# Evidence of CPV2c introgression into Croatia and novel insights into phylogeny and cell tropism

**DOI:** 10.1038/s41598-019-53422-9

**Published:** 2019-11-15

**Authors:** Dinko Novosel, Tamas Tuboly, Gyula Balka, Levente Szeredi, Ivana Lojkic, Andreja Jungic, Zaklin Acinger-Rogic, Tahar Ait-Ali, Attila Csagola

**Affiliations:** 1 Department of Animal science, Faculty of Agriculture University of Zagreb, Svetosimunska 25, 10000 Zagreb, Croatia; 2 Department of Microbiology and Infectious Diseases, Immunology, University of Veterinary Medicine, István u. 2, 1078 Budapest, Hungary; 3 Department of Pathology, University of Veterinary Medicine, István u. 2, 1078 Budapest, Hungary; 4 Veterinary Diagnostic Directorate National Food Chain Safety Office Budapest, Tábornok u, 2 H-1143 Hungary; 5 Department of Virology, Croatian Veterinary Institute, Savska cesta 143, 10000 Zagreb, Croatia; 6 Veterinary and Food Safety Directorate, Ministry of Agriculture, Planinska 2a, 10000 Zagreb, Croatia; 7 ANSES Fougeres Laboratory, 10B rue Claude Bourgelat, Javene, CS 40608 France; 8 Department of Pathology, Croatian Veterinary Institute, Savska cesta 143, 10000 Zagreb, Croatia

**Keywords:** Genome evolution, Viral pathogenesis

## Abstract

Canine parvovirus type 2 (CPV2) emerged for the first time in 1978 and evolved into two antigenic variants CPV2a and CPV2b and the third new antigenic variant CPV2c reported in 2000 in Italy. During 2014 unexplained outbreaks of gastroenteritis were observed in kennels where an extensive vaccination program was ongoing and where vaccinated animals showed pathologic lesions consistent with typical parvovirosis. The aim of this study was to investigate whether CPV2 could have played a role in the emergence of these cases and to evaluate genetic or pathological specificities of the virus and the disease. Using PCR and phylogenetic analysis we showed that the CPV2c variant is circulating in Croatia and is in close relationships with isolates from North and South America. Histopathological lesions and cell tropism that are known for CPV2 we are reporting the identification of the virus in glial cells and ovaries. It seems that evolution of CPV and CPV2a-c and adaptation to dogs are two independent events. Croatian isolates had specific and some unique amino acid mutations under positive selection. The effect of the alterations on the immunoglobulin binding cannot be excluded.

## Introduction

Parvoviridae are a family of small DNA viruses divided into two subfamilies *Parvovirinae* and *Densovirinae*, which infect vertebrates and insects, respectively. Five genuses are members of the *Parvovirinae subfamily: Protoparvovirus*, *Erythroparvovirus*, *Dependoparvovirus*, *Amdoparvovirus* and *Bocaparvovirus*. Canine parvovirus type 2 (CPV2) is a member of the *Protoparvovirus* genus and is host species specific such as *Carnivore protoparvovirus 1*, Feline panleukopenia virus, mink enteritis virus (MEV) and the raccoon parvovirus. CPV2 is genetically and antigenically unrelated to Carnivore bocaparvovirus 1, previously known as Canine minute virus (CnMV) or Canine parvovirus type 1 (CPV1), which causes neonatal mortality^1^. Parvoviruses are small (25 nm in diameter), non-enveloped and genome consist of an approximately 5200 nucleotides. VP1 is formed of the entire VP2 protein plus the addition of an extra domain at the N-terminal end. VP2 which forms 90% of viral capsid structural protein represents the main determinant for host interaction^2^. For this reason, over the years most studies were focused on the evolution of the VP2 gene, with limited studies on the nonstructural genes^3^. Also, NS gene sequences available in GenBank are relatively few if compared to partial or total length VP2 sequences. CPV2 first emerged in 1978 and quickly evolved into two antigenic variants CPV2a and CPV2b^4–8^. In 2000 a novel antigenic variant (CPV2c) was identified in Italy and subsequently spread to the neighboring countries^9,10^ whereas novel CPV2a variants have emerged in Hungary^11^. According to previous reports CPV is a variant of FPV that acquired the ability to infect canines through the acquisition of a small number of mutations in the capsid protein (VP2) gene responsible of change in surface-exposed residues^12–14^. These viruses use the transferrin receptor type 1 (TfR) as their primary receptor for attaching to and infecting cells. Canine cells resist infection by FPV because that virus cannot bind to the canine TfR, in particular because of a unique glycosylation site present in the canine TfR^13,15–18^. Four amino acid changes at VP2 residues 87, 101, 300, and 305 characterize differences of CPV2 and the CPV-2a variant. These four mutations map to or near the capsid surface and influence infection by altering binding to the carnivore transferrin receptor (TfR), the host cell attachment protein for these viruses^19^. Only two VP2 amino acid residues at position 426 (asparagine to aspartic acid) and 555 (isoleucine to valine have been identified (CPV-2a to CPV-2b)^20^. The new CPV2c variant harbours a mutation at residue 426 (asparagine/isoleucine to glutamate)^21^. However, as the CPV-2b and -2c antigenic strains differ from CPV-2a at only one position (VP2 residue 426), they are now considered to be variants of CPV-2a rather than distinct subtypes^22^. Although the site at position 426 confers only insignificant main chain movement, recent report suggests that Asp introduces a negative charge that is possibly blocking the interaction of some of antibodies^23^.

In most CPV2c outbreaks reported in adult dogs, the animals have undergone a full vaccination protocol^24,25^. Even considering all case reports of vaccination failures, there was no robust evidence for the absence of cross-protection from clinical disease between old and novel variants of CPV2 and the main issue is still related to the interference of vaccination by the maternal immunity^26^. The original viral strain designated as CPV2, and distinct from CPV1, can cause severe and fatal outbreaks of hemorrhagic gastroenteritis as well as subacute myocarditis in kennels and dog shelters^27^. Active viral circulation has often been exploited as a vaccination strategy to provide immune protection of dog population to reduce mortality and spread of the virus^26^.

CPV2 enters the host by the oronasal route. The virus replicates primarily in lymphoid cells prior to spreading to other mitotically active tissues including bone marrow and the germinal epithelium of the crypts of the small intestine, causing diarrhea^9,28–30^. Diarrhea is usually severe and often hemorrhagic, with sloughing of intestinal mucosa and replacement by cuboidal epithelium associated with lymphopenia and neutropenia resulting from necrosis of precursor cells^31,32^. Atrophy of the villi, necrosis of the epithelial cells, distension of the Lieberkühn’s crypts containing erythrocytes, depletion of Peyer’s patches and severe hemorrhagic enteritis are common lesions observed in CPV2 cases. Usually, CPV2 causes disease in unvaccinated 1–6-month-old pups. Vaccinated pups are usually protected from the disease and from infection, unless immunization fails due to the presence of high titers of maternally derived antibodies^30,33,34^. Similarly, adult dogs (≥1 year) are usually not susceptible to CPV2 infection due to vaccination or previous infections with field strain^8,27^.

Prophylaxis of CPV2 infection relies mainly on extensive vaccination. In most cases these vaccines induce long-term immunity^35,36^ and consist of attenuated strains either the original CPV2 or its variant CPV2b^37^. During 2014, several unexplained outbreaks of hemorrhagic gastroenteritis were observed in kennels and dog shelters despite the ongoing extensive vaccination programs: necropsy revealed typical clinical signs of a parvovirosis infection. Therefore, the aim of this study was to investigate whether CPV2 could have played a role in the emergence of these cases and to evaluate genetic or pathological specificities of the virus and the disease using a range of methodological approaches including immunohistochemistry, *in situ* PCR, sequencing, phylogenetic and sequence analysis.

## Results

### Presence of CPV2 in samples and phylogenetic analysis

**Figure 1:**
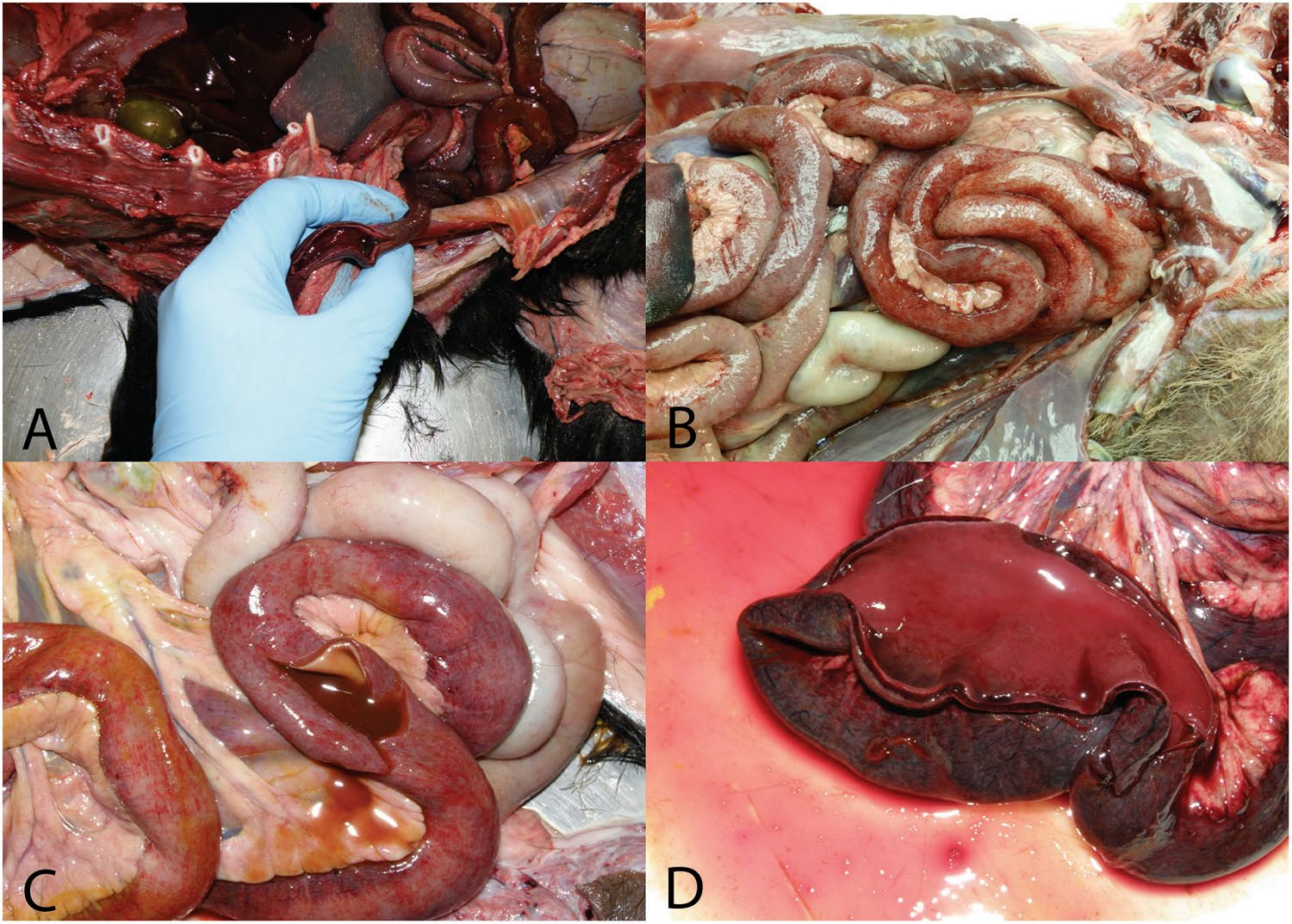
Typical lesions found in necropsied dogs in which CPV2 infection was suspected. (**A**) Severe hemorrhagic enterocolitis; (**B**) Intestinal ecchymosis; (**C**) Hemorrhagic enterocolitis; (**D**) Severe hemorrhagic inflammation of the intestinal mucosa.

Out of 11 animals that had a typical gross pathology lesions consistent for canine parvovirosis, 9 were confirmed positive for CPV2 by PCR (Fig. 1). Out of the 9 positive samples, we identified 5 novel nucleotide sequences encoding the VP2 region which were submitted to phylogenetic analysis. Single Breakpoint Recombination test confirmed that there was no recombination and recombinant strains in the dataset that was analyzed (Supplementary information Recombination test using Single break point analysis). A total of 23 different phylogeny models were tested (Table 1). According to results of the tests of different phylogeny models the age of The Most Common Recent Ancestor (TMCRA) is around 38–83 y with a Molecular clock of around 1E-4 mutations per base/per year/per site. The highest Akaike’s Information Criterion for Markov’s Chain Monte Carlo samples (AICM)^38^ was achieved when the codon Shapiro-Rambaut-Drummond 2006 model (SRD06) model and the highly parametric Coalescent Bayesian Skyline prior were used. We included Relaxed Molecular Clocks, uncorrelated lognormal and exponential, since we expected that the rate of evolution varied among the branches of the tree. When Strict clock was used, AICM was found a little bit lower. In both cases Molecular clock and Treehigh values were inside 1 standard deviation of the mean. In particular Relaxed lognormal molecular clock distribution had lower AICM than Relaxed exponential molecular clock. According to the phylogeny model when SRD06 codon model, Relaxed lognormal molecular clock and Coalescent Bayesian Skyline prior were used, TMCRA was estimated to be 57 y ago with a Molecular clock of 1,33E-4 (1,05E-4-1,63E-4) which was slower than previously estimated which is ranging between 1E-4 - 4E-4 mutations per year/per site^20^.

**Table 1:** Results of analysis of testing of different phylogeny models.

Substitution model	Molecular clock	Demographic model	AICM	AICM S.E.	Likelihood					Time Clock	Treeheight
					Mean	Stderr	Stdev	Variance	Median		
SRD06	lognormal	Skyline	14750,389	0,87	−6920,868	0,6955	21,3149	454,3267	−6920,4303	1,33E-04	56,53
YANG96	lognormal	Skyline	14814,664	1,319	−6881,9564	1,0399	22,9211	525,3759	−6881,4999	1,34E-04	57,52
GTR	strict	Skyline	15039,488	0,332	−7270,0426	0,5651	15,8019	249,7016	−7269,396	9,94E-05	63,54
TN	strict	Skyline	15045,206	0,288	−7269,9677	0,6691	15,8945	252,6351	−7269,3376	9,94E-05	63,34
GTR	strict	Exponential	15054,932	0,712	−7271,8802	0,567	15,987	255,5857	−7271,359	1,04E-04	60,39
HKY	strict	Skyline	15061,671	0,235	−7271,2307	0,6204	16,1123	259,6046	−7270,5718	9,95E-05	63,30
HKY	strict	Exponential	15103,558	0,373	−7274,2334	0,6181	16,6597	277,5455	−7273,6851	1,05E-04	60,23
GTR	strict	Constant	15176,989	0,259	−7294,2041	0,6241	17,1549	294,2903	−7293,6689	1,10E-04	75,21
GTR	lognormal	Skyline	15185,484	0,641	−7225,1862	0,5852	19,1717	367,5557	−7224,637	1,03E-04	59,93
HKY	strict	Constant	15185,713	0,302	−7296,122	0,6673	17,226	296,7344	−7295,705	1,11E-04	74,40
HKY	lognormal	Constant	15220,783	1,064	−7236,6342	0,7068	19,3328	373,7575	−7236,3062	1,36E-04	82,95
GTR	lognormal	Constant	15228,07	0,813	−7234,22	0,7667	19,4888	379,8152	−7233,8009	1,35E-04	83,79
GTR	lognormal	Exponent	15241,128	0,687	−7231,7305	0,6177	19,7189	388,8334	−7231,0663	1,10E-04	57,65
TN	lognormal	Constant	15243,848	0,787	−7233,4719	0,8295	19,7092	388,4522	−7233,0142	1,36E-04	83,58
HKY	lognormal	Exponential	15259,512	0,698	−7234,0025	0,7269	19,8935	395,7533	−7233,4183	1,16E-04	57,51
HKY	lognormal	Skyline	15274,597	0,836	−7227,9411	0,7945	20,2326	409,3574	−7227,2017	1,08E-04	60,17
JC	strict	Skyline	15568,929	0,213	−7564,2075	0,4269	14,841	220,2563	−7563,808	1,00E-04	63,12
JC	lognormal	Constant	15776,125	0,513	−7529,0754	0,6939	18,9469	358,9868	−7528,7064	1,33E-04	83,02
HKY	exponential	Exponential	20933,113	0,456	−10156,8428	0,3262	17,5986	309,7125	−10156,4941	1,32E-02	38,04
HKY	exponential	Skyline	20992,627	0,725	−10155,6312	0,6637	18,4576	340,6823	−10155,5158	1,34E-02	38,01
GTR	exponential	Skyline	21011,845	0,809	−10195,5903	0,3443	17,6163	310,3323	−10195,2899	1,41E-02	38,01
HKY	exponential	Constant	21078,474	0,966	−10216,2308	0,5423	17,9724	323,006	−10216,1617	1,55E-02	38,01
GTR	exponential	Constant	21583,068	1,086	−10292,0014	1,3058	22,3502	499,5326	−10293,6091	1,48E-02	38,01

**Figure 2:**
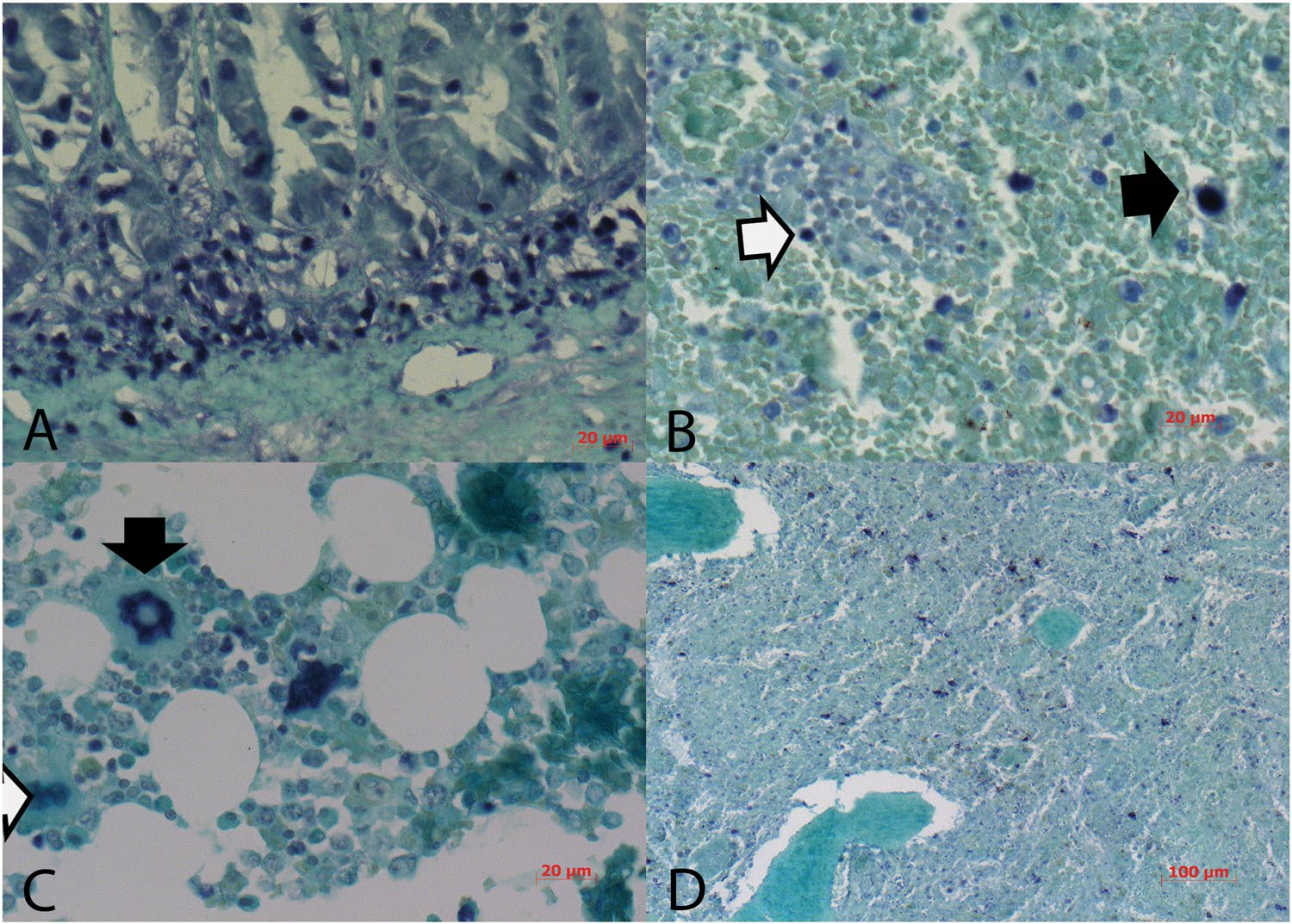
MCA tree revealed phylogenetic relationship of Croatian VP2 sequences. Sequences data are provided in taxa name. Each subtype/clade was colored differently; CPV2a dark blue, CPV2b blue, CPV2c light blue, FPV brown, MEV grey-green, yellow vaccine and red Croatian sequences. Red taxa name represent sequences originated from *Felides:* cat (*Felis catus*), bobcat (*Lynx rufus*), puma (*Puma concolor*) and leopard (*Pantera pardus*); blue taxa from *Mustelides*: Fisher (*Pekania pennant*) and Mink (*Neovision vision*) and green from other *Canides*, coyote (*Canis latrans*), grey wolf (*Canis lupus*), red fox (*Vulpes vulpes*), raccoon (*Procytor lotor*), raccoon dog (*Nyctereutes procyonoides*) and violet taxa from monkey. Scale length represents years.

The MCA tree showed that the five novel Croatian sequences belonged to subtype CPV2c (Fig. 2). HR442, HR774 and HR793 had the highest phylogenetic relationship with one Uruguayan sequence (KM457125) isolated from a dog and a North American sequence (KJ813846) isolated from a bobcat (*Lynx rufus*) and which is also in the same clade with French, Italian and other Uruguayan sequences. HR859 appeared to be a direct ancestor of Italian sequence (FJ005205) and in the same clade with French, North American and other Argentinian sequences. HR856 had, according to the tree topology, the same ancestor as the North American sequences (KJ813843) isolated from Bobcat and is in the same clade with Argentinian, Italian, French and Uruguayan sequences. According to this tree TMCRA of all the analyzed sequences was estimated to 57 y ago, while for CPV2a, b and c it was 39 y ago. Interestingly, some clades were specific but not exclusive to some species. In the MEV clade, a monkey isolate was also identified. In the clade with mostly from raccoon dog (*Nyctereutes procyonoides*) sequences we have also identified some early dog infection sequences and sequences mostly of vaccine origin. In the CPV2abc clade, virus isolates were mostly isolated from dog but also from puma (*Puma concolor*), coyote (*Canis latrans*), bobcat, grey wolf (*Canis lupus*), raccoon (*Procyon lotor*), fisher (*Pekania pennant*), cat (*Felis silvestra*), red panda (*Ailurus fulgens*) and mink (*Neovision vison*). In the FPV clade, sequences were also from many different species including grey wolf, fisher, raccoon, bobcat and red fox (*Vulpes Vulpes*). The Tree topology suggested a phylogenetic relationship but, in most of the cases, the posterior probability value was too low (PP < 0.95) to support diversifications. Only three clear branches were well supported by posterior probability: “old” CPV clade, MEV clade and FPV clade. Negative purifying selection pressure was identified in 23 codon positions. Synonymous mutations were observed mostly in vaccine strains and strains isolated from wild animals.

**Figure 3:**
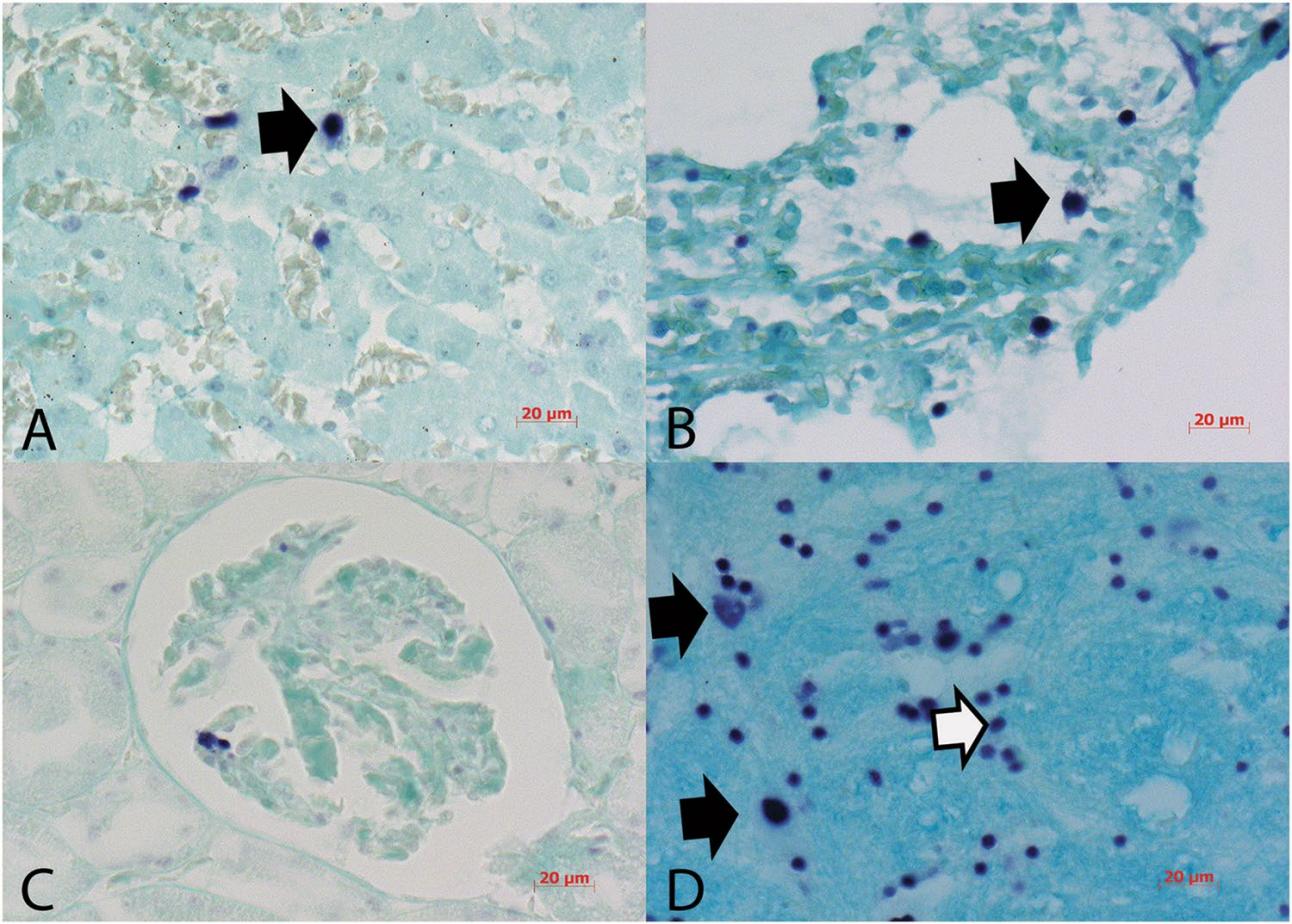
Skyplot to reconstruct historical population size. Axis values presents effective population size; scale length represents years; blue area is confidential interval.

Bayesian skyplot analysis showed that historical population size experienced significant growth bursts between 1973 and 1982 and again between 1998 and 2001 (Fig. 3). Since 2005 population size started to decline to reach an apparent steady-state from 2009. All five Croatian isolates had specific amino acid mutations M87L, L101I which is under episodic diversifying positive selection, A300G which is under episodic pervasive positive selection. D305Y^39^ which are present in CPV2a while A297S, which is typical for new variants 2a and 2b, was not observed^27,40^. It is notable that Q297S that is typical for South East Asia and F267Y, Y324I, T440A which is under episodic pervasive and diversifying positive selection) that are responsible for vaccine failures were not identified in any of the Croatian isolates^29,32^. In contrast HR856 harboured the specific mutation Q7H that is unique at this position together with G496A. While G496 of HR856 sequence is replaced with an A (mutation in second position of the codon) in raccoon it is replaced with a S (mutation in the first position of the codon). Croatian sequences had N426E which is under episodic diversifying positive selection that is typical for CPV2c^41–44^. Besides Croatian sequences, N426E was also observed in Italian, French, German, Argentinian and Australian isolated from dog and from North American sequences (Idaho, Montana and Colorado) isolated from dog, puma, bobcat and coyote. N426D was an amino acid mutation of interest since it resulted in the discrimination between CPV and CPV2a, CPV2b and was also associated with the binding affinity to the feline transferrin receptor as well as being involved in the replication of the virus in cats^6,20,45^. The third variant N426E was critical to help distinguish between CPV2a, CPV2b and CPV2c strains, but is obvious that CPV2c strains are able to replicate in felines too. Mutations at amino acid residue 324 may have had an impact on parvovirus host range^46^. Selection pressure analysis further revealed that 97 codons are under negative selection pressure or 99,203% sites while only 0,797% sites where under diversifying selection (Supplementary information Mixed Effects Model of Evolution, Fixed Effects Liklihood, Single-Likelihood Ancestor Counting, A Fast, Unconstrained Bayesian AppRoximation for Inferring Selection, Brench-Site Unrestricted Statistical Test for Episodic Diversification, The adaptive Branch-Site Random Effects Likelihood method, Detect relaxed selection in codon-based phylogenetic framework).

### Histopathology and ***in situ*** detection of CPV2 in organs

**Figure 4:**
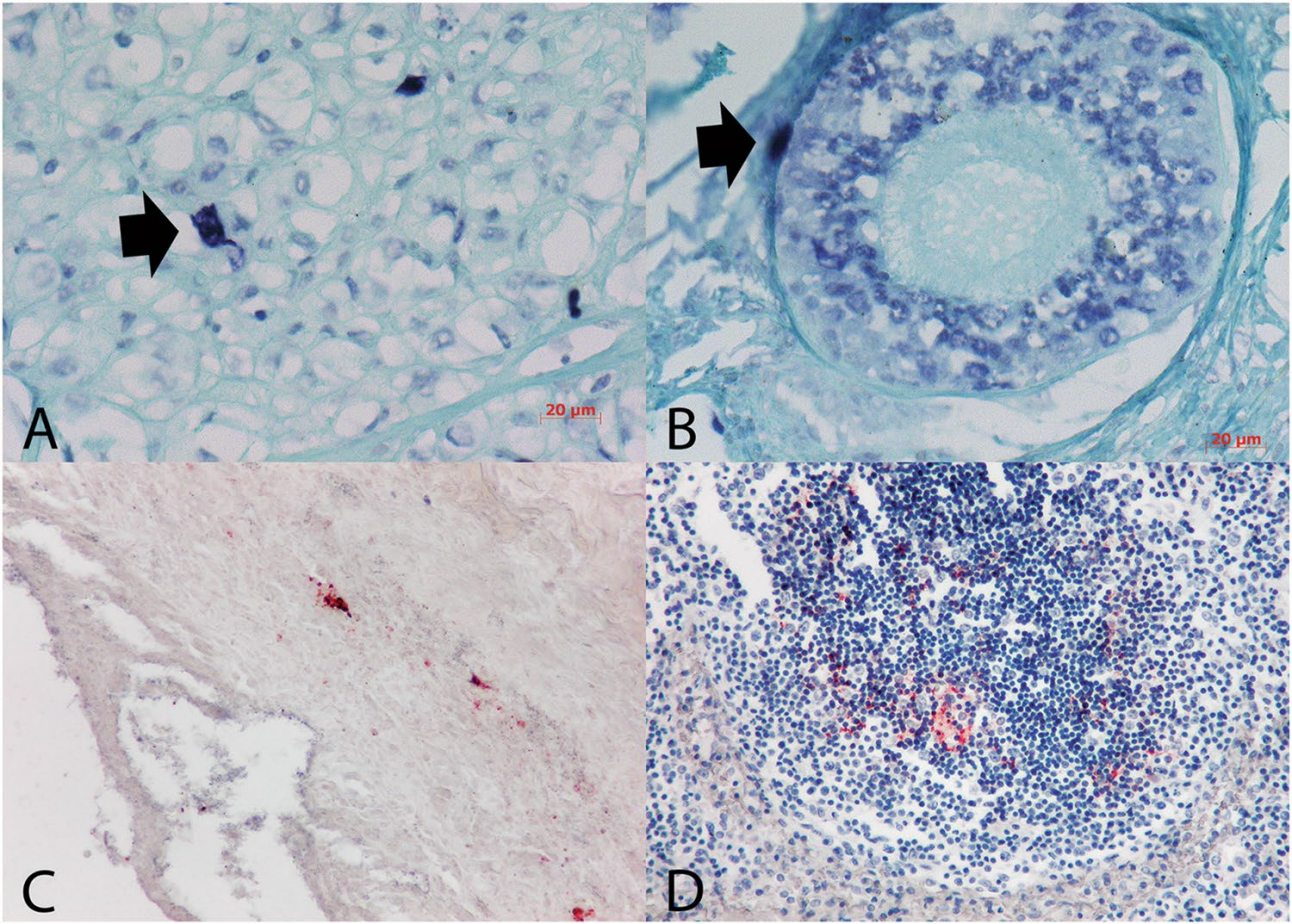
(**A**) Ileum with strong staining of CPV2 in submucosa and crypts; (**B**) Lymph nodes, bold arrow marks positive monocyte, white arrow marks lymphocyte; (**C**) Bone marrow, bold arrow marks positive megakaryocyte, white arrow marks lymphoblast; (**D**) Spleen, positive monocytes and lymphocytes. IS PCR for the detection of CPV2, NBT chromogen and counterstained with Fast Green.

The histopathologic lesions observed in the sections after H&E staining were necrotic and hemorrhagic enteritis of the small intestine, with dilated crypts often associated with the regeneration of epithelium (Fig. 4A). Shortened and fused intestinal villi, lymphoid depletion in the lymph nodes, spleen and tonsils were noticed. In the spleen, macrophages with golden-brown pigment were often observed except in one sample where foci of necrosis were seen. In liver samples, lymphocytic hepatitis with strong blood congestion, perivascular lymphocytic cuffing and weak infiltration inside sinusoidal vessels were also observed. In bone marrow, aplastic pancytopenia was present. Mild intramuscular bleedings and myofibrillar degeneration were present in myocardium. Interstitial pneumonia with proliferation of pneumocytes type 2 and strong congestions were observed in lungs whereas in pancreas congestion was hardly noticeable. Epithelial cells in tubules of the kidney cortex were affected by vacuolar degeneration. Apoptosis of astrocytes in brain and cerebellum were noticed besides micro gliosis (Fig. 4D).

**Figure 5:**
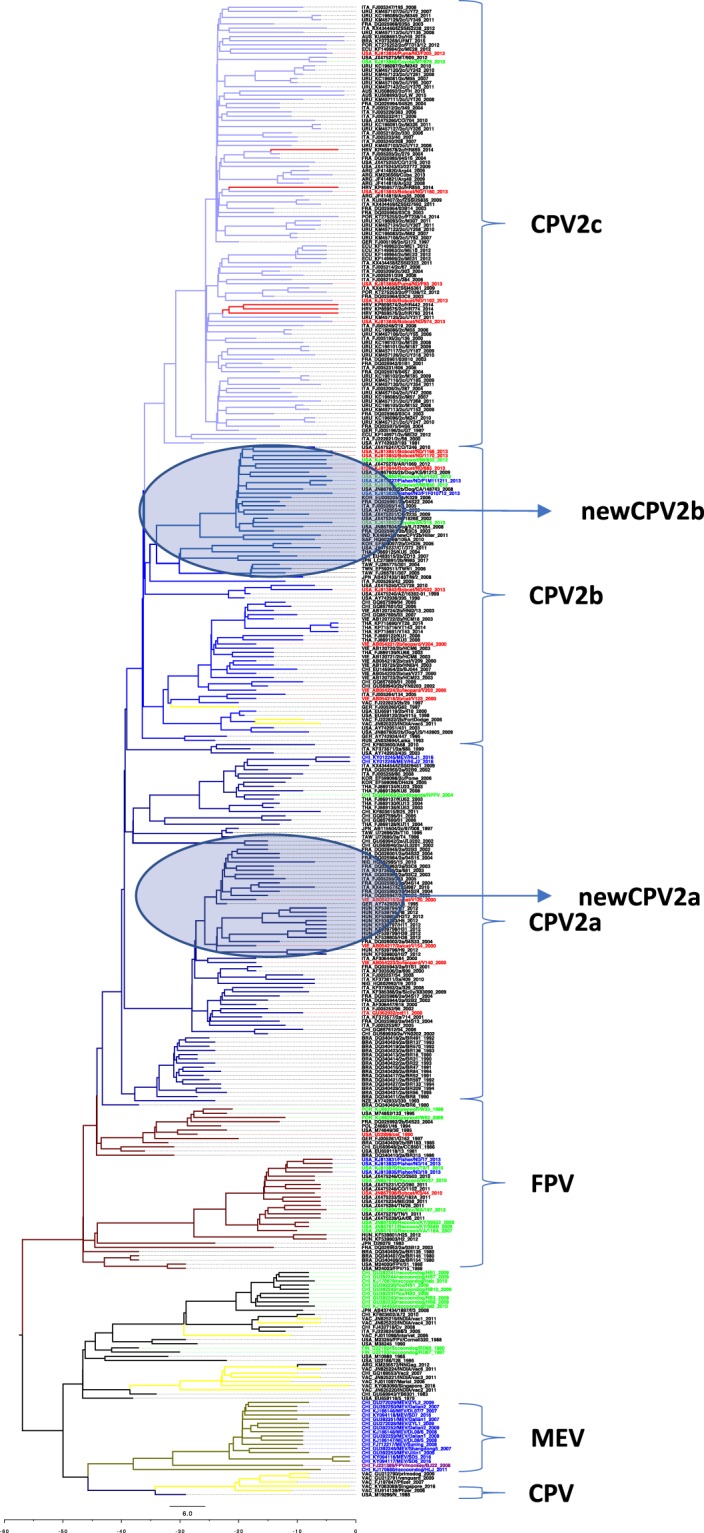
(**A**) Liver, bold arrow marks positive Kupffer cell; (**B**) lungs, bold arrow marks alveolar macrophage; (**C**) kidney, positive glomerular cell; (**D**) Cerebellum, bold arrows marks neurons and white arrow marks glia cells. IS PCR for the detection of CPV2, NBT chromogen, counterstained with Fast Green.

**Figure 6:**
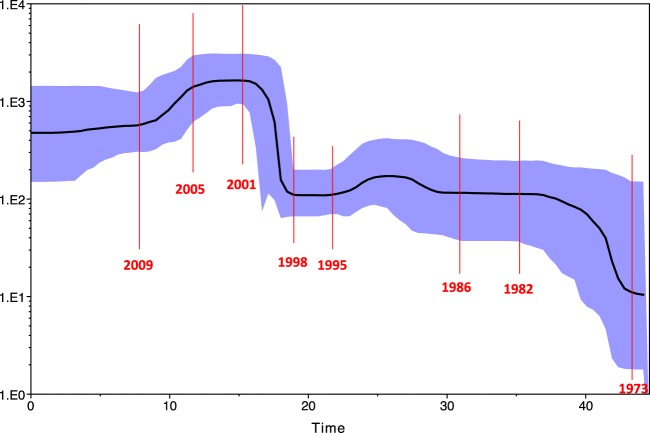
(**A**) Corpus luteum, bold arrow mark macrophages; (**B**) Graafian follicle, bold arrow marks granulosa cells. IS PCR for the detection of CPV2, NBT chromogen and counterstaining with Fast Green; (**C**) Ileum with low amount of CPV2 antigen, ABC chromogen and counterstaining with Heamtoxylin, 200x; (**D**) Lymph node with low amount of CPV2 antigen is noticeable in a follicle, IHC to detect CPV2, ABC chromogen and counterstained with Heamtoxylin, 400x.

CPV2 was readily detected using *in situ* PCR (IS PCR) in a wide range of organs (Figs 4–6). Eighteen amplification cycles were sufficient to detect the virus in ileum (Fig. 4A), colon, pancreas, liver (Fig. 5A), ovaries (Fig. 6A,B), lymph node (Fig. 4B), tonsils, pancreas, spleen (Fig. 4D), bone marrow (Fig. 4C) and lung (Fig. 5D) whereas thymus, heart, brain, cerebellum and kidney were negative. Strong positive signals were noticed when the amplification cycle number was raised to 30 for samples from brain, cerebellum (Fig. 5D) and kidney (Fig. 5C) whereas myocardium remained negative. In the ileum, intense staining was observed in submucosal inflammatory cells, goblet cells and enterocytes (Fig. 4A), in both cytoplasm and nuclei. In some strong infections, CPV2 genome was also identified in *tunica muscularis* and more specifically in aggregates around blood vessels. In some cases, staining was so intense that it was not possible to discriminate which cells in the mucosa or the submucosa were indeed infected. CPV2 was detected in the epithelium lining the gastric pits but not in glands. In contrast, staining was randomly observed in a few cells in the submucosa of the colon. Besides the ileum, a strong CPV2 signal was noticed in mesenteric lymph nodes (Fig. 4B) as well as in monocytes and lymphocytes of the spleen (Fig. 4D). Megakaryocytes and lymphoblasts harbored positive signal in bone marrow (Fig. 4C). Kupffer cells in the liver showed intense CPV2 signal (Fig. 5A) and nearby hepatocytes, and infected cells were aggregated around blood vessels. In lungs, lymphocytes and alveolar macrophages appeared to be the only infected cells (Fig. 5D). Kidneys were negative for CPV2 presence except for one clinical sample where the positive staining was found in glomerular cells (Fig. 5C). Further positive signals were observed in neurons and glia cells of the brain (Fig. 5D) as well as in macrophages in corpus luteum of ovaries (Fig. 5A) and in cells of *membrana granul*osa in Graafian follicle (Fig. 6B).

Samples that showed strong positive signals by IS PCR for the presence of CPV2 nucleic acid were submitted for the detection of CPV2 antigen using immunohistochemistry (IHC) (Fig. 6C,D). In four animals IS PCR detected a strong signal in a wide range of organs while IHC barely detected a weak signal for CPV2 antigen just in lymph nodes and intestines in two animals out of four. All other organs and animals remained negative by IHC. Based on the intensity and the distribution of the signal, abundant DNA or phagocytosed virions were highlighted in Kupffer cells in liver, megakaryocytes in bone marrow, monocytes, alveolar macrophages, macrophages in corpus luteum and neurons.

## Discussion

This report describes the exploitation of complementary approaches including phylogenetic analysis, amino acid analysis, Bayesian skyplot, recombination and selection pressure test. IS PCR and IHC was used to confirm the presence of CPV2 in all the submitted clinical samples that we have investigated. In this study, we have analysed one gene locus, VP2, to acquire a snapshot of “Carnivore” Parvovirus evolution. Our analysis indicated that TMCRA sequence was dated 57 y ago (95% HPD 42–74 y) which would suggest that around 1960 (1943–1975) FPV, MEV and CPV started to diversify. CPV2 started to branch from FPV 43 y ago, around 1978 which coincides with the first outbreak of CPV2 in the same year. Our observation with Skyplot may suggest that an occurrence of each new genogroup resulted in population size expansion while the use of new vaccine resulted in population size decrease. The expansion shift in Skyplot was visible around 1978 when the disease was reported as significant, and which was subsequently followed by a large one with the occurrence of CPV2c. In contrast, the implementation of an effective vaccine in 1981^47,48^ may have led to a stabilization of the viral population. The first strain of CPV was replaced by CPV-2a globally during 1979 and 1980, which represents the common ancestor of all the CPVs currently circulating in dogs world^3,49^. Clade FPV-CPV2a-c (Fig. 2) is supported by statistically significant PP 0,941 (Supplementary information Posterior probability tree) and this evolutionary analysis confirms that CPV2a-c has evolved from FPV. But, advanced Bayesian MCMC as a very strong phylogeny tool reveals that CPV and CPV2a-c do not have the same close TMCRA (Fig. 2), CPV2 evolved together with MEV (PP is 0,4666, too low to be statistically significant therefore branching between MEV and CPV is unconfident) and not FPV. These results are interesting since they strongly suggest that the evolution of VP2 of CPV and CPV2a-c are two independent events. This important observation may explain one of the major biological differences between CPV2 and CPV2a which is the ability of the latter to infect cats *in vitro*^50,51^. Our analysis seems to indicate that, based on the VP2 region we have analysed, CPV2, FPV, MEV may potentially be one transspecies virus which can infect the *Order Carnivore*. Although our phylogenetic results of the Croatian strains showed close relationship with isolates that originated from the North and South America in neither case was the tree topology supported by robust statistically significant posterior probability. All Croatian strains had codons under positive selection which means that those strains have some desirable traits. Those strains have some positive traits which offer them advantage, better chance for survival. Mutations of codons (amino acids) in Croatian strains are at the antibody footprint on the surface of CPV2^23^. Immune response means selection pressure is as evolution engine. An amino acid change is able to alter the structure of antibody binding site on the surface even if this mutation is not affecting directly the antibody binding site. The effect of the alterations on the immunoglobulin binding cannot be excluded.

Selection analysis is in consistence with skyplot, that CVP2 population was in last period stabile, not significant intensification or relaxation of selection trend in population.

In this study, we have exploited IS PCR for the detection of CPV2. Our data suggest IS PCR method is more sensitive than IHC without compromising on the specificity of the detection of CPV2 and could represent a versatile method that could greatly enhance pathology analysis. We were able to consistently identify the presence of CPV2 in glial cells and ovaries. Glial cell proliferation was associated with neuronophagia (degenerative or dead neuron was engulfed by microgliocytes) as previously reported^52^. This finding contrasts greatly with the current knowledge indicating that CPV2 has only a tropism for highly mitotically active tissues, whereas neurons do not replicate and originate from stem cells^28^. In contrast, glial cells are capable of mitosis. Astrocytes are star-shaped glial cells that have also been observed to turn into neurons by virtue of the pluripotency of the stem cell. IS PCR revealed that CPV2 was present in both, glia cells and neurons; with perhaps glial cells apparently being more predominantly infected than neurons. CPV2 was also identified in macrophages in *corpus lutea* and in Graafian follicles. This observation is consistent with the known tropism of CPV2 for the monocyte/macrophage cell lineage. *Corpus luteum* is a special type of cicatrix, and during the process of healing, the granulomatous tissue is gradually replacing ovulating Graafian follicles and - as a consequence - it is expected to be populated with macrophages*. Membrana granulosa* is consisting of granulosa cells which are somatic cells and are mitotically active.

## Conclusion

An important finding of this work is that CPV2c isolates circulate in Croatia. Our analysis indicated that TMCRA sequence was dated 57 y ago which would coincide with the first MEV outbreak in Canada. CPV2a-c started to branch from FPV just around the first outbreak of CPV2 which seems to have an evolutionary history related to MEV. In each clade a wide range of different species of the Order *Carnivorae* were observed which indicate that *Protoparvoviruse*s may potentially be one transspecies virus which can occasionally infect the Order *Carnivore*. Phylogenetic results of the Croatian strains showed close relationships with isolates that originated from the North and South of America, Italy and France. Historical population size experienced significant growth bursts between 1973 and 1982 and again between 1998 and 2001, Since 2005 population size started to decline to reach an apparent steady-state from 2009. Croatian isolates had specific and some unique amino acid mutations under positive selection. The effect of the alterations on the immunoglobulin binding cannot be excluded.

Finally, in this study, we implemented a highly sensitive *in situ* method to discover the presence of CPV2 in cell types and organs such as glial cells and ovaries. Further work is required to confirm if this novel observation is indeed associated to the CPV2c variant only.

## Material and Methods

### Ethical statement

In this study, the tissue samples that were analysed originated exclusively from dead animal cases caused by natural infection that were routinely submitted for necropsy to the Department of Pathology at the Croatian Veterinary Institute. Therefore, no institutional or licensing committee approval was needed since necropsy and tissue sampling is a common practice in pathology laboratories.

### Samples

In 2014, 7 dogs and 4 puppies (3, 4, 6 and 6 weeks old) were submitted to the Croatian Veterinary Institute for necropsy. These animals harbored pathological lesions typically associated with canine parvovirosis. Two animals were bitches originating from kennels where an outbreak of hemorrhagic enteritis occurred causing high mortality rates. While all bitches were routinely vaccinated most of their puppies died by the age of 1 month. Five dogs that originated from shelters received one shot of vaccine whereas the dog that was privately owned was regularly vaccinated against CPV2. From 2 litters, unrelated to the examined bitches, which died from hemorrhagic diarrhea, altogether 4 puppies were submitted for necropsy. Puppies were vaccinated twice, with a polyvalent vaccine, while all other animals were vaccinated several times according to standard prophylaxic measures and manufactures recommendations. The polyvalent vaccine included Canine parvovirus, Canine distemper, Canine adenovirus type 2, Canine parainfluenza virus, two serovariants of *Leptospira canicola* and *icteroheamorrhagiae*. All samples are geographically un-related and originated from city of Zagreb, middle and northern Croatia and from Dalmatia region. During the post-mortem examinations of the first six animals a tissue set was obtained for histopathological examinations, including ileum, mesenterial lymph node, spleen, liver, whereas in five other animals brain, heart, lung, kidney, pancreas, tonsils, thymus, bone marrow, submandibular, praescapular and inguinal lymph nodes, uterus and ovaries were also sampled. All tissue specimens were fixed in 10% buffered formaldehyde solution at room temperature for 2 days, dehydrated through different grades of ethanol and in xylene and embedded in paraffin. 3–4 µm tissues and mounted on Super Frost glass slides (Thermo Scientific). Subsequently they were routinely stained with the standard technique of Hematoxylin and Eosin and examined under light microscope. Sampled parts of organs of ileum, spleen and lymph nodes were stored at −18 °C.

### Classical PCR and phylogenetic analysis

To assess the background level of CPV2 infected sections, pooled parts of ileum, lymph node and spleen were submitted to PCR, and the VP2 encoding sequence was determined according to a protocol described previously^11^. The five Croatian sequences were aligned with the following reference sequences (GenBank accession numbers): CPV2a (M74849)^53^, CPV2b (M24003)^6^, CPV2c (FJ222821)^25,54^, newCPV2a (EU213073); and newCPV2b (AB054218)^55^ and CPV, Feline parvovirsu (FPV) and MEV altogether 356 sequences, selected using BlastTool to find most simmilar and dissimilar sequences to Croatian sequences randomly, and also known sequences from neigbor countries. Taxa name is consist of folowing informations: “Country of origin_Gene accsess number/subtype/host/strain data/strain data_year of isolation”. If host species is not menthion in taxa name then the host is dog or in case of MEV then the host is mink. Subtype was added if the appropriate metadata was present in the Genbank entry for a specific sequence. Sequences were aligned using the ClustalW program in MEGA 6 software^56^ to generate.nexus file and to perform amino acid sequence analysis. Prior to the phylogenetic analyses the dataset as checked for evidence of possible recombination events using a Single Breakpoint Recombination test implemented in HyPhy software^57^. Bayesian pylogeny was calculate using BEAST software package BEAST v2.4.8 and BEAST v1.4.3. Program was run with Markov Chain Monte Carlo length until Efective sample size values were over 200 for Likelihood, Posterior, Prior and Timeclock. Calculation were performed using different substitution models: Generalise times reversible (GTR)^58^, Hasegawa-Kishino-Yano (HKY)^59^, Jukes-Cantor 69 (JC69)^60,61^ and Tamura-Nei (TN93)^62^ including 2 codon models SRD06^63^ and YANG96^64^ where using strict molecular clock and relaxed lognormal and exponential molecular clock^65^; and different population size prior: Coalescent Constant, Coalescent Exponential and Coalescent Bayesian Skyline model. To compare different phylogenetic models BEAST log files were analyzed in Tracer v1.6 to calculate AICM parametar and which model better fits^38,66^. Selected tree file was compiled in TreeAnnotator v2.4.7 from BEAST package and te Most common ancestor (MCA) tree was constructed in FigTree v1.4.3^67^. Amino acid sequence analyisis was perfromed using MEGA 6 software. To investigate population history bayesian skyplot was calculated in Tracer v1.6.0 software^68^. To evaluate selection presure we used Fixed Effects Liklihood (FEL)^69^, A Fast, Unconstrained Bayesian AppRoximation for Inferring Selection (FUBAR)^70^, Detect Individual Sites Subject to Episodic Diversifying Selection - Mixed Effects Model of Evolution (MEME)^71^, Single-Likelihood Ancestor Counting (SLAC)^69^, Brench-Site Unrestricted Statistical Test for Episodic Diversification (BUSTED)^72^, Detect relaxed selection in codon-based phylogenetic framework (RELAX)^73^ and An adaptive branch-site REL test for episodic diversification The adaptive Branch-Site Random Effects Likelihood method (aBSREL)^74,75^ implemented in HyPhy software^57^ where p < 0.1 as a default value to detect selection pressuer and p < 0.05 were considered as statisticaly significant.

### Immunohistochemistry

Immunohistochemical labeling of CPV2 antigen was performed with anti-CPV2 mouse monoclonal antibody (Mab) (A3B10, VMRD, Pullman, USA). After dewaxing of sections, antigen retrieval was performed with 0.1% protease XIV solution (Sigma Aldrich Co.) at 37 °C for 10 min. Endogenous peroxidase was blocked with 3% H_2_0_2_ solution for 10 min and non-specific immune reactions were blocked with incubation of 2% solution of skimmed milk powder for 20 minutes. The sections were incubated with the primary antibody at a dilution of 1:300 overnight at 4 °C. Reaction was visualized using anti-mouse secondary antibody (DAKO EnVision^TM^ + HRP Mouse Kit, K4001) and AEC chromogen (DAKO AEC + substrate-chromogen system K3469) according to the manufacturer’s instructions. The slides were counterstained with Mayer’s hematoxylin and mounted with glycerol-gelatine. As a negative control, an additional section was incubated in a similar manner with PBS. As a positive control organ from a dog that died from parvovirus enteritis were used.

### *In situ* PCR

Direct *in situ* (IS) PCR was performed as previously described^76–81^ with some modifications. For IS PCR specific primers was applied to amplify a long sequence 681 bp product as was described previously^6,21,82^ and were further adjust for IS PCR^77–81^, activation step 5 min at 95 °C, annealing 2 min at 56 °C, elongation 2 min at 72 °C, denaturation 45 s at 95 °C and final elongation 7 min at 72 °C. In the first trail, all organs were performed with just 18 cycles for amplification. In a subsequent trial, all negative slides were repeated using 30 cycles for amplification. To exclude unspecific primer amplification, positive samples were repeatedly tested with only forward or reverse primer. As a negative control ileum and lymph nodes obtained from a dog that was negative for the presence of CPV2 by classic PCR were used.

## Supplementary information

Recombination test using Single break point analysis

Mixed Effects Model of Evolution

Fixed Effects Liklihood

Single-Likelihood Ancestor Counting

A Fast, Unconstrained Bayesian AppRoximation for Inferring Selection

Brench-Site Unrestricted Statistical Test for Episodic Diversification

The adaptive Branch-Site Random Effects Likelihood method

Detect relaxed selection in codon-based phylogenetic framework

Posterior probability tree
