# Supplementary material for: Evidence of CPV2c introgression into Croatia and novel insights into phylogeny and cell tropism
Source: Sci Rep. 2019 Nov 15;9:16909. doi: 10.1038/s41598-019-53422-9 (PMC6858334; doi:10.1038/s41598-019-53422-9)
Supplement: Supplementary file 1 — Recombination test using Single break point analysis [file 41598_2019_53422_MOESM1_ESM.pdf]

Evidence of CPV2c introgression into Croatia and novel insights into phylogeny and cell tropism

Dinko Novosel, Tamas Tuboly, Gyula Balka, Levente Szeredi, Ivana Lojkic, Andreja Jungic, Zaklin Acinger Rogic, Tahar Ait Ali, Attila Csagola

Supplementary info file 1.
Results of Recombination test using Single break point analysis in HYPHY 2.3.13. software.

```
    /HYPHY 2.3.13.20180601beta(MPI) for Darwin on x86_64\
***************** TYPES OF STANDARD ANALYSES *****************


    (1) Selection Analyses
    (2) Evolutionary Hypothesis Testing
    (3) Relative evolutionary rate inference
    (4) Basic Analyses
    (5) Codon Selection Analyses
    (6) Compartmentalization
    (7) Data File Tools
    (8) Miscellaneous
    (9) Model Comparison
    (10) Kernel Analysis Tools
    (11) Molecular Clock
    (12) Phylogeny Reconstruction
    (13) Positive Selection
    (14) Recombination
    (15) Selection/Recombination
    (16) Relative Rate
    (17) Relative Ratio
    (18) Substitution Rates

 Please select type of analyses you want to list (or press ENTER to
process custom batch file):14

***************** FILES IN 'Recombination' *****************


    (1) Screen an alignment using GARD (requires an MPI
environment).
    (2) Process GARD results.
    (3) A Likelihood Ratio Test to detect conflicting phylogenetic
signal Huelsenbeck and Bull, 1996. [Contributed by Olivier Fedrigo].
    (4) Search an alignment for a single breakpoint.
    (5) Plot genetic distances (similarity) of one sequence against
all others in an alignment, using a sliding window. Optionally,
determine NJ-based clustering and bootstrap support in every window.
This is a HyPhy adaptation of the excellent (but Windows only tool)
```

```
SimPlot (and/or VarPlot) written by Stuart Ray (http://
sray.med.som.jhmi.edu/SCRoftware/simplot/)

 Please select the analysis you would like to perform (or press
ENTER to return to the list of analysis types):4


            +---------+
            |Data type|
            +---------+


    (1):[Nucleotide] Nucleotide data.
    (2):[Codon] Codon (several available genetic codes).

Please choose an option (or press q to cancel selection):1

Locate a nucleotide data  file: (`/Users/dinkonovosel/hyphy/res/
TemplateBatchFiles/`) 1

Locate a nucleotide data  file: (`/Users/dinkonovosel/hyphy/res/
TemplateBatchFiles/`) /Users/dinkonovosel/CPV_VP2_cod.fas

There are 298 potential breakpoints.


            +----------+
            |KH Testing|
            +----------+


    (1):[Skip] Use only AIC to measure goodness of fit.
    (2):[Run 1] Verify conflicting phylogenetic signal with KH
resampling, swapping trees between partitions for the test
    (3):[Run 2] Verify conflicting phylogenetic signal with KH
resampling, using the joint tree as the null.

Please choose an option (or press q to cancel selection):1


              +--------------------------+
              | Select a standard model. |
              +--------------------------+



    (CUSTOM):Custom 4x4 reversible model (defined as in 010023)
with choice of several equilibrium frequencies options.
    (F81):Felsenstein 81 (F81).Rate heterogeneity (and HM spatial
correlation) optional.
    (F84):Felsenstein 84. Rate heterogeneity (and HM spatial
correlation) optional.
    (F84P):Felsenstein 84, compatible with PHYLIP's dnaml version.
```

```
User chooses ts/tv ratio. Rate heterogeneity (and HM spatial
correlation) optional.
    (GRM):General Reversible Model.Local or global parameters.
Possible Rate heterogeneity (and HM spatial correlation).
    (HKY85):Hasegawa et al.,1985. Local or global parameters.
Possible Rate heterogeneity (and HM spatial correlation).
    (JC69):Jukes-Cantor 1969. Rate heterogeneity (and HM spatial
correlation) optional.
    (K2P):Kimura 2 parameter (K2P).Local or global parameters.
Possible Rate heterogeneity (and HM spatial correlation).
    (K3ST):Kimura(1981) 3 substitution type model. Local or global
parameters. Possible Rate heterogeneity (and HM spatial
correlation).
    (NRM):Non reversible model, constrained to have observed counts
as its equilibrium frequencies. 11 parameters (8 rate parameteres +
3 frequency parameters).
    (NRM+FREQS):Non reversible model, NOT constrained to have
observed counts as its equilibrium frequencies. 11 parameters.
    (TRN):Tamura and Nei 1993. Local or global parameters. Local or
global parameters. Possible Rate heterogeneity (and HM spatial
correlation).

 Please type in the abbreviation for the model you want to use:NRM

Save results to: (`/Users/dinkonovosel/hyphy/res/TemplateBatchFiles/
`) /Users/dinkonovosel/output

1). Single partition analysis
Log Likelihood = -6490.33700241856;
Shared Parameters:
GA=0.9623750663265883
GC=0.1335566667929499
GT=0.157141482600193
CT=0.7426581885794767
AC=0.1230222119233418
AT=0.1192611546636207
CG=0.1117794129802484
CA=0.154016677060744
TA=AT+(AC-CA)*0.542755952673748+(1-
GA)*0.6821334429009175=0.1281039497559198
TC=CT+(CA-AC)*1.194527793611708+(CG-
GC)*0.6821334429009175=0.7648269455140476
TG=GT+(GA-1)*1.194527793611708+(GC-
CG)*0.542755952673748=0.1240171877342364

Tree
givenTree=((((((((((((((((((((((((((((VIE_AB054215_2a_cat_V120_2
000:0.00114701,
(FRA_DQ025947_2a_02B5_2002:0.000573883,ITA_FJ005255_333_2005:0.00114
744)Node33:0)Node31:0,FRA_DQ025962_2a_03C6_2003:0.00057373)Node30:0,
FRA_DQ026001_2a_04S32_2004:0.000573954)Node29:0,GER_AY742935_U6_1995
:
0)Node28:0,FRA_DQ025945_2a_02B3_2002:0)Node27:0,ITA_KF373580_2a_581_
```

2003:0)Node26:0,FRA_DQ025958_2a_03C2_2003:0.000573699)Node25:0,
((FRA_DQ025983_2a_04S14_2004:0,FRA_DQ025993_2a_04S24_2004:0.00172177
)Node43:0,ITA_KX434457_987_10_2010:0.000573468)Node42:0.000573931)No
de24:0,
(FRA_DQ025984_2a_04S15_2004:1.1776e-20,ITA_FJ005252_96_2002:0.001148
44)Node47:0.000574022)Node23:0.000573882,
(((((((VIE_AB054217_2a_cat_V154_2000:0.00172421,HUN_KF539796_H_9_201
2:0.00057404)Node56:0,HUN_KF539800_H_27_2012:0.000574008)Node55:0.00
0574025,
((HUN_KF539793_H_5_2012:0,HUN_KF539797_H_11_2012:0.00114879)Node61:0
.000574223,
((HUN_KF539794_H_7_2012:0,HUN_KF539795_H_8_2012:0.00114824)Node65:0,
HUN_KF539804_H_212_2012:0.00172402)Node64:0)Node60:0.00057375)Node54
:0,
(CHI_GQ857612_CPV08_04_2008:0.000573694,CHI_GU569939_2a_YN0202_2002:
0.00057441)Node69:0.00114872)Node53:0,
(((((((ITA_AF306446_584_2000:0.000574184,
(FRA_DQ025982_2a_04S13_2004:0.00114774,ITA_FJ005253_67_2005:0.001147
81)Node80:0)Node78:0,FRA_DQ025986_2a_04S17_2004:0.000574066)Node77:0
,ITA_KF373577_2a_714_2001:0.000573882)Node76:0,
((HUN_KF539798_H_31_2012:0.00057354,HUN_KF539799_H_39_2012:0.0011492
)Node86:0.000574689,HUN_KF539805_H_36_2012:0)Node85:0.000574241)Node
75:0,NIG_HQ602992_19_10_2010:0.000574066)Node74:0,ITA_AF306447_618_2
000:0)Node73:0,FRA_DQ025944_2a_02B2_2002:0)Node72:0)Node52:0,
((ITA_AF393506_2a_699_2000:0,FRA_DQ025943_2a_01S1_2001:0.00057373)No
de94:0,ITA_KF385388_2a_Sicily_X83090_2009:0.000574201)Node93:0.00057
3882)Node51:0,ITA_KF373592_2a_329_2008:0.000574186)Node50:0)Node22:0
,FRA_DQ026002_2a_04S33_2004:0.000574066)Node21:0.000574048,NIG_HQ602
995_15_10_2010:0.000574065)Node20:0,
(VIE_AB054223_2c_leopard_V140_2000:0.00114811,ITA_GU362932_cat11_200
8:0.0017256)Node101:0)Node19:0.000574066,NZE_AY742933_339_1993:0.001
724)Node18:0,(((((((JPN_AB437434_1887_f_3_2008:0.00114794,
(((CHI_GU392236_fox_HB1_2009:0,
((CHI_GU392241_raccoondog_HB1_2009:0.000574222,
(CHI_GU392242_raccoondog_HB10_2009:0.00114814,CHI_GU392244_raccoondo
g_HB7_2009:0.00114851)Node120:0)Node118:0,CHI_KJ170679_raccoondog_He
b10_2_2010:0.000574587)Node117:0)Node115:0.000574003,
((CHI_GU392239_raccoondog_HB6_2009:0.000574282,CHI_GU392240_raccoond
og_HB3_2009:0)Node125:0,CHI_KJ194463_raccoondog_HeB10_3_2010:0.00057
4216)Node124:0)Node114:0,CHI_GU392237_fox_HB2_2009:0.0017234)Node113
:0.000575528)Node111:0.00114863,
((((((USA_EU659116_CPV_5_1979:0,FIN_U22193_raccoondog_RD87_1987:0.00
114934)Node135:0,USA_M23255_FPV_Cornell320_1988:0)Node134:0,USA_M382
45_1990:0)Node133:0,FIN_U22192_raccoondog_RD_80_1980:0)Node132:0,
(((((((VAC_FJ011097_Merial_2006:0.00172753,CHI_GQ169553_Vac2_2007:0.
00172586)Node147:0.000573813,VAC_KY083090_Singapore_2016:0.00288073)
Node146:0,(CHI_GU569943_YB8301_1983:0.00114871,
(VAC_JN625224_INDIA_vac6_2011:0.00114834,ARG_KM236572_NNGag_2012:0)N
ode153:0)Node151:0.00114893)Node145:0.000766742,VAC_FJ011098_Interve
t_2006:0.00326032)Node144:0,VAC_JN625220_INDIA_vac2_2011:0.00366751)
Node143:0.000947955,VAC_JN625221_INDIA_vac3_2011:0.00114152)Node142:
0.000583916,(((ITA_FJ222824_388_05_3_2005:0,
(CHI_FJ432718_CPV_Cv_2008:0.000574405,

(VAC_JN625219_INDIA_vac1_2011:0.00114858,CHI_KF803602_2010_BJ_A72_20
10:0)Node165:0.000574286)Node163:0)Node161:0,VAC_JN625222_INDIA_vac4
_2011:0.00114859)Node160:0.00114882,
(USA_M10989_1985:0.00461932,USA_U22186_CPV_128_1995:0)Node169:0)Node
159:0.000574523)Node141:0)Node131:0.000574752,
(((VAC_EU914139_Pfizer__2006:0.00230165,(VAC_FJ197847_Pfizer_2007:0,
(VAC_GU212790_primodog_2009:0.00114883,VAC_GU212791_vanguard_2009:0.
000571316)Node178:0.00172792)Node176:0.000574336)Node174:0,VAC_KY083
089_Singapore_2016:0)Node173:0.00114902,USA_M19296_CPV_N_1988:0.0005
75297)Node172:0.000574159)Node130:0)Node110:0.000574597,
(CHI_FJ231389_FPV_monkey_BJ_22_2008:0.00289005,CHI_KJ170680_raccoond
og_HLJ11_1_2011:0.00115229)Node183:0.00114744)Node109:0,CHI_KF803600
_2010_BJ_A68_2010:0.0034576)Node108:0.00114895,
((((((USA_JN867598_Bobcat_KS_44_2010:0.000574774,USA_KJ813832_Fisher
_ND_14_2013:0)Node192:0.000574776,
(USA_KJ813831_Fisher_ND_17_2013:0,USA_KJ813835_Fisher_ND_19_2013:0.0
005748)Node195:0)Node191:0,(((USA_JN867618__Raccoon_WI_37_2010:0,
((USA_JX475233_SC_182_A_2011:0.000574383,USA_JX475246_CO_2503_2010:0
.
000574442)Node203:0,USA_JX475234_ME_258_2011:0.000574383)Node202:0)N
ode200:0,USA_JX475231_CO_280_2011:0)Node199:0,USA_JX475248_CO_1102_2
011:0.000574578)Node198:0.000574615)Node190:0.000574418,USA_KJ813870
_Raccoon_TX_1_2013:0)Node189:0.00115022,
((USA_JN867599_Raccoon_KY_39552_2009:0,USA_JN867611_Raccoon_KY_358_B
_2009:0.000573955)Node211:0.000574299,
(((USA_JN867610_Raccoon_VA_118_A_2007:0.000574406,
(USA_JX475239_GA_06_2011:0.000574336,USA_JX475279_TN_1_2011:0.001148
63)Node218:0)Node216:0,USA_KJ813890_Redfox_MA_197_2012:0.000574406)N
ode215:0,USA_JX475284_TN_26_2011:0)Node214:0)Node210:0.00115107)Node
188:0.000573697,
(HUN_KF539801_H_25_2012:0.000574226,HUN_KF539803_H_2_2012:0.00057553
8)Node223:0.00114934)Node187:0)Node107:0.000574687,
((JPN_D26079_1993:0.00114924,
(USA_EU659118_CPV_13_1981:0.00114864,CHI_GU569948_2a_CC8601_1986:0.0
00574676)Node229:0)Node227:0.000574223,
((FRA_DQ025952_2a_03B12_2003:0.00172432,
(((BRA_DQ340404_2a_BR6_1980:0,BRA_DQ340410_2a_BR315_1986:0.00172468)
Node237:0,
(USA_M24000_FPV_CPV_31_1988:0.000574817,USA_M24003_FPV_CPV_15_1988:0
)Node240:0.00057433)Node236:0,BRA_DQ340405_2a_BR135_1980:0)Node235:0
)Node233:0,
(BRA_DQ340407_2a_BR145_1980:0,BRA_DQ340408_2a_BR154_1980:0)Node244:0
.000574634)Node232:0)Node226:0)Node106:0.000574602,
(((((((FRA_DQ025950_2a_02B9_2002:0,ITA_KX434454_29451_09_2009:0)Node
253:0,KOR_EF599098_2c_Pome_2006:0.00344892)Node252:0.000573882,CHI_D
Q354068_2a_redpanda_RPPV_2004:0.000574296)Node251:0,
(KOR_EF599096_DH426_2005:0.000574066,
((THA_FJ869126_KU5_2008:0.000573882,THA_FJ869134_KU23_2003:0)Node261
:
0,THA_FJ869137_KU52_2003:0)Node260:0)Node258:0)Node250:0.000573988,I
TA_FJ005258_80_2008:0.000574048)Node249:0,
((THA_FJ869130_KU13_2004:0,THA_FJ869138_KU53_2003:0)Node267:0.000574
373,CHI_KF803615_2011_BJ_B25_2011:0)Node266:0)Node248:0,

(CHI_GU569942_2a_JL0202_2002:0,CHI_GU569946_2a_JL0201_2002:0.0017251
)Node271:0.00114848)Node247:0.000574071)Node105:0)Node17:0,
(USA_AY742953_435_2003:0.00114799,ITA_KF373571_2a_685_1999:0)Node274
:0)Node16:0,
(ITA_FJ005257_54_2008:0.000574249,ITA_KF373611_2a_409_2010:0)Node277
:0.000574385)Node15:0.000574192,
(((((((((VIE_AB054218_2b_cat_V123_2000:0,VAC_FJ222823_2b_29_1997:0.
00172383)Node289:0,ITA_FJ005264_134_2005:0.00229936)Node288:0,
((((((((VIE_AB054219_2b_cat_V209_2000:0.000574353,
(VIE_AB054220_2b_cat_V217_2000:0.00114774,
(VIE_AB120723_2b_HCM_23_2003:0.000574205,CHI_EU145954_2b_BJ044_2007:
0.00114774)Node305:0)Node303:0)Node301:0,VIE_AB120720_2b_HCM_6_2003:
0.000574232)Node300:0,VIE_AB120725_2b_HNI_3_4_2003:0)Node299:0,VIE_A
B054224_2c_leopard_V203_2000:0.00172363)Node298:0,VIE_AB054221_2b_le
opard_V204_2000:0.000574048)Node297:0,VIE_AB120721_2b_HCM_8_2003:0.0
0172437)Node296:0,THA_FJ869139_KU66_2003:0.00057408)Node295:0,
((((VIE_AB120722_2b_HCM_18_2003:0.000574049,VIE_AB120724_2b_HNI_2_13
_2003:0)Node317:0,
(CHI_GQ857599_CPV05_04_2005:0.00057359,CHI_GQ857601_CPV06_02_2006:0.
000574658)Node320:0.000574068)Node316:0,
((THA_KP715690_VT28_2014:0,THA_KP715716_VT143_2014:0)Node324:0.00114
704,THA_KP715691_VT43_2014:0)Node323:0.00172421)Node315:0,CHI_GQ8576
05_CPV07_03_2007:0.00114771)Node314:0.00114831)Node294:0,
(THA_FJ869122_KU1_2008:0.00114846,THA_FJ869123_KU3_2008:0)Node329:0.
00057408)Node293:0.000574066)Node287:0,
(((((JPN_AB115504_2c_97_008_1997:0.000574092,TAW_U72696_2b_T10_1996:
0)Node336:0.000574117,TAW_U72695_2a_T4_1996:0)Node335:0.000574676,
(CHI_GQ857596_CPV05_01_2005:0.000574283,CHI_GQ857600_CPV06_01_2006:0
.
000574966)Node340:0.00057428)Node334:0,THA_FJ869125_KU5_2004:0.00114
81)Node333:0.000574387,(((BRA_DQ340411_2a_BR8_1990:0.00114801,
(BRA_DQ340428_2a_BR209_1994:0,BRA_DQ340431_2a_BR56_1995:0.000574509)
Node348:0)Node346:0.000574242,
((((((((((BRA_DQ340413_2a_BR18_1990:0.000574385,BRA_DQ340419_2a_BR5
70_1992:0.000574352)Node361:0,BRA_DQ340423_2a_BR136_1993:0.000574346
)Node360:0,BRA_DQ340414_2a_BR31_1990:0)Node359:0,BRA_DQ340416_2a_BR4
7_1991:0)Node358:0,BRA_DQ340417_2a_BR52_1991:0)Node357:0,BRA_DQ34041
8_2a_BR491_1992:0)Node356:0,BRA_DQ340424_2a_BR137_1993:0)Node355:0,B
RA_DQ340426_2a_BR84_1994:0)Node354:0,BRA_DQ340427_2a_BR133_1994:0)No
de353:0,BRA_DQ340421_2a_BR597_1992:0.000574385)Node352:0,BRA_DQ34042
2_2a_BR22_1993:0.000574384)Node351:0)Node345:0.00057425,THA_FJ869128
_KU11_2004:0.000574386)Node344:0)Node332:0.000574529)Node286:0.00057
4174,((((JPN_AB437433_1887_M_2_2008:0.000574066,
((TWN_EF592511_TWN1_2006:0.00057397,TAW_FJ265775_CPV301_2004:0.00114
816)Node381:0,TAW_FJ265781_CPV307_2005:0)Node380:0)Node378:0.0005738
69,JPN_LC270891_2b_9985_2017:0.00114792)Node377:0,CHI_EU483515_2b_ZD
13_2007:0.00057373)Node376:0.000573666,
(USA_JX475237_CT_372_2011:0,KOR_EF599097_2b_DH326_2006:0.000573992)N
ode387:0.00057463)Node375:0.000574345)Node285:0,
(CHI_GQ857609_CPV08_01_2008:0.00057335,CHI_GU569940_2b_YN0203_2002:0
.000574786)Node390:0.0017258)Node284:0,
(((((((((((USA_AY742955_436_2003:0,
(((USA_JX475278_AR_1069_2012:0.000573659,

(USA_KJ813851_Bobcat_ND_1168_2013:0.000573659,
(USA_KJ813881_Graywolf_MI_832_2012:0.00114698,USA_KJ813882_Raccoon_N
J_1423_2012:0.000573647)Node412:0)Node410:0)Node408:0,USA_KJ813844_B
obcat_ND_885_2013:0.000574342)Node407:0,USA_KJ813828_Fisher_F1F01071
2_2013:0.00114778)Node406:0)Node404:0,FRA_DQ025991_2b_04S22_2004:0)N
ode403:0,USA_JN867603_2b_Dog_KS_81213_2009:0.000573647)Node402:0,USA
_JX475251_CO_2235_2009:0.000573659)Node401:0,USA_KJ813852_Bobcat_ND_
1170_2013:0.000573821)Node400:0,USA_JX475247_CO_1246_2010:0.00057365
7)Node399:0,
(KOR_EU009205_2b_K029_2006:0.000573659,USA_JN867602_2b_Dog_CA_148743
_2008:0.000573647)Node422:0)Node398:0,
((USA_JN867604_Dog_IL_137654_2008:0.000573883,USA_JX475242_WI_18268_
2002:0.000573931)Node426:0,USA_KJ813892_Coyote_AK_218_2013:0.0005738
83)Node425:0.000574088)Node397:0,
(USA_KJ813827_Fisher_F1M111211_2013:0,USA_KJ813873_Graywolf_MI_850_2
012:0.000573573)Node430:0.000573887)Node396:0,FRA_DQ025961_2b_03C5_2
003:0.000573821)Node395:0.00114802,ITA_FJ005265_140_2005:0)Node394:0
.00114808,ITA_FJ005263_42_2005:0)Node393:0.000574434)Node283:0,
(USA_AY742932_193_1991:0,
((USA_AY742951_431_2003:0,USA_JN867605_2b_Dog_US_142805_2009:0.00057
3751)Node439:0,
(((USA_EU659119_2b_CPV_410_2000:0,USA_EU659120_2b_CPV_411a_1998:0.00
0573917)Node444:0.000573752,VAC_JN625223_INDIA_vac5_2011:0.00057374)
Node443:0,VAC_FJ222822_2b_FortDodge_2008:0.000573752)Node442:0.00057
3751)Node438:0.00114813)Node436:0)Node282:0,
((((FRA_DQ025992_2b_04S23_2004:0.00114814,
(((POR_KU662349_greywolf_W33_1996:0,POR_KU662350_greywolf_W52_2005:0
.
00230328)Node456:0,USA_M74852_133_1995:0)Node455:0,POL_Z46651_46_199
4:0)Node454:0)Node452:0,
(USA_M74849_39_1995:0,USA_U22896_cat_1990:0.000574577)Node461:0.0005
74424)Node451:0,GER_FJ005261_G162_1997:0.00172478)Node450:0,BRA_DQ34
0409_2b_BR183_1985:0.000574393)Node449:0.000574103)Node281:0,
(SAF_HQ602969_22_10SA_2010:0,
(ECU_KF149971_2c_ME32_2012:0.00172742,IND_KX469432_newCPV_2b_Hiller_
2011:0.00172671)Node468:0.000571826)Node466:0.000574041)Node280:0)No
de14:0.000574275,
((GER_AY742934_447_1995:0.000572667,RUS_JN033694_Laika_1993:0.002301
19)Node472:0.000575963,(USA_AY742936_395_1998:0,
(USA_JX475240_AZ_16382_01_1999:0,
(USA_JX475250_CO_728_2010:0.000573565,USA_KJ813842_Bobcat_ND_502_201
3:0.000573565)Node479:0)Node477:0.00114773)Node475:0)Node471:0.00057
3965)Node13:0,
(GER_FJ005260_G82_1997:0.00172323,USA_KJ813846_Bobcat_ND_974_2013:0.
000573934)Node482:0)Node12:0.00057418,ITA_FJ222821_2c_56_2000:0.0005
74037)Node11:0,((FRA_DQ025985_04S16_2004:0.000574076,
(ITA_FJ005205_2c_279_2004:0,HRV_KP859578_2c_HR859_2014:0.000573546)N
ode489:0.00057378)Node487:0.000574301,ITA_FJ005232_411_2006:0.001147
32)Node486:0)Node10:0,URU_KM457126_2c_UY318_2010:0.00114769)Node9:0,
(((URU_KC196081_2c_M95_2007:0,URU_KM457109_2c_UY95_2007:0)Node496:0.
00114794,
((URU_KC196097_2c_M242_2010:0,URU_KM457120_2c_UY242_2010:0)Node500:0
,URU_KM457123_2c_UY261_2008:0)Node499:0)Node495:0.000574201,

((URU_KC196093_2c_M307_2011:0,URU_KM457122_2c_UY258_2010:0)Node505:0
,URU_KM457124_2c_UY307_2011:0)Node504:0)Node494:0.000574191)Node8:0,
URU_KM457142_2c_UY370_2011:0)Node7:0,
((URU_KC196083_2c_M82_2007:0,URU_KM457108_2c_UY82_2007:0)Node511:0.0
0114775,URU_KM457125_2c_UY317_2011:0)Node510:0)Node6:0,
(((((FRA_DQ025964_03C8_2003:0,POR_KT275253_2c_PT036_12_2012:0.000573
965)Node519:0,ITA_KX434456_45361_09_2009:0)Node518:0.000574199,
(URU_KC196086_2c_M55_2006:0,URU_KM457106_2c_UY55_2006:0)Node523:0.00
114787)Node517:0,AUS_KU508691_2c_HB_2015:0)Node516:0,
(FRA_DQ025994_04S25_2004:0.00057386,POR_KT275255_2c_PT238_14_2014:0.
00114789)Node527:0)Node515:0)Node5:0,
(((FRA_DQ025942_01B1_2001:0,URU_KC196107_2c_M129_2008:0.00114739)Nod
e532:0,
(URU_KC196101_2c_M187_2009:0,URU_KM457117_2c_UY187_2009:0)Node535:0.
000573858)Node531:0,
(GER_FJ005199_2c_G172_1997:0.000573892,URU_KC196085_2c_M57_2007:0.00
0574044)Node538:0)Node530:0)Node4:0,
(ITA_FJ005195_2c_136_2000:0.000574286,
((((ARG_JF414818_Arg32_2008:0.000573734,ARG_KM236569_Cuba_2013:0.000
573765)Node546:0,ARG_JF414821_Arg48_2009:0.000573733)Node545:0,ARG_J
F414820_Arg44_2009:0)Node544:0.00057389,USA_KJ813858_Puma_ND_F93_201
3:0.00057386)Node543:0)Node541:0)Node3:0,
((((FRA_DQ025969_03S5_2003:0.000574018,USA_JX475252_CO_1316_2010:0.0
00573859)Node555:0,
(((URU_KC196096_2c_M247_2010:0,URU_KM457121_2c_UY247_2010:0)Node560:
0.00057387,USA_KJ813848_Bobcat_ND_1162_2013:0.000574044)Node559:0,
(URU_KC196102_2c_M185_2009:0,URU_KM457116_2c_UY185_2009:0)Node564:0.
00057386)Node558:0)Node554:0,
(((FRA_DQ025975_04S6_2004:0.000574044,HRV_KP859577_2c_HR856_2014:0.0
0114898)Node569:0,
((((ECU_KF149962_2c_ME1_2012:0,ECU_KF149963_2c_ME10_2012:0.000573734
)Node575:0,ECU_KF149964_2c_ME23_2012:0.000573734)Node574:0,ECU_KF149
969_2c_ME31_2012:0.000573734)Node573:0.00057389,URU_KM457130_2c_UY35
4_2011:0)Node572:0)Node568:0,
(ITA_FJ005240_208_2007:0.000574044,ITA_FJ005248_219_2008:0.000574018
)Node581:0)Node567:0)Node553:0,
(((ITA_FJ005206_2c_287_2004:0,URU_KM457131_2c_UY368_2011:0.00114739)
Node586:0,
(((ITA_FJ005214_2c_67_2006:0.000573888,BRA_KY073269_UFMT_2015:0.0005
73884)Node591:0.00057423,URU_KM457111_2c_UY120_2008:0)Node590:0,
(URU_KC196089_2c_M349_2011:0,URU_KM457129_2c_UY349_2011:0)Node595:0.
000573847)Node589:0)Node585:0,
((ITA_FJ005226_383_2006:0.000574044,URU_KM457104_2c_UY47_2006:0.0005
73891)Node599:0,
(ECU_KF149984_2c_ME28_2012:0.000574044,AUS_KU508693_2c_LW_2015:0.000
574023)Node602:0)Node598:0)Node584:0)Node552:0)Node2:0,
(((((FRA_DQ025951_03B10_2003:0.000573892,ITA_FJ005231_406_2006:0.001
14732)Node609:0,HRV_KP859575_2c_HR774_2014:0)Node608:0,
(ITA_FJ005212_2c_349_2004:0.000574199,
(URU_KC196105_2c_M152_2008:0,URU_KM457113_2c_UY152_2009:0)Node615:0.
00114804)Node613:0)Node607:0,
((FRA_DQ025954_03B14_2003:0.000574044,USA_KJ813843_Bobcat_ND_1160_20
13:0.0011481)Node619:0,AUS_KU508692_2c_FH_2015:0)Node618:0)Node606:0

```
,
(((USA_JX475273_MT_909_2012:0.000574113,USA_KJ813888_Coyote_MT_878_2
012:0)Node625:0.000574126,
(URU_KM457107_2c_UY72_2007:0,POR_KT275252_2c_PT013_12_2012:0.0011473
9)Node628:0)Node624:0,
(USA_KJ813854_Puma_ND_F205_2013:0.00114739,URU_KM457112_2c_UY135_200
8:0)Node631:0)Node623:0)Node605:0)Node1:0,
(((((FRA_DQ025960_03C4_2003:0.000573892,ARG_JF414819_Arg35_2008:0.00
114732)Node638:0,HRV_KP859576_2c_HR793_2014:0)Node637:0,
(ITA_FJ005247_195_2008:0.000573892,ITA_KX434460_52238_12_2012:0.0011
4798)Node642:0)Node636:0,
(((ITA_FJ005209_2c_303_2004:0,ITA_FJ005251_239_2008:0.000573734)Node
647:0.000573891,URU_KM457103_2c_UY12_2006:0.00114798)Node646:0,HRV_K
P859574_2c_HR442_2014:0)Node645:0)Node635:0,
(((GER_FJ005196_2c_G7_1997:0.000573892,
(URU_KC196091_2c_M326_2011:0,URU_KM457127_2c_UY326_2011:0)Node656:0.
00114819)Node654:0,ITA_KX434458_2323_11_2011:0)Node653:0,
((ITA_FJ005218_2c_330_2006:0.000574026,ITA_FJ005233_40_2007:0)Node66
1:0.000573838,USA_JX475260_CO_704_2010:0.000573892)Node660:0)Node652
:0)Node634:0,
((FRA_DQ025965_03C9_2003:0.000574044,USA_JX475243_ID_22772_2009:0.00
0573891)Node666:0,(FRA_DQ025976_04S7_2004:0.000574184,
(ITA_FJ005216_2c_284_2006:0.00057386,
(ITA_KU508407_2c_25835_09_2009:0,ITA_KX434459_27692_1_11_2011:0.0005
73734)Node673:0.000573891)Node671:0)Node669:0)Node665:0);

 AIC  = 14346.67400483711

c-AIC = 15228.96295667847

 BIC  = 23175.62792501746
2). Looking for a breakpoint...

Breakpoint at position     17. dAIC =    -1321.32 dAICc =  -10051.13
dBIC =  -10046.86null
Breakpoint at position     20. dAIC =    -1314.29 dAICc =  -10044.09
dBIC =  -10039.83null
Breakpoint at position     35. dAIC =    -1144.19 dAICc =   -9873.99
dBIC =   -9869.73null
Breakpoint at position     36. dAIC =    -1061.71 dAICc =   -9791.51
dBIC =   -9787.25null
Breakpoint at position     59. dAIC =     -943.98 dAICc =   -9673.79
dBIC =   -9669.52null
Breakpoint at position     60. dAIC =     -937.27 dAICc =   -9667.08
dBIC =   -9662.81null
Breakpoint at position     62. dAIC =     -933.14 dAICc =   -9662.94
dBIC =   -9658.68null
Breakpoint at position     67. dAIC =     -929.41 dAICc =   -9659.21
dBIC =   -9654.95null
Breakpoint at position     74. dAIC =     -921.50 dAICc =   -9651.31
dBIC =   -9647.04null
Breakpoint at position     95. dAIC =     -897.15 dAICc =   -9626.96
dBIC =   -9622.69null
```

Breakpoint at position 101. dAIC = −889.79 dAICc = −9619.60
dBIC = −9615.33null
Breakpoint at position 112. dAIC = −877.18 dAICc = −9606.98
dBIC = −9602.72null
Breakpoint at position 119. dAIC = −872.58 dAICc = −9602.38
dBIC = −9598.12null
Breakpoint at position 121. dAIC = −867.16 dAICc = −9596.96
dBIC = −9592.70null
Breakpoint at position 128. dAIC = −862.89 dAICc = −9592.70
dBIC = −9588.43null
Breakpoint at position 129. dAIC = −801.14 dAICc = −9530.94
dBIC = −9526.68null
Breakpoint at position 134. dAIC = −759.86 dAICc = −9489.67
dBIC = −9485.40null
Breakpoint at position 143. dAIC = −739.18 dAICc = −9468.98
dBIC = −9464.72null
Breakpoint at position 146. dAIC = −591.69 dAICc = −9321.49
dBIC = −9317.23null
Breakpoint at position 147. dAIC = −586.91 dAICc = −9316.71
dBIC = −9312.45null
Breakpoint at position 165. dAIC = −579.67 dAICc = −9309.48
dBIC = −9305.21null
Breakpoint at position 179. dAIC = −573.78 dAICc = −9303.59
dBIC = −9299.32null
Breakpoint at position 197. dAIC = −565.86 dAICc = −9295.66
dBIC = −9291.40null
Breakpoint at position 221. dAIC = −555.19 dAICc = −9284.99
dBIC = −9280.73null
Breakpoint at position 227. dAIC = −553.79 dAICc = −9283.59
dBIC = −9279.33null
Breakpoint at position 233. dAIC = −554.79 dAICc = −9284.60
dBIC = −9280.33null
Breakpoint at position 234. dAIC = −554.03 dAICc = −9283.84
dBIC = −9279.57null
Breakpoint at position 238. dAIC = −552.32 dAICc = −9282.13
dBIC = −9277.86null
Breakpoint at position 239. dAIC = −551.37 dAICc = −9281.18
dBIC = −9276.91null
Breakpoint at position 245. dAIC = −533.64 dAICc = −9263.45
dBIC = −9259.18null
Breakpoint at position 246. dAIC = −534.38 dAICc = −9264.19
dBIC = −9259.92null
Breakpoint at position 247. dAIC = −531.84 dAICc = −9261.65
dBIC = −9257.38null
Breakpoint at position 251. dAIC = −547.55 dAICc = −9277.36
dBIC = −9273.09null
Breakpoint at position 257. dAIC = −543.32 dAICc = −9273.13
dBIC = −9268.86null
Breakpoint at position 258. dAIC = −526.78 dAICc = −9256.59
dBIC = −9252.32null
Breakpoint at position 260. dAIC = −516.30 dAICc = −9246.10
dBIC = −9241.84null
Breakpoint at position 261. dAIC = −515.54 dAICc = −9245.35

```
dBIC =   −9241.08null
Breakpoint at position    266. dAIC =     −511.64 dAICc =   −9241.44
dBIC =   −9237.18null
Breakpoint at position    278. dAIC =     −494.93 dAICc =   −9224.73
dBIC =   −9220.47null
Breakpoint at position    291. dAIC =     −486.95 dAICc =   −9216.76
dBIC =   −9212.49null
Breakpoint at position    301. dAIC =     −459.47 dAICc =   −9189.27
dBIC =   −9185.01null
Breakpoint at position    302. dAIC =     −404.36 dAICc =   −9134.16
dBIC =   −9129.90null
Breakpoint at position    307. dAIC =     −401.75 dAICc =   −9131.56
dBIC =   −9127.29null
Breakpoint at position    311. dAIC =     −395.43 dAICc =   −9125.24
dBIC =   −9120.97null
Breakpoint at position    313. dAIC =     −392.33 dAICc =   −9122.14
dBIC =   −9117.87null
Breakpoint at position    317. dAIC =     −389.01 dAICc =   −9118.81
dBIC =   −9114.55null
Breakpoint at position    328. dAIC =     −389.85 dAICc =   −9119.65
dBIC =   −9115.39null
Breakpoint at position    329. dAIC =     −388.68 dAICc =   −9118.48
dBIC =   −9114.22null
Breakpoint at position    330. dAIC =     −385.81 dAICc =   −9115.61
dBIC =   −9111.35null
Breakpoint at position    332. dAIC =     −375.52 dAICc =   −9105.32
dBIC =   −9101.06null
Breakpoint at position    334. dAIC =     −367.52 dAICc =   −9097.32
dBIC =   −9093.06null
Breakpoint at position    339. dAIC =     −366.53 dAICc =   −9096.33
dBIC =   −9092.07null
Breakpoint at position    343. dAIC =     −367.30 dAICc =   −9097.10
dBIC =   −9092.84null
Breakpoint at position    353. dAIC =     −366.32 dAICc =   −9096.12
dBIC =   −9091.86null
Breakpoint at position    356. dAIC =     −363.43 dAICc =   −9093.24
dBIC =   −9088.97null
Breakpoint at position    368. dAIC =     −359.69 dAICc =   −9089.50
dBIC =   −9085.24null
Breakpoint at position    370. dAIC =     −356.88 dAICc =   −9086.68
dBIC =   −9082.42null
Breakpoint at position    380. dAIC =     −356.00 dAICc =   −9085.80
dBIC =   −9081.54null
Breakpoint at position    383. dAIC =     −356.05 dAICc =   −9085.86
dBIC =   −9081.59null
Breakpoint at position    390. dAIC =     −355.74 dAICc =   −9085.54
dBIC =   −9081.28null
Breakpoint at position    404. dAIC =     −411.23 dAICc =   −9141.04
dBIC =   −9136.77null
Breakpoint at position    407. dAIC =     −407.96 dAICc =   −9137.76
dBIC =   −9133.50null
Breakpoint at position    414. dAIC =     −406.69 dAICc =   −9136.49
dBIC =   −9132.23null
```

Breakpoint at position 416. dAIC = −407.12 dAICc = −9136.93
dBIC = −9132.66null
Breakpoint at position 425. dAIC = −405.99 dAICc = −9135.79
dBIC = −9131.53null
Breakpoint at position 434. dAIC = −406.85 dAICc = −9136.66
dBIC = −9132.39null
Breakpoint at position 449. dAIC = −417.11 dAICc = −9146.92
dBIC = −9142.65null
Breakpoint at position 459. dAIC = −403.11 dAICc = −9132.92
dBIC = −9128.65null
Breakpoint at position 466. dAIC = −404.13 dAICc = −9133.93
dBIC = −9129.67null
Breakpoint at position 467. dAIC = −404.90 dAICc = −9134.70
dBIC = −9130.44null
Breakpoint at position 470. dAIC = −402.61 dAICc = −9132.41
dBIC = −9128.15null
Breakpoint at position 482. dAIC = −398.08 dAICc = −9127.88
dBIC = −9123.62null
Breakpoint at position 489. dAIC = −395.37 dAICc = −9125.18
dBIC = −9120.91null
Breakpoint at position 491. dAIC = −390.63 dAICc = −9120.44
dBIC = −9116.17null
Breakpoint at position 503. dAIC = −366.88 dAICc = −9096.68
dBIC = −9092.42null
Breakpoint at position 504. dAIC = −367.84 dAICc = −9097.65
dBIC = −9093.38null
Breakpoint at position 506. dAIC = −365.92 dAICc = −9095.73
dBIC = −9091.46null
Breakpoint at position 513. dAIC = −374.51 dAICc = −9104.31
dBIC = −9100.05null
Breakpoint at position 518. dAIC = −370.60 dAICc = −9100.41
dBIC = −9096.14null
Breakpoint at position 533. dAIC = −369.89 dAICc = −9099.70
dBIC = −9095.43null
Breakpoint at position 536. dAIC = −388.45 dAICc = −9118.25
dBIC = −9113.99null
Breakpoint at position 551. dAIC = −388.82 dAICc = −9118.63
dBIC = −9114.36null
Breakpoint at position 563. dAIC = −382.28 dAICc = −9112.08
dBIC = −9107.82null
Breakpoint at position 574. dAIC = −379.33 dAICc = −9109.13
dBIC = −9104.87null
Breakpoint at position 584. dAIC = −380.01 dAICc = −9109.81
dBIC = −9105.55null
Breakpoint at position 587. dAIC = −375.02 dAICc = −9104.82
dBIC = −9100.56null
Breakpoint at position 590. dAIC = −373.29 dAICc = −9103.09
dBIC = −9098.83null
Breakpoint at position 593. dAIC = −371.71 dAICc = −9101.52
dBIC = −9097.25null
Breakpoint at position 611. dAIC = −364.28 dAICc = −9094.09
dBIC = −9089.82null
Breakpoint at position 626. dAIC = −360.68 dAICc = −9090.49

```
dBIC =   -9086.22null
Breakpoint at position    630. dAIC =     -361.90 dAICc =   -9091.70
dBIC =   -9087.44null
Breakpoint at position    631. dAIC =     -360.68 dAICc =   -9090.48
dBIC =   -9086.22null
Breakpoint at position    632. dAIC =     -360.70 dAICc =   -9090.50
dBIC =   -9086.24null
Breakpoint at position    633. dAIC =     -358.47 dAICc =   -9088.27
dBIC =   -9084.01null
Breakpoint at position    638. dAIC =     -355.75 dAICc =   -9085.56
dBIC =   -9081.29null
Breakpoint at position    645. dAIC =     -353.64 dAICc =   -9083.45
dBIC =   -9079.19null
Breakpoint at position    647. dAIC =     -337.49 dAICc =   -9067.29
dBIC =   -9063.03null
Breakpoint at position    653. dAIC =     -333.03 dAICc =   -9062.83
dBIC =   -9058.57null
Breakpoint at position    654. dAIC =     -300.11 dAICc =   -9029.92
dBIC =   -9025.65null
Breakpoint at position    655. dAIC =     -303.58 dAICc =   -9033.38
dBIC =   -9029.12null
Breakpoint at position    659. dAIC =     -327.40 dAICc =   -9057.20
dBIC =   -9052.94null
Breakpoint at position    669. dAIC =     -327.15 dAICc =   -9056.95
dBIC =   -9052.69null
Breakpoint at position    673. dAIC =     -328.52 dAICc =   -9058.33
dBIC =   -9054.06null
Breakpoint at position    674. dAIC =     -327.57 dAICc =   -9057.38
dBIC =   -9053.11null
Breakpoint at position    675. dAIC =     -330.43 dAICc =   -9060.23
dBIC =   -9055.97null
Breakpoint at position    676. dAIC =     -329.41 dAICc =   -9059.21
dBIC =   -9054.95null
Breakpoint at position    677. dAIC =     -325.71 dAICc =   -9055.51
dBIC =   -9051.25null
Breakpoint at position    680. dAIC =     -327.14 dAICc =   -9056.94
dBIC =   -9052.68null
Breakpoint at position    686. dAIC =     -327.62 dAICc =   -9057.43
dBIC =   -9053.16null
Breakpoint at position    692. dAIC =     -327.15 dAICc =   -9056.95
dBIC =   -9052.69null
Breakpoint at position    694. dAIC =     -325.17 dAICc =   -9054.98
dBIC =   -9050.71null
Breakpoint at position    696. dAIC =     -321.47 dAICc =   -9051.27
dBIC =   -9047.01null
Breakpoint at position    698. dAIC =     -307.27 dAICc =   -9037.07
dBIC =   -9032.81null
Breakpoint at position    701. dAIC =     -303.52 dAICc =   -9033.32
dBIC =   -9029.06null
Breakpoint at position    704. dAIC =     -302.48 dAICc =   -9032.28
dBIC =   -9028.02null
Breakpoint at position    719. dAIC =     -302.26 dAICc =   -9032.07
dBIC =   -9027.80null
```

Breakpoint at position 722. dAIC = −298.61 dAICc = −9028.41
dBIC = −9024.15null
Breakpoint at position 728. dAIC = −296.96 dAICc = −9026.76
dBIC = −9022.50null
Breakpoint at position 729. dAIC = −296.30 dAICc = −9026.11
dBIC = −9021.84null
Breakpoint at position 731. dAIC = −316.51 dAICc = −9046.32
dBIC = −9042.05null
Breakpoint at position 733. dAIC = −315.78 dAICc = −9045.59
dBIC = −9041.32null
Breakpoint at position 734. dAIC = −315.15 dAICc = −9044.96
dBIC = −9040.69null
Breakpoint at position 739. dAIC = −310.47 dAICc = −9040.27
dBIC = −9036.01null
Breakpoint at position 743. dAIC = −309.70 dAICc = −9039.51
dBIC = −9035.24null
Breakpoint at position 749. dAIC = −311.58 dAICc = −9041.39
dBIC = −9037.12null
Breakpoint at position 755. dAIC = −305.76 dAICc = −9035.56
dBIC = −9031.30null
Breakpoint at position 759. dAIC = −288.88 dAICc = −9018.68
dBIC = −9014.42null
Breakpoint at position 761. dAIC = −291.09 dAICc = −9020.89
dBIC = −9016.63null
Breakpoint at position 764. dAIC = −293.29 dAICc = −9023.09
dBIC = −9018.83null
Breakpoint at position 766. dAIC = −285.11 dAICc = −9014.92
dBIC = −9010.65null
Breakpoint at position 767. dAIC = −284.65 dAICc = −9014.45
dBIC = −9010.19null
Breakpoint at position 793. dAIC = −278.73 dAICc = −9008.54
dBIC = −9004.27null
Breakpoint at position 794. dAIC = −275.60 dAICc = −9005.41
dBIC = −9001.14null
Breakpoint at position 799. dAIC = −270.46 dAICc = −9000.27
dBIC = −8996.00null
Breakpoint at position 800. dAIC = −270.14 dAICc = −8999.94
dBIC = −8995.68null
Breakpoint at position 804. dAIC = −266.91 dAICc = −8996.72
dBIC = −8992.45null
Breakpoint at position 806. dAIC = −266.57 dAICc = −8996.38
dBIC = −8992.11null
Breakpoint at position 807. dAIC = −267.33 dAICc = −8997.14
dBIC = −8992.87null
Breakpoint at position 808. dAIC = −265.97 dAICc = −8995.77
dBIC = −8991.51null
Breakpoint at position 811. dAIC = −266.19 dAICc = −8995.99
dBIC = −8991.73null
Breakpoint at position 818. dAIC = −262.99 dAICc = −8992.79
dBIC = −8988.53null
Breakpoint at position 820. dAIC = −262.51 dAICc = −8992.32
dBIC = −8988.05null
Breakpoint at position 824. dAIC = −259.46 dAICc = −8989.27

```
dBIC =   -8985.00null
Breakpoint at position    839. dAIC =     -253.88 dAICc =   -8983.68
dBIC =   -8979.42null
Breakpoint at position    843. dAIC =     -255.37 dAICc =   -8985.17
dBIC =   -8980.91null
Breakpoint at position    851. dAIC =     -257.74 dAICc =   -8987.54
dBIC =   -8983.28null
Breakpoint at position    852. dAIC =     -253.15 dAICc =   -8982.95
dBIC =   -8978.69null
Breakpoint at position    854. dAIC =     -252.99 dAICc =   -8982.79
dBIC =   -8978.53null
Breakpoint at position    860. dAIC =     -250.54 dAICc =   -8980.35
dBIC =   -8976.08null
Breakpoint at position    870. dAIC =     -250.70 dAICc =   -8980.51
dBIC =   -8976.24null
Breakpoint at position    872. dAIC =     -250.68 dAICc =   -8980.48
dBIC =   -8976.22null
Breakpoint at position    881. dAIC =     -249.36 dAICc =   -8979.17
dBIC =   -8974.90null
Breakpoint at position    884. dAIC =     -246.55 dAICc =   -8976.36
dBIC =   -8972.09null
Breakpoint at position    887. dAIC =     -247.57 dAICc =   -8977.38
dBIC =   -8973.11null
Breakpoint at position    888. dAIC =     -213.14 dAICc =   -8942.94
dBIC =   -8938.68null
Breakpoint at position    889. dAIC =     -213.12 dAICc =   -8942.93
dBIC =   -8938.66null
Breakpoint at position    890. dAIC =     -216.01 dAICc =   -8945.81
dBIC =   -8941.55null
Breakpoint at position    896. dAIC =     -216.04 dAICc =   -8945.85
dBIC =   -8941.58null
Breakpoint at position    897. dAIC =     -218.93 dAICc =   -8948.73
dBIC =   -8944.47null
Breakpoint at position    898. dAIC =     -243.97 dAICc =   -8973.78
dBIC =   -8969.51null
Breakpoint at position    899. dAIC =     -243.14 dAICc =   -8972.95
dBIC =   -8968.68null
Breakpoint at position    901. dAIC =     -241.78 dAICc =   -8971.58
dBIC =   -8967.32null
Breakpoint at position    911. dAIC =     -243.52 dAICc =   -8973.32
dBIC =   -8969.06null
Breakpoint at position    912. dAIC =     -216.61 dAICc =   -8946.41
dBIC =   -8942.15null
Breakpoint at position    920. dAIC =     -217.25 dAICc =   -8947.06
dBIC =   -8942.79null
Breakpoint at position    925. dAIC =     -219.36 dAICc =   -8949.16
dBIC =   -8944.90null
Breakpoint at position    926. dAIC =     -222.46 dAICc =   -8952.27
dBIC =   -8948.00null
Breakpoint at position    931. dAIC =     -221.29 dAICc =   -8951.10
dBIC =   -8946.83null
Breakpoint at position    937. dAIC =     -221.37 dAICc =   -8951.17
dBIC =   -8946.91null
```

```
Breakpoint at position    941. dAIC =     −221.92 dAICc =   −8951.72
dBIC =   −8947.46null
Breakpoint at position    944. dAIC =     −221.71 dAICc =   −8951.51
dBIC =   −8947.25null
Breakpoint at position    945. dAIC =     −209.49 dAICc =   −8939.29
dBIC =   −8935.03null
Breakpoint at position    947. dAIC =     −228.82 dAICc =   −8958.63
dBIC =   −8954.36null
Breakpoint at position    962. dAIC =     −234.46 dAICc =   −8964.26
dBIC =   −8960.00null
Breakpoint at position    963. dAIC =     −232.21 dAICc =   −8962.01
dBIC =   −8957.75null
Breakpoint at position    969. dAIC =     −236.19 dAICc =   −8965.99
dBIC =   −8961.73null
Breakpoint at position    970. dAIC =     −208.60 dAICc =   −8938.41
dBIC =   −8934.14null
Breakpoint at position    971. dAIC =     −207.55 dAICc =   −8937.35
dBIC =   −8933.09null
Breakpoint at position    974. dAIC =     −215.33 dAICc =   −8945.14
dBIC =   −8940.87null
Breakpoint at position    989. dAIC =     −217.04 dAICc =   −8946.85
dBIC =   −8942.58null
Breakpoint at position    995. dAIC =     −217.08 dAICc =   −8946.88
dBIC =   −8942.62null
Breakpoint at position   1004. dAIC =     −219.07 dAICc =   −8948.88
dBIC =   −8944.61null
Breakpoint at position   1010. dAIC =     −221.24 dAICc =   −8951.04
dBIC =   −8946.78null
Breakpoint at position   1019. dAIC =     −224.26 dAICc =   −8954.06
dBIC =   −8949.80null
Breakpoint at position   1025. dAIC =     −223.30 dAICc =   −8953.10
dBIC =   −8948.84null
Breakpoint at position   1037. dAIC =     −224.73 dAICc =   −8954.53
dBIC =   −8950.27null
Breakpoint at position   1038. dAIC =     −226.45 dAICc =   −8956.26
dBIC =   −8951.99null
Breakpoint at position   1040. dAIC =     −227.44 dAICc =   −8957.25
dBIC =   −8952.98null
Breakpoint at position   1046. dAIC =     −225.87 dAICc =   −8955.67
dBIC =   −8951.41null
Breakpoint at position   1052. dAIC =     −223.56 dAICc =   −8953.36
dBIC =   −8949.10null
Breakpoint at position   1061. dAIC =     −224.97 dAICc =   −8954.78
dBIC =   −8950.51null
Breakpoint at position   1082. dAIC =     −227.09 dAICc =   −8956.89
dBIC =   −8952.63null
Breakpoint at position   1088. dAIC =     −225.67 dAICc =   −8955.47
dBIC =   −8951.21null
Breakpoint at position   1091. dAIC =     −253.13 dAICc =   −8982.94
dBIC =   −8978.67null
Breakpoint at position   1097. dAIC =     −253.11 dAICc =   −8982.92
dBIC =   −8978.65null
Breakpoint at position   1098. dAIC =     −254.35 dAICc =   −8984.15
```

```
dBIC =   -8979.89null
Breakpoint at position   1122. dAIC =      -349.96 dAICc =   -9079.77
dBIC =   -9075.50null
Breakpoint at position   1124. dAIC =      -352.61 dAICc =   -9082.42
dBIC =   -9078.15null
Breakpoint at position   1127. dAIC =      -354.10 dAICc =   -9083.90
dBIC =   -9079.64null
Breakpoint at position   1128. dAIC =      -355.46 dAICc =   -9085.26
dBIC =   -9081.00null
Breakpoint at position   1129. dAIC =      -356.87 dAICc =   -9086.67
dBIC =   -9082.41null
Breakpoint at position   1145. dAIC =      -357.87 dAICc =   -9087.67
dBIC =   -9083.41null
Breakpoint at position   1155. dAIC =      -392.74 dAICc =   -9122.54
dBIC =   -9118.28null
Breakpoint at position   1156. dAIC =      -389.74 dAICc =   -9119.54
dBIC =   -9115.28null
Breakpoint at position   1160. dAIC =      -394.54 dAICc =   -9124.34
dBIC =   -9120.08null
Breakpoint at position   1166. dAIC =      -391.74 dAICc =   -9121.55
dBIC =   -9117.28null
Breakpoint at position   1181. dAIC =      -394.14 dAICc =   -9123.94
dBIC =   -9119.68null
Breakpoint at position   1187. dAIC =      -390.55 dAICc =   -9120.36
dBIC =   -9116.09null
Breakpoint at position   1189. dAIC =      -392.79 dAICc =   -9122.60
dBIC =   -9118.33null
Breakpoint at position   1193. dAIC =      -391.81 dAICc =   -9121.62
dBIC =   -9117.35null
Breakpoint at position   1215. dAIC =      -394.34 dAICc =   -9124.15
dBIC =   -9119.88null
Breakpoint at position   1220. dAIC =      -393.50 dAICc =   -9123.31
dBIC =   -9119.04null
Breakpoint at position   1226. dAIC =      -395.45 dAICc =   -9125.25
dBIC =   -9120.99null
Breakpoint at position   1235. dAIC =      -397.49 dAICc =   -9127.29
dBIC =   -9123.03null
Breakpoint at position   1236. dAIC =      -399.40 dAICc =   -9129.20
dBIC =   -9124.94null
Breakpoint at position   1238. dAIC =      -401.33 dAICc =   -9131.13
dBIC =   -9126.87null
Breakpoint at position   1244. dAIC =      -403.36 dAICc =   -9133.16
dBIC =   -9128.90null
Breakpoint at position   1247. dAIC =      -417.14 dAICc =   -9146.94
dBIC =   -9142.68null
Breakpoint at position   1250. dAIC =      -418.41 dAICc =   -9148.22
dBIC =   -9143.95null
Breakpoint at position   1252. dAIC =      -425.92 dAICc =   -9155.72
dBIC =   -9151.46null
Breakpoint at position   1253. dAIC =      -427.95 dAICc =   -9157.75
dBIC =   -9153.49null
Breakpoint at position   1264. dAIC =      -426.72 dAICc =   -9156.53
dBIC =   -9152.26null
```

```
Breakpoint at position   1274. dAIC =     −405.97 dAICc =   −9135.78
dBIC =   −9131.51null
Breakpoint at position   1275. dAIC =     −537.95 dAICc =   −9267.75
dBIC =   −9263.49null
Breakpoint at position   1277. dAIC =     −460.71 dAICc =   −9190.52
dBIC =   −9186.25null
Breakpoint at position   1284. dAIC =     −463.80 dAICc =   −9193.60
dBIC =   −9189.34null
Breakpoint at position   1286. dAIC =     −467.24 dAICc =   −9197.05
dBIC =   −9192.78null
Breakpoint at position   1289. dAIC =     −512.60 dAICc =   −9242.41
dBIC =   −9238.14null
Breakpoint at position   1295. dAIC =     −511.86 dAICc =   −9241.67
dBIC =   −9237.40null
Breakpoint at position   1297. dAIC =     −513.90 dAICc =   −9243.71
dBIC =   −9239.44null
Breakpoint at position   1302. dAIC =     −516.27 dAICc =   −9246.07
dBIC =   −9241.81null
Breakpoint at position   1309. dAIC =     −516.89 dAICc =   −9246.70
dBIC =   −9242.43null
Breakpoint at position   1313. dAIC =     −522.68 dAICc =   −9252.49
dBIC =   −9248.22null
Breakpoint at position   1317. dAIC =     −565.50 dAICc =   −9295.31
dBIC =   −9291.04null
Breakpoint at position   1325. dAIC =     −566.58 dAICc =   −9296.38
dBIC =   −9292.12null
Breakpoint at position   1334. dAIC =     −584.30 dAICc =   −9314.11
dBIC =   −9309.84null
Breakpoint at position   1337. dAIC =     −587.24 dAICc =   −9317.05
dBIC =   −9312.78null
Breakpoint at position   1340. dAIC =     −590.50 dAICc =   −9320.30
dBIC =   −9316.04null
Breakpoint at position   1349. dAIC =     −594.43 dAICc =   −9324.23
dBIC =   −9319.97null
Breakpoint at position   1353. dAIC =     −594.94 dAICc =   −9324.75
dBIC =   −9320.48null
Breakpoint at position   1361. dAIC =     −598.92 dAICc =   −9328.73
dBIC =   −9324.46null
Breakpoint at position   1362. dAIC =     −604.50 dAICc =   −9334.31
dBIC =   −9330.04null
Breakpoint at position   1373. dAIC =     −609.08 dAICc =   −9338.88
dBIC =   −9334.62null
Breakpoint at position   1379. dAIC =     −615.59 dAICc =   −9345.39
dBIC =   −9341.13null
Breakpoint at position   1386. dAIC =     −618.56 dAICc =   −9348.37
dBIC =   −9344.10null
Breakpoint at position   1403. dAIC =     −624.25 dAICc =   −9354.05
dBIC =   −9349.79null
Breakpoint at position   1406. dAIC =     −627.58 dAICc =   −9357.38
dBIC =   −9353.12null
Breakpoint at position   1427. dAIC =     −633.91 dAICc =   −9363.71
dBIC =   −9359.45null
Breakpoint at position   1445. dAIC =     −639.43 dAICc =   −9369.24
```

```
dBIC =   -9364.97null
Breakpoint at position   1451. dAIC =     -643.42 dAICc =   -9373.22
dBIC =   -9368.96null
Breakpoint at position   1466. dAIC =     -645.07 dAICc =   -9374.88
dBIC =   -9370.61null
Breakpoint at position   1472. dAIC =     -649.79 dAICc =   -9379.60
dBIC =   -9375.33null
Breakpoint at position   1481. dAIC =     -668.75 dAICc =   -9398.55
dBIC =   -9394.29null
Breakpoint at position   1484. dAIC =     -672.63 dAICc =   -9402.43
dBIC =   -9398.17null
Breakpoint at position   1485. dAIC =     -672.55 dAICc =   -9402.35
dBIC =   -9398.09null
Breakpoint at position   1486. dAIC =     -673.56 dAICc =   -9403.36
dBIC =   -9399.10null
Breakpoint at position   1499. dAIC =     -675.77 dAICc =   -9405.58
dBIC =   -9401.31null
Breakpoint at position   1502. dAIC =     -678.24 dAICc =   -9408.04
dBIC =   -9403.78null
Breakpoint at position   1503. dAIC =     -679.20 dAICc =   -9409.00
dBIC =   -9404.74null
Breakpoint at position   1505. dAIC =     -677.09 dAICc =   -9406.89
dBIC =   -9402.63null
Breakpoint at position   1508. dAIC =     -746.13 dAICc =   -9475.93
dBIC =   -9471.67null
Breakpoint at position   1511. dAIC =     -750.34 dAICc =   -9480.14
dBIC =   -9475.88null
Breakpoint at position   1520. dAIC =     -794.47 dAICc =   -9524.27
dBIC =   -9520.01null
Breakpoint at position   1526. dAIC =     -796.45 dAICc =   -9526.26
dBIC =   -9521.99null
Breakpoint at position   1545. dAIC =     -815.21 dAICc =   -9545.02
dBIC =   -9540.75null
Breakpoint at position   1565. dAIC =     -839.04 dAICc =   -9568.85
dBIC =   -9564.59null
Breakpoint at position   1568. dAIC =     -843.73 dAICc =   -9573.53
dBIC =   -9569.27null
Breakpoint at position   1580. dAIC =     -843.71 dAICc =   -9573.51
dBIC =   -9569.25null
Breakpoint at position   1595. dAIC =     -862.12 dAICc =   -9591.93
dBIC =   -9587.66null
Breakpoint at position   1599. dAIC =     -864.33 dAICc =   -9594.13
dBIC =   -9589.87null
Breakpoint at position   1601. dAIC =     -865.48 dAICc =   -9595.28
dBIC =   -9591.02null
Breakpoint at position   1604. dAIC =     -866.68 dAICc =   -9596.49
dBIC =   -9592.22null
Breakpoint at position   1613. dAIC =     -872.02 dAICc =   -9601.82
dBIC =   -9597.56null
Breakpoint at position   1622. dAIC =     -874.55 dAICc =   -9604.35
dBIC =   -9600.09null
Breakpoint at position   1637. dAIC =     -879.50 dAICc =   -9609.31
dBIC =   -9605.04null
```

```
Breakpoint at position   1640. dAIC =     -884.89 dAICc =   -9614.70
dBIC =   -9610.43null
Breakpoint at position   1646. dAIC =     -892.14 dAICc =   -9621.94
dBIC =   -9617.68null
Breakpoint at position   1655. dAIC =     -910.22 dAICc =   -9640.02
dBIC =   -9635.76null
Breakpoint at position   1658. dAIC =     -984.05 dAICc =   -9713.86
dBIC =   -9709.59null
Breakpoint at position   1662. dAIC =     -989.80 dAICc =   -9719.60
dBIC =   -9715.34null
Breakpoint at position   1664. dAIC =     -993.05 dAICc =   -9722.85
dBIC =   -9718.59null
Breakpoint at position   1669. dAIC =     -995.07 dAICc =   -9724.87
dBIC =   -9720.61null
Breakpoint at position   1679. dAIC =    -1000.48 dAICc =   -9730.28
dBIC =   -9726.02null
Breakpoint at position   1683. dAIC =    -1013.06 dAICc =   -9742.86
dBIC =   -9738.60null
Breakpoint at position   1690. dAIC =    -1016.60 dAICc =   -9746.41
dBIC =   -9742.14null
Breakpoint at position   1694. dAIC =    -1018.20 dAICc =   -9748.00
dBIC =   -9743.74null
Breakpoint at position   1702. dAIC =    -1038.53 dAICc =   -9768.33
dBIC =   -9764.07null
Breakpoint at position   1707. dAIC =    -1035.25 dAICc =   -9765.05
dBIC =   -9760.79null
Breakpoint at position   1708. dAIC =    -1048.26 dAICc =   -9778.06
dBIC =   -9773.80null
Breakpoint at position   1709. dAIC =    -1114.97 dAICc =   -9844.78
dBIC =   -9840.51null
Breakpoint at position   1715. dAIC =    -1200.88 dAICc =   -9930.69
dBIC =   -9926.42null
Breakpoint at position   1717. dAIC =    -1251.57 dAICc =   -9981.38
dBIC =   -9977.11null
Breakpoint at position   1718. dAIC =    -1260.22 dAICc =   -9990.02
dBIC =   -9985.76null
Breakpoint at position   1721. dAIC =    -1292.72 dAICc =  -10022.53
dBIC =  -10018.26null
Breakpoint at position   1723. dAIC =    -1320.55 dAICc =  -10050.35
dBIC =  -10046.09null
Breakpoint at position   1739. dAIC =    -1330.36 dAICc =  -10060.17
dBIC =  -10055.90null
Breakpoint at position   1741. dAIC =    -1346.04 dAICc =  -10075.85
dBIC =  -10071.58null
AIC

There seems to be NO recombination in this alignment


AIC-c

There seems to be NO recombination in this alignment
```

BIC

There seems to be NO recombination in this alignment
