# Supplementary material for: Evidence of CPV2c introgression into Croatia and novel insights into phylogeny and cell tropism
Source: Sci Rep. 2019 Nov 15;9:16909. doi: 10.1038/s41598-019-53422-9 (PMC6858334; doi:10.1038/s41598-019-53422-9)
Supplement: Supplementary file 2 — Mixed Effects Model of Evolution [file 41598_2019_53422_MOESM2_ESM.pdf]

Evidence of CPV2c introgression into Croatia and novel insights into phylogeny and cell tropism

Dinko Novosel, Tamas Tuboly, Gyula Balka, Levente Szeredi, Ivana Lojkic, Andreja Jungic, Zaklin Acinger Rogic, Tahar Ait Ali, Attila Csagola

Supplementary info file 2.

Results of selection using Mixed Effects Model of Evolution method

```
   /HYPHY 2.3.13.20180601beta(MPI) for Darwin on x86_64\
***************** TYPES OF STANDARD ANALYSES *****************


    (1) Selection Analyses
    (2) Evolutionary Hypothesis Testing
    (3) Relative evolutionary rate inference
    (4) Basic Analyses
    (5) Codon Selection Analyses
    (6) Compartmentalization
    (7) Data File Tools
    (8) Miscellaneous
    (9) Model Comparison
    (10) Kernel Analysis Tools
    (11) Molecular Clock
    (12) Phylogeny Reconstruction
    (13) Positive Selection
    (14) Recombination
    (15) Selection/Recombination
    (16) Relative Rate
    (17) Relative Ratio
    (18) Substitution Rates

1
 Please select type of analyses you want to list (or press ENTER to
process custom batch file):


***************** FILES IN 'Selection Analyses' *****************


    (1) [MEME] Test for episodic site-level selection using MEME
```

```
(Mixed Effects Model of Evolution).
    (2) [FEL] Test for pervasive site-level selection using FEL
(Fixed Effects Likelihood).
    (3) [SLAC] Test for pervasive site-level selection using SLAC
(Single Likelihood Ancestor Counting).
    (4) [FUBAR] Test for pervasive site-level selection using FUBAR
(Fast Unconstrained Bayesian AppRoximation for inferring selection).
    (5) [BUSTED] Test for episodic gene-wide selection using BUSTED
(Branch-site Unrestricted Statistical Test of Episodic
Diversification).
    (6) [aBSREL] Test for lineage-specific evolution using the
branch-site method aBS-REL (Adaptive Branch-Site Random Effects
Likelihood).
    (7) [RELAX] Test for relaxation of selection pressure along a
specified set of test branches using RELAX (a random effects test of
selection relaxation).

 Please select the analysis you would like to perform (or press
ENTER to return to the list of analysis types):1

Analysis Description
--------------------
MEME (Mixed Effects Model of Evolution) estimates a site-wise
synonymous
(&alpha;) and a two-category mixture of non-synonymous (&beta;-,
with
proportion p-, and &beta;+ with proportion [1-p-]) rates, and uses a
likelihood ratio test to determine if &beta;+ > &alpha; at a site.
The
estimates aggregate information over a proportion of branches at a
site,
so the signal is derived from episodic diversification, which is a
combination of strength of selection [effect size] and the
proportion of
the tree affected. A subset of branches can be selected for testing
as
well, in which case an additional (nuisance) parameter will be
inferred
-- the non-synonymous rate on branches NOT selected for testing.
Multiple partitions within a NEXUS file are also supported for
recombination - aware analysis.

- __Requirements__: in-frame codon alignment and a phylogenetic tree

- __Citation__: Detecting Individual Sites Subject to Episodic
Diversifying Selection.
_PLoS Genet_ 8(7): e1002764.

- __Written by__: Sergei L. Kosakovsky Pond, Steven Weaver

- __Contact Information__: spond@temple.edu

- __Analysis Version__: 2.0.1
```

####Choose Genetic Code

1. [**Universal**] Universal code. (Genebank transl_table=1).
2. [**Vertebrate mtDNA**] Vertebrate mitochondrial DNA code. (Genebank transl_table=2).
3. [**Yeast mtDNA**] Yeast mitochondrial DNA code. (Genebank transl_table=3).
4. [**Mold/Protozoan mtDNA**] Mold, Protozoan and Coelenterate mitochondrial DNA and the Mycloplasma/Spiroplasma code. (Genebank transl_table=4).
5. [**Invertebrate mtDNA**] Invertebrate mitochondrial DNA code. (Genebank transl_table=5).
6. [**Ciliate Nuclear**] Ciliate, Dasycladacean and Hexamita Nuclear code. (Genebank transl_table=6).
7. [**Echinoderm mtDNA**] Echinoderm mitochondrial DNA code. (Genebank transl_table=9).
8. [**Euplotid Nuclear**] Euplotid Nuclear code. (Genebank transl_table=10).
9. [**Alt. Yeast Nuclear**] Alternative Yeast Nuclear code. (Genebank transl_table=12).
10. [**Ascidian mtDNA**] Ascidian mitochondrial DNA code. (Genebank transl_table=13).
11. [**Flatworm mtDNA**] Flatworm mitochondrial DNA code. (Genebank transl_table=14).
12. [**Blepharisma Nuclear**] Blepharisma Nuclear code. (Genebank transl_table=15).
13. [**Chlorophycean mtDNA**] Chlorophycean Mitochondrial Code (transl_table=16).
14. [**Trematode mtDNA**] Trematode Mitochondrial Code (transl_table=21).
15. [**Scenedesmus obliquus mtDNA**] Scenedesmus obliquus mitochondrial Code (transl_table=22).
16. [**Thraustochytrium mtDNA**] Thraustochytrium Mitochondrial Code (transl_table=23).
17. [**Pterobranchia mtDNA**] Pterobranchia Mitochondrial Code (transl_table=24).
18. [**SR1 and Gracilibacteria**] Candidate Division SR1 and Gracilibacteria Code (transl_table=25).
19. [**Pachysolen Nuclear**] Pachysolen tannophilus Nuclear Code (transl_table=26).

>Please choose an option (or press q to cancel selection):1

>Select a coding sequence alignment file (`/Users/dinkonovosel/hyphy/res/TemplateBatchFiles/SelectionAnalyses/`) /Users/dinkonovosel/CPV_VP2_cod_sel.txt

>A tree was found in the data file:
`((ITA/FJ005218/2c/330/2006,ITA/FJ005233/40/2007),POR/KT275253/2c/

PT036/12/2012),(URU/KC196096/2c/M247/2010,URU/KM457121/2c/
UY247/2010)),(URU/KC196086/2c/M55/2006,URU/KM457106/2c/
UY55/2006)),USA/JX475260/CO/704/2010),ITA/FJ005247/195/2008),ITA/
FJ005226/383/2006),GER/FJ005196/2c/G7/1997),FRA/
DQ025994/04S25/2004),FRA/DQ025960/03C4/2003),FRA/
DQ025951/03B10/2003),FRA/DQ025954/03B14/2003),USA/KJ813848/Bobcat/
ND/1162/2013),URU/KM457104/2c/UY47/2006),FRA/
DQ025969/03S5/2003),USA/KJ813858/Puma/ND/F93/2013),ITA/
FJ005248/219/2008),GER/FJ005199/2c/G172/1997),ITA/
FJ005240/208/2007),URU/KC196085/2c/M57/2007),FRA/
DQ025975/04S6/2004),USA/JX475243/ID/22772/2009),USA/JX475252/CO/
1316/2010),FRA/DQ025965/03C9/2003),ECU/KF149984/2c/ME28/2012),(ECU/
KF149962/2c/ME1/2012,(ECU/KF149963/2c/ME10/2012,(ECU/KF149964/2c/
ME23/2012,ECU/KF149969/2c/ME31/2012)))),(ARG/JF414820/Arg44/2009,
(ARG/KM236569/Cuba/2013,(ARG/JF414818/Arg32/2008,ARG/JF414821/
Arg48/2009)))),(ITA/FJ005216/2c/284/2006,(ITA/KU508407/2c/
25835/09/2009,ITA/KX434459/27692/1/11/2011))),(((URU/KM457122/2c/
UY258/2010,URU/KM457124/2c/UY307/2011),URU/KC196093/2c/M307/2011),
((URU/KC196081/2c/M95/2007,URU/KM457109/2c/UY95/2007),(URU/
KC196097/2c/M242/2010,(URU/KM457120/2c/UY242/2010,URU/KM457123/2c/
UY261/2008))))),(URU/KM457103/2c/UY12/2006,(ITA/FJ005209/2c/
303/2004,ITA/FJ005251/239/2008))),(URU/KC196083/2c/M82/2007,URU/
KM457108/2c/UY82/2007)),URU/KC196107/2c/M129/2008),URU/KM457131/2c/
UY368/2011),POR/KT275252/2c/PT013/12/2012),ITA/FJ005231/406/2006),
(ITA/FJ005214/2c/67/2006,BRA/KY073269/UFMT/2015)),ARG/JF414819/
Arg35/2008),USA/KJ813854/Puma/ND/F205/2013),ITA/FJ005232/411/2006),
(((URU/KC196105/2c/M152/2008,URU/KM457113/2c/UY152/2009),ITA/
FJ005212/2c/349/2004),(POR/KT275255/2c/PT238/14/2014,(ITA/
KX434460/52238/12/2012,((URU/KC196091/2c/M326/2011,URU/KM457127/2c/
UY326/2011),((((((((((URU/KC196102/2c/M185/2009,URU/KM457116/2c/
UY185/2009),(HRV/KP859577/2c/HR856/2014,ITA/KX434458/2323/11/2011)),
(URU/KC196101/2c/M187/2009,URU/KM457117/2c/UY187/2009)),AUS/
KU508693/2c/LW/2015),(URU/KC196089/2c/M349/2011,URU/KM457129/2c/
UY349/2011)),ITA/FJ005195/2c/136/2000),FRA/DQ025976/04S7/2004),(USA/
JX475273/MT/909/2012,USA/KJ813888/Coyote/MT/878/2012)),USA/KJ813843/
Bobcat/ND/1160/2013),(FRA/DQ025942/01B1/2001,(FRA/
DQ025964/03C8/2003,(ITA/FJ005206/2c/287/2004,(URU/KM457107/2c/
UY72/2007,(URU/KM457111/2c/UY120/2008,(URU/KM457112/2c/UY135/2008,
(URU/KM457125/2c/UY317/2011,(URU/KM457130/2c/UY354/2011,(URU/
KM457142/2c/UY370/2011,(HRV/KP859574/2c/HR442/2014,(HRV/KP859575/2c/
HR774/2014,(HRV/KP859576/2c/HR793/2014,(AUS/KU508691/2c/HB/2015,
(AUS/KU508692/2c/FH/2015,ITA/
KX434456/45361/09/2009)))))))))))))))))))),URU/KM457126/2c/
UY318/2010),(FRA/DQ025985/04S16/2004,(ITA/FJ005205/2c/279/2004,HRV/
KP859578/2c/HR859/2014))),ITA/FJ222821/2c/56/2000),(GER/FJ005260/
G82/1997,USA/KJ813846/Bobcat/ND/974/2013)),((GER/
AY742934/447/1995,RUS/JN033694/Laika/1993),(USA/AY742936/395/1998,
(USA/JX475240/AZ/16382-01/1999,(USA/JX475250/CO/728/2010,USA/
KJ813842/Bobcat/ND/502/2013))))),((((((((((((((((((USA/KJ813828/
Fisher/F1F010712/2013,USA/KJ813881/Graywolf/MI/832/2012),USA/
KJ813844/Bobcat/ND/885/2013),USA/KJ813882/Raccoon/NJ/1423/2012),USA/
KJ813851/Bobcat/ND/1168/2013),USA/JX475278/AR/1069/2012),KOR/
EU009205/2b/K029/2006),USA/JX475247/CO/1246/2010),(USA/KJ813892/
Coyote/AK/218/2013,(USA/JN867604/Dog/IL/137654/2008,USA/JX475242/WI/

18268/2002))),SAF/HQ602969/22/10SA/2010),(USA/AY742955/436/2003,FRA/
DQ025991/2b/04S22/2004)),USA/JN867602/2b/Dog/CA/148743/2008),USA/
JX475251/CO/2235/2009),USA/JN867603/2b/Dog/KS/81213/2009),USA/
KJ813852/Bobcat/ND/1170/2013),FRA/DQ025961/2b/03C5/2003),(USA/
KJ813827/Fisher/F1M111211/2013,USA/KJ813873/Graywolf/MI/850/2012)),
(ECU/KF149971/2c/ME32/2012,IND/KX469432/newCPV/2b/Hiller/2011)),
((ITA/FJ005263/42/2005,ITA/FJ005265/140/2005),(((((((((USA/
M74849/39/1995,USA/U22896/cat/1990),FRA/DQ025992/2b/04S23/2004),USA/
M74852/133/1995),POR/KU662349/greywolf/W33/1996),(POR/KU662350/
greywolf/W52/2005,POL/Z46651/46/1994)),GER/FJ005261/G162/1997),BRA/
DQ340409/2b/BR183/1985),USA/AY742932/193/1991),((USA/
AY742951/431/2003,USA/JN867605/2b/Dog/US/142805/2009),(VAC/
FJ222822/2b/FortDodge/2008,(VAC/JN625223/INDIA/vac5/2011,(USA/
EU659119/2b/CPV/410/2000,USA/EU659120/2b/CPV/411a/1998))))),((CHI/
GQ857609/CPV08/01/2008,CHI/GU569940/2b/YN0203/2002),((((((JPN/
AB115504/2c/97/008/1997,TAW/U72696/2b/T10/1996),TAW/U72695/2a/
T4/1996),(CHI/GQ857596/CPV05/01/2005,CHI/GQ857600/
CPV06/01/2006)),THA/FJ869125/KU5/2004),(USA/JX475237/CT/
372/2011,KOR/EF599097/2b/DH326/2006)),(CHI/EU483515/2b/ZD13/2007,
(JPN/LC270891/2b/9985/2017,(JPN/AB437433/1887/M/2/2008,(TAW/
FJ265781/CPV307/2005,(TWN/EF592511/TWN1/2006,TAW/FJ265775/
CPV301/2004)))))),(((VIE/AB054218/2b/cat/V123/2000,VAC/FJ222823/2b/
29/1997),ITA/FJ005264/134/2005),((THA/FJ869122/KU1/2008,THA/
FJ869123/KU3/2008),(((((THA/KP715690/VT28/2014,THA/KP715716/
VT143/2014),THA/KP715691/VT43/2014),(VIE/AB120722/2b/HCM/
18/2003,VIE/AB120724/2b/HNI/2/13/2003)),(CHI/GQ857599/
CPV05/04/2005,CHI/GQ857601/CPV06/02/2006)),CHI/GQ857605/
CPV07/03/2007),(THA/FJ869139/KU66/2003,(VIE/AB120721/2b/HCM/8/2003,
(VIE/AB054221/2b/leopard/V204/2000,(VIE/AB054224/2c/leopard/
V203/2000,(VIE/AB120725/2b/HNI/3/4/2003,(VIE/AB120723/2b/HCM/
23/2003,(VIE/AB120720/2b/HCM/6/2003,(VIE/AB054219/2b/cat/V209/2000,
(VIE/AB054220/2b/cat/V217/2000,CHI/EU145954/2b/
BJ044/20O7))))))))))))))))),(ITA/FJ005257/54/2008,ITA/KF373611/2a/
409/2010)),(NZE/AY742933/339/1993,((VIE/AB054223/2c/leopard/
V140/2000,ITA/GU362932/cat11/2008),(NIG/HQ602995/15/10/2010,
(((((((((FRA/DQ025947/2a/02B5/2002,FRA/DQ026001/2a/04S32/2004),FRA/
DQ025962/2a/03C6/2003),ITA/KF373580/2a/581/2003),(GER/AY742935/
U6/1995,FRA/DQ025945/2a/02B3/2002)),VIE/AB054215/2a/cat/
V120/2000),ITA/FJ005255/333/2005),FRA/DQ025958/2a/03C2/2003),(ITA/
KX434457/987/10/2010,(FRA/DQ025983/2a/04S14/2004,FRA/DQ025993/2a/
04S24/2004))),(FRA/DQ025984/2a/04S15/2004,ITA/FJ005252/96/2002)),
(FRA/DQ026002/2a/04S33/2004,(ITA/KF373592/2a/329/2008,(((ITA/
AF393506/2a/699/2000,FRA/DQ025943/2a/01S1/2001),ITA/KF385388/2a/
Sicily/X83090/2009),((CHI/GQ857612/CPV08/04/2008,CHI/GU569939/2a/
YN0202/2002),((((HUN/KF539794/H/7/2012,HUN/KF539795/H/8/2012),HUN/
KF539804/H/212/2012),(HUN/KF539793/H/5/2012,HUN/KF539797/H/
11/2012)),(HUN/KF539800/H/27/2012,(VIE/AB054217/2a/cat/
V154/2000,HUN/KF539796/H/9/2012))),(((HUN/KF539798/H/31/2012,HUN/
KF539799/H/39/2012),HUN/KF539805/H/36/2012),(ITA/AF306447/618/2000,
(FRA/DQ025944/2a/02B2/2002,(NIG/HQ602992/19/10/2010,(ITA/
AF306446/584/2000,(FRA/DQ025986/2a/04S17/2004,(ITA/KF373577/2a/
714/2001,(FRA/DQ025982/2a/04S13/2004,ITA/
FJ005253/67/2005)))))))))))))))),(((((((THA/FJ869126/
KU5/2008,THA/FJ869137/KU52/2003),THA/FJ869134/KU23/2003),CHI/

DQ354068/2a/redpanda/RPPV/2004),KOR/EF599096/DH426/2005),(ITA/
FJ005258/80/2008,(KOR/EF599098/2c/Pome/2006,(FRA/DQ025950/2a/
02B9/2002,ITA/KX434454/29451/09/2009)))),(THA/FJ869130/KU13/2004,
(THA/FJ869138/KU53/2003,CHI/KF803615/2011/BJ/B25/2011))),(CHI/
GU569942/2a/JL0202/2002,CHI/GU569946/2a/JL0201/2002)),((USA/
AY742953/435/2003,ITA/KF373571/2a/685/1999),(THA/FJ869128/KU11/2004,
(((BRA/DQ340428/2a/BR209/1994,BRA/DQ340431/2a/BR56/1995),BRA/
DQ340411/2a/BR8/1990),(BRA/DQ340422/2a/BR22/1993,(BRA/DQ340421/2a/
BR597/1992,((((BRA/DQ340419/2a/BR570/1992,BRA/DQ340423/2a/
BR136/1993),BRA/DQ340413/2a/BR18/1990),BRA/DQ340427/2a/BR133/1994),
(BRA/DQ340414/2a/BR31/1990,(BRA/DQ340416/2a/BR47/1991,(BRA/
DQ340417/2a/BR52/1991,(BRA/DQ340418/2a/BR491/1992,(BRA/DQ340424/2a/
BR137/1993,BRA/DQ340426/2a/BR84/1994)))))))))))),CHI/KF803600/2010/
BJ/A68/2010),(((USA/EU659118/CPV/13/1981,CHI/GU569948/2a/
CC8601/1986),JPN/D26079/1993),((BRA/DQ340407/2a/BR145/1980,BRA/
DQ340408/2a/BR154/1980),(FRA/DQ025952/2a/03B12/2003,(BRA/
DQ340404/2a/BR6/1980,(BRA/DQ340405/2a/BR135/1980,(BRA/DQ340410/2a/
BR315/1986,(USA/M24000/FPV/CPV/31/1988,USA/M24003/FPV/CPV/
15/1988)))))))),((USA/JN867599/Raccoon/KY/39552/2009,USA/JN867611/
Raccoon/KY/358-B/2009),(USA/JN867610/Raccoon/VA/118-A/2007,(USA/
KJ813890/Redfox/MA/197/2012,(USA/JX475284/TN/26/2011,(USA/JX475239/
GA/06/2011,USA/JX475279/TN/1/2011)))))),(HUN/KF539801/H/25/2012,HUN/
KF539803/H/2/2012)),(USA/KJ813870/Raccoon/TX/1/2013,(((USA/JN867598/
Bobcat/KS/44/2010,USA/KJ813832/Fisher/ND/14/2013),(USA/KJ813831/
Fisher/ND/17/2013,USA/KJ813835/Fisher/ND/19/2013)),(USA/JX475234/ME/
258/2011,(USA/JN867618//Raccoon/WI/37/2010,(USA/JX475231/CO/
280/2011,(USA/JX475248/CO/1102/2011,(USA/JX475233/SC/182-A/2011,USA/
JX475246/CO/2503/2010))))))),((CHI/FJ231389/FPV/monkey/
BJ-22/2008,CHI/KJ170680/raccoondog/HLJ11/1/2011),(((((((((CHI/
GU392242/raccoondog/HB10/2009,CHI/GU392244/raccoondog/HB7/2009),CHI/
KJ170679/raccoondog/Heb10/2/2010),CHI/GU392241/raccoondog/
HB1/2009),CHI/GU392236/fox/HB1/2009),(CHI/GU392240/raccoondog/
HB3/2009,(CHI/GU392239/raccoondog/HB6/2009,CHI/KJ194463/raccoondog/
HeB10/3/2010))),CHI/GU392237/fox/HB2/2009),(VAC/FJ011098/Intervet/
2006,(VAC/JN625222/INDIA/vac4/2011,(ITA/FJ222824/388/05/3/2005,(CHI/
FJ432718/CPV/Cv/2008,(VAC/JN625219/INDIA/vac1/2011,CHI/
KF803602/2010/BJ/A72/2010)))))),JPN/AB437434/1887/f/3/2008),
((((((VAC/GU212790/primodog/2009,VAC/GU212791/vanguard/2009),VAC/
FJ197847/Pfizer/2007),VAC/EU914139/Pfizer//2006),VAC/KY083089/
Singapore/2016),USA/M19296/CPV/N/1988),(((((USA/M23255/FPV/
Cornell320/1988,USA/M38245/1990),USA/EU659116/CPV/5/1979),(FIN/
U22192/raccoondog/RD-80/1980,FIN/U22193/raccoondog/RD87/1987)),(USA/
M10989/1985,USA/U22186/CPV/128/1995)),(VAC/JN625221/INDIA/vac3/2011,
(VAC/JN625220/INDIA/vac2/2011,(((VAC/FJ011097/Merial/2006,CHI/
GQ169553/Vac2/2007),VAC/KY083090/Singapore/2016),(CHI/GU569943/
YB8301/1983,(VAC/JN625224/INDIA/vac6/2011,ARG/KM236572/NNGag/
2012))))))))))`

>Would you like to use it (y/n)? y

>Loaded a multiple sequence alignment with **339** sequences,
581 codons, and **1** partitions from `/Users/dinkonovosel/
CPV_VP2_cod_sel.txt`

####Choose the set of branches to test for selection

1. [**All**] Include all branches in the analysis
2. [**Internal**] Include all internal branches in the analysis
3. [**Leaves**] Include all leaf branches in the analysis
4. [**Unlabeled branches**] Set of 675 unlabeled branches

>Please choose an option (or press q to cancel selection):1

>Select the p-value threshold to use when testing for selection
(permissible range = [0,1], default value = 0.1): 0.1

Branches to include in the MEME analysis
Selected 675 branches to include in the MEME analysis:
`ITA_FJ005218_2c_330_2006, ITA_FJ005233_40_2007, Node53,
POR_KT275253_2c_PT036_12_2012, Node52, URU_KC196096_2c_M247_2010,
URU_KM457121_2c_UY247_2010, Node57, Node51,
URU_KC196086_2c_M55_2006, URU_KM457106_2c_UY55_2006, Node60, Node50,
USA_JX475260_CO_704_2010, Node49, ITA_FJ005247_195_2008, Node48,
ITA_FJ005226_383_2006, Node47, GER_FJ005196_2c_G7_1997, Node46,
FRA_DQ025994_04S25_2004, Node45, FRA_DQ025960_03C4_2003, Node44,
FRA_DQ025951_03B10_2003, Node43, FRA_DQ025954_03B14_2003, Node42,
USA_KJ813848_Bobcat_ND_1162_2013, Node41, URU_KM457104_2c_UY47_2006,
Node40, FRA_DQ025969_03S5_2003, Node39,
USA_KJ813858_Puma_ND_F93_2013, Node38, ITA_FJ005248_219_2008,
Node37, GER_FJ005199_2c_G172_1997, Node36, ITA_FJ005240_208_2007,
Node35, URU_KC196085_2c_M57_2007, Node34, FRA_DQ025975_04S6_2004,
Node33, USA_JX475243_ID_22772_2009, Node32,
USA_JX475252_CO_1316_2010, Node31, FRA_DQ025965_03C9_2003, Node30,
ECU_KF149984_2c_ME28_2012, Node29, ECU_KF149962_2c_ME1_2012,
ECU_KF149963_2c_ME10_2012, ECU_KF149964_2c_ME23_2012,
ECU_KF149969_2c_ME31_2012, Node88, Node86, Node84, Node28,
ARG_JF414820_Arg44_2009, ARG_KM236569_Cuba_2013,
ARG_JF414818_Arg32_2008, ARG_JF414821_Arg48_2009, Node95, Node93,
Node91, Node27, ITA_FJ005216_2c_284_2006,
ITA_KU508407_2c_25835_09_2009, ITA_KX434459_27692_1_11_2011,
Node100, Node98, Node26, URU_KM457122_2c_UY258_2010,
URU_KM457124_2c_UY307_2011, Node105, URU_KC196093_2c_M307_2011,
Node104, URU_KC196081_2c_M95_2007, URU_KM457109_2c_UY95_2007,
Node110, URU_KC196097_2c_M242_2010, URU_KM457120_2c_UY242_2010,
URU_KM457123_2c_UY261_2008, Node115, Node113, Node109, Node103,
Node25, URU_KM457103_2c_UY12_2006, ITA_FJ005209_2c_303_2004,
ITA_FJ005251_239_2008, Node120, Node118, Node24,
URU_KC196083_2c_M82_2007, URU_KM457108_2c_UY82_2007, Node123,
Node23, URU_KC196107_2c_M129_2008, Node22,
URU_KM457131_2c_UY368_2011, Node21, POR_KT275252_2c_PT013_12_2012,
Node20, ITA_FJ005231_406_2006, Node19, ITA_FJ005214_2c_67_2006,
BRA_KY073269_UFMT_2015, Node130, Node18, ARG_JF414819_Arg35_2008,
Node17, USA_KJ813854_Puma_ND_F205_2013, Node16,
ITA_FJ005232_411_2006, Node15, URU_KC196105_2c_M152_2008,
URU_KM457113_2c_UY152_2009, Node138, ITA_FJ005212_2c_349_2004,

Node137, POR_KT275255_2c_PT238_14_2014, ITA_KX434460_52238_12_2012,
URU_KC196091_2c_M326_2011, URU_KM457127_2c_UY326_2011, Node147,
URU_KC196102_2c_M185_2009, URU_KM457116_2c_UY185_2009, Node159,
HRV_KP859577_2c_HR856_2014, ITA_KX434458_2323_11_2011, Node162,
Node158, URU_KC196101_2c_M187_2009, URU_KM457117_2c_UY187_2009,
Node165, Node157, AUS_KU508693_2c_LW_2015, Node156,
URU_KC196089_2c_M349_2011, URU_KM457129_2c_UY349_2011, Node169,
Node155, ITA_FJ005195_2c_136_2000, Node154, FRA_DQ025976_04S7_2004,
Node153, USA_JX475273_MT_909_2012, USA_KJ813888_Coyote_MT_878_2012,
Node174, Node152, USA_KJ813843_Bobcat_ND_1160_2013, Node151,
FRA_DQ025942_01B1_2001, FRA_DQ025964_03C8_2003,
ITA_FJ005206_2c_287_2004, URU_KM457107_2c_UY72_2007,
URU_KM457111_2c_UY120_2008, URU_KM457112_2c_UY135_2008,
URU_KM457125_2c_UY317_2011, URU_KM457130_2c_UY354_2011,
URU_KM457142_2c_UY370_2011, HRV_KP859574_2c_HR442_2014,
HRV_KP859575_2c_HR774_2014, HRV_KP859576_2c_HR793_2014,
AUS_KU508691_2c_HB_2015, AUS_KU508692_2c_FH_2015,
ITA_KX434456_45361_09_2009, Node204, Node202, Node200, Node198,
Node196, Node194, Node192, Node190, Node188, Node186, Node184,
Node182, Node180, Node178, Node150, Node146, Node144, Node142,
Node136, Node14, URU_KM457126_2c_UY318_2010, Node13,
FRA_DQ025985_04S16_2004, ITA_FJ005205_2c_279_2004,
HRV_KP859578_2c_HR859_2014, Node210, Node208, Node12,
ITA_FJ222821_2c_56_2000, Node11, GER_FJ005260_G82_1997,
USA_KJ813846_Bobcat_ND_974_2013, Node214, Node10,
GER_AY742934_447_1995, RUS_JN033694_Laika_1993, Node218,
USA_AY742936_395_1998, USA_JX475240_AZ_16382_01_1999,
USA_JX475250_CO_728_2010, USA_KJ813842_Bobcat_ND_502_2013, Node225,
Node223, Node221, Node217, Node9,
USA_KJ813828_Fisher_F1F010712_2013,
USA_KJ813881_Graywolf_MI_832_2012, Node245,
USA_KJ813844_Bobcat_ND_885_2013, Node244,
USA_KJ813882_Raccoon_NJ_1423_2012, Node243,
USA_KJ813851_Bobcat_ND_1168_2013, Node242,
USA_JX475278_AR_1069_2012, Node241, KOR_EU009205_2b_K029_2006,
Node240, USA_JX475247_CO_1246_2010, Node239,
USA_KJ813892_Coyote_AK_218_2013, USA_JN867604_Dog_IL_137654_2008,
USA_JX475242_WI_18268_2002, Node256, Node254, Node238,
SAF_HQ602969_22_10SA_2010, Node237, USA_AY742955_436_2003,
FRA_DQ025991_2b_04S22_2004, Node260, Node236,
USA_JN867602_2b_Dog_CA_148743_2008, Node235,
USA_JX475251_CO_2235_2009, Node234,
USA_JN867603_2b_Dog_KS_81213_2009, Node233,
USA_KJ813852_Bobcat_ND_1170_2013, Node232,
FRA_DQ025961_2b_03C5_2003, Node231,
USA_KJ813827_Fisher_F1M111211_2013,
USA_KJ813873_Graywolf_MI_850_2012, Node268, Node230,
ECU_KF149971_2c_ME32_2012, IND_KX469432_newCPV_2b_Hiller_2011,
Node271, Node229, ITA_FJ005263_42_2005, ITA_FJ005265_140_2005,
Node275, USA_M74849_39_1995, USA_U22896_cat_1990, Node287,
FRA_DQ025992_2b_04S23_2004, Node286, USA_M74852_133_1995, Node285,
POR_KU662349_greywolf_W33_1996, Node284,
POR_KU662350_greywolf_W52_2005, POL_Z46651_46_1994, Node293,
Node283, GER_FJ005261_G162_1997, Node282,

BRA_DQ340409_2b_BR183_1985, Node281, USA_AY742932_193_1991, Node280,
USA_AY742951_431_2003, USA_JN867605_2b_Dog_US_142805_2009, Node300,
VAC_FJ222822_2b_FortDodge_2008, VAC_JN625223_INDIA_vac5_2011,
USA_EU659119_2b_CPV_410_2000, USA_EU659120_2b_CPV_411a_1998,
Node307, Node305, Node303, Node299, Node279,
CHI_GQ857609_CPV08_01_2008, CHI_GU569940_2b_YN0203_2002, Node311,
JPN_AB115504_2c_97_008_1997, TAW_U72696_2b_T10_1996, Node320,
TAW_U72695_2a_T4_1996, Node319, CHI_GQ857596_CPV05_01_2005,
CHI_GQ857600_CPV06_01_2006, Node324, Node318, THA_FJ869125_KU5_2004,
Node317, USA_JX475237_CT_372_2011, KOR_EF599097_2b_DH326_2006,
Node328, Node316, CHI_EU483515_2b_ZD13_2007,
JPN_LC270891_2b_9985_2017, JPN_AB437433_1887_M_2_2008,
TAW_FJ265781_CPV307_2005, TWN_EF592511_TWN1_2006,
TAW_FJ265775_CPV301_2004, Node339, Node337, Node335, Node333,
Node331, Node315, VIE_AB054218_2b_cat_V123_2000,
VAC_FJ222823_2b_29_1997, Node344, ITA_FJ005264_134_2005, Node343,
THA_FJ869122_KU1_2008, THA_FJ869123_KU3_2008, Node349,
THA_KP715690_VT28_2014, THA_KP715716_VT143_2014, Node357,
THA_KP715691_VT43_2014, Node356, VIE_AB120722_2b_HCM_18_2003,
VIE_AB120724_2b_HNI_2_13_2003, Node361, Node355,
CHI_GQ857599_CPV05_04_2005, CHI_GQ857601_CPV06_02_2006, Node364,
Node354, CHI_GQ857605_CPV07_03_2007, Node353,
THA_FJ869139_KU66_2003, VIE_AB120721_2b_HCM_8_2003,
VIE_AB054221_2b_leopard_V204_2000,
VIE_AB054224_2c_leopard_V203_2000, VIE_AB120725_2b_HNI_3_4_2003,
VIE_AB120723_2b_HCM_23_2003, VIE_AB120720_2b_HCM_6_2003,
VIE_AB054219_2b_cat_V209_2000, VIE_AB054220_2b_cat_V217_2000,
CHI_EU145954_2b_BJ044_2007, Node384, Node382, Node380, Node378,
Node376, Node374, Node372, Node370, Node368, Node352, Node348,
Node342, Node314, Node310, Node278, Node274, Node228, Node8,
ITA_FJ005257_54_2008, ITA_KF373611_2a_409_2010, Node387, Node7,
NZE_AY742933_339_1993, VIE_AB054223_2c_leopard_V140_2000,
ITA_GU362932_cat11_2008, Node393, NIG_HQ602995_15_10_2010,
FRA_DQ025947_2a_02B5_2002, FRA_DQ026001_2a_04S32_2004, Node407,
FRA_DQ025962_2a_03C6_2003, Node406, ITA_KF373580_2a_581_2003,
Node405, GER_AY742935_U6_1995, FRA_DQ025945_2a_02B3_2002, Node412,
Node404, VIE_AB054215_2a_cat_V120_2000, Node403,
ITA_FJ005255_333_2005, Node402, FRA_DQ025958_2a_03C2_2003, Node401,
ITA_KX434457_987_10_2010, FRA_DQ025983_2a_04S14_2004,
FRA_DQ025993_2a_04S24_2004, Node420, Node418, Node400,
FRA_DQ025984_2a_04S15_2004, ITA_FJ005252_96_2002, Node423, Node399,
FRA_DQ026002_2a_04S33_2004, ITA_KF373592_2a_329_2008,
ITA_AF393506_2a_699_2000, FRA_DQ025943_2a_01S1_2001, Node432,
ITA_KF385388_2a_Sicily_X83090_2009, Node431,
CHI_GQ857612_CPV08_04_2008, CHI_GU569939_2a_YN0202_2002, Node437,
HUN_KF539794_H_7_2012, HUN_KF539795_H_8_2012, Node444,
HUN_KF539804_H_212_2012, Node443, HUN_KF539793_H_5_2012,
HUN_KF539797_H_11_2012, Node448, Node442, HUN_KF539800_H_27_2012,
VIE_AB054217_2a_cat_V154_2000, HUN_KF539796_H_9_2012, Node453,
Node451, Node441, HUN_KF539798_H_31_2012, HUN_KF539799_H_39_2012,
Node458, HUN_KF539805_H_36_2012, Node457, ITA_AF306447_618_2000,
FRA_DQ025944_2a_02B2_2002, NIG_HQ602992_19_10_2010,
ITA_AF306446_584_2000, FRA_DQ025986_2a_04S17_2004,
ITA_KF373577_2a_714_2001, FRA_DQ025982_2a_04S13_2004,

ITA_FJ005253_67_2005, Node474, Node472, Node470, Node468, Node466, Node464, Node462, Node456, Node440, Node436, Node430, Node428, Node426, Node398, Node396, Node392, Node390, Node6, THA_FJ869126_KU5_2008, THA_FJ869137_KU52_2003, Node484, THA_FJ869134_KU23_2003, Node483, CHI_DQ354068_2a_redpanda_RPPV_2004, Node482, KOR_EF599096_DH426_2005, Node481, ITA_FJ005258_80_2008, KOR_EF599098_2c_Pome_2006, FRA_DQ025950_2a_02B9_2002, ITA_KX434454_29451_09_2009, Node494, Node492, Node490, Node480, THA_FJ869130_KU13_2004, THA_FJ869138_KU53_2003, CHI_KF803615_2011_BJ_B25_2011, Node499, Node497, Node479, CHI_GU569942_2a_JL0202_2002, CHI_GU569946_2a_JL0201_2002, Node502, Node478, USA_AY742953_435_2003, ITA_KF373571_2a_685_1999, Node506, THA_FJ869128_KU11_2004, BRA_DQ340428_2a_BR209_1994, BRA_DQ340431_2a_BR56_1995, Node513, BRA_DQ340411_2a_BR8_1990, Node512, BRA_DQ340422_2a_BR22_1993, BRA_DQ340421_2a_BR597_1992, BRA_DQ340419_2a_BR570_1992, BRA_DQ340423_2a_BR136_1993, Node524, BRA_DQ340413_2a_BR18_1990, Node523, BRA_DQ340427_2a_BR133_1994, Node522, BRA_DQ340414_2a_BR31_1990, BRA_DQ340416_2a_BR47_1991, BRA_DQ340417_2a_BR52_1991, BRA_DQ340418_2a_BR491_1992, BRA_DQ340424_2a_BR137_1993, BRA_DQ340426_2a_BR84_1994, Node537, Node535, Node533, Node531, Node529, Node521, Node519, Node517, Node511, Node509, Node505, Node477, Node5, CHI_KF803600_2010_BJ_A68_2010, Node4, USA_EU659118_CPV_13_1981, CHI_GU569948_2a_CC8601_1986, Node543, JPN_D26079_1993, Node542, BRA_DQ340407_2a_BR145_1980, BRA_DQ340408_2a_BR154_1980, Node548, FRA_DQ025952_2a_03B12_2003, BRA_DQ340404_2a_BR6_1980, BRA_DQ340405_2a_BR135_1980, BRA_DQ340410_2a_BR315_1986, USA_M24000_FPV_CPV_31_1988, USA_M24003_FPV_CPV_15_1988, Node559, Node557, Node555, Node553, Node551, Node547, Node541, Node3, USA_JN867599_Raccoon_KY_39552_2009, USA_JN867611_Raccoon_KY_358_B_2009, Node563, USA_JN867610_Raccoon_VA_118_A_2007, USA_KJ813890_Redfox_MA_197_2012, USA_JX475284_TN_26_2011, USA_JX475239_GA_06_2011, USA_JX475279_TN_1_2011, Node572, Node570, Node568, Node566, Node562, Node2, HUN_KF539801_H_25_2012, HUN_KF539803_H_2_2012, Node575, Node1, USA_KJ813870_Raccoon_TX_1_2013, USA_JN867598_Bobcat_KS_44_2010, USA_KJ813832_Fisher_ND_14_2013, Node582, USA_KJ813831_Fisher_ND_17_2013, USA_KJ813835_Fisher_ND_19_2013, Node585, Node581, USA_JX475234_ME_258_2011, USA_JN867618_Raccoon_WI_37_2010, USA_JX475231_CO_280_2011, USA_JX475248_CO_1102_2011, USA_JX475233_SC_182_A_2011, USA_JX475246_CO_2503_2010, Node596, Node594, Node592, Node590, Node588, Node580, Node578, CHI_FJ231389_FPV_monkey_BJ_22_2008, CHI_KJ170680_raccoondog_HLJ11_1_2011, Node600, CHI_GU392242_raccoondog_HB10_2009, CHI_GU392244_raccoondog_HB7_2009, Node611, CHI_KJ170679_raccoondog_Heb10_2_2010, Node610, CHI_GU392241_raccoondog_HB1_2009, Node609, CHI_GU392236_fox_HB1_2009, Node608, CHI_GU392240_raccoondog_HB3_2009, CHI_GU392239_raccoondog_HB6_2009, CHI_KJ194463_raccoondog_HeB10_3_2010, Node619, Node617, Node607, CHI_GU392237_fox_HB2_2009, Node606, VAC_FJ011098_Intervet_2006, VAC_JN625222_INDIA_vac4_2011, ITA_FJ222824_388_05_3_2005, CHI_FJ432718_CPV_Cv_2008, VAC_JN625219_INDIA_vac1_2011,

CHI_KF803602_2010_BJ_A72_2010, Node631, Node629, Node627, Node625, Node623, Node605, JPN_AB437434_1887_f_3_2008, Node604, VAC_GU212790_primodog_2009, VAC_GU212791_vanguard_2009, Node640, VAC_FJ197847_Pfizer_2007, Node639, VAC_EU914139_Pfizer_2006, Node638, VAC_KY083089_Singapore_2016, Node637, USA_M19296_CPV_N_1988, Node636, USA_M23255_FPV_Cornell320_1988, USA_M38245_1990, Node651, USA_EU659116_CPV_5_1979, Node650, FIN_U22192_raccoondog_RD_80_1980, FIN_U22193_raccoondog_RD87_1987, Node655, Node649, USA_M10989_1985, USA_U22186_CPV_128_1995, Node658, Node648, VAC_JN625221_INDIA_vac3_2011, VAC_JN625220_INDIA_vac2_2011, VAC_FJ011097_Merial_2006, CHI_GQ169553_Vac2_2007, Node667, VAC_KY083090_Singapore_2016, Node666, CHI_GU569943_YB8301_1983, VAC_JN625224_INDIA_vac6_2011, ARG_KM236572_NNGag_2012, Node673, Node671, Node665, Node663, Node661, Node647, Node635, Node603, Node599`

Obtaining branch lengths and nucleotide substitution biases under the nucleotide GTR model

* Log(L) = -6510.63, AIC-c = 14388.84 (683 estimated parameters)

Obtaining the global omega estimate based on relative GTR branch lengths and nucleotide substitution biases

* Log(L) = -6211.05, AIC-c = 13806.96 (690 estimated parameters)
* non-synonymous/synonymous rate ratio for *test* = 0.1168

Improving branch lengths, nucleotide substitution biases, and global dN/dS ratios under a full codon model

* Log(L) = -6211.05
* non-synonymous/synonymous rate ratio for *test* = 0.1166

For partition 1 these sites are significant at p <=0.1

Codon	Partition	alpha	beta+	p+	LRT	Episodic selection detected?	# branches
83	1	0.000	212.449	0.005	10.433	Yes, p = 0.0024	1
110	1	0.000	89.156	0.010	6.817	Yes, p = 0.0148	1
111	1	0.907	978.574	0.004	7.439	Yes, p = 0.0108	1
226	1	0.000	60.473	0.015	7.126	Yes, p = 0.0127	2
270	1	0.000	518.603	0.004	11.692	Yes, p = 0.0012	1
440	1	0.000	76.097	0.047	3.383	Yes, p = 0.0873	6

** Found _6_ sites under episodic diversifying positive selection at p <= 0.1**
