# Supplementary material for: Evidence of CPV2c introgression into Croatia and novel insights into phylogeny and cell tropism
Source: Sci Rep. 2019 Nov 15;9:16909. doi: 10.1038/s41598-019-53422-9 (PMC6858334; doi:10.1038/s41598-019-53422-9)
Supplement: Supplementary file 3 — Fixed Effects Liklihood [file 41598_2019_53422_MOESM3_ESM.pdf]

Evidence of CPV2c introgression into Croatia and novel insights into phylogeny and cell tropism

Dinko Novosel, Tamas Tuboly, Gyula Balka, Levente Szeredi, Ivana Lojkic, Andreja Jungic, Zaklin Acinger Rogic, Tahar Ait Ali, Attila Csagola

Supplementary info file 3.
Results of selection using Fixed Effects Liklihood method

```
     /HYPHY 2.3.13.20180530beta(MPI) for Linux on x86_64\
***************** TYPES OF STANDARD ANALYSES *****************


	(1) Selection Analyses
	(2) Evolutionary Hypothesis Testing
	(3) Relative evolutionary rate inference
	(4) Basic Analyses
	(5) Codon Selection Analyses
	(6) Compartmentalization
	(7) Data File Tools
	(8) Miscellaneous
	(9) Model Comparison
	(10) Kernel Analysis Tools
	(11) Molecular Clock
	(12) Phylogeny Reconstruction
	(13) Positive Selection
	(14) Recombination
	(15) Selection/Recombination
	(16) Relative Rate
	(17) Relative Ratio
	(18) Substitution Rates

1
 Please select type of analyses you want to list (or press ENTER to process custom batch file):



***************** FILES IN 'Selection Analyses' *****************


	(1) [MEME] Test for episodic site-level selection using MEME (Mixed Effects Model of Evolution).
	(2) [FEL] Test for pervasive site-level selection using FEL (Fixed Effects Likelihood).
```

(3) [SLAC] Test for pervasive site-level selection using SLAC (Single Likelihood Ancestor Counting).
(4) [FUBAR] Test for pervasive site-level selection using FUBAR (Fast Unconstrained Bayesian AppRoximation for inferring selection).
(5) [BUSTED] Test for episodic gene-wide selection using BUSTED (Branch-site Unrestricted Statistical Test of Episodic Diversification).
(6) [aBSREL] Test for lineage-specific evolution using the branch-site method aBS-REL (Adaptive Branch-Site Random Effects Likelihood).
(7) [RELAX] Test for relaxation of selection pressure along a specified set of test branches using RELAX (a random effects test of selection relaxation).

Please select the analysis you would like to perform (or press ENTER to return to the list of analysis types):2

Analysis Description

FEL (Fixed Effects Likelihood) estimates site-wise synonymous (α) and non-synonymous (β) rates, and uses a likelihood ratio test to determine if beta &neq; alpha at a site. The estimates aggregate information over all branches, so the signal is derived from pervasive diversification or conservation. A subset of branches can be selected for testing as well, in which case an additional (nuisance) parameter will be inferred -- the non-synonymous rate on branches NOT selected for testing. Multiple partitions within a NEXUS file are also supported for recombination - aware analysis.

- __Requirements__: in-frame codon alignment and a phylogenetic tree

- __Citation__: Not So Different After All: A Comparison of Methods for Detecting Amino
Acid Sites Under Selection (2005). _Mol Biol Evol_ 22 (5): 1208-1222

- __Written by__: Sergei L Kosakovsky Pond and Simon DW Frost

- __Contact Information__: spond@temple.edu

- __Analysis Version__: 2.00

####Choose Genetic Code

1. [**Universal**] Universal code. (Genebank transl_table=1).
2. [**Vertebrate mtDNA**] Vertebrate mitochondrial DNA code. (Genebank transl_table=2).
3. [**Yeast mtDNA**] Yeast mitochondrial DNA code. (Genebank transl_table=3).
4. [**Mold/Protozoan mtDNA**] Mold, Protozoan and Coelenterate mitochondrial DNA and the Mycloplasma/Spiroplasma code. (Genebank transl_table=4).
5. [**Invertebrate mtDNA**] Invertebrate mitochondrial DNA code. (Genebank transl_table=5).

6. [**Ciliate Nuclear**] Ciliate, Dasycladacean and Hexamita Nuclear code. (Genebank transl_table=6).
7. [**Echinoderm mtDNA**] Echinoderm mitochondrial DNA code. (Genebank transl_table=9).
8. [**Euplotid Nuclear**] Euplotid Nuclear code. (Genebank transl_table=10).
9. [**Alt. Yeast Nuclear**] Alternative Yeast Nuclear code. (Genebank transl_table=12).
10. [**Ascidian mtDNA**] Ascidian mitochondrial DNA code. (Genebank transl_table=13).
11. [**Flatworm mtDNA**] Flatworm mitochondrial DNA code. (Genebank transl_table=14).
12. [**Blepharisma Nuclear**] Blepharisma Nuclear code. (Genebank transl_table=15).
13. [**Chlorophycean mtDNA**] Chlorophycean Mitochondrial Code (transl_table=16).
14. [**Trematode mtDNA**] Trematode Mitochondrial Code (transl_table=21).
15. [**Scenedesmus obliquus mtDNA**] Scenedesmus obliquus mitochondrial Code (transl_table=22).
16. [**Thraustochytrium mtDNA**] Thraustochytrium Mitochondrial Code (transl_table=23).
17. [**Pterobranchia mtDNA**] Pterobranchia Mitochondrial Code (transl_table=24).
18. [**SR1 and Gracilibacteria**] Candidate Division SR1 and Gracilibacteria Code (transl_table=25).
19. [**Pachysolen Nuclear**] Pachysolen tannophilus Nuclear Code (transl_table=26).

>Please choose an option (or press q to cancel selection):1

>Select a coding sequence alignment file (`/home/opcestocarstvo/hyphy/res/TemplateBatchFiles/SelectionAnalyses/`) /home/opcestocarstvo/BEAST2/CPV_VP2_cod_sel.txt

>A tree was found in the data file: `((ITA/FJ005218/2c/330/2006,ITA/FJ005233/40/2007),POR/KT275253/2c/PT036/12/2012),(URU/KC196096/2c/M247/2010,URU/KM457121/2c/UY247/2010)),(URU/KC196086/2c/M55/2006,URU/KM457106/2c/UY55/2006)),USA/JX475260/CO/704/2010),ITA/FJ005247/195/2008),ITA/FJ005226/383/2006),GER/FJ005196/2c/G7/1997),FRA/DQ025994/04S25/2004),FRA/DQ025960/03C4/2003),FRA/DQ025951/03B10/2003),FRA/DQ025954/03B14/2003),USA/KJ813848/Bobcat/ND/1162/2013),URU/KM457104/2c/UY47/2006),FRA/DQ025969/03S5/2003),USA/KJ813858/Puma/ND/F93/2013),ITA/FJ005248/219/2008),GER/FJ005199/2c/G172/1997),ITA/FJ005240/208/2007),URU/KC196085/2c/M57/2007),FRA/DQ025975/04S6/2004),USA/JX475243/ID/22772/2009),USA/JX475252/CO/1316/2010),FRA/DQ025965/03C9/2003),ECU/KF149984/2c/ME28/2012),(ECU/KF149962/2c/ME1/2012,(ECU/KF149963/2c/ME10/2012,(ECU/KF149964/2c/ME23/2012,ECU/KF149969/2c/ME31/2012)))),(ARG/JF414820/Arg44/2009,(ARG/KM236569/Cuba/2013,(ARG/JF414818/Arg32/2008,ARG/JF414821/Arg48/2009)))),(ITA/FJ005216/2c/284/2006,(ITA/KU508407/2c/25835/09/2009,ITA/

KX434459/27692/1/11/2011))),(((URU/KM457122/2c/UY258/2010,URU/
KM457124/2c/UY307/2011),URU/KC196093/2c/M307/2011),((URU/KC196081/2c/
M95/2007,URU/KM457109/2c/UY95/2007),(URU/KC196097/2c/M242/2010,(URU/
KM457120/2c/UY242/2010,URU/KM457123/2c/UY261/2008))))),(URU/
KM457103/2c/UY12/2006,(ITA/FJ005209/2c/303/2004,ITA/FJ005251/239/2008))),
(URU/KC196083/2c/M82/2007,URU/KM457108/2c/UY82/2007)),URU/KC196107/2c/
M129/2008),URU/KM457131/2c/UY368/2011),POR/KT275252/2c/
PT013/12/2012),ITA/FJ005231/406/2006),(ITA/FJ005214/2c/67/2006,BRA/
KY073269/UFMT/2015)),ARG/JF414819/Arg35/2008),USA/KJ813854/Puma/ND/
F205/2013),ITA/FJ005232/411/2006),(((URU/KC196105/2c/M152/2008,URU/
KM457113/2c/UY152/2009),ITA/FJ005212/2c/349/2004),(POR/KT275255/2c/
PT238/14/2014,(ITA/KX434460/52238/12/2012,((URU/KC196091/2c/
M326/2011,URU/KM457127/2c/UY326/2011),(((((((((URU/KC196102/2c/
M185/2009,URU/KM457116/2c/UY185/2009),(HRV/KP859577/2c/HR856/2014,ITA/
KX434458/2323/11/2011)),(URU/KC196101/2c/M187/2009,URU/KM457117/2c/
UY187/2009)),AUS/KU508693/2c/LW/2015),(URU/KC196089/2c/M349/2011,URU/
KM457129/2c/UY349/2011)),ITA/FJ005195/2c/136/2000),FRA/
DQ025976/04S7/2004),(USA/JX475273/MT/909/2012,USA/KJ813888/Coyote/MT/
878/2012)),USA/KJ813843/Bobcat/ND/1160/2013),(FRA/DQ025942/01B1/2001,
(FRA/DQ025964/03C8/2003,(ITA/FJ005206/2c/287/2004,(URU/KM457107/2c/
UY72/2007,(URU/KM457111/2c/UY120/2008,(URU/KM457112/2c/UY135/2008,
(URU/KM457125/2c/UY317/2011,(URU/KM457130/2c/UY354/2011,(URU/
KM457142/2c/UY370/2011,(HRV/KP859574/2c/HR442/2014,(HRV/KP859575/2c/
HR774/2014,(HRV/KP859576/2c/HR793/2014,(AUS/KU508691/2c/HB/2015,(AUS/
KU508692/2c/FH/2015,ITA/KX434456/45361/09/2009))))))))))))))))))),URU/
KM457126/2c/UY318/2010),(FRA/DQ025985/04S16/2004,(ITA/FJ005205/2c/
279/2004,HRV/KP859578/2c/HR859/2014))),ITA/FJ222821/2c/56/2000),(GER/
FJ005260/G82/1997,USA/KJ813846/Bobcat/ND/974/2013)),((GER/
AY742934/447/1995,RUS/JN033694/Laika/1993),(USA/AY742936/395/1998,(USA/
JX475240/AZ/16382-01/1999,(USA/JX475250/CO/728/2010,USA/KJ813842/Bobcat/
ND/502/2013))))),(((((((((((((((((USA/KJ813828/Fisher/F1F010712/2013,USA/
KJ813881/Graywolf/MI/832/2012),USA/KJ813844/Bobcat/ND/885/2013),USA/
KJ813882/Raccoon/NJ/1423/2012),USA/KJ813851/Bobcat/ND/1168/2013),USA/
JX475278/AR/1069/2012),KOR/EU009205/2b/K029/2006),USA/JX475247/CO/
1246/2010),(USA/KJ813892/Coyote/AK/218/2013,(USA/JN867604/Dog/IL/
137654/2008,USA/JX475242/WI/18268/2002))),SAF/HQ602969/22/10SA/2010),
(USA/AY742955/436/2003,FRA/DQ025991/2b/04S22/2004)),USA/JN867602/2b/
Dog/CA/148743/2008),USA/JX475251/CO/2235/2009),USA/JN867603/2b/Dog/KS/
81213/2009),USA/KJ813852/Bobcat/ND/1170/2013),FRA/DQ025961/2b/
03C5/2003),(USA/KJ813827/Fisher/F1M111211/2013,USA/KJ813873/Graywolf/MI/
850/2012)),(ECU/KF149971/2c/ME32/2012,IND/KX469432/newCPV/2b/Hiller/
2011)),((ITA/FJ005263/42/2005,ITA/FJ005265/140/2005),(((((((((USA/
M74849/39/1995,USA/U22896/cat/1990),FRA/DQ025992/2b/04S23/2004),USA/
M74852/133/1995),POR/KU662349/greywolf/W33/1996),(POR/KU662350/greywolf/
W52/2005,POL/Z46651/46/1994)),GER/FJ005261/G162/1997),BRA/DQ340409/2b/
BR183/1985),USA/AY742932/193/1991),((USA/AY742951/431/2003,USA/
JN867605/2b/Dog/US/142805/2009),(VAC/FJ222822/2b/FortDodge/2008,(VAC/
JN625223/INDIA/vac5/2011,(USA/EU659119/2b/CPV/410/2000,USA/EU659120/2b/
CPV/411a/1998))))),((CHI/GQ857609/CPV08/01/2008,CHI/GU569940/2b/
YN0203/2002),(((((((JPN/AB115504/2c/97/008/1997,TAW/U72696/2b/

T10/1996),TAW/U72695/2a/T4/1996),(CHI/GQ857596/CPV05/01/2005,CHI/GQ857600/CPV06/01/2006)),THA/FJ869125/KU5/2004),(USA/JX475237/CT/372/2011,KOR/EF599097/2b/DH326/2006)),(CHI/EU483515/2b/ZD13/2007,(JPN/LC270891/2b/9985/2017,(JPN/AB437433/1887/M/2/2008,(TAW/FJ265781/CPV307/2005,(TWN/EF592511/TWN1/2006,TAW/FJ265775/CPV301/2004)))))),(((VIE/AB054218/2b/cat/V123/2000,VAC/FJ222823/2b/29/1997),ITA/FJ005264/134/2005),((THA/FJ869122/KU1/2008,THA/FJ869123/KU3/2008),((((((THA/KP715690/VT28/2014,THA/KP715716/VT143/2014),THA/KP715691/VT43/2014),(VIE/AB120722/2b/HCM/18/2003,VIE/AB120724/2b/HNI/2/13/2003)),(CHI/GQ857599/CPV05/04/2005,CHI/GQ857601/CPV06/02/2006)),CHI/GQ857605/CPV07/03/2007),(THA/FJ869139/KU66/2003,(VIE/AB120721/2b/HCM/8/2003,(VIE/AB054221/2b/leopard/V204/2000,(VIE/AB054224/2c/leopard/V203/2000,(VIE/AB120725/2b/HNI/3/4/2003,(VIE/AB120723/2b/HCM/23/2003,(VIE/AB120720/2b/HCM/6/2003,(VIE/AB054219/2b/cat/V209/2000,(VIE/AB054220/2b/cat/V217/2000,CHI/EU145954/2b/BJ044/20O7)))))))))))))))))),(ITA/FJ005257/54/2008,ITA/KF373611/2a/409/2010)),(NZE/AY742933/339/1993,((VIE/AB054223/2c/leopard/V140/2000,ITA/GU362932/cat11/2008),(NIG/HQ602995/15/10/2010,(((((((((FRA/DQ025947/2a/02B5/2002,FRA/DQ026001/2a/04S32/2004),FRA/DQ025962/2a/03C6/2003),ITA/KF373580/2a/581/2003),(GER/AY742935/U6/1995,FRA/DQ025945/2a/02B3/2002)),VIE/AB054215/2a/cat/V120/2000),ITA/FJ005255/333/2005),FRA/DQ025958/2a/03C2/2003),(ITA/KX434457/987/10/2010,(FRA/DQ025983/2a/04S14/2004,FRA/DQ025993/2a/04S24/2004))),(FRA/DQ025984/2a/04S15/2004,ITA/FJ005252/96/2002)),(FRA/DQ026002/2a/04S33/2004,(ITA/KF373592/2a/329/2008,(((ITA/AF393506/2a/699/2000,FRA/DQ025943/2a/01S1/2001),ITA/KF385388/2a/Sicily/X83090/2009),((CHI/GQ857612/CPV08/04/2008,CHI/GU569939/2a/YN0202/2002),(((((HUN/KF539794/H/7/2012,HUN/KF539795/H/8/2012),HUN/KF539804/H/212/2012),(HUN/KF539793/H/5/2012,HUN/KF539797/H/11/2012)),(HUN/KF539800/H/27/2012,(VIE/AB054217/2a/cat/V154/2000,HUN/KF539796/H/9/2012))),(((HUN/KF539798/H/31/2012,HUN/KF539799/H/39/2012),HUN/KF539805/H/36/2012),(ITA/AF306447/618/2000,(FRA/DQ025944/2a/02B2/2002,(NIG/HQ602992/19/10/2010,(ITA/AF306446/584/2000,(FRA/DQ025986/2a/04S17/2004,(ITA/KF373577/2a/714/2001,(FRA/DQ025982/2a/04S13/2004,ITA/FJ005253/67/2005))))))))))))))))),((((((((THA/FJ869126/KU5/2008,THA/FJ869137/KU52/2003),THA/FJ869134/KU23/2003),CHI/DQ354068/2a/redpanda/RPPV/2004),KOR/EF599096/DH426/2005),(ITA/FJ005258/80/2008,(KOR/EF599098/2c/Pome/2006,(FRA/DQ025950/2a/02B9/2002,ITA/KX434454/29451/09/2009)))),(THA/FJ869130/KU13/2004,(THA/FJ869138/KU53/2003,CHI/KF803615/2011/BJ/B25/2011))),(CHI/GU569942/2a/JL0202/2002,CHI/GU569946/2a/JL0201/2002)),((USA/AY742953/435/2003,ITA/KF373571/2a/685/1999),(THA/FJ869128/KU11/2004,(((BRA/DQ340428/2a/BR209/1994,BRA/DQ340431/2a/BR56/1995),BRA/DQ340411/2a/BR8/1990),(BRA/DQ340422/2a/BR22/1993,(BRA/DQ340421/2a/BR597/1992,((((BRA/DQ340419/2a/BR570/1992,BRA/DQ340423/2a/BR136/1993),BRA/DQ340413/2a/BR18/1990),BRA/DQ340427/2a/BR133/1994),(BRA/DQ340414/2a/BR31/1990,(BRA/DQ340416/2a/BR47/1991,(BRA/DQ340417/2a/BR52/1991,(BRA/DQ340418/2a/BR491/1992,(BRA/DQ340424/2a/BR137/1993,BRA/DQ340426/2a/BR84/1994)))))))))))),CHI/KF803600/2010/BJ/A68/2010),(((USA/EU659118/CPV/13/1981,CHI/GU569948/2a/CC8601/1986),JPN/D26079/1993),((BRA/DQ340407/2a/BR145/1980,BRA/DQ340408/2a/BR154/1980),(FRA/DQ025952/2a/03B12/2003,(BRA/DQ340404/2a/BR6/1980,(BRA/

DQ340405/2a/BR135/1980,(BRA/DQ340410/2a/BR315/1986,(USA/M24000/FPV/CPV/31/1988,USA/M24003/FPV/CPV/15/1988)))))))),((USA/JN867599/Raccoon/KY/39552/2009,USA/JN867611/Raccoon/KY/358-B/2009),(USA/JN867610/Raccoon/VA/118-A/2007,(USA/KJ813890/Redfox/MA/197/2012,(USA/JX475284/TN/26/2011,(USA/JX475239/GA/06/2011,USA/JX475279/TN/1/2011)))))),(HUN/KF539801/H/25/2012,HUN/KF539803/H/2/2012)),(USA/KJ813870/Raccoon/TX/1/2013,(((USA/JN867598/Bobcat/KS/44/2010,USA/KJ813832/Fisher/ND/14/2013),(USA/KJ813831/Fisher/ND/17/2013,USA/KJ813835/Fisher/ND/19/2013)),(USA/JX475234/ME/258/2011,(USA/JN867618//Raccoon/WI/37/2010,(USA/JX475231/CO/280/2011,(USA/JX475248/CO/1102/2011,(USA/JX475233/SC/182-A/2011,USA/JX475246/CO/2503/2010))))))),((CHI/FJ231389/FPV/monkey/BJ-22/2008,CHI/KJ170680/raccoondog/HLJ11/1/2011),((((((((CHI/GU392242/raccoondog/HB10/2009,CHI/GU392244/raccoondog/HB7/2009),CHI/KJ170679/raccoondog/Heb10/2/2010),CHI/GU392241/raccoondog/HB1/2009),CHI/GU392236/fox/HB1/2009),(CHI/GU392240/raccoondog/HB3/2009,(CHI/GU392239/raccoondog/HB6/2009,CHI/KJ194463/raccoondog/HeB10/3/2010))),CHI/GU392237/fox/HB2/2009),(VAC/FJ011098/Intervet/2006,(VAC/JN625222/INDIA/vac4/2011,(ITA/FJ222824/388/05/3/2005,(CHI/FJ432718/CPV/Cv/2008,(VAC/JN625219/INDIA/vac1/2011,CHI/KF803602/2010/BJ/A72/2010)))))),JPN/AB437434/1887/f/3/2008),((((((VAC/GU212790/primodog/2009,VAC/GU212791/vanguard/2009),VAC/FJ197847/Pfizer/2007),VAC/EU914139/Pfizer//2006),VAC/KY083089/Singapore/2016),USA/M19296/CPV/N/1988),(((((USA/M23255/FPV/Cornell320/1988,USA/M38245/1990),USA/EU659116/CPV/5/1979),(FIN/U22192/raccoondog/RD-80/1980,FIN/U22193/raccoondog/RD87/1987)),(USA/M10989/1985,USA/U22186/CPV/128/1995)),(VAC/JN625221/INDIA/vac3/2011,(VAC/JN625220/INDIA/vac2/2011,(((VAC/FJ011097/Merial/2006,CHI/GQ169553/Vac2/2007),VAC/KY083090/Singapore/2016),(CHI/GU569943/YB8301/1983,(VAC/JN625224/INDIA/vac6/2011,ARG/KM236572/NNGag/2012))))))))))`

>Would you like to use it (y/n)? y

>Loaded a multiple sequence alignment with **339** sequences, **581** codons, and **1** partitions from `/home/opcestocarstvo/BEAST2/CPV_VP2_cod_sel.txt`

####Choose the set of branches to test for selection

1. [**All**] Include all branches in the analysis
2. [**Internal**] Include all internal branches in the analysis
3. [**Leaves**] Include all leaf branches in the analysis
4. [**Unlabeled branches**] Set of 675 unlabeled branches

>Please choose an option (or press q to cancel selection):1

####Use synonymous rate variation? Strongly recommended YES for selection inference.

1. [**Yes**] [Recommended] Consider synonymous rate variation (dS varies across sites).

2. [**No**] Ignore synonymous rate variation (dS := 1 at each site).

>Please choose an option (or press q to cancel selection):1
Yes

>Select the p-value threshold to use when testing for selection (permissible range = [0,1], default value = 0.1): 0.1

Branches to include in the FEL analysis
Selected 675 branches to include in FEL calculations:
`ITA_FJ005218_2c_330_2006, ITA_FJ005233_40_2007, Node53, POR_KT275253_2c_PT036_12_2012, Node52, URU_KC196096_2c_M247_2010, URU_KM457121_2c_UY247_2010, Node57, Node51, URU_KC196086_2c_M55_2006, URU_KM457106_2c_UY55_2006, Node60, Node50, USA_JX475260_CO_704_2010, Node49, ITA_FJ005247_195_2008, Node48, ITA_FJ005226_383_2006, Node47, GER_FJ005196_2c_G7_1997, Node46, FRA_DQ025994_04S25_2004, Node45, FRA_DQ025960_03C4_2003, Node44, FRA_DQ025951_03B10_2003, Node43, FRA_DQ025954_03B14_2003, Node42, USA_KJ813848_Bobcat_ND_1162_2013, Node41, URU_KM457104_2c_UY47_2006, Node40, FRA_DQ025969_03S5_2003, Node39, USA_KJ813858_Puma_ND_F93_2013, Node38, ITA_FJ005248_219_2008, Node37, GER_FJ005199_2c_G172_1997, Node36, ITA_FJ005240_208_2007, Node35, URU_KC196085_2c_M57_2007, Node34, FRA_DQ025975_04S6_2004, Node33, USA_JX475243_ID_22772_2009, Node32, USA_JX475252_CO_1316_2010, Node31, FRA_DQ025965_03C9_2003, Node30, ECU_KF149984_2c_ME28_2012, Node29, ECU_KF149962_2c_ME1_2012, ECU_KF149963_2c_ME10_2012, ECU_KF149964_2c_ME23_2012, ECU_KF149969_2c_ME31_2012, Node88, Node86, Node84, Node28, ARG_JF414820_Arg44_2009, ARG_KM236569_Cuba_2013, ARG_JF414818_Arg32_2008, ARG_JF414821_Arg48_2009, Node95, Node93, Node91, Node27, ITA_FJ005216_2c_284_2006, ITA_KU508407_2c_25835_09_2009, ITA_KX434459_27692_1_11_2011, Node100, Node98, Node26, URU_KM457122_2c_UY258_2010, URU_KM457124_2c_UY307_2011, Node105, URU_KC196093_2c_M307_2011, Node104, URU_KC196081_2c_M95_2007, URU_KM457109_2c_UY95_2007, Node110, URU_KC196097_2c_M242_2010, URU_KM457120_2c_UY242_2010, URU_KM457123_2c_UY261_2008, Node115, Node113, Node109, Node103, Node25, URU_KM457103_2c_UY12_2006, ITA_FJ005209_2c_303_2004, ITA_FJ005251_239_2008, Node120, Node118, Node24, URU_KC196083_2c_M82_2007, URU_KM457108_2c_UY82_2007, Node123, Node23, URU_KC196107_2c_M129_2008, Node22, URU_KM457131_2c_UY368_2011, Node21, POR_KT275252_2c_PT013_12_2012, Node20, ITA_FJ005231_406_2006, Node19, ITA_FJ005214_2c_67_2006, BRA_KY073269_UFMT_2015, Node130, Node18, ARG_JF414819_Arg35_2008, Node17, USA_KJ813854_Puma_ND_F205_2013, Node16, ITA_FJ005232_411_2006, Node15, URU_KC196105_2c_M152_2008, URU_KM457113_2c_UY152_2009, Node138, ITA_FJ005212_2c_349_2004, Node137, POR_KT275255_2c_PT238_14_2014, ITA_KX434460_52238_12_2012, URU_KC196091_2c_M326_2011, URU_KM457127_2c_UY326_2011, Node147,

URU_KC196102_2c_M185_2009, URU_KM457116_2c_UY185_2009, Node159, HRV_KP859577_2c_HR856_2014, ITA_KX434458_2323_11_2011, Node162, Node158, URU_KC196101_2c_M187_2009, URU_KM457117_2c_UY187_2009, Node165, Node157, AUS_KU508693_2c_LW_2015, Node156, URU_KC196089_2c_M349_2011, URU_KM457129_2c_UY349_2011, Node169, Node155, ITA_FJ005195_2c_136_2000, Node154, FRA_DQ025976_04S7_2004, Node153, USA_JX475273_MT_909_2012, USA_KJ813888_Coyote_MT_878_2012, Node174, Node152, USA_KJ813843_Bobcat_ND_1160_2013, Node151, FRA_DQ025942_01B1_2001, FRA_DQ025964_03C8_2003, ITA_FJ005206_2c_287_2004, URU_KM457107_2c_UY72_2007, URU_KM457111_2c_UY120_2008, URU_KM457112_2c_UY135_2008, URU_KM457125_2c_UY317_2011, URU_KM457130_2c_UY354_2011, URU_KM457142_2c_UY370_2011, HRV_KP859574_2c_HR442_2014, HRV_KP859575_2c_HR774_2014, HRV_KP859576_2c_HR793_2014, AUS_KU508691_2c_HB_2015, AUS_KU508692_2c_FH_2015, ITA_KX434456_45361_09_2009, Node204, Node202, Node200, Node198, Node196, Node194, Node192, Node190, Node188, Node186, Node184, Node182, Node180, Node178, Node150, Node146, Node144, Node142, Node136, Node14, URU_KM457126_2c_UY318_2010, Node13, FRA_DQ025985_04S16_2004, ITA_FJ005205_2c_279_2004, HRV_KP859578_2c_HR859_2014, Node210, Node208, Node12, ITA_FJ222821_2c_56_2000, Node11, GER_FJ005260_G82_1997, USA_KJ813846_Bobcat_ND_974_2013, Node214, Node10, GER_AY742934_447_1995, RUS_JN033694_Laika_1993, Node218, USA_AY742936_395_1998, USA_JX475240_AZ_16382_01_1999, USA_JX475250_CO_728_2010, USA_KJ813842_Bobcat_ND_502_2013, Node225, Node223, Node221, Node217, Node9, USA_KJ813828_Fisher_F1F010712_2013, USA_KJ813881_Graywolf_MI_832_2012, Node245, USA_KJ813844_Bobcat_ND_885_2013, Node244, USA_KJ813882_Raccoon_NJ_1423_2012, Node243, USA_KJ813851_Bobcat_ND_1168_2013, Node242, USA_JX475278_AR_1069_2012, Node241, KOR_EU009205_2b_K029_2006, Node240, USA_JX475247_CO_1246_2010, Node239, USA_KJ813892_Coyote_AK_218_2013, USA_JN867604_Dog_IL_137654_2008, USA_JX475242_WI_18268_2002, Node256, Node254, Node238, SAF_HQ602969_22_10SA_2010, Node237, USA_AY742955_436_2003, FRA_DQ025991_2b_04S22_2004, Node260, Node236, USA_JN867602_2b_Dog_CA_148743_2008, Node235, USA_JX475251_CO_2235_2009, Node234, USA_JN867603_2b_Dog_KS_81213_2009, Node233, USA_KJ813852_Bobcat_ND_1170_2013, Node232, FRA_DQ025961_2b_03C5_2003, Node231, USA_KJ813827_Fisher_F1M111211_2013, USA_KJ813873_Graywolf_MI_850_2012, Node268, Node230, ECU_KF149971_2c_ME32_2012, IND_KX469432_newCPV_2b_Hiller_2011, Node271, Node229, ITA_FJ005263_42_2005, ITA_FJ005265_140_2005, Node275, USA_M74849_39_1995, USA_U22896_cat_1990, Node287, FRA_DQ025992_2b_04S23_2004, Node286, USA_M74852_133_1995, Node285, POR_KU662349_greywolf_W33_1996, Node284, POR_KU662350_greywolf_W52_2005, POL_Z46651_46_1994, Node293, Node283, GER_FJ005261_G162_1997, Node282, BRA_DQ340409_2b_BR183_1985,

Node281, USA_AY742932_193_1991, Node280, USA_AY742951_431_2003, USA_JN867605_2b_Dog_US_142805_2009, Node300, VAC_FJ222822_2b_FortDodge_2008, VAC_JN625223_INDIA_vac5_2011, USA_EU659119_2b_CPV_410_2000, USA_EU659120_2b_CPV_411a_1998, Node307, Node305, Node303, Node299, Node279, CHI_GQ857609_CPV08_01_2008, CHI_GU569940_2b_YN0203_2002, Node311, JPN_AB115504_2c_97_008_1997, TAW_U72696_2b_T10_1996, Node320, TAW_U72695_2a_T4_1996, Node319, CHI_GQ857596_CPV05_01_2005, CHI_GQ857600_CPV06_01_2006, Node324, Node318, THA_FJ869125_KU5_2004, Node317, USA_JX475237_CT_372_2011, KOR_EF599097_2b_DH326_2006, Node328, Node316, CHI_EU483515_2b_ZD13_2007, JPN_LC270891_2b_9985_2017, JPN_AB437433_1887_M_2_2008, TAW_FJ265781_CPV307_2005, TWN_EF592511_TWN1_2006, TAW_FJ265775_CPV301_2004, Node339, Node337, Node335, Node333, Node331, Node315, VIE_AB054218_2b_cat_V123_2000, VAC_FJ222823_2b_29_1997, Node344, ITA_FJ005264_134_2005, Node343, THA_FJ869122_KU1_2008, THA_FJ869123_KU3_2008, Node349, THA_KP715690_VT28_2014, THA_KP715716_VT143_2014, Node357, THA_KP715691_VT43_2014, Node356, VIE_AB120722_2b_HCM_18_2003, VIE_AB120724_2b_HNI_2_13_2003, Node361, Node355, CHI_GQ857599_CPV05_04_2005, CHI_GQ857601_CPV06_02_2006, Node364, Node354, CHI_GQ857605_CPV07_03_2007, Node353, THA_FJ869139_KU66_2003, VIE_AB120721_2b_HCM_8_2003, VIE_AB054221_2b_leopard_V204_2000, VIE_AB054224_2c_leopard_V203_2000, VIE_AB120725_2b_HNI_3_4_2003, VIE_AB120723_2b_HCM_23_2003, VIE_AB120720_2b_HCM_6_2003, VIE_AB054219_2b_cat_V209_2000, VIE_AB054220_2b_cat_V217_2000, CHI_EU145954_2b_BJ044_20O7, Node384, Node382, Node380, Node378, Node376, Node374, Node372, Node370, Node368, Node352, Node348, Node342, Node314, Node310, Node278, Node274, Node228, Node8, ITA_FJ005257_54_2008, ITA_KF373611_2a_409_2010, Node387, Node7, NZE_AY742933_339_1993, VIE_AB054223_2c_leopard_V140_2000, ITA_GU362932_cat11_2008, Node393, NIG_HQ602995_15_10_2010, FRA_DQ025947_2a_02B5_2002, FRA_DQ026001_2a_04S32_2004, Node407, FRA_DQ025962_2a_03C6_2003, Node406, ITA_KF373580_2a_581_2003, Node405, GER_AY742935_U6_1995, FRA_DQ025945_2a_02B3_2002, Node412, Node404, VIE_AB054215_2a_cat_V120_2000, Node403, ITA_FJ005255_333_2005, Node402, FRA_DQ025958_2a_03C2_2003, Node401, ITA_KX434457_987_10_2010, FRA_DQ025983_2a_04S14_2004, FRA_DQ025993_2a_04S24_2004, Node420, Node418, Node400, FRA_DQ025984_2a_04S15_2004, ITA_FJ005252_96_2002, Node423, Node399, FRA_DQ026002_2a_04S33_2004, ITA_KF373592_2a_329_2008, ITA_AF393506_2a_699_2000, FRA_DQ025943_2a_01S1_2001, Node432, ITA_KF385388_2a_Sicily_X83090_2009, Node431, CHI_GQ857612_CPV08_04_2008, CHI_GU569939_2a_YN0202_2002, Node437, HUN_KF539794_H_7_2012, HUN_KF539795_H_8_2012, Node444, HUN_KF539804_H_212_2012, Node443, HUN_KF539793_H_5_2012, HUN_KF539797_H_11_2012, Node448, Node442, HUN_KF539800_H_27_2012, VIE_AB054217_2a_cat_V154_2000, HUN_KF539796_H_9_2012, Node453, Node451, Node441, HUN_KF539798_H_31_2012, HUN_KF539799_H_39_2012,

Node458, HUN_KF539805_H_36_2012, Node457, ITA_AF306447_618_2000, FRA_DQ025944_2a_02B2_2002, NIG_HQ602992_19_10_2010, ITA_AF306446_584_2000, FRA_DQ025986_2a_04S17_2004, ITA_KF373577_2a_714_2001, FRA_DQ025982_2a_04S13_2004, ITA_FJ005253_67_2005, Node474, Node472, Node470, Node468, Node466, Node464, Node462, Node456, Node440, Node436, Node430, Node428, Node426, Node398, Node396, Node392, Node390, Node6, THA_FJ869126_KU5_2008, THA_FJ869137_KU52_2003, Node484, THA_FJ869134_KU23_2003, Node483, CHI_DQ354068_2a_redpanda_RPPV_2004, Node482, KOR_EF599096_DH426_2005, Node481, ITA_FJ005258_80_2008, KOR_EF599098_2c_Pome_2006, FRA_DQ025950_2a_02B9_2002, ITA_KX434454_29451_09_2009, Node494, Node492, Node490, Node480, THA_FJ869130_KU13_2004, THA_FJ869138_KU53_2003, CHI_KF803615_2011_BJ_B25_2011, Node499, Node497, Node479, CHI_GU569942_2a_JL0202_2002, CHI_GU569946_2a_JL0201_2002, Node502, Node478, USA_AY742953_435_2003, ITA_KF373571_2a_685_1999, Node506, THA_FJ869128_KU11_2004, BRA_DQ340428_2a_BR209_1994, BRA_DQ340431_2a_BR56_1995, Node513, BRA_DQ340411_2a_BR8_1990, Node512, BRA_DQ340422_2a_BR22_1993, BRA_DQ340421_2a_BR597_1992, BRA_DQ340419_2a_BR570_1992, BRA_DQ340423_2a_BR136_1993, Node524, BRA_DQ340413_2a_BR18_1990, Node523, BRA_DQ340427_2a_BR133_1994, Node522, BRA_DQ340414_2a_BR31_1990, BRA_DQ340416_2a_BR47_1991, BRA_DQ340417_2a_BR52_1991, BRA_DQ340418_2a_BR491_1992, BRA_DQ340424_2a_BR137_1993, BRA_DQ340426_2a_BR84_1994, Node537, Node535, Node533, Node531, Node529, Node521, Node519, Node517, Node511, Node509, Node505, Node477, Node5, CHI_KF803600_2010_BJ_A68_2010, Node4, USA_EU659118_CPV_13_1981, CHI_GU569948_2a_CC8601_1986, Node543, JPN_D26079_1993, Node542, BRA_DQ340407_2a_BR145_1980, BRA_DQ340408_2a_BR154_1980, Node548, FRA_DQ025952_2a_03B12_2003, BRA_DQ340404_2a_BR6_1980, BRA_DQ340405_2a_BR135_1980, BRA_DQ340410_2a_BR315_1986, USA_M24000_FPV_CPV_31_1988, USA_M24003_FPV_CPV_15_1988, Node559, Node557, Node555, Node553, Node551, Node547, Node541, Node3, USA_JN867599_Raccoon_KY_39552_2009, USA_JN867611_Raccoon_KY_358_B_2009, Node563, USA_JN867610_Raccoon_VA_118_A_2007, USA_KJ813890_Redfox_MA_197_2012, USA_JX475284_TN_26_2011, USA_JX475239_GA_06_2011, USA_JX475279_TN_1_2011, Node572, Node570, Node568, Node566, Node562, Node2, HUN_KF539801_H_25_2012, HUN_KF539803_H_2_2012, Node575, Node1, USA_KJ813870_Raccoon_TX_1_2013, USA_JN867598_Bobcat_KS_44_2010, USA_KJ813832_Fisher_ND_14_2013, Node582, USA_KJ813831_Fisher_ND_17_2013, USA_KJ813835_Fisher_ND_19_2013, Node585, Node581, USA_JX475234_ME_258_2011, USA_JN867618_Raccoon_WI_37_2010, USA_JX475231_CO_280_2011, USA_JX475248_CO_1102_2011, USA_JX475233_SC_182_A_2011, USA_JX475246_CO_2503_2010, Node596, Node594, Node592, Node590, Node588, Node580, Node578, CHI_FJ231389_FPV_monkey_BJ_22_2008, CHI_KJ170680_raccoondog_HLJ11_1_2011, Node600, CHI_GU392242_raccoondog_HB10_2009, CHI_GU392244_raccoondog_HB7_2009, Node611,

CHI_KJ170679_raccoondog_Heb10_2_2010, Node610, CHI_GU392241_raccoondog_HB1_2009, Node609, CHI_GU392236_fox_HB1_2009, Node608, CHI_GU392240_raccoondog_HB3_2009, CHI_GU392239_raccoondog_HB6_2009, CHI_KJ194463_raccoondog_HeB10_3_2010, Node619, Node617, Node607, CHI_GU392237_fox_HB2_2009, Node606, VAC_FJ011098_Intervet_2006, VAC_JN625222_INDIA_vac4_2011, ITA_FJ222824_388_05_3_2005, CHI_FJ432718_CPV_Cv_2008, VAC_JN625219_INDIA_vac1_2011, CHI_KF803602_2010_BJ_A72_2010, Node631, Node629, Node627, Node625, Node623, Node605, JPN_AB437434_1887_f_3_2008, Node604, VAC_GU212790_primodog_2009, VAC_GU212791_vanguard_2009, Node640, VAC_FJ197847_Pfizer_2007, Node639, VAC_EU914139_Pfizer_2006, Node638, VAC_KY083089_Singapore_2016, Node637, USA_M19296_CPV_N_1988, Node636, USA_M23255_FPV_Cornell320_1988, USA_M38245_1990, Node651, USA_EU659116_CPV_5_1979, Node650, FIN_U22192_raccoondog_RD_80_1980, FIN_U22193_raccoondog_RD87_1987, Node655, Node649, USA_M10989_1985, USA_U22186_CPV_128_1995, Node658, Node648, VAC_JN625221_INDIA_vac3_2011, VAC_JN625220_INDIA_vac2_2011, VAC_FJ011097_Merial_2006, CHI_GQ169553_Vac2_2007, Node667, VAC_KY083090_Singapore_2016, Node666, CHI_GU569943_YB8301_1983, VAC_JN625224_INDIA_vac6_2011, ARG_KM236572_NNGag_2012, Node673, Node671, Node665, Node663, Node661, Node647, Node635, Node603, Node599`

Obtaining branch lengths and nucleotide substitution biases under the nucleotide GTR model

* Log(L) = -6510.63, AIC-c = 14388.83 (683 estimated parameters)

Obtaining the global omega estimate based on relative GTR branch lengths and nucleotide substitution biases

* Log(L) = -6211.05, AIC-c = 13806.96 (690 estimated parameters)
* non-synonymous/synonymous rate ratio for *test* = 0.1167

Improving branch lengths, nucleotide substitution biases, and global dN/dS ratios under a full codon model

* Log(L) = -6211.05
* non-synonymous/synonymous rate ratio for *test* = 0.1165

For partition 1 these sites are significant at p <=0.1

Codon	Partition	alpha	beta	LRT	Selection detected?
6	1	2.732	0.000	4.183	Neg. p = 0.0408
12	1	16.165	0.000	25.499	Neg. p = 0.0000
32	1	7.755	0.000	13.480	Neg. p = 0.0002
20	1	12.064	0.000	20.105	Neg. p = 0.0000
40	1	3.433	0.000	4.533	Neg. p = 0.0332
34	1	2.637	0.000	4.542	Neg. p = 0.0331
43	1	2.719	0.000	4.597	Neg. p = 0.0320

45	1	2.288	0.000	6.129	Neg. p = 0.0133
48	1	2.341	0.000	6.071	Neg. p = 0.0137
49	1	3.695	0.000	11.998	Neg. p = 0.0005
60	1	4.802	0.000	7.900	Neg. p = 0.0049
76	1	7.258	0.000	6.443	Neg. p = 0.0111
74	1	3.340	0.000	6.539	Neg. p = 0.0106
82	1	0.992	0.000	3.318	Neg. p = 0.0685
84	1	2.851	0.000	6.185	Neg. p = 0.0129
86	1	7.266	0.000	6.009	Neg. p = 0.0142
101	1	12.882	1.173	11.877	Neg. p = 0.0006
89	1	2.365	0.000	3.900	Neg. p = 0.0483
104	1	4.819	0.000	6.626	Neg. p = 0.0101
106	1	1.630	0.000	3.310	Neg. p = 0.0689
118	1	1.634	0.000	3.613	Neg. p = 0.0573
119	1	2.735	0.000	4.185	Neg. p = 0.0408
123	1	1.647	0.000	3.254	Neg. p = 0.0712
127	1	2.365	0.000	3.296	Neg. p = 0.0694
142	1	4.770	0.000	7.879	Neg. p = 0.0050
135	1	1.760	0.000	5.803	Neg. p = 0.0160
136	1	1.368	0.000	3.282	Neg. p = 0.0701
145	1	3.445	0.000	4.540	Neg. p = 0.0331
157	1	2.717	0.000	4.257	Neg. p = 0.0391
161	1	1.630	0.000	3.237	Neg. p = 0.0720
169	1	2.231	0.000	3.581	Neg. p = 0.0584
168	1	14.755	0.000	12.209	Neg. p = 0.0005
178	1	7.199	0.000	6.098	Neg. p = 0.0135
184	1	1.630	0.000	3.237	Neg. p = 0.0720
188	1	1.634	0.000	3.391	Neg. p = 0.0656
196	1	5.391	0.000	9.148	Neg. p = 0.0025
179	1	12.809	0.000	19.736	Neg. p = 0.0000
198	1	7.266	0.000	5.319	Neg. p = 0.0211
197	1	7.133	0.000	5.489	Neg. p = 0.0191
209	1	1.782	0.000	3.751	Neg. p = 0.0528
204	1	5.393	0.000	5.605	Neg. p = 0.0179
216	1	5.598	0.321	7.267	Neg. p = 0.0070
220	1	1.567	0.000	3.175	Neg. p = 0.0748
213	1	4.819	0.000	6.626	Neg. p = 0.0101
227	1	1.105	0.000	4.155	Neg. p = 0.0415
229	1	1.634	0.000	3.241	Neg. p = 0.0718
231	1	7.021	0.000	5.947	Neg. p = 0.0147
235	1	2.722	0.000	4.599	Neg. p = 0.0320
234	1	7.200	0.000	5.930	Neg. p = 0.0149
243	1	15.056	0.000	11.094	Neg. p = 0.0009
241	1	2.716	0.000	4.173	Neg. p = 0.0411
244	1	12.251	0.536	6.329	Neg. p = 0.0119
240	1	28.972	0.000	24.042	Neg. p = 0.0000
248	1	7.266	0.000	6.009	Neg. p = 0.0142
254	1	2.223	0.000	3.574	Neg. p = 0.0587
252	1	6.498	0.000	13.142	Neg. p = 0.0003
269	1	7.198	0.351	3.421	Neg. p = 0.0644

273	1	7.272	0.000	5.966	Neg. p = 0.0146
280	1	2.357	0.000	3.291	Neg. p = 0.0696
284	1	1.634	0.000	3.391	Neg. p = 0.0656
285	1	1.364	0.000	3.275	Neg. p = 0.0704
291	1	1.446	0.000	3.052	Neg. p = 0.0807
299	1	1.647	0.000	3.627	Neg. p = 0.0569
295	1	2.706	0.000	4.088	Neg. p = 0.0432
304	1	2.706	0.000	4.588	Neg. p = 0.0322
307	1	1.638	0.000	3.617	Neg. p = 0.0572
316	1	6.923	0.765	6.747	Neg. p = 0.0094
314	1	2.737	0.000	4.472	Neg. p = 0.0344
321	1	7.184	0.372	3.317	Neg. p = 0.0686
315	1	2.717	0.000	4.595	Neg. p = 0.0321
330	1	3.473	0.000	4.553	Neg. p = 0.0329
332	1	1.781	0.000	3.751	Neg. p = 0.0528
325	1	6.871	0.000	9.049	Neg. p = 0.0026
337	1	2.735	0.000	4.607	Neg. p = 0.0318
335	1	1.163	0.000	3.851	Neg. p = 0.0497
340	1	1.640	0.000	3.397	Neg. p = 0.0653
349	1	1.634	0.000	3.297	Neg. p = 0.0694
351	1	0.968	0.000	3.821	Neg. p = 0.0506
342	1	14.756	0.000	10.618	Neg. p = 0.0011
363	1	1.634	0.000	3.613	Neg. p = 0.0573
366	1	3.302	0.000	6.614	Neg. p = 0.0101
364	1	3.936	0.000	13.596	Neg. p = 0.0002
376	1	1.640	0.000	3.247	Neg. p = 0.0715
382	1	3.587	0.000	7.510	Neg. p = 0.0061
394	1	1.638	0.000	3.301	Neg. p = 0.0692
387	1	2.335	0.000	3.879	Neg. p = 0.0489
396	1	1.166	0.000	3.857	Neg. p = 0.0496
407	1	1.633	0.000	3.612	Neg. p = 0.0574
398	1	7.199	0.000	5.505	Neg. p = 0.0190
409	1	7.199	0.000	5.303	Neg. p = 0.0213
412	1	1.641	0.000	3.620	Neg. p = 0.0571
413	1	7.265	0.352	3.437	Neg. p = 0.0637
416	1	12.665	0.000	16.794	Neg. p = 0.0000
429	1	4.969	0.384	5.888	Neg. p = 0.0152
430	1	1.826	0.000	4.379	Neg. p = 0.0364
425	1	4.647	0.000	9.600	Neg. p = 0.0019
432	1	1.630	0.000	3.237	Neg. p = 0.0720
438	1	2.706	0.000	4.588	Neg. p = 0.0322
442	1	3.416	0.000	4.524	Neg. p = 0.0334
447	1	5.394	0.347	2.958	Neg. p = 0.0855
445	1	8.001	0.000	12.332	Neg. p = 0.0004
450	1	2.652	0.000	4.113	Neg. p = 0.0426
446	1	7.266	0.000	6.009	Neg. p = 0.0142
458	1	7.266	0.000	6.009	Neg. p = 0.0142
468	1	2.374	0.000	3.302	Neg. p = 0.0692
460	1	3.289	0.000	6.635	Neg. p = 0.0100
469	1	3.450	0.000	4.542	Neg. p = 0.0331

482	1	2.717	0.000	4.004	Neg. p = 0.0454
476	1	2.717	0.000	4.155	Neg. p = 0.0415
484	1	1.646	0.000	3.326	Neg. p = 0.0682
489	1	2.706	0.000	4.167	Neg. p = 0.0412
491	1	4.802	0.000	6.615	Neg. p = 0.0101
500	1	1.619	0.000	3.299	Neg. p = 0.0693
495	1	2.717	0.000	4.095	Neg. p = 0.0430
501	1	2.318	0.000	3.867	Neg. p = 0.0492
494	1	16.038	0.000	16.090	Neg. p = 0.0001
503	1	1.958	0.000	6.787	Neg. p = 0.0092
504	1	2.718	0.000	4.095	Neg. p = 0.0430
509	1	2.365	0.000	3.939	Neg. p = 0.0472
507	1	5.868	0.000	12.443	Neg. p = 0.0004
522	1	4.920	0.000	9.921	Neg. p = 0.0016
523	1	2.727	0.000	4.161	Neg. p = 0.0414
527	1	6.878	0.000	5.424	Neg. p = 0.0199
532	1	4.765	0.000	7.799	Neg. p = 0.0052
535	1	7.134	0.000	5.489	Neg. p = 0.0191
538	1	2.373	0.000	3.905	Neg. p = 0.0481
547	1	1.638	0.000	3.245	Neg. p = 0.0716
546	1	7.266	0.000	6.009	Neg. p = 0.0142
549	1	4.786	0.000	6.605	Neg. p = 0.0102
552	1	14.756	0.000	12.864	Neg. p = 0.0003
553	1	10.421	0.000	16.132	Neg. p = 0.0001
565	1	6.943	0.000	5.927	Neg. p = 0.0149
570	1	5.256	1.168	3.072	Neg. p = 0.0797
574	1	7.136	0.000	11.766	Neg. p = 0.0006
572	1	16.180	0.000	32.437	Neg. p = 0.0000
580	1	2.736	0.000	4.107	Neg. p = 0.0427

** Found _0_ sites under pervasive positive diversifying and _136_ sites under negative selection at p <= 0.1**
