# Supplementary material for: Evidence of CPV2c introgression into Croatia and novel insights into phylogeny and cell tropism
Source: Sci Rep. 2019 Nov 15;9:16909. doi: 10.1038/s41598-019-53422-9 (PMC6858334; doi:10.1038/s41598-019-53422-9)
Supplement: Supplementary file 4 — Single-Likelihood Ancestor Counting [file 41598_2019_53422_MOESM4_ESM.pdf]

Evidence of CPV2c introgression into Croatia and novel insights into phylogeny and cell tropism

Dinko Novosel, Tamas Tuboly, Gyula Balka, Levente Szeredi, Ivana Lojkic, Andreja Jungic, Zaklin Acinger Rogic, Tahar Ait Ali, Attila Csagola

Supplementary info file 4.

Results of selection using Single-Likelihhod Ancestor Couting method

/HYPHY 2.3.13.20180530beta(MPI) for Linux on x86_64\

***************** TYPES OF STANDARD ANALYSES *****************

(1) Selection Analyses
(2) Evolutionary Hypothesis Testing
(3) Relative evolutionary rate inference
(4) Basic Analyses
(5) Codon Selection Analyses
(6) Compartmentalization
(7) Data File Tools
(8) Miscellaneous
(9) Model Comparison
(10) Kernel Analysis Tools
(11) Molecular Clock
(12) Phylogeny Reconstruction
(13) Positive Selection
(14) Recombination
(15) Selection/Recombination
(16) Relative Rate
(17) Relative Ratio
(18) Substitution Rates

1

Please select type of analyses you want to list (or press ENTER to process custom batch file):

***************** FILES IN 'Selection Analyses' *****************

(1) [MEME] Test for episodic site-level selection using MEME (Mixed Effects Model of Evolution).
(2) [FEL] Test for pervasive site-level selection using FEL (Fixed Effects Likelihood).

(3) [SLAC] Test for pervasive site-level selection using SLAC (Single Likelihood Ancestor Counting).
(4) [FUBAR] Test for pervasive site-level selection using FUBAR (Fast Unconstrained Bayesian AppRoximation for inferring selection).
(5) [BUSTED] Test for episodic gene-wide selection using BUSTED (Branch-site Unrestricted Statistical Test of Episodic Diversification).
(6) [aBSREL] Test for lineage-specific evolution using the branch-site method aBS-REL (Adaptive Branch-Site Random Effects Likelihood).
(7) [RELAX] Test for relaxation of selection pressure along a specified set of test branches using RELAX (a random effects test of selection relaxation).

Please select the analysis you would like to perform (or press ENTER to return to the list of analysis types):3

Analysis Description

SLAC (Single Likelihood Ancestor Counting) uses a maximum likelihood ancestral state reconstruction and minimum path substitution counting to estimate site - level dS and dN, and applies a simple binomial - based test to test if dS differs drom dN. The estimates aggregate information over all branches, so the signal is derived from pervasive diversification or conservation. A subset of branches can be selected for testing as well. Multiple partitions within a NEXUS file are also supported for recombination - aware analysis.

- __Requirements__: in-frame codon alignment and a phylogenetic tree

- __Citation__: Not So Different After All: A Comparison of Methods for Detecting Amino
Acid Sites Under Selection (2005). _Mol Biol Evol_ 22 (5): 1208-1222

- __Written by__: Sergei L Kosakovsky Pond and Simon DW Frost

- __Contact Information__: spond@temple.edu

- __Analysis Version__: 2.00

####Choose Genetic Code

1. [**Universal**] Universal code. (Genebank transl_table=1).
2. [**Vertebrate mtDNA**] Vertebrate mitochondrial DNA code. (Genebank transl_table=2).
3. [**Yeast mtDNA**] Yeast mitochondrial DNA code. (Genebank transl_table=3).
4. [**Mold/Protozoan mtDNA**] Mold, Protozoan and Coelenterate mitochondrial DNA and the Mycloplasma/Spiroplasma code. (Genebank transl_table=4).
5. [**Invertebrate mtDNA**] Invertebrate mitochondrial DNA code. (Genebank transl_table=5).
6. [**Ciliate Nuclear**] Ciliate, Dasycladacean and Hexamita Nuclear code.

(Genebank transl_table=6).
7. [**Echinoderm mtDNA**] Echinoderm mitochondrial DNA code. (Genebank transl_table=9).
8. [**Euplotid Nuclear**] Euplotid Nuclear code. (Genebank transl_table=10).
9. [**Alt. Yeast Nuclear**] Alternative Yeast Nuclear code. (Genebank transl_table=12).
10. [**Ascidian mtDNA**] Ascidian mitochondrial DNA code. (Genebank transl_table=13).
11. [**Flatworm mtDNA**] Flatworm mitochondrial DNA code. (Genebank transl_table=14).
12. [**Blepharisma Nuclear**] Blepharisma Nuclear code. (Genebank transl_table=15).
13. [**Chlorophycean mtDNA**] Chlorophycean Mitochondrial Code (transl_table=16).
14. [**Trematode mtDNA**] Trematode Mitochondrial Code (transl_table=21).
15. [**Scenedesmus obliquus mtDNA**] Scenedesmus obliquus mitochondrial Code (transl_table=22).
16. [**Thraustochytrium mtDNA**] Thraustochytrium Mitochondrial Code (transl_table=23).
17. [**Pterobranchia mtDNA**] Pterobranchia Mitochondrial Code (transl_table=24).
18. [**SR1 and Gracilibacteria**] Candidate Division SR1 and Gracilibacteria Code (transl_table=25).
19. [**Pachysolen Nuclear**] Pachysolen tannophilus Nuclear Code (transl_table=26).

>Please choose an option (or press q to cancel selection):1

>Select a coding sequence alignment file (`/home/opcestocarstvo/hyphy/res/TemplateBatchFiles/SelectionAnalyses/`) /home/opcestocartsvo/BEAST2/CPV_VP2_cod_sel.txt

>Select a coding sequence alignment file (`/home/opcestocarstvo/hyphy/res/TemplateBatchFiles/SelectionAnalyses/`) /home/opcestocarstvo/BEAST2/CPV_VP2_cod_sel.txt

>A tree was found in the data file: `((ITA/FJ005218/2c/330/2006,ITA/FJ005233/40/2007),POR/KT275253/2c/PT036/12/2012),(URU/KC196096/2c/M247/2010,URU/KM457121/2c/UY247/2010)),(URU/KC196086/2c/M55/2006,URU/KM457106/2c/UY55/2006)),USA/JX475260/CO/704/2010),ITA/FJ005247/195/2008),ITA/FJ005226/383/2006),GER/FJ005196/2c/G7/1997),FRA/DQ025994/04S25/2004),FRA/DQ025960/03C4/2003),FRA/DQ025951/03B10/2003),FRA/DQ025954/03B14/2003),USA/KJ813848/Bobcat/ND/1162/2013),URU/KM457104/2c/UY47/2006),FRA/DQ025969/03S5/2003),USA/KJ813858/Puma/ND/F93/2013),ITA/FJ005248/219/2008),GER/FJ005199/2c/G172/1997),ITA/FJ005240/208/2007),URU/KC196085/2c/M57/2007),FRA/DQ025975/04S6/2004),USA/JX475243/ID/22772/2009),USA/JX475252/CO/1316/2010),FRA/DQ025965/03C9/2003),ECU/KF149984/2c/ME28/2012),(ECU/

KF149962/2c/ME1/2012,(ECU/KF149963/2c/ME10/2012,(ECU/KF149964/2c/
ME23/2012,ECU/KF149969/2c/ME31/2012)))),(ARG/JF414820/Arg44/2009,(ARG/
KM236569/Cuba/2013,(ARG/JF414818/Arg32/2008,ARG/JF414821/Arg48/2009)))),
(ITA/FJ005216/2c/284/2006,(ITA/KU508407/2c/25835/09/2009,ITA/
KX434459/27692/1/11/2011))),(((URU/KM457122/2c/UY258/2010,URU/
KM457124/2c/UY307/2011),URU/KC196093/2c/M307/2011),((URU/KC196081/2c/
M95/2007,URU/KM457109/2c/UY95/2007),(URU/KC196097/2c/M242/2010,(URU/
KM457120/2c/UY242/2010,URU/KM457123/2c/UY261/2008))))),(URU/
KM457103/2c/UY12/2006,(ITA/FJ005209/2c/303/2004,ITA/FJ005251/239/2008))),
(URU/KC196083/2c/M82/2007,URU/KM457108/2c/UY82/2007)),URU/KC196107/2c/
M129/2008),URU/KM457131/2c/UY368/2011),POR/KT275252/2c/
PT013/12/2012),ITA/FJ005231/406/2006),(ITA/FJ005214/2c/67/2006,BRA/
KY073269/UFMT/2015)),ARG/JF414819/Arg35/2008),USA/KJ813854/Puma/ND/
F205/2013),ITA/FJ005232/411/2006),(((URU/KC196105/2c/M152/2008,URU/
KM457113/2c/UY152/2009),ITA/FJ005212/2c/349/2004),(POR/KT275255/2c/
PT238/14/2014,(ITA/KX434460/52238/12/2012,((URU/KC196091/2c/
M326/2011,URU/KM457127/2c/UY326/2011),((((((((((URU/KC196102/2c/
M185/2009,URU/KM457116/2c/UY185/2009),(HRV/KP859577/2c/HR856/2014,ITA/
KX434458/2323/11/2011)),(URU/KC196101/2c/M187/2009,URU/KM457117/2c/
UY187/2009)),AUS/KU508693/2c/LW/2015),(URU/KC196089/2c/M349/2011,URU/
KM457129/2c/UY349/2011)),ITA/FJ005195/2c/136/2000),FRA/
DQ025976/04S7/2004),(USA/JX475273/MT/909/2012,USA/KJ813888/Coyote/MT/
878/2012)),USA/KJ813843/Bobcat/ND/1160/2013),(FRA/DQ025942/01B1/2001,
(FRA/DQ025964/03C8/2003,(ITA/FJ005206/2c/287/2004,(URU/KM457107/2c/
UY72/2007,(URU/KM457111/2c/UY120/2008,(URU/KM457112/2c/UY135/2008,
(URU/KM457125/2c/UY317/2011,(URU/KM457130/2c/UY354/2011,(URU/
KM457142/2c/UY370/2011,(HRV/KP859574/2c/HR442/2014,(HRV/KP859575/2c/
HR774/2014,(HRV/KP859576/2c/HR793/2014,(AUS/KU508691/2c/HB/2015,(AUS/
KU508692/2c/FH/2015,ITA/KX434456/45361/09/2009)))))))))))))))))))),URU/
KM457126/2c/UY318/2010),(FRA/DQ025985/04S16/2004,(ITA/FJ005205/2c/
279/2004,HRV/KP859578/2c/HR859/2014))),ITA/FJ222821/2c/56/2000),(GER/
FJ005260/G82/1997,USA/KJ813846/Bobcat/ND/974/2013)),((GER/
AY742934/447/1995,RUS/JN033694/Laika/1993),(USA/AY742936/395/1998,(USA/
JX475240/AZ/16382-01/1999,(USA/JX475250/CO/728/2010,USA/KJ813842/Bobcat/
ND/502/2013))))),((((((((((((((((((USA/KJ813828/Fisher/F1F010712/2013,USA/
KJ813881/Graywolf/MI/832/2012),USA/KJ813844/Bobcat/ND/885/2013),USA/
KJ813882/Raccoon/NJ/1423/2012),USA/KJ813851/Bobcat/ND/1168/2013),USA/
JX475278/AR/1069/2012),KOR/EU009205/2b/K029/2006),USA/JX475247/CO/
1246/2010),(USA/KJ813892/Coyote/AK/218/2013,(USA/JN867604/Dog/IL/
137654/2008,USA/JX475242/WI/18268/2002))),SAF/HQ602969/22/10SA/2010),
(USA/AY742955/436/2003,FRA/DQ025991/2b/04S22/2004)),USA/JN867602/2b/
Dog/CA/148743/2008),USA/JX475251/CO/2235/2009),USA/JN867603/2b/Dog/KS/
81213/2009),USA/KJ813852/Bobcat/ND/1170/2013),FRA/DQ025961/2b/
03C5/2003),(USA/KJ813827/Fisher/F1M111211/2013,USA/KJ813873/Graywolf/MI/
850/2012)),(ECU/KF149971/2c/ME32/2012,IND/KX469432/newCPV/2b/Hiller/
2011)),((ITA/FJ005263/42/2005,ITA/FJ005265/140/2005),((((((((((USA/
M74849/39/1995,USA/U22896/cat/1990),FRA/DQ025992/2b/04S23/2004),USA/
M74852/133/1995),POR/KU662349/greywolf/W33/1996),(POR/KU662350/greywolf/
W52/2005,POL/Z46651/46/1994)),GER/FJ005261/G162/1997),BRA/DQ340409/2b/
BR183/1985),USA/AY742932/193/1991),((USA/AY742951/431/2003,USA/

JN867605/2b/Dog/US/142805/2009),(VAC/FJ222822/2b/FortDodge/2008,(VAC/JN625223/INDIA/vac5/2011,(USA/EU659119/2b/CPV/410/2000,USA/EU659120/2b/CPV/411a/1998))))),((CHI/GQ857609/CPV08/01/2008,CHI/GU569940/2b/YN0203/2002),(((((((JPN/AB115504/2c/97/008/1997,TAW/U72696/2b/T10/1996),TAW/U72695/2a/T4/1996),(CHI/GQ857596/CPV05/01/2005,CHI/GQ857600/CPV06/01/2006)),THA/FJ869125/KU5/2004),(USA/JX475237/CT/372/2011,KOR/EF599097/2b/DH326/2006)),(CHI/EU483515/2b/ZD13/2007,(JPN/LC270891/2b/9985/2017,(JPN/AB437433/1887/M/2/2008,(TAW/FJ265781/CPV307/2005,(TWN/EF592511/TWN1/2006,TAW/FJ265775/CPV301/2004)))))),(((VIE/AB054218/2b/cat/V123/2000,VAC/FJ222823/2b/29/1997),ITA/FJ005264/134/2005),((THA/FJ869122/KU1/2008,THA/FJ869123/KU3/2008),((((((THA/KP715690/VT28/2014,THA/KP715716/VT143/2014),THA/KP715691/VT43/2014),(VIE/AB120722/2b/HCM/18/2003,VIE/AB120724/2b/HNI/2/13/2003)),(CHI/GQ857599/CPV05/04/2005,CHI/GQ857601/CPV06/02/2006)),CHI/GQ857605/CPV07/03/2007),(THA/FJ869139/KU66/2003,(VIE/AB120721/2b/HCM/8/2003,(VIE/AB054221/2b/leopard/V204/2000,(VIE/AB054224/2c/leopard/V203/2000,(VIE/AB120725/2b/HNI/3/4/2003,(VIE/AB120723/2b/HCM/23/2003,(VIE/AB120720/2b/HCM/6/2003,(VIE/AB054219/2b/cat/V209/2000,(VIE/AB054220/2b/cat/V217/2000,CHI/EU145954/2b/BJ044/20O7)))))))))))))))))),(ITA/FJ005257/54/2008,ITA/KF373611/2a/409/2010)),(NZE/AY742933/339/1993,((VIE/AB054223/2c/leopard/V140/2000,ITA/GU362932/cat11/2008),(NIG/HQ602995/15/10/2010,((((((((((FRA/DQ025947/2a/02B5/2002,FRA/DQ026001/2a/04S32/2004),FRA/DQ025962/2a/03C6/2003),ITA/KF373580/2a/581/2003),(GER/AY742935/U6/1995,FRA/DQ025945/2a/02B3/2002)),VIE/AB054215/2a/cat/V120/2000),ITA/FJ005255/333/2005),FRA/DQ025958/2a/03C2/2003),(ITA/KX434457/987/10/2010,(FRA/DQ025983/2a/04S14/2004,FRA/DQ025993/2a/04S24/2004))),(FRA/DQ025984/2a/04S15/2004,ITA/FJ005252/96/2002)),(FRA/DQ026002/2a/04S33/2004,(ITA/KF373592/2a/329/2008,(((ITA/AF393506/2a/699/2000,FRA/DQ025943/2a/01S1/2001),ITA/KF385388/2a/Sicily/X83090/2009),((CHI/GQ857612/CPV08/04/2008,CHI/GU569939/2a/YN0202/2002),(((((HUN/KF539794/H/7/2012,HUN/KF539795/H/8/2012),HUN/KF539804/H/212/2012),(HUN/KF539793/H/5/2012,HUN/KF539797/H/11/2012)),(HUN/KF539800/H/27/2012,(VIE/AB054217/2a/cat/V154/2000,HUN/KF539796/H/9/2012))),(((HUN/KF539798/H/31/2012,HUN/KF539799/H/39/2012),HUN/KF539805/H/36/2012),(ITA/AF306447/618/2000,(FRA/DQ025944/2a/02B2/2002,(NIG/HQ602992/19/10/2010,(ITA/AF306446/584/2000,(FRA/DQ025986/2a/04S17/2004,(ITA/KF373577/2a/714/2001,(FRA/DQ025982/2a/04S13/2004,ITA/FJ005253/67/2005))))))))))))))))))),((((((((THA/FJ869126/KU5/2008,THA/FJ869137/KU52/2003),THA/FJ869134/KU23/2003),CHI/DQ354068/2a/redpanda/RPPV/2004),KOR/EF599096/DH426/2005),(ITA/FJ005258/80/2008,(KOR/EF599098/2c/Pome/2006,(FRA/DQ025950/2a/02B9/2002,ITA/KX434454/29451/09/2009)))),(THA/FJ869130/KU13/2004,(THA/FJ869138/KU53/2003,CHI/KF803615/2011/BJ/B25/2011))),(CHI/GU569942/2a/JL0202/2002,CHI/GU569946/2a/JL0201/2002)),((USA/AY742953/435/2003,ITA/KF373571/2a/685/1999),(THA/FJ869128/KU11/2004,(((BRA/DQ340428/2a/BR209/1994,BRA/DQ340431/2a/BR56/1995),BRA/DQ340411/2a/BR8/1990),(BRA/DQ340422/2a/BR22/1993,(BRA/DQ340421/2a/BR597/1992,((((BRA/DQ340419/2a/BR570/1992,BRA/DQ340423/2a/BR136/1993),BRA/DQ340413/2a/BR18/1990),BRA/DQ340427/2a/BR133/1994),(BRA/DQ340414/2a/BR31/1990,(BRA/DQ340416/2a/BR47/1991,(BRA/DQ340417/2a/BR52/1991,(BRA/DQ340418/2a/BR491/1992,(BRA/DQ340424/2a/

BR137/1993,BRA/DQ340426/2a/BR84/1994)))))))))))),CHI/KF803600/2010/BJ/A68/2010),(((USA/EU659118/CPV/13/1981,CHI/GU569948/2a/CC8601/1986),JPN/D26079/1993),((BRA/DQ340407/2a/BR145/1980,BRA/DQ340408/2a/BR154/1980),(FRA/DQ025952/2a/03B12/2003,(BRA/DQ340404/2a/BR6/1980,(BRA/DQ340405/2a/BR135/1980,(BRA/DQ340410/2a/BR315/1986,(USA/M24000/FPV/CPV/31/1988,USA/M24003/FPV/CPV/15/1988)))))))),((USA/JN867599/Raccoon/KY/39552/2009,USA/JN867611/Raccoon/KY/358-B/2009),(USA/JN867610/Raccoon/VA/118-A/2007,(USA/KJ813890/Redfox/MA/197/2012,(USA/JX475284/TN/26/2011,(USA/JX475239/GA/06/2011,USA/JX475279/TN/1/2011)))))),(HUN/KF539801/H/25/2012,HUN/KF539803/H/2/2012)),(USA/KJ813870/Raccoon/TX/1/2013,(((USA/JN867598/Bobcat/KS/44/2010,USA/KJ813832/Fisher/ND/14/2013),(USA/KJ813831/Fisher/ND/17/2013,USA/KJ813835/Fisher/ND/19/2013)),(USA/JX475234/ME/258/2011,(USA/JN867618//Raccoon/WI/37/2010,(USA/JX475231/CO/280/2011,(USA/JX475248/CO/1102/2011,(USA/JX475233/SC/182-A/2011,USA/JX475246/CO/2503/2010))))))),((CHI/FJ231389/FPV/monkey/BJ-22/2008,CHI/KJ170680/raccoondog/HLJ11/1/2011),(((((((((CHI/GU392242/raccoondog/HB10/2009,CHI/GU392244/raccoondog/HB7/2009),CHI/KJ170679/raccoondog/Heb10/2/2010),CHI/GU392241/raccoondog/HB1/2009),CHI/GU392236/fox/HB1/2009),(CHI/GU392240/raccoondog/HB3/2009,(CHI/GU392239/raccoondog/HB6/2009,CHI/KJ194463/raccoondog/HeB10/3/2010))),CHI/GU392237/fox/HB2/2009),(VAC/FJ011098/Intervet/2006,(VAC/JN625222/INDIA/vac4/2011,(ITA/FJ222824/388/05/3/2005,(CHI/FJ432718/CPV/Cv/2008,(VAC/JN625219/INDIA/vac1/2011,CHI/KF803602/2010/BJ/A72/2010)))))),JPN/AB437434/1887/f/3/2008),((((((VAC/GU212790/primodog/2009,VAC/GU212791/vanguard/2009),VAC/FJ197847/Pfizer/2007),VAC/EU914139/Pfizer//2006),VAC/KY083089/Singapore/2016),USA/M19296/CPV/N/1988),(((((USA/M23255/FPV/Cornell320/1988,USA/M38245/1990),USA/EU659116/CPV/5/1979),(FIN/U22192/raccoondog/RD-80/1980,FIN/U22193/raccoondog/RD87/1987)),(USA/M10989/1985,USA/U22186/CPV/128/1995)),(VAC/JN625221/INDIA/vac3/2011,(VAC/JN625220/INDIA/vac2/2011,(((VAC/FJ011097/Merial/2006,CHI/GQ169553/Vac2/2007),VAC/KY083090/Singapore/2016),(CHI/GU569943/YB8301/1983,(VAC/JN625224/INDIA/vac6/2011,ARG/KM236572/NNGag/2012))))))))))`

>Would you like to use it (y/n)? y

>Loaded a multiple sequence alignment with **339** sequences, **581** codons, and **1** partitions from `/home/opcestocarstvo/BEAST2/CPV_VP2_cod_sel.txt`

####Choose the set of branches to test for selection

1. [**All**] Include all branches in the analysis
2. [**Internal**] Include all internal branches in the analysis
3. [**Leaves**] Include all leaf branches in the analysis
4. [**Unlabeled branches**] Set of 675 unlabeled branches

>Please choose an option (or press q to cancel selection):1

>Select the number of samples used to assess ancestral reconstruction uncertainty [select 0 to skip] (permissible range = [0,100000], default value = 100, integer): 100

>Select the p-value threshold to use when testing for selection (permissible range = [0,1], default value = 0.1): 0.1

Branches to include in the SLAC analysis
Selected 675 branches to include in SLAC calculations:
`ITA_FJ005218_2c_330_2006, ITA_FJ005233_40_2007, Node53, POR_KT275253_2c_PT036_12_2012, Node52, URU_KC196096_2c_M247_2010, URU_KM457121_2c_UY247_2010, Node57, Node51, URU_KC196086_2c_M55_2006, URU_KM457106_2c_UY55_2006, Node60, Node50, USA_JX475260_CO_704_2010, Node49, ITA_FJ005247_195_2008, Node48, ITA_FJ005226_383_2006, Node47, GER_FJ005196_2c_G7_1997, Node46, FRA_DQ025994_04S25_2004, Node45, FRA_DQ025960_03C4_2003, Node44, FRA_DQ025951_03B10_2003, Node43, FRA_DQ025954_03B14_2003, Node42, USA_KJ813848_Bobcat_ND_1162_2013, Node41, URU_KM457104_2c_UY47_2006, Node40, FRA_DQ025969_03S5_2003, Node39, USA_KJ813858_Puma_ND_F93_2013, Node38, ITA_FJ005248_219_2008, Node37, GER_FJ005199_2c_G172_1997, Node36, ITA_FJ005240_208_2007, Node35, URU_KC196085_2c_M57_2007, Node34, FRA_DQ025975_04S6_2004, Node33, USA_JX475243_ID_22772_2009, Node32, USA_JX475252_CO_1316_2010, Node31, FRA_DQ025965_03C9_2003, Node30, ECU_KF149984_2c_ME28_2012, Node29, ECU_KF149962_2c_ME1_2012, ECU_KF149963_2c_ME10_2012, ECU_KF149964_2c_ME23_2012, ECU_KF149969_2c_ME31_2012, Node88, Node86, Node84, Node28, ARG_JF414820_Arg44_2009, ARG_KM236569_Cuba_2013, ARG_JF414818_Arg32_2008, ARG_JF414821_Arg48_2009, Node95, Node93, Node91, Node27, ITA_FJ005216_2c_284_2006, ITA_KU508407_2c_25835_09_2009, ITA_KX434459_27692_1_11_2011, Node100, Node98, Node26, URU_KM457122_2c_UY258_2010, URU_KM457124_2c_UY307_2011, Node105, URU_KC196093_2c_M307_2011, Node104, URU_KC196081_2c_M95_2007, URU_KM457109_2c_UY95_2007, Node110, URU_KC196097_2c_M242_2010, URU_KM457120_2c_UY242_2010, URU_KM457123_2c_UY261_2008, Node115, Node113, Node109, Node103, Node25, URU_KM457103_2c_UY12_2006, ITA_FJ005209_2c_303_2004, ITA_FJ005251_239_2008, Node120, Node118, Node24, URU_KC196083_2c_M82_2007, URU_KM457108_2c_UY82_2007, Node123, Node23, URU_KC196107_2c_M129_2008, Node22, URU_KM457131_2c_UY368_2011, Node21, POR_KT275252_2c_PT013_12_2012, Node20, ITA_FJ005231_406_2006, Node19, ITA_FJ005214_2c_67_2006, BRA_KY073269_UFMT_2015, Node130, Node18, ARG_JF414819_Arg35_2008, Node17, USA_KJ813854_Puma_ND_F205_2013, Node16, ITA_FJ005232_411_2006, Node15, URU_KC196105_2c_M152_2008, URU_KM457113_2c_UY152_2009, Node138, ITA_FJ005212_2c_349_2004, Node137, POR_KT275255_2c_PT238_14_2014, ITA_KX434460_52238_12_2012, URU_KC196091_2c_M326_2011, URU_KM457127_2c_UY326_2011, Node147, URU_KC196102_2c_M185_2009, URU_KM457116_2c_UY185_2009, Node159, HRV_KP859577_2c_HR856_2014, ITA_KX434458_2323_11_2011, Node162, Node158, URU_KC196101_2c_M187_2009, URU_KM457117_2c_UY187_2009, Node165, Node157, AUS_KU508693_2c_LW_2015, Node156,

URU_KC196089_2c_M349_2011, URU_KM457129_2c_UY349_2011, Node169, Node155, ITA_FJ005195_2c_136_2000, Node154, FRA_DQ025976_04S7_2004, Node153, USA_JX475273_MT_909_2012, USA_KJ813888_Coyote_MT_878_2012, Node174, Node152, USA_KJ813843_Bobcat_ND_1160_2013, Node151, FRA_DQ025942_01B1_2001, FRA_DQ025964_03C8_2003, ITA_FJ005206_2c_287_2004, URU_KM457107_2c_UY72_2007, URU_KM457111_2c_UY120_2008, URU_KM457112_2c_UY135_2008, URU_KM457125_2c_UY317_2011, URU_KM457130_2c_UY354_2011, URU_KM457142_2c_UY370_2011, HRV_KP859574_2c_HR442_2014, HRV_KP859575_2c_HR774_2014, HRV_KP859576_2c_HR793_2014, AUS_KU508691_2c_HB_2015, AUS_KU508692_2c_FH_2015, ITA_KX434456_45361_09_2009, Node204, Node202, Node200, Node198, Node196, Node194, Node192, Node190, Node188, Node186, Node184, Node182, Node180, Node178, Node150, Node146, Node144, Node142, Node136, Node14, URU_KM457126_2c_UY318_2010, Node13, FRA_DQ025985_04S16_2004, ITA_FJ005205_2c_279_2004, HRV_KP859578_2c_HR859_2014, Node210, Node208, Node12, ITA_FJ222821_2c_56_2000, Node11, GER_FJ005260_G82_1997, USA_KJ813846_Bobcat_ND_974_2013, Node214, Node10, GER_AY742934_447_1995, RUS_JN033694_Laika_1993, Node218, USA_AY742936_395_1998, USA_JX475240_AZ_16382_01_1999, USA_JX475250_CO_728_2010, USA_KJ813842_Bobcat_ND_502_2013, Node225, Node223, Node221, Node217, Node9, USA_KJ813828_Fisher_F1F010712_2013, USA_KJ813881_Graywolf_MI_832_2012, Node245, USA_KJ813844_Bobcat_ND_885_2013, Node244, USA_KJ813882_Raccoon_NJ_1423_2012, Node243, USA_KJ813851_Bobcat_ND_1168_2013, Node242, USA_JX475278_AR_1069_2012, Node241, KOR_EU009205_2b_K029_2006, Node240, USA_JX475247_CO_1246_2010, Node239, USA_KJ813892_Coyote_AK_218_2013, USA_JN867604_Dog_IL_137654_2008, USA_JX475242_WI_18268_2002, Node256, Node254, Node238, SAF_HQ602969_22_10SA_2010, Node237, USA_AY742955_436_2003, FRA_DQ025991_2b_04S22_2004, Node260, Node236, USA_JN867602_2b_Dog_CA_148743_2008, Node235, USA_JX475251_CO_2235_2009, Node234, USA_JN867603_2b_Dog_KS_81213_2009, Node233, USA_KJ813852_Bobcat_ND_1170_2013, Node232, FRA_DQ025961_2b_03C5_2003, Node231, USA_KJ813827_Fisher_F1M111211_2013, USA_KJ813873_Graywolf_MI_850_2012, Node268, Node230, ECU_KF149971_2c_ME32_2012, IND_KX469432_newCPV_2b_Hiller_2011, Node271, Node229, ITA_FJ005263_42_2005, ITA_FJ005265_140_2005, Node275, USA_M74849_39_1995, USA_U22896_cat_1990, Node287, FRA_DQ025992_2b_04S23_2004, Node286, USA_M74852_133_1995, Node285, POR_KU662349_greywolf_W33_1996, Node284, POR_KU662350_greywolf_W52_2005, POL_Z46651_46_1994, Node293, Node283, GER_FJ005261_G162_1997, Node282, BRA_DQ340409_2b_BR183_1985, Node281, USA_AY742932_193_1991, Node280, USA_AY742951_431_2003, USA_JN867605_2b_Dog_US_142805_2009, Node300, VAC_FJ222822_2b_FortDodge_2008, VAC_JN625223_INDIA_vac5_2011, USA_EU659119_2b_CPV_410_2000, USA_EU659120_2b_CPV_411a_1998,

Node307, Node305, Node303, Node299, Node279,
CHI_GQ857609_CPV08_01_2008, CHI_GU569940_2b_YN0203_2002, Node311,
JPN_AB115504_2c_97_008_1997, TAW_U72696_2b_T10_1996, Node320,
TAW_U72695_2a_T4_1996, Node319, CHI_GQ857596_CPV05_01_2005,
CHI_GQ857600_CPV06_01_2006, Node324, Node318,
THA_FJ869125_KU5_2004, Node317, USA_JX475237_CT_372_2011,
KOR_EF599097_2b_DH326_2006, Node328, Node316,
CHI_EU483515_2b_ZD13_2007, JPN_LC270891_2b_9985_2017,
JPN_AB437433_1887_M_2_2008, TAW_FJ265781_CPV307_2005,
TWN_EF592511_TWN1_2006, TAW_FJ265775_CPV301_2004, Node339,
Node337, Node335, Node333, Node331, Node315,
VIE_AB054218_2b_cat_V123_2000, VAC_FJ222823_2b_29_1997, Node344,
ITA_FJ005264_134_2005, Node343, THA_FJ869122_KU1_2008,
THA_FJ869123_KU3_2008, Node349, THA_KP715690_VT28_2014,
THA_KP715716_VT143_2014, Node357, THA_KP715691_VT43_2014, Node356,
VIE_AB120722_2b_HCM_18_2003, VIE_AB120724_2b_HNI_2_13_2003,
Node361, Node355, CHI_GQ857599_CPV05_04_2005,
CHI_GQ857601_CPV06_02_2006, Node364, Node354,
CHI_GQ857605_CPV07_03_2007, Node353, THA_FJ869139_KU66_2003,
VIE_AB120721_2b_HCM_8_2003, VIE_AB054221_2b_leopard_V204_2000,
VIE_AB054224_2c_leopard_V203_2000, VIE_AB120725_2b_HNI_3_4_2003,
VIE_AB120723_2b_HCM_23_2003, VIE_AB120720_2b_HCM_6_2003,
VIE_AB054219_2b_cat_V209_2000, VIE_AB054220_2b_cat_V217_2000,
CHI_EU145954_2b_BJ044_20O7, Node384, Node382, Node380, Node378,
Node376, Node374, Node372, Node370, Node368, Node352, Node348, Node342,
Node314, Node310, Node278, Node274, Node228, Node8,
ITA_FJ005257_54_2008, ITA_KF373611_2a_409_2010, Node387, Node7,
NZE_AY742933_339_1993, VIE_AB054223_2c_leopard_V140_2000,
ITA_GU362932_cat11_2008, Node393, NIG_HQ602995_15_10_2010,
FRA_DQ025947_2a_02B5_2002, FRA_DQ026001_2a_04S32_2004, Node407,
FRA_DQ025962_2a_03C6_2003, Node406, ITA_KF373580_2a_581_2003,
Node405, GER_AY742935_U6_1995, FRA_DQ025945_2a_02B3_2002, Node412,
Node404, VIE_AB054215_2a_cat_V120_2000, Node403,
ITA_FJ005255_333_2005, Node402, FRA_DQ025958_2a_03C2_2003, Node401,
ITA_KX434457_987_10_2010, FRA_DQ025983_2a_04S14_2004,
FRA_DQ025993_2a_04S24_2004, Node420, Node418, Node400,
FRA_DQ025984_2a_04S15_2004, ITA_FJ005252_96_2002, Node423, Node399,
FRA_DQ026002_2a_04S33_2004, ITA_KF373592_2a_329_2008,
ITA_AF393506_2a_699_2000, FRA_DQ025943_2a_01S1_2001, Node432,
ITA_KF385388_2a_Sicily_X83090_2009, Node431,
CHI_GQ857612_CPV08_04_2008, CHI_GU569939_2a_YN0202_2002, Node437,
HUN_KF539794_H_7_2012, HUN_KF539795_H_8_2012, Node444,
HUN_KF539804_H_212_2012, Node443, HUN_KF539793_H_5_2012,
HUN_KF539797_H_11_2012, Node448, Node442, HUN_KF539800_H_27_2012,
VIE_AB054217_2a_cat_V154_2000, HUN_KF539796_H_9_2012, Node453,
Node451, Node441, HUN_KF539798_H_31_2012, HUN_KF539799_H_39_2012,
Node458, HUN_KF539805_H_36_2012, Node457, ITA_AF306447_618_2000,
FRA_DQ025944_2a_02B2_2002, NIG_HQ602992_19_10_2010,
ITA_AF306446_584_2000, FRA_DQ025986_2a_04S17_2004,
ITA_KF373577_2a_714_2001, FRA_DQ025982_2a_04S13_2004,

ITA_FJ005253_67_2005, Node474, Node472, Node470, Node468, Node466, Node464, Node462, Node456, Node440, Node436, Node430, Node428, Node426, Node398, Node396, Node392, Node390, Node6, THA_FJ869126_KU5_2008, THA_FJ869137_KU52_2003, Node484, THA_FJ869134_KU23_2003, Node483, CHI_DQ354068_2a_redpanda_RPPV_2004, Node482, KOR_EF599096_DH426_2005, Node481, ITA_FJ005258_80_2008, KOR_EF599098_2c_Pome_2006, FRA_DQ025950_2a_02B9_2002, ITA_KX434454_29451_09_2009, Node494, Node492, Node490, Node480, THA_FJ869130_KU13_2004, THA_FJ869138_KU53_2003, CHI_KF803615_2011_BJ_B25_2011, Node499, Node497, Node479, CHI_GU569942_2a_JL0202_2002, CHI_GU569946_2a_JL0201_2002, Node502, Node478, USA_AY742953_435_2003, ITA_KF373571_2a_685_1999, Node506, THA_FJ869128_KU11_2004, BRA_DQ340428_2a_BR209_1994, BRA_DQ340431_2a_BR56_1995, Node513, BRA_DQ340411_2a_BR8_1990, Node512, BRA_DQ340422_2a_BR22_1993, BRA_DQ340421_2a_BR597_1992, BRA_DQ340419_2a_BR570_1992, BRA_DQ340423_2a_BR136_1993, Node524, BRA_DQ340413_2a_BR18_1990, Node523, BRA_DQ340427_2a_BR133_1994, Node522, BRA_DQ340414_2a_BR31_1990, BRA_DQ340416_2a_BR47_1991, BRA_DQ340417_2a_BR52_1991, BRA_DQ340418_2a_BR491_1992, BRA_DQ340424_2a_BR137_1993, BRA_DQ340426_2a_BR84_1994, Node537, Node535, Node533, Node531, Node529, Node521, Node519, Node517, Node511, Node509, Node505, Node477, Node5, CHI_KF803600_2010_BJ_A68_2010, Node4, USA_EU659118_CPV_13_1981, CHI_GU569948_2a_CC8601_1986, Node543, JPN_D26079_1993, Node542, BRA_DQ340407_2a_BR145_1980, BRA_DQ340408_2a_BR154_1980, Node548, FRA_DQ025952_2a_03B12_2003, BRA_DQ340404_2a_BR6_1980, BRA_DQ340405_2a_BR135_1980, BRA_DQ340410_2a_BR315_1986, USA_M24000_FPV_CPV_31_1988, USA_M24003_FPV_CPV_15_1988, Node559, Node557, Node555, Node553, Node551, Node547, Node541, Node3, USA_JN867599_Raccoon_KY_39552_2009, USA_JN867611_Raccoon_KY_358_B_2009, Node563, USA_JN867610_Raccoon_VA_118_A_2007, USA_KJ813890_Redfox_MA_197_2012, USA_JX475284_TN_26_2011, USA_JX475239_GA_06_2011, USA_JX475279_TN_1_2011, Node572, Node570, Node568, Node566, Node562, Node2, HUN_KF539801_H_25_2012, HUN_KF539803_H_2_2012, Node575, Node1, USA_KJ813870_Raccoon_TX_1_2013, USA_JN867598_Bobcat_KS_44_2010, USA_KJ813832_Fisher_ND_14_2013, Node582, USA_KJ813831_Fisher_ND_17_2013, USA_KJ813835_Fisher_ND_19_2013, Node585, Node581, USA_JX475234_ME_258_2011, USA_JN867618_Raccoon_WI_37_2010, USA_JX475231_CO_280_2011, USA_JX475248_CO_1102_2011, USA_JX475233_SC_182_A_2011, USA_JX475246_CO_2503_2010, Node596, Node594, Node592, Node590, Node588, Node580, Node578, CHI_FJ231389_FPV_monkey_BJ_22_2008, CHI_KJ170680_raccoondog_HLJ11_1_2011, Node600, CHI_GU392242_raccoondog_HB10_2009, CHI_GU392244_raccoondog_HB7_2009, Node611, CHI_KJ170679_raccoondog_Heb10_2_2010, Node610, CHI_GU392241_raccoondog_HB1_2009, Node609, CHI_GU392236_fox_HB1_2009, Node608, CHI_GU392240_raccoondog_HB3_2009, CHI_GU392239_raccoondog_HB6_2009,

CHI_KJ194463_raccoondog_HeB10_3_2010, Node619, Node617, Node607, CHI_GU392237_fox_HB2_2009, Node606, VAC_FJ011098_Intervet_2006, VAC_JN625222_INDIA_vac4_2011, ITA_FJ222824_388_05_3_2005, CHI_FJ432718_CPV_Cv_2008, VAC_JN625219_INDIA_vac1_2011, CHI_KF803602_2010_BJ_A72_2010, Node631, Node629, Node627, Node625, Node623, Node605, JPN_AB437434_1887_f_3_2008, Node604, VAC_GU212790_primodog_2009, VAC_GU212791_vanguard_2009, Node640, VAC_FJ197847_Pfizer_2007, Node639, VAC_EU914139_Pfizer_2006, Node638, VAC_KY083089_Singapore_2016, Node637, USA_M19296_CPV_N_1988, Node636, USA_M23255_FPV_Cornell320_1988, USA_M38245_1990, Node651, USA_EU659116_CPV_5_1979, Node650, FIN_U22192_raccoondog_RD_80_1980, FIN_U22193_raccoondog_RD87_1987, Node655, Node649, USA_M10989_1985, USA_U22186_CPV_128_1995, Node658, Node648, VAC_JN625221_INDIA_vac3_2011, VAC_JN625220_INDIA_vac2_2011, VAC_FJ011097_Merial_2006, CHI_GQ169553_Vac2_2007, Node667, VAC_KY083090_Singapore_2016, Node666, CHI_GU569943_YB8301_1983, VAC_JN625224_INDIA_vac6_2011, ARG_KM236572_NNGag_2012, Node673, Node671, Node665, Node663, Node661, Node647, Node635, Node603, Node599`

Obtaining branch lengths and nucleotide substitution biases under the nucleotide GTR model
* Log(L) = -6510.63, AIC-c = 14388.83 (683 estimated parameters)

Obtaining the global omega estimate based on relative GTR branch lengths and nucleotide substitution biases
* Log(L) = -6211.05, AIC-c = 13806.96 (690 estimated parameters)
* non-synonymous/synonymous rate ratio for *test* = 0.1167

Performing joint maximum likelihood ancestral state reconstruction

For partition 1 these sites are significant at p <=0.1

Codon	Partition	S	N	dS	dN	Selection detected?
12	1	9.000	0.000	11.697	0.000	Neg. p = 0.000
20	1	5.000	0.000	5.000	0.000	Neg. p = 0.004
32	1	3.000	0.000	3.000	0.000	Neg. p = 0.037
45	1	3.000	0.000	4.588	0.000	Neg. p = 0.010
48	1	4.000	0.000	5.003	0.000	Neg. p = 0.013
49	1	7.000	0.000	7.000	0.000	Neg. p = 0.000
60	1	2.000	0.000	2.616	0.000	Neg. p

						= 0.071
101	1	6.000	3.000	6.252	1.470	Neg. p = 0.035
135	1	3.000	0.000	3.752	0.000	Neg. p = 0.021
142	1	2.000	0.000	2.615	0.000	Neg. p = 0.071
168	1	2.000	0.000	3.062	0.000	Neg. p = 0.047
179	1	4.000	0.000	6.123	0.000	Neg. p = 0.002
204	1	1.000	0.000	4.225	0.000	Neg. p = 0.079
216	1	3.000	1.000	3.322	0.500	Neg. p = 0.092
240	1	4.000	0.000	6.124	0.000	Neg. p = 0.002
243	1	2.000	0.000	3.062	0.000	Neg. p = 0.047
252	1	4.000	0.000	4.000	0.000	Neg. p = 0.012
300	1	1.000	10.000	1.023	4.946	Pos. p = 0.082
316	1	4.000	2.000	4.190	0.978	Neg. p = 0.086
325	1	2.000	0.000	2.466	0.000	Neg. p = 0.073
335	1	2.000	0.000	2.501	0.000	Neg. p = 0.077
342	1	2.000	0.000	3.062	0.000	Neg. p = 0.061
364	1	8.000	0.000	8.000	0.000	Neg. p = 0.000
382	1	2.000	0.000	2.215	0.000	Neg. p = 0.097
396	1	2.000	0.000	2.501	0.000	Neg. p = 0.077
416	1	5.000	0.000	6.535	0.000	Neg. p = 0.004
425	1	3.000	0.000	3.000	0.000	Neg. p = 0.037
430	1	4.000	0.000	2.753	0.000	Neg. p = 0.068
440	1	0.000	6.000	0.000	3.001	Pos. p = 0.088
445	1	3.000	0.000	3.000	0.000	Neg. p = 0.037
494	1	3.000	0.000	4.593	0.000	Neg. p = 0.012
503	1	4.000	0.000	4.000	0.000	Neg. p

= 0.012 |

507	1	4.000	0.000	4.000	0.000	Neg. p = 0.012
522	1	3.000	0.000	3.000	0.000	Neg. p = 0.037
532	1	2.000	0.000	2.615	0.000	Neg. p = 0.069
552	1	2.000	0.000	3.062	0.000	Neg. p = 0.047
553	1	4.000	0.000	4.904	0.000	Neg. p = 0.005
572	1	10.000	0.000	10.000	0.000	Neg. p = 0.000
574	1	3.000	0.000	3.922	0.000	Neg. p = 0.019

Ancestor sampling analysis

>Generating 100 ancestral sequence samples to obtain confidence intervals

Done with ancestral sampling
Resampling results for partition 1

Codon	Partition	S [median, IQR]	N [median, IQR]	dS [median, IQR]	dN [median, IQR]	p-value [median, IQR]
12	1	9.00 [9.00-9.00]	0.00 [0.00-0.00]	11.70 [11.70-11.70]	0.00 [0.00-0.00]	0.00 [0.00-0.00]
20	1	5.00 [5.00-5.00]	0.00 [0.00-0.00]	5.00 [5.00-5.00]	0.00 [0.00-0.00]	0.00 [0.00-0.00]
32	1	3.00 [3.00-3.00]	0.00 [0.00-0.00]	3.00 [3.00-3.00]	0.00 [0.00-0.00]	0.04 [0.04-0.04]
45	1	3.00 [3.00-3.00]	0.00 [0.00-0.00]	4.59 [4.59-4.59]	0.00 [0.00-0.00]	0.01 [0.01-0.01]
48	1	4.00 [4.00-4.00]	0.00 [0.00-0.00]	5.00 [5.00-5.00]	0.00 [0.00-0.00]	0.01 [0.01-0.01]
49	1	7.00 [7.00-7.00]	0.00 [0.00-0.00]	7.00 [7.00-7.00]	0.00 [0.00-0.00]	0.00 [0.00-0.00]
60	1	2.00 [2.00-2.00]	0.00 [0.00-0.00]	2.62 [2.62-2.62]	0.00 [0.00-0.00]	0.07 [0.07-0.07]
101	1	6.00 [6.00-6.00]	3.00 [3.00-3.00]	6.25 [6.25-6.25]	1.47 [1.47-1.47]	0.03 [0.03-0.03]
135	1	3.00 [3.00-3.00]	0.00 [0.00-0.00]	3.75 [3.75-3.75]	0.00 [0.00-0.00]	0.02 [0.02-0.02]
142	1	2.00 [2.00-2.00]	0.00 [0.00-0.00]	2.62 [2.62-2.62]	0.00 [0.00-0.00]	0.07 [0.07-0.07]
168	1	2.00 [2.00-2.00]	0.00 [0.00-0.00]	3.06 [3.06-3.06]	0.00 [0.00-0.00]	0.05 [0.05-0.05]
179	1	4.00 [4.00-4.00]	0.00 [0.00-0.00]	6.12 [6.12-6.12]		

					0.00 [0.00-0.00]	0.00 [0.00-0.00]
204	1	1.00 [1.00-1.00]	0.00 [0.00-0.00]	4.22 [4.22-4.22]	0.00 [0.00-0.00]	0.08 [0.08-0.08]
216	1	3.00 [3.00-3.00]	1.00 [1.00-1.00]	3.32 [3.32-3.32]	0.50 [0.50-0.50]	0.09 [0.09-0.09]
240	1	4.00 [4.00-4.00]	0.00 [0.00-0.00]	6.12 [6.12-6.12]	0.00 [0.00-0.00]	0.00 [0.00-0.00]
243	1	2.00 [2.00-2.00]	0.00 [0.00-0.00]	3.06 [3.06-3.06]	0.00 [0.00-0.00]	0.05 [0.05-0.05]
252	1	4.00 [4.00-4.00]	0.00 [0.00-0.00]	4.00 [4.00-4.00]	0.00 [0.00-0.00]	0.01 [0.01-0.01]
300	1	1.00 [1.00-1.00]	10.00 [10.00-10.00]	1.02 [1.02-1.03]	4.95 [4.94-4.95]	0.08 [0.08-0.08]
316	1	4.00 [4.00-4.00]	2.00 [2.00-2.00]	4.19 [4.19-4.19]	0.98 [0.98-0.98]	0.09 [0.09-0.09]
325	1	2.00 [2.00-2.00]	0.00 [0.00-0.00]	2.47 [2.47-2.47]	0.00 [0.00-0.00]	0.07 [0.07-0.07]
335	1	2.00 [2.00-2.00]	0.00 [0.00-0.00]	2.50 [2.50-2.50]	0.00 [0.00-0.00]	0.08 [0.08-0.08]
342	1	2.00 [2.00-2.00]	0.00 [0.00-0.00]	3.06 [3.06-3.06]	0.00 [0.00-0.00]	0.06 [0.06-0.06]
364	1	8.00 [8.00-8.00]	0.00 [0.00-0.00]	8.00 [8.00-8.00]	0.00 [0.00-0.00]	0.00 [0.00-0.00]
382	1	2.00 [2.00-2.00]	0.00 [0.00-0.00]	2.21 [2.21-2.21]	0.00 [0.00-0.00]	0.10 [0.10-0.10]
396	1	2.00 [2.00-2.00]	0.00 [0.00-0.00]	2.50 [2.50-2.50]	0.00 [0.00-0.00]	0.08 [0.08-0.08]
416	1	5.00 [5.00-5.16]	0.00 [0.00-0.00]	6.54 [6.54-6.74]	0.00 [0.00-0.00]	0.00 [0.00-0.00]
425	1	3.00 [3.00-3.00]	0.00 [0.00-0.00]	3.00 [3.00-3.00]	0.00 [0.00-0.00]	0.04 [0.04-0.04]
430	1	4.00 [4.00-4.00]	0.00 [0.00-0.00]	2.75 [2.75-2.75]	0.00 [0.00-0.00]	0.07 [0.07-0.07]
440	1	0.00 [0.00-0.00]	6.00 [6.00-6.00]	0.00 [0.00-0.00]	3.00 [3.00-3.00]	0.09 [0.09-0.09]
445	1	3.00 [3.00-3.00]	0.00 [0.00-0.00]	3.00 [3.00-3.00]	0.00 [0.00-0.00]	0.04 [0.04-0.04]
494	1	3.00 [3.00-3.00]	0.00 [0.00-0.00]	4.59 [4.59-4.59]	0.00 [0.00-0.00]	0.01 [0.01-0.01]
503	1	4.00 [4.00-4.00]	0.00 [0.00-0.00]	4.00 [4.00-4.00]	0.00 [0.00-0.00]	0.01 [0.01-0.01]
507	1	4.00 [4.00-4.00]	0.00 [0.00-0.00]	4.00 [4.00-4.00]	0.00 [0.00-0.00]	0.01 [0.01-0.01]
522	1	3.00 [3.00-3.00]	0.00 [0.00-0.00]	3.00 [3.00-3.00]	0.00 [0.00-0.00]	0.04 [0.04-0.04]
532	1	2.00 [2.00-2.00]	0.00 [0.00-0.00]	2.62 [2.62-2.62]	0.00 [0.00-0.00]	0.07 [0.07-0.07]
552	1	2.00 [2.00-2.00]	0.00 [0.00-0.00]	3.06 [3.06-3.06]	0.00 [0.00-0.00]	0.05 [0.05-0.05]
553	1	4.00 [4.00-4.00]	0.00 [0.00-0.00]	4.90 [4.90-4.90]		

l   0.00 [0.00-0.00]   l   0.01 [0.01-0.01]   l
l   572   l   1   l 10.00 [10.00-10.00] l   0.00 [0.00-0.00]   l 10.00
[10.00-10.00]   l   0.00 [0.00-0.00]   l   0.00 [0.00-0.00]   l
l   574   l   1   l   3.00 [3.00-3.00]   l   0.00 [0.00-0.00]   l   3.92 [3.92-3.92]
l   0.00 [0.00-0.00]   l   0.02 [0.02-0.02]   l
