# Supplementary material for: Evidence of CPV2c introgression into Croatia and novel insights into phylogeny and cell tropism
Source: Sci Rep. 2019 Nov 15;9:16909. doi: 10.1038/s41598-019-53422-9 (PMC6858334; doi:10.1038/s41598-019-53422-9)
Supplement: Supplementary file 5 — A Fast, Unconstrained Bayesian AppRoximation for Inferring Selection [file 41598_2019_53422_MOESM5_ESM.pdf]

Evidence of CPV2c introgression into Croatia and novel insights into phylogeny and cell tropism

Dinko Novosel, Tamas Tuboly, Gyula Balka, Levente Szeredi, Ivana Lojkic, Andreja Jungic, Zaklin Acinger Rogic, Tahar Ait Ali, Attila Csagola

Supplementary info file 5.

Results of selection using A Fast, Unconstrained Bayesian AppRoximation for Inferring Selection method

```
   /HYPHY 2.3.13.20180601beta(MPI) for Darwin on x86_64\
***************** TYPES OF STANDARD ANALYSES *****************


     (1) Selection Analyses
     (2) Evolutionary Hypothesis Testing
     (3) Relative evolutionary rate inference
     (4) Basic Analyses
     (5) Codon Selection Analyses
     (6) Compartmentalization
     (7) Data File Tools
     (8) Miscellaneous
     (9) Model Comparison
     (10) Kernel Analysis Tools
     (11) Molecular Clock
     (12) Phylogeny Reconstruction
     (13) Positive Selection
     (14) Recombination
     (15) Selection/Recombination
     (16) Relative Rate
     (17) Relative Ratio
     (18) Substitution Rates

1
 Please select type of analyses you want to list (or press ENTER to
process custom batch file):


***************** FILES IN 'Selection Analyses' *****************


     (1) [MEME] Test for episodic site-level selection using MEME
(Mixed Effects Model of Evolution).
```

```
    (2) [FEL] Test for pervasive site-level selection using FEL
(Fixed Effects Likelihood).
    (3) [SLAC] Test for pervasive site-level selection using SLAC
(Single Likelihood Ancestor Counting).
    (4) [FUBAR] Test for pervasive site-level selection using FUBAR
(Fast Unconstrained Bayesian AppRoximation for inferring selection).
    (5) [BUSTED] Test for episodic gene-wide selection using BUSTED
(Branch-site Unrestricted Statistical Test of Episodic
Diversification).
    (6) [aBSREL] Test for lineage-specific evolution using the
branch-site method aBS-REL (Adaptive Branch-Site Random Effects
Likelihood).
    (7) [RELAX] Test for relaxation of selection pressure along a
specified set of test branches using RELAX (a random effects test of
selection relaxation).

 Please select the analysis you would like to perform (or press
ENTER to return to the list of analysis types):4

Analysis Description
--------------------
Perform a Fast Unbiased AppRoximate Bayesian (FUBAR) analysis of a
coding sequence alignment to determine whether some sites have been
subject to pervasive purifying or diversifying selection. v2.1
introduces two more methods for estimating the posterior
distribution of
grid weights: collapsed Gibbs MCMC (faster) and 0-th order Variation
Bayes approximation (fastest). Please note that a FUBAR analysis
generates a cache and a results JSON file in the same directory as
directory as the original alignment. HyPhy needs to have write
privileges to this directory. For example if the original file is in
/home/sergei/FUBAR/data/pol.nex then at the end of a FUBAR run,
there
will also exist FUBAR-generated files
/home/sergei/FUBAR/data/pol.nex.FUBAR.json,
/home/sergei/FUBAR/data/pol.nex.fubrar.cache. They also provide
checkpointing so that a partially completed analysis can be
restarted.

- __Requirements__: in-frame codon alignment (possibly partitioned)
and a phylogenetic tree
(one per partition)

- __Citation__: FUBAR: a fast, unconstrained bayesian approximation
for inferring
selection (2013), Mol Biol Evol. 30(5):1196-205

- __Written by__: Sergei L Kosakovsky Pond

- __Contact Information__: spond@temple.edu

- __Analysis Version__: 2.1
```

####Choose Genetic Code

1. [**Universal**] Universal code. (Genebank transl_table=1).
2. [**Vertebrate mtDNA**] Vertebrate mitochondrial DNA code. (Genebank transl_table=2).
3. [**Yeast mtDNA**] Yeast mitochondrial DNA code. (Genebank transl_table=3).
4. [**Mold/Protozoan mtDNA**] Mold, Protozoan and Coelenterate mitochondrial DNA and the Mycloplasma/Spiroplasma code. (Genebank transl_table=4).
5. [**Invertebrate mtDNA**] Invertebrate mitochondrial DNA code. (Genebank transl_table=5).
6. [**Ciliate Nuclear**] Ciliate, Dasycladacean and Hexamita Nuclear code. (Genebank transl_table=6).
7. [**Echinoderm mtDNA**] Echinoderm mitochondrial DNA code. (Genebank transl_table=9).
8. [**Euplotid Nuclear**] Euplotid Nuclear code. (Genebank transl_table=10).
9. [**Alt. Yeast Nuclear**] Alternative Yeast Nuclear code. (Genebank transl_table=12).
10. [**Ascidian mtDNA**] Ascidian mitochondrial DNA code. (Genebank transl_table=13).
11. [**Flatworm mtDNA**] Flatworm mitochondrial DNA code. (Genebank transl_table=14).
12. [**Blepharisma Nuclear**] Blepharisma Nuclear code. (Genebank transl_table=15).
13. [**Chlorophycean mtDNA**] Chlorophycean Mitochondrial Code (transl_table=16).
14. [**Trematode mtDNA**] Trematode Mitochondrial Code (transl_table=21).
15. [**Scenedesmus obliquus mtDNA**] Scenedesmus obliquus mitochondrial Code (transl_table=22).
16. [**Thraustochytrium mtDNA**] Thraustochytrium Mitochondrial Code (transl_table=23).
17. [**Pterobranchia mtDNA**] Pterobranchia Mitochondrial Code (transl_table=24).
18. [**SR1 and Gracilibacteria**] Candidate Division SR1 and Gracilibacteria Code (transl_table=25).
19. [**Pachysolen Nuclear**] Pachysolen tannophilus Nuclear Code (transl_table=26).

>Please choose an option (or press q to cancel selection):1

>Select a coding sequence alignment file (`/Users/dinkonovosel/hyphy/res/TemplateBatchFiles/SelectionAnalyses/`) /Users/dinkonovosel/CPV_VP2_cod_sel.txt

>A tree was found in the data file:
`((ITA/FJ005218/2c/330/2006,ITA/FJ005233/40/2007),POR/KT275253/2c/PT036/12/2012),(URU/KC196096/2c/M247/2010,URU/KM457121/2c/

UY247/2010)),(URU/KC196086/2c/M55/2006,URU/KM457106/2c/
UY55/2006)),USA/JX475260/CO/704/2010),ITA/FJ005247/195/2008),ITA/
FJ005226/383/2006),GER/FJ005196/2c/G7/1997),FRA/
DQ025994/04S25/2004),FRA/DQ025960/03C4/2003),FRA/
DQ025951/03B10/2003),FRA/DQ025954/03B14/2003),USA/KJ813848/Bobcat/
ND/1162/2013),URU/KM457104/2c/UY47/2006),FRA/
DQ025969/03S5/2003),USA/KJ813858/Puma/ND/F93/2013),ITA/
FJ005248/219/2008),GER/FJ005199/2c/G172/1997),ITA/
FJ005240/208/2007),URU/KC196085/2c/M57/2007),FRA/
DQ025975/04S6/2004),USA/JX475243/ID/22772/2009),USA/JX475252/CO/
1316/2010),FRA/DQ025965/03C9/2003),ECU/KF149984/2c/ME28/2012),(ECU/
KF149962/2c/ME1/2012,(ECU/KF149963/2c/ME10/2012,(ECU/KF149964/2c/
ME23/2012,ECU/KF149969/2c/ME31/2012)))),(ARG/JF414820/Arg44/2009,
(ARG/KM236569/Cuba/2013,(ARG/JF414818/Arg32/2008,ARG/JF414821/
Arg48/2009)))),(ITA/FJ005216/2c/284/2006,(ITA/KU508407/2c/
25835/09/2009,ITA/KX434459/27692/1/11/2011))),(((URU/KM457122/2c/
UY258/2010,URU/KM457124/2c/UY307/2011),URU/KC196093/2c/M307/2011),
((URU/KC196081/2c/M95/2007,URU/KM457109/2c/UY95/2007),(URU/
KC196097/2c/M242/2010,(URU/KM457120/2c/UY242/2010,URU/KM457123/2c/
UY261/2008))))),(URU/KM457103/2c/UY12/2006,(ITA/FJ005209/2c/
303/2004,ITA/FJ005251/239/2008))),(URU/KC196083/2c/M82/2007,URU/
KM457108/2c/UY82/2007)),URU/KC196107/2c/M129/2008),URU/KM457131/2c/
UY368/2011),POR/KT275252/2c/PT013/12/2012),ITA/FJ005231/406/2006),
(ITA/FJ005214/2c/67/2006,BRA/KY073269/UFMT/2015)),ARG/JF414819/
Arg35/2008),USA/KJ813854/Puma/ND/F205/2013),ITA/FJ005232/411/2006),
(((URU/KC196105/2c/M152/2008,URU/KM457113/2c/UY152/2009),ITA/
FJ005212/2c/349/2004),(POR/KT275255/2c/PT238/14/2014,(ITA/
KX434460/52238/12/2012,((URU/KC196091/2c/M326/2011,URU/KM457127/2c/
UY326/2011),(((((((((URU/KC196102/2c/M185/2009,URU/KM457116/2c/
UY185/2009),(HRV/KP859577/2c/HR856/2014,ITA/KX434458/2323/11/2011)),
(URU/KC196101/2c/M187/2009,URU/KM457117/2c/UY187/2009)),AUS/
KU508693/2c/LW/2015),(URU/KC196089/2c/M349/2011,URU/KM457129/2c/
UY349/2011)),ITA/FJ005195/2c/136/2000),FRA/DQ025976/04S7/2004),(USA/
JX475273/MT/909/2012,USA/KJ813888/Coyote/MT/878/2012)),USA/KJ813843/
Bobcat/ND/1160/2013),(FRA/DQ025942/01B1/2001,(FRA/
DQ025964/03C8/2003,(ITA/FJ005206/2c/287/2004,(URU/KM457107/2c/
UY72/2007,(URU/KM457111/2c/UY120/2008,(URU/KM457112/2c/UY135/2008,
(URU/KM457125/2c/UY317/2011,(URU/KM457130/2c/UY354/2011,(URU/
KM457142/2c/UY370/2011,(HRV/KP859574/2c/HR442/2014,(HRV/KP859575/2c/
HR774/2014,(HRV/KP859576/2c/HR793/2014,(AUS/KU508691/2c/HB/2015,
(AUS/KU508692/2c/FH/2015,ITA/
KX434456/45361/09/2009))))))))))))))))))),URU/KM457126/2c/
UY318/2010),(FRA/DQ025985/04S16/2004,(ITA/FJ005205/2c/279/2004,HRV/
KP859578/2c/HR859/2014))),ITA/FJ222821/2c/56/2000),(GER/FJ005260/
G82/1997,USA/KJ813846/Bobcat/ND/974/2013)),((GER/
AY742934/447/1995,RUS/JN033694/Laika/1993),(USA/AY742936/395/1998,
(USA/JX475240/AZ/16382-01/1999,(USA/JX475250/CO/728/2010,USA/
KJ813842/Bobcat/ND/502/2013))))),(((((((((((((((USA/KJ813828/
Fisher/F1F010712/2013,USA/KJ813881/Graywolf/MI/832/2012),USA/
KJ813844/Bobcat/ND/885/2013),USA/KJ813882/Raccoon/NJ/1423/2012),USA/
KJ813851/Bobcat/ND/1168/2013),USA/JX475278/AR/1069/2012),KOR/
EU009205/2b/K029/2006),USA/JX475247/CO/1246/2010),(USA/KJ813892/
Coyote/AK/218/2013,(USA/JN867604/Dog/IL/137654/2008,USA/JX475242/WI/
18268/2002))),SAF/HQ602969/22/10SA/2010),(USA/AY742955/436/2003,FRA/

DQ025991/2b/04S22/2004)),USA/JN867602/2b/Dog/CA/148743/2008),USA/
JX475251/CO/2235/2009),USA/JN867603/2b/Dog/KS/81213/2009),USA/
KJ813852/Bobcat/ND/1170/2013),FRA/DQ025961/2b/03C5/2003),(USA/
KJ813827/Fisher/F1M111211/2013,USA/KJ813873/Graywolf/MI/850/2012)),
(ECU/KF149971/2c/ME32/2012,IND/KX469432/newCPV/2b/Hiller/2011)),
((ITA/FJ005263/42/2005,ITA/FJ005265/140/2005),(((((((((USA/
M74849/39/1995,USA/U22896/cat/1990),FRA/DQ025992/2b/04S23/2004),USA/
M74852/133/1995),POR/KU662349/greywolf/W33/1996),(POR/KU662350/
greywolf/W52/2005,POL/Z46651/46/1994)),GER/FJ005261/G162/1997),BRA/
DQ340409/2b/BR183/1985),USA/AY742932/193/1991),((USA/
AY742951/431/2003,USA/JN867605/2b/Dog/US/142805/2009),(VAC/
FJ222822/2b/FortDodge/2008,(VAC/JN625223/INDIA/vac5/2011,(USA/
EU659119/2b/CPV/410/2000,USA/EU659120/2b/CPV/411a/1998))))),((CHI/
GQ857609/CPV08/01/2008,CHI/GU569940/2b/YN0203/2002),((((((JPN/
AB115504/2c/97/008/1997,TAW/U72696/2b/T10/1996),TAW/U72695/2a/
T4/1996),(CHI/GQ857596/CPV05/01/2005,CHI/GQ857600/
CPV06/01/2006)),THA/FJ869125/KU5/2004),(USA/JX475237/CT/
372/2011,KOR/EF599097/2b/DH326/2006)),(CHI/EU483515/2b/ZD13/2007,
(JPN/LC270891/2b/9985/2017,(JPN/AB437433/1887/M/2/2008,(TAW/
FJ265781/CPV307/2005,(TWN/EF592511/TWN1/2006,TAW/FJ265775/
CPV301/2004)))))),(((VIE/AB054218/2b/cat/V123/2000,VAC/FJ222823/2b/
29/1997),ITA/FJ005264/134/2005),((THA/FJ869122/KU1/2008,THA/
FJ869123/KU3/2008),((((((THA/KP715690/VT28/2014,THA/KP715716/
VT143/2014),THA/KP715691/VT43/2014),(VIE/AB120722/2b/HCM/
18/2003,VIE/AB120724/2b/HNI/2/13/2003)),(CHI/GQ857599/
CPV05/04/2005,CHI/GQ857601/CPV06/02/2006)),CHI/GQ857605/
CPV07/03/2007),(THA/FJ869139/KU66/2003,(VIE/AB120721/2b/HCM/8/2003,
(VIE/AB054221/2b/leopard/V204/2000,(VIE/AB054224/2c/leopard/
V203/2000,(VIE/AB120725/2b/HNI/3/4/2003,(VIE/AB120723/2b/HCM/
23/2003,(VIE/AB120720/2b/HCM/6/2003,(VIE/AB054219/2b/cat/V209/2000,
(VIE/AB054220/2b/cat/V217/2000,CHI/EU145954/2b/
BJ044/2007))))))))))))))))),(ITA/FJ005257/54/2008,ITA/KF373611/2a/
409/2010)),(NZE/AY742933/339/1993,((VIE/AB054223/2c/leopard/
V140/2000,ITA/GU362932/cat11/2008),(NIG/HQ602995/15/10/2010,
(((((((((FRA/DQ025947/2a/02B5/2002,FRA/DQ026001/2a/04S32/2004),FRA/
DQ025962/2a/03C6/2003),ITA/KF373580/2a/581/2003),(GER/AY742935/
U6/1995,FRA/DQ025945/2a/02B3/2002)),VIE/AB054215/2a/cat/
V120/2000),ITA/FJ005255/333/2005),FRA/DQ025958/2a/03C2/2003),(ITA/
KX434457/987/10/2010,(FRA/DQ025983/2a/04S14/2004,FRA/DQ025993/2a/
04S24/2004))),(FRA/DQ025984/2a/04S15/2004,ITA/FJ005252/96/2002)),
(FRA/DQ026002/2a/04S33/2004,(ITA/KF373592/2a/329/2008,(((ITA/
AF393506/2a/699/2000,FRA/DQ025943/2a/01S1/2001),ITA/KF385388/2a/
Sicily/X83090/2009),((CHI/GQ857612/CPV08/04/2008,CHI/GU569939/2a/
YN0202/2002),((((HUN/KF539794/H/7/2012,HUN/KF539795/H/8/2012),HUN/
KF539804/H/212/2012),(HUN/KF539793/H/5/2012,HUN/KF539797/H/
11/2012)),(HUN/KF539800/H/27/2012,(VIE/AB054217/2a/cat/
V154/2000,HUN/KF539796/H/9/2012))),(((HUN/KF539798/H/31/2012,HUN/
KF539799/H/39/2012),HUN/KF539805/H/36/2012),(ITA/AF306447/618/2000,
(FRA/DQ025944/2a/02B2/2002,(NIG/HQ602992/19/10/2010,(ITA/
AF306446/584/2000,(FRA/DQ025986/2a/04S17/2004,(ITA/KF373577/2a/
714/2001,(FRA/DQ025982/2a/04S13/2004,ITA/
FJ005253/67/2005)))))))))))))))),(((((((THA/FJ869126/
KU5/2008,THA/FJ869137/KU52/2003),THA/FJ869134/KU23/2003),CHI/
DQ354068/2a/redpanda/RPPV/2004),KOR/EF599096/DH426/2005),(ITA/

FJ005258/80/2008,(KOR/EF599098/2c/Pome/2006,(FRA/DQ025950/2a/
02B9/2002,ITA/KX434454/29451/09/2009)))),(THA/FJ869130/KU13/2004,
(THA/FJ869138/KU53/2003,CHI/KF803615/2011/BJ/B25/2011))),(CHI/
GU569942/2a/JL0202/2002,CHI/GU569946/2a/JL0201/2002)),((USA/
AY742953/435/2003,ITA/KF373571/2a/685/1999),(THA/FJ869128/KU11/2004,
(((BRA/DQ340428/2a/BR209/1994,BRA/DQ340431/2a/BR56/1995),BRA/
DQ340411/2a/BR8/1990),(BRA/DQ340422/2a/BR22/1993,(BRA/DQ340421/2a/
BR597/1992,((((BRA/DQ340419/2a/BR570/1992,BRA/DQ340423/2a/
BR136/1993),BRA/DQ340413/2a/BR18/1990),BRA/DQ340427/2a/BR133/1994),
(BRA/DQ340414/2a/BR31/1990,(BRA/DQ340416/2a/BR47/1991,(BRA/
DQ340417/2a/BR52/1991,(BRA/DQ340418/2a/BR491/1992,(BRA/DQ340424/2a/
BR137/1993,BRA/DQ340426/2a/BR84/1994)))))))))))),CHI/KF803600/2010/
BJ/A68/2010),(((USA/EU659118/CPV/13/1981,CHI/GU569948/2a/
CC8601/1986),JPN/D26079/1993),((BRA/DQ340407/2a/BR145/1980,BRA/
DQ340408/2a/BR154/1980),(FRA/DQ025952/2a/03B12/2003,(BRA/
DQ340404/2a/BR6/1980,(BRA/DQ340405/2a/BR135/1980,(BRA/DQ340410/2a/
BR315/1986,(USA/M24000/FPV/CPV/31/1988,USA/M24003/FPV/CPV/
15/1988)))))))),((USA/JN867599/Raccoon/KY/39552/2009,USA/JN867611/
Raccoon/KY/358-B/2009),(USA/JN867610/Raccoon/VA/118-A/2007,(USA/
KJ813890/Redfox/MA/197/2012,(USA/JX475284/TN/26/2011,(USA/JX475239/
GA/06/2011,USA/JX475279/TN/1/2011)))))),(HUN/KF539801/H/25/2012,HUN/
KF539803/H/2/2012)),(USA/KJ813870/Raccoon/TX/1/2013,(((USA/JN867598/
Bobcat/KS/44/2010,USA/KJ813832/Fisher/ND/14/2013),(USA/KJ813831/
Fisher/ND/17/2013,USA/KJ813835/Fisher/ND/19/2013)),(USA/JX475234/ME/
258/2011,(USA/JN867618//Raccoon/WI/37/2010,(USA/JX475231/CO/
280/2011,(USA/JX475248/CO/1102/2011,(USA/JX475233/SC/182-A/2011,USA/
JX475246/CO/2503/2010))))))),((CHI/FJ231389/FPV/monkey/
BJ-22/2008,CHI/KJ170680/raccoondog/HLJ11/1/2011),((((((((CHI/
GU392242/raccoondog/HB10/2009,CHI/GU392244/raccoondog/HB7/2009),CHI/
KJ170679/raccoondog/Heb10/2/2010),CHI/GU392241/raccoondog/
HB1/2009),CHI/GU392236/fox/HB1/2009),(CHI/GU392240/raccoondog/
HB3/2009,(CHI/GU392239/raccoondog/HB6/2009,CHI/KJ194463/raccoondog/
HeB10/3/2010))),CHI/GU392237/fox/HB2/2009),(VAC/FJ011098/Intervet/
2006,(VAC/JN625222/INDIA/vac4/2011,(ITA/FJ222824/388/05/3/2005,(CHI/
FJ432718/CPV/Cv/2008,(VAC/JN625219/INDIA/vac1/2011,CHI/
KF803602/2010/BJ/A72/2010)))))),JPN/AB437434/1887/f/3/2008),
((((((VAC/GU212790/primodog/2009,VAC/GU212791/vanguard/2009),VAC/
FJ197847/Pfizer/2007),VAC/EU914139/Pfizer//2006),VAC/KY083089/
Singapore/2016),USA/M19296/CPV/N/1988),(((((USA/M23255/FPV/
Cornell320/1988,USA/M38245/1990),USA/EU659116/CPV/5/1979),(FIN/
U22192/raccoondog/RD-80/1980,FIN/U22193/raccoondog/RD87/1987)),(USA/
M10989/1985,USA/U22186/CPV/128/1995)),(VAC/JN625221/INDIA/vac3/2011,
(VAC/JN625220/INDIA/vac2/2011,(((VAC/FJ011097/Merial/2006,CHI/
GQ169553/Vac2/2007),VAC/KY083090/Singapore/2016),(CHI/GU569943/
YB8301/1983,(VAC/JN625224/INDIA/vac6/2011,ARG/KM236572/NNGag/
2012))))))))))`

>Would you like to use it (y/n)? y

>Loaded a multiple sequence alignment with **339** sequences,
581 codons, and **1** partitions from `/Users/dinkonovosel/
CPV_VP2_cod_sel.txt`
> FUBAR will write cache and result files to _/Users/dinkonovosel/

CPV_VP2_cod_sel.txt.FUBAR.cache_ and _/Users/dinkonovosel/CPV_VP2_cod_sel.txt.FUBAR.json_, respectively

> Number of grid points per dimension (total number is D^2) (permissible range = [5,50], default value = 20, integer): 50

####Posterior estimation method

1. [**Metropolis-Hastings**] Full Metropolis-Hastings MCMC algorithm (slowest, original 2013 paper implementation)
2. [**Collapsed Gibbs**] Collapsed Gibbs sampler (intermediate speed)
3. [**Variational Bayes**] 0-th order Variational Bayes approximations (fastest, recommended default)

>Please choose an option (or press q to cancel selection):1
> Number of MCMC chains to run (permissible range = [2,20], default value = 5, integer): 10
> The length of each chain (permissible range = [5000,50000000], default value = 2000000, integer): 2000000
> Use this many samples as burn-in (permissible range = [100000,1900000], default value = 1000000, integer): 1000000
> How many samples should be drawn from each chain (permissible range = [50,1000000], default value = 100, integer): 200
> The concentration parameter of the Dirichlet prior (permissible range = [0.001,1], default value = 0.5): 0.5

Obtaining branch lengths and nucleotide substitution biases under the nucleotide GTR model
* Log(L) = -6510.63, AIC-c = 14388.84 (683 estimated parameters)
* Tree length (expected substitutions/site) for partition 1 : 0.278

Computing the phylogenetic likelihood function on the grid
* Determining appropriate tree scaling based on the best score from a 50 x 50 rate grid
* Best scaling achieved for
    * synonymous rate = 4.136
    * non-synonymous rate = 0.457
* Computing conditional site likelihoods on a 50 x 50 rate grid

Running MCMC chains to obtain a posterior sample of rate weights
* Using the following settings
    * Number of chains : 10
    * Steps/chain   : 2000000
    * Burn-in steps   : 1000000
    * Samples/chain   : 200
    * Dirichlet alpha : 0.5
* Running MCMC chain 1
* Running MCMC chain 2
* Running MCMC chain 3

```
* Running MCMC chain 4
* Running MCMC chain 5
* Running MCMC chain 6
* Running MCMC chain 7
* Running MCMC chain 8
* Running MCMC chain 9
* Running MCMC chain 10

### Tabulating site-level results
|     Codon      |   Partition    |     alpha      |      beta
|     N.eff      |Posterior prob for positive selection|
|:--------------:|:--------------:|:--------------:|:--------------:
|:--------------:|:-----------------------------------:|
|       13       |       1        |        0.963   |        7.320
|      170.237   |       Pos. posterior = 0.9663       |
|       44       |       1        |        0.966   |        6.268
|      183.732   |       Pos. posterior = 0.9585       |
|      386       |       1        |        0.973   |        7.261
|      171.023   |       Pos. posterior = 0.9584       |
|      426       |       1        |        1.694   |        6.749
|      121.746   |       Pos. posterior = 0.9258       |
|      440       |       1        |        0.750   |        7.706
|      203.816   |       Pos. posterior = 0.9850       |
----
## FUBAR inferred 5 sites subject to diversifying positive selection
at posterior probability >= 0.9
Of these,  0.21 are expected to be false positives (95% confidence
interval of 0-1 )
```
