# Supplementary material for: Evidence of CPV2c introgression into Croatia and novel insights into phylogeny and cell tropism
Source: Sci Rep. 2019 Nov 15;9:16909. doi: 10.1038/s41598-019-53422-9 (PMC6858334; doi:10.1038/s41598-019-53422-9)
Supplement: Supplementary file 6 — Brench-Site Unrestricted Statistical Test for Episodic Diversification [file 41598_2019_53422_MOESM6_ESM.pdf]

Evidence of CPV2c introgression into Croatia and novel insights into phylogeny and cell tropism

Dinko Novosel, Tamas Tuboly, Gyula Balka, Levente Szeredi, Ivana Lojkic, Andreja Jungic, Zaklin Acinger Rogic, Tahar Ait Ali, Attila Csagola

Supplementary info file 6.

Results of selection using Brench-Site Unrestricted Statistical Test for Episodic Diversification method

```
    /HYPHY 2.3.13.20180601beta(MPI) for Darwin on x86_64\
***************** TYPES OF STANDARD ANALYSES *****************


    (1) Selection Analyses
    (2) Evolutionary Hypothesis Testing
    (3) Relative evolutionary rate inference
    (4) Basic Analyses
    (5) Codon Selection Analyses
    (6) Compartmentalization
    (7) Data File Tools
    (8) Miscellaneous
    (9) Model Comparison
    (10) Kernel Analysis Tools
    (11) Molecular Clock
    (12) Phylogeny Reconstruction
    (13) Positive Selection
    (14) Recombination
    (15) Selection/Recombination
    (16) Relative Rate
    (17) Relative Ratio
    (18) Substitution Rates

1
 Please select type of analyses you want to list (or press ENTER to
process custom batch file):










***************** FILES IN 'Selection Analyses' *****************


    (1) [MEME] Test for episodic site-level selection using MEME
```

```
(Mixed Effects Model of Evolution).
    (2) [FEL] Test for pervasive site-level selection using FEL
(Fixed Effects Likelihood).
    (3) [SLAC] Test for pervasive site-level selection using SLAC
(Single Likelihood Ancestor Counting).
    (4) [FUBAR] Test for pervasive site-level selection using FUBAR
(Fast Unconstrained Bayesian AppRoximation for inferring selection).
    (5) [BUSTED] Test for episodic gene-wide selection using BUSTED
(Branch-site Unrestricted Statistical Test of Episodic
Diversification).
    (6) [aBSREL] Test for lineage-specific evolution using the
branch-site method aBS-REL (Adaptive Branch-Site Random Effects
Likelihood).
    (7) [RELAX] Test for relaxation of selection pressure along a
specified set of test branches using RELAX (a random effects test of
selection relaxation).

 Please select the analysis you would like to perform (or press
ENTER to return to the list of analysis types):5

Analysis Description
--------------------
BUSTED (branch-site unrestricted statistical test of episodic
diversification) uses a random effects branch-site model fitted
jointly
to all or a subset of tree branches in order to test for alignment-
wide
evidence of episodic diversifying selection. Assuming there is
evidence
of positive selection (i.e. there is an omega > 1), BUSTED will also
perform a quick evidence-ratio style analysis to explore which
individual sites may have been subject to selection.

- __Requirements__: in-frame codon alignment and a phylogenetic tree
(optionally annotated
with {})

- __Citation__: *Gene-wide identification of episodic selection*,
Mol Biol Evol.
32(5):1365-71

- __Written by__: Sergei L Kosakovsky Pond

- __Contact Information__: spond@temple.edu

- __Analysis Version__: 1.2



####Choose Genetic Code

1. [**Universal**] Universal code. (Genebank transl_table=1).
2. [**Vertebrate mtDNA**] Vertebrate mitochondrial DNA code.
(Genebank transl_table=2).
```

3. [**Yeast mtDNA**] Yeast mitochondrial DNA code. (Genebank transl_table=3).
4. [**Mold/Protozoan mtDNA**] Mold, Protozoan and Coelenterate mitochondrial DNA and the Mycloplasma/Spiroplasma code. (Genebank transl_table=4).
5. [**Invertebrate mtDNA**] Invertebrate mitochondrial DNA code. (Genebank transl_table=5).
6. [**Ciliate Nuclear**] Ciliate, Dasycladacean and Hexamita Nuclear code. (Genebank transl_table=6).
7. [**Echinoderm mtDNA**] Echinoderm mitochondrial DNA code. (Genebank transl_table=9).
8. [**Euplotid Nuclear**] Euplotid Nuclear code. (Genebank transl_table=10).
9. [**Alt. Yeast Nuclear**] Alternative Yeast Nuclear code. (Genebank transl_table=12).
10. [**Ascidian mtDNA**] Ascidian mitochondrial DNA code. (Genebank transl_table=13).
11. [**Flatworm mtDNA**] Flatworm mitochondrial DNA code. (Genebank transl_table=14).
12. [**Blepharisma Nuclear**] Blepharisma Nuclear code. (Genebank transl_table=15).
13. [**Chlorophycean mtDNA**] Chlorophycean Mitochondrial Code (transl_table=16).
14. [**Trematode mtDNA**] Trematode Mitochondrial Code (transl_table=21).
15. [**Scenedesmus obliquus mtDNA**] Scenedesmus obliquus mitochondrial Code (transl_table=22).
16. [**Thraustochytrium mtDNA**] Thraustochytrium Mitochondrial Code (transl_table=23).
17. [**Pterobranchia mtDNA**] Pterobranchia Mitochondrial Code (transl_table=24).
18. [**SR1 and Gracilibacteria**] Candidate Division SR1 and Gracilibacteria Code (transl_table=25).
19. [**Pachysolen Nuclear**] Pachysolen tannophilus Nuclear Code (transl_table=26).

>Please choose an option (or press q to cancel selection):1

>Select a coding sequence alignment file (`/Users/dinkonovosel/hyphy/res/TemplateBatchFiles/SelectionAnalyses/`) /Users/dinkonovosel/CPV_VP2_cod_sel.txt

>A tree was found in the data file:
`((ITA/FJ005218/2c/330/2006,ITA/FJ005233/40/2007),POR/KT275253/2c/PT036/12/2012),(URU/KC196096/2c/M247/2010,URU/KM457121/2c/UY247/2010)),(URU/KC196086/2c/M55/2006,URU/KM457106/2c/UY55/2006)),USA/JX475260/CO/704/2010),ITA/FJ005247/195/2008),ITA/FJ005226/383/2006),GER/FJ005196/2c/G7/1997),FRA/DQ025994/04S25/2004),FRA/DQ025960/03C4/2003),FRA/DQ025951/03B10/2003),FRA/DQ025954/03B14/2003),USA/KJ813848/Bobcat/ND/1162/2013),URU/KM457104/2c/UY47/2006),FRA/

DQ025969/03S5/2003),USA/KJ813858/Puma/ND/F93/2013),ITA/
FJ005248/219/2008),GER/FJ005199/2c/G172/1997),ITA/
FJ005240/208/2007),URU/KC196085/2c/M57/2007),FRA/
DQ025975/04S6/2004),USA/JX475243/ID/22772/2009),USA/JX475252/CO/
1316/2010),FRA/DQ025965/03C9/2003),ECU/KF149984/2c/ME28/2012),(ECU/
KF149962/2c/ME1/2012,(ECU/KF149963/2c/ME10/2012,(ECU/KF149964/2c/
ME23/2012,ECU/KF149969/2c/ME31/2012)))),(ARG/JF414820/Arg44/2009,
(ARG/KM236569/Cuba/2013,(ARG/JF414818/Arg32/2008,ARG/JF414821/
Arg48/2009)))),(ITA/FJ005216/2c/284/2006,(ITA/KU508407/2c/
25835/09/2009,ITA/KX434459/27692/1/11/2011))),(((URU/KM457122/2c/
UY258/2010,URU/KM457124/2c/UY307/2011),URU/KC196093/2c/M307/2011),
((URU/KC196081/2c/M95/2007,URU/KM457109/2c/UY95/2007),(URU/
KC196097/2c/M242/2010,(URU/KM457120/2c/UY242/2010,URU/KM457123/2c/
UY261/2008))))),(URU/KM457103/2c/UY12/2006,(ITA/FJ005209/2c/
303/2004,ITA/FJ005251/239/2008))),(URU/KC196083/2c/M82/2007,URU/
KM457108/2c/UY82/2007)),URU/KC196107/2c/M129/2008),URU/KM457131/2c/
UY368/2011),POR/KT275252/2c/PT013/12/2012),ITA/FJ005231/406/2006),
(ITA/FJ005214/2c/67/2006,BRA/KY073269/UFMT/2015)),ARG/JF414819/
Arg35/2008),USA/KJ813854/Puma/ND/F205/2013),ITA/FJ005232/411/2006),
(((URU/KC196105/2c/M152/2008,URU/KM457113/2c/UY152/2009),ITA/
FJ005212/2c/349/2004),(POR/KT275255/2c/PT238/14/2014,(ITA/
KX434460/52238/12/2012,((URU/KC196091/2c/M326/2011,URU/KM457127/2c/
UY326/2011),(((((((((URU/KC196102/2c/M185/2009,URU/KM457116/2c/
UY185/2009),(HRV/KP859577/2c/HR856/2014,ITA/KX434458/2323/11/2011)),
(URU/KC196101/2c/M187/2009,URU/KM457117/2c/UY187/2009)),AUS/
KU508693/2c/LW/2015),(URU/KC196089/2c/M349/2011,URU/KM457129/2c/
UY349/2011)),ITA/FJ005195/2c/136/2000),FRA/DQ025976/04S7/2004),(USA/
JX475273/MT/909/2012,USA/KJ813888/Coyote/MT/878/2012)),USA/KJ813843/
Bobcat/ND/1160/2013),(FRA/DQ025942/01B1/2001,(FRA/
DQ025964/03C8/2003,(ITA/FJ005206/2c/287/2004,(URU/KM457107/2c/
UY72/2007,(URU/KM457111/2c/UY120/2008,(URU/KM457112/2c/UY135/2008,
(URU/KM457125/2c/UY317/2011,(URU/KM457130/2c/UY354/2011,(URU/
KM457142/2c/UY370/2011,(HRV/KP859574/2c/HR442/2014,(HRV/KP859575/2c/
HR774/2014,(HRV/KP859576/2c/HR793/2014,(AUS/KU508691/2c/HB/2015,
(AUS/KU508692/2c/FH/2015,ITA/
KX434456/45361/09/2009)))))))))))))))))),URU/KM457126/2c/
UY318/2010),(FRA/DQ025985/04S16/2004,(ITA/FJ005205/2c/279/2004,HRV/
KP859578/2c/HR859/2014))),ITA/FJ222821/2c/56/2000),(GER/FJ005260/
G82/1997,USA/KJ813846/Bobcat/ND/974/2013)),((GER/
AY742934/447/1995,RUS/JN033694/Laika/1993),(USA/AY742936/395/1998,
(USA/JX475240/AZ/16382-01/1999,(USA/JX475250/CO/728/2010,USA/
KJ813842/Bobcat/ND/502/2013))))),((((((((((((((((USA/KJ813828/
Fisher/F1F010712/2013,USA/KJ813881/Graywolf/MI/832/2012),USA/
KJ813844/Bobcat/ND/885/2013),USA/KJ813882/Raccoon/NJ/1423/2012),USA/
KJ813851/Bobcat/ND/1168/2013),USA/JX475278/AR/1069/2012),KOR/
EU009205/2b/K029/2006),USA/JX475247/CO/1246/2010),(USA/KJ813892/
Coyote/AK/218/2013,(USA/JN867604/Dog/IL/137654/2008,USA/JX475242/WI/
18268/2002))),SAF/HQ602969/22/10SA/2010),(USA/AY742955/436/2003,FRA/
DQ025991/2b/04S22/2004)),USA/JN867602/2b/Dog/CA/148743/2008),USA/
JX475251/CO/2235/2009),USA/JN867603/2b/Dog/KS/81213/2009),USA/
KJ813852/Bobcat/ND/1170/2013),FRA/DQ025961/2b/03C5/2003),(USA/
KJ813827/Fisher/F1M111211/2013,USA/KJ813873/Graywolf/MI/850/2012)),
(ECU/KF149971/2c/ME32/2012,IND/KX469432/newCPV/2b/Hiller/2011)),
((ITA/FJ005263/42/2005,ITA/FJ005265/140/2005),(((((((((USA/

M74849/39/1995,USA/U22896/cat/1990),FRA/DQ025992/2b/04S23/2004),USA/
M74852/133/1995),POR/KU662349/greywolf/W33/1996),(POR/KU662350/
greywolf/W52/2005,POL/Z46651/46/1994)),GER/FJ005261/G162/1997),BRA/
DQ340409/2b/BR183/1985),USA/AY742932/193/1991),((USA/
AY742951/431/2003,USA/JN867605/2b/Dog/US/142805/2009),(VAC/
FJ222822/2b/FortDodge/2008,(VAC/JN625223/INDIA/vac5/2011,(USA/
EU659119/2b/CPV/410/2000,USA/EU659120/2b/CPV/411a/1998))))),((CHI/
GQ857609/CPV08/01/2008,CHI/GU569940/2b/YN0203/2002),((((((JPN/
AB115504/2c/97/008/1997,TAW/U72696/2b/T10/1996),TAW/U72695/2a/
T4/1996),(CHI/GQ857596/CPV05/01/2005,CHI/GQ857600/
CPV06/01/2006)),THA/FJ869125/KU5/2004),(USA/JX475237/CT/
372/2011,KOR/EF599097/2b/DH326/2006)),(CHI/EU483515/2b/ZD13/2007,
(JPN/LC270891/2b/9985/2017,(JPN/AB437433/1887/M/2/2008,(TAW/
FJ265781/CPV307/2005,(TWN/EF592511/TWN1/2006,TAW/FJ265775/
CPV301/2004)))))),(((VIE/AB054218/2b/cat/V123/2000,VAC/FJ222823/2b/
29/1997),ITA/FJ005264/134/2005),((THA/FJ869122/KU1/2008,THA/
FJ869123/KU3/2008),((((((THA/KP715690/VT28/2014,THA/KP715716/
VT143/2014),THA/KP715691/VT43/2014),(VIE/AB120722/2b/HCM/
18/2003,VIE/AB120724/2b/HNI/2/13/2003)),(CHI/GQ857599/
CPV05/04/2005,CHI/GQ857601/CPV06/02/2006)),CHI/GQ857605/
CPV07/03/2007),(THA/FJ869139/KU66/2003,(VIE/AB120721/2b/HCM/8/2003,
(VIE/AB054221/2b/leopard/V204/2000,(VIE/AB054224/2c/leopard/
V203/2000,(VIE/AB120725/2b/HNI/3/4/2003,(VIE/AB120723/2b/HCM/
23/2003,(VIE/AB120720/2b/HCM/6/2003,(VIE/AB054219/2b/cat/V209/2000,
(VIE/AB054220/2b/cat/V217/2000,CHI/EU145954/2b/
BJ044/2007))))))))))))))))),(ITA/FJ005257/54/2008,ITA/KF373611/2a/
409/2010)),(NZE/AY742933/339/1993,((VIE/AB054223/2c/leopard/
V140/2000,ITA/GU362932/cat11/2008),(NIG/HQ602995/15/10/2010,
(((((((((FRA/DQ025947/2a/02B5/2002,FRA/DQ026001/2a/04S32/2004),FRA/
DQ025962/2a/03C6/2003),ITA/KF373580/2a/581/2003),(GER/AY742935/
U6/1995,FRA/DQ025945/2a/02B3/2002)),VIE/AB054215/2a/cat/
V120/2000),ITA/FJ005255/333/2005),FRA/DQ025958/2a/03C2/2003),(ITA/
KX434457/987/10/2010,(FRA/DQ025983/2a/04S14/2004,FRA/DQ025993/2a/
04S24/2004))),(FRA/DQ025984/2a/04S15/2004,ITA/FJ005252/96/2002)),
(FRA/DQ026002/2a/04S33/2004,(ITA/KF373592/2a/329/2008,(((ITA/
AF393506/2a/699/2000,FRA/DQ025943/2a/01S1/2001),ITA/KF385388/2a/
Sicily/X83090/2009),((CHI/GQ857612/CPV08/04/2008,CHI/GU569939/2a/
YN0202/2002),(((((HUN/KF539794/H/7/2012,HUN/KF539795/H/8/2012),HUN/
KF539804/H/212/2012),(HUN/KF539793/H/5/2012,HUN/KF539797/H/
11/2012)),(HUN/KF539800/H/27/2012,(VIE/AB054217/2a/cat/
V154/2000,HUN/KF539796/H/9/2012))),(((HUN/KF539798/H/31/2012,HUN/
KF539799/H/39/2012),HUN/KF539805/H/36/2012),(ITA/AF306447/618/2000,
(FRA/DQ025944/2a/02B2/2002,(NIG/HQ602992/19/10/2010,(ITA/
AF306446/584/2000,(FRA/DQ025986/2a/04S17/2004,(ITA/KF373577/2a/
714/2001,(FRA/DQ025982/2a/04S13/2004,ITA/
FJ005253/67/2005))))))))))))))))),(((((((THA/FJ869126/
KU5/2008,THA/FJ869137/KU52/2003),THA/FJ869134/KU23/2003),CHI/
DQ354068/2a/redpanda/RPPV/2004),KOR/EF599096/DH426/2005),(ITA/
FJ005258/80/2008,(KOR/EF599098/2c/Pome/2006,(FRA/DQ025950/2a/
02B9/2002,ITA/KX434454/29451/09/2009)))),(THA/FJ869130/KU13/2004,
(THA/FJ869138/KU53/2003,CHI/KF803615/2011/BJ/B25/2011))),(CHI/
GU569942/2a/JL0202/2002,CHI/GU569946/2a/JL0201/2002)),((USA/
AY742953/435/2003,ITA/KF373571/2a/685/1999),(THA/FJ869128/KU11/2004,
(((BRA/DQ340428/2a/BR209/1994,BRA/DQ340431/2a/BR56/1995),BRA/

DQ340411/2a/BR8/1990),(BRA/DQ340422/2a/BR22/1993,(BRA/DQ340421/2a/
BR597/1992,((((BRA/DQ340419/2a/BR570/1992,BRA/DQ340423/2a/
BR136/1993),BRA/DQ340413/2a/BR18/1990),BRA/DQ340427/2a/BR133/1994),
(BRA/DQ340414/2a/BR31/1990,(BRA/DQ340416/2a/BR47/1991,(BRA/
DQ340417/2a/BR52/1991,(BRA/DQ340418/2a/BR491/1992,(BRA/DQ340424/2a/
BR137/1993,BRA/DQ340426/2a/BR84/1994)))))))))))),CHI/KF803600/2010/
BJ/A68/2010),(((USA/EU659118/CPV/13/1981,CHI/GU569948/2a/
CC8601/1986),JPN/D26079/1993),((BRA/DQ340407/2a/BR145/1980,BRA/
DQ340408/2a/BR154/1980),(FRA/DQ025952/2a/03B12/2003,(BRA/
DQ340404/2a/BR6/1980,(BRA/DQ340405/2a/BR135/1980,(BRA/DQ340410/2a/
BR315/1986,(USA/M24000/FPV/CPV/31/1988,USA/M24003/FPV/CPV/
15/1988))))))),((USA/JN867599/Raccoon/KY/39552/2009,USA/JN867611/
Raccoon/KY/358-B/2009),(USA/JN867610/Raccoon/VA/118-A/2007,(USA/
KJ813890/Redfox/MA/197/2012,(USA/JX475284/TN/26/2011,(USA/JX475239/
GA/06/2011,USA/JX475279/TN/1/2011)))))),(HUN/KF539801/H/25/2012,HUN/
KF539803/H/2/2012)),(USA/KJ813870/Raccoon/TX/1/2013,(((USA/JN867598/
Bobcat/KS/44/2010,USA/KJ813832/Fisher/ND/14/2013),(USA/KJ813831/
Fisher/ND/17/2013,USA/KJ813835/Fisher/ND/19/2013)),(USA/JX475234/ME/
258/2011,(USA/JN867618//Raccoon/WI/37/2010,(USA/JX475231/CO/
280/2011,(USA/JX475248/CO/1102/2011,(USA/JX475233/SC/182-A/2011,USA/
JX475246/CO/2503/2010))))))),((CHI/FJ231389/FPV/monkey/
BJ-22/2008,CHI/KJ170680/raccoondog/HLJ11/1/2011),((((((((CHI/
GU392242/raccoondog/HB10/2009,CHI/GU392244/raccoondog/HB7/2009),CHI/
KJ170679/raccoondog/Heb10/2/2010),CHI/GU392241/raccoondog/
HB1/2009),CHI/GU392236/fox/HB1/2009),(CHI/GU392240/raccoondog/
HB3/2009,(CHI/GU392239/raccoondog/HB6/2009,CHI/KJ194463/raccoondog/
HeB10/3/2010))),CHI/GU392237/fox/HB2/2009),(VAC/FJ011098/Intervet/
2006,(VAC/JN625222/INDIA/vac4/2011,(ITA/FJ222824/388/05/3/2005,(CHI/
FJ432718/CPV/Cv/2008,(VAC/JN625219/INDIA/vac1/2011,CHI/
KF803602/2010/BJ/A72/2010)))))),JPN/AB437434/1887/f/3/2008),
((((((VAC/GU212790/primodog/2009,VAC/GU212791/vanguard/2009),VAC/
FJ197847/Pfizer/2007),VAC/EU914139/Pfizer//2006),VAC/KY083089/
Singapore/2016),USA/M19296/CPV/N/1988),(((((USA/M23255/FPV/
Cornell320/1988,USA/M38245/1990),USA/EU659116/CPV/5/1979),(FIN/
U22192/raccoondog/RD-80/1980,FIN/U22193/raccoondog/RD87/1987)),(USA/
M10989/1985,USA/U22186/CPV/128/1995)),(VAC/JN625221/INDIA/vac3/2011,
(VAC/JN625220/INDIA/vac2/2011,(((VAC/FJ011097/Merial/2006,CHI/
GQ169553/Vac2/2007),VAC/KY083090/Singapore/2016),(CHI/GU569943/
YB8301/1983,(VAC/JN625224/INDIA/vac6/2011,ARG/KM236572/NNGag/
2012)))))))))))`

>Would you like to use it (y/n)? y

>Loaded a multiple sequence alignment with **339** sequences, **581** codons, and **1** partitions from `/Users/dinkonovosel/CPV_VP2_cod_sel.txt`

####Choose the set of branches to test for selection

1. [**All**] Include all branches in the analysis
2. [**Internal**] Include all internal branches in the analysis
3. [**Leaves**] Include all leaf branches in the analysis

4. [**Unlabeled branches**] Set of 675 unlabeled branches

>Please choose an option (or press q to cancel selection):1

Branches to test for selection in the BUSTED analysis
* Selected 675 branches to test in the BUSTED analysis:
`ITA_FJ005218_2c_330_2006, ITA_FJ005233_40_2007, Node53,
POR_KT275253_2c_PT036_12_2012, Node52, URU_KC196096_2c_M247_2010,
URU_KM457121_2c_UY247_2010, Node57, Node51,
URU_KC196086_2c_M55_2006, URU_KM457106_2c_UY55_2006, Node60, Node50,
USA_JX475260_CO_704_2010, Node49, ITA_FJ005247_195_2008, Node48,
ITA_FJ005226_383_2006, Node47, GER_FJ005196_2c_G7_1997, Node46,
FRA_DQ025994_04S25_2004, Node45, FRA_DQ025960_03C4_2003, Node44,
FRA_DQ025951_03B10_2003, Node43, FRA_DQ025954_03B14_2003, Node42,
USA_KJ813848_Bobcat_ND_1162_2013, Node41, URU_KM457104_2c_UY47_2006,
Node40, FRA_DQ025969_03S5_2003, Node39,
USA_KJ813858_Puma_ND_F93_2013, Node38, ITA_FJ005248_219_2008,
Node37, GER_FJ005199_2c_G172_1997, Node36, ITA_FJ005240_208_2007,
Node35, URU_KC196085_2c_M57_2007, Node34, FRA_DQ025975_04S6_2004,
Node33, USA_JX475243_ID_22772_2009, Node32,
USA_JX475252_CO_1316_2010, Node31, FRA_DQ025965_03C9_2003, Node30,
ECU_KF149984_2c_ME28_2012, Node29, ECU_KF149962_2c_ME1_2012,
ECU_KF149963_2c_ME10_2012, ECU_KF149964_2c_ME23_2012,
ECU_KF149969_2c_ME31_2012, Node88, Node86, Node84, Node28,
ARG_JF414820_Arg44_2009, ARG_KM236569_Cuba_2013,
ARG_JF414818_Arg32_2008, ARG_JF414821_Arg48_2009, Node95, Node93,
Node91, Node27, ITA_FJ005216_2c_284_2006,
ITA_KU508407_2c_25835_09_2009, ITA_KX434459_27692_1_11_2011,
Node100, Node98, Node26, URU_KM457122_2c_UY258_2010,
URU_KM457124_2c_UY307_2011, Node105, URU_KC196093_2c_M307_2011,
Node104, URU_KC196081_2c_M95_2007, URU_KM457109_2c_UY95_2007,
Node110, URU_KC196097_2c_M242_2010, URU_KM457120_2c_UY242_2010,
URU_KM457123_2c_UY261_2008, Node115, Node113, Node109, Node103,
Node25, URU_KM457103_2c_UY12_2006, ITA_FJ005209_2c_303_2004,
ITA_FJ005251_239_2008, Node120, Node118, Node24,
URU_KC196083_2c_M82_2007, URU_KM457108_2c_UY82_2007, Node123,
Node23, URU_KC196107_2c_M129_2008, Node22,
URU_KM457131_2c_UY368_2011, Node21, POR_KT275252_2c_PT013_12_2012,
Node20, ITA_FJ005231_406_2006, Node19, ITA_FJ005214_2c_67_2006,
BRA_KY073269_UFMT_2015, Node130, Node18, ARG_JF414819_Arg35_2008,
Node17, USA_KJ813854_Puma_ND_F205_2013, Node16,
ITA_FJ005232_411_2006, Node15, URU_KC196105_2c_M152_2008,
URU_KM457113_2c_UY152_2009, Node138, ITA_FJ005212_2c_349_2004,
Node137, POR_KT275255_2c_PT238_14_2014, ITA_KX434460_52238_12_2012,
URU_KC196091_2c_M326_2011, URU_KM457127_2c_UY326_2011, Node147,
URU_KC196102_2c_M185_2009, URU_KM457116_2c_UY185_2009, Node159,
HRV_KP859577_2c_HR856_2014, ITA_KX434458_2323_11_2011, Node162,
Node158, URU_KC196101_2c_M187_2009, URU_KM457117_2c_UY187_2009,
Node165, Node157, AUS_KU508693_2c_LW_2015, Node156,
URU_KC196089_2c_M349_2011, URU_KM457129_2c_UY349_2011, Node169,
Node155, ITA_FJ005195_2c_136_2000, Node154, FRA_DQ025976_04S7_2004,
Node153, USA_JX475273_MT_909_2012, USA_KJ813888_Coyote_MT_878_2012,
Node174, Node152, USA_KJ813843_Bobcat_ND_1160_2013, Node151,

FRA_DQ025942_01B1_2001, FRA_DQ025964_03C8_2003,
ITA_FJ005206_2c_287_2004, URU_KM457107_2c_UY72_2007,
URU_KM457111_2c_UY120_2008, URU_KM457112_2c_UY135_2008,
URU_KM457125_2c_UY317_2011, URU_KM457130_2c_UY354_2011,
URU_KM457142_2c_UY370_2011, HRV_KP859574_2c_HR442_2014,
HRV_KP859575_2c_HR774_2014, HRV_KP859576_2c_HR793_2014,
AUS_KU508691_2c_HB_2015, AUS_KU508692_2c_FH_2015,
ITA_KX434456_45361_09_2009, Node204, Node202, Node200, Node198,
Node196, Node194, Node192, Node190, Node188, Node186, Node184,
Node182, Node180, Node178, Node150, Node146, Node144, Node142,
Node136, Node14, URU_KM457126_2c_UY318_2010, Node13,
FRA_DQ025985_04S16_2004, ITA_FJ005205_2c_279_2004,
HRV_KP859578_2c_HR859_2014, Node210, Node208, Node12,
ITA_FJ222821_2c_56_2000, Node11, GER_FJ005260_G82_1997,
USA_KJ813846_Bobcat_ND_974_2013, Node214, Node10,
GER_AY742934_447_1995, RUS_JN033694_Laika_1993, Node218,
USA_AY742936_395_1998, USA_JX475240_AZ_16382_01_1999,
USA_JX475250_CO_728_2010, USA_KJ813842_Bobcat_ND_502_2013, Node225,
Node223, Node221, Node217, Node9,
USA_KJ813828_Fisher_F1F010712_2013,
USA_KJ813881_Graywolf_MI_832_2012, Node245,
USA_KJ813844_Bobcat_ND_885_2013, Node244,
USA_KJ813882_Raccoon_NJ_1423_2012, Node243,
USA_KJ813851_Bobcat_ND_1168_2013, Node242,
USA_JX475278_AR_1069_2012, Node241, KOR_EU009205_2b_K029_2006,
Node240, USA_JX475247_CO_1246_2010, Node239,
USA_KJ813892_Coyote_AK_218_2013, USA_JN867604_Dog_IL_137654_2008,
USA_JX475242_WI_18268_2002, Node256, Node254, Node238,
SAF_HQ602969_22_10SA_2010, Node237, USA_AY742955_436_2003,
FRA_DQ025991_2b_04S22_2004, Node260, Node236,
USA_JN867602_2b_Dog_CA_148743_2008, Node235,
USA_JX475251_CO_2235_2009, Node234,
USA_JN867603_2b_Dog_KS_81213_2009, Node233,
USA_KJ813852_Bobcat_ND_1170_2013, Node232,
FRA_DQ025961_2b_03C5_2003, Node231,
USA_KJ813827_Fisher_F1M111211_2013,
USA_KJ813873_Graywolf_MI_850_2012, Node268, Node230,
ECU_KF149971_2c_ME32_2012, IND_KX469432_newCPV_2b_Hiller_2011,
Node271, Node229, ITA_FJ005263_42_2005, ITA_FJ005265_140_2005,
Node275, USA_M74849_39_1995, USA_U22896_cat_1990, Node287,
FRA_DQ025992_2b_04S23_2004, Node286, USA_M74852_133_1995, Node285,
POR_KU662349_greywolf_W33_1996, Node284,
POR_KU662350_greywolf_W52_2005, POL_Z46651_46_1994, Node293,
Node283, GER_FJ005261_G162_1997, Node282,
BRA_DQ340409_2b_BR183_1985, Node281, USA_AY742932_193_1991, Node280,
USA_AY742951_431_2003, USA_JN867605_2b_Dog_US_142805_2009, Node300,
VAC_FJ222822_2b_FortDodge_2008, VAC_JN625223_INDIA_vac5_2011,
USA_EU659119_2b_CPV_410_2000, USA_EU659120_2b_CPV_411a_1998,
Node307, Node305, Node303, Node299, Node279,
CHI_GQ857609_CPV08_01_2008, CHI_GU569940_2b_YN0203_2002, Node311,
JPN_AB115504_2c_97_008_1997, TAW_U72696_2b_T10_1996, Node320,
TAW_U72695_2a_T4_1996, Node319, CHI_GQ857596_CPV05_01_2005,
CHI_GQ857600_CPV06_01_2006, Node324, Node318, THA_FJ869125_KU5_2004,
Node317, USA_JX475237_CT_372_2011, KOR_EF599097_2b_DH326_2006,

Node328, Node316, CHI_EU483515_2b_ZD13_2007,
JPN_LC270891_2b_9985_2017, JPN_AB437433_1887_M_2_2008,
TAW_FJ265781_CPV307_2005, TWN_EF592511_TWN1_2006,
TAW_FJ265775_CPV301_2004, Node339, Node337, Node335, Node333,
Node331, Node315, VIE_AB054218_2b_cat_V123_2000,
VAC_FJ222823_2b_29_1997, Node344, ITA_FJ005264_134_2005, Node343,
THA_FJ869122_KU1_2008, THA_FJ869123_KU3_2008, Node349,
THA_KP715690_VT28_2014, THA_KP715716_VT143_2014, Node357,
THA_KP715691_VT43_2014, Node356, VIE_AB120722_2b_HCM_18_2003,
VIE_AB120724_2b_HNI_2_13_2003, Node361, Node355,
CHI_GQ857599_CPV05_04_2005, CHI_GQ857601_CPV06_02_2006, Node364,
Node354, CHI_GQ857605_CPV07_03_2007, Node353,
THA_FJ869139_KU66_2003, VIE_AB120721_2b_HCM_8_2003,
VIE_AB054221_2b_leopard_V204_2000,
VIE_AB054224_2c_leopard_V203_2000, VIE_AB120725_2b_HNI_3_4_2003,
VIE_AB120723_2b_HCM_23_2003, VIE_AB120720_2b_HCM_6_2003,
VIE_AB054219_2b_cat_V209_2000, VIE_AB054220_2b_cat_V217_2000,
CHI_EU145954_2b_BJ044_2007, Node384, Node382, Node380, Node378,
Node376, Node374, Node372, Node370, Node368, Node352, Node348,
Node342, Node314, Node310, Node278, Node274, Node228, Node8,
ITA_FJ005257_54_2008, ITA_KF373611_2a_409_2010, Node387, Node7,
NZE_AY742933_339_1993, VIE_AB054223_2c_leopard_V140_2000,
ITA_GU362932_cat11_2008, Node393, NIG_HQ602995_15_10_2010,
FRA_DQ025947_2a_02B5_2002, FRA_DQ026001_2a_04S32_2004, Node407,
FRA_DQ025962_2a_03C6_2003, Node406, ITA_KF373580_2a_581_2003,
Node405, GER_AY742935_U6_1995, FRA_DQ025945_2a_02B3_2002, Node412,
Node404, VIE_AB054215_2a_cat_V120_2000, Node403,
ITA_FJ005255_333_2005, Node402, FRA_DQ025958_2a_03C2_2003, Node401,
ITA_KX434457_987_10_2010, FRA_DQ025983_2a_04S14_2004,
FRA_DQ025993_2a_04S24_2004, Node420, Node418, Node400,
FRA_DQ025984_2a_04S15_2004, ITA_FJ005252_96_2002, Node423, Node399,
FRA_DQ026002_2a_04S33_2004, ITA_KF373592_2a_329_2008,
ITA_AF393506_2a_699_2000, FRA_DQ025943_2a_01S1_2001, Node432,
ITA_KF385388_2a_Sicily_X83090_2009, Node431,
CHI_GQ857612_CPV08_04_2008, CHI_GU569939_2a_YN0202_2002, Node437,
HUN_KF539794_H_7_2012, HUN_KF539795_H_8_2012, Node444,
HUN_KF539804_H_212_2012, Node443, HUN_KF539793_H_5_2012,
HUN_KF539797_H_11_2012, Node448, Node442, HUN_KF539800_H_27_2012,
VIE_AB054217_2a_cat_V154_2000, HUN_KF539796_H_9_2012, Node453,
Node451, Node441, HUN_KF539798_H_31_2012, HUN_KF539799_H_39_2012,
Node458, HUN_KF539805_H_36_2012, Node457, ITA_AF306447_618_2000,
FRA_DQ025944_2a_02B2_2002, NIG_HQ602992_19_10_2010,
ITA_AF306446_584_2000, FRA_DQ025986_2a_04S17_2004,
ITA_KF373577_2a_714_2001, FRA_DQ025982_2a_04S13_2004,
ITA_FJ005253_67_2005, Node474, Node472, Node470, Node468, Node466,
Node464, Node462, Node456, Node440, Node436, Node430, Node428,
Node426, Node398, Node396, Node392, Node390, Node6,
THA_FJ869126_KU5_2008, THA_FJ869137_KU52_2003, Node484,
THA_FJ869134_KU23_2003, Node483, CHI_DQ354068_2a_redpanda_RPPV_2004,
Node482, KOR_EF599096_DH426_2005, Node481, ITA_FJ005258_80_2008,
KOR_EF599098_2c_Pome_2006, FRA_DQ025950_2a_02B9_2002,
ITA_KX434454_29451_09_2009, Node494, Node492, Node490, Node480,
THA_FJ869130_KU13_2004, THA_FJ869138_KU53_2003,
CHI_KF803615_2011_BJ_B25_2011, Node499, Node497, Node479,

CHI_GU569942_2a_JL0202_2002, CHI_GU569946_2a_JL0201_2002, Node502, Node478, USA_AY742953_435_2003, ITA_KF373571_2a_685_1999, Node506, THA_FJ869128_KU11_2004, BRA_DQ340428_2a_BR209_1994, BRA_DQ340431_2a_BR56_1995, Node513, BRA_DQ340411_2a_BR8_1990, Node512, BRA_DQ340422_2a_BR22_1993, BRA_DQ340421_2a_BR597_1992, BRA_DQ340419_2a_BR570_1992, BRA_DQ340423_2a_BR136_1993, Node524, BRA_DQ340413_2a_BR18_1990, Node523, BRA_DQ340427_2a_BR133_1994, Node522, BRA_DQ340414_2a_BR31_1990, BRA_DQ340416_2a_BR47_1991, BRA_DQ340417_2a_BR52_1991, BRA_DQ340418_2a_BR491_1992, BRA_DQ340424_2a_BR137_1993, BRA_DQ340426_2a_BR84_1994, Node537, Node535, Node533, Node531, Node529, Node521, Node519, Node517, Node511, Node509, Node505, Node477, Node5, CHI_KF803600_2010_BJ_A68_2010, Node4, USA_EU659118_CPV_13_1981, CHI_GU569948_2a_CC8601_1986, Node543, JPN_D26079_1993, Node542, BRA_DQ340407_2a_BR145_1980, BRA_DQ340408_2a_BR154_1980, Node548, FRA_DQ025952_2a_03B12_2003, BRA_DQ340404_2a_BR6_1980, BRA_DQ340405_2a_BR135_1980, BRA_DQ340410_2a_BR315_1986, USA_M24000_FPV_CPV_31_1988, USA_M24003_FPV_CPV_15_1988, Node559, Node557, Node555, Node553, Node551, Node547, Node541, Node3, USA_JN867599_Raccoon_KY_39552_2009, USA_JN867611_Raccoon_KY_358_B_2009, Node563, USA_JN867610_Raccoon_VA_118_A_2007, USA_KJ813890_Redfox_MA_197_2012, USA_JX475284_TN_26_2011, USA_JX475239_GA_06_2011, USA_JX475279_TN_1_2011, Node572, Node570, Node568, Node566, Node562, Node2, HUN_KF539801_H_25_2012, HUN_KF539803_H_2_2012, Node575, Node1, USA_KJ813870_Raccoon_TX_1_2013, USA_JN867598_Bobcat_KS_44_2010, USA_KJ813832_Fisher_ND_14_2013, Node582, USA_KJ813831_Fisher_ND_17_2013, USA_KJ813835_Fisher_ND_19_2013, Node585, Node581, USA_JX475234_ME_258_2011, USA_JN867618_Raccoon_WI_37_2010, USA_JX475231_CO_280_2011, USA_JX475248_CO_1102_2011, USA_JX475233_SC_182_A_2011, USA_JX475246_CO_2503_2010, Node596, Node594, Node592, Node590, Node588, Node580, Node578, CHI_FJ231389_FPV_monkey_BJ_22_2008, CHI_KJ170680_raccoondog_HLJ11_1_2011, Node600, CHI_GU392242_raccoondog_HB10_2009, CHI_GU392244_raccoondog_HB7_2009, Node611, CHI_KJ170679_raccoondog_Heb10_2_2010, Node610, CHI_GU392241_raccoondog_HB1_2009, Node609, CHI_GU392236_fox_HB1_2009, Node608, CHI_GU392240_raccoondog_HB3_2009, CHI_GU392239_raccoondog_HB6_2009, CHI_KJ194463_raccoondog_HeB10_3_2010, Node619, Node617, Node607, CHI_GU392237_fox_HB2_2009, Node606, VAC_FJ011098_Intervet_2006, VAC_JN625222_INDIA_vac4_2011, ITA_FJ222824_388_05_3_2005, CHI_FJ432718_CPV_Cv_2008, VAC_JN625219_INDIA_vac1_2011, CHI_KF803602_2010_BJ_A72_2010, Node631, Node629, Node627, Node625, Node623, Node605, JPN_AB437434_1887_f_3_2008, Node604, VAC_GU212790_primodog_2009, VAC_GU212791_vanguard_2009, Node640, VAC_FJ197847_Pfizer_2007, Node639, VAC_EU914139_Pfizer_2006, Node638, VAC_KY083089_Singapore_2016, Node637, USA_M19296_CPV_N_1988, Node636, USA_M23255_FPV_Cornell320_1988, USA_M38245_1990, Node651, USA_EU659116_CPV_5_1979, Node650, FIN_U22192_raccoondog_RD_80_1980, FIN_U22193_raccoondog_RD87_1987, Node655, Node649, USA_M10989_1985, USA_U22186_CPV_128_1995, Node658, Node648, VAC_JN625221_INDIA_vac3_2011, VAC_JN625220_INDIA_vac2_2011,

```
VAC_FJ011097_Merial_2006, CHI_GQ169553_Vac2_2007, Node667,
VAC_KY083090_Singapore_2016, Node666, CHI_GU569943_YB8301_1983,
VAC_JN625224_INDIA_vac6_2011, ARG_KM236572_NNGag_2012, Node673,
Node671, Node665, Node663, Node661, Node647, Node635, Node603,
Node599`


### Obtaining branch lengths and nucleotide substitution biases
under the nucleotide GTR model
* Log(L) = -6510.63, AIC-c = 14388.84 (683 estimated parameters)

### Obtaining the global omega estimate based on relative GTR branch
lengths and nucleotide substitution biases
* Log(L) = -6211.05, AIC-c = 13806.96 (690 estimated parameters)
* non-synonymous/synonymous rate ratio for *test* =   0.1168

### Improving branch lengths, nucleotide substitution biases, and
global dN/dS ratios under a full codon model
* Log(L) = -6211.05, AIC-c = 13806.96 (690 estimated parameters)
* non-synonymous/synonymous rate ratio for *test* =   0.1166

### Performing the full (dN/dS > 1 allowed) branch-site model fit
#NEXUS

BEGIN TAXA;
    DIMENSIONS NTAX = 339;
    TAXLABELS
        'VIE_AB054215_2a_cat_V120_2000'
'VIE_AB054217_2a_cat_V154_2000' 'VIE_AB054218_2b_cat_V123_2000'
'VIE_AB054219_2b_cat_V209_2000' 'VIE_AB054220_2b_cat_V217_2000'
'VIE_AB054221_2b_leopard_V204_2000'
'VIE_AB054223_2c_leopard_V140_2000'
'VIE_AB054224_2c_leopard_V203_2000' 'JPN_AB115504_2c_97_008_1997'
'VIE_AB120720_2b_HCM_6_2003' 'VIE_AB120721_2b_HCM_8_2003'
'VIE_AB120722_2b_HCM_18_2003' 'VIE_AB120723_2b_HCM_23_2003'
'VIE_AB120724_2b_HNI_2_13_2003' 'VIE_AB120725_2b_HNI_3_4_2003'
'JPN_AB437433_1887_M_2_2008' 'JPN_AB437434_1887_f_3_2008'
'ITA_AF306446_584_2000' 'ITA_AF306447_618_2000'
'ITA_AF393506_2a_699_2000' 'USA_AY742932_193_1991'
'NZE_AY742933_339_1993' 'GER_AY742934_447_1995'
'GER_AY742935_U6_1995' 'USA_AY742936_395_1998'
'USA_AY742951_431_2003' 'USA_AY742953_435_2003'
'USA_AY742955_436_2003' 'JPN_D26079_1993' 'FRA_DQ025942_01B1_2001'
'FRA_DQ025943_2a_01S1_2001' 'FRA_DQ025944_2a_02B2_2002'
'FRA_DQ025945_2a_02B3_2002' 'FRA_DQ025947_2a_02B5_2002'
'FRA_DQ025950_2a_02B9_2002' 'FRA_DQ025951_03B10_2003'
'FRA_DQ025952_2a_03B12_2003' 'FRA_DQ025954_03B14_2003'
'FRA_DQ025958_2a_03C2_2003' 'FRA_DQ025960_03C4_2003'
'FRA_DQ025961_2b_03C5_2003' 'FRA_DQ025962_2a_03C6_2003'
'FRA_DQ025964_03C8_2003' 'FRA_DQ025965_03C9_2003'
'FRA_DQ025969_03S5_2003' 'FRA_DQ025975_04S6_2004'
'FRA_DQ025976_04S7_2004' 'FRA_DQ025982_2a_04S13_2004'
'FRA_DQ025983_2a_04S14_2004' 'FRA_DQ025984_2a_04S15_2004'
'FRA_DQ025985_04S16_2004' 'FRA_DQ025986_2a_04S17_2004'
```

'FRA_DQ025991_2b_04S22_2004' 'FRA_DQ025992_2b_04S23_2004'
'FRA_DQ025993_2a_04S24_2004' 'FRA_DQ025994_04S25_2004'
'FRA_DQ026001_2a_04S32_2004' 'FRA_DQ026002_2a_04S33_2004'
'BRA_DQ340404_2a_BR6_1980' 'BRA_DQ340405_2a_BR135_1980'
'BRA_DQ340407_2a_BR145_1980' 'BRA_DQ340408_2a_BR154_1980'
'BRA_DQ340409_2b_BR183_1985' 'BRA_DQ340410_2a_BR315_1986'
'BRA_DQ340411_2a_BR8_1990' 'BRA_DQ340413_2a_BR18_1990'
'BRA_DQ340414_2a_BR31_1990' 'BRA_DQ340416_2a_BR47_1991'
'BRA_DQ340417_2a_BR52_1991' 'BRA_DQ340418_2a_BR491_1992'
'BRA_DQ340419_2a_BR570_1992' 'BRA_DQ340421_2a_BR597_1992'
'BRA_DQ340422_2a_BR22_1993' 'BRA_DQ340423_2a_BR136_1993'
'BRA_DQ340424_2a_BR137_1993' 'BRA_DQ340426_2a_BR84_1994'
'BRA_DQ340427_2a_BR133_1994' 'BRA_DQ340428_2a_BR209_1994'
'BRA_DQ340431_2a_BR56_1995' 'CHI_DQ354068_2a_redpanda_RPPV_2004'
'TWN_EF592511_TWN1_2006' 'KOR_EF599096_DH426_2005'
'KOR_EU009205_2b_K029_2006' 'CHI_EU145954_2b_BJ044_2007'
'CHI_EU483515_2b_ZD13_2007' 'USA_EU659116_CPV_5_1979'
'USA_EU659118_CPV_13_1981' 'USA_EU659119_2b_CPV_410_2000'
'USA_EU659120_2b_CPV_411a_1998' 'VAC_EU914139_Pfizer_2006'
'ITA_FJ005195_2c_136_2000' 'GER_FJ005196_2c_G7_1997'
'GER_FJ005199_2c_G172_1997' 'ITA_FJ005205_2c_279_2004'
'ITA_FJ005206_2c_287_2004' 'ITA_FJ005209_2c_303_2004'
'ITA_FJ005212_2c_349_2004' 'ITA_FJ005214_2c_67_2006'
'ITA_FJ005216_2c_284_2006' 'ITA_FJ005218_2c_330_2006'
'ITA_FJ005226_383_2006' 'ITA_FJ005231_406_2006'
'ITA_FJ005232_411_2006' 'ITA_FJ005233_40_2007'
'ITA_FJ005240_208_2007' 'ITA_FJ005247_195_2008'
'ITA_FJ005248_219_2008' 'ITA_FJ005251_239_2008'
'ITA_FJ005252_96_2002' 'ITA_FJ005253_67_2005'
'ITA_FJ005255_333_2005' 'ITA_FJ005257_54_2008'
'ITA_FJ005258_80_2008' 'GER_FJ005260_G82_1997'
'GER_FJ005261_G162_1997' 'ITA_FJ005263_42_2005'
'ITA_FJ005264_134_2005' 'ITA_FJ005265_140_2005'
'VAC_FJ011097_Merial_2006' 'VAC_FJ011098_Intervet_2006'
'VAC_FJ197847_Pfizer_2007' 'ITA_FJ222821_2c_56_2000'
'VAC_FJ222822_2b_FortDodge_2008' 'VAC_FJ222823_2b_29_1997'
'ITA_FJ222824_388_05_3_2005' 'CHI_FJ231389_FPV_monkey_BJ_22_2008'
'TAW_FJ265775_CPV301_2004' 'TAW_FJ265781_CPV307_2005'
'CHI_FJ432718_CPV_Cv_2008' 'THA_FJ869122_KU1_2008'
'THA_FJ869123_KU3_2008' 'THA_FJ869125_KU5_2004'
'THA_FJ869126_KU5_2008' 'THA_FJ869128_KU11_2004'
'THA_FJ869130_KU13_2004' 'THA_FJ869134_KU23_2003'
'THA_FJ869137_KU52_2003' 'THA_FJ869138_KU53_2003'
'THA_FJ869139_KU66_2003' 'CHI_GQ169553_Vac2_2007'
'CHI_GQ857596_CPV05_01_2005' 'CHI_GQ857599_CPV05_04_2005'
'CHI_GQ857600_CPV06_01_2006' 'CHI_GQ857601_CPV06_02_2006'
'CHI_GQ857605_CPV07_03_2007' 'CHI_GQ857609_CPV08_01_2008'
'CHI_GQ857612_CPV08_04_2008' 'VAC_GU212790_primodog_2009'
'VAC_GU212791_vanguard_2009' 'ITA_GU362932_cat11_2008'
'CHI_GU392236_fox_HB1_2009' 'CHI_GU392237_fox_HB2_2009'
'CHI_GU392239_raccoondog_HB6_2009'
'CHI_GU392240_raccoondog_HB3_2009'
'CHI_GU392241_raccoondog_HB1_2009'
'CHI_GU392242_raccoondog_HB10_2009'

'CHI_GU392244_raccoondog_HB7_2009' 'CHI_GU569939_2a_YN0202_2002'
'CHI_GU569940_2b_YN0203_2002' 'CHI_GU569942_2a_JL0202_2002'
'CHI_GU569943_YB8301_1983' 'CHI_GU569946_2a_JL0201_2002'
'CHI_GU569948_2a_CC8601_1986' 'SAF_HQ602969_22_10SA_2010'
'NIG_HQ602992_19_10_2010' 'NIG_HQ602995_15_10_2010'
'ARG_JF414818_Arg32_2008' 'ARG_JF414819_Arg35_2008'
'ARG_JF414820_Arg44_2009' 'ARG_JF414821_Arg48_2009'
'RUS_JN033694_Laika_1993' 'VAC_JN625219_INDIA_vac1_2011'
'VAC_JN625220_INDIA_vac2_2011' 'VAC_JN625221_INDIA_vac3_2011'
'VAC_JN625222_INDIA_vac4_2011' 'VAC_JN625223_INDIA_vac5_2011'
'VAC_JN625224_INDIA_vac6_2011' 'USA_JN867598_Bobcat_KS_44_2010'
'USA_JN867599_Raccoon_KY_39552_2009'
'USA_JN867602_2b_Dog_CA_148743_2008'
'USA_JN867603_2b_Dog_KS_81213_2009'
'USA_JN867604_Dog_IL_137654_2008'
'USA_JN867605_2b_Dog_US_142805_2009'
'USA_JN867610_Raccoon_VA_118_A_2007'
'USA_JN867611_Raccoon_KY_358_B_2009'
'USA_JN867618_Raccoon_WI_37_2010' 'USA_JX475231_CO_280_2011'
'USA_JX475233_SC_182_A_2011' 'USA_JX475234_ME_258_2011'
'USA_JX475237_CT_372_2011' 'USA_JX475239_GA_06_2011'
'USA_JX475240_AZ_16382_01_1999' 'USA_JX475242_WI_18268_2002'
'USA_JX475243_ID_22772_2009' 'USA_JX475246_CO_2503_2010'
'USA_JX475247_CO_1246_2010' 'USA_JX475248_CO_1102_2011'
'USA_JX475250_CO_728_2010' 'USA_JX475251_CO_2235_2009'
'USA_JX475252_CO_1316_2010' 'USA_JX475260_CO_704_2010'
'USA_JX475273_MT_909_2012' 'USA_JX475278_AR_1069_2012'
'USA_JX475279_TN_1_2011' 'USA_JX475284_TN_26_2011'
'URU_KC196081_2c_M95_2007' 'URU_KC196083_2c_M82_2007'
'URU_KC196085_2c_M57_2007' 'URU_KC196086_2c_M55_2006'
'URU_KC196089_2c_M349_2011' 'URU_KC196091_2c_M326_2011'
'URU_KC196093_2c_M307_2011' 'URU_KC196096_2c_M247_2010'
'URU_KC196097_2c_M242_2010' 'URU_KC196101_2c_M187_2009'
'URU_KC196102_2c_M185_2009' 'URU_KC196105_2c_M152_2008'
'URU_KC196107_2c_M129_2008' 'ECU_KF149962_2c_ME1_2012'
'ECU_KF149963_2c_ME10_2012' 'ECU_KF149964_2c_ME23_2012'
'ECU_KF149969_2c_ME31_2012' 'ECU_KF149971_2c_ME32_2012'
'ECU_KF149984_2c_ME28_2012' 'ITA_KF373571_2a_685_1999'
'ITA_KF373577_2a_714_2001' 'ITA_KF373580_2a_581_2003'
'ITA_KF373592_2a_329_2008' 'ITA_KF373611_2a_409_2010'
'ITA_KF385388_2a_Sicily_X83090_2009' 'HUN_KF539793_H_5_2012'
'HUN_KF539794_H_7_2012' 'HUN_KF539795_H_8_2012'
'HUN_KF539796_H_9_2012' 'HUN_KF539797_H_11_2012'
'HUN_KF539798_H_31_2012' 'HUN_KF539799_H_39_2012'
'HUN_KF539800_H_27_2012' 'HUN_KF539801_H_25_2012'
'HUN_KF539803_H_2_2012' 'HUN_KF539804_H_212_2012'
'HUN_KF539805_H_36_2012' 'CHI_KF803600_2010_BJ_A68_2010'
'CHI_KF803602_2010_BJ_A72_2010' 'CHI_KF803615_2011_BJ_B25_2011'
'CHI_KJ170679_raccoondog_Heb10_2_2010'
'CHI_KJ170680_raccoondog_HLJ11_1_2011'
'CHI_KJ194463_raccoondog_HeB10_3_2010'
'USA_KJ813827_Fisher_F1M111211_2013'
'USA_KJ813828_Fisher_F1F010712_2013'
'USA_KJ813831_Fisher_ND_17_2013' 'USA_KJ813832_Fisher_ND_14_2013'

```
'USA_KJ813835_Fisher_ND_19_2013' 'USA_KJ813842_Bobcat_ND_502_2013'
'USA_KJ813843_Bobcat_ND_1160_2013' 'USA_KJ813844_Bobcat_ND_885_2013'
'USA_KJ813846_Bobcat_ND_974_2013' 'USA_KJ813848_Bobcat_ND_1162_2013'
'USA_KJ813851_Bobcat_ND_1168_2013'
'USA_KJ813852_Bobcat_ND_1170_2013' 'USA_KJ813854_Puma_ND_F205_2013'
'USA_KJ813858_Puma_ND_F93_2013' 'USA_KJ813870_Raccoon_TX_1_2013'
'USA_KJ813873_Graywolf_MI_850_2012'
'USA_KJ813881_Graywolf_MI_832_2012'
'USA_KJ813882_Raccoon_NJ_1423_2012'
'USA_KJ813888_Coyote_MT_878_2012' 'USA_KJ813890_Redfox_MA_197_2012'
'USA_KJ813892_Coyote_AK_218_2013' 'ARG_KM236569_Cuba_2013'
'ARG_KM236572_NNGag_2012' 'URU_KM457103_2c_UY12_2006'
'URU_KM457104_2c_UY47_2006' 'URU_KM457106_2c_UY55_2006'
'URU_KM457107_2c_UY72_2007' 'URU_KM457108_2c_UY82_2007'
'URU_KM457109_2c_UY95_2007' 'URU_KM457111_2c_UY120_2008'
'URU_KM457112_2c_UY135_2008' 'URU_KM457113_2c_UY152_2009'
'URU_KM457116_2c_UY185_2009' 'URU_KM457117_2c_UY187_2009'
'URU_KM457120_2c_UY242_2010' 'URU_KM457121_2c_UY247_2010'
'URU_KM457122_2c_UY258_2010' 'URU_KM457123_2c_UY261_2008'
'URU_KM457124_2c_UY307_2011' 'URU_KM457125_2c_UY317_2011'
'URU_KM457126_2c_UY318_2010' 'URU_KM457127_2c_UY326_2011'
'URU_KM457129_2c_UY349_2011' 'URU_KM457130_2c_UY354_2011'
'URU_KM457131_2c_UY368_2011' 'URU_KM457142_2c_UY370_2011'
'THA_KP715690_VT28_2014' 'THA_KP715691_VT43_2014'
'THA_KP715716_VT143_2014' 'HRV_KP859574_2c_HR442_2014'
'HRV_KP859575_2c_HR774_2014' 'HRV_KP859576_2c_HR793_2014'
'HRV_KP859577_2c_HR856_2014' 'HRV_KP859578_2c_HR859_2014'
'POR_KT275252_2c_PT013_12_2012' 'POR_KT275253_2c_PT036_12_2012'
'POR_KT275255_2c_PT238_14_2014' 'ITA_KU508407_2c_25835_09_2009'
'AUS_KU508691_2c_HB_2015' 'AUS_KU508692_2c_FH_2015'
'AUS_KU508693_2c_LW_2015' 'POR_KU662349_greywolf_W33_1996'
'POR_KU662350_greywolf_W52_2005' 'ITA_KX434454_29451_09_2009'
'ITA_KX434456_45361_09_2009' 'ITA_KX434457_987_10_2010'
'ITA_KX434458_2323_11_2011' 'ITA_KX434459_27692_1_11_2011'
'ITA_KX434460_52238_12_2012' 'IND_KX469432_newCPV_2b_Hiller_2011'
'BRA_KY073269_UFMT_2015' 'VAC_KY083089_Singapore_2016'
'VAC_KY083090_Singapore_2016' 'JPN_LC270891_2b_9985_2017'
'USA_M10989_1985' 'USA_M19296_CPV_N_1988'
'USA_M23255_FPV_Cornell320_1988' 'USA_M24000_FPV_CPV_31_1988'
'USA_M24003_FPV_CPV_15_1988' 'USA_M38245_1990' 'USA_M74849_39_1995'
'USA_M74852_133_1995' 'USA_U22186_CPV_128_1995'
'FIN_U22192_raccoondog_RD_80_1980' 'FIN_U22193_raccoondog_RD87_1987'
'USA_U22896_cat_1990' 'TAW_U72695_2a_T4_1996'
'TAW_U72696_2b_T10_1996' 'POL_Z46651_46_1994'
'KOR_EF599097_2b_DH326_2006' 'KOR_EF599098_2c_Pome_2006' ;
END;

BEGIN CHARACTERS;
    DIMENSIONS NCHAR = 1743;
    FORMAT
        DATATYPE = DNA
        GAP=-
        MISSING=?
    ;
```

```
MATRIX
    'VIE_AB054215_2a_cat_V120_2000'
ATGAGTGATGGAGCAGTTCAACCAGACGGTGGTCAGCCTGCTGTCAGAAATGAAAGAGCTACAGGATC
TGGGAACGGGTCTGGAGGCGGGGGTGGTGGTGGTTCTGGGGGTGTGGGGATTTCTACGGGTACTTTCA
ATAATCAGACGGAATTTAAATTTTTGGAAAACGGATGGGTGGAAATCACAGCAAACTCAAGCAGACTT
GTACATTTAAATATGCCAGAAAGTGAAAATTATAGAAGAGTGGTTGTAAATAATTTGGATAAAACTGC
AGTTAACGGAAACATGGCTTTAGATGATACTCATGCACAAATTGTAACACCTTGGTCATTGGTTGATG
CAAATGCTTGGGGAGTTTGGTTTAATCCAGGAGATTGGCAACTAATTGTTAATACTATGAGTGAGTTG
CATTTAGTTAGTTTTGAACAAGAAATTTTTAATGTTGTTTTAAAGACTGTTTCAGAATCTGCTACTCA
GCCACCAACTAAAGTTTATAATAATGATTTAACTGCATCATTGATGGTTGCATTAGATAGCAATAATA
CTATGCCATTTACTCCAGCAGCTATGAGATCTGAGACATTGGGTTTTTATCCATGGAAACCAACCATA
CCAACTCCATGGAGATATTATTTTCAATGGGATAGAACATTAATACCATCTCATACTGGAACTAGTGG
CACACCAACAAATATATACCATGGTACAGATCCAGATGATGTTCAATTTTATACTATTGAAAATTCTG
TGCCAGTACACTTACTAAGGACAGGTGATGAATTTGCTACAGGAACATTTTTTTTTGATTGTAAACCA
TGTAGACTAACACATACATGGCAAACAAATAGAGCATTGGGCTTACCACCATTTCTAAATTCTTTGCC
TCAAGCTGAAGGAGGTACTAACTTTGGTTATATAGGAGTTCAACAAGATAAAAGACGTGGTGTAACTC
AAATGGGAAATACAAACTATATTACTGAAGCTACTATTATGAGACCAGCTGAGGTTGGTTATAGTGCA
CCATATTATTCTTTTGAGGCGTCTACACAAGGGCCATTTAAAACACCTATTGCAGCAGGACGGGGGGG
AGCGCAAACAGATGAAAATCAAGCAGCAGATGGTGATCCAAGATATGCATTTGGTAGACAACATGGTC
AAAAAACTACCACAACAGGAGAAACACCTGAGAGATTTACATATATAGCACATCAAGATACAGGAAGA
TATCCAGAAGGAGATTGGATTCAAAATATTAACTTTAACCTTCCTGTAACAAATGATAATGTATTGCT
ACCAACAGATCCAATTGGAGGTAAAACAGGAATTAACTATACTAATATATTTAATACTTATGGTCCTT
TAACTGCATTAAATAATGTACCACCAGTTTATCCAAATGGTCAAATTTGGGATAAAGAATTTGATACT
GACTTAAAACCAAGACTTCATGTAAATGCACCATTTGTTTGTCAAAATAATTGTCCTGGTCAATTATT
TGTAAAAGTTGCGCCTAATTTAACAAATGAATATGATCCTGATGCATCTGCTAATATGTCAAGAATTG
TAACTTACTCAGATTTTTGGTGGAAAGGTAAATTAGTATTTAAAGCTAAACTAAGAGCCTCTCATACT
TGGAATCCAATTCAGCAAATGAGTATTAATGTAGATAACCAATTTAACTATGTACCAAGTAATATTGG
AGGTATGAAGATTGTATATGAAAAATCTCAACTAGCACCTAGA
    'VIE_AB054217_2a_cat_V154_2000'
ATGAGTGATGGAGGAGTTCAACCAGACGGTGGTCAACCTGCTGTCAGAAATGAAAGAGCTACAGGATC
TGGGAACGGGTCTGGAGGCGGGGGTGGTGGTGGTTCTGGGGGTGTGGGGATTTCTACGGGTACTTTTA
ATAATCAGACGGAATTTAAATTTTTGGAAAACGGATGGGTGGAAATCACAGCAAACTCAAGCAGACTT
GTACATTTAAATATGCCAGAAAGTGAAAATTATAGAAGAGTGGTTGTAAATAATTTGGATAAAACTGC
AGTTAACGGAAACATGGCTTTAGATGATACTCATGCACAAATTGTAACACCTTGGTCATTGGTTGATG
CAAATGCTTGGGGAGTTTGGTTTAATCCAGGAGATTGGCAACTAATTGTTAATACTATGAGTGAGTTG
CATTTAGTTAGTTTTGAACAAGAAATTTTTAATGTTGTTTTAAAGACTGTTTCAGAATCTGCTACTCA
GCCACCAACTAAAGTTTATAATAATGATTTAACTGCATCATTGATGGTTGCATTAGATAGCAATAATA
CTATGCCATTTACTCCAGCAGCTATGAGATCTGAGACATTGGGTTTTTATCCATGGAAACCAACCATA
CCAACTCCATGGAGATATTATTTTCAATGGGATAGAACATTAATACCATCTCATACTGGAACTAGTGG
CACACCAACAAATATATACCATGGTACAGATCCAGATGATGTTCAATTTTATACTATTGAAAATTCTG
TGCCAGTACACTTACTAAGAACAGGTGATGAATTTGCTACAGGAACATTTTTTTTTGATTGTAAACCA
TGTAGACTAACACATACATGGCAAACAAATAGAGCATTGGGCTTACCACCATTTCTAAATTCTTTGCC
TCAAGCTGAAGGAGGTACTAACTTTGGTTATATAGGAGTTCAACAAGATAAAAGACGTGGTGTAACTC
AAATGGGAAATACAAACTATATTACTGAAGCTACTATTATGAGACCAGCTGAGGTTGGTTATAGTGCA
CCATATTATTCTTTTGAGGCGTCTACACAAGGGCCATTTAAAACACCTATTGCAGCAGGACGGGGGGG
AGCGCAAACAGATGAAAATCAAGCAGCAGATGGTGATCCAAGATATGCATTTGGTAGACAACATGGTC
AAAAAACTACCACAACAGGAGAAACACCTGAGAGATTTACATATATAGCACATCAAGATACAGGAAGA
TATCCAGAAGGAGATTGGATTCAGAATATTAACTTTAACCTTCCTGTAACAAATGATAATGTATTGCT
ACCGACAGATCCAATTGGAGGTAAAACAGGAATTAACTATACTAATATATTTAATACTTATGGTCCTT
TAACTGCATTAAATAATGTACCACCAGTTTATCCAAATGGTCAAATTTGGGATAAAGAATTTGATACT
GACTTAAAACCAAGACTTCATGTAAATGCACCATTTGTTTGTCAAAATAATTGTCCTGGTCAATTATT
TGTAAAAGTTGCGCCTAATTTAACAAATGAATATGATCCTGATGCATCTGCTAATATGTCAAGAATTG
TAACTTACTCAGATTTTTGGTGGAAAGGTAAATTAGTATTTAAAGCTAAACTAAGAGCCTCTCATACT
```

TGGAATCCAATTCAACAAATGAGTATTAATGTAGATAACCAATTTAACTATGTACCAAGTAATATTGG
AGGTATGAAGATTGTATATGAAAAATCTCAACTAGCACCTAGA

'VIE_AB054218_2b_cat_V123_2000'

ATGAGTGATGGAGCAGTTCAACCAGACGGTGGTCAACCTGCTGTCAGAAATGAAAGAGCTACAGGATC
TGGGAACGGGTCTGGAGGCGGGGGTGGTGGTGGTTCTGGGGGTGTGGGGATTTCTACGGGTACTTTCA
ATAATCAGACAGAATTTAAATTTTTGGAAAACGGATGGGTGGAAATCACAGCAAACTCAAGCAGACTT
GTACATTTAAATATGCCAGAAAGTGAAAATTATAGAAGAGTGGTTGTAAATAATTTGGATAAAACTGC
AGTTAACGGAAACATGGCTTTAGATGATACTCATGCACAAATTGTAACACCTTGGTCATTGGTTGATG
CAAATGCTTGGGGAGTTTGGTTTAATCCAGGAGATTGGCAACTAATTGTTAATACTATGAGTGAGTTG
CATTTAGTTAGTTTTGAACAAGAAATTTTTAATGTTGTTTTAAAGACTGTTTCAGAATCTGCTACTCA
GCCACCAACTAAAGTTTATAATAATGATTTAACTGCATCATTGATGGTTGCATTAGATAGTAATAATA
CTATGCCATTTACTCCAGCAGCTATGAGATCTGAGACATTGGGTTTTTATCCATGGAAACCAACCATA
CCAACTCCATGGAGATATTATTTTCAATGGGATAGAACATTAATACCATCTCATACTGGAACTAGTGG
CACACCAACAAATATATACCATGGTACAGATCCAGATGATGTTCAATTTTATACTATTGAAAATTCTG
TGCCAGTACACTTACTAAGAACAGGTGATGAATTTGCTACAGGAACATTTTTTTTTGATTGTAAACCA
TGTAGACTAACACATACATGGCAAACAAATAGAGCATTGGGCTTACCACCATTTCTAAATTCTTTGCC
TCAAGCTGAAGGAGGTACTAACTTTGGTTATATAGGAGTTCAACAAGATAAAAGACGTGGTGTAACTC
AAATGGGAAATACAAACTATATTACTGAAGCTACTATTATGAGACCAGCTGAGGTTGGTTATAGTGCA
CCATATTATTCTTTTGAGGCGTCTACACAAGGGCCATTTAAAACACCTATTGCAGCAGGACGGGGGGG
AGCGCAAACAGATGAAAATCAAGCAGCAGATGGTGATCCAAGATATGCATTTGGTAGACAACATGGTC
AAAAAACTACCACAACAGGAGAAACACCTGAGAGATTTACATATATAGCACATCAAGATACAGGAAGA
TATCCAGAAGGAGATTGGATTCAAAATATTAACTTTAACCTTCCTGTAACAGATGATAATGTATTGCT
ACCAACAGATCCAATTGGAGGTAAAACAGGAATTAACTATACTAATATATTTAATACTTATGGTCCTT
TAACTGCATTAAATAATGTACCACCAGTTTATCCAAATGGTCAAATTTGGGATAAAGAATTTGATACT
GACTTAAAACCAAGACTTCATGTAAATGCACCATTTGTTTGTCAAAATAATTGTCCTGGTCAATTATT
TGTAAAAGTTGCGCCTAATTTAACAAATGAATATGATCCTGATGCATCTGCTAATATGTCAAGAATTG
TAACTTACTCAGATTTTTGGTGGAAAGGTAAATTAGTATTTAAAGCTAAACTAAGAGCCTCTCATACT
TGGAATCCAATTCAACAAATGAGTATTAATGTAGATAACCAATTTAACTATGTACCAAGTAATATTGG
AGGTATGAAAATTGTATATGAAAAATCTCAACTAGCACCTAGA

'VIE_AB054219_2b_cat_V209_2000'

ATGAGTGATGGAGCAGTTCAACCAGACGGTGGTCAACCTGCTGTCAGAAATGAAAGAGCTACAGGATC
TGGGAACGGGTCTGGAGGCGGGGGTGGTGGTGGTTCTGGGGGTGTGGGGATTTCTACGGGTACTTTCA
ATAATCAGACAGAATTTAAATTTTTGGAAAACGGATGGGTGGAAATCACAGCAAACTCAAGCAGACTT
GTACATTTAAATATGCCAGAAAGTGAAAATTATAGAAGAGTGGTTGTAAATAATTTGGATAAAACTGC
AGTTAACGGAAACATGGCTTTAGATGATACTCATGCACAAATTGTAACACCTTGGTCATTGGTTGATG
CAAATGCTTGGGGAGTTTGGTTTAATCCAGGAGATTGGCAACTAATTGTTAATACTATGAGTGAGTTG
CATTTAGTTAGTTTTGAACAAGAAATTTTTAATGTTGTTTTAAAGACTGTTTCAGAATCTGCTACTCA
GCCACCAACTAAAGTTTATAATAATGATTTAACTGCATCATTGATGGTTGCATTAGATAGTAATAATA
CTATGCCATTTACTCCAGCAGCTATGAGATCTGAGACATTGGGTTTTTATCCATGGAAACCAACCATA
CCAACTCCATGGAGATATTATTTTCAATGGGATAGAACATTAATACCATCTCATACTGGAACTAGTGG
CACACCAACAAATATATACCATGGTACAGATCCAGATGATGTTCAATTTTATACTATTGAAAATTCTG
TGCCAGTACACTTACTAAGAACAGGTGATGAATTTGCTACAGGAACATTTTTTTTTGATTGTAAACCA
TGTAGACTAACACATACATGGCAAACAAATAGAGCATTGGGCTTACCACCATTTCTAAATTCTTTGCC
TCAAGCTGAAGGAGGTACTAACTTTGGTTATATAGGAGTTCAACAAGATAAAAGACGTGGTGTAACTC
AAATGGGAAATACAAACTATATTACTGAAGCTACTATTATGAGACCAGCTGAGGTTGGTTATAGTGCA
CCATATTATTCTTTTGAGGCGTCTACACAAGGGCCATTTAAAACACCTATTGCAGCAGGACGGGGGGG
AGCGCAAACAGATGAAAATCAAGCAGCAGATGGTGATCCAAGATATGCATTTGGTAGACAACATGGTC
AAAAAACTACCACAACAGGAGAAACACCTGAGAGATTTACATATATAGCACATCAAGATCCAGGAAGA
TATCCAGAAGGAGATTGGATTCAAAATATTAACTTTAACCTTCCTGTAACAGATGATAATGTATTGCT
ACCAACAGATCCAATTGGAGGTAAAACAGGAATTAACTATACTAATATATTTAATACTTATGGTCCTT
TAACTGCATTAAATAATGTACCACCAGTTTATCCAAATGGTCAAATTTGGGATAAAGAATTTGATACT
GACTTAAAACCAAGACTTCATGTAAATGCACCATTTGTTTGTCAAAATAATTGTCCTGGTCAATTATT
TGTAAAAGTTGCGCCTAATTTAACAAATGAATATGATCCTGATGCATCTGCTAATATGTCAAGAATTG
TAACTTACTCAGATTTTTGGTGGAAAGGTAAATTAGTATTTAAAGCTAAACTAAGAGCCTCTCATACT

TGGAATCCAATTCAACAAATGAGTATCAATGTAGATAACCAATTTAACTATGTACCAAGTAATATTGG
AGGTATGAAAATTGTATATGAAAAATCTCAACTAGCACCTAGA

'VIE_AB054220_2b_cat_V217_2000'

ATGAGTGATGGAGCAGTTCAACCAGACGGTGGTCAACCTGCTGTCAGAAATGAAAGAGCTACAGGATC
TGGGAACGGGTCTGGAGGCGGGGGTGGTGGTGGTTCTGGGGGTGTGGGGATTTCTACGGGTACTTTCA
ATAATCAGACAGAATTTAAATTTTTGGAAAACGGATGGGTGGAAATCACAGCAAACTCAAGCAGACTT
GTACATTTAAATATGCCAGAAAGTGAAAATTATAGAAGAGTGGTTGTAAATAATTTGGATAAAACTGC
AGTTAACGGAAACATGGCTTTAGATGATACTCATGCACAAATTGTAACACCTTGGTCATTGGTTGATG
CAAATGCTTGGGGAGTTTGGTTTAATCCAGGAGATTGGCAGCTAATTGTTAATACTATGAGTGAGTTG
CATTTAGTTAGTTTTGAACAAGAAATTTTTAATGTTGTTTTAAAGACTGTTTCAGAATCTGCTACTCA
GCCACCAACTAAAGTTTATAATAATGATTTAACTGCATCATTGATGGTTGCATTAGATAGTAATAATA
CTATGCCATTTACTCCAGCAGCTATGAGATCTGAGACATTGGGTTTTTATCCATGGAAACCAACCATA
CCAACTCCATGGAGATATTATTTTCAATGGGATAGAACATTAATACCATCTCATACTGGAACTAGTGG
CACACCAACAAATATATACCATGGTACAGATCCAGATGATGTTCAATTTTATACTATTGAAAATTCTG
TGCCAGTACACTTACTAAGAACAGGTGATGAATTTGCTACAGGAACATTTTTTTTTGATTGTAAACCA
TGTAGACTAACACATACATGGCAAACAAATAGAGCATTGGGCTTACCACCATTTCTAAATTCTTTGCC
TCAAGCTGAAGGAGGTACTAACTTTGGTTATATAGGAGTTCAACAAGATAAAAGACGTGGTGTAACTC
AAATGGGAAATACAAACTATATTACTGAAGCTACTATTATGAGACCAGCTGAGGTTGGTTATAGTGCA
CCATATTATTCTTTTGAGGCGTCTACACAAGGGCCATTTAAAACACCTATTGCAGCAGGACGGGGGGG
AGCGCAAACAGATGAAAATCAAGCAGCAGATGGTGATCCAAGATATGCATTTGGTAGGCAACATGGTC
AAAAAACTACCACAACAGGAGAAACACCTGAGAGATTTACATATATAGCACATCAAGATACAGGAAGA
TATCCAGAAGGAGATTGGATTCAAAATATTAACTTTAACCTTCCTGTAACAGATGATAATGTATTGCT
ACCAACAGATCCAATTGGAGGTAAAACAGGAATTAACTATACTAATATATTTAATACTTATGGTCCTT
TAACTGCATTAAATAATGTACCACCAGTTTATCCAAATGGTCAAATTTGGGATAAAGAATTTGATACT
GACTTAAAACCAAGACTTCATGTAAATGCACCATTTGTTTGTCAAAATAATTGTCCTGGTCAATTATT
TGTAAAAGTTGCGCCTAATTTAACAAATGAATATGATCCTGATGCATCTGCTAATATGTCAAGAATTG
TAACTTACTCAGATTTTTGGTGGAAAGGTAAATTAGTATTTAAAGCTAAACTAAGAGCCTCTCATACT
TGGAATCCAATTCAACAAATGAGTATCAATGTAGATAACCAATTTAACTATGTACCAAGTAATATTGG
AGGTATGAAAATTGTATATGAAAAATCTCAACTAGCACCTAGA

'VIE_AB054221_2b_leopard_V204_2000'

ATGAGTGATGGAGCAGTTCAACCAGACGGTGGTCAACCTGCTGTCAGAAATGAAAGAGCTACAGGATC
TGGGAACGGGTCTGGAGGCGGGGGTGGTGGTGGTTCTGGGGGTGTGGGGATTTCTACGGGTACTTTCA
ATAATCAGACAGAATTTAAATTTTTGGAAAACGGATGGGTGGAAATCACAGCAAACTCAAGCAGACTT
GTACATTTAAATATGCCAGAAAGTGAAAATTATAGAAGAGTGGTTGTAAATAATTTGGATAAAACTGC
AGTTAACGGAAACATGGCTTTAGATGATACTCATGCACAAATTGTAACACCTTGGTCATTGGTTGATG
CAAATGCTTGGGGAGTTTGGTTTAATCCAGGAGATTGGCAACTAATTGTTAATACTATGAGTGAGTTG
CATTTAGTTAGTTTTGAACAAGAAATTTTTAATGTTGTTTTAAAGACTGTTTCAGAATCTGCTACTCA
GCCACCAACTAAAGTTTATAATAATGATTTAACTGCATCATTGATGGTTGCATTAGATAGTAATAATA
CTATGCCATTTACTCCAGCAGCTATGAGATCTGAGACATTGGGTTTTTATCCATGGAAACCAACCATA
CCAACTCCATGGAGATATTATTTTCAATGGGATAGAACATTAATACCATCTCATACTGGAACTAGTGG
CACACCAACAAATATATACCATGGTACAGATCCAGATGATGTTCAATTTTATACTATTGAAAATTCTG
TGCCAGTACACTTACTAAGAACAGGTGATGAATTTGCTACAGGAACATTTTTTTTTGATTGTAAACCA
TGTAGACTAACACATACATGGCAAACAAATAGAGCATTGGGCTTACCACCATTTCTAAATTCTTTGCC
TCAAGCTGAAGGAGGTACTAACTTTGGTTATATAGGAGTTCAACAAGATAAAAGACGTGGTGTAACTC
AAATGGGAAATACAAACTATATTACTGAAGCTACTATTATGAGACCAGCTGAGGTTGGTTATAGTGCA
CCATATTATTCTTTTGAGGCGTCTACACAAGGGCCATTTAAAACACCTATTGCAGCAGGACGGGGGGG
AGCGCAAACAGATGAAAATCAAGCAGCAGATGGTGATCCAAGATATGCATTTGGTAGACAACATGGTC
AAAAAACTACCACAACAGGAGAAACACCTGAGAGATTTACATATATAGCACATCAAGATACAGGAAGA
TATCCAGAAGGAGATTGGATTCAAAATATTAACTTTAACCTTCCTGTAACAGATGATAATGTATTGCT
ACCAACAGATCCAATTGGAGGTAAAGCAGGAATTAACTATACTAATATATTTAATACTTATGGTCCTT
TAACTGCATTAAATAATGTACCACCAGTTTATCCAAATGGTCAAATTTGGGATAAAGAATTTGATACT
GACTTAAAACCAAGACTTCATGTAAATGCACCATTTGTTTGTCAAAATAATTGTCCTGGTCAATTATT
TGTAAAAGTTGCGCCTAATTTAACAAATGAATATGATCCTGATGCATCTGCTAATATGTCAAGAATTG
TAACTTACTCAGATTTTTGGTGGAAAGGTAAATTAGTATTTAAAGCTAAACTAAGAGCCTCTCATACT

TGGAATCCAATTCAACAAATGAGTATCAATGTAGATAACCAATTTAACTATGTACCAAGTAATATTGG
AGGTATGAAAATTGTATATGAAAAATCTCAACTAGCACCTAGA

'VIE_AB054223_2c_leopard_V140_2000'

ATGAGTGATGGAGCAGTTCAACCAGACGGTGGTCAACCTGCTGTCAGAAATGAAAGAGCTACAGGATC
TGGGAACGGGTCTGGAGGCGGGGGTGGTGGTGGTTCTGGGGGTGTGGGGATTTCTACGGGTACTTTCA
ATAATCAGACGGAATTTAAATTTTTGGAAAACGGATGGGTGGAAATCACAGCAAACTCAAGCAGACTT
GTACATTTAAATATGCCAGAAAGTGAAAATTATAGAAGAGTGGTTGTAAATAATTTGGATAAAACTGC
AGTTAACGGAAACATGGCTTTAGATGATACTCATGCACAAATTGTAACACCTTGGTCATTGGTTGATG
CAAATGCTTGGGGAGTTTGGTTTAATCCAGGAGATTGGCAACTAATTGTTAATACTATGAGTGAGTTG
CATTTAGTTAGTTTTGAACAAGAAATTTTTAATGTTGTTTTAAAGACTGTTTCAGAATCTGCTACTCA
GCCACCAACTAAAGTTTATAATAATGATTTAACTGCATCATTGATGGTTGCATTAGATAGCAATAATA
CTATGCCATTTACTCCAGCAGCTATGAGATCTGAGACATTGGGTTTTTATCCATGGAAACCAACCATA
CCAACTCCATGGAGATATTATTTTCAATGGGATAGAACATTAATACCATCTCATACTGGAACTAGTGG
CACACCAACAAATATATACCATGGTACAGATCCAGATGATGTTCAATTTTATACTATTGAAAATTCTG
TGCCAGTACACTTACTAAGAACAGGTGATGAATTTGCTACAGGAACATTTTTTTTTGATTGTAAACCA
TGTAGACTAACACATACATGGCAAACAAATAGAGCATTGGGCTTACCACCATTTCTAAATTCTTTGCC
TCAAGCTGAAGGAGATACTAACTTTGGTTATATAGGAGTTCAACAAGATAAAAGACGTGGTGTAACTC
AAATGGGAAATACAAACTATATTACTGAAGCTACTATTATGAGACCAGCTGAGGTTGGTTATAGTGCA
CCATATTATTCTTTTGAGGCGTCTACACAAGGGCCATTTAAAACACCTATTGCAGCAGGACGGGGGGG
AGCGCAAACAGATGAAAATCAAGCAGCAGATGGTGATCCAAGATATGCATTTGGTAGACAACATGGTC
AAAAAACTACCACAACAGGAGAAACACCTGAGAGATTTACATATATAGCACATCAAGATACAGGAAGA
TATCCAGAAGGAGATTGGATTCAAAATATTAACTTTAACCTTCCTGTAACAAATGATAATGTATTGCT
ACCAACAGATCCAATTGGAGGTAAAACAGGAATTAACTATACTAATATATTTAATACTTATGGTCCTT
TAACTGCATTAAATAATGTACCACCAGTTTATCCAAATGGTCAAATTTGGGATAAAGAATTTGATACT
GACTTAAAACCAAGACTTCATGTAAATGCACCATTTGTTTGTCAAAATAATTGTCCTGGTCAATTATT
TGTAAAAGTTGCGCCTAATTTAACAAATGAATATGATCCTGATGCATCTGCTAATATGTCAAGAATTG
TAACTTACTCAGATTTTTGGTGGAAAGGTAAATTAGTATTTAAAGCTAAACTAAGAGCCTCTCATACT
TGGAATCCAATTCAGCAAATGAGTATTAATGTAGATAACCAATTTAACTATGTACCAAGTAATATTGG
AGGTATGAAAATTGTATATGAAAAATCTCAACTAGCACCTAGA

'VIE_AB054224_2c_leopard_V203_2000'

ATGAGTGATGGAGCAGTTCAACCAGACGGTGGTCAACCTGCTGTCAGAAATGAAAGAGCTACAGGATC
TGGGAACGGGTCTGGAGGCGGGGGTGGTGGTGGTTCTGGGGGTGTGGGGATTTCTACGGGTACTTTCA
ATAATCAGACAGAATTTAAATTTTTGGAAAACGGATGGGTGGAAATCACAGCAAACTCAAGCAGACTT
GTACATTTAAATATGCCAGAAAGTGAAAATTATAGAAGAGTGGTTGTAAATAATTTGGATAAAACTGC
AGTTAACGGAAACATGGCTTTAGATGATACTCATGCACAAATTGTAACACCTTGGTCATTGGTTGATG
CAAATGCTTGGGGAGTTTGGTTTAATCCAGGAGATTGGCAACTAATTGTTAATACTATGAGTGAGTTG
CATTTAGTTAGTTTTGAACAAGAAATTTTTAATGTTGTTTTAAAGACTGTTTCAGAATCTGCTACTCA
GCCACCAACTAAAGTTTATAATAATGATTTAACTGCATCATTGATGGTTGCATTAGATAGTAATAATA
CTATGCCGTTTACTCCAGCAGCTATGAGATCTGAGACATTGGGTTTTTATCCATGGAAACCAACCATA
CCAACTCCATGGAGATATTATTTTCAATGGGATAGAACATTAATACCATCTCATACTGGAACTAGTGG
CACACCAACAAATATATACCATGGTACAGATCCAGATGATGTTCAATTTTATACTATTGAAAATTCTG
TGCCAGTACACTTACTAAGAACAGGTGATGAATTTGCTACAGGAACATTTTTTTTTGATTGTAAACCA
TGTAGACTAACACATACATGGCAAACAAATAGAGCATTGGGCTTACCACCATTTCTAAATTCTTTGCC
TCAAGCTGAAGGAGATACTAACTTTGGTTATATAGGAGTTCAACAAGATAAAAGACGTGGTGTAACTC
AAATGGGAAATACAAACTATATTACTGAAGCTACTATTATGAGACCAGCTGAGGTTGGTTATAGTGCA
CCATACTATTCTTTTGAGGCGTCTACACAAGGGCCATTTAAAACACCTATTGCAGCAGGACGGGGGGG
AGCGCAAACAGATGAAAATCAAGCAGCAGATGGTGATCCAAGATATGCATTTGGTAGACAACATGGTC
AAAAAACTACCACAACAGGAGAAACACCTGAGAGATTTACATATATAGCACATCAAGATACAGGAAGA
TATCCAGAAGGAGATTGGATTCAAAATATTAACTTTAACCTTCCTGTAACAGATGATAATGTATTGCT
ACCAACAGATCCAATTGGAGGTAAAACAGGAATTAACTATACTAATATATTTAATACTTATGGTCCTT
TAACTGCATTAAATAATGTACCACCAGTTTATCCAAATGGTCAAATTTGGGATAAAGAATTTGATACT
GACTTAAAACCAAGACTTCATGTAAATGCACCATTTGTTTGTCAAAATAATTGTCCTGGTCAATTATT
TGTAAAAGTTGCGCCTAATTTAACAAATGAATATGATCCTGATGCATCTGCTAATATGTCAAGAATTG
TAACTTACTCAGATTTTTGGTGGAAAGGTAAATTAGTATTTAAAGCTAAACTAAGAGCCTCTCATACT

TGGAATCCAATTCAACAAATGAGTATCAATGTAGATAACCAATTTAACTATGTACCAAGTAATATTGG
AGGTATGAAAATTGTATATGAAAAATCTCAACTAGCACCTAGA
'JPN_AB115504_2c_97_008_1997'
ATGAGTGATGGAGCAGTTCAACCAGACGGTGGTCAACCTGCTGTCAGAAATGAAAGAGCTACAGGATC
TGGGAACGGGTCTGGAGGCGGGGGTGGTGGTGGTTCTGGGGGTGTGGGGATTTCTACGGGTACTTTCA
ATAATCAGACAGAATTTAAATTTTTGGAAAACGGATGGGTGGAAATCACAGCAAACTCAAGTAGACTT
GTACATTTAAATATGCCAGAAAGTGAAAATTATAGAAGAGTGGTTGTAAATAATTTGGATAAAACTGC
AGTTAACGGAAACATGGCTTTAGATGATACCCATGCACAAATTGTAACACCTTGGTCATTGGTTGATG
CAAATGCTTGGGGAGTTTGGTTTAATCCAGGAGATTGGCAACTAATTGTTAATACTATGAGTGAGTTG
CATTTAGTTAGTTTTGAACAAGAAATTTTTAATGTTGTTTTAAAGACTGTTTCAGAATCTGCTACTCA
GCCACCAACTAAAGTTTATAATAATGATTTAACTGCATCATTGATGGTTGCATTAGATAGTAATAATA
CTATGCCATTTACTCCAGCAGCTATGAGATCTGAGACATTGGGTTTTTATCCATGGAAACCAACCATA
CCAACTCCATGGAGATATTATTTTCAATGGGATAGAACATTAATACCATCTCATACTGGAACTAGTGG
GACACCAACAAATATATACCATGGTACAGATCCAGATGATGTTCAATTTTATACTATTGAAAATTCTG
TGCCAGTACACTTACTAAGAACAGGTGATGAATTTGCTACAGGAACATTTTTTTTTGATTGTAAACCA
TGTAGACTAACACATACATGGCAAACAAATAGAGCATTGGGCTTACCACCATTTCTAAATTCTTTGCC
TCAAGCTGAAGGAGGTACTAACTTTGGTTATATAGGAGTTCAACAAGATAAAAGACGTGGTGTAACTC
AAATGGGAAATACAAACTATATTACTGAAGCTACTATTATGAGACCAGCTGAGGTTGGTTATAGTGCA
CCATATTATTCTTTTGAGGCGTCTACACAAGGGCCATTTAAAACACCTATTGCAGCAGGACGGGGGGG
AGCGCAAACAGATGAAAATCAAGCAGCAGATGGTGATCCAAGATATGCATTTGGTAGACAACATGGTC
AAAAAACTACCACAACAGGAGAAACACCTGAGAGATTTACATATATAGCACATCAAGATACAGGAAGA
TATCCAGAAGGAGATTGGATTCAAAATATTAACTTTAACCTTCCTGTAACAGATGATAATGTATTGCT
ACCAACAGATCCAATTGGAGGTAAAACAGGAATTAACTATACTAATATATTTAATACTTATGGTCCTT
TAACTGCATTAAATAATGTACCACCAGTTTATCCAAATGGTCAAATTTGGGATAAAGAATTTGATACT
GACTTAAAACCAAGACTTCATGTAAATGCACCATTTGTTTGTCAAAATAATTGTCCTGGTCAATTATT
TGTAAAAGTTGCGCCTAATTTAACAAATGAATATGATCCTGATGCATCTGCTAATATGTCAAGAATTG
TAACTTACTCAGATTTTTGGTGGAAAGGTAAATTAGTATTTAAAGCTAAACTAAGAGCCTCTCATACT
TGGAATCCAATTCAACAAATGAGTATTAATGTAGATAACCAATTTAACTATGTACCAAGTAATATTGG
AGGTATGAAAATTGTATATGAAAAATCTCAACTAGCACCTAGA
'VIE_AB120720_2b_HCM_6_2003'
ATGAGTGATGGAGCAGTTCAACCAGACGGTGGTCAACCTGCTGTCAGAAATGAAAGAGCTACAGGATC
TGGGAACGGGTCTGGAGGCGGGGGTGGTGGTGGTTCTGGGGGTGTGGGGATTTCTACGGGTACTTTCA
ATAATCAGACAGAATTTAAATTTTTGGAAAACGGATGGGTGGAAATCACAGCAAACTCAAGCAGACTT
GTACATTTAAATATGCCAGAAAGTGAAAATTATAGAAGAGTGGTTGTAAATAATTTGGATAAAACTGC
AGTTAACGGAAACATGGCTTTAGATGATACTCATGCACAAATTGTAACACCTTGGTCATTGGTTGATG
CAAATGCTTGGGGAGTTTGGTTTAATCCAGGAGATTGGCAACTAATTGTTAATACTATGAGTGAGTTG
CATTTAGTTAGTTTTGAACAAGAAATTTTTAATGTTGTTTTAAAGACTGTTTCAGAATCTGCTACTCA
GCCACCAACTAAAGTTTATAATAATGATTTAACTGCATCATTGATGGTTGCATTAGATAGTAATAATA
CTATGCCATTTACTCCAGCAGCTATGAGATCTGAGACATTGGGTTTTTATCCATGGAAACCAACCATA
CCAACTCCATGGAGATATTATTTTCAATGGGATAGAACATTAATACCATCTCATACTGGAACTAGTGG
CACACCAACAAATATATACCATGGTACAGATCCAGATGATGTTCAATTTTATACTATTGAAAATTCTG
TGCCAGTACACTTACTAAGAACAGGTGATGAATTTGCTACAGGAACATTTTTTTTTGATTGTAAACCA
TGTAGACTAACACATACATGGCAAACAAATAGAGCATTGGGCTTACCACCATTTCTAAATTCTTTGCC
TCAAGCTGAAGGAGGTACTAACTTTGGTTATATAGGAGTTCAACAAGATAAAAGACGTGGTGTAACTC
AAATGGGAAATACAAACTATATTACTGAAGCTACTATTATGAGACCAGCTGAGGTTGGTTATAGTGCA
CCATATTATTCTTTTGAAGCGTCTACACAAGGGCCATTTAAAACACCTATTGCAGCAGGACGGGGGGG
AGCGCAAACAGATGAAAATCAAGCAGCAGATGGTGATCCAAGATATGCATTTGGTAGACAACATGGTC
AAAAAACTACCACAACAGGAGAAACACCTGAGAGATTTACATATATAGCACATCAAGATACAGGAAGA
TATCCAGAAGGAGATTGGATTCAAAATATTAACTTTAACCTTCCTGTAACAGATGATAATGTATTGCT
ACCAACAGATCCAATTGGAGGTAAAACAGGAATTAACTATACTAATATATTTAATACTTATGGTCCTT
TAACTGCATTAAATAATGTACCACCAGTTTATCCAAATGGTCAAATTTGGGATAAAGAATTTGATACT
GACTTAAAACCAAGACTTCATGTAAATGCACCATTTGTTTGTCAAAATAATTGTCCTGGTCAATTATT
TGTAAAAGTTGCGCCTAATTTAACAAATGAATATGATCCTGATGCATCTGCTAATATGTCAAGAATTG
TAACTTACTCAGATTTTTGGTGGAAAGGTAAATTAGTATTTAAAGCTAAACTAAGAGCCTCTCATACT

TGGAATCCAATTCAACAAATGAGTATCAATGTAGATAACCAATTTAACTATGTACCAAGTAATATTGG
AGGTATGAAAATTGTATATGAAAAATCTCAACTAGCACCTAGA
'VIE_AB120721_2b_HCM_8_2003'
ATGAGTGATGGAGCAGTTCAACCAGACGGTGGTCAATCTGCTGTCAGAAATGAAAGAGCTACAGGATC
TGGGAACGGGTCTGGAGGCGGGGGTGGTGGTGGTTCTGGGGGTGTGGGGATTTCTACGGGTACTTTCA
ATAATCAGACAGAATTTAAATTTTTGGAAAACGGATGGGTGGAAATCACAGCAAACTCAAGCAGACTT
GTACATTTAAATATGCCAGAAAGTGAAAATTATAGAAGAGTGGTTGTAAATAATTTGGATAAAACTGC
AGTTAACGGAAACATGGCTTTAGATGATACTCATGCACAAATTGTGACACCTTGGTCATTGGTTGATG
CAAATGCTTGGGGAGTTTGGTTTAATCCAGGAGATTGGCAACTAATTGTTAATACTATGAGTGAGTTG
CATTTAGTTAGTTTTGAACAAGAAATTTTTAATGTTGTTTTAAAGACTGTTTCAGAATCTGCTACTCA
GCCACCAACTAAAGTTTATAATAATGATTTAACTGCATCATTGATGGTTGCATTAGATAGTAATAATA
CTATGCCATTTACTCCAGCAGCTATGAGATCTGAGACATTGGGTTTTTATCCATGGAAACCAACCATA
CCAACTCCATGGAGATATTATTTTCAATGGGATAGAACATTAATACCATCTCATACTGGAACTAGTGG
CACACCAACAAATATATACCATGGTACAGATCCAGATGATGTTCAATTTTATACTATTGAAAATTCTG
TGCCAGTACACTTACTAAGAACAGGTGATGAATTTGCTACAGGAAAATTTTTTTTTGATTGTAAACCA
TGTAGACTAACACATACATGGCAAACAAATAGAGCATTGGGCTTACCACCATTTCTAAATTCTTTGCC
TCAAGCTGAAGGAGGTACTAACTTTGGTTATATAGGAGTTCAACAAGATAAAAGACGTGGTGTAACTC
AAATGGGAAATACAAACTATATTACTGAAGCTACTATTATGAGACCAGCTGAGGTTGGTTATAGTGCA
CCATATTATTCTTTTGAGGCGTCTACACAAGGGCCATTTAAAACACCTATTGCAGCAGGACGGGGGGG
AGCGCAAACAGATGAAAATCAAGCAGCAGATGGTGATCCAAGATATGCATTTGGTAGACAACATGGTC
AAAAAACTACCACAACAGGAGAAACACCTGAGAGATTTACATATATAGCACATCAAGATACAGGAAGA
TATCCAGAAGGAGATTGGATTCAAAATATTAACTTTAACCTTCCTGTAACAGATGATAATGTATTGCT
ACCAACAGATCCAATTGGAGGTAAAACAGGAATTAACTATACTAATATATTTAATACTTATGGTCCTT
TAACTGCATTAAATAATGTACCACCAGTTTATCCAAATGGTCAAATTTGGGATAAAGAATTTGATACT
GACTTAAAACCAAGACTTCATGTAAATGCACCATTTGTTTGTCAAAATAATTGTCCTGGTCAATTATT
TGTAAAAGTTGCGCCTAATTTAACAAATGAATATGATCCTGATGCATCTGCTAATATGTCAAGAATTG
TAACTTACTCAGATTTTTGGTGGAAAGGTAAATTAGTATTTAAAGCTAAACTAAGAGCCTCTCATACT
TGGAATCCAATTCAACAAATGAGTATCAATGTAGATAACCAATTTAACTATGTACCAAGTAATATTGG
AGGTATGAAAATTGTATATGAAAAATCTCAACTAGCACCTAGA
'VIE_AB120722_2b_HCM_18_2003'
ATGAGTGATGGAGCAGTTCAACCAGACGGTGGTCAACCTGCTGTCAGAAATGAAAGAGCTACAGGATC
TGGGAACGGGTCTGGAGGCGGGGGTGGTGGTGGTTCTGGGGGTGTGGGGATTTCTACGGGTACTTTCA
ATAATCAGACAGAATTTAAATTTTTGGAAAACGGATGGGTGGAAATCACAGCAAACTCAAGCAGACTT
GTACATTTAAATATGCCAGAAAGTGAAAATTATAGAAGAGTGGTTGTAAATAATTTGGATAAAACTGC
AGTTAACGGAAACATGGCTTTAGATGATACTCATGCACAAATTGTAACACCTTGGTCATTGGTTGATG
CAAATGCTTGGGGAGTTTGGTTTAATCCAGGAGATTGGCAACTAATTGTTAATACTATGAGTGAGTTA
CATTTAGTTAGTTTTGAACAAGAAATTTTTAATGTTGTTTTAAAGACTGTTTCAGAATCTGCTACTCA
GCCACCAACTAAAGTTTATAATAATGATTTAACTGCATCATTGATGGTTGCATTAGATAGTAATAATA
CTATGCCATTTACTCCAGCAGCTATGAGATCTGAGACATTGGGTTTTTATCCATGGAAACCAACCATA
CCAACTCCATGGAGATATTATTTTCAATGGGATAGAACATTAATACCATCTCATACTGGAACTAGTGG
CACACCAACAAATATATACCATGGTACAGATCCAGATGATGTTCAATTTTACACTATTGAAAATTCTG
TGCCAGTACACTTACTAAGAACAGGTGATGAATTTGCTACAGGAACATTTTATTTTGATTGTAAACCA
TGTAGACTAACACATACATGGCAAACAAATAGAGCATTGGGCTTACCACCATTTCTAAATTCTTTGCC
TCAAGCTGAAGGAGGTACTAACTTTGGTTATATAGGAGTTCAACAAGATAAAAGACGTGGTGTAACTC
AAATGGGAAATACAAACTATATTACTGAAGCTACTATTATGAGACCAGCTGAGGTTGGTTATAGTGCA
CCATATTATTCTTTTGAGGCGTCTACACAAGGGCCATTTAAAACACCTATTGCAGCAGGACGGGGGGG
AGCGCAAACAGATGAAAATCAAGCAGCAGATGGTGATCCAAGATATGCATTTGGTAGACAACATGGTC
AAAAAACTACCACAACAGGAGAAACACCTGAGAGATTTACATATATAGCACATCAAGATACAGGAAGA
TATCCAGAAGGAGATTGGATTCAAAATATTAACTTTAACCTTCCTGTAACAGATGATAATGTATTGCT
ACCAACAGATCCAATTGGAGGTAAAACAGGAATTAACTATACTAATATATTTAATACTTATGGTCCTT
TAACTGCATTAAATAATGTACCACCAGTTTATCCAAATGGTCAAATTTGGGATAAAGAATTTGATACT
GACTTAAAACCAAGACTTCATGTAAATGCACCATTTGTTTGTCAAAATAATTGTCCTGGTCAATTATT
TGTAAAAGTTGCGCCTAATTTAACAAATGAATATGATCCTGATGCATCTGCTAATATGTCAAGAATTG
TAACTTACTCAGATTTTTGGTGGAAAGGTAAATTAGTATTTAAAGCTAAACTAAGAGCCTCTCATACT

TGGAATCCAATTCAACAAATGAGTATCAATGTAGATAACCAATTTAACTATGTACCAAGTAATATTGG
AGGTATGAAAATTGTATATGAAAAATCTCAACTAGCACCTAGA
'VIE_AB120723_2b_HCM_23_2003'
ATGAGTGATGGAGCAGTTCAACCAGACGGTGGTCAACCTGCTGTCAGAAATGAAAGAGCTACAGGATC
TGGGAACGGGTCTGGAGGCGGGGGTGGTGGTGGTTCTGGGGGTGTGGGGATTTCTACGGGTACTTTTA
ATAATCAGACAGAATTTAAATTTTTGGAAAACGGATGGGTGGAAATCACAGCAAACTCAAGCAGACTT
GTACATTTAAATATGCCAGAAAGTGAAAATTATAGAAGAGTGGTTGTAAATAATTTGGATAAAACTGC
AGTTAACGGAAACATGGCTTTAGATGATACTCATGCACAAATTGTAACACCTTGGTCATTGGTTGATG
CAAATGCTTGGGGAGTTTGGTTTAATCCAGGAGATTGGCAACTAATTGTTAATACTATGAGTGAGTTG
CATTTAGTTAGTTTTGAACAAGAAATTTTTAATGTTGTTTTAAAGACTGTTTCAGAATCTGCTACTCA
GCCACCAACTAAAGTTTATAATAATGATTTAACTGCATCATTGATGGTTGCATTAGATAGTAATAATA
CTATGCCATTTACTCCAGCAGCTATGAGATCTGAGACATTGGGTTTTTATCCATGGAAACCAACCATA
CCAACTCCATGGAGATATTATTTTCAATGGGATAGAACATTAATACCATCTCATACTGGAACTAGTGG
CACACCAACAAATATATACCATGGTACAGATCCAGATGATGTTCAATTTTATACTATTGAAAATTCTG
TGCCAGTACACTTACTAAGAACAGGTGATGAATTTGCTACAGGAACATTTTTTTTTGATTGTAAACCA
TGTAGACTAACACATACATGGCAAACAAATAGAGCATTGGGCTTACCACCATTTCTAAATTCTTTGCC
TCAAGCTGAAGGAGGTACTAACTTTGGTTATATAGGAGTTCAACAAGATAAAAGACGTGGTGTAACTC
AAATGGGAAATACAAACTATATTACTGAAGCTACTATTATGAGACCAGCTGAGGTTGGTTATAGTGCA
CCATATTATTCTTTTGAGGCGTCTACACAAGGGCCATTTAAAACACCTATTGCAGCAGGACGGGGGGG
AGCGCAAACAGATGAAAATCAAGCAGCAGATGGTGATCCAAGATATGCATTTGGTAGACAACATGGTC
AAAAAACTACCACAACAGGAGAAACACCTGAGAGATTTACATATATAGCACATCAAGATACAGGAAGA
TATCCAGAAGGAGATTGGATTCAAAATATTAACTTTAACCTTCCTGTAACAGATGATAATGTATTGCT
ACCAACAGATCCAATTGGAGGTAAAACAGGAATTAACTATACTAATATATTTAATACTTATGGTCCTT
TAACTGCATTAAATAATGTACCACCAGTTTATCCAAATGGTCAAATTTGGGATAAAGAATTTGATACT
GACTTAAAACCAAGACTTCATGTAAATGCACCATTTGTTTGTCAAAATAATTGTCCTGGTCAATTATT
TGTAAAAGTTGCGCCTAATTTAACAAATGAATATGATCCTGATGCATCTGCTAATATGTCAAGAATTG
TAACTTACTCAGATTTTTGGTGGAAAGGTAAATTAGTATTTAAAGCTAAACTAAGAGCCTCTCATACT
TGGAATCCAATTCAACAAATGAGTATCAATGTAGATAACCAATTTAACTATGTACCAAGTAATATTGG
AGGTATGAAAATTGTATATGAAAAATCTCAACTAGCACCTAGA
'VIE_AB120724_2b_HNI_2_13_2003'
ATGAGTGATGGAGCAGTTCAACCAGACGGTGGTCAACCTGCTGTCAGAAATGAAAGAGCTACAGGATC
TGGGAACGGGTCTGGAGGCGGGGGTGGTGGTGGTTCTGGGGGTGTGGGGATTTCTACGGGTACTTTCA
ATAATCAGACAGAATTTAAATTTTTGGAAAACGGATGGGTGGAAATCACAGCAAACTCAAGCAGACTT
GTACATTTAAATATGCCAGAAAGTGAAAATTATAGAAGAGTGGTTGTAAATAATTTGGATAAAACTGC
AGTTAACGGAAACATGGCTTTAGATGATACTCATGCACAAATTGTAACACCTTGGTCATTGGTTGATG
CAAATGCTTGGGGAGTTTGGTTTAATCCAGGAGATTGGCAACTAATTGTTAATACTATGAGTGAGTTG
CATTTAGTTAGTTTTGAACAAGAAATTTTTAATGTTGTTTTAAAGACTGTTTCAGAATCTGCTACTCA
GCCACCAACTAAAGTTTATAATAATGATTTAACTGCATCATTGATGGTTGCATTAGATAGTAATAATA
CTATGCCATTTACTCCAGCAGCTATGAGATCTGAGACATTGGGTTTTTATCCATGGAAACCAACCATA
CCAACTCCATGGAGATATTATTTTCAATGGGATAGAACATTAATACCATCTCATACTGGAACTAGTGG
CACACCAACAAATATATACCATGGTACAGATCCAGATGATGTTCAATTTTACACTATTGAAAATTCTG
TGCCAGTACACTTACTAAGAACAGGTGATGAATTTGCTACAGGAACATTTTATTTTGATTGTAAACCA
TGTAGACTAACACATACATGGCAAACAAATAGAGCATTGGGCTTACCACCATTTCTAAATTCTTTGCC
TCAAGCTGAAGGAGGTACTAACTTTGGTTATATAGGAGTTCAACAAGATAAAAGACGTGGTGTAACTC
AAATGGGAAATACAAACTATATTACTGAAGCTACTATTATGAGACCAGCTGAGGTTGGTTATAGTGCA
CCATATTATTCTTTTGAGGCGTCTACACAAGGGCCATTTAAAACACCTATTGCAGCAGGACGGGGGGG
AGCGCAAACAGATGAAAATCAAGCAGCAGATGGTGATCCAAGATATGCATTTGGTAGACAACATGGTC
AAAAAACTACCACAACAGGAGAAACACCTGAGAGATTTACATATATAGCACATCAAGATACAGGAAGA
TATCCAGAAGGAGATTGGATTCAAAATATTAACTTTAACCTTCCTGTAACAGATGATAATGTATTGCT
ACCAACAGATCCAATTGGAGGTAAAACAGGAATTAACTATACTAATATATTTAATACTTATGGTCCTT
TAACTGCATTAAATAATGTACCACCAGTTTATCCAAATGGTCAAATTTGGGATAAAGAATTTGATACT
GACTTAAAACCAAGACTTCATGTAAATGCACCATTTGTTTGTCAAAATAATTGTCCTGGTCAATTATT
TGTAAAAGTTGCGCCTAATTTAACAAATGAATATGATCCTGATGCATCTGCTAATATGTCAAGAATTG
TAACTTACTCAGATTTTTGGTGGAAAGGTAAATTAGTATTTAAAGCTAAACTAAGAGCCTCTCATACT

TGGAATCCAATTCAACAAATGAGTATCAATGTAGATAACCAATTTAACTATGTACCAAGTAATATTGG
AGGTATGAAAATTGTATATGAAAAATCTCAACTAGCACCTAGA
'VIE_AB120725_2b_HNI_3_4_2003'
ATGAGTGATGGAGCAGTTCAACCAGACGGTGGTCAACCTGCTGTCAGAAATGAAAGAGCTACAGGATC
TGGGAACGGGTCTGGAGGCGGGGGTGGTGGTGGTTCTGGGGGTGTGGGGATTTCTACGGGTACTTTCA
ATAATCAGACAGAATTTAAATTTTTGGAAAACGGATGGGTGGAAATCACAGCAAACTCAAGCAGACTT
GTACATTTAAATATGCCAGAAAGTGAAAATTATAGAAGAGTGGTTGTAAATAATTTGGATAAAACTGC
AGTTAACGGAAACATGGCTTTAGATGATACTCATGCACAAATTGTAACACCTTGGTCATTGGTTGATG
CAAATGCTTGGGGAGTTTGGTTTAATCCAGGAGATTGGCAACTAATTGTTAATACTATGAGTGAGTTG
CATTTAGTTAGTTTTGAACAAGAAATTTTTAATGTTGTTTTAAAGACTGTTTCAGAATCTGCTACTCA
GCCACCAACTAAAGTTTATAATAATGATTTAACTGCATCATTGATGGTTGCATTAGATAGTAATAATA
CTATGCCATTTACTCCAGCAGCTATGAGATCTGAGACATTGGGTTTTTATCCATGGAAACCAACCATA
CCAACTCCATGGAGATATTATTTTCAATGGGATAGAACATTAATACCATCTCATACTGGAACTAGTGG
CACACCAACAAATATATACCATGGTACAGATCCAGATGATGTTCAATTTTATACTATTGAAAATTCTG
TGCCAGTACACTTACTAAGAACAGGTGATGAATTTGCTACAGGAACATTTTTTTTTGATTGTAAACCA
TGTAGACTAACACATACATGGCAAACAAATAGAGCATTGGGCTTACCACCATTTCTAAATTCTTTGCC
TCAAGCTGAAGGAGGTACTAACTTTGGTTATATAGGAGTTCAACAAGATAAAAGACGTGGTGTAACTC
AAATGGGAAATACAAACTATATTACTGAAGCTACTATTATGAGACCAGCTGAGGTTGGTTATAGTGCA
CCATATTATTCTTTTGAGGCGTCTACACAAGGGCCATTTAAAACACCTATTGCAGCAGGACGGGGGGG
AGCGCAAACAGATGAAAATCAAGCAGCAGATGGTGATCCAAGATATGCATTTGGTAGACAACATGGTC
AAAAAACTACCACAACAGGAGAAACACCTGAGAGATTTACATATATAGCACATCAAGATACAGGAAGA
TATCCAGAAGGAGATTGGATTCAAAATATTAACTTTAACCTTCCTGTAACAGATGATAATGTATTGCT
ACCAACAGATCCAATTGGAGGTAAAACAGGAATTAACTATACTAATATATTTAATACTTATGGTCCTT
TAACTGCATTAAATAATGTACCACCAGTTTATCCAAATGGTCAAATTTGGGATAAAGAATTTGATACT
GACTTAAAACCAAGACTTCATGTAAATGCACCATTTGTTTGTCAAAATAATTGTCCTGGTCAATTATT
TGTAAAAGTTGCGCCTAATTTAACAAATGAATATGATCCTGATGCATCTGCTAATATGTCAAGAATTG
TAACTTACTCAGATTTTTGGTGGAAAGGTAAATTAGTATTTAAAGCTAAACTAAGAGCCTCTCATACT
TGGAATCCAATTCAACAAATGAGTATCAATGTAGATAACCAATTTAACTATGTACCAAGTAATATTGG
AGGTATGAAAATTGTATATGAAAAATCTCAACTAGCACCTAGA
'JPN_AB437433_1887_M_2_2008'
ATGAGTGATGGAGCAGTTCAACCAGACGGTGGTCAGCCTGCTGTCAGAAATGAAAGAGCTACAGGATC
TGGGAACGGGTCTGGAGGCGGGGGTGGTGGTGGTTCTGGGGGTGTGGGGATTTCTACGGGTACTTTCA
ATAATCAGACGGAATTTAAATTTTTGGAAAACGGATGGGTGGAAATCACAGCAAACTCAAGCAGACTT
GTACATTTAAATATGCCAGAAAGTGAAAATTATAGAAGAGTGGTTGTAAATAATTTGGATAAAACTGC
AGTTAACGGAAACATGGCTTTAGATGATACCCATGCACAAATTGTAACACCTTGGTCATTGGTTGATG
CAAATGCTTGGGGAGTTTGGTTTAATCCAGGAGATTGGCAACTAATTGTTAATACTATGAGTGAGTTG
CATTTAGTTAGTTTTGAACAAGAAATTTTTAATGTTGTTTTAAAGACTGTTTCAGAATCTGCTACTCA
GCCACCAACTAAAGTTTATAATAATGATTTAACTGCATCATTGATGGTTGCATTAGATAGTAATAATA
CTATGCCATTTACTCCAGCAGCTATGAGATCTGAGACATTGGGTTTTTATCCATGGAAACCAACCATA
CCAACTCCATGGAGATATTATTTTCAATGGGATAGAACATTAATACCATCTCATACTGGAACTAGTGG
CACACCAACAAATATATACCATGGTACAGATCCAGATGATGTTCAATTTTATACTATTGAAAATTCTG
TGCCAGTACACTTACTAAGAACAGGTGATGAATTTGCTACAGGAACATTTTTTTTTGATTGTAAACCA
TGTAGACTAACACATACATGGCAAACAAATAGAGCATTAGGCTTACCACCATTTCTAAATTCTTTGCC
TCAAGCTGAAGGAGGTACTAACTTTGGTTATATAGGAGTTCAACAAGATAAAAGACGTGGTGTAACTC
AAATGGGAAATACAAACTATATTACTGAAGCTACTATTATGAGACCAGCTGAGGTTGGTTATAGTGCA
CCATATTATTCTTTTGAGGCGTCTACACAAGGGCCATTTAAAACACCTATTGCAGCAGGACGGGGGGG
AGCGCAAACAGATGAAAATCAAGCAGCAGATGGTGATCCAAGATATGCATTTGGTAGACAACATGGTC
AAAAAACTACCACAACAGGAGAAACACCTGAGAGATTTACATATATAGCACATCAAGATACAGGAAGA
TATCCAGAAGGAGATTGGATTCAAAATATTAACTTTAACCTTCCTGTAACAGATGATAATGTATTGCT
ACCAACAGATCCAATTGGAGGTAAAACAGGAATTAACTATACTAATATATTTAATACTTATGGTCCTT
TAACTGCATTAAATAATGTACCACCAGTTTATCCAAATGGTCAAATTTGGGATAAAGAATTTGATACT
GACTTAAAACCAAGACTTCATGTAAATGCACCATTTGTTTGTCAAAATAATTGTCCTGGTCAATTATT
TGTAAAAGTTGCACCTAATTTAACAAATGAATATGATCCTGATGCATCTGCTAATATGTCAAGAATTG
TAACTTACTCAGATTTTTGGTGGAAAGGTAAATTAGTATTTAAAGCTAAACTAAGAGCCTCTCATACT

TGGAATCCAATTCAACAAATGAGTATTAATGTAGATAACCAATTTAACTATGTACCAAGTAATATTGG
AGGTATGAAAATTGTATATGAAAAATCTCAACTAGCACCTAGA
'JPN_AB437434_1887_f_3_2008'
ATGAGTGATGGAGCAGTTCAACCAGACGGTGGTCAACCTGCTGTCAGAAATGAAAGAGCTACAGGATC
TGGGAACGGGTCTGGAGGCGGGGGTGGTGGTGGTTCTGGGGGTGTGGGGATTTCTACGGGTACTTTCA
ATAATCAGACGGAATTTAAATTTTTGGAAAACGGATGGGTGGAAATCACAGCAAACTCAAGCAGACTT
GTACATTTAAATATGCCAGAAAGTGAAAATTATAGAAGAGTGGTTGTGAATAATATGGATAAAACTGC
AGTTAACGGAAACATGGCTTTAGATGATATTCATGCACAAATTGTAACACCTTGGTCATTGGTTGATG
CAAATGCTTGGGGAGTTTGGTTTAATCCAGGAGATTGGCAACTAATTGTTAATACTATGAGTGAGTTG
CATTTAGTTAGTTTTGAACAAGAAATTTTTAATGTTGTTTTAAAGACTGTTTCAGAATCTGCTACTCA
GCCACCAACTAAAGTTTATAATAATGATTTAACTGCATCATTGATGGTTGCATTAGATAGTAATAATA
CTATGCCATTTACTCCAGCAGCTATGAGATCTGAGACATTGGGTTTTTATCCATGGAAACCAACCATA
CCAACTCCATGGAGATATTATTTTCAATGGGATAGAACATTAGTACCATCTCATACTGGAACTAGTGG
CACACCAACAAATATATACCATGGTACAGATCCAGATGATGTTCAATTTTATACTATTGAAAATTCTG
TGCCAGTACACTTACTAAGAACAGGTGATGAATTTGCTACAGGAACATTTTTTTTTGATTGTAAACCA
TGTAGACTAACACATACATGGCAAACAAATAGAGCATTGGGCTTACCACCATTTCTAAATTCTTTGCC
TCAATCTGAAGGAGCTACTAACTTTGGTGATATAGGAGTTCAACAAGATAAAAGACGTGGTGTAACTC
AAATGGGAAATACAAACTATATTACTGAAGCTACTATTATGAGACCAGCTGAGGTTGGTTATAGTGCA
CCATATTATTCTTTTGAGGCGTCTACACAAGGGCCATTTAAAACACCTATTGCAGCAGGACGGGGGGG
AGCGCAAACAGATGAAAATCAAGCAGCAGATGGTGATCCAAGATATGCATTTGGTAGACAACATGGTC
AAAAAACTACCACAACAGGAGAAACACCTGAGAGATTTACATATATAGCACATCAAGATACAGGAAGA
TATCCAGAAGGAGATTGGATTCAAAATATTAACTTTAACCTTCCTGTAACAGATGATAATGTATTGCT
ACCAACAGATCCAATTGGAGGTAAAACAGGAATTAACTATACTAATATATTTAATACTTATGGTCCTT
TAACTGCATTAAATAATGTACCACCAGTTTATCCAAATGGTCAAATTTGGGATAAAGAATTTGATACT
GACTTAAAACCAAGACTTCATGTAAATGCACCATTTGTTTGTCAAAATAATTGTCCTGGTCAATTATT
TGTAAAAGTTGCACCTAATTTAACAAATGAATATGATCCTGATGCATCTGCTAATATGTCAAGAATTG
TAACTTACTCAGATTTTTGGTGGAAAGGTAAATTAGTATTTAAAGCTAAACTAAGAGCCTCTCATACT
TGGAATCCAATTCAACAAATGAGTATTAATGTAGATAACCAATTTAACTATGTACCAAGTAATATTGG
AGGTATGAAAATTGTATATGAAAAATCTCAACTAGCACCTAGA
'ITA_AF306446_584_2000'
ATGAGTGATGGAGCAGTTCAACCAGACGGTGGTCAACCTGCTGTCAGAAATGAAAGAGCTACAGGATC
TGGGAATGGGTCTGGAGGCGGGGGTGGTGGTGGTTCTGGGGGTGTGGGGATTTCTACGGGTACTTTCA
ATAATCAGACGGAATTTAAATTTTTGGAAAACGGATGGGTGGAAATCACAGCAAACTCAAGCAGACTT
GTACATTTAAATATGCCAGAAAGTGAAAATTATAGAAGAGTGGTTGTAAATAATTTGGATAAAACTGC
AGTTAACGGAAACATGGCTTTAGATGATACTCATGCACAAATTGTAACACCTTGGTCATTGGTTGATG
CAAATGCTTGGGGAGTTTGGTTTAATCCAGGAGATTGGCAACTAATTGTTAATACTATGAGTGAGTTG
CATTTAGTTAGTTTTGAACAAGAAATTTTTAATGTTGTTTTAAAGACTGTTTCAGAATCTGCTACTCA
GCCACCAACTAAAGTTTATAATAATGATTTAACTGCATCATTGATGGTTGCATTAGATAGCAATAATA
CTATGCCATTTACTCCAGCAGCTATGAGATCTGAGACATTGGGTTTTTATCCATGGAAACCAACCATA
CCAACTCCATGGAGATATTATTTTCAATGGGATAGAACATTAATACCATCTCATACTGGAACTAGTGG
CACACCAACAAATATATACCATGGTACAGATCCAGATGATGTTCAATTTTATACTATTGAAAATTCTG
TGCCAGTACACTTACTAAGAACAGGTGATGAATTTGCTACAGGAACATTTTTTTTTGATTGTAAACCA
TGTAGACTAACACATACATGGCAAACAAATAGAGCATTGGGCTTACCACCATTTCTAAATTCTTTGCC
TCAAGCTGAAGGAGGTACTAACTTTGGTTATATAGGAGTTCAACAAGATAAAAGACGTGGTGTAACTC
AAATGGGAAATACAAACTATATTACTGAAGCTACTATTATGAGACCAGCTGAGGTTGGTTATAGTGCA
CCATATTATTCTTTTGAGGCGTCTACACAAGGGCCATTTAAAACACCTATTGCAGCAGGACGGGGGGG
AGCGCAAACAGATGAAAATCAAGCAGCAGATGGTGATCCAAGATATGCATTTGGTAGACAACATGGTC
AAAAAACTACCACAACAGGAGAAACACCTGAGAGATTTACATATATAGCACATCAAGATACAGGAAGA
TATCCAGAAGGAGATTGGATTCAAAATATTAACTTTAACCTTCCTGTAACAAATGATAATGTATTGCT
ACCAACAGATCCAATTGGAGGTAAAACAGGAATTAACTATACTAATATATTTAATACTTATGGTCCTT
TAACTGCATTAAATAATGTACCACCAGTTTATCCAAATGGTCAAATTTGGGATAAAGAATTTGATACT
GACTTAAAACCAAGACTTCATGTAAATGCACCATTTGTTTGTCAAAATAATTGTCCTGGTCAATTATT
TGTAAAAGTTGCGCCTAATTTAACAAATGAATATGATCCTGATGCATCTGCTAATATGTCAAGAATTG
TAACTTACTCAGATTTTTGGTGGAAAGGTAAATTAGTATTTAAAGCTAAACTAAGAGCCTCTCATACT

TGGAATCCAATTCAACAAATGAGTATTAATGTAGATAACCAATTTAACTATGTACCAAGTAATATTGG
AGGTATGAAGATTGTATATGAAAAATCTCAACTAGCACCTAGA
'ITA_AF306447_618_2000'
ATGAGTGATGGAGCAGTTCAACCAGACGGTGGTCAACCTGCTGTCAGAAATGAAAGAGCTACAGGATC
TGGGAACGGGTCTGGAGGCGGGGGTGGTGGTGGTTCTGGGGGTGTGGGGATTTCTACGGGTACTTTCA
ATAATCAGACGGAATTTAAATTTTTGGAAAACGGATGGGTGGAAATCACAGCAAACTCAAGCAGACTT
GTACATTTAAATATGCCAGAAAGTGAAAATTATAGAAGAGTGGTTGTAAATAATTTGGATAAAACTGC
AGTTAACGGAAACATGGCTTTAGATGATACTCATGCACAAATTGTAACACCTTGGTCATTGGTTGATG
CAAATGCTTGGGGAGTTTGGTTTAATCCAGGAGATTGGCAACTAATTGTTAATACTATGAGTGAGTTG
CATTTAGTTAGTTTTGAACAAGAAATTTTTAATGTTGTTTTAAAGACTGTTTCAGAATCTGCTACTCA
GCCACCAACTAAAGTTTATAATAATGATTTAACTGCATCATTGATGGTTGCATTAGATAGCAATAATA
CTATGCCATTTACTCCAGCAGCTATGAGATCTGAGACATTGGGTTTTTATCCATGGAAACCAACCATA
CCAACTCCATGGAGATATTATTTTCAATGGGATAGAACATTAATACCATCTCATACTGGAACTAGTGG
CACACCAACAAATATATACCATGGTACAGATCCAGATGATGTTCAATTTTATACTATTGAAAATTCTG
TGCCAGTACACTTACTAAGAACAGGTGATGAATTTGCTACAGGAACATTTTTTTTTGATTGTAAACCA
TGTAGACTAACACATACATGGCAAACAAATAGAGCATTGGGCTTACCACCATTTCTAAATTCTTTGCC
TCAAGCTGAAGGAGGTACTAACTTTGGTTATATAGGAGTTCAACAAGATAAAAGACGTGGTGTAACTC
AAATGGGAAATACAAACTATATTACTGAAGCTACTATTATGAGACCAGCTGAGGTTGGTTATAGTGCA
CCATATTATTCTTTTGAGGCGTCTACACAAGGGCCATTTAAAACACCTATTGCAGCAGGACGGGGGGG
AGCGCAAACAGATGAAAATCAAGCAGCAGATGGTGATCCAAGATATGCATTTGGTAGACAACATGGTC
AAAAAACTACCACAACAGGAGAAACACCTGAGAGATTTACATATATAGCACATCAAGATACAGGAAGA
TATCCAGAAGGAGATTGGATTCAAAATATTAACTTTAACCTTCCTGTAACAAATGATAATGTATTGCT
ACCAACAGATCCAATTGGAGGTAAAACAGGAATTAACTATACTAATATATTTAATACTTATGGTCCTT
TAACTGCATTAAATAATGTACCACCAGTTTATCCAAATGGTCAAATTTGGGATAAAGAATTTGATACT
GACTTAAAACCAAGACTTCATGTAAATGCACCATTTGTTTGTCAAAATAATTGTCCTGGTCAATTATT
TGTAAAAGTTGCGCCTAATTTAACAAATGAATATGATCCTGATGCATCTGCTAATATGTCAAGAATTG
TAACTTACTCAGATTTTTGGTGGAAAGGTAAATTAGTATTTAAAGCTAAACTAAGAGCCTCTCATACT
TGGAATCCAATTCAACAAATGAGTATTAATGTAGATAACCAATTTAACTATGTACCAAGTAATATTGG
AGGTATGAAGATTGTATATGAAAAATCTCAACTAGCACCTAGA
'ITA_AF393506_2a_699_2000'
ATGAGTGATGGAGCAGTTCAACCAGACGGTGGTCAACCTGCTGTCAGAAATGAAAGAGCTACAGGATC
TGGGAACGGGTCTGGAGGCGGGGGTGGTGGTGGTTCTGGGGGTGTGGGGATTTCTACGGGTACTTTCA
ATAATCAGACGGAATTTAAATTTTTGGAAAACGGATGGGTGGAAATCACAGCAAACTCAAGCAGACTT
GTACATTTAAATATGCCAGAAAGTGAAAATTATAGAAGAGTGGTTGTAAATAATTTGGATAAAACTGC
AGTTAACGGAAACATGGCTTTAGATGATACTCATGCACAAATTGTAACACCTTGGTCATTGGTTGATG
CAAATGCTTGGGGAGTTTGGTTTAATCCAGGAGATTGGCAACTAATTGTTAATACTATGAGTGAGTTG
CATTTAGTTAGTTTTGAACAAGAAATTTTTAATGTTGTTTTAAAGACTGTTTCAGAATCTGCTACTCA
GCCACCAACTAAAGTTTATAATAATGATTTAACTGCATCATTGATGGTTGCATTAGATAGCAATAATA
CTATGCCATTTACTCCAGCAGCTATGAGATCTGAGACATTGGGTTTTTATCCATGGAAACCAACCATA
CCAACTCCATGGAGATATTATTTTCAATGGGATAGAACATTAATACCATCTCATACTGGAACTAGTGG
CACACCAACAAATATATACCATGGTACAGATCCAGATGATGTTCAATTTTATACTATTGAAAATTCTG
TGCCAGTACACTTACTAAGAACAGGTGATGAATTTGCTACAGGAACATTTTTTTTTGATTGTAAACCA
TGTAGACTAACACATACATGGCAAACAAATAGAGCATTGGGCTTACCACCATTTCTAAATTCTTTGCC
TCAAGCTGAAGGAGGTACTAACTTTGGTTATATAGGAGTTCAACAAGATAAAAGACGTGGTGTAACTC
AAATGGGAAATACAAACTATATTACTGAAGCTACTATTATGAGACCAGCTGAGGTTGGTTATAGTGCA
CCATATTATTCTTTTGAGGCGTCTACACAAGGGCCATTTAAAACACCTATTGCAGCAGGACGGGGGGG
AGCGCAAACAGATGAAAATCAAGCAGCAGATGGTGATCCAAGATATGCATTTGGTAGACAACATGGTC
AAAAAACTACCACAACAGGAGAAACACCTGAGAGATTTACATATATAGCACATCAAGATACAGGAAGA
TATCCAGAAGGAGATTGGATTCAAAATATTAACTTTAACCTTCCTGTAACAAATGATAATGTATTGCT
ACCAACAGATCCAATTGGAGGTAAAGCAGGAATTAACTATACTAATATATTTAATACTTATGGTCCTT
TAACTGCATTAAATAATGTACCACCAGTTTATCCAAATGGTCAAATTTGGGATAAAGAATTTGATACT
GACTTAAAACCAAGACTTCATGTAAATGCACCATTTGTTTGTCAAAATAATTGTCCTGGTCAATTATT
TGTAAAAGTTGCGCCTAATTTAACAAATGAATATGATCCTGATGCATCTGCTAATATGTCAAGAATTG
TAACTTACTCAGATTTTTGGTGGAAAGGTAAATTAGTATTTAAAGCTAAACTAAGAGCCTCTCATACT

TGGAATCCAATTCAACAAATGAGTATTAATGTAGATAACCAATTTAACTATGTACCAAGTAATATTGG
AGGTATGAAGATTGTATATGAAAAATCTCAACTAGCACCTAGA

'USA_AY742932_193_1991'

ATGAGTGATGGAGCAGTTCAACCAGACGGTGGTCAACCTGCTGTCAGAAATGAAAGAGCTACAGGATC
TGGGAACGGGTCTGGAGGCGGGGGTGGTGGTGGTTCTGGGGGTGTGGGGATTTCTACGGGTACTTTCA
ATAATCAGACGGAATTTAAATTTTTGGAAAACGGATGGGTGGAAATCACAGCAAACTCAAGCAGACTT
GTACATTTAAATATGCCAGAAAGTGAAAATTATAGAAGAGTGGTTGTAAATAATTTGGATAAAACTGC
AGTTAACGGAAACATGGCTTTAGATGATACTCATGCACAAATTGTAACACCTTGGTCATTGGTTGATG
CAAATGCTTGGGGAGTTTGGTTTAATCCAGGAGATTGGCAACTAATTGTTAATACTATGAGTGAGTTG
CATTTAGTTAGTTTTGAACAAGAAATTTTTAATGTTGTTTTAAAGACTGTTTCAGAATCTGCTACTCA
GCCACCAACTAAAGTTTATAATAATGATTTAACTGCATCATTGATGGTTGCATTAGATAGTAATAATA
CTATGCCATTTACTCCAGCAGCTATGAGATCTGAGACATTGGGTTTTTATCCATGGAAACCAACCATA
CCAACTCCATGGAGATATTATTTTCAATGGGATAGAACATTAATACCATCTCATACTGGAACTAGTGG
CACACCAACAAATATATACCATGGTACAGATCCAGATGATGTTCAATTTTATACTATTGAAAATTCTG
TGCCAGTACACTTACTAAGAACAGGTGATGAATTTGCTACAGGAACATTTTTTTTTGATTGTAAACCA
TGTAGACTAACACATACATGGCAAACAAATAGAGCATTGGGCTTACCACCATTTCTAAATTCTTTGCC
TCAAGCTGAAGGAGGTACTAACTTTGGTTATATAGGAGTTCAACAAGATAAAAGACGTGGTGTAACTC
AAATGGGAAATACAAACTATATTACTGAAGCTACTATTATGAGACCAGCTGAGGTTGGTTATAGTGCA
CCATATTATTCTTTTGAGGCGTCTACACAAGGGCCATTTAAAACACCTATTGCAGCAGGACGGGGGGG
AGCGCAAACAGATGAAAATCAAGCAGCAGATGGTGATCCAAGATATGCATTTGGTAGACAACATGGTC
AAAAAACTACCACAACAGGAGAAACACCTGAGAGATTTACATATATAGCACATCAAGATACAGGAAGA
TATCCAGAAGGAGATTGGATTCAAAATATTAACTTTAACCTTCCTGTAACAGATGATAATGTATTGCT
ACCAACAGATCCAATTGGAGGTAAAACAGGAATTAACTATACTAATATATTTAATACTTATGGTCCTT
TAACTGCATTAAATAATGTACCACCAGTTTATCCAAATGGTCAAATTTGGGATAAAGAATTTGATACT
GACTTAAAACCAAGACTTCATGTAAATGCACCATTTGTTTGTCAAAATAATTGTCCTGGTCAATTATT
TGTAAAAGTTGCGCCTAATTTAACAAATGAATATGATCCTGATGCATCTGCTAATATGTCAAGAATTG
TAACTTACTCAGATTTTTGGTGGAAAGGTAAATTAGTATTTAAAGCTAAACTAAGAGCCTCTCATACT
TGGAATCCAATTCAACAAATGAGTATTAATGTAGATAACCAATTTAACTATGTACCAAGTAATATTGG
AGGTATGAAAATTGTATATGAAAAATCTCAACTAGCACCTAGA

'NZE_AY742933_339_1993'

ATGAGTGATGGAGCAGTTCAACCAGACGGTGGTCAGCCTGCTGTCAGAAATGAAAGAGCTACAGGATC
TGGGAACGGGTCTGGAGGCGGGGGTGGTGGTGGTTCTGGGGGTGTGGGGATTTCTACGGGTACTTTCA
ATAATCAGACGGAATTTAAATTTTTGGAAAACGGATGGGTGGAAATCACAGCAAACTCAAGCAGACTT
GTACATTTAAATATGCCAGAAAGTGAAAATTATAGAAGAGTGGTTGTAAATAATTTGGATAAAACTGC
AGTTAACGGAAACATGGCTTTAGATGATACTCATGCACAAATTGTAACACCTTGGTCATTGGTTGATG
CAAATGCTTGGGGAGTTTGGTTTAATCCAGGAGATTGGCAACTAATTGTTAATACTATGAGTGAGTTA
CATTTAGTTAGTTTTGAACAAGAAATTTTTAATGTTGTTTTAAAGACTGTTTCAGAATCTGCTACTCA
GCCACCAACTAAAGTCTATAATAATGATTTAACTGCATCATTGATGGTTGCATTAGATAGTAATAATA
CTATGCCATTTACTCCAGCAGCTATGAGATCTGAGACATTGGGTTTTTATCCATGGAAACCAACCATA
CCAACTCCATGGAGATATTATTTTCAATGGGATAGAACATTAATACCATCTCATACTGGAACTAGTGG
CACACCAACAAATATATACCATGGTACAGATCCAGATGATGTTCAATTTTATACTATTGAAAATTCTG
TGCCAGTACACTTACTAAGAACAGGTGATGAATTTGCTACAGGAACATTTTTTTTTGATTGTAAACCA
TGTAGACTAACACATACATGGCAAACAAATAGAGCATTGGGCTTACCACCATTTCTAAATTCTTTGCC
TCAAGCTGAAGGAGGTACTAACTTTGGTTATATAGGAGTTCAACAAGATAAAAGACGTGGTGTAACTC
AAATGGGAAATACAAACTATATTACTGAAGCTACTATTATGAGACCAGCTGAGGTTGGTTATAGTGCA
CCATATTATTCTTTTGAGGCGTCTACACAAGGGCCATTTAAAACACCTATTGCAGCAGGACGGGGGGG
AGCGCAAACAGATGAAAATCAAGCAGCAGATGGTGATCCAAGATATGCATTTGGTAGACAACATGGTC
AAAAAACTACCACAACAGGAGAAACACCTGAGAGATTTACATATATAGCACATCAAGATACAGGAAGA
TATCCAGAAGGAGATTGGATTCAAAATATTAACTTTAACCTTCCTGTAACAAATGATAATGTATTGCT
ACCAACAGATCCAATTGGAGGTAAAACAGGAATTAACTATACTAATATATTTAATACTTATGGTCCTT
TAACTGCATTAAATAATGTACCACCAGTTTATCCAAATGGTCAAATTTGGGATAAAGAATTTGATACT
GACTTAAAACCAAGACTTCATGTAAATGCACCATTTGTTTGTCAAAATAATTGTCCTGGTCAATTATT
TGTAAAAGTTGCGCCTAATTTAACAAATGAATATGATCCTGATGCATCTGCTAATATGTCAAGAATTG
TAACTTACTCAGATTTTTGGTGGAAAGGTAAATTAGTATTTAAAGCTAAACTAAGAGCCTCTCATACT

TGGAATCCAATTCAACAAATGAGTATTAATGTAGATAACCAATTTAACTATGTACCAAGTAATATTGG
AGGTATGAAAATTGTATATGAAAAATCTCAACTAGCACCTAGA
'GER_AY742934_447_1995'
ATGAGTGATGGAGCAGTTCAACCAGACGGTGGTCAACCTGCTGTCAGAAATGAAAGAGCTACAGGATC
TGGGAACGGGTCTGGAGGCGGGGGTGGTGGTGGTTCTGGGGGTGTGGGGATTTCTACGGGTACTTTCA
ATAATCAGACGGAATTTAAATTTTTGGAAAACGGATGGGTGGAAATCACAGCAAACTCAAGCAGACTT
GTACATTTAAATATGCCAGAAAGTGAAAATTATAGAAGAGTGGTTGTAAATAATTTGGATAAAACTGC
AGTTAACGGAAACATGGCTTTAGATGATACTCATGCACAAATTGTAACACCTTGGTCATTGGTTGATG
CAAATGCTTGGGGAGTTTGGTTTAATCCAGGAGATTGGCAACTAATTGTTAATACTATGAGTGAGTTG
CATTTAGTTAGTTTTGAACAAGAAATTTTTAATGTTGTTTTAAAGACTGTTTCAGAATCTGCTACTCA
GCCACCAACTAAAGTTTATAATAATGATTTAACTGCATCATTGATGGTTGCATTAGATAGCAATAATA
CTATGCCATTTACTCCAGCAGCTATGAGATCTGAGACATTGGGTTTTTATCCATGGAAACCAACCATA
CCAACTCCATGGAGATATTATTTTCAATGGGATAGAACATTAATACCATCTCATACTGGAACTAGTGG
CACACCAACAAATATATACCATGGTACAGATCCAGATGATGTTCAATTTTATACTATTGAAAATTCTG
TGCCAGTACACTTACTAAGAACAGGTGATGAATTTGCTACAGGAACATTTTTTTTTGATTGTAAACCA
TGTAGACTAACACATACATGGCAAACAAATAGAGCATTGGGCTTACCACCATTTCTAAATTCTTTGCC
TCAAGCTGAAGGAGGTACTAACTTTGGTTATATAGGAGTTCAACAAGATAAAAGACGTGGTGTAACTC
AAATGGGAAATACAAACTATATTACTGAAGCTACTATTATGAGACCAGCTGAGGTTGGTTATAGTGCA
CCATATTATTCTTTTGAGGCGTCTACACAAGGGCCATTTAAAACACCTATTGCAGCAGGACGGGGGGG
AGCGCAAACAGATGAAAATCAAGCAGCAGATGGTGATCCAAGATATGCATTTGGTAGACAACATGGTC
AAAAAACTACCACAACAGGAGAAACACCTGAGAGATTTACATATATAGCACATCAAGATACAGGAAGA
TATCCAGAAGGAGATTGGATTCAAAATATTAACTTTAACCTTCCTGTAACAGATGATAATGTATTGCT
ACCAACAGATCCAATTGGAGGTAAAACAGGAATTAACTATACTAATATATTTAATACTTATGGTCCTT
TAACTGCATTAAATAATGTACCACCAGTTTATCCAAATGGTCAGATTTGGGATAAAGAATTTGATACT
GACTTAAAACCAAGACTTCATGTAAATGCACCATTTGTTTGTCAAAATAATTGTCCTGGTCAATTATT
TGTAAAAGTTGCGCCTAATTTAACAAATGAATATGATCCTGATGCATCTGCTAATATGTCAAGAATTG
TAACTTACTCAGATTTTTGGTGGAAAGGTAAATTAGTATTTAAAGCTAAACTAAGAGCCTCTCATACT
TGGAATCCAATTCAACAAATGAGTATTAATGTAGATAACCAATTTAACTATGTACCAAGTAATATTGG
AGGTATGAAAATTGTATATGAAAAATCTCAACTAGCACCTAGA
'GER_AY742935_U6_1995'
ATGAGTGATGGAGCAGTTCAACCAGACGGTGGTCAGCCTGCTGTCAGAAATGAAAGAGCTACAGGATC
TGGGAACGGGTCTGGAGGCGGGGGTGGTGGTGGTTCTGGGGGTGTGGGGATTTCTACGGGTACTTTCA
ATAATCAGACGGAATTTAAATTTTTGGAAAACGGATGGGTGGAAATCACAGCAAACTCAAGCAGACTT
GTACATTTAAATATGCCAGAAAGTGAAAATTATAGAAGAGTGGTTGTAAATAATTTGGATAAAACTGC
AGTTAACGGAAACATGGCTTTAGATGATACTCATGCACAAATTGTAACACCTTGGTCATTGGTTGATG
CAAATGCTTGGGGAGTTTGGTTTAATCCAGGAGATTGGCAACTAATTGTTAATACTATGAGTGAGTTG
CATTTAGTTAGTTTTGAACAAGAAATTTTTAATGTTGTTTTAAAGACTGTTTCAGAATCTGCTACTCA
GCCACCAACTAAAGTTTATAATAATGATTTAACTGCATCATTGATGGTTGCATTAGATAGCAATAATA
CTATGCCATTTACTCCAGCAGCTATGAGATCTGAGACATTGGGTTTTTATCCATGGAAACCAACCATA
CCAACTCCATGGAGATATTATTTTCAATGGGATAGAACATTAATACCATCTCATACTGGAACTAGTGG
CACACCAACAAATATATACCATGGTACAGATCCAGATGATGTTCAATTTTATACTATTGAAAATTCTG
TGCCAGTACACTTACTAAGAACAGGTGATGAATTTGCTACAGGAACATTTTTTTTTGATTGTAAACCA
TGTAGACTAACACATACATGGCAAACAAATAGAGCATTGGGCTTACCACCATTTCTAAATTCTTTGCC
TCAAGCTGAAGGAGGTACTAACTTTGGTTATATAGGAGTTCAACAAGATAAAAGACGTGGTGTAACTC
AAATGGGAAATACAAACTATATTACTGAAGCTACTATTATGAGACCAGCTGAGGTTGGTTATAGTGCA
CCATATTATTCTTTTGAGGCGTCTACACAAGGGCCATTTAAAACACCTATTGCAGCAGGACGGGGGGG
AGCGCAAACAGATGAAAATCAAGCAGCAGATGGTGATCCAAGATATGCATTTGGTAGACAACATGGTC
AAAAAACTACCACAACAGGAGAAACACCTGAGAGATTTACATATATAGCACATCAAGATACAGGAAGA
TATCCAGAAGGAGATTGGATTCAAAATATTAACTTTAACCTTCCTGTAACAAATGATAATGTATTGCT
ACCAACAGATCCAATTGGAGGTAAAACAGGAATTAACTATACTAATATATTTAATACTTATGGTCCTT
TAACTGCATTAAATAATGTACCACCAGTTTATCCAAATGGTCAAATTTGGGATAAAGAATTTGATACT
GACTTAAAACCAAGACTTCATGTAAATGCACCATTTGTTTGTCAAAATAATTGTCCTGGTCAATTATT
TGTAAAAGTTGCGCCTAATTTAACAAATGAATATGATCCTGATGCATCTGCTAATATGTCAAGAATTG
TAACTTACTCAGATTTTTGGTGGAAAGGTAAATTAGTATTTAAAGCTAAACTAAGAGCCTCTCATACT

TGGAATCCAATTCAACAAATGAGTATTAATGTAGATAACCAATTTAACTATGTACCAAGTAATATTGG
AGGTATGAAGATTGTATATGAAAAATCTCAACTAGCACCTAGA

'USA_AY742936_395_1998'

ATGAGTGATGGAGCAGTTCAACCAGACGGTGGTCAACCTGCTGTCAGAAATGAAAGAGCAACAGGATC
TGGGAACGGGTCTGGAGGCGGGGGTGGTGGTGGTTCTGGGGGTGTGGGGATTTCTACGGGTACTTTCA
ATAATCAGACGGAATTTAAATTTTTGGAAAACGGATGGGTGGAAATCACAGCAAACTCAAGCAGACTT
GTACATTTAAATATGCCAGAAAGTGAAAATTATAGAAGAGTGGTTGTAAATAATTTGGATAAAACTGC
AGTTAACGGAAACATGGCTTTAGATGATACTCATGCACAAATTGTAACACCTTGGTCATTGGTTGATG
CAAATGCTTGGGGAGTTTGGTTTAATCCAGGAGATTGGCAACTAATTGTTAATACTATGAGTGAGTTG
CATTTAGTTAGTTTTGAACAAGAAATTTTTAATGTTGTTTTAAAGACTGTTTCAGAATCTGCTACTCA
GCCACCAACTAAAGTTTATAATAATGATTTAACTGCATCATTGATGGTTGCATTAGATAGCAATAATA
CTATGCCATTTACTCCAGCAGCTATGAGATCTGAGACATTGGGTTTTTATCCATGGAAACCAACCATA
CCAACTCCATGGAGATATTATTTTCAATGGGATAGAACATTAATACCATCTCATACTGGAACTAGTGG
CACACCAACAAATATATACCATGGTACAGATCCAGATGATGTTCAATTTTATACTATTGAAAATTCTG
TGCCAGTACACTTACTAAGAACAGGTGATGAATTTGCTACAGGAACATTTTTTTTTGATTGTAAACCA
TGTAGACTAACACATACATGGCAAACAAATAGAGCATTGGGCTTACCACCATTTCTAAATTCTTTGCC
TCAAGCTGAAGGAGGTACTAACTTTGGTTATATAGGAGTTCAACAAGATAAAAGACGTGGTGTAACTC
AAATGGGAAATACAAACTATATTACTGAAGCTACTATTATGAGACCAGCTGAGGTTGGTTATAGTGCA
CCATATTATTCTTTTGAGGCGTCTACACAAGGGCCATTTAAAACACCTATTGCAGCAGGACGGGGGGG
AGCGCAAACAGATGAAAATCAAGCAGCAGATGGTGATCCAAGATATGCATTTGGTAGACAACATGGTC
AAAAAACTACCACAACAGGAGAAACACCTGAGAGATTTACATATATAGCACATCAAGATACAGGAAGA
TATCCAGAAGGAGATTGGATTCAAAATATTAACTTTAACCTTCCTGTAACAGATGATAATGTATTGCT
ACCAACAGATCCAATTGGAGGTAAAACAGGAATTAACTATACTAATATATTTAATACTTATGGTCCTT
TAACTGCATTAAATAATGTACCACCAGTTTATCCAAATGGTCAAATTTGGGATAAAGAATTTGATACT
GACTTAAAACCAAGACTTCATGTAAATGCACCATTTGTTTGTCAAAATAATTGTCCTGGTCAATTATT
TGTAAAAGTTGCGCCTAATTTAACAAATGAATATGATCCTGATGCATCTGCTAATATGTCAAGAATTG
TAACTTACTCAGATTTTTGGTGGAAAGGTAAATTAGTATTTAAAGCTAAACTAAGAGCCTCTCATACT
TGGAATCCAATTCAACAAATGAGTATTAATGTAGATAACCAATTTAACTATGTACCAAGTAATATTGG
AGGTATGAAAATTGTATATGAAAAATCTCAACTAGCACCTAGA

'USA_AY742951_431_2003'

ATGAGTGATGGAGCAGTTCAACCAGACGGTGGTCAACCTGCTGTCAGAAATGAAAGAGCTACAGGATC
TGGGAACGGGTCTGGAGGCGGGGGTGGTGGTGGTTCTGGGGGTGTGGGGATTTCTACGGGTACTTTCA
ATAATCAGACGGAATTTAAATTTTTGGAAAACGGATGGGTGGAAATCACAGCAAACTCAAGCAGACTT
GTACATTTAAATATGCCAGAAAGTGAAAATTATAGAAGAGTGGTTGTAAATAATTTGGATAAAACTGC
AGTTAACGGAAACATGGCTTTAGATGATACTCATGCACAAATTGTAACACCTTGGTCATTGGTTGATG
CAAATGCTTGGGGAGTTTGGTTTAATCCAGGAGATTGGCAACTAATTGTTAATACTATGAGTGAGTTG
CATTTAGTTAGTTTTGAACAAGAAATTTTTAATGTTGTTTTAAAGACTGTTTCAGAATCTGCTACTCA
GCCACCAACTAAAGTTTATAATAATGATTTAACTGCATCATTGATGGTTGCATTAGATAGTAATAATA
CTATGCCATTTACTCCAGCAGCTATGAGATCTGAGACATTGGGTTTTTATCCATGGAAACCAACCATA
CCAACTCCATGGAGATATTATTTTCAATGGGATAGAACATTAATACCATCTCATACTGGAACTAGTGG
CACACCAACAAATATATACCATGGTACAGATCCAGATGATGTTCAATTTTATACTATTGAAAATTCTG
TGCCAGTACACTTACTAAGAACAGGTGATGAATTTGCTACAGGAACATTTTTTTTTGATTGTAAACCA
TGTAGACTAACACATACATGGCAAACAAATAGAGCATTGGGCTTACCACCATTTCTAAATTCTTTGCC
TCAAGCTGAAGGAGGTACTAACTTTGGTTATATAGGAGTTCAACAAGATAAAAGACGTGGTGTAACTC
AAATGGGAAAAACAAACTATATTACTGAAGCTACTATTATGAGACCAGCTGAGGTTGGTTATAGTGCA
CCATATTATTCTTTTGAGGCGTCTACACAAGGGCCATTTAAAACACCTATTGCAGCAGGACGGGGGGG
AGCGCAAACAGATGAAAATCAAGCAGCAGATGGTGATCCAAGATATGCATTTGGTAGACAACATGGTC
AAAAAACTACCACAACAGGAGAAACACCTGAGAGATTTACATATATAGCACATCAAGATACAGGAAGA
TATCCAGAAGGAGATTGGATTCAAAATATTAACTTTAACCTTCCTGTAACAGATGATAATGTATTGCT
ACCAACAGATCCAATTGGAGGTAAAACAGGAATTAACTATACTAATATATTTAATACTTATGGTCCTT
TAACTGCATTAAATAATGTACCACCAGTTTATCCAAATGGTCAAATTTGGGATAAAGAATTTGATACT
GACTTAAAACCAAGACTTCATGTAAATGCACCATTTGTTTGTCAAAATAATTGTCCTGGTCAATTATT
TGTAAAAGTTGCGCCTAATTTAACAAATGAATATGATCCTGATGCATCTGCTAATATGTCAAGAATTG
TAACTTACTCAGATTTTTGGTGGAAAGGTAAATTAGTATTTAAAGCTAAACTAAGAGCCTCTCATACT

TGGAATCCAATTCAACAAATGAGTATTAATGTAGATAACCAATTTAACTATGTACCAAGTAATATTGG
AGGTATGAAAATTGTATATGAAAGATCTCAACTAGCACCTAGA

'USA_AY742953_435_2003'

ATGAGTGATGGAGCAGTTCAACCAGACGGTGGTCAACCTGCTGTCAGAAATGAAAGAGCTACAGGATC
TGGGAACGGGTCTGGAGGCGGGGGTGGTGGTGGTTCTGGGGGTGTGGGGATTTCTACGGGTACTTTCA
ATAATCAGACGGAATTTAAATTTTTGGAAAACGGATGGGTGGAAATCACAGCAAACTCAAGCAGACTT
GTACATTTAAATATGCCAGAAAGTGAAAATTATAGAAGAGTGGTTGTAAATAATTTGGATAAAACTGC
AGTTAACGGAAACATGGCTTTAGATGATACTCATGCACAAATTGTAACACCTTGGTCATTGGTTGATG
CAAATGCTTGGGGAGTTTGGTTTAATCCAGGAGATTGGCAACTAATTGTTAATACTATGAGTGAGTTG
CATTTAGTTAGTTTTGAACAAGAAATTTTTAATGTTGTTTTAAAGACTGTTTCAGAATCTGCTACTCA
GCCACCAACTAAAGTTTATAATAATGATTTAACTGCATCATTGATGGTTGCATTAGATAGTAATAATA
CTATGCCATTTACTCCAGCAGCTATGAGATCTGAGACATTGGGTTTTTATCCATGGAAACCAACCATA
CCAACTCCATGGAGATATTATTTTCAATGGGATAGAACATTAATACCATCTCATACTGGAACTAGTGG
CACACCAACAAATATATACCATGGTACAGATCCAGATGATGTTCAATTTTATACTATTGAAAATTCTG
TGCCAGTACACTTACTAAGAACAGGTGATGAATTTGCTACAGGAACATTTTTTTTTGATTGTAAACCA
TGTAGACTAACACATACATGGCAAACAAATAGAGCATTGGGCTTACCACCATTTCTAAATTCTTTGCC
TCAAGCTGAAGGAGGTACTAACTTTGGTTATATAGGAGTTCAACAAGATAAAAGACGTGGTGTAACTC
AAATGGGAAATACAAACTATATTACTGAAGCTACTATTATGAGACCAGCTGAGGTTGGTTATAGTGCA
CCATATTATTCTTTTGAGGCGTCTACACAAGGGCCATTTAAAACACCTATTGCAGCAGGACGGGGGGG
AGCGCAAACAGATGAAAATCAAGCAGCAGATGGTGATCCAAGATATGCATTTGGTAGACAACATGGTC
AAAAAACTACCACAACAGGAGAAACACCTGAGAGATTTACATATATAGCACATCAAGATACAGGAAGA
TATCCAGAAGGAGATTGGATTCAAAATATTAACTTTAACCTTCCTGTAACAAATGATAATGTATTGCT
ACCAACAGATCCAATTGGAGGTAAAACAGGAATTAACTATACTAATATATTTAATACTTATGGTCCTT
TAACTGCATTAAATAATGTGCCACCAGTTTATCCAAATGGTCAAATTTGGGATAAAGAATTTGATACT
GACTTAAAACCAAGACTTCATGTAAATGCACCATTTGTTTGTCAAAATAATTGTCCTGGTCAATTATT
TGTAAAAGTTGCGCCTAATTTAACAAATGAATATGATCCTGATGCATCTGCTAATATGTCAAGAATTG
TAACTTACTCAGATTTTTGGTGGAAAGGTAAATTAGTATTTAAAGCTAAACTAAGAGCCTCTCATACT
TGGAATCCAATTCAACAAATGAGTATTAATGTAGATAACCAATTTAACTATGTACCAAGTAATATTGG
AGGTATGAAAATTGTGTATGAAAAATCTCAACTAGCACCTAGA

'USA_AY742955_436_2003'

ATGAGTGATGGAGCAGTTCAACCAGACGGTGGTCAGCCTGCTGTCAGAAATGAAAGAGCTACAGGATC
TGGGAACGGGTCTGGAGGCGGGGGTGGTGGTGGTTCTGGGGGTGTGGGGATTTCTACGGGTACTTTCA
ATAATCAGACGGAATTTAAATTTTTGGAAAACGGATGGGTGGAAATCACAGCAAACTCAAGCAGACTT
GTACATTTAAATATGCCAGAAAGTGAAAATTATAGAAGAGTGGTTGTAAATAATTTGGATAAAACTGC
AGTTAACGGAAACATGGCTTTAGATGATACTCATGCACAAATTGTAACACCTTGGTCATTGGTTGATG
CAAATGCTTGGGGAGTTTGGTTTAATCCAGGAGATTGGCAACTAATTGTTAATACTATGAGTGAGTTG
CATTTAGTTAGTTTTGAACAAGAAATTTTTAATGTTGTTTTAAAGACTGTTTCAGAATCTGCTACTCA
GCCACCAACTAAAGTTTATAATAATGATTTAACTGCATCATTGATGGTTGCATTAGATAGTAATAATA
CTATGCCATTTACTCCAGCAGCTATGAGATCTGAGACATTGGGTTTTTATCCATGGAAACCAACCATA
CCAACTCCATGGAGATATTATTTTCAATGGGATAGAACATTAATACCATCTCATACTGGAACTAGTGG
CACACCAACAAATATATACCATGGTACAGATCCAGATGATGTTCAATTTTATACTATTGAAAATTCTG
TGCCAGTACACTTACTAAGAACAGGTGATGAATTTGCTACAGGAACATTTTTTTTTGATTGTAAACCA
TGTAGACTAACACATACATGGCAAACAAATAGAGCATTGGGCTTACCACCATTTCTAAATTCTTTGCC
TCAAGCTGAAGGAGGTACTAACTTTGGTTATATAGGAGTTCAACAAGATAAAAGACGTGGTGTAACTC
AAATGGGAAATACAAACTATATTACTGAAGCTACTATTATGAGACCAGCTGAGGTTGGTTATAGTGCA
CCATATTATTCTTTTGAGGCGTCTACACAAGGGCCATTTAAAACACCTATTGCAGCAGGACGGGGGGG
AGCGCAAACAGATGAAAATCAAGCAGCAGATGGTGATCCAAGATATGCATTTGGTAGACAACATGGTC
AAAAAACTACCACAACAGGAGAAACACCTGAGAGATTTACATATATAGCACATCAAGATACAGGAAGA
TATCCAGAAGGAGATTGGATTCAAAATATTAACTTTAACCTTCCTGTAACAGATGATAATGTATTGCT
ACCAACAGATCCAATTGGAGGTAAAACAGGAATTAACTATACTAATATATTTAATACTTATGGTCCTT
TAACTGCATTAAATAATGTACCACCAGTTTATCCAAATGGTCAAATTTGGGATAAAGAATTTGATACT
GACTTAAAACCAAGACTTCATGTAAATGCACCATTTGTTTGTCAAAATAATTGCCCTGGTCAATTATT
TGTAAAAGTTGCGCCTAATTTAACAAATGAATATGATCCTGATGCATCTGCTAATATGTCAAGAATTG
TAACTTACTCAGATTTTTGGTGGAAAGGTAAATTAGTATTTAAAGCTAAACTAAGAGCCTCTCATACT

TGGAATCCAATTCAACAAATGAGTATTAATGTAGATAACCAATTTAACTATGTACCAAGTAATATTGG
AGGTATGAAAATTGTCTATGAAAAATCTCAACTAGCACCTAGA

'JPN_D26079_1993'

ATGAGTGATGGAGCAGTTCAACCAGACGGTGGTCAACCTGCTGTCAGAAATGAAAGAGCTACAGGATC
TGGGAACGGGTCTGGAGGCGGGGGTGGTGGTGGTTCTGGGGGTGTGGGGATTTCTACGGGTACTTTCA
ATAATCAGACGGAATTTAAATTTTTGGAAAACGGATGGGTGGAAATCACAGCAAACTCAAGCAGACTT
GTACATTTAAATATGCCAGAAAGTGAAAATTATAGAAGAGTGGTTGTAAATAATTTGGATAAAACTGC
AGTTAACGGAAACATGGCTTTAGATGATACACATGCACAAATTGTAACACCTTGGTCATTGGTTGATG
CAAATGCTTGGGGAGTTTGGTTTAATCCAGGAGATTGGCAACTAATTGTTAATACTATGAGTGAGTTG
CATTTAGTTAGTTTTGAACAAGAAATTTTTAATGTTGTTTTAAAGACTGTTTCAGAATCTGCTACTCA
GCCACCAACTAAAGTTTATAATAATGATTTAACTGCATCATTGATGGTTGCATTAGATAGTAATAATA
CTATGCCATTTACTCCAGCAGCTATGAGATCTGAGACATTGGGTTTTTATCCATGGAAACCAACCATA
CCAACTCCATGGAGATATTATTTTCAATGGGATAGAACATTAATACCATCTCATACTGGAACTAGTGG
CACACCAACAAATATATACCATGGTACAGATCCAGATGATGTTCAATTTTATACTATTGAAAATTCTG
TGCCAGTACACTTACTAAGAACAGGTGATGAATTTGCTACAGGAACATTTTTTTTTGATTGTAAACCA
TGTAGACTAACACATACATGGCAAACAAATAGAGCATTGGGCTTACCACCATTTCTAAATTCTTTGCC
TCAATCTGAAGGAGGTACTAACTTTGGTTATATAGGAGTTCAACAAGATAAAAGACGTGGTGTAACTC
AAATGGGAAATACAAACTATATTACTGAAGCTACTATTATGAGACCAGCTGAGGTTGGTTATAGTGCA
CCATATTATTCTTTTGAGGCGTCTACACAAGGGCCATTTAAAACACCTATTGCAGCAGGACGGGGGGG
AGCGCAAACAGATGAAAATCAAGCAGCAGATGGTGATCCAAGATATGCATTTGGTAGACAACATGGTC
AAAAAACTACCACAACAGGAGAAACACCTGAGAGATTTACATATATAGCACATCAAGATACAGGAAGA
TATCCAGAAGGAGATTGGATTCAAAATATTAACTTTAACCTTCCTGTAACAAATGATAATGTATTACT
ACCAACAGATCCAATTGGAGGTAAAACAGGAATTAACTATACTAATATATTTAATACTTATGGTCCTT
TAACTGCATTAAATAATGTACCACCAGTTTATCCAAATGGTCAAATTTGGGATAAAGAATTTGATACT
GACTTAAAACCAAGACTTCATGTAAATGCACCATTTGTTTGTCAAAATAATTGTCCTGGTCAATTATT
TGTAAAAGTTGCGCCTAATTTAACAAATGAATATGATCCTGATGCATCTGCTAATATGTCAAGAATTG
TAACTTACTCAGATTTTTGGTGGAAAGGTAAATTAGTATTTAAAGCTAAACTAAGAGCCTCTCATACT
TGGAATCCAATTCAACAAATGAGTATTAATGTAGATAACCAATTTAACTATGTACCAAGTAATATTGG
AGGTATGAAAATTGTATATGAAAAATCTCAACTAGCACCTAGA

'FRA_DQ025942_01B1_2001'

ATGAGTGATGGAGCAGTTCAACCAGACGGTGGTCAACCTGCTGTCAGAAATGAAAGAGCAACAGGATC
TGGGAACGGGTCTGGAGGCGGGGGTGGTGGTGGTTCTGGGGGTGTGGGGATTTCTACGGGTACTTTCA
ATAATCAGACGGAATTTAAATTTTTGGAAAACGGATGGGTGGAAATCACAGCAAACTCAAGCAGACTT
GTACATTTAAATATGCCAGAAAGTGAAAATTATAGAAGAGTGGTTGTAAATAATTTGGATAAAACTGC
AGTTAACGGAAACATGGCTTTAGATGATACTCATGCACAAATTGTAACACCTTGGTCATTGGTTGATG
CAAATGCTTGGGGAGTTTGGTTTAATCCAGGAGATTGGCAACTAATTGTTAATACTATGAGTGAGTTG
CATTTAGTTAGTTTTGAACAAGAAATTTTTAATGTTGTTTTAAAGACTGTTTCAGAATCTGCTACTCA
GCCACCAACTAAAGTTTATAATAATGATTTAACTGCATCATTGATGGTTGCATTAGATAGTAATAATA
CTATGCCATTTACTCCAGCAGCTATGAGATCTGAGACATTGGGTTTTTATCCATGGAAACCAACCATA
CCAACTCCATGGAGATATTATTTTCAATGGGATAGAACATTAATACCATCTCATACTGGAACTAGTGG
CACACCAACAAATATATACCATGGTACAGATCCAGATGATGTTCAATTTTATACTATTGAAAATTCTG
TGCCAGTACACTTACTAAGAACAGGTGATGAATTTGCTACAGGAACATTTTTTTTTGATTGTAAACCA
TGTAGACTAACACATACATGGCAAACAAATAGAGCATTGGGCTTACCACCATTTCTAAATTCTTTGCC
TCAAGCTGAAGGAGGTACTAACTTTGGTTATATAGGAGTTCAACAAGATAAAAGACGTGGTGTAACTC
AAATGGGAAATACAAACTATATTACTGAAGCTACTATTATGAGACCAGCTGAGGTTGGTTATAGTGCA
CCATATTATTCTTTTGAGGCGTCTACACAAGGGCCATTTAAAACACCTATTGCAGCAGGACGGGGGGG
AGCGCAAACAGATGAAAATCAAGCAGCAGATGGTGATCCAAGATATGCATTTGGTAGACAACATGGTC
AAAAAACTACCACAACAGGAGAAACACCTGAGAGATTTACATATATAGCACATCAAGATACAGGAAGA
TATCCAGAAGGAGATTGGATTCAAAATATTAACTTTAACCTTCCTGTAACAGAAGATAATGTATTGCT
ACCAACAGATCCAATTGGAGGTAAAACAGGAATTAACTATACTAATATATTTAATACTTATGGTCCTT
TAACTGCATTAAATAATGTACCACCAGTTTATCCAAATGGTCAAATTTGGGATAAAGAATTTGATACT
GACTTAAAACCAAGACTTCATGTAAATGCACCATTTGTTTGTCAAAATAATTGTCCTGGTCAATTATT
TGTAAAAGTTGCGCCTAATTTAACAAATGAATATGATCCTGATGCATCTGCTAATATGTCAAGAATTG
TAACTTACTCAGATTTTTGGTGGAAAGGTAAATTAGTATTTAAAGCTAAACTAAGAGCCTCTCATACT

TGGAATCCAATTCAACAAATGAGTATTAATGTAGATAACCAATTTAACTATGTACCAAGTAATATTGG
AGGTATGAAAATTGTATATGAAAAATCTCAACTAGCACCTAGA

'FRA_DQ025943_2a_01S1_2001'

ATGAGTGATGGAGCAGTTCAACCAGACGGTGGTCAACCTGCTGTCAGAAATGAAAGAGCTACAGGATC
TGGGAACGGGTCTGGAGGCGGGGGTGGTGGTGGTTCTGGGGGTGTGGGGATTTCTACGGGTACTTTCA
ATAATCAGACGGAATTTAAATTTTTGGAAAACGGATGGGTGGAAATCACAGCAAACTCAAGCAGACTT
GTACATTTAAATATGCCAGAAAGTGAAAATTATAGAAGAGTGGTTGTAAATAATTTGGATAAAACTGC
AGTTAACGGAAACATGGCTTTAGATGATACTCATGCACAAATTGTAACACCTTGGTCATTGGTTGATG
CAAATGCTTGGGGAGTTTGGTTTAATCCAGGAGATTGGCAACTAATTGTTAATACTATGAGTGAGTTG
CATTTAGTTAGTTTTGAACAAGAAATTTTTAATGTTGTTTTAAAGACTGTTTCAGAATCTGCTACTCA
GCCACCAACTAAAGTTTATAATAATGACTTAACTGCATCATTGATGGTTGCATTAGATAGCAATAATA
CTATGCCATTTACTCCAGCAGCTATGAGATCTGAGACATTGGGTTTTTATCCATGGAAACCAACCATA
CCAACTCCATGGAGATATTATTTTCAATGGGATAGAACATTAATACCATCTCATACTGGAACTAGTGG
CACACCAACAAATATATACCATGGTACAGATCCAGATGATGTTCAATTTTATACTATTGAAAATTCTG
TGCCAGTACACTTACTAAGAACAGGTGATGAATTTGCTACAGGAACATTTTTTTTTGATTGTAAACCA
TGTAGACTAACACATACATGGCAAACAAATAGAGCATTGGGCTTACCACCATTTCTAAATTCTTTGCC
TCAAGCTGAAGGAGGTACTAACTTTGGTTATATAGGAGTTCAACAAGATAAAAGACGTGGTGTAACTC
AAATGGGAAATACAAACTATATTACTGAAGCTACTATTATGAGACCAGCTGAGGTTGGTTATAGTGCA
CCATATTATTCTTTTGAGGCGTCTACACAAGGGCCATTTAAAACACCTATTGCAGCAGGACGGGGGGG
AGCGCAAACAGATGAAAATCAAGCAGCAGATGGTGATCCAAGATATGCATTTGGTAGACAACATGGTC
AAAAAACTACCACAACAGGAGAAACACCTGAGAGATTTACATATATAGCACATCAAGATACAGGAAGA
TATCCAGAAGGAGATTGGATTCAAAATATTAACTTTAACCTTCCTGTAACAAATGATAATGTATTGCT
ACCAACAGATCCAATTGGAGGTAAAGCAGGAATTAACTATACTAATATATTTAATACTTATGGTCCTT
TAACTGCATTAAATAATGTACCACCAGTTTATCCAAATGGTCAAATTTGGGATAAAGAATTTGATACT
GACTTAAAACCAAGACTTCATGTAAATGCACCATTTGTTTGTCAAAATAATTGTCCTGGTCAATTATT
TGTAAAAGTTGCGCCTAATTTAACAAATGAATATGATCCTGATGCATCTGCTAATATGTCAAGAATTG
TAACTTACTCAGATTTTTGGTGGAAAGGTAAATTAGTATTTAAAGCTAAACTAAGAGCCTCTCATACT
TGGAATCCAATTCAACAAATGAGTATTAATGTAGATAACCAATTTAACTATGTACCAAGTAATATTGG
AGGTATGAAGATTGTATATGAAAAATCTCAACTAGCACCTAGA

'FRA_DQ025944_2a_02B2_2002'

ATGAGTGATGGAGCAGTTCAACCAGACGGTGGTCAACCTGCTGTCAGAAATGAAAGAGCTACAGGATC
TGGGAACGGGTCTGGAGGCGGGGGTGGTGGTGGTTCTGGGGGTGTGGGGATTTCTACGGGTACTTTCA
ATAATCAGACGGAATTTAAATTTTTGGAAAACGGATGGGTGGAAATCACAGCAAACTCAAGCAGACTT
GTACATTTAAATATGCCAGAAAGTGAAAATTATAGAAGAGTGGTTGTAAATAATTTGGATAAAACTGC
AGTTAACGGAAACATGGCTTTAGATGATACTCATGCACAAATTGTAACACCTTGGTCATTGGTTGATG
CAAATGCTTGGGGAGTTTGGTTTAATCCAGGAGATTGGCAACTAATTGTTAATACTATGAGTGAGTTG
CATTTAGTTAGTTTTGAACAAGAAATTTTTAATGTTGTTTTAAAGACTGTTTCAGAATCTGCTACTCA
GCCACCAACTAAAGTTTATAATAATGATTTAACTGCATCATTGATGGTTGCATTAGATAGCAATAATA
CTATGCCATTTACTCCAGCAGCTATGAGATCTGAGACATTGGGTTTTTATCCATGGAAACCAACCATA
CCAACTCCATGGAGATATTATTTTCAATGGGATAGAACATTAATACCATCTCATACTGGAACTAGTGG
CACACCAACAAATATATACCATGGTACAGATCCAGATGATGTTCAATTTTATACTATTGAAAATTCTG
TGCCAGTACACTTACTAAGAACAGGTGATGAATTTGCTACAGGAACATTTTTTTTTGATTGTAAACCA
TGTAGACTAACACATACATGGCAAACAAATAGAGCATTGGGCTTACCACCATTTCTAAATTCTTTGCC
TCAAGCTGAAGGAGGTACTAACTTTGGTTATATAGGAGTTCAACAAGATAAAAGACGTGGTGTAACTC
AAATGGGAAATACAAACTATATTACTGAAGCTACTATTATGAGACCAGCTGAGGTTGGTTATAGTGCA
CCATATTATTCTTTTGAGGCGTCTACACAAGGGCCATTTAAAACACCTATTGCAGCAGGACGGGGGGG
AGCGCAAACAGATGAAAATCAAGCAGCAGATGGTGATCCAAGATATGCATTTGGTAGACAACATGGTC
AAAAAACTACCACAACAGGAGAAACACCTGAGAGATTTACATATATAGCACATCAAGATACAGGAAGA
TATCCAGAAGGAGATTGGATTCAAAATATTAACTTTAACCTTCCTGTAACAAATGATAATGTATTGCT
ACCAACAGATCCAATTGGAGGTAAAACAGGAATTAACTATACTAATATATTTAATACTTATGGTCCTT
TAACTGCATTAAATAATGTACCACCAGTTTATCCAAATGGTCAAATTTGGGATAAAGAATTTGATACT
GACTTAAAACCAAGACTTCATGTAAATGCACCATTTGTTTGTCAAAATAATTGTCCTGGTCAATTATT
TGTAAAAGTTGCGCCTAATTTAACAAATGAATATGATCCTGATGCATCTGCTAATATGTCAAGAATTG
TAACTTACTCAGATTTTTGGTGGAAAGGTAAATTAGTATTTAAAGCTAAACTAAGAGCCTCTCATACT

TGGAATCCAATTCAACAAATGAGTATTAATGTAGATAACCAATTTAACTATGTACCAAGTAATATTGG
AGGTATGAAGATTGTATATGAAAAATCTCAACTAGCACCTAGA
'FRA_DQ025945_2a_02B3_2002'
ATGAGTGATGGAGCAGTTCAACCAGACGGTGGTCAGCCTGCTGTCAGAAATGAAAGAGCTACAGGATC
TGGGAACGGGTCTGGAGGCGGGGGTGGTGGTGGTTCTGGGGGTGTGGGGATTTCTACGGGTACTTTCA
ATAATCAGACGGAATTTAAATTTTTGGAAAACGGATGGGTGGAAATCACAGCAAACTCAAGCAGACTT
GTACATTTAAATATGCCAGAAAGTGAAAATTATAGAAGAGTGGTTGTAAATAATTTGGATAAAACTGC
AGTTAACGGAAACATGGCTTTAGATGATACTCATGCACAAATTGTAACACCTTGGTCATTGGTTGATG
CAAATGCTTGGGGAGTTTGGTTTAATCCAGGAGATTGGCAACTAATTGTTAATACTATGAGTGAGTTG
CATTTAGTTAGTTTTGAACAAGAAATTTTTAATGTTGTTTTAAAGACTGTTTCAGAATCTGCTACTCA
GCCACCAACTAAAGTTTATAATAATGATTTAACTGCATCATTGATGGTTGCATTAGATAGCAATAATA
CTATGCCATTTACTCCAGCAGCTATGAGATCTGAGACATTGGGTTTTTATCCATGGAAACCAACCATA
CCAACTCCATGGAGATATTATTTTCAATGGGATAGAACATTAATACCATCTCATACTGGAACTAGTGG
CACACCAACAAATATATACCATGGTACAGATCCAGATGATGTTCAATTTTATACTATTGAAAATTCTG
TGCCAGTACACTTACTAAGAACAGGTGATGAATTTGCTACAGGAACATTTTTTTTTGATTGTAAACCA
TGTAGACTAACACATACATGGCAAACAAATAGAGCATTGGGCTTACCACCATTTCTAAATTCTTTGCC
TCAAGCTGAAGGAGGTACTAACTTTGGTTATATAGGAGTTCAACAAGATAAAAGACGTGGTGTAACTC
AAATGGGAAATACAAACTATATTACTGAAGCTACTATTATGAGACCAGCTGAGGTTGGTTATAGTGCA
CCATATTATTCTTTTGAGGCGTCTACACAAGGGCCATTTAAAACACCTATTGCAGCAGGACGGGGGGG
AGCGCAAACAGATGAAAATCAAGCAGCAGATGGTGATCCAAGATATGCATTTGGTAGACAACATGGTC
AAAAAACTACCACAACAGGAGAAACACCTGAGAGATTTACATATATAGCACATCAAGATACAGGAAGA
TATCCAGAAGGAGATTGGATTCAAAATATTAACTTTAACCTTCCTGTAACAAATGATAATGTATTGCT
ACCAACAGATCCAATTGGAGGTAAAACAGGAATTAACTATACTAATATATTTAATACTTATGGTCCTT
TAACTGCATTAAATAATGTACCACCAGTTTATCCAAATGGTCAAATTTGGGATAAAGAATTTGATACT
GACTTAAAACCAAGACTTCATGTAAATGCACCATTTGTTTGTCAAAATAATTGTCCTGGTCAATTATT
TGTAAAAGTTGCGCCTAATTTAACAAATGAATATGATCCTGATGCATCTGCTAATATGTCAAGAATTG
TAACTTACTCAGATTTTTGGTGGAAAGGTAAATTAGTATTTAAAGCTAAACTAAGAGCCTCTCATACT
TGGAATCCAATTCAACAAATGAGTATTAATGTAGATAACCAATTTAACTATGTACCAAGTAATATTGG
AGGTATGAAGATTGTATATGAAAAATCTCAACTAGCACCTAGA
'FRA_DQ025947_2a_02B5_2002'
ATGAGTGATGGAGCAGTTCAACCAGACGGTGGTCAGCCTGCTGTCAGAAATGAAAGAGCTACAGGATC
TGGGAACGGGTCTGGAGGCGGGGGTGGTGGTGGTTCTGGGGGTGTGGGGATTTCTACGGGTACTTTCA
ATAATCAGACGGAATTTAAATTTTTGGAAAACGGATGGGTGGAAATCACAGCAAACTCAAGCAGACTT
GTACATTTAAATATGCCAGAAAGTGAAAATTATAGAAGAGTGGTTGTAAATAATTTGGATAAAACTGC
AGTTAACGGAAACATGGCTTTAGATGATACTCATGCACAAATTGTAACACCTTGGTCATTGGTTGATG
CAAATGCTTGGGGAGTTTGGTTTAATCCAGGAGATTGGCAACTAATTGTTAATACTATGAGTGAGTTG
CATTTAGTTAGTTTTGAACAAGAAATTTTTAATGTTGTTTTAAAGACTGTTTCAGAATCTGCTACTCA
GCCACCAACTAAAGTTTATAATAATGATTTAACTGCATCATTGATGGTTGCATTAGATAGCAATAATA
CTATGCCATTTACTCCAGCAGCTATGAGATCTGAGACATTGGGTTTTTATCCATGGAAACCAACCATA
CCAACTCCATGGAGATATTATTTTCAATGGGATAGAACATTAATACCATCTCATACTGGAACTAGTGG
CACACCAACAAATATATACCATGGTACAGATCCAGATGATGTTCAATTTTATACTATTGAAAATTCTG
TGCCAGTACACTTACTAAGAACAGGTGATGAATTTGCTACAGGAACATTTTTTTTTGATTGTAAACCA
TGTAGACTAACACATACATGGCAAACAAATAGAGCATTGGGCTTACCACCATTTCTAAATTCTTTGCC
TCAAGCTGAAGGAGGTACTAACTTTGGTTATATAGGAGTTCAACAAGATAAAAGACGTGGTGTAACTC
AAATGGGAAATACAAACTATATTACTGAAGCTACTATTATGAGACCAGCTGAGGTTGGTTATAGTGCA
CCATATTATTCTTTTGAGGCGTCTACACAAGGGCCATTTAAAACACCTATTGCAGCAGGACGAGGGGG
AGCGCAAACAGATGAAAATCAAGCAGCAGATGGTGATCCAAGATATGCATTTGGTAGACAACATGGTC
AAAAAACTACCACAACAGGAGAAACACCTGAGAGATTTACATATATAGCACATCAAGATACAGGAAGA
TATCCAGAAGGAGATTGGATTCAAAATATTAACTTTAACCTTCCTGTAACAAATGATAATGTATTGCT
ACCAACAGATCCAATTGGAGGTAAAACAGGAATTAACTATACTAATATATTTAATACTTATGGTCCTT
TAACTGCATTAAATAATGTACCACCAGTTTATCCAAATGGTCAAATTTGGGATAAAGAATTTGATACT
GACTTAAAACCAAGACTTCATGTAAATGCACCATTTGTTTGTCAAAATAATTGTCCTGGTCAATTATT
TGTAAAAGTTGCGCCTAATTTAACAAATGAATATGATCCTGATGCATCTGCTAATATGTCAAGAATTG
TAACTTACTCAGATTTTTGGTGGAAAGGTAAATTAGTATTTAAAGCTAAACTAAGAGCCTCTCATACT

TGGAATCCAATTCAACAAATGAGTATTAATGTAGATAACCAATTTAACTATGTACCAAGTAATATTGG
AGGTATGAAGATTGTATATGAAAAATCTCAACTAGCACCTAGA
'FRA_DQ025950_2a_02B9_2002'
ATGAGTGATGGAGCAGTTCAACCAGACGGTGGTCAGCCTGCTGTCAGAAATGAAAGAGCTACAGGATC
TGGGAACGGGTCTGGAGGCGGGGGTGGTGGTGGTTCTGGGGGTGTGGGGATTTCTACGGGTACTTTCA
ATAATCAGACGGAATTTAAATTTTTGGAAAACGGATGGGTGGAAATCACAGCAAACTCAAGCAGACTT
GTACATTTAAATATGCCAGAAAGTGAAAATTATAGAAGAGTGGTTGTAAATAATTTGGATAAAACTGC
AGTTAACGGAAACATGGCTTTAGATGATACCCATGCACAAATTGTAACACCTTGGTCATTGGTTGATG
CAAATGCTTGGGGAGTTTGGTTTAATCCAGGAGATTGGCAACTAATTGTTAATACTATGAGTGAGTTG
CATTTAGTTAGTTTTGAACAAGAAATTTTTAATGTTGTTTTAAAGACTGTTTCAGAATCTGCTACTCA
GCCACCAACTAAAGTTTATAATAATGATTTAACTGCATCATTGATGGTTGCATTAGATAGTAATAATA
CTATGCCATTTACTCCAGCAGCTATGAGATCTGAGACATTGGGTTTTTATCCATGGAAACCAACCATA
CCAACTCCATGGAGATATTATTTTCAATGGGATAGAACATTAATACCATCTCATACTGGAACTAGTGG
CACACCAACAAATATATACCATGGTACAGATCCAGATGATGTTCAATTTTATACTATTGAAAATTCTG
TGCCAGTACACTTACTAAGAACAGGTGATGAATTTGCTACAGGAACATTTTTTTTTGATTGTAAACCA
TGTAGACTAACACATACATGGCAAACAAATAGAGCATTGGGCTTACCACCATTTCTAAATTCTTTGCC
TCAAGCTGAAGGAGGTACTAACTTTGGTTATATAGGAGTTCAACAAGATAAAAGACGTGGTGTAACTC
AAATGGGAAATACAAACTATATTACTGAAGCTACTATTATGAGACCAGCTGAGGTTGGTTATAGTGCA
CCATATTATTCTTTTGAGGCGTCTACACAAGGGCCATTTAAAACACCTATTGCAGCAGGACGGGGGGG
AGCGCAAACAGATGAAAATCAAGCAGCAGATGGTGATCCAAGATATGCATTTGGTAGACAACATGGTC
AAAAAACTACCACAACAGGAGAAACACCTGAGAGATTTACATATATAGCACATCAAGATACAGGAAGA
TATCCAGAAGGAGATTGGATTCAAAATATTAACTTTAACCTTCCTGTAACAAATGATAATGTATTGCT
ACCAACAGATCCAATTGGAGGTAAAGCAGGAATTAACTATACTAATATATTTAATACTTATGGTCCTT
TAACTGCATTAAATAATGTACCACCAGTTTATCCAAATGGTCAAATTTGGGATAAAGAATTTGATACT
GACTTAAAACCAAGACTTCATGTAAATGCACCATTTGTTTGTCAAAATAATTGTCCTGGTCAATTATT
TGTAAAAGTTGCGCCTAATTTAACAAATGAATATGATCCTGATGCATCTGCTAATATGTCAAGAATTG
TAACTTACTCAGATTTTTGGTGGAAAGGTAAATTAGTATTTAAAGCTAAACTAAGAGCCTCTCATACT
TGGAATCCAATTCAACAAATGAGTATTAATGTAGATAACCAATTTAACTATGTACCAAGTAATATTGG
AGGTATGAAAATTGTATATGAAAAATCTCAACTAGCACCTAGA
'FRA_DQ025951_03B10_2003'
ATGAGTGATGGAGCAGTTCAACCAGACGGTGGTCAACCTGCTGTCAGAAATGAAAGAGCAACAGGATC
TGGGAACGGGTCTGGAGGCGGGGGTGGTGGTGGTTCTGGGGGTGTGGGGATTTCTACGGGTACTTTCA
ATAATCAGACGGAATTTAAATTTTTGGAAAACGGATGGGTGGAAATCACAGCAAACTCAAGCAGACTT
GTACATTTAAATATGCCAGAAAGTGAAAATTATAGAAGAGTGGTTGTAAATAACTTGGATAAAACTGC
AGTTAACGGAAACATGGCTTTAGATGATACTCATGCACAAATTGTAACACCTTGGTCATTGGTTGATG
CAAATGCTTGGGGAGTTTGGTTTAATCCAGGAGATTGGCAACTAATTGTTAATACTATGAGTGAGTTG
CATTTAGTTAGTTTTGAACAAGAAATTTTTAATGTTGTTTTAAAGACTGTTTCAGAATCTGCTACTCA
GCCACCAACTAAAGTTTATAATAATGATTTAACTGCATCATTGATGGTTGCATTAGATAGTAATAATA
CTATGCCATTTACTCCAGCAGCTATGAGATCTGAGACATTGGGTTTTTATCCATGGAAACCAACCATA
CCAACTCCATGGAGATATTATTTTCAATGGGATAGAACATTAATACCATCTCATACTGGAACTAGTGG
CACACCAACAAATATATACCATGGTACAGATCCAGATGATGTTCAATTTTATACTATTGAAAATTCTG
TGCCAGTACACTTACTAAGAACAGGTGATGAATTTGCTACAGGAACATTTTTTTTTGATTGTAAACCA
TGTAGACTAACACATACATGGCAAACAAATAGAGCATTGGGCTTACCACCATTTCTAAATTCTTTGCC
TCAAGCTGAAGGAGGTACTAACTTTGGTTATATAGGAGTTCAACAAGATAAAAGACGTGGTGTAACTC
AAATGGGAAATACAAACTATATTACTGAAGCTACTATTATGAGACCAGCTGAGGTTGGTTATAGTGCA
CCATATTATTCTTTTGAGGCGTCTACACAAGGGCCATTTAAAACACCTATTGCAGCAGGACGGGGGGG
AGCGCAAACAGATGAAAATCAAGCAGCAGATGGTGATCCAAGATATGCATTTGGTAGACAACATGGTC
AAAAAACTACCACAACAGGAGAAACACCTGAGAGATTTACATATATAGCACATCAAGATACAGGAAGA
TATCCAGAAGGAGATTGGATTCAAAATATTAACTTTAACCTTCCTGTAACAGAAGATAATGTATTGCT
ACCAACAGATCCAATTGGAGGTAAAACAGGAATTAACTATACTAATATATTTAATACTTATGGTCCTT
TAACTGCATTAAATAATGTACCACCAGTTTATCCAAATGGTCAAATTTGGGATAAAGAATTTGATACT
GACTTAAAACCAAGACTTCATGTAAATGCACCATTTGTTTGTCAAAATAATTGTCCTGGTCAATTATT
TGTAAAAGTTGCGCCTAATTTAACAAATGAATATGATCCTGATGCATCTGCTAATATGTCAAGAATTG
TAACTTACTCAGATTTTTGGTGGAAAGGTAAATTAGTATTTAAAGCTAAACTAAGAGCCTCTCATACT

TGGAATCCAATTCAACAAATGAGTATTAATGTAGATAACCAATTTAACTATGTACCAAGTAATATTGG
AGGTATGAAAATTGTATATGAAAAATCTCAACTAGCACCTAGA
'FRA_DQ025952_2a_03B12_2003'
ATGAGTGATGGAGCAGTTCAACCAGACGGTGGTCAACCTGCTGTCAGAAATGAAAGAGCTACAGGATC
TGGGAACGGGTCTGGAGGCGGGGGTGGTGGTGGTTCTGGGGGTGTGGGGATTTCTACGGGTACTTTCA
ATAATCAGACGGAATTTAAATTTTTGGAAAACGGATGGGTGGAAATCACAGCAAACTCAAGCAGACTT
GTACATTTAAATATGCCAGAAAGTGAAAATTATAGAAGAGTGGTTGTAAATAATTTGGATAAAACTGC
AGTTAACGGAAACATGGCTTTAGATGATACTCATGCACAAATTGTAACACCTTGGTCATTGGTTGATG
CAAATGCTTGGGGAGTTTGGTTTAATCCAGGAGATTGGCAACTAATTGTTAATACTATGAGTGAGTTG
CATTTAGTTAGTTTTGAACAAGAAATTTTTAATGTTGTTTTAAAGACTGTTTCAGAATCTGCTACTCA
GCCACCAACTAAAGTTTATAATAATGATTTAACTGCATCATTGATGGTTGCATTAGATAGTAATAATA
CTATGCCATTTACTCCAGCAGCTATGAGATCTGAGACATTGGGTTTTTATCCATGGAAACCAACCATA
CCAACTCCATGGAGATATTATTTTCAATGGGATAGAACATTAATACCATCTCATACTGGAACTAGTGG
CACACCAACAAATATATACCATGGTACAGATCCAGATGATGTTCAATTTTATACTATTGAAAATTCTG
TGCCAGTACACTTACTAAGAACAGGTGATGAATTTGCTACAGGAACATTTTTTTTTGATTGTAAACCA
TGTAGACTAACACATACATGGCAAACAAATAGAGCATTGGGCTTACCACCATTTCTAAATTCTTTGCC
TCAATCTGAAGGAGGTACTAACTTTGGTTATATAGGAGTTCAACAAGATAAAAGACGTGGTGTAACTC
AAATGGGAAATACAAACTATATCACTGAAGCTACTATTATGAGACCAGCTGAGGTTGGTTATAGTGCA
CCATATTATTCTTTTGAGGCGTCTACACAAGGGCCATTTAAAACACCTATTGCAGCAGGACGGGGGGG
AGCGCAAACAGATGAAAATCAAGCAGCAGATGGTGATCCAAGATATGCATTTGGTAGACAACATGGTC
AAAAAACTACCACAACAGGAGAAACACCTGAGAGATTTACATATATAGCACATCAAGATACAGGAAGA
TATCCAGAAGGAGATTGGATTCAAAATATTAACTTTAACCTTCCTGTAACAAATGATAATGTATTGCT
ACCAACAGATCCAATTGGAGGTAAAACAGGAATCAACTATACTAATATATTTAATACTTATGGTCCTT
TAACTGCATTAAATAATGTACCACCAGTTTATCCAAATGGTCAAATTTGGGATAAAGAATTTGATACT
GACTTAAAACCAAGACTTCATGTAAATGCACCATTTGTTTGTCAAAATAATTGTCCTGGTCAATTATT
TGTAAAAGTTGCGCCTAATTTAACAAATGAATATGATCCTGATGCATCTGCTAATATGTCAAGAATTG
TAACTTACTCAGATTTTTGGTGGAAAGGTAAATTAGTATTTAAAGCTAAACTAAGAGCCTCTCATACT
TGGAATCCAATTCAACAAATGAGTATTAATGTAGATAACCAATTTAACTATGTACCAAGTAATATTGG
AGGTATGAAAATTGTATATGAGAAATCTCAACTAGCACCTAGA
'FRA_DQ025954_03B14_2003'
ATGAGTGATGGAGCAGTTCAACCAGACGGTGGTCAACCTGCTGTCAGAAATGAAAGAGCAACAGGATC
TGGGAACGGGTCTGGAGGCGGGGGTGGTGGTGGTTCTGGGGGTGTGGGGATTTCTACGGGTACTTTCA
ATAATCAGACGGAATTTAAATTTTTGGAAAACGGATGGGTGGAAATCACAGCAAACTCAAGCAGACTT
GTACATTTAAATATGCCAGAAAGTGAAAATTATAGAAGAGTGGTTGTAAATAATTTGGATAAAACTGC
AGTTAACGGAAACATGGCTTTAGATGATACTCATGCACAAATTGTAACACCTTGGTCATTGGTTGATG
CAAATGCTTGGGGAGTTTGGTTTAATCCAGGAGATTGGCAACTAATTGTTAATACTATGAGTGAGTTG
CATTTAGTTAGTTTTGAACAAGAAATTTTTAATGTTGTTTTAAAGACTGTTTCAGAATCTGCTACTCA
GCCACCAACTAAAGTTTATAATAATGATTTAACTGCATCATTGATGGTTGCATTAGATAGTAATAATA
CTATGCCATTTACTCCAGCAGCTATGAGATCTGAGACATTGGGTTTTTATCCATGGAAACCAACCATA
CCAACTCCATGGAGATATTATTTTCAATGGGATAGAACATTAATACCATCTCATACTGGAACTAGTGG
CACACCAACAAATATATACCATGGTACAGATCCAGATGATGTTCAATTTTATACTATTGAAAATTCTG
TGCCAGTACACTTACTAAGAACAGGTGATGAATTTGCTACAGGAACATTTTTTTTTGATTGTAAACCA
TGTAGACTAACACATACATGGCAAACAAATAGAGCATTGGGCTTACCACCATTTCTAAATTCTTTGCC
TCAAGCTGAAGGAGGTACTAACTTTGGTTATATAGGAGTTCAACAAGATAAAAGACGTGGTGTAACTC
AAATGGGAAATACAAACTATATTACTGAAGCTACTATTATGAGACCAGCTGAGGTTGGTTATAGTGCA
CCATATTATTCTTTTGAGGCGTCTACACAAGGGCCATTTAAAACACCTATTGCAGCAGGACGGGGGGG
AGCGCAAACAGATGAAAATCAAGCAGCAGATGGTGATCCAAGATATGCATTTGGTAGACAACATGGTC
AAAAAACTACCACAACAGGAGAAACACCTGAGAGATTTACATATATAGCACATCAAGATACAGGAAGA
TATCCAGAAGGAGATTGGATTCAAAATATTAACTTTAACCTTCCTGTAACAGAAGATAATATATTGCT
ACCAACAGATCCAATTGGAGGTAAAACAGGAATTAACTATACTAATATATTTAATACTTATGGTCCTT
TAACTGCATTAAATAATGTACCACCAGTTTATCCAAATGGTCAAATTTGGGATAAAGAATTTGATACT
GACTTAAAACCAAGACTTCATGTAAATGCACCATTTGTTTGTCAAAATAATTGTCCTGGTCAATTATT
TGTAAAAGTTGCGCCTAATTTAACAAATGAATATGATCCTGATGCATCTGCTAATATGTCAAGAATTG
TAACTTACTCAGATTTTTGGTGGAAAGGTAAATTAGTATTTAAAGCTAAACTAAGAGCCTCTCATACT

TGGAATCCAATTCAACAAATGAGTATTAATGTAGATAACCAATTTAACTATGTACCAAGTAATATTGG
AGGTATGAAAATTGTATATGAAAAATCTCAACTAGCACCTAGA
'FRA_DQ025958_2a_03C2_2003'
ATGAGTGATGGAGCAGTTCAACCAGACGGTGGTCAGCCTGCTGTCAGAAATGAAAGAGCTACAGGATC
TGGGAACGGGTCTGGAGGCGGGGGTGGTGGTGGTTCTGGGGGTGTGGGGATTTCTACGGGTACTTTCA
ATAATCAGACGGAATTTAAATTTTTGGAAAACGGATGGGTGGAAATCACAGCAAACTCAAGCAGACTT
GTACATTTAAATATGCCAGAAAGTGAAAATTATAGAAGAGTGGTTGTAAATAATTTGGATAAAACTGC
AGTTAACGGAAACATGGCTTTAGATGATACTCATGCACAAATTGTAACACCTTGGTCATTGGTTGATG
CAAATGCTTGGGGAGTTTGGTTTAATCCAGGAGATTGGCAACTAATTGTTAATACTATGAGTGAGTTG
CATTTAGTTAGTTTTGAACAAGAAATTTTTAATGTTGTTTTAAAGACTGTTTCAGAATCTGCTACTCA
GCCACCAACTAAAGTTTATAATAATGATTTAACTGCATCATTGATGGTTGCATTAGATAGCAATAATA
CTATGCCATTTACTCCAGCAGCTATGAGATCTGAGACATTGGGTTTTTATCCATGGAAACCAACCATA
CCAACTCCATGGAGATATTATTTTCAATGGGATAGAACATTAATACCATCTCATACTGGAACTAGTGG
CACACCAACAAATATATACCATGGTACAGATCCAGATGATGTTCAATTTTATACTATTGAAAATTCTG
TGCCAGTACACTTACTAAGAACAGGTGATGAATTTGCTACAGGAACATTTTTTTTTGATTGTAAACCA
TGTAGACTAACACATACATGGCAAACAAATAGAGCATTGGGCTTACCACCATTTCTAAATTCTTTGCC
TCAAGCTGAAGGAGGTACTAACTTTGGTTATATAGGAGTTCAACAAGATAAAAGACGTGGTGTAACTC
AAATGGGAAATACAAACTATATTACTGAAGCTACTATTATGAGACCAGCTGAGGTTGGTTATAGTGCA
CCATATTATTCTTTTGAGGCGTCTACACAAGGGCCATTTAAAACACCTATTGCAGCAGGACGGGGGGG
AGCGCAAACAGATGAAAATCAAGCAGCAGATGGTGATCCAAGATATGCATTTGGTAGACAACATGGTC
AAAAAACTACCACAACAGGAGAAACACCTGAGAGATTTACATATATAGCACATCAAGATACAGGAAGA
TATCCAGAAGGAGATTGGATTCAAAATATTAACTTTAACCTTCCTGTAACAAATGATAATGTATTGCT
ACCAACAGATCCAATTGGAGGTAAAACAGGAATTAACTATACTAATATATTTAATACTTATGGTCCTT
TAACTGCATTAAATAATGTGCCACCAGTTTATCCAAATGGTCAAATTTGGGATAAAGAATTTGATACT
GACTTAAAACCAAGACTTCATGTAAATGCACCATTTGTTTGTCAAAATAATTGTCCTGGTCAATTATT
TGTAAAAGTTGCGCCTAATTTAACAAATGAATATGATCCTGATGCATCTGCTAATATGTCAAGAATTG
TAACTTACTCAGATTTTTGGTGGAAAGGTAAATTAGTATTTAAAGCTAAACTAAGAGCCTCTCATACT
TGGAATCCAATTCAACAAATGAGTATTAATGTAGATAACCAATTTAACTATGTACCAAGTAATATTGG
AGGTATGAAGATTGTATATGAAAAATCTCAACTAGCACCTAGA
'FRA_DQ025960_03C4_2003'
ATGAGTGATGGAGCAGTTCAACCAGACGGTGGTCAACCTGCTGTCAGAAATGAAAGAGCAACAGGATC
TGGGAACGGGTCTGGAGGCGGGGGTGGTGGTGGTTCTGGGGGTGTGGGGATTTCTACGGGTACTTTCA
ATAATCAGACGGAATTTAAATTTTTGGAAAACGGATGGGTGGAAATCACAGCAAACTCAAGCAGACTT
GTACATTTAAATATGCCAGAAAGTGAAAATTATAGAAGAGTGGTTGTAAATAATTTGGATAAAACTGC
AGTTAACGGAAACATGGCTTTAGATGATACTCATGCACAAATTGTAACACCTTGGTCATTGGTTGATG
CAAATGCTTGGGGAGTTTGGTTTAATCCAGGAGATTGGCAACTAATTGTTAATACTATGAGTGAGTTG
CATTTAGTTAGTTTTGAACAAGAAATTTTTAATGTTGTTTTAAAGACTGTTTCAGAATCTGCTACTCA
GCCACCAACTAAAGTTTATAATAATGATTTAACTGCATCATTGATGGTTGCATTAGATAGTAATAATA
CTATGCCATTTACTCCAGCAGCTATGAGATCTGAGACATTGGGTTTTTATCCATGGAAACCAACCATA
CCAACTCCATGGAGATATTATTTTCAATGGGATAGAACATTAATACCATCTCATACTGGAACTAGTGG
CACACCAACAAATATATACCATGGTACAGATCCAGATGATGTTCAATTTTATACTATTGAAAATTCTG
TGCCAGTACACTTACTAAGAACAGGTGATGAATTTGCTACAGGAACATTTTTTTTTGATTGTAAACCA
TGTAGACTAACACATACATGGCAAACAAATAGAGCATTGGGCTTACCACCATTTCTAAATTCTTTGCC
TCAAGCTGAAGGAGGTACTAACTTTGGTTATATAGGAGTTCAACAAGATAAAAGACGTGGTGTAACTC
AAATGGGAAATACAAACTATATTACTGAAGCTACTATTATGAGACCAGCTGAGGTTGGTTATAGTGCA
CCATATTATTCTTTTGAGGCGTCTACACAAGGGCCATTTAAAACACCTATTGCAGCAGGACGGGGGGG
AGCGCAAACAGATGAAAATCAAGCAGCAGATGGTGATCCAAGATATGCATTTGGTAGACAACATGGTC
AAAAAACTACCACAACAGGAGAAACACCTGAGAGATTTACATATATAGCACATCAAGATACAGGAAGA
TATCCAGAAGGAGATTGGATTCAAAATATTAACTTTAACCTTCCTGTAACAGAAGATAATGTATTGCT
ACCAACAGATCCAATTGGAGGTAAAACAGGAATTAACTATACTAATATATTTAATACTTATGGTCCTT
TAACTGCATTAAATAATGTACCACCAGTTTATCCAAATGGTCAAATTTGGGATAAAGAATTTGATACC
GACTTAAAACCAAGACTTCATGTAAATGCACCATTTGTTTGTCAAAATAATTGTCCTGGTCAATTATT
TGTAAAAGTTGCGCCTAATTTAACAAATGAATATGATCCTGATGCATCTGCTAATATGTCAAGAATTG
TAACTTACTCAGATTTTTGGTGGAAAGGTAAATTAGTATTTAAAGCTAAACTAAGAGCCTCTCATACT

TGGAATCCAATTCAACAAATGAGTATTAATGTAGATAACCAATTTAACTATGTACCAAGTAATATTGG
AGGTATGAAAATTGTATATGAAAAATCTCAACTAGCACCTAGA
'FRA_DQ025961_2b_03C5_2003'
ATGAGTGATGGAGCAGTTCAACCAGACGGTGGTCAGCCTGCTGTCAGAAATGAAAGAGCTACAGGATC
TGGGAACGGGTCTGGAGGCGGGGGTGGTGGTGGTTCTGGGGGTGTGGGGATTTCTACGGGTACTTTCA
ATAATCAGACGGAATTTAAATTTTTGGAAAACGGATGGGTGGAAATCACAGCAAACTCAAGCAGACTT
GTACATTTAAATATGCCAGAAAGTGAAAATTATAGAAGAGTGGTTGTAAATAATTTGGATAAAACTGC
AGTTAACGGAAACATGGCTTTAGATGATACTCATGCACAAATTGTAACACCTTGGTCATTGGTTGATG
CAAATGCTTGGGGAGTTTGGTTTAATCCAGGAGATTGGCAACTAATTGTTAATACTATGAGTGAGTTG
CATTTAGTTAGTTTTGAACAAGAAATTTTTAATGTTGTTTTAAAGACTGTTTCAGAATCTGCTACTCA
GCCACCAACTAAAGTTTATAATAATGATTTAACTGCATCATTGATGGTTGCATTAGATAGTAATAATA
CTATGCCATTTACTCCAGCAGCTATGAGATCTGAGACATTGGGTTTTTATCCATGGAAACCAACCATA
CCAACTCCATGGAGATATTATTTTCAATGGGATAGAACATTAATACCATCTCATACTGGAACTAGTGG
CACACCAACAAATATATACCATGGTACAGATCCAGATGATGTTCAATTTTATACTATTGAAAATTCTG
TGCCAGTACACTTACTAAGAACAGGTGATGAATTTGCTACAGGAACATTTTTTTTTGATTGTAAACCA
TGTAGACTAACACATACATGGCAAACAAATAGAGCATTGGGCTTACCACCATTTCTAAATTCTTTGCC
TCAAGCTGAAGGAGGTACTAACTTTGGTTATATAGGAGTTCAACAAGATAAAAGACGTGGTGTAACTC
AAATGGGAAATACAAACTATATTACTGAAGCTACTATTATGAGACCAGCTGAGGTTGGTTATAGTGCA
CCATATTATTCTTTTGAGGCGTCTACACAAGGGCCATTTAAAACACCTATTGCAGCAGGACGGGGGGG
AGCACAAACAGATGAAAATCAAGCAGCAGATGGTGATCCAAGATATGCATTTGGTAGACAACATGGTC
AAAAAACTACCACAACAGGAGAAACACCTGAGAGATTTACATATATAGCACATCAAGATACAGGAAGA
TATCCAGAAGGAGATTGGATTCAAAATATTAACTTTAACCTTCCTGTAACAGATGATAATGTATTGCT
ACCAACAGATCCAATTGGAGGTAAAACAGGAATTAACTATACTAATATATTTAATACTTATGGTCCTT
TAACTGCATTAAATAATGTACCACCAGTTTATCCAAATGGTCAAATTTGGGATAAAGAATTTGATACT
GACTTAAAACCAAGACTTCATGTAAATGCACCATTTGTTTGTCAAAATAATTGCCCTGGTCAATTATT
TGTAAAAGTTGCGCCTAATTTAACAAATGAATATGATCCTGATGCATCTGCTAATATGTCAAGAATTG
TAACTTACTCAGATTTTTGGTGGAAAGGTAAATTAGTATTTAAAGCTAAACTAAGAGCCTCTCATACT
TGGAATCCAATTCAACAAATGAGTATTAATGTAGATAACCAATTTAACTATGTACCAAGTAATATTGG
AGGTATGAAAATTGTCTATGAAAAATCTCAACTAGCACCTAGA
'FRA_DQ025962_2a_03C6_2003'
ATGAGTGATGGAGCAGTTCAACCAGACGGTGGTCAGCCTGCTGTCAGAAATGAAAGAGCTACAGGATC
TGGGAACGGGTCTGGAGGCGGGGGTGGTGGTGGTTCTGGGGGTGTGGGGATTTCTACGGGTACTTTCA
ATAATCAGACGGAATTTAAATTTTTGGAAAACGGATGGGTGGAAATCACAGCAAACTCAAGCAGACTT
GTACATTTAAATATGCCAGAAAGTGAAAATTATAGAAGAGTGGTTGTAAATAATTTGGATAAAACTGC
AGTTAACGGAAACATGGCTTTAGATGATACTCATGCACAAATTGTAACACCTTGGTCATTGGTTGATG
CAAATGCTTGGGGAGTTTGGTTTAATCCAGGAGATTGGCAACTAATTGTTAATACTATGAGTGAGTTG
CATTTAGTTAGTTTTGAACAAGAAATTTTTAATGTTGTTTTAAAGACTGTTTCAGAATCTGCTACTCA
GCCACCAACTAAAGTTTATAATAATGATTTAACTGCATCATTGATGGTTGCATTAGATAGCAATAATA
CTATGCCATTTACTCCAGCAGCTATGAGATCTGAGACATTGGGTTTTTATCCATGGAAACCAACCATA
CCAACTCCATGGAGATATTATTTTCAATGGGATAGAACATTAATACCATCTCATACTGGAACTAGTGG
CACACCAACAAATATATACCATGGTACAGATCCAGATGATGTTCAATTTTATACTATTGAAAACTCTG
TGCCAGTACACTTACTAAGAACAGGTGATGAATTTGCTACAGGAACATTTTTTTTTGATTGTAAACCA
TGTAGACTAACACATACATGGCAAACAAATAGAGCATTGGGCTTACCACCATTTCTAAATTCTTTGCC
TCAAGCTGAAGGAGGTACTAACTTTGGTTATATAGGAGTTCAACAAGATAAAAGACGTGGTGTAACTC
AAATGGGAAATACAAACTATATTACTGAAGCTACTATTATGAGACCAGCTGAGGTTGGTTATAGTGCA
CCATATTATTCTTTTGAGGCGTCTACACAAGGGCCATTTAAAACACCTATTGCAGCAGGACGGGGGGG
AGCGCAAACAGATGAAAATCAAGCAGCAGATGGTGATCCAAGATATGCATTTGGTAGACAACATGGTC
AAAAAACTACCACAACAGGAGAAACACCTGAGAGATTTACATATATAGCACATCAAGATACAGGAAGA
TATCCAGAAGGAGATTGGATTCAAAATATTAACTTTAACCTTCCTGTAACAAATGATAATGTATTGCT
ACCAACAGATCCAATTGGAGGTAAAACAGGAATTAACTATACTAATATATTTAATACTTATGGTCCTT
TAACTGCATTAAATAATGTACCACCAGTTTATCCAAATGGTCAAATTTGGGATAAAGAATTTGATACT
GACTTAAAACCAAGACTTCATGTAAATGCACCATTTGTTTGTCAAAATAATTGTCCTGGTCAATTATT
TGTAAAAGTTGCGCCTAATTTAACAAATGAATATGATCCTGATGCATCTGCTAATATGTCAAGAATTG
TAACTTACTCAGATTTTTGGTGGAAAGGTAAATTAGTATTTAAAGCTAAACTAAGAGCCTCTCATACT

TGGAATCCAATTCAACAAATGAGTATTAATGTAGATAACCAATTTAACTATGTACCAAGTAATATTGG
AGGTATGAAGATTGTATATGAAAAATCTCAACTAGCACCTAGA

'FRA_DQ025964_03C8_2003'

ATGAGTGATGGAGCAGTTCAACCAGACGGTGGTCAACCTGCTGTCAGAAATGAAAGAGCAACAGGATC
TGGGAACGGGTCTGGAGGCGGGGGTGGTGGTGGTTCTGGGGGTGTGGGGATTTCTACGGGTACTTTCA
ATAATCAGACGGAATTTAAATTTTTGGAAAACGGATGGGTGGAAATCACAGCAAACTCAAGCAGACTT
GTACATTTAAATATGCCAGAAAGTGAAAATTATAGAAGAGTGGTTGTAAATAATTTGGATAAAACTGC
AGTTAACGGAAACATGGCTTTAGATGATACTCATGCACAAATTGTAACACCTTGGTCATTGGTTGATG
CAAATGCTTGGGGAGTTTGGTTTAATCCAGGAGATTGGCAACTAATTGTTAATACTATGAGTGAGTTG
CATTTAGTTAGTTTTGAACAAGAAATTTTTAATGTTGTTTTAAAGACTGTTTCAGAATCTGCTACTCA
GCCACCAACTAAAGTTTATAATAATGATTTAACTGCATCATTGATGGTTGCATTAGATAGTAATAATA
CTATGCCATTTACTCCAGCAGCTATGAGATCTGAGACATTGGGTTTTTATCCATGGAAACCAACCATA
CCAACTCCATGGAGATATTATTTTCAATGGGATAGAACATTAATACCATCTCATACTGGAACTAGTGG
CACACCAACAAATATATACCATGGTACAGATCCAGATGATGTTCAATTTTATACTATTGAAAATTCTG
TGCCAGTACACTTACTAAGAACAGGTGATGAATTTGCTACAGGAACATTTTTTTTTGATTGTAAACCA
TGTAGACTAACACATACATGGCAAACAAATAGAGCATTGGGCTTACCACCATTTCTAAATTCTTTGCC
TCAAGCTGAAGGAGGTACTAACTTTGGTTATATAGGAGTTCAACAAGATAAAAGACGTGGTGTAACTC
AAATGGGAAATACAAACTATATTACTGAAGCTACTATTATGAGACCAGCTGAGGTTGGTTATAGTGCA
CCATATTATTCTTTTGAGGCGTCTACACAAGGGCCATTTAAAACACCTATTGCAGCAGGACGGGGGGG
AGCGCAAACAGATGAAAATCAAGCAGCAGATGGTGATCCAAGATATGCATTTGGTAGACAACATGGTC
AAAAAACTACCACAACAGGAGAAACACCTGAGAGATTTACATATATAGCACATCAAGATACAGGAAGA
TATCCAGAAGGAGATTGGATTCAAAATATTAACTTTAACCTTCCTGTAACAGAAGATAATGTATTGCT
ACCAACAGATCCAATTGGAGGTAAAACAGGAATTAACTATACTAATATATTTAATACTTATGGTCCTT
TAACTGCATTAAATAATGTACCACCAGTTTATCCAAATGGTCAAATTTGGGATAAAGAATTTGATACT
GACTTAAAACCAAGACTTCATGTAAATGCACCATTTGTTTGTCAAAATAATTGTCCTGGTCAATTATT
TGTAAAAGTTGCGCCTAATTTAACAAATGAATATGATCCTGATGCATCTGCTAATATGTCAAGAATTG
TAACTTACTCAGATTTTTGGTGGAAAGGTAAATTAGTATTTAAAGCTAAACTAAGAGCCTCTCATACT
TGGAATCCAATTCAACAAATGAGTATTAATGTAGATAACCAATTTAACTATGTACCAAGTAATATTGG
AGGTATGAAAATTGTTTATGAAAAATCTCAACTAGCACCTAGA

'FRA_DQ025965_03C9_2003'

ATGAGTGATGGAGCAGTTCAACCAGACGGTGGTCAACCTGCTGTCAGAAATGAAAGAGCAACAGGATC
TGGGAACGGGTCTGGAGGCGGGGGTGGTGGTGGTTCTGGGGGTGTGGGGATTTCTACGGGTACTTTCA
ATAATCAGACGGAATTTAAATTTTTGGAAAACGGATGGGTGGAAATCACAGCAAACTCAAGCAGACTT
GTACATTTAAATATGCCAGAAAGTGAAAATTATAGAAGAGTGGTTGTAAATAATTTGGATAAAACTGC
AGTTAACGGAAACATGGCTTTAGATGATACTCATGCACAAATTGTAACACCTTGGTCATTAGTTGATG
CAAATGCTTGGGGAGTTTGGTTTAATCCAGGAGATTGGCAACTAATTGTTAATACTATGAGTGAGTTG
CATTTAGTTAGTTTTGAACAAGAAATTTTTAATGTTGTTTTAAAGACTGTTTCAGAATCTGCTACTCA
GCCACCAACTAAAGTTTATAATAATGATTTAACTGCATCATTGATGGTTGCATTAGATAGTAATAATA
CTATGCCATTTACTCCAGCAGCTATGAGATCTGAGACATTGGGTTTTTATCCATGGAAACCAACCATA
CCAACTCCATGGAGATATTATTTTCAATGGGATAGAACATTAATACCATCTCATACTGGAACTAGTGG
CACACCAACAAATATATACCATGGTACAGATCCAGATGATGTTCAATTTTATACTATTGAAAATTCTG
TGCCAGTACACTTACTAAGAACAGGTGATGAATTTGCTACAGGAACATTTTTTTTTGATTGTAAACCA
TGTAGACTAACACATACATGGCAAACAAATAGAGCATTGGGCTTACCACCATTTCTAAATTCTTTGCC
TCAAGCTGAAGGAGGTACTAACTTTGGTTATATAGGAGTTCAACAAGATAAAAGACGTGGTGTAACTC
AAATGGGAAATACAAACTATATTACTGAAGCTACTATTATGAGACCAGCTGAGGTTGGTTATAGTGCA
CCATATTATTCTTTTGAGGCGTCTACACAAGGGCCATTTAAAACACCTATTGCAGCAGGACGGGGGGG
AGCGCAAACAGATGAAAATCAAGCAGCAGATGGTGATCCAAGATATGCATTTGGTAGACAACATGGTC
AAAAAACTACCACAACAGGAGAAACACCTGAGAGATTTACATATATAGCACATCAAGATACAGGAAGA
TATCCAGAAGGAGATTGGATTCAAAATATTAACTTTAACCTTCCTGTAACAGAAGATAATGTATTGCT
ACCAACAGATCCAATTGGAGGTAAAACAGGAATTAACTATACTAATATATTTAATACTTATGGTCCTT
TAACTGCATTAAATAATGTACCACCAGTTTATCCAAATGGTCAAATTTGGGATAAAGAATTTGATACT
GACTTAAAACCAAGACTTCATGTAAATGCACCATTTGTTTGTCAAAATAATTGTCCTGGTCAATTATT
TGTAAAAGTTGCGCCTAATTTAACAAATGAATATGATCCTGATGCATCTGCTAATATGTCAAGAATTG
TAACTTACTCAGATTTTTGGTGGAAAGGTAAATTAGTATTTAAAGCTAAACTAAGAGCCTCTCATACT

TGGAATCCAATTCAACAAATGAGTATTAATGTAGATAACCAATTTAACTATGTACCAAGTAATATTGG
AGGTATGAAAATTGTATATGAAAAATCTCAACTAGCACCTAGA

'FRA_DQ025969_03S5_2003'

ATGAGTGATGGAGCAGTTCAACCAGACGGTGGTCAACCTGCTGTCAGAAATGAAAGAGCAACAGGATC
TGGGAACGGGTCTGGAGGCGGGGGTGGTGGTGGTTCTGGGGGTGTGGGGATTTCTACGGGTACTTTCA
ATAATCAGACGGAATTTAAATTTTTGGAAAACGGATGGGTGGAAATCACAGCAAACTCAAGCAGACTT
GTACATTTAAATATGCCAGAAAGTGAAAATTATAGAAGAGTGGTTGTAAATAATTTGGATAAAACTGC
AGTTAACGGAAACATGGCTTTAGATGATACTCATGCACAAATTGTAACACCTTGGTCATTGGTTGATG
CAAATGCTTGGGGAGTTTGGTTTAATCCAGGAGATTGGCAACTAATTGTTAATACTATGAGTGAGTTG
CATTTAGTTAGTTTTGAACAAGAAATTTTTAATGTTGTTTTAAAGACTGTTTCAGAATCTGCTACTCA
GCCACCAACTAAAGTTTATAATAATGATTTAACTGCATCATTGATGGTTGCATTAGATAGTAATAATA
CTATGCCATTTACTCCAGCAGCTATGAGATCTGAGACATTGGGTTTTTATCCATGGAAACCAACCATA
CCAACTCCATGGAGATATTATTTTCAATGGGATAGAACATTAATACCATCTCATACTGGAACTAGTGG
CACACCAACAAATATATATCATGGTACAGATCCAGATGATGTTCAATTTTATACTATTGAAAATTCTG
TGCCAGTACACTTACTAAGAACAGGTGATGAATTTGCTACAGGAACATTTTTTTTTGATTGTAAACCA
TGTAGACTAACACATACATGGCAAACAAATAGAGCATTGGGCTTACCACCATTTCTAAATTCTTTGCC
TCAAGCTGAAGGAGGTACTAACTTTGGTTATATAGGAGTTCAACAAGATAAAAGACGTGGTGTAACTC
AAATGGGAAATACAAACTATATTACTGAAGCTACTATTATGAGACCAGCTGAGGTTGGTTATAGTGCA
CCATATTATTCTTTTGAGGCGTCTACACAAGGGCCATTTAAAACACCTATTGCAGCAGGACGGGGGGG
AGCGCAAACAGATGAAAATCAAGCAGCAGATGGTGATCCAAGATATGCATTTGGTAGACAACATGGTC
AAAAAACTACCACAACAGGAGAAACACCTGAGAGATTTACATATATAGCACATCAAGATACAGGAAGA
TATCCAGAAGGAGATTGGATTCAAAATATTAACTTTAACCTTCCTGTAACAGAAGATAATGTATTGCT
ACCAACAGATCCAATTGGAGGTAAAACAGGAATTAACTATACTAATATATTTAATACTTATGGTCCTT
TAACTGCATTAAATAATGTACCACCAGTTTATCCAAATGGTCAAATTTGGGATAAAGAATTTGATACT
GACTTAAAACCAAGACTTCATGTAAATGCACCATTTGTTTGTCAAAATAATTGTCCTGGTCAATTATT
TGTAAAAGTTGCGCCTAATTTAACAAATGAATATGATCCTGATGCATCTGCTAATATGTCAAGAATTG
TAACTTACTCAGATTTTTGGTGGAAAGGTAAATTAGTATTTAAAGCTAAACTAAGAGCCTCTCATACT
TGGAATCCAATTCAACAAATGAGTATTAATGTAGATAACCAATTTAACTATGTACCAAGTAATATTGG
AGGTATGAAAATTGTATATGAAAAATCTCAACTAGCACCTAGA

'FRA_DQ025975_04S6_2004'

ATGAGTGATGGAGCAGTTCAACCAGACGGTGGTCAACCTGCTGTCAGAAATGAAAGAGCAACAGGATC
TGGGAACGGGTCTGGAGGCGGGGGTGGTGGTGGTTCTGGGGGTGTGGGGATTTCTACGGGTACTTTCA
ATAATCAGACGGAATTTAAATTTTTGGAAAACGGATGGGTGGAAATCACAGCAAACTCAAGCAGACTT
GTACATTTAAATATGCCAGAAAGTGAAAATTATAGAAGAGTGGTTGTAAATAATTTGGATAAAACTGC
AGTTAACGGAAACATGGCTTTAGATGATACTCATGCACAAATTGTAACACCTTGGTCATTGGTTGATG
CAAATGCTTGGGGAGTTTGGTTTAATCCAGGAGATTGGCAACTAATTGTTAATACTATGAGTGAGTTG
CATTTAGTTAGTTTTGAACAAGAAATTTTTAATGTTGTTTTAAAGACTGTTTCAGAATCTGCTACTCA
GCCACCAACTAAAGTTTATAATAATGATTTAACTGCATCATTGATGGTTGCATTAGATAGTAATAATA
CTATGCCATTTACTCCAGCAGCTATGAGATCTGAGACATTGGGTTTTTATCCATGGAAACCAACCATA
CCAACTCCATGGAGATATTATTTTCAATGGGATAGAACATTAATACCATCTCATACTGGAACTAGTGG
CACACCAACAAATATATACCATGGTACAGATCCAGATGATGTTCAATTTTATACTATTGAAAATTCTG
TGCCAGTACACTTACTAAGAACAGGTGATGAATTTGCTACAGGAACATTTTTTTTTGATTGTAAACCA
TGTAGACTAACACATACATGGCAAACAAATAGAGCATTGGGCTTACCACCATTTCTAAATTCTTTGCC
TCAAGCTGAAGGAGGTACTAACTTTGGTTATATAGGAGTTCAACAAGATAAAAGACGTGGTGTAACTC
AAATGGGAAATACAAACTATATTACTGAAGCTACTATTATGAGACCAGCTGAGGTTGGTTATAGTGCA
CCATATTATTCTTTTGAGGCGTCTACACAAGGGCCATTTAAAACACCTATTGCAGCAGGACGGGGGGG
AGCGCAAACAGATGAAAATCAAGCAGCAGATGGTGATCCAAGATATGCATTTGGTAGACAACATGGTC
AAAAAACTACCACAACAGGAGAAACACCTGAGAGATTTACATATATAGCACATCAAGATACAGGAAGA
TATCCAGAAGGAGATTGGATTCAAAATATTAACTTTAACCTTCCTGTAACAGAAGATAATGTATTGCT
ACCAACAGATCCAATTGGAGGTAAAACAGGAATTAACTATACTAATATATTTAATACTTATGGTCCTT
TAACTGCATTAAATAATGTACCACCAGTTTATCCAAATGGTCAAATTTGGGATAAAGAATTTGATACT
GACTTAAAACCAAGACTTCATGTAAATGCACCATTTGTTTGTCAAAATAATTGTCCTGGTCAATTATT
TGTAAAAGTTGCACCTAATTTAACAAATGAATATGATCCTGATGCATCTGCTAATATGTCAAGAATTG
TAACTTACTCAGATTTTTGGTGGAAAGGTAAATTAGTATTTAAAGCTAAACTAAGAGCCTCTCATACT

TGGAATCCAATTCAACAAATGAGTATTAATGTAGATAACCAATTTAACTATGTACCAAGTAATATTGG
AGGTATGAAAATTGTATATGAAAAATCTCAACTAGCACCTAGA
'FRA_DQ025976_04S7_2004'
ATGAGTGATGGAGCAGTTCAACCAGACGGTGGTCAACCTGCTGTCAGAAATGAAAGAGCAACAGGATC
TGGGAACGGGTCTGGAGGCGGGGGTGGTGGTGGTTCTGGGGGTGTGGGGATTTCTACGGGTACTTTCA
ATAATCAGACGGAATTTAAATTTTTGGAAAACGGATGGGTGGAAATCACAGCAAACTCAAGCAGACTT
GTACATTTAAATATGCCAGAAAGTGAAAATTATAGAAGAGTGGTTGTAAATAATTTGGATAAAACTGC
AGTTAACGGAAACATGGCTTTAGATGATACTCATGCACAAATTGTAACACCTTGGTCATTGGTTGATG
CAAATGCTTGGGGAGTATGGTTTAATCCAGGAGATTGGCAACTAATTGTTAATACTATGAGTGAGTTG
CATTTAGTTAGTTTTGAACAAGAAATTTTTAATGTTGTTTTAAAGACTGTTTCAGAATCTGCTACTCA
GCCACCAACTAAAGTTTATAATAATGATTTAACTGCATCATTGATGGTTGCATTAGATAGTAATAATA
CTATGCCATTTACTCCAGCAGCTATGAGATCTGAGACATTGGGTTTTTATCCATGGAAACCAACCATA
CCAACTCCATGGAGATATTATTTTCAATGGGATAGAACATTAATACCATCTCATACTGGAACTAGTGG
CACACCAACAAATATATACCATGGTACAGATCCAGATGATGTTCAATTTTATACTATTGAAAATTCTG
TGCCAGTACACTTACTAAGAACAGGTGATGAATTTGCTACAGGAACATTTTTTTTTGATTGTAAACCA
TGTAGACTAACACATACATGGCAAACAAATAGAGCATTGGGCTTACCACCATTTCTAAATTCTTTGCC
TCAAGCTGAAGGAGGTACTAACTTTGGTTATATAGGAGTTCAACAAGATAAAAGACGTGGTGTAACTC
AAATGGGAAATACAAACTATATTACTGAAGCTACTATTATGAGACCAGCTGAGGTTGGTTATAGTGCA
CCATATTATTCTTTTGAGGCGTCTACACAAGGGCCATTTAAAACACCTATTGCAGCAGGACGGGGGGG
AGCGCAAACAGATGAAAATCAAGCAGCAGATGGTGATCCAAGATATGCATTTGGTAGACAACATGGTC
AAAAAACTACCACAACAGGAGAAACACCTGAGAGATTTACATATATAGCACATCAAGATACAGGAAGA
TATCCAGAAGGAGATTGGATTCAAAATATTAACTTTAACCTTCCTGTAACAGAAGATAATGTATTGCT
ACCAACAGATCCAATTGGAGGTAAAACAGGAATTAACTATACTAATATATTTAATACTTATGGTCCTT
TAACTGCATTAAATAATGTACCACCAGTTTATCCAAATGGTCAAATTTGGGATAAAGAATTTGATACT
GACTTAAAACCAAGACTTCATGTAAATGCACCATTTGTTTGTCAAAATAATTGTCCTGGTCAATTATT
TGTAAAAGTTGCGCCTAATTTAACAAATGAATATGATCCTGATGCATCTGCTAATATGTCAAGAATTG
TAACTTACTCAGATTTTTGGTGGAAAGGTAAATTAGTATTTAAAGCTAAACTAAGAGCCTCTCATACT
TGGAATCCAATTCAACAAATGAGTATTAATGTAGATAACCAATTTAACTATGTACCAAGTAATATTGG
AGGTATGAAAATTGTATATGAAAAATCTCAACTAGCACCTAGA
'FRA_DQ025982_2a_04S13_2004'
ATGAGTGATGGAGCAGTTCAACCAGACGGTGGTCAACCTGCTGTCAGAAATGAAAGAGCTACAGGATC
TGGGAACGGGTCTGGAGGCGGGGGTGGTGGTGGTTCTGGGGGTGTGGGGATTTCTACGGGTACTTTCA
ATAATCAGACGGAATTTAAATTTTTGGAAAACGGATGGGTGGAAATCACAGCAAACTCAAGCAGACTT
GTACATTTAAATATGCCAGAAAGTGAAAATTATAGAAGAGTGGTTGTAAATAATTTGGATAAAACTGC
AGTTAACGGAAACATGGCTTTAGATGATACTCATGCACAAATTGTAACACCTTGGTCATTGGTTGATG
CAAATGCTTGGGGAGTTTGGTTTAATCCAGGAGATTGGCAACTAATTGTTAATACTATGAGTGAGTTG
CATTTAGTTAGTTTTGAACAAGAAATTTTTAATGTTGTTTTAAAGACTGTTTCAGAATCTGCTACTCA
GCCACCAACTAAAGTTTATAATAATGATTTAACTGCATCATTGATGGTTGCATTAGATAGCAATAATA
CTATGCCATTTACTCCAGCAGCTATGAGATCTGAGACATTGGGTTTTTATCCATGGAAACCAACCATA
CCAACTCCATGGAGATATTATTTTCAATGGGATAGAACATTAATACCATCTCATACTGGAACTAGTGG
CACACCAACAAATATATACCATGGTACAGATCCAGATGATGTTCAATTTTATACTATTGAAAATTCTG
TGCCAGTACACTTACTAAGAACAGGTGATGAATTTGCTACAGGAACATTTTTTTTTGATTGTAAACCA
TGTAGACTAACACATACATGGCAAACAAATAGAGCATTGGGCTTACCACCATTTCTAAATTCTTTGCC
TCAAGCTGAAGGAGGTACTAACTTTGGTTATATAGGAGTTCAGCAAGATAAAAGACGTGGTGTAACTC
AAATGGGAAATACAAACTATATTACTGAAGCTACTATTATGAGACCAGCTGAGGTTGGTTATAGTGCA
CCATATTATTCTTTTGAGGCGTCTACACAAGGGCCATTTAAAACACCTATTGCAGCAGGACGGGGGGG
AGCGCAAACAGATGAAAATCAAGCAGCAGATGGTGATCCAAGATATGCATTTGGTAGACAACATGGTC
AAAAAACTACCACAACAGGAGAAACACCTGAGAGATTTACATATATAGCACATCAAGATACAGGAAGA
TATCCAGAAGGAGATTGGATTCAAAATATTAACTTTAACCTTCCTGTAACAAATGATAATGTATTGCT
ACCAACAGATCCAATTGGAGGTAAAACAGGAATTAACTATACTAATATATTTAATACTTATGGTCCTT
TAACTGCATTAAATAATGTACCACCAGTTTATCCAAATGGTCAAATTTGGGATAAAGAATTTGATACT
GACTTAAAACCAAGACTTCATGTAAATGCACCATTTGTTTGTCAAAATAATTGTCCTGGTCAATTATT
TGTAAAAGTTGCGCCTAATTTAACAAATGAATATGATCCTGATGCATCTGCTAATATGTCAAGAATTG
TAACTTACTCAGATTTTTGGTGGAAAGGTAAATTAATATTTAAAGCTAAACTAAGAGCCTCTCATACT

TGGAATCCAATTCAACAAATGAGTATTAATGTAGATAACCAATTTAACTATGTACCAAGTAATATTGG
AGGTATGAAGATTGTATATGAAAAATCTCAACTAGCACCTAGA

'FRA_DQ025983_2a_04S14_2004'

ATGAGTGATGGAGCAGTTCAACCAGACGGTGGTCAGCCTGCTGTCAGAAATGAAAGAGCGACAGGATC
TGGGAACGGGTCTGGAGGCGGGGGTGGTGGTGGTTCTGGGGGTGTGGGGATTTCTACGGGTACTTTCA
ATAATCAGACGGAATTTAAATTTTTGGAAAACGGATGGGTGGAAATCACAGCAAACTCAAGCAGACTT
GTACATTTAAATATGCCAGAAAGTGAAAATTATAGAAGAGTGGTTGTAAATAATTTGGATAAAACTGC
AGTTAACGGAAACATGGCTTTAGATGATACTCATGCACAAATTGTAACACCTTGGTCATTGGTTGATG
CAAATGCTTGGGGAGTTTGGTTTAATCCAGGAGATTGGCAACTAATTGTTAATACTATGAGTGAGTTG
CATTTAGTTAGTTTTGAACAAGAAATTTTTAATGTTGTTTTAAAGACTGTTTCAGAATCTGCTACTCA
GCCACCAACTAAAGTTTATAATAATGATTTAACTGCATCATTGATGGTTGCATTAGATAGCAATAATA
CTATGCCATTTACTCCAGCAGCTATGAGATCTGAGACATTGGGTTTTTATCCATGGAAACCAACCATA
CCAACTCCATGGAGATATTATTTTCAATGGGATAGAACATTAATACCATCTCATACTGGAACTAGTGG
CACACCAACAAATATATACCATGGTACAGATCCAGATGATGTTCAATTTTATACTATTGAAAATTCTG
TGCCAGTACACTTACTAAGAACAGGTGATGAATTTGCTACAGGAACATTTTTTTTTGATTGTAAACCA
TGTAGACTAACACATACATGGCAAACAAATAGAGCATTGGGCTTACCACCATTTCTAAATTCTTTGCC
TCAAGCTGAAGGAGGTACTAACTTTGGTTATATAGGAGTTCAACAAGATAAAAGACGTGGTGTAACTC
AAATGGGAAATACAAACTATATTACTGAAGCTACTATTATGAGACCAGCTGAGGTTGGTTATAGTGCA
CCATATTATTCTTTTGAGGCGTCTACACAAGGGCCATTTAAAACACCTATTGCAGCAGGACGGGGGGG
AGCGCAAACAGATGAAAATCAAGCAGCAGATGGTGATCCAAGATATGCATTTGGTAGACAACATGGTC
AAAAAACTACCACAACAGGAGAAACACCTGAGAGATTTACATATATAGCACATCAAGATACAGGAAGA
TATCCAGAAGGAGATTGGATTCAAAATATTAACTTTAACCTTCCTGTAACAAATGATAATGTATTGCT
ACCAACAGATCCAATTGGAGGTAAAACAGGAATTAACTATACTAATATATTTAATACTTATGGTCCTT
TAACTGCATTAAATAATGTACCACCAGTTTATCCAAATGGTCAAATTTGGGATAAAGAATTTGATACT
GACTTAAAACCAAGACTTCATGTAAATGCACCATTTGTTTGTCAAAATAATTGTCCTGGTCAATTATT
TGTAAAAGTTGCGCCTAATTTAACAAATGAATATGATCCTGATGCATCTGCTAATATGTCAAGAATTG
TAACTTACTCAGATTTTTGGTGGAAAGGTAAATTAGTATTTAAAGCTAAACTAAGAGCCTCTCATACT
TGGAATCCAATTCAACAAATGAGTATTAATGTAGATAACCAATTTAACTATGTACCAAGTAATATTGG
AGGTATGAAGATTGTATATGAAAAATCTCAACTAGCACCTAGA

'FRA_DQ025984_2a_04S15_2004'

ATGAGTGATGGAGCAGTTCAACCAGACGGTGGTCAGCCTGCTGTCAGAAATGAAAGAGCTACAGGATC
TGGGAACGGGTCTGGAGGCGGGGGTGGTGGTGGTTCTGGGGGTGTGGGGATTTCTACGGGTACTTTCA
ATAATCAGACGGAATTTAAATTTTTGGAAAACGGATGGGTGGAAATCACAGCAAACTCAAGCAGACTT
GTACATTTAAATATGCCAGAAAGTGAAAATTATAGAAGAGTGGTTGTAAATAATTTGGATAAAACTGC
AGTTAACGGAAACATGGCTTTAGATGATACTCATGCACAAATTGTAACACCTTGGTCATTGGTTGATG
CAAATGCTTGGGGAGTTTGGTTTAATCCAGGAGATTGGCAACTAATTGTTAATACTATGAGTGAGTTG
CATTTAGTTAGTTTTGAACAAGAAATTTTTAATGTTGTTTTAAAGACTGTTTCAGAATCTGCTACTCA
GCCACCAACTAAAGTTTATAATAATGATTTAACTGCATCATTGATGGTTGCATTAGATAGCAATAATA
CTATGCCATTTACTCCAGCAGCTATGAGATCTGAGACATTGGGTTTTTATCCATGGAAACCAACCATA
CCAACTCCATGGAGATATTATTTTCAATGGGATAGAACATTAATACCATCTCATACTGGAACTAGTGG
CACACCAACAAATATATACCATGGTACAGATCCAGATGATGTTCAATTTTATACTATTGAAAATTCTG
TGCCAGTACACTTACTAAGAACAGGTGATGAATTTGCTACAGGAACATTTTTTTTTGATTGTAAACCA
TGTAGACTAACACATACATGGCAAACAAATAGAGCATTGGGCTTACCACCATTTCTAAATTCTTTGCC
TCAAGCTGAAGGAGGTACTAACTTTGGTTATATAGGAGTTCAACAAGATAAAAGACGTGGTGTAACTC
AAATGGGAAATACAAACTATATTACTGAAGCTACTATTATGAGACCAGCTGAGGTTGGTTATAGTGCA
CCATATTATTCTTTTGAGGCGTCTACACAAGGGCCATTTAAAACACCTATTGCAGCAGGACGGGGGGG
AGCACAAACAGATGAAAATCAAGCAGCAGATGGTGATCCAAGATATGCATTTGGTAGACAACATGGTC
AAAAAACTACCACAACAGGAGAAACACCTGAGAGATTTACATATATAGCACATCAAGATACAGGAAGA
TATCCAGAAGGAGATTGGATTCAAAATATTAACTTTAACCTTCCTGTAACAAATGATAATGTATTGCT
ACCAACAGATCCAATTGGAGGTAAAACAGGAATTAACTATACTAATATATTTAATACTTATGGTCCTT
TAACTGCATTAAATAATGTACCACCAGTTTATCCAAATGGTCAAATTTGGGATAAAGAATTTGATACT
GACTTAAAACCAAGACTTCATGTAAATGCACCATTTGTTTGTCAAAATAATTGTCCTGGTCAATTATT
TGTAAAAGTTGCGCCTAATTTAACAAATGAATATGATCCTGATGCATCTGCTAATATGTCAAGAATTG
TAACTTACTCAGATTTTTGGTGGAAAGGTAAATTAGTATTTAAAGCTAAACTAAGAGCCTCTCATACT

TGGAATCCAATTCAACAAATGAGTATTAATGTAGATAACCAATTTAACTATGTACCAAGTAATATTGG
AGGTATGAAGATTGTATATGAAAAATCTCAACTAGCACCTAGA
'FRA_DQ025985_04S16_2004'
ATGAGTGATGGAGCAGTTCAACCAGACGGTGGTCAACCTGCTGTCAGAAATGAAAGAGCAACAGGATC
TGGGAACGGGTCTGGAGGCGGGGGTGGTGGTGGTTCTGGGGGTGTGGGGATTTCTACGGGTACTTTCA
ATAATCAGACGGAATTTAAATTTTTGGAAAACGGATGGGTGGAAATCACAGCAAACTCAAGCAGACTT
GTACATTTAAATATGCCAGAAAGTGAAAATTATAGAAGAGTGGTTGTAAATAATTTGGATAAAACTGC
AGTTAACGGAAACATGGCTTTAGATGATACTCATGCACAAATTGTAACACCTTGGTCATTGGTTGATG
CAAATGCTTGGGGAGTTTGGTTTAATCCAGGAGATTGGCAACTAATTGTTAATACTATGAGTGAGTTG
CATTTAGTTAGTTTTGAACAAGAAATTTTTAATGTTGTTTTAAAGACTGTTTCAGAATCTGCTACTCA
GCCACCAACTAAAGTTTATAATAATGATTTAACTGCATCATTGATGGTTGCATTAGATAGTAATAATA
CTATGCCATTTACTCCAGCAGCTATGAGATCTGAGACATTGGGTTTTTATCCATGGAAACCAACCATA
CCAACTCCATGGAGATATTATTTTCAATGGGATAGAACATTAATACCATCTCATACTGGAACTAGTGG
CACACCAACAAATATATACCATGGTACAGATCCAGATGATGTTCAATTTTATACTATTGAAAATTCTG
TGCCAGTACACTTACTAAGAACAGGTGATGAATTTGCTACAGGAACATTTTTTTTTGATTGTAAACCA
TGTAGACTAACACATACATGGCAAACAAATAGAGCATTGGGCTTACCACCATTTCTAAATTCTTTGCC
TCAAGCTGAAGGAGGTACTAACTTTGGTTATATAGGAGTTCAACAAGATAAAAGACGCGGTGTAACTC
AAATGGGAAATACAAACTATATTACTGAAGCTACTATTATGAGACCAGCTGAGGTTGGTTATAGTGCA
CCATATTATTCTTTTGAGGCGTCTACACAAGGGCCATTTAAAACACCTATTGCAGCAGGACGGGGGGG
AGCGCAAACAGATGAAAATCAAGCAGCAGATGGTGATCCAAGATATGCATTTGGTAGACAACATGGTC
AAAAAACTACCACAACAGGAGAAACACCTGAGAGATTTACATATATAGCACATCAAGATACAGGAAGA
TATCCAGAAGGAGATTGGATTCAAAATATTAACTTTAACCTTCCTGTAACAGAAGATAATGTATTGCT
ACCAACAGATCCAATTGGAGGTAAAACAGGAATTAACTATACTAATATATTTAATACTTATGGTCCTT
TAACTGCATTAAATAATGTACCACCAGTTTATCCAAATGGTCAAATTTGGGATAAAGAATTTGATACT
GACTTAAAACCAAGACTTCATGTAAATGCACCATTTGTTTGTCAAAATAATTGTCCTGGTCAATTATT
TGTAAAAGTTGCGCCTAATTTAACAAATGAATATGATCCTGATGCATCTGCTAATATGTCAAGAATTG
TAACTTACTCAGATTTTTGGTGGAAAGGTAAATTAGTATTTAAAGCTAAACTAAGAGCCTCTCATACT
TGGAATCCAATTCAACAAATGAGTATTAATGTAGATAACCAATTTAACTATGTACCAAGTAATATTGG
AGGTATGAAGATTGTATATGAAAAATCTCAACTAGCACCTAGA
'FRA_DQ025986_2a_04S17_2004'
ATGAGTGATGGAGCAGTTCAACCAGACGGTGGTCAACCTGCTGTCAGAAATGAAAGAGCTACAGGATC
TGGGAACGGGTCTGGAGGCGGGGGTGGTGGTGGTTCTGGGGGTGTGGGGATTTCTACGGGTACTTTCA
ATAATCAGACGGAATTTAAATTTTTGGAAAACGGATGGGTGGAAATCACAGCAAACTCAAGCAGACTT
GTACATTTAAATATGCCAGAAAGTGAAAATTATAGAAGAGTGGTTGTAAATAATTTGGATAAAACTGC
AGTTAACGGAAACATGGCTTTAGATGATACTCATGCACAAATTGTAACACCTTGGTCATTGGTTGATG
CAAATGCTTGGGGAGTTTGGTTTAATCCAGGAGATTGGCAACTAATTGTTAATACTATGAGTGAGTTG
CATTTAGTTAGTTTTGAACAAGAAATTTTTAATGTTGTTTTAAAGACTGTTTCAGAATCTGCTACTCA
GCCACCAACTAAAATTTATAATAATGATTTAACTGCATCATTGATGGTTGCATTAGATAGCAATAATA
CTATGCCATTTACTCCAGCAGCTATGAGATCTGAGACATTGGGTTTTTATCCATGGAAACCAACCATA
CCAACTCCATGGAGATATTATTTTCAATGGGATAGAACATTAATACCATCTCATACTGGAACTAGTGG
CACACCAACAAATATATACCATGGTACAGATCCAGATGATGTTCAATTTTATACTATTGAAAATTCTG
TGCCAGTACACTTACTAAGAACAGGTGATGAATTTGCTACAGGAACATTTTTTTTTGATTGTAAACCA
TGTAGACTAACACATACATGGCAAACAAATAGAGCATTGGGCTTACCACCATTTCTAAATTCTTTGCC
TCAAGCTGAAGGAGGTACTAACTTTGGTTATATAGGAGTTCAACAAGATAAAAGACGTGGTGTAACTC
AAATGGGAAATACAAACTATATTACTGAAGCTACTATTATGAGACCAGCTGAGGTTGGTTATAGTGCA
CCATATTATTCTTTTGAGGCGTCTACACAAGGGCCATTTAAAACACCTATTGCAGCAGGACGGGGGGG
AGCGCAAACAGATGAAAATCAAGCAGCAGATGGTGATCCAAGATATGCATTTGGTAGACAACATGGTC
AAAAAACTACCACAACAGGAGAAACACCTGAGAGATTTACATATATAGCACATCAAGATACAGGAAGA
TATCCAGAAGGAGATTGGATTCAAAATATTAACTTTAACCTTCCTGTAACAAATGATAATGTATTGCT
ACCAACAGATCCAATTGGAGGTAAAACAGGAATTAACTATACTAATATATTTAATACTTATGGTCCTT
TAACTGCATTAAATAATGTACCACCAGTTTATCCAAATGGTCAAATTTGGGATAAAGAATTTGATACT
GACTTAAAACCAAGACTTCATGTAAATGCACCATTTGTTTGTCAAAATAATTGTCCTGGTCAATTATT
TGTAAAAGTTGCGCCTAATTTAACAAATGAATATGATCCTGATGCATCTGCTAATATGTCAAGAATTG
TAACTTACTCAGATTTTTGGTGGAAAGGTAAATTAGTATTTAAAGCTAAACTAAGAGCCTCTCATACT

TGGAATCCAATTCAACAAATGAGTATTAATGTAGATAACCAATTTAACTATGTACCAAGTAATATTGG
AGGTATGAAGATTGTATATGAAAAATCTCAACTAGCACCTAGA
'FRA_DQ025991_2b_04S22_2004'
ATGAGTGATGGAGCAGTTCAACCAGACGGTGGTCAGCCTGCTGTCAGAAATGAAAGAGCTACAGGATC
TGGGAACGGGTCTGGAGGCGGGGGTGGTGGTGGTTCTGGGGGTGTGGGGATTTCTACGGGTACTTTCA
ATAATCAGACGGAATTTAAATTTTTGGAAAACGGATGGGTGGAAATCACAGCAAACTCAAGCAGACTT
GTACATTTAAATATGCCAGAAAGTGAAAATTATAGAAGAGTGGTTGTAAATAATTTGGATAAAACTGC
AGTTAACGGAAACATGGCTTTAGATGATACTCATGCACAAATTGTAACACCTTGGTCATTGGTTGATG
CAAATGCTTGGGGAGTTTGGTTTAATCCAGGAGATTGGCAACTAATTGTTAATACTATGAGTGAGTTG
CATTTAGTTAGTTTTGAACAAGAAATTTTTAATGTTGTTTTAAAGACTGTTTCAGAATCTGCTACTCA
GCCACCAACTAAAGTTTATAATAATGATTTAACTGCATCATTGATGGTTGCATTAGATAGTAATAATA
CTATGCCATTTACTCCAGCAGCTATGAGATCTGAGACATTGGGTTTTTATCCATGGAAACCAACCATA
CCAACTCCATGGAGATATTATTTTCAATGGGATAGAACATTAATACCATCTCATACTGGAACTAGTGG
CACACCAACAAATATATACCATGGTACAGATCCAGATGATGTTCAATTTTATACTATTGAAAATTCTG
TGCCAGTACACTTACTAAGAACAGGTGATGAATTTGCTACAGGAACATTTTTTTTTGATTGTAAACCA
TGTAGACTAACACATACATGGCAAACAAATAGAGCATTGGGCTTACCACCATTTCTAAATTCTTTGCC
TCAAGCTGAAGGAGGTACTAACTTTGGTTATATAGGAGTTCAACAAGATAAAAGACGTGGTGTAACTC
AAATGGGAAATACAAACTATATTACTGAAGCTACTATTATGAGACCAGCTGAGGTTGGTTATAGTGCA
CCATATTATTCTTTTGAGGCGTCTACACAAGGGCCATTTAAAACACCTATTGCAGCAGGACGGGGGGG
AGCGCAAACAGATGAAAATCAAGCAGCAGATGGTGATCCAAGATATGCATTTGGTAGACAACATGGTC
AAAAAACTACCACAACAGGAGAAACACCTGAGAGATTTACATATATAGCACATCAAGATACAGGAAGA
TATCCAGAAGGAGATTGGATTCAAAATATTAACTTTAACCTTCCTGTAACAGATGATAATGTATTGCT
ACCAACAGATCCAATTGGAGGTAAAACAGGAATTAACTATACTAATATATTTAATACTTATGGTCCTT
TAACTGCATTAAATAATGTACCACCAGTTTATCCAAATGGTCAAATTTGGGATAAAGAATTTGATACT
GACTTAAAACCAAGACTTCATGTAAATGCACCATTTGTTTGTCAAAATAATTGCCCTGGTCAATTATT
TGTAAAAGTTGCGCCTAATTTAACAAATGAATATGATCCTGATGCATCTGCTAATATGTCAAGAATTG
TAACTTACTCAGATTTTTGGTGGAAAGGTAAATTAGTATTTAAAGCTAAACTAAGAGCCTCTCATACT
TGGAATCCAATTCAACAAATGAGTATTAATGTAGATAACCAATTTAACTATGTACCAAGTAATATTGG
AGGTATGAAAATTGTCTATGAAAAATCTCAACTAGCACCTAGA
'FRA_DQ025992_2b_04S23_2004'
ATGAGTGATGGAGCAGTTCAACCAGACGGTGGTCAACCTGCTGTCAGAAATGAAAGAGCTACAGGATC
TGGGAACGGGTCTGGAGGCGGGGGTGGTGGTGGTTCTGGGGGTGTGGGGATTTCTACGGGTACTTTCA
ATAATCAGACGGAATTTAAATTTTTGGAAAACGGATGGGTGGAAATCACAGCAAACTCAAGCAGACTT
GTACATTTAAATATGCCAGAAAGTGAAAATTATAGAAGAGTGGTTGTAAATAATTTGGATAAAACTGC
AGTTAACGGAAACATGGCTTTAGATGATACTCATGCACAAATTGTAACACCTTGGTCATTGGTTGATG
CAAATGCTTGGGGAGTTTGGTTTAATCCAGGAGATTGGCAACTAATTGTTAATACTATGAGTGAGTTG
CATTTAGTTAGTTTTGAGCAAGAAATTTTTAATGTTGTTTTAAAGACTGTTTCAGAATCTGCTACTCA
GCCACCAACTAAAGTTTATAATAATGATTTAACTGCATCATTGATGGTTGCATTAGATAGTAATAATA
CTATGCCATTTACTCCAGCAGCTATGAGATCTGAGACATTGGGTTTTTATCCATGGAAACCAACCATA
CCAACTCCATGGAGATATTATTTTCAATGGGATAGAACATTAATACCATCTCATACTGGAACTAGTGG
CACACCAACAAATATATACCATGGTACAGATCCAGATGATGTTCAATTTTATACTATTGAAAATTCTG
TGCCAGTGCACTTACTAAGAACAGGTGATGAATTTGCTACAGGAACATTTTTTTTTGATTGTAAACCA
TGTAGACTAACACATACATGGCAAACAAATAGAGCATTGGGCTTACCACCATTTCTAAATTCTTTGCC
TCAATCTGAAGGAGGTACTAACTTTGGTTATATAGGAGTTCAACAAGATAAAAGACGTGGTGTAACTC
AAATGGGAAATACAAACTATATTACTGAAGCTACTATTATGAGACCAGCTGAGGTTGGTTATAGTGCA
CCATATTATTCTTTTGAGGCGTCTACACAAGGGCCATTTAAAACACCTATTGCAGCAGGACGGGGGGG
AGCGCAAACAGATGAAAATCAAGCAGCAGATGGTGATCCAAGATATGCATTTGGTAGACAACATGGTC
AAAAAACTACCACAACAGGAGAAACACCTGAGAGATTTACATATATAGCACATCAAGATACAGGAAGA
TATCCAGAAGGAGATTGGATTCAAAATATTAACTTTAACCTTCCTGTAACAGATGATAATGTATTGCT
ACCAACAGATCCAATTGGAGGTAAAACAGGAATTAACTATACTAATATATTTAATACTTATGGTCCTT
TAACTGCATTAAATAATGTACCACCAGTTTATCCAAATGGTCAAATTTGGGATAAAGAATTTGATACT
GACTTAAAACCAAGACTTCATGTAAATGCACCATTTGTTTGTCAAAATAATTGTCCTGGTCAATTATT
TGTAAAAGTTGCGCCTAATTTAACAAATGAATATGATCCTGATGCATCTGCTAATATGTCAAGAATTG
TAACTTACTCAGATTTTTGGTGGAAAGGTAAATTAGTATTTAAAGCTAAACTAAGAGCCTCTCATACT

TGGAATCCAATTCAACAAATGAGTATTAATGTAGATAACCAATTTAACTATGTACCAAGTAATATTGG
AGGTATGAAAATTGTATATGAAAAATCTCAACTAGCACCTAGA
'FRA_DQ025993_2a_04S24_2004'
ATGAGTGATGGAGCAGTTCAACCAGACGGTGGTCAGCCTGCTGTCAGAAATGAAAGAGCGACAGGATC
TGGGAACGGGTCTGGAGGCGGGGGTGGTGGTGGTTCTGGGGGTGTGGGGATTTCTACGGGTACTTTCA
ATAATCAGACGGAATTTAAATTTTTGGAAAACGGATGGGTGGAAATCACAGCAAACTCAAGCAGACTT
GTACATTTAAATATGCCAGAAAGTGAAAATTATAGAAGAGTGGTTGTAAATAATTTGGATAAAACTGC
AGTTAACGGAAACATGGCTTTAGATGATACTCATGCACAAATTGTAACACCTTGGTCATTGGTTGATG
CAAATGCTTGGGGAGTTTGGTTTAATCCAGGAGATTGGCAACTAATTGTTAATACTATGAGTGAGTTG
CATTTAGTTAGTTTTGAGCAAGAAATTTTTAATGTTGTTTTAAAGACTGTTTCAGAATCTGCTACTCA
GCCACCAACTAAAGTTTATAATAATGATTTAACTGCATCATTAATGGTTGCATTAGATAGCAATAATA
CTATGCCATTTACTCCAGCAGCTATGAGATCTGAGACATTGGGTTTTTATCCATGGAAACCAACCATA
CCAACTCCATGGAGATATTATTTTCAATGGGATAGAACATTAATACCATCTCATACTGGAACTAGTGG
CACACCAACAAATATATACCATGGTACAGATCCAGATGATGTTCAATTTTATACTATTGAAAATTCTG
TGCCAGTACACTTACTAAGAACAGGTGATGAATTTGCTACAGGAACATTTTTTTTTGATTGTAAACCA
TGTAGACTAACACATACATGGCAAACAAATAGAGCATTGGGCTTACCACCATTTCTAAATTCTTTGCC
TCAAGCTGAAGGAGGTACTAACTTTGGTTATATAGGAGTTCAACAAGATAAAAGACGTGGTGTAACTC
AAATGGGAAATACAAACTATATTACTGAAGCTACTATTATGAGACCAGCTGAGGTTGGTTATAGTGCA
CCATATTATTCTTTTGAGGCGTCTACACAAGGGCCATTTAAAACACCTATTGCAGCAGGACGGGGGGG
AGCGCAAACAGATGAAAATCAAGCAGCAGATGGTGATCCAAGATATGCATTTGGTAGACAACATGGTC
AAAAAACTACCACAACAGGAGAAACACCTGAGAGATTTACATATATAGCACATCAAGATACAGGAAGA
TATCCAGAAGGAGATTGGATTCAAAATATTAACTTTAACCTTCCTGTAACAAATGATAATGTATTGCT
ACCAACAGATCCAATTGGAGGTAAAACAGGAATTAACTATACTAATATATTTAATACTTATGGTCCTT
TAACTGCATTAAATAATGTACCACCAGTTTATCCAAATGGTCAAATTTGGGATAAAGAATTTGATACT
GACTTAAAACCAAGACTTCATGTAAATGCACCATTTGTTTGTCAAAATAATTGTCCTGGTCAATTATT
TGTAAAAGTTGCGCCTAATTTAACAAATGAATATGATCCTGATGCATCTGCTAATATGTCAAGAATTG
TAACTTACTCAGATTTTTGGTGGAAAGGTAAATTAGTATTCAAAGCTAAACTAAGAGCCTCTCATACT
TGGAATCCAATTCAACAAATGAGTATTAATGTAGATAACCAATTTAACTATGTACCAAGTAATATTGG
AGGTATGAAGATTGTATATGAAAAATCTCAACTAGCACCTAGA
'FRA_DQ025994_04S25_2004'
ATGAGTGATGGAGCAGTTCAACCAGACGGTGGTCAACCTGCTGTCAGAAATGAAAGAGCAACAGGATC
TGGGAACGGGTCTGGAGGCGGGGGTGGTGGTGGTTCTGGGGGTGTGGGGATTTCTACGGGTACTTTCA
ATAATCAGACGGAATTTAAATTTTTGGAAAACGGATGGGTGGAAATCACAGCAAACTCAAGCAGACTT
GTACATTTAAATATGCCAGAAAGTGAAAATTATAGAAGAGTGGTTGTAAATAATTTGGATAAAACTGC
AGTTAACGGAAACATGGCTTTAGATGATACTCATGCACAAATTGTAACACCTTGGTCATTGGTTGATG
CAAATGCTTGGGGAGTTTGGTTTAATCCAGGAGATTGGCAACTAATTGTTAATACTATGAGTGAGTTG
CATTTAGTTAGTTTTGAACAAGAAATTTTTAATGTTGTTTTAAAGACTGTTTCAGAATCTGCTACTCA
GCCACCAACTAAAGTTTATAATAATGATTTAACTGCATCATTGATGGTTGCATTAGATAGTAATAATA
CTATGCCATTTACTCCAGCAGCTATGAGATCTGAGACATTGGGTTTTTATCCATGGAAACCAACCATA
CCAACTCCATGGAGATATTATTTTCAATGGGATAGAACATTAATACCATCTCATACTGGAACTAGTGG
CACACCAACAAATATATACCATGGTACAGATCCAGATGATGTTCAATTTTATACTATTGAAAATTCTG
TGCCAGTACACTTACTAAGAACAGGTGATGAATTTGCTACAGGAACATTTTTTTTTGATTGTAAACCA
TGTAGACTAACACATACATGGCAAACAAATAGAGCATTGGGCTTACCACCATTTCTAAATTCTTTGCC
TCAAGCTGAAGGAGGTACTAACTTTGGTTATATAGGAGTTCAACAAGATAAAAGACGTGGTGTAACTC
AAATGGGAAATACAAACTATATTACTGAAGCTACTATTATGAGACCAGCTGAGGTTGGTTATAGTGCA
CCATATTATTCTTTTGAGGCGTCTACACAAGGGCCATTTAAAACACCTATTGCAGCAGGACGGGGGGG
AGCGCAAACAGATGAAAATCAAGCAGCAGATGGTGATCCAAGATATGCATTTGGTAGACAACATGGTC
AAAAAACTACCACAACAGGAGAAACGCCTGAGAGATTTACATATATAGCACATCAAGATACAGGAAGA
TATCCAGAAGGAGATTGGATTCAAAATATTAACTTTAACCTTCCTGTAACAGAAGATAATGTATTGCT
ACCAACAGATCCAATTGGAGGTAAAACAGGAATTAACTATACTAATATATTTAATACTTATGGTCCTT
TAACTGCATTAAATAATGTACCACCAGTTTATCCAAATGGTCAAATTTGGGATAAAGAATTTGATACT
GACTTAAAACCAAGACTTCATGTAAATGCACCATTTGTTTGTCAAAATAATTGTCCTGGTCAATTATT
TGTAAAAGTTGCGCCTAATTTAACAAATGAATATGATCCTGATGCATCTGCTAATATGTCAAGAATTG
TAACTTACTCAGATTTTTGGTGGAAAGGTAAATTAGTATTTAAAGCTAAACTAAGAGCCTCTCATACT

TGGAATCCAATTCAACAAATGAGTATTAATGTAGATAACCAATTTAACTATGTACCAAGTAATATTGG
AGGTATGAAAATTGTATATGAAAAATCTCAACTAGCACCTAGA

'FRA_DQ026001_2a_04S32_2004'

ATGAGTGATGGAGCAGTTCAACCAGACGGTGGTCAGCCTGCTGTCAGAAATGAAAGAGCTACAGGATC
TGGGAACGGGTCTGGAGGCGGGGGTGGTGGTGGTTCTGGGGGTGTGGGGATTTCTACGGGTACTTTCA
ATAATCAGACGGAATTTAAATTTTTGGAAAACGGATGGGTGGAAATCACAGCAAACTCAAGCAGACTT
GTACATTTAAATATGCCAGAAAGTGAAAATTATAGAAGAGTGGTTGTAAATAATTTGGATAAAACTGC
AGTTAACGGAAACATGGCTTTAGATGATACTCATGCACAAATTGTAACACCTTGGTCATTGGTTGATG
CAAATGCTTGGGGAGTTTGGTTTAATCCAGGAGATTGGCAACTAATTGTTAATACTATGAGTGAGTTG
CATTTAGTTAGTTTTGAACAAGAAATTTTTAATGTTGTTTTAAAGACTGTTTCAGAATCTGCTACTCA
GCCACCAACTAAAGTTTATAATAATGATTTAACTGCATCATTGATGGTTGCATTAGATAGCAATAATA
CTATGCCATTTACTCCAGCAGCTATGAGATCTGAGACATTGGGTTTTTATCCATGGAAACCAACCATA
CCAACTCCATGGAGATATTATTTTCAATGGGATAGAACATTAATACCATCTCATACTGGAACTAGTGG
CACACCAACAAATATATACCATGGTACAGATCCAGATGATGTTCAATTTTATACTATTGAAAATTCTG
TGCCAGTACACTTACTAAGAACAGGTGATGAATTTGCTACAGGAACATTTTTTTTTGATTGTAAACCA
TGTAGACTAACACATACATGGCAAACAAATAGAGCATTGGGCTTACCACCATTTCTAAATTCTTTGCC
TCAAGCTGAAGGAGGTACTAACTTTGGTTATATAGGAGTTCAACAAGATAAAAGACGTGGTGTAACTC
AAATGGGAAATACAAACTATATTACTGAAGCTACTATAATGAGACCAGCTGAGGTTGGTTATAGTGCA
CCATATTATTCTTTTGAGGCGTCTACACAAGGGCCATTTAAAACACCTATTGCAGCAGGACGGGGGGG
AGCGCAAACAGATGAAAATCAAGCAGCAGATGGTGATCCAAGATATGCATTTGGTAGACAACATGGTC
AAAAAACTACCACAACAGGAGAAACACCTGAGAGATTTACATATATAGCACATCAAGATACAGGAAGA
TATCCAGAAGGAGATTGGATTCAAAATATTAACTTTAACCTTCCTGTAACAAATGATAATGTATTGCT
ACCAACAGATCCAATTGGAGGTAAAACAGGAATTAACTATACTAATATATTTAATACTTATGGTCCTT
TAACTGCATTAAATAATGTACCACCAGTTTATCCAAATGGTCAAATTTGGGATAAAGAATTTGATACT
GACTTAAAACCAAGACTTCATGTAAATGCACCATTTGTTTGTCAAAATAATTGTCCTGGTCAATTATT
TGTAAAAGTTGCGCCTAATTTAACAAATGAATATGATCCTGATGCATCTGCTAATATGTCAAGAATTG
TAACTTACTCAGATTTTTGGTGGAAAGGTAAATTAGTATTTAAAGCTAAACTAAGAGCCTCTCATACT
TGGAATCCAATTCAACAAATGAGTATTAATGTAGATAACCAATTTAACTATGTACCAAGTAATATTGG
AGGTATGAAGATTGTATATGAAAAATCTCAACTAGCACCTAGA

'FRA_DQ026002_2a_04S33_2004'

ATGAGTGATGGAGCAGTTCAACCAGACGGTGGTCAACCTGCTGTCAGAAATGAAAGAGCTACAGGATC
TGGGAACGGGTCTGGAGGCGGGGGTGGTGGTGGTTCTGGGGGTGTGGGGATTTCTACGGGTACTTTCA
ATAATCAGACAGAATTTAAATTTTTGGAAAACGGATGGGTGGAAATCACAGCAAACTCAAGCAGACTT
GTACATTTAAATATGCCAGAAAGTGAAAATTATAGAAGAGTGGTTGTAAATAATTTGGATAAAACTGC
AGTTAACGGAAACATGGCTTTAGATGATACTCATGCACAAATTGTAACACCTTGGTCATTGGTTGATG
CAAATGCTTGGGGAGTTTGGTTTAATCCAGGAGATTGGCAACTAATTGTTAATACTATGAGTGAGTTG
CATTTAGTTAGTTTTGAACAAGAAATTTTTAATGTTGTTTTAAAGACTGTTTCAGAATCTGCTACTCA
GCCACCAACTAAAGTTTATAATAATGATTTAACTGCATCATTGATGGTTGCATTAGATAGCAATAATA
CTATGCCATTTACTCCAGCAGCTATGAGATCTGAGACATTGGGTTTTTATCCATGGAAACCAACCATA
CCAACTCCATGGAGATATTATTTTCAATGGGATAGAACATTAATACCATCTCATACTGGAACTAGTGG
CACACCAACAAATATATACCATGGTACAGATCCAGATGATGTTCAATTTTATACTATTGAAAATTCTG
TGCCAGTACACTTACTAAGAACAGGTGATGAATTTGCTACAGGAACATTTTTTTTTGATTGTAAACCA
TGTAGACTAACACATACATGGCAAACAAATAGAGCATTGGGCTTACCACCATTTCTAAATTCTTTGCC
TCAAGCTGAAGGAGGTACTAACTTTGGTTATATAGGAGTTCAACAAGATAAAAGACGTGGTGTAACTC
AAATGGGAAATACAAACTATATTACTGAAGCTACTATTATGAGACCAGCTGAGGTTGGTTATAGTGCA
CCATATTATTCTTTTGAGGCGTCTACACAAGGGCCATTTAAAACACCTATTGCAGCAGGACGGGGGGG
AGCGCAAACAGATGAAAATCAAGCAGCAGATGGTGATCCAAGATATGCATTTGGTAGACAACATGGTC
AAAAAACTACCACAACAGGAGAAACACCTGAGAGATTTACATATATAGCACATCAAGATACAGGAAGA
TATCCAGAAGGAGATTGGATTCAAAATATTAACTTTAACCTTCCTGTAACAAATGATAATGTATTGCT
ACCAACAGATCCAATTGGAGGTAAAACAGGAATTAACTATACTAATATATTTAATACTTATGGTCCTT
TAACTGCATTAAATAATGTACCACCAGTTTATCCAAATGGTCAAATTTGGGATAAAGAATTTGATACT
GACTTAAAACCAAGACTTCATGTAAATGCACCATTTGTTTGTCAAAATAATTGTCCTGGTCAATTATT
TGTAAAAGTTGCGCCTAATTTAACAAATGAATATGATCCTGATGCATCTGCTAATATGTCAAGAATTG
TAACTTACTCAGATTTTTGGTGGAAAGGTAAATTAGTATTTAAAGCTAAACTAAGAGCCTCTCATACT

TGGAATCCAATTCAACAAATGAGTATTAATGTAGATAACCAATTTAACTATGTACCAAGTAATATTGG
AGGTATGAAGATTGTATATGAAAAATCTCAACTAGCACCTAGA
'BRA_DQ340404_2a_BR6_1980'
ATGAGTGATGGAGCAGTTCAACCAGACGGTGGTCAACCTGCTGTCAGAAATGAAAGAGCTACAGGATC
TGGGAACGGGTCTGGAGGCGGGGGTGGTGGTGGTTCTGGGGGTGTGGGGATTTCTACGGGTACTTTCA
ATAATCAGACGGAATTTAAATTTTTGGAAAACGGATGGGTGGAAATCACAGCAAACTCAAGCAGACTT
GTACATTTAAATATGCCAGAAAGTGAAAATTATAGAAGAGTGGTTGTAAATAATTTGGATAAAACTGC
AGTTAACGGAAACATGGCTTTAGATGATACTCATGCACAAATTGTAACACCTTGGTCATTGGTTGATG
CAAATGCTTGGGGAGTTTGGTTTAATCCAGGAGATTGGCAACTAATTGTTAATACTATGAGTGAGTTG
CATTTAGTTAGTTTTGAACAAGAAATTTTTAATGTTGTTTTAAAGACTGTTTCAGAATCTGCTACTCA
GCCACCAACTAAAGTTTATAATAATGATTTAACTGCATCATTGATGGTTGCATTAGATAGTAATAATA
CTATGCCATTTACTCCAGCAGCTATGAGATCTGAGACATTGGGTTTTTATCCATGGAAACCAACCATA
CCAACTCCATGGAGATATTATTTTCAATGGGATAGAACATTAATACCATCTCATACTGGAACTAGTGG
CACACCAACAAATATATACCATGGTACAGATCCAGATGATGTTCAATTTTATACTATTGAAAATTCTG
TGCCAGTACACTTACTAAGAACAGGTGATGAATTTGCTACAGGAACATTTTTTTTTGATTGTAAACCA
TGTAGACTAACACATACATGGCAAACAAATAGAGCATTGGGCTTACCACCATTTCTAAATTCTTTGCC
TCAATCTGAAGGAGGTACTAACTTTGGTTATATAGGAGTTCAACAAGATAAAAGACGTGGTGTAACTC
AAATGGGAAATACAAACTATATTACTGAAGCTACTATTATGAGACCAGCTGAGGTTGGTTATAGTGCA
CCATATTATTCTTTTGAGGCGTCTACACAAGGGCCATTTAAAACACCTATTGCAGCAGGACGGGGGGG
AGCGCAAACAGATGAAAATCAAGCAGCAGATGGTGATCCAAGATATGCATTTGGTAGACAACATGGTC
AAAAAACTACCACAACAGGAGAAACACCTGAGAGATTTACATATATAGCACATCAAGATACAGGAAGA
TATCCAGAAGGAGATTGGATTCAAAATATTAACTTTAACCTTCCTGTAACAAATGATAATGTATTGCT
ACCAACAGATCCAATTGGAGGTAAAACAGGAATTAACTATACTAATATATTTAATACTTATGGTCCTT
TAACTGCATTAAATAATGTACCACCAGTTTATCCAAATGGTCAAATTTGGGATAAAGAATTTGATACT
GACTTAAAACCAAGACTTCATGTAAATGCACCATTTGTTTGTCAAAATAATTGTCCTGGTCAATTATT
TGTAAAAGTTGCGCCTAATTTAACAAATGAATATGATCCTGATGCATCTGCTAATATGTCAAGAATTG
TAACTTACTCAGATTTTTGGTGGAAAGGTAAATTAGTATTTAAAGCTAAACTAAGAGCCTCTCATACT
TGGAATCCAATTCAACAAATGAGTATTAATGTAGATAACCAATTTAACTATGTACCAAGTAATATTGG
AGGTATGAAAATTGTATATGAAAAATCTCAACTAGCACCTAGA
'BRA_DQ340405_2a_BR135_1980'
ATGAGTGATGGAGCAGTTCAACCAGACGGTGGTCAACCTGCTGTCAGAAATGAAAGAGCTACAGGATC
TGGGAACGGGTCTGGAGGCGGGGGTGGTGGTGGTTCTGGGGGTGTGGGGATTTCTACGGGTACTTTCA
ATAATCAGACGGAATTTAAATTTTTGGAAAACGGATGGGTGGAAATCACAGCAAACTCAAGCAGACTT
GTACATTTAAATATGCCAGAAAGTGAAAATTATAGAAGAGTGGTTGTAAATAATTTGGATAAAACTGC
AGTTAACGGAAACATGGCTTTAGATGATACTCATGCACAAATTGTAACACCTTGGTCATTGGTTGATG
CAAATGCTTGGGGAGTTTGGTTTAATCCAGGAGATTGGCAACTAATTGTTAATACTATGAGTGAGTTG
CATTTAGTTAGTTTTGAACAAGAAATTTTTAATGTTGTTTTAAAGACTGTTTCAGAATCTGCTACTCA
GCCACCAACTAAAGTTTATAATAATGATTTAACTGCATCATTGATGGTTGCATTAGATAGTAATAATA
CTATGCCATTTACTCCAGCAGCTATGAGATCTGAGACATTGGGTTTTTATCCATGGAAACCAACCATA
CCAACTCCATGGAGATATTATTTTCAATGGGATAGAACATTAATACCATCTCATACTGGAACTAGTGG
CACACCAACAAATATATACCATGGTACAGATCCAGATGATGTTCAATTTTATACTATTGAAAATTCTG
TGCCAGTACACTTACTAAGAACAGGTGATGAATTTGCTACAGGAACATTTTTTTTTGATTGTAAACCA
TGTAGACTAACACATACATGGCAAACAAATAGAGCATTGGGCTTACCACCATTTCTAAATTCTTTGCC
TCAATCTGAAGGAGGTACTAACTTTGGTTATATAGGAGTTCAACAAGATAAAAGACGTGGTGTAACTC
AAATGGGAAATACAAACTATATTACTGAAGCTACTATTATGAGACCAGCTGAGGTTGGTTATAGTGCA
CCATATTATTCTTTTGAGGCGTCTACACAAGGGCCATTTAAAACACCTATTGCAGCAGGACGGGGGGG
AGCGCAAACAGATGAAAATCAAGCAGCAGATGGTGATCCAAGATATGCATTTGGTAGACAACATGGTC
AAAAAACTACCACAACAGGAGAAACACCTGAGAGATTTACATATATAGCACATCAAGATACAGGAAGA
TATCCAGAAGGAGATTGGATTCAAAATATTAACTTTAACCTTCCTGTAACAAATGATAATGTATTGCT
ACCAACAGATCCAATTGGAGGTAAAACAGGAATTAACTATACTAATATATTTAATACTTATGGTCCTT
TAACTGCATTAAATAATGTACCACCAGTTTATCCAAATGGTCAAATTTGGGATAAAGAATTTGATACT
GACTTAAAACCAAGACTTCATGTAAATGCACCATTTGTTTGTCAAAATAATTGTCCTGGTCAATTATT
TGTAAAAGTTGCGCCTAATTTAACAAATGAATATGATCCTGATGCATCTGCTAATATGTCAAGAATTG
TAACTTACTCAGATTTTTGGTGGAAAGGTAAATTAGTATTTAAAGCTAAACTAAGAGCCTCTCATACT

TGGAATCCAATTCAACAAATGAGTATTAATGTAGATAACCAATTTAACTATGTACCAAGTAATATTGG
AGGTATGAAAATTGTATATGAAAAATCTCAACTAGCACCTAGA
'BRA_DQ340407_2a_BR145_1980'
ATGAGTGATGGAGCAGTTCAACCAGACGGTGGTCAACCTGCTGTCAGAAATGAAAGAGCTACAGGATC
TGGGAACGGGTCTGGAGGCGGGGGTGGTGGTGGTTCTGGGGGTGTGGGGATTTCTACGGGTACTTTCA
ATAATCAGACGGAATTTAAATTTTTGGAAAACGGATGGGTGGAAATCACAGCAAACTCAAGCAGACTT
GTACATTTAAATATGCCAGAAAGTGAAAATTATAGAAGAGTGGTTGTAAATAATTTGGATAAAACTGC
AGTTAACGGAAACATGGCTTTAGATGATACTCATGCACAAATTGTAACACCTTGGTCATTGGTTGATG
CAAATGCTTGGGGAGTTTGGTTTAATCCAGGAGATTGGCAACTAATTGTTAATACTATGAGTGAGTTG
CATTTAGTTAGTTTTGAACAAGAAATTTTTAATGTTGTTTTAAAGACTGTTTCAGAATCTGCTACTCA
GCCACCAACTAAAGTTTATAATAATGATTTAACTGCATCATTGATGGTTGCATTAGATAGTAATAATA
CTATGCCATTTACTCCAGCAGCTATGAGATCTGAGACATTGGGTTTTTATCCATGGAAACCAACCATA
CCAACTCCATGGAGATATTATTTTCAATGGGATAGAACATTAATACCATCTCATACTGGAACTAGTGG
CACACCAACAAATATATACCATGGTACAGATCCAGATGATGTTCAATTTTATACTATTGAAAATTCTG
TGCCAGTACACTTACTAAGAACAGGTGATGAATTTGCTACAGGAACATTTTTTTTTGATTGTAAACCA
TGTAGACTAACACATACATGGCAAACAAATAGAGCATTGGGCTTACCACCATTTCTAAATTCTTTGCC
TCAATCTGAAGGAGGTACTAACTTTGGTTATATAGGAGTTCAACAAGATAAAAGACGTGGTGTAACTC
AAATGGGAAATACAAACTATATTACTGAAGCTACTATTATGAGACCAGCTGAGGTTGGTTATAGTGCA
CCATATTATTCTTTTGAGGCGTCTACACAAGGGCCATTTAAAACACCTATTGCAGCAGGACGGGGGGG
AGCACAAACAGATGAAAATCAAGCAGCAGATGGTGATCCAAGATATGCATTTGGTAGACAACATGGTC
AAAAAACTACCACAACAGGAGAAACACCTGAGAGATTTACATATATAGCACATCAAGATACAGGAAGA
TATCCAGAAGGAGATTGGATTCAAAATATTAACTTTAACCTTCCTGTAACAAATGATAATGTATTGCT
ACCAACAGATCCAATTGGAGGTAAAACAGGAATTAACTATACTAATATATTTAATACTTATGGTCCTT
TAACTGCATTAAATAATGTACCACCAGTTTATCCAAATGGTCAAATTTGGGATAAAGAATTTGATACT
GACTTAAAACCAAGACTTCATGTAAATGCACCATTTGTTTGTCAAAATAATTGTCCTGGTCAATTATT
TGTAAAAGTTGCGCCTAATTTAACAAATGAATATGATCCTGATGCATCTGCTAATATGTCAAGAATTG
TAACTTACTCAGATTTTTGGTGGAAAGGTAAATTAGTATTTAAAGCTAAACTAAGAGCCTCTCATACT
TGGAATCCAATTCAACAAATGAGTATTAATGTAGATAACCAATTTAACTATGTACCAAGTAATATTGG
AGGTATGAAAATTGTATATGAAAAATCTCAACTAGCACCTAGA
'BRA_DQ340408_2a_BR154_1980'
ATGAGTGATGGAGCAGTTCAACCAGACGGTGGTCAACCTGCTGTCAGAAATGAAAGAGCTACAGGATC
TGGGAACGGGTCTGGAGGCGGGGGTGGTGGTGGTTCTGGGGGTGTGGGGATTTCTACGGGTACTTTCA
ATAATCAGACGGAATTTAAATTTTTGGAAAACGGATGGGTGGAAATCACAGCAAACTCAAGCAGACTT
GTACATTTAAATATGCCAGAAAGTGAAAATTATAGAAGAGTGGTTGTAAATAATTTGGATAAAACTGC
AGTTAACGGAAACATGGCTTTAGATGATACTCATGCACAAATTGTAACACCTTGGTCATTGGTTGATG
CAAATGCTTGGGGAGTTTGGTTTAATCCAGGAGATTGGCAACTAATTGTTAATACTATGAGTGAGTTG
CATTTAGTTAGTTTTGAACAAGAAATTTTTAATGTTGTTTTAAAGACTGTTTCAGAATCTGCTACTCA
GCCACCAACTAAAGTTTATAATAATGATTTAACTGCATCATTGATGGTTGCATTAGATAGTAATAATA
CTATGCCATTTACTCCAGCAGCTATGAGATCTGAGACATTGGGTTTTTATCCATGGAAACCAACCATA
CCAACTCCATGGAGATATTATTTTCAATGGGATAGAACATTAATACCATCTCATACTGGAACTAGTGG
CACACCAACAAATATATACCATGGTACAGATCCAGATGATGTTCAATTTTATACTATTGAAAATTCTG
TGCCAGTACACTTACTAAGAACAGGTGATGAATTTGCTACAGGAACATTTTTTTTTGATTGTAAACCA
TGTAGACTAACACATACATGGCAAACAAATAGAGCATTGGGCTTACCACCATTTCTAAATTCTTTGCC
TCAATCTGAAGGAGGTACTAACTTTGGTTATATAGGAGTTCAACAAGATAAAAGACGTGGTGTAACTC
AAATGGGAAATACAAACTATATTACTGAAGCTACTATTATGAGACCAGCTGAGGTTGGTTATAGTGCA
CCATATTATTCTTTTGAGGCGTCTACACAAGGGCCATTTAAAACACCTATTGCAGCAGGACGGGGGGG
AGCACAAACAGATGAAAATCAAGCAGCAGATGGTGATCCAAGATATGCATTTGGTAGACAACATGGTC
AAAAAACTACCACAACAGGAGAAACACCTGAGAGATTTACATATATAGCACATCAAGATACAGGAAGA
TATCCAGAAGGAGATTGGATTCAAAATATTAACTTTAACCTTCCTGTAACAAATGATAATGTATTGCT
ACCAACAGATCCAATTGGAGGTAAAACAGGAATTAACTATACTAATATATTTAATACTTATGGTCCTT
TAACTGCATTAAATAATGTACCACCAGTTTATCCAAATGGTCAAATTTGGGATAAAGAATTTGATACT
GACTTAAAACCAAGACTTCATGTAAATGCACCATTTGTTTGTCAAAATAATTGTCCTGGTCAATTATT
TGTAAAAGTTGCGCCTAATTTAACAAATGAATATGATCCTGATGCATCTGCTAATATGTCAAGAATTG
TAACTTACTCAGATTTTTGGTGGAAAGGTAAATTAGTATTTAAAGCTAAACTAAGAGCCTCTCATACT

TGGAATCCAATTCAACAAATGAGTATTAATGTAGATAACCAATTTAACTATGTACCAAGTAATATTGG
AGGTATGAAAATTGTATATGAAAAATCTCAACTAGCACCTAGA
'BRA_DQ340409_2b_BR183_1985'
ATGAGTGATGGAGCAGTTCAACCAGACGGTGGTCAACCTGCTGTCAGAAATGAAAGAGCTACAGGATC
TGGGAACGGGTCTGGAGGCGGGGGTGGTGGTGGTTCTGGGGGTGTGGGGATTTCTACGGGTACTTTCA
ATAATCAGACGGAATTTAAATTTTTGGAAAACGGATGGGTGGAAATCACAGCAAACTCAAGCAGACTT
GTACATTTAAATATGCCAGAAAGTGAAAATTATAGAAGAGTGGTTGTAAATAATTTGGATAAAACTGC
AGTTAACGGAAACATGGCTTTAGATGATACTCATGCACAAATTGTAACACCTTGGTCATTGGTTGATG
CAAATGCTTGGGGAGTTTGGTTTAATCCAGGAGATTGGCAACTAATTGTTAATACTATGAGTGAGTTG
CATTTAGTTAGTTTTGAACAAGAAATTTTTAATGTTGTTTTAAAGACTGTTTCAGAATCTGCTACTCA
GCCACCAACTAAAGTTTATAATAATGATTTAACTGCATCATTGATGGTTGCATTAGATAGTAATAATA
CTATGCCATTTACTCCAGCAGCTATGAGATCTGAGACATTGGGTTTTTATCCATGGAAACCAACCATA
CCAACTCCATGGAGATATTATTTTCAATGGGATAGAACATTAATACCATCTCATACTGGAACTAGTGG
CACACCAACAAATATATACCATGGTACAGATCCAGATGATGTTCAATTTTATACTATTGAAAATTCTG
TGCCAGTACACTTACTAAGAACAGGTGATGAATTTGCTACAGGAACATTTTTTTTTGATTGTAAACCA
TGTAGACTAACACATACATGGCAAACAAATAGAGCATTGGGCTTACCACCATTTCTAAATTCTTTGCC
TCAATCTGAAGGAGGTACTAACTTTGGTTATATAGGAGTTCAACAAGATAAAAGACGTGGTGTAACTC
AAATGGGAAATACAAACTATATTACTGAAGCTACTATTATGAGACCAGCTGAGGTTGGTTATAGTGCA
CCATATTATTCTTTTGAGGCGTCTACACAAGGGCCATTTAAAACACCTATTGCAGCAGGACGGGGGGG
AGCGCAAACAGATGAAAATCAAGCAGCAGATGGTGATCCAAGATATGCATTTGGTAGACAACATGGTC
AAAAAACTACCACAACAGGAGAAACACCTGAGAGATTTACATATATAGCACATCAAGATACAGGAAGA
TATCCAGAAGGAGATTGGATTCAAAATATTAACTTTAACCTTCCTGTAACAGATGATAATGTATTACT
ACCAACAGATCCAATTGGAGGTAAAACAGGAATTAACTATACTAATATATTTAATACTTATGGTCCTT
TAACTGCATTAAATAATGTACCACCAGTTTATCCAAATGGTCAAATTTGGGATAAAGAATTTGATACT
GACTTAAAACCAAGACTTCATGTAAATGCACCATTTGTTTGTCAAAATAATTGTCCTGGTCAATTATT
TGTAAAAGTTGCGCCTAATTTAACAAATGAATATGATCCTGATGCATCTGCTAATATGTCAAGAATTG
TAACTTACTCAGATTTTTGGTGGAAAGGTAAATTAGTATTTAAAGCTAAACTAAGAGCCTCTCATACT
TGGAATCCAATTCAACAAATGAGTATTAATGTAGATAACCAATTTAACTATGTACCAAGTAATATTGG
AGGTATGAAAATTGTATATGAAAAATCTCAACTAGCACCTAGA
'BRA_DQ340410_2a_BR315_1986'
ATGAGTGATGGAGCAGTTCAACCAGACGGTGGTCAACCTGCTGTCAGAAATGAAAGAGCTACAGGATC
TGGGAACGGGTCTGGAGGCGGGGGTGGTGGTGGTTCTGGGGGTGTGGGGATTTCTACGGGTACTTTCA
ATAATCAGACGGAATTTAAATTTTTGGAAAACGGATGGGTGGAAATCACAGCAAACTCAAGCAGACTT
GTACATTTAAATATGCCAGAAAGTGAAAATTATAGAAGAGTGGTTGTAAATAATTTGGATAAAACTGC
AGTTAACGGAAACATGGCTTTAGATGATACTCATGCACAAATTGTAACACCTTGGTCATTGGTTGATG
CAAATGCTTGGGGAGTTTGGTTTAATCCAGGAGATTGGCAACTAATTGTTAATACTATGAGTGAGTTG
CATTTAGTTAGTTTTGAACAAGAAATTTTTAATGTTGTTTTAAAGACTGTTTCAGAATCTGCTACTCA
GCCACCAACTAAAGTTTATAATAATGATTTAACTGCATCATTGATGGTTGCATTAGATAGTAATAATA
CTATGCCATTTACTCCAGCAGCTATGAGATCTGAGACATTGGGTTTTTATCCATGGAAACCAACCATA
CCAACTCCATGGAGATATTATTTTCAATGGGATAGAACATTAATACCATCTCATACTGGAACTGATGG
CACACCAACAAATATATACCATGGTACAGATCCAGATGATGTTCAATTTTATACTATTGAAAATTCTG
TGCCAGTACACTTACTAAGAACAGGTGATGAATTTGCTACAGGAACATTTTTTTTTGATTGTAAACCA
TGTAGACTAACACATACATGGCAGACAAATAGAGCATTGGGCTTACCACCATTTCTAAATTCTTTGCC
TCAATCTGAAGGAGGTACTAACTTTGGTTATATAGGAGTTCAACAAGATAAAAGACGTGGTGTAACTC
AAATGGGAAATACAAACTATATTACTGAAGCTACTATTATGAGACCAGCTGAGGTTGGTTATAGTGCA
CCATATTATTCTTTTGAGGCGTCTACACAAGGGCCATTTAAAACACCTATTGCAGCAGGACGGGGGGG
AGCGCAAACAGATGAAAATCAAGCAGCAGATGGTGATCCAAGATATGCATTTGGTAGACAACATGGTC
AAAAAACTACCACAACAGGAGAAACACCTGAGAGATTTACATATATAGCACATCAAGATACAGGAAGA
TATCCAGAAGGAGATTGGATTCAAAATATTAACTTTAACCTTCCTGTAACAAATGATAATGTATTGCT
ACCAACAGATCCAATTGGAGGTAAAACAGGAATTAACTATACTAATATATTTAATACTTATGGTCCTT
TAACTGCATTAAATAATGTACCACCAGTTTATCCAAATGGTCAAATTTGGGATAAAGAATTTGATACT
GACTTAAAACCAAGACTTCATGTAAATGCACCATTTGTTTGTCAAAATAATTGTCCTGGTCAATTATT
TGTAAAAGTTGCGCCTAATTTAACAAATGAATATGATCCTGATGCATCTGCTAATATGTCAAGAATTG
TAACTTACTCAGATTTTTGGTGGAAAGGTAAATTAGTATTTAAAGCTAAACTAAGAGCCTCTCATACT

TGGAATCCAATTCAACAAATGAGTATTAATGTAGATAACCAATTTAACTATGTACCAAGTAATATTGG
AGGTATGAAAATTGTATATGAAAAATCTCAACTAGCACCTAGA
'BRA_DQ340411_2a_BR8_1990'
ATGAGTGATGGAGCAGTTCAACCAGACGGTGGTCAACCTGCTGTCAGAAATGAAAGAGCTACAGGATC
TGGGAACGGGTCTGGAGGCGGGGGTGGTGGTGGTTCTGGGGGTGTGGGGATTTCTACGGGTACTTTCA
ATAATCAGACGGAATTTAAATTTTTGGAAAACGGATGGGTGGAAATCACAGCAAACTCAAGCAGACTT
GTACATTTAAATATGCCAGAAAGTGAAAATTATAGAAGAGTGGTTGTAAATAATTTGGATAAAACTGC
AGTTAATGGAAACATGGCTTTAGATGATACTCATGCACAAATTGTAACACCTTGGTCATTGGTTGATG
CAAATGCTTGGGGAGTTTGGTTTAATCCAGGAGATTGGCAACTAATTGTTAATACTATGAGTGAGTTG
CATTTAGTTAGTTTTGAACAAGAAATTTTTAATGTTGTTTTAAAGACTGTTTCAGAATCTGCTACTCA
GCCACCAACTAAAGTTTATAATAATGATTTAACTGCATCATTGATGGTTGCATTAGATAGTAATAATA
CTATGCCATTTACTCCAGCAGCTATGAGATCTGAGACATTGGGTTTTTATCCATGGAAACCAACCATA
CCAACTCCATGGAGATATTATTTTCAATGGGATAGAACATTAATACCGTCTCATACTGGAACTAGTGG
CACACCAACAAATATATACCATGGTACAGATCCAGATGATGTTCAATTTTATACTATTGAAAATTCTG
TGCCAGTACACTTACTAAGAACAGGTGATGAATTTGCTACAGGAACATTTTTTTTTGATTGTAAACCA
TGTAGACTAACACATACATGGCAAACAAATAGAGCATTGGGCTTACCACCATTTCTAAATTCTTTGCC
TCAAGCTGAAGGAGGTACTAACTTTGGTTATATAGGAGTTCAACAAGATAAAAGACGTGGTGTAACTC
AAATGGGAAATACAAACTATATTACTGAAGCTACTATTATGAGACCAGCTGAGGTTGGTTATAGTGCA
CCATATTATTCTTTTGAGGCGTCTACACAAGGGCCATTTAAAACACCTATTGCAGCAGGACGGGGGGG
AGCGCAAACAGATGAAAATCAAGCAGCAGATGGTGATCCAAGATATGCATTTGGTAGACAACATGGTC
AAAAAACTACCACAACAGGAGAAACACCTGAGAGATTCACATATATAGCACATCAAGATACAGGAAGA
TATCCAGAAGGAGATTGGATTCAAAATATTAACTTTAACCTTCCTGTAACAAATGATAATGTATTGCT
ACCAACAGATCCAATTGGAGGTAAAACAGGAATTAACTATACTAATATATTTAATACTTATGGTCCTT
TAACTGCATTAAATAATGTACCACCAGTTTATCCAAATGGTCAAATTTGGGATAAAGAATTTGATACT
GACTTAAAACCAAGACTTCATGTAAATGCACCATTTGTTTGTCAAAATAATTGTCCTGGTCAATTATT
TGTAAAAGTTGCGCCTAATTTAACAAATGAATATGATCCTGATGCATCTGCTAATATGTCAAGAATTG
TAACTTACTCAGATTTTTGGTGGAAAGGTAAATTAGTATTTAAAGCTAAACTAAGAGCCTCTCATACT
TGGAATCCAATTCAACAAATGAGTATTAATGTAGATAACCAATTTAACTATGTACCAAGTAATATTGG
AGGTATGAAAATTGTATATGAAAAATCTCAACTAGCACCTAGA
'BRA_DQ340413_2a_BR18_1990'
ATGAGTGATGGAGCAGTTCAACCAGACGGTGGTCAACCTGCTGTCAGAAATGAAAGAGCTACAGGATC
TGGGAACGGGTCTGGAGGCGGGGGTGGTGGTGGTTCTGGGGGTGTGGGGATTTCTACGGGTACTTTCA
ATAATCAGACAGAATTTAAATTTTTGGAAAACGGATGGGTGGAAATCACAGCAAACTCAAGCAGACTT
GTACATTTAAATATGCCAGAAAGTGAAAATTATAGAAGAGTGGTTGTAAATAATTTGGATAAAACTGC
AGTTAACGGAAACATGGCTTTAGATGATACTCATGCACAAATTGTAACACCTTGGTCATTGGTTGATG
CAAATGCTTGGGGAGTTTGGTTTAATCCAGGAGATTGGCAACTAATTGTTAATACTATGAGTGAGTTG
CATTTAGTTAGTTTTGAACAAGAAATTTTTAATGTTGTTTTAAAGACTGTTTCAGAATCTGCTACTCA
GCCACCAACTAAAGTTTATAATAATGATTTAACTGCATCATTGATGGTTGCATTAGATAGTAATAATA
CTATGCCATTTACTCCAGCAGCTATGAGATCTGAGACATTGGGTTTTTATCCATGGAAACCAACCATA
CCAACTCCATGGAGATATTATTTTCAATGGGATAGAACATTAATACCGTCTCATACTGGAACTAGTGG
CACACCAACAAATATATACCATGGTACAGATCCAGATGATGTTCAATTTTATACTATTGAAAATTCTG
TGCCAGTACACTTACTAAGAACAGGTGATGAATTTGCTACAGGAACATTTTTTTTTGATTGTAAACCA
TGTAGACTAACACATACATGGCAAACAAATAGAGCATTGGGCTTACCACCATTTCTAAATTCTTTGCC
TCAAGCTGAAGGAGGTACTAACTTTGGTTATATAGGAGTTCAACAAGATAAAAGACGTGGTGTAACTC
AAATGGGAAATACAAACTATATTACTGAAGCTACTATTATGAGGCCAGCTGAGGTTGGTTATAGTGCA
CCATATTATTCTTTTGAGGCGTCTACACAAGGGCCATTTAAAACACCTATTGCAGCAGGACGGGGGGG
AGCGCAAACAGATGAAAATCAAGCAGCAGATGGTGATCCAAGATATGCATTTGGTAGACAACATGGTC
AAAAAACTACCACAACAGGAGAAACACCTGAGAGATTTACATATATAGCACATCAAGATACAGGAAGA
TATCCAGAAGGAGATTGGATTCAAAATATTAACTTTAACCTTCCTGTAACAAATGATAATGTATTGCT
ACCAACAGATCCAATTGGAGGTAAAACAGGAATTAACTATACTAATATATTTAATACTTATGGTCCTT
TAACTGCATTAAATAATGTACCACCAGTTTATCCAAATGGTCAAATTTGGGATAAAGAATTTGATACT
GACTTAAAACCAAGACTTCATGTAAATGCACCATTTGTTTGTCAAAATAATTGTCCTGGTCAATTATT
TGTAAAAGTTGCGCCTAATTTAACAAATGAATATGATCCTGATGCATCTGCTAATATGTCAAGAATTG
TAACTTACTCAGATTTTTGGTGGAAAGGTAAATTAGTATTTAAAGCTAAACTAAGAGCCTCTCATACT

TGGAATCCAATTCAACAAATGAGTATTAATGTAGATAACCAATTTAACTATGTACCAAGTAATATTGG
AGGTATGAAAATTGTATATGAAAAATCTCAACTAGCACCTAGA
'BRA_DQ340414_2a_BR31_1990'
ATGAGTGATGGAGCAGTTCAACCAGACGGTGGTCAACCTGCTGTCAGAAATGAAAGAGCTACAGGATC
TGGGAACGGGTCTGGAGGCGGGGGTGGTGGTGGTTCTGGGGGTGTGGGGATTTCTACGGGTACTTTCA
ATAATCAGACAGAATTTAAATTTTTGGAAAACGGATGGGTGGAAATCACAGCAAACTCAAGCAGACTT
GTACATTTAAATATGCCAGAAAGTGAAAATTATAGAAGAGTGGTTGTAAATAATTTGGATAAAACTGC
AGTTAACGGAAACATGGCTTTAGATGATACTCATGCACAAATTGTAACACCTTGGTCATTGGTTGATG
CAAATGCTTGGGGAGTTTGGTTTAATCCAGGAGATTGGCAACTAATTGTTAATACTATGAGTGAGTTG
CATTTAGTTAGTTTTGAACAAGAAATTTTTAATGTTGTTTTAAAGACTGTTTCAGAATCTGCTACTCA
GCCACCAACTAAAGTTTATAATAATGATTTAACTGCATCATTGATGGTTGCATTAGATAGTAATAATA
CTATGCCATTTACTCCAGCAGCTATGAGATCTGAGACATTGGGTTTTTATCCATGGAAACCAACCATA
CCAACTCCATGGAGATATTATTTTCAATGGGATAGAACATTAATACCGTCTCATACTGGAACTAGTGG
CACACCAACAAATATATACCATGGTACAGATCCAGATGATGTTCAATTTTATACTATTGAAAATTCTG
TGCCAGTACACTTACTAAGAACAGGTGATGAATTTGCTACAGGAACATTTTTTTTTGATTGTAAACCA
TGTAGACTAACACATACATGGCAAACAAATAGAGCATTGGGCTTACCACCATTTCTAAATTCTTTGCC
TCAAGCTGAAGGAGGTACTAACTTTGGTTATATAGGAGTTCAACAAGATAAAAGACGTGGTGTAACTC
AAATGGGAAATACAAACTATATTACTGAAGCTACTATTATGAGACCAGCTGAGGTTGGTTATAGTGCA
CCATATTATTCTTTTGAGGCGTCTACACAAGGGCCATTTAAAACACCTATTGCAGCAGGACGGGGGGG
AGCGCAAACAGATGAAAATCAAGCAGCAGATGGTGATCCAAGATATGCATTTGGTAGACAACATGGTC
AAAAAACTACCACAACAGGAGAAACACCTGAGAGATTTACATATATAGCACATCAAGATACAGGAAGA
TATCCAGAAGGAGATTGGATTCAAAATATTAACTTTAACCTTCCTGTAACAAATGATAATGTATTGCT
ACCAACAGATCCAATTGGAGGTAAAACAGGAATTAACTATACTAATATATTTAATACTTATGGTCCTT
TAACTGCATTAAATAATGTACCACCAGTTTATCCAAATGGTCAAATTTGGGATAAAGAATTTGATACT
GACTTAAAACCAAGACTTCATGTAAATGCACCATTTGTTTGTCAAAATAATTGTCCTGGTCAATTATT
TGTAAAAGTTGCGCCTAATTTAACAAATGAATATGATCCTGATGCATCTGCTAATATGTCAAGAATTG
TAACTTACTCAGATTTTTGGTGGAAAGGTAAATTAGTATTTAAAGCTAAACTAAGAGCCTCTCATACT
TGGAATCCAATTCAACAAATGAGTATTAATGTAGATAACCAATTTAACTATGTACCAAGTAATATTGG
AGGTATGAAAATTGTATATGAAAAATCTCAACTAGCACCTAGA
'BRA_DQ340416_2a_BR47_1991'
ATGAGTGATGGAGCAGTTCAACCAGACGGTGGTCAACCTGCTGTCAGAAATGAAAGAGCTACAGGATC
TGGGAACGGGTCTGGAGGCGGGGGTGGTGGTGGTTCTGGGGGTGTGGGGATTTCTACGGGTACTTTCA
ATAATCAGACAGAATTTAAATTTTTGGAAAACGGATGGGTGGAAATCACAGCAAACTCAAGCAGACTT
GTACATTTAAATATGCCAGAAAGTGAAAATTATAGAAGAGTGGTTGTAAATAATTTGGATAAAACTGC
AGTTAACGGAAACATGGCTTTAGATGATACTCATGCACAAATTGTAACACCTTGGTCATTGGTTGATG
CAAATGCTTGGGGAGTTTGGTTTAATCCAGGAGATTGGCAACTAATTGTTAATACTATGAGTGAGTTG
CATTTAGTTAGTTTTGAACAAGAAATTTTTAATGTTGTTTTAAAGACTGTTTCAGAATCTGCTACTCA
GCCACCAACTAAAGTTTATAATAATGATTTAACTGCATCATTGATGGTTGCATTAGATAGTAATAATA
CTATGCCATTTACTCCAGCAGCTATGAGATCTGAGACATTGGGTTTTTATCCATGGAAACCAACCATA
CCAACTCCATGGAGATATTATTTTCAATGGGATAGAACATTAATACCGTCTCATACTGGAACTAGTGG
CACACCAACAAATATATACCATGGTACAGATCCAGATGATGTTCAATTTTATACTATTGAAAATTCTG
TGCCAGTACACTTACTAAGAACAGGTGATGAATTTGCTACAGGAACATTTTTTTTTGATTGTAAACCA
TGTAGACTAACACATACATGGCAAACAAATAGAGCATTGGGCTTACCACCATTTCTAAATTCTTTGCC
TCAAGCTGAAGGAGGTACTAACTTTGGTTATATAGGAGTTCAACAAGATAAAAGACGTGGTGTAACTC
AAATGGGAAATACAAACTATATTACTGAAGCTACTATTATGAGACCAGCTGAGGTTGGTTATAGTGCA
CCATATTATTCTTTTGAGGCGTCTACACAAGGGCCATTTAAAACACCTATTGCAGCAGGACGGGGGGG
AGCGCAAACAGATGAAAATCAAGCAGCAGATGGTGATCCAAGATATGCATTTGGTAGACAACATGGTC
AAAAAACTACCACAACAGGAGAAACACCTGAGAGATTTACATATATAGCACATCAAGATACAGGAAGA
TATCCAGAAGGAGATTGGATTCAAAATATTAACTTTAACCTTCCTGTAACAAATGATAATGTATTGCT
ACCAACAGATCCAATTGGAGGTAAAACAGGAATTAACTATACTAATATATTTAATACTTATGGTCCTT
TAACTGCATTAAATAATGTACCACCAGTTTATCCAAATGGTCAAATTTGGGATAAAGAATTTGATACT
GACTTAAAACCAAGACTTCATGTAAATGCACCATTTGTTTGTCAAAATAATTGTCCTGGTCAATTATT
TGTAAAAGTTGCGCCTAATTTAACAAATGAATATGATCCTGATGCATCTGCTAATATGTCAAGAATTG
TAACTTACTCAGATTTTTGGTGGAAAGGTAAATTAGTATTTAAAGCTAAACTAAGAGCCTCTCATACT

TGGAATCCAATTCAACAAATGAGTATTAATGTAGATAACCAATTTAACTATGTACCAAGTAATATTGG
AGGTATGAAAATTGTATATGAAAAATCTCAACTAGCACCTAGA
'BRA_DQ340417_2a_BR52_1991'
ATGAGTGATGGAGCAGTTCAACCAGACGGTGGTCAACCTGCTGTCAGAAATGAAAGAGCTACAGGATC
TGGGAACGGGTCTGGAGGCGGGGGTGGTGGTGGTTCTGGGGGTGTGGGGATTTCTACGGGTACTTTCA
ATAATCAGACAGAATTTAAATTTTTGGAAAACGGATGGGTGGAAATCACAGCAAACTCAAGCAGACTT
GTACATTTAAATATGCCAGAAAGTGAAAATTATAGAAGAGTGGTTGTAAATAATTTGGATAAAACTGC
AGTTAACGGAAACATGGCTTTAGATGATACTCATGCACAAATTGTAACACCTTGGTCATTGGTTGATG
CAAATGCTTGGGGAGTTTGGTTTAATCCAGGAGATTGGCAACTAATTGTTAATACTATGAGTGAGTTG
CATTTAGTTAGTTTTGAACAAGAAATTTTTAATGTTGTTTTAAAGACTGTTTCAGAATCTGCTACTCA
GCCACCAACTAAAGTTTATAATAATGATTTAACTGCATCATTGATGGTTGCATTAGATAGTAATAATA
CTATGCCATTTACTCCAGCAGCTATGAGATCTGAGACATTGGGTTTTTATCCATGGAAACCAACCATA
CCAACTCCATGGAGATATTATTTTCAATGGGATAGAACATTAATACCGTCTCATACTGGAACTAGTGG
CACACCAACAAATATATACCATGGTACAGATCCAGATGATGTTCAATTTTATACTATTGAAAATTCTG
TGCCAGTACACTTACTAAGAACAGGTGATGAATTTGCTACAGGAACATTTTTTTTTGATTGTAAACCA
TGTAGACTAACACATACATGGCAAACAAATAGAGCATTGGGCTTACCACCATTTCTAAATTCTTTGCC
TCAAGCTGAAGGAGGTACTAACTTTGGTTATATAGGAGTTCAACAAGATAAAAGACGTGGTGTAACTC
AAATGGGAAATACAAACTATATTACTGAAGCTACTATTATGAGACCAGCTGAGGTTGGTTATAGTGCA
CCATATTATTCTTTTGAGGCGTCTACACAAGGGCCATTTAAAACACCTATTGCAGCAGGACGGGGGGG
AGCGCAAACAGATGAAAATCAAGCAGCAGATGGTGATCCAAGATATGCATTTGGTAGACAACATGGTC
AAAAAACTACCACAACAGGAGAAACACCTGAGAGATTTACATATATAGCACATCAAGATACAGGAAGA
TATCCAGAAGGAGATTGGATTCAAAATATTAACTTTAACCTTCCTGTAACAAATGATAATGTATTGCT
ACCAACAGATCCAATTGGAGGTAAAACAGGAATTAACTATACTAATATATTTAATACTTATGGTCCTT
TAACTGCATTAAATAATGTACCACCAGTTTATCCAAATGGTCAAATTTGGGATAAAGAATTTGATACT
GACTTAAAACCAAGACTTCATGTAAATGCACCATTTGTTTGTCAAAATAATTGTCCTGGTCAATTATT
TGTAAAAGTTGCGCCTAATTTAACAAATGAATATGATCCTGATGCATCTGCTAATATGTCAAGAATTG
TAACTTACTCAGATTTTTGGTGGAAAGGTAAATTAGTATTTAAAGCTAAACTAAGAGCCTCTCATACT
TGGAATCCAATTCAACAAATGAGTATTAATGTAGATAACCAATTTAACTATGTACCAAGTAATATTGG
AGGTATGAAAATTGTATATGAAAAATCTCAACTAGCACCTAGA
'BRA_DQ340418_2a_BR491_1992'
ATGAGTGATGGAGCAGTTCAACCAGACGGTGGTCAACCTGCTGTCAGAAATGAAAGAGCTACAGGATC
TGGGAACGGGTCTGGAGGCGGGGGTGGTGGTGGTTCTGGGGGTGTGGGGATTTCTACGGGTACTTTCA
ATAATCAGACAGAATTTAAATTTTTGGAAAACGGATGGGTGGAAATCACAGCAAACTCAAGCAGACTT
GTACATTTAAATATGCCAGAAAGTGAAAATTATAGAAGAGTGGTTGTAAATAATTTGGATAAAACTGC
AGTTAACGGAAACATGGCTTTAGATGATACTCATGCACAAATTGTAACACCTTGGTCATTGGTTGATG
CAAATGCTTGGGGAGTTTGGTTTAATCCAGGAGATTGGCAACTAATTGTTAATACTATGAGTGAGTTG
CATTTAGTTAGTTTTGAACAAGAAATTTTTAATGTTGTTTTAAAGACTGTTTCAGAATCTGCTACTCA
GCCACCAACTAAAGTTTATAATAATGATTTAACTGCATCATTGATGGTTGCATTAGATAGTAATAATA
CTATGCCATTTACTCCAGCAGCTATGAGATCTGAGACATTGGGTTTTTATCCATGGAAACCAACCATA
CCAACTCCATGGAGATATTATTTTCAATGGGATAGAACATTAATACCGTCTCATACTGGAACTAGTGG
CACACCAACAAATATATACCATGGTACAGATCCAGATGATGTTCAATTTTATACTATTGAAAATTCTG
TGCCAGTACACTTACTAAGAACAGGTGATGAATTTGCTACAGGAACATTTTTTTTTGATTGTAAACCA
TGTAGACTAACACATACATGGCAAACAAATAGAGCATTGGGCTTACCACCATTTCTAAATTCTTTGCC
TCAAGCTGAAGGAGGTACTAACTTTGGTTATATAGGAGTTCAACAAGATAAAAGACGTGGTGTAACTC
AAATGGGAAATACAAACTATATTACTGAAGCTACTATTATGAGACCAGCTGAGGTTGGTTATAGTGCA
CCATATTATTCTTTTGAGGCGTCTACACAAGGGCCATTTAAAACACCTATTGCAGCAGGACGGGGGGG
AGCGCAAACAGATGAAAATCAAGCAGCAGATGGTGATCCAAGATATGCATTTGGTAGACAACATGGTC
AAAAAACTACCACAACAGGAGAAACACCTGAGAGATTTACATATATAGCACATCAAGATACAGGAAGA
TATCCAGAAGGAGATTGGATTCAAAATATTAACTTTAACCTTCCTGTAACAAATGATAATGTATTGCT
ACCAACAGATCCAATTGGAGGTAAAACAGGAATTAACTATACTAATATATTTAATACTTATGGTCCTT
TAACTGCATTAAATAATGTACCACCAGTTTATCCAAATGGTCAAATTTGGGATAAAGAATTTGATACT
GACTTAAAACCAAGACTTCATGTAAATGCACCATTTGTTTGTCAAAATAATTGTCCTGGTCAATTATT
TGTAAAAGTTGCGCCTAATTTAACAAATGAATATGATCCTGATGCATCTGCTAATATGTCAAGAATTG
TAACTTACTCAGATTTTTGGTGGAAAGGTAAATTAGTATTTAAAGCTAAACTAAGAGCCTCTCATACT

TGGAATCCAATTCAACAAATGAGTATTAATGTAGATAACCAATTTAACTATGTACCAAGTAATATTGG
AGGTATGAAAATTGTATATGAAAAATCTCAACTAGCACCTAGA
'BRA_DQ340419_2a_BR570_1992'
ATGAGTGATGGAGCAGTTCAACCAGACGGTGGTCAACCTGCTGTCAGAAATGAAAGAGCTACAGGATC
TGGGAACGGGTCTGGAGGCGGGGGTGGTGGTGGTTCTGGGGGTGTGGGGATTTCTACGGGTACTTTCA
ATAATCAGACAGAATTTAAATTTTTGGAAAACGGATGGGTGGAAATCACAGCAAACTCAAGCAGACTT
GTACATTTAAATATGCCAGAAAGTGAAAATTATAGAAGAGTGGTTGTAAATAATTTGGATAAAACTGC
AGTTAACGGAAACATGGCTTTAGATGATACTCATGCACAAATTGTAACACCTTGGTCATTGGTTGATG
CAAATGCTTGGGGAGTTTGGTTTAATCCAGGAGATTGGCAACTAATTGTTAATACTATGAGTGAGTTG
CATTTAGTTAGTTTTGAACAAGAAATTTTTAATGTTGTTTTAAAGACTGTTTCAGAATCTGCTACTCA
GCCACCAACTAAAGTTTATAATAATGATTTAACTGCATCATTGATGGTTGCATTAGATAGTAATAATA
CTATGCCATTTACTCCAGCAGCTATGAGATCTGAGACATTGGGTTTTTATCCATGGAAACCAACCATA
CCAACTCCATGGAGATATTATTTTCAATGGGATAGAACATTAATACCGTCTCATACTGGAACTAGTGG
CACACCAACAAATATATACCATGGTACAGATCCAGATGATGTTCAATTTTATACTATTGAAAATTCTG
TGCCAGTACACTTACTAAGAACAGGTGATGAATTTGCTACAGGAACATTTTTTTTTGATTGTAAACCA
TGTAGACTAACACATACATGGCAAACAAATAGAGCATTGGGCTTACCACCATTTCTAAATTCTTTGCC
TCAAGCTGAAGGAGGTACTAACTTTGGTTATATAGGAGTTCAACAAGATAAAAGACGTGGTGTAACTC
AAATGGGAAATACAAACTATATTACTGAAGCTACTATTATGAGACCAGCTGAGGTTGGTTATAGTGCA
CCATATTATTCTTTTGAGGCGTCTACACAAGGGCCATTTAAAACACCTATTGCAGCAGGACGGGGGGG
AGCGCAAACAGATGAAAATCAAGCAGCAGATGGTGATCCAAGATATGCATTTGGTAGACAACATGGTC
AAAAAACTACCACAACAGGAGAAACACCTGAGAGATTTACATATATAGCACATCAAGATACAGGAAGA
TATCCAGAAGGAGATTGGATTCAAAATATAAACTTTAACCTTCCTGTAACAAATGATAATGTATTGCT
ACCAACAGATCCAATTGGAGGTAAAACAGGAATTAACTATACTAATATATTTAATACTTATGGTCCTT
TAACTGCATTAAATAATGTACCACCAGTTTATCCAAATGGTCAAATTTGGGATAAAGAATTTGATACT
GACTTAAAACCAAGACTTCATGTAAATGCACCATTTGTTTGTCAAAATAATTGTCCTGGTCAATTATT
TGTAAAAGTTGCGCCTAATTTAACAAATGAATATGATCCTGATGCATCTGCTAATATGTCAAGAATTG
TAACTTACTCAGATTTTTGGTGGAAAGGTAAATTAGTATTTAAAGCTAAACTAAGAGCCTCTCATACT
TGGAATCCAATTCAACAAATGAGTATTAATGTAGATAACCAATTTAACTATGTACCAAGTAATATTGG
AGGTATGAAAATTGTATATGAAAAATCTCAACTAGCACCTAGA
'BRA_DQ340421_2a_BR597_1992'
ATGAGTGATGGAGCAGTTCAACCAGACGGTGGTCAACCTGCTGTCAGAAATGAAAGAGCTACAGGATC
TGGGAACGGGTCTGGAGGCGGGGGTGGTGGTGGTTCTGGGGGTGTGGGGATTTCTACGGGTACTTTCA
ATAATCAGACAGAATTTAAATTTTTGGAAAACGGATGGGTGGAAATCACAGCAAACTCAAGCAGACTT
GTACATTTAAATATGCCAGAAAGTGAAAATTATAGAAGAGTGGTTGTAAATAATTTGGATAAAACTGC
AGTTAACGGAAACATGGCTTTAGATGATACTCATGCACAAATTGTAACACCTTGGTCATTGGTTGATG
CAAATGCTTGGGGAGTTTGGTTTAATCCAGGAGATTGGCAACTAATTGTTAATACTATGAGTGAGTTG
CATTTAGTTAGTTTTGAACAAGAAATTTTTAATGTTGTTTTAAAGACTGTTTCAGAATCTGCTACTCA
GCCACCAACTAAAGTTTATAATAATGATTTAACTGCATCATTGATGGTTGCATTAGATAGTAATAATA
CTATGCCATTTACTCCAGCAGCTATGAGATCTGAGACATTGGGTTTTTATCCATGGAAACCAACCATA
CCAACTCCATGGAGATATTATTTTCAATGGGATAGGACATTAATACCGTCTCATACTGGAACTAGTGG
CACACCAACAAATATATACCATGGTACAGATCCAGATGATGTTCAATTTTATACTATTGAAAATTCTG
TGCCAGTACACTTACTAAGAACAGGTGATGAATTTGCTACAGGAACATTTTTTTTTGATTGTAAACCA
TGTAGACTAACACATACATGGCAAACAAATAGAGCATTGGGCTTACCACCATTTCTAAATTCTTTGCC
TCAAGCTGAAGGAGGTACTAACTTTGGTTATATAGGAGTTCAACAAGATAAAAGACGTGGTGTAACTC
AAATGGGAAATACAAACTATATTACTGAAGCTACTATTATGAGACCAGCTGAGGTTGGTTATAGTGCA
CCATATTATTCTTTTGAGGCGTCTACACAAGGGCCATTTAAAACACCTATTGCAGCAGGACGGGGGGG
AGCGCAAACAGATGAAAATCAAGCAGCAGATGGTGATCCAAGATATGCATTTGGTAGACAACATGGTC
AAAAAACTACCACAACAGGAGAAACACCTGAGAGATTTACATATATAGCACATCAAGATACAGGAAGA
TATCCAGAAGGAGATTGGATTCAAAATATTAACTTTAACCTTCCTGTAACAAATGATAATGTATTGCT
ACCAACAGATCCAATTGGAGGTAAAACAGGAATTAACTATACTAATATATTTAATACTTATGGTCCTT
TAACTGCATTAAATAATGTACCACCAGTTTATCCAAATGGTCAAATTTGGGATAAAGAATTTGATACT
GACTTAAAACCAAGACTTCATGTAAATGCACCATTTGTTTGTCAAAATAATTGTCCTGGTCAATTATT
TGTAAAAGTTGCGCCTAATTTAACAAATGAATATGATCCTGATGCATCTGCTAATATGTCAAGAATTG
TAACTTACTCAGATTTTTGGTGGAAAGGTAAATTAGTATTTAAAGCTAAACTAAGAGCCTCTCATACT

TGGAATCCAATTCAACAAATGAGTATTAATGTAGATAACCAATTTAACTATGTACCAAGTAATATTGG
AGGTATGAAAATTGTATATGAAAAATCTCAACTAGCACCTAGA
'BRA_DQ340422_2a_BR22_1993'
ATGAGTGATGGAGCAGTTCAACCAGACGGTGGTCAACCTGCTGTCAGAAATGAAAGAGCTACAGGATC
TGGGAACGGGTCTGGAGGCGGGGGTGGTGGTGGTTCTGGGGGTGTGGGGATTTCTACGGGTACTTTCA
ATAATCAGACAGAATTTAAATTTTTGGAAAACGGATGGGTGGAAATCACAGCAAACTCAAGCAGACTT
GTACATTTAAATATGCCAGAAAGTGAAAATTATAGAAGAGTGGTTGTAAATAATTTGGATAAAACTGC
AGTTAACGGAAACATGGCTTTAGATGATACTCATGCACAAATTGTAACACCTTGGTCATTGGTTGATG
CAAATGCTTGGGGAGTTTGGTTTAATCCAGGAGATTGGCAACTAATTGTTAATACTATGAGTGAGTTG
CATTTAGTTAGTTTTGAACAAGAAATTTTTAATGTTGTTTTAAAGACTGTTTCAGAATCTGCTACTCA
GCCACCAACTAAAGTTTATAATAATGATTTAACTGCATCATTGATGGTTGCATTAGATAGTAATAATA
CTATGCCATTTACTCCAGCAGCTATGAGATCTGAGACATTGGGTTTTTATCCATGGAAACCAACCATA
CCAACTCCATGGAGATATTATTTTCAATGGGATAGAACATTAATACCGTCTCATACTGGAACTAGTGG
CACACCAACAAATATATACCATGGTACAGATCCAGATGATGTTCAATTTTATACTATTGAAAATTCTG
TGCCAGTACACTTACTAAGAACAGGTGATGAATTTGCTACAGGAACATTTTTTTTTGATTGTAAACCA
TGTAGACTAACACATACATGGCAAACAAATAGAGCATTGGGCTTACCACCATTTCTAAATTCTTTGCC
TCAAGCTGAAGGAGGTACTAACTTTGGTTATATAGGAGTTCAACAAGATAAAAGACGTGGTGTAACTC
AAATGGGAAATACAAACTATATTACTGAAGCTACTATTATGAGACCAGCTGAGGTTGGTTATAGTGCA
CCATATTATTCTTTTGAGGCGTCTACACAAGGGCCATTTAAAACACCTATTGCAGCAGGACGGGGGGG
AGCGCAAACAGATGAAAATCAAGCAGCAGATGGTGATCCAAGATATGCATTTGGTAGACAACATGGTC
AAAAAACTACCACAACAGGAGAAACACCTGAGAGATTTACATATATAGCACATCAAGATACAGGAAGA
TATCCAGAAGGAGATTGGATTCAAAATATTAACTTTAACCTTCCTGTAACAAATGATAATGTATTGCT
ACCAACAGATCCAATTGGAGGTAAAGCAGGAATTAACTATACTAATATATTTAATACTTATGGTCCTT
TAACTGCATTAAATAATGTACCACCAGTTTATCCAAATGGTCAAATTTGGGATAAAGAATTTGATACT
GACTTAAAACCAAGACTTCATGTAAATGCACCATTTGTTTGTCAAAATAATTGTCCTGGTCAATTATT
TGTAAAAGTTGCGCCTAATTTAACAAATGAATATGATCCTGATGCATCTGCTAATATGTCAAGAATTG
TAACTTACTCAGATTTTTGGTGGAAAGGTAAATTAGTATTTAAAGCTAAACTAAGAGCCTCTCATACT
TGGAATCCAATTCAACAAATGAGTATTAATGTAGATAACCAATTTAACTATGTACCAAGTAATATTGG
AGGTATGAAAATTGTATATGAAAAATCTCAACTAGCACCTAGA
'BRA_DQ340423_2a_BR136_1993'
ATGAGTGATGGAGCAGTTCAACCAGACGGTGGTCAACCTGCTGTCAGAAATGAAAGAGCTACAGGATC
TGGGAACGGGTCTGGAGGCGGGGGTGGTGGTGGTTCTGGGGGTGTGGGGATTTCTACGGGTACTTTCA
ATAATCAGACAGAATTTAAATTTTTGGAAAACGGATGGGTGGAAATCACAGCAAACTCAAGCAGACTT
GTACATTTAAATATGCCAGAAAGTGAAAATTATAGAAGAGTGGTTGTAAATAATTTGGATAAAACTGC
AGTTAACGGAAACATGGCTTTAGATGATACTCATGCACAAATTGTAACACCTTGGTCATTGGTTGATG
CAAATGCTTGGGGAGTTTGGTTTAATCCAGGAGATTGGCAACTAATTGTTAATACTATGAGTGAGTTG
CATTTAGTTAGTTTTGAACAAGAAATTTTTAATGTTGTTTTAAAGACTGTTTCAGAATCTGCTACTCA
GCCACCAACTAAAGTTTATAATAATGATTTAACTGCATCATTGATGGTTGCATTAGATAGTAATAATA
CTATGCCATTTACTCCAGCAGCTATGAGATCTGAGACATTGGGTTTTTATCCATGGAAACCAACCATA
CCAACTCCATGGAGATATTATTTTCAATGGGATAGAACATTAATACCGTCTCATACTGGAACTAGTGG
CACACCAACAAATATATACCATGGTACAGATCCAGATGATGTTCAATTTTATACTATTGAAAATTCTG
TGCCAGTACACTTACTAAGAACAGGTGATGAATTTGCTACAGGAACATTTTTTTTTGATTGTAAACCA
TGTAGACTAACACATACATGGCAAACAAATAGAGCATTGGGCTTACCACCATTTCTAAATTCTTTGCC
TCAAGCTGAAGGAGGTACTAACTTTGGTTATATAGGAGTTCAACAAGATAAAAGACGTGGCGTAACTC
AAATGGGAAATACAAACTATATTACTGAAGCTACTATTATGAGACCAGCTGAGGTTGGTTATAGTGCA
CCATATTATTCTTTTGAGGCGTCTACACAAGGGCCATTTAAAACACCTATTGCAGCAGGACGGGGGGG
AGCGCAAACAGATGAAAATCAAGCAGCAGATGGTGATCCAAGATATGCATTTGGTAGACAACATGGTC
AAAAAACTACCACAACAGGAGAAACACCTGAGAGATTTACATATATAGCACATCAAGATACAGGAAGA
TATCCAGAAGGAGATTGGATTCAAAATATTAACTTTAACCTTCCTGTAACAAATGATAATGTATTGCT
ACCAACAGATCCAATTGGAGGTAAAACAGGAATTAACTATACTAATATATTTAATACTTATGGTCCTT
TAACTGCATTAAATAATGTACCACCAGTTTATCCAAATGGTCAAATTTGGGATAAAGAATTTGATACT
GACTTAAAACCAAGACTTCATGTAAATGCACCATTTGTTTGTCAAAATAATTGTCCTGGTCAATTATT
TGTAAAAGTTGCGCCTAATTTAACAAATGAATATGATCCTGATGCATCTGCTAATATGTCAAGAATTG
TAACTTACTCAGATTTTTGGTGGAAAGGTAAATTAGTATTTAAAGCTAAACTAAGAGCCTCTCATACT

TGGAATCCAATTCAACAAATGAGTATTAATGTAGATAACCAATTTAACTATGTACCAAGTAATATTGG
AGGTATGAAAATTGTATATGAAAAATCTCAACTAGCACCTAGA
'BRA_DQ340424_2a_BR137_1993'
ATGAGTGATGGAGCAGTTCAACCAGACGGTGGTCAACCTGCTGTCAGAAATGAAAGAGCTACAGGATC
TGGGAACGGGTCTGGAGGCGGGGGTGGTGGTGGTTCTGGGGGTGTGGGGATTTCTACGGGTACTTTCA
ATAATCAGACAGAATTTAAATTTTTGGAAAACGGATGGGTGGAAATCACAGCAAACTCAAGCAGACTT
GTACATTTAAATATGCCAGAAAGTGAAAATTATAGAAGAGTGGTTGTAAATAATTTGGATAAAACTGC
AGTTAACGGAAACATGGCTTTAGATGATACTCATGCACAAATTGTAACACCTTGGTCATTGGTTGATG
CAAATGCTTGGGGAGTTTGGTTTAATCCAGGAGATTGGCAACTAATTGTTAATACTATGAGTGAGTTG
CATTTAGTTAGTTTTGAACAAGAAATTTTTAATGTTGTTTTAAAGACTGTTTCAGAATCTGCTACTCA
GCCACCAACTAAAGTTTATAATAATGATTTAACTGCATCATTGATGGTTGCATTAGATAGTAATAATA
CTATGCCATTTACTCCAGCAGCTATGAGATCTGAGACATTGGGTTTTTATCCATGGAAACCAACCATA
CCAACTCCATGGAGATATTATTTTCAATGGGATAGAACATTAATACCGTCTCATACTGGAACTAGTGG
CACACCAACAAATATATACCATGGTACAGATCCAGATGATGTTCAATTTTATACTATTGAAAATTCTG
TGCCAGTACACTTACTAAGAACAGGTGATGAATTTGCTACAGGAACATTTTTTTTTGATTGTAAACCA
TGTAGACTAACACATACATGGCAAACAAATAGAGCATTGGGCTTACCACCATTTCTAAATTCTTTGCC
TCAAGCTGAAGGAGGTACTAACTTTGGTTATATAGGAGTTCAACAAGATAAAAGACGTGGTGTAACTC
AAATGGGAAATACAAACTATATTACTGAAGCTACTATTATGAGACCAGCTGAGGTTGGTTATAGTGCA
CCATATTATTCTTTTGAGGCGTCTACACAAGGGCCATTTAAAACACCTATTGCAGCAGGACGGGGGGG
AGCGCAAACAGATGAAAATCAAGCAGCAGATGGTGATCCAAGATATGCATTTGGTAGACAACATGGTC
AAAAAACTACCACAACAGGAGAAACACCTGAGAGATTTACATATATAGCACATCAAGATACAGGAAGA
TATCCAGAAGGAGATTGGATTCAAAATATTAACTTTAACCTTCCTGTAACAAATGATAATGTATTGCT
ACCAACAGATCCAATTGGAGGTAAAACAGGAATTAACTATACTAATATATTTAATACTTATGGTCCTT
TAACTGCATTAAATAATGTACCACCAGTTTATCCAAATGGTCAAATTTGGGATAAAGAATTTGATACT
GACTTAAAACCAAGACTTCATGTAAATGCACCATTTGTTTGTCAAAATAATTGTCCTGGTCAATTATT
TGTAAAAGTTGCGCCTAATTTAACAAATGAATATGATCCTGATGCATCTGCTAATATGTCAAGAATTG
TAACTTACTCAGATTTTTGGTGGAAAGGTAAATTAGTATTTAAAGCTAAACTAAGAGCCTCTCATACT
TGGAATCCAATTCAACAAATGAGTATTAATGTAGATAACCAATTTAACTATGTACCAAGTAATATTGG
AGGTATGAAAATTGTATATGAAAAATCTCAACTAGCACCTAGA
'BRA_DQ340426_2a_BR84_1994'
ATGAGTGATGGAGCAGTTCAACCAGACGGTGGTCAACCTGCTGTCAGAAATGAAAGAGCTACAGGATC
TGGGAACGGGTCTGGAGGCGGGGGTGGTGGTGGTTCTGGGGGTGTGGGGATTTCTACGGGTACTTTCA
ATAATCAGACAGAATTTAAATTTTTGGAAAACGGATGGGTGGAAATCACAGCAAACTCAAGCAGACTT
GTACATTTAAATATGCCAGAAAGTGAAAATTATAGAAGAGTGGTTGTAAATAATTTGGATAAAACTGC
AGTTAACGGAAACATGGCTTTAGATGATACTCATGCACAAATTGTAACACCTTGGTCATTGGTTGATG
CAAATGCTTGGGGAGTTTGGTTTAATCCAGGAGATTGGCAACTAATTGTTAATACTATGAGTGAGTTG
CATTTAGTTAGTTTTGAACAAGAAATTTTTAATGTTGTTTTAAAGACTGTTTCAGAATCTGCTACTCA
GCCACCAACTAAAGTTTATAATAATGATTTAACTGCATCATTGATGGTTGCATTAGATAGTAATAATA
CTATGCCATTTACTCCAGCAGCTATGAGATCTGAGACATTGGGTTTTTATCCATGGAAACCAACCATA
CCAACTCCATGGAGATATTATTTTCAATGGGATAGAACATTAATACCGTCTCATACTGGAACTAGTGG
CACACCAACAAATATATACCATGGTACAGATCCAGATGATGTTCAATTTTATACTATTGAAAATTCTG
TGCCAGTACACTTACTAAGAACAGGTGATGAATTTGCTACAGGAACATTTTTTTTTGATTGTAAACCA
TGTAGACTAACACATACATGGCAAACAAATAGAGCATTGGGCTTACCACCATTTCTAAATTCTTTGCC
TCAAGCTGAAGGAGGTACTAACTTTGGTTATATAGGAGTTCAACAAGATAAAAGACGTGGTGTAACTC
AAATGGGAAATACAAACTATATTACTGAAGCTACTATTATGAGACCAGCTGAGGTTGGTTATAGTGCA
CCATATTATTCTTTTGAGGCGTCTACACAAGGGCCATTTAAAACACCTATTGCAGCAGGACGGGGGGG
AGCGCAAACAGATGAAAATCAAGCAGCAGATGGTGATCCAAGATATGCATTTGGTAGACAACATGGTC
AAAAAACTACCACAACAGGAGAAACACCTGAGAGATTTACATATATAGCACATCAAGATACAGGAAGA
TATCCAGAAGGAGATTGGATTCAAAATATTAACTTTAACCTTCCTGTAACAAATGATAATGTATTGCT
ACCAACAGATCCAATTGGAGGTAAAACAGGAATTAACTATACTAATATATTTAATACTTATGGTCCTT
TAACTGCATTAAATAATGTACCACCAGTTTATCCAAATGGTCAAATTTGGGATAAAGAATTTGATACT
GACTTAAAACCAAGACTTCATGTAAATGCACCATTTGTTTGTCAAAATAATTGTCCTGGTCAATTATT
TGTAAAAGTTGCGCCTAATTTAACAAATGAATATGATCCTGATGCATCTGCTAATATGTCAAGAATTG
TAACTTACTCAGATTTTTGGTGGAAAGGTAAATTAGTATTTAAAGCTAAACTAAGAGCCTCTCATACT

TGGAATCCAATTCAACAAATGAGTATTAATGTAGATAACCAATTTAACTATGTACCAAGTAATATTGG
AGGTATGAAAATTGTATATGAAAAATCTCAACTAGCACCTAGA
'BRA_DQ340427_2a_BR133_1994'
ATGAGTGATGGAGCAGTTCAACCAGACGGTGGTCAACCTGCTGTCAGAAATGAAAGAGCTACAGGATC
TGGGAACGGGTCTGGAGGCGGGGGTGGTGGTGGTTCTGGGGGTGTGGGGATTTCTACGGGTACTTTCA
ATAATCAGACAGAATTTAAATTTTTGGAAAACGGATGGGTGGAAATCACAGCAAACTCAAGCAGACTT
GTACATTTAAATATGCCAGAAAGTGAAAATTATAGAAGAGTGGTTGTAAATAATTTGGATAAAACTGC
AGTTAACGGAAACATGGCTTTAGATGATACTCATGCACAAATTGTAACACCTTGGTCATTGGTTGATG
CAAATGCTTGGGGAGTTTGGTTTAATCCAGGAGATTGGCAACTAATTGTTAATACTATGAGTGAGTTG
CATTTAGTTAGTTTTGAACAAGAAATTTTTAATGTTGTTTTAAAGACTGTTTCAGAATCTGCTACTCA
GCCACCAACTAAAGTTTATAATAATGATTTAACTGCATCATTGATGGTTGCATTAGATAGTAATAATA
CTATGCCATTTACTCCAGCAGCTATGAGATCTGAGACATTGGGTTTTTATCCATGGAAACCAACCATA
CCAACTCCATGGAGATATTATTTTCAATGGGATAGAACATTAATACCGTCTCATACTGGAACTAGTGG
CACACCAACAAATATATACCATGGTACAGATCCAGATGATGTTCAATTTTATACTATTGAAAATTCTG
TGCCAGTACACTTACTAAGAACAGGTGATGAATTTGCTACAGGAACATTTTTTTTTGATTGTAAACCA
TGTAGACTAACACATACATGGCAAACAAATAGAGCATTGGGCTTACCACCATTTCTAAATTCTTTGCC
TCAAGCTGAAGGAGGTACTAACTTTGGTTATATAGGAGTTCAACAAGATAAAAGACGTGGTGTAACTC
AAATGGGAAATACAAACTATATTACTGAAGCTACTATTATGAGACCAGCTGAGGTTGGTTATAGTGCA
CCATATTATTCTTTTGAGGCGTCTACACAAGGGCCATTTAAAACACCTATTGCAGCAGGACGGGGGGG
AGCGCAAACAGATGAAAATCAAGCAGCAGATGGTGATCCAAGATATGCATTTGGTAGACAACATGGTC
AAAAAACTACCACAACAGGAGAAACACCTGAGAGATTTACATATATAGCACATCAAGATACAGGAAGA
TATCCAGAAGGAGATTGGATTCAAAATATTAACTTTAACCTTCCTGTAACAAATGATAATGTATTGCT
ACCAACAGATCCAATTGGAGGTAAAACAGGAATTAACTATACTAATATATTTAATACTTATGGTCCTT
TAACTGCATTAAATAATGTACCACCAGTTTATCCAAATGGTCAAATTTGGGATAAAGAATTTGATACT
GACTTAAAACCAAGACTTCATGTAAATGCACCATTTGTTTGTCAAAATAATTGTCCTGGTCAATTATT
TGTAAAAGTTGCGCCTAATTTAACAAATGAATATGATCCTGATGCATCTGCTAATATGTCAAGAATTG
TAACTTACTCAGATTTTTGGTGGAAAGGTAAATTAGTATTTAAAGCTAAACTAAGAGCCTCTCATACT
TGGAATCCAATTCAACAAATGAGTATTAATGTAGATAACCAATTTAACTATGTACCAAGTAATATTGG
AGGTATGAAAATTGTATATGAAAAATCTCAACTAGCACCTAGA
'BRA_DQ340428_2a_BR209_1994'
ATGAGTGATGGAGCAGTTCAACCAGACGGTGGTCAACCTGCTGTCAGAAATGAAAGAGCTACAGGATC
TGGGAACGGGTCTGGAGGCGGGGGTGGTGGTGGTTCTGGGGGTGTGGGGATTTCTACGGGTACTTTCA
ATAATCAGACGGAATTTAAATTTTTGGAAAACGGATGGGTGGAAATCACAGCAAACTCAAGCAGACTT
GTACATTTAAATATGCCAGAAAGTGAAAATTATAGAAGAGTGGTTGTAAATAATTTGGATAAAACTGC
AGTTAACGGAAACATGGCTTTAGATGATACTCATGCACAAATTGTAACACCTTGGTCATTGGTTGATG
CAAATGCTTGGGGAGTTTGGTTTAATCCAGGAGATTGGCAACTAATTGTTAATACTATGAGTGAGTTG
CATTTAGTTAGTTTTGAACAAGAAATTTTTAATGTTGTTTTAAAGACTGTTTCAGAATCTGCTACTCA
GCCACCAACTAAAGTTTATAATAATGATTTAACTGCATCATTGATGGTTGCATTAGATAGTAATAATA
CTATGCCATTTACTCCAGCAGCTATGAGATCTGAGACATTGGGTTTTTATCCATGGAAACCAACCATA
CCAACTCCATGGAGATATTATTTTCAATGGGATAGAACATTAATACCGTCTCATACTGGAACTAGTGG
CACACCAACAAATATATACCATGGTACAGATCCAGATGATGTTCAATTTTATACTATTGAAAATTCTG
TGCCAGTACACTTACTAAGAACAGGTGATGAATTTGCTACAGGAACATTTTTTTTTGATTGTAAACCA
TGTAGACTAACACATACATGGCAAACAAATAGAGCATTGGGCTTACCACCATTTCTAAATTCTTTGCC
TCAAGCTGAAGGAGGTACTAACTTTGGTTATATAGGAGTTCAACAAGATAAAAGACGTGGTGTAACTC
AAATGGGAAATACAAACTATATTACTGAAGCTACTATTATGAGACCAGCTGAGGTTGGTTATAGTGCA
CCATATTATTCTTTTGAGGCGTCTACACAAGGGCCATTTAAAACACCTATTGCAGCAGGACGGGGGGG
AGCGCAAACAGATGAAAATCAAGCAGCAGATGGTGATCCAAGATATGCATTTGGTAGACAACATGGTC
AAAAAACTACCACAACAGGAGAAACACCTGAGAGATTTACATATATAGCACATCAAGATACAGGAAGA
TATCCAGAAGGAGATTGGATTCAAAATATTAACTTTAACCTTCCTGTAACAAATGATAATGTATTGCT
ACCAACAGATCCAATTGGAGGTAAAACAGGAATTAACTATACTAATATATTTAATACTTATGGTCCTT
TAACTGCATTAAATAATGTACCACCAGTTTATCCAAATGGTCAAATTTGGGATAAAGAATTTGATACT
GACTTAAAACCAAGACTTCATGTAAATGCACCATTTGTTTGTCAAAATAATTGTCCTGGTCAATTATT
TGTAAAAGTTGCGCCTAATTTAACAAATGAATATGATCCTGATGCATCTGCTAATATGTCAAGAATTG
TAACTTACTCAGATTTTTGGTGGAAAGGTAAATTAGTATTTAAAGCTAAACTAAGAGCCTCTCATACT

TGGAATCCAATTCAACAAATGAGTATTAATGTAGATAACCAATTTAACTATGTACCAAGTAATATTGG
AGGTATGAAAATTGTATATGAAAAATCTCAACTAGCACCTAGA

'BRA_DQ340431_2a_BR56_1995'

ATGAGTGATGGAGCAGTTCAACCAGACGGTGGTCAACCTGCTGTCAGAAATGAAAGAGCTACAGGATC
TGGGAACGGGTCTGGAGGCGGGGGTGGTGGTGGTTCTGGGGGTGTGGGGATTTCTACGGGTACTTTCA
ATAATCAGACGGAATTTAAATTTTTGGAAAACGGATGGGTGGAAATCACAGCAAACTCAAGCAGACTT
GTACATTTAAATATGCCAGAAAGTGAAAATTATAGAAGAGTGGTTGTAAATAATTTGGATAAAACTGC
AGTTAACGGAAACATGGCTTTAGATGATACTCATGCACAAATTGTAACACCTTGGTCATTGGTTGATG
CAAATGCTTGGGGAGTTTGGTTTAATCCAGGAGATTGGCAACTAATTGTTAATACTATGAGTGAGTTG
CATTTAGTTAGTTTTGAACAAGAAATTTTTAATGTTGTTTTAAAGACTGTTTCAGAATCTGCTACTCA
GCCACCAACTAAAGTTTATAATAATGATTTAACTGCATCATTGATGGTTGCATTAGATAGTAATAATA
CTATGCCATTTACTCCAGCAGCTATGAGATCTGAGACATTGGGTTTTTATCCATGGAAACCAACCATA
CCAACTCCATGGAGATATTATTTTCAATGGGATAGAACATTAATACCGTCTCATACTGGAACTAGTGG
CACACCAACAAATATATACCATGGTACAGATCCAGATGATGTTCAATTTTATACTATTGAAAATTCTG
TGCCAGTACACTTACTAAGAACAGGTGATGAATTTGCTACAGGAACATTTTTTTTTGATTGTAAACCA
TGTAGACTAACACATACATGGCAAACAAATAGAGCATTGGGCTTACCACCATTTCTAAATTCTTTGCC
TCAAGCTGAAGGAGGTACTAACTTTGGTTATATAGGAGTTCAACAAGATAAAAGACGTGGTGTAACTC
AAATGGGAAATACAAACTATATTACTGAAGCTACTATTATGAGACCAGCTGAGGTTGGTTATAGTGCA
CCATATTATTCTTTTGAGGCGTCTACACAAGGGCCATTTAAAACACCTATTGCAGCAGGACGGGGGGG
AGCGCAAACAGATGAAAATCAAGCAGCAGATGGTGATCCAAGATATGCATTTGGTAGACAACATGGTC
AAAAAACTACCACAACAGGAGAAACACCTGAGAGATTTACATATATAGCACATCAAGATACAGGAAGA
TATCCAGAAGGAGATTGGATTCAAAATATTAACTTTAACCTTCCTGTAACAAATGATAATGTATTGCT
ACCAACAGATCCAATTGGAGGTAAAACAGGAATTAACTATACTAATATATTTAATACTTATGGTCCTT
TAACTGCATTAAATAATGTACCACCAGTTTATCCAAATGGTCAAATTTGGGATAAAGAATTTGATACT
GACTTAAAACCAAGACTTCATGTAAATGCACCATTTGTTTGTCAAAATAATTGTCCTGGTCAATTATT
TGTAAAAGTTGCGCCTAATTTAACAAATGAATATGATCCTGATGCATCTGCTAATATGTCAAGAATTG
TAACTTACTCAGATTTTTGGTGGAAAGGTAAATTAGTATTTAAAGCTAAACTAAGAGCCTCTCATACT
TGGAATCCAATTCAACAAATGAGTATTAATGTAGATAACCAATTTAACTATGTACCAAGTAATATTGG
AGGTATGAAAATTGTATATGAAAAATCTCAACTAGCACCTACA

'CHI_DQ354068_2a_redpanda_RPPV_2004'

ATGAGTGATGGAGCAGTTCAACCAGACGGTGGTCAGCCTGCTGTCAGAAATGAAAGAGCTACAGGATC
TGGGAACGGGTCTGGAGGCGGGGGTGGTGGTGGTTCTGGGGGTGTGGGGATTTCTACGGGTACTTTCA
ATAATCAGACGGAATTTAAATTTTTGGAAAACGGATGGGTGGAAATCACAGCAAACTCAAGCAGACTT
GTACATTTAAATATGCCAGAAAGTGAAAATTATAGAAGAGTGGTTGTAAATAATTTGGATAAAACTGC
AGTTAACGGAAACATGGCTTTAGATGATACCCATGCACAAATTGTAACACCTTGGTCATTGGTTGATG
CAAATGCTTGGGGAGTTTGGTTTAATCCAGGAGATTGGCAACTAATTGTTAATACTATGAGTGAGTTG
CATTTAGTTAGTTTTGAACAAGAAATTTTTAATGTTGTTTTAAAGACTGTTTCAGAATCTGCTACTCA
GCCACCAACTAAAGTTTATAATAATGATTTAACTGCATCATTGATGGTTGCATTAGATAGTAATAATA
CTATGCCATTTACTCCAGCAGCTATGAGATCTGAGACATTGGGTTTTTATCCATGGAAACCAACCATA
CCAACTCCATGGAGATATTATTTTCAATGGGATAGAACATTAATACCATCTCATACTGGAACTAGTGG
CACACCAACAAATATATACCATGGTACAGATCCAGATGATGTTCAATTTTATACTATTGAAAATTCTG
TGCCAGTACACTTACTAAGAACAGGTGATGAATTTGCTACAGGAACATTTTTTTTTGATTGTAAACCA
TGTAGACTAACACATACATGGCAAACAAATAGAGCATTGGGCTTACCACCATTTCTAAATTCTTTGCC
TCAAGCTGAAGGAGTTACTAACTTTGGTTATATAGGAGTTCAACAAGATAAAAGACGTGGTGTAACTC
AAATGGGAAATACAAACTATATTACTGAAGCTACTATTATGAGACCAGCTGAGGTTGGTTATAGTGCA
CCATATTATTCTTTTGAGGCGTCTACACAAGGGCCATTTAAAACACCTATTGCAGCAGGACGGGGGGG
AGCGCAAACAGATGAAAATCAAGCAGCAGATGGTGATCCAAGATATGCATTTGGTAGACAACATGGTC
AAAAAACTACCACAACAGGAGAAACACCTGAGAGATTTACATATATAGCACATCAAGATACAGGAAGA
TATCCAGAAGGAGATTGGATTCAAAATATTAACTTTAACCTTCCTGTAACAAATGATAATGTATTGCT
ACCAACAGATCCAATTGGAGGTAAAACAGGAATTAACTATACTAATATATTTAATACTTATGGTCCTT
TAACTGCATTAAATAATGTACCACCAGTTTATCCAAATGGTCAAATTTGGGATAAAGAATTTGATACT
GACTTAAAACCAAGACTTCATGTAAATGCACCATTTGTTTGTCAAAATAATTGTCCTGGTCAATTATT
TGTAAAAGTTGCGCCTAATTTAACAAATGAATATGATCCTGATGCATCTGCTAATATGTCAAGAATTG
TAACTTACTCAGATTTTTGGTGGAAAGGTAAATTAGTATTTAAAGCTAAACTAAGAGCCTCTCATACT

TGGAATCCAATTCAACAAATGAGTATTAATGTAGATAACCAATTTAACTATGTACCAAGTAATATTGG
AGGTATGAAAATTGTATATGAAAAATCTCAACTAGCACCTAGA
'TWN_EF592511_TWN1_2006'
ATGAGTGATGGAGCAGTTCAACCAGACGGTGGTCAGCCTGCTGTCAGAAATGAAAGAGCTACAGGATC
TGGGAACGGGTCTGGAGGCGGGGGTGGTGGTGGTTCTGGGGGTGTGGGGATTTCTACGGGTACTTTCA
ATAATCAGACGGAATTTAAATTTTTGGAAAACGGATGGGTGGAAATCACAGCAAACTCAAGCAGACTT
GTACATTTAAATATGCCAGAAAGTGAAAATTATAGAAGAGTGGTTGTAAATAATTTGGATAAAACTGC
AGTTAACGGAAACATGGCTTTAGATGATACCCATGCACAAATTGTAACACCTTGGTCATTGGTTGATG
CAAATGCTTGGGGAGTTTGGTTTAATCCAGGAGATTGGCAACTAATTGTTAATACTATGAGTGAGTTG
CATTTAGTTAGTTTTGAACAAGAAATTTTTAATGTTGTTTTAAAGACTGTTTCAGAATCTGCTACTCA
GCCACCAACTAAAGTTTATAATAATGATTTAACTGCATCATTGATGGTTGCATTAGATAGTAATAATA
CTATGCCATTTACTCCAGCAGCTATGAGATCTGAGACATTGGGTTTTTATCCATGGAAACCAACCATA
CCAACTCCATGGAGATATTATTTTCAATGGGATAGAACATTAATACCATCTCATACTGGAACTAGTGG
CACACCAACAAATATATACCATGGTACAGATCCAGATGATGTTCAATTTTATACTATTGAAAATTCTG
TGCCAGTACACTTACTAAGAACAGGTGATGAATTTGCTACAGGAACATTTTTTTTTGATTGTAAACCA
TGTAGACTAACACATACATGGCAAACAAATAGAGCATTGGGCTTACCACCATTTCTAAATTCTTTGCC
TCAAGCTGAAGGAGGTACTAACTTTGGTTATATAGGAGTTCAACAAGATAAAAGACGTGGTGTAACTC
AAATGGGAAATACAAACTATATTACTGAAGCTACTATTATGAGACCAGCTGAGGTTGGATATAGTGCA
CCATATTATTCTTTTGAGGCGTCTACACAAGGGCCATTTAAAACACCTATTGCAGCAGGACGGGGGGG
AGCGCAAACAGATGAAAATCAAGCAGCAGATGGTGATCCAAGATATGCATTTGGTAGACAACATGGTC
AAAAAACTACCACAACAGGAGAAACACCTGAGAGATTTACATATATAGCACATCAAGATACAGGAAGA
TATCCAGAAGGAGATTGGATTCAAAATATTAACTTTAACCTTCCTGTAACAGATGATAATGTATTGCT
ACCAACAGATCCAATTGGAGGTAAAACAGGAATTAACTATACTAATATATTTAATACTTATGGTCCTT
TAACTGCATTAAATAATGTACCACCAGTTTATCCAAATGGTCAAATTTGGGATAAAGAATTTGATACT
GACTTAAAACCAAGACTTCATGTAAATGCACCATTTGTTTGTCAAAATAATTGTCCTGGTCAATTATT
TGTAAAAGTTGCACCTAATTTAACAAATGAATATGATCCTGATGCATCTGCTAATATGTCAAGAATTG
TAACTTACTCAGATTTTTGGTGGAAAGGTAAATTAGTATTTAAAGCTAAACTAAGAGCCTCTCATACT
TGGAATCCAATTCAACAAATGAGTATTAATGTAGATAACCAATTTAACTATGTACCAAGTAATATTGG
AGGTATGAAAATTGTATATGAAAAATCTCAACTAGCACCTAGA
'KOR_EF599096_DH426_2005'
ATGAGTGATGGAGCAGTTCAACCAGACGGTGGTCAGCCTGCTGTCAGAAATGAAAGAGCTACAGGATC
TGGGAACGGGTCTGGAGGCGGGGGTGGTGGTGGTTCTGGGGGTGTGGGGATTTCTACGGGTACTTTCA
ATAATCAGACGGAATTTAAATTTTTGGAAAACGGATGGGTGGAAATCACAGCAAACTCAAGCAGACTT
GTACATTTAAATATGCCAGAAAGTGAAAATTATAGAAGAGTGGTTGTAAATAATTTGGATAAAACTGC
AGTTAACGGAAACATGGCTTTAGATGATACCCATGCACAAATTGTAACACCTTGGTCATTGGTTGATG
CAAATGCTTGGGGAGTTTGGTTTAATCCAGGAGATTGGCAACTAATTGTTAATACTATGAGTGAATTG
CATTTAGTTAGTTTTGAACAAGAAATTTTTAATGTTGTTTTAAAGACTGTTTCAGAATCTGCTACTCA
GCCACCAACTAAAGTTTATAATAATGATTTAACTGCATCATTGATGGTTGCATTAGATAGTAATAATA
CTATGCCATTTACTCCAGCAGCTATGAGATCTGAGACATTGGGTTTTTATCCATGGAAACCAACCATA
CCAACTCCATGGAGATATTATTTTCAATGGGATAGAACATTAATACCATCTCATACTGGAACTAGTGG
CACACCAACAAATATATACCATGGTACAGATCCAGATGATGTTCAATTTTATACTATTGAAAATTCTG
TGCCAGTACACTTACTAAGAACAGGTGATGAATTTGCTACAGGAACATTTTTTTTTGATTGTAAACCA
TGTAGACTAACACATACATGGCAAACAAATAGAGCATTGGGCTTACCACCATTTCTAAATTCTTTGCC
TCAAGCTGAAGGAGGTACTAACTTTGGTTATATAGGAGTTCAACAAGATAAAAGACGTGGTGTAACTC
AAATGGGAAATACAAACTATATTACTGAAGCTACTATTATGAGACCAGCTGAGGTTGGTTATAGTGCA
CCATATTATTCTTTTGAGGCGTCTACACAAGGGCCATTTAAAACACCTATTGCAGCAGGACGGGGGGG
AGCGCAAACAGATGAAAATCAAGCAGCAGATGGTGATCCAAGATATGCATTTGGTAGACAACATGGTC
AAAAAACTACCACAACAGGAGAAACACCTGAGAGATTTACATATATAGCACATCAAGATACAGGAAGA
TATCCAGAAGGAGATTGGATTCAAAATATTAACTTTAACCTTCCTGTAACAAATGATAATGTATTGCT
ACCAACAGATCCAATTGGAGGTAAAACAGGAATTAACTATACTAATATATTTAATACTTATGGTCCTT
TAACTGCATTAAATAATGTACCACCAGTTTATCCAAATGGTCAAATTTGGGATAAAGAATTTGATACT
GACTTAAAACCAAGACTTCATGTAAATGCACCATTTGTTTGTCAAAATAATTGTCCTGGTCAATTATT
TGTAAAAGTTGCGCCTAATTTAACAAATGAATATGATCCTGATGCATCTGCTAATATGTCAAGAATTG
TAACTTACTCAGATTTTTGGTGGAAAGGTAAATTAGTATTTAAAGCTAAACTAAGAGCCTCTCATACT

TGGAATCCAATTCAACAAATGAGTATTAATGTAGATAACCAATTTAACTATGTACCAAGTAATATTGG
AGGTATGAAAATTGTATATGAAAAATCTCAACTAGCACCTAGA

'KOR_EU009205_2b_K029_2006'

ATGAGTGATGGAGCAGTTCAACCAGACGGTGGTCAGCCTGCTGTCAGAAATGAAAGAGCTACAGGATC
TGGGAACGGGTCTGGAGGCGGGGGTGGTGGTGGTTCTGGGGGTGTGGGGATTTCTACGGGTACTTTCA
ATAATCAGACGGAATTTAAATTTTTGGAAAACGGATGGGTGGAAATCACAGCAAACTCAAGCAGACTT
GTACATTTAAATATGCCAGAAAGTGAAAATTATAGAAGAGTGGTTGTAAATAATTTGGATAAAACTGC
AGTTAACGGAAACATGGCTTTAGATGATACTCATGCACAAATTGTAACACCTTGGTCATTGGTTGATG
CAAATGCTTGGGGAGTTTGGTTTAATCCAGGAGATTGGCAACTAATTGTTAATACTATGAGTGAGTTG
CATTTAGTTAGTTTTGAACAAGAAATTTTTAATGTTGTTTTAAAGACTGTTTCAGAATCTGCTACTCA
GCCACCAACTAAAGTTTATAATAATGATTTAACTGCATCATTGATGGTTGCATTAGATAGTAATAATA
CTATGCCATTTACTCCAGCAGCTATGAGATCTGAGACATTGGGTTTTTATCCATGGAAACCAACCATA
CCAACTCCATGGAGATATTATTTTCAATGGGATAGAACATTAATACCATCTCATACTGGAACTAGTGG
CACACCAACAAATATATACCATGGTACAGATCCAGATGATGTTCAATTTTATACTATTGAAAATTCTG
TGCCAGTACACTTACTAAGAACAGGTGATGAATTTGCTACAGGAACATTTTTTTTTGATTGTAAACCA
TGTAGACTAACACATACATGGCAAACAAATAGAGCATTGGGCTTACCACCATTTCTAAATTCTTTGCC
TCAAGCTGAAGGAGGTACTAACTTTGGTTATATAGGAGTTCAACAAGATAAAAGACGTGGTGTAACTC
AAATGGGAAATACAAACTATATCACTGAAGCTACTATTATGAGACCAGCTGAGGTTGGTTATAGTGCA
CCATATTATTCTTTTGAGGCGTCTACACAAGGGCCATTTAAAACACCTATTGCAGCAGGACGGGGGGG
AGCGCAAACAGATGAAAATCAAGCAGCAGATGGTGATCCAAGATATGCATTTGGTAGACAACATGGTC
AAAAAACTACCACAACAGGAGAAACACCTGAGAGATTTACATATATAGCACATCAAGATACAGGAAGA
TATCCAGAAGGAGATTGGATTCAAAATATTAACTTTAACCTTCCTGTAACAGATGATAATGTATTGCT
ACCAACAGATCCAATTGGAGGTAAAACAGGAATTAACTATACTAATATATTTAATACTTATGGTCCTT
TAACTGCATTAAATAATGTACCACCAGTTTATCCAAATGGTCAAATTTGGGATAAAGAATTTGATACT
GACTTAAAACCAAGACTTCATGTAAATGCACCATTTGTTTGTCAAAATAATTGCCCTGGTCAATTATT
TGTAAAAGTTGCGCCTAATTTAACAAATGAATATGATCCTGATGCATCTGCTAATATGTCAAGAATTG
TAACTTACTCAGATTTTTGGTGGAAAGGTAAATTAGTATTTAAAGCTAAACTAAGAGCCTCTCATACT
TGGAATCCAATTCAACAAATGAGTATTAATGTAGATAACCAATTTAACTATGTACCAAGTAATATTGG
AGGTATGAAAATTGTCTATGAAAAATCTCAACTAGCACCTAGA

'CHI_EU145954_2b_BJ044_20O7'

ATGAGTGATGGAGCAGTTCAACCAGACGGTGGTCAACCTGCTGTCAGAAATGAAAGAGCTACAGGATC
TGGGAACGGGTCTGGAGGCGGGGGTGGTGGTGGTTCTGGGGGTGTGGGGATTTCTACGGGTACTTTCA
ATAATCAGACAGAATTTAAATTTTTGGAAAACGGATGGGTGGAAATCACAGCAAACTCAAGCAGACTT
GTACATTTAAATATGCCAGAAAGTGAAAATTATAGAAGAGTGGTTGTAAATAATTTGGATAAAACTGC
AGTTAACGGAAACATGGCTTTAGATGATACTCATGCACAAATTGTAACACCTTGGTCATTGGTTGATG
CAAATGCTTGGGGAGTTTGGTTTAATCCAGGAGATTGGCAACTAATTGTTAATACTATGAGTGAGTTG
CATTTAGTTAGTTTTGAACAAGAAATTTTTAATGTTGTTTTAAAGACTGTTTCAGAATCTGCTACTCA
GCCACCAACTAAAGTTTATAATAATGATTTAACTGCATCATTGATGGTTGCATTAGATAGTAATAATA
CTATGCCATTTACTCCAGCAGCTATGAGATCTGAGACATTGGGTTTTTATCCATGGAAACCAACCATA
CCAACTCCATGGAGATATTATTTTCAATGGGATAGAACATTAATACCATCTCATACTGGAACTAGTGG
CACACCGACAAATATATACCATGGTACAGATCCAGATGATGTTCAATTTTATACTATTGAAAATTCTG
TGCCAGTACACTTACTAAGAACAGGTGATGAATTTGCTACAGGAACATTTTTTTTTGATTGTAAACCA
TGTAGACTGACACATACATGGCAAACAAATAGAGCATTGGGCTTACCACCATTTCTAAATTCTTTGCC
TCAAGCTGAAGGAGGTACTAACTTTGGTTATATAGGAGTTCAACAAGATAAAAGACGTGGTGTAACTC
AAATGGGAAATACAAACTATATTACTGAAGCTACTATTATGAGACCAGCTGAGGTTGGTTATAGTGCA
CCATATTATTCTTTTGAGGCGTCTACACAAGGGCCATTTAAAACACCTATTGCAGCAGGACGGGGGGG
AGCGCAAACAGATGAAAATCAAGCAGCAGATGGTGATCCAAGATATGCATTTGGTAGACAACATGGTC
AAAAAACTACCACAACAGGAGAAACACCTGAGAGATTTACATATATAGCACATCAAGATACAGGAAGA
TATCCAGAAGGAGATTGGATTCAAAATATTAACTTTAACCTTCCTGTAACAGATGATAATGTATTGCT
ACCAACAGATCCAATTGGAGGTAAAACAGGAATTAACTATACTAATATATTTAATACTTATGGTCCTT
TAACTGCATTAAATAATGTACCACCAGTTTATCCAAATGGTCAAATTTGGGATAAAGAATTTGATACT
GACTTAAAACCAAGACTTCATGTAAATGCACCATTTGTTTGTCAAAATAATTGTCCTGGTCAATTATT
TGTAAAAGTTGCGCCTAATTTAACAAATGAATATGATCCTGATGCATCTGCTAATATGTCAAGAATTG
TAACTTACTCAGATTTTTGGTGGAAAGGTAAATTAGTATTTAAAGCTAAACTAAGAGCCTCTCATACT

TGGAATCCAATTCAACAAATGAGTATCAATGTAGATAACCAATTTAACTATGTACCAAGTAATATTGG
AGGTATGAAAATTGTATATGAAAAATCTCAACTAGCACCTAGA

'CHI_EU483515_2b_ZD13_2007'

ATGAGTGATGGAGCAGTTCAACCAGACGGTGGTCAGCCTGCTGTCAGAAATGAAAGAGCTACAGGATC
TGGGAACGGGTCTGGAGGCGGGGGTGGTGGTGGTTCTGGGGGTGTGGGGATTTCTACGGGTACTTTCA
ATAATCAGACGGAATTTAAATTTTTGGAAAACGGATGGGTGGAAATCACAGCAAACTCAAGCAGACTT
GTACATTTAAATATGCCAGAAAGTGAAAATTATAGAAGAGTGGTTGTAAATAATTTGGATAAAACTGC
AGTTAACGGAAACATGGCTTTAGATGATACCCATGCACAAATTGTAACACCTTGGTCATTGGTTGATG
CAAATGCTTGGGGAGTTTGGTTTAATCCAGGAGATTGGCAACTAATTGTTAATACTATGAGTGAGTTG
CATTTAGTTAGTTTTGAACAAGAAATTTTTAATGTTGTTTTAAAGACTGTTTCAGAATCTGCTACTCA
GCCACCAACTAAAGTTTATAATAATGATTTAACTGCATCATTGATGGTTGCATTAGATAGTAATAATA
CTATGCCATTTACTCCAGCAGCTATGAGATCTGAGACATTGGGTTTTTATCCATGGAAACCAACCATA
CCAACTCCATGGAGATATTATTTTCAATGGGATAGAACATTAATACCATCTCATACTGGAACTAGTGG
CACACCAACAAATATATACCATGGTACAGATCCAGATGATGTTCAATTTTATACTATTGAAAATTCTG
TGCCAGTACACTTACTAAGAACAGGTGATGAATTTGCTACAGGAACATTTTTTTTTGATTGTAAACCA
TGTAGACTAACACATACATGGCAAACAAATAGAGCATTGGGCTTACCACCATTTCTAAATTCTTTGCC
TCAAGCTGAAGGAGGTACTAACTTTGGTTATATAGGAGTTCAACAAGATAAAAGACGTGGTGTAACTC
AAATGGGAAATACAAACTATATTACTGAAGCTACTATTATGAGACCAGCTGAGGTTGGTTATAGTGCA
CCATATTATTCTTTTGAGGCGTCTACACAAGGGCCATTTAAAACACCTATTGCAGCAGGACGGGGGGG
AGCGCAAACAGATGAAAATCAAGCAGCAGATGGTGATCCAAGATATGCATTTGGTAGACAACATGGTC
AAAAAACTACCACAACAGGAGAAACACCTGAGAGATTTACATATATAGCACATCAAGATACAGGAAGA
TATCCAGAAGGAGATTGGATTCAAAATATTAACTTTAACCTTCCTGTAACAGATGATAATGTATTGCT
ACCAACAGATCCAATTGGAGGTAAAACAGGAATTAACTATACTAATATATTTAATACTTATGGTCCTT
TAACTGCATTAAATAATGTACCACCAGTTTATCCAAATGGTCAAATTTGGGATAAAGAATTTGATACT
GACTTAAAACCAAGACTTCATGTAAATGCACCATTTGTTTGTCAAAATAATTGTCCTGGTCAATTATT
TGTAAAAGTTGCGCCTAATTTAACAAATGAATATGATCCTGATGCATCTGCTAATATGTCAAGAATTG
TAACTTACTCAGATTTTTGGTGGAAAGGTAAATTAGTATTTAAAGCTAAACTAAGAGCCTCTCATACT
TGGAATCCAATTCAACAAATGAGTATCAATGTAGATAACCAATTTAACTATGTACCAAGTAATATTGG
AGGTATGAAAATTGTATATGAAAAATCTCAACTAGCACCTAGA

'USA_EU659116_CPV_5_1979'

ATGAGTGATGGAGCAGTTCAACCAGACGGTGGTCAACCTGCTGTCAGAAATGAAAGAGCTACAGGATC
TGGGAACGGGTCTGGAGGCGGGGGTGGTGGTGGTTCTGGGGGTGTGGGGATTTCTACGGGTACTTTCA
ATAATCAGACGGAATTTAAATTTTTGGAAAACGGATGGGTGGAAATCACAGCAAACTCAAGCAGACTT
GTACATTTAAATATGCCAGAAAGTGAAAATTATAGAAGAGTGGTTGTAAATAATATGGATAAAACTGC
AGTTAACGGAAACATGGCTTTAGATGATATTCATGCACAAATTGTAACACCTTGGTCATTGGTTGATG
CAAATGCTTGGGGAGTTTGGTTTAATCCAGGAGATTGGCAACTAATTGTTAATACTATGAGTGAGTTG
CATTTAGTTAGTTTTGAACAAGAAATTTTTAATGTTGTTTTAAAGACTGTTTCAGAATCTGCTACTCA
GCCACCAACTAAAGTTTATAATAATGATTTAACTGCATCATTGATGGTTGCATTAGATAGTAATAATA
CTATGCCATTTACTCCAGCAGCTATGAGATCTGAGACATTGGGTTTTTATCCATGGAAACCAACCATA
CCAACTCCATGGAGATATTATTTTCAATGGGATAGAACATTAATACCATCTCATACTGGAACTAGTGG
CACACCAACAAATATATACCATGGTACAGATCCAGATGATGTTCAATTTTATACTATTGAAAATTCTG
TGCCAGTACACTTACTAAGAACAGGTGATGAATTTGCTACAGGAACATTTTTTTTTGATTGTAAACCA
TGTAGACTAACACATACATGGCAAACAAATAGAGCATTGGGCTTACCACCATTTCTAAATTCTTTGCC
TCAATCTGAAGGAGCTACTAACTTTGGTGATATAGGAGTTCAACAAGATAAAAGACGTGGTGTAACTC
AAATGGGAAATACAAACTATATTACTGAAGCTACTATTATGAGACCAGCTGAGGTTGGTTATAGTGCA
CCATATTATTCTTTTGAGGCGTCTACACAAGGGCCATTTAAAACACCTATTGCAGCAGGACGGGGGGG
AGCGCAAACAGATGAAAATCAAGCAGCAGATGGTAATCCAAGATATGCATTTGGTAGACAACATGGTC
AAAAAACTACCACAACAGGAGAAACACCTGAGAGATTTACATATATAGCACATCAAGATACAGGAAGA
TATCCAGAAGGAGATTGGATTCAAAATATTAACTTTAACCTTCCTGTAACAAATGATAATGTATTGCT
ACCAACAGATCCAATTGGAGGTAAAACAGGAATTAACTATACTAATATATTTAATACTTATGGTCCTT
TAACTGCATTAAATAATGTACCACCAGTTTATCCAAATGGTCAAATTTGGGATAAAGAATTTGATACT
GACTTAAAACCAAGACTTCATGTAAATGCACCATTTGTTTGTCAAAATAATTGTCCTGGTCAATTATT
TGTAAAAGTTGCGCCTAATTTAACAAATGAATATGATCCTGATGCATCTGCTAATATGTCAAGAATTG
TAACTTACTCAGATTTTTGGTGGAAAGGTAAATTAGTATTTAAAGCTAAACTAAGAGCCTCTCATACT

TGGAATCCAATTCAACAAATGAGTATTAATGTAGATAACCAATTTAACTATGTACCAAGTAATATTGG
AGGTATGAAAATTGTATATGAAAAATCTCAACTAGCACCTAGA
'USA_EU659118_CPV_13_1981'
ATGAGTGATGGAGCAGTTCAACCAGACGGTGGTCAACCTGCTGTCAGAAATGAAAGAGCTACAGGATC
TGGGAACGGGTCTGGAGGCGGGGGTGGTGGTGGTTCTGGGGGTGTGGGGATTTCTACGGGTACTTTCA
ATAATCAGACGGAATTTAAATTTTTGGAAAACGGATGGGTGGAAATCACAGCAAACTCAAGCAGACTT
GTACATTTAAATATGCCAGAAAGTGAAAATTATAGAAGAGTGGTTGTAAATAATTTGGATAAAACTGC
AGTTAACGGAAACATGGCTTTAGATGATACCCATGCACAAATTGTAACACCTTGGTCATTGGTTGATG
CAAATGCTTGGGGAGTTTGGTTTAATCCAGGAGATTGGCAACTAATTGTTAATACTATGAGTGAGTTG
CATTTAGTTAGTTTTGAACAAGAAATTTTTAATGTTGTTTTAAAGACTGTTTCAGAATCTGCTACTCA
GCCACCAACTAAAGTTTATAATAATGATCTAACTGCATCATTGATGGTTGCATTAGATAGTAATAATA
CTATGCCATTTACTCCAGCAGCTATGAGATCTGAGACATTGGGCTTTTATCCATGGAAACCAACCATA
CCAACTCCATGGAGATATTATTTTCAATGGGATAGAACATTAATACCATCTCATACTGGAACTAGTGG
CACACCAACAAATATATACCATGGTACAGATCCAGATGATGTTCAATTTTATACTATTGAAAATTCTG
TGCCAGTACACTTACTAAGAACAGGTGATGAATTTGCTACAGGAACATTTTTTTTTGATTGTAAACCA
TGTAGACTAACACATACATGGCAAACAAATAGAGCATTGGGCTTACCACCATTTCTAAATTCTTTGCC
TCAATCTGAAGGAGGTACTAACTTTGGTTATATAGGAGTTCAACAAGATAAAAGACGTGGTGTAACTC
AAATGGGAAATACAAACTATATTACTGAAGCTACTATTATGAGACCAGCTGAGGTTGGTTATAGTGCA
CCATATTATTCTTTTGAGGCGTCTACACAAGGGCCATTTAAAACACCTATTGCAGCAGGACGGGGGGG
AGCGCAAACAGATGAAAATCAAGCAGCAGATGGTGATCCAAGATATGCATTTGGTAGACAACATGGTC
AAAAAACTACCACAACAGGAGAAACACCTGAGAGATTTACATATATAGCACATCAAGATACAGGAAGA
TATCCAGAAGGAGATTGGATTCAAAATATTAACTTTAACCTTCCTGTAACAAATGATAATGTATTGCT
ACCAACAGATCCAATTGGAGGTAAAACAGGAATTAACTATACTAATATATTTAATACTTATGGTCCTT
TAACTGCATTAAATAATGTACCACCAGTTTATCCAAATGGTCAAATTTGGGATAAAGAATTTGATACT
GACTTAAAACCAAGACTTCATGTAAATGCACCATTTGTTTGTCAAAATAATTGTCCTGGTCAATTATT
TGTAAAAGTTGCGCCTAATTTAACAAATGAATATGATCCTGATGCATCTGCTAATATGTCAAGAATTG
TAACTTACTCAGATTTTTGGTGGAAAGGTAAATTAGTATTTAAAGCTAAACTAAGAGCCTCTCATACT
TGGAATCCAATTCAACAAATGAGTATTAATGTAGATAACCAATTTAACTATGTACCAAGTAATATTGG
AGGTATGAAAATTGTATATGAAAAATCTCAACTAGCACCTAGA
'USA_EU659119_2b_CPV_410_2000'
ATGAGTGATGGAGCAGTTCAACCAGACGGTGGTCAACCTGCTGTCAGAAATGAAAGAGCTACAGGATC
TGGGAACGGGTCTGGAGGCGGGGGTGGTGGTGGTTCTGGGGGTGTGGGGATTTCTACGGGTACTTTCA
ATAATCAGACGGAATTTAAATTTTTGGAAAACGGATGGGTGGAAATCACAGCAAACTCAAGCAGACTT
GTACATTTAAATATGCCAGAAAGTGAAAATTATAGAAGAGTGGTTGTAAATAATTTGGATAAAACTGC
AGTTAACGGAAACATGGCTTTAGATGATACTCATGCACAAATTGTAACACCTTGGTCATTGGTTGATG
CAAATGCTTGGGGAGTTTGGTTTAATCCAGGAGATTGGCAACTAATTGTTAATACTATGAGTGAGTTG
CATTTAGTTAGTTTTGAACAAGAAATTTTTAATGTTGTTTTAAAGACTGTTTCAGAATCTGCTACTCA
GCCACCAACTAAAGTTTATAATAATGATTTAACTGCATCATTGATGGTTGCATTAGATAGTAATAATA
CTATGCCATTTACTCCAGCAGCTATGAGATCTGAGACATTGGGTTTTTATCCATGGAAACCAACCATA
CCAACTCCATGGAGATATTATTTTCAATGGGATAGAACATTAATACCATCTCATACTGGAACTAGTGG
CACACCAACAAATATATACCATGGTACAGATCCAGATGATGTTCAATTTTATACTATTGAAAATTCTG
TGCCAGTACACTTACTAAGAACAGGTGATGAATTTGCTACAGGAACATTTTTTTTTGATTGTAAACCA
TGTAGACTAACACATACATGGCAAACAAATAGAGCATTGGGCTTACCACCATTTCTAAATTCTTTGCC
TCAAGCTGAAGGAGGTACTAACTTTGGTTATATAGGAGTTCAACAAGATAAAAGACGTGGTGTAACTC
AAATGGGAAAAACAAACTATATTACTGAAGCTACTATTATGAGACCAGCTGAGGTTGGTTATAGTGCA
CCATATTATTCTTTTGAGGCGTCTACACAAGGGCCATTTAAAACACCTATTGCAGCAGGACGGGGGGG
AGCGCAAACAGATGAAAATCAAGCAGCAGATGGTGATCCAAGATATGCATTTGGTAGACAACATGGTC
AAAAAACTACCACAACAGGAGAAACACCTGAGAGATTTACATATATAGCACATCAAGATACAGGAAGA
TATCCAGAAGGAAATTGGATTCAAAATATTAACTTTAACCTTCCTGTAACAGATGATAATGTATTGCT
ACCAACAGATCCAATTGGAGGTAAAACAGGAATTAACTATACTAATATATTTAATACTTATGGTCCTT
TAACTGCATTAAATAATGTACCACCAGTTTATCCAAATGGTCAAATTTGGGATAAAGAATTTGATACT
GACTTAAAACCAAGACTTCATGTAAATGCACCATTTGTTTGTCAAAATAATTGTCCTGGTCAATTATT
TGTAAAAGTTGCGCCTAATTTAACAAATGAATATGATCCTGATGCATCTGCTAATATGTCAAGAATTG
TAACTTACTCAGATTTTTGGTGGAAAGGTAAATTAGTATTTAAAGCTAAACTAAGAGCCTCTCATACT

TGGAATCCAATTCAACAAATGAGTATTAATGTAGATAACCAATTTAACTATGTACCAAGTAATATTGG
AGGTATGGAAATTGTATATGAAAGATCTCAACTAGCACCTAGA
'USA_EU659120_2b_CPV_411a_1998'
ATGAGTGATGGAGCAGTTCAACCAGACGGTGGTCAACCTGCTGTCAGAAATGAAAGAGCTACAGGATC
TGGGAACGGGTCTGGAGGCGGGGGTGGTGGTGGTTCTGGGGGTGTGGGGATTTCTACGGGTACTTTCA
ATAATCAGACGGAATTTAAATTTTTGGAAAACGGATGGGTGGAAATCACAGCAAACTCAAGCAGACTT
GTACATTTAAATATGCCAGAAAGTGAAAATTATAGAAGAGTGGTTGTAAATAATTTGGATAAAACTGC
AGTTAACGGAAACATGGCTTTAGATGATACTCATGCACAAATTGTAACACCTTGGTCATTGGTTGATG
CAAATGCTTGGGGAGTTTGGTTTAATCCAGAAGATTGGCAACTAATTGTTAATACTATGAGTGAGTTG
CATTTAGTTAGTTTTGAACAAGAAATTTTTAATGTTGTTTTAAAGACTGTTTCAGAATCTGCTACTCA
GCCACCAACTAAAGTTTATAATAATGATTTAACTGCATCATTGATGGTTGCATTAGATAGTAATAATA
CTATGCCATTTACTCCAGCAGCTATGAGATCTGAGACATTGGGTTTTTATCCATGGAAACCAACCATA
CCAACTCCATGGAGATATTATTTTCAATGGGATAGAACATTAATACCATCTCATACTGGAACTAGTGG
CACACCAACAAATATATACCATGGTACAGATCCAGATGATGTTCAATTTTATACTATTGAAAATTCTG
TGCCAGTACACTTACTAAGAACAGGTGATGAATTTGCTACAGGAACATTTTTTTTTGATTGTAAACCA
TGTAGACTAACACATACATGGCAAACAAATAGAGCATTGGGCTTACCACCATTTCTAAATTCTTTGCC
TCAAGCTGAAGGAGGTACTAACTTTGGTTATATAGGAGTTCAACAAGATAAAAGACGTGGTGTAACTC
AAATGGGAAAAACAAACTATATTACTGAAGCTACTATTATGAGACCAGCTGAGGTTGGTTATAGTGCA
CCATATTATTCTTTTGAGGCGTCTACACAAGGGCCATTTAAAACACCTATTGCAGCAGGACGGGGGGG
AGCGCAAACAGATGAAAATCAAGCAGCAGATGGTGATCCAAGATATGCATTTGGTAGACAACATGGTC
AAAAAACTACCACAACAGGAGAAACACCTGAGAGATTTACATATATAGCACATCAAGATACAGGAAGA
TATCCAGAAGGAAATTGGATTCAAAATATTAACTTTAACCTTCCTGTAACAGATGATAATGTATTGCT
ACCAACAGATCCAATTGGAGGTAAAACAGGAATTAACTATACTAATATATTTAATACTTATGGTCCTT
TAACTGCATTAAATAATGTACCACCAGTTTATCCAAATGGTCAAATTTGGGATAAAGAATTTGATACT
GACTTAAAACCAAGACTTCATGTAAATGCACCATTTGTTTGTCAAAATAATTGTCCTGGTCAATTATT
TGTAAAAGTTGCGCCTAATTTAACAAATGAATATGATCCTGATGCATCTGCTAATATGTCAAGAATTG
TAACTTACTCAGATTTTTGGTGGAAAGGTAAATTAGTATTTAAAGCTAAACTAAGAGCCTCTCATACT
TGGAATCCAATTCAACAAATGAGTATTAATGTAGATAACCAATTTAACTATGTACCAAGTAATATTGG
AGGTATGGAAATTGTATATGAAAGATCTCAACTAGCACCTAGA
'VAC_EU914139_Pfizer_2006'
ATGAGTGATGGAGCAGTTCAACCAGACGGTGGTCAACCTGCTGTCAGAAATGAAAGAGCTACAGGATC
TGGGAACGGGTCTGGAGGCGGGGGTGGTGGTGGTTCTGGGGGTGTGGGGATTTCTACGGGTGCTTTCA
ATAATCAGACGGAATTTAAATTTTTGGAAAACGGATGGGTGGAAATCACAGCAAACTCAAGCAGACTT
GTACATTTAAATATGCCAGAAAGTGAAAATTATAGAAGAGTGGTTGTAAATAATATGGATAAAACTGC
AGTTAACGGAAACATGGCTTTAGATGATATTCATGCACAAATTGTAACACCTTGGTCATTGGTTGATG
CAAATGCTTGGGGAGTTTGGTTTAATCCAGGAGATTGGCAACTAATTGTTAATACTATGAGTGAGTTG
CATTTAGTTAGTTTTGAACAAGAAATTTTTAATGTTGTTTTAAAGACTGTTTCAGAATCTGCTACTCA
GCCACCAACTAAAGTTTATAATAATGATTTAACTGCATCATTGATGGTTGCATTAGATAGTAATAATA
CTATGCCATTTACTCCAGCAGCTATGAGATCTGAGACATTGGGTTTTTATCCATGGAAACCAACCATA
CCAACTCCATGGAGATATTATTTTCAATGGGATAGAACATTAAAACCATCTCATACTGGAACTAGTGG
CACACCAACAAATATATACCATGGTACAGATCCAGATGATGTTCAATTTTATACTATTGAAAATTCTG
TGCCAGTACACTTACTAAGAACAGGTGATGAATTTGCTACAGGAACATTTTTTTTTGATGTTAAACCA
TGTAGACTAACACATACATGGCAAACAAATAGAGCATTGGGCTTACCACCATTTCTAAATTCTTTGCC
TCAATCTGAAGGAGCTACTAACTTTGGTGATATAGGAGTTCAACAAGATAAAAGACGTGGTGTAACTC
AAATGGGAAATACAAACTATATTACTGAAGCTACTATTATGAGACCAGCTGAGGTTGGTTATAGTGCA
CCATATTATTCTTTTGAGGCGTCTACACAAGGGCCATTTAAAACACCTATTGCAGCAGGACGGGGGGG
AGCGCAAACAGATGAAAATCAAGCAGCAGATGGTGATCCAAGATATGCATTTGGTAGACAACATGGTC
GAAAAACTACCACAACAGGAGAAACACCTGAGAGATTTACATATATAGCACATCAAGATACAGGAAGA
TATCCAGAAGGAGATTGGATTCAAAATATTAACTTTAACCTTCCTGTAACGAATGATAATGTATTGCT
ACCAACAGATCCAATTGGAGGTAAAACAGGAATTAACTATACTAATATATTTAATACTTATGGTCCTT
TAACTGCATTAAATAATGTACCACCAGTTTATCCAAATGGTCAAATTTGGGATAAAGAATTTGATACT
GACTTAAAACCAAGACTTCATGTAAATGCACCATTTGTTTGTCAAAATAATTGTCCTGGTCAATTATT
TGTAAAAGTTGCGCCTAATTTAACAAATGAATATGATCCTGATGCATCTGCTAATATGTCAAGAATTG
TAACTTACTCAGATTTTTGGTGGAAAGGTAAATTAGTATTTAAAGCTAAACTAAGAGCCTCTCATACT

TGGAATCCAATTCAACAAATGAGTATTAATGTAGATAACCAATTTAACTATGTACCAAGTAATATTGG
AGGTATGAAAATTGTATTTGAAAAATCTCAACTAGCACCTAGA
'ITA_FJ005195_2c_136_2000'
ATGAGTGATGGAGCAGTTCAACCAGACGGTGGTCAACCTGCTGTCAGAAATGAAAGAGCAACAGGATC
TGGGAACGGGTCTGGAGGCGGGGGTGGTGGTGGTTCTGGGGGTGTGGGGATTTCTACGGGTACTTTCA
ATAATCAGACGGAATTTAAATTTTTGGAAAACGGATGGGTGGAAATCACAGCAAACTCAAGCAGACTT
GTACATTTAAATATGCCAGAAAGTGAAAATTATAGAAGAGTGGTTGTAAATAATTTGGATAAAACTGC
AGTTAACGGAAACATGGCTTTAGATGATACTCATGCACAAATTGTAACACCTTGGTCATTGGTTGATG
CAAATGCTTGGGGAGTTTGGTTTAATCCAGGAGATTGGCAACTAATTGTTAATACTATGAGTGAGTTG
CATTTAGTTAGTTTTGAACAAGAAATTTTTAATGTTGTTTTAAAGACTGTTTCAGAATCTGCTACTCA
GCCACCAACTAAAGTTTATAATAATGATTTAACTGCATCATTGATGGTTGCATTAGATAGTAATAATA
CTATGCCATTTACTCCAGCAGCTATGAGATCTGAGACATTGGGTTTTTATCCATGGAAACCAACCATA
CCAACTCCATGGAGATATTATTTTCAATGGGATAGAACATTAATACCATCTCATACTGGAACTAGTGG
CACACCAACAAATATATACCATGGTACAGATCCAGATGATGTTCAATTTTATACTATTGAAAATTCTG
TGCCAGTACACTTACTAAGAACAGGTGATGAATTTGCTACAGGAACATTTTTTTTTGATTGTAAACCA
TGTAGACTAACACATACATGGCAAACAAATAGAGCATTGGGCTTACCACCATTTCTAAATTCTTTGCC
TCAAGCTGAAGGAGGTACTAACTTTGGTTATATAGGAGTTCAACAAGATAAAAGACGTGGTGTAACTC
AAATGGGAAATACAAACTATATTACTGAAGCTACTATTATGAGACCAGCTGAGGTTGGTTATAGTGCA
CCATATTATTCTTTTGAGGCGTCTACACAAGGGCCATTTAAAACACCTATTGCAGCAGGACGGGGGGG
AGCGCAAACAGATGAAAATCAAGCAGCAGATGGTGATCCAAGATATGCATTTGGTAGACAACATGGTC
AAAAAACTACCACAACAGGAGAAACACCTGAGAGATTTACATATATAGCACATCAAGATACAGGAAGA
TATCCAGAAGGAGATTGGATTCAAAATATTAACTTTAACCTTCCTGTAACAGAAGATAATGTATTGCT
ACCAACAGATCCAATTGGAGGTAAAACAGGAATTAACTATACTAATATATTTAATACTTATGGTCCTT
TAACTGCATTAAATAATGTACCACCAGTTTATCCAAATGGTCAAATTTGGGATAAAGAATTTGATACT
GACTTAAAACCAAGACTTCATGTAAATGCACCATTTGTTTGTCAAAATAATTGTCCTGGTCAATTATT
TGTAAAAGTTGCGCCTAATTTAACAAATGAATATGATCCTGATGCATCTGCTAATATGTCAAGAATTG
TAACTTACTCAGATTTTTGGTGGAAAGGTAAATTAGTATTTAAAGCTAAACTAAGAGCCTCTCATACT
TGGAATCCAATTCAACAAATGAGTATTAATGTAGATAACCAATTTAAGTATGTACCAAGTAATATTGG
AGGTATGAAAATTGTATATGAAAAATCTCAACTAGCACCTAGA
'GER_FJ005196_2c_G7_1997'
ATGAGTGATGGAGCAGTTCAACCAGACGGTGGTCAACCTGCTGTCAGAAATGAAAGAGCAACAGGATC
TGGGAACGGGTCTGGAGGCGGGGGTGGTGGTGGTTCTGGGGGTGTGGGGATTTCTACGGGTACTTTCA
ATAATCAGACGGAATTTAAATTTTTGGAAAACGGATGGGTGGAAATCACAGCAAACTCAAGCAGACTT
GTACATTTAAATATGCCAGAAAGTGAAAATTATAGAAGAGTGGTTGTAAATAATTTGGATAAAACTGC
AGTTAACGGAAACATGGCTTTAGATGATACTCATGCACAAATTGTAACACCTTGGTCATTGGTTGATG
CAAATGCTTGGGGAGTTTGGTTTAATCCAGGAGATTGGCAACTAATTGTTAATACTATGAGTGAGTTG
CATTTAGTTAGTTTTGAACAAGAAATTTTTAATGTTGTTTTAAAGACTGTTTCAGAATCTGCTACTCA
GCCACCAACTAAAGTTTATAATAATGATTTAACTGCATCATTGATGGTTGCATTAGATAGTAATAATA
CTATGCCATTTACTCCAGCAGCTATGAGATCTGAGACATTGGGTTTTTATCCATGGAAACCAACCATA
CCAACTCCATGGAGATATTATTTTCAATGGGATAGAACATTAATACCATCTCATACTGGAACTAGTGG
CACACCAACAAATATATACCATGGTACAGATCCAGATGATGTTCAATTTTATACTATTGAAAATTCTG
TGCCAGTACACTTACTAAGAACAGGTGATGAATTTGCTACAGGAACATTTTTTTTTGATTGTAAACCA
TGTAGACTAACACATACATGGCAAACAAATAGAGCATTGGGCTTACCACCATTTCTAAATTCTTTGCC
TCAAGCTGAAGGAGGTACTAACTTTGGTTATATAGGAGTTCAACAAGATAAAAGACGTGGTGTAACTC
AAATGGGAAATACAAACTATATTACTGAAGCTACTATTATGAGACCAGCTGAGGTTGGTTATAGTGCA
CCATATTATTCTTTTGAGGCGTCTACACAAGGGCCATTTAAAACACCTATTGCAGCAGGACGGGGGGG
AGCGCAAACAGATGAAAATCAAGCAGCAGATGGTGATCCAAGATATGCATTTGGTAGACAACATGGTC
AAAAAACTACCACAACAGGAGAAACACCTGAGAGATTTACATATATAGCACATCAAGATACAGGAAGA
TATCCAGAAGGAGATTGGATTCAAAATATTAACTTTAACCTTCCTGTAACAGAAGATAATGTATTGCT
ACCAACAGATCCAATTGGAGGTAAAACAGGAATTAACTATACTAATATATTTAATACTTATGGTCCTT
TAACTGCATTAAATAATGTACCACCAGTTTATCCAAATGGTCAAATCTGGGATAAAGAATTTGATACT
GACTTAAAACCAAGACTTCATGTAAATGCACCATTTGTTTGTCAAAATAATTGTCCTGGTCAATTATT
TGTAAAAGTTGCGCCTAATTTAACAAATGAATATGATCCTGATGCATCTGCTAATATGTCAAGAATTG
TAACTTACTCAGATTTTTGGTGGAAAGGTAAATTAGTATTTAAAGCTAAACTAAGAGCCTCTCATACT

TGGAATCCAATTCAACAAATGAGTATTAATGTAGATAACCAATTTAACTATGTACCAAGTAATATTGG
AGGTATGAAAATTGTATATGAAAAATCTCAACTAGCACCTAGA
'GER_FJ005199_2c_G172_1997'
ATGAGTGATGGAGCAGTTCAACCAGACGGTGGTCAACCTGCTGTCAGAAATGAAAGAGCAACAGGATC
TGGGAACGGGTCTGGAGGCGGGGGTGGTGGTGGTTCTGGGGGTGTGGGGATTTCTACGGGTACTTTCA
ATAATCAGACGGAATTTAAATTTTTGGAAAACGGATGGGTGGAAATCACAGCAAACTCAAGCAGACTT
GTACATTTAAATATGCCAGAAAGTGAAAATTATAGAAGAGTGGTTGTAAATAATTTGGATAAAACTGC
AGTTAACGGAAACATGGCTTTAGATGATACTCATGCACAAATTGTAACACCTTGGTCATTGGTTGATG
CAAATGCTTGGGGAGTTTGGTTTAATCCAGGAGATTGGCAACTAATTGTTAATACTATGAGTGAGTTG
CATTTAGTTAGTTTTGAACAAGAAATTTTTAATGTTGTTTTAAAGACTGTTTCAGAATCTGCTACTCA
GCCACCAACTAAAGTTTATAATAATGATTTAACTGCATCATTGATGGTTGCATTAGATAGTAATAATA
CTATGCCATTTACTCCAGCAGCTATGAGATCTGAGACATTGGGTTTTTATCCATGGAAACCAACCATA
CCAACTCCATGGAGATATTATTTTCAATGGGATAGAACATTAATACCATCTCATACTGGAACTAGTGG
CACACCAACAAATATATACCATGGTACAGATCCAGATGATGTTCAATTTTATACTATTGAAAATTCTG
TGCCAGTACACTTACTAAGAACAGGTGATGAATTTGCTACAGGAACATTTTTTTTTGATTGTAAACCA
TGTAGACTAACACATACATGGCAAACAAATAGAGCATTGGGCTTACCACCATTTCTAAATTCTTTGCC
TCAAGCTGAAGGAGGTACTAACTTTGGTTATATAGGAGTTCAACAAGATAAAAGACGTGGTGTAACTC
AAATGGGAAATACAAACTATATTACTGAAGCTACTATTATGAGACCAGCTGAGGTTGGTTATAGTGCA
CCATACTATTCTTTTGAGGCGTCTACACAAGGGCCATTTAAAACACCTATTGCAGCAGGACGGGGGGG
AGCGCAAACAGATGAAAATCAAGCAGCAGATGGTGATCCAAGATATGCATTTGGTAGACAACATGGTC
AAAAAACTACCACAACAGGAGAAACACCTGAGAGATTTACATATATAGCACATCAAGATACAGGAAGA
TATCCAGAAGGAGATTGGATTCAAAATATTAACTTTAACCTTCCTGTAACAGAAGATAATGTATTGCT
ACCAACAGATCCAATTGGAGGTAAAACAGGAATTAACTATACTAATATATTTAATACTTATGGTCCTT
TAACTGCATTAAATAATGTACCACCAGTTTATCCAAATGGTCAAATTTGGGATAAAGAATTTGATACT
GACTTAAAACCAAGACTTCATGTAAATGCACCATTTGTTTGTCAAAATAATTGTCCTGGTCAATTATT
TGTAAAAGTTGCGCCTAATTTAACAAATGAATATGATCCTGATGCATCTGCTAATATGTCAAGAATTG
TAACTTACTCAGATTTTTGGTGGAAAGGTAAATTAGTATTTAAAGCTAAACTAAGAGCCTCTCATACT
TGGAATCCAATTCAACAAATGAGTATTAATGTAGATAACCAATTTAACTATGTACCAAGTAATATTGG
AGGTATGAAAATTGTATATGAAAAATCTCAACTAGCACCTAGA
'ITA_FJ005205_2c_279_2004'
ATGAGTGATGGAGCAGTTCAACCAGACGGTGGTCAACCTGCTGTCAGAAATGAAAGAGCAACAGGATC
TGGGAACGGGTCTGGAGGCGGGGGTGGTGGTGGTTCTGGGGGTGTGGGGATTTCTACGGGTACTTTCA
ATAATCAGACGGAATTTAAATTTTTGGAAAACGGATGGGTGGAAATCACAGCAAACTCAAGCAGACTT
GTACATTTAAATATGCCAGAAAGTGAAAATTATAGAAGAGTGGTTGTAAATAATTTGGATAAAACTGC
AGTTAACGGAAACATGGCTTTAGATGATACTCATGCACAAATTGTAACACCTTGGTCATTGGTTGATG
CAAATGCTTGGGGAGTTTGGTTTAATCCAGGAGATTGGCAACTAATTGTTAATACTATGAGTGAGTTG
CATTTAGTTAGTTTTGAACAAGAAATTTTTAATGTTGTTTTAAAGACTGTTTCAGAATCTGCTACTCA
GCCACCAACTAAAGTTTATAATAATGATTTAACTGCATCATTGATGGTTGCATTAGATAGTAATAATA
CTATGCCATTTACTCCAGCAGCTATGAGATCTGAGACATTGGGTTTTTATCCATGGAAACCAACCATA
CCAACTCCATGGAGATATTATTTTCAATGGGATAGAACATTAATACCATCTCATACTGGAACTAGTGG
CACACCAACAAATATATACCATGGTACAGATCCAGATGATGTTCAATTTTATACTATTGAAAATTCTG
TGCCAGTACACTTACTAAGAACAGGTGATGAATTTGCTACAGGAACATTTTTTTTTGATTGTAAACCA
TGTAGACTAACACATACATGGCAAACAAATAGAGCATTGGGCTTACCACCATTTCTAAATTCTTTGCC
TCAAGCTGAAGGAGGTACTAACTTTGGTTATATAGGAGTTCAACAAGATAAAAGACGTGGTGTAACTC
AAATGGGAAATACAAACTATATTACTGAAGCTACTATTATGAGACCAGCTGAGGTTGGTTATAGTGCA
CCATATTATTCTTTTGAGGCGTCTACACAAGGGCCATTTAAAACACCTATTGCAGCAGGACGGGGGGG
AGCGCAAACAGATGAAAATCAAGCAGCAGATGGTGATCCAAGATATGCATTTGGTAGACAACATGGTC
AAAAAACTACCACAACAGGAGAAACACCTGAGAGATTTACATATATAGCACATCAAGATACAGGAAGA
TATCCAGAAGGAGATTGGATTCAAAATATTAACTTTAACCTTCCTGTAACAGAAGATAATGTATTGCT
ACCAACAGATCCAATTGGAGGTAAAACAGGAATTAACTATACTAATATATTTAATACTTATGGTCCTT
TAACTGCATTAAATAATGTACCACCAGTTTATCCAAATGGTCAAATTTGGGATAAAGAATTTGATACT
GACTTAAAACCAAGACTTCATGTAAATGCACCATTTGTTTGTCAAAATAATTGTCCTGGTCAATTATT
TGTAAAAGTTGCGCCTAATTTAACAAATGAATATGATCCTGATGCATCTGCTAATATGTCAAGAATTG
TGACTTACTCAGATTTTTGGTGGAAAGGTAAATTAGTATTTAAAGCTAAACTAAGAGCCTCTCATACT

TGGAATCCAATTCAACAAATGAGTATTAATGTAGATAACCAATTTAACTATGTACCAAGTAATATTGG
AGGTATGAAGATTGTATATGAAAAATCTCAACTAGCACCTAGA

'ITA_FJ005206_2c_287_2004'

ATGAGTGATGGAGCAGTTCAACCAGACGGTGGTCAACCTGCTGTCAGAAATGAAAGAGCAACAGGATC
TGGGAACGGGTCTGGAGGCGGGGGTGGTGGTGGTTCTGGGGGTGTGGGGATTTCTACGGGTACTTTCA
ATAATCAGACGGAATTTAAATTTTTGGAAAACGGATGGGTGGAAATCACAGCAAACTCAAGCAGACTT
GTACATTTAAATATGCCAGAAAGTGAAAATTATAGAAGAGTGGTTGTAAATAATTTGGATAAAACTGC
AGTTAACGGAAACATGGCTTTAGATGATACTCATGCACAAATTGTAACACCTTGGTCATTGGTTGATG
CAAATGCTTGGGGAGTTTGGTTTAATCCAGGAGATTGGCAACTAATTGTTAATACTATGAGTGAGTTG
CATTTAGTTAGTTTTGAACAAGAAATTTTTAATGTTGTTTTAAAGACTGTTTCAGAATCTGCTACTCA
GCCACCAACTAAAGTTTATAATAATGATTTAACTGCATCATTGATGGTTGCATTAGATAGTAATAATA
CTATGCCATTTACTCCAGCAGCTATGAGATCTGAGACATTGGGTTTTTATCCATGGAAACCAACCATA
CCAACTCCATGGAGATATTATTTTCAATGGGATAGAACATTAATACCATCTCATACTGGAACTAGTGG
CACACCAACAAATATATACCATGGTACAGATCCAGATGATGTTCAATTTTATACTATTGAAAATTCTG
TGCCAGTACACTTACTAAGAACAGGTGATGAATTTGCTACAGGAACATTTTTTTTTGATTGTAAACCA
TGTAGACTAACACATACATGGCAAACAAATAGAGCATTGGGCTTACCACCATTTCTAAATTCTTTGCC
TCAAGCTGAAGGAGGTACTAACTTTGGTTATATAGGAGTTCAACAAGATAAAAGACGTGGTGTAACTC
AAATGGGAAATACAAACTATATTACTGAAGCTACTATTATGAGACCAGCTGAGGTTGGTTATAGTGCA
CCATATTATTCTTTTGAGGCGTCTACACAAGGGCCATTTAAAACACCTATTGCAGCAGGACGGGGGGG
AGCGCAAACAGATGAAAATCAAGCAGCAGATGGTGATCCAAGATATGCATTTGGTAGACAACATGGTC
AAAAAACTACCACAACAGGAGAAACACCTGAGAGATTTACATATATAGCACATCAAGATACAGGAAGA
TATCCAGAAGGAGATTGGATTCAAAATATTAACTTTAACCTTCCTGTAACAGAAGATAATGTATTGCT
ACCAACAGATCCAATTGGAGGTAAAACAGGAATTAACTATACTAATATATTTAATACTTATGGTCCTT
TAACTGCATTAAATAATGTACCACCAGTTTATCCAAATGGTCAAATTTGGGATAAAGAATTTGATACT
GACTTAAAACCAAGACTTCATGTAAATGCACCATTTGTTTGTCAAAATAATTGTCCTGGTCAATTATT
TGTAAAAGTTGCGCCTAATTTAACAAATGAATATGATCCTGATGCATCTGCTAATATGTCAAGAATTG
TAACTTACTCAGATTTTTGGTGGAAAGGTAAATTAGTATTTAAAGCTAAACTAAGAGCCTCTCATACT
TGGAATCCAATTCAACAAATGAGTATTAATGTAGATAACCAATTTAACTATGTACCAAGTAATATTGG
AGGTATGAAAATTGTATATGAAAAATCTCAACTAGCACCTAGA

'ITA_FJ005209_2c_303_2004'

ATGAGTGATGGAGCAGTTCAACCAGACGGTGGTCAACCTGCTGTCAGAAATGAAAGAGCAACAGGATC
TGGGAACGGGTCTGGAGGCGGGGGTGGTGGTGGTTCTGGGGGTGTGGGGATTTCTACGGGTACTTTCA
ATAATCAGACGGAATTTAAATTTTTGGAAAACGGATGGGTGGAAATCACAGCAAACTCAAGCAGACTT
GTACATTTAAATATGCCAGAAAGTGAAAATTATAGAAGAGTGGTTGTAAATAATTTGGATAAAACTGC
AGTTAACGGAAACATGGCTTTAGATGATACTCATGCACAAATTGTAACACCTTGGTCATTGGTTGATG
CAAATGCTTGGGGAGTTTGGTTTAATCCAGGAGATTGGCAACTAATTGTTAATACTATGAGTGAGTTG
CATTTAGTTAGTTTTGAACAAGAAATTTTTAATGTTGTTTTAAAGACTGTTTCAGAATCTGCTACTCA
GCCACCAACTAAAGTTTATAATAATGATTTAACTGCATCATTGATGGTTGCATTAGATAGTAATAATA
CTATGCCATTTACTCCAGCAGCTATGAGATCTGAGACATTGGGTTTCTATCCATGGAAACCAACCATA
CCAACTCCATGGAGATATTATTTTCAATGGGATAGAACATTAATACCATCTCATACTGGAACTAGTGG
CACACCAACAAATATATACCATGGTACAGATCCAGATGATGTTCAATTTTATACTATTGAAAATTCTG
TGCCAGTACACTTACTAAGAACAGGTGATGAATTTGCTACAGGAACATTTTTTTTTGATTGTAAACCA
TGTAGACTAACACATACATGGCAAACAAATAGAGCATTGGGCTTACCACCATTTCTAAATTCTTTGCC
TCAAGCTGAAGGAGGTACTAACTTTGGTTATATAGGAGTTCAACAAGATAAAAGACGTGGTGTAACTC
AAATGGGAAATACAAACTATATTACTGAAGCTACTATTATGAGACCAGCTGAGGTTGGTTATAGTGCA
CCATATTATTCTTTTGAGGCGTCTACACAAGGGCCATTTAAAACACCTATTGCAGCAGGACGGGGGGG
AGCGCAAACAGATGAAAATCAAGCAGCAGATGGTGATCCAAGATATGCATTTGGTAGACAACATGGTC
AAAAAACTACCACAACAGGAGAAACACCTGAGAGATTTACATATATAGCACATCAAGATACAGGAAGA
TATCCAGAAGGAGATTGGATTCAAAATATTAACTTTAACCTTCCTGTAACAGAAGATAATGTATTGCT
ACCAACAGATCCAATTGGAGGTAAAACAGGAATTAACTATACTAATATATTTAATACTTATGGTCCTT
TAACTGCATTAAATAATGTACCACCAGTTTATCCAAATGGTCAAATTTGGGATAAAGAATTTGATACT
GACTTAAAACCAAGACTTCATGTAAATGCACCATTTGTTTGTCAAAATAATTGTCCTGGTCAATTATT
TGTAAAAGTTGCGCCTAATTTAACAAATGAATATGATCCTGATGCATCTGCTAATATGTCAAGAATTG
TAACTTACTCAGATTTTTGGTGGAAAGGTAAATTAGTATTTAAAGCTAAACTAAGAGCCTCTCATACT

TGGAATCCAATTCAACAAATGAGTATTAATGTAGATAACCAATTTAACTATGTACCAAGTAATATTGG
AGGTATGAAAATTGTATATGAAAAATCTCAACTAGCACCTAGA
'ITA_FJ005212_2c_349_2004'
ATGAGTGATGGAGCAGTTCAACCAGACGGTGGTCAACCTGCTGTCAGAAATGAAAGAGCAACAGGATC
TGGGAACGGGTCTGGAGGCGGGGGTGGTGGTGGTTCTGGGGGTGTGGGGATTTCTACGGGTACTTTCA
ATAATCAGACGGAATTTAAATTTTTGGAAAACGGATGGGTGGAAATCACAGCAAACTCAAGCAGACTT
GTACATTTAAATATGCCAGAAAGTGAAAATTATAGAAGAGTGGTTGTAAATAATTTGGATAAAACTGC
AGTTAACGGAAACATGGCTTTAGATGATACTCATGCACAAATTGTAACACCTTGGTCATTGGTTGATG
CAAATGCTTGGGGAGTTTGGTTTAATCCAGGAGATTGGCAACTAATTGTTAATACTATGAGTGAGTTG
CATTTAGTTAGTTTTGAACAAGAAATTTTTAATGTTGTTTTAAAGACTGTTTCAGAATCTGCTACTCA
GCCACCAACTAAAGTTTATAATAATGATTTAACTGCATCATTGATGGTTGCATTAGATAGTAATAATA
CTATGCCATTTACTCCAGCAGCTATGAGATCTGAGACATTGGGTTTTTATCCATGGAAACCAACCATA
CCAACTCCATGGAGATATTATTTTCAATGGGATAGAACATTAATACCATCTCATACTGGAACTAGTGG
CACACCAACAAATATATACCATGGTACAGATCCAGATGATGTTCAATTTTATACTATTGAAAATTCTG
TGCCAGTACACTTACTAAGAACAGGTGATGAATTTGCTACAGGAACATTTTTTTTTGATTGTAAACCA
TGTAGACTAACACATACATGGCAAACAAATAGAGCATTGGGCTTACCACCATTTCTAAATTCTTTGCC
TCAAGCTGAAGGAGGTACTAACTTTGGTTATATAGGAGTTCAACAAGATAAAAGACGTGGTGTAACTC
AAATGGGAAATACAAACTATATTACTGAAGCTACTATTATGAGACCAGCTGAGGTTGGTTATAGTGCA
CCATATTATTCTTTTGAGGCGTCTACACAAGGGCCATTTAAAACACCTATTGCAGCAGGACGGGGGGG
AGCGCAAACAGATGAAAATCAAGCAGCAGATGGTGATCCAAGATATGCATTTGGTAGACAACATGGTC
AAAAAACTACCACAACAGGAGAAACACCTGAGAGATTTACATATATAGCACATCAAGATACAGGAAGA
TATCCAGAAGGAGATTGGATTCAAAATATTAACTTTAACCTTCCTGTAACAGAAGATAATGTATTGCT
ACCAACAGATCCAATTGGAGGTAAAACAGGAATTAACTATACTAATATTTTTAATACTTATGGTCCTT
TAACTGCATTAAATAATGTACCACCAGTTTATCCAAATGGTCAAATTTGGGATAAAGAATTTGATACT
GACTTAAAACCAAGACTTCATGTAAATGCACCATTTGTTTGTCAAAATAATTGTCCTGGTCAATTATT
TGTAAAAGTTGCGCCTAATTTAACAAATGAATATGATCCTGATGCATCTGCTAATATGTCAAGAATTG
TAACTTACTCAGATTTTTGGTGGAAAGGTAAATTAGTATTTAAAGCTAAACTAAGAGCCTCTCATACT
TGGAATCCAATTCAACAAATGAGTATTAATGTAGATAACCAATTTAACTATGTACCAAGTAATATTGG
AGGTATGAAAATTGTATATGAAAAATCTCAACTAGCACCTAGA
'ITA_FJ005214_2c_67_2006'
ATGAGTGATGGAGCAGTTCAACCAGACGGTGGTCAACCTGCTGTCAGAAATGAAAGAGCAACAGGATC
TGGGAACGGGTCTGGAGGCGGGGGTGGTGGTGGTTCTGGGGGTGTGGGGATTTCTACGGGTACTTTCA
ATAATCAGACGGAATTTAAATTTTTGGAAAACGGATGGGTGGAAATCACAGCAAACTCAAGCAGACTT
GTACATTTAAATATGCCAGAAAGTGAAAATTATAGAAGAGTGGTTGTAAATAATTTGGATAAAACTGC
AGTTAACGGAAACATGGCTTTAGATGATACTCATGCACAAATTGTAACACCTTGGTCATTGGTTGATG
CAAATGCTTGGGGAGTTTGGTTTAATCCGGGAGATTGGCAACTAATTGTTAATACTATGAGTGAGTTG
CATTTAGTTAGTTTTGAACAAGAAATTTTTAATGTTGTTTTAAAGACTGTTTCAGAATCTGCTACTCA
GCCACCAACTAAAGTTTATAATAATGATTTAACTGCATCATTGATGGTTGCATTAGATAGTAATAATA
CTATGCCATTTACTCCAGCAGCTATGAGATCTGAGACATTGGGTTTTTATCCATGGAAACCAACCATA
CCAACTCCATGGAGATATTATTTTCAATGGGATAGAACATTAATACCATCTCATACTGGAACTAGTGG
CACACCAACAAACATATACCATGGTACAGATCCAGATGATGTTCAATTTTATACTATTGAAAATTCTG
TGCCAGTACACTTACTAAGAACAGGTGATGAATTTGCTACAGGAACATTTTTTTTTGATTGTAAACCA
TGTAGACTAACACATACATGGCAAACAAATAGAGCATTGGGCTTACCACCATTTCTAAATTCTTTGCC
TCAAGCTGAAGGAGGTACTAACTTTGGTTATATAGGAGTTCAACAAGATAAAAGACGTGGTGTAACTC
AAATGGGAAATACAAACTATATTACTGAAGCTACTATTATGAGACCAGCTGAGGTTGGTTATAGTGCA
CCATATTATTCTTTTGAGGCGTCTACACAAGGGCCATTTAAAACACCTATTGCAGCAGGACGGGGGGG
AGCGCAAACAGATGAAAATCAAGCAGCAGATGGTGATCCAAGATATGCATTTGGTAGACAACATGGTC
AAAAAACTACCACAACAGGAGAAACACCTGAGAGATTTACATATATAGCACATCAAGATACAGGAAGA
TATCCAGAAGGAGATTGGATTCAAAATATTAACTTTAACCTTCCTGTAACAGAAGATAATGTATTGCT
ACCAACAGATCCAATTGGAGGTAAAACAGGAATTAACTATACTAATATATTTAATACTTATGGTCCTT
TAACTGCATTAAATAATGTACCACCAGTTTATCCAAATGGTCAAATTTGGGATAAAGAATTTGATACT
GACTTAAAACCAAGACTTCATGTAAATGCACCATTTGTTTGTCAAAATAATTGTCCTGGTCAATTATT
TGTAAAAGTTGCGCCTAATTTAACAAATGAATATGATCCTGATGCATCTGCTAATATGTCAAGAATTG
TAACTTACTCAGATTTTTGGTGGAAAGGTAAATTAGTATTTAAAGCTAAACTAAGAGCCTCTCATACT

TGGAATCCAATTCAACAAATGAGTATTAATGTAGATAACCAATTTAACTATGTACCAAGTAATATTGG
AGGTATGAAAATTGTATATGAAAAATCTCAACTAGCACCTAGA

'ITA_FJ005216_2c_284_2006'

ATGAGTGATGGAGCAGTTCAACCAGACGGTGGTCAACCTGCTGTCAGAAATGAAAGAGCAACAGGATC
TGGGAACGGGTCTGGAGGCGGGGGTGGTGGTGGTTCTGGGGGTGTGGGGATTTCTACGGGTACTTTCA
ATAATCAGACGGAATTTAAATTTTTGGAAAACGGATGGGTGGAAATCACAGCAAACTCAAGCAGACTT
GTACATTTAAATATGCCAGAAAGTGAAAATTATAGAAGAGTGGTTGTAAATAATTTGGATAAAACTGC
AGTTAACGGAAACATGGCTTTAGATGATACTCATGCACAAATTGTAACACCTTGGTCATTGGTTGATG
CAAATGCTTGGGGAGTTTGGTTTAATCCAGGAGATTGGCAACTAATTGTTAATACTATGAGTGAGTTG
CATTTAGTTAGTTTTGAACAAGAAATTTTTAATGTTGTTTTAAAGACTGTTTCAGAATCTGCTACTCA
GCCACCAACTAAAGTTTATAATAATGATTTAACTGCATCATTGATGGTTGCATTAGATAGTAATAATA
CTATGCCATTTACTCCAGCAGCTATGAGATCTGAGACATTGGGTTTTTATCCATGGAAACCAACCATA
CCAACTCCATGGAGATATTATTTTCAATGGGATAGAACATTAATACCATCTCATACTGGAACTAGTGG
CACACCAACAAATATATACCATGGTACAGATCCAGATGATGTTCAATTTTATACTATTGAAAATTCTG
TGCCAGTACACTTACTAAGAACAGGTGATGAATTTGCTACAGGAACATTTTTTTTTGATTGTAAACCA
TGTAGACTAACACATACATGGCAAACAAATAGAGCATTGGGCTTACCACCATTTCTAAATTCTTTGCC
TCAAGCTGAAGGAGGTACTAACTTTGGTTATATAGGAGTTCAACAAGATAAAAGACGTGGTGTGACTC
AAATGGGAAATACAAACTATATTACTGAAGCTACTATTATGAGACCAGCTGAGGTTGGTTATAGTGCA
CCATATTATTCTTTTGAGGCGTCTACACAAGGGCCATTTAAAACACCTATTGCAGCAGGACGGGGGGG
AGCGCAAACAGATGAAAATCAAGCAGCAGATGGTGATCCAAGATATGCATTTGGTAGACAACATGGTC
AAAAAACTACCACAACAGGAGAAACACCTGAGAGATTTACATATATAGCACATCAAGATACAGGAAGA
TATCCAGAAGGAGATTGGATTCAAAATATTAACTTTAACCTTCCTGTAACAGAAGATAATGTATTGCT
ACCAACAGATCCAATTGGAGGTAAAACAGGAATTAACTATACTAATATATTTAATACTTATGGTCCTT
TAACTGCATTAAATAATGTACCACCAGTTTATCCAAATGGTCAAATTTGGGATAAAGAATTTGATACT
GACTTAAAACCAAGACTTCATGTAAATGCACCATTTGTTTGTCAAAATAATTGTCCTGGTCAATTATT
TGTAAAAGTTGCGCCTAATTTAACAAATGAATATGATCCTGATGCATCTGCTAATATGTCAAGAATTG
TAACTTACTCAGATTTTTGGTGGAAAGGTAAATTAGTATTTAAAGCTAAACTAAGAGCCTCTCATACT
TGGAATCCAATTCAACAAATGAGTATTAATGTAGATAACCAATTTAACTATGTACCAAGTAATATTGG
AGGTATGAAAATTGTATATGAAAAATCTCAACTAGCACCTAGA

'ITA_FJ005218_2c_330_2006'

ATGAGTGATGGAGCAGTTCAACCAGACGGTGGTCAACCTGCTGTCAGAAATGAAAGAGCAACAGGATC
TGGGAACGGGTCTGGAGGCGGGGGTGGTGGTGGTTCTGGGGGTGTGGGGATTTCTACGGGTACTTTCA
ATAATCAGACGGAATTTAAATTTTTGGAAAACGGATGGGTGGAAATCACAGCAAACTCAAGCAGACTT
GTACATTTAAATATGCCAGAAAGTGAAAATTATAGAAGAGTGGTTGTAAATAATTTGGATAAAACTGC
AGTTAACGGAAACATGGCTTTAGATGATACTCATGCACAAATTGTAACACCTTGGTCATTGGTTGATG
CAAATGCTTGGGGAGTTTGGTTTAATCCAGGAGATTGGCAACTAATTGTTAATACTATGAGTGAGTTG
CATTTAGTTAGTTTTGAACAAGAAATTTTTAATGTTGTTTTAAAGACTGTTTCAGAATCTGCTACTCA
GCCACCGACTAAAGTTTATAATAATGATTTAACTGCATCATTGATGGTTGCATTAGATAGTAATAATA
CTATGCCATTTACTCCAGCAGCTATGAGATCTGAGACATTGGGTTTTTATCCATGGAAACCAACCATA
CCAACTCCATGGAGATATTATTTTCAATGGGATAGAACATTAATACCATCTCATACTGGAACTAGTGG
CACACCAACAAATATATACCATGGTACAGATCCAGATGATGTTCAATTTTATACTATTGAAAATTCTG
TGCCAGTACACTTACTAAGAACAGGTGATGAATTTGCTACAGGAACATTTTTTTTTGATTGTAAACCA
TGTAGACTAACACATACATGGCAAACAAATAGAGCATTGGGCTTACCACCATTTCTAAATTCTTTGCC
TCAAGCTGAAGGAGGTACTAACTTTGGTTATATAGGAGTTCAACAAGATAAAAGACGTGGTGTAACTC
AAATGGGAAATACAAACTATATTACTGAAGCTACTATTATGAGACCAGCTGAGGTTGGTTATAGTGCA
CCATATTATTCTTTTGAGGCGTCTACACAAGGGCCATTTAAAACACCTATTGCAGCAGGACGGGGGGG
AGCGCAAACAGATGAAAATCAAGCAGCAGATGGTGATCCAAGATATGCATTTGGTAGACAACATGGTC
AAAAAACTACCACAACAGGAGAAACACCTGAGAGATTTACATATATAGCACATCAAGATACAGGAAGA
TATCCAGAAGGAGATTGGATTCAAAATATTAACTTTAACCTTCCTGTAACAGAAGATAATGTATTGCT
ACCAACAGATCCAATTGGAGGTAAAACAGGAATTAACTATACTAATATATTTAATACTTATGGTCCTT
TAACTGCATTAAATAATGTACCACCAGTTTATCCAAATGGTCAAATTTGGGATAAAGAATTTGATACT
GACTTAAAACCAAGACTTCATGTTAATGCACCATTTGTTTGTCAAAATAATTGTCCTGGTCAATTATT
TGTAAAAGTTGCGCCTAATTTAACAAATGAATATGATCCTGATGCATCTGCTAATATGTCAAGAATTG
TAACTTACTCAGATTTTTGGTGGAAAGGTAAATTAGTATTTAAAGCTAAACTAAGAGCCTCTCATACT

TGGAATCCAATTCAACAAATGAGTATTAATGTAGATAACCAATTTAACTATGTACCAAGTAATATTGG
AGGTATGAAAATTGTATATGAAAAATCTCAACTAGCACCTAGA
'ITA_FJ005226_383_2006'
ATGAGTGATGGAGCAGTTCAACCAGACGGTGGTCAACCTGCTGTCAGAAATGAAAGAGCAACAGGATC
TGGGAACGGGTCTGGAGGCGGGGGTGGTGGTGGTTCTGGGGGTGTGGGGATTTCTACGGGTACTTTCA
ATAATCAGACGAAATTTAAATTTTTGGAAAACGGATGGGTGGAAATCACAGCAAACTCAAGCAGACTT
GTACATTTAAATATGCCAGAAAGTGAAAATTATAGAAGAGTGGTTGTAAATAATTTGGATAAAACTGC
AGTTAACGGAAACATGGCTTTAGATGATACTCATGCACAAATTGTAACACCTTGGTCATTGGTTGATG
CAAATGCTTGGGGAGTTTGGTTTAATCCAGGAGATTGGCAACTAATTGTTAATACTATGAGTGAGTTG
CATTTAGTTAGTTTTGAACAAGAAATTTTTAATGTTGTTTTAAAGACTGTTTCAGAATCTGCTACTCA
GCCACCAACTAAAGTTTATAATAATGATTTAACTGCATCATTGATGGTTGCATTAGATAGTAATAATA
CTATGCCATTTACTCCAGCAGCTATGAGATCTGAGACATTGGGTTTTTATCCATGGAAACCAACCATA
CCAACTCCATGGAGATATTATTTTCAATGGGATAGAACATTAATACCATCTCATACTGGAACTAGTGG
CACACCAACAAATATATACCATGGTACAGATCCAGATGATGTTCAATTTTATACTATTGAAAATTCTG
TGCCAGTACACTTACTAAGAACAGGTGATGAATTTGCTACAGGAACATTTTTTTTTGATTGTAAACCA
TGTAGACTAACACATACATGGCAAACAAATAGAGCATTGGGCTTACCACCATTTCTAAATTCTTTGCC
TCAAGCTGAAGGAGGTACTAACTTTGGTTATATAGGAGTTCAACAAGATAAAAGACGTGGTGTAACTC
AAATGGGAAATACAAACTATATTACTGAAGCTACTATTATGAGACCAGCTGAGGTTGGTTATAGTGCA
CCATATTATTCTTTTGAGGCGTCTACACAAGGGCCATTTAAAACACCTATTGCAGCAGGACGGGGGGG
AGCGCAAACAGATGAAAATCAAGCAGCAGATGGTGATCCAAGATATGCATTTGGTAGACAACATGGTC
AAAAAACTACCACAACAGGAGAAACACCTGAGAGATTTACATATATAGCACATCAAGATACAGGAAGA
TATCCAGAAGGAGATTGGATTCAAAATATTAACTTTAACCTTCCTGTAACAGAAGATAATGTATTGCT
ACCAACAGATCCAATTGGAGGTAAAACAGGAATTAACTATACTAATATATTTAATACTTATGGTCCTT
TAACTGCATTAAATAATGTACCACCAGTTTATCCAAATGGTCAAATTTGGGATAAAGAATTTGATACT
GACTTAAAACCAAGACTTCATGTAAATGCACCATTTGTTTGTCAAAATAATTGTCCTGGTCAATTATT
TGTAAAAGTTGCGCCTAATTTAACAAATGAATATGATCCTGATGCATCTGCTAATATGTCAAGAATTG
TAACTTACTCAGATTTTTGGTGGAAAGGTAAATTAGTATTTAAAGCTAAACTAAGAGCCTCTCATACT
TGGAATCCAATTCAACAAATGAGTATTAATGTAGATAACCAATTTAACTATGTACCAAGTAATATTGG
AGGTATGAAAATTGTATATGAAAAATCTCAACTAGCACCTAGA
'ITA_FJ005231_406_2006'
ATGAGTGATGGAGCAGTTCAACCAGACGGTGGTCAACCTGCTGTCAGAAATGAAAGAGCAACAGGATC
TGGGAACGGGTCTGGAGGCGGGGGTGGTGGTGGTTCTGGGGGTGTGGGGATTTCTACGGGTACTTTCA
ATAATCAGACGGAATTTAAATTTTTGGAAAACGGATGGGTGGAAATCACAGCAAACTCAAGCAGACTT
GTACATTTAAATATGCCAGAAAGTGAAAATTATAGAAGAGTGGTTGTAAATAATTTGGATAAAACTGC
AGTTAACGGAAACATGGCTTTAGATGATACTCATGCACAAATTGTAACACCTTGGTCATTGGTTGATG
CAAATGCTTGGGGAGTTTGGTTTAATCCAGGAGATTGGCAACTGATTGTTAATACTATGAGTGAGTTG
CATTTAGTTAGTTTTGAACAAGAAATTTTTAATGTTGTTTTAAAGACTGTTTCAGAATCTGCTACTCA
GCCACCAACTAAAGTTTATAATAATGATTTAACTGCATCATTGATGGTTGCATTAGATAGTAATAATA
CTATGCCATTTACTCCAGCGGCTATGAGATCTGAGACATTGGGTTTTTATCCATGGAAACCAACCATA
CCAACTCCATGGAGATATTATTTTCAATGGGATAGAACATTAATACCATCTCATACTGGAACTAGTGG
CACACCAACAAATATATACCATGGTACAGATCCAGATGATGTTCAATTTTATACTATTGAAAATTCTG
TGCCAGTACACTTACTAAGAACAGGTGATGAATTTGCTACAGGAACATTTTTTTTTGATTGTAAACCA
TGTAGACTAACACATACATGGCAAACAAATAGAGCATTGGGCTTACCACCATTTCTAAATTCTTTGCC
TCAAGCTGAAGGAGGTACTAACTTTGGTTATATAGGAGTTCAACAAGATAAAAGACGTGGTGTAACTC
AAATGGGAAATACAAACTATATTACTGAAGCTACTATTATGAGACCAGCTGAGGTTGGTTATAGTGCA
CCATATTATTCTTTTGAGGCGTCTACACAAGGGCCATTTAAAACACCTATTGCAGCAGGACGGGGGGG
AGCGCAAACAGATGAAAATCAAGCAGCAGATGGTGATCCAAGATATGCATTTGGTAGACAACATGGTC
AAAAAACTACCACAACAGGAGAAACACCTGAGAGATTTACATATATAGCACATCAAGATACAGGAAGA
TATCCAGAAGGAGATTGGATTCAAAATATTAACTTTAACCTTCCTGTAACAGAAGATAATGTATTGCT
ACCAACAGATCCAATTGGAGGTAAAACAGGAATTAACTATACTAATATATTTAATACTTATGGTCCTT
TAACTGCATTAAATAATGTACCACCAGTTTATCCAAATGGTCAAATTTGGGATAAAGAATTTGATACT
GACTTAAAACCAAGACTTCATGTAAATGCACCATTTGTTTGTCAAAATAATTGTCCTGGTCAATTATT
TGTAAAAGTTGCGCCTAATTTAACAAATGAATATGATCCTGATGCATCTGCTAATATGTCAAGAATTG
TAACTTACTCAGATTTTTGGTGGAAAGGTAAATTAGTATTTAAAGCTAAACTAAGAGCCTCTCATACT

TGGAATCCAATTCAACAAATGAGTATTAATGTAGATAACCAATTTAACTATGTACCAAGTAATATTGG
AGGTATGAAAATTGTATATGAAAAATCTCAACTAGCACCTAGA
'ITA_FJ005232_411_2006'
ATGAGTGATGGAGCAGTTCAACCAGACGGTGGTCAACCTGCTGTCAGAAATGAAAGAGCAACAGGATC
TGGGAACGGGTCTGGAGGCGGGGGTGGTGGTGGTTCTGGGGGTGTGGGGATTTCTACGGGTACTTTCA
ATAATCAGACGGAATTTAAATTTTTGGAAAACGGATGGGTGGAAATCACAGCAAACTCAAGCAGACTT
GTACATTTAAATATGCCAGAAAGTGAAAATTATAGAAGAGTGGTTGTAAATAATTTGGATAAAACTGC
AGTTAACGGAAACATGGCTTTAGATGATACTCATGCACAAATTGTAACACCTTGGTCATTGGTTGATG
CAAATGCTTGGGGAGTTTGGTTTAATCCAGGAGATTGGCAACTAATTGTTAATACTATGAGTGAGTTG
CATTTAGTTAGTTTTGAACAAGAAATTTTTAATGTTGTTTTAAAGACTGTTTCAGAATCTGCTACTCA
GCCACCAACTAAAGTTTATAATAATGATTTAACTGCATCATTGATGGTTGCATTAGATAGTAATAATA
CTATGCCATTTACTCCAGCAGCTATGAGATCTGAGACATTGGGTTTTTATCCATGGAAACCAACCATA
CCAACTCCATGGAGATATTATTTTCAATGGGATAGAACATTAATACCATCTCATACTGGAACTAGTGG
CACACCAACAAATATATACCATGGTACAGATCCAGATGATGTTCAATTTTATACTATTGAAAATTCTG
TGCCAGTACACTTACTAAGAACAGGTGATGAATTTGCTACAGGAACATTTTTTTTTGATTGTAAACCA
TGTAGACTAACACATACATGGCAAACAAATAGAGCATTGGGCTTACCACCATTTCTAAATTCTTTGCC
TCAAGCTGAAGGAGGTACTAACTTTGGTTATATAGGAGTTCAACAAGATAAAAGACGTGGTGTAACTC
AAATGGGAAATACAAACTATATTACTGAAGCTACTATTATGAGACCAGCTGAGGTTGGTTATAGTGCA
CCATATTATTCTTTTGAGGCGTCTACACAAGGGCCATTTAAAACACCTATTGCAGCAGGACGGGGGGG
AGCGCAAACAGATGAAAATCAAGCAGCAGATGGTGATCCAAGATATGCATTTGGTAGACAACATGGTC
AAAAAACTACCACAACAGGAGAAACACCTGAGAGATTTACATATATAGCACATCAAGATACAGGAAGA
TATCCAGAAGGAGATTGGATTCAAAATATTAACTTTAACCTTCCTGTAACGGAAGATAATGTATTGCT
ACCAACAGATCCAATTGGAGGTAAAACAGGAATTAACTATACTAATATATTTAATACTTATGGTCCTT
TAACTGCATTAAATAATGTACCACCAGTTTATCCAAATGGTCAAATTTGGGATAAAGAATTTGATACT
GACTTAAAACCAAGACTTCATGTAAATGCACCATTTGTTTGTCAAAATAATTGTCCTGGTCAATTATT
TGTAAAAGTTGCGCCTAATTTAACGAATGAATATGATCCTGATGCATCTGCTAATATGTCAAGAATTG
TAACTTACTCAGATTTTTGGTGGAAAGGTAAATTAGTATTTAAAGCTAAACTAAGAGCCTCTCATACT
TGGAATCCAATTCAACAAATGAGTATTAATGTAGATAACCAATTTAACTATGTACCAAGTAATATTGG
AGGTATGAAAATTGTATATGAAAAATCTCAACTAGCACCTAGA
'ITA_FJ005233_40_2007'
ATGAGTGATGGAGCAGTTCAACCAGACGGTGGTCAACCTGCTGTCAGAAATGAAAGAGCAACAGGATC
TGGGAACGGGTCTGGAGGCGGGGGTGGTGGTGGTTCTGGGGGTGTGGGGATTTCTACGGGTACTTTCA
ATAATCAGACGGAATTTAAATTTTTGGAAAACGGATGGGTGGAAATCACAGCAAACTCAAGCAGACTT
GTACATTTAAATATGCCAGAAAGTGAAAATTATAGAAGAGTGGTTGTAAATAATTTGGATAAAACTGC
AGTTAACGGAAACATGGCTTTAGATGATACTCATGCACAAATTGTAACACCTTGGTCATTGGTTGATG
CAAATGCTTGGGGAGTTTGGTTTAATCCAGGAGATTGGCAACTAATTGTTAATACTATGAGTGAGTTG
CATTTAGTTAGTTTTGAACAAGAAATTTTTAATGTTGTTTTAAAGACTGTTTCAGAATCTGCTACTCA
GCCACCGACTAAAGTTTATAATAATGATTTAACTGCATCATTGATGGTTGCATTAGATAGTAATAATA
CTATGCCATTTACTCCAGCAGCTATGAGATCTGAGACATTGGGTTTTTATCCATGGAAACCAACCATA
CCAACTCCATGGAGATATTATTTTCAATGGGATAGAACATTAATACCATCTCATACTGGAACTAGTGG
CACACCAACAAATATATACCATGGTACAGATCCAGATGATGTTCAATTTTATACTATTGAAAATTCTG
TGCCAGTACACTTACTAAGAACAGGTGATGAATTTGCTACAGGAACATTTTTTTTTGATTGTAAACCA
TGTAGACTAACACATACATGGCAAACAAATAGAGCATTGGGCTTACCACCATTTCTAAATTCTTTGCC
TCAAGCTGAAGGAGGTACTAACTTTGGTTATATAGGAGTTCAACAAGATAAAAGACGTGGTGTAACTC
AAATGGGAAATACAAACTATATTACTGAAGCTACTATTATGAGACCAGCTGAGGTTGGTTATAGTGCA
CCATATTATTCTTTTGAGGCGTCTACACAAGGGCCATTTAAAACACCTATTGCAGCAGGACGGGGGGG
AGCGCAAACAGATGAAAATCAAGCAGCAGATGGTGATCCAAGATATGCATTTGGTAGACAACATGGTC
AAAAAACTACCACAACAGGAGAAACACCTGAGAGATTTACATATATAGCACATCAAGATACAGGAAGA
TATCCAGAAGGAGATTGGATTCAAAATATTAACTTTAACCTTCCTGTAACAGAAGATAATGTATTGCT
ACCAACAGATCCAATTGGAGGTAAAACAGGAATTAACTATACTAATATATTTAATACTTATGGTCCTT
TAACTGCATTAAATAATGTACCACCAGTTTATCCAAATGGTCAAATTTGGGATAAAGAATTTGATACT
GACTTAAAACCAAGACTTCATGTAAATGCACCATTTGTTTGTCAAAATAATTGTCCTGGTCAATTATT
TGTAAAAGTTGCGCCTAATTTAACAAATGAATATGATCCTGATGCATCTGCTAATATGTCAAGAATTG
TAACTTACTCAGATTTTTGGTGGAAAGGTAAATTAGTATTTAAAGCTAAACTAAGAGCCTCTCATACT

TGGAATCCAATTCAACAAATGAGTATTAATGTAGATAACCAATTTAACTATGTACCAAGTAATATTGG
AGGTATGAAAATTGTATATGAAAAATCTCAACTAGCACCTAGA
'ITA_FJ005240_208_2007'
ATGAGTGATGGAGCAGTTCAACCAGACGGTGGTCAACCTGCTGTCAGAAATGAAAGAGCAACAGGATC
TGGGAACGGGTCTGGAGGCGGGGGTGGTGGTGGTTCTGGGGGTGTGGGGATTTCTACGGGTACTTTCA
ATAATCAGACGGAATTTAAATTTTTGGAAAACGGATGGGTGGAAATCACAGCAAACTCAAGCAGACTT
GTACATTTAAATATGCCAGAAAGTGAAAATTATAGAAGAGTGGTTGTAAATAATTTGGATAAAACTGC
AGTTAACGGAAACATGGCTTTAGATGATACTCATGCACAAATTGTAACACCTTGGTCATTGGTTGATG
CAAATGCTTGGGGAGTTTGGTTTAATCCAGGAGATTGGCAACTAATTGTTAATACTATGAGTGAGTTG
CATTTAGTTAGTTTTGAACAAGAAATTTTTAATGTTGTTTTAAAGACTGTTTCAGAATCTGCTACTCA
GCCACCAACTAAAGTTTATAATAATGATTTAACTGCATCATTGATGGTTGCATTAGATAGTAATAATA
CTATGCCATTTACTCCAGCAGCTATGAGATCTGAGACATTGGGTTTTTATCCATGGAAACCAACCATA
CCAACTCCATGGAGATATTATTTTCAATGGGATAGAACATTAATACCATCTCATACTGGAACTAGTGG
CACACCAACAAATATATACCATGGTACAGATCCAGATGATGTTCAATTTTATACTATTGAAAATTCTG
TGCCAGTACACTTACTAAGAACAGGTGATGAATTTGCTACAGGAACATTTTTTTTTGATTGTAAACCA
TGTAAACTAACACATACATGGCAAACAAATAGAGCATTGGGCTTACCACCATTTCTAAATTCTTTGCC
TCAAGCTGAAGGAGGTACTAACTTTGGTTATATAGGAGTTCAACAAGATAAAAGACGTGGTGTAACTC
AAATGGGAAATACAAACTATATTACTGAAGCTACTATTATGAGACCAGCTGAGGTTGGTTATAGTGCA
CCATATTATTCTTTTGAGGCGTCTACACAAGGGCCATTTAAAACACCTATTGCAGCAGGACGGGGGGG
AGCGCAAACAGATGAAAATCAAGCAGCAGATGGTGATCCAAGATATGCATTTGGTAGACAACATGGTC
AAAAAACTACCACAACAGGAGAAACACCTGAGAGATTTACATATATAGCACATCAAGATACAGGAAGA
TATCCAGAAGGAGATTGGATTCAAAATATTAACTTTAACCTTCCTGTAACAGAAGATAATGTATTGCT
ACCAACAGATCCAATTGGAGGTAAAACAGGAATTAACTATACTAATATATTTAATACTTATGGTCCTT
TAACTGCATTAAATAATGTACCACCAGTTTATCCAAATGGTCAAATTTGGGATAAAGAATTTGATACT
GACTTAAAACCAAGACTTCATGTAAATGCACCATTTGTTTGTCAAAATAATTGTCCTGGTCAATTATT
TGTAAAAGTTGCGCCTAATTTAACAAATGAATATGATCCTGATGCATCTGCTAATATGTCAAGAATTG
TAACTTACTCAGATTTTTGGTGGAAAGGTAAATTAGTATTTAAAGCTAAACTAAGAGCCTCTCATACT
TGGAATCCAATTCAACAAATGAGTATTAATGTAGATAACCAATTTAACTATGTACCAAGTAATATTGG
AGGTATGAAAATTGTATATGAAAAATCTCAACTAGCACCTAGA
'ITA_FJ005247_195_2008'
ATGAGTGATGGAGCAGTTCAACCAGACGGTGGTCAACCTGCTGTCAGAAATGAAAGAGCAACAGGATC
TGGGAACGGGTCTGGAGGCGGGGGTGGTGGTGGTTCTGGGGGTGTGGGGATTTCTACGGGTACTTTCA
ATAATCAGACGGAATTTAAATTTTTGGAAAACGGATGGGTGGAAATCACAGCAAACTCAAGCAGACTT
GTACATTTAAATATGCCAGAAAGTGAAAATTATAGAAGAGTGGTTGTAAATAATTTGGATAAAACTGC
AGTTAACGGAAACATGGCTTTAGATGATACTCATGCACAAATTGTAACACCTTGGTCATTGGTTGATG
CAAATGCTTGGGGAGTTTGGTTTAATCCAGGAGATTGGCAACTAATTGTTAATACTATGAGTGAGTTG
CATTTAGTTAGTTTTGAACAAGAAATTTTTAATGTTGTTTTAAAGACTGTTTCAGAATCTGCTACTCA
GCCACCAACTAAAGTTTATAATAATGATTTAACTGCATCATTGATGGTTGCATTAGATAGTAATAATA
CTATGCCATTTACTCCAGCAGCTATGAGATCTGAGACATTGGGTTTTTATCCATGGAAACCAACCATA
CCAACTCCATGGAGATATTATTTTCAATGGGATAGAACATTAATACCATCTCATACTGGAACTAGTGG
CACACCAACAAATATATACCATGGTACAGATCCAGATGATGTTCAATTTTATACTATTGAAAATTCTG
TGCCAGTACACTTACTAAGAACAGGTGATGAATTTGCTACAGGAACATTTTTTTTTGATTGTAAACCA
TGTAGACTAACACATACATGGCAAACAAATAGAGCATTGGGCTTACCACCATTTCTAAATTCTTTGCC
TCAAGCTGAAGGAGGTACTAACTTTGGTTATATAGGAGTTCAACAAGATAAAAGACGTGGTGTAACTC
AAATGGGAAATACAAACTATATTACTGAAGCTACTATTATGAGACCAGCTGAGGTTGGTTATAGTGCA
CCATATTATTCTTTTGAGGCGTCTACACAAGGGCCATTTAAAACACCTATTGCAGCAGGACGGGGGGG
AGCGCAAACAGATGAAAATCAAGCAGCAGATGGTGATCCAAGATATGCATTTGGTAGACAACATGGTC
AAAAAACTACCACAACAGGAGAAACACCTGAGAGATTTACATATATAGCACATCAAGATACAGGAAGA
TATCCAGAAGGAGATTGGATTCAAAATATTAACTTTAACCTTCCTGTAACAGAAGATAATGTATTGCT
ACCAACAGATCCAATTGGAGGTAAAACAGGAATTAACTATACTAATATATTTAATACTTATGGTCCTT
TAACTGCATTAAACAATGTACCACCAGTTTATCCAAATGGTCAAATTTGGGATAAAGAATTTGATACT
GACTTAAAACCAAGACTTCATGTAAATGCACCATTTGTTTGTCAAAATAATTGTCCTGGTCAATTATT
TGTAAAAGTTGCGCCTAATTTAACAAATGAATATGATCCTGATGCATCTGCTAATATGTCAAGAATTG
TAACTTACTCAGATTTTTGGTGGAAAGGTAAATTAGTATTTAAAGCTAAACTAAGAGCCTCTCATACT

TGGAATCCAATTCAACAAATGAGTATTAATGTAGATAACCAATTTAACTATGTACCAAGTAATATTGG
AGGTATGAAAATTGTATATGAAAAATCTCAACTAGCACCTAGA
'ITA_FJ005248_219_2008'
ATGAGTGATGGAGCAGTTCAACCAGACGGTGGTCAACCTGCTGTCAGAAATGAAAGAGCAACAGGATC
TGGGAACGGGTCTGGAGGCGGGGGTGGTGGTGGTTCTGGGGGTGTGGGGATTTCTACGGGTACTTTCA
ATAATCAGACGGAATTTAAATTTTTGGAAAACGGATGGGTGGAAATCACAGCAAACTCAAGCAGACTT
GTACATTTAAATATGCCAGAAAGTGAAAATTATAGAAGAGTGGTTGTAAATAATTTGGATAAAACTGC
AGTTAACGGAAACATGGCTTTAGATGATACTCATGCACAAATTGTAACACCTTGGTCATTGGTTGATG
CAAATGCTTGGGGAGTTTGGTTTAATCCAGGAGATTGGCAACTAATTGTTAATACTATGAGTGAGTTG
CATTTAGTTAGTTTTGAACAAGAAATTTTTAATGTTGTTTTAAAGACTGTTTCAGAATCTGCTACTCA
GCCACCAACTAAAGTTTATAATAATGATTTAACTGCATCATTGATGGTTGCATTAGATAGTAATAATA
CTATGCCATTTACTCCAGCAGCTATGAGATCTGAGACATTGGGTTTTTATCCATGGAAACCAACCATA
CCAACTCCATGGAGATATTATTTTCAATGGGATAGAACATTAATACCATCTCATACTGGAACTAGTGG
CACACCAACAAATATATACCATGGTACAGATCCAGATGATGTTCAATTTTATACTATTGAAAATTCTG
TGCCAGTACACTTACTAAGAACAGGTGATGAATTTGCTACAGGAATATTTTTTTTTGATTGTAAACCA
TGTAGACTAACACATACATGGCAAACAAATAGAGCATTGGGCTTACCACCATTTCTAAATTCTTTGCC
TCAAGCTGAAGGAGGTACTAACTTTGGTTATATAGGAGTTCAACAAGATAAAAGACGTGGTGTAACTC
AAATGGGAAATACAAACTATATTACTGAAGCTACTATTATGAGACCAGCTGAGGTTGGTTATAGTGCA
CCATATTATTCTTTTGAGGCGTCTACACAAGGGCCATTTAAAACACCTATTGCAGCAGGACGGGGGGG
AGCGCAAACAGATGAAAATCAAGCAGCAGATGGTGATCCAAGATATGCATTTGGTAGACAACATGGTC
AAAAAACTACCACAACAGGAGAAACACCTGAGAGATTTACATATATAGCACATCAAGATACAGGAAGA
TATCCAGAAGGAGATTGGATTCAAAATATTAACTTTAACCTTCCTGTAACAGAAGATAATGTATTGCT
ACCAACAGATCCAATTGGAGGTAAAACAGGAATTAACTATACTAATATATTTAATACTTATGGTCCTT
TAACTGCATTAAATAATGTACCACCAGTTTATCCAAATGGTCAAATTTGGGATAAAGAATTTGATACT
GACTTAAAACCAAGACTTCATGTAAATGCACCATTTGTTTGTCAAAATAATTGTCCTGGTCAATTATT
TGTAAAAGTTGCGCCTAATTTAACAAATGAATATGATCCTGATGCATCTGCTAATATGTCAAGAATTG
TAACTTACTCAGATTTTTGGTGGAAAGGTAAATTAGTATTTAAAGCTAAACTAAGAGCCTCTCATACT
TGGAATCCAATTCAACAAATGAGTATTAATGTAGATAACCAATTTAACTATGTACCAAGTAATATTGG
AGGTATGAAAATTGTATATGAAAAATCTCAACTAGCACCTAGA
'ITA_FJ005251_239_2008'
ATGAGTGATGGAGCAGTTCAACCAGACGGTGGTCAACCTGCTGTCAGAAATGAAAGAGCAACAGGATC
TGGGAACGGGTCTGGAGGCGGGGGTGGTGGTGGTTCTGGGGGTGTGGGGATTTCTACGGGTACTTTCA
ATAATCAGACGGAATTTAAATTTTTGGAAAACGGATGGGTGGAAATCACAGCAAACTCAAGCAGACTT
GTACATTTAAATATGCCAGAAAGTGAAAATTATAGAAGAGTGGTTGTAAATAATTTGGATAAAACTGC
AGTTAACGGAAACATGGCTTTAGATGATACTCATGCACAAATTGTAACACCTTGGTCATTGGTTGATG
CAAATGCTTGGGGAGTTTGGTTTAATCCAGGAGATTGGCAACTAATTGTTAATACTATGAGTGAGTTG
CATTTAGTTAGTTTTGAACAAGAAATTTTTAATGTTGTTTTAAAGACTGTTTCAGAATCTGCTACTCA
GCCACCAACTAAAGTTTATAATAATGATTTAACTGCATCATTGATGGTTGCATTAGATAGTAATAATA
CTATGCCATTTACTCCAGCAGCTATGAGATCTGAGACATTGGGTTTCTATCCATGGAAACCAACCATA
CCAACTCCATGGAGATATTATTTTCAATGGGATAGAACATTAATACCATCTCATACTGGAACTAGTGG
CACACCAACAAATATATACCATGGTACAGATCCAGATGATGTTCAATTTTATACTATTGAAAATTCTG
TGCCAGTACACTTACTAAGAACAGGTGATGAATTTGCTACAGGAACATTTTTTTTTGATTGTAAACCA
TGTAGACTAACACATACATGGCAAACAAATAGAGCATTGGGCTTACCACCATTTCTAAATTCTTTGCC
TCAAGCTGAAGGAGGTACTAACTTTGGTTATATAGGAGTTCAACAAGATAAAAGACGTGGTGTAACTC
AAATGGGAAATACAAACTATATTACTGAAGCTACTATTATGAGACCAGCTGAGGTTGGTTATAGTGCA
CCATATTATTCTTTTGAGGCGTCTACACAAGGGCCATTTAAAACACCTATTGCAGCAGGACGGGGGGG
AGCGCAAACGGATGAAAATCAAGCAGCAGATGGTGATCCAAGATATGCATTTGGTAGACAACATGGTC
AAAAAACTACCACAACAGGAGAAACACCTGAGAGATTTACATATATAGCACATCAAGATACAGGAAGA
TATCCAGAAGGAGATTGGATTCAAAATATTAACTTTAACCTTCCTGTAACAGAAGATAATGTATTGCT
ACCAACAGATCCAATTGGAGGTAAAACAGGAATTAACTATACTAATATATTTAATACTTATGGTCCTT
TAACTGCATTAAATAATGTACCACCAGTTTATCCAAATGGTCAAATTTGGGATAAAGAATTTGATACT
GACTTAAAACCAAGACTTCATGTAAATGCACCATTTGTTTGTCAAAATAATTGTCCTGGTCAATTATT
TGTAAAAGTTGCGCCTAATTTAACAAATGAATATGATCCTGATGCATCTGCTAATATGTCAAGAATTG
TAACTTACTCAGATTTTTGGTGGAAAGGTAAATTAGTATTTAAAGCTAAACTAAGAGCCTCTCATACT

TGGAATCCAATTCAACAAATGAGTATTAATGTAGATAACCAATTTAACTATGTACCAAGTAATATTGG
AGGTATGAAAATTGTATATGAAAAATCTCAACTAGCACCTAGA

'ITA_FJ005252_96_2002'

ATGAGTGATGGAGCAGTTCAACCAGACGGTGGTCAACCTGCTGTCAGAAATGAAAGAGCTACAGGATC
TGGGAACGGGTCTGGAGGCGGGGGTGGTGGTGGTTCTGGGGGTGTGGGGATTTCTACGGGTACTTTCA
ATAATCAGACGGAATTTAAATTTTTGGAAAACGGATGGGTGGAAATCACAGCAAACTCAAGCAGACTT
GTACATTTAAATATGCCAGAAAGTGAAAATTATAGAAGAGTGGTTGTAAATAATTTGGATAAAACTGC
AGTTAACGGAAACATGGCTTTAGATGATACTCATGCACAAATTGTAACACCTTGGTCATTGGTTGATG
CAAATGCTTGGGGAGTTTGGTTTAATCCAGGAGATTGGCAACTAATTGTTAATACTATGAGTGAGTTG
CATTTAGTTAGTTTTGAACAAGAAATTTTTAATGTTGTTTTAAAGACTGTTTCAGAATCTGCTACTCA
GCCACCAACTAAAGTTTATAATAATGATTTAACTGCATCATTGATGGTTGCATTAGATAGCAATAATA
CTATGCCATTTACTCCAGCAGCTATGAGATCTGAGACATTGGGTTTTTATCCATGGAAACCAACCATA
CCAACTCCATGGAGATATTATTTTCAATGGGATAGAACATTAATACCATCTCATACTGGAACTAGTGG
CACACCAACAAATATATACCATGGTACAGATCCAGATGATGTTCAATTTTATACTATTGAAAATTCTG
TGCCAGTACACTTACTAAGAACAGGTGATGAATTTGCTACAGGAACATTTTTTTTTGATTGTAAACCA
TGTAGACTAACACATACATGGCAAACAAATAGAGCATTGGGCTTACCACCATTTCTAAATTCTTTGCC
TCAAGCTGAAGGAGGTACTAACTTTGGTTATATAGGAGTTCAACAAGATAAAAGACGTGGTGTAACTC
AAATGGGAAATACAAACTATATTACTGAAGCTACTATTATGAGACCAGCTGAGGTTGGTTATAGTGCA
CCATATTATTCTTTTGAGGCGTCTACACAAGGGCCATTTAAAACACCTATTGCAGCAGGACGGGGGGG
AGCACAAACAGATGAAAATCAAGCAGCAGATGGTGATCCAAGATATGCATTTGGTAGACAACATGGTC
AAAAAACTACCACAACAGGAGAAACACCTGAGAGATTTACATATATAGCACATCAAGATACAGGAAGA
TACCCAGAAGGAGATTGGATTCAAAATATTAACTTTAACCTTCCTGTAACAAATGATAATGTATTGCT
ACCAACAGATCCAATTGGAGGTAAAACAGGAATTAACTATACTAATATATTTAATACTTATGGTCCTT
TAACTGCATTAAATAATGTACCACCAGTTTATCCAAATGGTCAAATTTGGGATAAAGAATTTGATACT
GACTTAAAACCAAGACTTCATGTAAATGCACCATTTGTTTGTCAAAATAATTGTCCTGGTCAATTATT
TGTAAAAGTTGCGCCTAATTTAACAAATGAATATGATCCTGATGCATCTGCTAATATGTCAAGAATTG
TAACTTACTCAGATTTTTGGTGGAAAGGTAAATTAGTATTTAAAGCTAAACTAAGAGCCTCTCATACT
TGGAATCCAATTCAACAAATGAGTATTAATGTAGATAACCAATTTAACTATGTACCAAGTAATATTGG
AGGTATGAAGATTGTATATGAAAAATCTCAACTAGCACCTAGA

'ITA_FJ005253_67_2005'

ATGAGTGATGGAGCAGTTCAACCAGACGGTGGTCAACCTGCTGTCAGAAATGAAAGAGCTACAGGATC
TGGGAACGGGTCTGGAGGCGGGGGTGGTGGTGGTTCTGGGGGTGTGGGGATTTCTACGGGTACTTTCA
ATAATCAGACGGAATTTAAATTTTTGGAAAACGGATGGGTGGAAATCACAGCAAACTCAAGCAGACTT
GTACATTTAAATATGCCAGAAAGTGAAAATTATAGAAGAGTGGTTGTAAATAATTTGGATAAAACTGC
AGTTAACGGAAACATGGCTTTAGATGATACTCATGCACAAATTGTAACACCTTGGTCATTGGTTGATG
CAAATGCTTGGGGAGTTTGGTTTAATCCAGGAGATTGGCAACTAATTGTTAATACTATGAGTGAGTTG
CATTTAGTTAGTTTTGAACAAGAAATTTTTAATGTTGTTTTAAAGACTGTTTCAGAATCTGCTACTCA
GCCACCAACTAAAGTTTATAATAATGATTTAACTGCATCATTGATGGTTGCATTAGATAGCAATAATA
CTATGCCATTTACTCCAGCAGCTATGAGATCTGAGACATTGGGTTTTTATCCATGGAAACCAACCATA
CCAACTCCATGGAGATATTATTTTCAATGGGATAGAACATTAATACCATCTCATACTGGAACTAGTGG
CACACCAACAAATATATACCATGGTACAGATCCAGATGATGTTCAATTTTATACTATTGAAAATTCTG
TGCCAGTACACTTACTAAGAACAGGTGATGAATTTGCTACAGGAACATTTTTTTTTGATTGTAAACCA
TGTAGACTAACACATACATGGCAAACAAATAGAGCATTGGGCTTACCACCATTTCTAAATTCTTTGCC
TCAAGCTGAAGGAGGTACTAACTTTGGTTATATAGGAGTTCAACAAGATAAAAGACGTGGTGTAACTC
AAATGGGAAATACAAACTATATTACTGAAGCTACTATTATGAGACCAGCTGAGGTTGGTTATAGTGCA
CCATATTATTCTTTTGAGGCGTCTACACAAGGGCCATTTAAAACACCTATTGCAGCAGGACGGGGGGG
AGCGCAAACAGATGAAAATCAAGCAGCAGATGGTGATCCAAGATATGCATTTGGTAGACAACATGGTC
AAAAAACTACCACAACAGGAGAAACACCTGAGAGATTTACATATATAGCACATCAAGATACAGGAAGA
TATCCAGAAGGAGATTGGATTCAAAATACTAACTTTAACCTTCCTGTAACAAATGATAATGTATTGCT
ACCAACAGATCCAATTGAAGGTAAAACAGGAATTAACTATACTAATATATTTAATACTTATGGTCCTT
TAACTGCATTAAATAATGTACCACCAGTTTATCCAAATGGTCAAATTTGGGATAAAGAATTTGATACT
GACTTAAAACCAAGACTTCATGTAAATGCACCATTTGTTTGTCAAAATAATTGTCCTGGTCAATTATT
TGTAAAAGTTGCGCCTAATTTAACAAATGAATATGATCCTGATGCATCTGCTAATATGTCAAGAATTG
TAACTTACTCAGATTTTTGGTGGAAAGGTAAATTAGTATTTAAAGCTAAACTAAGAGCCTCTCATACT

TGGAATCCAATTCAACAAATGAGTATTAATGTAGATAACCAATTTAACTATGTACCAAGTAATATTGG
AGGTATGAAGATTGTATATGAAAAATCTCAACTAGCACCTAGA

'ITA_FJ005255_333_2005'

ATGAGTGATGGAGCAGTTCAACCAGACGGTGGTCAGCCTGCTGTCAGAAATGAAAGAGCTACAGGATC
TGGGAACGGGTCTGGAGGCGGGGGTGGTGGTGGTTCTGGGGGTGTGGGGATTTCTACGGGTACTTTCA
ATAATCAGACGGAATTTAAATTTTTGGAAAACGGATGGGTGGAAATCACAGCAAACTCAAGCAGACTT
GTACATTTAAATATGCCAGAAAGTGAAAATTATAGAAGAGTGGTTGTAAATAATTTGGATAAAACTGC
AGTTAACGGAAACATGGCTTTAGATGATACTCATGCACAAATTGTAACACCTTGGTCATTGGTTGATG
CAAATGCTTGGGGAGTTTGGTTTAATCCAGGAGATTGGCAACTAATTGTTAATACTATGAGTGAGTTG
CATTTAGTTAGTTTTGAACAAGAAATTTTTAATGTTGTTTTAAAGACTGTTTCAGAATCTGCTACTCA
GCCACCAACTAAAGTTTATAATAATGATTTAACTGCATCATTGATGGTTGCATTAGATAGCAATAATA
CTATGCCATTTACTCCAGCAGCTATGAGATCTGAGACATTAGGTTTTTATCCATGGAAACCAACCATA
CCAACTCCATGGAGATATTATTTTCAATGGGATAGAACATTAATACCATCTCATACTGGAACTAGTGG
CACACCAACAAATATATACCATGGTACAGATCCAGATGATGTTCAATTTTATACTATTGAAAATTCTG
TGCCAGTACACTTACTAAGAACAGGTGATGAATTTGCTACAGGAACATTTTTTTTTGATTGTAAACCA
TGTAGACTAACACATACATGGCAAACAAATAGAGCATTGGGCTTACCACCATTTCTAAATTCTTTGCC
TCAAGCTGAAGGAGGTACTAACTTTGGTTATATAGGAGTTCAACAAGATAAAAGACGTGGTGTAACTC
AAATGGGAAATACAAACTACATTACTGAAGCTACTATTATGAGACCAGCTGAGGTTGGTTATAGTGCA
CCATATTATTCTTTTGAGGCGTCTACACAAGGGCCATTTAAAACACCTATTGCAGCAGGACGGGGGGG
AGCGCAAACAGATGAAAATCAAGCAGCAGATGGTGATCCAAGATATGCATTTGGTAGACAACATGGTC
AAAAAACTACCACAACAGGAGAAACACCTGAGAGATTTACATATATAGCACATCAAGATACAGGAAGA
TATCCAGAAGGAGATTGGATTCAAAATATTAACTTTAACCTTCCTGTAACAAATGATAATGTATTGCT
ACCAACAGATCCAATTGGAGGTAAAACAGGAATTAACTATACTAATATATTTAATACTTATGGTCCTT
TAACTGCATTAAATAATGTACCACCAGTTTATCCAAATGGTCAAATTTGGGATAAAGAATTTGATACT
GACTTAAAACCAAGACTTCATGTAAATGCACCATTTGTTTGTCAAAATAATTGTCCTGGTCAATTATT
TGTAAAAGTTGCGCCTAATTTAACAAATGAATATGATCCTGATGCATCTGCTAATATGTCAAGAATTG
TAACTTACTCAGATTTTTGGTGGAAAGGTAAATTAGTATTTAAAGCTAAACTAAGAGCCTCTCATACT
TGGAATCCAATTCAACAAATGAGTATTAATGTAGATAACCAATTTAACTATGTACCAAGTAATATTGG
AGGTATGAAGATTGTATATGAAAAATCTCAACTAGCACCTAGA

'ITA_FJ005257_54_2008'

ATGAGTGATGGAGCAGTTCAACCAGACGGTGGTCAACCTGCTGTCAGAAATGAAAGAGCAACAGGATC
TGGGAACGGGTCTGGAGGCGGGGGTGGTGGTGGTTCTGGGGGTGTGGGGATTTCTACGGGTACTTTCA
ATAATCAGACGGAATTTAAATTTTTGGAAAACGGATGGGTGGAAATCACAGCAAACTCAAGCAGACTT
GTACATTTAAATATGCCAGAAAGTGAAAATTATAGAAGAGTGGTTGTAAATAATTTGGATAAAACTGC
AGTTAACGGAAACATGGCTTTAGATGATACTCATGCACAAATTGTAACACCTTGGTCATTGGTTGATG
CAAATGCTTGGGGAGTTTGGTTTAATCCAGGAGATTGGCAACTAATTGTTAATACTATGAGTGAGTTG
CATTTAGTTAGTTTTGAACAAGAAATTTTTAATGTTGTTTTAAAGACTGTTTCAGAATCTGCTACTCA
GCCACCAACTAAAGTTTATAATAATGATTTAACTGCATCATTGATGGTTGCATTAGATAGTAATAATA
CTATGCCATTTACTCCAGCAGCTATGAGATCTGAGACATTGGGTTTTTATCCATGGAAACCAACCATA
CCAACTCCATGGAGATATTATTTTCAATGGGATAGAACATTAATACCATCTCATACTGGAACTAGTGG
CACACCAACAAATATATACCATGGTACAGATCCAGATGATGTTCAATTTTATACTATTGAAAATTCTG
TGCCAGTACACTTACTAAGAACAGGTGATGAATTTGCTACAGGAACATTTTTTTTTGATTGTAAACCA
TGTAGACTAACACATACATGGCAAACAAATAGAGCATTGGGCTTACCACCATTTCTAAATTCTTTGCC
TCAAGCTGAAGGAGGTACTAACTTTGGTTATATAGGAGTTCAACAAGATAAAAGACGTGGTGTAACTC
AAATGGGAAATACAAACTATATTACTGAAGCTACTATTATGAGACCAGCTGAGGTTGGTTATAGTGCA
CCATATTATTCTTTTGAGGCGTCTACACAAGGGCCATTTAAAACACCTATTGCAGCAGGACGGGGGGG
AGCGCAAACAGATGAAAATCAAGCAGCAGATGGTGATCCAAGATATGCATTTGGTAGACAACATGGTC
AAAAAACTACCACAACAGGAGAAACACCTGAGAGATTTACATATATAGCACATCAAGATACAGGAAGA
TATCCAGAAGGAGATTGGATTCAAAATATTAACTTTAACCTTCCTGTAACAAATGATAATGTATTGCT
ACCAACAGATCCAATTGGAGGTAAAACAGGAATTAACTATACTAATATATTTAATACTTATGGTCCTT
TAACTGCATTAAATAATGTACCACCAGTTTATCCAAATGGTCAAATTTGGGATAAAGAATTTGATACT
GACTTAAAACCAAGACTTCATGTAAATGCACCATTTGTTTGTCAAAATAATTGTCCTGGTCAATTATT
TGTAAAAGTTGCGCCTAATTTAACAAATGAATATGATCCTGATGCATCTGCTAATATGTCAAGAATTG
TAACTTACTCAGATTTTTGGTGGAAAGGTAAATTAGTATTTAAAGCTAAACTAAGAGCCTCTCATACT

TGGAATCCAATTCAACAAATGAGTATTAATGTAGATAACCAATTTAACTATGTACCAAGTAATATTGG
AGGTATGAAGATTGTATATGAAAAATCTCAACTAGCACCTAGA
'ITA_FJ005258_80_2008'
ATGAGTGATGGAGCAGTTCAACCAGACGGTGGTCAACCTGCTGTCAGAAATGAAAGAGCTACAGGATC
TGGGAACGGGTCTGGAGGCGGGGGTGGTGGTGGTTCTGGGGGTGTGGGGATTTCTACGGGTACTTTCA
ATAATCAGACGGAATTTAAATTTTTGGAAAACGGATGGGTGGAAATCACAGCAAACTCAAGCAGACTT
GTACATTTAAATATGCCAGAAAGTGAAAATTATAGAAGAGTGGTTGTAAATAATTTGGATAAAACTGC
AGTTAACGGAAACATGGCTTTAGATGATACCCATGCACAAATTGTAACACCTTGGTCATTGGTTGATG
CAAATGCTTGGGGAGTTTGGTTTAATCCAGGAGATTGGCAACTAATTGTTAATACTATGAGTGAGTTG
CATTTAGTTAGTTTTGAACAAGAAATTTTTAATGTTGTTTTAAAGACTGTTTCAGAATCTGCTACTCA
GCCACCAACTAAAGTTTATAATAATGATTTAACTGCATCATTGATGGTTGCATTAGATAGTAATAATA
CTATGCCATTTACTCCAGCAGCTATGAGATCTGAGACATTGGGTTTTTATCCATGGAAACCAACCATA
CCAACTCCATGGAGATATTATTTTCAATGGGATAGAACATTAATACCATCTCATACTGGAACTAGTGG
CACACCAACAAATATATACCATGGTACAGATCCAGATGATGTTCAATTTTATACTATTGAAAATTCTG
TGCCAGTACACTTACTAAGAACAGGTGATGAATTTGCTACAGGAACATTTTTTTTTGATTGTAAACCA
TGTAGACTAACACATACATGGCAAACAAATAGAGCATTGGGCTTACCACCATTTCTAAATTCTTTGCC
TCAAGCTGAAGGAGGTACTAACTTTGGTTATATAGGAGTTCAACAAGATAAAAGACGTGGTGTAACTC
AAATGGGAAATACAAACTATATTACTGAAGCTACTATTATGAGACCAGCTGAGGTTGGTTATAGTGCA
CCATATTATTCTTTTGAGGCGTCTACACAAGGGCCATTTAAAACACCTATTGCAGCAGGACGGGGGGG
AGCGCAAACAGATGAAAATCAAGCAGCAGATGGTGATCCAAGATATGCATTTGGTAGACAACATGGTC
AAAAAACTACCACAACAGGAGAAACACCTGAGAGATTTACATATATAGCACATCAAGATACAGGAAGA
TATCCAGAAGGAGATTGGATTCAAAATATTAACTTTAACCTTCCTGTAACAAATGATAATGTATTGCT
ACCAACAGATCCAATTGGAGGTAAAGCAGGAATTAACTATACTAATATATTTAATACTTATGGTCCTT
TAACTGCATTAAATAATGTACCACCAGTTTATCCAAATGGTCAAATTTGGGATAAAGAATTTGATACT
GACTTAAAACCAAGACTTCATGTAAATGCACCATTTGTTTGTCAAAATAATTGTCCTGGTCAATTATT
TGTAAAAGTTGCGCCTAATTTAACAAATGAATATGATCCTGATGCATCTGCTAATATGTCAAGAATTG
TAACTTACTCAGATTTTTGGTGGAAAGGTAAATTAGTATTTAAAGCTAAACTAAGAGCCTCTCATACT
TGGAATCCAATTCAACAAATGAGTATTAATGTAGATAACCAATTTAACTATGTACCAAGTAATATTGG
AGGTATGAAAATTGTATATGAAAAATCTCAACTAGCACCTAGA
'GER_FJ005260_G82_1997'
ATGAGTGATGGAGCAGTTCAACCAGACGGTGGTCAACCTGCTGTCAGAAATGAAAGAGCAACAGGATC
TGGGAACGGGTCTGGAGGCGGGGGTGGTGGTGGTTCTGGGGGTGTGGGGATTTCTACGGGTACTTTCA
ATAATCAGACAGAATTTAAATTTTTGGAAAACGGATGGGTGGAAATCACAGCAAACTCAAGCAGACTT
GTACATTTAAATATGCCAGAAAGTGAAAATTATAGAAGAGTGGTTGTAAATAATTTGGATAAAACTGC
AGTTAACGGAAACATGGCTTTAGATGATACTCATGCACAAATTGTAACACCTTGGTCATTGGTTGATG
CAAATGCTTGGGGAGTTTGGTTTAATCCAGGAGATTGGCAACTAATTGTTAATACTATGAGTGAGTTG
CATTTAGTTAGTTTTGAACAAGAAATTTTTAATGTTGTTTTAAAGACTGTTTCAGAATCTGCTACTCA
GCCACCAACTAAAGTTTATAATAATGATTTAACTGCATCATTGATGGTTGCATTAGATAGTAATAATA
CTATGCCATTTACTCCAGCAGCTATGAGATCTGAGACATTGGGTTTTTATCCATGGAAACCAACCATA
CCAACTCCATGGAGATATTATTTTCAATGGGATAGAACATTAATACCATCTCATACTGGAACTAGTGG
CACACCAACAAATATATACCATGGTACAGATCCAGATGATGTTCAATTTTATACTATTGAAAATTCTG
TGCCAGTACACTTACTAAGAACAGGTGATGAATTTGCTACAGGAACATTTTTTTTTGATTGTAAACCA
TGTAGACTAACACATACATGGCAAACAAATAGAGCATTGGGCTTACCACCATTTCTAAATTCTTTGCC
TCAAGCTGAAGGAGGTACTAACTTTGGTTATATAGGAGTTCAACAAGATAAAAGACGTGGTGTAACTC
AAATGGGAAATACAAACTATATTACTGAAGCTACTATTATGAGACCAGCTGAGGTTGGTTATAGTGCA
CCATATTATTCTTTTGAGGCGTCTACACAAGGGCCATTTAAAACACCTATTGCAGCAGGACGGGGGGG
AGCGCAAACAGATGAAAATCAAGCAGCAGATGGTGATCCAAGATATGCATTTGGTAGACAACATGGTC
AAAAAACTACCACAACAGGAGAAACACCTGAGAGATTTACATATATAGCACATCAAGATACAGGGAGA
TATCCAGAAGGAGATTGGATTCAAAATATTAACTTTAACCTTCCTGTAACAGATGATAATGTATTGCT
ACCAACAGATCCAATTGGAGGTAAAACAGGAATTAACTATACTAATATATTTAATACTTATGGTCCTT
TAACTGCATTAAATAATGTACCACCAGTTTATCCAAATGGTCAAATTTGGGATAAAGAATTTGATACT
GACTTAAAACCAAGACTTCATGTAAATGCACCATTTGTTTGTCAAAATAATTGCCCTGGTCAATTATT
TGTAAAAGTTGCGCCTAATTTAACAAATGAATATGATCCTGATGCATCTGCTAATATGTCAAGAATTG
TAACTTACTCAGATTTTTGGTGGAAAGGTAAATTAGTATTTAAAGCTAAACTAAGAGCCTCTCATACT

TGGAATCCAATTCAACAAATGAGTATTAATGTAGATAACCAATTTAACTATGTACCAAGTAATATTGG
AGGTATGAAAATTGTATATGAAAAATCTCAACTAGCACCTAGA
'GER_FJ005261_G162_1997'
ATGAGTGATGGAGCAGTTCAACCAGACGGTGGTCAACCTGCTGTCAGAAATGAAAGAGCTACAGGATC
TGGGAACGGGTCTGGAGGCGGGGGTGGTGGTGGTTCTGGGGGTGTGGGGATTTCTACGGGTACTTTCA
ATAATCAAACGGAATTTAAATTTTTGGAAAACGGATGGGTGGAAATCACAGCAAACTCAAGCAGACTT
GTACATTTAAATATGCCAGAAAGTGAAAATTATAGAAGAGTGGTTGTAAATAATTTGGATAAAACTGC
AGTTAACGGAAACATGGCTTTAGATGATACTCATGCACAAATTGTAACACCTTGGTCATTGGTTGATG
CAAATGCTTGGGGAGTTTGGTTTAATCCAGGAGATTGGCAACTAATTGTTAATACTATGAGTGAGTTG
CATTTAGTTAGTTTTGAACAAGAAATTTTTAATGTTGTTTTAAAGACTGTTTCAGAATCTGCTACTCA
GCCACCAACTAAAGTTTATAATAATGACTTAACTGCATCATTGATGGTTGCATTAGATAGTAATAATA
CTATGCCATTTACTCCAGCAGCTATGAGATCTGAGACATTGGGTTTTTATCCATGGAAACCAACCATA
CCAACTCCATGGAGATATTATTTTCAATGGGATAGAACATTAATACCATCTCATACTGGAACTAGTGG
CACACCAACAAATATATACCATGGTACAGATCCAGATGATGTTCAATTTTATACTATTGAAAATTCTG
TGCCAGTACACTTACTAAGAACAGGTGATGAATTTGCTACAGGAACATTTTTTTTTGATTGTAAACCA
TGTAGACTAACACATACATGGCAAACAAATAGAGCATTGGGCTTACCACCATTTCTAAATTCTTTGCC
TCAATCTGAAGGAGGTACTAACTTTGGTTATATAGGAGTTCAACAAGATAAAAGACGTGGTGTAACTC
AAATGGGAAATACAAACTATATTACTGAAGCTACTATTATGAGACCAGCTGAGGTTGGTTATAGTGCA
CCATATTATTCTTTTGAGGCGTCTACACAAGGGCCATTTAAAACACCTATTGCAGCAGGACGGGGGGG
AGCGCAAACAGATGAAAATCAAGCAGCAGATGGTGATCCAAGATATGCATTTGGTAGACAACATGGTC
AAAAAACTACCACAACAGGAGAAACACCTGAAAGATTTACATATATAGCACATCAAGATACAGGAAGA
TATCCAGAAGGAGATTGGATTCAAAATATTAACTTTAACCTTCCTGTAACAGATGATAATGTATTGCT
ACCAACAGATCCAATTGGAGGTAAAACAGGAATTAACTATACTAATATATTTAATACTTATGGTCCTT
TAACTGCATTAAATAATGTACCACCAGTTTATCCAAATGGTCAAATTTGGGATAAAGAATTTGATACT
GACTTAAAACCAAGACTTCATGTAAATGCACCATTTGTTTGTCAAAATAATTGTCCTGGTCAATTATT
TGTAAAAGTTGCGCCTAATTTAACAAATGAATATGATCCTGATGCATCTGCTAATATGTCAAGAATTG
TAACTTACTCAGATTTTTGGTGGAAAGGTAAATTAGTATTTAAAGCTAAACTAAGAGCCTCTCATACT
TGGAATCCAATTCAACAAATGAGTATTAATGTAGATAACCAATTTAACTATGTACCAAGTAATATTGG
AGGTATGAAAATTGTATATGAAAAATCTCAACTAGCACCTAGA
'ITA_FJ005263_42_2005'
ATGAGTGATGGAGCAGTTCAACCAGACGGTGGTCAAGCTGCTGTCAGAAATGAAAGAGCTACAGGATC
TGGGAACGGGTCTGGAGGCGGGGGTGGTGGTGGTTCTGGGGGTGTGGGGATTTCTACGGGTACTTTCA
ATAATCAGACGGAATTTAAATTTTTGGAAAACGGATGGGTGGAAATCACAGCAAACTCAAGCAGACTT
GTACATTTAAATATGCCAGAAAGTGAAAATTATAGAAGAGTGGTTGTAAATAATTTGGATAAAACTGC
AGTTAACGGAAACATGGCTTTAGATGATACTCATGCACAAATTGTAACACCTTGGTCATTGGTTGATG
CAAATGCTTGGGGAGTTTGGTTTAATCCAGGAGATTGGCAACTAATTGTTAATACTATGAGTGAGTTG
CATTTAGTTAGTTTTGAACAAGAAATTTTTAATGTTGTTTTAAAGACTGTTTCAGAATCTGCTACTCA
GCCACCAACTAAAGTTTATAATAATGATTTAACTGCATCATTGATGGTTGCATTAGATAGTAATAATA
CTATGCCATTTACTCCAGCAGCTATGAGATCTGAGACATTGGGTTTTTATCCATGGAAACCAACCATA
CCAACTCCATGGAGATATTATTTTCAATGGGATAGAACATTAATACCATCTCATACTGGAACTAGTGG
CACACCAACAAATATATACCATGGTACAGATCCAGATGATGTTCAATTTTATACTATTGAAAATTCTG
TGCCAGTACACTTACTAAGAACAGGTGATGAATTTGCTACAGGAACATTTTTTTTTGATTGTAAACCA
TGTAGACTAACACATACATGGCAAACAAATAGAGCATTGGGCTTACCACCATTTCTAAATTCTTTGCC
TCAAGCTGAAGGAGGTACTAACTTTGGTTATATAGGAGTTCAACAAGATAAAAGACGTGGTGTAACTC
AAATGGGAAATACAAACTATATTACTGAAGCTACTATTATGAGACCAGCTGAGGTTGGTTATAGTGCA
CCATATTATTCTTTTGAGGCGTCTACACAAGGGCCATTTAAAACACCTATTGCAGCAGGACGGGGGGG
AGCGCAAACAGATGAAAATCAAGCAGCAGATGGTGATCCAAGATATGCATTTGGTAGACAACATGGTC
AAAAAACTACCACAACAGGAGAAACACCTGAGAGATTTACATATATAGCACATCAAGATACAGGAAGA
TATCCAGAAGGAGATTGGATTCAAAATATTAACTTTAACCTTCCTGTAACAGATGATAATGTATTGCT
ACCAACAGATCCAATTGGAGGTAAAACAGGAATTAACTATACTAATATATTTAATACTTATGGTCCTT
TAACTGCATTAAATAATGTACCACCAGTTTATCCAAATGGTCAAATTTGGGATAAAGAATTTGATACT
GACTTAAAACCAAGACTTCATGTAAATGCACCATTTGTTTGTCAAAATAATTGTCCTGGTCAATTATT
TGTAAAAGTTGCGCCTAATTTAACAAATGAATATGATCCTGATGCATCTGCTAATATGTCAAGAATTG
TAACTTACTCAGATTTTTGGTGGAAAGGTAAATTAGTATTTAAAGCTAAACTAAGAGCCTCTCATACT

TGGAATCCAATTCAACAAATGAGTATTAATGTAGATAACCAATTTAACTATGTACCAAGTAATATTGG
AGGTATGAAAATTGTATATGAAAAATCTCAACTAGCACCTAGA
'ITA_FJ005264_134_2005'
ATGAGTGATGGAGCAGTTCAACCAGACGGTGGTCAACCTGCTGTCAGAAATGAAAGAGCTACAGGATC
TGGGAACGGGTCTGGAGGCGGGGGTGGTGGTGGTTCTGGGGGTGTGGGGATTTCTACGGGTACTTTCA
ATAATCAGACAGAATTTAAATTTTTGGAAAACGGATGGGTGGAAATCACAGCAAACTCAAGCAGACTT
GTACATTTAAATATGCCAGAAAGTGAAAATTATAGAAGAGTAGTTGTAAATAATTTGGATAAAACTGC
AGTTAACGGAAACATGGCTTTAGATGATACTCATGCACAAATTGTAACACCTTGGTCATTGGTTGATG
CAAATGCTTGGGGAGTTTGGTTTAATCCAGGAGATTGGCAACTAATTGTTAATACTATGAGTGAGTTG
CATTTAGTTAGTTTTGAACAAGAAATTTTTAATGTTGTTTTAAAGACTGTTTCAGAATCTGCTACTCA
GCCACCAACTAAAGTTTATAATAATGATTTAACTGCATCATTGATGGTTGCATTAGATAGTAATAATA
CTATGCCATTTACTCCAGCAGCTATGAGATCTGAGACATTGGGTTTTTATCCATGGAAACCAACCATA
CCAACTCCATGGAGATATTATTTTCAATGGGATAGAACATTAATACCATCTCATACTGGAACTAGTGG
CACACCAACAAATATATACCATGGTACAGATCCAGATGACGTTCAATTTTATACTATTGAAAATTCTG
TGCCAGTGCACTTACTAAGAACAGGTGATGAATTTGCTACAGGAACATTTTTTTTTGATTGTAAACCA
TGTAGACTAACACATACATGGCAAACAAATAGAGCACTGGGCTTACCACCATTTCTAAATTCTTTGCC
TCAAGCTGAAGGAGGTACTAACTTTGGTTATATAGGAGTTCAACAAGATAAAAGACGTGGTGTAACTC
AAATGGGAAATACAAACTATATTACTGAAGCTACTATTATGAGACCAGCTGAGGTTGGTTATAGTGCA
CCATATTATTCTTTTGAGGCGTCTACACAAGGGCCATTTAAAACACCTATTGCAGCAGGACGGGGGGG
AGCGCAAACAGATGAAAATCAAGCAGCAGATGGTGATCCAAGATATGCATTTGGTAGACAACATGGTC
AAAAAACTACCACAACAGGAGAAACACCTGAGAGATTTACATATATAGCACATCAAGATACAGGAAGA
TATCCAGAAGGAGATTGGATTCAAAATATTAACTTTAACCTTCCTGTAACAGATGATAATGTATTGCT
ACCAACAGATCCAATTGGAGGTAAAACAGGAATTAACTATACTAATATATTTAATACTTATGGTCCTT
TAACTGCATTAAATAATGTACCACCAGTTTATCCAAATGGTCAAATTTGGGATAAAGAATTTGATACT
GACTTAAAACCAAGACTTCATGTAAATGCACCATTTGTTTGTCAAAATAATTGTCCTGGTCAATTATT
TGTAAAAGTTGCGCCTAATTTAACAAATGAATATGATCCTGATGCATCTGCTAATATGTCAAGAATTG
TAACTTACTCAGATTTTTGGTGGAAAGGTAAATTAGTATTTAAAGCTAAACTAAGAGCCTCTCATACT
TGGAATCCAATTCAACAAATGAGTATTAATGTAGATAACCAATTTAACTATGTACCAAGTAATATTGG
AGGTATGAAAATTGTATATGAAAAATCTCAACTAGCACCTAGA
'ITA_FJ005265_140_2005'
ATGAGTGATGGAGCAGTTCAACCAGACGGTGGTCAAGCTGCTGTCAGAAATGAAAGAGCTACAGGATC
TGGGAACGGGTCTGGAGGCGGGGGTGGTGGTGGTTCTGGGGGTGTGGGGATTTCTACGGGTACTTTCA
ATAATCAGACGGAATTTAAATTTTTGGAAAACGGATGGGTGGAAATCACAGCAAACTCAAGCAGACTT
GTACATTTAAATATGCCAGAAAGTGAAAATTATAGAAGAGTGGTTGTAAATAATTTGGATAAAACTGC
AGTTAACGGAAACATGGCTTTAGATGATACTCATGCACAAATTGTAACACCTTGGTCATTGGTTGATG
CAAATGCTTGGGGAGTTTGGTTTAATCCAGGAGATTGGCAACTAATTGTTAATACTATGAGTGAGTTG
CATTTAGTTAGTTTTGAACAAGAAATTTTTAATGTTGTTTTAAAGACTGTTTCAGAATCTGCTACTCA
GCCACCAACTAAAGTTTATAATAATGATTTAACTGCATCATTGATGGTTGCATTAGATAGTAATAATA
CTATGCCATTTACTCCAGCAGCTATGAGATCTGAGACATTGGGTTTTTATCCATGGAAACCAACCATA
CCAACTCCATGGAGATATTATTTTCAATGGGATAGAACATTAATACCATCTCATACTGGAACTAGTGG
CACACCAACAAATATATACCATGGTACAGATCCAGATGATGTTCAATTTTATACTATTGAAAATTCTG
TGCCAGTACACTTACTAAGAACAGGTGATGAATTTGCTACAGGAACATTTTTTTTTGATTGTAAACCA
TGTAGACTAACACATACATGGCAAACAAATAGAGCATTGGGCTTACCACCATTTCTAAATTCTTTGCC
TCAAGCTGAAGGAGGTACTAACTTTGGTTATATAGGAGTTCAACAAGATAAAAGACGTGGTGTAACTC
AAATGGGAAATACAAACTATATTACTGAAGCTACTATTATGAGACCAGCTGAGGTTGGTTATAGTGCA
CCATATTATTCTTTTGAGGCGTCTACACAAGGGCCATTTAAAACACCTATTGCAGCAGGACGGGGGGG
AGCGCAAACAGATGAAAATCAAGCAGCAGATGGTGATCCAAGATATGCATTTGGTAGACAACATGGTC
AAAAAACTACCACAACAGGAGAAACACCTGAGAGATTTACATATATAGCACATCAAGATACAGGAAGA
TATCCAGAAGGAGATTGGATTCAAAATATTAACTTTAACCTTCCTGTAACAGATGATAATGTATTGCT
ACCAACAGATCCAATTGGAGGTAAAACAGGAATTAACTATACTAATATATTTAATACTTATGGTCCTT
TAACTGCATTAAATAATGTACCACCAGTTTATCCAAATGGTCAAATTTGGGATAAAGAATTTGATACT
GACTTAAAACCAAGACTTCATGTAAATGCACCATTTGTTTGTCAAAATAATTGCCCTGGTCAATTATT
TGTAAAAGTTGCGCCTAATTTAACAAATGAATATGATCCTGATGCATCTGCTAATATGTCAAGAATTG
TAACTTACTCAGATTTTTGGTGGAAAGGTAAATTAGTATTTAAAGCTAAACTAAGAGCCTCTCATACT

TGGAATCCAATTCAACAAATGAGTATTAATGTAGATAACCAATTTAACTATGTACCAAGTAATATTGG
AGGTATGAAAATTGTCTATGAAAAATCTCAACTAGCACCTAGA
'VAC_FJ011097_Merial_2006'
ATGAGTGATGGAGCAGTTCAACCAGACGGTGGTCAACCTGCTGTCAGAAATGAAAGAGCTACAGGATC
TGGGAACGGGTCTGGAGGCGGGGGTGGTGGTGGTTCTGGGGGTGTGGGGATTTCTACGGGTGCTTTCA
ATAATCAGACGGAATTTAAATTTTTGGAAAACGGATGGGTGGAAATCACAGCAAACTCAAGCAGACTT
GTACATTTAAATATGCCAGAAAGCGAAAATTATAGAAGAGTGGTTGTAAATAATATGGATAAAACTGC
AGTTAACGGAAACATGGCTTTAGATGATATTCATGCACAAATTGTAACACCTTGGTCATTGGTTGATG
CAAATGCTTGGGGAGTTTGGTTTAATCCAGGAGATTGGCAACTAATTGTTAATACTATGAGTGAGTTG
CATTTAGTTAGTTTTGAACAAGAAATTTTTAATGTTGTTTTAAAGACTGTTTCAGAATCTGCTACTCA
GCCACCAACTAAAGTTTATAATAATGATTTAACTGCATCATTGATGGTTGCATTAGATAGTAATAATA
CTATGCCATTTACTCCAGCAGCTATGAGATCTGAGACATTGGGTTTTTATCCATGGAAACCAACCATA
CCAACTCCATGGAGATATTATTTTCAATGGGATAGAACATTAATACCATCTCATACTGGAACTAGTGG
CACACCAACAAATATATATCATGGTACAGATCCAGATGATGTTCAATTTTATACTATTGAAAATTCTG
TGCCAGTACACTTACTAAGAACAGGTGATGAATTTGCTACAGGAACATTTTTTTTTGATTGTAGACCA
TGTAGACTAACACATACATGGCAAACAAATAGAGCATTGGGCTTACCACCATTTCTAAATTCTTTGCC
TCAATCTGAAGGAGCTACTAACTTTGGTGATATAGGAGTTCAACAAGATAAAAGACGTGGTATAACTC
AAATGGGAAATACAAACTATATTACTGAAGCTACTATTATGAGACCAGCTGAGGTTGGTTATAGTGCA
CCATATTATTCTTTTGAGGCGTCTACACAAGGGCCATTTAAAACACCTATTGCAGCAGGACGGGGGGG
AGCGCAAACAGATGAAAATCAAGCAGCAGATGGTAATCCAAGATATGCATTTGGTAGACAACATGGTC
AAAAAACTACCACAACAGGAGAAACACCTGAGAGATTTACATATATAGCACATCAAGATACAGGAAGA
TATCCAGAAGGAGATTGGATTCAAAATATTAACTTTAACCTTCCTGTAACAAATGATAATGTATTGCT
ACCAACAGATCCAATTGGAGGTAAAACAGGAATTAACTATACTAATATATTTAATACTTATGGTCCTT
TAACTGCATTAAATAATGTACCACCAGTTTATCCAAATGGTCAAATTTGGGATAAAGAATTTGATACT
GACTTAAAACCAAGACTTCATGTAAATGCACCATTTGTTTGTCAAAATAATTGTCCTGGTCAATTATT
TGTAAAAGTTGCGCCTAATTTAACGAATGAATATGATCCTGATGCATCTGCTAATATGTCAAGAATTG
TAACTTACTCAGATTTTTGGTGGAAAGGTAAGTTAGTATTTAAAGCTAAACTAAGAGCCTCTCATACT
TGGAATCCAATTCAACAAATGAGTATTAATGTAGATAACCAATTTAACTATGTACCAAGTAATATTGG
AGGTATGACAATTGTATATGAAAAATCTCAACTAGCACCTAGA
'VAC_FJ011098_Intervet_2006'
ATGAGTGATGGAGCAGTTCAACCAGACGGTGGTCAACCTGCTGTCAGAAATGAAAGAGCTACAGGATC
TGGGAACGGGTCTGGAGGCGGGGGTGGTGGTGGTTCTGGGGGTGTGGGGATTTCTACGGGTACTTTCA
ATAATCAGACGGAATTTAAATTTTTGGAAAACGGATGGGTGGAAATCACAGCAAACTCAAGCAGACTT
GTACATTTAAATATGCCAGAAAGTGAAAATTATAGAAGAGTGGTTGTGAATAATATGGATAAAACTGC
AGTTAACGGAAACATGGCTTTAGATGATATTCATGCACAAATTGTAACACCTTGGTCATTGGTTGATG
CAAATGCTTGGGGAGTTTGGTTTAATCCAGGAGATTGGCAACTAATTGTTAATACTATGAGTGAGTTG
CATTTAGTTAGTTTTGAACAAGAAATTTTTAATGTTGTTTTAAAGACTGTTTCAGAATCTGCTACTCA
GCCACCAACTAAAGTTTATAATAATGATTTAACTGCATCATTGATGGTTGCATTAGATAGTAATAATA
CTATGCCATTTACTCCAGCAGCTATGAGATCTGAGACATTGGGTTTTTATCCATGGAAACCAACCATA
CCAACTCCATGGAGATATTATTTTCAATGGGATAGAACATTAGTACCATCTCATACTGGAACTAGTGG
CACACCAACAAATATATACCATGGTACAGATCCAGATGATGTTCAATTTTATACTATTGAAAATTCTG
TGCCAGTACACTTACTGAGAACAGGTGATGAATTTGCTACAGGAACATTTTTTTTTGATTGTAAACCA
TGTAGACTAACACATACATGGCAAACAAATAGAGCATTGGGCTTACCACCATTTCTAAATTCTTTGCC
TCAATCTGAAGGAGCTACTAACTTTGGTGATATAGGAGTTCAACAAGATAAAAGACGTGGTATAACTC
AAATGGGAAATACAAACTATATTACTGAAGCTACTATTATGAGACCAGCTGAGGTTGGTTATAGTGCA
CCATATTATTCTTTTGAGGCGTCTACACAAGGGCCATTTAAAACACCTATTGCAGCAGGACGGGGGGG
AGCGCAAACAGATGAAAATCAAGCAGCAGATGGTAATCCAAGATATGCATTTGGTAGACAACATGGTC
AAAAAACTACCACAACAGGAGAAACACCTGAGAGATTTACATATATAGCACATCAAGATACAGGAAGA
TATCCAGAAGGAGATTGGATTCAAAATATTAACTTTAACCTTCCTGTAACAAATGATAATGTATTGCT
ACCAACAGATCCAATTGGAGGTAAAACAGGAATTAACTATACTAATATATTTAATACTTATGGTCCTT
TAACTGCATTAAATAATGTACCACCAGTTTATCCAAATGGTCAAATTTGGGATAAAGAATTTGATACT
GACTTAAAACCAAGACTTCATGTAAATGCACCATTTGTTTGTCAAAATAATTGTCCTGGTCAATTATT
TGTAAAAGTTGCGCCTAATTTAACGAATGAATATGATCCTGATGCATCTGCTAATATGTCAAGAATTG
TAACTTACTCAGATTTTTGGTGGAAAGGTAAGTTAGTATTTAAAGCTAAACTAAGAGCCTCTCATACT

TGGAATCCAATTCAACAAATGAGTATTAATGTAGATAACCAATTTAACTATGTACCAAGTAATATTGG
AGGTATGACAATTGTATATGAAAAATCTCAACTAGCACCTAGA

'VAC_FJ197847_Pfizer_2007'

ATGAGTGATGGAGCAGTTCAACCAGACGGTGGTCAACCTGCTGTCAGAAATGAAAGAGCTACAGGATC
TGGGAACGGGTCTGGAGGCGGGGGTGGTGGTGGTTCTGGGGGTGTGGGGATTTCTACGGGTGCTTTCA
ATAATCAGACGGAATTTAAATTTTTGGAAAACGGATGGGTGGAAATCACAGCAAACTCAAGCAGACTT
GTACATTTAAATATGCCAGAAAGTGAAAATTATAGAAGAGTGGTTGTAAATAATATGGATAAAACTGC
AGTTAACGGAAACATGGCTTTAGATGATATTCATGCACAAATTGTAACACCTTGGTCATTGGTTGATG
CAAATGCTTGGGGAGTTTGGTTTAATCCAGGAGATTGGCAACTAATTGTTAATACTATGAGTGAGTTG
CATTTAGTTAGTTTTGAACAAGAAATTTTTAATGTTGTTTTAAAGACTGTTTCAGAATCTGCTACTCA
GCCACCAACTAAAGTTTATAATAATGATTTAACTGCATCATTGATGGTTGCATTAGATAGTAATAATA
CTATGCCATTTACTCCAGCAGCTATGAGATCTGAGACATTGGGTTTTTATCCATGGAAACCAACCATA
CCAACTCCATGGAGATATTATTTTCAATGGGATAGAACATTAATACCATCTCATACTGGAACTAGTGG
CACACCAACAAATATATACCATGGTACAGATCCAGATGATGTTCAATTTTATACTATTGAAAATTCTG
TGCCAGTACACTTACTAAGAACAGGTGATGAATTTGCTACAGGAACATTTTTTTTTGATTGTAAACCA
TGTAGACTAACACATACATGGCAAACAAATAGAGCATTGGGCTTACCACCATTTCTAAATTCTTTGCC
TCAATCTGAAGGAGCTACTAACTTTGGTGATATAGGAGTTCAACAAGATAAAAGACGTGGTGTAACTC
AAATGGGAAATACAAACTATATTACTGAAGCTACTATTATGAGACCAGCTGAGGTTGGTTATAGTGCA
CCATATTATTCTTTTGAGGCGTCTACACAAGGGCCATTTAAAACACCTATTGCAGCAGGACGGGGGGG
AGCGCAAACAGATGAAAATCAAGCAGCAGATGGTGAACCAAGATATGCATTTGGTAGACAACATGGTC
AAAAAACTACCACAACAGGAGAAACACCTGAGAGATTTACATATATAGCACATCAAGATACAGGAAGA
TATCCAGAAGGAGATTGGATTCAAAATATTAACTTTAACCTTCCTGTAACGAATGATAATGTATTGCT
ACCAACAGATCCAATTGGAGGTAAAACAGGAATTAACTATACTAATATATTTAATACTTATGGTCCTT
TAACTGCATTAAATAATGTACCACCAGTTTATCCAAATGGTCAAATTTGGGATAAAGAATTTGATACT
GACTTAAAACCAAGACTTCATGTAAATGCACCATTTGTTTGTCAAAATAATTGTCCTGGTCAATTATT
TGTAAAAGTTGCGCCTAATTTAACAAATGAATATGATCCTGATGCATCTGCTAATATGTCAAGAATTG
TAACTTACTCAGATTTTTGGTGGAAAGGTAAATTAGTATTTAAAGCTAAACTAAGAGCCTCTCATACT
TGGAATCCAATTCAACAAATGAGTATTAATGTAGATAACCAATTTAACTATGTACCAAGTAATATTGG
AGGTATGAAAATTGTATTTGAAAAATCTCAACTAGCACCTAGA

'ITA_FJ222821_2c_56_2000'

ATGAGTGATGGAGCAGTTCAACCAGACGGTGGTCAACCTGCTGTCAGAAATGAAAGAGCTACAGGATC
TGGGAACGGGTCTGGAGGCGGGGGTGGTGGTGGTTCTGGGGGTGTGGGGATTTCTACGGGTACTTTCA
ATAATCAGACGGAATTTAAATTTTTGGAAAACGGATGGGTGGAAATCACAGCAAACTCAAGCAGACTT
GTACATTTAAATATGCCAGAAAGTGAAAATTATAGAAGAGTGGTTGTAAATAATTTGGATAAAACTGC
AGTTAACGGAAACATGGCTTTAGATGATACTCATGCACAAATTGTAACACCTTGGTCATTGGTTGATG
CAAATGCTTGGGGAGTTTGGTTTAATCCAGGAGATTGGCAACTAATTGTTAATACTATGAGTGAGTTG
CATTTAGTTAGTTTTGAACAAGAAATTTTTAATGTTGTTTTAAAGACTGTTTCAGAATCTGCTACTCA
GCCACCAACTAAAGTTTATAATAATGATTTAACTGCATCATTGATGGTTGCATTAGATAGTAATAATA
CTATGCCATTTACTCCAGCAGCTATGAGATCTGAGACATTGGGTTTTTATCCATGGAAACCAACCATA
CCAACTCCATGGAGATATTATTTTCAATGGGATAGAACATTAATACCATCTCATACTGGAACTAGTGG
CACACCAACAAATATATACCATGGTACAGATCCAGATGATGTTCAATTTTATACTATTGAAAATTCTG
TGCCAGTACACTTACTAAGAACAGGTGATGAATTTGCTACAGGAACATTTTTTTTTGATTGTAAACCA
TGTAGACTAACACATACATGGCAAACAAATAGAGCATTGGGCTTACCACCATTTCTAAATTCTTTGCC
TCAAGCTGAAGGAGGTACTAACTTTGGTTATATAGGAGTTCAACAAGATAAAAGACGTGGTGTAACTC
AAATGGGAAATACAAACTATATTACTGAAGCTACTATTATGAGACCAGCTGAGGTTGGTTATAGTGCA
CCATATTATTCTTTTGAGGCGTCTACACAAGGGCCATTTAAAACACCTATTGCAGCAGGACGGGGGGG
AGCGCAAACAGATGAAAATCAAGCAGCAGATGGTGATCCAAGATATGCATTTGGTAGACAACATGGTC
AAAAAACTACCACAACAGGAGAAACACCTGAGAGATTTACATATATAGCACATCAAGATACAGGAAGA
TATCCAGAAGGAGATTGGATTCAAAATATTAACTTTAACCTTCCTGTAACAGAAGATAATGTATTGCT
ACCAACAGATCCAATTGGAGGTAAAACAGGAATTAACTATACTAATATATTTAATACTTATGGTCCTT
TAACTGCATTAAATAATGTACCACCAGTTTATCCAAATGGTCAAATTTGGGATAAAGAATTTGATACT
GACTTAAAACCAAGACTTCATGTAAATGCACCATTTGTTTGTCAAAATAATTGTCCTGGTCAATTATT
TGTAAAAGTTGCGCCTAATTTAACAAATGAATATGATCCTGATGCATCTGCTAATATGTCAAGAATTG
TAACTTACTCAGATTTTTGGTGGAAAGGTAAATTAGTATTTAAAGCTAAACTAAGAGCCTCTCATACT

TGGAATCCAATTCAACAAATGAGTATTAATGTAGATAACCAATTTAACTATGTACCAAGTAATATTGG
AGGTATGAAAATTGTATATGAAAAATCTCAACTAGCACCTAGA

'VAC_FJ222822_2b_FortDodge_2008'

ATGAGTGATGGAGCAGTTCAACCAGACGGTGGTCAACCTGCTGTCAGAAATGAAAGAGCTACAGGATC
TGGGAACGGGTCTGGAGGCGGGGGTGGTGGTGGTTCTGGGGGTGTGGGGATTTCTACGGGTACTTTCA
ATAATCAGACGGAATTTAAATTTTTGGAAAACGGATGGGTGGAAATCACAGCAAACTCAAGCAGACTT
GTACATTTAAATATGCCAGAAAGTGAAAATTATAGAAGAGTGGTTGTAAATAATTTGGATAAAACTGC
AGTTAACGGAAACATGGCTTTAGATGATACTCATGCACAAATTGTAACACCTTGGTCATTGGTTGATG
CAAATGCTTGGGGAGTTTGGTTTAATCCAGGAGATTGGCAACTAATTGTTAATACTATGAGTGAGTTG
CATTTAGTTAGTTTTGAACAAGAAATTTTTAATGTTGTTTTAAAGACTGTTTCAGAATCTGCTACTCA
GCCACCAACTAAAGTTTATAATAATGATTTAACTGCATCATTGATGGTTGCATTAGATAGTAATAATA
CTATGCCATTTACTCCAGCAGCTATGAGATCTGAGACATTGGGTTTTTATCCATGGAAACCAACCATA
CCAACTCCATGGAGATATTATTTTCAATGGGATAGAACATTAATACCATCTCATACTGGAACTAGTGG
CACACCAACAAATATATACCATGGTACAGATCCAGATGATGTTCAATTTTATACTATTGAAAATTCTG
TGCCAGTACACTTACTAAGAACAGGTGATGAATTTGCTACAGGAACATTTTTTTTTGATTGTAAACCA
TGTAGACTAACACATACATGGCAAACAAATAGAGCATTGGGCTTACCACCATTTCTAAATTCTTTGCC
TCAAGCTGAAGGAGGTACTAACTTTGGTTATATAGGAGTTCAACAAGATAAAAGACGTGGTGTAACTC
AAATGGGAAAAACAAACTATATTACTGAAGCTACTATTATGAGACCAGCTGAGGTTGGTTATAGTGCA
CCATATTATTCTTTTGAGGCGTCTACACAAGGGCCATTTAAAACACCTATTGCAGCAGGACGGGGGGG
AGCGCAAACAGATGAAAATCAAGCAGCAGATGGTGATCCAAGATATGCATTTGGTAGACAACATGGTC
AAAAAACTACCACAACAGGAGAAACACCTGAGAGATTTACATATATAGCACATCAAGATACAGGAAGA
TATCCAGAAGGAGATTGGATTCAAAATATTAACTTTAACCTTCCTGTAACAGATGATAATGTATTGCT
ACCAACAGATCCAATTGGAGGTAAAACAGGAATTAACTATACTAATATATTTAATACTTATGGTCCTT
TAACTGCATTAAATAATGTACCACCAGTTTATCCAAATGGTCAAATTTGGGATAAAGAATTTGATACT
GACTTAAAACCAAGACTTCATGTAAATGCACCATTTGTTTGTCAAAATAATTGTCCTGGTCAATTATT
TGTAAAAGTTGCGCCTAATTTAACAAATGAATATGATCCTGATGCATCTGCTAATATGTCAAGAATTG
TAACTTACTCAGATTTTTGGTGGAAAGGTAAATTAGTATTTAAAGCTAAACTAAGAGCCTCTCATACT
TGGAATCCAATTCAACAAATGAGTATTAATGTAGATAACCAATTTAACTATGTACCAAGTAATATTGG
AGGTATGGAAATTGTATATGAAAAATCTCAACTAGCACCTAGA

'VAC_FJ222823_2b_29_1997'

ATGAGTGATGGAGCAGTTCAACCAGACGGTGGTCAACCTGCTGTCAGAAATGAAAGAGCTACAGGATT
TGGGAACGGGTCTGGAGGCGGGGGTGGTGGTGGTTCTGGGGGTGTGGGGATTTCTACGGGTACTTTCA
ATAATCAGACAGAATTTAAATTTTTGGAAAACGGATGGGTGGAAATCACAGCAAACTCAAGCAGACTT
GTACATTTAAATATGCCAGAAAGTGAAAATTATAGAAGAGTGGTTGTAAATAATTTGGATAAAACTGC
AGTTAACGGAAACATGGCTTTAGATGATACTCATGCACAAATTGTAACACCTTGGTCATTGGTTGATG
CAAATGCTTGGGGAGTTTGGTTTAATCCAGGAGATTGGCAACTAATTGTTAATACTATGAGTGAGTTG
CATTTAGTTAGTTTTGAACAAGAAATTTTTAATGTTGTTTTAAAGACTGTTTCAGAATCTGCTACTCA
GCCACCAACTAAAGTTTATAATAATGATTTAACTGCATCATTGATGGTTGCATTAGATAGTAATAATA
CTATGCCATTTACTCCAGCAGCTATGAGATCTGAGACATTGGGTTTTTATCCATGGAAACCAACCATA
CCAACTCCATGGAGATATTATTTTCAATGGGATAGGACATTAATACCATCTCATACTGGAACTAGTGG
CACACCAACAAATATATACCATGGTACAGATCCAGATGATGTTCAATTTTATACTATTGAAAATTCTG
TGCCAGTACACTTACTAAGAACAGGTGATGAATTTGCTACAGGAACATTTTTTTTTGATTGTAAACCA
TGTAGACTAACACATACATGGCAAACAAATAGAGCATTGGGCTTACCACCATTTCTAAATTCTTTGCC
TCAAGCTGAAGGAGGTACTAACTTTGGTTATATAGGAGTTCAACAAGATAAAAGACGTGGTGTAACTC
AAATGGGAAATACAAACTATATTACTGAAGCTACTATTATGAGACCAGCTGAGGTTGGTTATAGTGCA
CCATATTATTCTTTTGAGGCGTCTACACAAGGGCCATTTAAAACACCTATTGCAGCAGGACGGGGGGG
AGCGCAAACAGATGAAAATCAAGCAGCAGATGGTGATCCAAGATATGCATTTGGTAGACAACATGGTC
AAAAAACTACCACAACAGGAGAAACACCTGAGAGATTTACATATATAGCACATCAAGATACAGGAAGA
TATCCAGAAGGAGATTGGATTCAAAATATTAACTTTAACCTTCCTGTAACAGATGATAATGTATTGCT
ACCAACAGATCCAATTGGAGGTAAAACAGGAATTAACTATACTAATATATTTAATACTTATGGTCCTT
TAACTGCATTAAATAATGTACCACCAGTTTATCCAAATGGTCAAATTTGGGATAAAGAATTTGATACT
GACTTAAAACCAAGACTTCATGTAAATGCACCATTTGTTTGTCAAAATAATTGTCCTGGTCAATTATT
TGTAAAAGTTGCGCCTAATTTAACAAATGAATATGATCCTGATGCATCTGCTAATATGTCAAGAATTG
TAACTTACTCAGATTTTTGGTGGAAAGGTAAATTAGTGTTTAAAGCTAAACTAAGAGCCTCTCATACT

TGGAATCCAATTCAACAAATGAGTATTAATGTAGATAACCAATTTAACTATGTACCAAGTAATATTGG
AGGTATGAAAATTGTATATGAAAAATCTCAACTAGCACCTAGA
'ITA_FJ222824_388_05_3_2005'
ATGAGTGATGGAGCAGTTCAACCAGACGGTGGTCAACCTGCTGTCAGAAATGAAAGAGCTACAGGATC
TGGGAACGGGTCTGGAGGCGGGGGTGGTGGTGGTTCTGGGGGTGTGGGGATTTCTACGGGTACTTTCA
ATAATCAGACGGAATTTAAATTTTTGGAAAACGGATGGGTGGAAATCACAGCAAACTCAAGCAGACTT
GTACATTTAAATATGCCAGAAAGTGAAAATTATAGAAGAGTGGTTGTGAATAATATGGATAAAACTGC
AGTTAACGGAAACATGGCTTTAGATGATATTCATGCACAAATTGTAACACCTTGGTCATTGGTTGATG
CAAATGCTTGGGGAGTTTGGTTTAATCCAGGAGATTGGCAACTAATTGTTAATACTATGAGTGAGTTG
CATTTAGTTAGTTTTGAACAAGAAATTTTTAATGTTGTTTTAAAGACTGTTTCAGAATCTGCTACTCA
GCCACCAACTAAAGTTTATAATAATGATTTAACTGCATCATTGATGGTTGCATTAGATAGTAATAATA
CTATGCCATTTACTCCAGCAGCTATGAGATCTGAGACATTGGGTTTTTATCCATGGAAACCAACCATA
CCAACTCCATGGAGATATTATTTTCAATGGGATAGAACATTAGTACCATCTCATACTGGAACTAGTGG
CACACCAACAAATATATACCATGGTACAGATCCAGATGATGTTCAATTTTATACTATTGAAAATTCTG
TGCCAGTACACTTACTAAGAACAGGTGATGAATTTGCTACAGGAACATTTTTTTTTGATTGTAAACCA
TGTAGACTAACACATACATGGCAAACAAATAGAGCATTGGGCTTACCACCATTTCTAAATTCTTTGCC
TCAATCTGAAGGAGCTACTAACTTTGGTGATATAGGAGTTCAACAAGATAAAAGACGTGGTGTAACTC
AAATGGGAAATACAAACTATATTACTGAAGCTACTATTATGAGACCAGCTGAGGTTGGTTATAGTGCA
CCATATTATTCTTTTGAGGCGTCTACACAAGGGCCATTTAAAACACCTATTGCAGCAGGACGGGGGGG
AGCGCAAACAGATGAAAATCAAGCAGCAGATGGTAATCCAAGATATGCATTTGGTAGACAACATGGTA
AAAAAACTACCACAACAGGAGAAACACCTGAGAGATTTACATATATAGCACATCAAGATACAGGAAGA
TATCCAGAAGGAGATTGGATTCAAAATATTAACTTTAACCTTCCTGTAACAAATGATAATGTATTGCT
ACCAACAGATCCAATTGGAGGTAAAACAGGAATTAACTATACTAATATATTTAATACTTATGGTCCTT
TAACTGCATTAAATAATGTACCACCAGTTTATCCAAATGGTCAAATTTGGGATAAAGAATTTGATACT
GACTTAAAACCAAGACTTCATGTAAATGCACCATTTGTTTGTCAAAATAATTGTCCTGGTCAATTATT
TGTAAAAGTTGCGCCTAATTTAACAAATGAATATGATCCTGATGCATCTGCTAATATGTCAAGAATTG
TAACTTACTCAGATTTTTGGTGGAAAGGTAAATTAGTATTTAAAGCTAAACTAAGAGCCTCTCATACT
TGGAATCCAATTCAACAAATGAGTATTAATGTAGATAACCAATTTAACTATGTACCAAGTAATATTGG
AGGTATGAAAATTGTATATGAAAAATCTCAACTAGCACCTAGA
'CHI_FJ231389_FPV_monkey_BJ_22_2008'
ATGAGTGATGGAGCAGTTCAACCAGACGGTGGTCAACCTGCTGTCAGAAATGAAAGAGCTACAGGATC
TGGGAACGGGTCTGGAGGCGGGGGTGGTGGTGGTTCTGGGGGTGTGGGGATTTCTACGGGTACTTTCA
ATAATCAGACGGAATTTAAATTTTTGGAAAACGGATGGGTGGAAATCACAGCAAACTCAAGCAGACTT
GTACATTTAAATATGCCAGAAAGTGAAAATTATAAAAGAGTAGTTGTAAATAATATGGATAAAACTGC
AGTTAAAGGAAACATGGCTTTAGATGATATTCATGTACAAATTGTAACACCTTGGTCATTGGTTGATG
CAAATGCTTGGGGAGTTTGGTTTAATCCAGGAGATTGGCAACTAATTGTTAATACTATGAGTGAGTTG
CATTTAGTTAGTTTTGAACAAGAAATTTTTAATGTTGTTTTAAAGACTGTTTCAGAATCTGCTACTCA
GCCACCAACTAAAGTTTATAATAATGATTTAACTGCATCATTGATGGTTGCATTAGATAGTAATAATA
CTATGCCATTTACTCCAGCAGCTATGAGATCTGAGACATTGGGTTTTTATCCATGGAAACCAACCATA
CCAACTCCATGGAGATATTATTTTCAATGGGATAGAACATTAATACCATCTCATACTGGAACTAGTGG
CACACCAACAAATATATACCATGGTACAGATCCAGATGATGTTCAATTTTATACTATTGAAAATTCTG
TGCCAGTACACTTACTAAGAACAGGTGATGAATTTGCTACAGGAACATTTTTTTTTGATTGTAAACCA
TGTAGACTAACACATACATGGCAAACAAATAGAGCATTGGGCTTACCACCATTTTTAAATTCTTTGCC
TCAATCTGAAGGAGCTACTAACTTTGGTGATATAGGAGTTCAACAAGATAAAAGACGTGGTGTAACTC
AAATGGGAAATACAAACTATATTACTGAAGCTACTATTATGAGACCAGCTGAGGTTGGTTATAGTGCA
CCATATTATTCTTTTGAGGCGTCTACACAAGGGCCATTTAAAACACCTATTGCAGCAGGACGGGGGGG
AGCGCAAACAGATGAAAATCAAGCAGCAGATGGTGATCCAAGATATGCATTTGGTAGACAACATGGTC
AAAAAACTACCACAACAGGAGAAACACCTGAGAGATTTACATATATAGCACATCAAGATACAGGAAGA
TATCCAGAAGGAGATTGGATTCAAAATATTAACTTTAACCTTCCTGTAACAAATGATAATGTATTGCT
ACCAACAGATCCAATTGGAGGTAAAACAGGAATTAACTATACTAATATATTTAATACTTATGGTCCTT
TAACTGCATTAAATAATGTACCACCAGTTTATCCAAATGGTCAAATTTGGGATAAAGAATTTGATACT
GACTTAAAACCAAGACTTCATGTAAATGCACCATTTGTTTGTCAAAATAATTGTCCTGGTCAATTATT
TGTAAAAGTTGCGCCTAATTTAACAAATGAATATGATCCTGATGCATCTGCTAATATGTCAAGAATTG
TAACTTACTCAGATTTTTGGTGGAAAGGTAAATTAGTATTTAAAGCTAAACTAAGAGCCTCTCATACT

TGGAATCCAATTCAACAAATGAGTATTAATGTAGATAACCAATTTAACTATGTACCAAGTAATATTGG
AGCTATGAAAATTGTATATGAAAAATCTCAACTAGCACCTAGA

'TAW_FJ265775_CPV301_2004'

ATGAGTGATGGAGCAGTTCAACCAGACGGTGGTCAGCCTGCTGTCAGAAATGAAAGAGCTACTGGATC
TGGGAACGGGTCTGGAGGCGGGGGTGGTGGTGGTTCTGGGGGTGTGGGGATTTCTACGGGTACTTTCA
ATAATCAGACGGAATTTAAATTTTTGGAAAACGGATGGGTGGAAATCACAGCAAACTCAAGCAGACTT
GTACATTTAAATATGCCAGAAAGTGAAAATTATAGAAGAGTGGTTGTAAATAATTTGGATAAAACTGC
AGTTAACGGAAACATGGCTCTAGATGATACCCATGCACAAATTGTAACACCTTGGTCATTGGTTGATG
CAAATGCTTGGGGAGTTTGGTTTAATCCAGGAGATTGGCAACTAATTGTTAATACTATGAGTGAGTTG
CATTTAGTTAGTTTTGAACAAGAAATTTTTAATGTTGTTTTAAAGACTGTTTCAGAATCTGCTACTCA
GCCACCAACTAAAGTTTATAATAATGATTTAACTGCATCATTGATGGTTGCATTAGATAGTAATAATA
CTATGCCATTTACTCCAGCAGCTATGAGATCTGAGACATTGGGTTTTTATCCATGGAAACCAACCATA
CCAACTCCATGGAGATATTATTTTCAATGGGATAGAACATTAATACCATCTCATACTGGAACTAGTGG
CACACCAACAAATATATACCATGGTACAGATCCAGATGATGTTCAATTTTATACTATTGAAAATTCTG
TGCCAGTACACTTACTAAGAACAGGTGATGAATTTGCTACAGGAACATTTTTTTTTGATTGTAAACCA
TGTAGACTAACACATACATGGCAAACAAATAGAGCATTGGGCTTACCACCATTTCTAAATTCTTTGCC
TCAAGCTGAAGGAGGTACTAACTTTGGTTATATAGGAGTTCAACAAGATAAAAGACGTGGTGTAACTC
AAATGGGAAATACAAACTATATTACTGAAGCTACTATTATGAGACCAGCTGAGGTTGGTTATAGTGCA
CCATATTATTCTTTTGAGGCGTCTACACAAGGGCCATTTAAAACACCTATTGCAGCAGGACGGGGGGG
AGCGCAAACAGATGAAAATCAAGCAGCAGATGGTGATCCAAGATATGCATTTGGTAGACAACATGGTC
AAAAAACTACCACAACAGGAGAAACACCTGAGAGATTTACATATATAGCACATCAAGATACAGGAAGA
TATCCAGAAGGAGATTGGATTCAAAATATTAACTTTAACCTTCCTGTAACAGATGATAATGTATTGCT
ACCAACAGATCCAATTGGAGGTAAAACAGGAATTAACTATACTAATATATTTAATACTTATGGTCCTT
TAACTGCATTAAATAATGTACCACCAGTTTATCCAAATGGTCAAATTTGGGATAAAGAATTTGATACT
GACTTAAAACCAAGACTTCATGTAAATGCACCATTTGTTTGTCAAAATAATTGTCCTGGTCAATTATT
TGTAAAAGTTGCACCTAATTTAACAAATGAATATGATCCTGATGCATCTGCTAATATGTCAAGAATTG
TAACTTACTCAGATTTTTGGTGGAAAGGTAAATTAGTATTTAAAGCTAAACTAAGAGCCTCTCATACT
TGGAATCCAATTCAACAAATGAGTATTAATGTAGATAACCAATTTAACTATGTACCAAGTAATATTGG
AGGTATGAAAATTGTATATGAAAAATCTCAACTAGCACCTAGA

'TAW_FJ265781_CPV307_2005'

ATGAGTGATGGAGCAGTTCAACCAGACGGTGGTCAGCCTGCTGTCAGAAATGAAAGAGCTACAGGATC
TGGGAACGGGTCTGGAGGCGGGGGTGGTGGTGGTTCTGGGGGTGTGGGGATTTCTACGGGTACTTTCA
ATAATCAGACGGAATTTAAATTTTTGGAAAACGGATGGGTGGAAATCACAGCAAACTCAAGCAGACTT
GTACATTTAAATATGCCAGAAAGTGAAAATTATAGAAGAGTGGTTGTAAATAATTTGGATAAAACTGC
AGTTAACGGAAACATGGCTTTAGATGATACCCATGCACAAATTGTAACACCTTGGTCATTGGTTGATG
CAAATGCTTGGGGAGTTTGGTTTAATCCAGGAGATTGGCAACTAATTGTTAATACTATGAGTGAGTTG
CATTTAGTTAGTTTTGAACAAGAAATTTTTAATGTTGTTTTAAAGACTGTTTCAGAATCTGCTACTCA
GCCACCAACTAAAGTTTATAATAATGATTTAACTGCATCATTGATGGTTGCATTAGATAGTAATAATA
CTATGCCATTTACTCCAGCAGCTATGAGATCTGAGACATTGGGTTTTTATCCATGGAAACCAACCATA
CCAACTCCATGGAGATATTATTTTCAATGGGATAGAACATTAATACCATCTCATACTGGAACTAGTGG
CACACCAACAAATATATACCATGGTACAGATCCAGATGATGTTCAATTTTATACTATTGAAAATTCTG
TGCCAGTACACTTACTAAGAACAGGTGATGAATTTGCTACAGGAACATTTTTTTTTGATTGTAAACCA
TGTAGACTAACACATACATGGCAAACAAATAGAGCATTGGGCTTACCACCATTTCTAAATTCTTTGCC
TCAAGCTGAAGGAGGTACTAACTTTGGTTATATAGGAGTTCAACAAGATAAAAGACGTGGTGTAACTC
AAATGGGAAATACAAACTATATTACTGAAGCTACTATTATGAGACCAGCTGAGGTTGGTTATAGTGCA
CCATATTATTCTTTTGAGGCGTCTACACAAGGGCCATTTAAAACACCTATTGCAGCAGGACGGGGGGG
AGCGCAAACAGATGAAAATCAAGCAGCAGATGGTGATCCAAGATATGCATTTGGTAGACAACATGGTC
AAAAAACTACCACAACAGGAGAAACACCTGAGAGATTTACATATATAGCACATCAAGATACAGGAAGA
TATCCAGAAGGAGATTGGATTCAAAATATTAACTTTAACCTTCCTGTAACAGATGATAATGTATTGCT
ACCAACAGATCCAATTGGAGGTAAAACAGGAATTAACTATACTAATATATTTAATACTTATGGTCCTT
TAACTGCATTAAATAATGTACCACCAGTTTATCCAAATGGTCAAATTTGGGATAAAGAATTTGATACT
GACTTAAAACCAAGACTTCATGTAAATGCACCATTTGTTTGTCAAAATAATTGTCCTGGTCAATTATT
TGTAAAAGTTGCACCTAATTTAACAAATGAATATGATCCTGATGCATCTGCTAATATGTCAAGAATTG
TAACTTACTCAGATTTTTGGTGGAAAGGTAAATTAGTATTTAAAGCTAAACTAAGAGCCTCTCATACT

TGGAATCCAATTCAACAAATGAGTATTAATGTAGATAACCAATTTAACTATGTACCAAGTAATATTGG
AGGTATGAAAATTGTATATGAAAAATCTCAACTAGCACCTAGA

'CHI_FJ432718_CPV_Cv_2008'

ATGAGTGATGGAGCAGTTCAACCAGACGGTGGTCAACCTGCTGTCAGAAATGAAAGAGCTACAGGATC
TGGGAACGGGTCTGGAGGCGGGGGTGGTGGTGGTTCTGGGGGTGTGGGGATTTCTACGGGTACTTTCA
ATAATCAGACGGAATTTAAATTTTTGGAAAACGGATGGGTGGAAATCACAGCAAACTCAAGCAGACTT
GTACATTTAAATATGCCAGAAAGTGAAAATTATAGAAGAGTGGTTGTGAATAATATGGATAAAACTGC
AGTTAACGGAAACATGGCTTTAGATGATATTCATGCACAAATTGTAACACCTTGGTCATTGGTTGATG
CAAATGCTTGGGGAGTTTGGTTTAATCCAGGAGATTGGCAACTAATTGTTAATACTATGAGTGAGTTG
CATTTAGTTAGTTTTGAACAAGAAATTTTTAATGTTGTTTTAAAGACTGTTTCAGAATCTGCTACTCA
GCCACCAACTAAAGTTTATAATAATGATTTAACTGCATCATTGATGGTTGCATTAGATAGTAATAATA
CTATGCCATTTACTCCAGCAGCTATGAGATCTGAGACATTGGGTTTTTATCCATGGAAACCAACCATA
CCAACTCCATGGAGATATTATTTTCAATGGGATAGAACATTAGTACCATCTCATACTGGAACTAGTGG
CACACCAACAAATATATACCATGGTACAGATCCAGATGATGTTCAATTTCATACTATTGAAAATTCTG
TGCCAGTACACTTACTAAGAACAGGTGATGAATTTGCTACAGGAACATTTTTTTTTGATTGTAAACCA
TGTAGACTAACACATACATGGCAAACAAATAGAGCATTGGGCTTACCACCATTTCTAAATTCTTTGCC
TCAATCTGAAGGAGCTACTAACTTTGGTGATATAGGAGTTCAACAAGATAAAAGACGTGGTGTAACTC
AAATGGGAAATACAAACTATATTACTGAAGCTACTATTATGAGACCAGCTGAGGTTGGTTATAGTGCA
CCATATTATTCTTTTGAGGCGTCTACACAAGGGCCATTTAAAACACCTATTGCAGCAGGACGGGGGGG
AGCGCAAACAGATGAAAATCAAGCAGCAGATGGTAATCCAAGATATGCATTTGGTAGACAACATGGTA
AAAAAACTACCACAACAGGAGAAACACCTGAGAGATTTACATATATAGCACATCAAGATACAGGAAGA
TATCCAGAAGGAGATTGGATTCAAAATATTAACTTTAACCTTCCTGTAACAAATGATAATGTATTGCT
ACCAACAGATCCAATTGGAGGTAAAACAGGAATTAACTATACTAATATATTTAATACTTATGGTCCTT
TAACTGCATTAAATAATGTACCACCAGTTTATCCAAATGGTCAAATTTGGGATAAAGAATTTGATACT
GACTTAAAACCAAGACTTCATGTAAATGCACCATTTGTTTGTCAAAATAATTGTCCTGGTCAATTATT
TGTAAAAGTTGCGCCTAATTTAACAAATGAATATGATCCTGATGCATCTGCTAATATGTCAAGAATTG
TAACTTACTCAGATTTTTGGTGGAAAGGTAAATTAGTATTTAAAGCTAAACTAAGAGCCTCTCATACT
TGGAATCCAATTCAACAAATGAGTATTAATGTAGATAACCAATTTAACTATGTACCAAGTAATATTGG
AGGTATGAAAATTGTATATGAAAAATCTCAACTAGCACCTAGA

'THA_FJ869122_KU1_2008'

ATGAGTGATGGAGCAGTTCAACCAGACGGTGGTCAACCTGCTGTCAGAAATGAAAGAGCTACAGGATC
TGGGAACGGGTCTGGAGGCGGGGGTGGTGGTGGTTCTGGGGGTGTGGGGATTTCTACGGGTACTTTCA
ATAATCAGACAGAATTTAAATTTTTGGAAAACGGATGGGTGGAAATCACAGCAAACTCAAGCAGACTT
GTACATTTAAATATGCCAGAAAGTGAAAATTATAGAAGAGTGGTTGTAAATAATTTGGATAAAACTGC
AGTTAACGGAAACATGGCTTTAGATGATACTCATGCACAAATTGTAACACCTTGGTCATTGGTTGATG
CAAATGCTTGGGGAGTTTGGTTTAATCCAGGAGATTGGCAACTAATTGTTAATACTATGAGTGAGTTG
CATTTAGTTAGTTTTGAACAAGAAATTTTTAATGTTGTTTTAAAGACTGTTTCAGAATCTGCTACTCA
GCCACCAACTAAAGTTTATAATAATGATTTAACTGCATCATTGATGGTTGCATTAGATAGTAATAATA
CTATGCCATTTACTCCAGCAGCTATGAGATCTGAGACATTGGGTTTTTATCCATGGAAACCAACCATA
CCAACTCCATGGAGATATTATTTTCAATGGGATAGAACATTAATACCATCTCATACTGGAACTAGTGG
CACACCAACAAATATATACCATGGTACAGATCCAGATGATGTTCAATTTTATACTATTGAAAATTCTG
TGCCAGTACACTTACTAAGAACAGGTGATGAATTTGCTACAGGAACATTTTTTTTTGATTGTAAACCA
TGTAGACTAACACATACATGGCAAACAAATAGAGCATTGGGCTTACCACCATTTCTAAATTCTTTGCC
TCAAGCTGAAGGAGGTACTAACTTTGGTTATATAGGAGTTCAACAAGATAAAAGACGTGGTGTAACTC
AAATGGGAAATACAAACTATATTACTGAAGCTACTATTATGAGACCAGCTGAGGTTGGTTATAGTGCA
CCATATTATTCTTTTGAGGCGTCTACACAAGGGCCATTTAAAACACCTATTGCAGCAGGACGGGGGGG
AGCGCAAACAGATGAAAATCAAGCAGCAGATGGTGATCCCAGATATGCATTTGGTAGACAACATGGTC
AAAAAACTACCACAACAGGAGAAACACCTGAGAGATTTACATATATAGCACATCAAGATACAGGAAGA
TATCCAGAAGGAGATTGGATTCAAAATATTAACTTTAACCTTCCTGTAACAGATGATAATGTATTGCT
ACCAACAGATCCAATTGGAGGTAAAACAGGAATTAACTATACCAATATATTTAATACTTATGGTCCTT
TAACTGCATTAAATAATGTACCACCAGTTTATCCAAATGGTCAAATTTGGGATAAAGAATTTGATACT
GACTTAAAACCAAGACTTCATGTAAATGCACCATTTGTTTGTCAAAATAATTGTCCTGGTCAATTATT
TGTAAAAGTTGCGCCTAATTTAACAAATGAATATGATCCTGATGCATCTGCTAATATGTCAAGAATTG
TAACTTACTCAGATTTTTGGTGGAAAGGTAAATTAGTATTTAAAGCTAAACTAAGAGCCTCTCATACT

TGGAATCCAATTCAACAAATGAGTATTAATGTAGATAACCAATTTAACTATGTACCAAGTAATATTGG
AGGTATGAAAATTGTATATGAAAAATCTCAACTAGCACCTAGA
'THA_FJ869123_KU3_2008'
ATGAGTGATGGAGCAGTTCAACCAGACGGTGGTCAACCTGCTGTCAGAAATGAAAGAGCTACAGGATC
TGGGAACGGGTCTGGAGGCGGGGGTGGTGGTGGTTCTGGGGGTGTGGGGATTTCTACGGGTACTTTCA
ATAATCAGACAGAATTTAAATTTTTGGAAAACGGATGGGTGGAAATCACAGCAAACTCAAGCAGACTT
GTACATTTAAATATGCCAGAAAGTGAAAATTATAGAAGAGTGGTTGTAAATAATTTGGATAAAACTGC
AGTTAACGGAAACATGGCTTTAGATGATACTCATGCACAAATTGTAACACCTTGGTCATTGGTTGATG
CAAATGCTTGGGGAGTTTGGTTTAATCCAGGAGATTGGCAACTAATTGTTAATACTATGAGTGAGTTG
CATTTAGTTAGTTTTGAACAAGAAATTTTTAATGTTGTTTTAAAGACTGTTTCAGAATCTGCTACTCA
GCCACCAACTAAAGTTTATAATAATGATTTAACTGCATCATTGATGGTTGCATTAGATAGTAATAATA
CTATGCCATTTACTCCAGCAGCTATGAGATCTGAGACATTGGGTTTTTATCCATGGAAACCAACCATA
CCAACTCCATGGAGATATTATTTTCAATGGGATAGAACATTAATACCATCTCATACTGGAACTAGTGG
CACACCAACAAATATATACCATGGTACAGATCCAGATGATGTTCAATTTTATACTATTGAAAATTCTG
TGCCAGTACACTTACTAAGAACAGGTGATGAATTTGCTACAGGAACATTTTTTTTTGATTGTAAACCA
TGTAGACTAACACATACATGGCAAACAAATAGAGCATTGGGCTTACCACCATTTCTAAATTCTTTGCC
TCAAGCTGAAGGAGGTACTAACTTTGGTTATATAGGAGTTCAACAAGATAAAAGACGTGGTGTAACTC
AAATGGGAAATACAAACTATATTACTGAAGCTACTATTATGAGACCAGCTGAGGTTGGTTATAGTGCA
CCATATTATTCTTTTGAGGCGTCTACACAAGGGCCATTTAAAACACCTATTGCAGCAGGACGGGGGGG
AGCGCAAACAGATGAAAATCAAGCAGCAGATGGTGATCCAAGATATGCATTTGGTAGACAACATGGTC
AAAAAACTACCACAACAGGAGAAACACCTGAGAGATTTACATATATAGCACATCAAGATACAGGAAGA
TATCCAGAAGGAGATTGGATTCAAAATATTAACTTTAACCTTCCTGTAACAGATGATAATGTATTGCT
ACCAACAGATCCAATTGGAGGTAAAACAGGAATTAACTATACCAATATATTTAATACTTATGGTCCTT
TAACTGCATTAAATAATGTACCACCAGTTTATCCAAATGGTCAAATTTGGGATAAAGAATTTGATACT
GACTTAAAACCAAGACTTCATGTAAATGCACCATTTGTTTGTCAAAATAATTGTCCTGGTCAATTATT
TGTAAAAGTTGCGCCTAATTTAACAAATGAATATGATCCTGATGCATCTGCTAATATGTCAAGAATTG
TAACTTACTCAGATTTTTGGTGGAAAGGTAAATTAGTATTTAAAGCTAAACTAAGAGCCTCTCATACT
TGGAATCCAATTCAACAAATGAGTATCAATGTAGATAACCAATTTAACTATGTACCAAGTAATATTGG
AGGTATGAAAATTGTATATGAAAAATCTCAACTAGCACCTAGA
'THA_FJ869125_KU5_2004'
ATGAGTGATGGAGCAGTTCAACCAGACGGTGGTCAACCTGCTGTCAGAAATGAAAGAGCTACAGGATC
TGGGAACGGGTCTGGAGGCGGGGGTGGTGGTGGTTCTGGGGGTGTGGGGATTTCTACGGGTACTTTCA
ATAATCAGACAGAATTTAAATTTTTGGAAAACGGATGGGTGGAAATCACAGCAAACTCAAGCAGACTT
GTACATTTAAATATGCCAGAAAGTGAAAATTATAGAAGAGTGGTTGTAAATAATTTGGATAAAACTGC
AGTTAACGGAAACATGGCTTTAGATGATACCCATGCACAAATTGTAACACCTTGGTCATTGGTTGATG
CAAATGCTTGGGGAGTTTGGTTTAATCCAGGAGATTGGCAACTAATTGTTAATACTATGAGTGAGTTG
CATTTAGTTAGTTTTGAACAAGAAATTTTTAATGTTGTTTTAAAGACTGTTTCAGAATCTGCTACTCA
GCCACCAACTAAAGTTTATAATAATGATTTAACTGCATCATTGATGGTTGCATTAGATAGTAATAATA
CTATGCCATTTACTCCAGCAGCTATGAGATCTGAGACATTGGGTTTTTATCCATGGAAACCAACCATA
CCAACTCCATGGAGATATTATTTTCAATGGGATAGAACATTAATACCATCTCATACTGGAACTAGTGG
CACACCAACAAATATATACCATGGTACAGATCCAGATGATGTTCAATTTTATACTATTGAAAATTCTG
TGCCAGTACACTTACTAAGAACAGGTGATGAATTTGCTACAGGAACATTTTTTTTTGATTGTAAACCA
TGTAGACTAACACATACATGGCAAACAAATAGAGCATTGGGCTTACCACCATTTCTAAATTCTTTGCC
TCAAGCTGAAGGAGGTACTAACTTTGGTTATATAGGAGTTCAACAAGATAAAAGACGTGGTGTAACTC
AAATGGGAAATACAAACTATATTACTGAAGCTACTATTATGAGACCAGCTGAGGTTGGTTATAGTGCA
CCATATTATTCTTTTGAGGCGTCTACACAAGGGCCATTTAAAACACCTATTGCAGCAGGACGGGGGGG
AGCGCAAACAGATGAAAATCAAGCAGCAGATGGTGATCCAAGATATGCATTTGGTAGACAACATGGTC
AAAAAACTACCACAACAGGAGAAACACCTGAGAGATTTACATATATAGCACATCAAGATACAGGAAGA
TATCCAGAAGGAGATTGGATTCAAAATATTAACTTTAACCTTCCTGTAACAGATGATAATGTATTGCT
ACCAACAGATCCAATTGGAGGTAAAACAGGAATTAACTATACTAATATATTTAATACTTATGGTCCTT
TAACTGCATTAAATAATGTACCACCAGTTTATCCAAATGGTCAAATTTGGGATAAAGAATTTGATACT
GACTTAAAACCAAGACTTCATGTAAATGCACCATTTGTTTGTCAAAATAATTGTCCTGGTCAATTATT
TGTAAAAGTTGCGCCTAATTTAACAAATGAATATGATCCTGATGCATCTGCTAATATGTCAAGAATTG
TAACTTACTCAGATTTTTGGTGGAAAGGTAAATTAGTATTTAAAGCTAAACTAAGAGCCTCTCATACT

TGGAATCCAATTCAACAAATGAGTATTAATGTAGATAACCAATTTAACTATGTACCAAGTAATATTGG
AGGTATGAAAATTGTATATGAAAAATCTCAACTAGCACCTAGA
'THA_FJ869126_KU5_2008'
ATGAGTGATGGAGCAGTTCAACCAGACGGTGGTCAGCCTGCTGTCAGAAATGAAAGAGCTGCAGGATC
TGGGAACGGGTCTGGAGGCGGGGGTGGTGGTGGTTCTGGGGGTGTGGGGATTTCTACGGGTACTTTCA
ATAATCAGACGGAATTTAAATTTTTGGAAAACGGATGGGTGGAAATCACAGCAAACTCAAGCAGACTT
GTACATTTAAATATGCCAGAAAGTGAAAATTATAGAAGAGTGGTTGTAAATAATTTGGATAAAACTGC
AGTTAACGGAAACATGGCTTTAGATGATACCCATGCACAAATTGTAACACCTTGGTCATTGGTTGATG
CAAATGCTTGGGGAGTTTGGTTTAATCCAGGAGATTGGCAACTAATTGTTAATACTATGAGTGAGTTG
CATTTAGTTAGTTTTGAACAAGAAATTTTTAATGTTGTTTTAAAGACTGTTTCAGAATCTGCTACTCA
GCCACCAACTAAAGTTTATAATAATGATTTAACTGCATCATTGATGGTTGCATTAGATAGTAATAATA
CTATGCCATTTACTCCAGCAGCTATGAGATCTGAGACATTGGGTTTTTATCCATGGAAACCAACCATA
CCAACTCCATGGAGATATTATTTTCAATGGGATAGAACATTAATACCATCTCATACTGGAACTAGTGG
CACACCAACAAATATATACCATGGTACAGATCCAGATGATGTTCAATTTTATACTATTGAAAATTCTG
TGCCAGTACACTTACTAAGAACAGGTGATGAATTTGCTACAGGAACATTTTTTTTTGATTGTAAACCA
TGTAGACTAACACATACATGGCAAACAAATAGAGCATTGGGCTTACCACCATTTCTAAATTCTTTGCC
TCAAGCTGAAGGAGGTACTAACTTTGGTTATATAGGAGTTCAACAAGATAAAAGACGTGGTGTAACTC
AAATGGGAAATACAAACTATATTACTGAAGCTACTATTATGAGACCAGCTGAGGTTGGTTATAGTGCA
CCATATTATTCTTTTGAGGCGTCTACACAAGGGCCATTTAAAACACCTATTGCAGCAGGACGGGGGGG
AGCGCAAACAGATGAAAATCAAGCAGCAGATGGTGATCCAAGATATGCATTTGGTAGACAACATGGTC
AAAAAACTACCACAACAGGAGAAACACCTGAGAGATTTACATATATAGCACATCAAGATACAGGAAGA
TATCCAGAAGGAGATTGGATTCAAAATATTAACTTTAACCTTCCTGTAACAAATGATAATGTATTGCT
ACCAACAGATCCAATTGGAGGTAAAACAGGAATTAACTATACTAATATATTTAATACTTATGGTCCTT
TAACTGCATTAAATAATGTACCACCAGTTTATCCAAATGGTCAAATTTGGGATAAAGAATTTGATACT
GACTTAAAACCAAGACTTCATGTAAATGCACCATTTGTTTGTCAAAATAATTGTCCTGGTCAATTATT
TGTAAAAGTTGCGCCTAATTTAACAAATGAATATGATCCTGATGCATCTGCTAATATGTCAAGAATTG
TAACTTACTCAGATTTTTGGTGGAAAGGTAAATTAGTATTTAAAGCTAAACTAAGAGCCTCTCATACT
TGGAATCCAATTCAACAAATGAGTATTAATGTAGATAACCAATTTAACTATGTACCAAGTAATATTGG
AGGTATGAAAATTGTATATGAAAAATCTCAACTAGCACCTAGA
'THA_FJ869128_KU11_2004'
ATGAGTGATGGAGCAGTTCAACCAGACGGTGGTCAACCTGCTGTCAGAAATGAAAGAGCTACAGGATC
TGGGAACGGGTCTGGAGGCGGGGGTGGTGGTGGTTCTGGGGGTGTGGGGATTTCTACGGGTACTTTCA
ATAATCAGACAGAATTTAAATTTTTGGAAAACGGATGGGTGGAAATCACAGCAAACTCAAGCAGACTT
GTACATTTAAATATGCCAGAAAGTGAAAATTATAGAAGAGTGGTTGTAAATAATTTGGATAAAACTGC
AGTTAACGGAAACATGGCTTTAGATGATACTCATGCACAAATTGTAACACCTTGGTCATTGGTTGATG
CAAATGCTTGGGGAGTTTGGTTTAATCCAGGAGATTGGCAACTAATTGTTAATACTATGAGTGAGTTG
CATTTAGTTAGTTTTGAACAAGAAATTTTTAATGTTGTTTTAAAGACTGTTTCAGAATCTGCTACTCA
GCCACCAACTAAAGTTTATAATAATGATTTAACTGCATCATTGATGGTTGCATTAGATAGTAATAATA
CTATGCCATTTACTCCAGCAGCTATGAGATCTGAGACATTGGGTTTTTATCCATGGAAACCAACCATA
CCAACTCCATGGAGATATTATTTTCAATGGGATAGAACATTAATACCATCTCATACTGGAACTAGTGG
CACACCAACAAATATATACCATGGTACAGATCCAGATGATGTTCAATTTTATACTATTGAAAATTCTG
TGCCAGTACACTTACTAAGAACAGGTGATGAATTTGCTACAGGAACATTTTTTTTTGATTGTAAACCA
TGTAGACTAACACATACATGGCAAACAAATAGAGCATTGGGCTTACCACCATTTCTAAATTCTTTGCC
TCAAGCTGAAGGAGGTACTAACTTTGGTTATATAGGAGTTCAACAAGATAAAAGACGTGGTGTAACTC
AAATGGGAAATACAAACTATATTACTGAAGCTACTATTATGAGACCAGCTGAGGTTGGTTATAGTGCA
CCATATTATTCTTTTGAGGCGTCTACACAAGGGCCATTTAAAACACCTATTGCAGCAGGACGGGGGGG
AGCGCAAACAGATGAAAATCAAGCAGCAGATGGTGACCCAAGATATGCATTTGGTAGACAACATGGTC
AAAAAACTACCACAACAGGAGAAACACCTGAGAGATTTACATATATAGCACATCAAGATACAGGAAGA
TATCCAGAAGGAGATTGGATTCAAAATATTAACTTTAACCTTCCTGTAACAAATGATAATGTATTGCT
ACCAACAGATCCAATTGGAGGTAAAACAGGAATTAACTATACTAATATATTTAATACTTATGGTCCTT
TAACTGCATTAAATAATGTACCACCAGTTTATCCAAATGGTCAAATTTGGGATAAAGAATTTGATACT
GACTTAAAACCAAGACTTCATGTAAATGCACCATTTGTTTGTCAAAATAATTGTCCTGGTCAATTATT
TGTAAAAGTTGCGCCTAATTTAACAAATGAATATGATCCTGATGCATCTGCTAATATGTCAAGAATTG
TAACTTACTCAGATTTTTGGTGGAAAGGTAAATTAGTATTTAAAGCTAAACTAAGAGCCTCTCATACT

TGGAATCCAATTCAACAAATGAGTATTAATGTAGATAACCAATTTAACTATGTACCAAGTAATATTGG
AGGTATGAAAATTGTATATGAAAAATCTCAACTAGCACCTAGA
'THA_FJ869130_KU13_2004'
ATGAGTGATGGAGCAGTTCAACCAGACGGTGGTCAACCTGCTGTCAGAAATGAAAGAGCTACAGGATC
TGGGAACGGGTCTGGAGGCGGGGGTGGTGGTGGTTCTGGGGGTGTGGGGATTTCTACGGGTACTTTCA
ATAATCAGACGGAATTTAAATTTTTGGAAAACGGATGGGTGGAAATCACAGCAAACTCAAGCAGACTT
GTACATTTAAATATGCCAGAAAGTGAAAATTATAGAAGAGTGGTTGTAAATAATTTGGATAAAACTGC
AGTTAACGGAAACATGGCTTTAGATGATACCCATGCACAAATTGTAACACCTTGGTCATTGGTTGATG
CAAATGCTTGGGGAGTTTGGTTTAATCCAGGAGATTGGCAACTAATTGTTAATACTATGAGTGAGTTG
CATTTAGTTAGTTTTGAACAAGAAATTTTTAATGTTGTTTTAAAGACTGTTTCAGAATCTGCTACTCA
GCCACCAACTAAAGTTTATAATAATGATTTAACTGCATCATTGATGGTTGCATTAGATAGTAATAATA
CTATGCCATTTACTCCAGCAGCTATGAGATCTGAGACATTGGGTTTTTATCCATGGAAACCAACCATA
CCAACTCCATGGAGATATTATTTTCAATGGGATAGAACATTAATACCATCTCATACTGGAACTAGTGG
CACACCAACAAATATATACCATGGTACAGATCCAGATGATGTTCAATTTTATACTATTGAAAATTCTG
TGCCAGTACACTTACTAAGAACAGGTGATGAATTTGCTACAGGAACATTTTTTTTTGATTGTAAACCA
TGTAGACTAACACATACATGGCAAACAAATAGAGCATTGGGCTTACCACCATTTCTAAATTCTTTGCC
TCAAGCTGAAGGAGGTACTAACTTTGGTTATATAGGAGTTCAACAAGATAAAAGACGTGGTGTAACTC
AAATGGGAAATACAAACTATATTACTGAAGCTACTATTATGAGACCAGCTGAGGTTGGTTATAGTGCA
CCATATTATTCTTTTGAGGCGTCTACACAAGGGCCATTTAAAACACCTATTGCAGCAGGACGGGGGGG
AGCGCAAACAGATGAAAATCAAGCAGCAGATGGTGATCCAAGATATGCATTTGGTAGACAACATGGTC
AAAAAACTACCACAACAGGAGAAACACCTGAGAGATTTACATATATAGCACATCAAGATACAGGAAGA
TATCCAGAAGGAGATTGGATTCAAAATATTAACTTTAACCTTCCTGTAACAAATGATAATGTATTGCT
ACCAACAGATCCAATTGGAGGTAAAACAGGAATTAACTATACTAATATATTTAATACTTATGGTCCTT
TAACTGCATTAAATAATGTACCACCAGTTTATCCAAATGGTCAAATTTGGGATAAAGAATTTGATACT
GACTTAAAACCAAGACTTCATGTAAATGCACCATTTGTTTGTCAAAATAATTGTCCTGGTCAATTATT
TGTAAAAGTTGCGCCTAATTTAACAAATGAATATGATCCTGATGCATCTGCTAATATGTCAAGAATTG
TAACTTACTCAGATTTTTGGTGGAAAGGTAAATTAGTATTTAAAGCTAAACTAAGAGCCTCTCATACT
TGGAATCCAATTCAACAAATGAGTATTAATGTAGATAACCAATTTAACTATGTACCAAGTAATATTGG
AGGTATGAAAATTGTCTATGAAAAATCTCAACTAGCACCTAGA
'THA_FJ869134_KU23_2003'
ATGAGTGATGGAGCAGTTCAACCAGACGGTGGTCAGCCTGCTGTCAGAAATGAAAGAGCTACAGGATC
TGGGAACGGGTCTGGAGGCGGGGGTGGTGGTGGTTCTGGGGGTGTGGGGATTTCTACGGGTACTTTCA
ATAATCAGACGGAATTTAAATTTTTGGAAAACGGATGGGTGGAAATCACAGCAAACTCAAGCAGACTT
GTACATTTAAATATGCCAGAAAGTGAAAATTATAGAAGAGTGGTTGTAAATAATTTGGATAAAACTGC
AGTTAACGGAAACATGGCTTTAGATGATACCCATGCACAAATTGTAACACCTTGGTCATTGGTTGATG
CAAATGCTTGGGGAGTTTGGTTTAATCCAGGAGATTGGCAACTAATTGTTAATACTATGAGTGAGTTG
CATTTAGTTAGTTTTGAACAAGAAATTTTTAATGTTGTTTTAAAGACTGTTTCAGAATCTGCTACTCA
GCCACCAACTAAAGTTTATAATAATGATTTAACTGCATCATTGATGGTTGCATTAGATAGTAATAATA
CTATGCCATTTACTCCAGCAGCTATGAGATCTGAGACATTGGGTTTTTATCCATGGAAACCAACCATA
CCAACTCCATGGAGATATTATTTTCAATGGGATAGAACATTAATACCATCTCATACTGGAACTAGTGG
CACACCAACAAATATATACCATGGTACAGATCCAGATGATGTTCAATTTTATACTATTGAAAATTCTG
TGCCAGTACACTTACTAAGAACAGGTGATGAATTTGCTACAGGAACATTTTTTTTTGATTGTAAACCA
TGTAGACTAACACATACATGGCAAACAAATAGAGCATTGGGCTTACCACCATTTCTAAATTCTTTGCC
TCAAGCTGAAGGAGGTACTAACTTTGGTTATATAGGAGTTCAACAAGATAAAAGACGTGGTGTAACTC
AAATGGGAAATACAAACTATATTACTGAAGCTACTATTATGAGACCAGCTGAGGTTGGTTATAGTGCA
CCATATTATTCTTTTGAGGCGTCTACACAAGGGCCATTTAAAACACCTATTGCAGCAGGACGGGGGGG
AGCGCAAACAGATGAAAATCAAGCAGCAGATGGTGATCCAAGATATGCATTTGGTAGACAACATGGTC
AAAAAACTACCACAACAGGAGAAACACCTGAGAGATTTACATATATAGCACATCAAGATACAGGAAGA
TATCCAGAAGGAGATTGGATTCAAAATATTAACTTTAACCTTCCTGTAACAAATGATAATGTATTGCT
ACCAACAGATCCAATTGGAGGTAAAACAGGAATTAACTATACTAATATATTTAATACTTATGGTCCTT
TAACTGCATTAAATAATGTACCACCAGTTTATCCAAATGGTCAAATTTGGGATAAAGAATTTGATACT
GACTTAAAACCAAGACTTCATGTAAATGCACCATTTGTTTGTCAAAATAATTGTCCTGGTCAATTATT
TGTAAAAGTTGCGCCTAATTTAACAAATGAATATGATCCTGATGCATCTGCTAATATGTCAAGAATTG
TAACTTACTCAGATTTTTGGTGGAAAGGTAAATTAGTATTTAAAGCTAAACTAAGAGCCTCTCATACT

TGGAATCCAATTCAACAAATGAGTATTAATGTAGATAACCAATTTAACTATGTACCAAGTAATATTGG
AGGTATGAAAATTGTATATGAAAAATCTCAACTAGCACCTAGA

'THA_FJ869137_KU52_2003'

ATGAGTGATGGAGCAGTTCAACCAGACGGTGGTCAGCCTGCTGTCAGAAATGAAAGAGCTACAGGATC
TGGGAACGGGTCTGGAGGCGGGGGTGGTGGTGGTTCTGGGGGTGTGGGGATTTCTACGGGTACTTTCA
ATAATCAGACGGAATTTAAATTTTTGGAAAACGGATGGGTGGAAATCACAGCAAACTCAAGCAGACTT
GTACATTTAAATATGCCAGAAAGTGAAAATTATAGAAGAGTGGTTGTAAATAATTTGGATAAAACTGC
AGTTAACGGAAACATGGCTTTAGATGATACCCATGCACAAATTGTAACACCTTGGTCATTGGTTGATG
CAAATGCTTGGGGAGTTTGGTTTAATCCAGGAGATTGGCAACTAATTGTTAATACTATGAGTGAGTTG
CATTTAGTTAGTTTTGAACAAGAAATTTTTAATGTTGTTTTAAAGACTGTTTCAGAATCTGCTACTCA
GCCACCAACTAAAGTTTATAATAATGATTTAACTGCATCATTGATGGTTGCATTAGATAGTAATAATA
CTATGCCATTTACTCCAGCAGCTATGAGATCTGAGACATTGGGTTTTTATCCATGGAAACCAACCATA
CCAACTCCATGGAGATATTATTTTCAATGGGATAGAACATTAATACCATCTCATACTGGAACTAGTGG
CACACCAACAAATATATACCATGGTACAGATCCAGATGATGTTCAATTTTATACTATTGAAAATTCTG
TGCCAGTACACTTACTAAGAACAGGTGATGAATTTGCTACAGGAACATTTTTTTTTGATTGTAAACCA
TGTAGACTAACACATACATGGCAAACAAATAGAGCATTGGGCTTACCACCATTTCTAAATTCTTTGCC
TCAAGCTGAAGGAGGTACTAACTTTGGTTATATAGGAGTTCAACAAGATAAAAGACGTGGTGTAACTC
AAATGGGAAATACAAACTATATTACTGAAGCTACTATTATGAGACCAGCTGAGGTTGGTTATAGTGCA
CCATATTATTCTTTTGAGGCGTCTACACAAGGGCCATTTAAAACACCTATTGCAGCAGGACGGGGGGG
AGCGCAAACAGATGAAAATCAAGCAGCAGATGGTGATCCAAGATATGCATTTGGTAGACAACATGGTC
AAAAAACTACCACAACAGGAGAAACACCTGAGAGATTTACATATATAGCACATCAAGATACAGGAAGA
TATCCAGAAGGAGATTGGATTCAAAATATTAACTTTAACCTTCCTGTAACAAATGATAATGTATTGCT
ACCAACAGATCCAATTGGAGGTAAAACAGGAATTAACTATACTAATATATTTAATACTTATGGTCCTT
TAACTGCATTAAATAATGTACCACCAGTTTATCCAAATGGTCAAATTTGGGATAAAGAATTTGATACT
GACTTAAAACCAAGACTTCATGTAAATGCACCATTTGTTTGTCAAAATAATTGTCCTGGTCAATTATT
TGTAAAAGTTGCGCCTAATTTAACAAATGAATATGATCCTGATGCATCTGCTAATATGTCAAGAATTG
TAACTTACTCAGATTTTTGGTGGAAAGGTAAATTAGTATTTAAAGCTAAACTAAGAGCCTCTCATACT
TGGAATCCAATTCAACAAATGAGTATTAATGTAGATAACCAATTTAACTATGTACCAAGTAATATTGG
AGGTATGAAAATTGTATATGAAAAATCTCAACTAGCACCTAGA

'THA_FJ869138_KU53_2003'

ATGAGTGATGGAGCAGTTCAACCAGACGGTGGTCAACCTGCTGTCAGAAATGAAAGAGCTACAGGATC
TGGGAACGGGTCTGGAGGCGGGGGTGGTGGTGGTTCTGGGGGTGTGGGGATTTCTACGGGTACTTTCA
ATAATCAGACGGAATTTAAATTTTTGGAAAACGGATGGGTGGAAATCACAGCAAACTCAAGCAGACTT
GTACATTTAAATATGCCAGAAAGTGAAAATTATAGAAGAGTGGTTGTAAATAATTTGGATAAAACTGC
AGTTAACGGAAACATGGCTTTAGATGATACCCATGCACAAATTGTAACACCTTGGTCATTGGTTGATG
CAAATGCTTGGGGAGTTTGGTTTAATCCAGGAGATTGGCAACTAATTGTTAATACTATGAGTGAGTTG
CATTTAGTTAGTTTTGAACAAGAAATTTTTAATGTTGTTTTAAAGACTGTTTCAGAATCTGCTACTCA
GCCACCAACTAAAGTTTATAATAATGATTTAACTGCATCATTGATGGTTGCATTAGATAGTAATAATA
CTATGCCATTTACTCCAGCAGCTATGAGATCTGAGACATTGGGTTTTTATCCATGGAAACCAACCATA
CCAACTCCATGGAGATATTATTTTCAATGGGATAGAACATTAATACCATCTCATACTGGAACTAGTGG
CACACCAACAAATATATACCATGGTACAGATCCAGATGATGTTCAATTTTATACTATTGAAAATTCTG
TGCCAGTACACTTACTAAGAACAGGTGATGAATTTGCTACAGGAACATTTTTTTTTGATTGTAAACCA
TGTAGACTAACACATACATGGCAAACAAATAGAGCATTGGGCTTACCACCATTTCTAAATTCTTTGCC
TCAAGCTGAAGGAGGTACTAACTTTGGTTATATAGGAGTTCAACAAGATAAAAGACGTGGTGTAACTC
AAATGGGAAATACAAACTATATTACTGAAGCTACTATTATGAGACCAGCTGAGGTTGGTTATAGTGCA
CCATATTATTCTTTTGAGGCGTCTACACAAGGGCCATTTAAAACACCTATTGCAGCAGGACGGGGGGG
AGCGCAAACAGATGAAAATCAAGCAGCAGATGGTGATCCAAGATATGCATTTGGTAGACAACATGGTC
AAAAAACTACCACAACAGGAGAAACACCTGAGAGATTTACATATATAGCACATCAAGATACAGGAAGA
TATCCAGAAGGAGATTGGATTCAAAATATTAACTTTAACCTTCCTGTAACAAATGATAATGTATTGCT
ACCAACAGATCCAATTGGAGGTAAAACAGGAATTAACTATACTAATATATTTAATACTTATGGTCCTT
TAACTGCATTAAATAATGTACCACCAGTTTATCCAAATGGTCAAATTTGGGATAAAGAATTTGATACT
GACTTAAAACCAAGACTTCATGTAAATGCACCATTTGTTTGTCAAAATAATTGTCCTGGTCAATTATT
TGTAAAAGTTGCGCCTAATTTAACAAATGAATATGATCCTGATGCATCTGCTAATATGTCAAGAATTG
TAACTTACTCAGATTTTTGGTGGAAAGGTAAATTAGTATTTAAAGCTAAACTAAGAGCCTCTCATACT

TGGAATCCAATTCAACAAATGAGTATTAATGTAGATAACCAATTTAACTATGTACCAAGTAATATTGG
AGGTATGAAAATTGTCTATGAAAAATCTCAACTAGCACCTAGA
'THA_FJ869139_KU66_2003'
ATGAGTGATGGAGCAGTTCAACCAGACGGTGGTCAACCTGCTGTCAGAAATGAAAGAGCTACAGGATC
TGGGAACGGGTCTGGAGGCGGGGGTGGTGGTGGTTCTGGGGGTGTGGGGATTTCTACGGGTACTTTCA
ATAATCAGACAGAATTTAAATTTTTGGAAAACGGATGGGTGGAAATCACAGCAAACTCAAGCAGACTT
GTACATTTAAATATGCCAGAAAGTGAAAATTATAGAAGAGTGGTTGTAAATAATTTGGATAAAACTGC
AGTTAACGGAAACATGGCTTTAGATGATACCCATGCACAAATTGTAACACCTTGGTCATTGGTTGATG
CAAATGCTTGGGGAGTTTGGTTTAATCCAGGAGATTGGCAACTAATTGTTAATACTATGAGTGAGTTG
CATTTAGTTAGTTTTGAACAAGAAATTTTTAATGTTGTTTTAAAGACTGTTTCAGAATCTGCTACTCA
GCCACCAACTAAAGTTTATAATAATGATTTAACTGCATCATTGATGGTTGCATTAGATAGTAATAATA
CTATGCCATTTACTCCAGCAGCTATGAGATCTGAGACATTGGGTTTTTATCCATGGAAACCAACCATA
CCAACTCCATGGAGATATTATTTTCAATGGGATAGAACATTAATACCATCTCATACTGGAACTAGTGG
CACACCAACAAATATATACCATGGTACAGATCCAGATGATGTTCAATTTTATACTATTGAAAATTCTG
TGCCAGTACACTTACTAAGAACAGGTGATGAATTTGCTACAGGAACATTTTTTTTTGATTGTAAACCA
TGTAGACTAACACATACATGGCAAACAAATAGAGCATTGGGCTTACCACCATTTCTAAATTCTTTGCC
TCAAGCTGAAGGAGGTACTAACTTTGGTTATATAGGAGTTCAACAAGATAAAAGACGTGGTGTAACTC
AAATGGGAAATACAAACTATATTACTGAAGCTACTATTATGAGACCAGCTGAGGTTGGTTATAGTGCA
CCATATTATTCTTTTGAGGCGTCTACACAAGGGCCATTTAAAACACCTATTGCAGCAGGACGGGGGGG
AGCGCAAACAGATGAAAATCAAGCAGCAGATGGTGATCCAAGATATGCATTTGGTAGACAACATGGTC
AAAAAACTACCACAACAGGAGAAACACCTGAGAGATTTACATATATAGCACATCAAGATACAGGAAGA
TATCCAGAAGGAGATTGGATTCAAAATATTAACTTTAACCTTCCTGTAACAGATGATAATGTATTGCT
ACCAACAGATCCAATTGGAGGTAAAACAGGAATTAACTATACTAATATATTTAATACTTATGGTCCTT
TAACTGCATTAAATAATGTACCACCAGTTTATCCAAATGGTCAAATTTGGGATAAAGAATTTGATACT
GACTTAAAACCAAGACTTCATGTAAATGCACCATTTGTTTGTCAAAATAATTGTCCTGGTCAATTATT
TGTAAAAGTTGCGCCTAATTTAACAAATGAATATGATCCTGATGCATCTGCTAATATGTCAAGAATTG
TAACTTACTCAGATTTTTGGTGGAAAGGTAAATTAGTATTTAAAGCTAAACTAAGAGCCTCTCATACT
TGGAATCCAATTCAACAAATGAGTATCAATGTAGATAACCAATTTAACTATGTACCAAGTAATATTGG
AGGTATGAAAATTGTATATGAAAAATCTCAACTAGCACCTAGA
'CHI_GQ169553_Vac2_2007'
ATGAGTGATGGAGCAGTTCAACCAGACGGTGGTCAACCTGCTGTCAGAAATGAAAGAGCTACAGGATC
TGGGAACGGGTCTGGAGGCGGGGGTGGTGGTGGTTCTGGGGGTGTGGGGATTTCTACGGGTGCTTTCA
ATAATCAGACGGAATTTAAATTTTTGGAAAACGGATGGGTGGAAATCACAGCAAACTCAAGCAGACTT
GTACATTTAAATATGCCAGAAAGTGAAAATTATAGAAGAGTGGTTGTAAATAATATGGATAAAACTGC
AGTTAACGGAAACATGGCTTTAGATGATATTCATGCACAAATTGTAACACCTTGGTCATTGGTTGATG
CAAATGCTTGGGGAGTTTGGTTTAATCCAGGAGATTGGCAACTAATTGTTAATACTATGAGTGAGTTG
CATTTAGTTAGTTTTGAACAAGAAATCTTTAATGTTGTTTTAAAGACTGTTTCAGAATCTGCTACTCA
GCCACCAACTAAAGTTTATAATAATGATTTAACTGCATCATTGATGGTTGCATTAGATAGTAATAATA
CTATGCCATTTACTCCAGCAGCTATGAGATCTGAGACATTGGGTTTTTATCCATGGAAACCAACCATA
CCAACTCCATGGAGATATTATTTTCAATGGGATAGAACATTAATACCATCTCATACTGGAACTAGTGG
CACACCAACAAATATATATCATGGTACAGATCCAGATGATGTTCAATTTTATACTATTGAAAATTCTG
TGCCAGTACACTTACTAAGAACAGGTGATGAATTTGCTACAGGAACATTTTTTTTTGATTGTAGACCA
TGTAGACTAACACATACATGGCAAACAAATAGAGCATTGGGCTTACCACCATTTCTAAATTCTTTGCC
TCAATCTGAAGGAGCTACTAACTTTGGTGATATAGGAGTTCAACAAGATAAAAGACGTGGTATAACTC
AAATGGGAAATACAAACTATATTACTGAAGCTACTATTATGAGACCAGCTGAGGTTGGTTATAGTGCA
CCATATTATTCTTTTGAGGCGTCTACACAAGGGCCATTTAAAACACCTATTGCAGCAGGACGGGGGGG
AGCGCAAACAGATGAAAATCAAGCAGCAGATGGTAATCCAAGATATGCATTTGGTAGGCAACATGGTC
AAAAAACTACCACAACAGGAGAAACACCTGAGAGATTTACATATATAGCACATCAAGATACAGGAAGA
TATCCAGAAGGAGATTGGATTCAAAATATTAACTTTAACCTTCCTGTAACAAATGATAATGTATTGCT
ACCAACAGATCCAATTGGAGGTAAAACAGGAATTAACTATACTAATATATTTAATACTTATGGTCCTT
TAACTGCATTAAATAATGTACCACCAGTTTATCCAAATGGTCAAATTTGGGATAAAGAATTTGATACT
GACTTAAAACCAAGACTTCATGTAAATGCACCATTTGTTTGTCAAAATAATTGTCCTGGTCAATTATT
TGTAAAAGTTGCGCCTAATTTAACGAATGAATATGATCCTGATGCATCTGCTAATATGTCAAGAATTG
TAACTTACTCAGATTTTTGGTGGAAAGGTAAATTAGTATTTAAAGCTAAACTAAGAGCCTCTCATACT

TGGAATCCAATTCAACAAATGAGTATTAATGTAGATAGCCAATTTAACTATGTACCAAGTAATATTGG
AGGTATGAAAATTGTATATGAAAAATCTCAACTAGCACCTAGA
'CHI_GQ857596_CPV05_01_2005'
ATGAGTGATGGAGCAGTTCAACCAGACGGTGGTCAACCTGCTGTCAGAAATGAAAGAGCTACAGGATC
TGGGAACGGGTCTGGAGGCGGGGGTGGTGGTGGTTCTGGGGGTGTGGGGATTTCTACGGGTACTTTCA
ATAATCAGACAGAATTTAAATTTTTGGAAAACGGATGGGTGGAAATCACAGCAAACTCAAGCAGACTT
GTACATTTAAATATGCCAGAAAGTGAAAATTATAGAAGAGTGGTTGTAAATAATTTGGATAAAACTGC
AGTTAACGGAAACATGGCTTTAGATGATACCCATGCACAAATTGTAACACCTTGGTCATTGGTTGATG
CAAATGCTTGGGGAGTTTGGTTTAATCCAGGAGATTGGCAACTAATTGTTAATACTATGAGTGAATTG
CATTTAGTTAGTTTTGAACAAGAAATTTTTAATGTTGTTTTAAAGACTGTTTCAGAATCTGCTACTCA
GCCACCAACTAAAGTTTATAATAATGATTTAACTGCATCATTGATGGTTGCATTAGATAGTAATAATA
CTATGCCATTTACTCCAGCAGCTATGAGATCTGAGACATTGGGTTTTTATCCATGGAAACCAACCATA
CCAACTCCATGGAGATATTATTTTCAATGGGATAGAACATTAATACCATCTCATACTGGAACTAGTGG
CACACCAACAAATATATACCATGGTACAGATCCAGATGATGTTCAATTTTATACTATTGAAAATTCTG
TGCCAGTACACTTACTAAGAACAGGTGATGAATTTGCTACAGGAACATTTTTTTTTGATTGTAAACCA
TGTAGACTAACACATACATGGCAAACAAATAGAGCATTGGGCTTACCACCATTTCTAAATTCTTTGCC
TCAAGCTGAAGGAGGTACTAACTTTGGTTATATAGGAGTTCAACAAGATAAAAGACGTGGTGTAACTC
AAATGGGAAATACAAACTATATTACTGAAGCTACTATTATGAGACCAGCTGAGGTTGGTTATAGTGCA
CCATATTATTCTTTTGAGGCGTCTACACAAGGGCCATTTAAAACACCTATTGCAGCAGGACGGGGGGG
AGCGCAAACAGATGAAAATCAAGCAGCAGATGGTGATCCAAGATATGCATTTGGTAGACAACATGGTC
AAAAAACTACCACAACAGGAGAAACACCTGAGAGATTTACATATATAGCACATCAAGATACAGGAAGA
TATCCAGAAGGAGATTGGATTCAGAATATTAACTTTAACCTTCCTGTAACAAATGATAATGTATTGCT
ACCAACAGATCCAATTGGAGGTAAAACAGGAATTAACTATACTAATATATTTAATACTTATGGTCCTT
TAACTGCATTAAATAATGTACCACCAGTTTATCCAAATGGTCAAATTTGGGATAAAGAATTTGATACT
GACTTAAAACCAAGACTTCATGTAAATGCACCATTTGTTTGTCAAAATAATTGTCCTGGTCAATTATT
TGTAAAAGTTGCGCCTAATTTAACAAATGAATATGATCCTGATGCATCTGCTAATATGTCAAGAATTG
TAACTTACTCAGATTTTTGGTGGAAAGGTAAATTAGTATTTAAAGCTAAACTAAGAGCCTCTCATACT
TGGAATCCAATTCAACAAATGAGTATTAATGTAGATAACCAATTTAACTATGTACCAAGTAATATTGG
AGGTATGAAAATTGTATATGAAAAATCTCAACTAGCACCTAGA
'CHI_GQ857599_CPV05_04_2005'
ATGAGTGATGGAGCAGTTCAACCAGACGGTGGTCAACCTGCTGTCAGAAATGAAAGAGCTACAGGATC
TGGGAACGGGTCTGGAGGCGGGGGTGGTGGTGGTTCTGGGGGTGTGGGGATTTCTACGGGTACTTTCA
ATAATCAGACAGAATTTAAATTTTTGGAAAACGGATGGGTGGAAATCACAGCAAACTCAAGCAGACTT
GTACATTTAAATATGCCAGAAAGTGAAAATTATAGAAGAGTGGTTGTAAATAATTTGGATAAAACTGC
AGTTAACGGAAACATGGCTTTAGATGATACTCATGCACAAATTGTAACACCTTGGTCATTGGTTGATG
CAAATGCTTGGGGAGTTTGGTTTAATCCAGGAGATTGGCAACTAATTGTTAATACTATGAGTGAGTTG
CATTTAGTTAGTTTTGAACAAGAAATTTTTAATGTTGTTTTAAAGACTGTTTCAGAATCTGCTACTCA
GCCACCAACTAAAGTTTATAATAATGATTTAACTGCATCATTGATGGTTGCATTAGATAGTAATAATA
CTATGCCATTTACTCCAGCAGCTATGAGATCTGAGACATTGGGTTTTTATCCATGGAAACCAACCATA
CCAACTCCATGGAGATATTATTTTCAATGGGATAGAACATTAATACCATCTCATACTGGAACTAGTGG
CACACCAACAAATATATACCATGGTACAGATCCAGATGATGTTCAATTTTACACTATTGAAAATTCTG
TGCCAGTACACTTACTAAGAACAGGTGATGAATTTGCTACAGGAACATTTTATTTTGATTGTAAACCA
TGTAGACTAACACATACATGGCAAACAAATAGAGCATTGGGCTTACCACCATTTCTAAATTCTTTGCC
TCAAGCTGAAGGGGGTACTAACTTTGGTTATATAGGAGTTCAACAAGATAAAAGACGTGGTGTAACTC
AAATGGGAAATACAAACTATATTACTGAAGCTACTATTATGAGACCAGCTGAGGTTGGTTATAGTGCA
CCATATTATTCTTTTGAGGCGTCTACACAAGGGCCATTTAAAACACCTATTGCAGCAGGACGGGGGGG
AGCGCAAACAGATGAAAATCAAGCAGCAGATGGTGATCCAAGATATGCATTTGGTAGACAACATGGTC
AAAAAACTACCACAACAGGAGAAACACCTGAGAGATTTACATATATAGCACATCAAGATACAGGAAGA
TATCCAGAAGGAGATTGGATTCAGAATATTAACTTTAACCTTCCTGTAACAGATGATAATGTATTGCT
ACCAACAGATCCAATTGGAGGTAAAACAGGAATTAACTATACTAATATATTTAATACTTATGGTCCTT
TAACTGCATTAAATAATGTACCACCAGTTTATCCAAATGGTCAAATTTGGGATAAAGAATTTGATACT
GACTTAAAACCAAGACTTCATGTAAATGCACCATTTGTTTGTCAAAATAATTGTCCTGGTCAATTATT
TGTAAAAGTTGCGCCTAATTTAACAAATGAATATGATCCTGATGCATCTGCTAATATGTCAAGAATTG
TAACTTACTCAGATTTTTGGTGGAAAGGTAAATTAGTATTTAAAGCTAAACTAAGAGCCTCTCATACT

TGGAATCCAATTCAACAAATGAGTATCAATGTAGATAACCAATTTAACTATGTACCAAGTAATATTGG
AGGTATGAAAATTGTATATGAAAAATCTCAACTAGCACCTAGA
'CHI_GQ857600_CPV06_01_2006'
ATGAGTGATGGAGCAGTTCAACCAGACGGTGGTCAACCTGCTGTCAGAAATGAAAGAGCTACAGGATC
TGGGAACGGGTCTGGAGGCGGGGGTGGTGGTGGTTCTGGGGGTGTGGGGATTTCTACGGGTACTTTCA
ATAATCAGACAGAATTTAAATTTTTGGAAAACGGATGGGTGGAAATCACAGCAAACTCAAGCAGACTT
GTACATTTAAATATGCCAGAAAGTGAAAATTATAGAAGAGTGGTTGTAAATAATTTGGATAAAACTGC
AGTTAACGGAAACATGGCTTTAGATGATACCCATGCACAAATTGTAACACCTTGGTCATTGGTTGATG
CAAATGCTTGGGGAGTTTGGTTTAATCCAGGAGATTGGCAACTAATTGTTAATACTATGAGTGAGTTG
CATTTAGTTAGTTTTGAACAAGAAATTTTTAATGTTGTTTTAAAGACTGTTTCAGAATCTGCTACTCA
GCCACCAACTAAAGTTTATAATAATGATTTAACTGCATCATTGATGGTTGCATTAGATAGTAATAATA
CTATGCCATTTACTCCAGCAGCTATGAGATCTGAGACATTGGGTTTTTATCCATGGAAACCAACCATA
CCAACTCCATGGAGATATTATTTTCAATGGGATAGAACATTAATACCATCTCATACTGGAACTAGTGG
CACACCAACAAATATATACCATGGTACAGATCCAGATGATGTTCAATTTTATACTATTGAAAATTCTG
TGCCAGTACACTTACTAAGAACAGGTGATGAATTTGCTACAGGAACATTTTTTTTTGATTGTAAACCA
TGTAGACTAACACATACATGGCAAACAAATAGAGCATTGGGCTTACCACCATTTCTAAATTCTTTGCC
TCAATCTGAAGGAGGTACTAACTTTGGTTATATAGGAGTTCAACAAGATAAAAGACGTGGTGTAACTC
AAATGGGAAATACAAACTATATTACTGAAGCTACTATTATGAGACCAGCTGAGGTTGGTTATAGTGCA
CCATATTATTCTTTTGAGGCGTCTACACAAGGGCCATTTAAAACACCTATTGCAGCAGGACGGGGGGG
AGCGCAAACAGATGAAAATCAAGCAGCAGATGGTGATCCAAGATATGCATTTGGTAGACAACATGGTC
AAAAAACTACCACAACAGGAGAAACACCTGAGAGATTTACATATATAGCACATCAAGATACAGGAAGA
TATCCAGAAGGAGATTGGATTCAGAATATTAACTTTAACCTTCCTGTAACAAATGATAATGTATTGCT
ACCAACAGATCCAATTGGAGGTAAAACAGGAATTAACTATACTAATATATTTAATACTTATGGTCCTT
TAACTGCATTAAATAATGTACCACCAGTTTATCCAAATGGTCAAATTTGGGATAAAGAATTTGATACT
GACTTAAAACCAAGACTTCATGTAAATGCACCATTTGTTTGTCAAAATAATTGTCCTGGTCAATTATT
TGTAAAAGTTGCGCCTAATTTAACAAATGAATATGATCCTGATGCATCTGCTAATATGTCAAGAATTG
TAACTTACTCAGATTTTTGGTGGAAAGGTAAATTAGTATTTAAAGCTAAACTAAGAGCCTCTCATACT
TGGAATCCAATTCAACAAATGAGTATTAATGTAGATAACCAATTTAACTATGTACCAAGTAATATTGG
AGGTATGAAAATTGTATATGAAAAATCTCAACTAGCACCTAGA
'CHI_GQ857601_CPV06_02_2006'
ATGAGTGATGGAGCAGTTCAACCAGACGGTGGTCAACCTGCTGTCAGAAATGAAAGAGCTACAGGATC
TGGGAACGGGTCTGGAGGCGGGGGTGGTGGTGGTTCTGGGGGTGTGGGGATTTCTACGGGTACTTTCA
ATAATCAGACAGAATTTAAATTTTTGGAAAACGGATGGGTGGAAATCACAGCAAACTCAAGCAGACTT
GTACATTTAAATATGCCAGAAAGTGAAAATTATAGAAGAGTGGTTGTAAATAATTTGGATAAAACTGC
AGTTAACGGAAACATGGCTTTAGATGATACTCATGCACAAATTGTAACACCTTGGTCATTGGTTGATG
CAAATGCTTGGGGAGTTTGGTTTAATCCAGGAGATTGGCAACTAATTGTTAATACTATGAGTGAGTTG
CATTTAGTTAGTTTTGAACAAGAAATTTTTAATGTTGTTTTAAAGACTGTTTCAGAATCTGCTACTCA
GCCACCAACTAAAGTTTATAATAATGATTTAACTGCATCATTGATGGTTGCATTAGATAGTAATAATA
CTATGCCATTTACTCCAGCAGCTATGAGATCTGAGACATTGGGTTTTTATCCATGGAAACCAACCATA
CCAACTCCATGGAGATATTATTTTCAATGGGATAGAACATTAATACCATCTCATACTGGAACTAGTGG
CACACCAACAAATATATACCATGGTACAGATCCAGATGATGTTCAATTTTACACTATTGAAAATTCTG
TGCCAGTACACTTACTAAGAACAGGTGATGAATTTGCTACAGGAACATTTTATTTTGATTGTAAACCA
TGTAGACTAACACATACATGGCAAACAAATAGAGCATTGGGCTTACCACCATTTCTAAATTCTTTGCC
TCAAGCTGAAGGAGGTACTAACTTTGGTTATATAGGAGTTCAACAAGATAAAAGACGTGGTGTAACTC
AAATGGGAAATACAAACTATATTACTGAAGCTACTATTATGAGACCAGCTGAGGTTGGTTATAGTGCA
CCATATTATTCTTTTGAGGCGTCTACACAAGGGCCATTTAAAACACCTATTGCAGCAGGACGGGGGGG
AGCGCAAACAGATGAAAATCAAGCAGCAGATGGTGATCCAAGATATGCATTTGGTAGACAACATGGTC
AAAAAACTACCACAACAGGAGAAACACCTGAGAGATTTACATATATAGCACATCAAGATACAGGAAGA
TATCCAGAAGGAGATTGGATTCAGAATATTAACTTTAACCTTCCTGTAACAGATGATAATGTATTGCT
ACCAACAGATCCAATTGGAGGTAAAACAGGAATTAACTATACTAATATATTTAATACTTATGGTCCTT
TAACTGCATTAAATAATGTACCACCAGTTTATCCAAATGGTCAAATTTGGGATAAAGAATTTGATACT
GACTTAAAACCAAGACTTCATGTAAATGCACCATTTGTTTGTCAAAATAATTGTCCTGGTCAATTATT
TGTAAAAGTTGCGCCTAATTTAACAAATGAATATGATCCTGATGCATCTGCTAATATGTCAAGAATTG
TAACGTACTCAGATTTTTGGTGGAAAGGTAAATTAGTATTTAAAGCTAAACTAAGAGCCTCTCATACT

TGGAATCCAATTCAACAAATGAGTATCAATGTAGATAACCAATTTAACTATGTACCAAGTAATATTGG
AGGTATGAAAATTGTATATGAAAAATCTCAACTAGCACCTAGA
'CHI_GQ857605_CPV07_03_2007'
ATGAGTGATGGAGCAGTTCAACCAGACGGTGGTCAACCTGCTGTCAGAAATGAAAGAGCTACAGGATC
TGGGAACGGGTCTGGAGGCGGGGGTGGTGGTGGTTCTGGGGGTGTGGGGATTTCTACGGGTACTTTCA
ATAATCAGACGGAATTTAAATTTTTGGAAAACGGATGGGTGGAAATCACAGCAAACTCAAGCAGACTT
GTACATTTAAATATGCCAGAAAGTGAAAATTATAGAAGAGTGGTTGTAAATAATTTGGATAAAACTGC
AGTTAACGGAAACATGGCTTTAGATGATACTCATGCACAAATTGTAACACCTTGGTCATTGGTTGATG
CAAATGCTTGGGGAGTTTGGTTTAATCCAGGAGATTGGCAACTAATTGTTAATACTATGAGTGAGTTG
CATTTAGTTAGTTTTGAACAAGAAATTTTTAATGTTGTTTTAAAGACTGTTTCAGAATCTGCTACTCA
GCCACCAACTAAAGTTTATAATAATGATTTAACTGCATCATTGATGGTTGCATTAGATAGTAATAATA
CTATGCCATTTACTCCAGCAGCTATGAGATCTGAGACATTGGGTTTTTATCCATGGAAACCAACCATA
CCAACTCCATGGAGATATTATTTTCAATGGGATAGAACATTAATACCATCTCATACTGGAACTAGTGG
CACACCAACAAATATATACCATGGTACAGATCCAGATGATGTTCAATTTTACACTATTGAAAATTCTG
TGCCAGTACACTTACTAAAAACAGGTGATGAATTTGCTACAGGAACATTTTATTTTGATTGTAAACCA
TGTAGACTAACACATACATGGCAAACAAATAGAGCATTGGGCTTACCACCATTTCTAAATTCTTTGCC
TCAAGCTGAAGGAGGTACTAACTTTGGTTATATAGGAGTTCAACAAGATAAAAGACGTGGTGTAACTC
AAATGGGAAATACAAACTATATTACTGAAGCTACTATTATGAGACCAGCTGAGGTTGGTTATAGTGCA
CCATATTATTCTTTTGAGGCGTCTACACAAGGGCCATTTAAAACACCTATTGCAGCAGGACGGGGGGG
AGCGCAAACAGATGAAAATCAAGCAGCAGATGGTGATCCAAGATATGCATTTGGTAGACAACATGGTC
AAAAAACTACCACAACAGGAGAAACACCTGAGAGATTTACATATATAGCACATCAAGATACAGGAAGA
TATCCAGAAGGAGATTGGATTCAAAATATTAACTTTAACCTTCCTGTAACAGATGATAATGTATTGCT
ACCAACAGATCCAATTGGAGGTAAAACAGGAATTAACTATACTAATATATTTAATACTTATGGTCCTT
TAACTGCATTAAATAATGTACCACCAGTTTATCCAAATGGTCAAATTTGGGATAAAGAATTTGATACT
GACTTAAAACCAAGACTTCATGTAAATGCACCATTTGTTTGTCAAAATAATTGTCCTGGTCAATTATT
TGTAAAAGTTGCGCCTAATTTAACAAATGAATATGATCCTGATGCATCTGCTAATATGTCAAGAATTG
TAACTTACTCAGATTTTTGGTGGAAAGGTAAATTAGTATTTAAAGCTAAACTAAGAGCCTCTCATACT
TGGAATCCAATTCAACAAATGAGTATCAATGTAGATAACCAATTTAACTATGTACCAAGTAATATTGG
AGGTATGAAAATTGTATATGAAAAATCTCAACTAGCACCTAGA
'CHI_GQ857609_CPV08_01_2008'
ATGAGTGATGGAGCAGTTCAACCAGACGGTGGTCAACCTGCTGTCAGAAATGAAAGAGCTACAGGATC
TGGGAACGGGTCTGGAGGCGGGGGTGGTGGTGGTTCTGGGGGTGTGGGGATTTCTACGGGTACTTTCA
ATAATCAGACGGAATTTAAATTTTTGGAAAACGGATGGGTGGAAATCACAGCAAACTCAAGCAGACTT
GTACATTTAAATATGCCAGAAAGTGAAAATTATAGAAGAGTGGTTGTAAATAATTTGGATAAAACTGC
AGTTAACGGAAACATGGCTTTAGATGATACTCATGCACAAATTGTAACACCTTGGTCATTGGTTGATG
CAAATGCTTGGGGAGTTTGGTTTAATCCAGGAGATTGGCAACTAATTGTTAATACTATGAGTGAGTTG
CATTTAGTTAGTTTTGAACAAGAAATTTTTAATGTTGTTTTGAAGACTGTTTCAGAATCTGCTACTCA
GCCACCAACTAAAGTTTATAATAATGATTTAACTGCATCATTGATGGTTGCATTAGATAGTAATAATA
CTATGCCATTTACTCCAGCAGCTATGAGATCTGAGACATTGGGTTTTTATCCATGGAAACCAACCATA
CCAACTCCATGGAGATATTATTTTCAATGGGATAGAACATTAATACCATCTCATACTGGAACTAGTGG
CACACCAACAAATATATACCATGGTACAGATCCAGATGATGTTCAATTTTATACTATTGAAAATTCTG
TGCCAGTACACTTACTAAGAACAGGTGATGAATTTGCTACAGGAACATTTTTTTTTGATTGTAAACCA
TGTAGACTAACACATACATGGCAAACAAATAGAGCATTGGGCTTACCACCATTTCTAAATTCTTTGCC
TCAAGCTGAAGGAGGTACTAACTTTGGTTATATAGGAGTTCAACAAGATAAAAGACGTGGTGTAACTC
AAATGGGAAATACAAACTATATTACTGAAGCTACTATTATGAGACCAGCTGAGGTTGGTTATAGTGCA
CCATATTATTCTTTTGAGGCGTCTACACAAGGGCCATTTAAAACACCTATTGCAGCAGGACGGGGGGG
AGCGCAAACAGATGAAAATCAAGCAGCAGATGGTGATCCAAGATATGCATTTGGTAGACAACATGGTC
AAAAAACTACCACAACAGGAGAAACACCTGAGAGATTTACATATATAGCACATCAAGATACAGGAAGA
TATCCAGAAGGAGATTGGATTCAGAATATTAACTTTAACCTTCCTGTAACAGATGATAATGTTTTGCT
ACCAACAGATCCAATTGGAGGTAAAACAGGAATTAACTATACTAATATATTTAATACTTATGGTCCTT
TAACTGCATTAAATAATGTACCACCAGTTTATCCAAATGGTCAAATTTGGGATAAAGAATTTGATACT
GACTTAAAACCAAGACTTCATGTAAATGCACCATTTGTTTGTCAAAATAATTGTCCTGGTCAATTATT
TGTAAAAGTTGCGCCTAATTTAACAAATGAATATGATCCTGATGCATCTGCTAATATGTCAAGAATTG
TAACTTACTCAGATTTTTGGTGGAAAGGTAAATTAGTATTTAAAGCTAAACTAAGAGCCTCTCATACT

TGGAATCCAATTCAACAAATGAGTATCAATGTAGATAACCAATTTAACTATGTACCAAGTAATATTGG
AGGTATGAAAATTGTATATGAAAAATCTCAACTAGCACCTAGA
'CHI_GQ857612_CPV08_04_2008'
ATGAGTGATGGAGCAGTTCAACCAGACGGTGGTCAACCTGCTGTCAGAAATGAAAGAGCTACAGGATC
TGGGAACGGGTCTGGAGGCGGGGGTGGTGGTGGTTCTGGGGGTGTGGGGATTTCTACGGGTACTTTCA
ATAATCAGACGGAATTTAAATTTTTGGAAAACGGATGGGTGGAAATCACAGCAAACTCAAGCAGACTT
GTACATTTAAATATGCCAGAAAGTGAAAATTATAGAAGAGTGGTTGTAAATAATTTGGATAAAACTGC
AGTTAACGGAAACATGGCTTTAGATGATACTCATGCACAAATTGTAACACCTTGGTCATTGGTTGATG
CAAATGCTTGGGGAGTTTGGTTTAATCCAGGAGATTGGCAACTAATTGTTAATACTATGAGTGAGTTG
CATTTAGTTAGTTTTGAACAAGAAATTTTTAATGTTGTTTTAAAGACTGTTTCAGAATCTGCTACTCA
GCCACCAACTAAAGTTTATAATAATGATTTAACTGCATCATTGATGGTTGCATTAGATAGCAATAATA
CTATGCCATTTACTCCAGCAGCTATGAGATCTGAGACATTGGGTTTTTATCCATGGAAACCAACCATA
CCAACTCCATGGAGATATTATTTTCAATGGGATAGAACATTAATACCATCTCATACTGGAACTAGTGG
CACACCAACAAATATATACCATGGTACAGATCCAGATGATGTTCAATTTTATACTATTGAAAATTCTG
TACCAGTACACTTACTAAGAACAGGTGATGAATTTGCTACAGGAACATTTTTTTTTGATTGTAAACCA
TGTAGACTAACACATACATGGCAAACAAATAGAGCATTGGGCTTACCACCATTTCTAAATTCTTTGCC
TCAAGCTGAAGGAGGTACTAACTTTGGTTATATAGGAGTTCAACAAGATAAAAGACGTGGTGTAACTC
AAATGGGAAATACAAACTATATTACTGAAGCTACTATTATGAGACCAGCTGAGGTTGGTTATAGTGCA
CCATATTATTCTTTTGAGGCGTCTACACAAGGGCCATTTAAAACACCTATTGCAGCAGGACGGGGGGG
AGCGCAAACAGATGAAAATCAAGCAGCAGATGGTGATCCAAGATATGCATTTGGTAGACAACATGGTC
AAAAAACTACCACAACAGGAGAAACACCTGAGAGATTTACATATATAGCACATCAAGATACAGGAAGA
TATCCAGAAGGAGATTGGATTCAGAATATTAACTTTAACCTTCCTGTAACAAATGATAATGTATTGCT
ACCAACAGATCCAATTGGAGGTAAAACAGGAATTAACTATACTAATATATTTAATACTTATGGTCCTT
TAACTGCATTAAATAATGTACCACCAGTTTATCCAAATGGTCAAATTTGGGATAAAGAATTTGATACT
GACTTAAAACCAAGACTTCATGTAAATGCACCATTTGTTTGTCAAAATAATTGTCCTGGTCAATTATT
TGTGAAAGTTGCGCCTAATTTAACAAATGAATATGATCCTGATGCATCTGCTAATATGTCAAGAATTG
TAACTTACTCAGATTTTTGGTGGAAAGGTAAATTAGTATTTAAAGCTAAACTAAGAGCCTCTCATACT
TGGAATCCAATTCAACAAATGAGTATTAATGTAGATAACCAATTTAACTATGTACCAAGTAATATTGG
AGGTATGAAGATTGTATATGAAAAATCTCAACTAGCACCTAGA
'VAC_GU212790_primodog_2009'
ATGAGTGATGGAGCAGTTCAACCAGACGGTGGTCAACCTGCTGTCAGAAATGAAAGAGCTACAGGATC
TGGGAACGGGTCTGGAGGCGGGGGTGGGGGTGGGTCTGGGGGTGGGGGGATTTCTACGGGTGCTTTCA
ATAATCAGACGGAATTTAAATTTTTGGAAAACGGATGGGTGGAAATCACAGCAAACTCAAGCAGACTT
GTACATTTAAATATGCCAGAAAGTGAAAATTATAGAAGAGTGGTTGTAAATAATATGGATAAAACTGC
AGTTAACGGAAACATGGCTTTAGATGATATTCATGCACAAATTGTAACACCTTGGTCATTGGTTGATG
CAAATGCTTGGGGAGTTTGGTTTAATCCAGGAGATTGGCAACTAATTGTTAATACTATGAGTGAGTTG
CATTTAGTTAGTTTTGAACAAGAAATTTTTAATGTTGTTTTAAAGACTGTTTCAGAATCTGCTACTCA
GCCACCAACTAAAGTTTATAATAATGATTTAACTGCATCATTGATGGTTGCATTAGATAGTAATAATA
CTATGCCATTTACTCCAGCAGCTATGAGATCTGAGACATTGGGTTTTTATCCATGGAAACCAACCATA
CCAACTCCATGGAGATATCATTTTCAATGGGATAGAACATTAATACCATCTCATACTGGAACTAGTGG
CACACCAACAAATATATACCATGGTACAGATCCAGATGATGTTCAATTTTATACTATTGAAAATTCTG
TGCCAGTACACTTACTAAGAACAGGTGATGAATTTGCTACAGGAACATTTTTTTTTGATTGTACACCA
TGTAGACTAACACATACATGGCAAACAAATAGAGCATTGGGCTTACCACCATTTCTAAATTCTTTGCC
TCAATCTGAAGGAGCTACTAACTTTGGTGATATAGGAGTTCAACAAGATAAAAGACGTGGTGTAACTC
AAATGGGAAATACAAACTATATTACTGAAGCTACTATTATGAGACCAGCTGAGGTTGGTTATAGTGCA
CCATATTATTCTTTTGAGGCGTCTACACAAGGGCCATTTAAAACACCTATTGCAGCAGGACGGGGGGG
AGCGCAAACAGATGAAAATCAAGCAGCAGATGGTGAACCAAGATATGCATTTGGTAGACAACATGGTC
AAAAAACTACCACAACAGGAGAAACACCTGAGAGATTTACATATATAGCACATCAAGATACAGGAAGA
TATCCAGAAGGAGATTGGATTCAAAATATTAACTTTAACCTTCCTGTAACGAATGATAATGTATTGCT
ACCAACAGATCCAATTGGAGGTAAAACAGGAATTAACTATACTAATATATTTAATACTTATGGTCCTT
TAACTGCATTAAATAATGTACCACCAGTTTATCCAAATGGTCAAATTTGGGATAAAGAATTTGATACT
GACTTAAAACCAAGACTTCATGTAAATGCACCATTTGTTTGTCAAAATAATTGTCCTGGTCAATTATT
TGTAAAAGTTGCGCCTAATTTAACAAATGAATATGATCCTGATGCATCTGCTAATATGTCAAGAATTG
TAACTTACTCAGATTTTTGGTGGAAAGGTAAATTAGTATTTAAAGCTAAACTAAGAGCCTCTCATACT

TGGAATCCAATTCAACAAATGAGTATTAATGTAGATAACCAATTTAACTATGTACCAAGTAATATTGG
AGGTATGAAAATTGTATTTGAAAAATCTCAACTAGCACCTAGA
'VAC_GU212791_vanguard_2009'
ATGAGTGATGGAGCAGTTCAACCAGACGGTGGTCAACCTGCTGTCAGAAATGAAAGAGCTACAGGATC
TGGGAACGGGTCTGGAGGCGGGGGTGGGGGTGGGTCTGGGGGTGGGGGGATTTCTACGGGTGCTTTCA
ATAATCAGACGGAATTTAAATTTTTGGAAAACGGATGGGTGGAAATCACAGCAAACTCAAGCAGACTT
GTACATTTAAATATGCCAGAAAGTGAAAATTATAGAAGAGTGGTTGTAAATAATATGGATAAAACTGC
AGTTAACGGAAACATGGCTTTAGATGATATTCATGCACAAATTGTAACACCTTGGTCATTGGTTGATG
CAAATGCTTGGGGAGTTTGGTTTAATCCAGGAGATTGGCAACTAATTGTTAATACTATGAGTGAGTTG
CATTTAGTTAGTTTTGAACAAGAAATTTTTAATGTTGTTTTAAAGACTGTTTCAGAATCTGCTACTCA
GCCACCAACTAAAGTTTATAATAATGATTTAACTGCATCATTGATGGTTGCATTAGATAGTAATAATA
CTATGCCATTTACTCCAGCAGCTATGAGATCTGAGACATTGGGTTTTTATCCATGGAAACCAACCATA
CCAACTCCATGGAGATATTATTTTCAATGGGATAGGACATTAATACCATCTCATACTGGAACTAGTGG
CACACCAACAAATATATACCATGGTACAGATCCAGATGATGTTCAATTTTATACTATTGAAAATTCTG
TGCCAGTACACTTACTAAGAACAGGTGATGAATTTGCTACAGGAACATTTTTTTTTGATTGTAAACCA
TGTAGACTAACACATACATGGCAAACAAATAGAGCATTGGGCTTACCACCATTTCTAAATTCTTTGCC
TCAATCTGAAGGAGCTACTAACTTTGGTGATATAGGAGTTCAACAAGATAAAAGACGTGGTGTAACTC
AAATGGGAAATACAAACTATATTACTGAAGCTACTATTATGAGACCAGCTGAGGTTGGTTATAGTGCA
CCATATTATTCTTTTGAGGCGTCTACACAAGGGCCATTTAAAACACCTATTGCAGCAGGACGGGGGGG
AGCGCAAACAGATGAAAATCAAGCAGCAGATGGTGAACCAAGATATGCATTTGGTAGACAACATGGTC
AAAAAACTACCACAACAGGAGAAACACCTGAGAGATTTACATATATAGCACATCAAGATACAGGAAGA
TATCCAGAAGGAGATTGGATTCAAAATATTAACTTTAACCTTCCTGTAACGAATGATAATGTATTGCT
ACCAACAGATCCAATTGGAGGTAAAACAGGAATTAACTATACTAATATATTTAATACTTATGGTCCTT
TAACTGCATTAAATAATGTACCACCAGTTTATCCAAATGGTCAAATTTGGGATAAAGAATTTGATACT
GACTTAAAACCAAGACTTCATGTAAATGCACCATTTGTTTGTCAAAATAATTGTCCTGGTCAATTATT
TGTAAAAGTTGCGCCTAATTTAACAAATGAATATGATCCTGATGCATCTGCTAATATGTCAAGAATTG
TAACTTACTCAGATTTTTGGTGGAAAGGTAAATTAGTATTTAAAGCTAAACTAAGAGCCTCTCATACT
TGGAATCCAATTCAACAAATGAGTATTAATGTAGATAACCAATTTAACTATGTACCAAGTAATATTGG
AGGTATGAAAATTGTATTTGAAAAATCTCAACTAGCACCTAGA
'ITA_GU362932_cat11_2008'
ATGAGTGATGGAGCAGTTCAACCAGACGGTGGTCAACCTGCTGTCAGAAATGAAAGAGCTACAGGATC
TGGGAACGGGTCTGGAGGCGGGGGTGGTGGTGGTTCTGGGGGTGTGGGGATTTCTACGGGTACTTTCA
ATAATCAGACGGAATTTAAATTTTTGGAAAACGGATGGGTGGAAATCACAGCAAACTCAAGCAGACTT
GTACATTTAAATATGCCAGAAAGTGAAAATTATAGAAGAGTGGTTGTAAATAATTTGGATAAAACTGC
AGTTAACGGAAACATGGCTTTAGATGATACTCATGCACAAATTGTAACACCTTGGTCATTGGTTGATG
CAAATGCTTGGGGAGTTTGGTTTAATCCAGGAGATTGGCAACTAATTGTTAATACTATGAGTGAGTTG
CATTTAGTTAGTTTTGAACAAGAAATTTTTAATGTTGTTTTAAAGACTGTTTCAGAATCTGCTACTCA
GCCACCAACTAAAGTTTATAATAATGATTTAACTGCATCATTGATGGTTGCATTAGATAGCAATAATA
CTATGCCATTTACTCCAGCAGCTATGAGATCTGAGACATTGGGTTTTTATCCATGGAAACCAACCATA
CCAACTCCATGGAGATATTATTTTCAATGGGATAGAACATTAATACCATCTCATACTGGAACTAGTGG
CACACCAACAAATATATACCATGGTACAGATCCAGATGATGTTCAATTTTATACTATTGAAAATTCTG
TGCCAGTACACTTACTAAGAACAGGTGATGAATTTGCTACAGGAACATTTTTTTTTGATTGTAAACCA
TGTAGACTAACACATACATGGCAAACAAATAGAGCATTGGGCTTACCACCATTTCTAAATTCTTTGCC
TCAAGCTGAAGGAGGTACTAACTTTGGTTATATAGGAGTTCAACAAGATAAAAGACGTGGTGTAACTC
AAATGGGAAATACAAACTATATTACTGAAGCTACTATTATGAGACCAGCTGAGGTTGGTTATAGTGCA
CCATATTATTCTTTTGAGGCGTCTACACAAGGGCCATTTAAAACACCTATTGCAGCAGGACGGGGGGG
AGCACAAACAGATGAAAATCAAGCAGCAGATGGTGATCCAAGATATGCATTTGGTAGACAACATGGTC
AAAAAACTACCACAACAGGAGAAACACCTGAGAGATTTACATATATAGCACATCAAGATACAGGAAGA
TATCCAGAAGGAGATTGGATTCAAAATATTAACTTTAACCTTCCTGTAACAAATGATAATGTATTGCT
ACCAACAGATCCAATTGGAGGTAAAACAGGAATTAACTATACTAATATATTTAATACTTATGGTCCTT
TAACTGCATTAAATAATGTACCACCAGTTTATCCAAATGGTCAAATTTGGGATAAAGAATTTGATACT
GACTTAAAACCAAGACTTCATGTAAATGCACCATTTGTTTGTCAAAATAATTGTCCTGGTCAATTATT
TGTAAAAGTTGCGCCTAATTTAACAAATGAATATGATCCTGATGCATCTGCTAATATGTCAAGAATTG
TAACTTACTCAGATTTTTGGTGGAAAGGTAAATTAGTATTTAAAGCTAAACTAAGAGCCTCTCATACT

TGGAATCCAATTCAACAAATGAGTATTAATGTAGATAACCAATTTAACTATGTACCAAATAATATTGG
AGCTATGAAAATTGTATATGAAAAATCTCAACTAGCACCTAGA
'CHI_GU392236_fox_HB1_2009'
ATGAGTGATGGAGCAGTTCAACCAGACGGTGGTCAATCTGCTGTCAGAAATGAAAGAGCTACAGGATC
TGGGAACGGGTCTGGAGGCGGGGGTGGTGGTGGTTCTGGGGGTGTGGGGATTTCTACGGGTACTTTCA
ATAATCAGACGGAATTTAAATTTTTGGAAAACGGATGGGTGGAAATCACAGCAAACTCAAGCAGACTT
GTACATTTAAATATGCCAGAAAGTGAAAATTATAGAAGAGTGGTTGTGAATAATATGGATAAAACTGC
AGTTAACGGAAACATGGCTTTAGATGATATTCATGCACAAATTGTAACACCTTGGTCATTGGTTGATG
CAAATGCTTGGGGAGTTTGGTTTAATCCAGGAGATTGGCAACTAATTGTTAATACTATGAGTGAGTTG
CATTTAGTTAGTTTTGAACAAGAAATTTTTAATGTTGTTTTAAAGACTGTTTCAGAATCTGCTACTCA
GCCACCAACTAAAGTTTATAATAATGATTTAACTGCATCATTGATGGTTGCATTAGATAGTAATAATA
CTATGCCATTTACTCCAGCAGCTATGAGATCTGAGACATTGGGTTTTTATCCATGGAAACCAACCATA
CCAACTCCATGGAGATATTATTTTCAATGGGATAGAACATTAGTACCATCTCATACTGGAACTAGTGG
CACACCAACAAATATATACCATGGTACAGATCCAGATGATGTTCAATTTTATACTATTGAAAATTCTG
TGCCAGTACACTTACTAAGAACAGGTGATGAATTTGCTACAGGAACATTTTTTTTTGATTGTAAACCA
TGTAGACTAACACATACATGGCAAACAAATAGAGCATTGGGCTTACCACCATTTCTAAATTCTTTGCC
TCAATCTGAAGGAGCTACTAACTTTGGTGATATAGGAGTTCAACAAGATAAAAGACGTGGTGTAACTC
AAATGGGAAATACAAACTATATTACTGAAGCTACTATTATGAGACCAGCTGAGGTTGGTTATAGTGCA
CCATATTATTCTTTTGAGGCGTCTACACAAGGGCCATTTAAAACACCTATTGCAGCAGGACGGGGGGG
AGCGCAAACAGATGAAAATCAAGCAGCAGATGGTGATCCAAGATATGCATTTGGTAGACAACATGGTC
AAAAAACTACCACAACAGGAGAAACACCTGAGAGATTTACATATATAGCACATCAAGATACAGGAAGA
TATCCAGAAGGAGATTGGATTCAAAATATTAACTTTAACCTTCCTGTAACAAATGATAATGTATTGCT
ACCAACAGATCCAATTGGAGGTAAAACAGGAATTAACTATACTAATATATTTAATACTTATGGTCCTT
TAACTGCATTAAATAATGTACCACCAGTTTATCCAAATGGTCAAATTTGGGATAAAGAATTTGATACT
GACTTAAAACCAAGACTTCATGTAAATGCACCATTTGTTTGTCAAAATAATTGTCCTGGTCAATTATT
TGTAAAAGTTGCGCCTAATTTAACAAATGAATATGATCCTGATGCATCTGCTAATATGTCAAGAATTG
TAACTTACTCAGATTTTTGGTGGAAAGGTAAATTAGTATTTAAAGCTAAACTAAGAGCCTCTCATACT
TGGAATCCAATTCAACAAATGAGTATTAATGTAGATAACCAATTTAACTATCTACCAAGTAATATTGG
AGGTATGAAAATTGTATATGAAAAATCTCAACTAGCACCTAGA
'CHI_GU392237_fox_HB2_2009'
ATGAGTGATGGAGCAGTTCAACCAGACGGTGGTCAGCCTGCTGTCAGAAATGAAAGAGCTACAGGATC
TGGGAACGGGTCTGGAGGCGGGGGTGGTGGTGGTTCTGGGGGTGTGGGGATTTCTACGGGTACTTTCA
ATAATCAGACGGAATTTAAATTTTTGGAAAACGGATGGGTGGAAATCACAGCAAACTCAAGCAGACTT
GTACATTTAAATATGCCAGAAAGTGAAAATTATAGAAGAGTGGTTGTGAATAATATGGATAAAACTGC
AGTTAACGGAAACATGGCTTTAGATGATATTCATGCACAAATTGTAACACCTTGGTCATTGGTTGATG
CAAATGCTTGGGGAGTTTGGTTTAATCCAGGAGATTGGCAACTAATTGTTAATACTATGAGTGAGTTG
CATTTAGTTAGTTTTGAACAAGAAATTTTTAATGTTGTTTTAAAGACTGTTTCAGAATCTGCTACTCA
GCCACCAACTAAAGTTTATAATAATGATTTAACTGCATCATTGATGGTTGCATTAGATAGTAATAATA
CTATGCCATTTACTCCAGCAGCTATGAGATCTGAGACATTGGGCTTTTATCCATGGAAACCAACCATA
CCAACTCCATGGAGATATTATTTTCAATGGGATAGAACATTAGTACCATCTCATACTGGAACTAGTGG
CACACCAACAAATATATACCATGGTACAGATCCAGATGATGTTCAATTTTATACTATTGAAAATTCTG
TGCCAGTACACTTACTAAGAACAGGTGATGAATTTGCTACAGGAACATTTTTTTTTGATTGTAAACCA
TGTAGACTAACACATACATGGCAAACAAATAGAGCATTGGGCTTACCACCATTTCTAAATTCTTTGCC
TCAATCTGAAGGAGCTACTAACTTTGGTGATATAGGAGTTCAACAAGATAAAAGACGTGGTGTAACTC
AAATGGGAAATACAAACTATATTACTGAAGCTACTATTATGAGACCAGCTGAGGTTGGTTATAGTGCA
CCATATTATTCTTTTGAGGCGTCTACACAAGGGCCATTTAAAACACCTATTGCAGCAGGACGGGGGGG
AGCGCAAACAGATGAAAATCAAGCAGCAGATGGTGATCCAAGATATGCATTTGGTAGACAACATGGTC
AAAAAACTACCACAACAGGAGAAACACCTGAGAGATTTACATATATAGCACATCAAGATACAGGAAGA
TATCCAGAAGGAGATTGGATTCAAAATATTAACTTTAACCCTCCTGTAACAAATGATAATGTATTGCT
ACCAACAGATCCAATTGGAGGTAAAACAGGAATTAACTATACTAATATATTTAATACTTATGGTCCTT
TAACTGCATTAAATAATGTACCACCAGTTTATCCAAATGGTCAAATTTGGGATAAAGAATTTGATACT
GACTTAAAACCAAGACTTCATGTAAATGCACCATTTGTTTGTCAAAATAATTGTCCTGGTCAATTATT
TGTAAAAGTTGCGCCTAATTTAACAAATGAATATGATCCTGATGCATCTGCTAATATGTCAAGAATTG
TAACTTACTCAGATTTTTGGTGGAAAGGTAAATTAGTATTTAAAGCTAAACTAAGAGCCTCTCATACT

TGGAATCCAATTCAACAAATGAGTATTAATGTAGATAACCAATTTAACTATCTACCAAGTAATATTGG
AGGTATGAAAATTGTATATGAAAAATCTCAACTAGCACCTAGA
'CHI_GU392239_raccoondog_HB6_2009'
ATGAGTGATGGAGCAGTTCAACCAGACGGTGGTCAACCTGCTGTCAGAAATGAAAGAGCTACAGGATC
TGGGAACGGGTCTGGAGGCGGGGGTGGTGGTGGTTCTGGGGGTGTGGGGATTTTTACGGGTACTTTCA
ATAATCAGACGGAATTTAAATTTTTGGAAAACGGATGGGTGGAAATCACAGCAAACTCAAGCAGACTT
GTACATTTAAATATGCCAGAAAGTGAAAATTATAGAAGAGTGGTTGTGAATAATATGGATAAAACTGC
AGTTAACGGAAACATGGCTTTAGATGATATTCATGCACAAATTGTAACACCTTGGTCATTGGTTGATG
CAAATGCTTGGGGAGTTTGGTTTAATCCAGGAGATTGGCAACTAATTGTTAATACTATGAGTGAGTTG
CATTTAGTTAGTTTTGAACAAGAAATTTTTAATGTTGTTTTAAAGACTGTTTCAGAATCTGCTACTCA
GCCACCAACTAAAGTTTATAATAATGATTTAACTGCATCATTGATGGTTGCATTAGATAGTAATAATA
CTATGCCATTTACTCCAGCAGCTATGAGATCTGAGACATTGGGTTTTTATCCATGGAAACCAACCATA
CCAACTCCATGGAGATATTATTTTCAATGGGATAGAACATTAGTACCATCTCATACTGGAACTAGTGG
CACACCAACAAATATATACCATGGTACAGATCCAGATGATGTTCAATTTTATACTATTGAAAATTCTG
TGCCAGTACACTTACTAAGAACAGGTGATGAATTTGCTACAGGAACATTTTTTTTTGATTGTAAACCA
TGTAGACTAACACATACATGGCAAACAAATAGAGCATTGGGCTTACCACCATTTCTAAATTCTTTGCC
TCAATCTGAAGGAGCTACTAACTTTGGTGATATAGGAGTTCAACAAGATAAAAGACGTGGTGTAACTC
AAATGGGAAATACAAACTATATTACTGAAGCTACTATTATGAGACCAGCTGAGGTTGGTTATAGTGCA
CCATATTATTCTTTTGAGGCGTCTACACAAGGGCCATTTAAAACACCTATTGCAGCAGGACGGGGGGG
AGCGCAAACAGATGAAAATCAAGCAGCAGATGGTGATCCAAGATATGCATTTGGTAGACAACATGGTC
AAAAAACTACCACAACAGGAGAAACACCTGAGAGATTTACATATATAGCACATCAAGATACAGGAAGA
TATCCAGAAGGAGATTGGATTCAAAATATTAACTTTAACCTTCCTGTAACAAATGATAATGTATTGCT
ACCAACAGATCCAATTGGAGGTAAAACAGGAATTAACTATACTAATATATTTAATACTTATGGTCCTT
TAACTGCATTAAATAATGTACCACCAGTTTATCCAAATGGTCAAATTTGGGATAAAGAATTTGATACT
GACTTAAAACCAAGACTTCATGTAAATGCACCATTTGTTTGTCAAAATAATTGTCCTGGTCAATTATT
TGTAAAAGTTGCGCCTAATTTAACAAATGAATATGATCCTGATGCATCTGCTAATATGTCAAGAATTG
TAACTTACTCAGATTTTTGGTGGAAAGGTAAATTAGTATTTAAAGCTAAACTAAGAGCCTCTCATACT
TGGAATCCAATTCAACAAATGAGTATTAATGTAGATAACCAATTTAACTATCTACCAAGTAATATTGG
AGGTATGAAAATTGTATATGAAAAATCTCAACTAGCACCTAGA
'CHI_GU392240_raccoondog_HB3_2009'
ATGAGTGATGGAGCAGTTCAACCAGACGGTGGTCAACCTGCTGTCAGAAATGAAAGAGCTACAGGATC
TGGGAACGGGTCTGGAGGCGGGGGTGGTGGTGGTTCTGGGGGTGTGGGGATTTCTACGGGTACTTTCA
ATAATCAGACGGAATTTAAATTTTTGGAAAACGGATGGGTGGAAATCACAGCAAACTCAAGCAGACTT
GTACATTTAAATATGCCAGAAAGTGAAAATTATAGAAGAGTGGTTGTGAATAATATGGATAAAACTGC
AGTTAACGGAAACATGGCTTTAGATGATATTCATGCACAAATTGTAACACCTTGGTCATTGGTTGATG
CAAATGCTTGGGGAGTTTGGTTTAATCCAGGAGATTGGCAACTAATTGTTAATACTATGAGTGAGTTG
CATTTAGTTAGTTTTGAACAAGAAATTTTTAATGTTGTTTTAAAGACTGTTTCAGAATCTGCTACTCA
GCCACCAACTAAAGTTTATAATAATGATTTAACTGCATCATTGATGGTTGCATTAGATAGTAATAATA
CTATGCCATTTACTCCAGCAGCTATGAGATCTGAGACATTGGGTTTTTATCCATGGAAACCAACCATA
CCAACTCCATGGAGATATTATTTTCAATGGGATAGAACATTAGTACCATCTCATACTGGAACTAGTGG
CACACCAACAAATATATACCATGGTACAGATCCAGATGATGTTCAATTTTATACTATTGAAAATTCTG
TGCCAGTACACTTACTAAGAACAGGTGATGAATTTGCTACAGGAACATTTTTTTTTGATTGTAAACCA
TGTAGACTAACACATACATGGCAAACAAATAGAGCATTGGGCTTACCACCATTTCTAAATTCTTTGCC
TCAATCTGAAGGAGCTACTAACTTTGGTGATATAGGAGTTCAACAAGATAAAAGACGTGGTGTAACTC
AAATGGGAAATACAAACTATATTACTGAAGCTACTATTATGAGACCAGCTGAGGTTGGTTATAGTGCA
CCATATTATTCTTTTGAGGCGTCTACACAAGGGCCATTTAAAACACCTATTGCAGCAGGACGGGGGGG
AGCGCAAACAGATGAAAATCAAGCAGCAGATGGTGATCCAAGATATGCATTTGGTAGACAACATGGTC
AAAAAACTACCACAACAGGAGAAACACCTGAGAGATTTACATATATAGCACATCAAGATACAGGAAGA
TATCCAGAAGGAGATTGGATTCAAAATATTAACTTTAACCTTCCTGTAACAAATGATAATGTATTGCT
ACCAACAGATCCAATTGGAGGTAAAACAGGAATTAACTATACTAATATATTTAATACTTATGGTCCTT
TAACTGCATTAAATAATGTACCACCAGTTTATCCAAATGGTCAAATTTGGGATAAAGAATTTGATACT
GACTTAAAACCAAGACTTCATGTAAATGCACCATTTGTTTGTCAAAATAATTGTCCTGGTCAATTATT
TGTAAAAGTTGCGCCTAATTTAACAAATGAATATGATCCTGATGCATCTGCTAATATGTCAAGAATTG
TAACTTACTCAGATTTTTGGTGGAAAGGTAAATTAGTATTTAAAGCTAAACTAAGAGCCTCTCATACT

TGGAATCCAATTCAACAAATGAGTATTAATGTAGATAACCAATTTAACTATCTACCAAGTAATATTGG
AGGTATGAAAATTGTATATGAAAAATCTCAACTAGCACCTAGA
'CHI_GU392241_raccoondog_HB1_2009'
ATGAGTGATGGAGCAGTTCAACCAGACGGTGGTCAATCTGCTGTCAGAAATGAAAGAGCTACAGGATC
TGGGAACGGGTCTGGAGGCGGGGGTGGTGGTGGTTCTGGGGGTGTGGGGATTTCTACGGGTACTTTCA
ATAATCAGACGGAATTTAAATTTTTGGAAAACGGATGGGTGGAAATCACAGCAAACTCAAGCAGACTT
GTACATTTAAATATGCCAGAAAGTGAAAATTATAGAAGAGTGGTTGTGAATAATATGGATAAAACTGC
AGTTAACGGAAACATGGCTTTAGATGATATTCATGCACAAATTGTAACACCTTGGTCATTGGTTGATG
CAAATGCTTGGGGAGTTTGGTTTAATCCAGGAGATTGGCAACTAATTGTTAATACTATGAGTGAGTTG
CATTTAGTTAGTTTTGAACAAGAAATTTTTAATGTTGTTTTAAAGACTGTTTCAGAATCTGCTACTCA
GCCACCAACTAAAGTTTATAATAATGATTTAACTGCATCATTGATGGTTGCATTAGATAGTAATAATA
CTATGCCATTTACTCCAGCAGCTATGAGATCTGAGACATTGGGTTTTTATCCATGGAAACCAACCATA
CCAACTCCATGGAGATATTATTTTCAATGGGATAGAACATTAGTACCATCTCATACTGGAACTAGTGG
CACACCAACAAATATATACCATGGTACAGATCCAGATGATGTTCAATTTTATACTATTGAAAATTCTG
TGCCAGTACACTTACTAAGAACAGGTGATGAATTTGCTACAGGAACATTTTTTTTTGATTGTAAACCA
TGTAGACTAACACATACATGGCAAACAAATAGAGCATTGGGCTTACCACCATTTCTAAATTCTTTGCC
TCAATCTGAAGGAGCTACTAACTTTGGTGATATAGGAGTTCAACAAGATAAAAGACGTGGTGTAACTC
AAATGGGAAATACAAACTATATTACTGAAGCTACTATTATGAGACCAGCTGAGGTTGGTTATAGTGCA
CCATATTATTCTTTTGAGGCGTCTACACAAGGGCCATTTAAAACACCTATTGCAGCAGGACGGGGGGG
AGCGCAAACAGATGAAAATCAAGCAGCAGATGGTGATCCAAGATATGCATTTGGTAGACAACATGGTC
AAAAAACTACCACAACAGGAGAAACACCTGAGAGATTTACATATATAGCACATCAAGATACAGGAAGA
TATCCAGAAGGAGATTGGATTCAAAATATTAACTTTAACCTTCCTGTAACAAATGATAATGTATTGCT
ACCAACAGATCCAATTGGAGGTAAAACAGGAATTAACTATACTAATATATTTAATACTTATGGTCCTT
TAACTGCATTAAATAATGTACCACCAGTTTATCCAAATGGTCAAATTTGGGATAAAGAATTTGATACT
GACTTAAAACCAAGACTTCATGTAAATGCACCATTTGTTTGTCAAAATAATTGTCCTGGTCAATTATT
TGTAAAAGTTGCGCCTAATTTAACAAATGAATATGATCCTGATGCATCTGCTAATATGTCAAGAATTG
TAACTTACTCAGATTTTTGGTGGAAAGGTAAATTAGTATTTAAAGCTAAACTAAGAGCCTCTCATACT
TGGAACCCAATTCAACAAATGAGTATTAATGTAGATAACCAATTTAACTATCTACCAAGTAATATTGG
AGGTATGAAAATTGTATATGAAAAATCTCAACTAGCACCTAGA
'CHI_GU392242_raccoondog_HB10_2009'
ATGAGTGATGGAGCAGTTCAACCAGACGGTGGTCAATCTGCTGTCAGAAATGAAAGAGCTACAGGATC
TGGGAACGGGTCTGGAGGCGGGGGTGGTGGTGGTTCTGGGGGTGTGGGGATTTCTACGGGTACTTTCA
ATAATCAGACGGAATTTAAATTTTTGGAAAACGGATGGGTGGAAATCACAGCAAACTCAAGCAGACTT
GTACATTTAAATATGCCAGAAAGTGAAAATTATAGAAGAGTGGTTGTGAATAATATGGATAAAACTGC
AGTTAACGGAAACATGGCTTTAGATGATATTCATGCACAAATTGTAACACCTTGGTCATTGGTTGATG
CAAATGCTTGGGGAGTTTGGTTTAATCCAGGAGATTGGCAACTAATTGTTAATACTATGAGTGAGTTG
CATTTAGTTAGTTTTGAACAAGAAATTTTTAATGTTGTTTTAAAGACTGTTTCAGAATCTGCTACTCA
GCCACCAACTAAAGTTTATAATAATGATTTAACTGCATCATTGATGGTTGCATTAGATAGTAATAATA
CTATGCCATTTACTCCAGCAGCTATGAGATCTGAGACATTGGGTTTTTATCCATGGAAACCAACCATA
CCAACTCCATGGAGATATTATTTTCAATGGGATAGAACATTAGTACCATCTCATACTGGAACTAGTGG
CACACCAACAAATATATACCATGGTACAGATCCAGATGATGTTCAATTTTATACTATTGAAAATTCTG
TGCCAGTACACTTACTAAGAACAGGTGATGAATTTGCTACAGGAACATTTTTTTTTGATTGTAAACCA
TGTAGACTAACACATACATGGCAAACAGATAGAGCATTGGGCTTACCACCATTTCTAAATTCTTTGCC
TCAATCTGAAGGAGCTACTAACTTTGGTGATATAGGAGTTCAACAAGGTAAAAGACGTGGTGTAACTC
AAATGGGAAATACAAACTATATTACTGAAGCTACTATTATGAGACCAGCTGAGGTTGGTTATAGTGCA
CCATATTATTCTTTTGAGGCGTCTACACAAGGGCCATTTAAAACACCTATTGCAGCAGGACGGGGGGG
AGCGCAAACAGATGAAAATCAAGCAGCAGATGGTGATCCAAGATATGCATTTGGTAGACAACATGGTC
AAAAAACTACCACAACAGGAGAAACACCTGAGAGATTTACATATATAGCACATCAAGATACAGGAAGA
TATCCAGAAGGAGATTGGATTCAAAATATTAACTTTAACCTTCCTGTAACAAATGATAATGTATTGCT
ACCAACAGATCCAATTGGAGGTAAAACAGGAATTAACTATACTAATATATTTAATACTTATGGTCCTT
TAACTGCATTAAATAATGTACCACCAGTTTATCCAAATGGTCAAATTTGGGATAAAGAATTTGATACT
GACTTAAAACCAAGACTTCATGTAAATGCACCATTTGTTTGTCAAAATAATTGTCCTGGTCAATTATT
TGTAAAAGTTGCGCCTAATTTAACAAATGAATATGATCCTGATGCATCTGCTAATATGTCAAGAATTG
TAACTTACTCAGATTTTTGGTGGAAAGGTAAATTAGTATTTAAAGCTAAACTAAGAGCCTCTCATACT

TGGAATCCAATTCAACAAATGAGTATTAATGTAGATAACCAATTTAACTATCTACCAAGTAATATTGG
AGGTATGAAAATTGTATATGAAAAATCTCAACTAGCACCTAGA

'CHI_GU392244_raccoondog_HB7_2009'

ATGAGTGATGGAGCAGTTCAACCAGACGGTGGTCAATCTGCTGTCAGAAATGAAAGAGCTACAGGATC
TGGGAACGGGTCTGGAGGCGGGGGTGGTGGTGGTTCTGGGGGTGTGGGGATTTCTACGGGTACTTTCA
ATAATCAGACGGAATTTAAATTTTTGGAAAACGGATGGGTGGAAATCACAGCAAACTCAAGCAGACTT
GTACATTTAAATATGCCAGAAAGTGAAAATTATAGAAGAGTGGTTGTGAATAATATGAATAAAACTGC
AGTTAACGGAAACATGGCTTTAGATGATATTCATGCACAAATTGTAACACCTTGGTCATTGGTTGATG
CAAATGCTTGGGGAGTTTGGTTTAATCCAGGAGATTGGCAACTAATTGTTAATACTATGAGTGAGTTG
CATTTAGTTAGTTTTGAACAAGAAATTTTTAATGTTGTTTTAAAGACTGTTTCAGAATCTGCTACTCA
GCCACCAACTAAAGTTTATAATAATGATTTAACTGCATCATTGATGGTTGCATTAGATAGTAATAATA
CTATGCCATTTACTCCAGCAGCTATGAGATCTGAGACATTGGGTTTTTATCCATGGAAACCAACCATA
CCAACTCCATGGAGATATTATTTTCAATGGGATAGAACATTAGTACCATCTCATACTGGAACTAGTGG
CACACCAACAAATATATACCATGGTACAGATCCAGATGATGTTCAATTTTATACTATTGAAAATTCTG
TGCCAGTACACTTACTAAGAACAGGTGATGAATTTGCTACAGGAACGTTTTTTTTTGATTGTAAACCA
TGTAGACTAACACATACATGGCAAACAAATAGAGCATTGGGCTTACCACCATTTCTAAATTCTTTGCC
TCAATCTGAAGGAGCTACTAACTTTGGTGATATAGGAGTTCAACAAGATAAAAGACGTGGTGTAACTC
AAATGGGAAATACAAACTATATTACTGAAGCTACTATTATGAGACCAGCTGAGGTTGGTTATAGTGCA
CCATATTATTCTTTTGAGGCGTCTACACAAGGGCCATTTAAAACACCTATTGCAGCAGGACGGGGGGG
AGCGCAAACAGATGAAAATCAAGCAGCAGATGGTGATCCAAGATATGCATTTGGTAGACAACATGGTC
AAAAAACTACCACAACAGGAGAAACACCTGAGAGATTTACATATATAGCACATCAAGATACAGGAAGA
TATCCAGAAGGAGATTGGATTCAAAATATTAACTTTAACCTTCCTGTAACAAATGATAATGTATTGCT
ACCAACAGATCCAATTGGAGGTAAAACAGGAATTAACTATACTAATATATTTAATACTTATGGTCCTT
TAACTGCATTAAATAATGTACCACCAGTTTATCCAAATGGTCAAATTTGGGATAAAGAATTTGATACT
GACTTAAAACCAAGACTTCATGTAAATGCACCATTTGTTTGTCAAAATAATTGTCCTGGTCAATTATT
TGTAAAAGTTGCGCCTAATTTAACAAATGAATATGATCCTGATGCATCTGCTAATATGTCAAGAATTG
TAACTTACTCAGATTTTTGGTGGAAAGGTAAATTAGTATTTAAAGCTAAACTAAGAGCCTCTCATACT
TGGAATCCAATTCAACAAATGAGTATTAATGTAGATAACCAATTTAACTATCTACCAAGTAATATTGG
AGGTATGAAAATTGTATATGAAAAATCTCAACTAGCACCTAGA

'CHI_GU569939_2a_YN0202_2002'

ATGAGTGATGGAGCAGTTCAACCAGACGGTGGTCAACCTGCTGTCAGAAATGAAAGAGCTACAGGATC
TGGGAACGGGTCTGGAGGCGGGGGTGGTGGTGGTTCTGGGGGTGTGGGGATTTCTACGGGTACTTTCA
ATAATCAGACGGAATTTAAATTTTTGGAAAACGGATGGGTGGAAATCACAGCAAACTCAAGCAGACTT
GTACATTTAAATATGCCAGAAAGTGAAAATTATAGAAGAGTGGTTGTAAATAATTTGGATAAAACTGC
AGTTAACGGAAACATGGCTTTAGATGATACTCATGCACAAATTGTAACACCTTGGTCATTGGTTGATG
CAAATGCTTGGGGAGTTTGGTTTAATCCAGGAGATTGGCAACTAATTGTTAATACTATGAGTGAGTTG
CATTTAGTTAGTTTTGAACAAGAAATTTTTAATGTTGTTTTAAAGACTGTTTCAGAATCTGCTACTCA
GCCACCAACTAAAGTTTATAATAATGATTTAACTGCATCATTGATGGTTGCATTAGATAGCAATAATA
CTATGCCATTTACTCCAGCAGCTATGAGATCTGAGACATTGGGTTTTTATCCATGGAAACCAACCATA
CCAACTCCATGGAGATATTATTTTCAATGGGATAGAACATTAATACCATCTCATACTGGAACTAGTGG
CACACCAACAAATATATACCATGGTACAGATCCAGATGATGTTCAATTTTATACTATTGAAAATTCTG
TACCAGTACACTTACTAAGAACAGGTGATGAATTTGCTACAGGAACATTTTTTTTTGATTGTAAACCA
TGTAGACTAACACATACATGGCAAACAAATAGAGCATTGGGCTTACCACCATTTCTAAATTCTTTGCC
TCAAGCTGAAGGAGGTACTAACTTTGGTTATATAGGAGTTCAACAAGATAAAAGACGTGGTGTAACTC
AAATGGGAAATACAAACTATATTACTGAAGCTACTATTATGAGACCAGCTGAGGTTGGTTATAGTGCA
CCATATTATTCTTTTGAGGCGTCTACACAAGGGCCATTTAAAACACCTATTGCAGCAGGACGGGGGGG
AGCGCAAACAGATGAAAATCAAGCAGCAGATGGTAATCCAAGATATGCATTTGGTAGACAACATGGTC
AAAAAACTACCACAACAGGAGAAACACCTGAGAGATTTACATATATAGCACATCAAGATACAGGAAGA
TATCCAGAAGGAGATTGGATTCAAAATATTAACTTTAACCTTCCTGTAACAAATGATAATGTATTGCT
ACCAACAGATCCAATTGGAGGTAAAACAGGAATTAACTATACTAATATATTTAATACTTATGGTCCTT
TAACTGCATTAAATAATGTACCACCAGTTTATCCAAATGGTCAAATTTGGGATAAAGAATTTGATACT
GACTTAAAACCAAGACTTCATGTAAATGCACCATTTGTTTGTCAAAATAATTGTCCTGGTCAATTATT
TGTGAAAGTTGCGCCTAATTTAACAAATGAATATGATCCTGATGCATCTGCTAATATGTCAAGAATTG
TAACTTACTCAGATTTTTGGTGGAAAGGTAAATTAGTATTTAAAGCTAAACTAAGAGCCTCTCATACT

TGGAATCCAATTCAACAAATGAGTATTAATGTAGATAACCAATTTAACTATGTACCAAGTAATATTGG
AGGTATGAAGATTGTATATGAAAAATCTCAACTAGCACCTAGA
'CHI_GU569940_2b_YN0203_2002'
ATGAGTGATGGAGCAGTTCAACCAGACGGTGGTCAACCTGCTGTCAGAAATGAAAGAGCTACAGGATC
TGGGAACGGGTCTGGAGGCGGGGGTGGTGGTGGTTCTGGGGGTGTGGGGATTTCTACGGGTACTTTCA
ATAATCAGACGGAATTTAAATTTTTGGAAAACGGATGGGTGGAAATCACAGCAAACTCAAGCAGACTT
GTACATTTAAATATGCCAGAAAGTGAAAATTATAGAAGAGTGGTTGTAAATAATTTGGATAAAACTGC
AGTTAACGGAAACATGGCTTTAGATGATACTCATGCACAAATTGTAACACCTTGGTCATTGGTTGATG
CAAATGCTTGGGGAGTTTGGTTTAATCCAGGAGATTGGCAACTAATTGTTAATACTATGAGTGAGTTG
CATTTAGTTAGTTTTGAACAAGAAATTTTTAATGTTGTTTTGAAGACTGTTTCAGAATCTGCTACTCA
GCCACCAACTAAAGTTTATAATAATGATTTAACTGCATCATTGATGGTTGCATTAGATAGTAATAATA
CTATGCCATTTACTCCAGCAGCTATGAGATCTGAGACATTGGGTTTTTATCCATGGAAACCAACCATA
CCAACTCCATGGAGATATTATTTTCAATGGGATAGAACATTAATACCATCTCATACTGGAACTAGTGG
CACACCAACAAATATATACCATGGTACAGATCCAGATGATGTTCAATTTTATACTATTGAAAATTCTG
TGCCAGTACACTTACTAAGAACAGGTGATGAATTTGCTACAGGAACATTTTTTTTTGATTGTAAACCA
TGTAGACTAACACATACATGGCAAACAAATAGAGCATTGGGCTTACCACCATTTCTAAATTCTTTGCC
TCAAGCTGAAGGAGGTACTAACTTTGGTTATATAGGAGTTCAACAAGATAAAAGACGTGGTGTAACTC
AAATGGGAAATACAAACTATATTACTGAAGCTACTATTATGAGACCAGCTGAGGTTGGTTATAGTGCA
CCATATTATTCTTTTGAGGCGTCTACACAAGGGCCATTTAAAACACCTATTGCAGCAGGACGGGGGGG
AGCGCAAACAGATGAAAATCAAGCAGCAGATGGTAATCCAAGATATGCATTTGGTAGACAACATGGTC
AAAAAACTACCACAACAGGAGAAACACCTGAGAGATTTACATATATAGCACATCAAGATACAGGAAGA
TATCCAGAAGGAGATTGGATTCAAAATATTAACTTTAACCTTCCTGTAACAGATGATAATGTTTTGCT
ACCAACAGATCCAATTGGAGGTAAAACAGGAATTAACTATACTAATATATTTAATACTTATGGTCCTT
TAACTGCATTAAATAATGTACCACCAGTTTATCCAAATGGTCAAATTTGGGATAAAGAATTTGATACT
GACTTAAAACCAAGACTTCATGTAAATGCACCATTTGTTTGTCAAAATAATTGTCCTGGTCAATTATT
TGTAAAAGTTGCGCCTAATTTAACAAATGAATATGATCCTGATGCATCTGCTAATATGTCAAGAATTG
TAACTTACTCAGATTTTTGGTGGAAAGGTAAATTAGTATTTAAAGCTAAACTAAGAGCCTCTCATACT
TGGAATCCAATTCAACAAATGAGTATCAATGTAGATAACCAATTTAACTATGTACCAAGTAATATTGG
AGGTATGAAAATTGTATATGAAAAATCTCAACTAGCACCTAGA
'CHI_GU569942_2a_JL0202_2002'
ATGAGTGATGGAGCAGTTCAACCAGACGGTGGTCAACCTGCTGTCAGAAATGAAAGAGCTACAGGATC
TGGGAACGGGTCTGGAGGCGGGGGTGGTGGTGGTTCTGGGGGTGTGGGGATTTCTACGGGTACTTTCA
ATAATCAGACGGAATTTAAATTTTTGGAAAACGGATGGGTGGAAATCACAGCAAACTCAAGCAGACTT
GTACATTTAAATATGCCAGAAAGTGAAAATTATAGAAGAGTGGTTGTAAATAATTTGGATAAAACTGC
AGTTAACGGAAACATGGCTTTAGATGATACCCATGCACAAATTGTAACACCTTGGTCATTGGTTGATG
CAAATGCTTGGGGAGTTTGGTTTAATCCAGGAGATTGGCAACTAATTGTTAATACTATGAGTGAATTG
CATTTAGTTAGTTTTGAACAAGAAATTTTTAATGTTGTTTTAAAGACTGTTTCAGAATCTGCTACTCA
GCCACCAACTAAAGTTTATAATAATGATTTAACTGCATCATTGATGGTTGCATTAGATAGTAATAATA
CTATGCCATTTACTCCAGCAGCTATGAGATCTGAGACATTGGGTTTTTATCCATGGAAACCAACCATA
CCAACTCCATGGAGATATTATTTTCAATGGGATAGAACATTAATACCATCTCATACTGGAACTAGTGG
CACACCAACAAATATATACCATGGTACAGATCCAGATGATGTTCAATTTTATACTATTGAAAATTCTG
TGCCAGTACACTTACTAAGAACAGGTGATGAATTTGCTACAGGAACATTTTTTTTTGATTGTAAACCA
TGTAGACTAACACATACATGGCAAACAAATAGAGCATTGGGCTTACCACCATTTCTAAATTCTTTGCC
TCAAGCTGAAGGAGGTACTAACTTTGGTTATATAGGAGTTCAACAAGATAAAAGACGTGGTGTAACTC
AAATGGGAAATACAAACTATATTACTGAAGCTACTATTATGAGACCAGCTGAGGTTGGTTATAGTGCA
CCATATTATTCTTTTGAGGCGTCTACACAAGGGCCATTTAAAACACCTATTGCAGCAGGACGGGGGGG
AGCGCAAACAGATGAAAATCAAGCAGCAGATGGTAATCCAAGATATGCATTTGGTAGACAACATGGTC
AAAAAACTACCACAACAGGAGAAACACCTGAGAGATTTACATATATAGCACATCAAGATACAGGAAGA
TATCCAGAAGGAGATTGGATTCAAAATATTAACTTTAACCTTCCTGTAACAAATGATAATGTATTGCT
ACCAACAGATCCAATTGGAGGTAAAACAGGAATTAACTATACTAATATATTTAATACTTATGGTCCTT
TAACTGCATTAAATAATGTACCACCAGTTTATCCAAATGGTCAAATTTGGGATAAAGAATTTGATACT
GACTTAAAACCAAGACTTCATGTAAATGCACCATTTGTTTGTCAAAATAATTGTCCTGGTCAATTATT
TGTAAAAGTTGCGCCTAATTTAACAAATGAATATGATCCTGATGCATCTGCTAATATGTCAAGAATTG
TAACTTACTCAGATTTTTGGTGGAAAGGTAAATTAGTATTTAAAGCTAAACTAAGAGCCTCTCATACT

TGGAATCCAATTCAACAAATGAGTATTAATGTAGATAACCAATTTAACTATGTACCAAGTAATATTGG
AGGTATGAAAATTGTATATGAAAAATCTCAACTAGCACCTAGA
'CHI_GU569943_YB8301_1983'
ATGAGTGATGGAGCAGTTCAACCAGACGGTGGTCAACCTGCTGTCAGAAATGAAAGAGCTACAGGATC
TGGGAACGGGTCTGGAGGCGGGGGTGGTGGTGGTTCTGGGGGTGTGGGGATTTCTACGGGTACTTTCA
ATAATCAGACGGAATTTAAATTTTTGGAAAACGGATGGGTGGAAATCACAGCAAACTCAAGCAGACTT
GTACATTTAAATATGCCAGAAAGTGAAAATTATAGAAGAGTGGTTGTAAATAATATGGATAAAACTGC
AGTTAACGGAAACATGGCTTTAGATGATATTCATGCACAAATTGTAACACCTTGGTCATTGGTTGATG
CAAATGCTTGGGGAGTTTGGTTTAATCCAGGAGATTGGCAACTAATTGTTAATACTATGAGTGAGTTG
CATTTAGTTAGTTTTGAACAAGAAATTTTTAATGTTGTTTTAAAGACTGTTTCAGAATCTGCTACTCA
GCCACCAACTAAAGTTTATAATAATGATTTAACTGCATCATTGATGGTTGCATTAGATAGTAATAATA
CTATGCCATTTACTCCAGCAGCTATGAGATCTGAGACATTGGGTTTTTATCCATGGAAACCAACCATA
CCAACTCCATGGAGATATTATTTTCAATGGGATAGAACATTAATACCATCTCATACTGGAACTAGTGG
CACACCAACAAATATATACCATGGTACAGATCCAGATGATGTTCAATTTTATACTATTGAAAATTCTG
TGCCAGTGCACTTACTAAGAACAGGTGATGAATTTGCTACAGGAACATTTTTTTTTGATTGTAGACCA
TGTAGACTAACACATACATGGCAAACAAATAGAGCATTGGGCTTACCACCATTTCTAAATTCTTTGCC
TCAATCTGAAGGAGATATTAACTTTGGTGATATAGGAGTTCAACAAGATAAAAGACGTGGTATAACTC
AAATGGGAAATACAAACTATATTACTGAAGCTACTATTATGAGACCAGCTGAGGTTGGTTATAGTGCA
CCATATTATTCTTTTGAGGCGTCTACACAAGGGCCATTTAAAACACCTATTGCAGCAGGACGGGGGGG
AGCGCAAACAGATGAAAATCAAGCAGCAGATGGTAATCCAAGATATGCATTTGGTAGACAACATGGTC
AAAAAACTACCACAACAGGAGAAACACCTGAGAGATTTACATATATAGCACATCAAGATACAGGAAGA
TATCCAGAAGGAGATTGGATTCAAAATATTAACTTTAACCTTCCTGTAACAAATGATAATGTATTGCT
ACCAACAGATCCAATTGGAGGTAAAACAGGAATTAACTATACTAATATATTTAATACTTATGGTCCTT
TAACTGCATTAAATAATGTACCACCAGTTTATCCAAATGGTCAAATTTGGGATAAAGAATTTGATACT
GACTTAAAACCAAGACTTCATGTAAATGCACCATTTGTTTGTCAAAATAATTGTCCTGGTCAATTATT
TGTAAAAGTTGCGCCTAATTTAACGAATGAATATGATCCTGATGCATCTGCTAATATGTCAAGAATTG
TAACTTACTCAGATTTTTGGTGGAAAGGTAAATTAGTATTTAAAGCTAAACTAAGAGCCTCTCATACT
TGGAATCCAATTCAACAAATGAGTATTAATGTAGATAACCAATTTAACTATGTACCAAGTAATATTGG
AGGTATGAAAATTGTATATGAAAAATCTCAACTAGCACCTAGA
'CHI_GU569946_2a_JL0201_2002'
ATGAGTGATGGAGCAGTTCAACCAGACGGTGGTCAACCTGCTGTCAGAAATGAAAGAGCTACAGGATC
TGGGAACGGGTCTGGAGGCGGGGGTGGTGGTGGTTCTGGGGGTGTGGGGATTTCTACGGGTACTTTCA
ATAATCAGACGGAATTTAAATTTTTGGAAAACGGATGGGTGGAAATCACAGCAAACTCAAGCAGACTT
GTACATTTAAATATGCCAGAAAGTGAAAATTATAGAAGAGTGGTTGTAAATAATTTGGATAAAACTGC
AGTTAACGGAAACATGGCTTTAGATGATACTCATGCACAAATTGTAACACCTTGGTCATTGGTTGATG
CAAATGCTTGGGGAGTTTGGTTTAATCCAGGAGATTGGCAACTAATTGTTAATACTATGAGTGAATTG
CATTTAGTTAGTTTTGAACAAGAAATTTTTAATGTTGTTTTAAAGACTGTTTCAGAATCTGCTACTCA
GCCACCAACTAAAGTTTATAATAATGATTTAACTGCATCATTGATGGTTGCATTAGATAGTAATAATA
CTATGCCATTTACTCCAGCAGCTATGAGATCTGAGACATTGGGTTTTTATCCATGGAAACCAACCATA
CCAACTCCATGGAGATATTATTTTCAATGGGATAGAACATTAATACCATCTCATACTGGAACTAGTGG
CACACCAACAAATATATACCATGGTACAGATCCAGATGATGTTCAATTTTATACTATTGAAAATTCTG
TGCCAGTACACTTACTAAGAACAGGTGATGAATTTGCTACAGGAACATTTTTTTTTGATTGTAAACCA
TGTAGACTAACACATACATGGCAAACAAATAGAGCATTGGGCTTACCACCATTTCTAAATTCTTTGCC
TCAAGCTGAAGGAGGTACTAACTTTGGTTATATAGGAGTTCAACAAGATAAAAGACGTGGTGTAACTC
AAATGGGAAATACAAACTATATTACTGAAGCTACTATTATGAGACCAGCTGAGGTTGGTTATAGTGCA
CCATATTATTCTTTTGAGGCGTCTACACAAGGGCCATTTAAAACACCTATTGCAGCAGGACGGGGGGG
AGCGCAAACAGATGAAAATCAAGCAGCAGATGGTAATCCAAGATATGCATTTGGTAGACAACATGGTC
AAAAAACTACCACAACAGGAGAAACACCTGAGAGATTTACATATATAGCACATCAAGATACAGGAAGA
TATCCAGAAGGAGATTGGATTCAAAATATTAACTTTAACCTTCCTGTAACAAATGATAATGTATTGCT
ACCAACAGATCCAATTGGAGGTAAAACAGGAATTAACTATACTAATATATTTAATACTTATGGTCCTT
TAACTGCATTAAATAATGTACCACCAGTTTATCCAAATGGTCAAATTTGGGATAAAGAATTTGATACT
GACTTAAAACCAAGACTTCATGTAAATGCACCATTTGTTTGTCAAAATAATTGTCCTGGTCAATTATT
TGTAAAAGTTGCGCCTAATTTAACAAATGAATATGATCCTGATGCATCTGCTAATATGTCAAGAATTG
TGACTTACTCAGATTTTTGGTGGAAAGGTAAATTAGTATTTAAAGCTAAACTAAGAGCCTCTCATACT

TGGAATCCAATTCAACAAATGAGTATTAATGTAGATAACCAATTTAACTATGTACCAAATAATATTGG
AGGTATGAAAATTGTATATGAAAAATCTCAACTAGCACCTAGA
'CHI_GU569948_2a_CC8601_1986'
ATGAGTGATGGAGCAGTTCAACCAGACGGTGGTCAACCTGCTGTCAGAAATGAAAGAGCTACAGGATC
TGGGAACGGGTCTGGAGGCGGGGGTGGTGGTGGTTCTGGGGGTGTGGGGATTTCTACGGGTACTTTCA
ATAATCAGACGGAATTTAAATTTTTGGAAAACGGATGGGTGGAAATCACAGCAAACTCAAGCAGACTT
GTACATTTAAATATGCCAGAAAGTGAAAATTATAGAAGAGTGGTTGTAAATAATTTGGATAAAACTGC
AGTTAACGGAAACATGGCTTTAGATGATACCCATGCACAAATTGTAACACCTTGGTCATTGGTTGATG
CAAATGCTTGGGGAGTTTGGTTTAATCCAGGAGATTGGCAACTAATTGTTAATACTATGAGTGAGTTG
CATTTAGTTAGTTTTGAACAAGAAATTTTTAATGTTGTTTTAAAGACTGTTTCAGAATCTGCTACTCA
GCCACCAACTAAAGTTTATAATAATGATTTAACTGCATCATTGATGGTTGCATTAGATAGTAATAATA
CTATGCCATTTACTCCAGCAGCTATGAGATCTGAGACATTGGGTTTTTATCCATGGAAACCAACCATA
CCAACTCCATGGAGATATTATTTTCAATGGGATAGAACATTAATACCATCTCATACTGGAACTAGTGG
CACACCAACAAATATATACCATGGTACAGATCCAGATGATGTTCAATTTTATACTATTGAAAATTCTG
TGCCAGTACACTTACTAAGAACAGGTGATGAATTTGCTACAGGAACATTTTTTTTTGATTGTAAACCA
TGTAGACTAACACATACATGGCAAACAAATAGAGCATTGGGCTTACCACCATTTCTAAATTCTTTGCC
TCAATCTGAAGGAGGTACTAACTTTGGTTATATAGGAGTTCAACAAGATAAAAGACGTGGTGTAACTC
AAATGGGAAATACAAACTATATTACTGAAGCTACTATTATGAGACCAGCTGAGGTTGGTTATAGTGCA
CCATATTATTCTTTTGAGGCGTCTACACAAGGGCCATTTAAAACACCTATTGCAGCAGGACGGGGGGG
AGCGCAAACAGATGAAAATCAAGCAGCAGATGGTAATCCAAGATATGCATTTGGTAGACAACATGGTC
AAAAAACTACCACAACAGGAGAAACACCTGAGAGATTTACATATATAGCACATCAAGATACAGGAAGA
TATCCAGAAGGAGATTGGATTCAAAATATTAACTTTAACCTTCCTGTAACAAATGATAATGTATTGCT
ACCAACAGATCCAATTGGAGGTAAAACAGGAATTAACTATACTAATATATTTAATACTTATGGTCCTT
TAACTGCATTAAATAATGTACCACCAGTTTATCCAAATGGTCAAATTTGGGATAAAGAATTTGATACT
GACTTAAAACCAAGACTTCATGTAAATGCACCATTTGTTTGTCAAAATAATTGTCCTGGTCAATTATT
TGTAAAAGTTGCGCCTAATTTAACAAATGAATATGATCCTGATGCATCTGCTAATATGTCAAGAATTG
TAACTTACTCAGATTTTTGGTGGAAAGGTAAATTAGTATTTAAAGCTAAACTAAGAGCCTCTCATACT
TGGAATCCAATTCAACAAATGAGTATTAATGTAGATAACCAATTTAACTATGTACCAAGTAATATTGG
AGGTATGAAAATTGTATATGAAAAATCTCAACTAGCACCTAGA
'SAF_HQ602969_22_10SA_2010'
ATGAGTGATGGAGCAGTTCAACCAGACGGTGGTCAGCCTGCTGTCAGAAATGAAAGAGCTACAGGATC
TGGGAACGGGTCTGGAGGCGGGGGTGGTGGTGGTTCTGGGGGTGTGGGGATTTCTACGGGTACTTTCA
ATAATCAGACGGAATTTAAATTTTTGGAAAACGGATGGGTGGAAATCACAGCAAACTCAAGCAGACTT
GTACATTTAAATATGCCAGAAAGTGAAAATTATAGAAGAGTGGTTGTAAATAATTTGGATAAAACTGC
AGTTAACGGAAACATGGCTTTAGATGATACTCATGCACAAATTGTAACACCTTGGTCATTGGTTGATG
CAAATGCTTGGGGAGTTTGGTTTAATCCAGGAGATTGGCAACTAATTGTTAATACTATGAGTGAGTTG
CATTTAGTTAGTTTTGAACAAGAAATTTTTAATGTTGTTTTAAAGACTGTTTCAGAATCTGCTACTCA
GCCACCAACTAAAGTTTATAATAATGATTTAACTGCATCATTGATGGTTGCATTAGATAGTAATAATA
CTATGCCATTTACTCCAGCAGCTATGAGATCTGAGACATTGGGTTTTTATCCATGGAAACCAACCATA
CCAACTCCATGGAGATATTATTTTCAATGGGATAGAACATTAATACCATCTCATACTGGAACTAGTGG
CACACCAACAAATATATACCATGGTACAGATCCAGATGATGTTCAATTTTATACTATTGAAAATTCTG
TGCCAGTACACTTACTAAGAACAGGTGATGAATTTGCTACAGGAACATTTTTTTTTGATTGTAAACCA
TGTAGACTAACACATACATGGCAAACAAATAGAGCATTGGGCTTACCACCATTTCTAAATTCTTTGCC
TCAAGCTGAAGGAGGTACTAACTTTGGTTATATAGGAGTTCAACAAGATAAAAGACGTGGTGTAACTC
AAATGGGAAATACAAACTATATTACTGAAGCTACTATTATGAGACCAGCTGAGGTTGGTTATAGTGCA
CCATATTATTCTTTTGAGGCGTCTACACAAGGGCCATTTAAAACACCTATTGCAGCAGGACGGGGGGG
AGCGCAAACAGATGAAAATCAAGCAGCAGATGGTGATCCAAGATATGCATTTGGTAGACAACATGGTC
AAAAAACTACCACAACAGGAGAAACACCTGAGAGATTTACATATATAGCACATCAAGATACAGGAAGA
TATCCAGAAGGAGATTGGATTCAAAATATTAACTTTAACCTTCCTGTAACAGATGATAATGTATTGCT
ACCAACAGATCCAATTGGAGGTAAAACAGGAATTAACTATACTAATATATTTAATACTTATGGTCCTT
TAACTGCATTAAATAATGTACCACCAGTTTATCCAAATGGTCAAATTTGGGATAAAGAATTTGATACT
GACTTAAAACCAAGACTTCATGTAAATGCACCATTTGTTTGTCAAAATAATTGYCCTGGTCAATTATT
TGTAAAAGTTGCGCCTAATTTAACAAATGAATATGATCCTGATGCATCTGCTAATATGTCAAGAATTG
TAACTTACTCAGATTTTTGGTGGAAAGGTAAATTAGTATTTAAAGCTAAACTAAGAGCCTCTCATACT

TGGAATCCAATTCAACAAATGAGTATTAATGTAGATAACCAATTTAACTATGTACCAAGTAATATTGG
AGGTATGAAAATTGTMTATGAAAAATCTCAACTAGCACCTAGA

'NIG_HQ602992_19_10_2010'

ATGAGTGATGGAGCAGTTCAACCAGACGGTGGTCAACCTGCTGTCAGAAATGAAAGAGCTACAGGATC
TGGGAACGGGTCTGGAGGCGGGGGTGGTGGTGGTTCTGGGGGTGTGGGGATTTCTACGGGTACTTTCA
ATAATCAGACGGAATTTAAATTTTTGGAAAACGGATGGGTGGAAATCACAGCAAACTCAAGCAGACTT
GTACATTTAAATATGCCAGAAAGTGAAAATTATAGAAGAGTGGTTGTAAATAATTTGGATAAAACTGC
AGTTAACGGAAACATGGCTTTAGATGATACTCATGCACAAATTGTAACACCTTGGTCATTGGTTGATG
CAAATGCTTGGGGAGTTTGGTTTAATCCAGGAGATTGGCAACTAATTGTTAATACTATGAGTGAGTTG
CATTTAGTTAGTTTTGAACAAGAAATTTTTAATGTTGTTTTAAAGACTGTTTCAGAATCTGCTACTCA
GCCACCAACTAAAGTTTATAATAATGATTTAACTGCATCATTGATGGTTGCATTAGATAGCAATAATA
CTATGCCATTTACTCCAGCAGCTATGAGATCTGAGACATTGGGTTTTTATCCATGGAAACCAACCATA
CCAACTCCATGGAGATATTATTTTCAATGGGATAGAACATTAATACCATCTCATACTGGAACTAGTGG
CACACCAACAAATATATACCATGGTACAGATCCAGATGATGTTCAATTTTATACTATTGAAAATTCTG
TGCCAGTACACTTACTAAGAACAGGTGATGAATTTGCTACAGGAACATTTTTTTTTGATTGTAAACCA
TGTAGACTAACACATACATGGCAAACAAATAGAGCATTAGGCTTACCACCATTTCTAAATTCTTTGCC
TCAAGCTGAAGGAGGTACTAACTTTGGTTATATAGGAGTTCAACAAGATAAAAGACGTGGTGTAACTC
AAATGGGAAATACAAACTATATTACTGAAGCTACTATTATGAGACCAGCTGAGGTTGGTTATAGTGCA
CCATATTATTCTTTTGAGGCGTCTACACAAGGGCCATTTAAAACACCTATTGCAGCAGGACGGGGGGG
AGCGCAAACAGATGAAAATCAAGCAGCAGATGGTGATCCAAGATATGCATTTGGTAGACAACATGGTC
AAAAAACTACCACAACAGGAGAAACACCTGAGAGATTTACATATATAGCACATCAAGATACAGGAAGA
TATCCAGAAGGAGATTGGATTCAAAATATTAACTTTAACCTTCCTGTAACAAATGATAATGTATTGCT
ACCAACAGATCCAATTGGAGGTAAAACAGGAATTAACTATACTAATATATTTAATACTTATGGTCCTT
TAACTGCATTAAATAATGTACCACCAGTTTATCCAAATGGTCAAATTTGGGATAAAGAATTTGATACT
GACTTAAAACCAAGACTTCATGTAAATGCACCATTTGTTTGTCAAAATAATTGTCCTGGTCAATTATT
TGTAAAAGTTGCGCCTAATTTAACAAATGAATATGATCCTGATGCATCTGCTAATATGTCAAGAATTG
TAACTTACTCAGATTTTTGGTGGAAAGGTAAATTAGTATTTAAAGCTAAACTAAGAGCCTCTCATACT
TGGAATCCAATTCAACAAATGAGTATTAATGTAGATAACCAATTTAACTATGTACCAAGTAATATTGG
AGGTATGAAGATTGTATATGAAAAATCTCAACTAGCACCTAGA

'NIG_HQ602995_15_10_2010'

ATGAGTGATGGAGCAGTTCAACCAGACGGTGGTCAGCCTGCTGTCAGAAATGAAAGAGCTACAGGATC
TGGGAACGGGTCTGGAGGCGGGGGTGGTGGTGGTTCTGGGGGTGTGGGGATTTCTACGGGTACTTTCA
ATAATCAGACGGAATTTAAATTTTTGGAAAACGGATGGGTGGAAATCACAGCAAACTCAAGCAGACTT
GTACATTTAAATATGCCAGAAAGTGAAAATTATAGAAGAGTGGTTGTAAATAATTTGGATAAAACTGC
AGTTAACGGAAACATGGCTTTAGATGATACTCATGCACAAATTGTAACACCTTGGTCATTGGTTGATG
CAAATGCTTGGGGAGTTTGGTTTAATCCAGGAGATTGGCAACTAATTGTTAATACTATGAGTGAGTTG
CATTTAGTTAGTTTTGAACAAGAAATTTTTAATGTTGTTTTAAAGACTGTTTCAGAATCTGCTACTCA
GCCACCAACTAAAGTTTATAATAATGATTTAACTGCATCATTGATGGTTGCATTAGATAGCAATAATA
CTATGCCATTTACTCCAGCAGCTATGAGATCTGAGACATTGGGTTTTTATCCATGGAAACCAACCATA
CCAACTCCATGGAGATATTATTTTCAATGGGATAGAACATTAATACCATCTCATACTGGAACTAGTGG
CACACCAACAAATATATACCATGGTACAGATCCAGATGATGTTCAATTTTATACTATTGAAAATTCTG
TGCCAGTACACTTACTAAGAACAGGTGATGAATTTGCTACAGGAACATTTTTTTTTGATTGTAAACCA
TGTAGACTAACACATACATGGCAAACAAATAGAGCATTGGGCTTACCACCATTTCTAAATTCTTTGCC
TCAAGCTGAAGGAGGTACTAACTTTGGTTATATAGGAGTTCAACAAGATAAAAGACGTGGTGTAACTC
AAATGGGAAATACAAACTATATTACTGAAGCTACTATTATGAGACCAGCTGAGGTTGGTTATAGTGCA
CCATATTATTCTTTTGAGGCGTCTACACAAGGGCCATTTAAAACACCTATTGCAGCAGGACGGGGGGG
AGCGCAAACAGATGAAAATCAAGCAGCAGATGGTGATCCAAGATATGCATTTGGTAGACAACATGGTC
AAAAAACTACCACAACAGGAGAAACACCTGAGAGATTTACATATATAGCACATCAAGATACAGGAAGA
TATCCAGAAGGAGATTGGATTCAAAATATTAACTTTAACCTTCCTGTAACAAATGATAATGTATTGCT
ACCAACAGATCCAATTGGAGGTAAAACAGGAATTAACTATACTAATATATTTAATACTTATGGTCCTT
TAACTGCATTAAATAATGTACCACCAGTTTATCCAAATGGTCAAATTTGGGATAAAGAATTTGATACT
GACTTAAAACCAAGACTTCATGTAAATGCACCATTTGTTTGTCAAAATAATTGTCCTGGTCAATTATT
TGTAAAAGTTGCGCCTAATTTAACAAATGAATATGATCCTGATGCATCTGCTAATATGTCAAGAATTG
TAACTTACTCAGATTTTTGGTGGAAAGGTAAATTAGTATTTAAAGCTAAACTAAGAGCCTCTCATACT

TGGAATCCAATTCAACAAATGAGTATTAATGTAGATAACCAATTTAACTATGTACCAAGTAATATTGG
AGGTATGAAAATTGTATATGAAAAATCTCAACTAGCACCTAGA
'ARG_JF414818_Arg32_2008'
ATGAGTGATGGAGCAGTTCAACCAGACGGTGGTCAACCTGCTGTCAGAAATGAAAGAGCAACAGGATC
TGGGAACGGGTCTGGAGGCGGGGGTGGTGGTGGTTCTGGGGGTGTGGGGATTTCTACGGGTACTTTCA
ATAATCAGACGGAATTTAAATTTTTGGAAAACGGATGGGTGGAAATCACAGCAAACTCAAGCAGACTT
GTACATTTAAATATGCCAGAAAGTGAAAATTATAGAAGAGTGGTTGTAAATAATTTGGATAAAACTGC
AGTTAACGGAAACATGGCTTTAGATGATACTCATGCACAAATTGTAACACCTTGGTCATTGGTTGATG
CAAATGCTTGGGGAGTTTGGTTTAATCCAGGAGATTGGCAACTAATTGTTAATACTATGAGTGAGTTG
CATTTAGTTAGTTTTGAACAAGAAATTTTTAATGTTGTTTTAAAGACTGTTTCAGAATCTGCTACTCA
GCCACCAACTAAAGTTTATAATAATGATTTAACTGCATCATTGATGGTTGCATTAGATAGTAATAATA
CTATGCCATTTACTCCAGCAGCTATGAGATCTGAGACATTGGGTTTTTATCCATGGAAACCAACCATA
CCAACTCCATGGAGATATTATTTTCAATGGGATAGAACATTAATACCATCTCATACTGGAACCAGTGG
CACACCAACAAATATATACCATGGTACAGATCCAGATGATGTTCAATTTTATACTATTGAAAATTCTG
TGCCAGTACACTTACTAAGAACAGGTGATGAATTTGCTACAGGAACATTTTTTTTTGATTGTAAACCA
TGTAGACTAACACATACATGGCAAACAAATAGAGCATTGGGCTTACCACCATTTCTAAATTCTTTGCC
TCAAGCTGAAGGAGGTACTAACTTTGGTTATATAGGAGTTCAACAAGATAAAAGACGTGGTGTAACTC
AAATGGGAAATACAAACTATATTACTGAAGCTACTATTATGAGACCAGCTGAGGTTGGTTATAGTGCA
CCATATTATTCTTTTGAGGCGTCTACACAAGGGCCATTTAAAACACCTATTGCAGCAGGACGGGGGGG
AGCGCAAACAGATGAAAATCAAGCAGCAGATGGTGATCCAAGATATGCATTTGGTAGACAACATGGTC
AAAAAACTACCACAACAGGAGAAACACCTGAGAGATTTACATATATAGCACATCAAGATACAGGAAGA
TATCCAGAAGGAGATTGGATTCAAAATATTAACTTTAACCTTCCTGTAACAGAAGATAATGTATTGCT
ACCAACAGATCCAATTGGAGGTAAAACAGGAATTAACTATACTAATATATTTAATACTTATGGTCCTT
TAACTGCATTAAATAATGTACCACCAGTTTATCCAAATGGTCAAATTTGGGATAAAGAATTTGATACT
GACTTAAAACCAAGACTTCATGTAAATGCACCATTTGTTTGTCAAAATAATTGTCCTGGTCAATTATT
TGTAAAAGTTGCGCCTAATTTAACAAATGAATATGATCCTGATGCATCTGCTAATATGTCAAGAATTG
TAACTTACTCAGATTTTTGGTGGAAAGGTAAATTAGTATTTAAAGCTAAGCTAAGAGCCTCTCATACT
TGGAATCCAATTCAACAAATGAGTATTAATGTAGATAACCAATTTAACTATGTACCAAGTAATATTGG
AGGTATGAAAATTGTATATGAAAAATCTCAACTAGCACCTAGA
'ARG_JF414819_Arg35_2008'
ATGAGTGATGGAGCAGTTCAACCAGACGGTGGTCAACCTGCTGTCAGAAATGAAAGAGCAACAGGATC
TGGGAACGGGTCTGGAGGCGGGGGTGGTGGTGGTTCTGGGGGTGTGGGGATTTCTACGGGTACTTTCA
ATAATCAGACGGAATTTAAATTTTTGGAAAACGGATGGGTGGAGATCACAGCAAACTCAAGCAGACTT
GTACATTTAAATATGCCAGAAAGTGAAAATTATAGAAGAGTGGTTGTAAATAATTTGGATAAAACTGC
AGTTAACGGAAACATGGCTTTAGATGATACTCATGCACAAATTGTAACACCTTGGTCATTGGTTGATG
CAAATGCTTGGGGAGTTTGGTTTAATCCAGGAGATTGGCAACTAATTGTTAATACTATGAGTGAGTTG
CATTTAGTTAGTTTTGAACAAGAAATTTTTAATGTTGTTTTAAAGACTGTTTCAGAATCTGCTACTCA
GCCACCAACTAAAGTTTATAATAATGATTTAACTGCATCATTGATGGTTGCATTAGATAGTAATAATA
CTATGCCATTTACTCCAGCAGCTATGAGATCTGAGACATTGGGTTTTTATCCATGGAAACCAACCATA
CCAACTCCATGGAGATATTATTTTCAATGGGATAGAACATTAATACCATCTCATACTGGAACTAGTGG
CACACCAACAAATATATACCATGGTACAGATCCAGATGATGTTCAATTTTATACTATTGAAAATTCTG
TGCCAGTACACTTACTAAGAACAGGTGATGAATTTGCTACAGGAACATTTTTTTTTGATTGTAAACCA
TGTAGACTAACACATACATGGCAAACAAATAGAGCATTGGGCTTACCACCATTTCTAAATTCTTTGCC
TCAAGCTGAAGGAGGTACTAACTTTGGTTATATAGGAGTTCAACAAGATAAAAGACGTGGTGTAACTC
AAATGGGAAATACAAACTATATTACTGAAGCTACTATTATGAGACCAGCTGAGGTTGGTTATAGTGCA
CCATATTATTCTTTTGAGGCGTCTACACAAGGGCCATTTAAAACACCTATTGCAGCAGGACGGGGGGG
GGCGCAAACAGATGAAAATCAAGCAGCAGATGGTGATCCAAGATATGCATTTGGTAGACAACATGGTC
AAAAAACTACCACAACAGGAGAAACACCTGAGAGATTTACATATATAGCACATCAAGATACAGGAAGA
TATCCAGAAGGAGATTGGATTCAAAATATTAACTTTAACCTTCCTGTAACAGAAGATAATGTATTGCT
ACCAACAGATCCAATTGGAGGTAAAACAGGAATTAACTATACTAATATATTTAATACTTATGGTCCTT
TAACTGCATTAAATAATGTACCACCAGTTTATCCAAATGGTCAAATTTGGGATAAAGAATTTGATACT
GACTTAAAACCAAGACTTCATGTAAATGCACCATTTGTTTGTCAAAATAATTGTCCTGGTCAATTATT
TGTAAAAGTTGCGCCTAATTTAACAAATGAATATGATCCTGATGCATCTGCTAATATGTCAAGAATTG
TAACTTACTCAGATTTTTGGTGGAAAGGTAAATTAGTATTTAAAGCTAAACTAAGAGCCTCTCATACT

TGGAATCCAATTCAACAAATGAGTATTAATGTAGATAACCAATTTAACTATGTACCAAGTAATATTGG
AGGTATGAAAATTGTATATGAAAAATCTCAACTAGCACCTAGA
'ARG_JF414820_Arg44_2009'
ATGAGTGATGGAGCAGTTCAACCAGACGGTGGTCAACCTGCTGTCAGAAATGAAAGAGCAACAGGATC
TGGGAACGGGTCTGGAGGCGGGGGTGGTGGTGGTTCTGGGGGTGTGGGGATTTCTACGGGTACTTTCA
ATAATCAGACGGAATTTAAATTTTTGGAAAACGGATGGGTGGAAATCACAGCAAACTCAAGCAGACTT
GTACATTTAAATATGCCAGAAAGTGAAAATTATAGAAGAGTGGTTGTAAATAATTTGGATAAAACTGC
AGTTAACGGAAACATGGCTTTAGATGATACTCATGCACAAATTGTAACACCTTGGTCATTGGTTGATG
CAAATGCTTGGGGAGTTTGGTTTAATCCAGGAGATTGGCAACTAATTGTTAATACTATGAGTGAGTTG
CATTTAGTTAGTTTTGAACAAGAAATTTTTAATGTTGTTTTAAAGACTGTTTCAGAATCTGCTACTCA
GCCACCAACTAAAGTTTATAATAATGATTTAACTGCATCATTGATGGTTGCATTAGATAGTAATAATA
CTATGCCATTTACTCCAGCAGCTATGAGATCTGAGACATTGGGTTTTTATCCATGGAAACCAACCATA
CCAACTCCATGGAGATATTATTTTCAATGGGATAGAACATTAATACCATCTCATACTGGAACCAGTGG
CACACCAACAAATATATACCATGGTACAGATCCAGATGATGTTCAATTTTATACTATTGAAAATTCTG
TGCCAGTACACTTACTAAGAACAGGTGATGAATTTGCTACAGGAACATTTTTTTTTGATTGTAAACCA
TGTAGACTAACACATACATGGCAAACAAATAGAGCATTGGGCTTACCACCATTTCTAAATTCTTTGCC
TCAAGCTGAAGGAGGTACTAACTTTGGTTATATAGGAGTTCAACAAGATAAAAGACGTGGTGTAACTC
AAATGGGAAATACAAACTATATTACTGAAGCTACTATTATGAGACCAGCTGAGGTTGGTTATAGTGCA
CCATATTATTCTTTTGAGGCGTCTACACAAGGGCCATTTAAAACACCTATTGCAGCAGGACGGGGGGG
AGCGCAAACAGATGAAAATCAAGCAGCAGATGGTGATCCAAGATATGCATTTGGTAGACAACATGGTC
AAAAAACTACCACAACAGGAGAAACACCTGAGAGATTTACATATATAGCACATCAAGATACAGGAAGA
TATCCAGAAGGAGATTGGATTCAAAATATTAACTTTAACCTTCCTGTAACAGAAGATAATGTATTGCT
ACCAACAGATCCAATTGGAGGTAAAACAGGAATTAACTATACTAATATATTTAATACTTATGGTCCTT
TAACTGCATTAAATAATGTACCACCAGTTTATCCAAATGGTCAAATTTGGGATAAAGAATTTGATACT
GACTTAAAACCAAGACTTCATGTAAATGCACCATTTGTTTGTCAAAATAATTGTCCTGGTCAATTATT
TGTAAAAGTTGCGCCTAATTTAACAAATGAATATGATCCTGATGCATCTGCTAATATGTCAAGAATTG
TAACTTACTCAGATTTTTGGTGGAAAGGTAAATTAGTATTTAAAGCTAAACTAAGAGCCTCTCATACT
TGGAATCCAATTCAACAAATGAGTATTAATGTAGATAACCAATTTAACTATGTACCAAGTAATATTGG
AGGTATGAAAATTGTATATGAAAAATCTCAACTAGCACCTAGA
'ARG_JF414821_Arg48_2009'
ATGAGTGATGGAGCAGTTCAACCAGACGGTGGTCAACCTGCTGTCAGAAATGAAAGAGCAACAGGATC
TGGGAACGGGTCTGGAGGCGGGGGTGGTGGTGGTTCTGGGGGTGTGGGGATTTCTACGGGTACTTTCA
ATAATCAGACGGAATTTAAATTTTTGGAAAACGGATGGGTGGAAATCACAGCAAACTCAAGCAGACTT
GTACATTTAAATATGCCAGAAAGTGAAAATTATAGAAGAGTGGTTGTAAATAATTTGGATAAAACTGC
AGTTAACGGAAACATGGCTTTAGATGATACTCATGCACAAATTGTAACACCTTGGTCATTGGTTGATG
CAAATGCTTGGGGAGTTTGGTTTAATCCAGGAGATTGGCAACTAATTGTTAATACTATGAGTGAGTTG
CATTTAGTTAGTTTTGAACAAGAAATTTTTAATGTTGTTTTAAAGACTGTTTCAGAATCTGCTACTCA
GCCACCAACTAAAGTTTATAATAATGATTTAACTGCATCATTGATGGTTGCATTAGATAGTAATAATA
CTATGCCATTTACTCCAGCAGCTATGAGATCTGAGACATTGGGTTTTTATCCATGGAAACCAACCATA
CCAACTCCATGGAGATATTATTTTCAATGGGATAGAACATTAATACCATCTCATACTGGAACCAGTGG
CACACCAACAAATATATACCATGGTACAGATCCAGATGATGTTCAATTTTATACTATTGAAAATTCTG
TGCCAGTACACTTACTAAGAACAGGTGATGAATTTGCTACAGGAACATTTTTTTTTGATTGTAAACCA
TGTAGACTAACACATACATGGCAAACAAATAGAGCATTGGGCTTACCACCATTTCTAAATTCTTTGCC
TCAAGCTGAAGGAGGTACTAACTTTGGTTATATAGGAGTTCAACAAGATAAAAGACGTGGTGTAACTC
AAATGGGAAATACAAACTATATTACTGAAGCTACTATTATGAGACCAGCTGAGGTTGGTTATAGTGCA
CCATATTATTCTTTTGAGGCGTCTACACAAGGGCCATTTAAAACACCTATTGCAGCAGGACGGGGGGG
AGCGCAAACAGATGAAAATCAAGCAGCAGATGGTGATCCAAGATATGCATTTGGTAGACAACATGGTC
AAAAAACTACCACAACAGGAGAAACACCTGAGAGATTTACATATATAGCACATCAAGATACAGGAAGA
TATCCAGAAGGAGATTGGATTCAAAATATTAACTTTAACCTTCCTGTAACAGAAGATAATGTATTGCT
ACCAACAGATCCAATTGGAGGTAAAGCAGGAATTAACTATACTAATATATTTAATACTTATGGTCCTT
TAACTGCATTAAATAATGTACCACCAGTTTATCCAAATGGTCAAATTTGGGATAAAGAATTTGATACT
GACTTAAAACCAAGACTTCATGTAAATGCACCATTTGTTTGTCAAAATAATTGTCCTGGTCAATTATT
TGTAAAAGTTGCGCCTAATTTAACAAATGAATATGATCCTGATGCATCTGCTAATATGTCAAGAATTG
TAACTTACTCAGATTTTTGGTGGAAAGGTAAATTAGTATTTAAAGCTAAACTAAGAGCCTCTCATACT

TGGAATCCAATTCAACAAATGAGTATTAATGTAGATAACCAATTTAACTATGTACCAAGTAATATTGG
AGGTATGAAAATTGTATATGAAAAATCTCAACTAGCACCTAGA
'RUS_JN033694_Laika_1993'
ATGAGTGATGGAGCAGTTCAACCAGACGGTGGTCAACCTGCTGTCAGAAATGAAAGAGCTACAGGATC
TGGGAACGGGTCTGGAGGCGGGGGTGGTGGTGGTTCTGGGGGTGTGGGGATTTCTACGGGTACTTTCA
ATAATCAGACGGAATTTAAATTTTTGGAAAACGGATGGGTGGAAATCACAGCAAACTCAAGCAGACTT
GTACATTTAAATATGCCAGAAAGTGAAAATTATAGAAGAGTGAATGTAAATAATTTGGATAAAACTGC
AGTTAACGGAAACATGGCTTTAGATGATACTCATGCACAAATTGTAACACCTTGGTCATTGGTTGATG
CAAATGCTTGGGGAGTTTGGTTTAATCCAGGAGATTGGCAACTAATTGTTAATACTATGAGTGAGTTG
CATTTAGTTAGTTTTGAACAAGAAATTTTTAATGTTGTTTTAAAGACTGTTTCAGAATCTGCTACTCA
GCCACCAACTAAAGTTTATAATAATGATTTAACTGCATCATTGATGGTTGCATTAGATAGCAATAATA
CTATGCCATTTACTCCAGCAGCTATGAGATCTGAGACATTGGGTTTTTATCCATGGAAACCAACCATA
CCAACTCCATGGAGATATTATTTTCAATGGGATAGAACATTAATACCATCTCATACTGGAACTAGTGG
CACACCAACAAATATATACCATGGTACAGATCCAGATGATGTTCAATTTTATACTATTGAAAATTCTG
TGCCAGTACACTTACTAAGAACAGGTGATGAATTTGCTACAGGAACATTTTTTTTTGATTGTAAACCA
TGTAGACTAACACATACATGGCAAACAAATAGAGCATTGGGCTTACCACCATTTCTAAATTCTTTACC
TCAAGCTGAAGGAGGTACTAACTTTGGTTATATAGGAGTTCAACAAGATAAAAGACGTGGTGTAACTC
AAATGGGAAATACAAACTATATTACTGAAGCTACTATTATGAGACCAGCTGAGGTTGGTTATAGTGCA
CCATATTATTCTTTTGAGGCGTCTACACAAGGGCCATTTAAAACACCTATTGCAGCAGGACGGGGGGG
AGCGCAAACAGATGAAAATCAAGCAGCAGATGGTGATCCAAGATATGCATTTGGTAGACAACATGGTC
AAAAAACTACCACAACAGGAGAAACACCTGAGAGATTTACATATATAGCACATCAAGATACAGGAAGA
TATCCAGAAGGAGATTGGATTCAAAATATTAACTTTAACCTTCCTGTAACAGATGATAATGTATTGCT
ACCAACAGATCCAATTGGAGGTAAAACAGGAATTAACTATACTAATATATTTAATACTTATGGTCCTT
TAACTGCATTAAATAATGTACCACCAGTTTATCCAAATGGTCAAATTTGGGATAAAGAATTTGATACT
GACTTAAAACCAAGACTTCATGTAAATGCACCATTTGTTTGTCAAAATAATTGTCCTGGTCAATTATT
TGTAAAAGTTGCGCCTAATTTAACAAATGAATATGATCCTGATGCATCTGCTAATATGTCAAGAATTG
TGACTTACTCAGATTTTTGGTGGAAAGGTAAATTAGTATTTAAAGCTAAACTAAGAGCCTCTCATACT
TGGAATCCAATTCAACAAATGAGTATTAATGTAGATAACCAATTTAACTATGTACCAAGTAATATTGG
AGGTATGAAAATTGTATATGAAAAATCTCAACTAGCACCTAGA
'VAC_JN625219_INDIA_vac1_2011'
ATGAGTGATGGAGCAGTTCAACCAGACGGTGGTCAACCTGCTGTCAGAAATGAAAGAGCTACAGGATC
TGGGAACGGGTCTGGAGGCGGGGGTGGCGGTGGTTCTGGGGGTGTGGGGATTTCTACGGGTACTTTCA
ATAATCAGACGGAATTTAAATTTTTGGAAAACGGATGGGTGGAAATCACAGCAAACTCAAGCAGACTT
GTACATTTAAATATGCCAGAAAGTGAAAATTATAGAAGAGTGGTTGTGAATAATATGGATAAAACTGC
AGTTAACGGAAACATGGCTTTAGATGATATTCATGCACAAATTGTAACACCTTGGTCATTGGTTGATG
CAAATGCTTGGGGAGTTTGGTTTAATCCAGGAGATTGGCAACTAATTGTTAATACTATGAGTGAGTTG
CATTTAGTTAGTTTTGAACAAGAAATTTTTAATGTTGTTTTAAAGACTGTTTCAGAATCTGCTACTCA
GCCACCAACTAAAGTTTATAATAATGATTTAACTGCATCATTGATGGTTGCATTAGATAGTAATAATA
CTATGCCATTTACTCCAGCAGCTATGAGATCTGAGACATTGGGTTTTTATCCATGGAAACCAACCATA
CCAACTCCATGGAGATATTATTTTCAATGGGATAGAACATTAGTACCATCTCATACTGGAACTAGTGG
CACACCAACAAATATATACCATGGTACAGATCCAGATGATGTTCAATTTTATACTATTGAAAATTCTG
TGCCAGTACACTTACTAAGAACAGGTGATGAATTTGCTACAGGAACATTTTTTTTTGATTGTAAACCA
TGTAGACTAACACATACATGGCAAACAAATAGAGCATTGGGCTTACCACCATTTCTAAATTCTTTGCC
TCAATCTGAAGGAGCTACTAACTTTGGTGATATAGGAGTTCAACAAGATAAAAGACGTGGTGTAACTC
AAATGGGAAATACAAACTATATTACTGAAGCTACTATTATGAGACCAGCTGAGGTTGGTTATAGTGCA
CCATATTATTCTTTTGAGACGTCTACACAAGGGCCATTTAAAACACCTATTGCAGCAGGACGGGGGGG
AGCGCAAACAGATGAAAATCAAGCAGCAGATGGTAATCCAAGATATGCATTTGGTAGACAACATGGTA
AAAAAACTACCACAACAGGAGAAACACCTGAGAGATTTACATATATAGCACATCAAGATACAGGAAGA
TATCCAGAAGGAGATTGGATTCAAAATATTAACTTTAACCTTCCTGTAACAAATGATAATGTATTGCT
ACCAACAGATCCAATTGGAGGTAAAACAGGAATTAACTATACTAATATATTTAATACTTATGGTCCTT
TAACTGCATTAAATAATGTACCACCAGTTTATCCAAATGGTCAAATTTGGGATAAAGAATTTGATACT
GACTTAAAACCAAGACTTCATGTAAATGCACCATTTGTTTGTCAAAATAATTGTCCTGGTCAATTATT
TGTAAAAGTTGCGCCTAATTTAACAAATGAATATGATCCTGATGCATCTGCTAATATGTCAAGAATTG
TAACTTACTCAGATTTTTGGTGGAAAGGTAAATTAGTATTTAAAGCTAAACTAAGAGCCTCTCATACT

TGGAATCCAATTCAACAAATGAGCATTAATGTAGATAACCAATTTAACTATGTACCAAGTAATATTGG
AGGTATGAAAATTGTATATGAAAAATCTCAACTAGCACCTAGA
'VAC_JN625220_INDIA_vac2_2011'
ATGAGTGATGGAGCAGTTCAACCAGACGGTGGTCAACCTGCTGTCAGAAATGAAAGAGCTACAGGATC
TGGGAACGGGTCTGGAGGCGGGGGTGGCGGTGGTTCTGGGGGTGTGGGGATTTCTACGGGTACTTTCA
ATAATCAGACGGAATTTAAATTTTTGGAAAACGGATGGGTGGAAATCACAGCAAACTCAAGCAGACTT
GTACATTTAAATATGCCAGAAAGTGAAAATTATAGAAGAGTGGTTGTAAATAATATGGATAAAACTGC
AGTTAACGGAAACATGGCTTTAGATGATATTCATGCACAAATTGTAACACCTTGGTCATTGGTTGATG
CAAATGCTTGGGGAGTTTGGTTTAATCCAGGAGATTGGCAACTAATTGTTAATACTATGAGTGAGTTG
CATTTAGTTAGTTTTGAACAAGAAATTTTTAATGTTGTTTTAAAGACTGTTTCAGAATCTGCTACTCA
GCCACCAACTAAAGTTTATAATAATGATTTAACTGCATCATTGATGGTTGCATTAGATAGTAATAATA
CTATGCCATTTACTCCAGCAGCTATGAGATCTGAGACATTGGGTTTTTATCCATGGAAACCAACCATA
CCAACTCCATGGAGATATTACTTTCAATGGGATAGAACATTAATACCATCTCATACTGGAACTAGTGG
CACACCAACAAATATATACCATGGTACAGATCCAGATGATGTTCAATTTTATACTATTGAAAATTCTG
TGCCAGTACACTTACTAAGAACAGGTGATGAATTTGCTACAGGAACATTTTTTTTTGATTGTAGACCA
TGTAGACTAACACATACATGGCAAACAAATAGAGCATTGGGCTTACCACCATTTCTAAATTCTTTGCC
TCAATCTGAAGGAGCTACTAACTTTGGTGATATAGGAGTTCAACAAGATAAAAGACGTGGTATAACTC
AAATGGGAAATACAAACTATATTACTGAAGCTACTATTATGAGACCAGCTGAGGTTGGTTATAGTGCA
CCATATTATTCTTTTGAGGCGTCTACACAAGGGCCATTTAAAACACCTATTGCAGCAGGACGGGGGGG
AGCGCAAACAGATGAAAATCAAGCAGCAGATGGTAATCCAAGATATGCATTTGGTAGACAACATGGTC
AAAAAACTACCACAACAGGAGAAACACCTGAGAGATTTACATATATAGCACATCAAGATACAGGAAGA
TATCCAGAAGGAGATTGGATTCAAAATATTAACTTTAACCTTCCTGTAACAAATGATAATGTATTGCT
ACCAACAGATCCAATTGGAGGTAAAACAGGAATTAACTATACTAATATATTTAATACTTATGGTCCTT
TAACTGCATTAAATAATGTACCACCAATTTATCCAAATGGTCAAATTTGGGATAAAGAATTTGATACT
GACTTAAAACCAAGACTTCATGTAAATGCACCATTTGTTTGTCAAAATAATTGTCCTGGTCAATTATT
TGTAAAAGTTGCGCCTAATTTAACAAATGAATATGATCCTGATGCATCTGCTAATATGTCAAGAATTG
TAACTTACTCAGATTTCTGGTGGAAAGGTAAATTAGTATTTAAAGCTAAACTAAGAGCCTCTCATACT
TGGAATCCAATTCAACAAATGAGTATTAATGTAGATAACCAATTTAACTATGTACCAAGTAATATTGG
AGGTATGAAAATTGTATTTGAAAAATCTCAACTAGCACCTAGA
'VAC_JN625221_INDIA_vac3_2011'
ATGAGTGATGGAGCAGTTCAACCAGACGGTGGTCAACCTGCTGTCAGAAATGAAAGAGCTACAGGATC
TGGGAACGGGTCTGGAGGCGGGGGTGGTGGTGGTTCTGGGGGTGTGGGGATTTCTACGGGTGCTTTCA
ATAATCAGACGGAATTTAAATTTTTGGAAAACGGATGGGTGGAAATCACAGCAAACTCAAGCAGACTT
GTACATTTAAATATGCCAGAAAGTGAAAATTATAGAAGAGTGGTTGTAAATAATATGGATAAAACTGC
AGTTAACGGAAACATGGCTTTAGATGATATTCATGCACAAATTGTAACACCTTGGTCATTGGTTGATG
CAAATGCTTGGGGAGTTTGGTTTAATCCAGGAGATTGGCAACTAATTGTTAATACTATGAGTGAGTTG
CATTTAGTTAGTTTTGAACAAGAAATTTTTAATGTTGTTTTAAAGACTGTTTCAGAATCTGCTACTCA
GCCACCAACTAAAGTTTATAATAATGATTTAACTGCATCATTGATGGTTGCATTAGATAGTAATAATA
CTATGCCATTTACTCCAGCAGCTATGAGATCTGAGACATTGGGTTTTTATCCATGGAAACCAACCATA
CCAACTCCATGGAGATATTATTTTCAATGGGATAGAACATTAATACCATCTCATACTGGAACTAGTGG
CACACCAACAAATATATACCATGGTACAGATCCAGATGATGTTCAATTTTATACTATTGAAAATTCTG
TGCCAGTACACTTACTAAGAACAGGTGATGAATTTGCTACAGGAACATTTTTTTTTGATTGTAAACCA
TGTAGACTAACACATACATGGCAAACAAATAGAGCATTGGGCTTACCACCATTTCTAAATTCTTTGCC
TCAATCTGAAGGAGCTACTAACTTTGGTGATATAGGAGTTCAACAAGATAAAAGACGTGGTGTAACTC
AAATGGGAAATACAAACTATATTACTGAAGCTACTATTATGAGACCAGCTGAGGTTGGTTATAGTGCA
CCATATTATTCTTTTGAGGCGTCTACACAAGGGCCATTTAAAACACCTATTGCAGCAGGACGGGGGGG
AGCGCAAACAGATGAAAATCAAGCAGCAGATGGTAATCCAAGATATGCATTTGGTAGACAACATGGTC
AAAAAACTACCACAACAGGAGAAACACCTGAGAGATTTACATATATAGCACATCAAGATACAGGAAGA
TATCCAGAAGGAGATTGGATTCAAAAAATTAACTTTAACCTTCCTGTAACAAATGATAATGTATTGCT
ACCAACAGATCCAATTGGAGGTAAAACAGGAATTAACTATACTAATATATTTAATACTTATGGTCCTT
TAACTGCATTAAATAATGTACCACCAGTTTATCCAAATGGTCAAATTTGGGATAAAGAATTTGATACT
GACTTAAAACCAAGACTTCATGTAAATGCACCATTTGTTTGTCAAAATAATTGTCCTGGTCAATTATT
TGTAAAAGTTGCGCCTAATTTAACGAATGAATATGATCCTGATGCATCTGCTAATATGTCAAGAATTG
TAACTTACTCAGATTTTTGGTGGAAAGGTAAATTAGTATTTAAAGCTAAACTAAGAGCCTCTCATACT

TGGAATCCAATTCAACAAATGAGTATTAATGTAGATAACCAATTTAACTATGTACCAAGTAATATTGG
AGGTATGAAAATTGTATATGAAAAATCTCAACTAGCACCTAGA
'VAC_JN625222_INDIA_vac4_2011'
ATGAGTGATGGAGCAGTTCAACCAGACGGTGGTCAACCTGCTGTCAGAAATGAAAGAGCTACAGGATC
TGGGAACGGGTCTGGAGGCGGGGGTGGTGGTGGTTCTGGGGGTGTGGGGATTTCTACGGGTGCTTTCA
ATAATCAGACGGAATTTAAATTTTTGGAAAACGGATGGGTGGAAATCACAGCAAACTCAAGCAGACTT
GTACATTTAAATATGCCAGAAAGTGAAAATTATAGAAGAGTGGTTGTGAATAATATGGATAAAACTGC
AGTTAACGGAAACATGGCTTTAGATGATATTCATGCACAAATTGTAACACCTTGGTCATTGGTTGATG
CAAATGCTTGGGGAGTTTGGTTTAATCCAGGAGATTGGCAACTAATTGTTAATACTATGAGTGAGTTG
CATTTAGTTAGTTTTGAACAAGAAATTTTTAATGTTGTTTTAAAGACTGTTTCAGAATCTGCTACTCA
GCCACCAACTAAAGTTTATAATAATGATTTAACTGCATCATTGATGGTTGCATTAGATAGTAATAATA
CTATGCCATTTACTCCAGCAGCTATGAGATCTGAGACATTGGGTTTTTATCCATGGAAACCAACCATA
CCAACTCCATGGAGATATTATTTTCAATGGGATAGAACATTAGTACCATCTCATACTGGAACTAGTGG
CACACCAACAAATATATACCATGGTACAGATCCAGATGATGTTCAATTTTATACTATTGAAAATTCTG
TGCCAGTACACTTACTAAGAACAGGTGATGAATTTGCTACAGGAACATTTTTTTTTGATTGTAAACCA
TGTAGACTAACACATACATGGCAAACAAATAGAGCATTGGGCTTACCACCATTTCTAAATTCTTTGCC
TCAATCTGAAGGAGCTACTAACTTTGGTGATATAGGAGTTCAACAAGATAAAAGACGTGGTGTAACTC
AAATGGGAAATACAAACTATATTACTGAAGCTACTATTATGAGACCAGCTGAGGTTGGTTATAGTGCA
CCATATTATTCTTTTGAGGCGTCTACACAAGGGCCATTTAAAACACCTATTGCAGCAGGACGGGGGGG
AGCGCAAACAGATGAAAATCAAGCAGCAGATGGTAATCCAAGATATGCATTTGGTAGACAACATGGTA
AAAAAACTACCACAACAGGAGAAACACCTGAGAGATTTACATATATAGCACATCAAGATACAGGAAGA
TATCCAGAAGGAGATTGGATTCAAAAAATTAACTTTAACCTTCCTGTAACAAATGATAATGTATTGCT
ACCAACAGATCCAATTGGAGGTAAAACAGGAATTAACTATACTAATATATTTAATACTTATGGTCCTT
TAACTGCATTAAATAATGTACCACCAGTTTATCCAAATGGTCAAATTTGGGATAAAGAATTTGATACT
GACTTAAAACCAAGACTTCATGTAAATGCACCATTTGTTTGTCAAAATAATTGTCCTGGTCAATTATT
TGTAAAAGTTGCGCCTAATTTAACAAATGAATATGATCCTGATGCATCTGCTAATATGTCAAGAATTG
TAACTTACTCAGATTTTTGGTGGAAAGGTAAATTAGTATTTAAAGCTAAACTAAGAGCCTCTCATACT
TGGAATCCAATTCAACAAATGAGTATTAATGTAGATAACCAATTTAACTATGTACCAAGTAATATTGG
AGGTATGAAAATTGTATATGAAAAATCTCAACTAGCACCTAGA
'VAC_JN625223_INDIA_vac5_2011'
ATGAGTGATGGAGCAGTTCAACCAGACGGTGGTCAACCTGCTGTCAGAAATGAAAGAGCTACAGGATC
TGGGAACGGGTCTGGAGGCGGGGGTGGTGGTGGTTCTGGGGGTGTGGGGATTTCTACGGGTACTTTCA
ATAATCAGACGGAATTTAAATTTTTGGAAAACGGATGGGTGGAAATCACAGCAAACTCAAGCAGACTT
GTACATTTAAATATGCCAGAAAGTGAAAATTATAGAAGAGTGGTTGTAAATAATTTGGATAAAACTGC
AGTTAACGGAAACATGGCTTTAGATGATACTCATGCACAAACTGTAACACCTTGGTCATTGGTTGATG
CAAATGCTTGGGGAGTTTGGTTTAATCCAGGAGATTGGCAACTAATTGTTAATACTATGAGTGAGTTG
CATTTAGTTAGTTTTGAACAAGAAATTTTTAATGTTGTTTTAAAGACTGTTTCAGAATCTGCTACTCA
GCCACCAACTAAAGTTTATAATAATGATTTAACTGCATCATTGATGGTTGCATTAGATAGTAATAATA
CTATGCCATTTACTCCAGCAGCTATGAGATCTGAGACATTGGGTTTTTATCCATGGAAACCAACCATA
CCAACTCCATGGAGATATTATTTTCAATGGGATAGAACATTAATACCATCTCATACTGGAACTAGTGG
CACACCAACAAATATATACCATGGTACAGATCCAGATGATGTTCAATTTTATACTATTGAAAATTCTG
TGCCAGTACACTTACTAAGAACAGGTGATGAATTTGCTACAGGAACATTTTTTTTTGATTGTAAACCA
TGTAGACTAACACATACATGGCAAACAAATAGAGCATTGGGCTTACCACCATTTCTAAATTCTTTGCC
TCAAGCTGAAGGAGGTACTAACTTTGGTTATATAGGAGTTCAACAAGATAAAAGACGTGGTGTAACTC
AAATGGGAAAAACAAACTATATTACTGAAGCTACTATTATGAGACCAGCTGAGGTTGGTTATAGTGCA
CCATATTATTCTTTTGAGGCGTCTACACAAGGGCCATTTAAAACACCTATTGCAGCAGGACGGGGGGG
AGCGCAAACAGATGAAAATCAAGCAGCAGATGGTGATCCAAGATATGCATTTGGTAGACAACATGGTC
AAAAAACTACCACAACAGGAGAAACACCTGAGAGATTTACATATATAGCACATCAAGATACAGGAAGA
TATCCAGAAGGAGATTGGATTCAAAATATTAACTTTAACCTTCCTGTAACAGATGATAATGTATTGCT
ACCAACAGATCCAATTGGAGGTAAAACAGGAATTAACTATACTAATATATTTAATACTTATGGTCCTT
TAACTGCATTAAATAATGTACCACCAGTTTATCCAAATGGTCAAATTTGGGATAAAGAATTTGATACT
GACTTAAAACCAAGACTTCATGTAAATGCACCATTTGTTTGTCAAAATAATTGTCCTGGTCAATTATT
TGTAAAAGTTGCGCCTAATTTAACAAATGAATATGATCCTGATGCATCTGCTAATATGTCAAGAATTG
TAACTTACTCAGATTTTTGGTGGAAAGGTAAATTAGTATTTAAAGCTAAACTAAGAGCCTCTCATACT

TGGAATCCAATTCAACAAATGAGTATTAATGTAGATAACCAATTTAACTATGTACCAAGTAATATTGG
AGGTATGGAAATTGTATATGAAAGATCTCAACTAGCACCTAGA
'VAC_JN625224_INDIA_vac6_2011'
ATGAGTGATGGAGCAGTTCAACCAGACGGTGGTCAACCTGCTGTCAGAAATGAAAGAGCTACAGGATC
TGGGAACGGGTCTGGAGGCGGGGGTGGTGGTGGTTCTGGGGGTGTGGGGATTTCTACGGGTGCTTTCA
ATAATCAGACGGAATTTAAATTTTTGGAAAACGGATGGGTGGAAATCACAGCAAACTCAAGCAGACTT
GTACATTTAAATATGCCAGAAAGTGAAAATTATAGAAGAGTGGTTGTAAATAATATGGATAAAACTGC
AGTTAACGGAAACATGGCTTTAGATGATATTCATGCACAAATTGTAACACCTTGGTCATTGGTTGATG
CAAATGCTTGGGGAGTTTGGTTTAATCCAGGAGATTGGCAACTAATTGTTAATACTATGAGTGAGTTG
CATTTAGTTAGTTTTGAACAAGAAATTTTTAATGTTGTTTTAAAGACTGTTTCAGAATCTGCTACTCA
GCCACCAACTAAAGTTTATAATAATGATTTAACTGCATCATTGATGGTTGCATTAGATAGTAATAATA
CTATGCCATTTACTCCAGCAGCTATGAGATCTGAGACATTGGGTTTTTATCCATGGAAACCAACCATA
CCAACTCCATGGAGATATTATTTTCAATGGGATAGAACATTAATACCATCTCATACTGGAACTAGTGG
CACACCAACAAATATATACCATGGTACAGATCCAGATGATGTTCAATTTTATACTATTGAAAATTCTG
TGCCAGTACACTTACTAAGAACAGGTGATGAATTTGCTACAGGAACATTTTTTTTTGATTGTAGACCA
TGTAGACTAACACATACATGGCAAACAAATAGAGCATTGGGCTTACCACCATTTCTAAATTCTTTGCC
TCAATCTGAAGGAGATATTAACTTTGGTGATATAGGAGTTCAACAAGATAAAAGACGTGGTATAACTC
AAATGGGAAATACAAACTATATTACTGAAGCTACTATTATGAGACCAGCTGAGGTTGGTTATAGTGCA
CCATATTATTCTTTTGAGGCGTCTACACAAGGGCCATTTAAAACACCTATTGCAGCAGGACGGGGGGG
AGCGCAAACAGATGAAAATCAAGCAGCAGATGGTAATCCAAGATATGCATTTGGTAGACAACATGGTC
AAAAAACTACCACAACAGGAGAAACACCTGAGAGATTTACATATATAGCACATCAAGATACAGGAAGA
TATCCAGAAGGAGATTGGATTCAAAATATTAACTTTAACCTTCCTGTAACAAATGATAATGTATTGCT
ACCAACAGATCCAATTGGAGGTAAAACAGGAATTAACTATACTAATATATTTAATACTTATGGTCCTT
TAGCTGCATTAAATAATGTACCACCAGTTTATCCAAATGGTCAAATTTGGGATAAAGAATTTGATACT
GACTTAAAACCAAGACTTCATGTAAATGCACCATTTGTTTGTCAAAATAATTGTCCTGGTCAATTATT
TGTAAAAGTTGCGCCTAATTTAACGAATGAGTATGATCCTGATGCATCTGCTAATATGTCAAGAATTG
TAACTTACTCAGATTTTTGGTGGAAAGGTAAATTAGTATTTAAAGCTAAACTAAGAGCCTCTCATACT
TGGAATCCAATTCAACAAATGAGTATTAATGTAGATAACCAATTTAACTATGTACCAAGTAATATTGG
AGGTATGAAAATTGTATATGAAAAATCTCAACTAGCACCTAGA
'USA_JN867598_Bobcat_KS_44_2010'
ATGAGTGATGGAGCAGTTCAACCAGACGGTGGTCAACCTGCTGTCAGAAATGAAAGAGCTACAGGATC
TGGGAACGGGTCTGGAGGCGGGGGTGGTGGTGGTTCTGGGGGTGTGGGGATTTCTACGGGTACTTTCA
ATAATCAGACGGAATTTAAATTTTTGGAAAACGGATGGGTGGAAATCACAGCAAACTCAAGCAGACTT
GTACATTTAAATATGCCAGAAAGTGAAAATTATAGAAGAGTGGTTGTAAATAATTTAGATAAAACTGC
AGTTAACGGAAACATGGCTTTAGATGATACTCATGCACAAATTGTAACACCTTGGTCATTGGTTGATG
CAAATGCTTGGGGAGTTTGGTTTAATCCAGGAGATTGGCAACTAATTGTTAATACTATGAGTGAGTTG
CATTTAGTTAGTTTTGAACAAGAAATTTTTAATGTTGTTTTAAAGACTGTTTCAGAATCTGCTACTCA
GCCACCAACTAAAGTTTATAATAATGATTTAACTGCATCATTGATGGTTGCATTAGATAGTAATAATA
CTATGCCATTTACTCCAGCAGCTATGAGATCTGAGACATTGGGTTTTTATCCATGGAAACCAACCATA
CCAACTCCATGGAGATATTATTTTCAATGGGATAGAACATTAATACCATCTCATACTGGAACTAGTGG
CACACCAACAAATACATACCATGGTACAGATCCAGATGATGTTCAATTTTATACTATTGAAAATTCTG
TGCCAGTACACTTACTAAGAACAGGTGATGAATTTGCTACAGGAACATTTTTTTTTGATTGTAAACCA
TGTAGACTAACACATACATGGCAAACAAATAGAGCATTGGGCTTACCACCATTTCTAAATTCTTTGCC
TCAATCTGAAGGAGATACTAACTTTGGTGATATAGGAGTTCAACAAGATAAAAGACGTGGTGTAACTC
AAATGGGAAATACAAACTATATTACTGAAGCTACTATTATGAGACCAGCTGAGGTTGGTTATAGTGCA
CCATATTATTCTTTTGAGGCGTCTACACAAGGGCCATTTAAAACACCTATTGCAGCAGGACGGGGGGG
AGCGCAAACAGATGAAAATCAAGCAGCAGATGGTGATCCAAGATATGCATTTGGTAGACAACATGGTC
AAAAAACTACCACAACAGGAGAAACACCTGAGAGATTTACATATATAGCACATCAAGATACAGGAAGA
TATCCAGAAGGAGATTGGATTCAAAATATTAACTTTAACCTTCCTGTAACAAATGATAATGTATTGCT
ACCAACAGATCCAATTGGAGGTAAAACAGGAATTAACTATACTAATATATTTAATACTTATGGTCCTT
TAACTGCATTAAATAATGTACCACCAGTTTATCCAAATGGTCAAATTTGGGATAAAGAATTTGATACT
GACTTAAAACCAAGACTTCATGTAAATGCACCATTTGTTTGTCAAAATAATTGTCCTGGTCAATTATT
TGTAAAAGTTGCACCTAATTTAACAAATGAATATGATCCTGATGCATCTGCTAATATGTCAAGAATTG
TAACTTACTCAGATTTTTGGTGGAAAGGTAAATTAGTATTTAAAGCTAAACTAAGAGCCTCTCATACT

TGGAATCCAATTCAACAAATGAGTATTAATGTAGATAACCAATTTAACTATGTACCAAGTAATATTGG
AGGTATGAAAATTGTATATGAAAAATCTCAACTAGCACCTAGA

'USA_JN867599_Raccoon_KY_39552_2009'

ATGAGTGATGGAGCAGTTCAACCAGACGGTGGTCAACCTGCTGTCAGAAATGAAAGAGCTACAGGATC
TGGGAACGGGTCTGGAGGCGGGGGTGGTGGTGGTTCTGGGGGTGTGGGGATTTCTACGGGTACTTTCA
ATAATCAGACGGAATTTAAATTTTTGGAAAACGGATGGGTGGAAATCACAGCAAACTCAAGCAGACTT
GTACATTTAAATATGCCAGAAAGTGAAAATTATAGAAGAGTGGTTGTAAATAATTTGGATAAAACTGC
AGTTAACGGAAACATGGCTTTAGATGATACTCATGCACAAATTGTAACACCTTGGTCATTGGTTGATG
CAAATGCTTGGGGAGTTTGGTTTAATCCAGGAGATTGGCAACTAATTGTTAATACTATGAGTGAGTTG
CATTTAGTTAGTTTTGAACAAGAAATTTTTAATGTTGTTTTAAAGACTGTTTCAGAATCTGCTACTCA
GCCACCAACTAAAGTTTATAATAATGATTTAACTGCATCATTGATGGTTGCATTAGATAGTAATAATA
CTATGCCATTTACTCCAGCAGCTATGAGATCTGAGACATTGGGTTTTTATCCATGGAAACCAACCATA
CCAACTCCATGGAGATATTATTTTCAATGGGATAGAACATTAATACCATCTCATACTAGAACTAGTGG
CACACCAACAAATATATACCATGGTACAGATCCAGATGATGTTCAATTTTATACTATTGAAAATTCTG
TGCCAGTACACTTACTAAGAACAGGTGATGAATTTGCTACAGGAACATTTTTTTTTGATTGTAAACCA
TGTAGACTAACACATACATGGCAAACAAATAGAGCATTGGGCTTACCACCATTTCTAAATTCTTTGCC
TCAAGCTGAAGGAGATACTAACTTTGGTGATATAGGAGTTCAACAAGATAAAAGACGTGGTGTGACTC
AAATGGGAAATACAAACTATATTACTGAAGCTACTATTATGAGACCAGCTGAGGTTGGTTATAGTGCA
CCATATTATTCTTTTGAGGCGTCTACACAAGGGCCATTTAAAACACCTATTGCAGCAGGACGGGGGGG
AGCGCAAACAGATGAAAATCAAGCAGCAGATGGTGATCCAAGATATGCATTTGGTAGACAACATGGTC
AAAAAACTACCACAACAGGAGAAACACCTGAGAGATTTACATATATAGCACATCAAGATACAGGAAGA
TATCCAGAAGGAGATTGGATTCAAAATATTAACTTTAACCTTCCTGTAACAAATGATAATGTATTGCT
ACCAACAGATCCAATTGGAGGTAAAACAGGAATTAACTATACTAATATATTTAATACTTATGGTCCTT
TAACTGCATTAAATAATGTACCACCAGTTTATCCAAATGGTCAAATTTGGGATAAAGAATTTGATACT
GACTTAAAACCAAGACTTCATGTAAATGCACCATTTGTTTGTCAAAATAATTGTCCTGGTCAATTATT
TGTAAAAGTTGCGCCTAATTTAACAAATGAATATGATCCTGATGCATCTGCTAATATGTCAAGAATTG
TAACTTACTCAGATTTTTGGTGGAAAGGTAAATTAGTATTTAAAGCTAAACTAAGAGCCTCTCATACT
TGGAATCCAATTCAACAAATGAGTATTAATGTAGATAACCAATTTAACTATGTACCAAGTAATATTGG
AGGTATGAAAATTGTATATGAAAAATCTCAACTAGCACCTAGA

'USA_JN867602_2b_Dog_CA_148743_2008'

ATGAGTGATGGAGCAGTTCAACCAGACGGTGGTCAGCCTGCTGTCAGAAATGAAAGAGCTACAGGATC
TGGGAACGGGTCTGGAGGCGGGGGTGGTGGTGGTTCTGGGGGTGTGGGGATTTCTACGGGTACTTTCA
ATAATCAGACGGAATTTAAATTTTTGGAAAACGGATGGGTGGAAATCACAGCAAACTCAAGCAGACTT
GTACATTTAAATATGCCAGAAAGTGAAAATTATAGAAGAGTGGTTGTAAATAATTTGGATAAAACTGC
AGTTAACGGAAACATGGCTTTAGATGATACTCATGCACAAATTGTAACACCTTGGTCATTGGTTGATG
CAAATGCTTGGGGAGTTTGGTTTAATCCAGGAGATTGGCAACTAATTGTTAATACTATGAGTGAGTTG
CATTTAGTTAGTTTTGAACAAGAAATTTTTAATGTTGTTTTAAAGACTGTTTCAGAATCTGCTACTCA
GCCACCAACTAAAGTTTATAATAATGATTTAACTGCATCATTGATGGTTGCATTAGATAGTAATAATA
CTATGCCATTTACTCCAGCAGCTATGAGATCTGAGACATTGGGTTTTTATCCATGGAAACCAACCATA
CCAACTCCATGGAGATATTATTTTCAATGGGATAGAACATTAATACCATCTCATACTGGAACTAGTGG
CACACCAACAAATATATACCATGGTACAGATCCAGATGATGTTCAATTTTATACTATTGAAAATTCTG
TGCCAGTACACTTACTAAGAACAGGTGATGAATTTGCTACAGGAACATTTTTTTTTGATTGTAAACCA
TGTAGACTAACACATACATGGCAAACAAATAGAGCATTGGGCTTACCACCATTTCTAAATTCTTTGCC
TCAAGCTGAAGGAGGTACTAACTTTGGTTATATAGGAGTTCAACAAGATAAAAGACGTGGTGTAACTC
AAATGGGAAATACAAACTATATTACTGAAGCTACTATTATGAGACCAGCTGAGGTTGGTTATAGTGCA
CCATATTATTCTTTTGAGGCGTCTACACAAGGGCCATTTAAAACACCTATTGCAGCAGGACGGGGGGG
AGCGCAAACAGATGAAAATCAAGCAGCAGATGGTGATCCAAGATATGCATTTGGTAGACAACATGGTC
AAAAAACTACCACAACAGGAGAAACACCTGAGAGATTTACATATATAGCACATCAAGATACAGGAAGA
TATCCAGAAGGAGATTGGATTCAAAATATTAACTTTAACCTTCCTGTAACAGATGATAATGTGTTGCT
ACCAACAGATCCAATTGGAGGTAAAACAGGAATTAACTATACTAATATATTTAATACTTATGGTCCTT
TAACTGCATTAAATAATGTACCACCAGTTTATCCAAATGGTCAAATTTGGGATAAAGAATTTGATACT
GACTTAAAACCAAGACTTCATGTAAATGCACCATTTGTTTGTCAAAATAATTGCCCTGGTCAATTATT
TGTAAAAGTTGCGCCTAATTTAACAAATGAATATGATCCTGATGCATCTGCTAATATGTCAAGAATTG
TAACTTACTCAGATTTTTGGTGGAAAGGTAAATTAGTATTTAAAGCTAAACTAAGAGCCTCTCATACT

TGGAATCCAATTCAACAAATGAGTATTAATGTAGATAACCAATTTAACTATGTACCAAGTAATATTGG
AGGTATGAAAATTGTCTATGAAAAATCTCAACTAGCACCTAGA

'USA_JN867603_2b_Dog_KS_81213_2009'

ATGAGTGATGGAGCAGTTCAACCAGACGGTGGTCAGCCTGCTGTCAGAAATGAAAGAGCTACAGGATC
TGGGAACGGGTCTGGAGGCGGGGGTGGTGGTGGTTCTGGGGGTGTGGGGATTTCTACGGGTACTTTCA
ATAATCAGACGGAATTTAAATTTTTGGAAAACGGATGGGTGGAAATCACAGCAAACTCAAGCAGACTT
GTACATTTAAATATGCCAGAAAGTGAAAATTATAGAAGAGTGGTTGTAAATAATTTGGATAAAACTGC
AGTTAACGGAAACATGGCTTTAGATGATACTCATGCACAAATTGTAACACCTTGGTCATTGGTTGATG
CAAATGCTTGGGGAGTTTGGTTTAATCCAGGAGATTGGCAACTAATTGTTAATACTATGAGTGAGTTG
CATTTAGTTAGTTTTGAACAAGAAATTTTTAATGTTGTTTTAAAGACTGTTTCAGAATCTGCTACTCA
GCCACCAACTAAAGTTTATAATAATGATTTAACTGCATCATTGATGGTTGCATTAGATAGTAATAATA
CTATGCCATTTACTCCAGCAGCTATGAGATCTGAGACATTGGGTTTTTATCCATGGAAACCAACCATA
CCAACTCCATGGAGATATTATTTTCAGTGGGATAGAACATTAATACCATCTCATACTGGAACTAGTGG
CACACCAACAAATATATACCATGGTACAGATCCAGATGATGTTCAATTTTATACTATTGAAAATTCTG
TGCCAGTACACTTACTAAGAACAGGTGATGAATTTGCTACAGGAACATTTTTTTTTGATTGTAAACCA
TGTAGACTAACACATACATGGCAAACAAATAGAGCATTGGGCTTACCACCATTTCTAAATTCTTTGCC
TCAAGCTGAAGGAGGTACTAACTTTGGTTATATAGGAGTTCAACAAGATAAAAGACGTGGTGTAACTC
AAATGGGAAATACAAACTATATTACTGAAGCTACTATTATGAGACCAGCTGAGGTTGGTTATAGTGCA
CCATATTATTCTTTTGAGGCGTCTACACAAGGGCCATTTAAAACACCTATTGCAGCAGGACGGGGGGG
AGCGCAAACAGATGAAAATCAAGCAGCAGATGGTGATCCAAGATATGCATTTGGTAGACAACATGGTC
AAAAAACTACCACAACAGGAGAAACACCTGAGAGATTTACATATATAGCACATCAAGATACAGGAAGA
TATCCAGAAGGAGATTGGATTCAAAATATTAACTTTAACCTTCCTGTAACAGATGATAATGTATTGCT
ACCAACAGATCCAATTGGAGGTAAAACAGGAATTAACTATACTAATATATTTAATACTTATGGTCCTT
TAACTGCATTAAATAATGTACCACCAGTTTATCCAAATGGTCAAATTTGGGATAAAGAATTTGATACT
GACTTAAAACCAAGACTTCATGTAAATGCACCATTTGTTTGTCAAAATAATTGCCCTGGTCAATTATT
TGTAAAAGTTGCGCCTAATTTAACAAATGAATATGATCCTGATGCATCTGCTAATATGTCAAGAATTG
TAACTTACTCAGATTTTTGGTGGAAAGGTAAATTAGTATTTAAAGCTAAACTAAGAGCCTCTCATACT
TGGAATCCAATTCAACAAATGAGTATTAATGTAGATAACCAATTTAACTATGTACCAAGTAATATTGG
AGGTATGAAAATTGTCTATGAAAAATCTCAACTAGCACCTAGA

'USA_JN867604_Dog_IL_137654_2008'

ATGAGTGATGGAGCAGTTCAACCAGACGGTGGTCAGCCTGCTGTCAGAAATGAAAGAGCTACAGGATC
TGGGAACGGGTCTGGAGGCGGGGGTGGTGGTGGTTCTGGGGGTGTGGGGATTTCTACGGGTACTTTCA
ATAATCAGACGGAATTTAAATTTTTGGAAAACGGATGGGTGGAAATCACAGCAAACTCAAGCAGACTT
GTACATTTAAATATGCCAGAAAGTGAAAATTATAGAAGAGTGGTTGTAAATAATTTGGATAAAACTGC
AGTTAACGGAAACATGGCTTTAGATGATACTCATGCACAAATTGTAACACCTTGGTCATTGGTTGATG
CAAATGCTTGGGGAGTTTGGTTTAATCCAGGAGATTGGCAACTAATTGTTAATACTATGAGTGAGTTG
CATTTAGTTAGTTTTGAACAAGAAATTTTTAATGTTGTTTTAAAGACTGTTTCAGAATCTGCTACTCA
GCCACCAACTAAAGTTTATAATAATGATTTAACTGCATCATTGATGGTTGCATTAGATAGTAATAATA
CTATGCCATTTACTCCAGCAGCTATGAGATCTGAGACATTAGGTTTTTATCCATGGAAACCAACCATA
CCAACTCCATGGAGATATTATTTTCAATGGGATAGAACATTAATACCATCTCATACTGGAACTAGTGG
CACACCAACAAATATATACCATGGTACAGATCCAGATGATGTTCAATTTTATACTATTGAAAATTCTG
TGCCAGTACACTTACTAAGAACAGGTGATGAATTTGCTACAGGAACATTTTTTTTTGATTGTAAACCA
TGTAGACTAACACATACATGGCAAACAAATAGAGCATTGGGCTTACCACCATTTCTAAATTCTTTGCC
TCAAGCTGAAGGAGGTACTAACTTTGGTTATATAGGAGTTCAACAAGATAAAAGACGTGGTGTAACTC
AAATGGGAAATACAAACTATATTACTGAAGCTACTATTATGAGACCAGCTGAGGTTGGTTATAGTGCA
CCATATTATTCTTTTGAGGCGTCTACACAAGGGCCATTTAAAACACCTATTGCAGCAGGACGGGGGGG
AGCGCAAACAGATGAAAATCAAGCAGCAGATGGTGATCCAAGATATGCATTTGGTAGACAACATGGTC
AAAAAACTACAACAACAGGAGAAACACCTGAGAGATTTACATATATAGCACATCAAGATACAGGAAGA
TATCCAGAAGGAGATTGGATTCAAAATATTAACTTTAACCTTCCTGTAACAGATGATAATGTATTGCT
ACCAACAGATCCAATTGGAGGTAAAACAGGAATTAACTATACTAATATATTTAATACTTATGGTCCTT
TAACTGCATTAAATAATGTACCACCAGTTTATCCAAATGGTCAAATTTGGGATAAAGAATTTGATACT
GACTTAAAACCAAGACTTCATGTAAATGCACCATTTGTTTGTCAAAATAATTGCCCTGGTCAATTATT
TGTAAAAGTTGCGCCTAATTTAACAAATGAATATGATCCTGATGCATCTGCTAATATGTCAAGAATTG
TAACTTACTCAGATTTTTGGTGGAAAGGTAAATTAGTATTTAAAGCTAAACTAAGAGCCTCTCATACT

TGGAATCCAATTCAACAAATGAGTATTAATGTAGATAACCAATTTAACTATGTACCAAGTAATATTGG
AGGTATGAAAATTGTCTATGAAAAATCTCAACTAGCACCTAGA

'USA_JN867605_2b_Dog_US_142805_2009'

ATGAGTGATGGAGCAGTTCAACCAGACGGTGGTCAACCTGCTGTCAGAAATGAAAGAGCTACAGGATC
TGGGAACGGGTCTGGAGGCGGGGGTGGTGGTGGTTCTGGGGGTGTGGGGATTTCTACGGGTACTTTCA
ATAATCAGACGGAATTTAAATTTTTGGAAAACGGATGGGTGGAAATCACAGCAAACTCAAGCAGACTT
GTACATTTAAATATGCCAGAAAGTGAAAATTATAGAAGAGTGGTTGTAAATAATTTGGATAAAACTGC
AGTTAACGGAAACATGGCTTTAGATGATACTCATGCACAAATTGTAACACCTTGGTCATTGGTTGATG
CAAATGCTTGGGGAGTTTGGTTTAATCCAGGAGATTGGCAACTAATTGTTAATACTATGAGTGAGTTG
CATTTAGTTAGTTTTGAACAAGAAATTTTTAATGTTGTTTTAAAGACTGTTTCAGAATCTGCTACTCA
GCCACCAACTAAAGTTTATAATAATGATTTAACTGCATCATTGATGGTTGCATTAGATAGTAATAATA
CTATGCCATTTACTCCAGCAGCTATGAGATCTGAGACATTGGGTTTTTATCCATGGAAACCAACCATA
CCAACTCCATGGAGATATTATTTTCAATGGGATAGAACATTAATACCATCTCATACTGGAACTAGTGG
CACACCAACAAATATATACCATGGTACAGATCCAGATGATGTTCAATTTTATACTATTGAAAATTCTG
TGCCAGTACACTTACTAAGAACAGGTGATGAATTTGCTACAGGAACATTTTTTTTTGATTGTAAACCA
TGTAGACTAACACATACATGGCAAACAAATAGAGCATTGGGCTTACCACCATTTCTAAATTCTTTGCC
TCAAGCTGAAGGAGGTACTAACTTTGGTTATATAGGAGTTCAACAAGATAAAAGACGTGGTGTAACTC
AAATGGGAAAAACAAACTATATTACTGAAGCTACTATTATGAGACCAGCTGAGGTTGGTTATAGTGCA
CCATATTATTCTTTTGAGGCGTCTACACAAGGGCCATTTAAAACACCTATTGCAGCAGGACGGGGGGG
AGCGCAAACAGATGAAAATCAAGCAGCAGATGGTGATCCAAGATATGCATTTGGTAGACAACATGGTC
AAAAAACTACCACAACAGGAGAAACACCTGAGAGATTTACATATATAGCACATCAAGATACAGGAAGA
TATCCAGAAGGAGATTGGATTCAAAATATTAACTTTAACCTTCCTGTAACAGATGATAATGTATTGCT
ACCAACAGATCCAATTGGAGGTAAAACAGGAATTAACTATACTAATATATTTAATACTTATGGTCCTT
TAACTGCATTAAATAATGTACCACCAGTTTATCCAAATGGTCAAATTTGGGATAAAGAATTTGATACT
GACTTAAAACCAAGACTTCATGTAAATGCACCATTTGTTTGTCAAAATAATTGTCCTGGTCAATTATT
TGTAAAAGTTGCGCCTAATTTAACAAATGAATATGATCCTGATGCATCTGCTAATATGTCAAGAATTG
TAACTTACTCAGATTTTTGGTGGAAAGGTAAATTAGTATTTAAAGCTAAACTAAGAGCCTCTCATACT
TGGAATCCAATTCAACAAATGAGTATTAATGTAGATAACCAATTTAACTATGTACCAAGTAATATTGG
AGGTATGAAAATTGTATGTGAAAGATCTCAACTAGCACCTAGA

'USA_JN867610_Raccoon_VA_118_A_2007'

ATGAGTGATGGAGCAGTTCAACCAGACGGTGGTCAACCTGCTGTCAGAAATGAAAGAGCTACAGGATC
TGGGAACGGGTCTGGAGGCGGGGGTGGTGGTGGTTCTGGGGGTGTGGGGATTTCTACGGGTACTTTCA
ATAATCAGACGGAATTTAAATTTTTGGAAAACGGATGGGTGGAAATCACAGCAAACTCAAGCAGACTT
GTACATTTAAATATGCCAGAAAGTGAAAATTATAGAAGAGTGGTTGTAAATAATTTGGATAAAACTGC
AGTTAACGGAAACATGGCTTTAGATGATACTCATGCACAAATTGTAACACCTTGGTCATTGGTTGATG
CAAATGCTTGGGGAGTTTGGTTTAATCCAGGAGATTGGCAACTAATTGTTAATACTATGAGTGAGTTG
CATTTAGTTAGTTTTGAACAAGAAATTTTTAATGTTGTTTTAAAGACTGTTTCAGAATCTGCTACTCA
GCCACCAACTAAAGTTTATAATAATGATTTAACTGCATCATTGATGGTTGCATTAGATAGTAATAATA
CTATGCCATTTACTCCAGCAGCTATGAGATCTGAGACATTGGGTTTTTATCCATGGAAACCAACCATA
CCAACTCCATGGAGATATTATTTTCAATGGGATAGAACATTAATACCATCTCATACTAGAACTAGTGG
CACACCAACAAATATATACCATGGTACAGATCCAGATGATGTTCAATTTTATACTATTGAAAATTCTG
TGCCAGTACACTTACTAAGAACAGGTGATGAATTTGCTACAGGAACATTTTTTTTTGATTGTAAACCA
TGTAGACTAACACATACATGGCAAACAAATAGAGCATTGGGCTTACCACCATTTCTAAATTCTTTGCC
TCAAGCTGAAGGAGATACTAACTTTGGTGATATAGGAGTTCAACAAGATAAAAGACGTGGTGTAACTC
AAATGGGAAATACAAACTATATTACTGAAGCTACTATTATGAGACCAGCTGAAGTTGGTTATAGTGCA
CCATATTATTCTTTTGAGGCGTCTACACAAGGGCCATTTAAAACACCTATTGCAGCAGGACGGGGGGG
AGCGCAAACAGATGAAAATCAAGCAGCAGATGGTGATCCAAGATATGCATTTGGTAGACAACATGGTC
AAAAAACTACCACAACAGGAGAAACACCTGAGAGATTTACATATATAGCACATCAAGATACAGGAAGA
TATCCAGAAGGAGATTGGATTCAAAATATTAACTTTAACCTTCCTGTAACAAATGATAATGTATTGCT
ACCAACAGATCCAATTGGAGGTAAAACAGGAATTAACTATACTAATATATTTAATACTTATGGTCCTT
TAACTGCATTAAATAATGTACCACCAGTTTATCCAAATGGTCAAATTTGGGATAAAGAATTTGATACT
GACTTAAAACCAAGACTTCATGTAAATGCACCATTTGTTTGTCAAAATAATTGTCCTGGTCAATTATT
TGTAAAAGTTGCGCCTAATTTAACAAATGAATATGATCCTGATGCATCTGCTAATATGTCAAGAATTG
TAACTTACTCAGATTTTTGGTGGAAAGGTAAATTAGTATTTAAAGCTAAACTAAGAGCCTCTCATACT

TGGAATCCAATTCAACAAATGAGTATTAATGTAGATAACCAATTTAACTATGTACCAAGTAATATTGG
AGGTATGAAAATTGTATATGAAAAATCTCAACTAGCACCTAGA
'USA_JN867611_Raccoon_KY_358_B_2009'
ATGAGTGATGGAGCAGTTCAACCAGACGGTGGTCAACCTGCTGTCAGAAATGAAAGAGCTACAGGATC
TGGGAACGGGTCTGGAGGCGGGGGTGGTGGTGGTTCTGGGGGTGTGGGGATTTCTACGGGTACTTTCA
ATAATCAGACGGAATTTAAATTTTTGGAAAACGGATGGGTGGAAATCACAGCAAACTCAAGCAGACTT
GTACATTTAAATATGCCAGAAAGTGAAAATTATAGAAGAGTGGTTGTAAATAATTTGGATAAAACTGC
AGTTAACGGAAACATGGCTTTAGATGATACTCATGCACAAATTGTAACACCTTGGTCATTGGTTGATG
CAAATGCTTGGGGAGTTTGGTTTAATCCAGGAGATTGGCAACTAATTGTTAATACTATGAGTGAGTTA
CATTTAGTTAGTTTTGAACAAGAAATTTTTAATGTTGTTTTAAAGACTGTTTCAGAATCTGCTACTCA
GCCACCAACTAAAGTTTATAATAATGATTTAACTGCATCATTGATGGTTGCATTAGATAGTAATAATA
CTATGCCATTTACTCCAGCAGCTATGAGATCTGAGACATTGGGTTTTTATCCATGGAAACCAACCATA
CCAACTCCATGGAGATATTATTTTCAATGGGATAGAACATTAATACCATCTCATACTAGAACTAGTGG
CACACCAACAAATATATACCATGGTACAGATCCAGATGATGTTCAATTTTATACTATTGAAAATTCTG
TGCCAGTACACTTACTAAGAACAGGTGATGAATTTGCTACAGGAACATTTTTTTTTGATTGTAAACCA
TGTAGACTAACACATACATGGCAAACAAATAGAGCATTGGGCTTACCACCATTTCTAAATTCTTTGCC
TCAAGCTGAAGGAGATACTAACTTTGGTGATATAGGAGTTCAACAAGATAAAAGACGTGGTGTGACTC
AAATGGGAAATACAAACTATATTACTGAAGCTACTATTATGAGACCAGCTGAGGTTGGTTATAGTGCA
CCATATTATTCTTTTGAGGCGTCTACACAAGGGCCATTTAAAACACCTATTGCAGCAGGACGGGGGGG
AGCGCAAACAGATGAAAATCAAGCAGCAGATGGTGATCCAAGATATGCATTTGGTAGACAACATGGTC
AAAAAACTACCACAACAGGAGAAACACCTGAGAGATTTACATATATAGCACATCAAGATACAGGAAGA
TATCCAGAAGGAGATTGGATTCAAAATATTAACTTTAACCTTCCTGTAACAAATGATAATGTATTGCT
ACCAACAGATCCAATTGGAGGTAAAACAGGAATTAACTATACTAATATATTTAATACTTATGGTCCTT
TAACTGCATTAAATAATGTACCACCAGTTTATCCAAATGGTCAAATTTGGGATAAAGAATTTGATACT
GACTTAAAACCAAGACTTCATGTAAATGCACCATTTGTTTGTCAAAATAATTGTCCTGGTCAATTATT
TGTAAAAGTTGCGCCTAATTTAACAAATGAATATGATCCTGATGCATCTGCTAATATGTCAAGAATTG
TAACTTACTCAGATTTTTGGTGGAAAGGTAAATTAGTATTTAAAGCTAAACTAAGAGCCTCTCATACT
TGGAATCCAATTCAACAAATGAGTATTAATGTAGATAACCAATTTAACTATGTACCAAGTAATATTGG
AGGTATGAAAATTGTATATGAAAAATCTCAACTAGCACCTAGA
'USA_JN867618_Raccoon_WI_37_2010'
ATGAGTGATGGAGCAGTTCAACCAGACGGTGGTCAACCTGCTGTCAGAAATGAAAGAGCTACAGGATC
TGGGAACGGGTCTGGAGGCGGGGGTGGTGGTGGTTCTGGGGGTGTGGGGATTTCTACGGGTACTTTCA
ATAATCAGACGGAATTTAAATTTTTGGAAAACGGATGGGTGGAAATCACAGCAAACTCAAGCAGACTT
GTACATTTAAATATGCCAGAAAGTGAAAATTATAGAAGAGTGGTTGTAAATAATTTAGATAAAACTGC
AGTTAACGGAAACATGGCTTTAGATGATACTCATGCACAAATTGTAACACCTTGGTCATTGGTTGATG
CAAATGCTTGGGGAGTTTGGTTTAATCCAGGAGATTGGCAACTAATTGTTAATACTATGAGTGAGTTG
CATTTAGTTAGTTTTGAACAAGAAATTTTTAATGTTGTTTTAAAGACTGTTTCAGAATCTGCTACTCA
GCCACCAACTAAAGTTTATAATAATGATTTAACTGCATCATTGATGGTTGCATTAGATAGTAATAATA
CTATGCCATTTACTCCAGCAGCTATGAGATCTGAGACATTGGGTTTTTATCCATGGAAACCAACCATA
CCAACTCCATGGAGATATTATTTTCAATGGGATAGAACATTAATACCATCTCATACTGGAACTAGTGG
CACACCAACAAATACATACCATGGTACAGATCCAGATGATGTTCAATTTTATACTATTGAAAATTCTG
TGCCAGTACACTTACTAAGAACAGGTGATGAATTTGCTACAGGAACATTTTTTTTTGATTGTAAACCA
TGTAGACTAACACATACATGGCAAACAAATAGAGCATTGGGCTTACCACCATTTCTAAATTCTTTGCC
TCAATCTGAAGGAGATACTAACTTTGGTGATATAGGAGTTCAACAAGATAAAAGACGTGGTGTAACTC
AAATGGGAAATACAAACTATATTACTGAAGCTACTATTATGAGACCAGCTGAGGTTGGTTATAGTGCA
CCATATTATTCTTTTGAGGCGTCTACACAAGGGCCATTTAAAACACCTATTGCAGCAGGACGGGGGGG
AGCGCAAACAGATGAAAATCAAGCAGCAGATGGTGATCCAAGATATGCATTTGGTAGACAACATGGTC
AAAAAACTACCACAACAGGAGAAACACCTGAGAGATTTACATATATAGCACATCAAGATACAGGAAGA
TATCCAGAAGGAGATTGGATTCAAAATATTAACTTTAACCTTCCTGTAACAAATGATAATGTATTGCT
ACCAACAGATCCAATTGGAGGTAAAACAGGAATTAACTATACTAATATATTTAATACTTATGGTCCTT
TAACTGCATTAAATAATGTACCACCAGTTTATCCAAATGGTCAAATTTGGGATAAAGAATTTGATACT
GACTTAAAACCAAGACTTCATGTAAATGCACCATTTGTTTGTCAAAATAATTGTCCTGGTCAATTATT
TGTAAAAGTTGCGCCTAATTTAACAAATGAATATGATCCTGATGCATCTGCTAATATGTCAAGAATTG
TAACTTACTCAGATTTTTGGTGGAAAGGTAAATTAGTATTTAAAGCTAAACTAAGAGCCTCTCATACT

TGGAATCCAATTCAACAAATGAGTATTAATGTAGATAACCAATTTAACTATGTACCAAGTAATATTGG
AGGTATGAAAATTGTATATGAAAAATCTCAACTAGCACCTAGA

'USA_JX475231_CO_280_2011'

ATGAGTGATGGAGCAGTTCAACCAGACGGTGGTCAACCTGCTGTCAGAAATGAAAGAGCTACAGGATC
TGGGAACGGGTCTGGAGGCGGGGGTGGTGGTGGTTCTGGGGGTGTGGGGATTTCTACGGGTACTTTCA
ATAATCAGACGGAATTTAAATTTTTGGAAAACGGATGGGTGGAAATCACAGCAAACTCAAGCAGACTT
GTACATTTAAATATGCCAGAAAGTGAAAATTATAGAAGAGTGGTTGTAAATAATTTAGATAAAACTGC
AGTTAACGGAAACATGGCTTTAGATGATACTCATGCACAAATTGTAACACCTTGGTCATTGGTTGATG
CAAATGCTTGGGGAGTTTGGTTTAATCCAGGAGATTGGCAACTAATTGTTAATACTATGAGTGAGTTG
CATTTAGTTAGTTTTGAACAAGAAATTTTTAATGTTGTTTTAAAGACTGTTTCAGAATCTGCTACTCA
GCCACCAACTAAAGTTTATAATAATGATTTAACTGCATCATTGATGGTTGCATTAGATAGTAATAATA
CTATGCCATTTACTCCAGCAGCTATGAGATCTGAGACATTGGGTTTTTATCCATGGAAACCAACCATA
CCAACTCCATGGAGATATTATTTTCAATGGGATAGAACATTAATACCATCTCATACTGGAACTAGTGG
CACACCAACAAATACATACCATGGTACAGATCCAGATGATGTTCAATTTTATACTATTGAAAATTCTG
TGCCAGTACACTTACTAAGAACAGGTGATGAATTTGCTACAGGAACATTTTTTTTTGATTGTAAACCA
TGTAGACTAACACATACATGGCAAACAAATAGAGCATTGGGCTTACCACCATTTCTAAATTCTTTGCC
TCAATCTGAAGGAGATACTAACTTTGGTGATATAGGAGTTCAACAAGATAAAAGACGTGGTGTAACTC
AAATGGGAAATACAAACTATATTACTGAAGCTACTATTATGAGACCAGCTGAGGTTGGTTATAGTGCA
CCATATTATTCTTTTGAGGCGTCTACACAAGGGCCATTTAAAACACCTATTGCAGCAGGACGGGGGGG
AGCGCAAACAGATGAAAATCAAGCAGCAGATGGTGATCCAAGATATGCATTTGGTAGACAACATGGTC
AAAAAACTACCACAACAGGAGAAACACCTGAGAGATTTACATATATAGCACATCAAGATACAGGAAGA
TATCCAGAAGGAGATTGGATTCAAAATATTAACTTTAACCTTCCTGTAACAAATGATAATGTATTGCT
ACCAACAGATCCAATTGGAGGTAAAACAGGAATTAACTATACTAATATATTTAATACTTATGGTCCTT
TAACTGCATTAAATAATGTACCACCAGTTTATCCAAATGGTCAAATTTGGGATAAAGAATTTGATACT
GACTTAAAACCAAGACTTCATGTAAATGCACCATTTGTTTGTCAAAATAATTGTCCTGGTCAATTATT
TGTAAAAGTTGCGCCTAATTTAACAAATGAATATGATCCTGATGCATCTGCTAATATGTCAAGAATTG
TAACTTACTCAGATTTTTGGTGGAAAGGTAAATTAGTATTTAAAGCTAAACTAAGAGCCTCTCATACT
TGGAATCCAATTCAACAAATGAGTATTAATGTAGATAACCAATTTAACTATGTACCAAGTAATATTGG
AGGTATGAAAATTGTATATGAAAAATCTCAACTAGCACCTAGA

'USA_JX475233_SC_182_A_2011'

ATGAGTGATGGAGCAGTTCAACCAGACGGTGGTCAACCTGCTGTCAGAAATGAAAGAGCTACAGGATC
TGGGAACGGGTCTGGAGGCGGGGGTGGTGGTGGTTCTGGGGGTGTGGGGATTTCTACGGGTACTTTCA
ATAATCAGACGGAATTTAAATTTTTGGAAAACGGATGGGTGGAAATCACAGCAAACTCAAGCAGACTT
GTACATTTAAATATGCCAGAAAGTGAAAATTATAGAAGAGTGGTTGTAAATAATTTAGATAAAACTGC
AGTTAACGGAAACATGGCTTTAGATGATACTCATGCACAAATTGTAACACCTTGGTCATTGGTTGATG
CAAATGCTTGGGGAGTTTGGTTTAATCCAGGAGATTGGCAACTAATTGTTAATACTATGAGTGAGTTG
CATTTAGTTAGTTTTGAACAAGAAATTTTTAATGTTGTTTTAAAGACTGTTTCAGAATCTGCTACTCA
GCCACCAACTAAAGTTTATAATAATGATTTAACTGCATCATTGATGGTTGCATTAGATAGTAATAATA
CTATGCCATTTACTCCAGCAGCTATGAGATCTGAGACATTGGGTTTTTATCCATGGAAACCAACCATA
CCAACTCCATGGAGATATTATTTTCAATGGGATAGAACATTAATACCATCTCATACTGGAACTAGTGG
CACACCAACAAATACATACCATGGTACAGATCCAGATGATGTTCAATTTTATACTATTGAAAATTCTG
TGCCAGTACACTTACTAAGAACAGGTGATGAATTTGCTACAGGAACATTTTTTTTTGATTGTAAACCA
TGTAGACTAACACATACATGGCAAACAAATAGAGCATTGGGCTTACCACCATTTCTAAATTCTTTGCC
TCAATCTGAAGGAGATACTAACTTTGGTGATATAGGGGTTCAACAAGATAAAAGACGTGGTGTAACTC
AAATGGGAAATACAAACTATATTACTGAAGCTACTATTATGAGACCAGCTGAGGTTGGTTATAGTGCA
CCATATTATTCTTTTGAGGCGTCTACACAAGGGCCATTTAAAACACCTATTGCAGCAGGACGGGGGGG
AGCGCAAACAGATGAAAATCAAGCAGCAGATGGTGATCCAAGATATGCATTTGGTAGACAACATGGTC
AAAAAACTACCACAACAGGAGAAACACCTGAGAGATTTACATATATAGCACATCAAGATACAGGAAGA
TATCCAGAAGGAGATTGGATTCAAAATATTAACTTTAACCTTCCTGTAACAAATGATAATGTATTGCT
ACCAACAGATCCAATTGGAGGTAAAACAGGAATTAACTATACTAATATATTTAATACTTATGGTCCTT
TAACTGCATTAAATAATGTACCACCAGTTTATCCAAATGGTCAAATTTGGGATAAAGAATTTGATACT
GACTTAAAACCAAGACTTCATGTAAATGCACCATTTGTTTGTCAAAATAATTGTCCTGGTCAATTATT
TGTAAAAGTTGCGCCTAATTTAACAAATGAATATGATCCTGATGCATCTGCTAATATGTCAAGAATTG
TAACTTACTCAGATTTTTGGTGGAAAGGTAAATTAGTATTTAAAGCTAAACTAAGAGCCTCTCATACT

TGGAATCCAATTCAACAAATGAGTATTAATGTAGATAACCAATTTAACTATGTACCAAGTAATATTGG
AGGTATGAAAATTGTATATGAAAAATCTCAACTAGCACCTAGA
'USA_JX475234_ME_258_2011'
ATGAGTGATGGAGCAGTTCAACCAGACGGTGGTCAACCTGCTGTCAGAAATGAAAGAGCTACAGGATC
TGGGAACGGGTCTGGAGGCGGGGGTGGTGGTGGTTCTGGGGGTGTGGGGATTTCTACGGGTACTTTCA
ATAATCAGACGGAATTTAAATTTTTGGAAAACGGATGGGTGGAGATCACAGCAAACTCAAGCAGACTT
GTACATTTAAATATGCCAGAAAGTGAAAATTATAGAAGAGTGGTTGTAAATAATTTAGATAAAACTGC
AGTTAACGGAAACATGGCTTTAGATGATACTCATGCACAAATTGTAACACCTTGGTCATTGGTTGATG
CAAATGCTTGGGGAGTTTGGTTTAATCCAGGAGATTGGCAACTAATTGTTAATACTATGAGTGAGTTG
CATTTAGTTAGTTTTGAACAAGAAATTTTTAATGTTGTTTTAAAGACTGTTTCAGAATCTGCTACTCA
GCCACCAACTAAAGTTTATAATAATGATTTAACTGCATCATTGATGGTTGCATTAGATAGTAATAATA
CTATGCCATTTACTCCAGCAGCTATGAGATCTGAGACATTGGGTTTTTATCCATGGAAACCAACCATA
CCAACTCCATGGAGATATTATTTTCAATGGGATAGAACATTAATACCATCTCATACTGGAACTAGTGG
CACACCAACAAATACATACCATGGTACAGATCCAGATGATGTTCAATTTTATACTATTGAAAATTCTG
TGCCAGTACACTTACTAAGAACAGGTGATGAATTTGCTACAGGAACATTTTTTTTTGATTGTAAACCA
TGTAGACTAACACATACATGGCAAACAAATAGAGCATTGGGCTTACCACCATTTCTAAATTCTTTGCC
TCAATCTGAAGGAGATACTAACTTTGGTGATATAGGAGTTCAACAAGATAAAAGACGTGGTGTAACTC
AAATGGGAAATACAAACTATATTACTGAAGCTACTATTATGAGACCAGCTGAGGTTGGTTATAGTGCA
CCATATTATTCTTTTGAGGCGTCTACACAAGGGCCATTTAAAACACCTATTGCAGCAGGACGGGGGGG
AGCGCAAACAGATGAAAATCAAGCAGCAGATGGTGATCCAAGATATGCATTTGGTAGACAACATGGTC
AAAAAACTACCACAACAGGAGAAACACCTGAGAGATTTACATATATAGCACATCAAGATACAGGAAGA
TATCCAGAAGGAGATTGGATTCAAAATATTAACTTTAACCTTCCTGTAACAAATGATAATGTATTGCT
ACCAACAGATCCAATTGGAGGTAAAACAGGAATTAACTATACTAATATATTTAATACTTATGGTCCTT
TAACTGCATTAAATAATGTACCACCAGTTTATCCAAATGGTCAAATTTGGGATAAAGAATTTGATACT
GACTTAAAACCAAGACTTCATGTAAATGCACCATTTGTTTGTCAAAATAATTGTCCTGGTCAATTATT
TGTAAAAGTTGCGCCTAATTTAACAAATGAATATGATCCTGATGCATCTGCTAATATGTCAAGAATTG
TAACTTACTCAGATTTTTGGTGGAAAGGTAAATTAGTATTTAAAGCTAAACTAAGAGCCTCTCATACT
TGGAATCCAATTCAACAAATGAGTATTAATGTAGATAACCAATTTAACTATGTACCAAGTAATATTGG
AGGTATGAAAATTGTATATGAAAAATCTCAACTAGCACCTAGA
'USA_JX475237_CT_372_2011'
ATGAGTGATGGAGCAGTACAACCAGACGGTGGTCAACCTGCTGTCAGAAATGAAAGAGCTACAGGATC
TGGGAACGGGTCTGGAGGCGGGGGTGGTGGTGGTTCTGGGGGTGTGGGGATTTCTACGGGTACTTTCA
ATAATCAGACGGAATTTAAATTTTTGGAAAACGGATGGGTGGAAATCACAGCAAACTCAAGCAGACTT
GTACATTTAAATATGCCAGAAAGTGAAAATTATAGAAGAGTGGTTGTAAATAATTTGGATAAAACTGC
AGTTAACGGAAACATGGCTTTAGATGATACCCATGCACAAATTGTAACACCTTGGTCATTGGTTGATG
CAAATGCTTGGGGAGTTTGGTTTAATCCAGGAGATTGGCAACTAATTGTTAATACTATGAGTGAGTTG
CATTTAGTTAGTTTTGAACAAGAAATTTTTAATGTTGTTTTAAAGACTGTTTCAGAATCTGCTACTCA
GCCACCAACTAAAGTTTATAATAATGATTTAACTGCATCATTGATGGTTGCATTAGATAGTAATAATA
CTATGCCATTTACTCCAGCAGCTATGAGATCTGAGACATTGGGTTTTTATCCATGGAAACCAACCATA
CCAACTCCATGGAGATATTATTTTCAATGGGATAGAACATTAATACCATCTCATACTGGAACTAGTGG
CACACCAACAAATATATACCATGGTACAGATCCAGATGATGTTCAATTTTATACTATTGAAAATTCTG
TGCCAGTACACTTACTAAGAACAGGTGATGAATTTGCTACAGGAACATTTTTTTTTGATTGTAAACCA
TGTAGACTAACACATACATGGCAAACAAATAGAGCATTGGGCTTACCACCATTTCTAAATTCTTTGCC
TCAAGCTGAAGGAGGTACTAACTTTGGTTATATAGGAGTTCAACAAGATAAAAGACGTGGTGTAACTC
AAATGGGAAATACAAACTATATTACTGAAGCTACTATTATGAGACCAGCTGAGGTTGGTTATAGTGCA
CCATATTATTCTTTTGAGGCGTCTACACAAGGGCCATTTAAAACACCTATTGCAGCAGGACGGGGGGG
AGCGCAAACAGATGAAAATCAAGCAGCAGATGGTGATCCAAGATATGCATTTGGTAGACAACATGGTC
AAAAAACTACCACAACAGGAGAAACACCTGAGAGATTTACATATATAGCACATCAAGATACAGGAAGA
TATCCAGAAGGAGATTGGATTCAAAATATTAACTTTAACCTTCCTGTAACAGATGATAATGTATTGCT
ACCAACAGATCCAATTGGAGGTAAAACAGGAATTAACTATACTAATATATTTAATACTTATGGTCCTT
TAACTGCATTAAATAATGTACCACCAGTTTATCCAAATGGTCAAATTTGGGATAAAGAATTTGATACT
GACTTAAAACCAAGACTTCATGTAAATGCACCATTTGTTTGTCAAAATAATTGTCCTGGTCAATTATT
TGTAAAAGTTGCGCCTAATTTAACAAATGAATATGATCCTGATGCATCTGCTAATATGTCAAGAATTG
TAACTTACTCAGATTTTTGGTGGAAAGGTAAATTAGTATTTAAAGCTAAACTAAGAGCCTCTCATACT

TGGAATCCAATTCAACAAATGAGTATTAATGTAGATAACCAATTTAACTATGTACCAAGTAATATTGG
AGGTATGAAAATTGTATATGAAAAATCTCAACTAGCACCTAGA
'USA_JX475239_GA_06_2011'
ATGAGTGATGGAGCAGTTCAACCAGACGGTGGTCAACCTGCTGTCAGAAATGAAAGAGCTACAGGATC
TGGGAACGGGTCTGGAGGCGGGGGTGGTGGTGGTTCTGGGGGTGTGGGGATTTCTACGGGTACTTTCA
ATAATCAGACGGAATTTAAATTTTTGGAAAACGGATGGGTGGAAATCACAGCAAACTCAAGCAGACTT
GTACATTTAAATATGCCAGAAAGTGAAAATTATAGAAGAGTGGTTGTAAATAATTTGGATAAAACTGC
AGTTAACGGAAACATGGCTTTAGATGATACTCATGCACAAATTGTAACACCTTGGTCATTGGTTGATG
CAAATGCTTGGGGAGTTTGGTTTAATCCAGGAGATTGGCAACTAATTGTTAATACTATGAGTGAGTTG
CATTTAGTTAGTTTTGAACAAGAAATTTTTAATGTTGTTTTAAAGACTGTTTCAGAATCTGCTACTCA
GCCACCAACTAAAGTTTATAATAATGATTTAACTGCATCATTGATGGTTGCATTAGATAGTAATAATA
CTATGCCATTTACTCCAGCAGCTATGAGATCTGAGACATTGGGTTTTTATCCATGGAAACCAACCATA
CCAACTCCATGGAGATATTATTTTCAATGGGATAGAACATTAATACCATCTCATACTAGAACTAGTGG
CACACCAACAAATATATACCATGGTACAGATCCAGATGATGTTCAATTTTATACTATTGAAAATTCTG
TGCCAGTACACTTACTAAGAACAGGTGATGAATTTGCTACAGGAACATTTTTTTTTGATTGTAAACCA
TGTAGACTAACACATACATGGCAAACAAATAGAGCATTGGGCTTACCACCATTTCTAAATTCTTTGCC
TCAAGCTGAAGGAGATACTAACTTTGGTGATATAGGAGTTCAACAAGATAAAAGACGTGGTGTAACTC
AAATGGGAAATACAAACTATATTACTGAAGCTACTATTATGAGACCAGCTGAGGTTGGTTATAGTGCA
CCATATTATTCTTTTGAGGCGTCTACACAAGGGCCATTTAAAACACCTATTGCAGCAGGACGGGGGGG
AGCGCAAACAGATGAAAATCAAGCAGCAGATGGTGATCCAAGATATGCATTTGGTAGACAACATGGTC
AAAAAACTACCACAACAGGAGAAACACCTGAGAGATTTACATATATAGCACATCAAGATACAGGAAGA
TATCCAGAAGGAGACTGGATTCAAAATATTAACTTTAACCTTCCTGTAACAAATGATAATGTATTGCT
ACCAACAGATCCAATTGGAGGTAAAACAGGAATTAACTATACTAATATATTTAATACTTATGGTCCTT
TAACTGCATTAAATAATGTACCACCAGTTTATCCAAATGGTCAAATTTGGGATAAAGAATTTGATACT
GACTTAAAACCAAGACTTCATGTAAATGCACCATTTGTTTGTCAAAATAATTGTCCTGGTCAATTATT
TGTAAAAGTTGCGCCTAATTTAACAAATGAATATGATCCTGATGCATCTGCTAATATGTCAAGAATTG
TAACTTACTCAGATTTTTGGTGGAAAGGTAAATTAGTATTTAAAGCTAAACTAAGAGCCTCTCATACT
TGGAATCCAATTCAACAAATGAGTATTAATGTAGATAACCAATTTAACTATGTACCAAGTAATATTGG
AGGTATGAAAATTGTATATGAAAAATCTCAACTAGCACCTAGA
'USA_JX475240_AZ_16382_01_1999'
ATGAGTGATGGAGCAGTTCAACCAGACGGTGGTCAACCTGCTGTCAGAAATGAAAGAGCAACAGGATC
TGGGAACGGGTCTGGAGGCGGGGGTGGTGGTGGTTCTGGGGGTGTGGGGATTTCTACGGGTACTTTCA
ATAATCAGACGGAATTTAAATTTTTGGAAAACGGATGGGTGGAAATCACAGCAAACTCAAGCAGACTT
GTACATTTAAATATGCCAGAAAGTGAAAATTATAGAAGAGTGGTTGTAAATAATTTGGATAAAACTGC
AGTTAACGGAAACATGGCTTTAGATGATACTCATGCACAAATTGTAACACCTTGGTCATTGGTTGATG
CAAATGCTTGGGGAGTTTGGTTTAATCCAGGAGATTGGCAACTAATTGTTAATACTATGAGTGAGTTG
CATTTAGTTAGTTTTGAACAAGAAATTTTTAATGTTGTTTTAAAGACTGTTTCAGAATCTGCTACTCA
GCCACCAACTAAAGTTTATAATAATGATTTAACTGCATCATTGATGGTTGCATTAGATAGCAATAATA
CTATGCCATTTACTCCAGCAGCTATGAGATCTGAGACATTGGGTTTTTATCCATGGAAACCAACCATA
CCAACTCCATGGAGATATTATTTTCAATGGGATAGAACATTAATACCATCTCATACTGGAACTAGTGG
CACACCAACAAATATATACCATGGTACAGATCCAGATGATGTTCAATTTTATACTATTGAAAATTCTG
TGCCAGTACACTTACTAAGAACAGGTGATGAATTTGCTACAGGAACATTTTTTTTTGATTGTAAACCA
TGTAGACTAACACATACATGGCAAACAAATAGAGCATTGGGCTTACCACCATTTCTAAATTCTTTGCC
TCAAGCTGAAGGAGGTACTAACTTTGGTTATATAGGAGTTCAACAAGATAAAAGACGTGGTGTAACTC
AAATGGGAAATACAAACTATATTACTGAAGCTACTATTATGAGACCAGCTGAGGTTGGTTATAGTGCA
CCATATTATTCTTTTGAGGCGTCTACACAAGGGCCATTTAAAACACCTATTGCAGCAGGACGGGGGGG
AGCGCAAACAGATGAAAATCAAGCAGCAGATGGTGATCCAAGATATGCATTTGGTAGACAACATGGTC
AAAAGACTACCACAACAGGAGAAACACCTGAGAGATTTACATATATAGCACATCAAGATACAGGAAGA
TATCCAGAAGGAGATTGGATTCAAAATATTAACTTTAACCTTCCTGTAACAGATGATAATGTATTGCT
ACCAACAGATCCAATTGGAGGTAAAACAGGAATTAACTATACTAATATATTTAATACTTATGGTCCTT
TAACTGCATTAAATAATGTACCACCAGTTTATCCAAATGGTCAAATTTGGGATAAAGAATTTGATACT
GACTTAAAACCAAGACTTCATGTAAATGCACCATTTGTTTGTCAAAATAATTGTCCTGGTCAATTATT
TGTAAAAGTTGCGCCTAATTTAACAAATGAATATGATCCTGATGCATCTGCTAATATGTCAAGAATTG
TAACTTACTCAGATTTTTGGTGGAAAGGTAAATTAGTATTTAAAGCTAAACTAAGAGCCTCTCATACT

TGGAATCCAATTCAACAAATGAGTATTAATGTAGATAACCAATTTAACTATGTACCAAGTAATATTGG
AGGTATGAAAATTGTATATGAAAAATCTCAACTAGCACCAAGA
'USA_JX475242_WI_18268_2002'
ATGAGTGATGGAGCAGTTCAACCAGACGGTGGTCAGCCTGCTGTCAGAAATGAAAGAGCTACAGGATC
TGGGAACGGGTCTGGAGGCGGGGGTGGTGGTGGTTCTGGGGGTGTGGGGATTTCTACGGGTACTTTCA
ATAATCAGACGGAATTTAAATTTTTGGAAAACGGATGGGTGGAAATCACAGCAAACTCAAGCAGACTT
GTACATTTAAATATGCCAGAAAGTGAAAATTATAGAAGAGTGGTTGTAAATAATTTGGATAAAACTGC
AGTTAACGGAAACATGGCTTTAGATGATACTCATGCACAAATTGTAACACCTTGGTCATTGGTTGATG
CAAATGCTTGGGGAGTTTGGTTTAATCCAGGAGATTGGCAACTAATTGTTAATACTATGAGTGAGTTG
CATTTAGTTAGTTTTGAACAAGAAATTTTTAATGTTGTTTTAAAGACTGTTTCAGAATCTGCTACTCA
GCCACCAACTAAAGTTTATAATAATGATTTAACTGCATCATTGATGGTTGCATTAGATAGTAATAATA
CTATGCCATTTACTCCAGCAGCTATGAGATCTGAGACATTGGGTTTTTATCCATGGAAACCAACCATA
CCAACTCCATGGAGATATTATTTTCAATGGGATAGAACATTAATACCATCTCATACTGGAACTAGTGG
CACACCAACAAATATATACCATGGGACAGATCCAGATGATGTTCAATTTTATACTATTGAAAATTCTG
TGCCAGTACACTTACTAAGAACAGGTGATGAATTTGCTACAGGAACATTTTTTTTTGATTGTAAACCA
TGTAGACTAACACATACATGGCAAACAAATAGAGCATTGGGCTTACCACCATTTCTAAATTCTTTGCC
TCAAGCTGAAGGAGGTACTAACTTTGGTTATATAGGAGTTCAACAAGATAAAAGACGTGGTGTAACTC
AAATGGGAAATACAAACTATATTACTGAAGCTACTATTATGAGACCAGCTGAGGTTGGTTATAGTGCA
CCATATTATTCTTTTGAGGCGTCTACACAAGGGCCATTTAAAACACCTATTGCAGCAGGACGGGGGGG
AGCGCAAACAGATGAAAATCAAGCAGCAGATGGTGATCCAAGATATGCATTTGGTAGACAACATGGTC
AAAAAACTACAACAACAGGAGAAACACCTGAGAGATTTACATATATAGCACATCAAGATACAGGAAGA
TATCCAGAAGGAGATTGGATTCAAAATATTAACTTTAACCTTCCTGTAACAGATGATAATGTATTGCT
ACCAACAGATCCAATTGGAGGTAAAACAGGAATTAACTATACTAATATATTTAATACTTATGGTCCTT
TAACTGCATTAAATAATGTACCACCAGTTTATCCAAATGGTCAAATTTGGGATAAAGAATTTGATACT
GACTTAAAACCAAGACTTCATGTAAATGCACCATTTGTTTGTCAAAATAATTGCCCTGGTCAATTATT
TGTAAAAGTTGCGCCTAATTTAACAAATGAATATGATCCTGATGCATCTGCTAATATGTCAAGAATTG
TAACTTACTCAGATTTTTGGTGGAAAGGTAAATTAGTATTTAAAGCTAAACTAAGAGCCTCTCATACT
TGGAATCCAATTCAACAAATGAGTATTAATGTAGATAACCAATTTAACTATGTACCAAGTAATATTGG
AGGTATGAAAATTGTCTATGAAAAATCTCAACTAGCACCTAGA
'USA_JX475243_ID_22772_2009'
ATGAGTGATGGAGCAGTTCAACCAGACGGTGGTCAACCTGCTGTCAGAAATGAAAGAGCAACAGGATC
TGGGAACGGGTCTGGAGGCGGGGGTGGTGGTGGTTCTGGGGGTGTGGGGATTTCTACGGGTACTTTCA
ATAATCAGACGGAATTTAAATTTTTGGAAAACGGATGGGTGGAAATCACAGCAAACTCAAGCAGACTT
GTACATTTAAATATGCCAGAAAGTGAAAATTATAGAAGAGTGGTTGTAAATAATTTGGATAAAACTGC
AGTTAACGGAAACATGGCTTTAGATGATACTCATGCACAAATTGTAACACCTTGGTCATTGGTTGATG
CAAATGCTTGGGGAGTTTGGTTTAATCCAGGAGATTGGCAACTAATTGTTAATACTATGAGTGAGTTG
CATTTAGTTAGTTTTGAACAAGAAATTTTTAATGTTGTTTTAAAGACTGTTTCAGAATCTGCTACTCA
GCCACCAACTAAAGTTTATAATAATGATTTAACTGCATCATTGATGGTTGCATTAGATAGTAATAATA
CTATGCCATTTACTCCAGCAGCTATGAGATCTGAGACATTGGGTTTTTATCCATGGAAACCAACCATA
CCAACTCCATGGAGATATTATTTTCAATGGGATAGAACATTAATACCATCTCATACTGGAACTAGTGG
CACACCAACAAATATATACCATGGTACAGATCCAGATGATGTTCAATTCTATACTATTGAAAATTCTG
TGCCAGTACACTTACTAAGAACAGGTGATGAATTTGCTACAGGAACATTTTTTTTTGATTGTAAACCA
TGTAGACTAACACATACATGGCAAACAAATAGAGCATTGGGCTTACCACCATTTCTAAATTCTTTGCC
TCAAGCTGAAGGAGGTACTAACTTTGGTTATATAGGAGTTCAACAAGATAAAAGACGTGGTGTAACTC
AAATGGGAAATACAAACTATATTACTGAAGCTACTATTATGAGACCAGCTGAGGTTGGTTATAGTGCA
CCATATTATTCTTTTGAGGCGTCTACACAAGGGCCATTTAAAACACCTATTGCAGCAGGACGGGGGGG
AGCGCAAACAGATGAAAATCAAGCAGCAGATGGTGATCCAAGATATGCATTTGGTAGACAACATGGTC
AAAAAACTACCACAACAGGAGAAACACCTGAGAGATTTACATATATAGCACATCAAGATACAGGAAGA
TATCCAGAAGGAGATTGGATTCAAAATATTAACTTTAACCTTCCTGTAACAGAAGATAATGTATTGCT
ACCAACAGATCCAATTGGAGGTAAAACAGGAATTAACTATACTAATATATTTAATACTTATGGTCCTT
TAACTGCATTAAATAATGTACCACCAGTTTATCCAAATGGTCAAATTTGGGATAAAGAATTTGATACT
GACTTAAAACCAAGACTTCATGTAAATGCACCATTTGTTTGTCAAAATAATTGTCCTGGTCAATTATT
TGTAAAAGTTGCGCCTAATTTAACAAATGAATATGATCCTGATGCATCTGCTAATATGTCAAGAATTG
TAACTTACTCAGATTTTTGGTGGAAAGGTAAATTAGTATTTAAAGCTAAACTAAGAGCCTCTCATACT

TGGAATCCAATTCAACAAATGAGTATTAATGTAGATAACCAATTTAACTATGTACCAAGTAATATTGG
AGGTATGAAAATTGTATATGAAAAATCTCAACTAGCACCTAGA

'USA_JX475246_CO_2503_2010'

ATGAGTGATGGAGCAGTTCAACCAGACGGTGGTCAACCTGCTGTCAGAAATGAAAGAGCTACAGGATC
TGGGAACGGGTCTGGAGGCGGGGGTGGTGGTGGTTCTGGGGGTGTGGGGATTTCTACGGGTACTTTCA
ATAATCAGACGGAATTTAAATTTTTGGAAAACGGATGGGTGGAAATCACAGCAAACTCAAGCAGACTT
GTACATTTAAATATGCCAGAAAGTGAAAATTATAGAAGAGTGGTTGTAAATAATTTAGATAAAACTGC
AGTTAACGGAAACATGGCTTTAGATGATACTCATGCACAAATTGTAACACCTTGGTCATTGGTTGATG
CAAATGCTTGGGGAGTTTGGTTTAATCCAGGAGATTGGCAACTAATTGTTAATACTATGAGTGAGTTG
CATTTAGTTAGTTTTGAACAAGAAATTTTTAATGTTGTTTTAAAGACTGTTTCAGAATCTGCTACTCA
GCCACCAACTAAAGTTTATAATAATGATTTAACTGCATCATTGATGGTTGCATTAGATAGTAATAATA
CTATGCCATTTACTCCAGCAGCTATGAGATCTGAGACATTGGGTTTTTATCCATGGAAACCAACCATA
CCAACTCCATGGAGATATTATTTTCAATGGGATAGAACATTAATACCATCTCATACTGGAACTAGTGG
CACACCAACAAATACATACCATGGTACAGATCCAGATGATGTTCAATTTTATACTATTGAAAATTCTG
TGCCAGTACACTTACTAAGAACAGGTGATGAATTTGCTACAGGAACATTTTTTTTTGATTGTAAACCA
TGTAGACTAACACATACATGGCAAACAAATAGAGCATTGGGCTTACCACCATTTCTAAATTCTTTGCC
TCAATCTGAAGGAGATACTAACTTTGGTGATATAGGAGTTCAACAAGATAAAAGACGTGGTGTAACTC
AAATGGGAAATACAAACTATATTACTGAAGCTACTATTATGAGACCAGCTGAGGTTGGTTATAGTGCA
CCATATTATTCTTTTGAGGCGTCTACACAAGGGCCATTTAAAACACCTATTGCAGCAGGACGGGGGGG
AGCGCAAACAGATGAAAATCAAGCAGCAGATGGTGATCCAAGATATGCATTTGGTAGACAACATGGTC
AAAAAACTACCACAACAGGAGAAACACCTGAGAGATTTACATATATAGCACATCAAGATACAGGAAGA
TATCCAGAAGGAGATTGGATTCAAAATATTAACTTTAACCTTCCTGTAACAAATGATAATGTATTGCT
ACCAACAGATCCAATTGGAGGTAAAACAGGAATTAACTATACTAATATATTTAATACTTATGGTCCTT
TAACTGCATTAAATAATGTACCACCAGTTTATCCAAATGGTCAAATTTGGGATAAAGAATTTGATACT
GACTTAAAACCAAGACTCCATGTAAATGCACCATTTGTTTGTCAAAATAATTGTCCTGGTCAATTATT
TGTAAAAGTTGCGCCTAATTTAACAAATGAATATGATCCTGATGCATCTGCTAATATGTCAAGAATTG
TAACTTACTCAGATTTTTGGTGGAAAGGTAAATTAGTATTTAAAGCTAAACTAAGAGCCTCTCATACT
TGGAATCCAATTCAACAAATGAGTATTAATGTAGATAACCAATTTAACTATGTACCAAGTAATATTGG
AGGTATGAAAATTGTATATGAAAAATCTCAACTAGCACCTAGA

'USA_JX475247_CO_1246_2010'

ATGAGTGATGGAGCAGTTCAACCAGACGGTGGTCAGCCTGCTGTCAGAAATGAAAGAGCTACAGGATC
TGGGAACGGGTCTGGAGGCGGGGGTGGTGGTGGTTCTGGGGGTGTGGGGATTTCTACGGGTACTTTCA
ATAATCAGACGGAATTTAAATTTTTGGAAAACGGATGGGTGGAAATCACAGCAAACTCAAGCAGACTT
GTACATTTAAATATGCCAGAAAGTGAAAATTATAGAAGAGTGGTTGTAAATAATTTGGATAAAACTGC
AGTTAACGGAAACATGGCTTTAGATGATACTCATGCACAAATTGTAACACCTTGGTCATTGGTTGATG
CAAATGCTTGGGGAGTTTGGTTTAATCCAGGAGATTGGCAACTAATTGTTAATACTATGAGTGAGTTG
CATTTAGTTAGTTTTGAACAAGAAATTTTTAATGTTGTTTTAAAGACTGTTTCAGAATCTGCTACTCA
GCCACCAACTAAAGTTTATAATAATGATTTAACTGCATCATTGATGGTTGCATTAGATAGTAATAATA
CTATGCCATTTACTCCAGCAGCTATGAGATCTGAGACATTGGGTTTTTATCCATGGAAACCAACCATA
CCAACTCCATGGAGATATTATTTTCAATGGGATAGAACATTAATACCATCTCATACTGGAACTAGTGG
CACACCAACAAATATATACCATGGTACAGATCCAGATGATGTTCAATTTTACACTATTGAAAATTCTG
TGCCAGTACACTTACTAAGAACAGGTGATGAATTTGCTACAGGAACATTTTTTTTTGATTGTAAACCA
TGTAGACTAACACATACATGGCAAACAAATAGAGCATTGGGCTTACCACCATTTCTAAATTCTTTGCC
TCAAGCTGAAGGAGGTACTAACTTTGGTTATATAGGAGTTCAACAAGATAAAAGACGTGGTGTAACTC
AAATGGGAAATACAAACTATATTACTGAAGCTACTATTATGAGACCAGCTGAGGTTGGTTATAGTGCA
CCATATTATTCTTTTGAGGCGTCTACACAAGGGCCATTTAAAACACCTATTGCAGCAGGACGGGGGGG
AGCGCAAACAGATGAAAATCAAGCAGCAGATGGTGATCCAAGATATGCATTTGGTAGACAACATGGTC
AAAAAACTACCACAACAGGAGAAACACCTGAGAGATTTACATATATAGCACATCAAGATACAGGAAGA
TATCCAGAAGGAGATTGGATTCAAAATATTAACTTTAACCTTCCTGTAACAGATGATAATGTATTGCT
ACCAACAGATCCAATTGGAGGTAAAACAGGAATTAACTATACTAATATATTTAATACTTATGGTCCTT
TAACTGCATTAAATAATGTACCACCAGTTTATCCAAATGGTCAAATTTGGGATAAAGAATTTGATACT
GACTTAAAACCAAGACTTCATGTAAATGCACCATTTGTTTGTCAAAATAATTGCCCTGGTCAATTATT
TGTAAAAGTTGCGCCTAATTTAACAAATGAATATGATCCTGATGCATCTGCTAATATGTCAAGAATTG
TAACTTACTCAGATTTTTGGTGGAAAGGTAAATTAGTATTTAAAGCTAAACTAAGAGCCTCTCATACT

TGGAATCCAATTCAACAAATGAGTATTAATGTAGATAACCAATTTAACTATGTACCAAGTAATATTGG
AGGTATGAAAATTGTCTATGAAAAATCTCAACTAGCACCTAGA
'USA_JX475248_CO_1102_2011'
ATGAGTGATGGAGCAGTTCAACCAGACGGTGGTCAACCTGCTGTCAGAAATGAAAGAGCTACAGGATC
TGGGAACGGGTCTGGAGGCGGGGGTGGTGGTGGTTCTGGGGGTGTGGGGATTTCTACGGGTACTTTCA
ATAATCAGACGGAATTTAAATTTTTGGAAAACGGATGGGTGGAAATCACAGCAAACTCAAGCAGACTT
GTACATTTAAATATGCCTGAAAGTGAAAATTATAGAAGAGTGGTTGTAAATAATTTAGATAAAACTGC
AGTTAACGGAAACATGGCTTTAGATGATACTCATGCACAAATTGTAACACCTTGGTCATTGGTTGATG
CAAATGCTTGGGGAGTTTGGTTTAATCCAGGAGATTGGCAACTAATTGTTAATACTATGAGTGAGTTG
CATTTAGTTAGTTTTGAACAAGAAATTTTTAATGTTGTTTTAAAGACTGTTTCAGAATCTGCTACTCA
GCCACCAACTAAAGTTTATAATAATGATTTAACTGCATCATTGATGGTTGCATTAGATAGTAATAATA
CTATGCCATTTACTCCAGCAGCTATGAGATCTGAGACATTGGGTTTTTATCCATGGAAACCAACCATA
CCAACTCCATGGAGATATTATTTTCAATGGGATAGAACATTAATACCATCTCATACTGGAACTAGTGG
CACACCAACAAATACATACCATGGTACAGATCCAGATGATGTTCAATTTTATACTATTGAAAATTCTG
TGCCAGTACACTTACTAAGAACAGGTGATGAATTTGCTACAGGAACATTTTTTTTTGATTGTAAACCA
TGTAGACTAACACATACATGGCAAACAAATAGAGCATTGGGCTTACCACCATTTCTAAATTCTTTGCC
TCAATCTGAAGGAGATACTAACTTTGGTGATATAGGAGTTCAACAAGATAAAAGACGTGGTGTAACTC
AAATGGGAAATACAAACTATATTACTGAAGCTACTATTATGAGACCAGCTGAGGTTGGTTATAGTGCA
CCATATTATTCTTTTGAGGCGTCTACACAAGGGCCATTTAAAACACCTATTGCAGCAGGACGGGGGGG
AGCGCAAACAGATGAAAATCAAGCAGCAGATGGTGATCCAAGATATGCATTTGGTAGACAACATGGTC
AAAAAACTACCACAACAGGAGAAACACCTGAGAGATTTACATATATAGCACATCAAGATACAGGAAGA
TATCCAGAAGGAGATTGGATTCAAAATATTAACTTTAACCTTCCTGTAACAAATGATAATGTATTGCT
ACCAACAGATCCAATTGGAGGTAAAACAGGAATTAACTATACTAATATATTTAATACTTATGGTCCTT
TAACTGCATTAAATAATGTACCACCAGTTTATCCAAATGGTCAAATTTGGGATAAAGAATTTGATACT
GACTTAAAACCAAGACTTCATGTAAATGCACCATTTGTTTGTCAAAATAATTGTCCTGGTCAATTATT
TGTAAAAGTTGCGCCTAATTTAACAAATGAATATGATCCTGATGCATCTGCTAATATGTCAAGAATTG
TAACTTACTCAGATTTTTGGTGGAAAGGTAAATTAGTATTTAAAGCTAAACTAAGAGCCTCTCATACT
TGGAATCCAATTCAACAAATGAGTATTAATGTAGATAACCAATTTAACTATGTACCAAGTAATATTGG
AGGTATGAAAATTGTATATGAAAAATCTCAACTAGCACCTAGA
'USA_JX475250_CO_728_2010'
ATGAGTGATGGAGCAGTTCAACCAGACGGTGGTCAACCTGCTGTCAGAAATGAAAGAGCAACAGGATC
TGGGAACGGGTCTGGAGGCGGGGGTGGTGGTGGTTCTGGGGGTGTGGGGATTTCTACGGGTACTTTCA
ATAATCAGACGGAATTTAAATTTTTGGAAAACGGATGGGTGGAAATCACAGCAAACTCAAGCAGACTT
GTACATTTAAATATGCCAGAAAGTGAAAATTATAGAAGAGTGGTTGTAAATAATTTGGATAAAACTGC
AGTTAACGGAAACATGGCTTTAGATGATACTCATGCACAAATTGTAACACCTTGGTCATTGGTTGATG
CAAATGCTTGGGGAGTTTGGTTTAATCCAGGAGATTGGCAACTAATTGTTAATACTATGAGTGAGTTG
CATTTAGTTAGTTTTGAACAAGAAATTTTTAATGTTGTTTTAAAGACTGTTTCAGAATCTGCCACTCA
GCCACCAACTAAAGTTTATAATAATGATTTAACTGCATCATTGATGGTTGCATTAGATAGCAATAATA
CTATGCCATTTACTCCAGCAGCTATGAGATCTGAGACATTGGGTTTTTATCCATGGAAACCAACCATA
CCAACTCCATGGAGATATTATTTTCAATGGGATAGAACATTAATACCATCTCATACTGGAACTAGTGG
CACACCAACAAATATATACCATGGTACAGATCCAGATGATGTTCAATTTTATACTATTGAAAATTCTG
TGCCAGTACACTTACTAAGAACAGGTGATGAATTTGCTACAGGAACATTTTTTTTTGATTGTAAACCA
TGTAGACTAACACATACATGGCAAACAAATAGAGCATTGGGCTTACCACCATTTCTAAATTCTTTGCC
TCAAGCTGAAGGAGGTACTAACTTTGGTTATATAGGAGTTCAACAAGATAAAAGACGTGGTGTAACTC
AAATGGGAAATACAAACTATATTACTGAAGCTACTATTATGAGACCAGCTGAGGTTGGTTATAGTGCA
CCATATTATTCTTTTGAGGCGTCTACACAAGGGCCATTTAAAACACCTATTGCAGCAGGACGGGGGGG
AGCGCAAACAGATGAAAATCAAGCAGCAGATGGTGATCCAAGATATGCATTTGGTAGACAACATGGTC
AAAAGACTACCACAACAGGAGAAACACCTGAGAGATTTACATATATAGCACATCAAGATACAGGAAGA
TATCCAGAAGGAGATTGGATTCAAAATATTAACTTTAACCTTCCTGTAACAGATGATAATGTATTGCT
ACCAACAGATCCAATTGGAGGTAAAACAGGAATTAACTATACTAATATATTTAATACTTATGGTCCTT
TAACTGCATTAAATAATGTACCACCAGTTTATCCAAATGGTCAAATTTGGGATAAAGAATTTGATACT
GACTTAAAACCAAGACTTCATGTAAATGCACCATTTGTTTGTCAAAATAATTGTCCTGGTCAATTATT
TGTAAAAGTTGCGCCTAATTTAACAAATGAATATGATCCTGATGCATCTGCTAATATGTCAAGAATTG
TAACTTACTCAGATTTTTGGTGGAAAGGTAAATTAGTATTTAAAGCTAAACTAAGAGCCTCTCATACT

TGGAATCCAATTCAACAAATGAGTATTAATGTAGATAACCAATTTAACTATGTACCAAGTAATATTGG
AGGTATGAAAATTGTATATGAAAAATCTCAACTAGCACCAAGA

'USA_JX475251_CO_2235_2009'

ATGAGTGATGGAGCAGTTCAACCAGACGGTGGTCAGCCTGCTGTCAGAAATGAAAGAGCTACAGGATC
TGGGAACGGGTCTGGAGGCGGGGGTGGTGGTGGTTCTGGGGGTGTGGGGATTTCTACGGGTACTTTCA
ATAATCAGACGGAATTTAAATTTTTGGAAAACGGATGGGTGGAAATCACAGCAAACTCAAGCAGACTT
GTACATTTAAATATGCCAGAAAGTGAAAATTATAGAAGAGTGGTTGTAAATAATTTGGATAAAACTGC
AGTTAACGGAAACATGGCTTTAGATGATACTCATGCACAAATTGTAACACCTTGGTCATTGGTTGATG
CAAATGCTTGGGGAGTTTGGTTTAATCCAGGAGATTGGCAACTAATTGTTAATACTATGAGTGAGTTG
CATTTAGTTAGTTTTGAACAAGAAATTTTTAATGTTGTTTTAAAGACTGTTTCAGAATCTGCTACTCA
GCCACCAACTAAAGTTTATAATAATGATTTAACTGCATCATTGATGGTTGCATTAGATAGTAATAATA
CTATGCCATTTACTCCAGCAGCTATGAGATCTGAGACATTGGGTTTTTATCCATGGAAACCAACCATA
CCAACTCCATGGAGATATTATTTTCAATGGGATAGAACATTAATACCATCTCATACTGGAACTAGTGG
CACACCAACAAATATATACCATGGTACAGATCCAGATGATGTTCAATTCTATACTATTGAAAATTCTG
TGCCAGTACACTTACTAAGAACAGGTGATGAATTTGCTACAGGAACATTTTTTTTTGATTGTAAACCA
TGTAGACTAACACATACATGGCAAACAAATAGAGCATTGGGCTTACCACCATTTCTAAATTCTTTGCC
TCAAGCTGAAGGAGGTACTAACTTTGGTTATATAGGAGTTCAACAAGATAAAAGACGTGGTGTAACTC
AAATGGGAAATACAAACTATATTACTGAAGCTACTATTATGAGACCAGCTGAGGTTGGTTATAGTGCA
CCATATTATTCTTTTGAGGCGTCTACACAAGGGCCATTTAAAACACCTATTGCAGCAGGACGGGGGGG
AGCGCAAACAGATGAAAATCAAGCAGCAGATGGTGATCCAAGATATGCATTTGGTAGACAACATGGTC
AAAAAACTACCACAACAGGAGAAACACCTGAGAGATTTACATATATAGCACATCAAGATACAGGAAGA
TATCCAGAAGGAGATTGGATTCAAAATATTAACTTTAACCTTCCTGTAACAGATGATAATGTATTGCT
ACCAACAGATCCAATTGGAGGTAAAACAGGAATTAACTATACTAATATATTTAATACTTATGGTCCTT
TAACTGCATTAAATAATGTACCACCAGTTTATCCAAATGGTCAAATTTGGGATAAAGAATTTGATACT
GACTTAAAACCAAGACTTCATGTAAATGCACCATTTGTTTGTCAAAATAATTGCCCTGGTCAATTATT
TGTAAAAGTTGCGCCTAATTTAACAAATGAATATGATCCTGATGCATCTGCTAATATGTCAAGAATTG
TAACTTACTCAGATTTTTGGTGGAAAGGTAAATTAGTATTTAAAGCTAAACTAAGAGCCTCTCATACT
TGGAATCCAATTCAACAAATGAGTATTAATGTAGATAACCAATTTAACTATGTACCAAGTAATATTGG
AGGTATGAAAATTGTCTATGAAAAATCTCAACTAGCACCTAGA

'USA_JX475252_CO_1316_2010'

ATGAGTGATGGAGCAGTTCAACCAGACGGTGGTCAACCTGCTGTCAGAAATGAAAGAGCAACAGGATC
TGGGAACGGGTCTGGAGGCGGGGGTGGTGGTGGTTCTGGGGGTGTGGGGATTTCTACGGGTACTTTCA
ATAATCAGACGGAATTTAAATTTTTGGAAAACGGATGGGTGGAAATCACAGCAAACTCAAGCAGACTT
GTACATTTAAATATGCCAGAAAGTGAAAATTATAGAAGAGTGGTTGTAAATAATTTGGATAAAACTGC
AGTTAACGGAAACATGGCTTTAGATGATACTCATGCACAAATTGTAACACCTTGGTCATTGGTTGATG
CAAATGCTTGGGGAGTTTGGTTTAATCCAGGAGATTGGCAACTAATTGTTAATACTATGAGTGAGTTG
CATTTAGTTAGTTTTGAACAAGAAATTTTTAATGTTGTTTTAAAGACTGTTTCAGAATCTGCTACTCA
GCCACCAACTAAAGTTTATAATAATGATTTAACTGCATCATTGATGGTTGCATTAGATAGTAATAATA
CTATGCCATTTACTCCAGCAGCTATGAGATCTGAGACATTGGGTTTTTATCCATGGAAACCAACCATA
CCAACTCCATGGAGATATTATTTTCAGTGGGATAGAACATTAATACCATCTCATACTGGAACTAGTGG
CACACCAACAAATATATACCATGGTACAGATCCAGATGATGTTCAATTTTATACTATTGAAAATTCTG
TGCCAGTACACTTACTAAGAACAGGTGATGAATTTGCTACAGGAACATTTTTTTTTGATTGTAAACCA
TGTAGACTAACACATACATGGCAAACAAATAGAGCATTGGGCTTACCACCATTTCTAAATTCTTTGCC
TCAAGCTGAAGGAGGTACTAACTTTGGTTATATAGGAGTTCAACAAGATAAAAGACGTGGTGTAACTC
AAATGGGAAATACAAACTATATTACTGAAGCTACTATTATGAGACCAGCTGAGGTTGGTTATAGTGCA
CCATATTATTCTTTTGAGGCGTCTACACAAGGGCCATTTAAAACACCTATTGCAGCAGGACGGGGGGG
AGCGCAAACAGATGAAAATCAAGCAGCAGATGGTGATCCAAGATATGCATTTGGTAGACAACATGGTC
AAAAAACTACCACAACAGGAGAAACACCTGAGAGATTTACATATATAGCACATCAAGATACAGGAAGA
TATCCAGAAGGAGATTGGATTCAAAATATTAACTTTAACCTTCCTGTAACAGAAGATAATGTATTGCT
ACCAACAGATCCAATTGGAGGTAAAACAGGAATTAACTATACTAATATATTTAATACTTATGGTCCTT
TAACTGCATTAAATAATGTACCACCAGTTTATCCAAATGGTCAAATTTGGGATAAAGAATTTGATACT
GACTTAAAACCAAGACTTCATGTAAATGCACCATTTGTTTGTCAAAATAATTGTCCTGGTCAATTATT
TGTAAAAGTTGCGCCTAATTTAACAAATGAATATGATCCTGATGCATCTGCTAATATGTCAAGAATTG
TAACTTACTCAGATTTTTGGTGGAAAGGTAAATTAGTATTTAAAGCTAAACTAAGAGCCTCTCATACT

TGGAATCCAATTCAACAAATGAGTATTAATGTAGATAACCAATTTAACTATGTACCAAGTAATATTGG
AGGTATGAAAATTGTATATGAAAAATCTCAACTAGCACCTAGA
'USA_JX475260_CO_704_2010'
ATGAGTGATGGAGCAGTTCAACCAGACGGTGGTCAACCTGCTGTCAGAAATGAAAGAGCAACAGGATC
TGGGAACGGGTCTGGAGGCGGGGGTGGTGGTGGTTCTGGGGGTGTGGGGATTTCTACGGGTACTTTCA
ATAATCAGACGGAATTTAAATTTTTGGAAAACGGATGGGTGGAAATCACAGCAAACTCAAGCAGACTT
GTACATTTAAATATGCCAGAAAGTGAAAATTATAGAAGAGTGGTTGTAAATAATTTGGATAAAACTGC
AGTTAACGGAAACATGGCTTTAGATGATACTCATGCACAAATTGTAACACCTTGGTCATTGGTTGATG
CAAATGCTTGGGGAGTTTGGTTTAATCCAGGAGATTGGCAACTAATTGTTAATACTATGAGTGAGTTG
CATTTAGTTAGTTTTGAACAAGAAATTTTTAATGTTGTTTTAAAGACTGTTTCAGAATCTGCTACTCA
GCCACCAACTAAAGTTTATAATAATGATTTAACTGCATCATTGATGGTTGCATTAGATAGTAATAATA
CTATGCCATTTACTCCAGCAGCTATGAGATCTGAGACATTGGGTTTTTATCCATGGAAACCAACCATA
CCAACTCCATGGAGATATTATTTTCAATGGGATAGAACATTAATACCATCTCATACTGGAACTAGTGG
CACACCAACAAATATATACCATGGTACAGATCCAGATGATGTTCAATTTTATACTATTGAAAATTCTG
TGCCAGTACACTTACTAAGAACAGGTGATGAATTTGCTACAGGAACATTTTTCTTTGATTGTAAACCA
TGTAGACTAACACATACATGGCAAACAAATAGAGCATTGGGCTTACCACCATTTCTAAATTCTTTGCC
TCAAGCTGAAGGAGGTACTAACTTTGGTTATATAGGAGTTCAACAAGATAAAAGACGTGGTGTAACTC
AAATGGGAAATACAAACTATATTACTGAAGCTACTATTATGAGACCAGCTGAGGTTGGTTATAGTGCA
CCATATTATTCTTTTGAGGCGTCTACACAAGGGCCATTTAAAACACCTATTGCAGCAGGACGGGGGGG
AGCGCAAACAGATGAAAATCAAGCAGCAGATGGTGATCCAAGATATGCATTTGGTAGACAACATGGTC
AAAAAACTACCACAACAGGAGAAACACCTGAGAGATTTACATATATAGCACATCAAGATACAGGAAGA
TATCCAGAAGGAGATTGGATTCAAAATATTAACTTTAACCTTCCTGTAACAGAAGATAATGTATTGCT
ACCAACAGATCCAATTGGAGGTAAAACAGGAATTAACTATACTAATATATTTAATACTTATGGTCCTT
TAACTGCATTAAATAATGTACCACCAGTTTATCCAAATGGTCAAATTTGGGATAAAGAATTTGATACT
GACTTAAAACCAAGACTTCATGTAAATGCACCATTTGTTTGTCAAAATAATTGTCCTGGTCAATTATT
TGTAAAAGTTGCGCCTAATTTAACAAATGAATATGATCCTGATGCATCTGCTAATATGTCAAGAATTG
TAACTTACTCAGATTTTTGGTGGAAAGGTAAATTAGTATTTAAAGCTAAACTAAGAGCCTCTCATACT
TGGAATCCAATTCAACAAATGAGTATTAATGTAGATAACCAATTTAACTATGTACCAAGTAATATTGG
AGGTATGAAAATTGTATATGAAAAATCTCAACTAGCACCTAGA
'USA_JX475273_MT_909_2012'
ATGAGTGATGGAGCAGTTCAACCAGACGGTGGTCAACCTGCTGTCAGAAATGAAAGAGCAACAGGATC
TGGGAACGGGTCTGGAGGCGGGGGTGGTGGTGGTTCTGGGGGTGTGGGGATTTCTACGGGTACTTTCA
ATAATCAGACGGAATTTAAATTTTTGGAAAACGGATGGGTGGAAATCACAGCAAACTCAAGCAGACTT
GTACATTTAAATATGCCAGAAAGTGAAAATTATAGAAGAGTGGTTGTAAATAATTTGGATAAAACTGC
AGTTAACGGAAACATGGCTTTAGATGATACTCATGCACAAATTGTAACACCTTGGTCATTGGTTGATG
CAAATGCTTGGGGAGTTTGGTTTAATCCAGGAGATTGGCAACTAATTGTTAATACTATGAGTGAGTTG
CATTTAGTTAGTTTTGAACAAGAAATTTTTAATGTTGTTTTAAAGACTGTTTCAGAATCTGCTACTCA
GCCACCAACTAAAGTTTATAATAATGATTTAACTGCATCATTGATGGTTGCATTAGATAGTAATAATA
CTATGCCATTTACTCCAGCAGCTATGAGATCTGAGACATTGGGTTTTTATCCATGGAAACCAACCATA
CCAACTCCATGGAGATATTATATTCAATGGGATAGAACATTAATACCATCTCATACTGGAACTAGTGG
CACACCAACAAATATATACCATGGTACAGATCCAGATGATGTTCAATTTTATACTATTGAAAATTCTG
TGCCAGTACACTTACTAAGAACAGGTGATGAATTTGCTACAGGAACATTTTTTTTTGATTGTAAACCA
TGTAGACTAACACATACATGGCAAACAAATAGAGCATTGGGCTTACCACCATTTCTAAATTCTTTGCC
TCAAGCTGAAGGAGGTACTAACTTTGGTTATATAGGAGTTCAACAAGATAAAAGACGTGGTGTAACTC
AAATGGGAAATACAAACTATATTACTGAAGCTACTATTATGAGACCAGCTGAGGTTGGTTATAGTGCA
CCATATTATTCTTTTGAGGCGTCTACACAAGGACCATTTAAAACACCTATTGCAGCAGGACGGGGGGG
AGCGCAAACAGATGAAAATCAAGCAGCAGATGGTGATCCAAGATATGCATTTGGTAGACAACATGGTC
AAAAAACTACCACAACAGGAGAAACACCTGAGAGATTTACATATATAGCACATCAAGATACAGGAAGA
TATCCAGAAGGAGATTGGATTCAAAATATTAACTTTAACCTTCCTGTAACAGAAGATAATGTATTGCT
ACCAACAGATCCAATTGGAGGTAAAACAGGAATTAACTATACTAATATATTTAATACTTATGGTCCTT
TAACTGCATTAAATAATGTACCACCAGTTTATCCAAATGGTCAAATTTGGGATAAAGAATTTGATACT
GACTTAAAACCAAGACTTCATGTAAATGCACCATTTGTTTGTCAAAATAATTGTCCTGGTCAATTATT
TGTAAAAGTTGCGCCTAATTTAACAAATGAATATGATCCTGATGCATCTGCTAATATGTCAAGAATTG
TAACTTACTCAGATTTTTGGTGGAAAGGTAAATTAGTATTTAAAGCTAAACTAAGAGCCTCTCATACT

TGGAATCCAATTCAACAAATGAGTATTAATGTAGATAACCAATTTAACTATGTACCAAGTAATATTGG
AGGTATGAAAATTGTATATGAAAAATCTCAACTAGCACCTAGA
'USA_JX475278_AR_1069_2012'
ATGAGTGATGGAGCAGTTCAACCAGACGGTGGTCAGCCTGCTGTCAGAAATGAAAGAGCTACAGGATC
TGGGAACGGGTCTGGAGGCGGGGGTGGTGGTGGTTCTGGGGGTGTGGGGATTTCTACGGGTACTTTCA
ATAATCAGACGGAATTTAAATTTTTGGAAAACGGATGGGTGGAAATCACAGCAAACTCAAGCAGACTT
GTACATTTAAATATGCCAGAAAGTGAAAATTATAGAAGAGTGGTTGTAAATAATTTGGATAAAACTGC
AGTTAACGGAAACATGGCTTTAGATGATACTCATGCACAAATTGTAACACCTTGGTCATTGGTTGATG
CAAATGCTTGGGGAGTTTGGTTTAATCCAGGAGATTGGCAACTAATTGTTAATACTATGAGTGAGTTG
CATTTAGTTAGTTTTGAACAAGAAATTTTTAATGTTGTTTTAAAGACTGTTTCAGAATCTGCTACTCA
GCCACCAACTAAAGTTTATAATAATGATTTAACTGCATCATTGATGGTTGCATTAGATAGTAATAATA
CTATGCCATTTACTCCAGCAGCTATGAGATCTGAGACATTGGGTTTTTACCCATGGAAACCAACCATA
CCAACTCCATGGAGATATTATTTTCAATGGGATAGAACATTAATACCATCTCATACTGGAACTAGTGG
CACACCAACAAATATATACCATGGTACAGATCCAGATGATGTTCAATTTTATACTATTGAAAATTCTG
TGCCAGTACACTTACTAAGAACAGGTGATGAATTTGCTACAGGAACATTTTTTTTTGATTGTAAACCA
TGTAGACTAACACATACATGGCAAACAAATAGAGCATTGGGCTTACCACCATTTCTAAATTCTTTGCC
TCAAGCTGAAGGAGGTACTAACTTTGGTTATATAGGAGTTCAACAAGATAAAAGACGTGGTGTAACTC
AAATGGGAAATACAAACTATATTACTGAAGCTACTATTATGAGACCAGCTGAGGTTGGTTATAGTGCA
CCATATTATTCTTTTGAGGCGTCTACACAAGGGCCATTTAAAACACCTATTGCAGCAGGACGGGGGGG
AGCGCAAACAGATGAAAATCAAGCAGCAGATGGTGATCCAAGATATGCATTTGGTAGACAACATGGTC
AAAAAACTACCACAACAGGAGAAACACCTGAGAGATTTACATATATAGCACATCAAGATACAGGAAGA
TATCCAGAAGGAGATTGGATTCAAAATATTAACTTTAACCTTCCTGTAACAGATGATAATGTATTGCT
ACCAACAGATCCAATTGGAGGTAAAACAGGAATTAACTATACTAATATATTTAATACTTATGGTCCTT
TAACTGCATTAAATAATGTACCACCAGTTTATCCAAATGGTCAAATTTGGGATAAAGAATTTGATACT
GACTTAAAACCAAGACTTCATGTAAATGCACCATTTGTTTGTCAAAATAATTGCCCTGGTCAATTATT
TGTAAAAGTTGCGCCTAATTTAACAAATGAATATGATCCTGATGCATCTGCTAATATGTCAAGAATTG
TAACTTACTCAGATTTTTGGTGGAAAGGTAAATTAGTATTTAAAGCTAAACTAAGAGCCTCTCATACT
TGGAATCCAATTCAACAAATGAGTATTAATGTAGATAACCAATTTAACTATGTACCAAGTAATATTGG
AGGTATGAAAATTGTCTATGAAAAATCTCAACTAGCACCTAGA
'USA_JX475279_TN_1_2011'
ATGAGTGATGGAGCAGTTCAACCAGACGGTGGTCAACCTGCTGTCAGAAATGAAAGAGCTACAGGATC
TGGGAACGGGTCTGGAGGCGGGGGTGGTGGTGGTTCTGGGGGTGTGGGGATCTCTACGGGTACTTTCA
ATAATCAGACGGAATTTAAATTTTTGGAAAACGGATGGGTGGAAATCACAGCAAACTCAAGCAGACTT
GTACATTTAAATATGCCAGAAAGTGAAAATTATAGAAGAGTGGTTGTAAATAATTTGGATAAAACTGC
AGTTAACGGAAACATGGCTTTAGATGATACTCATGCACAAATTGTAACACCTTGGTCATTGGTTGATG
CAAATGCTTGGGGAGTTTGGTTTAATCCAGGAGATTGGCAACTAATTGTTAATACTATGAGTGAGTTG
CATTTAGTTAGTTTTGAACAAGAAATTTTTAATGTTGTTTTAAAGACTGTTTCAGAATCTGCTACTCA
GCCACCAACTAAAGTTTATAATAATGATTTAACTGCATCATTGATGGTTGCATTAGATAGTAATAATA
CTATGCCATTTACTCCAGCAGCTATGAGATCTGAGACATTGGGTTTTTATCCATGGAAACCAACCATA
CCAACTCCATGGAGATATTATTTTCAATGGGATAGAACATTAATACCATCTCATACTAGAACTAGTGG
CACACCAACAAATATATACCATGGTACAGATCCAGATGATGTTCAATTTTATACTATTGAAAATTCTG
TGCCAGTACACTTACTAAGAACAGGTGATGAATTTGCTACAGGAACATTTTTTTTTAATTGTAAACCA
TGTAGACTAACACATACATGGCAAACAAATAGAGCATTGGGCTTACCACCATTTCTAAATTCTTTGCC
TCAAGCTGAAGGAGATACTAACTTTGGTGATATAGGAGTTCAACAAGATAAAAGACGTGGTGTAACTC
AAATGGGAAATACAAACTATATTACTGAAGCTACTATTATGAGACCAGCTGAGGTTGGTTATAGTGCA
CCATATTATTCTTTTGAGGCGTCTACACAAGGGCCATTTAAAACACCTATTGCAGCAGGACGGGGGGG
AGCGCAAACAGATGAAAATCAAGCAGCAGATGGTGATCCAAGATATGCATTTGGTAGACAACATGGTC
AAAAAACTACCACAACAGGAGAAACACCTGAGAGATTTACATATATAGCACATCAAGATACAGGAAGA
TATCCAGAAGGAGATTGGATTCAAAATATTAACTTTAACCTTCCTGTAACAAATGATAATGTATTGCT
ACCAACAGATCCAATTGGAGGTAAAACAGGAATTAACTATACTAATATATTTAATACTTATGGTCCTT
TAACTGCATTAAATAATGTACCACCAGTTTATCCAAATGGTCAAATTTGGGATAAAGAATTTGATACT
GACTTAAAACCAAGACTTCATGTAAATGCACCATTTGTTTGTCAAAATAATTGTCCTGGTCAATTATT
TGTAAAAGTTGCGCCTAATTTAACAAATGAATATGATCCTGATGCATCTGCTAATATGTCAAGAATTG
TAACTTACTCAGATTTTTGGTGGAAAGGTAAATTAGTATTTAAAGCTAAACTAAGAGCCTCTCATACT

TGGAATCCAATTCAACAAATGAGTATTAATGTAGATAACCAATTTAACTATGTACCAAGTAATATTGG
AGGTATGAAAATTGTATATGAAAAATCTCAACTAGCACCTAGA

'USA_JX475284_TN_26_2011'

ATGAGTGATGGAGCAGTTCAACCAGACGGTGGTCAACCTGCTGTCAGAAATGAAAGAGCTACAGGATC
TGGGAACGGGTCTGGAGGCGGGGGTGGTGGTGGTTCTGGGGGTGTGGGGATTTCTACGGGTACTTTCA
ATAATCAGACGGAATTTAAATTTTTGGAAAACGGATGGGTGGAAATCACAGCAAACTCAAGCAGACTT
GTACATTTAAATATGCCAGAAAGTGAAAATTATAGAAGAGTGGTTGTAAATAATTTGGATAAAACTGC
AGTTAACGGAAACATGGCTTTAGATGATACTCATGCACAAATTGTAACACCTTGGTCATTGGTTGATG
CAAATGCTTGGGGAGTTTGGTTTAATCCAGGAGATTGGCAACTAATTGTTAATACTATGAGTGAGTTG
CATTTAGTTAGTTTTGAACAAGAAATTTTTAATGTTGTTTTAAAGACTGTTTCAGAATCTGCTACTCA
GCCACCAACTAAAGTTTATAATAATGATTTAACTGCATCATTGATGGTTGCATTAGATAGTAATAATA
CTATGCCATTTACTCCAGCAGCTATGAGATCTGAGACATTGGGTTTTTATCCATGGAAACCAACCATA
CCAACTCCATGGAGATATTATTTTCAATGGGATAGAACATTAATACCATCTCATACTAGAACTAGTGG
CACACCAACAAATATATACCATGGTACAGATCCAGATGATGTTCAATTTTATACTATTGAAAATTCTG
TGCCAGTACACTTACTAAGAACAGGTGATGAATTTGCTACAGGAACATTTTTTTTTGATTGTAAACCA
TGTAGACTAACACATACATGGCAAACAAATAGAGCATTGGGCTTACCACCATTTCTAAATTCTTTGCC
TCAAGCTGAAGGAGATACTAACTTTGGTGATATAGGAGTTCAACAAGATAAAAGACGTGGTGTAACTC
AAATGGGAAATACAAACTATATTACTGAAGCTACTATTATGAGACCAGCTGAGGTTGGTTATAGTGCA
CCATATTATTCTTTTGAGGCGTCTACACAAGGGCCATTTAAAACACCTATTGCAGCAGGACGGGGGGG
AGCGCAAACAGATGAAAATCAAGCAGCAGATGGTGATCCAAGATATGCATTTGGTAGACAACATGGTC
AAAAAACTACCACAACAGGAGAAACACCTGAGAGATTTACATATATAGCACATCAAGATACAGGAAGA
TATCCAGAAGGAGATTGGATTCAAAATATTAACTTTAACCTTCCTGTAACAAATGATAATGTATTGCT
ACCAACAGATCCAATTGGAGGTAAAACAGGAATTAACTATACTAATATATTTAATACTTATGGTCCTT
TAACTGCATTAAATAATGTACCACCAGTTTATCCAAATGGTCAAATTTGGGATAAAGAATTTGATACT
GACTTAAAACCAAGACTTCATGTAAATGCACCATTTGTTTGTCAAAATAATTGTCCTGGTCAATTATT
TGTAAAAGTTGCGCCTAATTTAACAAATGAATATGATCCTGATGCATCTGCTAATATGTCAAGAATTG
TAACTTACTCAGATTTTTGGTGGAAAGGTAAATTAGTATTTAAAGCTAAACTAAGAGCCTCTCATACT
TGGAATCCAATTCAACAAATGAGTATTAATGTAGATAACCAATTTAACTATGTACCAAGTAATATTGG
AGGTATGAAAATTGTATATGAAAAATCTCAACTAGCACCTAGA

'URU_KC196081_2c_M95_2007'

ATGAGTGATGGAGCAGTTCAACCAGACGGTGGTCAATCTGCTGTCAGAAATGAAAGAGCAACAGGATC
TGGGAACGGGTCTGGAGGCGGGGGTGGTGGTGGTTCTGGGGGTGTGGGGATTTCTACGGGTACTTTCA
ATAATCAGACGGAATTTAAATTTTTGGAAAACGGATGGGTGGAAATCACAGCAAACTCAAGCAGACTT
GTACATTTAAATATGCCAGAAAGTGAAAATTATAGAAGAGTGGTTGTAAATAATTTGGATAAAACTGC
AGTTAACGGAAACATGGCTTTAGATGATACTCATGCACAAATTGTAACACCTTGGTCATTGGTTGATG
CAAATGCTTGGGGAGTTTGGTTTAATCCAGGAGATTGGCAACTAATTGTTAATACTATGAGTGAGTTG
CATTTAGTTAGTTTTGAACAAGAAATTTTTAATGTTGTTTTAAAGACTGTTTCAGAATCTGCTACTCA
GCCACCAACTAAAGTTTATAATAATGATTTAACTGCATCATTGATGGTTGCATTAGATAGTAATAATA
CTATGCCATTTACTCCAGCAGCTATGAGATCTGAGACATTGGGTTTTTATCCATGGAAACCAACCATA
CCAACTCCATGGAGATATTATTTTCAATGGGATGGAACATTAATACCATCTCATACTGGAACTAGTGG
CACACCAACAAATATATACCATGGTACAGATCCAGATGATGTTCAATTTTATACTATTGAAAATTCTG
TGCCAGTACACTTACTAAGAACAGGTGATGAATTTGCTACAGGAACATTTTTTTTTGATTGTAAACCA
TGTAGACTAACACATACATGGCAAACAAATAGAGCATTGGGCTTACCACCATTTCTAAATTCTTTGCC
TCAAGCTGAAGGAGGTACTAACTTTGGTTATATAGGAGTTCAACAAGATAAAAGACGTGGTGTAACTC
AAATGGGAAATACAAACTATATTACTGAAGCTACTATTATGAGACCAGCTGAGGTTGGTTATAGTGCA
CCATATTATTCTTTTGAGGCGTCTACACAAGGGCCATTTAAAACACCTATTGCAGCAGGACGGGGGGG
AGCGCAAACAGATGAAAATCAAGCAGCAGATGGTGATCCAAGATATGCATTTGGTAGACAACATGGTC
AAAAAACTACCACAACAGGAGAAACACCTGAGAGATTTACATATATAGCACATCAAGATACAGGAAGA
TATCCAGAAGGAGATTGGATTCAAAATATTAACTTTAACCTTCCTGTAACAGAAGATAATGTATTGCT
ACCAACAGATCCAATTGGAGGTAAAACAGGAATTAACTATACTAATATATTTAATACTTATGGTCCTT
TAACTGCATTAAATAATGTACCACCAGTTTATCCAAATGGTCAAATTTGGGATAAAGAATTTGATACT
GACTTAAAACCAAGACTTCATGTAAATGCACCATTTGTTTGTCAAAATAATTGTCCTGGTCAATTATT
TGTAAAAGTTGCGCCTAATTTAACAAATGAATATGATCCTGATGCATCTGCTAATATGTCAAGAATTG
TAACTTACTCAGATTTTTGGTGGAAAGGTAAATTAGTATTTAAAGCTAAACTAAGAGCCTCTCATACT

TGGAATCCAATTCAACAAATGAGTATTAATGTAGATAACCAATTTAACTATGTACCAAGTAATATTGG
AGGTATGAAAATTGTATATGAAAAATCTCAACTAGCACCTAGA

'URU_KC196083_2c_M82_2007'

ATGAGTGATGGAGCAGTTCAACCAGACGGTGGTCAACCTGCTGTCAGAAATGAAAGAGCAACAGGATC
TGGGAACGGGTCTGGAGGCGGGGGTGGTGGTGGTTCTGGGGGTGTGGGGATTTCTACGGGTACTTTCA
ATAATCAAACGGAATTTAAATTTTTGGAAAACGGATGGGTGGAAATCACAGCAAACTCAAGCAGACTT
GTACATTTAAATATGCCAGAAAGTGAAAATTATAGAAGAGTGGTTGTAAATAATTTGGATAAAACTGC
AGTTAACGGAAACATGGCTTTAGATGATACTCATGCACAAATTGTAACACCTTGGTCATTGGTTGATG
CAAATGCTTGGGGAGTTTGGTTTAATCCAGGAGATTGGCAACTAATTGTTAATACTATGAGTGAGTTG
CATTTAGTTAGTTTTGAACAAGAAATTTTTAATGTTGTTTTAAAGACTGTTTCAGAATCTGCTACTCA
GCCACCAACTAAAGTTTATAATAATGATTTAACTGCATCATTGATGGTTGCATTAGATAGTAATAATA
CTATGCCATTTACTCCAGCAGCTATGAGATCTGAGACATTGGGTTTTTATCCATGGAAACCAACCATA
CCAACTCCATGGAGATATTATTTTCAATGGGATAGAACATTAATACCATCTCATACTGGAACTAGTGG
CACACCAACAAATATATACCATGGTACAGATCCAGATGATGTTCAATTTTATACTATTGAAAATTCTG
TGCCAGTACACTTACTAAGAACAGGTGATGAATTTGCTACAGGAACATTTTTTTTTGATTGTAAACCA
TGTAGACTAACACATACATGGCAAACAAATAGAGCATTGGGCTTACCACCATTTCTAAATTCTTTGCC
TCAAGCTGAAGGAGGTACTAACTTTGGTTATATAGGAGTTCAACAAGATAAAAGACGTGGTGTAACTC
AAATGGGAAATACAAACTATATTACTGAAGCTACTATTATGAGACCAGCTGAGGTTGGTTATAGTGCA
CCATATTATTCTTTTGAGGCGTCTACACAAGGGCCATTTAAAACACCTATTGCAGCAGGACGGGGGGG
AGCGCAAACAGATGAAAATCAAGCAGCAGATGGTGATCCAAGATATGCATTTGGTAGACAACATGGTC
AAAAAACTACCACAACAGGAGAAACACCTGAGAGATTTACATATATAGCACATCAAGATACAGGAAGA
TATCCAGAAGGAGATTGGATTCAAAATATTAACTTTAACCTTCCTGTAACAGAAGATAATGTATTGCT
ACCAACAGATCCAATTGGAGGTAAAACAGGAATTAACTATACTAATATATTTAATACTTATGGTCCTT
TAACTGCATTAAATAATGTACCACCAGTTTATCCAAATGGTCAAATTTGGGATAAAGAATTTGATACT
GACTTAAAACCAAGACTTCATGTAAATGCACCATTTGTTTGTCAAAATAATTGTCCTGGTCAATTATT
TGTAAAAGTTGCGCCTAATTTAACAAATGAATATGATCCTGATGCATCTGCTAATATGTCAAGAATTG
TAACTTACTCAGATTTTTGGTGGAAAGGTAAATTAGTATTTAAAGCTAAACTAAGAGCCTCTCATACT
TGGAATCCAATTCAACAAATGAGCATTAATGTAGATAACCAATTTAACTATGTACCAAGTAATATTGG
AGGTATGAAAATTGTATATGAAAAATCTCAACTAGCACCTAGA

'URU_KC196085_2c_M57_2007'

ATGAGTGATGGAGCAGTTCAACCAGACGGTGGTCAACCTGCTGTCAGAAATGAAAGAGCAACAGGATC
TGGGAACGGGTCTGGAGGCGGGGGTGGTGGTGGTTCTGGGGGTGTGGGGATTTCTACGGGTACTTTCA
ATAATCAGACGGAATTTAAATTTTTGGAAAACGGATGGGTGGAAATCACAGCAAACTCAAGCAGACTT
GTACATTTAAATATGCCAGAAAGTGAAAATTATAGAAGAGTGGTTGTAAATAATTTGGATAAAACTGC
AGTTAACGGAAACATGGCTTTAGATGATACTCATGCACAAATTGTAACACCTTGGTCATTGGTTGATG
CAAATGCTTGGGGAGTTTGGTTTAATCCAGGAGATTGGCAACTAATTGTTAATACTATGAGTGAGTTG
CATTTAGTTAGTTTTGAACAAGAAATTTTTAATGTTGTTTTAAAGACTGTTTCAGAATCTGCTACTCA
GCCACCAACTAAAGTTTATAATAATGATTTAACTGCATCATTGATGGTTGCATTAGATAGTAATAATA
CTATGCCATTTACTCCAGCAGCTATGAGATCTGAGACATTGGGTTTTTATCCATGGAAACCAACCATA
CCAACTCCATGGAGATATTATTTTCAATGGGATAGAACATTAATACCATCTCATACTGGAACTAGTGG
CACACCAACAAATATATACCATGGTACAGATCCAGATGATGTTCAATTTTATACTATTGAAAATTCTG
TGCCAGTACACTTACTAAGAACAGGTGATGAATTTGCTACAGGAACATTTTTTTTTGATTGTAAACCA
TGTAGACTAACACATACATGGCAAACAAATAGAGCATTGGGCTTACCACCATTTCTAAATTCTTTGCC
TCAAGCTGAAGGAGGTACTAACTTTGGTTATATAGGAGTTCAACAAGATAAAAGACGTGGTGTAACTC
AAATGGGAAATACAAACTATATTACTGAAGCTACTATTATGAGACCAGCTGAAGTTGGTTATAGTGCA
CCATATTATTCTTTTGAGGCGTCTACACAAGGGCCATTTAAAACACCTATTGCAGCAGGACGGGGGGG
AGCGCAAACAGATGAAAATCAAGCAGCAGATGGTGATCCAAGATATGCATTTGGTAGACAACATGGTC
AAAAAACTACCACAACAGGAGAAACACCTGAGAGATTTACATATATAGCACATCAAGATACAGGAAGA
TATCCAGAAGGAGATTGGATTCAAAATATTAACTTTAACCTTCCTGTAACAGAAGATAATGTATTGCT
ACCAACAGATCCAATTGGAGGTAAAACAGGAATTAACTATACTAATATATTTAATACTTATGGTCCTT
TAACTGCATTAAATAATGTACCACCAGTTTATCCAAATGGTCAAATTTGGGATAAAGAATTTGATACT
GACTTAAAACCAAGACTTCATGTAAATGCACCATTTGTTTGTCAAAATAATTGTCCTGGTCAATTATT
TGTAAAAGTTGCGCCTAATTTAACAAATGAATATGATCCTGATGCATCTGCTAATATGTCAAGAATTG
TAACTTACTCAGATTTTTGGTGGAAAGGTAAATTAGTATTTAAAGCTAAACTAAGAGCCTCTCATACT

TGGAATCCAATTCAACAAATGAGTATTAATGTAGATAACCAATTTAACTATGTACCAAGTAATATTGG
AGGTATGAAAATTGTATATGAAAAATCTCAACTAGCACCTAGA
'URU_KC196086_2c_M55_2006'
ATGAGTGATGGAGCAGTTCAACCAGACGGTGGTCAACCTGCTGTCAGAAATGAAAGAGCAACAGGATC
TGGGAACGGGTCTGGAGGCGGGGGTGGTGGTGGTTCTGGGGGTGTGGGGATTTCTACGGGTACTTTCA
ATAATCAGACGGAATTTAAATTTTTGGAAAACGGATGGGTGGAAATCACAGCAAACTCAAGCAGACTT
GTACATTTAAATATGCCAGAAAGTGAAAATTATAGAAGAGTGGTTGTAAATAATTTGGATAAAACTGC
AGTTAACGGAAACATGGCTTTAGATGATACTCATGCACAAATTGTAACACCTTGGTCATTGGTTGATA
CAAATGCTTGGGGAGTTTGGTTTAATCCAGGAGATTGGCAACTAATTGTTAATACTATGAGTGAGTTG
CATTTAGTTAGTTTTGAACAAGAAATTTTTAATGTTGTTTTAAAGACTGTTTCAGAATCTGCTACTCA
GCCACCAACTAAAGTTTATAATAATGATTTAACTGCATCATTGATGGTTGCATTAGATAGTAATAATA
CTATGCCATTTACTCCAGCAGCTATGAGATCTGAGACATTGGGTTTTTATCCATGGAAACCAACCATA
CCAACTCCATGGAGATATTATTTTCAATGGGATAGAACATTAATACCATCTCATACTGGAACTAGTGG
CACACCAACAAATATATACCATGGTACAGATCCAGATGATGTTCAATTTTATACTATTGAAAATTCTG
TGCCAGTACACTTACTAAGAACAGGTGATGAATTTGCTACAGGAACATTTTTTTTTGATTGTAAACCA
TGTAGACTAACACATACATGGCAAACAAATAGAGCATTGGGCTTACCACCATTTCTAAATTCTTTGCC
TCAAGCTGAAGGAGGTACTAACTTTGGTTATATAGGAGTTCAACAAGATAAAAGACGTGGTGTAACTC
AAATGGGAAATACAAACTATATTACTGAAGCTACTATTATGAGACCAGCTGAGGTTGGTTATAGTGCA
CCATATTATTCTTTTGAGGCGTCTACACAAGGGCCATTTAAAACACCTATTGCAGCAGGACGGGGGGG
AGCGCAAACAGATGAAAATCAAGCAGCAGATGGTGATCCAAGATATGCATTTGGTAGACAACATGGTC
AAAAAACTACCACAACAGGAGAAACACCTGAGAGATTTACATATATAGCACATCAAGATACAGGAAGA
TATCCAGAAGGAGATTGGATTCAAAATATTAACTTTAACCTTCCTGTAACAGAAGATAATGTATTGCT
ACCAACAGATCCAATTGGAGGTAAAACAGGAATTAACTATACTAATATATTTAATACTTATGGTCCTT
TAACTGCATTAAATAATGTACCACCAGTTTATCCAAATGGTCAAATTTGGGATAAAGAATTTGATACT
GACTTAAAACCAAGACTTCATGTAAATGCACCATTTGTTTGTCAAAATAATTGTCCTGGTCAATTATT
TGTAAAAGTTGCGCCTAATTTAACAAATGAATATGATCCTGATGCATCTGCTAATATGTCAAGAATTG
TAACTTACTCAGATTTTTGGTGGAAAGGTAAATTAGTATTTAAAGCTAAACTAAGAGCCTCTCATACT
TGGAATCCAATTCAACAAATGAGTATTAATGTAGATAACCAATTTAACTATGTACCAAGTAATATTGG
AGGTATGAAAATTGTGTATGAAAAATCTCAACTAGCACCTAGA
'URU_KC196089_2c_M349_2011'
ATGAGTGATGGAGCAGTTCAACCAGACGGTGGTCAACCTGCTGTCAGAAATGAAAGAGCAACAGGATC
TGGGAACGGGTCTGGAGGCGGGGGTGGTGGTGGTTCTGGGGGTGTGGGGATTTCTACGGGTACTTTCA
ATAATCAGACGGAATTTAAATTTTTGGAAAACGGATGGGTGGAAATCACAGCAAACTCAAGCAGACTT
GTACATTTAAATATGCCAGAAAGTGAAAATTATAGAAGAGTGGTTGTAAATAATTTGGATAAAACTGC
AGTTAACGGAAACATGGCTTTAGATGATACTCATGCACAAATTGTAACACCTTGGTCATTGGTTGATG
CAAATGCTTGGGGAGTTTGGTTTAATCCAGGAGATTGGCAACTAATTGTTAATACTATGAGTGAGTTG
CATTTAGTTAGTTTTGAACAAGAAATTTTTAATGTTGTTTTAAAGACTGTTACAGAATCTGCTACTCA
GCCACCAACTAAAGTTTATAATAATGATTTAACTGCATCATTGATGGTTGCATTAGATAGTAATAATA
CTATGCCATTTACTCCAGCAGCTATGAGATCTGAGACATTGGGTTTTTATCCATGGAAACCAACCATA
CCAACTCCATGGAGATATTATTTTCAATGGGATAGAACATTAATACCATCTCATACTGGAACTAGTGG
CACACCAACAAATATATACCATGGTACAGATCCAGATGATGTTCAATTTTATACTATTGAAAATTCTG
TGCCAGTACACTTACTAAGAACAGGTGATGAATTTGCTACAGGAACATTTTTTTTTGATTGTAAACCA
TGTAGACTAACACATACATGGCAAACAAATAGAGCATTGGGCTTACCACCATTTCTAAATTCTTTGCC
TCAAGCTGAAGGAGGTACTAACTTTGGTTATATAGGAGTTCAACAAGATAAAAGACGTGGTGTAACTC
AAATGGGAAATACAAACTATATTACTGAAGCTACTATTATGAGACCAGCTGAGGTTGGTTATAGTGCA
CCATATTATTCTTTTGAGGCGTCTACACAAGGGCCATTTAAAACACCTATTGCAGCAGGACGGGGGGG
AGCGCAAACAGATGAAAATCAAGCAGCAGATGGTGATCCAAGATATGCATTTGGTAGACAACATGGTC
AAAAAACTACCACAACAGGAGAAACACCTGAGAGATTTACATATATAGCACATCAAGATACAGGAAGA
TATCCAGAAGGAGATTGGATTCAAAATATTAACTTTAACCTTCCTGTAACAGAAGATAATGTATTGCT
ACCAACAGATCCAATTGGAGGTAAAACAGGAATTAACTATACTAATATATTTAATACTTATGGTCCTT
TAACTGCATTAAATAATGTACCACCAGTTTATCCAAATGGTCAAATTTGGGATAAAGAATTTGATACT
GACTTAAAACCAAGACTTCATGTAAATGCACCATTTGTTTGTCAAAATAATTGTCCTGGTCAATTATT
TGTAAAAGTTGCGCCTAATTTAACAAATGAATATGATCCTGATGCATCTGCTAATATGTCAAGAATTG
TAACTTACTCAGATTTTTGGTGGAAAGGTAAATTAGTATTTAAAGCTAAACTAAGAGCCTCTCATACT

TGGAATCCAATTCAACAAATGAGTATTAATGTAGATAACCAATTTAACTATGTACCAAGTAATATTGG
AGGTATGAAAATTGTATATGAAAAATCTCAACTAGCACCTAGA

'URU_KC196091_2c_M326_2011'

ATGAGTGATGGAGCAGTTCAACCAGACGGTGGTCAACCTGCTGTCAGAAATGAAAGAGCAACAGGATC
TGGGAACGGGTCTGGAGGCGGGGGTGGTGGTGGTTCTGGGGGTGTGGGGATTTCTACGGGTACTTTCA
ATAATCAGACGGAATTTAAATTTTTGGAAAACGGATGGGTGGAAATCACAGCAAACTCAAGCAGACTT
GTACATTTAAATATGCCAGAAAGTGAAAATTATAGAAGAGTGGTTGTAAATAATTTGGATAAAACTGC
AGTTAACGGAAACATGGCTTTAGATGATACTCATGCACAAATTGTAACACCTTGGTCATTGGTTGATG
CAAATGCTTGGGGAGTTTGGTTTAATCCAGGAGATTGGCAACTAATTGTTAATACTATGAGTGAGTTG
CATTTAGTTAGTTTTGAACAAGAAATTTTTAATGTTGTTTTAAAGACTGTTTCAGAATCTGCTACTCA
GCCACCAACTAAAGTTTATAATAATGATTTAACTGCATCATTGATGGTTGCATTAGATAGTAATAATA
CTATGCCATTTACTCCAGCAGCTATGAGATCTGAGACATTGGGTTTTTATCCATGGAAACCAACCATA
CCAACTCCATGGAGATATTATTTTCAATGGGATAGAACATTAATACCATCTCATACTGGAACTAGTGG
CACACCAACAAATATATACCATGGTACAGATCCAGATGATGTTCAATTTTATACTATTGAAAATTCTG
TGCCAGTACACTTACTAAGAACAGGTGATGAATTTGCTACAGGAACATTTTTTTTTGATTGTAAACCA
TGTAGACTAACACATACATGGCAAACAAATAGAGCATTGGGCTTACCACCATTTCTAAATTCTTTGCC
TCAAGCAGAAGGAGGTACTAACTTTGGTTATATAGGAGTTCAACAAGATAAAAGACGTGGTGTAACTC
AAATGGGAAATACAAACTATATTACTGAAGCTACTATTATGAGACCAGCTGAGGTTGGTTATAGTGCA
CCATATTATTCTTTTGAGGCGTCTACACAAGGGCCATTTAAAACACCTATTGCAGCAGGACGGGGGGG
AGCGCAAACAGATGAAAATCAAGCAGCAGATGGTGATCCAAGATATGCATTTGGTAGACAACATGGTC
AAAAAACTACCACAACAGGAGAAACACCTGAGAGATTTACATATATAGCACATCAAGATACAGGAAGA
TATCCAGAAGGAGATTGGATTCAAAATATTAACTTTAACCTTCCTGTAACAGAAGATAATGTATTGCT
ACCAACAGATCCAATTGGAGGTAAAACAGGAATTAACTATACTAATATATTTAATACTTATAGTCCTT
TAACTGCATTAAATAATGTACCACCAGTTTATCCAAATGGTCAAATTTGGGATAAAGAATTTGATACT
GACTTAAAACCAAGACTTCATGTAAATGCACCATTTGTTTGTCAAAATAATTGTCCTGGTCAATTATT
TGTAAAAGTTGCGCCTAATTTAACAAATGAATATGATCCTGATGCATCTGCTAATATGTCAAGAATTG
TAACTTACTCAGATTTTTGGTGGAAAGGTAAATTAGTATTTAAAGCTAAACTAAGAGCCTCTCATACT
TGGAATCCAATTCAACAAATGAGTATTAATGTAGATAACCAATTTAACTATGTACCAAGTAATATTGG
AGGTATGAAAATTGTATATGAAAAATCTCAACTAGCACCTAGA

'URU_KC196093_2c_M307_2011'

ATGAGTGATGGAGCAGTTCAACCAGACGGTGGTCAACCTGCTGTCAGAAATGAAAGAGCAACAGGATC
TGGGAACGGGTCTGGAGGCGGGGGTGGTGGTGGTTCTGGGGGTGTGGGGATTTCTACGGGTACTTTCA
ATAATCAGACGGAATTTAAATTTTTGGAAAACGGATGGGTGGAAATCACAGCAAACTCAAGCAGACTT
GTACATTTAAATATGCCAGAAAGTGAAAATTATAGAAGAGTGGTTGTAAATAATTTGGATAAAACTGC
AGTTAACGGAAACATGGCTTTAGATGATACTCATGCACAAATTGTAACACCTTGGTCATTGGTTGATG
CAAATGCTTGGGGAGTTTGGTTTAATCCAGGAGATTGGCAACTAATTGTTAATACTATGAGTGAGTTG
CATTTAGTTAGTTTTGAACAAGAAATTTTTAATGTTGTTTTAAAGACTGTTTCAGAATCTGCTACTCA
GCCACCAACTAAAGTTTATAATAATGATTTAACTGCATCATTGATGGTTGCATTAGATAGTAATAATA
CTATGCCATTTACTCCAGCAGCTATGAGATCTGAGACATTGGGTTTTTATCCATGGAAACCAACCATA
CCAACTCCATGGAGATATTATTTTCAATGGGATAGAACATTAATACCATCTCATACTGGAACTAGTGG
CACACCAACAAATATATACCATGGTACAGATCCAGATGATGTTCAATTTTATACTATTGAAAATTCTG
TGCCAGTACACTTACTAAGAACAGGTGATGAATTTGCTACAGGAACATTTTTTTTTGATTGTAAACCA
TGTAGACTAACACATACATGGCAAACAAATAGAGCATTGGGCTTACCACCATTTCTAAATTCTTTGCC
TCAAGCTGAAGGAGGTACTAACTTTGGTTATATAGGAGTTCAACAAGATAAAAGACGTGGTGTAACTC
AAATGGGAAATACAAACTATATTACTGAAGCTACTATTATGAGACCAGCTGAGGTTGGTTATAGTGCA
CCATATTATTCTTTTGAGGCGTCTACACAAGGGCCATTTAAAACACCTATTGCAGCAGGACGGGGGGG
AGCGCAAACAGATGAAAATCAAGCAGCAGATGGTGATCCAAGATATGCATTTGGTAGACAACATGGTC
AAAAAACTACCACAACAGGAGAAACACCTGAGAGATTTACATATATAGCACATCAAGATACAGGAAGA
TATCCAGAAGGAGATTGGATTCAAAATATTAACTTTAACCTTCCTGTAACAGAAGATAATGTATTACT
ACCAACAGATCCAATTGGAGGTAAAACAGGAATTAACTATACTAATATATTTAATACTTATGGTCCTT
TAACTGCATTAAATAATGTACCACCAGTTTATCCAAATGGTCAAATTTGGGATAAAGAATTTGATACT
GACTTAAAACCAAGACTTCATGTAAATGCACCATTTGTTTGTCAAAATAATTGTCCTGGTCAATTATT
TGTAAAAGTTGCGCCTAATTTAACAAATGAATATGATCCTGATGCATCTGCTAATATGTCAAGAATTG
TAACTTACTCAGATTTTTGGTGGAAAGGTAAATTAGTATTTAAAGCTAAACTAAGAGCCTCTCATACT

TGGAATCCAATTCAACAAATGAGTATTAATGTAGATAACCAATTTAACTATGTACCAAGTAATATTGG
AGGTATGAAAATTGTATATGAAAAATCTCAACTAGCACCTAGA
'URU_KC196096_2c_M247_2010'
ATGAGTGATGGAGCAGTTCAACCAGACGGTGGTCAACCTGCTGTCAGAAATGAAAGAGCAACAGGATC
TGGGAACGGGTCTGGAGGCGGGGGTGGTGGTGGTTCTGGGGGTGTGGGGATTTCTACGGGTACTTTCA
ATAATCAGACGGAATTTAAATTTTTGGAAAACGGATGGGTGGAAATCACAGCAAACTCAAGCAGACTT
GTACATTTAAATATGCCAGAAAGTGAAAATTATAGAAGAGTGGTTGTAAATAATTTGGATAAAACTGC
AGTTAACGGAAACATGGCTTTAGATGATACTCATGCACAAATTGTAACACCTTGGTCATTGGTTGATG
CAAATGCTTGGGGAGTTTGGTTTAATCCAGGAGATTGGCAACTAATTGTTAATACTATGAGTGAGTTG
CATTTAGTTAGTTTTGAACAAGAAATTTTTAATGTTGTTTTAAAGACTGTTTCAGAATCTGCTACTCA
GCCACCAACTAAAGTTTATAATAATGATTTAACTGCATCATTGATGGTTGCATTAGATAGTAATAATA
CTATGCCATTTACTCCAGCAGCTATGAGATCTGAGACATTGGGTTTTTATCCATGGAAACCAACCATA
CCAACTCCATGGAGATATTATTTTCAATGGGATAGAACATTAATACCATCTCATACTGGAACTAGTGG
CACACCAACAAATATATACCATGGTACAGATCCAGATGATGTTCAATTTTATACTATTGAAAATTCTG
TGCCAGTACACTTACTAAGAACAGGTGATGAATTTGCTACAGGAACATTTTTTTTTGATTGTAAACCA
TGTAGACTAACACATACATGGCAAACAAATAGAGCATTGGGCTTACCACCATTTCTAAATTCTTTGCC
TCAAGCTGAAGGAGGTACTAACTTTGGTTATATAGGAGTTCAACAAGATAAAAGACGTGGTGTAACTC
AAATGGGAAATACAAACTATATTACTGAAGCTACTATTATGAGACCAGCTGAGGTTGGTTATAGTGCA
CCATATTATTCTTTTGAGGCGTCTACACAAGGGCCATTTAAAACACCTATTGCAGCAGGACGGGGGGG
AGCGCAAACAGATGAAAATCAAGCAGCAGATGGTGATCCAAGATATGCATTTGGTAGACAACATGGTC
AAAAAACTACCACAACAGGAGAAACACCTGAGAGATTTACATATATAGCACATCAAGATACAGGAAGA
TATCCAGAAGGAGATTGGATTCAAAATATTAACTTTAACCTTCCTGTAACAGAAGATAATGTATTGCT
ACCAACAGATCCAATTGGAGGTAAAACAGGAATTAACTATACTAATATATTTAATACTTATGGTCCTT
TAACTGCATTAAATAATGTACCACCAGTTTATCCAAATGGTCAAATTTGGGATAAAGAATTTGATACT
GACTTAAAACCAAGACTTCATGTAAATGCACCATTTGTTTGTCAAAATAATTGTCCCGGTCAATTATT
TGTAAAAGTTGCGCCTAATTTAACAAATGAATATGATCCTGATGCATCTGCTAATATGTCAAGAATTG
TAACTTACTCAGATTTTTGGTGGAAAGGTAAATTAGTATTTAAAGCTAAACTAAGAGCCTCTCATACT
TGGAATCCAATTCAACAAATGAGTATTAATGTAGATAACCAATTTAACTATGTACCAAGTAATATTGG
AGGTATGAAAATTGTATATGAAAAATCTCAACTAGCACCTAGA
'URU_KC196097_2c_M242_2010'
ATGAGTGATGGAGCAGTTCAACCAGACGGTGGTCAATCTGCTGTCAGAAATGAAAGAGCAACAGGATC
TGGGAACGGGTCTGGAGGCGGGGGTGGTGGTGGTTCTGGGGGTGTGGGGATTTCTACGGGTACTTTCA
ATAATCAGACGGAATTTAAATTTTTGGAAAACGGATGGGTGGAAATCACAGCAAACTCAAGCAGACTT
GTACATTTAAATATGCCAGAAAGTGAAAATTATAGAAGAGTGGTTGTAAATAATTTGGATAAAACTGC
AGTTAACGGAAACATGGCTTTAGATGATACTCATGCACAAATTGTAACACCTTGGTCATTGGTTGATG
CAAATGCTTGGGGAGTTTGGTTTAATCCAGGAGATTGGCAACTAATTGTTAATACTATGAGTGAGTTG
CATTTAGTTAGTTTTGAACAAGAAATTTTTAATGTTGTTTTAAAGACTGTTTCAGAATCTGCTACTCA
GCCACCAACTAAAGTTTATAATAATGATTTAACTGCATCATTGATGGTTGCATTAGATAGTAATAATA
CTATGCCATTTACTCCAGCAGCTATGAGATCTGAGACATTGGGTTTTTATCCATGGAAACCAACCATA
CCAACTCCATGGAGATATTATTTTCAATGGGATAGAACATTAATACCATCTCATACTGGAACTAGTGG
CACACCAACAAATATATACCATGGTACAGATCCAGATGATGTTCAATTTTATACTATTGAAAATTCTG
TGCCAGTACACTTACTAAGAACAGGTGATGAATTTGCTACAGGAACATTTTTTTTTGATTGTAAACCA
TGTAGACTAACACATACATGGCAAACAAATAGAGCATTGGGCTTACCACCATTTCTAAATTCTTTGCC
TCAAGCTGAAGGAGGTACTAACTTTGGTTATATAGGAGTTCAACAAGATAAAAGACGTGGTGTAACTC
AAATGGGAAATACAAACTATATTACTGAAGCTACTATTATGAGACCAGCTGAGGTTGGTTATAGTGCA
CCATATTATTCTTTTGAGGCGTCTACACAAGGGCCATTTAAAACACCTATTGCAGCAGGACGGGGGGG
AGCGCAAACAGATGAAAATCAAGCAGCAGATGGTGATCCAAGATATGCATTTGGTAGACAACATGGTC
AAAAAACTACCACAACAGGAGAAACACCTGAGAGATTTACATATATAGCACATCAAGATACAGGAAGA
TATCCAGAAGGAGATTGGATTCAAAATATTAACTTTAACCTTCCTGTAACAGAAGATAATGTATTACT
ACCAACAGATCCAATTGGAGGTAAAACAGGAATTAACTATACTAATATATTTAATACTTATGGTCCTT
TAACTGCATTAAATAATGTACCACCAGTTTATCCAAATGGTCAAATTTGGGATAAAGAATTTGATACT
GACTTAAAACCAAGACTTCATGTAAATGCACCATTTGTTTGTCAAAATAATTGTCCTGGTCAATTATT
TGTAAAAGTTGCGCCTAATTTAACAAATGAATATGATCCTGATGCATCTGCTAATATGTCAAGAATTG
TAACTTACTCAGATTTTTGGTGGAAAGGTAAATTAGTATTTAAAGCTAAACTAAGAGCCTCTCATACT

TGGAATCCAATTCAACAAATGAGTATTAATGTAGATAACCAATTTAACTATGTACCAAGTAATATTGG
AGGTATGAAAATTGTATATGAAAAATCTCAACTAGCACCTAGA
'URU_KC196101_2c_M187_2009'
ATGAGTGATGGAGCAGTTCAACCAGACGGTGGTCAACCTGCTGTCAGAAATGAAAGAGCAACAGGATC
TGGGAACGGGTCTGGAGGCGGGGGTGGTGGTGGTTCTGGGGGTGTGGGGATTTCTACGGGTACTTTCA
ATAATCAGACGGAATTTAAATTTTTGGAAAACGGATGGGTGGAAATCACAGCAAACTCAAGCAGACTT
GTACATTTAAATATGCCAGAAAGTGAAAATTATAGAAGAGTGGTTGTAAATAATTTGGATAAAACTGC
AGTTAACGGAAACATGGCTTTAGATGATACTCATGCACAAATTGTAACACCTTGGTCATTGGTTGATG
CAAATGCTTGGGGAGTTTGGTTTAATCCAGGAGATTGGCAACTAATTGTTAATACTATGAGTGAGTTG
CATTTAGTTAGTTTTGAACAAGAAATTTTTAATGTTGTTTTAAAGACTGTTTCAGAATCTGCTACTCA
GCCACCAACTAAAGTTTATAATAATGATTTAACTGCATCATTGATGGTTGCATTAGATAGTAATAATA
CTATGCCATTTACTCCAGCAGCTATGAGATCTGAGACATTGGGTTTTTATCCATGGAAACCAACCATA
CCAACTCCATGGAGATATTATTTTCAATGGGATAGAACATTAATACCATCTCATACTGGAACTAGTGG
CACACCAACAAATATATACCATGGTACAGATCCAGATGATGTTCAATTTTATACTATTGAAAATTCTG
TGCCAGTACACTTACTAAGAACAGGTGATGAATTTGCTACAGGAACATTTTTTTTTGATTGTAAACCA
TGTAGACTAACACATACATGGCAAACAAATAGAGCATTGGGCTTACCACCATTTCTAAATTCTTTGCC
TCAAGCTGAAGGAGGTACTAACTTTGGTTATATAGGAGTTCAACAAGATAAAAGACGTGGTGTAACTC
AAATGGGAAATACAAACTATATTACTGAAGCTACTATTATGAGACCAGCTGAGGTTGGTTATAGTGCA
CCATATTATTCTTTTGAGGCGTCTACACAAGGGCCATTTAAAACACCTATTGCAGCAGGACGGGGGGG
AGCGCAAACAGATGAAAATCAAGCAGCAGATGGTGATCCAAGATATGCATTTGGTAGACAACATGGTC
AAAAAACTACCACAACAGGAGAAACACCTGAGAGATTTACATATATAGCACATCAAGATACAGGAAGA
TATCCAGAAGGCGATTGGATTCAAAATATTAACTTTAACCTTCCTGTAACAGAAGATAATGTATTGCT
ACCAACAGATCCAATTGGAGGTAAAACAGGAATTAACTATACTAATATATTTAATACTTATGGTCCTT
TAACTGCATTAAATAATGTACCACCAGTTTATCCAAATGGTCAAATTTGGGATAAAGAATTTGATACT
GACTTAAAACCAAGACTTCATGTAAATGCACCATTTGTTTGTCAAAATAATTGTCCTGGTCAATTATT
TGTAAAAGTTGCGCCTAATTTAACAAATGAATATGATCCTGATGCATCTGCTAATATGTCAAGAATTG
TAACTTACTCAGATTTTTGGTGGAAAGGTAAATTAGTATTTAAAGCTAAACTAAGAGCCTCTCATACT
TGGAATCCAATTCAACAAATGAGTATTAATGTAGATAACCAATTTAACTATGTACCAAGTAATATTGG
AGGTATGAAAATTGTATATGAAAAATCTCAACTAGCACCTAGA
'URU_KC196102_2c_M185_2009'
ATGAGTGATGGAGCAGTTCAACCAGACGGTGGTCAACCTGCTGTCAGAAATGAAAGAGCAACAGGATC
TGGGAACGGGTCTGGAGGCGGGGGTGGTGGTGGTTCTGGGGGTGTGGGGATTTCTACGGGTACTTTCA
ATAATCAGACGGAATTTAAATTTTTGGAAAACGGATGGGTGGAAATCACAGCAAACTCAAGCAGACTT
GTACATTTAAATATGCCAGAAAGTGAAAATTATAGAAGAGTGGTTGTAAATAATTTGGATAAAACTGC
AGTTAACGGAAACATGGCTTTAGATGATACTCATGCACAAATTGTAACACCTTGGTCATTGGTTGATG
CAAATGCTTGGGGAGTTTGGTTTAATCCAGGAGATTGGCAACTAATTGTTAATACTATGAGTGAGTTG
CATTTAGTTAGTTTTGAACAAGAAATTTTTAATGTTGTTTTAAAGACTGTTTCAGAATCTGCTACTCA
GCCACCAACTAAAGTTTATAATAATGATTTAACTGCATCATTGATGGTTGCATTAGATAGTAATAATA
CTATGCCATTTACTCCAGCAGCTATGAGATCTGAGACATTGGGTTTTTATCCATGGAAACCAACCATA
CCAACTCCATGGAGATATTATTTTCAATGGGATAGAACATTAATACCATCTCATACTGGAACTAGTGG
CACACCAACAAATATATACCATGGTACAGATCCAGATGATGTTCAATTTTATACTATTGAAAATTCTG
TGCCAGTACACTTACTAAGAACAGGTGATGAATTTGCTACAGGAACATTTTTTTTTGATTGTAAACCA
TGTAGACTAACACATACATGGCAAACAAATAGAGCATTGGGCTTACCACCATTTCTAAATTCTTTGCC
TCAAGCTGAAGGAGGTACTAACTTTGGTTATATAGGAGTTCAACAAGATAAAAGACGTGGTGTAACTC
AAATGGGAAATACAAACTATATTACTGAAGCTACTATTATGAGACCAGCTGAGGTTGGTTATAGTGCA
CCATATTATTCTTTTGAGGCGTCTACACAAGGGCCATTTAAAACACCTATTGCAGCAGGACGGGGGGG
AGCGCAAACAGATGAAAATCAAGCAGCAGATGGTGATCCACGATATGCATTTGGTAGACAACATGGTC
AAAAAACTACCACAACAGGAGAAACACCTGAGAGATTTACATATATAGCACATCAAGATACAGGAAGA
TATCCAGAAGGAGATTGGATTCAAAATATTAACTTTAACCTTCCTGTAACAGAAGATAATGTATTGCT
ACCAACAGATCCAATTGGAGGTAAAACAGGAATTAACTATACTAATATATTTAATACTTATGGTCCTT
TAACTGCATTAAATAATGTACCACCAGTTTATCCAAATGGTCAAATTTGGGATAAAGAATTTGATACT
GACTTAAAACCAAGACTTCATGTAAATGCACCATTTGTTTGTCAAAATAATTGTCCTGGTCAATTATT
TGTAAAAGTTGCGCCTAATTTAACAAATGAATATGATCCTGATGCATCTGCTAATATGTCAAGAATTG
TAACTTACTCAGATTTTTGGTGGAAAGGTAAATTAGTATTTAAAGCTAAACTAAGAGCCTCTCATACT

TGGAATCCAATTCAACAAATGAGTATTAATGTAGATAACCAATTTAACTATGTACCAAGTAATATTGG
AGGTATGAAAATTGTATATGAAAAATCTCAACTAGCACCTAGA
'URU_KC196105_2c_M152_2008'
ATGAGTGATGGAGCAGTTCAACCAGACGGTGGTCAACCTGCTGTCAGAAATGAAAGAGCAACAGGATC
TGGGAACGGGTCTGGAGGCGGGGGTGGTGGTGGTTCTGGGGGTGTGGGGATTTCTACGGGTACTTTCA
ATAATCAGACGGAATTTAAATTTTTGGAAAACGGATGGGTGGAAATCACAGCAAACTCAAGCAGACTT
GTACATTTAAATATGCCAGAAAGTGAAAATTATAGAAGAGTGGTTGTAAATAATTTGGATAAAACTGC
AGTTAACGGAAACATGGCTTTAGATGATACTCATGCACAAATTGTAACACCTTGGTCATTGGTTGATG
CAAATGCTTGGGGAGTTTGGTTTAATCCAGGAGATTGGCAACTAATTGTTAATACTATGAGTGAGTTG
CATTTAGTTAGTTTTGAACAAGAAATTTTTAATGTTGTTTTAAAGACTGTTTCAGAATCTGCTACTCA
GCCACCAACTAAAGTTTATAATAATGATTTAACTGCATCATTGATGGTTGCATTAGATAGTAATAATA
CTATGCCATTTACTCCAGCAGCTATGAGATCTGAGACATTGGGTTTTTATCCATGGAAACCAACCATA
CCAACTCCATGGAGATATTATTTTCAATGGGATAGAACATTAATACCATCTCATACTGGAACTAGTGG
CACACCAACAAATATATACCATGGTACAGATCCAGATGATGTTCAATTTTATACTATTGAAAATTCTG
TGCCAGTACACTTACTAAGAACAGGTGATGAATTTGCTACAGGAACATTTTTTTTTGATTGTAAACCA
TGTAGACTAACACATACATGGCAAACAAATAGAGCATTGGGCTTACCACCATTTCTAAATTCTTTGCC
TCAAGCTGAAGGAGGTACTAACTTTGGCTATATAGGAGTTCAACAAGATAAAAGACGTGGTGTAACTC
AAATGGGAAATACAAACTATATTACTGAAGCTACTATTATGAGACCAGCTGAGGTTGGTTATAGTGCA
CCATATTATTCTTTTGAGGCGTCTACACAAGGGCCATTTAAAACACCTATTGCAGCAGGACGGGGGGG
AGCGCAAACAGATGAAAATCAAGCAGCAGATGGTGATCCAAGATATGCATTTGGTAGACAACATGGTC
AAAAAACTACCACAACAGGAGAAACACCTGAGAGATTTACATATATAGCACATCAAGATACAGGAAGA
TATCCAGAAGGAGATTGGATTCAAAATATTAACTTTAACCTTCCTGTAACAGAAGATAATGTATTGCT
ACCAACAGATCCAATTGGAGGTAAAACAGGAATTAACTATACTAATATGTTTAATACTTATGGTCCTT
TAACTGCATTAAATAATGTACCACCAGTTTATCCAAATGGTCAAATTTGGGATAAAGAATTTGATACT
GACTTAAAACCAAGACTTCATGTAAATGCACCATTTGTTTGTCAAAATAATTGTCCTGGTCAATTATT
TGTAAAAGTTGCGCCTAATTTAACAAATGAATATGATCCTGATGCATCTGCTAATATGTCAAGAATTG
TAACTTACTCAGATTTTTGGTGGAAAGGTAAATTAGTATTTAAAGCTAAACTAAGAGCCTCTCATACT
TGGAATCCAATTCAACAAATGAGTATTAATGTAGATAACCAATTTAACTATGTACCAAGTAATATTGG
AGGTATGAAAATTGTATATGAAAAATCTCAACTAGCACCTAGA
'URU_KC196107_2c_M129_2008'
ATGAGTGATGGAGCAGTTCAACCAGACGGTGGTCAACCTGCTGTCAGAAATGAAAGAGCAACAGGATC
TGGGAACGGGTCTGGAGGCGGGGGTGGTGGTGGTTCTGGGGGTGTGGGGATTTCTACGGGTACTTTCA
ATAATCAGACGGAATTTAAATTTTTGGAAAACGGATGGGTGGAAATCACAGCAAACTCAAGCAGACTT
GTACATTTAAATATGCCAGAAAGTGAAAATTATAGAAGAGTGGTTGTAAATAATTTGGATAAAACTGC
AGTTAACGGAAACATGGCTTTAGATGATACTCATGCACAAATTGTAACACCTTGGTCATTGGTTGATG
CAAATGCTTGGGGAGTTTGGTTTAATCCAGGAGATTGGCAACTAATTGTTAATACTATGAGTGAGTTG
CATTTAGTTAGTTTTGAACAAGAAATTTTTAATGTTGTTTTAAAGACTGTTTCAGAATCTGCTACTCA
GCCACCAACTAAAGTTTATAATAATGATTTAACTGCATCATTGATGGTTGCATTAGATAGTAATAATA
CTATGCCATTTACTCCAGCAGCTATGAGATCTGAGACATTGGGTTTTTATCCATGGAAACCAACCATA
CCAACTCCATGGAGATATTATTTTCAATGGGATAGAACATTAATACCATCTCATACTGGAACTAGTGG
CACACCAACAAATATATACCATGGTACAGATCCAGATGATGTTCAATTTTATACTATTGAAAATTCTG
TGCCAGTACACTTACTAAGAACAGGTGATGAATTTGCTACAGGAACATTTTTTTTTGATTGTAAACCA
TGTAGACTAACACATACATGGCAAACAAATAGAGCGTTGGGCTTACCACCATTTCTAAATTCTTTGCC
TCAAGCTGAAGGAGGTACTAACTTTGGTTATATAGGAGTTCAACAAGATAAAAGACGTGGTGTAACTC
AAATGGGAAATACAAACTATATTACTGAAGCTACTATTATGAGACCAGCTGAGGTTGGTTATAGTGCA
CCATATTATTCTTTTGAGGCGTCTACACAAGGGCCATTTAAAACACCTATTGCAGCAGGACGGGGGGG
AGCGCAAACAGATGAAAATCAAGCAGCAGATGGTGATCCAAGATATGCATTTGGTAGACAACATGGTC
AAAAAACTACCACAACAGGAGAAACACCTGAGAGATTTACATATATAGCACATCAAGATACAGGAAGA
TATCCAGAAGGAGATTGGATTCAAAATATTAACTTTAACCTTCCTGTAACAGAAGATAATGTATTGCT
ACCAACAGATCCAATTGGAGGTAAAACAGGAATTAACTATACTAATATATTTAATACTTATGGTCCTT
TAACTGCATTAAATAATGTACCACCAGTTTATCCAAATGGTCAAATTTGGGATAAAGAATTTGATACT
GACTTAAAACCAAGACTTCATGTAAATGCACCATTTGTCTGTCAAAATAATTGTCCTGGTCAATTATT
TGTAAAAGTTGCGCCTAATTTAACAAATGAATATGATCCTGATGCATCTGCTAATATGTCAAGAATTG
TAACTTACTCAGATTTTTGGTGGAAAGGTAAATTAGTATTTAAAGCTAAACTAAGAGCCTCTCATACT

TGGAATCCAATTCAACAAATGAGTATTAATGTAGATAACCAATTTAACTATGTACCAAGTAATATTGG
AGGTATGAAAATTGTATATGAAAAATCTCAACTAGCACCTAGA
'ECU_KF149962_2c_ME1_2012'
ATGAGTGATGGAGCAGTTCAACCAGACGGTGGTCAACCTGCTGTCAGAAATGAAAGAGCAACAGGATC
TGGGAACGGGTCTGGAGGCGGGGGTGGTGGTGGTTCTGGGGGTGTGGGGATTTCTACGGGTACTTTCA
ATAATCAGACGGAATTTAAATTTTTGGAAAACGGATGGGTGGAAATCACAGCAAACTCAAGCAGACTT
GTACATTTAAATATGCCAGAAAGTGAAAATTATAGAAGAGTGGTTGTAAATAATTTGGATAAAACTGC
AGTTAACGGAAACATGGCTTTAGATGATACTCATGCACAAATTGTAACACCTTGGTCATTGGTTGATG
CAAATGCTTGGGGAGTTTGGTTTAATCCAGGAGATTGGCAACTAATTGTTAATACTATGAGTGAGTTG
CATTTAGTTAGTTTTGAACAAGAAATTTTTAATGTTGTTTTAAAGACTGTTTCAGAATCTGCTACTCA
GCCACCAACTAAAGTTTATAATAATGATTTAACTGCATCATTGATGGTTGCATTAGATAGTAATAATA
CTATGCCATTTACTCCAGCAGCTATGAGATCTGAGACATTGGGTTTTTATCCATGGAAACCAACCATA
CCAACTCCATGGAGATATTATTTTCAATGGGATAGAACATTAATACCATCTCATACTGGAACTAGTGG
CACACCAACAAATATATACCATGGTACAGATCCAGATGATGTTCAATTTTATACTATTGAAAATTCTG
TGCCAGTACACTTACTAAGAACAGGTGATGAATTTGCTACAGGAACATTTTTTTTTGATTGTAAACCA
TGTAGACTAACACATACATGGCAAACAAATAGAGCATTGGGCTTACCACCATTTCTAAATTCTTTGCC
TCAAGCTGAAGGAGGTACTAACTTTGGTTATATAGGAGTTCAACAAGATAAAAGACGTGGTGTAACTC
AAATGGGAAATACAAACTATATTACTGAAGCTACTATTATGAGACCAGCTGAGGTTGGTTATAGTGCA
CCATATTATTCTTTTGAGGCGTCTACACAAGGGCCATTTAAAACACCTATTGCAGCAGGACGGGGGGG
AGCGCAAACAGATGAAAATCAAGCAGCAGATGGTGATCCAAGATATGCATTTGGTAGACAACATGGTC
AAAAAACTACCACAACAGGAGAAACACCTGAGAGATTTACATATATAGCACATCAAGATACAGGAAGA
TATCCAGAAGGAGATTGGATTCAAAATATTAACTTTAACCTTCCTGTAACAGAAGATAATGTATTGCT
ACCAACAGATCCAATTGGAGGTAAAACAGGAATTAACTATACTAATATATTTAATACCTATGGTCCTT
TAACTGCATTAAATAATGTACCACCAGTTTATCCAAATGGTCAAATTTGGGATAAAGAATTTGATACT
GACTTAAAACCAAGACTTCATGTAAATGCACCATTTGTTTGTCAAAATAATTGTCCTGGTCAATTATT
TGTAAAAGTTGCGCCTAATTTAACAAATGAATATGATCCTGATGCATCTGCTAATATGTCAAGAATTG
TAACTTACTCAGATTTTTGGTGGAAAGGTAAATTAGTATTTAAAGCTAAACTAAGAGCCTCTCATACT
TGGAATCCAATTCAACAAATGAGTATTAATGTAGATAACCAATTTAACTATGTACCAAGTAATATTGG
AGGTATGAAAATTGTATATGAAAAATCTCAACTAGCACCTAGA
'ECU_KF149963_2c_ME10_2012'
ATGAGTGATGGAGCAGTTCAACCAGACGGTGGTCAACCTGCTGTCAGAAATGAAAGAGCAACAGGATC
TGGGAACGGGTCTGGAGGCGGGGGTGGTGGTGGTTCTGGGGGTGTGGGGATTTCTACGGGTACTTTCA
ATAATCAGACGGAATTTAAATTTTTGGAAAACGGATGGGTGGAAATCACAGCAAACTCAAGCAGACTT
GTACATTTAAATATGCCAGAAAGTGAAAATTATAGAAGAGTGGTTGTAAATAATTTGGATAAAACTGC
AGTTAACGGAAACATGGCTTTAGATGATACTCATGCACAAATTGTAACACCTTGGTCATTGGTTGATG
CAAATGCTTGGGGAGTTTGGTTTAATCCAGGAGATTGGCAACTAATTGTTAATACTATGAGTGAGTTG
CATTTAGTTAGTTTTGAACAAGAAATTTTTAATGTTGTTTTAAAGACTGTTTCAGAATCTGCTACTCA
GCCACCAACTAAAGTTTATAATAATGATTTAACTGCATCATTGATGGTTGCATTAGATAGTAATAATA
CTATGCCATTTACTCCAGCAGCTATGAGATCTGAGACATTGGGTTTTTATCCATGGAAACCAACCATA
CCAACTCCATGGAGATATTATTTTCAATGGGATAGAACATTAATACCATCTCATACTGGAACTAGTGG
CACACCAACAAATATATACCATGGTACAGATCCAGATGATGTTCAATTTTATACTATTGAAAATTCTG
TGCCAGTACACTTACTAAGAACAGGTGATGAATTTGCTACAGGAACATTTTTTTTTGATTGTAAACCA
TGTAGACTAACACATACATGGCAAACAAATAGAGCATTGGGCTTACCACCATTTCTGAATTCTTTGCC
TCAAGCTGAAGGAGGTACTAACTTTGGTTATATAGGAGTTCAACAAGATAAAAGACGTGGTGTAACTC
AAATGGGAAATACAAACTATATTACTGAAGCTACTATTATGAGACCAGCTGAGGTTGGTTATAGTGCA
CCATATTATTCTTTTGAGGCGTCTACACAAGGGCCATTTAAAACACCTATTGCAGCAGGACGGGGGGG
AGCGCAAACAGATGAAAATCAAGCAGCAGATGGTGATCCAAGATATGCATTTGGTAGACAACATGGTC
AAAAAACTACCACAACAGGAGAAACACCTGAGAGATTTACATATATAGCACATCAAGATACAGGAAGA
TATCCAGAAGGAGATTGGATTCAAAATATTAACTTTAACCTTCCTGTAACAGAAGATAATGTATTGCT
ACCAACAGATCCAATTGGAGGTAAAACAGGAATTAACTATACTAATATATTTAATACCTATGGTCCTT
TAACTGCATTAAATAATGTACCACCAGTTTATCCAAATGGTCAAATTTGGGATAAAGAATTTGATACT
GACTTAAAACCAAGACTTCATGTAAATGCACCATTTGTTTGTCAAAATAATTGTCCTGGTCAATTATT
TGTAAAAGTTGCGCCTAATTTAACAAATGAATATGATCCTGATGCATCTGCTAATATGTCAAGAATTG
TAACTTACTCAGATTTTTGGTGGAAAGGTAAATTAGTATTTAAAGCTAAACTAAGAGCCTCTCATACT

TGGAATCCAATTCAACAAATGAGTATTAATGTAGATAACCAATTTAACTATGTACCAAGTAATATTGG
AGGTATGAAAATTGTATATGAAAAATCTCAACTAGCACCTAGA
'ECU_KF149964_2c_ME23_2012'
ATGAGTGATGGAGCAGTTCAACCAGACGGTGGTCAACCTGCTGTCAGAAATGAAAGAGCAACAGGATC
TGGGAACGGGTCTGGAGGCGGGGGTGGTGGTGGTTCTGGGGGTGTGGGGATTTCTACGGGTACTTTCA
ATAATCAGACGGAATTTAAATTTTTGGAAAACGGATGGGTGGAAATCACAGCAAACTCAAGCAGACTT
GTACATTTAAATATGCCAGAAAGTGAAAATTATAGAAGAGTGGTTGTAAATAATTTGGATAAAACTGC
AGTTAACGGAAACATGGCTTTAGATGATACTCATGCACAAATTGTAACACCTTGGTCATTGGTTGATG
CAAATGCTTGGGGAGTTTGGTTTAATCCAGGAGATTGGCAACTAATTGTTAATACTATGAGTGAGTTG
CATTTAGTTAGTTTTGAACAAGAAATTTTTAATGTTGTTTTAAAGACTGTTTCAGAATCTGCTACTCA
GCCACCAACTAAAGTTTATAATAATGATTTAACTGCATCATTGATGGTTGCATTAGATAGTAATAATA
CTATGCCATTTACTCCAGCAGCTATGAGATCTGAGACATTGGGTTTTTATCCATGGAAACCAACCATA
CCAACTCCATGGAGGTATTATTTTCAATGGGATAGAACATTAATACCATCTCATACTGGAACTAGTGG
CACACCAACAAATATATACCATGGTACAGATCCAGATGATGTTCAATTTTATACTATTGAAAATTCTG
TGCCAGTACACTTACTAAGAACAGGTGATGAATTTGCTACAGGAACATTTTTTTTTGATTGTAAACCA
TGTAGACTAACACATACATGGCAAACAAATAGAGCATTGGGCTTACCACCATTTCTAAATTCTTTGCC
TCAAGCTGAAGGAGGTACTAACTTTGGTTATATAGGAGTTCAACAAGATAAAAGACGTGGTGTAACTC
AAATGGGAAATACAAACTATATTACTGAAGCTACTATTATGAGACCAGCTGAGGTTGGTTATAGTGCA
CCATATTATTCTTTTGAGGCGTCTACACAAGGGCCATTTAAAACACCTATTGCAGCAGGACGGGGGGG
AGCGCAAACAGATGAAAATCAAGCAGCAGATGGTGATCCAAGATATGCATTTGGTAGACAACATGGTC
AAAAAACTACCACAACAGGAGAAACACCTGAGAGATTTACATATATAGCACATCAAGATACAGGAAGA
TATCCAGAAGGAGATTGGATTCAAAATATTAACTTTAACCTTCCTGTAACAGAAGATAATGTATTGCT
ACCAACAGATCCAATTGGAGGTAAAACAGGAATTAACTATACTAATATATTTAATACCTATGGTCCTT
TAACTGCATTAAATAATGTACCACCAGTTTATCCAAATGGTCAAATTTGGGATAAAGAATTTGATACT
GACTTAAAACCAAGACTTCATGTAAATGCACCATTTGTTTGTCAAAATAATTGTCCTGGTCAATTATT
TGTAAAAGTTGCGCCTAATTTAACAAATGAATATGATCCTGATGCATCTGCTAATATGTCAAGAATTG
TAACTTACTCAGATTTTTGGTGGAAAGGTAAATTAGTATTTAAAGCTAAACTAAGAGCCTCTCATACT
TGGAATCCAATTCAACAAATGAGTATTAATGTAGATAACCAATTTAACTATGTACCAAGTAATATTGG
AGGTATGAAAATTGTATATGAAAAATCTCAACTAGCACCTAGA
'ECU_KF149969_2c_ME31_2012'
ATGAGTGATGGAGCAGTTCAACCAGACGGTGGTCAACCTGCTGTCAGAAATGAAAGAGCAACAGGATC
TGGGAACGGGTCTGGAGGCGGGGGTGGTGGTGGTTCTGGGGGTGTGGGGATTTCTACGGGTACTTTCA
ATAATCAGACGGAATTTAAATTTTTGGAAAACGGATGGGTGGAAATCACAGCAAACTCAAGCAGACTT
GTACATTTAAATATGCCAGAAAGTGAAAATTATAGAAGAGTGGTTGTAAATAATTTGGATAAAACTGC
AGTTAACGGAAACATGGCTTTAGATGATACTCATGCACAAATTGTAACACCTTGGTCATTGGTTGATG
CAAATGCTTGGGGAGTTTGGTTTAATCCAGGAGATTGGCAACTAATTGTTAATACTATGAGTGAGTTG
CATTTAGTTAGTTTTGAACAAGAAATTTTTAATGTTGTTTTAAAGACTGTTTCAGAATCTGCTACTCA
GCCACCAACTAAAGTTTATAATAATGATTTAACTGCATCATTGATGGTTGCATTAGATAGTAATAATA
CTATGCCATTTACTCCAGCAGCTATGAGATCTGAGACATTGGGTTTTTATCCATGGAAACCAACCATA
CCAACTCCATGGAGATATTATTTTCAATGGGATAGAACATTAATACCATCTCATACTGGAACTAGTGG
CACACCAACAAATATATACCATGGTACAGATCCAGATGATGTTCAATTTTATACTATTGAAAATTCTG
TGCCAGTACACTTACTAAGAACAGGTGATGAATTTGCTACAGGAACATTTTTTTTTGATTGTAAACCA
TGTAGACTAACACATACATGGCAAACAAATAGAGCATTGGGCTTGCCACCATTTCTAAATTCTTTGCC
TCAAGCTGAAGGAGGTACTAACTTTGGTTATATAGGAGTTCAACAAGATAAAAGACGTGGTGTAACTC
AAATGGGAAATACAAACTATATTACTGAAGCTACTATTATGAGACCAGCTGAGGTTGGTTATAGTGCA
CCATATTATTCTTTTGAGGCGTCTACACAAGGGCCATTTAAAACACCTATTGCAGCAGGACGGGGGGG
AGCGCAAACAGATGAAAATCAAGCAGCAGATGGTGATCCAAGATATGCATTTGGTAGACAACATGGTC
AAAAAACTACCACAACAGGAGAAACACCTGAGAGATTTACATATATAGCACATCAAGATACAGGAAGA
TATCCAGAAGGAGATTGGATTCAAAATATTAACTTTAACCTTCCTGTAACAGAAGATAATGTATTGCT
ACCAACAGATCCAATTGGAGGTAAAACAGGAATTAACTATACTAATATATTTAATACCTATGGTCCTT
TAACTGCATTAAATAATGTACCACCAGTTTATCCAAATGGTCAAATTTGGGATAAAGAATTTGATACT
GACTTAAAACCAAGACTTCATGTAAATGCACCATTTGTTTGTCAAAATAATTGTCCTGGTCAATTATT
TGTAAAAGTTGCGCCTAATTTAACAAATGAATATGATCCTGATGCATCTGCTAATATGTCAAGAATTG
TAACTTACTCAGATTTTTGGTGGAAAGGTAAATTAGTATTTAAAGCTAAACTAAGAGCCTCTCATACT

TGGAATCCAATTCAACAAATGAGTATTAATGTAGATAACCAATTTAACTATGTACCAAGTAATATTGG
AGGTATGAAAATTGTATATGAAAAATCTCAACTAGCACCTAGA
'ECU_KF149971_2c_ME32_2012'
ATGAGTGATGGAGCAGTTCAACCAGACGGTGGTCAGCCTGCTGTCAGAAATGAAAGAGCTACAGGATC
TGGGAACGGGTCTGGAGGCGGGGGTGGTGGTGGTTCTGGGGGTGTGGGGATTTCTACGGGTACTTTCA
ATAATCAGACGGAATTTAAATTTTTGGAAAACGGATGGGTGGAAATCACAGCAAACTCAAGCAGACTT
GTACATTTAAATATGCCAGAAAGTGAAAATTATAGAAGAGTGGTTGTAAATAATTTGGATAAAACTGC
AGTTAACGGAAACATGGCTTTAGATGATACTCATGCACAAATTGTAACACCTTGGTCATTGGTTGATG
CAAATGCTTGGGGAGTTTGGTTTAATCCAGGAGATTGGCAACTAATTGTTAATACTATGAGTGAGTTG
CATTTAATTAGTTTTGAACAAGAAATTTTTAATGTTGTTTTAAAGACTGTTTCAGAATCTGCTACTCA
GCCACCAACTAAAGTTTATAATAATGATTTAACTGCATCATTGATGGTTGCATTAGATAGTAATAATA
CTATGCCATTTACTCCAGCAGCTATGAGATCTGAGACATTGGGTTTTTATCCATGGAAACCAACCATA
CCAACTCCATGGAGATATTATTTTCAATGGGATAGAACATTAATACCATCTCATACTGGAACTAGTGG
CACACCAACAAATATATACCATGGTACAGATCCAGATGATGTTCAATTTTATACTATTGAAAATTCTG
TGCCAGTACACTTGCTAAGAACAGGTGATGAATTTGCTACAGGAACATTTTTTTTTGATTGTAAACCA
TGCAGACTAACACATACATGGCAAACAAATAGAGCATTGGGCTTACCACCATTTCTAAATTCTTTGCC
TCAAGCTGAAGGAGGTACTAACTTTGGTTATATAGGAGTTCAACAAGATAAAAGACGTGGTGTAACTC
AAATGGGAAATACAAACTATATTACTGAAGCTACTATTATGAGACCAGCTGAGGTTGGTTATAGTGCA
CCATATTATTCTTTTGAGGCGTCTACACAAGGGCCATTTAAAACACCTATTGCAGCAGGACGGGGGGG
AGCGCAAACAGATGAAAATCAAGCAGCAGATGGTGATCCAAGATATGCATTTGGTAGACAACATGGTC
AAAAAACTACCACAACAGGAGAAACACCTGAGAGATTTACATATATAGCACATCAAGATACAGGAAGA
TATCCAGAAGGAGATTGGATTCAAAATATTAACTTTAACCTTCCTGTAACAGAAGATAATGTATTGCT
ACCAACAGATCCAATTGGAGGTAAAACAGGAATTAACTATACTAATATATTTAATACTTATGGTCCTT
TAACTGCATTAAATAATGTACCACCAGTTTATCCAAATGGTCAAATTTGGGATAAAGAATTTGATACT
GACTTAAAACCAAGACTTCATGTAAATGCACCATTTGTTTGTCAAAATAATTGTCCTGGTCAATTATT
TGTAAAAGTTGCGCCTAATTTAACAAATGAATATGATCCTGATGCATCTGCTAATATGTCAAGAATTG
TAACTTACTCAGATTTTTGGTGGAAAGGTAAATTAGTATTTAAAGCTAAACTAAGAGCCTCTCATACT
TGGAATCCAATTCAACAAATGAGTATTAATGTAGATAACCAATTTAACTATGTACCAAGTAATATTGG
AGGTATGAAAATTGTATATGAAAAATCTCAACTAGCACCTAGA
'ECU_KF149984_2c_ME28_2012'
ATGAGTGATGGAGCAGTTCAACCAGACGGTGGTCAACCTGCTGTCAGAAATGAAAGAGCAACAGGATC
TGGGAACGGGTCTGGAGGCGGGGGTGGTGGTGGTTCTGGGGGTGTGGGGATTTCTACGGGTACTTTCA
ATAATCAGACGGAATTTAAATTTTTGGAAAACGGATGGGTGGAAATCACAGCAAACTCAAGCAGACTT
GTACATTTAAATATGCCAGAAAGTGAAAATTATAGAAGAGTGGTTGTAAATAATTTGGATAAAACTGC
AGTTAACGGAAACATGGCTTTAGATGATACTCATGCACAAATTGTAACACCTTGGTCATTGGTTGATG
CAAATGCTTGGGGAGTTTGGTTTAATCCAGGAGATTGGCAACTAATTGTTAATACTATGAGTGAGTTG
CATTTAGTTAGTTTTGAACAAGAAATTTTTAATGTTGTTTTAAAGACTGTTTCAGAATCTGCTACTCA
GCCACCAACTAAAGTTTATAATAATGATTTAACTGCATCATTGATGGTTGCATTAGATAGTAATAATA
CTATGCCATTTACTCCAGCAGCTATGAGATCTGAGACATTGGGTTTTTATCCATGGAAACCAACCATA
CCAACTCCATGGAGATATTATTTTCAATGGGATAGAACATTAATACCATCTCATACTGGAACTAGTGG
CACACCAACAAATATATACCATGGTACAGATCCAGATGATGTTCAATTTTATACTATTGAAAATTCTG
TGCCAGTACACTTACTAAGAACAGGTGATGAATTTGCTACAGGAACATTTTTTTTTGATTGTAAACCA
TGTAGACTAACACATACATGGCAAACAAATAGAGCATTGGGCTTACCACCATTTCTAAATTCTTTGCC
TCAAGCTGAAGGAGGTACTAACTTTGGTTATATAGGAGTTCAACAAGATAAAAGACGTGGTGTAACTC
AAATGGGAAATACAAACTATATTACTGAAGCTACTATTATGAGACCAGCTGAGGTTGGTTATAGTGCA
CCATATTATTCTTTTGAGGCGTCTACACAAGGGCCATTTAAAACACCTATTGCAGCAGGACGGGGGGG
AGCACAAACAGATGAAAATCAAGCAGCAGATGGTGATCCAAGATATGCATTTGGTAGACAACATGGTC
AAAAAACTACCACAACAGGAGAAACACCTGAGAGATTTACATATATAGCACATCAAGATACAGGAAGA
TATCCAGAAGGAGATTGGATTCAAAATATTAACTTTAACCTTCCTGTAACAGAAGATAATGTATTGCT
ACCAACAGATCCAATTGGAGGTAAAACAGGAATTAACTATACTAATATATTTAATACTTATGGTCCTT
TAACTGCATTAAATAATGTACCACCAGTTTATCCAAATGGTCAAATTTGGGATAAAGAATTTGATACT
GACTTAAAACCAAGACTTCATGTAAATGCACCATTTGTTTGTCAAAATAATTGTCCTGGTCAATTATT
TGTAAAAGTTGCGCCTAATTTAACAAATGAATATGATCCTGATGCATCTGCTAATATGTCAAGAATTG
TAACTTACTCAGATTTTTGGTGGAAAGGTAAATTAGTATTTAAAGCTAAACTAAGAGCCTCTCATACT

TGGAATCCAATTCAACAAATGAGTATTAATGTAGATAACCAATTTAACTATGTACCAAGTAATATTGG
AGGTATGAAAATTGTATATGAAAAATCTCAACTAGCACCTAGA
'ITA_KF373571_2a_685_1999'
ATGAGTGATGGAGCAGTTCAACCAGACGGTGGTCAACCTGCTGTCAGAAATGAAAGAGCTACAGGATC
TGGGAACGGGTCTGGAGGCGGGGGTGGTGGTGGTTCTGGGGGTGTGGGGATTTCTACGGGTACTTTCA
ATAATCAGACGGAATTTAAATTTTTGGAAAACGGATGGGTGGAAATCACAGCAAACTCAAGCAGACTT
GTACATTTAAATATGCCAGAAAGTGAAAATTATAGAAGAGTGGTTGTAAATAATTTGGATAAAACTGC
AGTTAACGGAAACATGGCTTTAGATGATACTCATGCACAAATTGTAACACCTTGGTCATTGGTTGATG
CAAATGCTTGGGGAGTTTGGTTTAATCCAGGAGATTGGCAACTAATTGTTAATACTATGAGTGAGTTG
CATTTAGTTAGTTTTGAACAAGAAATTTTTAATGTTGTTTTAAAGACTGTTTCAGAATCTGCTACTCA
GCCACCAACTAAAGTTTATAATAATGATTTAACTGCATCATTGATGGTTGCATTAGATAGTAATAATA
CTATGCCATTTACTCCAGCAGCTATGAGATCTGAGACATTGGGTTTTTATCCATGGAAACCAACCATA
CCAACTCCATGGAGATATTATTTTCAATGGGATAGAACATTAATACCATCTCATACTGGAACTAGTGG
CACACCAACAAATATATACCATGGTACAGATCCAGATGATGTTCAATTTTATACTATTGAAAATTCTG
TGCCAGTACACTTACTAAGAACAGGTGATGAATTTGCTACAGGAACATTTTTTTTTGATTGTAAACCA
TGTAGACTAACACATACATGGCAAACAAATAGAGCATTGGGCTTACCACCATTTCTAAATTCTTTGCC
TCAAGCTGAAGGAGGTACTAACTTTGGTTATATAGGAGTTCAACAAGATAAAAGACGTGGTGTAACTC
AAATGGGAAATACAAACTATATTACTGAAGCTACTATTATGAGACCAGCTGAGGTTGGTTATAGTGCA
CCATATTATTCTTTTGAGGCGTCTACACAAGGGCCATTTAAAACACCTATTGCAGCAGGACGGGGGGG
AGCGCAAACAGATGAAAATCAAGCAGCAGATGGTGATCCAAGATATGCATTTGGTAGACAACATGGTC
AAAAAACTACCACAACAGGAGAAACACCTGAGAGATTTACATATATAGCACATCAAGATACAGGAAGA
TATCCAGAAGGAGATTGGATTCAAAATATTAACTTTAACCTTCCTGTAACAAATGATAATGTATTGCT
ACCAACAGATCCAATTGGAGGTAAAACAGGAATTAACTATACTAATATATTTAATACTTATGGTCCTT
TAACTGCATTAAATAATGTACCACCAGTTTATCCAAATGGTCAAATTTGGGATAAAGAATTTGATACT
GACTTAAAACCAAGACTTCATGTAAATGCACCATTTGTTTGTCAAAATAATTGTCCTGGTCAATTATT
TGTAAAAGTTGCGCCTAATTTAACAAATGAATATGATCCTGATGCATCTGCTAATATGTCAAGAATTG
TAACTTACTCAGATTTTTGGTGGAAAGGTAAATTAGTATTTAAAGCTAAACTAAGAGCCTCTCATACT
TGGAATCCAATTCAACAAATGAGTATTAATGTAGATAACCAATTTAACTATGTACCAAGTAATATTGG
AGGTATGAAAATTGTATATGAAAAATCTCAACTAGCACCTAGA
'ITA_KF373577_2a_714_2001'
ATGAGTGATGGAGCAGTTCAACCAGACGGTGGTCAACCTGCTGTCAGAAATGAAAGAGCTACAGGATC
TGGGAACGGGTCTGGAGGCGGGGGTGGTGGTGGTTCTGGGGGTGTGGGGATTTCTACGGGTACTTTCA
ATAATCAGACGGAATTTAAATTTTTGGAAAACGGATGGGTGGAAATCACAGCAAACTCAAGCAGACTT
GTACATTTAAATATGCCAGAAAGTGAAAATTATAGAAGAGTGGTTGTAAATAATTTGGATAAAACTGC
AGTTAACGGAAACATGGCTTTAGATGATACTCATGCACAAATTGTAACACCTTGGTCATTGGTTGATG
CAAATGCTTGGGGAGTTTGGTTTAATCCAGGAGATTGGCAACTAATTGTTAATACTATGAGTGAGTTG
CATTTAGTTAGTTTTGAACAAGAAATTTTTAATGTTGTTTTAAAGACTGTTTCAGAATCTGCTACTCA
GCCACCAACTAAAGTTTATAATAATGATTTAACTGCATCATTGATGGTTGCATTAGATAGCAATAATA
CTATGCCATTTACTCCAGCAGCTATGAGATCTGAGACATTGGGTTTTTATCCATGGAAACCAACCATA
CCAACTCCATGGAGATATTATTTTCAATGGGATAGAACATTAATACCATCTCATACTGGAACTAGTGG
CACACCAACAAATATATACCATGGTACAGATCCAGATGATGTTCAATTTTATACTATTGAAAATTCTG
TGCCAGTACACTTACTAAGAACAGGTGATGAATTTGCTACAGGAACATTTTTTTTTGATTGTAAACCA
TGTAGACTAACACATACATGGCAAACAAATAGAGCATTGGGCTTACCACCATTTCTAAATTCTTTGCC
TCAAGCTGAAGGAGGTACTAACTTTGGTTATATAGGAGTTCAACAAGATAAAAGACGTGGTGTAACTC
AAATGGGAAATACAAACTATATTACTGAAGCTACTATTATGAGACCAGCTGAGGTTGGTTATAGTGCA
CCATATTATTCTTTTGAGGCGTCTACACAAGGGCCATTTAAAACACCTATTGCAGCAGGACGGGGGGG
AGCGCAAACAGATGAAAATCAAGCAGCAGATGGTGATCCAAGATATGCATTTGGTAGACAACATGGTC
AAAAAACTACCACAACAGGAGAAACACCTGAGAGATTTACATATATAGCACATCAAGATACAGGAAGA
TATCCAGAAGGAGATTGGATTCAAAATATTAACTTTAACCTTCCTGTAACAAATGATAATGTATTGCT
ACCAACAGATCCAATTGGAGGTAAAACAGGAATTAACTATACTAATATATTTAATACTTATGGTCCTT
TAACTGCATTAAATAATGTACCACCAGTTTATCCAAATGGTCAAATTTGGGATAAAGAATTTGATACT
GACTTAAAACCAAGACTTCATGTAAATGCACCATTTGTTTGTCAAAATAATTGTCCTGGTCAATTATT
TGTAAAAGTTGCGCCTAATTTAACAAATGAATATGATCCTGATGCATCTGCTAATATGTCAAGAATTG
TAACTTACTCAGATTTTTGGTGGAAAGGTAAATTAGTATTTAAAGCTAAACTAAGAGCCTCTCATACT

TGGAATCCGATTCAACAAATGAGTATTAATGTAGATAACCAATTTAACTATGTACCAAGTAATATTGG
AGGTATGAAGATTGTATATGAAAAATCTCAACTAGCACCTAGA

'ITA_KF373580_2a_581_2003'

ATGAGTGATGGAGCAGTTCAACCAGACGGTGGTCAGCCTGCTGTCAGAAATGAAAGAGCTACAGGATC
TGGGAACGGGTCTGGAGGCGGGGGTGGTGGTGGTTCTGGGGGTGTGGGGATTTCTACGGGTACTTTCA
ATAATCAGACGGAATTTAAATTTTTGGAAAACGGATGGGTGGAAATCACAGCAAACTCAAGCAGACTT
GTACATTTAAATATGCCAGAAAGTGAAAATTATAGAAGAGTGGTTGTAAATAATTTGGATAAAACTGC
AGTTAACGGAAACATGGCTTTAGATGATACTCATGCACAAATTGTAACACCTTGGTCATTGGTTGATG
CAAATGCTTGGGGAGTTTGGTTTAATCCAGGAGATTGGCAACTAATTGTTAATACTATGAGTGAGTTG
CATTTAGTTAGTTTTGAACAAGAAATTTTTAATGTTGTTTTAAAGACTGTTTCAGAATCTGCTACTCA
GCCACCAACTAAAGTTTATAATAATGATTTAACTGCATCATTGATGGTTGCATTAGATAGCAATAATA
CTATGCCATTTACTCCAGCAGCTATGAGATCTGAGACATTGGGTTTTTATCCATGGAAACCAACCATA
CCAACTCCATGGAGATATTATTTTCAATGGGATAGAACATTAATACCATCTCATACTGGAACTAGTGG
CACACCAACAAATATATACCATGGTACAGATCCAGATGATGTTCAATTTTATACTATTGAAAATTCTG
TGCCAGTACACTTACTAAGAACAGGTGATGAATTTGCTACAGGAACATTTTTTTTTGATTGTAAACCA
TGTAGACTAACACATACATGGCAAACAAATAGAGCATTGGGCTTACCACCATTTCTAAATTCTTTGCC
TCAAGCTGAAGGAGGTACTAACTTTGGTTATATAGGAGTTCAACAAGATAAAAGACGTGGTGTAACTC
AAATGGGAAATACAAACTATATTACTGAAGCTACTATTATGAGACCAGCTGAGGTTGGTTATAGTGCA
CCATATTATTCTTTTGAGGCGTCTACACAAGGGCCATTTAAAACACCTATTGCAGCAGGACGGGGGGG
AGCGCAAACAGATGAAAATCAAGCAGCAGATGGTGATCCAAGATATGCATTTGGTAGACAACATGGTC
AAAAAACTACCACAACAGGAGAAACACCTGAGAGATTTACATATATAGCACATCAAGATACAGGAAGA
TATCCAGAAGGAGATTGGATTCAAAATATTAACTTTAACCTTCCTGTAACAAATGATAATGTATTGCT
ACCAACAGATCCAATTGGAGGTAAAACAGGAATTAACTATACTAATATATTTAATACTTATGGTCCTT
TAACTGCATTAAATAATGTACCACCAGTTTATCCAAATGGTCAAATTTGGGATAAAGAATTTGATACT
GACTTAAAACCAAGACTTCATGTAAATGCACCATTTGTTTGTCAAAATAATTGTCCTGGTCAATTATT
TGTAAAAGTTGCGCCTAATTTAACAAATGAATATGATCCTGATGCATCTGCTAATATGTCAAGAATTG
TAACTTACTCAGATTTTTGGTGGAAAGGTAAATTAGTATTTAAAGCTAAACTAAGAGCCTCTCATACT
TGGAATCCAATTCAACAAATGAGTATTAATGTAGATAACCAATTTAACTATGTACCAAGTAATATTGG
AGGTATGAAGATTGTATATGAAAAATCTCAACTAGCACCTAGA

'ITA_KF373592_2a_329_2008'

ATGAGTGATGGAGCAGTTCAACCAGACGGTGGTCAATCTGCTGTCAGAAATGAAAGAGCTACAGGATC
TGGGAACGGGTCTGGAGGCGGGGGTGGTGGTGGTTCTGGGGGTGTGGGGATTTCTACGGGTACTTTCA
ATAATCAGACGGAATTTAAATTTTTGGAAAACGGATGGGTGGAAATCACAGCAAACTCAAGCAGACTT
GTACATTTAAATATGCCAGAAAGTGAAAATTATAGAAGAGTGGTTGTAAATAATTTGGATAAAACTGC
AGTTAACGGAAACATGGCTTTAGATGATACTCATGCACAAATTGTAACACCTTGGTCATTGGTTGATG
CAAATGCTTGGGGAGTTTGGTTTAATCCAGGAGATTGGCAACTAATTGTTAATACTATGAGTGAGTTG
CATTTAGTTAGTTTTGAACAAGAAATTTTTAATGTTGTTTTAAAGACTGTTTCAGAATCTGCTACTCA
GCCACCAACTAAAGTTTATAATAATGATTTAACTGCATCATTGATGGTTGCATTAGATAGCAATAATA
CTATGCCATTTACTCCAGCAGCTATGAGATCTGAGACATTGGGTTTTTATCCATGGAAACCAACCATA
CCAACTCCATGGAGATATTATTTTCAATGGGATAGAACATTAATACCATCTCATACTGGAACTAGTGG
CACACCAACAAATATATACCATGGTACAGATCCAGATGATGTTCAATTTTATACTATTGAAAATTCTG
TGCCAGTACACTTACTAAGAACAGGTGATGAATTTGCTACAGGAACATTTTTTTTTGATTGTAAACCA
TGTAGACTAACACATACATGGCAAACAAATAGAGCATTGGGCTTACCACCATTTCTAAATTCTTTGCC
TCAAGCTGAAGGAGGTACTAACTTTGGTTATATAGGAGTTCAACAAGATAAAAGACGTGGTGTAACTC
AAATGGGAAATACAAACTATATTACTGAAGCTACTATTATGAGACCAGCTGAGGTTGGTTATAGTGCA
CCATATTATTCTTTTGAGGCGTCTACACAAGGGCCATTTAAAACACCTATTGCAGCAGGACGGGGGGG
AGCGCAAACAGATGAAAATCAAGCAGCAGATGGTGATCCAAGATATGCATTTGGTAGACAACATGGTC
AAAAAACTACCACAACAGGAGAAACACCTGAGAGATTTACATATATAGCACATCAAGATACAGGAAGA
TATCCAGAAGGAGATTGGATTCAAAATATTAACTTTAACCTTCCTGTAACAAATGATAATGTATTGCT
ACCAACAGATCCAATTGGAGGTAAAACAGGAATTAACTATACTAATATATTTAATACTTATGGTCCTT
TAACTGCATTAAATAATGTACCACCAGTTTATCCAAATGGTCAAATTTGGGATAAAGAATTTGATACT
GACTTAAAACCAAGACTTCATGTAAATGCACCATTTGTTTGTCAAAATAATTGTCCTGGTCAATTATT
TGTAAAAGTTGCGCCTAATTTAACAAATGAATATGATCCTGATGCATCTGCTAATATGTCAAGAATTG
TAACTTACTCAGATTTTTGGTGGAAAGGTAAATTAGTATTTAAAGCTAAACTAAGAGCCTCTCATACT

TGGAATCCAATTCAACAAATGAGTATTAATGTAGATAACCAATTTAACTATGTACCAAGTAATATTGG
AGGTATGAAGATTGTATATGAAAAATCTCAACTAGCACCTAGA
'ITA_KF373611_2a_409_2010'
ATGAGTGATGGAGCAGTTCAACCAGACGGTGGTCAACCTGCTGTCAGAAATGAAAGAGCTACAGGATC
TGGGAACGGGTCTGGAGGCGGGGGTGGTGGTGGTTCTGGGGGTGTGGGGATTTCTACGGGTACTTTCA
ATAATCAGACGGAATTTAAATTTTTGGAAAACGGATGGGTGGAAATCACAGCAAACTCAAGCAGACTT
GTACATTTAAATATGCCAGAAAGTGAAAATTATAGAAGAGTGGTTGTAAATAATTTGGATAAAACTGC
AGTTAACGGAAACATGGCTTTAGATGATACTCATGCACAAATTGTAACACCTTGGTCATTGGTTGATG
CAAATGCTTGGGGAGTTTGGTTTAATCCAGGAGATTGGCAACTAATTGTTAATACTATGAGTGAGTTG
CATTTAGTTAGTTTTGAACAAGAAATTTTTAATGTTGTTTTAAAGACTGTTTCAGAATCTGCTACTCA
GCCACCAACTAAAGTTTATAATAATGATTTAACTGCATCATTGATGGTTGCATTAGATAGTAATAATA
CTATGCCATTTACTCCAGCAGCTATGAGATCTGAGACATTGGGTTTTTATCCATGGAAACCAACCATA
CCAACTCCATGGAGATATTATTTTCAATGGGATAGAACATTAATACCATCTCATACTGGAACTAGTGG
CACACCAACAAATATATACCATGGTACAGATCCAGATGATGTTCAATTTTATACTATTGAAAATTCTG
TGCCAGTACACTTACTAAGAACAGGTGATGAATTTGCTACAGGAACATTTTTTTTTGATTGTAAACCA
TGTAGACTAACACATACATGGCAAACAAATAGAGCATTGGGCTTACCACCATTTCTAAATTCTTTGCC
TCAAGCTGAAGGAGGTACTAACTTTGGTTATATAGGAGTTCAACAAGATAAAAGACGTGGTGTAACTC
AAATGGGAAATACAAACTATATTACTGAAGCTACTATTATGAGACCAGCTGAGGTTGGTTATAGTGCA
CCATATTATTCTTTTGAGGCGTCTACACAAGGGCCATTTAAAACACCTATTGCAGCAGGACGGGGGGG
AGCGCAAACAGATGAAAATCAAGCAGCAGATGGTGATCCAAGATATGCATTTGGTAGACAACATGGTC
AAAAAACTACCACAACAGGAGAAACACCTGAGAGATTTACATATATAGCACATCAAGATACAGGAAGA
TATCCAGAAGGAGATTGGATTCAAAATATTAACTTTAACCTTCCTGTAACAAATGATAATGTATTGCT
ACCAACAGATCCAATTGGAGGTAAAACAGGAATTAACTATACTAATATATTTAATACTTATGGTCCTT
TAACTGCATTAAATAATGTACCACCAGTTTATCCAAATGGTCAAATTTGGGATAAAGAATTTGATACT
GACTTAAAACCAAGACTTCATGTAAATGCACCATTTGTTTGTCAAAATAATTGTCCTGGTCAATTATT
TGTAAAAGTTGCGCCTAATTTAACAAATGAATATGATCCTGATGCATCTGCTAATATGTCAAGAATTG
TAACTTACTCAGATTTTTGGTGGAAAGGTAAATTAGTATTTAAAGCTAAACTAAGAGCCTCTCATACT
TGGAATCCAATTCAACAAATGAGTATTAATGTAGATAACCAATTTAACTATGTACCAAGTAATATTGG
AGGTATGAAGATTGTATATGAAAAATCTCAACTAGCACCTAGA
'ITA_KF385388_2a_Sicily_X83090_2009'
ATGAGTGATGGAGCAGTTCAACCAGACGGTGGTCAACCTGCTGTCAGAAATGAAAGAGCTACAGGATC
TGGGAACGGGTCTGGAGGCGGGGGTGGTGGTGGTTCTGGGGGTGTGGGGATTTCTACGGGTACTTTCA
ATAATCAGACGGAATTTAAATTTTTGGAAAACGGATGGGTGGAAATCACAGCAAACTCAAGCAGACTT
GTACATTTAAATATGCCAGAAAGTGAAAATTATAGAAGAGTGGTTGTAAATAATTTGGATAAAACTGC
AGTTAACGGAAACATGGCTTTAGATGATACTCATGCACAAATTGTAACACCTTGGTCATTGGTTGATG
CAAATGCTTGGGGAGTTTGGTTTAATCCAGGAGATTGGCAACTAATTGTTAATACTATGAGTGAGTTG
CATTTAGTTAGTTTTGAACAAGAAATTTTTAATGTTGTTTTAAAGACTGTTTCAGAATCTGCTACTCA
GCCACCAACTAAAGTTTATAATAATGATTTAACTGCATCATTGATGGTTGCATTAGATAGCAATAATA
CTATGCCATTTACTCCAGCAGCTATGAGATCTGAGACATTGGGTTTTTATCCATGGAAACCAACCATA
CCAACTCCATGGAGATATTATTTTCAATGGGATAGAACATTAATACCATCTCATACTGGAACTAGTGG
CACACCAACAAATATATACCATGGTACAGATCCAGATGATGTTCAATTTTATACTATTGAAAATTCTG
TGCCAGTACACTTACTAAGAACAGGTGATGAATTTGCTACAGGAACATTTTTTTTTGATTGTAAACCA
TGTAGACTAACACATACATGGCAAACAAATAGAGCATTGGGCTTACCACCATTTCTAAATTCTTTGCC
TCAAGCTGAAGGAGGTACTAACTTTGGTTATATAGGAGTTCAACAAGATAAAAGACGTGGTGTAACTC
AAATGGGAAATACAAACTATATTACTGAAGCTACTATTATGAGACCAGCTGAGGTTGGTTATAGTGCA
CCATATTATTCTTTTGAGGCGTCTACACAAGGGCCATTTAAAACACCTATTGCAGCAGGACGGGGGGG
AGCGCAAACAGATGAAAATCAAGCAGCAGATGGTGATCCAAGATATGCATTTGGTAGACAACATGGTC
AAAAAACTACCACAACAGGAGAAACACCTGAGAGATTTACATATATAGCACATCAAGATACAGGAAGA
TATCCAGAAGGAGATTGGATTCAAAATATTAACTTTAACCTTCCTGTAACAAATGATAATGTATTGCT
ACCAACAGATCCAATTGGAGGTAAATCAGGAATTAACTATACTAATATATTTAATACTTATGGTCCTT
TAACTGCATTAAATAATGTACCACCAGTTTATCCAAATGGTCAAATTTGGGATAAAGAATTTGATACT
GACTTAAAACCAAGACTTCATGTAAATGCACCATTTGTTTGTCAAAATAATTGTCCTGGTCAATTATT
TGTAAAAGTTGCGCCTAATTTAACAAATGAATATGATCCTGATGCATCTGCTAATATGTCAAGAATTG
TAACTTACTCAGATTTTTGGTGGAAAGGTAAATTAGTATTTAAAGCTAAACTAAGAGCCTCTCATACT

TGGAATCCAATTCAACAAATGAGTATTAATGTAGATAACCAATTTAACTATGTACCAAGTAATATTGG
AGGTATGAAGATTGTATATGAAAAATCTCAACTAGCACCTAGA

'HUN_KF539793_H_5_2012'

ATGAGTGATGGAGCAGTTCAACCAGACGGTGGTCAACCTGCTGTCAGAAATGAAAGAGCTACAGGATC
TGGGAACGGGTCTGGAGGCGGGGGTGGTGGTGGTTCTGGGGGTGTGGGGATTTCTACGGGTACTTTTA
ATAATCAGACGGAATTTAAATTTTTGGAAAACGGATGGGTGGAAATCACAGCAAACTCAAGCAGACTT
GTACATTTAAATATGCCAGAAAGTGAAAATTATAGAAGAGTGGTTGTAAATAATTTGGATAAAACTGC
AGTTAACGGAAACATGGCTTTAGATGATACTCATGCACAAATTGTAACACCTTGGTCATTGGTTGATG
CAAATGCTTGGGGAGTTTGGTTTAATCCAGGAGATTGGCAACTAATTGTTAATACTATGAGTGAGTTG
CATTTAGTTAGTTTTGAACAAGAAATTTTTAATGTTGTTTTAAAGACTGTTTCAGAATCTGCTACTCA
GCCACCAACTAAAGTTTATAATAATGATTTAACTGCATCATTGATGGTTGCATTAGATAGCAATAATA
CTATGCCATTTACTCCAGCAGCTATGAGATCTGAGACATTGGGTTTTTATCCATGGAAACCAACCATA
CCAACTCCATGGAGATATTATTTTCAATGGGATAGAACATTAATACCATCTCATACTGGAACTAGTGG
CACACCAACAAATATATACCATGGTACAGATCCAGATGATGTTCAATTTTATACTATTGAAAATTCTG
TGCCAGTACACTTACTAAGAACAGGTGATGAATTTGCTACAGGAACATTTTTTTTTGATTGTAAACCA
TGTAGACTAACACATACATGGCAAACAAATAGAGCATTGGGCTTACCACCATTTCTAAATTCTTTGCC
TCAAGCTGAAGGAGGTACTAACTTTGGTTATATAGGAGTTCAACAAGATAAAAGACGTGGTGTAACTC
AAATGGGAAATACAAACTATATTACTGAAGCTACTATTATGAGACCAGCTGAGGTTGGTTATAGTGCA
CCATATTATTCTTTTGAGGCGTCTACACAAGGGCCATTTAAAACACCTATTGCAGCAGGACGGGGGGG
AGCGCAAACAGATGAAAATCAAGCAGCAGATGGTGATCCAAGATATGCATTTGGTAGACAACATGGTC
AAAAAACTACCACAACAGGAGAAACACCTGAGAGATTTACATATATAGCACATCAAGATACAGGAAGA
TATCCAGAAGGAGATTGGATTCAAAATATTAACTTTAACCTTCCTGTAACAAATGATAATGTATTGCT
ACCAACAGATCCAATTGGAGGTAAAACAGGAATTAACTATACTAATATATTTAATACTTATGGTCCTT
TAACTGCATTAAATAATGTACCACCAGTTTATCCAAATGGTCAAATTTGGGATAAAGAATTTGATACT
GACTTAAAACCAAGACTTCATGTAAATGCACCATTTGTTTGTCAAAATAATTGTCCTGGTCAATTATT
TGTAAAAGTTGCGCCTAATTTAACAAATGAATATGATCCTGATGCATCTACTAATATGTCAAGAATTG
TAACTTACTCAGATTTTTGGTGGAAAGGTAAATTAGTATTTAAAGCTAAACTAAGAGCCTCTCATACT
TGGAATCCAATTCAACAAATGAGTATTAATGTAGATAACCAATTTAACTATGTACCAAGTAATATTGG
AGGTATGAAGATTGTATATGAAAAATCTCAACTAGCACCTAGA

'HUN_KF539794_H_7_2012'

ATGAGTGATGGAGCAGTTCAACCAGACGGTGGTCAACCTGCTGTCAGAAATGAAAGAGCTACAGGATC
TGGGAACGGGTCTGGAGGCGGGGGTGGTGGTGGTTCTGGGGGTGTGGGGATTTCTACGGGTACTTTCA
ATAATCAGACGGAATTTAAATTTTTGGAAAACGGATGGGTGGAAATCACAGCAAACTCAAGCAGACTT
GTACATTTAAATATGCCAGAAAGTGAAAATTATAGAAGAGTGGTTGTAAATAATTTGGATAAAACTGC
AGTTAACGGAAACATGGCTTTAGATGATACTCATGCACAAATTGTAACACCTTGGTCATTGGTTGATG
CAAATGCTTGGGGAGTTTGGTTTAATCCAGGAGATTGGCAACTAATTGTTAATACTATGAGTGAGTTG
CATTTAGTTAGTTTTGAACAAGAAATTTTTAATGTTGTTTTAAAGACTGTTTCAGAATCTGCTACTCA
GCCACCAACTAAAGTTTATAATAATGATTTAACTGCATCATTGATGGTTGCATTAGATAGCAATAATA
CTATGCCATTTACTCCAGCAGCTATGAGATCTGAGACATTGGGTTTTTATCCATGGAAACCAACCATA
CCAACTCCATGGAGATATTATTTTCAATGGGATAGAACATTAATACCATCTCATACTGGAACTAGTGG
CACACCAACAAATATATACCATGGTACAGATCCAGATGATGTTCAATTTTATACTATTGAAAATTCTG
TGCCAGTACACTTACTAAGAACAGGTGATGAATTTGCTACAGGAACATTTTTTTTTGATTGTAAACCA
TGTAGACTAACACATACATGGCAAACAAATAGAGCATTGGGCTTACCACCATTTCTAAATTCTTTGCC
TCAAGCTGAAGGAGGTACTAACTTTGGTTATATAGGAGTTCAACAAGATAAAAGACGTGGTGTAACTC
AAATGGGAAATACAAACTATATTACTGAAGCTACTATTATGAGACCAGCTGAGGTTGGTTATAGTGCA
CCATATTATTCTTTTGAGGCGTCTACACAAGGGCCATTTAAAACACCTATTGCAGCAGGACGGGGGGG
AGCGCAAACAGATGAAAATCAAGCAGCAGATGGTGATCCAAGATATGCATTTGGTAGACAACATGGTC
AAAAAACTACCACAACAGGAGAAACACCTGAGAGATTTACATATATAGCACATCAAGATACAGGAAGA
TATCCAGAAGGAGATTGGATTCAAAATATTAACTTTAACCTTCCTGTAACAAATGATAATGTATTGCT
ACCAACAGATCCAATTGGAGGTAAAACAGGAATTAACTATACTAATATATTTAATACTTATGGTCCTT
TAACTGCATTAAATAATGTACCACCAGTTTATCCAAATGGTCAAATTTGGGATAAAGAATTTGATACT
GACTTAAAACCAAGACTTCATGTAAATGCACCATTTGTTTGTCAAAATAATTGTCCTGGTCAATTATT
TGTAAAAGTTGCGCCTAATTTAACAAATGAATATGATCCTGATGCATCTACTAATATGTCAAGAATTG
TAACTTACTCAGATTTTTGGTGGAAAGGTAAATTAGTATTTAAAGCTAAACTAAGAGCCTCTCATACT

TGGAATCCAATTCAACAAATGAGTATTAATGTAGATAACCAATTTAACTATGTACCAAGTAATATTGG
AGGTATGAAGATTGTATATGAAAAATCTCAACTAGCACCTAGA
'HUN_KF539795_H_8_2012'
ATGAGTGATGGAGCAGTTCAACCAGACGGTGGTCAACCTGCTGTCAGAAATGAAAGAGCTACAGGATC
TGGGAACGGGTCTGGAGGCGGGGGTGGTGGTGGTTCTGGGGGTGTGGGGATTTCTACGGGTACTTTCA
ATAATCAGACGGAATTTAAATTTTTGGAAAACGGATGGGTGGAAATCACAGCAAACTCAAGCAGACTT
GTACATTTAAATATGCCAGAAAGTGAAAATTATAGAAGAGTGGTTGTAAATAATTTGGATAAAACTGC
AGTTAACGGAAACATGGCTTTAGATGATACTCATGCACAAATTGTAACACCTTGGTCATTGGTTGATG
CAAATGCTTGGGGAGTTTGGTTTAATCCAGGAGATTGGCAACTAATTGTTAATACTATGAGTGAGTTG
CATTTAGTTAGTTTTGAACAAGAAATTTTTAATGTTGTTTTAAAGACTGTTTCAGAATCAGCTACTCA
GCCACCAACTAAAGTTTATAATAATGATTTAACTGCATCATTGATGGTTGCATTAGATAGCAATAATA
CTATGCCATTTACTCCAGCAGCTATGAGATCTGAGACATTGGGTTTTTATCCATGGAAACCAACCATA
CCAACTCCATGGAGATATTATTTTCAATGGGATAGAACATTAATACCATCTCATACTGGAACTAGTGG
CACACCAACAAATATATACCATGGTACAGATCCAGATGATGTTCAATTTTATACTATTGAAAATTCTG
TGCCAGTACACTTACTAAGAACAGGTGATGAATTTGCTACAGGAACATTTTTTTTTGATTGTAAACCA
TGTAGACTAACACATACATGGCAAACAAATAGAGCATTGGGCTTACCACCATTTCTAAATTCTTTGCC
TCAAGCTGAAGGAGGTACTAACTTTGGTTATATAGGAGTTCAACAAGATAAAAGACGTGGTGTAACTC
AAATGGGAAATACAAACTATATTACTGAAGCTACTATTATGAGACCAGCTGAGGTTGGTTATAGTGCA
CCATATTATTCTTTTGAGGCGTCTACGCAAGGGCCATTTAAAACACCTATTGCAGCAGGACGGGGGGG
AGCGCAAACAGATGAAAATCAAGCAGCAGATGGTGATCCAAGATATGCATTTGGTAGACAACATGGTC
AAAAAACTACCACAACAGGAGAAACACCTGAGAGATTTACATATATAGCACATCAAGATACAGGAAGA
TATCCAGAAGGAGATTGGATTCAAAATATTAACTTTAACCTTCCTGTAACAAATGATAATGTATTGCT
ACCAACAGATCCAATTGGAGGTAAAACAGGAATTAACTATACTAATATATTTAATACTTATGGTCCTT
TAACTGCATTAAATAATGTACCACCAGTTTATCCAAATGGTCAAATTTGGGATAAAGAATTTGATACT
GACTTAAAACCAAGACTTCATGTAAATGCACCATTTGTTTGTCAAAATAATTGTCCTGGTCAATTATT
TGTAAAAGTTGCGCCTAATTTAACAAATGAATATGATCCTGATGCATCTACTAATATGTCAAGAATTG
TAACTTACTCAGATTTTTGGTGGAAAGGTAAATTAGTATTTAAAGCTAAACTAAGAGCCTCTCATACT
TGGAATCCAATTCAACAAATGAGTATTAATGTAGATAACCAATTTAACTATGTACCAAGTAATATTGG
AGGTATGAAGATTGTATATGAAAAATCTCAACTAGCACCTAGA
'HUN_KF539796_H_9_2012'
ATGAGTGATGGAGCAGTTCAACCAGACGGTGGTCAACCTGCTGTCAGAAATGAAAGAGCTACAGGATC
TGGGAACGGGTCTGGAGGCGGGGGTGGTGGTGGTTCTGGGGGTGTGGGGATTTCTACGGGTACTTTTA
ATAATCAGACGGAATTTAAATTTTTGGAAAACGGATGGGTGGAAATCACAGCAAACTCAAGCAGACTT
GTACATTTAAATATGCCAGAAAGTGAAAATTATAGAAGAGTGGTTGTAAATAATTTGGATAAAACTGC
AGTTAACGGAAACATGGCTTTAGATGATACTCATGCACAAATTGTAACACCTTGGTCATTGGTTGATG
CAAATGCTTGGGGAGTTTGGTTTAATCCAGGAGATTGGCAACTAATTGTTAATACTATGAGTGAGTTG
CATTTAGTTAGTTTTGAACAAGAAATTTTTAATGTTGTTTTAAAGACTGTTTCAGAATCTGCTACTCA
GCCACCAACTAAAGTTTATAATAATGATTTAACTGCATCATTGATGGTTGCATTAGATAGCAATAATA
CTATGCCATTTACTCCAGCAGCTATGAGATCTGAGACATTGGGTTTTTATCCATGGAAACCAACCATA
CCAACTCCATGGAGATATTATTTTCAATGGGATAGAACATTAATACCATCTCATACTGGAACTAGTGG
CACACCAACAAATATATACCATGGTACAGATCCAGATGATGTTCAATTTTATACTATTGAAAATTCTG
TGCCAGTACACTTACTAAGAACAGGTGATGAATTTGCTACAGGAACATTTTTTTTTGATTGTAAACCA
TGTAGACTAACACATACATGGCAAACAAATAGAGCATTGGGCTTACCACCATTTCTAAATTCTTTGCC
TCAAGCTGAAGGAGGTACTAACTTTGGTTATATAGGAGTTCAACAAGATAAAAGACGTGGTGTAACTC
AAATGGGAAATACAAACTATATTACTGAAGCTACTATTATGAGACCAGCTGAGGTTGGTTATAGTGCA
CCATATTATTCTTTTGAGGCGTCTACACAAGGGCCATTTAAAACACCTATTGCAGCAGGACGGGGGGG
AGCGCAAACAGATGAAAATCAAGCAGCAGATGGTGATCCAAGATATGCATTTGGTAGACAACATGGTC
AAAAAACTACCACAACAGGAGAAACACCTGAGAGATTTACATATATAGCACATCAAGATACAGGAAGA
TATCCAGAAGGAGATTGGATTCAAAATATTAACTTTAACCTTCCTGTAACAAATGATAATGTATTGCT
ACCAACAGATCCAATTGGAGGTAAAACAGGAATTAACTATACTAATATATTTAATACTTATGGTCCTT
TAACTGCATTAAATAATGTACCACCAGTTTATCCAAATGGTCAAATTTGGGATAAAGAATTTGATACT
GACTTAAAACCAAGACTTCATGTAAATGCACCATTTGTTTGTCAAAATAATTGTCCTGGTCAATTATT
TGTAAAAGTTGCGCCCAATTTAACAAATGAATATGATCCTGATGCATCTGCTAATATGTCAAGAATTG
TAACTTACTCAGATTTTTGGTGGAAAGGTAAATTAGTATTTAAAGCTAAACTAAGAGCCTCTCATACT

TGGAATCCAATTCAACAAATGAGTATTAATGTAGATAACCAATTTAACTATGTACCAAGTAATATTGG
AGGTATGAAGATTGTATATGAAAAATCTCAACTAGCACCTAGA

'HUN_KF539797_H_11_2012'

ATGAGTGATGGAGCAGTTCAACCAGACGGTGGTCAACCTGCTGTCAGAAATGAAAGAGCTACAGGATC
TGGGAACGGGTCTGGAGGCGGGGGTGGTGGTGGTTCTGGGGGTGTGGGGATTTCTACGGGTACTTTTA
ATAATCAGACGGAATTTAAATTTTTGGAAAACGGATGGGTGGAAATCACAGCAAACTCAAGCAGACTT
GTACATTTAAATATGCCAGAAAGTGAAAATTATAGAAGAGTGGTTGTAAATAATTTGGATAAAACTGC
AGTTAACGGAAACATGGCTTTAGATGATACTCATGCACAAATTGTAACACCTTGGTCATTGGTTGATG
CAAATGCTTGGGGAGTTTGGTTTAATCCAGGAGATTGGCAACTAATTGTTAATACTATGAGTGAGTTG
CATTTAGTTAGTTTTGAACAAGAAATTTTTAATGTTGTTTTAAAGACTGTTTCAGAATCTGCTACTCA
GCCACCAACTAAAGTTTATAATAATGATTTAACTGCATCATTGATGGTTGCATTAGATAGCAATAATA
CTATGCCATTTACTCCAGCAGCTATGAGATCTGAGACATTGGGTTTTTATCCATGGAAACCAACCATA
CCAACTCCATGGAGATATTATTTTCAATGGGATAGAACATTAATACCATCTCATACTGGAATTAGGGG
CACACCAACAAATATATACCATGGTACAGATCCAGATGATGTTCAATTTTATACTATTGAAAATTCTG
TGCCAGTACACTTACTAAGAACAGGTGATGAATTTGCTACAGGAACATTTTTTTTTGATTGTAAACCA
TGTAGACTAACACATACATGGCAAACAAATAGAGCATTGGGCTTACCACCATTTCTAAATTCTTTGCC
TCAAGCTGAAGGAGGTACTAACTTTGGTTATATAGGAGTTCAACAAGATAAAAGACGTGGTGTAACTC
AAATGGGAAATACAAACTATATTACTGAAGCTACTATTATGAGACCAGCTGAGGTTGGTTATAGTGCA
CCATATTATTCTTTTGAGGCGTCTACACAAGGGCCATTTAAAACACCTATTGCAGCAGGACGGGGGGG
AGCGCAAACAGATGAAAATCAAGCAGCAGATGGTGATCCAAGATATGCATTTGGTAGACAACATGGTC
AAAAAACTACCACAACAGGAGAAACACCTGAGAGATTTACATATATAGCACATCAAGATACAGGAAGA
TATCCAGAAGGAGATTGGATTCAAAATATTAACTTTAACCTTCCTGTAACAAATGATAATGTATTGCT
ACCAACAGATCCAATTGGAGGTAAAACAGGAATTAACTATACTAATATATTTAATACTTATGGTCCTT
TAACTGCATTAAATAATGTACCACCAGTTTATCCAAATGGTCAAATTTGGGATAAAGAATTTGATACT
GACTTAAAACCAAGACTTCATGTAAATGCACCATTTGTTTGTCAAAATAATTGTCCTGGTCAATTATT
TGTAAAAGTTGCGCCTAATTTAACAAATGAATATGATCCTGATGCATCTACTAATATGTCAAGAATTG
TAACTTACTCAGATTTTTGGTGGAAAGGTAAATTAGTATTTAAAGCTAAACTAAGAGCCTCTCATACT
TGGAATCCAATTCAACAAATGAGTATTAATGTAGATAACCAATTTAACTATGTACCAAGTAATATTGG
AGGTATGAAGATTGTATATGAAAAATCTCAACTAGCACCTAGA

'HUN_KF539798_H_31_2012'

ATGAGTGATGGAGCAGTTCAACCAGACGGTGGTCAACCTGCTGTCAGAAATGAAAGAGCTACAGGATC
TGGGAACGGGTCTGGAGGCGGGGGTGGTGGTGGTTCTGGGGGTGTGGGGATTTCTACGGGTACTTTCA
ATAATCAGACGGAATTTAAATTTTTGGAAAACGGATGGGTGGAAATCACAGCAAACTCAAGCAGACTT
GTACATTTAAATATGCCAGAAAGTGAAAATTATAGAAGAGTGGTTGTAAATAATTTGGATAAAACTGC
AGTTAACGGAAACATGGCTTTAGATGATACTCATGCACAAATTGTAACACCTTGGTCATTGGTTGATG
CAAATGCTTGGGGAGTTTGGTTTAATCCAGGAGATTGGCAACTAATTGTTAATACTATGAGTGAGTTG
CATTTAGTTAGTTTTGAACAAGAAATTTTTAATGTTGTTTTAAAGACTGTTTCAGAATCTGCTACTCA
GCCACCAACTAAAGTTTATAATAATGATTTAACTGCATCATTGATGGTTGCATTAGATAGCAATAATA
CTATGCCATTTACTCCAGCAGCTATGAGATCTGAGACATTGGGTTTTTATCCATGGAAACCAACCATA
CCAACTCCATGGAGATATTTTTTTCAATGGGATAGAACATTAATACCATCTCATACTGGAACTAGTGG
CACACCAACAAATATATACCATGGTACAGATCCAGATGATGTTCAATTTTATACTATTGAAAATTCTG
TGCCAGTACACTTACTAAGAACAGGTGATGAATTTGCTACAGGAACATTTTTTTTTGATTGTAAACCA
TGTAGACTAACACATACATGGCAAACAAATAGAGCATTGGGCTTACCACCATTTCTAAATTCTTTGCC
TCAAGCTGAAGGAGGTACTAACTTTGGTTATATAGGAGTTCAACAAGATAAAAGACGTGGTGTAACTC
AAATGGGAAATACAAACTATATTACTGAAGCTACTATTATGAGACCAGCTGAGGTTGGTTATAGTGCA
CCATATTATTCTTTTGAGGCGTCTACACAAGGGCCATTTAAAACACCTATTGCAGCAGGACGGGGGGG
AGCGCAAACAGATGAAAATCAAGCAGCAGATGGTGATCCAAGATATGCATTTGGTAGACAACATGGTC
AAAAAACTACCACAACAGGAGAAACACCTGAGAGATTTACATATATAGCACATCAAGATACAGGAAGA
TATCCAGAAGGAGATTGGATTCAAAATATTAACTTTAACCTTCCTGTAACAAATGATAATGTCTTGCT
ACCAACAGATCCAATTGGAGGTAAAACAGGAATTAACTATACTAATATATTTAATACTTATGGTCCTT
TAACTGCATTAAATAATGTACCACCAGTTTATCCAAATGGTCAAATTTGGGATAAAGAATTTGATACT
GACTTAAAACCAAGACTTCATGTAAATGCACCATTTGTTTGTCAAAATAATTGTCCTGGTCAATTATT
TGTAAAAGTTGCGCCTAATTTAACAAATGAATATGATCCTGATGCATCTGCTAATATGTCAAGAATTG
TAACTTACTCAGATTTTTGGTGGAAAGGTAAATTAGTATTTAAAGCTAAACTAAGAGCCTCTCATACT

TGGAATCCAATTCAACAAATGAGTATTAATGTAGATAACCAATTTAACTATGTACCAAGTAATATTGG
AGGTATGAAGATTGTATACGAAAAATCTCAACTAGCACCTAGA
'HUN_KF539799_H_39_2012'
ATGAGTGATGGAGCAGTTCAACCAGACGGTGGTCAACCTGCTGTCAGAAATGAAAGAGCTACAGGATC
TGGGAACGGGTCTGGAGGCGGGGGTGGTGGTGGTTCTGGGGGTGTGGGGATTTCTACGGGGACTTTCA
ATAATCAGACGGAATTTAAATTTTTGGAAAACGGATGGGTGGAAATCACAGCAAACTCAAGCAGACTT
GTACATTTAAATATGCCAGAAAGTGAAAATTATAGAAGAGTGGTTGTAAATAATTTGGATAAAACTGC
AGTTAACGGAAACATGGCTTTAGATGATACTCATGCACAAATTGTAACACCTTGGTCATTGGTTGATG
CAAATGCTTGGGGAGTTTGGTTTAATCCAGGAGATTGGCAACTAATTGTTAATACTATGAGTGAGTTG
CATTTAGTTAGTTTTGAACAAGAAATTTTTAATGTTGTTTTAAAGACTGTTTCAGAATCTGCTACTCA
GCCACCAACTAAAGTTTATAATAATGATTTAACTGCATCATTGATGGTTGCATTAGATAGCAATAATA
CTATGCCATTTACTCCAGCAGCTATGAGATCTGAGACATTGGGTTTTTATCCATGGAAACCAACCATT
CCAACTCCATGGAGATATTTTTTTCAATGGGATAGAACATTAATACCATCTCATACTGGAACTAGTGG
CACACCAACAAATATATACCATGGTACAGATCCAGATGATGTTCAATTTTATACTATTGAAAATTCTG
TGCCAGTACACTTACTAAGAACAGGTGATGAATTTGCTACAGGAACATTTTTTTTTGATTGTAAACCA
TGTAGACTAACACATACATGGCAAACAAATAGAGCATTGGGCTTACCACCATTTCTAAATTCTTTGCC
TCAAGCTGAAGGAGGTACTAACTTTGGTTATATAGGAGTTCAACAAGATAAAAGACGTGGTGTAACTC
AAATGGGAAATACAAACTATATTACTGAAGCTACTATTATGAGACCAGCTGAGGTTGGTTATAGTGCA
CCATATTATTCTTTTGAGGCGTCTACACAAGGGCCATTTAAAACACCTATTGCAGCAGGACGGGGGGG
AGCGCAAACAGATGAAAATCAAGCAGCAGATGGTGATCCAAGATATGCATTTGGTAGACAACATGGTC
AAAAAACTACCACAACAGGAGAAACACCTGAGAGATTTACATATATAGCACATCAAGATACAGGAAGA
TATCCAGAAGGAGATTGGATTCAAAATATTAACTTTAACCTTCCTGTAACAAATGATAATGTCTTGCT
ACCAACAGATCCAATTGGAGGTAAAACAGGAATTAACTATACTAATATATTTAATACTTATGGTCCTT
TAACTGCATTAAATAATGTACCACCAGTTTATCCAAATGGTCAAATTTGGGATAAAGAATTTGATACT
GACTTAAAACCAAGACTTCATGTAAATGCACCATTTGTTTGTCAAAATAATTGTCCTGGTCAATTATT
TGTAAAAGTTGCGCCTAATTTAACAAATGAATATGATCCTGATGCATCTGCTAATATGTCAAGAATTG
TAACTTACTCAGATTTTTGGTGGAAAGGTAAATTAGTATTTAAAGCTAAACTAAGAGCCTCTCATACT
TGGAATCCAATTCAACAAATGAGTATTAATGTAGATAACCAATTTAACTATGTACCAAGTAATATTGG
AGGTATGAAGATTGTATATGAAAAATCTCAACTAGCACCTAGA
'HUN_KF539800_H_27_2012'
ATGAGTGATGGAGCAGTTCAACCAGACGGTGGTCAACCTGCTGTCAGAAATGAAAGAGCTACAGGATC
TGGGAACGGGTCTGGAGGCGGGGGTGGTGGTGGTTCTGGGGGTGTGGGGATTTCTACGGGTACTTTTA
ATAATCAGACGGAATTTAAATTTTTGGAAAACGGATGGGTGGAAATCACAGCAAACTCAAGCAGACTT
GTACATTTAAATATGCCAGAAAGTGAAAATTATAGAAGAGTGGTTGTAAATAATTTGGATAAAACTGC
AGTTAACGGAAACATGGCTTTAGATGATACTCATGCACAAATTGTAACACCTTGGTCATTGGTTGATG
CAAATGCTTGGGGAGTTTGGTTTAATCCAGGAGATTGGCAACTAATTGTTAATACTATGAGTGAGTTG
CATTTAGTTAGTTTTGAACAAGAAATTTTTAATGTTGTTTTAAAGACTGTTTCAGAATCTGCTACTCA
GCCACCAACTAAAGTTTATAATAATGATTTAACTGCATCATTGATGGTTGCATTAGATAGCAATAATA
CTATGCCATTTACTCCAGCAGCTATGAGATCTGAGACATTGGGTTTTTATCCATGGAAACCAACCATA
CCAACTCCATGGAGATATTATTTTCAATGGGATAGAACATTAATACCATCTCATACTGGAACTAGTGG
CACACCAACAAATATATACCATGGTACAGATCCAGATGATGTTCAATTTTATACTATTGAAAATTCTG
TGCCAGTACACTTACTAAGAACAGGTGATGAATTTGCTACAGGAACATTTTTTTTTGATTGTAAACCA
TGTAGACTAACACATACATGGCAAACAAATAGAGCATTGGGCTTACCACCATTTCTAAATTCTTTGCC
TCAAGCTGAAGGAGGTACTAACTTTGGTTATATAGGAGTTCAACAAGATAAAAGACGTGGTGTAACTC
AAATGGGAAATACAAACTATATTACTGAAGCTACTATTATGAGACCAGCTGAGGTTGGTTATAGTGCA
CCATATTATTCTTTTGAGGCGTCTACACAAGGGCCATTTAAAACACCTATTGCAGCAGGACGGGGGGG
AGCGCAAACAGATGAAAATCAAGCAGCAGATGGTGATCCAAGATATGCATTTGGTAGACAACATGGTC
AAAAAACTACCACAACAGGAGAAACACCTGAGAGATTTACATATATAGCACATCAAGATACAGGAAGA
TATCCAGAAGGAGATTGGATTCAAAATATTAACTTTAACCTTCCTGTAACAAATGATAATGTATTGCT
ACCAACAGATCCAATTGGAGGTAAAACAGGAATTAACTATACTAATATATTTAATACTTATGGTCCTT
TAACTGCATTAAATAATGTACCACCAGTTTATCCAAATGGTCAAATTTGGGATAAAGAATTTGATACT
GACTTAAAACCAAGACTTCATGTAAATGCACCATTTGTTTGTCAAAATAATTGTCCTGGTCAATTATT
TGTAAAAGTTGCGCCTAATTTAACAAATGAATATGATCCTGATGCATCTGCTAATATGTCAAGAATTG
TAACTTACTCAGATTTTTGGTGGAAAGGTAAATTAGTATTTAAAGCTAAACTAAGAGCCTCTCATACT

TGGAATCCAATTCAACAAATGAGTATTAATGTAGATAACCAATTTAACTATGTACCAAGTAATATTGG
AGGTATGAAGATTGTGTATGAAAAATCTCAACTAGCACCTAGA
'HUN_KF539801_H_25_2012'
ATGAGTGATGGAGCAGTTCAACCAGACGGTGGTCAACCTGCTGTCAGAAATGAAAGAGCTACAGGATC
TGGGAACGGGTCTGGAGGCGGGGGTGGTGGTGGTTCTGGGGGTGTGGGGATTTCTACGGGTACTTTCA
ATAATCAGACGGAATTTAAATTTTTGGAAAACGGATGGGTGGAAATCACAGCAAACTCAAGCAGACTT
GTACATTTAAATATGCCAGAAAGTGAAAATTATAGAAGAGTGGTTGTAAATAATTTGGATAAAACTGC
AGTTAACGGAAACATGGCTTTAGATGATACTCATGCACAAATTGTAACACCTTGGTCATTGGTTGATG
CAAATGCTTGGGGAGTTTGGTTTAATCCAGGAGATTGGCAACTAATTGTTAATACTATGAGTGAGTTG
CATTTAGTTAGTTTTGAACAAGAAATTTTTAATGTTGTTTTAAAGACTGTTTCAGAATCTGCTACTCA
GCCACCAACTAAAGTTTATAATAATGATTTAACTGCATCATTGATGGTTGCATTAGATAGCAATAATA
CTATGCCATTTACTCCAGCAGCTATGAGATCTGAGACATTGGGTTTTTATCCATGGAAACCAACCATA
CCAACTCCATGGAGATATTATTTTCAATGGGATAGAACATTAATACCATCTCATACTGGAACTAGTGG
CACACCAACAAATATATACCATGGTACAGATCCAGATGATGTTCAATTTTATACTATTGAAAATTCTG
TGCCAGTACACTTACTAAGAACAGGTGATGAATTTGCTACAGGAACATTTTTTTTTGATTGTAAACCA
TGTAGACTAACACATACATGGCAAACAAATAGAGCATTGGGCTTACCACCATTTCTAAATTCTTTGCC
TCAATCTGAAGGAGGTACTAACTTTGGTGATATAGGAGTTCAACAAGATAAAAGACGTGGTGTAACTC
AAATGGGAAATACAAACTATATTACTGAAGCTACTATTATGAGACCAGCTGAGGTTGGTTATAGTGCA
CCATATTATTCTTTTGAGGCGTCTACACAAGGGCCATTTAAAACACCTATTGCAGCAGGACGGGGGGG
AGCGCAAACAGATGAAAATCAAGCAGCAGATGGTAATCCAAGATATGCATTTGGTAGACAACATGGTA
AAAAAACTACCACAACAGGAGAAACACCTGAGAGATTTACATATATAGCACATCAAGATACAGGAAGA
TATCCAGAAGGAGATTGGATTCAAAATATTAACTTTAACCTTCCTGTAACAAATGATAATGTATTGCT
ACCAACAGATCCAATTGGAGGTAAAACAGGAATTAACTATACTAATATATTTAATACTTATGGTCCTT
TAACTGCATTAAATAATGTACCACCAGTTTATCCAAATGGTCAAATTTGGGATAAAGAATTTGATACT
GACTTAAAACCAAGACTTCATGTAAATGCACCATTTGTTTGTCAAAATAATTGTCCTGGTCAATTATT
TGTAAAAGTTGCGCCTAATTTAACAAATGAATATGATCCTGATGCATCTGCTAATATGTCAAGAATTG
TAACTTACTCAGATTTTTGGTGGAAAGGTAAATTAGTATTTAAAGCTAAACTAAGAGCCTCTCATACT
TGGAATCCAATTCAACAAATGAGTATTAATGTAGATAACCAATTTAACTATGTACCAAGTAATATTGG
AGGTATGAAAATTGTATATGAAAAATCTCAACTAGCACCTAGA
'HUN_KF539803_H_2_2012'
ATGAGTGATGGAGCAGTTCAACCAGACGGTGGTCAACCTGCTGTCAGAAATGAAAGAGCTACAGGATC
TGGGAACGGGTCTGGAGGCGGGGGTGGTGGTGGTTCTGGGGGTGTGGGGATTTCTACGGGTACTTTCA
ATAATCAGACGGAATTTAAATTTTTGGAAAACGGATGGGTGGAAATCACAGCAAACTCAAGCAGACTT
GTACATTTAAATATGCCAGAAAGTGAAAATTATAGAAGAGTGGTTGTAAATAATTTGGATAAAACTGC
AGTTAACGGAAACATGGCTTTAGATGATACTCATGCACAAATTGTAACACCTTGGTCATTGGTTGATG
CAAATGCTTGGGGAGTTTGGTTTAATCCAGGAGATTGGCAACTAATTGTTAATACTATGAGTGAGTTG
CATTTAGTTAGTTTTGAACAAGAAATTTTTAATGTTGTTTTAAAGACTGTTTCAGAATCTGCTACTCA
GCCACCAACTAAAGTTTATAATAATGATTTAACTGCATCATTGATGGTTGCATTAGATAGTAATAATA
CTATGCCATTTACTCCAGCAGCTATGAGATCTGAGACATTGGGTTTTTATCCATGGAAACCAACCATA
CCAACTCCATGGAGATATTATTTTCAATGGGATAGAACATTAATACCATCTCATACTGGAACTAGTGG
CACACCAACAAATATATACCATGGTACAGATCCAGATGATGTTCAATTTTATACTATTGAAAATTCTG
TGCCAGTACACTTACTAAGAACAGGTGATGAATTTGCTACAGGAACATTTTTTTTTGATTGTAAACCA
TGTAGACTAACACATACATGGCAAACAAATAGAGCATTGGGCTTACCACCATTTCTAAATTCTTTGCC
TCAATCTGAAGGAGGTACTAACTTTGGTGATATAGGAGTTCAACAAGATAAAAGACGTGGTGTAACTC
AAATGGGAAATACAAACTATATTACTGAAGCTACTATTATGAGACCAGCTGAGGTTGGTTATAGTGCA
CCATATTATTCTTTTGAGTCGTCTACACAAGGGCCATTTAAAACACCTATTGCAGCAGGACGGGGGGG
AGCGCAAACAGATGAAAATCAAGCAGCAGATGGTAATCCAAGATATGCATTTGGTAGACAACATGGTA
AAAAAACTACCACAACAGGAGAAACACCTGAGAGATTTACATATATAGCACATCAAGATACAGGAAGA
TATCCAGAAGGAGATTGGATTCAAAATATTAACTTTAACCTTCCTGTAACAAATGATAATGTATTGCT
ACCAACAGATCCAATTGGAGGTAAAACAGGAATTAACTATACTAATATATTTAATACTTATGGTCCTT
TAACTGCATTAAATAATGTACCACCAGTTTATCCAAATGGTCAAATTTGGGATAAAGAATTTGATACT
GACTTAAAACCAAGACTTCATGTAAATGCACCATTTGTTTGTCAAAATAATTGTCCTGGTCAATTATT
TGTAAAAGTTGCGCCTAATTTAACAAATGAATATGATCCTGATGCATCTGCTAATATGTCAAGAATTG
TAACTTACTCAGATTTTTGGTGGAAAGGTAAATTAGTATTTAAAGCTAAACTAAGAGCCTCTCATACT

TGGAATCCAATTCAACAAATGAGTATTAATGTAGATAACCAATTTAACTATGTACCAAGTAATATTGG
AGGTATGAAAATTGTATATGAAAAATCTCAACTAGCACCTAGA
'HUN_KF539804_H_212_2012'
ATGAGTGATGGAGCAGTTCAACCAGACGGTGGTCAACCTGCTGTCAGAAATGAAAGAGCTACAGGATC
TGGGAACGGGTCTGGAGGCGGGGGTGGTGGTGGTTCTGGGGGTGTGGGGATTTCTACGGGTACTTTCA
ATAATCAGACGGAATTTAAATTTTTGGAAAACGGATGGGTGGAAATCACAGCAAACTCAAGCAGACTT
GTACATTTAAATATGCCAGAAAGTGAAAATTATAGAAGAGTGGTTGTAAATAATTTGGATAAAACTGC
AGTTAACGGAAACATGGCTTTAGATGATACTCATGCACAAATTGTAACACCTTGGTCATTGGTTGATG
CAAATGCTTGGGGAGTTTGGTTTAATCCAGGAGATTGGCAACTAATTGTTAATACTATGAGTGAGTTG
CATTTAGTTAGTTTTGAACAAGAAATTTTTAATGTTGTTTTAAAGACTGTTTCAGAATTTGCTACTCA
GCCACCAACTAAAGTTTATAATAATGATTTAACTGCATCATTGATGGTTGCATTAGATAGCAATAATA
CTATGCCATTTACTCCAGCAGCTATGAGATTTGAGACATTGGGTTTTTATCCATGGAAACCAACCATA
CCAACTCCATGGAGATATTATTTTCAATGGGATAGAACATTAATACCATCTCATACTGGAACTAGTGG
CACACCAACAAATATATACCATGGTACAGATCCAGATGACGTTCAATTTTATACTATTGAAAATTCTG
TGCCAGTACACTTACTAAGAACAGGTGATGAATTTGCTACAGGAACATTTTTTTTTGATTGTAAACCA
TGTAGACTAACACATACATGGCAAACAAATAGAGCATTGGGCTTACCACCATTTCTAAATTCTTTGCC
TCAAGCTGAAGGAGGTACTAACTTTGGTTATATAGGAGTTCAACAAGATAAAAGACGTGGTGTAACTC
AAATGGGAAATACAAACTATATTACTGAAGCTACTATTATGAGACCAGCTGAGGTTGGTTATAGTGCA
CCATATTATTCTTTTGAGGCGTCTACACAAGGGCCATTTAAAACACCTATTGCAGCAGGACGGGGGGG
AGCGCAAACAGATGAAAATCAAGCAGCAGATGGTGATCCAAGATATGCATTTGGTAGACAACATGGTC
AAAAAACTACCACAACAGGAGAAACACCTGAGAGATTTACATATATAGCACATCAAGATACAGGAAGA
TATCCAGAAGGAGATTGGATTCAAAATATTAACTTTAACCTTCCTGTAACAAATGATAATGTATTGCT
ACCAACAGATCCAATTGGAGGTAAAACAGGAATTAACTATACTAATATATTTAATACTTATGGTCCTT
TAACTGCATTAAATAATGTACCACCAGTTTATCCAAATGGTCAAATTTGGGATAAAGAATTTGATACT
GACTTAAAACCAAGACTTCATGTAAATGCACCATTTGTTTGTCAAAATAATTGTCCTGGTCAATTATT
TGTAAAAGTTGCGCCTAATTTAACAAATGAATATGATCCTGATGCATCTACTAATATGTCAAGAATTG
TAACTTACTCAGATTTTTGGTGGAAAGGTAAATTAGTATTTAAAGCTAAACTAAGAGCCTCTCATACT
TGGAATCCAATTCAACAAATGAGTATTAATGTAGATAACCAATTTAACTATGTACCAAGTAATATTGG
AGGTATGAAGATTGTATATGAAAAATCTCAACTAGCACCTAGA
'HUN_KF539805_H_36_2012'
ATGAGTGATGGAGCAGTTCAACCAGACGGTGGTCAACCTGCTGTCAGAAATGAAAGAGCTACAGGATC
TGGGAACGGGTCTGGAGGCGGGGGTGGTGGTGGTTCTGGGGGTGTGGGGATTTCTACGGGTACTTTCA
ATAATCAGACGGAATTTAAATTTTTGGAAAACGGATGGGTGGAAATCACAGCAAACTCAAGCAGACTT
GTACATTTAAATATGCCAGAAAGTGAAAATTATAGAAGAGTGGTTGTAAATAATTTGGATAAAACTGC
AGTTAACGGAAACATGGCTTTAGATGATACTCATGCACAAATTGTAACACCTTGGTCATTGGTTGATG
CAAATGCTTGGGGAGTTTGGTTTAATCCAGGAGATTGGCAACTAATTGTTAATACTATGAGTGAGTTG
CATTTAGTTAGTTTTGAACAAGAAATTTTTAATGTTGTTTTAAAGACTGTTTCAGAATCTGCTACTCA
GCCACCAACTAAAGTTTATAATAATGATTTAACTGCATCATTGATGGTTGCATTAGATAGCAATAATA
CTATGCCATTTACTCCAGCAGCTATGAGATCTGAGACATTGGGTTTTTATCCATGGAAACCAACCATA
CCAACTCCATGGAGATATTATTTTCAATGGGATAGAACATTAATACCATCTCATACTGGAACTAGTGG
CACACCAACAAATATATACCATGGTACAGATCCAGATGATGTTCAATTTTATACTATTGAAAATTCTG
TGCCAGTACACTTACTAAGAACAGGTGATGAATTTGCTACAGGAACATTTTTTTTTGATTGTAAACCA
TGTAGACTAACACATACATGGCAAACAAATAGAGCATTGGGCTTACCACCATTTCTAAATTCTTTGCC
TCAAGCTGAAGGAGGTACTAACTTTGGTTATATAGGAGTTCAACAAGATAAAAGACGTGGTGTAACTC
AAATGGGAAATACAAACTATATTACTGAAGCTACTATTATGAGACCAGCTGAGGTTGGTTATAGTGCA
CCATATTATTCTTTTGAGGCGTCTACACAAGGGCCATTTAAAACACCTATTGCAGCAGGACGGGGGGG
AGCGCAAACAGATGAAAATCAAGCAGCAGATGGTGATCCAAGATATGCATTTGGTAGACAACATGGTC
AAAAAACTACCACAACAGGAGAAACACCTGAGAGATTTACATATATAGCACATCAAGATACAGGAAGA
TATCCAGAAGGAGATTGGATTCAAAATATTAACTTTAACCTTCCTGTAACAAATGATAATGTCTTGCT
ACCAACAGATCCAATTGGAGGTAAAACAGGAATTAACTATACTAATATATTTAATACTTATGGTCCTT
TAACTGCATTAAATAATGTACCACCAGTTTATCCAAATGGTCAAATTTGGGATAAAGAATTTGATACT
GACTTAAAACCAAGACTTCATGTAAATGCACCATTTGTTTGTCAAAATAATTGTCCTGGTCAATTATT
TGTAAAAGTTGCGCCTAATTTAACAAATGAATATGATCCTGATGCATCTGCTAATATGTCAAGAATTG
TAACTTACTCAGATTTTTGGTGGAAAGGTAAATTAGTATTTAAAGCTAAACTAAGAGCCTCTCATACT

TGGAATCCAATTCAACAAATGAGTATTAATGTAGATAACCAATTTAACTATGTACCAAGTAATATTGG
AGGTATGAAGATTGTATATGAAAAATCTCAACTAGCACCTAGA
'CHI_KF803600_2010_BJ_A68_2010'
ATGAGTGATGGAGCAGTTCAACCAGACGGTGGTCAACCTGCTGTCAGAAATGAAAGAGCTACAGGATC
TGGGAACGGGTCTGGAGGCGGGGGTGGTGGTGGTTCTGGGGGTGTGGGGATTTCTACGGGTACTTTCA
ATAATCAGACGGAATTTAAATTTTTGGAAAACGGATGGGTGGAAATCACAGCAAACTCAAGCAGACTT
GTACATTTAAATATGCCAGAAAGTGAAAATTATAGAAGAGTGGTTGTGAATAATATGGATAAAACTGC
AGTTAACGGAAACATGGCTTTAGATGATATTCATGCACAAATTGTAACACCTTGGTCATTGGTTGATG
CAAATGCTTGGGGAGTTTGGTTTAATCCAGGAGATTGGCAACTAATTGTTAATACTATGAGTGAGTTG
CATTTAGTTAGTTTTGAACAAGAAATTTTTAATGTTGTTTTAAAGACTGTTTCAGAATCTGCTACTCA
GCCACCAACTAAAGTTTATAATAATGATTTAACTGCATCATTGATGGTTGCATTAGATAGTAATAATA
CTATGCCATTTACTCCAGCAGCTATGAGATCTGAGACATTGGGTTTTTATCCATGGAAACCAACCATA
CCAACTCCATGGAGATATTATTTTCAATGGGATAGAACATTAATACCATCTCATACTGGAACTAGTGG
CACACCAACAAATATATACCATGGTACAGATCCAGATGACGTTCAATTTTATACTATTGAAAATTCTG
TGCCAGTACACTTACTAAGAACAGGTGATGAATTTGCTACAGGAACATTTTTTTTTGATTGTAAACCA
TGTAGACTAACACATACATGGCAAACAAATAGAGCATTGGGCTTACCACCATTTCTAAATTCTTTGCC
TCAAGCTGAAGGAGGTACTAACTTTGGTTATATAGGAGTTCAACAAGATAAAAGACGTGGTGTAACTC
AAATGGGAAATACAAACATTATTACTGAAGCTACTATTATGAGACCAGCTGAGGTTGGTTATAGTGCA
CCATATTATTCTTTTGAGGCGTCTACACAAGGGCCATTTAAAACACCTATTGCAGCAGGACGGGGGGG
AGCGCAAACAGATGAAAATCAAGCAGCAGATGGTGATCCAAGATATGCATTTGGTAGACAACATGGTC
AAAAAACTACCACAACAGGAGAAACACCTGAGAGATTTACATATATAGCACATCAAGATACAGGAAGA
TATCCAGAAGGAGATTGGATTCAAAATATTAACTTTAACCTTCCTGTAACAAATGATAATGTATTGCT
ACCAACAGATCCAATTGGAGGTAAAACAGGAATTAACTATACTAATATATTTAATACTTATGGTCCTT
TAACTGCATTAAATAATGTACCACCAGTTTATCCAAATGGTCAAATTTGGGATAAAGAATTTGATACT
GACTTAAAACCAAGACTTCATGTAAATGCACCATTTGTTTGTCAAAATAATTGTCCTGGTCAATTATT
TGTAAAAGTTGCGCCTAATTTAACAAATGAATATGATCCTGATGCATCTGCTAATATGTCAAGAATTG
TAACTTACTCAGATTTTTGGTGGAAAGGTAAATTAGTATTTAAAGCTAAACTAAGAGCCTCTCATACT
TGGAATCCAATTCAACAAATGAGTATTAATGTAGATAACCAATTTAACTATGTACCAAGTAATATTGG
AGGTATGAAAATTGTATATGAAAAATCTCAACTAGCACCTAGA
'CHI_KF803602_2010_BJ_A72_2010'
ATGAGTGATGGAGCAGTTCAACCAGACGGTGGTCAACCTGCTGTCAGAAATGAAAGAGCTACAGGATC
TGGGAACGGGTCTGGAGGCGGGGGTGGTGGTGGTTCTGGGGGTGTGGGGATTTCTACGGGTACTTTCA
ATAATCAGACGGAATTTAAATTTTTGGAAAACGGATGGGTGGAAATCACAGCAAACTCAAGCAGACTT
GTACATTTAAATATGCCAGAAAGTGAAAATTATAGAAGAGTGGTTGTGAATAATATGGATAAAACTGC
AGTTAACGGAAACATGGCTTTAGATGATATTCATGCACAAATTGTAACACCTTGGTCATTGGTTGATG
CAAATGCTTGGGGAGTTTGGTTTAATCCAGGAGATTGGCAACTAATTGTTAATACTATGAGTGAGTTG
CATTTAGTTAGTTTTGAACAAGAAATTTTTAATGTTGTTTTAAAGACTGTTTCAGAATCTGCTACTCA
GCCACCAACTAAAGTTTATAATAATGATTTAACTGCATCATTGATGGTTGCATTAGATAGTAATAATA
CTATGCCATTTACTCCAGCAGCTATGAGATCTGAGACATTGGGTTTTTATCCATGGAAACCAACCATA
CCAACTCCATGGAGATATTATTTTCAATGGGATAGAACATTAGTACCATCTCATACTGGAACTAGTGG
CACACCAACAAATATATACCATGGTACAGATCCAGATGATGTTCAATTTTATACTATTGAAAATTCTG
TGCCAGTACACTTACTAAGAACAGGTGATGAATTTGCTACAGGAACATTTTTTTTTGATTGTAAACCA
TGTAGACTAACACATACATGGCAAACAAATAGAGCATTGGGCTTACCACCATTTCTAAATTCTTTGCC
TCAATCTGAAGGAGCTACTAACTTTGGTGATATAGGAGTTCAACAAGATAAAAGACGTGGTGTAACTC
AAATGGGAAATACAAACTATATTACTGAAGCTACTATTATGAGACCAGCTGAGGTTGGTTATAGTGCA
CCATATTATTCTTTTGAGACGTCTACACAAGGGCCATTTAAAACACCTATTGCAGCAGGACGGGGGGG
AGCGCAAACAGATGAAAATCAAGCAGCAGATGGTAATCCAAGATATGCATTTGGTAGACAACATGGTA
AAAAAACTACCACAACAGGAGAAACACCTGAGAGATTTACATATATAGCACATCAAGATACAGGAAGA
TATCCAGAAGGAGATTGGATTCAAAATATTAACTTTAACCTTCCTGTAACAAATGATAATGTATTGCT
ACCAACAGATCCAATTGGAGGTAAAACAGGAATTAACTATACTAATATATTTAATACTTATGGTCCTT
TAACTGCATTAAATAATGTACCACCAGTTTATCCAAATGGTCAAATTTGGGATAAAGAATTTGATACT
GACTTAAAACCAAGACTTCATGTAAATGCACCATTTGTTTGTCAAAATAATTGTCCTGGTCAATTATT
TGTAAAAGTTGCGCCTAATTTAACAAATGAATATGATCCTGATGCATCTGCTAATATGTCAAGAATTG
TAACTTACTCAGATTTTTGGTGGAAAGGTAAATTAGTATTTAAAGCTAAACTAAGAGCCTCTCATACT

TGGAATCCAATTCAACAAATGAGTATTAATGTAGATAACCAATTTAACTATGTACCAAGTAATATTGG
AGGTATGAAAATTGTATATGAAAAATCTCAACTAGCACCTAGA
'CHI_KF803615_2011_BJ_B25_2011'
ATGAGTGATGGAGCAGTTCAACCAGACGGTGGTCAACCTGCTGTCAGAAATGAAAGAGCTACAGGATC
TGGGAACGGGTCTGGAGGCGGGGGTGGTGGTGGTTCTGGGGGTGTGGGGATTTCTACGGGTACTTTCA
ATAATCAGACGGAATTTAAATTTTTGGAAAACGGATGGGTGGAAATCACAGCAAACTCAAGCAGACTT
GTACATTTAAATATGCCAGAAAGTGAAAATTATAGAAGAGTGGTTGTAAATAATTTGGATAAAACTGC
AGTTAACGGAAACATGGCTTTAGATGATACCCATGCACAAATTGTAACACCTTGGTCATTGGTTGATG
CAAATGCTTGGGGAGTTTGGTTTAATCCAGGAGATTGGCAACTAATTGTTAATACTATGAGTGAGTTG
CATTTAGTTAGTTTTGAACAAGAAATTTTTAATGTTGTTTTAAAGACTGTTTCAGAATCTGCTACTCA
GCCACCAACTAAAGTTTATAATAATGATTTAACTGCATCATTGATGGTTGCATTAGATAGTAATAATA
CTATGCCATTTACTCCAGCAGCTATGAGATCTGAGACATTGGGTTTTTATCCATGGAAACCAACCATA
CCAACTCCATGGAGATATTATTTTCAATGGGATAGAACATTAATACCATCTCATACTGGAACTAGTGG
CACACCAACAAATATATACCATGGTACAGATCCAGATGATGTTCAATTTTATACTATTGAAAATTCTG
TGCCAGTACACTTACTAAGAACAGGTGATGAATTTGCTACAGGAACATTTTTTTTTGATTGTAAACCA
TGTAGACTAACACATACATGGCAAACAAATAGAGCATTGGGCTTACCACCATTTCTAAATTCTTTGCC
TCAAGCTGAAGGAGGTACTAACTTTGGTTATATAGGAGTTCAACAAGATAAAAGACGTGGTGTAACTC
AAATGGGAAATACAAACTATATTACTGAAGCTACTATTATGAGACCAGCTGAGGTTGGTTATAGTGCA
CCATATTATTCTTTTGAGGCGTCTACACAAGGGCCATTTAAAACACCTATTGCAGCAGGACGGGGGGG
AGCGCAAACAGATGAAAATCAAGCAGCAGATGGTGATCCAAGATATGCATTTGGTAGACAACATGGTC
AAAAAACTACCACAACAGGAGAAACACCTGAGAGATTTACATATATAGCACATCAAGATACAGGAAGA
TATCCAGAAGGAGATTGGATTCAAAATATTAACTTTAACCTTCCTGTAACAAATGATAATGTATTGCT
ACCAACAGATCCAATTGGAGGTAAAACAGGAATTAACTATACTAATATATTTAATACTTATGGTCCTT
TAACTGCATTAAATAATGTACCACCAGTTTATCCAAATGGTCAAATTTGGGATAAAGAATTTGATACT
GACTTAAAACCAAGACTTCATGTAAATGCACCATTTGTTTGTCAAAATAATTGTCCTGGTCAATTATT
TGTAAAAGTTGCGCCTAATTTAACAAATGAATATGATCCTGATGCATCTGCTAATATGTCAAGAATTG
TAACTTACTCAGATTTTTGGTGGAAAGGTAAATTAGTATTTAAAGCTAAACTAAGAGCCTCTCATACT
TGGAATCCAATTCAACAAATGAGTATTAATGTAGATAACCAATTTAACTATGTACCAAGTAATATTGG
AGGTATGAAAATTGTATATGAAAAATCTCAACTAGCACCTAGA
'CHI_KJ170679_raccoondog_Heb10_2_2010'
ATGAGTGATGGAGCAGTTCAACCAGACGGTGGTCAATCTGCTGTCAGAAATGAAAGAGCTACAGGATC
TGGGAACGGGTCTGGAGGCGGGGGTGGTGGTGGTTCTGGGGGTGTGGGGATTTCTACGGGTACTTTCA
ATAATCAGACGGAATTTAAATTTTTGGAAAACGGATGGGTGGAAATCACAGCAAACTCAAGCAGACTT
GTACATTTAAATATGCCAGAAAGTGAAAATTATAGAAGAGTGGTTGTGAATAATATGGATAAAACTGC
AGTTAACGGAAACATGGCTTTAGATGATATTCATGCACAAATTGTAACACCTTGGTCATTGGTTGATG
CAAATGCTTGGGGAGTTTGGTTTAATCCAGGAGATTGGCAACTAATTGTTAATACTATGAGTGAGTTG
CATTTAGTTAGTTTTGAACAAGAAATTTTTAATGTTGTTTTAAAGACTGTTTCAGAATCTGCTACTCA
GCCACCAACTAAAGTTTATAATAATGATTTAACTGCATCATTGATGGTTGCATTAGATAGTAATAATA
CTATGCCATTTACTCCAGCAGCTATGAGATCTGAGACATTGGGTTTTTATCCATGGAAACCAACCATA
CCAACTCCATGGAGATATTATTTTCAATGGGATAGAACATTAGTACCATCTCATACTGGAACTAGTGG
CACACCAACAAATATATACCATGGTACAGATCCAGATGATGTTCAATTTTATACTATTGAAAATTCTG
TGCCAGTACACTTACTAAGAACAGGTGATGAATTTGCTACAGGAACATTTTTTTTTGATTGTAAACCA
TGTAGACTAACACATACATGGCAAACAAATAGAGCATTGGGCTTACCACCATTTCTAAATTCTTTGCC
TCAATCTGAAGGAGCTACTAACTTTGGTGATATAGGAGTTCAACAAGATAAAAGACGTGGTGTAACTC
AAATGGGAAATACAAACTATATTACTGAAGCTACTATTATGAGACCAGCTGAGGTTGGTTATAGTGCA
CCATATTATTCTTTTGAGGCGTCTACACAAGGGCCATTTAACACACCTATTGCAGCAGGACGGGGGGG
AGCGCAAACAGATGAAAATCAAGCAGCAGATGGTGATCCAAGATATGCATTTGGTAGACAACATGGTC
AAAAAACTACCACAACAGGAGAAACACCTGAGAGATTTACATATATAGCACATCAAGATACAGGAAGA
TATCCAGAAGGAGATTGGATTCAAAATATTAACTTTAACCTTCCTGTAACAAATGATAATGTATTGCT
ACCAACAGATCCAATTGGAGGTAAAACAGGAATTAACTATACTAATATATTTAATACTTATGGTCCTT
TAACTGCATTAAATAATGTACCACCAGTTTATCCAAATGGTCAAATTTGGGATAAAGAATTTGATACT
GACTTAAAACCAAGACTTCATGTAAATGCACCATTTGTTTGTCAAAATAATTGTCCTGGTCAATTATT
TGTAAAAGTTGCGCCTAATTTAACAAATGAATATGATCCTGATGCATCTGCTAATATGTCAAGAATTG
TAACTTACTCAGATTTTTGGTGGAAAGGTAAATTAGTATTTAAAGCTAAACTAAGAGCCTCTCATACT

TGGAATCCAATTCAACAAATGAGTATTAATGTAGATAACCAATTTAACTATCTACCAAGTAATATTGG
AGGTATGAAAATTGTATATGAAAAATCTCAACTAGCACCTAGA
'CHI_KJ170680_raccoondog_HLJ11_1_2011'
ATGAGTGATGGAGCAGTTCAACCAGACGGTGGTCAACCTGCTGTCAGAAATGAAAGAGCTACAGGATC
TGGGAACGGGTCTGGAGGCGGGGGTGGTGGTGGTTCTGGGGGTGTGGGGATTTCTACGGGTACTTTCA
ATAATCAGACGGAATTTAAATTTTTGGAAAACGGATGGGTGGAAATCACAGCAAACTCAAGCAGACTT
GTACATTTAAATATGCCAGAAAGTGAAAATTATAAAAGAGTAGTTGTAAATAATATGGATAAAACTGC
AGTTAACGGAAACATGGCTTTAGATGATATTCATGCACAAATTGTAACACCTTGGTCATTGGTTGATG
CAAATGCTTGGGGAGTTTGGTTTAATCCAGGAGATTGGCAACTAATTGTTAATACTATGAGTGAGTTG
CATTTAGTTAGTTTTGAACAAGAAATTTTTAATGTTGTTTTAAAGACTGTTTCAGAATCTGCTACTCA
GCCACCAACTAAAGTTTATAATAATGATTTAACTGCATCATTGATGGTTGCATTAGATAGTAATAATA
CTATGCCATTTACTCCAGCAGCTATGAGATCTGAGACATTGGGTTTTTATCCATGGAAACCAACCATA
CCAACTCCATGGAGATATTATTTTCAATGGGATAGAACATTAATACCATCTCATACTGGAACTAGTGG
CACACCAACAAATATATACCATGGTACAGATCCAGATGATGTTCAATTTTATACTATTGAAAATTCTG
TGCCAGTACACTTACTAAGAACAGGTGATGAATTTGCTACAGGAACATTTTTTTTTGATTGTAAACCA
TGTAGACTAACACATACATGGCAAACAAATAGAGCATTGGGCTTACCACCATTTCTAAATTCTTTGCC
TCAATCTGAAGGAAGTACTAACTTTGGTGATATAGGAGTTCAACAAGATAAAAGACGTGGTGTAACTC
AAATGGGAAATACAAACTATATTACTGAAGCTACTATTATGAGACCAGCTGAGGTTGGTTATAGTGCA
CCATATTATTCTTTTGAGGCGTCTACACAAGGGCCATTTAAAACACCTATTGCAGCAGGACGGGGGGG
AGCGCAAACAGATGAAAATCAAGCAGCAGATGGTGATCCAAGATATGCATTTGGTAGACAACATGGTC
AAAAAACTACCACAACAGGAGAAACACCTGAGAGATTTACATATATAGCACATCAAGATACAGGAAGA
TATCCAGAAGGAGATTGGATTCAAAATATTAACTTTAACCTTCCTGTAACAAATGATAATGTATTGCT
ACCAACAGATCCAATTGGAGGTAAAACAGGAATTAACTATACTAATATATTTAATACTTATGGTCCTT
TAACTGCATTAAATAATGTACCACCAGTTTATCCAAATGGTCAAATTTGGGATAAAGAATTTGATACT
GACTTAAAACCAAGACTTCATGTAAATGCACCATTTGTTTGTCAAAATAATTGTCCTAGTCAATTATT
TGTAAAAGTTGCGCCTAATTTAACAAATGAATATGATCCTGATGCATCTGCTAATATGTCAAGAATTG
TAACTTACTCAGATTTTTGGTGGAAAGGTAAATTAGTATTTAAAGCTAAACTAAGAGCCTCTCATACT
TGGAATCCAATTCAACAAATGAGTATTAATGTAGATAACCAATTTAACTATGTACCAAGTAATATTGG
AGGTATGAAAATTGTATATGAAAAATCTCAACTAGCACCTAGA
'CHI_KJ194463_raccoondog_HeB10_3_2010'
ATGAGTGATGGAGCAGTTCAACCAGACGGTGGTCAACCTGCTGTCAGAAATGAAAGAGCTACAGGATC
TGGGAACGGGTCTGGAGGCGGGGGTGGTGGTGGTTCTGGGGGTGTGGGGATTTCTACGGGTACTTTCA
ATAATCAGACGGAATTTAAATTTTTGGAAAACGGATGGGTGGAAATCACAGCAAACTCAAGCAGACTT
GTACATTTAAATATGCCAGAAAGTGAAAATTATAGAAGAGTGGTTGTGAATAATATGGATAAAACTGC
AGTTAACGGAAACATGGCTTTAGATGATATTCATGCACAAATTGTAACACCTTGGTCATTGGTTGATG
CAAATGCTTGGGGAGTTTGGTTTAATCCAGGAGATTGGCAACTAATTGTTAATACTATGAGTGAGTTG
CATTTAGTTAGTTTTGAACAAGAAATTTTTAATGTTGTTTTAAAGACTGTTTCAGAATCTGCTACTCA
GCCACCAACTAAAGTTTATAATAATGATTTAACTGCATCATTGATGGTTGCATTAGATAGTAATAATA
CTATGCCATTTACTCCAGCAGCTATGAGATCTGAGACATTGGGTTTTTATCCATGGAAACCAACCATA
CCAACTCCATGGAGATATTATTTTCAATGGGATAGAACATTAGTACCATCTCATACTGGAACTAGTGG
CACACCAACAAATATATACCATGGTACAGATCCAGATGATGTTCAATTTTATACTATTGAAAATTCTG
TGCCAGTACACTTACTAAGAACAGGTGATGAATTTGCTACAGGAACATTTTTTTTTGATTGTAAACCA
TGTAGACTAACACATACATGGCAAACAAATAGAGCATTGGGCTTACCACCATTTCTAAATTCTTTGCC
TCAATCTGAAGGAGCTACTAACTTTGGTGATATAGGAGTTCAACAAGATAAAAGACGTGGTGTAACTC
AAATGGGAAATACAAACTATATTACTGAAGCTACTATTATGAGACCAGCTGAGGTTGGTTATAGTGCA
CCATATTATTCTTTTGAGGCGTCTACACAAGGGCCATTTAAAACACCTATTGCAGCAGGACGGGGGGG
AGCGCAAACAGATGAAAATCAAGCAGCAGATGGTGATCCAAGATATGCATTTGGTAGACAACATGGTC
AAAAAACTACCACAACAGGAGAAACACCTGAGAGATTTACATATATAGCACATCAAGATACAGGAAGA
TATCCAGAAGGAGATTGGATTCAAAATATTAACTTTAACCTTCCTGTAACAAATGATAATGTATTGCT
ACCAACAGATCCAATTGGAGGTAAAACAGGAATTAACTATACTAATATATTTAATACTTATGGTCCTT
TAACTGCATTAAATAATGTACCACCAGTTTATCCAAATGGTCAAATTTGGGATAAAGAATTTGATACT
GACTTAAAACCAAGACTTCATGTAAATGCACCATTTGTTTGTCAAAATAATTGTCCTGGTCAATTATT
TGTAAAAGTTGCGCCTAATTTAACAAATGAATATGATCCTGATGCATCTGCTAATATGTCAAGAATTG
TAACTTACTCAGATTTTTGGTGGAAAGGTAAATTAGTATTTAAAGCTAAACTAAGAGCCTCTCATACT

TGGAATCCAATTCAACAAATGAGTATTAATGTAGATAACCAATTTAACTATCTACCAAGTAATATTGG
AGGTATGAAAATTGTATTTGAAAAATCTCAACTAGCACCTAGA
'USA_KJ813827_Fisher_F1M111211_2013'
ATGAGTGATGGAGCAGTTCAACCAGACGGTGGTCAGCCTGCTGTCAGAAATGAAAGAGCTACAGGATC
TGGGAACGGGTCTGGAGGCGGGGGTGGTGGTGGTTCTGGGGGTGTGGGGATTTCTACGGGTACTTTCA
ATAATCAGACGGAATTTAAATTTTTGGAAAACGGATGGGTGGAAATCACAGCAAACTCAAGCAGACTT
GTACATTTAAATATGCCAGAAAGTGAAAATTATAGAAGAGTGGTTGTAAATAATTTGGATAAAACTGC
AGTTAACGGAAACATGGCTTTAGATGATACTCATGCACAAATTGTAACACCTTGGTCATTGGTTGATG
CAAATGCTTGGGGAGTTTGGTTTAATCCAGGAGATTGGCAACTAATTGTTAATACTATGAGTGAGTTG
CATTTAGTTAGTTTTGAACAAGAAATTTTTAATGTTGTTTTAAAGACTGTTTCAGAATCTGCTACTCA
GCCACCAACTAAAGTTTATAATAATGATTTAACTGCATCATTGATGGTTGCATTAGATAGTAATAATA
CTATGCCATTTACTCCAGCAGCTATGAGATCTGAGACATTGGGTTTTTATCCATGGAAACCAACCATA
CCAACTCCATGGAGATATTATATTCAATGGGATAGAACATTAATACCATCTCATACTGGAACTAGTGG
CACACCAACAAATATATACCATGGTACAGATCCAGATGATGTTCAATTTTATACTATTGAAAATTCTG
TGCCAGTACACTTACTAAGAACAGGTGATGAATTTGCTACAGGAACATTTTTTTTTGATTGTAAACCA
TGTAGACTAACACATACATGGCAAACAAATAGAGCATTGGGCTTACCACCATTTCTAAATTCTTTGCC
TCAAGCTGAAGGAGGTACTAACTTTGGTTATATAGGAGTTCAACAAGATAAAAGACGTGGTGTAACTC
AAATGGGAAATACAAACTATATTACTGAAGCTACTATTATGAGACCAGCTGAGGTTGGTTATAGTGCA
CCATATTATTCTTTTGAGGCGTCTACACAAGGGCCATTTAAAACACCTATTGCAGCAGGACGGGGGGG
AGCGCAAACAGATGAAAATCAAGCAGCAGATGGTGATCCAAGATATGCATTTGGTAGACAACATGGTC
AAAAAACTACCACAACAGGAGAAACACCTGAGAGATTTACATATATAGCACATCAAGATACAGGAAGA
TATCCAGAAGGAGATTGGATTCAAAATATTAACTTTAACCTTCCTGTAACAGATGATAATGTATTGCT
ACCAACAGATCCAATTGGAGGTAAAACAGGAATTAACTATACTAATATATTTAATACTTATGGTCCTT
TAACTGCATTAAATAATGTACCACCAGTTTATCCAAATGGTCAAATTTGGGATAAAGAATTTGATACT
GACTTAAAACCAAGACTTCATGTAAATGCACCATTTGTTTGTCAAAATAATTGCCCTGGTCAATTATT
TGTAAAAGTTGCGCCTAATTTAACAAATGAATATGATCCTGATGCATCTGCTAATATGTCAAGAATTG
TAACTTACTCAGATTTTTGGTGGAAAGGTAAATTAGTATTTAAAGCTAAACTAAGAGCCTCTCATACT
TGGAATCCAATTCAACAAATGAGTATTAATGTAGATAACCAATTTAACTATGTACCAAGTAATATTGG
AGGTATGAAAATTGTCTATGAAAAATCTCAACTAGCACCTAGA
'USA_KJ813828_Fisher_F1F010712_2013'
ATGAGTGATGGAGCAGTTCAACCAGACGGTGGTCAGCCTGCTGTCAGAAATGAAAGAGCTACAGGATC
TGGGAACGGGTCTGGAGGCGGGGGTGGTGGTGGTTCTGGGGGTGTGGGGATTTCTACGGGTACTTTCA
ATAATCAGACGGAATTTAAATTTTTGGAAAACGGATGGGTGGAAATCACAGCAAACTCAAGCAGACTT
GTACATTTAAATATGCCAGAAAGTGAAAATTATAGAAGAGTGGTTGTAAATAATTTGGATAAAACTGC
AGTTAACGGAAACATGGCTTTAGATGATACTCATGCACAAATTGTAACACCTTGGTCATTGGTTGATG
CAAATGCTTGGGGAGTTTGGTTTAATCCAGGAGATTGGCAACTAATTGTTAATACTATGAGTGAGTTG
CATTTAGTTAGTTTTGAACAAGAAATTTTTAATGTTGTTTTAAAGACTGTTTCAGAATCTGCTACTCA
GCCACCAACTAAAGTTTATAATAATGATTTAACTGCATCATTGATGGTTGCATTAGATAGTAATAATA
CTATGCCATTTACTCCAGCAGCTATGAGATCTGAGACATTGGGTTTTTATCCATGGAAACCAACCATA
CCAACTCCATGGAGATATTATTTTCAATGGGATAGAACATTAATACCATCTCATACTGGAACTAGTGG
CACACCAACAAATATATACCATGGTACAGATCCAGATGATGTTCAATTTTATACTATTGAAAATTCTG
TGCCAGTACACTTACTAAGAACAGGTGATGAATTTGCTACAGGAACATTTTTTTTTGATTGTAAACCA
TGTAGACTAACACATACATGGCAAACAAATAGAGCATTGGGCTTACCACCATTTCTAAATTCTTTGCC
TCAAGCTGAAGGAGGAACTAACTTTGGTTATATAGGAGTTCAACAAGATAAAAGACGTGGTGTAACTC
AAATGGGAAATACAAACTATATTACTGAAGCTACTATTATGAGACCAGCTGAGGTTGGTTATAGTGCA
CCATATTATTCTTTTGAGGCGTCTACACAAGGGCCATTTAAAACACCTATTGCAGCAGGACGGGGGGG
AGCGCAAACAGATGAAAATCAAGCAGCAGATGGTGATCCAAGATATGCATTTGGTAGACAACATGGTC
AAAAAACTACCACAACAGGAGAAACACCTGAAAGATTTACATATATAGCACATCAAGATACAGGAAGA
TATCCAGAAGGAGATTGGATTCAAAATATTAACTTTAACCTTCCTGTAACAGATGATAATGTATTGCT
ACCAACAGATCCAATTGGAGGTAAAACAGGAATTAACTATACTAATATATTTAATACTTATGGTCCTT
TAACTGCATTAAATAATGTACCACCAGTTTATCCAAATGGTCAAATTTGGGATAAAGAATTTGATACT
GACTTAAAACCAAGACTTCATGTAAATGCACCATTTGTTTGTCAAAATAATTGCCCTGGTCAATTATT
TGTAAAAGTTGCGCCTAATTTAACAAATGAATATGATCCTGATGCATCTGCTAATATGTCAAGAATTG
TAACTTACTCAGATTTTTGGTGGAAAGGTAAATTAGTATTTAAAGCTAAACTAAGAGCCTCTCATACT

TGGAATCCAATTCAACAAATGAGTATTAATGTAGATAACCAATTTAACTATGTACCAAGTAATATTGG
AGGTATGAAAATTGTCTATGAAAAATCTCAACTAGCACCTAGA
'USA_KJ813831_Fisher_ND_17_2013'
ATGAGTGATGGAGCAGTTCAACCAGACGGTGGTCAACCTGCTGTCAGAAATGAAAGAGCTACAGGATC
TGGGAACGGGTCTGGAGGCGGGGGTGGTGGTGGTTCTGGGGGTGTGGGGATTTCTACGGGTACTTTCA
ATAATCAGACGGAATTTAAATTTTTGGAAAACGGATGGGTGGAAATCACAGCAAACTCAAGCAGACTT
GTACATTTAAATATGCCAGAAAGTGAAAATTATAGAAGAGTGGTTGTAAATAATTTAGATAAAACTGC
AGTTAACGGAAACATGGCTTTAGATGATACTCATGCACAAATTGTAACACCTTGGTCATTGGTTGATG
CAAATGCTTGGGGAGTTTGGTTTAATCCAGGAGATTGGCAACTAATTGTTAATACTATGAGTGAGTTG
CATTTAGTTAGTTTTGAACAAGAAATTTTTAATGTTGTTTTAAAGACTGTTTCAGAATCTGCTACTCA
GCCACCAACTAAAGTTTATAATAATGATTTAACTGCATCATTGATGGTTGCATTAGATAGTAATAATA
CTATGCCATTTACTCCAGCAGCTATGAGATCTGAGACATTGGGTTTTTATCCATGGAAACCAACCATA
CCAACTCCATGGAGATATTATTTTCAATGGGATAGAACATTAATACCATCTCATACTGGAACTAGTGG
CACACCAACAAATACATACCATGGTACAGATCCAGATGATGTTCAATTTTATACTATTGAAAATTCTG
TGCCAGTACACTTACTAAGAACAGGTGATGAATTTGCTACAGGAACATTTTTTTTTGATTGTAAACCA
TGTAGACTAACACATACATGGCAAACAAATAGAGCATTGGGCTTACCACCATTTCTAAATTCTTTGCC
TCAATCTGAAGGAGATACTAACTTTGGTGATATAGGAGTTCAACAAGATAAAAGACGTGGTGTAACTC
AAATGGGAAATACAAACTATATTACTGAAGCTACTATTATGAGACCAGCTGAGGTTGGTTATAGTGCA
CCATATTATTCTTTTGAGGCGTCTACACAAGGGCCATTTAAAACACCTATTGCAGCAGGACGGGGGGG
AGCACAAACAGATGAAAATCAAGCAGCAGATGGTGATCCAAGATATGCATTTGGTAGACAACATGGTC
AAAAAACTACCACAACAGGAGAAACACCTGAGAGATTTACATATATAGCACATCAAGATACAGGAAGA
TATCCAGAAGGAGATTGGATTCAAAATATTAACTTTAACCTTCCTGTAACAAATGATAATGTATTGCT
ACCAACAGATCCAATTGGAGGTAAAACAGGAATTAACTATACTAATATATTTAATACTTATGGTCCTT
TAACTGCATTAAATAATGTACCACCAGTTTATCCAAATGGTCAAATTTGGGATAAAGAATTTGATACT
GACTTAAAACCAAGACTTCATGTAAATGCACCATTTGTTTGTCAAAATAATTGTCCTGGTCAATTATT
TGTAAAAGTTGCGCCTAATTTAACAAATGAATATGATCCTGATGCATCTGCTAATATGTCAAGAATTG
TAACTTACTCAGATTTTTGGTGGAAAGGTAAATTAGTATTTAAAGCTAAACTAAGAGCCTCTCATACT
TGGAATCCAATTCAACAAATGAGTATTAATGTAGATAACCAATTTAACTATGTACCAAGTAATATTGG
AGGTATGAAAATTGTATATGAAAAATCTCAACTAGCACCTAGA
'USA_KJ813832_Fisher_ND_14_2013'
ATGAGTGATGGAGCAGTTCAACCAGACGGTGGTCAACCTGCTGTCAGAAATGAAAGAGCTACAGGATC
TGGGAACGGGTCTGGAGGCGGGGGTGGTGGTGGTTCTGGGGGTGTGGGGATTTCTACGGGTACTTTCA
ATAATCAGACGGAATTTAAATTTTTGGAAAACGGATGGGTGGAAATCACAGCAAACTCAAGCAGACTT
GTACATTTAAATATGCCAGAAAGTGAAAATTATAGAAGAGTGGTTGTAAATAATTTAGATAAAACTGC
AGTTAACGGAAACATGGCTTTAGATGATACTCATGCACAAATTGTAACACCTTGGTCATTGGTTGATG
CAAATGCTTGGGGAGTTTGGTTTAATCCAGGAGATTGGCAACTAATTGTTAATACTATGAGTGAGTTG
CATTTAGTTAGTTTTGAACAAGAAATTTTTAATGTTGTTTTAAAGACTGTTTCAGAATCTGCTACTCA
GCCACCAACTAAAGTTTATAATAATGATTTAACTGCATCATTGATGGTTGCATTAGATAGTAATAATA
CTATGCCATTTACTCCAGCAGCTATGAGATCTGAGACATTGGGTTTTTATCCATGGAAACCAACCATA
CCAACTCCATGGAGATATTATTTTCAATGGGATAGAACATTAATACCATCTCATACTGGAACTAGTGG
CACACCAACAAATACATACCATGGTACAGATCCAGATGATGTTCAATTTTATACTATTGAAAATTCTG
TGCCAGTACACTTACTAAGAACAGGTGATGAATTTGCTACAGGAACATTTTTTTTTGATTGTAAACCA
TGTAGACTAACACATACATGGCAAACAAATAGAGCATTGGGCTTACCACCATTTCTAAATTCTTTGCC
TCAATCTGAAGGAGATACTAACTTTGGTGATATAGGAGTTCAACAAGATAAAAGACGTGGTGTAACTC
AAATGGGAAATACAAACTATATTACTGAAGCTACTATTATGAGACCAGCTGAGGTTGGTTATAGTGCA
CCATATTATTCTTTTGAGGCGTCTACACAAGGGCCATTTAAAACACCTATTGCAGCAGGACGGGGGGG
AGCACAAACAGATGAAAATCAAGCAGCAGATGGTGATCCAAGATATGCATTTGGTAGACAACATGGTC
AAAAAACTACCACAACAGGAGAAACACCTGAGAGATTTACATATATAGCACATCAAGATACAGGAAGA
TATCCAGAAGGAGATTGGATTCAAAATATTAACTTTAACCTTCCTGTAACAAATGATAATGTATTGCT
ACCAACAGATCCAATTGGAGGTAAAACAGGAATTAACTATACTAATATATTTAATACTTATGGTCCTT
TAACTGCATTAAATAATGTACCACCAGTTTATCCAAATGGTCAAATTTGGGATAAAGAATTTGATACT
GACTTAAAACCAAGACTTCATGTAAATGCACCATTTGTTTGTCAAAATAATTGTCCTGGTCAATTATT
TGTAAAAGTTGCACCTAATTTAACAAATGAATATGATCCTGATGCATCTGCTAATATGTCAAGAATTG
TAACTTACTCAGATTTTTGGTGGAAAGGTAAATTAGTATTTAAAGCTAAACTAAGAGCCTCTCATACT

TGGAATCCAATTCAACAAATGAGTATTAATGTAGATAACCAATTTAACTATGTACCAAGTAATATTGG
AGGTATGAAAATTGTATATGAAAAATCTCAACTAGCACCTAGA
'USA_KJ813835_Fisher_ND_19_2013'
ATGAGTGATGGAGCAGTTCAACCAGACGGTGGTCAACCTGCTGTCAGAAATGAAAGAGCTACAGGATC
TGGGAACGGGTCTGGAGGCGGGGGTGGTGGTGGTTCTGGGGGTGTGGGGATTTCTACGGGTACTTTCA
ATAATCAAACGGAATTTAAATTTTTGGAAAACGGATGGGTGGAAATCACAGCAAACTCAAGCAGACTT
GTACATTTAAATATGCCAGAAAGTGAAAATTATAGAAGAGTGGTTGTAAATAATTTAGATAAAACTGC
AGTTAACGGAAACATGGCTTTAGATGATACTCATGCACAAATTGTAACACCTTGGTCATTGGTTGATG
CAAATGCTTGGGGAGTTTGGTTTAATCCAGGAGATTGGCAACTAATTGTTAATACTATGAGTGAGTTG
CATTTAGTTAGTTTTGAACAAGAAATTTTTAATGTTGTTTTAAAGACTGTTTCAGAATCTGCTACTCA
GCCACCAACTAAAGTTTATAATAATGATTTAACTGCATCATTGATGGTTGCATTAGATAGTAATAATA
CTATGCCATTTACTCCAGCAGCTATGAGATCTGAGACATTGGGTTTTTATCCATGGAAACCAACCATA
CCAACTCCATGGAGATATTATTTTCAATGGGATAGAACATTAATACCATCTCATACTGGAACTAGTGG
CACACCAACAAATACATACCATGGTACAGATCCAGATGATGTTCAATTTTATACTATTGAAAATTCTG
TGCCAGTACACTTACTAAGAACAGGTGATGAATTTGCTACAGGAACATTTTTTTTTGATTGTAAACCA
TGTAGACTAACACATACATGGCAAACAAATAGAGCATTGGGCTTACCACCATTTCTAAATTCTTTGCC
TCAATCTGAAGGAGATACTAACTTTGGTGATATAGGAGTTCAACAAGATAAAAGACGTGGTGTAACTC
AAATGGGAAATACAAACTATATTACTGAAGCTACTATTATGAGACCAGCTGAGGTTGGTTATAGTGCA
CCATATTATTCTTTTGAGGCGTCTACACAAGGGCCATTTAAAACACCTATTGCAGCAGGACGGGGGGG
AGCACAAACAGATGAAAATCAAGCAGCAGATGGTGATCCAAGATATGCATTTGGTAGACAACATGGTC
AAAAAACTACCACAACAGGAGAAACACCTGAGAGATTTACATATATAGCACATCAAGATACAGGAAGA
TATCCAGAAGGAGATTGGATTCAAAATATTAACTTTAACCTTCCTGTAACAAATGATAATGTATTGCT
ACCAACAGATCCAATTGGAGGTAAAACAGGAATTAACTATACTAATATATTTAATACTTATGGTCCTT
TAACTGCATTAAATAATGTACCACCAGTTTATCCAAATGGTCAAATTTGGGATAAAGAATTTGATACT
GACTTAAAACCAAGACTTCATGTAAATGCACCATTTGTTTGTCAAAATAATTGTCCTGGTCAATTATT
TGTAAAAGTTGCGCCTAATTTAACAAATGAATATGATCCTGATGCATCTGCTAATATGTCAAGAATTG
TAACTTACTCAGATTTTTGGTGGAAAGGTAAATTAGTATTTAAAGCTAAACTAAGAGCCTCTCATACT
TGGAATCCAATTCAACAAATGAGTATTAATGTAGATAACCAATTTAACTATGTACCAAGTAATATTGG
AGGTATGAAAATTGTATATGAAAAATCTCAACTAGCACCTAGA
'USA_KJ813842_Bobcat_ND_502_2013'
ATGAGTGATGGAGCAGTTCAACCAGACGGTGGTCAACCTGCTGTCAGAAATGAAAGAGCAACAGGATC
TGGGAACGGGTCTGGAGGCGGGGGTGGTGGTGGTTCTGGGGGTGTGGGGATTTCTACGGGTACTTTCA
ATAATCAGACGGAATTTAAATTTTTGGAAAACGGATGGGTGGAAATCACAGCAAACTCAAGCAGACTT
GTACATTTAAATATGCCAGAAAGTGAAAATTATAGAAGAGTGGTTGTAAATAATTTGGATAAAACTGC
AGTTAACGGAAACATGGCTTTAGATGATACTCATGCACAAATTGTAACACCTTGGTCATTGGTTGATG
CAAATGCTTGGGGAGTTTGGTTTAATCCAGGAGATTGGCAACTAATTGTTAATACTATGAGTGAGTTG
CATTTAGTTAGTTTTGAACAAGAAATTTTTAATGTTGTTTTAAAGACTGTTTCAGAATCTGCTACTCA
GCCACCAACTAAAGTTTATAATAATGATTTAACTGCATCATTGATGGTTGCATTAGATAGCAATAATA
CTATGCCATTTACTCCAGCAGCTATGAGATCTGAGACATTGGGTTTTTATCCATGGAAACCAACCATA
CCAACTCCATGGAGATATTATTTTCAATGGGATAGAACATTAATACCATCTCATACTGGAACTAGTGG
CACACCAACAAATATATACCATGGTACAGATCCAGATGATGTTCAATTTTATACTATTGAAAATTCTG
TGCCAGTACACTTACTAAGAACAGGTGATGAATTTGCTACAGGAACATTTTTTTTTGATTGTAAACCA
TGTAGACTAACACATACATGGCAAACAAATAGAGCATTGGGCTTACCACCATTTCTAAATTCTTTGCC
TCAAGCTGAAGGAGGTACTAACTTTGGTTATATAGGAGTTCAACAAGATAAAAGACGTGGTGTAACTC
AAATGGGAAATACAAACTATATTACTGAAGCTACTATTATGAGACCAGCTGAGGTTGGTTATAGTGCA
CCATATTATTCTTTTGAGGCGTCTACACAAGGGCCATTTAAAACACCTATTGCAGCAGGACGGGGGGG
AGCGCAAACAGATGAAAATCAAGCAGCAGATGGTGATCCAAGATATGCATTTGGTAGACAACATGGTC
AAAAGACTACCACAACAGGAGAAACACCTGAGAGATTTACATATATAGCACATCAAGATACAGGAAGA
TATCCAGAAGGAGATTGGATTCAAAATATTAACTTTAACCTTCCTGTAACAGATGATAATGTATTGCT
ACCAACAGATCCAATTGGAGGTAAAACAGGAATTAACTATACTAACATATTTAATACTTATGGTCCTT
TAACTGCATTAAATAATGTACCACCAGTTTATCCAAATGGTCAAATTTGGGATAAAGAATTTGATACT
GACTTAAAACCAAGACTTCATGTAAATGCACCATTTGTTTGTCAAAATAATTGTCCTGGTCAATTATT
TGTAAAAGTTGCGCCTAATTTAACAAATGAATATGATCCTGATGCATCTGCTAATATGTCAAGAATTG
TAACTTACTCAGATTTTTGGTGGAAAGGTAAATTAGTATTTAAAGCTAAACTAAGAGCCTCTCATACT

TGGAATCCAATTCAACAAATGAGTATTAATGTAGATAACCAATTTAACTATGTACCAAGTAATATTGG
AGGTATGAAAATTGTATATGAAAAATCTCAACTAGCACCAAGA
'USA_KJ813843_Bobcat_ND_1160_2013'
ATGAGTGATGGAGCAGTTCAACCAGACGGTGGTCAACCTGCTGTCAGAAATGAAAGAGCAACAGGATC
TGGGAACGGGTCTGGAGGCGGGGGTGGTGGTGGTTCTGGGGGTGTGGGGATTTCTACGGGTACTTTCA
ATAATCAGACGGAATTTAAATTTTTGGAAAACGGATGGGTGGAAATCACAGCAAACTCAAGCAGACTT
GTACATTTAAATATGCCTGAAAGTGAAAATTATAGAAGAGTGGTTGTAAATAATTTGGATAAAACTGC
AGTTAACGGAAACATGGCTTTAGATGATACTCATGCACAAATTGTAACACCTTGGTCATTGGTTGATG
CAAATGCTTGGGGAGTTTGGTTTAATCCAGGAGATTGGCAACTAATTGTTAATACTATGAGTGAGTTG
CATTTAGTTAGTTTTGAACAAGAAATTTTTAATGTTGTTTTAAAGACTGTTTCAGAATCTGCTACTCA
GCCACCAACTAAAGTTTATAATAATGATTTAACTGCATCATTGATGGTTGCATTAGATAGTAATAATA
CTATGCCATTTACTCCAGCAGCTATGAGATCTGAGACATTGGGTTTTTATCCATGGAAACCAACCATA
CCAACTCCATGGAGATATTATTTTCAATGGGATAGAACATTAATACCATCTCATACTGGAACTAGTGG
CACACCAACAAATATACACCATGGTACAGATCCAGATGATGTTCAATTTTATACTATTGAAAATTCTG
TGCCAGTACACTTACTAAGAACAGGTGATGAATTTGCTACAGGAACATTTTTTTTTGATTGTAAACCA
TGTAGACTAACACATACATGGCAAACAAATAGAGCATTGGGCTTACCACCATTTCTAAATTCTTTGCC
TCAAGCTGAAGGAGGTACTAACTTTGGTTATATAGGAGTTCAACAAGATAAAAGACGTGGTGTAACTC
AAATGGGAAATACAAACTATATTACTGAAGCTACTATTATGAGACCAGCTGAGGTTGGTTATAGTGCA
CCATATTATTCTTTTGAGGCGTCTACACAAGGGCCATTTAAAACACCTATTGCAGCAGGACGGGGGGG
AGCGCAAACAGATGAAAATCAAGCAGCAGATGGTGATCCAAGATATGCATTTGGTAGACAACATGGTC
AAAAAACTACCACAACAGGAGAAACACCTGAGAGATTTACATATATAGCACATCAAGATACAGGAAGA
TATCCAGAAGGAGATTGGATTCAAAATATTAACTTTAACCTTCCTGTAACAGAAGATAATGTATTGCT
ACCAACAGATCCAATTGGAGGTAAAACAGGAATTAACTATACTAATATATTTAATACTTATGGTCCTT
TAACTGCATTAAATAATGTACCACCAGTTTATCCAAATGGTCAAATTTGGGATAAAGAATTTGATACT
GACTTAAAACCAAGACTTCATGTAAATGCACCATTTGTTTGTCAAAATAATTGTCCTGGTCAATTATT
TGTAAAAGTTGCGCCTAATTTAACAAATGAATATGATCCTGATGCATCTGCTAATATGTCAAGAATTG
TAACTTACTCAGATTTTTGGTGGAAAGGTAAATTAGTATTTAAAGCTAAACTAAGAGCCTCTCATACT
TGGAATCCAATTCAACAAATGAGTATTAATGTAGATAACCAATTTAACTATGTACCAAGTAATATTGG
AGGTATGAAAATTGTATATGAAAAATCTCAACTAGCACCTAGA
'USA_KJ813844_Bobcat_ND_885_2013'
ATGAGTGATGGAGCAGTTCAACCAGACGGTGGTCAGCCTGCTGTCAGAAATGAAAGAGCTACAGGATC
TGGGAACGGGTCTGGAGGCGGGGGTGGTGGTGGTTCTGGGGGTGTGGGGATTTCTACGGGTACTTTCA
ATAATCAGACGGAATTTAAATTTTTGGAAAACGGATGGGTGGAAATCACAGCAAACTCAAGCAGACTT
GTACATTTAAATATGCCAGAAAGTGAAAATTATAGAAGAGTGGTTGTAAATAATTTGGATAAAACTGC
AGTTAACGGAAACATGGCTTTAGATGATACTCATGCACAAATTGTAACACCTTGGTCATTGGTTGATG
CAAATGCTTGGGGAGTTTGGTTTAATCCAGGAGATTGGCAACTAATTGTTAATACTATGAGTGAGTTG
CATTTAGTTAGTTTTGAACAAGAAATTTTTAATGTTGTTTTAAAGACTGTTTCAGAATCTGCTACTCA
GCCACCAACTAAAGTTTATAATAATGATTTAACTGCATCATTGATGGTTGCATTAGATAGTAATAATA
CTATGCCATTTACTCCAGCAGCTATGAGATCTGAGACATTGGGTTTTTATCCATGGAAACCAACCATA
CCAACTCCATGGAGATATTATTTTCAATGGGATAGAACATTAATACCATCTCATACTGGAACTAGTGG
CACACCAACAAATATATACCATGGTACAGATCCAGATGATGTTCAATTTTATACTATTGAAAATTCTG
TGCCAGTACACTTACTAAGAACAGGTGATGAATTTGCTACAGGAACATTTTTTTTTGATTGTAAACCA
TGTAGACTAACACATACATGGCAAACAAATAGAGCATTGGGCTTACCACCATTTCTAAATTCTTTGCC
TCAAGCTGAAGGAGGTACTAACTTTGGTTATATAGGAGTTCAACAAGATAAAAGACGTGGTGTAACTC
AAATGGGAAATACAAACTATATTACTGAAGCTACTATTATGAGACCAGCTGAGGTTGGTTATAGTGCA
CCATATTATTCTTTTGAGGCGTCTACACAAGGGCCATTTAAAACACCTATTGCAGCAGGACGGGGGGG
AGCGCAAACAGATGAAAATCAAGCAGCAGATGGTGATCCAAGATATGCATTTGGTAGACAACATGGTC
AAAAAACTACCACAACAGGAGAAACACCTGAGACATTTACATATATAGCACATCAAGATACAGGAAGA
TATCCAGAAGGAGATTGGATTCAAAATATTAACTTTAACCTTCCTGTAACAGATGATAATGTATTGCT
ACCAACAGATCCAATTGGAGGTAAAACAGGAATTAACTATACTAATATATTTAATACTTATGGTCCTT
TAACTGCATTAAATAATGTACCACCAGTTTATCCAAATGGTCAAATTTGGGATAAAGAATTTGATACT
GACTTAAAACCAAGACTTCATGTAAATGCACCATTTGTTTGTCAAAATAATTGCCCTGGTCAATTATT
TGTAAAAGTTGCGCCTAATTTAACAAATGAATATGATCCTGATGCATCTGCTAATATGTCAAGAATTG
TAACTTACTCAGATTTTTGGTGGAAAGGTAAATTAGTATTTAAAGCTAAACTAAGAGCCTCTCATACT

TGGAATCCAATTCAACAAATGAGTATTAATGTAGATAACCAATTTAACTATGTACCAAGTAATATTGG
AGGTATGAAAATTGTCTATGAAAAATCTCAACTAGCACCTAGA
'USA_KJ813846_Bobcat_ND_974_2013'
ATGAGTGATGGAGCAGTTCAACCAGACGGTGGTCAACCTGCTGTCAGAAATGAAAGAGCAACAGGATC
TGGGAACGGGTCTGGAGGCGGGGGTGGTGGTGGTTCTGGGGGTGTGGGGATTTCTACGGGTACTTTCA
ATAATCAGACGGAATTTAAATTTTTGGAAGACGGATGGGTGGAAATCACAGCAAACTCAAGCAGACTT
GTACATTTAAATATGCCAGAAAGTGAAAATTATAGAAGAGTGGTTGTAAATAATTTGGATAAAACTGC
AGTTAACGGAAACATGGCTTTAGATGATACTCATGCACAAATTGTAACACCTTGGTCATTGGTTGATG
CAAATGCTTGGGGAGTTTGGTTTAATCCAGGAGATTGGCAACTAATTGTTAATACTATGAGTGAGTTG
CATTTAGTTAGTTTTGAACAAGAAATTTTTAATGTTGTTTTAAAGACTGTTTCAGAATCTGCTACTCA
GCCACCAACTAAAGTTTATAATAATGATTTAACTGCATCATTGATGGTTGCATTAGATAGTAATAATA
CTATGCCATTTACTCCAGCAGCTATGAGATCTGAGACATTGGGTTTTTATCCATGGAAACCAACCATA
CCAACTCCATGGAGATATTATTTTCAATGGGATAGAACATTAATACCATCTCATACTGGAACTAGTGG
CACACCAACAAATATATACCATGGTACAGATCCAGATGATGTTCAATTTTATACTATTGAAAATTCTG
TGCCAGTACACTTACTAAGAACAGGTGATGAATTTGCTACAGGAACATTTTTTTTTGATTGTAAACCA
TGTAGACTAACACATACATGGCAAACAAATAGAGCATTGGGCTTACCACCATTTCTAAATTCTTTGCC
TCAAGCTGAAGGAGGTACTAACTTTGGTTATATAGGAGTTCAACAAGATAAAAGACGTGGTGTAACTC
AAATGGGAAATACAAACTATATTACTGAAGCTACTATTATGAGACCAGCTGAGGTTGGTTATAGTGCA
CCATATTATTCTTTTGAGGCGTCTACACAAGGGCCATTTAAAACACCTATTGCAGCAGGACGGGGGGG
AGCGCAAACAGATGAAAATCAAGCAGCAGATGGTGATCCAAGATATGCATTTGGTAGACAACATGGTC
AAAAAACTACCACAACAGGAGAAACACCTGAGAGATTTACATATATAGCACATCAAGATACAGGAAGA
TATCCAGAAGGAGATTGGATTCAAAATATTAACTTTAACCTTCCTGTAACAGATGATAATGTATTGCT
ACCAACAGATCCAATTGGAGGTAAAACAGGAATTAACTATACTAATATATTTAATACTTATGGTCCTT
TAACTGCATTAAATAATGTACCACCAGTTTATCCAAATGGTCAAATTTGGGATAAAGAATTTGATACT
GACTTAAAACCAAGACTTCATGTAAATGCACCATTTGTTTGTCAAAATAATTGTCCTGGTCAATTATT
TGTAAAAGTTGCGCCTAATTTAACAAATGAATATGATCCTGATGCATCTGCTAATATGTCAAGAATTG
TAACTTACTCAGATTTTTGGTGGAAAGGTAAATTAGTATTTAAAGCTAAACTAAGAGCCTCTCATACT
TGGAATCCAATTCAACAAATGAGTATTAATGTAGATAACCAATTTAACTATGTACCAAGTAATATTGG
AGGTATGAAAATTGTATATGAAAAATCTCAACTAGCACCTAGA
'USA_KJ813848_Bobcat_ND_1162_2013'
ATGAGTGATGGAGCAGTTCAACCAGACGGTGGTCAACCTGCTGTCAGAAATGAAAGAGCAACAGGATC
TGGGAACGGGTCTGGAGGCGGGGGTGGTGGTGGTTCTGGGGGTGTGGGGATTTCTACGGGTACTTTCA
ATAATCAGACGGAATTTAAATTTTTGGAAAACGGATGGGTGGAAATCACAGCAAACTCAAGCAGACTT
GTACATTTAAATATGCCAGAAAGTGAAAATTATAGAAGAGTGGTTGTAAATAATTTGGATAAAACTGC
AGTTAACGGAAACATGGCTTTAGATGATACTCATGCACAAATTGTAACACCTTGGTCATTGGTTGATG
CAAATGCTTGGGGAGTTTGGTTTAATCCAGGAGATTGGCAACTAATTGTTAATACTATGAGTGAGTTG
CATTTAGTTAGTTTTGAACAAGAAATTTTTAATGTTGTTTTAAAGACTGTTTCAGAATCTGCTACTCA
GCCACCAACTAAAGTTTATAATAATGATTTAACTGCATCATTGATGGTTGCATTAGATAGTAATAATA
CTATGCCATTTACTCCAGCAGCTATGAGATCTGAGACATTGGGTTTTTATCCATGGAAACCAACCATA
CCAACTCCATGGAGATATTATTTTCAATGGGATAGAACATTAATACCATCTCATACTGGAACTAGTGG
CACACCAACAAATATATACCATGGTACAGATCCAGATGATGTTCAATTTTATACTATTGAAAATTCTG
TGCCAGTACACTTACTAAGAACAGGTGATGAATTTGCTACAGGAACATTTTTTTTTGATTGTAAACCA
TGTAGACTAACACATACATGGCAAACAAATAGAGCATTGGGCTTACCACCATTTCTAAATTCTTTGCC
TCAAGCTGAAGGAGGTACTAACTTTGGTTATATAGGAGTTCAACAAGATAAAAGACGTGGTGTAACTC
AAATGGGAAATACAAACTATATTACTGAAGCTACTATTATGAGACCAGCTGAGGTTGGTTATAGTGCA
CCATATTATTCTTTTGAGGCGTCTACACAAGGGCCATTTAAAACACCTATTGCAGCAGGACGGGGGGG
AGCGCAAACAGATGAAAATCAAGCAGCAGATGGTGATCCAAAATATGCATTTGGTAGACAACATGGTC
AAAAAACTACCACAACAGGAGAAACACCTGAGAGATTTACATATATAGCACATCAAGATACAGGAAGA
TATCCAGAAGGAGATTGGATTCAAAATATTAACTTTAACCTTCCTGTAACAGAAGATAATGTATTGCT
ACCAACAGATCCAATTGGAGGTAAAACAGGAATTAACTATACTAATATATTTAATACTTATGGTCCTT
TAACTGCATTAAATAATGTACCACCAGTTTATCCAAATGGTCAAATTTGGGATAAAGAATTTGATACT
GACTTAAAACCAAGACTTCATGTAAATGCACCATTTGTTTGTCAAAATAATTGTCCTGGTCAATTATT
TGTAAAAGTTGCGCCTAATTTAACAAATGAATATGATCCTGATGCATCTGCTAATATGTCAAGAATTG
TAACTTACTCAGATTTTTGGTGGAAAGGTAAATTAGTATTTAAAGCTAAACTAAGAGCCTCTCATACT

TGGAATCCAATTCAACAAATGAGTATTAATGTAGATAACCAATTTAACTATGTACCAAGTAATATTGG
AGGTATGAAAATTGTATATGAAAAATCTCAACTAGCACCTAGA
'USA_KJ813851_Bobcat_ND_1168_2013'
ATGAGTGATGGAGCAGTTCAACCAGACGGTGGTCAGCCTGCTGTCAGAAATGAAAGAGCTACAGGATC
TGGGAACGGGTCTGGAGGCGGGGGTGGTGGTGGTTCTGGGGGTGTGGGGATTTCTACGGGTACTTTCA
ATAATCAGACGGAATTTAAATTTTTGGAAAACGGATGGGTGGAAATCACAGCAAACTCAAGCAGACTT
GTACATTTAAATATGCCAGAAAGTGAAAATTATAGAAGAGTGGTTGTAAATAATTTGGATAAAACTGC
AGTTAACGGAAACATGGCTTTAGATGATACTCATGCACAAATTGTAACACCTTGGTCATTGGTTGATG
CAAATGCTTGGGGAGTTTGGTTTAATCCAGGAGATTGGCAACTAATTGTTAATACTATGAGTGAGTTG
CATTTAGTTAGTTTTGAACAAGAAATTTTTAATGTTGTTTTAAAGACTGTTTCAGAATCTGCTACTCA
GCCACCAACTAAAGTTTATAATAATGATTTAACTGCATCATTGATGGTTGCATTAGATAGTAATAATA
CTATGCCATTTACTCCAGCAGCTATGAGATCTGAGACATTGGGTTTTTATCCATGGAAACCAACCATA
CCAACTCCATGGAGATATTATTTTCAATGGGATAGAACATTAATACCATCTCATACTGGAACTAGTGG
CACACCAACAAATATATACCATGGTACAGATCCAGATGATGTTCAATTTTATACCATTGAAAATTCTG
TGCCAGTACACTTACTAAGAACAGGTGATGAATTTGCTACAGGAACATTTTTTTTTGATTGTAAACCA
TGTAGACTAACACATACATGGCAAACAAATAGAGCATTGGGCTTACCACCATTTCTAAATTCTTTGCC
TCAAGCTGAAGGAGGTACTAACTTTGGTTATATAGGAGTTCAACAAGATAAAAGACGTGGTGTAACTC
AAATGGGAAATACAAACTATATTACTGAAGCTACTATTATGAGACCAGCTGAGGTTGGTTATAGTGCA
CCATATTATTCTTTTGAGGCGTCTACACAAGGGCCATTTAAAACACCTATTGCAGCAGGACGGGGGGG
AGCGCAAACAGATGAAAATCAAGCAGCAGATGGTGATCCAAGATATGCATTTGGTAGACAACATGGTC
AAAAAACTACCACAACAGGAGAAACACCTGAGAGATTTACATATATAGCACATCAAGATACAGGAAGA
TATCCAGAAGGAGATTGGATTCAAAATATTAACTTTAACCTTCCTGTAACAGATGATAATGTATTGCT
ACCAACAGATCCAATTGGAGGTAAAACAGGAATTAACTATACTAATATATTTAATACTTATGGTCCTT
TAACTGCATTAAATAATGTACCACCAGTTTATCCAAATGGTCAAATTTGGGATAAAGAATTTGATACT
GACTTAAAACCAAGACTTCATGTAAATGCACCATTTGTTTGTCAAAATAATTGCCCTGGTCAATTATT
TGTAAAAGTTGCGCCTAATTTAACAAATGAATATGATCCTGATGCATCTGCTAATATGTCAAGAATTG
TAACTTACTCAGATTTTTGGTGGAAAGGTAAATTAGTATTTAAAGCTAAACTAAGAGCCTCTCATACT
TGGAATCCAATTCAACAAATGAGTATTAATGTAGATAACCAATTTAACTATGTACCAAGTAATATTGG
AGGTATGAAAATTGTCTATGAAAAATCTCAACTAGCACCTAGA
'USA_KJ813852_Bobcat_ND_1170_2013'
ATGAGTGATGGAGCAGTTCAACCAGACGGTGGTCAGCCTGCTGTCAGAAATGAAAGAGCTACAGGATC
TGGGAACGGGTCTGGAGGCGGGGGTGGTGGTGGTTCTGGGGGTGTGGGGATTTCTACGGGTACTTTCA
ATAATCAGACGGAATTTAAATTTTTGGAAAACGGATGGGTGGAAATCACAGCAAACTCAAGCAGACTT
GTACATTTAAATATGCCAGAAAGTGAAAATTATAGAAGAGTGGTTGTAAATAATTTGGATAAAACTGC
AGTTAACGGAAACATGGCTTTAGATGATACTCATGCACAAATTGTAACACCTTGGTCATTAGTTGATG
CAAATGCTTGGGGAGTTTGGTTTAATCCAGGAGATTGGCAACTAATTGTTAATACTATGAGTGAGTTG
CATTTAGTTAGTTTTGAACAAGAAATTTTTAATGTTGTTTTAAAGACTGTTTCAGAATCTGCTACTCA
GCCACCAACTAAAGTTTATAATAATGATTTAACTGCATCATTGATGGTTGCATTAGATAGTAATAATA
CTATGCCATTTACTCCAGCAGCTATGAGATCTGAGACATTGGGTTTTTATCCATGGAAACCAACCATA
CCAACTCCATGGAGATATTATTTTCAATGGGATAGAACATTAATACCATCTCATACTGGAACTAGTGG
CACACCAACAAATATATACCATGGTACAGATCCAGATGATGTTCAATTTTATACTATTGAAAATTCTG
TGCCAGTACACTTACTAAGAACAGGTGATGAATTTGCTACAGGAACATTTTTTTTTGATTGTAAACCA
TGTAGACTAACACATACATGGCAAACAAATAGAGCATTGGGCTTACCACCATTTCTAAATTCTTTGCC
TCAAGCTGAAGGAGGTACTAACTTTGGTTATATAGGAGTTCAACAAGATAAAAGACGTGGTGTAACTC
AAATGGGAAATACAAACTATATTACTGAAGCTACTATTATGAGACCAGCTGAGGTTGGTTATAGTGCA
CCATATTATTCTTTTGAGGCGTCTACACAAGGGCCATTTAAAACACCTATTGCAGCAGGACGGGGGGG
AGCGCAAACAGATGAAAATCAAGCAGCAGATGGTGATCCAAGATATGCATTTGGTAGACAACATGGTC
AAAAAACTACCACAACAGGAGAAACACCTGAGAGATTTACATATATAGCACATCAAGATACAGGAAGA
TATCCAGAAGGAGATTGGATTCAAAATATTAACTTTAACCTTCCTGTAACAGATGATAATGTATTGCT
ACCAACAGATCCAATTGGAGGTAAAACAGGAATTAACTATACTAATATATTTAATACTTATGGTCCTT
TAACTGCATTAAATAATGTACCACCAGTTTATCCAAATGGTCAAATTTGGGATAAAGAATTTGATACT
GACTTAAAACCAAGACTTCATGTAAATGCACCATTTGTTTGTCAAAATAATTGCCCTGGTCAATTATT
TGTAAAAGTTGCGCCTAATTTAACAAATGAATATGATCCTGATGCATCTGCTAATATGTCAAGAATTG
TAACTTACTCAGATTTTTGGTGGAAAGGTAAATTAGTATTTAAAGCTAAACTAAGAGCCTCTCATACT

TGGAATCCAATTCAACAAATGAGTATTAATGTAGATAACCAATTTAACTATGTACCAAGTAATATTGG
AGGTATGAAAATTGTCTATGAAAAATCTCAACTAGCACCTAGA
'USA_KJ813854_Puma_ND_F205_2013'
ATGAGTGATGGAGCAGTTCAACCAGACGGTGGTCAACCTGCTGTCAGAAATGAAAGAGCAACAGGATC
TGGGAACGGGTCTGGAGGCGGGGGTGGTGGTGGTTCTGGGGGTGTGGGGATTTCTACGGGTACTTTCA
ATAATCAGACGGAATTTAAATTTTTGGAAAACGGATGGGTGGAAATCACAGCAAACTCAAGCAGACTT
GTACATTTAAATATGCCAGAAAGTGAAAATTATAGAAGAGTGGTTGTAAATAATTTGGATAAAACTGC
AGTTAACGGAAACATGGCTTTAGATGATACTCATGCACAAATTGTAACACCTTGGTCATTGGTTGATG
CAAATGCTTGGGGAGTTTGGTTTAATCCAGGAGATTGGCAACTAATTGTTAATACTATGAGTGAGTTG
CATTTAGTTAGTTTTGAACAAGAAATTTTTAATGTTGTTTTAAAGACTGTTTCAGAATCTGCTACTCA
GCCACCAACTAAAGTTTATAATAATGATTTAACTGCATCATTGATGGTTGCATTAGATAGTAATAATA
CTATGCCATTTACTCCAGCAGCTATGAGATCTGAGACATTGGGTTTTTATCCATGGAAACCAACCATA
CCAACTCCATGGAGATATTATTTTCAATGGGATAGAACATTAATACCATCTCATACTGGAACTAGTGG
CACACCAACAAATATATACCATGGTACAGATCCAGATGATGTTCAATTTTATACTATTGAAAATTCTG
TGCCAGTACACTTACTAAGAACAGGTGATGAATTTGCTACAGGAACATTTTTTTTTGATTGTAAACCA
TGTAGACTAACACATACATGGCAAACAAATAGAGCATTGGGCTTACCACCATTTCTAAATTCTTTGCC
TCAAGCTGAAGGAGGTACTAACTTTGGTTATATAGGAGTTCAACAAGATAAAAGACGTGGTGTAACTC
AAATGGGAAATACAAACTATATTACTGAAGCTACTATTATGAGACCAGCTGAGGTTGGTTATAGTGCA
CCATATTATTCTTTTGAGGCGTCTACACAAGGGCCATTTAAAACACCTATTGCAGCAGGACGGGGGGG
AGCGCAAACAGATGAAAATCAAGCAGCAGATGGTGATCCAAGATATGCATTTGGTAGACAACATGGTC
AAAAAACTACCACAACAGGAGAAACACCTGAGAGATTTACATATATAGCACATCAAGATACAGGAAGA
TATCCAGAAGGAGATTGGATTCAAAATATTAACTTTAACCTTCCTGTAACAGAAGATAATGTATTGCT
ACCAACAGATCCAATTGGAGGCAAAACAGGAATTAACTATACTAATATATTTAATACTTATGGTCCTT
TAACTGCATTAAATAATGTACCACCAGTTTATCCAAATGGTCAAATTTGGGATAAAGAATTTGATACT
GACTTAAAACCAAGACTTCATGTAAATGCACCATTTGTTTGTCAGAATAATTGTCCTGGTCAATTATT
TGTAAAAGTTGCGCCTAATTTAACAAATGAATATGATCCTGATGCATCTGCTAATATGTCAAGAATTG
TAACTTACTCAGATTTTTGGTGGAAAGGTAAATTAGTATTTAAAGCTAAACTAAGAGCCTCTCATACT
TGGAATCCAATTCAACAAATGAGTATTAATGTAGATAACCAATTTAACTATGTACCAAGTAATATTGG
AGGTATGAAAATTGTATATGAAAAATCTCAACTAGCACCTAGA
'USA_KJ813858_Puma_ND_F93_2013'
ATGAGTGATGGAGCAGTTCAACCAGACGGTGGTCAACCTGCTGTCAGAAATGAAAGAGCAACAGGATC
TGGGAACGGGTCTGGAGGCGGGGGTGGTGGTGGTTCTGGGGGTGTGGGGATTTCTACGGGTACTTTCA
ATAATCAGACGGAATTTAAATTTTTGGAAAACGGATGGGTGGAAATCACAGCAAACTCAAGCAGACTT
GTACATTTAAATATGCCAGAAAGTGAAAATTATAGAAGAGTGGTTGTAAATAATTTGGATAAAACTGC
AGTTAACGGAAACATGGCTTTAGATGATACTCATGCACAGATTGTAACACCTTGGTCATTGGTTGATG
CAAATGCTTGGGGAGTTTGGTTTAATCCAGGAGATTGGCAACTAATTGTTAATACTATGAGTGAGTTG
CATTTAGTTAGTTTTGAACAAGAAATTTTTAATGTTGTTTTAAAGACTGTTTCAGAATCTGCTACTCA
GCCACCAACTAAAGTTTATAATAATGATTTAACTGCATCATTGATGGTTGCATTAGATAGTAATAATA
CTATGCCATTTACTCCAGCAGCTATGAGATCTGAGACATTGGGTTTTTATCCATGGAAACCAACCATA
CCAACTCCATGGAGATATTATTTTCAATGGGATAGAACATTAATACCATCTCATACTGGAACTAGTGG
CACACCAACAAATATATACCATGGTACAGATCCAGATGATGTTCAATTTTATACTATTGAAAATTCTG
TGCCAGTACACTTACTAAGAACAGGTGATGAATTTGCTACAGGAACATTTTTTTTTGATTGTAAACCA
TGTAGACTAACACATACATGGCAAACAAATAGAGCATTGGGCTTACCACCATTTCTAAATTCTTTGCC
TCAAGCTGAAGGAGGTACTAACTTTGGTTATATAGGAGTTCAACAAGATAAAAGACGTGGTGTAACTC
AAATGGGAAATACAAACTATATTACTGAAGCTACTATTATGAGACCAGCTGAGGTTGGTTATAGTGCA
CCATATTATTCTTTTGAGGCGTCTACACAAGGGCCATTTAAAACACCTATTGCAGCAGGACGGGGGGG
AGCGCAAACAGATGAAAATCAAGCAGCAGATGGTGATCCAAGATATGCATTTGGTAGACAACATGGTC
AAAAAACTACCACAACAGGAGAAACACCTGAGAGATTTACATATATAGCACATCAAGATACAGGAAGA
TATCCAGAAGGAGATTGGATTCAAAATATTAACTTTAACCTTCCTGTAACAGAAGATAATGTATTGCT
ACCAACAGATCCAATTGGAGGTAAAACAGGAATTAACTATACTAATATATTTAATACTTATGGTCCTT
TAACTGCATTAAATAATGTACCACCAGTTTATCCAAATGGTCAAATTTGGGATAAAGAATTTGATACT
GACTTAAAACCAAGACTTCATGTAAATGCACCATTTGTTTGTCAAAATAATTGTCCTGGTCAATTATT
TGTAAAAGTTGCGCCTAATTTAACAAATGAATATGATCCTGATGCATCTGCTAATATGTCAAGAATTG
TAACTTACTCAGATTTTTGGTGGAAAGGTAAATTAGTATTTAAAGCTAAACTAAGAGCCTCTCATACT

TGGAATCCAATTCAACAAATGAGTATTAATGTAGATAACCAATTTAACTATGTACCAAGTAATATTGG
AGGTATGAAAATTGTATATGAAAAATCTCAACTAGCACCTAGA
'USA_KJ813870_Raccoon_TX_1_2013'
ATGAGTGATGGAGCAGTTCAACCAGACGGTGGTCAACCTGCTGTCAGAAATGAAAGAGCTACAGGATC
TGGGAACGGGTCTGGAGGCGGGGGTGGTGGTGGTTCTGGGGGTGTGGGGATTTCTACGGGTACTTTCA
ATAATCAGACGGAATTTAAATTTTTGGAAAACGGATGGGTGGAAATCACAGCAAACTCAAGCAGACTT
GTACATTTAAATATGCCAGAAAGTGAAAATTATAGAAGAGTGGTTGTAAATAATTTGGATAAAACTGC
AGTTAACGGAAACATGGCTTTAGATGATACTCATGCACAAATTGTAACACCTTGGTCATTGGTTGATG
CAAATGCTTGGGGAGTTTGGTTTAATCCAGGAGATTGGCAACTAATTGTTAATACTATGAGTGAGTTG
CATTTAGTTAGTTTTGAACAAGAAATTTTTAATGTTGTTTTAAAGACTGTTTCAGAATCTGCTACTCA
GCCACCAACTAAAGTTTATAATAATGATTTAACTGCATCATTGATGGTTGCATTAGATAGTAATAATA
CTATGCCATTTACTCCAGCAGCTATGAGATCTGAGACATTGGGTTTTTATCCATGGAAACCAACCATA
CCAACTCCATGGAGATATTATTTTCAATGGGATAGAACATTAATACCATCTCATACTGGAACTAGTGG
CACACCAACAAATACATACCATGGTACAGATCCAGATGATGTTCAATTTTATACTATTGAAAATTCTG
TGCCAGTACACTTACTAAGAACAGGTGATGAATTTGCTACAGGAACATTTTTTTTTGATTGTAAACCA
TGTAGACTAACACATACATGGCAAACAAATAGAGCATTGGGCTTACCACCATTTCTAAATTCTTTGCC
TCAATCTGAAGGAGATACTAACTTTGGTGATATAGGAGTTCAACAAGATAAAAGACGTGGTGTAACTC
AAATGGGAAATACAAACTATATTACTGAAGCTACTATTATGAGACCAGCTGAGGTTGGTTATAGTGCA
CCATATTATTCTTTTGAGGCGTCTACACAAGGGCCATTTAAAACACCTATTGCAGCAGGACGGGGGGG
AGCACAAACAGATGAAAATCAAGCAGCAGATGGTGATCCAAGATATGCATTTGGTAGACAACATGGTC
AAAAAACTACCACAACAGGAGAAACACCTGAGAGATTTACATATATAGCACATCAAGATACAGGAAGA
TATCCAGAAGGAGATTGGATTCAAAATATTAACTTTAACCTTCCTGTAACAAATGATAATGTATTGCT
ACCAACAGATCCAATTGGAGGTAAAACAGGAATTAACTATACTAATATATTTAATACTTATGGTCCTT
TAACTGCATTAAATAATGTACCACCAGTTTATCCAAATGGTCAAATTTGGGATAAAGAATTTGATACT
GACTTAAAACCAAGACTTCATGTAAATGCACCATTTGTTTGTCAAAATAATTGTCCTGGTCAATTATT
TGTAAAAGTTGCGCCTAATTTAACAAATGAATATGATCCTGATGCATCTGCTAATATGTCAAGAATTG
TAACTTACTCAGATTTTTGGTGGAAAGGTAAATTAGTATTTAAAGCTAAACTAAGAGCCTCTCATACT
TGGAATCCAATTCAACAAATGAGTATTAATGTAGATAACCAATTTAACTATGTACCAAGTAATATTGG
AGGTATGAAAATTGTATATGAAAAATCTCAACTAGCACCTAGA
'USA_KJ813873_Graywolf_MI_850_2012'
ATGAGTGATGGAGCAGTTCAACCAGACGGTGGTCAGCCTGCTGTCAGAAATGAAAGAGCTACAGGATC
TGGGAACGGGTCTGGAGGCGGGGGTGGTGGTGGTTCTGGGGGTGTGGGGATTTCTACGGGTACTTTCA
ATAATCAGACGGAATTTAAATTTTTGGAAAACGGATGGGTGGAAATCACAGCAAACTCAAGCAGACTT
GTACATTTAAATATGCCAGAAAGTGAAAATTATAGAAGAGTGGTTGTAAATAATTTGGATAAAACTGC
AGTTAACGGAAACATGGCTTTAGATGATACTCATGCACAAATTGTAACACCTTGGTCATTGGTTGATG
CAAATGCTTGGGGAGTTTGGTTTAATCCAGGAGATTGGCAACTAATTGTTAATACTATGAGTGAGTTG
CATTTAGTTAGTTTTGAACAAGAAATTTTTAATGTTGTTTTAAAGACTGTTTCAGAATCTGCTACTCA
GCCACCAACTAAAGTTTATAATAATGATTTAACTGCATCATTGATGGTTGCATTAGATAGTAATAATA
CTATGCCATTTACTCCAGCAGCTATGAGATCTGAGACATTGGGTTTTTATCCATGGAAACCAACCATA
CCAACTCCATGGAGATATTATATTCAATGGGATAGAACATTAATACCATCTCATACTGGAACTAGTGG
CACACCAACAAATATATACCATGGTACAGATCCAGATGATGTTCAATTTTATACTATTGAAAATTCTG
TGCCAGTACACTTACTAAGAACAGGTGATGAATTTGCTACAGGAACATTTTTTTTTGATTGTAAACCA
TGTAGACTAACACATACATGGCAAACAAATAGAGCATTGGGCTTACCACCATTTCTAAATTCTTTGCC
TCAAGCTGAAGGAGGTACTAACTTTGGTTATATAGGAGTTCAACAAGATAAAAGACGTGGTGTAACTC
AAATGGGAAATACAAACTATATTACTGAAGCTACTATTATGAGACCAGCTGAGGTTGGTTATAGTGCA
CCATATTATTCTTTTGAGGCGTCTACACAAGGGCCATTTAAAACACCTATTGCAGCAGGACGGGGGGG
AGCGCAAACAGATGAAAATCAAGCAGCAGATGGTGATCCAAGATATGCATTTGGTAGACAACATGGTC
AAAAAACTACCACAACAGGAGAAACACCTGAGAGATTTACATATATAGCACATCAAGATACAGGAAGA
TATCCAGAAGGAGATTGGATTCAAAATATTAACTTTAACCTTCCTGTAACAGATGATAATGTATTGCT
ACCAACAGATCCAATTGGAGGTAAAACAGGAATTAACTATACTAATATATTTAATACTTATGGTCCTT
TAACTGCATTAAATAATGTACCACCAGTTTATCCAAATGGTCAAATTTGGGATAAAGAATTTGATACT
GACTTAAAACCAAGACTTCATGTAAATGCACCATTTGTTTGTCAGAATAATTGCCCTGGTCAATTATT
TGTAAAAGTTGCGCCTAATTTAACAAATGAATATGATCCTGATGCATCTGCTAATATGTCAAGAATTG
TAACTTACTCAGATTTTTGGTGGAAAGGTAAATTAGTATTTAAAGCTAAACTAAGAGCCTCTCATACT

TGGAATCCAATTCAACAAATGAGTATTAATGTAGATAACCAATTTAACTATGTACCAAGTAATATTGG
AGGTATGAAAATTGTCTATGAAAAATCTCAACTAGCACCTAGA
'USA_KJ813881_Graywolf_MI_832_2012'
ATGAGTGATGGAGCAGTTCAACCAGACGGTGGTCAGCCTGCTGTCAGAAATGAAAGAGCTACAGGATC
TGGGAACGGGTCTGGAGGCGGGGGTGGTGGTGGTTCTGGGGGTGTGGGGATTTCTACGGGTACTTTCA
ATAATCAGACGGAATTTAAATTTTTGGAAAACGGATGGGTGGAAATCACAGCAAACTCAAGCAGACTT
GTACATTTAAATATGCCAGAAAGTGAAAATTATAGGAGAGTGGTTGTAAATAATTTGGATAAAACTGC
AGTTAACGGAAACATGGCTTTAGATGATACTCATGCACAAATTGTAACACCTTGGTCATTGGTTGATG
CAAATGCTTGGGGAGTTTGGTTTAATCCAGGAGATTGGCAACTAATTGTTAATACTATGAGTGAGTTG
CATTTAGTTAGTTTTGAACAAGAAATTTTTAATGTTGTTTTAAAGACTGTTTCAGAATCTGCTACTCA
GCCACCAACTAAAGTTTATAATAATGATTTAACTGCATCATTGATGGTTGCATTAGATAGTAATAATA
CTATGCCATTTACTCCAGCAGCTATGAGATCTGAGACATTGGGTTTTTATCCATGGAAACCAACCATA
CCAACTCCATGGAGATATTATTTTCAATGGGATAGAACATTAATACCATCTCATACTGGAACTAGTGG
CACACCAACAAATATATACCACGGTACAGATCCAGATGATGTTCAATTTTATACTATTGAAAATTCTG
TGCCAGTACACTTACTAAGAACAGGTGATGAATTTGCTACAGGAACATTTTTTTTTGATTGTAAACCA
TGTAGACTAACACATACATGGCAAACAAATAGAGCATTGGGCTTACCACCATTTCTAAATTCTTTGCC
TCAAGCTGAAGGAGGTACTAACTTTGGTTATATAGGAGTTCAACAAGATAAAAGACGTGGTGTAACTC
AAATGGGAAATACAAACTATATTACTGAAGCTACTATTATGAGACCAGCTGAGGTTGGTTATAGTGCA
CCATATTATTCTTTTGAGGCGTCTACACAAGGGCCATTTAAAACACCTATTGCAGCAGGACGGGGGGG
AGCGCAAACAGATGAAAATCAAGCAGCAGATGGTGATCCAAGATATGCATTTGGTAGACAACATGGTC
AAAAAACTACCACAACAGGAGAAACACCTGAGAGATTTACATATATAGCACATCAAGATACAGGAAGA
TATCCAGAAGGAGATTGGATTCAAAATATTAACTTTAACCTTCCTGTAACAGATGATAATGTATTGCT
ACCAACAGATCCAATTGGAGGTAAAACAGGAATTAACTATACTAATATATTTAATACTTATGGTCCTT
TAACTGCATTAAATAATGTACCACCAGTTTATCCAAATGGTCAAATTTGGGATAAAGAATTTGATACT
GACTTAAAACCAAGACTTCATGTAAATGCACCATTTGTTTGTCAAAATAATTGCCCTGGTCAATTATT
TGTAAAAGTTGCGCCTAATTTAACAAATGAATATGATCCTGATGCATCTGCTAATATGTCAAGAATTG
TAACTTACTCAGATTTTTGGTGGAAAGGTAAATTAGTATTTAAAGCTAAACTAAGAGCCTCTCATACT
TGGAATCCAATTCAACAAATGAGTATTAATGTAGATAACCAATTTAACTATGTACCAAGTAATATTGG
AGGTATGAAAATTGTCTATGAAAAATCTCAACTAGCACCTAGA
'USA_KJ813882_Raccoon_NJ_1423_2012'
ATGAGTGATGGAGCAGTTCAACCAGACGGTGGTCAGCCTGCTGTCAGAAATGAAAGAGCTACAGGATC
TGGGAACGGGTCTGGAGGCGGGGGTGGTGGTGGTTCTGGGGGTGTGGGGATTTCTACGGGTACTTTCA
ATAATCAGACGGAATTTAAATTTTTGGAAAACGGATGGGTGGAAATCACAGCAAACTCAAGCAGACTT
GTACATTTAAATATGCCAGAAAGTGAAAATTATAGAAGAGTGGTTGTAAATAATTTGGATAAAACTGC
AGTTAACGGAAACATGGCTTTAGATGATACTCATGCACAAATTGTAACACCTTGGTCATTGGTTGATG
CAAATGCTTGGGGAGTTTGGTTTAATCCAGGAGATTGGCAACTAATTGTTAATACTATGAGTGAGTTG
CATTTAGTTAGTTTTGAACAAGAAATTTTTAATGTTGTTTTAAAGACTGTTTCAGAATCTGCTACTCA
GCCACCAACTAAAGTTTATAATAATGATTTAACTGCATCATTGATGGTTGCATTAGATAGTAATAATA
CTATGCCATTTACTCCAGCAGCTATGAGATCTGAGACATTGGGTTTTTATCCATGGAAACCAACCATA
CCAACTCCATGGAGATATTATTTTCAATGGGATAGAACATTAATACCATCTCATACTGGAACTAGTGG
CACACCAACAAATATATACCATGGTACAGATCCAGATGATGTTCAATTTTATACTATTGAAAATTCTG
TGCCAGTACACTTACTAAGAACAGGTGATGAATTTGCTACAGGAACATTTTTTTTTGATTGTAAACCA
TGTAGACTAACACATACATGGCAAACAAATAGAGCATTGGGCTTACCACCATTTCTAAATTCTTTGCC
TCAAGCTGAAGGAGGTACTAACTTTGGTTATATAGGAGTTCAACAAGATAAAAGACGTGGTGTAACTC
AAATGGGAAATACAAACTATATTACTGAAGCTACTATTATGAGACCAGCTGAGGTTGGTTATAGTGCA
CCATATTATTCTTTTGAGGCGTCTACACAAGGGCCATTTAAAACACCTATTGCAGCAGGACGGGGGGG
AGCGCAAACAGATGAAAATCAAGCAGCAGATGGTGATCCAAGATATGCATTTGGTAGACAACATGGTC
AAAAAACTACCACAACAGGAGAAACACCTGAGAGATTTACATATATAGCACATCAAGATACAGGAAGA
TATCCAGAAGGAGATTGGATTCAAAATATTAACTTTAACCTTCCTGTAACAGATGATAATGTATTGCT
ACCAACAGATCCAATTGGAGGTAAAACAGGAATTAACTATACTAATATATTTAATACTTATGGTCCTT
TGACTGCATTAAATAATGTACCACCAGTTTATCCAAATGGTCAAATTTGGGATAAAGAATTTGATACT
GACTTAAAACCAAGACTTCATGTAAATGCACCATTTGTTTGTCAAAATAATTGCCCTGGTCAATTATT
TGTAAAAGTTGCGCCTAATTTAACAAATGAATATGATCCTGATGCATCTGCTAATATGTCAAGAATTG
TAACTTACTCAGATTTTTGGTGGAAAGGTAAATTAGTATTTAAAGCTAAACTAAGAGCCTCTCATACT

TGGAATCCAATTCAACAAATGAGTATTAATGTAGATAACCAATTTAACTATGTACCAAGTAATATTGG
AGGTATGAAAATTGTCTATGAAAAATCTCAACTAGCACCTAGA
'USA_KJ813888_Coyote_MT_878_2012'
ATGAGTGATGGAGCAGTTCAACCAGACGGTGGTCAACCTGCTGTCAGAAATGAAAGAGCAACAGGATC
TGGGAACGGGTCTGGAGGCGGGGGTGGTGGTGGTTCTGGGGGTGTGGGGATTTCTACGGGTACTTTCA
ATAATCAGACGGAATTTAAATTTTTGGAAAACGGATGGGTGGAAATCACAGCAAACTCAAGCAGACTT
GTACATTTAAATATGCCAGAAAGTGAAAATTATAGAAGAGTGGTTGTAAATAATTTGGATAAAACTGC
AGTTAACGGAAACATGGCTTTAGATGATACTCATGCACAAATTGTAACACCTTGGTCATTGGTTGATG
CAAATGCTTGGGGAGTTTGGTTTAATCCAGGAGATTGGCAACTAATTGTTAATACTATGAGTGAGTTG
CATTTAGTTAGTTTTGAACAAGAAATTTTTAATGTTGTTTTAAAGACTGTTTCAGAATCTGCTACTCA
GCCACCAACTAAAGTTTATAATAATGATTTAACTGCATCATTGATGGTTGCATTAGATAGTAATAATA
CTATGCCATTTACTCCAGCAGCTATGAGATCTGAGACATTGGGTTTTTATCCATGGAAACCAACCATA
CCAACTCCATGGAGATATTATATTCAATGGGATAGAACATTAATACCATCTCATACTGGAACTAGTGG
CACACCAACAAATATATACCATGGTACAGATCCAGATGATGTTCAATTTTATACTATTGAAAATTCTG
TGCCAGTACACTTACTAAGAACAGGTGATGAATTTGCTACAGGAACATTTTTTTTTGATTGTAAACCA
TGTAGACTAACACATACATGGCAAACAAATAGAGCATTGGGCTTACCACCATTTCTAAATTCTTTGCC
TCAAGCTGAAGGAGGTACTAACTTTGGTTATATAGGAGTTCAACAAGATAAAAGACGTGGTGTAACTC
AAATGGGAAATACAAACTATATTACTGAAGCTACTATTATGAGACCAGCTGAGGTTGGTTATAGTGCA
CCATATTATTCTTTTGAGGCGTCTACACAAGGGCCATTTAAAACACCTATTGCAGCAGGACGGGGGGG
AGCGCAAACAGATGAAAATCAAGCAGCAGATGGTGATCCAAGATATGCATTTGGTAGACAACATGGTC
AAAAAACTACCACAACAGGAGAAACACCTGAGAGATTTACATATATAGCACATCAAGATACAGGAAGA
TATCCAGAAGGAGATTGGATTCAAAATATTAACTTTAACCTTCCTGTAACAGAAGATAATGTATTGCT
ACCAACAGATCCAATTGGAGGTAAAACAGGAATTAACTATACTAATATATTTAATACTTATGGTCCTT
TAACTGCATTAAATAATGTACCACCAGTTTATCCAAATGGTCAAATTTGGGATAAAGAATTTGATACT
GACTTAAAACCAAGACTTCATGTAAATGCACCATTTGTTTGTCAAAATAATTGTCCTGGTCAATTATT
TGTAAAAGTTGCGCCTAATTTAACAAATGAATATGATCCTGATGCATCTGCTAATATGTCAAGAATTG
TAACTTACTCAGATTTTTGGTGGAAAGGTAAATTAGTATTTAAAGCTAAACTAAGAGCCTCTCATACT
TGGAATCCAATTCAACAAATGAGTATTAATGTAGATAACCAATTTAACTATGTACCAAGTAATATTGG
AGGTATGAAAATTGTATATGAAAAATCTCAACTAGCACCTAGA
'USA_KJ813890_Redfox_MA_197_2012'
ATGAGTGATGGAGCAGTTCAACCAGACGGTGGTCAACCTGCTGTCAGAAATGAAAGAGCTACAGGATC
TGGGAACGGGTCTGGAGGCGGGGGTGGTGGTGGTTCTGGGGGTGTGGGGATTTCTACGGGTACTTTCA
ATAATCAGACGGAATTTAAATTTTTGGAAAACGGATGGGTGGAAATCACAGCAAACTCAAGCAGACTT
GTACATTTAAATATGCCAGAAAGTGAAAATTATAGAAGAGTGGTTGTAAATAATTTGGATAAAACTGC
AGTTAACGGAAACATGGCTTTAGATGATACTCATGCACAAATTGTAACACCTTGGTCATTGGTTGATG
CAAATGCTTGGGGAGTTTGGTTTAATCCAGGAGATTGGCAACTAATTGTTAATACTATGAGTGAGTTG
CATTTAGTTAGTTTTGAACAAGAAATTTTTAATGTTGTTTTAAAGACTGTTTCAGAATCTGCTACTCA
GCCACCAACTAAAGTTTATAATAATGATTTAACTGCATCATTGATGGTTGCATTAGATAGTAATAATA
CTATGCCATTTACTCCAGCAGCTATGAGATCTGAGACATTGGGTTTTTATCCATGGAAACCAACCATA
CCAACTCCATGGAGATATTATTTTCAATGGGATAGAACATTAATACCATCTCATACTAGAACTAGTGG
CACACCAACAAATATATACCATGGTACAGATCCAGATGATGTTCAATTTTATACTATTGAAAATTCTG
TGCCAGTACACTTACTAAGAACAGGTGATGAATTTGCTACAGGAACATTTTTTTTTGATTGTAAACCA
TGTAAACTAACACATACATGGCAAACAAATAGAGCATTGGGCTTACCACCATTTCTAAATTCTTTGCC
TCAAGCTGAAGGAGATACTAACTTTGGTGATATAGGAGTTCAACAAGATAAAAGACGTGGTGTAACTC
AAATGGGAAATACAAACTATATTACTGAAGCTACTATTATGAGACCAGCTGAGGTTGGTTATAGTGCA
CCATATTATTCTTTTGAGGCGTCTACACAAGGGCCATTTAAAACACCTATTGCAGCAGGACGGGGGGG
AGCGCAAACAGATGAAAATCAAGCAGCAGATGGTGATCCAAGATATGCATTTGGTAGACAACATGGTC
AAAAAACTACCACAACAGGAGAAACACCTGAGAGATTTACATATATAGCACATCAAGATACAGGAAGA
TATCCAGAAGGAGATTGGATTCAAAATATTAACTTTAACCTTCCTGTAACAAATGATAATGTATTGCT
ACCAACAGATCCAATTGGAGGTAAAACAGGAATTAACTATACTAATATATTTAATACTTATGGTCCTT
TAACTGCATTAAATAATGTACCACCAGTTTATCCAAATGGTCAAATTTGGGATAAAGAATTTGATACT
GACTTAAAACCAAGACTTCATGTAAATGCACCATTTGTTTGTCAAAATAATTGTCCTGGTCAATTATT
TGTAAAAGTTGCGCCTAATTTAACAAATGAATATGATCCTGATGCATCTGCTAATATGTCAAGAATTG
TAACTTACTCAGATTTTTGGTGGAAAGGTAAATTAGTATTTAAAGCTAAACTAAGAGCCTCTCATACT

TGGAATCCAATTCAACAAATGAGTATTAATGTAGATAACCAATTTAACTATGTACCAAGTAATATTGG
AGGTATGAAAATTGTATATGAAAAATCTCAACTAGCACCTAGA
'USA_KJ813892_Coyote_AK_218_2013'
ATGAGTGATGGAGCAGTTCAACCAGACGGTGGTCAGCCTGCTGTCAGAAATGAAAGAGCTACAGGATC
TGGGAACGGGTCTGGAGGCGGGGGTGGTGGTGGTTCTGGGGGTGTGGGGATTTCTACGGGTACTTTCA
ATAATCAAACGGAATTTAAATTTTTGGAAAACGGATGGGTGGAAATCACAGCAAACTCAAGCAGACTT
GTACATTTAAATATGCCAGAAAGTGAAAATTATAGAAGAGTGGTTGTAAATAATTTGGATAAAACTGC
AGTTAACGGAAACATGGCTTTAGATGATACTCATGCACAAATTGTAACACCTTGGTCATTGGTTGATG
CAAATGCTTGGGGAGTTTGGTTTAATCCAGGAGATTGGCAACTAATTGTTAATACTATGAGTGAGTTG
CATTTAGTTAGTTTTGAACAAGAAATTTTTAATGTTGTTTTAAAGACTGTTTCAGAATCTGCTACTCA
GCCACCAACTAAAGTTTATAATAATGATTTAACTGCATCATTGATGGTTGCATTAGATAGTAATAATA
CTATGCCATTTACTCCAGCAGCTATGAGATCTGAGACATTGGGTTTTTATCCATGGAAACCAACCATA
CCAACTCCATGGAGATATTATTTTCAATGGGATAGAACATTAATACCATCTCATACTGGAACTAGTGG
CACACCAACAAATATATACCATGGTACAGATCCAGATGATGTTCAATTTTATACTATTGAAAATTCTG
TGCCAGTACACTTACTAAGAACAGGTGATGAATTTGCTACAGGAACATTTTTTTTTGATTGTAAACCA
TGTAGACTAACACATACATGGCAAACAAATAGAGCATTGGGCTTACCACCATTTCTAAATTCTTTGCC
TCAAGCTGAAGGAGGTACTAACTTTGGTTATATAGGAGTTCAACAAGATAAAAGACGTGGTGTAACTC
AAATGGGAAATACAAACTATATTACTGAAGCTACTATTATGAGACCAGCTGAGGTTGGTTATAGTGCA
CCATATTATTCTTTTGAGGCGTCTACACAAGGGCCATTTAAAACACCTATTGCAGCAGGACGGGGGGG
AGCGCAAACAGATGAAAATCAAGCAGCAGATGGTGATCCAAGATATGCATTTGGTAGACAACATGGTC
AAAAAACTACAACAACAGGAGAAACACCTGAGAGATTTACATATATAGCACATCAAGATACAGGAAGA
TATCCAGAAGGAGATTGGATTCAAAATATTAACTTTAACCTTCCTGTAACAGATGATAATGTATTGCT
ACCAACAGATCCAATTGGAGGTAAAACAGGAATTAACTATACTAATATATTTAATACTTATGGTCCTT
TAACTGCATTAAATAATGTACCACCAGTTTATCCAAATGGTCAAATTTGGGATAAAGAATTTGATACT
GACTTAAAACCAAGACTTCATGTAAATGCACCATTTGTTTGTCAAAATAATTGCCCTGGTCAATTATT
TGTAAAAGTTGCGCCTAATTTAACAAATGAATATGATCCTGATGCATCTGCTAATATGTCAAGAATTG
TAACTTACTCAGATTTTTGGTGGAAAGGTAAATTAGTATTTAAAGCTAAACTAAGAGCCTCTCATACT
TGGAATCCAATTCAACAAATGAGTATTAATGTAGATAACCAATTTAACTATGTACCAAGTAATATTGG
AGGTATGAAAATTGTCTATGAAAAATCTCAACTAGCACCTAGA
'ARG_KM236569_Cuba_2013'
ATGAGTGATGGAGCAGTTCAACCAGACGGTGGTCAACCTGCTGTCAGAAATGAAAGAGCAACAGGATC
TGGGAACGGGTCTGGAGGCGGGGGTGGTGGTGGTTCTGGGGGTGTGGGGATTTCTACGGGTACTTTCA
ATAATCAGACGGAATTTAAATTTTTGGAAAACGGATGGGTGGAAATCACAGCAAACTCAAGCAGACTT
GTACATTTAAATATGCCAGAAAGTGAAAATTATAGAAGAGTGGTTGTAAATAATTTGGATAAAACTGC
AGTTAACGGAAACATGGCTTTAGATGATACTCATGCACAAATTGTAACACCTTGGTCATTGGTTGATG
CAAATGCTTGGGGAGTTTGGTTTAATCCAGGAGATTGGCAACTAATTGTTAATACTATGAGTGAGTTG
CATTTAGTTAGTTTTGAACAAGAAATTTTTAATGTTGTTTTAAAGACTGTTTCAGAATCTGCTACTCA
GCCACCAACTAAAGTTTATAATAATGATTTAACTGCATCATTGATGGTTGCATTAGATAGTAATAATA
CTATGCCATTTACTCCAGCAGCTATGAGATCTGAGACATTGGGTTTTTATCCATGGAAACCAACCATA
CCAACTCCATGGAGATATTATTTTCAATGGGATAGAACATTAATACCATCTCATACTGGAACCAGTGG
CACACCAACAAATATATACCATGGTACAGATCCAGATGATGTTCAATTTTATACTATTGAAAATTCTG
TGCCAGTACACTTACTAAGAACAGGTGATGAATTTGCTACAGGAACATTTTTTTTTGATTGTAAACCA
TGTAGACTAACACATACATGGCAAACAAATAGAGCATTGGGCTTACCACCATTTCTAAATTCTTTGCC
TCAAGCTGAAGGAGGTACTAACTTTGGTTATATAGGAGTTCAACAAGATAAAAGACGTGGTGTAACTC
AAATGGGAAACACAAACTATATTACTGAAGCTACTATTATGAGACCAGCTGAGGTTGGTTATAGTGCA
CCATATTATTCTTTTGAGGCGTCTACACAAGGGCCATTTAAAACACCTATTGCAGCAGGACGGGGGGG
AGCGCAAACAGATGAAAATCAAGCAGCAGATGGTGATCCAAGATATGCATTTGGTAGACAACATGGTC
AAAAAACTACCACAACAGGAGAAACACCTGAGAGATTTACATATATAGCACATCAAGATACAGGAAGA
TATCCAGAAGGAGATTGGATTCAAAATATTAACTTTAACCTTCCTGTAACAGAAGATAATGTATTGCT
ACCAACAGATCCAATTGGAGGTAAAACAGGAATTAACTATACTAATATATTTAATACTTATGGTCCTT
TAACTGCATTAAATAATGTACCACCAGTTTATCCAAATGGTCAAATTTGGGATAAAGAATTTGATACT
GACTTAAAACCAAGACTTCATGTAAATGCACCATTTGTTTGTCAAAATAATTGTCCTGGTCAATTATT
TGTAAAAGTTGCGCCTAATTTAACAAATGAATATGATCCTGATGCATCTGCTAATATGTCAAGAATTG
TAACTTACTCAGATTTTTGGTGGAAAGGTAAATTAGTATTTAAAGCTAAACTAAGAGCCTCTCATACT

TGGAATCCAATTCAACAAATGAGTATTAATGTAGATAACCAATTTAACTATGTACCAAGTAATATTGG
AGGTATGAAAATTGTATATGAAAAATCTCAACTAGCACCTAGA

'ARG_KM236572_NNGag_2012'

ATGAGTGATGGAGCAGTTCAACCAGACGGTGGTCAACCTGCTGTCAGAAATGAAAGAGCTACAGGATC
TGGGAACGGGTCTGGAGGCGGGGGTGGTGGTGGTTCTGGGGGTGTGGGGATTTCTACGGGTGCTTTCA
ATAATCAGACGGAATTTAAATTTTTGGAAAACGGATGGGTGGAAATCACAGCAAACTCAAGCAGACTT
GTACATTTAAATATGCCAGAAAGTGAAAATTATAGAAGAGTGGTTGTAAATAATATGGATAAAACTGC
AGTTAACGGAAACATGGCTTTAGATGATATTCATGCACAAATTGTAACACCTTGGTCATTGGTTGATG
CAAATGCTTGGGGAGTTTGGTTTAATCCAGGAGATTGGCAACTAATTGTTAATACTATGAGTGAGTTG
CATTTAGTTAGTTTTGAACAAGAAATTTTTAATGTTGTTTTAAAGACTGTTTCAGAATCTGCTACTCA
GCCACCAACTAAAGTTTATAATAATGATTTAACTGCATCATTGATGGTTGCATTAGATAGTAATAATA
CTATGCCATTTACTCCAGCAGCTATGAGATCTGAGACATTGGGTTTTTATCCATGGAAACCAACCATA
CCAACTCCATGGAGATATTATTTTCAATGGGATAGAACATTAATACCATCTCATACTGGAACTAGTGG
CACACCAACAAATATATACCATGGTACAGATCCAGATGATGTTCAATTTTATACTATTGAAAATTCTG
TGCCAGTACACTTACTAAGAACAGGTGATGAATTTGCTACAGGAACATTTTTTTTTGATTGTAGACCA
TGTAGACTAACACATACATGGCAAACAAATAGAGCATTGGGCTTACCACCATTTCTAAATTCTTTGCC
TCAATCTGAAGGAGATATTAACTTTGGTGATATAGGAGTTCAACAAGATAAAAGACGTGGTATAACTC
AAATGGGAAATACAAACTATATTACTGAAGCTACTATTATGAGACCAGCTGAGGTTGGTTATAGTGCA
CCATATTATTCTTTTGAGGCGTCTACACAAGGGCCATTTAAAACACCTATTGCAGCAGGACGGGGGGG
AGCGCAAACAGATGAAAATCAAGCAGCAGATGGTAATCCAAGATATGCATTTGGTAGACAACATGGTC
AAAAAACTACCACAACAGGAGAAACACCTGAGAGATTTACATATATAGCACATCAAGATACAGGAAGA
TATCCAGAAGGAGATTGGATTCAAAATATTAACTTTAACCTTCCTGTAACAAATGATAATGTATTGCT
ACCAACAGATCCAATTGGAGGTAAAACAGGAATTAACTATACTAATATATTTAATACTTATGGTCCTT
TAACTGCATTAAATAATGTACCACCAGTTTATCCAAATGGTCAAATTTGGGATAAAGAATTTGATACT
GACTTAAAACCAAGACTTCATGTAAATGCACCATTTGTTTGTCAAAATAATTGTCCTGGTCAATTATT
TGTAAAAGTTGCGCCTAATTTAACGAATGAATATGATCCTGATGCATCTGCTAATATGTCAAGAATTG
TAACTTACTCAGATTTTTGGTGGAAAGGTAAATTAGTATTTAAAGCTAAACTAAGAGCCTCTCATACT
TGGAATCCAATTCAACAAATGAGTATTAATGTAGATAACCAATTTAACTATGTACCAAGTAATATTGG
AGGTATGAAAATTGTATATGAAAAATCTCAACTAGCACCTAGA

'URU_KM457103_2c_UY12_2006'

ATGAGTGATGGAGCAGTTCAACCAGACGGTGGTCAACCTGCTGTCAGAAATGAAAGAGCAACAGGATC
TGGGAACGGGTCTGGAGGCGGGGGTGGTGGTGGTTCTGGGGGTGTGGGGATTTCTACGGGTACTTTCA
ATAATCAGACGGAATTTAAATTTTTGGAAAACGGATGGGTGGAAATCACAGCAAACTCAAGCAGACTT
GTACATTTAAATATGCCAGAAAGTGAAAATTATAGAAGAGTGGTTGTAAATAATTTGGATAAAACTGC
AGTTAACGGAAACATGGCTTTAGATGATACTCATGCACAAATTGTAACACCTTGGTCATTGGTTGATG
CAAATGCTTGGGGAGTTTGGTTTAATCCAGGAGATTGGCAACTAATTGTTGATACTATGAGTGAGTTG
CATTTAGTTAGTTTTGAACAAGAAATTTTTAATGTTGTTTTAAAGACTGTTTCAGAATCTGCTACTCA
GCCACCAACTAAAGTTTATAATAATGATTTAACTGCATCATTGATGGTTGCATTAGATAGTAATAATA
CTATGCCATTTACTCCAGCAGCTATGAGATCTGAGACATTGGGTTTTTATCCATGGAAACCAACCATA
CCAACTCCATGGAGATATTATTTTCAATGGGATAGAACATTAATACCATCTCATACTGGAACTAGTGG
CACACCAACAAATATATACCATGGTACAGATCCAGATGATGTTCAATTTTATACTATTGAAAATTCTG
TGCCAGTACACTTACTAAGAACAGGTGATGAATTTGCTACAGGAACATTTTTTTTTGATTGTAAACCA
TGTAGACTAACACATACATGGCAAACAAATAGAGCATTGGGCTTACCACCATTTCTAAATTCTTTGCC
TCAAGCTGAAGGAGGTACTAACTTTGGTTATATAGGAGTTCAACAAGATAAAAGACGTGGTGTAACTC
AAATGGGAAATACAAACTATATTACTGAAGCTACTATTATGAGACCAGCTGAGGTTGGTTATAGTGCA
CCATATTATTCTTTTGAGGCGTCTACACAAGGGCCATTTAAAACACCTATTGCAGCAGGACGGGGGGG
AGCGCAAACCGATGAAAATCAAGCAGCAGATGGTGATCCAAGATATGCATTTGGTAGACAACATGGTC
AAAAAACTACCACAACAGGAGAAACACCTGAGAGATTTACATATATAGCACATCAAGATACAGGAAGA
TATCCAGAAGGAGATTGGATTCAAAATATTAACTTTAACCTTCCTGTAACAGAAGATAATGTATTGCT
ACCAACAGATCCAATTGGAGGTAAAACAGGAATTAACTATACTAATATATTTAATACTTATGGTCCTT
TAACTGCATTAAATAATGTACCACCAGTTTATCCAAATGGTCAAATTTGGGATAAAGAATTTGATACT
GACTTAAAACCAAGACTTCATGTAAATGCACCATTTGTTTGTCAAAATAATTGTCCTGGTCAATTATT
TGTAAAAGTTGCGCCTAATTTAACAAATGAATATGATCCTGATGCATCTGCTAATATGTCAAGAATTG
TAACTTACTCAGATTTTTGGTGGAAAGGTAAATTAGTATTTAAAGCTAAACTAAGAGCCTCTCATACT

TGGAATCCAATTCAACAAATGAGTATTAATGTAGATAACCAATTTAACTATGTACCAAGTAATATTGG
AGGTATGAAAATTGTATATGAAAAATCTCAACTAGCACCTAGA
'URU_KM457104_2c_UY47_2006'
ATGAGTGATGGAGCAGTTCAACCAGACGGTGGTCAACCTGCTGTCAGAAATGAAAGAGCAACAGGATC
TGGGAACGGGTCTGGAGGCGGGGGTGGTGGTGGTTCTGGGGGTGTGGGGATTTCTACGGGTACTTTCA
ATAATCAGACGGAATTTAAATTTTTGGAAAACGGATGGGTGGAAATCACAGCAAACTCAAGCAGACTT
GTACATTTAAATATGCCAGAAAGTGAAAATTATAGAAGAGTGGTTGTAAATAATTTGGATAAAACTGC
AGTTAACGGAAACATGGCTTTAGATGATACTCATGCACAAATTGTAACACCTTGGTCATTGGTTGATG
CAAATGCTTGGGGAGTTTGGTTTAATCCAGGAGATTGGCAACTAATTGTTAATACTATGAGTGAGTTG
CATTTAGTTAGTTTTGAACAAGAAATTTTTAATGTTGTTTTAAAGACTGTTTCAGAATCTGCTACTCA
GCCACCAACTAAAGTTTATAATAATGATTTAACTGCACCATTGATGGTTGCATTAGATAGTAATAATA
CTATGCCATTTACTCCAGCAGCTATGAGATCTGAGACATTGGGTTTTTATCCATGGAAACCAACCATA
CCAACTCCATGGAGATATTATTTTCAATGGGATAGAACATTAATACCATCTCATACTGGAACTAGTGG
CACACCAACAAATATATACCATGGTACAGATCCAGATGATGTTCAATTTTATACTATTGAAAATTCTG
TGCCAGTACACTTACTAAGAACAGGTGATGAATTTGCTACAGGAACATTTTTTTTTGATTGTAAACCA
TGTAGACTAACACATACATGGCAAACAAATAGAGCATTGGGCTTACCACCATTTCTAAATTCTTTGCC
TCAAGCTGAAGGAGGTACTAACTTTGGTTATATAGGAGTTCAACAAGATAAAAGACGTGGTGTAACTC
AAATGGGAAATACAAACTATATTACTGAAGCTACTATTATGAGACCAGCTGAGGTTGGTTATAGTGCA
CCATATTATTCTTTTGAGGCGTCTACACAAGGGCCATTTAAAACACCTATTGCAGCAGGACGGGGGGG
AGCGCAAACAGATGAAAATCAAGCAGCAGATGGTGATCCAAGATATGCATTTGGTAGACAACATGGTC
AAAAAACTACCACAACAGGAGAAACACCTGAGAGATTTACATATATAGCACATCAAGATACAGGAAGA
TATCCAGAAGGAGATTGGATTCAAAATATTAACTTTAACCTTCCTGTAACAGAAGATAATGTATTGCT
ACCAACAGATCCAATTGGAGGTAAAACAGGAATTAACTATACTAATATATTTAATACTTATGGTCCTT
TAACTGCATTAAATAATGTACCACCAGTTTATCCAAATGGTCAAATTTGGGATAAAGAATTTGATACT
GACTTAAAACCAAGACTTCATGTAAATGCACCATTTGTTTGTCAAAATAATTGTCCTGGTCAATTATT
TGTAAAAGTTGCGCCTAATTTAACAAATGAATATGATCCTGATGCATCTGCTAATATGTCAAGAATTG
TAACTTACTCAGATTTTTGGTGGAAAGGTAAATTAGTATTTAAAGCTAAACTAAGAGCCTCTCATACT
TGGAATCCAATTCAACAAATGAGTATTAATGTAGATAACCAATTTAACTATGTACCAAGTAATATTGG
AGGTATGAAAATTGTATATGAAAAATCTCAACTAGCACCTAGA
'URU_KM457106_2c_UY55_2006'
ATGAGTGATGGAGCAGTTCAACCAGACGGTGGTCAACCTGCTGTCAGAAATGAAAGAGCAACAGGATC
TGGGAACGGGTCTGGAGGCGGGGGTGGTGGTGGTTCTGGGGGTGTGGGGATTTCTACGGGTACTTTCA
ATAATCAGACGGAATTTAAATTTTTGGAAAACGGATGGGTGGAAATCACAGCAAACTCAAGCAGACTT
GTACATTTAAATATGCCAGAAAGTGAAAATTATAGAAGAGTGGTTGTAAATAATTTGGATAAAACTGC
AGTTAACGGAAACATGGCTTTAGATGATACTCATGCACAAATTGTAACACCTTGGTCATTGGTTGATA
CAAATGCTTGGGGAGTTTGGTTTAATCCAGGAGATTGGCAACTAATTGTTAATACTATGAGTGAGTTG
CATTTAGTTAGTTTTGAACAAGAAATTTTTAATGTTGTTTTAAAGACTGTTTCAGAATCTGCTACTCA
GCCACCAACTAAAGTTTATAATAATGATTTAACTGCATCATTGATGGTTGCATTAGATAGTAATAATA
CTATGCCATTTACTCCAGCAGCTATGAGATCTGAGACATTGGGTTTTTATCCATGGAAACCAACCATA
CCAACTCCATGGAGATATTATTTTCAATGGGATAGAACATTAATACCATCTCATACTGGAACTAGTGG
CACACCAACAAATATATACCATGGTACAGATCCAGATGATGTTCAATTTTATACTATTGAAAATTCTG
TGCCAGTACACTTACTAAGAACAGGTGATGAATTTGCTACAGGAACATTTTTTTTTGATTGTAAACCA
TGTAGACTAACACATACATGGCAAACAAATAGAGCATTGGGCTTACCACCATTTCTAAATTCTTTGCC
TCAAGCTGAAGGAGGTACTAACTTTGGTTATATAGGAGTTCAACAAGATAAAAGACGTGGTGTAACTC
AAATGGGAAATACAAACTATATTACTGAAGCTACTATTATGAGACCAGCTGAGGTTGGTTATAGTGCA
CCATATTATTCTTTTGAGGCGTCTACACAAGGGCCATTTAAAACACCTATTGCAGCAGGACGGGGGGG
AGCGCAAACAGATGAAAATCAAGCAGCAGATGGTGATCCAAGATATGCATTTGGTAGACAACATGGTC
AAAAAACTACCACAACAGGAGAAACACCTGAGAGATTTACATATATAGCACATCAAGATACAGGAAGA
TATCCAGAAGGAGATTGGATTCAAAATATTAACTTTAACCTTCCTGTAACAGAAGATAATGTATTGCT
ACCAACAGATCCAATTGGAGGTAAAACAGGAATTAACTATACTAATATATTTAATACTTATGGTCCTT
TAACTGCATTAAATAATGTACCACCAGTTTATCCAAATGGTCAAATTTGGGATAAAGAATTTGATACT
GACTTAAAACCAAGACTTCATGTAAATGCACCATTTGTTTGTCAAAATAATTGTCCTGGTCAATTATT
TGTAAAAGTTGCGCCTAATTTAACAAATGAATATGATCCTGATGCATCTGCTAATATGTCAAGAATTG
TAACTTACTCAGATTTTTGGTGGAAAGGTAAATTAGTATTTAAAGCTAAACTAAGAGCCTCTCATACT

TGGAATCCAATTCAACAAATGAGTATTAATGTAGATAACCAATTTAACTATGTACCAAGTAATATTGG
AGGTATGAAAATTGTGTATGAAAAATCTCAACTAGCACCTAGA

'URU_KM457107_2c_UY72_2007'

ATGAGTGATGGAGCAGTTCAACCAGACGGTGGTCAACCTGCTGTCAGAAATGAAAGAGCAACAGGATC
TGGGAACGGGTCTGGAGGCGGGGGTGGTGGTGGTTCTGGGGGTGTGGGGATTTCTACGGGTACTTTCA
ATAATCAGACGGAATTTAAATTTTTGGAAAACGGATGGGTGGAAATCACAGCAAACTCAAGCAGACTT
GTACATTTAAATATGCCAGAAAGTGAAAATTATAGAAGAGTGGTTGTAAATAATTTGGATAAAACTGC
AGTTAACGGAAACATGGCTTTAGATGATACTCATGCACAAATTGTAACACCTTGGTCATTGGTTGATG
CAAATGCTTGGGGAGTTTGGTTTAATCCAGGAGATTGGCAACTAATTGTTAATACTATGAGTGAGTTG
CATTTAGTTAGTTTTGAACAAGAAATTTTTAATGTTGTTTTAAAGACTGTTTCAGAATCTGCTACTCA
GCCACCAACTAAAGTTTATAATAATGATTTAACTGCATCATTGATGGTTGCATTAGATAGTAATAATA
CTATGCCATTTACTCCAGCAGCTATGAGATCTGAGACATTGGGTTTTTATCCATGGAAACCAACCATA
CCAACTCCATGGAGATATTATTTTCAATGGGATAGAACATTAATACCATCTCATACTGGAACTAGTGG
CACACCAACAAATATATACCATGGTACAGATCCAGATGATGTTCAATTTTATACTATTGAAAATTCTG
TGCCAGTACACTTACTAAGAACAGGTGATGAATTTGCTACAGGAACATTTTTTTTTGATTGTAAACCA
TGTAGACTAACACATACATGGCAAACAAATAGAGCATTGGGCTTACCACCATTTCTAAATTCTTTGCC
TCAAGCTGAAGGAGGTACTAACTTTGGTTATATAGGAGTTCAACAAGATAAAAGACGTGGTGTAACTC
AAATGGGAAATACAAACTATATTACTGAAGCTACTATTATGAGACCAGCTGAGGTTGGTTATAGTGCA
CCATATTATTCTTTTGAGGCGTCTACACAAGGGCCATTTAAAACACCTATTGCAGCAGGACGGGGGGG
AGCGCAAACAGATGAAAATCAAGCAGCAGATGGTGATCCAAGATATGCATTTGGTAGACAACATGGTC
AAAAAACTACCACAACAGGAGAAACACCTGAGAGATTTACATATATAGCACATCAAGATACAGGAAGA
TATCCAGAAGGAGATTGGATTCAAAATATTAACTTTAACCTTCCTGTAACAGAAGATAATGTATTGCT
ACCAACAGATCCAATTGGAGGTAAAACAGGAATTAACTATACTAATATATTTAATACTTATGGTCCTT
TAACTGCATTAAATAATGTACCACCAGTTTATCCAAATGGTCAAATTTGGGATAAAGAATTTGATACT
GACTTAAAACCAAGACTTCATGTAAATGCACCATTTGTTTGTCAAAATAATTGTCCTGGTCAATTATT
TGTAAAAGTTGCGCCTAATTTAACAAATGAATATGATCCTGATGCATCTGCTAATATGTCAAGAATTG
TAACTTACTCAGATTTTTGGTGGAAAGGTAAATTAGTATTTAAAGCTAAACTAAGAGCCTCTCATACT
TGGAATCCAATTCAACAAATGAGTATTAATGTAGATAACCAATTTAACTATGTACCAAGTAATATTGG
AGGTATGAAAATTGTATATGAAAAATCTCAACTAGCACCTAGA

'URU_KM457108_2c_UY82_2007'

ATGAGTGATGGAGCAGTTCAACCAGACGGTGGTCAACCTGCTGTCAGAAATGAAAGAGCAACAGGATC
TGGGAACGGGTCTGGAGGCGGGGGTGGTGGTGGTTCTGGGGGTGTGGGGATTTCTACGGGTACTTTCA
ATAATCAAACGGAATTTAAATTTTTGGAAAACGGATGGGTGGAAATCACAGCAAACTCAAGCAGACTT
GTACATTTAAATATGCCAGAAAGTGAAAATTATAGAAGAGTGGTTGTAAATAATTTGGATAAAACTGC
AGTTAACGGAAACATGGCTTTAGATGATACTCATGCACAAATTGTAACACCTTGGTCATTGGTTGATG
CAAATGCTTGGGGAGTTTGGTTTAATCCAGGAGATTGGCAACTAATTGTTAATACTATGAGTGAGTTG
CATTTAGTTAGTTTTGAACAAGAAATTTTTAATGTTGTTTTAAAGACTGTTTCAGAATCTGCTACTCA
GCCACCAACTAAAGTTTATAATAATGATTTAACTGCATCATTGATGGTTGCATTAGATAGTAATAATA
CTATGCCATTTACTCCAGCAGCTATGAGATCTGAGACATTGGGTTTTTATCCATGGAAACCAACCATA
CCAACTCCATGGAGATATTATTTTCAATGGGATAGAACATTAATACCATCTCATACTGGAACTAGTGG
CACACCAACAAATATATACCATGGTACAGATCCAGATGATGTTCAATTTTATACTATTGAAAATTCTG
TGCCAGTACACTTACTAAGAACAGGTGATGAATTTGCTACAGGAACATTTTTTTTTGATTGTAAACCA
TGTAGACTAACACATACATGGCAAACAAATAGAGCATTGGGCTTACCACCATTTCTAAATTCTTTGCC
TCAAGCTGAAGGAGGTACTAACTTTGGTTATATAGGAGTTCAACAAGATAAAAGACGTGGTGTAACTC
AAATGGGAAATACAAACTATATTACTGAAGCTACTATTATGAGACCAGCTGAGGTTGGTTATAGTGCA
CCATATTATTCTTTTGAGGCGTCTACACAAGGGCCATTTAAAACACCTATTGCAGCAGGACGGGGGGG
AGCGCAAACAGATGAAAATCAAGCAGCAGATGGTGATCCAAGATATGCATTTGGTAGACAACATGGTC
AAAAAACTACCACAACAGGAGAAACACCTGAGAGATTTACATATATAGCACATCAAGATACAGGAAGA
TATCCAGAAGGAGATTGGATTCAAAATATTAACTTTAACCTTCCTGTAACAGAAGATAATGTATTGCT
ACCAACAGATCCAATTGGAGGTAAAACAGGAATTAACTATACTAATATATTTAATACTTATGGTCCTT
TAACTGCATTAAATAATGTACCACCAGTTTATCCAAATGGTCAAATTTGGGATAAAGAATTTGATACT
GACTTAAAACCAAGACTTCATGTAAATGCACCATTTGTTTGTCAAAATAATTGTCCTGGTCAATTATT
TGTAAAAGTTGCGCCTAATTTAACAAATGAATATGATCCTGATGCATCTGCTAATATGTCAAGAATTG
TAACTTACTCAGATTTTTGGTGGAAAGGTAAATTAGTATTTAAAGCTAAACTAAGAGCCTCTCATACT

TGGAATCCAATTCAACAAATGAGCATTAATGTAGATAACCAATTTAACTATGTACCAAGTAATATTGG
AGGTATGAAAATTGTATATGAAAAATCTCAACTAGCACCTAGA
'URU_KM457109_2c_UY95_2007'
ATGAGTGATGGAGCAGTTCAACCAGACGGTGGTCAATCTGCTGTCAGAAATGAAAGAGCAACAGGATC
TGGGAACGGGTCTGGAGGCGGGGGTGGTGGTGGTTCTGGGGGTGTGGGGATTTCTACGGGTACTTTCA
ATAATCAGACGGAATTTAAATTTTTGGAAAACGGATGGGTGGAAATCACAGCAAACTCAAGCAGACTT
GTACATTTAAATATGCCAGAAAGTGAAAATTATAGAAGAGTGGTTGTAAATAATTTGGATAAAACTGC
AGTTAACGGAAACATGGCTTTAGATGATACTCATGCACAAATTGTAACACCTTGGTCATTGGTTGATG
CAAATGCTTGGGGAGTTTGGTTTAATCCAGGAGATTGGCAACTAATTGTTAATACTATGAGTGAGTTG
CATTTAGTTAGTTTTGAACAAGAAATTTTTAATGTTGTTTTAAAGACTGTTTCAGAATCTGCTACTCA
GCCACCAACTAAAGTTTATAATAATGATTTAACTGCATCATTGATGGTTGCATTAGATAGTAATAATA
CTATGCCATTTACTCCAGCAGCTATGAGATCTGAGACATTGGGTTTTTATCCATGGAAACCAACCATA
CCAACTCCATGGAGATATTATTTTCAATGGGATGGAACATTAATACCATCTCATACTGGAACTAGTGG
CACACCAACAAATATATACCATGGTACAGATCCAGATGATGTTCAATTTTATACTATTGAAAATTCTG
TGCCAGTACACTTACTAAGAACAGGTGATGAATTTGCTACAGGAACATTTTTTTTTGATTGTAAACCA
TGTAGACTAACACATACATGGCAAACAAATAGAGCATTGGGCTTACCACCATTTCTAAATTCTTTGCC
TCAAGCTGAAGGAGGTACTAACTTTGGTTATATAGGAGTTCAACAAGATAAAAGACGTGGTGTAACTC
AAATGGGAAATACAAACTATATTACTGAAGCTACTATTATGAGACCAGCTGAGGTTGGTTATAGTGCA
CCATATTATTCTTTTGAGGCGTCTACACAAGGGCCATTTAAAACACCTATTGCAGCAGGACGGGGGGG
AGCGCAAACAGATGAAAATCAAGCAGCAGATGGTGATCCAAGATATGCATTTGGTAGACAACATGGTC
AAAAAACTACCACAACAGGAGAAACACCTGAGAGATTTACATATATAGCACATCAAGATACAGGAAGA
TATCCAGAAGGAGATTGGATTCAAAATATTAACTTTAACCTTCCTGTAACAGAAGATAATGTATTGCT
ACCAACAGATCCAATTGGAGGTAAAACAGGAATTAACTATACTAATATATTTAATACTTATGGTCCTT
TAACTGCATTAAATAATGTACCACCAGTTTATCCAAATGGTCAAATTTGGGATAAAGAATTTGATACT
GACTTAAAACCAAGACTTCATGTAAATGCACCATTTGTTTGTCAAAATAATTGTCCTGGTCAATTATT
TGTAAAAGTTGCGCCTAATTTAACAAATGAATATGATCCTGATGCATCTGCTAATATGTCAAGAATTG
TAACTTACTCAGATTTTTGGTGGAAAGGTAAATTAGTATTTAAAGCTAAACTAAGAGCCTCTCATACT
TGGAATCCAATTCAACAAATGAGTATTAATGTAGATAACCAATTTAACTATGTACCAAGTAATATTGG
AGGTATGAAAATTGTATATGAAAAATCTCAACTAGCACCTAGA
'URU_KM457111_2c_UY120_2008'
ATGAGTGATGGAGCAGTTCAACCAGACGGTGGTCAACCTGCTGTCAGAAATGAAAGAGCAACAGGATC
TGGGAACGGGTCTGGAGGCGGGGGTGGTGGTGGTTCTGGGGGTGTGGGGATTTCTACGGGTACTTTCA
ATAATCAGACGGAATTTAAATTTTTGGAAAACGGATGGGTGGAAATCACAGCAAACTCAAGCAGACTT
GTACATTTAAATATGCCAGAAAGTGAAAATTATAGAAGAGTGGTTGTAAATAATTTGGATAAAACTGC
AGTTAACGGAAACATGGCTTTAGATGATACTCATGCACAAATTGTAACACCTTGGTCATTGGTTGATG
CAAATGCTTGGGGAGTTTGGTTTAATCCAGGAGATTGGCAACTAATTGTTAATACTATGAGTGAGTTG
CATTTAGTTAGTTTTGAACAAGAAATTTTTAATGTTGTTTTAAAGACTGTTTCAGAATCTGCTACTCA
GCCACCAACTAAAGTTTATAATAATGATTTAACTGCATCATTGATGGTTGCATTAGATAGTAATAATA
CTATGCCATTTACTCCAGCAGCTATGAGATCTGAGACATTGGGTTTTTATCCATGGAAACCAACCATA
CCAACTCCATGGAGATATTATTTTCAATGGGATAGAACATTAATACCATCTCATACTGGAACTAGTGG
CACACCAACAAATATATACCATGGTACAGATCCAGATGATGTTCAATTTTATACTATTGAAAATTCTG
TGCCAGTACACTTACTAAGAACAGGTGATGAATTTGCTACAGGAACATTTTTTTTTGATTGTAAACCA
TGTAGACTAACACATACATGGCAAACAAATAGAGCATTGGGCTTACCACCATTTCTAAATTCTTTGCC
TCAAGCTGAAGGAGGTACTAACTTTGGTTATATAGGAGTTCAACAAGATAAAAGACGTGGTGTAACTC
AAATGGGAAATACAAACTATATTACTGAAGCTACTATTATGAGACCAGCTGAGGTTGGTTATAGTGCA
CCATATTATTCTTTTGAGGCGTCTACACAAGGGCCATTTAAAACACCTATTGCAGCAGGACGGGGGGG
AGCGCAAACAGATGAAAATCAAGCAGCAGATGGTGATCCAAGATATGCATTTGGTAGACAACATGGTC
AAAAAACTACCACAACAGGAGAAACACCTGAGAGATTTACATATATAGCACATCAAGATACAGGAAGA
TATCCAGAAGGAGATTGGATTCAAAATATTAACTTTAACCTTCCTGTAACAGAAGATAATGTATTGCT
ACCAACAGATCCAATTGGAGGTAAAACAGGAATTAACTATACTAATATATTTAATACTTATGGTCCTT
TAACTGCATTAAATAATGTACCACCAGTTTATCCAAATGGTCAAATTTGGGATAAAGAATTTGATACT
GACTTAAAACCAAGACTTCATGTAAATGCACCATTTGTTTGTCAAAATAATTGTCCTGGTCAATTATT
TGTAAAAGTTGCGCCTAATTTAACAAATGAATATGATCCTGATGCATCTGCTAATATGTCAAGAATTG
TAACTTACTCAGATTTTTGGTGGAAAGGTAAATTAGTATTTAAAGCTAAACTAAGAGCCTCTCATACT

TGGAATCCAATTCAACAAATGAGTATTAATGTAGATAACCAATTTAACTATGTACCAAGTAATATTGG
AGGTATGAAAATTGTATATGAAAAATCTCAACTAGCACCTAGA
'URU_KM457112_2c_UY135_2008'
ATGAGTGATGGAGCAGTTCAACCAGACGGTGGTCAACCTGCTGTCAGAAATGAAAGAGCAACAGGATC
TGGGAACGGGTCTGGAGGCGGGGGTGGTGGTGGTTCTGGGGGTGTGGGGATTTCTACGGGTACTTTCA
ATAATCAGACGGAATTTAAATTTTTGGAAAACGGATGGGTGGAAATCACAGCAAACTCAAGCAGACTT
GTACATTTAAATATGCCAGAAAGTGAAAATTATAGAAGAGTGGTTGTAAATAATTTGGATAAAACTGC
AGTTAACGGAAACATGGCTTTAGATGATACTCATGCACAAATTGTAACACCTTGGTCATTGGTTGATG
CAAATGCTTGGGGAGTTTGGTTTAATCCAGGAGATTGGCAACTAATTGTTAATACTATGAGTGAGTTG
CATTTAGTTAGTTTTGAACAAGAAATTTTTAATGTTGTTTTAAAGACTGTTTCAGAATCTGCTACTCA
GCCACCAACTAAAGTTTATAATAATGATTTAACTGCATCATTGATGGTTGCATTAGATAGTAATAATA
CTATGCCATTTACTCCAGCAGCTATGAGATCTGAGACATTGGGTTTTTATCCATGGAAACCAACCATA
CCAACTCCATGGAGATATTATTTTCAATGGGATAGAACATTAATACCATCTCATACTGGAACTAGTGG
CACACCAACAAATATATACCATGGTACAGATCCAGATGATGTTCAATTTTATACTATTGAAAATTCTG
TGCCAGTACACTTACTAAGAACAGGTGATGAATTTGCTACAGGAACATTTTTTTTTGATTGTAAACCA
TGTAGACTAACACATACATGGCAAACAAATAGAGCATTGGGCTTACCACCATTTCTAAATTCTTTGCC
TCAAGCTGAAGGAGGTACTAACTTTGGTTATATAGGAGTTCAACAAGATAAAAGACGTGGTGTAACTC
AAATGGGAAATACAAACTATATTACTGAAGCTACTATTATGAGACCAGCTGAGGTTGGTTATAGTGCA
CCATATTATTCTTTTGAGGCGTCTACACAAGGGCCATTTAAAACACCTATTGCAGCAGGACGGGGGGG
AGCGCAAACAGATGAAAATCAAGCAGCAGATGGTGATCCAAGATATGCATTTGGTAGACAACATGGTC
AAAAAACTACCACAACAGGAGAAACACCTGAGAGATTTACATATATAGCACATCAAGATACAGGAAGA
TATCCAGAAGGAGATTGGATTCAAAATATTAACTTTAACCTTCCTGTAACAGAAGATAATGTATTGCT
ACCAACAGATCCAATTGGAGGTAAAACAGGAATTAACTATACTAATATATTTAATACTTATGGTCCTT
TAACTGCATTAAATAATGTACCACCAGTTTATCCAAATGGTCAAATTTGGGATAAAGAATTTGATACT
GACTTAAAACCAAGACTTCATGTAAATGCACCATTTGTTTGTCAAAATAATTGTCCTGGTCAATTATT
TGTAAAAGTTGCGCCTAATTTAACAAATGAATATGATCCTGATGCATCTGCTAATATGTCAAGAATTG
TAACTTACTCAGATTTTTGGTGGAAAGGTAAATTAGTATTTAAAGCTAAACTAAGAGCCTCTCATACT
TGGAATCCAATTCAACAAATGAGTATTAATGTAGATAACCAATTTAACTATGTACCAAGTAATATTGG
AGGTATGAAAATTGTATATGAAAAATCTCAACTAGCACCTAGA
'URU_KM457113_2c_UY152_2009'
ATGAGTGATGGAGCAGTTCAACCAGACGGTGGTCAACCTGCTGTCAGAAATGAAAGAGCAACAGGATC
TGGGAACGGGTCTGGAGGCGGGGGTGGTGGTGGTTCTGGGGGTGTGGGGATTTCTACGGGTACTTTCA
ATAATCAGACGGAATTTAAATTTTTGGAAAACGGATGGGTGGAAATCACAGCAAACTCAAGCAGACTT
GTACATTTAAATATGCCAGAAAGTGAAAATTATAGAAGAGTGGTTGTAAATAATTTGGATAAAACTGC
AGTTAACGGAAACATGGCTTTAGATGATACTCATGCACAAATTGTAACACCTTGGTCATTGGTTGATG
CAAATGCTTGGGGAGTTTGGTTTAATCCAGGAGATTGGCAACTAATTGTTAATACTATGAGTGAGTTG
CATTTAGTTAGTTTTGAACAAGAAATTTTTAATGTTGTTTTAAAGACTGTTTCAGAATCTGCTACTCA
GCCACCAACTAAAGTTTATAATAATGATTTAACTGCATCATTGATGGTTGCATTAGATAGTAATAATA
CTATGCCATTTACTCCAGCAGCTATGAGATCTGAGACATTGGGTTTTTATCCATGGAAACCAACCATA
CCAACTCCATGGAGATATTATTTTCAATGGGATAGAACATTAATACCATCTCATACTGGAACTAGTGG
CACACCAACAAATATATACCATGGTACAGATCCAGATGATGTTCAATTTTATACTATTGAAAATTCTG
TGCCAGTACACTTACTAAGAACAGGTGATGAATTTGCTACAGGAACATTTTTTTTTGATTGTAAACCA
TGTAGACTAACACATACATGGCAAACAAATAGAGCATTGGGCTTACCACCATTTCTAAATTCTTTGCC
TCAAGCTGAAGGAGGTACTAACTTTGGCTATATAGGAGTTCAACAAGATAAAAGACGTGGTGTAACTC
AAATGGGAAATACAAACTATATTACTGAAGCTACTATTATGAGACCAGCTGAGGTTGGTTATAGTGCA
CCATATTATTCTTTTGAGGCGTCTACACAAGGGCCATTTAAAACACCTATTGCAGCAGGACGGGGGGG
AGCGCAAACAGATGAAAATCAAGCAGCAGATGGTGATCCAAGATATGCATTTGGTAGACAACATGGTC
AAAAAACTACCACAACAGGAGAAACACCTGAGAGATTTACATATATAGCACATCAAGATACAGGAAGA
TATCCAGAAGGAGATTGGATTCAAAATATTAACTTTAACCTTCCTGTAACAGAAGATAATGTATTGCT
ACCAACAGATCCAATTGGAGGTAAAACAGGAATTAACTATACTAATATGTTTAATACTTATGGTCCTT
TAACTGCATTAAATAATGTACCACCAGTTTATCCAAATGGTCAAATTTGGGATAAAGAATTTGATACT
GACTTAAAACCAAGACTTCATGTAAATGCACCATTTGTTTGTCAAAATAATTGTCCTGGTCAATTATT
TGTAAAAGTTGCGCCTAATTTAACAAATGAATATGATCCTGATGCATCTGCTAATATGTCAAGAATTG
TAACTTACTCAGATTTTTGGTGGAAAGGTAAATTAGTATTTAAAGCTAAACTAAGAGCCTCTCATACT

TGGAATCCAATTCAACAAATGAGTATTAATGTAGATAACCAATTTAACTATGTACCAAGTAATATTGG
AGGTATGAAAATTGTATATGAAAAATCTCAACTAGCACCTAGA
'URU_KM457116_2c_UY185_2009'
ATGAGTGATGGAGCAGTTCAACCAGACGGTGGTCAACCTGCTGTCAGAAATGAAAGAGCAACAGGATC
TGGGAACGGGTCTGGAGGCGGGGGTGGTGGTGGTTCTGGGGGTGTGGGGATTTCTACGGGTACTTTCA
ATAATCAGACGGAATTTAAATTTTTGGAAAACGGATGGGTGGAAATCACAGCAAACTCAAGCAGACTT
GTACATTTAAATATGCCAGAAAGTGAAAATTATAGAAGAGTGGTTGTAAATAATTTGGATAAAACTGC
AGTTAACGGAAACATGGCTTTAGATGATACTCATGCACAAATTGTAACACCTTGGTCATTGGTTGATG
CAAATGCTTGGGGAGTTTGGTTTAATCCAGGAGATTGGCAACTAATTGTTAATACTATGAGTGAGTTG
CATTTAGTTAGTTTTGAACAAGAAATTTTTAATGTTGTTTTAAAGACTGTTTCAGAATCTGCTACTCA
GCCACCAACTAAAGTTTATAATAATGATTTAACTGCATCATTGATGGTTGCATTAGATAGTAATAATA
CTATGCCATTTACTCCAGCAGCTATGAGATCTGAGACATTGGGTTTTTATCCATGGAAACCAACCATA
CCAACTCCATGGAGATATTATTTTCAATGGGATAGAACATTAATACCATCTCATACTGGAACTAGTGG
CACACCAACAAATATATACCATGGTACAGATCCAGATGATGTTCAATTTTATACTATTGAAAATTCTG
TGCCAGTACACTTACTAAGAACAGGTGATGAATTTGCTACAGGAACATTTTTTTTTGATTGTAAACCA
TGTAGACTAACACATACATGGCAAACAAATAGAGCATTGGGCTTACCACCATTTCTAAATTCTTTGCC
TCAAGCTGAAGGAGGTACTAACTTTGGTTATATAGGAGTTCAACAAGATAAAAGACGTGGTGTAACTC
AAATGGGAAATACAAACTATATTACTGAAGCTACTATTATGAGACCAGCTGAGGTTGGTTATAGTGCA
CCATATTATTCTTTTGAGGCGTCTACACAAGGGCCATTTAAAACACCTATTGCAGCAGGACGGGGGGG
AGCGCAAACAGATGAAAATCAAGCAGCAGATGGTGATCCACGATATGCATTTGGTAGACAACATGGTC
AAAAAACTACCACAACAGGAGAAACACCTGAGAGATTTACATATATAGCACATCAAGATACAGGAAGA
TATCCAGAAGGAGATTGGATTCAAAATATTAACTTTAACCTTCCTGTAACAGAAGATAATGTATTGCT
ACCAACAGATCCAATTGGAGGTAAAACAGGAATTAACTATACTAATATATTTAATACTTATGGTCCTT
TAACTGCATTAAATAATGTACCACCAGTTTATCCAAATGGTCAAATTTGGGATAAAGAATTTGATACT
GACTTAAAACCAAGACTTCATGTAAATGCACCATTTGTTTGTCAAAATAATTGTCCTGGTCAATTATT
TGTAAAAGTTGCGCCTAATTTAACAAATGAATATGATCCTGATGCATCTGCTAATATGTCAAGAATTG
TAACTTACTCAGATTTTTGGTGGAAAGGTAAATTAGTATTTAAAGCTAAACTAAGAGCCTCTCATACT
TGGAATCCAATTCAACAAATGAGTATTAATGTAGATAACCAATTTAACTATGTACCAAGTAATATTGG
AGGTATGAAAATTGTATATGAAAAATCTCAACTAGCACCTAGA
'URU_KM457117_2c_UY187_2009'
ATGAGTGATGGAGCAGTTCAACCAGACGGTGGTCAACCTGCTGTCAGAAATGAAAGAGCAACAGGATC
TGGGAACGGGTCTGGAGGCGGGGGTGGTGGTGGTTCTGGGGGTGTGGGGATTTCTACGGGTACTTTCA
ATAATCAGACGGAATTTAAATTTTTGGAAAACGGATGGGTGGAAATCACAGCAAACTCAAGCAGACTT
GTACATTTAAATATGCCAGAAAGTGAAAATTATAGAAGAGTGGTTGTAAATAATTTGGATAAAACTGC
AGTTAACGGAAACATGGCTTTAGATGATACTCATGCACAAATTGTAACACCTTGGTCATTGGTTGATG
CAAATGCTTGGGGAGTTTGGTTTAATCCAGGAGATTGGCAACTAATTGTTAATACTATGAGTGAGTTG
CATTTAGTTAGTTTTGAACAAGAAATTTTTAATGTTGTTTTAAAGACTGTTTCAGAATCTGCTACTCA
GCCACCAACTAAAGTTTATAATAATGATTTAACTGCATCATTGATGGTTGCATTAGATAGTAATAATA
CTATGCCATTTACTCCAGCAGCTATGAGATCTGAGACATTGGGTTTTTATCCATGGAAACCAACCATA
CCAACTCCATGGAGATATTATTTTCAATGGGATAGAACATTAATACCATCTCATACTGGAACTAGTGG
CACACCAACAAATATATACCATGGTACAGATCCAGATGATGTTCAATTTTATACTATTGAAAATTCTG
TGCCAGTACACTTACTAAGAACAGGTGATGAATTTGCTACAGGAACATTTTTTTTTGATTGTAAACCA
TGTAGACTAACACATACATGGCAAACAAATAGAGCATTGGGCTTACCACCATTTCTAAATTCTTTGCC
TCAAGCTGAAGGAGGTACTAACTTTGGTTATATAGGAGTTCAACAAGATAAAAGACGTGGTGTAACTC
AAATGGGAAATACAAACTATATTACTGAAGCTACTATTATGAGACCAGCTGAGGTTGGTTATAGTGCA
CCATATTATTCTTTTGAGGCGTCTACACAAGGGCCATTTAAAACACCTATTGCAGCAGGACGGGGGGG
AGCGCAAACAGATGAAAATCAAGCAGCAGATGGTGATCCAAGATATGCATTTGGTAGACAACATGGTC
AAAAAACTACCACAACAGGAGAAACACCTGAGAGATTTACATATATAGCACATCAAGATACAGGAAGA
TATCCAGAAGGCGATTGGATTCAAAATATTAACTTTAACCTTCCTGTAACAGAAGATAATGTATTGCT
ACCAACAGATCCAATTGGAGGTAAAACAGGAATTAACTATACTAATATATTTAATACTTATGGTCCTT
TAACTGCATTAAATAATGTACCACCAGTTTATCCAAATGGTCAAATTTGGGATAAAGAATTTGATACT
GACTTAAAACCAAGACTTCATGTAAATGCACCATTTGTTTGTCAAAATAATTGTCCTGGTCAATTATT
TGTAAAAGTTGCGCCTAATTTAACAAATGAATATGATCCTGATGCATCTGCTAATATGTCAAGAATTG
TAACTTACTCAGATTTTTGGTGGAAAGGTAAATTAGTATTTAAAGCTAAACTAAGAGCCTCTCATACT

TGGAATCCAATTCAACAAATGAGTATTAATGTAGATAACCAATTTAACTATGTACCAAGTAATATTGG
AGGTATGAAAATTGTATATGAAAAATCTCAACTAGCACCTAGA
'URU_KM457120_2c_UY242_2010'
ATGAGTGATGGAGCAGTTCAACCAGACGGTGGTCAATCTGCTGTCAGAAATGAAAGAGCAACAGGATC
TGGGAACGGGTCTGGAGGCGGGGGTGGTGGTGGTTCTGGGGGTGTGGGGATTTCTACGGGTACTTTCA
ATAATCAGACGGAATTTAAATTTTTGGAAAACGGATGGGTGGAAATCACAGCAAACTCAAGCAGACTT
GTACATTTAAATATGCCAGAAAGTGAAAATTATAGAAGAGTGGTTGTAAATAATTTGGATAAAACTGC
AGTTAACGGAAACATGGCTTTAGATGATACTCATGCACAAATTGTAACACCTTGGTCATTGGTTGATG
CAAATGCTTGGGGAGTTTGGTTTAATCCAGGAGATTGGCAACTAATTGTTAATACTATGAGTGAGTTG
CATTTAGTTAGTTTTGAACAAGAAATTTTTAATGTTGTTTTAAAGACTGTTTCAGAATCTGCTACTCA
GCCACCAACTAAAGTTTATAATAATGATTTAACTGCATCATTGATGGTTGCATTAGATAGTAATAATA
CTATGCCATTTACTCCAGCAGCTATGAGATCTGAGACATTGGGTTTTTATCCATGGAAACCAACCATA
CCAACTCCATGGAGATATTATTTTCAATGGGATAGAACATTAATACCATCTCATACTGGAACTAGTGG
CACACCAACAAATATATACCATGGTACAGATCCAGATGATGTTCAATTTTATACTATTGAAAATTCTG
TGCCAGTACACTTACTAAGAACAGGTGATGAATTTGCTACAGGAACATTTTTTTTTGATTGTAAACCA
TGTAGACTAACACATACATGGCAAACAAATAGAGCATTGGGCTTACCACCATTTCTAAATTCTTTGCC
TCAAGCTGAAGGAGGTACTAACTTTGGTTATATAGGAGTTCAACAAGATAAAAGACGTGGTGTAACTC
AAATGGGAAATACAAACTATATTACTGAAGCTACTATTATGAGACCAGCTGAGGTTGGTTATAGTGCA
CCATATTATTCTTTTGAGGCGTCTACACAAGGGCCATTTAAAACACCTATTGCAGCAGGACGGGGGGG
AGCGCAAACAGATGAAAATCAAGCAGCAGATGGTGATCCAAGATATGCATTTGGTAGACAACATGGTC
AAAAAACTACCACAACAGGAGAAACACCTGAGAGATTTACATATATAGCACATCAAGATACAGGAAGA
TATCCAGAAGGAGATTGGATTCAAAATATTAACTTTAACCTTCCTGTAACAGAAGATAATGTATTACT
ACCAACAGATCCAATTGGAGGTAAAACAGGAATTAACTATACTAATATATTTAATACTTATGGTCCTT
TAACTGCATTAAATAATGTACCACCAGTTTATCCAAATGGTCAAATTTGGGATAAAGAATTTGATACT
GACTTAAAACCAAGACTTCATGTAAATGCACCATTTGTTTGTCAAAATAATTGTCCTGGTCAATTATT
TGTAAAAGTTGCGCCTAATTTAACAAATGAATATGATCCTGATGCATCTGCTAATATGTCAAGAATTG
TAACTTACTCAGATTTTTGGTGGAAAGGTAAATTAGTATTTAAAGCTAAACTAAGAGCCTCTCATACT
TGGAATCCAATTCAACAAATGAGTATTAATGTAGATAACCAATTTAACTATGTACCAAGTAATATTGG
AGGTATGAAAATTGTATATGAAAAATCTCAACTAGCACCTAGA
'URU_KM457121_2c_UY247_2010'
ATGAGTGATGGAGCAGTTCAACCAGACGGTGGTCAACCTGCTGTCAGAAATGAAAGAGCAACAGGATC
TGGGAACGGGTCTGGAGGCGGGGGTGGTGGTGGTTCTGGGGGTGTGGGGATTTCTACGGGTACTTTCA
ATAATCAGACGGAATTTAAATTTTTGGAAAACGGATGGGTGGAAATCACAGCAAACTCAAGCAGACTT
GTACATTTAAATATGCCAGAAAGTGAAAATTATAGAAGAGTGGTTGTAAATAATTTGGATAAAACTGC
AGTTAACGGAAACATGGCTTTAGATGATACTCATGCACAAATTGTAACACCTTGGTCATTGGTTGATG
CAAATGCTTGGGGAGTTTGGTTTAATCCAGGAGATTGGCAACTAATTGTTAATACTATGAGTGAGTTG
CATTTAGTTAGTTTTGAACAAGAAATTTTTAATGTTGTTTTAAAGACTGTTTCAGAATCTGCTACTCA
GCCACCAACTAAAGTTTATAATAATGATTTAACTGCATCATTGATGGTTGCATTAGATAGTAATAATA
CTATGCCATTTACTCCAGCAGCTATGAGATCTGAGACATTGGGTTTTTATCCATGGAAACCAACCATA
CCAACTCCATGGAGATATTATTTTCAATGGGATAGAACATTAATACCATCTCATACTGGAACTAGTGG
CACACCAACAAATATATACCATGGTACAGATCCAGATGATGTTCAATTTTATACTATTGAAAATTCTG
TGCCAGTACACTTACTAAGAACAGGTGATGAATTTGCTACAGGAACATTTTTTTTTGATTGTAAACCA
TGTAGACTAACACATACATGGCAAACAAATAGAGCATTGGGCTTACCACCATTTCTAAATTCTTTGCC
TCAAGCTGAAGGAGGTACTAACTTTGGTTATATAGGAGTTCAACAAGATAAAAGACGTGGTGTAACTC
AAATGGGAAATACAAACTATATTACTGAAGCTACTATTATGAGACCAGCTGAGGTTGGTTATAGTGCA
CCATATTATTCTTTTGAGGCGTCTACACAAGGGCCATTTAAAACACCTATTGCAGCAGGACGGGGGGG
AGCGCAAACAGATGAAAATCAAGCAGCAGATGGTGATCCAAGATATGCATTTGGTAGACAACATGGTC
AAAAAACTACCACAACAGGAGAAACACCTGAGAGATTTACATATATAGCACATCAAGATACAGGAAGA
TATCCAGAAGGAGATTGGATTCAAAATATTAACTTTAACCTTCCTGTAACAGAAGATAATGTATTGCT
ACCAACAGATCCAATTGGAGGTAAAACAGGAATTAACTATACTAATATATTTAATACTTATGGTCCTT
TAACTGCATTAAATAATGTACCACCAGTTTATCCAAATGGTCAAATTTGGGATAAAGAATTTGATACT
GACTTAAAACCAAGACTTCATGTAAATGCACCATTTGTTTGTCAAAATAATTGTCCCGGTCAATTATT
TGTAAAAGTTGCGCCTAATTTAACAAATGAATATGATCCTGATGCATCTGCTAATATGTCAAGAATTG
TAACTTACTCAGATTTTTGGTGGAAAGGTAAATTAGTATTTAAAGCTAAACTAAGAGCCTCTCATACT

TGGAATCCAATTCAACAAATGAGTATTAATGTAGATAACCAATTTAACTATGTACCAAGTAATATTGG
AGGTATGAAAATTGTATATGAAAAATCTCAACTAGCACCTAGA
'URU_KM457122_2c_UY258_2010'
ATGAGTGATGGAGCAGTTCAACCAGACGGTGGTCAACCTGCTGTCAGAAATGAAAGAGCAACAGGATC
TGGGAACGGGTCTGGAGGCGGGGGTGGTGGTGGTTCTGGGGGTGTGGGGATTTCTACGGGTACTTTCA
ATAATCAGACGGAATTTAAATTTTTGGAAAACGGATGGGTGGAAATCACAGCAAACTCAAGCAGACTT
GTACATTTAAATATGCCAGAAAGTGAAAATTATAGAAGAGTGGTTGTAAATAATTTGGATAAAACTGC
AGTTAACGGAAACATGGCTTTAGATGATACTCATGCACAAATTGTAACACCTTGGTCATTGGTTGATG
CAAATGCTTGGGGAGTTTGGTTTAATCCAGGAGATTGGCAACTAATTGTTAATACTATGAGTGAGTTG
CATTTAGTTAGTTTTGAACAAGAAATTTTTAATGTTGTTTTAAAGACTGTTTCAGAATCTGCTACTCA
GCCACCAACTAAAGTTTATAATAATGATTTAACTGCATCATTGATGGTTGCATTAGATAGTAATAATA
CTATGCCATTTACTCCAGCAGCTATGAGATCTGAGACATTGGGTTTTTATCCATGGAAACCAACCATA
CCAACTCCATGGAGATATTATTTTCAATGGGATAGAACATTAATACCATCTCATACTGGAACTAGTGG
CACACCAACAAATATATACCATGGTACAGATCCAGATGATGTTCAATTTTATACTATTGAAAATTCTG
TGCCAGTACACTTACTAAGAACAGGTGATGAATTTGCTACAGGAACATTTTTTTTTGATTGTAAACCA
TGTAGACTAACACATACATGGCAAACAAATAGAGCATTGGGCTTACCACCATTTCTAAATTCTTTGCC
TCAAGCTGAAGGAGGTACTAACTTTGGTTATATAGGAGTTCAACAAGATAAAAGACGTGGTGTAACTC
AAATGGGAAATACAAACTATATTACTGAAGCTACTATTATGAGACCAGCTGAGGTTGGTTATAGTGCA
CCATATTATTCTTTTGAGGCGTCTACACAAGGGCCATTTAAAACACCTATTGCAGCAGGACGGGGGGG
AGCGCAAACAGATGAAAATCAAGCAGCAGATGGTGATCCAAGATATGCATTTGGTAGACAACATGGTC
AAAAAACTACCACAACAGGAGAAACACCTGAGAGATTTACATATATAGCACATCAAGATACAGGAAGA
TATCCAGAAGGAGATTGGATTCAAAATATTAACTTTAACCTTCCTGTAACAGAAGATAATGTATTACT
ACCAACAGATCCAATTGGAGGTAAAACAGGAATTAACTATACTAATATATTTAATACTTATGGTCCTT
TAACTGCATTAAATAATGTACCACCAGTTTATCCAAATGGTCAAATTTGGGATAAAGAATTTGATACT
GACTTAAAACCAAGACTTCATGTAAATGCACCATTTGTTTGTCAAAATAATTGTCCTGGTCAATTATT
TGTAAAAGTTGCGCCTAATTTAACAAATGAATATGATCCTGATGCATCTGCTAATATGTCAAGAATTG
TAACTTACTCAGATTTTTGGTGGAAAGGTAAATTAGTATTTAAAGCTAAACTAAGAGCCTCTCATACT
TGGAATCCAATTCAACAAATGAGTATTAATGTAGATAACCAATTTAACTATGTACCAAGTAATATTGG
AGGTATGAAAATTGTATATGAAAAATCTCAACTAGCACCTAGA
'URU_KM457123_2c_UY261_2008'
ATGAGTGATGGAGCAGTTCAACCAGACGGTGGTCAATCTGCTGTCAGAAATGAAAGAGCAACAGGATC
TGGGAACGGGTCTGGAGGCGGGGGTGGTGGTGGTTCTGGGGGTGTGGGGATTTCTACGGGTACTTTCA
ATAATCAGACGGAATTTAAATTTTTGGAAAACGGATGGGTGGAAATCACAGCAAACTCAAGCAGACTT
GTACATTTAAATATGCCAGAAAGTGAAAATTATAGAAGAGTGGTTGTAAATAATTTGGATAAAACTGC
AGTTAACGGAAACATGGCTTTAGATGATACTCATGCACAAATTGTAACACCTTGGTCATTGGTTGATG
CAAATGCTTGGGGAGTTTGGTTTAATCCAGGAGATTGGCAACTAATTGTTAATACTATGAGTGAGTTG
CATTTAGTTAGTTTTGAACAAGAAATTTTTAATGTTGTTTTAAAGACTGTTTCAGAATCTGCTACTCA
GCCACCAACTAAAGTTTATAATAATGATTTAACTGCATCATTGATGGTTGCATTAGATAGTAATAATA
CTATGCCATTTACTCCAGCAGCTATGAGATCTGAGACATTGGGTTTTTATCCATGGAAACCAACCATA
CCAACTCCATGGAGATATTATTTTCAATGGGATAGAACATTAATACCATCTCATACTGGAACTAGTGG
CACACCAACAAATATATACCATGGTACAGATCCAGATGATGTTCAATTTTATACTATTGAAAATTCTG
TGCCAGTACACTTACTAAGAACAGGTGATGAATTTGCTACAGGAACATTTTTTTTTGATTGTAAACCA
TGTAGACTAACACATACATGGCAAACAAATAGAGCATTGGGCTTACCACCATTTCTAAATTCTTTGCC
TCAAGCTGAAGGAGGTACTAACTTTGGTTATATAGGAGTTCAACAAGATAAAAGACGTGGTGTAACTC
AAATGGGAAATACAAACTATATTACTGAAGCTACTATTATGAGACCAGCTGAGGTTGGTTATAGTGCA
CCATATTATTCTTTTGAGGCGTCTACACAAGGGCCATTTAAAACACCTATTGCAGCAGGACGGGGGGG
AGCGCAAACAGATGAAAATCAAGCAGCAGATGGTGATCCAAGATATGCATTTGGTAGACAACATGGTC
AAAAAACTACCACAACAGGAGAAACACCTGAGAGATTTACATATATAGCACATCAAGATACAGGAAGA
TATCCAGAAGGAGATTGGATTCAAAATATTAACTTTAACCTTCCTGTAACAGAAGATAATGTATTACT
ACCAACAGATCCAATTGGAGGTAAAACAGGAATTAACTATACTAATATATTTAATACTTATGGTCCTT
TAACTGCATTAAATAATGTACCACCAGTTTATCCAAATGGTCAAATTTGGGATAAAGAATTTGATACT
GACTTAAAACCAAGACTTCATGTAAATGCACCATTTGTTTGTCAAAATAATTGTCCTGGTCAATTATT
TGTAAAAGTTGCGCCTAATTTAACAAATGAATATGATCCTGATGCATCTGCTAATATGTCAAGAATTG
TAACTTACTCAGATTTTTGGTGGAAAGGTAAATTAGTATTTAAAGCTAAACTAAGAGCCTCTCATACT

TGGAATCCAATTCAACAAATGAGTATTAATGTAGATAACCAATTTAACTATGTACCAAGTAATATTGG
AGGTATGAAAATTGTATATGAAAAATCTCAACTAGCACCTAGA
'URU_KM457124_2c_UY307_2011'
ATGAGTGATGGAGCAGTTCAACCAGACGGTGGTCAACCTGCTGTCAGAAATGAAAGAGCAACAGGATC
TGGGAACGGGTCTGGAGGCGGGGGTGGTGGTGGTTCTGGGGGTGTGGGGATTTCTACGGGTACTTTCA
ATAATCAGACGGAATTTAAATTTTTGGAAAACGGATGGGTGGAAATCACAGCAAACTCAAGCAGACTT
GTACATTTAAATATGCCAGAAAGTGAAAATTATAGAAGAGTGGTTGTAAATAATTTGGATAAAACTGC
AGTTAACGGAAACATGGCTTTAGATGATACTCATGCACAAATTGTAACACCTTGGTCATTGGTTGATG
CAAATGCTTGGGGAGTTTGGTTTAATCCAGGAGATTGGCAACTAATTGTTAATACTATGAGTGAGTTG
CATTTAGTTAGTTTTGAACAAGAAATTTTTAATGTTGTTTTAAAGACTGTTTCAGAATCTGCTACTCA
GCCACCAACTAAAGTTTATAATAATGATTTAACTGCATCATTGATGGTTGCATTAGATAGTAATAATA
CTATGCCATTTACTCCAGCAGCTATGAGATCTGAGACATTGGGTTTTTATCCATGGAAACCAACCATA
CCAACTCCATGGAGATATTATTTTCAATGGGATAGAACATTAATACCATCTCATACTGGAACTAGTGG
CACACCAACAAATATATACCATGGTACAGATCCAGATGATGTTCAATTTTATACTATTGAAAATTCTG
TGCCAGTACACTTACTAAGAACAGGTGATGAATTTGCTACAGGAACATTTTTTTTTGATTGTAAACCA
TGTAGACTAACACATACATGGCAAACAAATAGAGCATTGGGCTTACCACCATTTCTAAATTCTTTGCC
TCAAGCTGAAGGAGGTACTAACTTTGGTTATATAGGAGTTCAACAAGATAAAAGACGTGGTGTAACTC
AAATGGGAAATACAAACTATATTACTGAAGCTACTATTATGAGACCAGCTGAGGTTGGTTATAGTGCA
CCATATTATTCTTTTGAGGCGTCTACACAAGGGCCATTTAAAACACCTATTGCAGCAGGACGGGGGGG
AGCGCAAACAGATGAAAATCAAGCAGCAGATGGTGATCCAAGATATGCATTTGGTAGACAACATGGTC
AAAAAACTACCACAACAGGAGAAACACCTGAGAGATTTACATATATAGCACATCAAGATACAGGAAGA
TATCCAGAAGGAGATTGGATTCAAAATATTAACTTTAACCTTCCTGTAACAGAAGATAATGTATTACT
ACCAACAGATCCAATTGGAGGTAAAACAGGAATTAACTATACTAATATATTTAATACTTATGGTCCTT
TAACTGCATTAAATAATGTACCACCAGTTTATCCAAATGGTCAAATTTGGGATAAAGAATTTGATACT
GACTTAAAACCAAGACTTCATGTAAATGCACCATTTGTTTGTCAAAATAATTGTCCTGGTCAATTATT
TGTAAAAGTTGCGCCTAATTTAACAAATGAATATGATCCTGATGCATCTGCTAATATGTCAAGAATTG
TAACTTACTCAGATTTTTGGTGGAAAGGTAAATTAGTATTTAAAGCTAAACTAAGAGCCTCTCATACT
TGGAATCCAATTCAACAAATGAGTATTAATGTAGATAACCAATTTAACTATGTACCAAGTAATATTGG
AGGTATGAAAATTGTATATGAAAAATCTCAACTAGCACCTAGA
'URU_KM457125_2c_UY317_2011'
ATGAGTGATGGAGCAGTTCAACCAGACGGTGGTCAACCTGCTGTCAGAAATGAAAGAGCAACAGGATC
TGGGAACGGGTCTGGAGGCGGGGGTGGTGGTGGTTCTGGGGGTGTGGGGATTTCTACGGGTACTTTCA
ATAATCAGACGGAATTTAAATTTTTGGAAAACGGATGGGTGGAAATCACAGCAAACTCAAGCAGACTT
GTACATTTAAATATGCCAGAAAGTGAAAATTATAGAAGAGTGGTTGTAAATAATTTGGATAAAACTGC
AGTTAACGGAAACATGGCTTTAGATGATACTCATGCACAAATTGTAACACCTTGGTCATTGGTTGATG
CAAATGCTTGGGGAGTTTGGTTTAATCCAGGAGATTGGCAACTAATTGTTAATACTATGAGTGAGTTG
CATTTAGTTAGTTTTGAACAAGAAATTTTTAATGTTGTTTTAAAGACTGTTTCAGAATCTGCTACTCA
GCCACCAACTAAAGTTTATAATAATGATTTAACTGCATCATTGATGGTTGCATTAGATAGTAATAATA
CTATGCCATTTACTCCAGCAGCTATGAGATCTGAGACATTGGGTTTTTATCCATGGAAACCAACCATA
CCAACTCCATGGAGATATTATTTTCAATGGGATAGAACATTAATACCATCTCATACTGGAACTAGTGG
CACACCAACAAATATATACCATGGTACAGATCCAGATGATGTTCAATTTTATACTATTGAAAATTCTG
TGCCAGTACACTTACTAAGAACAGGTGATGAATTTGCTACAGGAACATTTTTTTTTGATTGTAAACCA
TGTAGACTAACACATACATGGCAAACAAATAGAGCATTGGGCTTACCACCATTTCTAAATTCTTTGCC
TCAAGCTGAAGGAGGTACTAACTTTGGTTATATAGGAGTTCAACAAGATAAAAGACGTGGTGTAACTC
AAATGGGAAATACAAACTATATTACTGAAGCTACTATTATGAGACCAGCTGAGGTTGGTTATAGTGCA
CCATATTATTCTTTTGAGGCGTCTACACAAGGGCCATTTAAAACACCTATTGCAGCAGGACGGGGGGG
AGCGCAAACAGATGAAAATCAAGCAGCAGATGGTGATCCAAGATATGCATTTGGTAGACAACATGGTC
AAAAAACTACCACAACAGGAGAAACACCTGAGAGATTTACATATATAGCACATCAAGATACAGGAAGA
TATCCAGAAGGAGATTGGATTCAAAATATTAACTTTAACCTTCCTGTAACAGAAGATAATGTATTGCT
ACCAACAGATCCAATTGGAGGTAAAACAGGAATTAACTATACTAATATATTTAATACTTATGGTCCTT
TAACTGCATTAAATAATGTACCACCAGTTTATCCAAATGGTCAAATTTGGGATAAAGAATTTGATACT
GACTTAAAACCAAGACTTCATGTAAATGCACCATTTGTTTGTCAAAATAATTGTCCTGGTCAATTATT
TGTAAAAGTTGCGCCTAATTTAACAAATGAATATGATCCTGATGCATCTGCTAATATGTCAAGAATTG
TAACTTACTCAGATTTTTGGTGGAAAGGTAAATTAGTATTTAAAGCTAAACTAAGAGCCTCTCATACT

TGGAATCCAATTCAACAAATGAGTATTAATGTAGATAACCAATTTAACTATGTACCAAGTAATATTGG
AGGTATGAAAATTGTATATGAAAAATCTCAACTAGCACCTAGA
'URU_KM457126_2c_UY318_2010'
ATGAGTGATGGAGCAGTTCAACCAGACGGTGGTCAACCTGCTGTCAGAAATGAAAGAGCAACAGGATC
TGGGAACGGGTCTGGAGGCGGGGGTGGTGGTGGTTCTGGGGGTGTGGGGATTTCTACGGGTACTTTCA
ATAATCAGACGGAATTTAAATTTTTGGAAAACGGATGGGTGGAAATCACAGCAAACTCAAGCAGACTT
GTACATTTAAATATGCCAGAAAGTGAAAATTATAGAAGAGTGGTTGTAAATAATTTGGATAAAACTGC
AGTTAACGGAAACATGGCTTTAGATGATACTCATGCACAAATTGTAACACCTTGGTCATTGGTTGATG
CAAATGCTTGGGGAGTTTGGTTTAATCCAGGAGATTGGCAACTAATTGTTAATACTATGAGTGAGTTG
CATTTAATTAGTTTTGAACAAGAAATTTTTAATGTTGTTTTAAAGACTGTTTCAGAATCTGCTACTCA
GCCACCAACTAAAGTTTATAATAATGATTTAACTGCATCATTGATGGTTGCATTAGATAGTAATAATA
CTATGCCATTTACTCCAGCAGCTATGAGATCTGAGACATTGGGTTTTTATCCATGGAAACCAACCATA
CCAACTCCATGGAGATATTATTTTCAATGGGATAGAACATTAATACCATCTCATACTGGAACTAGTGG
CACACCAACAAATATATACCATGGTACAGATCCAGATGATGTTCAATTTTATACTATTGAAAATTCTG
TGCCAGTACACTTACTAAGAACAGGTGATGAATTTGCTACAGGAACATTTTTTTTTGATTGTAAACCA
TGTAGACTAACACATACATGGCAAACAAATAGAGCATTGGGCTTACCACCATTTCTAAATTCTTTGCC
TCAAGCTGAAGGAGGTACTAACTTTGGTTATATAGGAGTTCAACAAGATAAAAGACGTGGTGTAACTC
AAATGGGAAATGCAAACTATATTACTGAAGCTACTATTATGAGACCAGCTGAGGTTGGTTATAGTGCA
CCATATTATTCTTTTGAGGCGTCTACACAAGGGCCATTTAAAACACCTATTGCAGCAGGACGGGGGGG
AGCGCAAACAGATGAAAATCAAGCAGCAGATGGTGATCCAAGATATGCATTTGGTAGACAACATGGTC
AAAAAACTACCACAACAGGAGAAACACCTGAGAGATTTACATATATAGCACATCAAGATACAGGAAGA
TATCCAGAAGGAGATTGGATTCAAAATATTAACTTTAACCTTCCTGTAACAGAAGATAATGTATTGCT
ACCAACAGATCCAATTGGAGGTAAAACAGGAATTAACTATACTAATATATTTAATACTTATGGTCCTT
TAACTGCATTAAATAATGTACCACCAGTTTATCCAAATGGTCAAATTTGGGATAAAGAATTTGATACT
GACTTAAAACCAAGACTTCATGTAAATGCACCATTTGTTTGTCAAAATAATTGTCCTGGTCAATTATT
TGTAAAAGTTGCGCCTAATTTAACAAATGAATATGATCCTGATGCATCTGCTAATATGTCAAGAATTG
TAACTTACTCAGATTTTTGGTGGAAAGGTAAATTAGTATTTAAAGCTAAACTAAGAGCCTCTCATACT
TGGAATCCAATTCAACAAATGAGTATTAATGTAGATAACCAATTTAACTATGTACCAAGTAATATTGG
AGGTATGAAAATTGTATATGAAAAATCTCAACTAGCACCTAGA
'URU_KM457127_2c_UY326_2011'
ATGAGTGATGGAGCAGTTCAACCAGACGGTGGTCAACCTGCTGTCAGAAATGAAAGAGCAACAGGATC
TGGGAACGGGTCTGGAGGCGGGGGTGGTGGTGGTTCTGGGGGTGTGGGGATTTCTACGGGTACTTTCA
ATAATCAGACGGAATTTAAATTTTTGGAAAACGGATGGGTGGAAATCACAGCAAACTCAAGCAGACTT
GTACATTTAAATATGCCAGAAAGTGAAAATTATAGAAGAGTGGTTGTAAATAATTTGGATAAAACTGC
AGTTAACGGAAACATGGCTTTAGATGATACTCATGCACAAATTGTAACACCTTGGTCATTGGTTGATG
CAAATGCTTGGGGAGTTTGGTTTAATCCAGGAGATTGGCAACTAATTGTTAATACTATGAGTGAGTTG
CATTTAGTTAGTTTTGAACAAGAAATTTTTAATGTTGTTTTAAAGACTGTTTCAGAATCTGCTACTCA
GCCACCAACTAAAGTTTATAATAATGATTTAACTGCATCATTGATGGTTGCATTAGATAGTAATAATA
CTATGCCATTTACTCCAGCAGCTATGAGATCTGAGACATTGGGTTTTTATCCATGGAAACCAACCATA
CCAACTCCATGGAGATATTATTTTCAATGGGATAGAACATTAATACCATCTCATACTGGAACTAGTGG
CACACCAACAAATATATACCATGGTACAGATCCAGATGATGTTCAATTTTATACTATTGAAAATTCTG
TGCCAGTACACTTACTAAGAACAGGTGATGAATTTGCTACAGGAACATTTTTTTTTGATTGTAAACCA
TGTAGACTAACACATACATGGCAAACAAATAGAGCATTGGGCTTACCACCATTTCTAAATTCTTTGCC
TCAAGCAGAAGGAGGTACTAACTTTGGTTATATAGGAGTTCAACAAGATAAAAGACGTGGTGTAACTC
AAATGGGAAATACAAACTATATTACTGAAGCTACTATTATGAGACCAGCTGAGGTTGGTTATAGTGCA
CCATATTATTCTTTTGAGGCGTCTACACAAGGGCCATTTAAAACACCTATTGCAGCAGGACGGGGGGG
AGCGCAAACAGATGAAAATCAAGCAGCAGATGGTGATCCAAGATATGCATTTGGTAGACAACATGGTC
AAAAAACTACCACAACAGGAGAAACACCTGAGAGATTTACATATATAGCACATCAAGATACAGGAAGA
TATCCAGAAGGAGATTGGATTCAAAATATTAACTTTAACCTTCCTGTAACAGAAGATAATGTATTGCT
ACCAACAGATCCAATTGGAGGTAAAACAGGAATTAACTATACTAATATATTTAATACTTATAGTCCTT
TAACTGCATTAAATAATGTACCACCAGTTTATCCAAATGGTCAAATTTGGGATAAAGAATTTGATACT
GACTTAAAACCAAGACTTCATGTAAATGCACCATTTGTTTGTCAAAATAATTGTCCTGGTCAATTATT
TGTAAAAGTTGCGCCTAATTTAACAAATGAATATGATCCTGATGCATCTGCTAATATGTCAAGAATTG
TAACTTACTCAGATTTTTGGTGGAAAGGTAAATTAGTATTTAAAGCTAAACTAAGAGCCTCTCATACT

TGGAATCCAATTCAACAAATGAGTATTAATGTAGATAACCAATTTAACTATGTACCAAGTAATATTGG
AGGTATGAAAATTGTATATGAAAAATCTCAACTAGCACCTAGA
'URU_KM457129_2c_UY349_2011'
ATGAGTGATGGAGCAGTTCAACCAGACGGTGGTCAACCTGCTGTCAGAAATGAAAGAGCAACAGGATC
TGGGAACGGGTCTGGAGGCGGGGGTGGTGGTGGTTCTGGGGGTGTGGGGATTTCTACGGGTACTTTCA
ATAATCAGACGGAATTTAAATTTTTGGAAAACGGATGGGTGGAAATCACAGCAAACTCAAGCAGACTT
GTACATTTAAATATGCCAGAAAGTGAAAATTATAGAAGAGTGGTTGTAAATAATTTGGATAAAACTGC
AGTTAACGGAAACATGGCTTTAGATGATACTCATGCACAAATTGTAACACCTTGGTCATTGGTTGATG
CAAATGCTTGGGGAGTTTGGTTTAATCCAGGAGATTGGCAACTAATTGTTAATACTATGAGTGAGTTG
CATTTAGTTAGTTTTGAACAAGAAATTTTTAATGTTGTTTTAAAGACTGTTACAGAATCTGCTACTCA
GCCACCAACTAAAGTTTATAATAATGATTTAACTGCATCATTGATGGTTGCATTAGATAGTAATAATA
CTATGCCATTTACTCCAGCAGCTATGAGATCTGAGACATTGGGTTTTTATCCATGGAAACCAACCATA
CCAACTCCATGGAGATATTATTTTCAATGGGATAGAACATTAATACCATCTCATACTGGAACTAGTGG
CACACCAACAAATATATACCATGGTACAGATCCAGATGATGTTCAATTTTATACTATTGAAAATTCTG
TGCCAGTACACTTACTAAGAACAGGTGATGAATTTGCTACAGGAACATTTTTTTTTGATTGTAAACCA
TGTAGACTAACACATACATGGCAAACAAATAGAGCATTGGGCTTACCACCATTTCTAAATTCTTTGCC
TCAAGCTGAAGGAGGTACTAACTTTGGTTATATAGGAGTTCAACAAGATAAAAGACGTGGTGTAACTC
AAATGGGAAATACAAACTATATTACTGAAGCTACTATTATGAGACCAGCTGAGGTTGGTTATAGTGCA
CCATATTATTCTTTTGAGGCGTCTACACAAGGGCCATTTAAAACACCTATTGCAGCAGGACGGGGGGG
AGCGCAAACAGATGAAAATCAAGCAGCAGATGGTGATCCAAGATATGCATTTGGTAGACAACATGGTC
AAAAAACTACCACAACAGGAGAAACACCTGAGAGATTTACATATATAGCACATCAAGATACAGGAAGA
TATCCAGAAGGAGATTGGATTCAAAATATTAACTTTAACCTTCCTGTAACAGAAGATAATGTATTGCT
ACCAACAGATCCAATTGGAGGTAAAACAGGAATTAACTATACTAATATATTTAATACTTATGGTCCTT
TAACTGCATTAAATAATGTACCACCAGTTTATCCAAATGGTCAAATTTGGGATAAAGAATTTGATACT
GACTTAAAACCAAGACTTCATGTAAATGCACCATTTGTTTGTCAAAATAATTGTCCTGGTCAATTATT
TGTAAAAGTTGCGCCTAATTTAACAAATGAATATGATCCTGATGCATCTGCTAATATGTCAAGAATTG
TAACTTACTCAGATTTTTGGTGGAAAGGTAAATTAGTATTTAAAGCTAAACTAAGAGCCTCTCATACT
TGGAATCCAATTCAACAAATGAGTATTAATGTAGATAACCAATTTAACTATGTACCAAGTAATATTGG
AGGTATGAAAATTGTATATGAAAAATCTCAACTAGCACCTAGA
'URU_KM457130_2c_UY354_2011'
ATGAGTGATGGAGCAGTTCAACCAGACGGTGGTCAACCTGCTGTCAGAAATGAAAGAGCAACAGGATC
TGGGAACGGGTCTGGAGGCGGGGGTGGTGGTGGTTCTGGGGGTGTGGGGATTTCTACGGGTACTTTCA
ATAATCAGACGGAATTTAAATTTTTGGAAAACGGATGGGTGGAAATCACAGCAAACTCAAGCAGACTT
GTACATTTAAATATGCCAGAAAGTGAAAATTATAGAAGAGTGGTTGTAAATAATTTGGATAAAACTGC
AGTTAACGGAAACATGGCTTTAGATGATACTCATGCACAAATTGTAACACCTTGGTCATTGGTTGATG
CAAATGCTTGGGGAGTTTGGTTTAATCCAGGAGATTGGCAACTAATTGTTAATACTATGAGTGAGTTG
CATTTAGTTAGTTTTGAACAAGAAATTTTTAATGTTGTTTTAAAGACTGTTTCAGAATCTGCTACTCA
GCCACCAACTAAAGTTTATAATAATGATTTAACTGCATCATTGATGGTTGCATTAGATAGTAATAATA
CTATGCCATTTACTCCAGCAGCTATGAGATCTGAGACATTGGGTTTTTATCCATGGAAACCAACCATA
CCAACTCCATGGAGATATTATTTTCAATGGGATAGAACATTAATACCATCTCATACTGGAACTAGTGG
CACACCAACAAATATATACCATGGTACAGATCCAGATGATGTTCAATTTTATACTATTGAAAATTCTG
TGCCAGTACACTTACTAAGAACAGGTGATGAATTTGCTACAGGAACATTTTTTTTTGATTGTAAACCA
TGTAGACTAACACATACATGGCAAACAAATAGAGCATTGGGCTTACCACCATTTCTAAATTCTTTGCC
TCAAGCTGAAGGAGGTACTAACTTTGGTTATATAGGAGTTCAACAAGATAAAAGACGTGGTGTAACTC
AAATGGGAAATACAAACTATATTACTGAAGCTACTATTATGAGACCAGCTGAGGTTGGTTATAGTGCA
CCATATTATTCTTTTGAGGCGTCTACACAAGGGCCATTTAAAACACCTATTGCAGCAGGACGGGGGGG
AGCGCAAACAGATGAAAATCAAGCAGCAGATGGTGATCCAAGATATGCATTTGGTAGACAACATGGTC
AAAAAACTACCACAACAGGAGAAACACCTGAGAGATTTACATATATAGCACATCAAGATACAGGAAGA
TATCCAGAAGGAGATTGGATTCAAAATATTAACTTTAACCTTCCTGTAACAGAAGATAATGTATTGCT
ACCAACAGATCCAATTGGAGGTAAAACAGGAATTAACTATACTAATATATTTAATACTTATGGTCCTT
TAACTGCATTAAATAATGTACCACCAGTTTATCCAAATGGTCAAATTTGGGATAAAGAATTTGATACT
GACTTAAAACCAAGACTTCATGTAAATGCACCATTTGTTTGTCAAAATAATTGTCCTGGTCAATTATT
TGTAAAAGTTGCGCCTAATTTAACAAATGAATATGATCCTGATGCATCTGCTAATATGTCAAGAATTG
TAACTTACTCAGATTTTTGGTGGAAAGGTAAATTAGTATTTAAAGCTAAACTAAGAGCCTCTCATACT

TGGAATCCAATTCAACAAATGAGTATTAATGTAGATAACCAATTTAACTATGTACCAAGTAATATTGG
AGGTATGAAAATTGTATATGAAAAATCTCAACTAGCACCTAGA
'URU_KM457131_2c_UY368_2011'
ATGAGTGATGGAGCAGTTCAACCAGACGGTGGTCAACCTGCTGTCAGAAATGAAAGAGCAACAGGATC
TGGGAACGGGTCTGGAGGCGGGGGTGGTGGTGGTTCTGGGGGTGTGGGGATTTCTACGGGTACTTTCA
ATAATCAGACGGAATTTAAATTTTTGGAAAACGGATGGGTGGAAATCACAGCAAACTCAAGCAGACTT
GTACATTTAAATATGCCAGAAAGTGAAAATTATAGAAGAGTGGTTGTAAATAATTTGGATAAAACTGC
AGTTAACGGAAACATGGCTTTAGATGATACTCATGCACAAATTGTAACACCTTGGTCATTGGTTGATG
CAAATGCTTGGGGAGTTTGGTTTAATCCAGGAGATTGGCAACTAATTGTTAATACTATGAGTGAGTTG
CATTTAGTTAGTTTTGAACAAGAAATTTTTAATGTTGTTTTAAAGACTGTTTCAGAATCTGCTACTCA
GCCACCAACTAAAGTTTATAATAATGATTTAACTGCATCATTGATGGTTGCATTAGACAGTAATAATA
CTATGCCATTTACTCCAGCAGCTATGAGATCTGAGACATTGGGTTTTTATCCATGGAAACCAACCATA
CCAACTCCATGGAGATATTATTTTCAATGGGATAGAACATTAATACCATCTCATACTGGAACTAGTGG
CACACCAACAAATATATACCATGGTACAGATCCAGATGATGTTCAATTTTATACTATTGAAAATTCTG
TGCCAGTACACTTACTAAGAACAGGTGATGAATTTGCTACAGGAACATTTTTTTTTGATTGTAAACCA
TGTAGACTAACACATACATGGCAAACAAATAGAGCATTGGGCTTACCACCATTTCTAAATTCTTTGCC
TCAAGCTGAAGGAGGTACTAACTTTGGTTATATAGGAGTTCAACAAGATAAAAGACGTGGTGTAACTC
AAATGGGAAATACAAACTATATTACTGAAGCTACTATTATGAGACCAGCTGAGGTTGGTTATAGTGCA
CCATATTATTCTTTTGAGGCGTCTACACAAGGGCCATTTAAAACACCTATTGCAGCAGGACGGGGGGG
AGCGCAAACAGATGAAAATCAAGCAGCAGATGGTGATCCAAGATATGCATTTGGTAGACAACATGGTC
AAAAAACTACCACAACAGGAGAAACACCTGAGAGATTTACATATATAGCACATCAAGATACAGGAAGA
TATCCAGAAGGAGATTGGATTCAAAATATTAACTTTAACCTTCCTGTAACAGAAGATAATGTATTGCT
ACCAACAGATCCAATTGGAGGTAAAACAGGAATTAACTATACTAATATATTTAATACTTATGGTCCTT
TAACTGCATTAAATAATGTACCACCAGTTTATCCAAATGGTCAAATTTGGGATAAAGAATTTGATACT
GACTTAAAACCAAGACTTCATGTAAATGCACCATTTGTTTGTCAAAATAATTGTCCTGGTCAATTATT
TGTAAAAGTTGCGCCTAATTTAACAAATGAATATGATCCTGATGCATCTGCTAATATGTCAAGAATTG
TAACTTACTCAGATTTTTGGTGGAAAGGTAAATTAGTATTTAAAGCTAAACTAAGAGCCTCTCATACT
TGGAATCCAATTCAACAAATGAGTATTAATGTGGATAACCAATTTAACTATGTACCAAGTAATATTGG
AGGTATGAAAATTGTATATGAAAAATCTCAACTAGCACCTAGA
'URU_KM457142_2c_UY370_2011'
ATGAGTGATGGAGCAGTTCAACCAGACGGTGGTCAACCTGCTGTCAGAAATGAAAGAGCAACAGGATC
TGGGAACGGGTCTGGAGGCGGGGGTGGTGGTGGTTCTGGGGGTGTGGGGATTTCTACGGGTACTTTCA
ATAATCAGACGGAATTTAAATTTTTGGAAAACGGATGGGTGGAAATCACAGCAAACTCAAGCAGACTT
GTACATTTAAATATGCCAGAAAGTGAAAATTATAGAAGAGTGGTTGTAAATAATTTGGATAAAACTGC
AGTTAACGGAAACATGGCTTTAGATGATACTCATGCACAAATTGTAACACCTTGGTCATTGGTTGATG
CAAATGCTTGGGGAGTTTGGTTTAATCCAGGAGATTGGCAACTAATTGTTAATACTATGAGTGAGTTG
CATTTAGTTAGTTTTGAACAAGAAATTTTTAATGTTGTTTTAAAGACTGTTTCAGAATCTGCTACTCA
GCCACCAACTAAAGTTTATAATAATGATTTAACTGCATCATTGATGGTTGCATTAGATAGTAATAATA
CTATGCCATTTACTCCAGCAGCTATGAGATCTGAGACATTGGGTTTTTATCCATGGAAACCAACCATA
CCAACTCCATGGAGATATTATTTTCAATGGGATAGAACATTAATACCATCTCATACTGGAACTAGTGG
CACACCAACAAATATATACCATGGTACAGATCCAGATGATGTTCAATTTTATACTATTGAAAATTCTG
TGCCAGTACACTTACTAAGAACAGGTGATGAATTTGCTACAGGAACATTTTTTTTTGATTGTAAACCA
TGTAGACTAACACATACATGGCAAACAAATAGAGCATTGGGCTTACCACCATTTCTAAATTCTTTGCC
TCAAGCTGAAGGAGGTACTAACTTTGGTTATATAGGAGTTCAACAAGATAAAAGACGTGGTGTAACTC
AAATGGGAAATACAAACTATATTACTGAAGCTACTATTATGAGACCAGCTGAGGTTGGTTATAGTGCA
CCATATTATTCTTTTGAGGCGTCTACACAAGGGCCATTTAAAACACCTATTGCAGCAGGACGGGGGGG
AGCGCAAACAGATGAAAATCAAGCAGCAGATGGTGATCCAAGATATGCATTTGGTAGACAACATGGTC
AAAAAACTACCACAACAGGAGAAACACCTGAGAGATTTACATATATAGCACATCAAGATACAGGAAGA
TATCCAGAAGGAGATTGGATTCAAAATATTAACTTTAACCTTCCTGTAACAGAAGATAATGTATTGCT
ACCAACAGATCCAATTGGAGGTAAAACAGGAATTAACTATACTAATATATTTAATACTTATGGTCCTT
TAACTGCATTAAATAATGTACCACCAGTTTATCCAAATGGTCAAATTTGGGATAAAGAATTTGATACT
GACTTAAAACCAAGACTTCATGTAAATGCACCATTTGTTTGTCAAAATAATTGTCCTGGTCAATTATT
TGTAAAAGTTGCGCCTAATTTAACAAATGAATATGATCCTGATGCATCTGCTAATATGTCAAGAATTG
TAACTTACTCAGATTTTTGGTGGAAAGGTAAATTAGTATTTAAAGCTAAACTAAGAGCCTCTCATACT

TGGAATCCAATTCAACAAATGAGTATTAATGTAGATAACCAATTTAACTATGTACCAAGTAATATTGG
AGGTATGAAAATTGTATATGAAAAATCTCAACTAGCACCTAGA

'THA_KP715690_VT28_2014'

ATGAGTGATGGAGCAGTTCAACCAGACGGTGGTCAACCTGCTGTCAGAAATGAAAGAGCTACAGGATC
TGGGAACGGGTCTGGAGGCGGGGGTGGTGGTGGTTCTGGGGGTGTGGGGATTTCTACGGGTACTTTCA
ATAATCAGACAGAATTTAAATTTTTGGAAAACGGATGGGTGGAAATCACAGCAAACTCAAGCAGACTT
GTACATTTAAATATGCCAGAAAGTGAAAATTATAGAAGAGTGGTTGTAAATAATTTGGATAAAACTGC
AGTTAACGGAAACATGGCTTTAGATGATACTCATGCACAAATTGTAACACCTTGGTCATTGGTTGATG
CAAATGCTTGGGGAGTTTGGTTTAATCCAGGAGATTGGCAACTAATTGTTAATACTATGAGTGAGTTG
CATTTAGTTAGTTTTGAACAAGAAATTTTTAATGTTGTTTTAAAGACTGTTTCAGAATCTGCTACTCA
GCCACCAACTAAAGTTTATAATAATGATTTAACTGCATCATTGATGGTTGCATTAGATAGTAATAATA
CTATGCCATTTACTCCAGCAGCTATGAGATCTGAGACATTGGGTTTTTATCCATGGAAACCAACCATA
CCAACTCCATGGAGATATTATTTTCAATGGGATAGAACATTAATACCATCTCATACTGGAACTAGTGG
CACACCAACAAATATATACCATGGTACAGATCCAGATGATGTTCAATTTTACACTATTGAAAATTCTG
TGCCAGTGCACTTACTAAGAACAGGTGATGAATTTGCTACAGGAACATTTTATTTTGATTGTAAACCA
TGTAGACTAACACATACATGGCAAACAAATAGAGCATTGGGCTTACCACCATTTCTAAATTCTTTGCC
TCAAGCTGAAGGAGGTACTAACTTTGGTTATATAGGAGTTCAACAAGATAAAAGACGTGGTGTGACTC
AAATGGGAAATACAAACATTATTACTGAAGCTACTATTATGAGACCAGCTGAGGTTGGTTATAGTGCA
CCATATTATTCTTTTGAGGCGTCTACACAAGGGCCATTTAAAACACCTATTGCAGCAGGACGGGGGGG
AGCGCAAACAGATGAAAATCAAGCAGCAGATGGTGATCCAAGATATGCATTTGGTAGACAACATGGTC
AAAAAACTACCACAACAGGAGAAACACCTGAGAGATTTACATATATAGCACATCAAGATACAGGAAGA
TATCCAGAAGGAGATTGGATTCAAAATATTAACTTTAACCTTCCTGTAACAGATGATAATGTATTGCT
ACCAACAGATCCAATTGGAGGTAAAACAGGAATTAACTATACCAATATATTTAATACTTATGGTCCTT
TAACTGCATTAAATAATGTACCACCAGTTTATCCAAATGGTCAAATTTGGGATAAAGAATTTGATACT
GACTTAAAACCAAGACTTCATGTAAATGCACCATTTGTTTGTCAAAATAATTGTCCTGGTCAATTATT
TGTAAAAGTTGCGCCTAATTTAACAAATGAATATGATCCTGATGCATCTGCTAATATGTCAAGAATTG
TAACTTACTCAGATTTTTGGTGGAAAGGTAAATTAGTATTTAAAGCTAAACTAAGAGCCTCTCATACT
TGGAATCCAATTCAACAAATGAGTATCAATGTAGATAACCAATTTAACTATGTACCAAGTAATATTGG
AGGTATGAAAATTGTATATGAAAAATCTCAACTAGCACCTAGA

'THA_KP715691_VT43_2014'

ATGAGTGATGGAGCAGTTCAACCAGACGGTGGTCAACCTGCTGTCAGAAATGAAAGAGCTACAGGATC
TGGGAACGGGTCTGGAGGCGGGGGTGGTGGTGGTTCTGGGGGTGTGGGGATTTCTACGGGTACTTTCA
ATAATCAGACAGAATTTAAATTTTTGGAAAACGGATGGGTGGAAATCACAGCAAACTCAAGCAGACTT
GTACATTTAAATATGCCAGAAAGTGAAAATTATAGAAGAGTGGTTGTAAATAATTTGGATAAAACTGC
AGTTAACGGAAACATGGCTTTAGATGATACTCATGCACAAATTGTAACACCTTGGTCATTGGTTGATG
CAAATGCTTGGGGAGTTTGGTTTAATCCAGGAGATTGGCAACTAATTGTTAATACTATGAGTGAGTTG
CATTTAGTTAGTTTTGAACAAGAAATTTTTAATGTTGTTTTAAAGACTGTTTCAGAATCTGCTACTCA
GCCACCAACTAAAGTTTATAATAATGATTTAACTGCATCATTGATGGTTGCATTAGATAGTAATAATA
CTATGCCATTTACTCCAGCAGCTATGAGATCTGAGACATTGGGTTTTTATCCATGGAAACCAACCATA
CCAACTCCATGGAGATATTATTTTCAATGGGATAGAACATTAATACCATCTCATACTGGAACTAGTGG
CACACCAACAAATATATACCATGGTACAGATCCAGATGATGTTCAATTTTACACTATTGAAAATTCTG
TGCCAGTGCACTTACTAAGAACAGGTGATGAATTTGCTACAGGAACATTTTATTTTGATTGTAAACCA
TGTAGACTAACACATACATGGCAAACAAATAGAGCATTGGGCTTACCACCATTTCTAAATTCTTTGCC
TCAAGCTGAAGGAGGTACTAACTTTGGTTATATAGGAGTTCAACAAGATAAAAGACGTGGTGTAACTC
AAATGGGAAATACAAACATTATTACTGAAGCTACTATTATGAGACCAGCTGAGGTTGGTTATAGTGCA
CCATATTATTCTTTTGAGGCGTCTACACAAGGGCCATTTAAAACACCTATTGCAGCAGGACGGGGGGG
AGCGCAAACAGATGAAAATCAAGCAGCAGATGGTGATCCAAGATATGCATTTGGTAGACAACATGGTC
AAAAAACTACCACAACAGGAGAAACACCTGAGAGATTTACATATATAGCACATCAAGATACAGGAAGA
TATCCAGAAGGAGATTGGATTCAAAATATTAACTTTAACCTTCCTGTAACAGATGATAATGTATTGCT
ACCAACAGATCCAATTGGAGGTAAAACAGGAATTAACTATACTAATATATTTAATACTTATGGTCCTT
TAACTGCATTAAATAATGTACCACCAGTTTATCCAAATGGTCAAATTTGGGATAAAGAATTTGATACT
GACTTAAAACCAAGACTTCATGTAAATGCACCATTTGTTTGTCAAAATAATTGTCCTGGTCAATTATT
TGTAAAAGTTGCGCCTAATTTAACAAATGAATATGATCCTGATGCATCTGCTAATATGTCAAGAATTG
TAACTTACTCAGATTTTTGGTGGAAAGGTAAATTAGTATTTAAAGCTAAACTAAGAGCCTCTCATACT

TGGAATCCAATTCAACAAATGAGTATCAATGTAGATAACCAATTTAACTATGTACCAAGTAATATTGG
AGGTATGAAAATTGTATATGAAAAATCTCAACTAGCACCTAGA

'THA_KP715716_VT143_2014'

ATGAGTGATGGAGCAGTTCAACCAGACGGTGGTCAACCTGCTGTCAGAAATGAAAGAGCTACAGGATC
TGGGAACGGGTCTGGAGGCGGGGGTGGTGGTGGTTCTGGGGGTGTGGGGATTTCTACGGGTACTTTCA
ATAATCAGACAGAATTTAAATTTTTGGAAAACGGATGGGTGGAAATCACAGCAAACTCAAGCAGACTT
GTACATTTAAATATGCCAGAAAGTGAAAATTATAGAAGAGTGGTTGTAAATAATTTGGATAAAACTGC
AGTTAACGGAAACATGGCTTTAGATGATACTCATGCACAAATTGTAACACCTTGGTCATTGGTTGATG
CAAATGCTTGGGGAGTTTGGTTTAATCCAGGAGATTGGCAACTAATTGTTAATACTATGAGTGAGTTG
CATTTAGTTAGTTTTGAACAAGAAATTTTTAATGTTGTTTTAAAGACTGTTTCAGAATCTGCTACTCA
GCCACCAACTAAAGTTTATAATAATGATTTAACTGCATCATTGATGGTTGCATTAGATAGTAATAATA
CTATGCCATTTACTCCAGCAGCTATGAGATCTGAGACATTGGGTTTTTATCCATGGAAACCAACCATA
CCAACTCCATGGAGATATTATTTTCAATGGGATAGAACATTAATACCATCTCATACTGGAACTAGTGG
CACACCAACAAATATATACCATGGTACAGATCCAGATGATGTTCAATTTTACACTATTGAAAATTCTG
TGCCAGTGCACTTACTAAGAACAGGTGATGAATTTGCTACAGGAACATTTTATTTTGATTGTAAACCA
TGTAGACTAACACATACATGGCAAACAAATAGAGCATTGGGCTTACCACCATTTCTAAATTCTTTGCC
TCAAGCTGAAGGAGGTACTAACTTTGGTTATATAGGAGTTCAACAAGATAAAAGACGTGGTGTAACTC
AAATGGGAAATACAAACATTATTACTGAAGCTACTATTATGAGACCAGCTGAGGTTGGTTATAGTGCA
CCATATTATTCTTTTGAGGCGTCTACACAAGGGCCATTTAAAACACCTATTGCAGCAGGACGGGGGGG
AGCGCAAACAGATGAAAATCAAGCAGCAGATGGTGATCCAAGATATGCATTTGGTAGACAACATGGTC
AAAAAACTACCACAACAGGAGAAACACCTGAGAGATTTACATATATAGCACATCAAGATACAGGAAGA
TATCCAGAAGGAGATTGGATTCAAAATATTAACTTTAACCTTCCTGTAACAGATGATAATGTATTGCT
ACCAACAGATCCAATTGGAGGTAAAACAGGAATTAACTATACCAATATATTTAATACTTATGGTCCTT
TAACTGCATTAAATAATGTACCACCAGTTTATCCAAATGGTCAAATTTGGGATAAAGAATTTGATACT
GACTTAAAACCAAGACTTCATGTAAATGCACCATTTGTTTGTCAAAATAATTGTCCTGGTCAATTATT
TGTAAAAGTTGCGCCTAATTTAACAAATGAATATGATCCTGATGCATCTGCTAATATGTCAAGAATTG
TAACTTACTCAGATTTTTGGTGGAAAGGTAAATTAGTATTTAAAGCTAAACTAAGAGCCTCTCATACT
TGGAATCCAATTCAACAAATGAGTATCAATGTAGATAACCAATTTAACTATGTACCAAGTAATATTGG
AGGTATGAAAATTGTATATGAAAAATCTCAACTAGCACCTAGA

'HRV_KP859574_2c_HR442_2014'

ATGAGTGATGGAGCAGTTCAACCAGACGGTGGTCAACCTGCTGTCAGAAATGAAAGAGCAACAGGATC
TGGGAACGGGTCTGGAGGCGGGGGTGGTGGTGGTTCTGGGGGTGTGGGGATTTCTACGGGTACTTTCA
ATAATCAGACGGAATTTAAATTTTTGGAAAACGGATGGGTGGAAATCACAGCAAACTCAAGCAGACTT
GTACATTTAAATATGCCAGAAAGTGAAAATTATAGAAGAGTGGTTGTAAATAATTTGGATAAAACTGC
AGTTAACGGAAACATGGCTTTAGATGATACTCATGCACAAATTGTAACACCTTGGTCATTGGTTGATG
CAAATGCTTGGGGAGTTTGGTTTAATCCAGGAGATTGGCAACTAATTGTTAATACTATGAGTGAGTTG
CATTTAGTTAGTTTTGAACAAGAAATTTTTAATGTTGTTTTAAAGACTGTTTCAGAATCTGCTACTCA
GCCACCAACTAAAGTTTATAATAATGATTTAACTGCATCATTGATGGTTGCATTAGATAGTAATAATA
CTATGCCATTTACTCCAGCAGCTATGAGATCTGAGACATTGGGTTTTTATCCATGGAAACCAACCATA
CCAACTCCATGGAGATATTATTTTCAATGGGATAGAACATTAATACCATCTCATACTGGAACTAGTGG
CACACCAACAAATATATACCATGGTACAGATCCAGATGATGTTCAATTTTATACTATTGAAAATTCTG
TGCCAGTACACTTACTAAGAACAGGTGATGAATTTGCTACAGGAACATTTTTTTTTGATTGTAAACCA
TGTAGACTAACACATACATGGCAAACAAATAGAGCATTGGGCTTACCACCATTTCTAAATTCTTTGCC
TCAAGCTGAAGGAGGTACTAACTTTGGTTATATAGGAGTTCAACAAGATAAAAGACGTGGTGTAACTC
AAATGGGAAATACAAACTATATTACTGAAGCTACTATTATGAGACCAGCTGAGGTTGGTTATAGTGCA
CCATATTATTCTTTTGAGGCGTCTACACAAGGGCCATTTAAAACACCTATTGCAGCAGGACGGGGGGG
AGCGCAAACAGATGAAAATCAAGCAGCAGATGGTGATCCAAGATATGCATTTGGTAGACAACATGGTC
AAAAAACTACCACAACAGGAGAAACACCTGAGAGATTTACATATATAGCACATCAAGATACAGGAAGA
TATCCAGAAGGAGATTGGATTCAAAATATTAACTTTAACCTTCCTGTAACAGAAGATAATGTATTGCT
ACCAACAGATCCAATTGGAGGTAAAACAGGAATTAACTATACTAATATATTTAATACTTATGGTCCTT
TAACTGCATTAAATAATGTACCACCAGTTTATCCAAATGGTCAAATTTGGGATAAAGAATTTGATACT
GACTTAAAACCAAGACTTCATGTAAATGCACCATTTGTTTGTCAAAATAATTGTCCTGGTCAATTATT
TGTAAAAGTTGCGCCTAATTTAACAAATGAATATGATCCTGATGCATCTGCTAATATGTCAAGAATTG
TAACTTACTCAGATTTTTGGTGGAAAGGTAAATTAGTATTTAAAGCTAAACTAAGAGCCTCTCATACT

TGGAATCCAATTCAACAAATGAGTATTAATGTAGATAACCAATTTAACTATGTACCAAGTAATATTGG
AGGTATGAAAATTGTATATGAAAAATCTCAACTAGCACCTAGA
'HRV_KP859575_2c_HR774_2014'
ATGAGTGATGGAGCAGTTCAACCAGACGGTGGTCAACCTGCTGTCAGAAATGAAAGAGCAACAGGATC
TGGGAACGGGTCTGGAGGCGGGGGTGGTGGTGGTTCTGGGGGTGTGGGGATTTCTACGGGTACTTTCA
ATAATCAGACGGAATTTAAATTTTTGGAAAACGGATGGGTGGAAATCACAGCAAACTCAAGCAGACTT
GTACATTTAAATATGCCAGAAAGTGAAAATTATAGAAGAGTGGTTGTAAATAATTTGGATAAAACTGC
AGTTAACGGAAACATGGCTTTAGATGATACTCATGCACAAATTGTAACACCTTGGTCATTGGTTGATG
CAAATGCTTGGGGAGTTTGGTTTAATCCAGGAGATTGGCAACTAATTGTTAATACTATGAGTGAGTTG
CATTTAGTTAGTTTTGAACAAGAAATTTTTAATGTTGTTTTAAAGACTGTTTCAGAATCTGCTACTCA
GCCACCAACTAAAGTTTATAATAATGATTTAACTGCATCATTGATGGTTGCATTAGATAGTAATAATA
CTATGCCATTTACTCCAGCAGCTATGAGATCTGAGACATTGGGTTTTTATCCATGGAAACCAACCATA
CCAACTCCATGGAGATATTATTTTCAATGGGATAGAACATTAATACCATCTCATACTGGAACTAGTGG
CACACCAACAAATATATACCATGGTACAGATCCAGATGATGTTCAATTTTATACTATTGAAAATTCTG
TGCCAGTACACTTACTAAGAACAGGTGATGAATTTGCTACAGGAACATTTTTTTTTGATTGTAAACCA
TGTAGACTAACACATACATGGCAAACAAATAGAGCATTGGGCTTACCACCATTTCTAAATTCTTTGCC
TCAAGCTGAAGGAGGTACTAACTTTGGTTATATAGGAGTTCAACAAGATAAAAGACGTGGTGTAACTC
AAATGGGAAATACAAACTATATTACTGAAGCTACTATTATGAGACCAGCTGAGGTTGGTTATAGTGCA
CCATATTATTCTTTTGAGGCGTCTACACAAGGGCCATTTAAAACACCTATTGCAGCAGGACGGGGGGG
AGCGCAAACAGATGAAAATCAAGCAGCAGATGGTGATCCAAGATATGCATTTGGTAGACAACATGGTC
AAAAAACTACCACAACAGGAGAAACACCTGAGAGATTTACATATATAGCACATCAAGATACAGGAAGA
TATCCAGAAGGAGATTGGATTCAAAATATTAACTTTAACCTTCCTGTAACAGAAGATAATGTATTGCT
ACCAACAGATCCAATTGGAGGTAAAACAGGAATTAACTATACTAATATATTTAATACTTATGGTCCTT
TAACTGCATTAAATAATGTACCACCAGTTTATCCAAATGGTCAAATTTGGGATAAAGAATTTGATACT
GACTTAAAACCAAGACTTCATGTAAATGCACCATTTGTTTGTCAAAATAATTGTCCTGGTCAATTATT
TGTAAAAGTTGCGCCTAATTTAACAAATGAATATGATCCTGATGCATCTGCTAATATGTCAAGAATTG
TAACTTACTCAGATTTTTGGTGGAAAGGTAAATTAGTATTTAAAGCTAAACTAAGAGCCTCTCATACT
TGGAATCCAATTCAACAAATGAGTATTAATGTAGATAACCAATTTAACTATGTACCAAGTAATATTGG
AGGTATGAAAATTGTATATGAAAAATCTCAACTAGCACCTAGA
'HRV_KP859576_2c_HR793_2014'
ATGAGTGATGGAGCAGTTCAACCAGACGGTGGTCAACCTGCTGTCAGAAATGAAAGAGCAACAGGATC
TGGGAACGGGTCTGGAGGCGGGGGTGGTGGTGGTTCTGGGGGTGTGGGGATTTCTACGGGTACTTTCA
ATAATCAGACGGAATTTAAATTTTTGGAAAACGGATGGGTGGAAATCACAGCAAACTCAAGCAGACTT
GTACATTTAAATATGCCAGAAAGTGAAAATTATAGAAGAGTGGTTGTAAATAATTTGGATAAAACTGC
AGTTAACGGAAACATGGCTTTAGATGATACTCATGCACAAATTGTAACACCTTGGTCATTGGTTGATG
CAAATGCTTGGGGAGTTTGGTTTAATCCAGGAGATTGGCAACTAATTGTTAATACTATGAGTGAGTTG
CATTTAGTTAGTTTTGAACAAGAAATTTTTAATGTTGTTTTAAAGACTGTTTCAGAATCTGCTACTCA
GCCACCAACTAAAGTTTATAATAATGATTTAACTGCATCATTGATGGTTGCATTAGATAGTAATAATA
CTATGCCATTTACTCCAGCAGCTATGAGATCTGAGACATTGGGTTTTTATCCATGGAAACCAACCATA
CCAACTCCATGGAGATATTATTTTCAATGGGATAGAACATTAATACCATCTCATACTGGAACTAGTGG
CACACCAACAAATATATACCATGGTACAGATCCAGATGATGTTCAATTTTATACTATTGAAAATTCTG
TGCCAGTACACTTACTAAGAACAGGTGATGAATTTGCTACAGGAACATTTTTTTTTGATTGTAAACCA
TGTAGACTAACACATACATGGCAAACAAATAGAGCATTGGGCTTACCACCATTTCTAAATTCTTTGCC
TCAAGCTGAAGGAGGTACTAACTTTGGTTATATAGGAGTTCAACAAGATAAAAGACGTGGTGTAACTC
AAATGGGAAATACAAACTATATTACTGAAGCTACTATTATGAGACCAGCTGAGGTTGGTTATAGTGCA
CCATATTATTCTTTTGAGGCGTCTACACAAGGGCCATTTAAAACACCTATTGCAGCAGGACGGGGGGG
AGCGCAAACAGATGAAAATCAAGCAGCAGATGGTGATCCAAGATATGCATTTGGTAGACAACATGGTC
AAAAAACTACCACAACAGGAGAAACACCTGAGAGATTTACATATATAGCACATCAAGATACAGGAAGA
TATCCAGAAGGAGATTGGATTCAAAATATTAACTTTAACCTTCCTGTAACAGAAGATAATGTATTGCT
ACCAACAGATCCAATTGGAGGTAAAACAGGAATTAACTATACTAATATATTTAATACTTATGGTCCTT
TAACTGCATTAAATAATGTACCACCAGTTTATCCAAATGGTCAAATTTGGGATAAAGAATTTGATACT
GACTTAAAACCAAGACTTCATGTAAATGCACCATTTGTTTGTCAAAATAATTGTCCTGGTCAATTATT
TGTAAAAGTTGCGCCTAATTTAACAAATGAATATGATCCTGATGCATCTGCTAATATGTCAAGAATTG
TAACTTACTCAGATTTTTGGTGGAAAGGTAAATTAGTATTTAAAGCTAAACTAAGAGCCTCTCATACT

TGGAATCCAATTCAACAAATGAGTATTAATGTAGATAACCAATTTAACTATGTACCAAGTAATATTGG
AGGTATGAAAATTGTATATGAAAAATCTCAACTAGCACCTAGA

'HRV_KP859577_2c_HR856_2014'

ATGAGTGATGGAGCAGTTCACCCAGACGGTGGTCAACCTGCTGTCAGAAATGAAAGAGCAACAGGATC
TGGGAACGGGTCTGGAGGCGGGGGTGGTGGTGGTTCTGGGGGTGTGGGGATTTCTACGGGTACTTTCA
ATAATCAGACGGAATTTAAATTTTTGGAAAACGGATGGGTGGAAATCACAGCAAACTCAAGCAGACTT
GTACATTTAAATATGCCAGAAAGTGAAAATTATAGAAGAGTGGTTGTAAATAATTTGGATAAAACTGC
AGTTAACGGAAACATGGCTTTAGATGATACTCATGCACAAATTGTAACACCTTGGTCATTGGTTGATG
CAAATGCTTGGGGAGTTTGGTTTAATCCAGGAGATTGGCAACTAATTGTTAATACTATGAGTGAGTTG
CATTTAGTTAGTTTTGAACAAGAAATTTTTAATGTTGTTTTAAAGACTGTTTCAGAATCTGCTACTCA
GCCACCAACTAAAGTTTATAATAATGATTTAACTGCATCATTGATGGTTGCATTAGATAGTAATAATA
CTATGCCATTTACTCCAGCAGCTATGAGATCTGAGACATTGGGTTTTTATCCATGGAAACCAACCATA
CCAACTCCATGGAGATATTATTTTCAATGGGATAGAACATTAATACCATCTCATACTGGAACTAGTGG
CACACCAACAAATATATACCATGGTACAGATCCAGATGATGTTCAATTTTATACTATTGAAAATTCTG
TGCCAGTACACTTACTAAGAACAGGTGATGAATTTGCTACAGGAACATTTTTTTTTGATTGTAAACCA
TGTAGACTAACACATACATGGCAAACAAATAGAGCATTGGGCTTACCACCATTTCTAAATTCTTTGCC
TCAAGCTGAAGGAGGTACTAACTTTGGTTATATAGGAGTTCAACAAGATAAAAGACGTGGTGTAACTC
AAATGGGAAATACAAACTATATTACTGAAGCTACTATTATGAGACCAGCTGAGGTTGGTTATAGTGCA
CCATATTATTCTTTTGAGGCGTCTACACAAGGGCCATTTAAAACACCTATTGCAGCAGGACGGGGGGG
AGCGCAAACAGATGAAAATCAAGCAGCAGATGGTGATCCAAGATATGCATTTGGTAGACAACATGGTC
AAAAAACTACCACAACAGGAGAAACACCTGAGAGATTTACATATATAGCACATCAAGATACAGGAAGA
TATCCAGAAGGAGATTGGATTCAAAATATTAACTTTAACCTTCCTGTAACAGAAGATAATGTATTGCT
ACCAACAGATCCAATTGGAGGTAAAACAGGAATTAACTATACTAATATATTTAATACTTATGGTCCTT
TAACTGCATTAAATAATGTACCACCAGTTTATCCAAATGGTCAAATTTGGGATAAAGAATTTGATACT
GACTTAAAACCAAGACTTCATGTAAATGCACCATTTGTTTGTCAAAATAATTGTCCTGCTCAATTATT
TGTAAAAGTTGCGCCTAATTTAACAAATGAATATGATCCTGATGCATCTGCTAATATGTCAAGAATTG
TAACTTACTCAGATTTTTGGTGGAAAGGTAAATTAGTATTTAAAGCTAAACTAAGAGCCTCTCATACT
TGGAATCCAATTCAACAAATGAGTATTAATGTAGATAACCAATTTAACTATGTACCAAGTAATATTGG
AGGTATGAAAATTGTATATGAAAAATCTCAACTAGCACCTAGA

'HRV_KP859578_2c_HR859_2014'

ATGAGTGATGGAGCAGTTCAACCAGACGGTGGTCAACCTGCTGTCAGAAATGAAAGAGCAACAGGATC
TGGGAACGGGTCTGGAGGCGGGGGTGGTGGTGGTTCTGGGGGTGTGGGGATTTCTACGGGTACTTTCA
ATAATCAGACGGAATTTAAATTTTTGGAAAACGGATGGGTGGAAATCACAGCAAACTCAAGCAGACTT
GTACATTTAAATATGCCAGAAAGTGAAAATTATAGAAGAGTGGTTGTAAATAATTTGGATAAAACTGC
AGTTAACGGAAACATGGCTTTAGATGATACTCATGCACAAATTGTAACACCTTGGTCATTGGTTGATG
CAAATGCTTGGGGAGTTTGGTTTAATCCAGGAGATTGGCAACTAATTGTTAATACTATGAGTGAGTTG
CATTTAGTTAGTTTTGAACAAGAAATTTTTAATGTTGTTTTAAAGACTGTTTCAGAATCTGCTACTCA
GCCACCAACTAAAGTTTATAATAATGATTTAACTGCATCATTGATGGTTGCATTAGATAGTAATAATA
CTATGCCATTTACTCCAGCAGCTATGAGATCTGAGACATTGGGTTTTTATCCATGGAAACCAACCATA
CCAACTCCATGGAGATATTATTTTCAATGGGATAGAACATTAATACCATCTCATACTGGAACTAGTGG
CACACCAACAAATATATACCATGGTACAGATCCAGATGATGTTCAATTTTATACTATTGAAAATTCTG
TGCCAGTACACTTACTAAGAACAGGTGATGAATTTGCTACAGGAACATTTTTTTTTGATTGTAAACCA
TGTAGACTAACACATACATGGCAAACAAATAGAGCATTGGGCTTACCACCATTTCTAAATTCTTTGCC
TCAAGCTGAAGGAGGTACTAACTTTGGTTATATAGGAGTTCAACAAGATAAAAGACGTGGTGTAACTC
AAATGGGAAATACAAACTATATTACTGAAGCTACTATTATGAGACCAGCTGAGGTTGGTTATAGTGCA
CCATATTATTCTTTTGAGGCGTCTACACAAGGGCCATTTAAAACACCTATTGCAGCAGGACGGGGGGG
AGCGCAAACAGATGAAAATCAAGCAGCAGATGGTGATCCAAGATATGCATTTGGTAGACAACATGGTC
AAAAAACTACCACAACAGGAGAAACACCTGAGAGATTTACATATATAGCACATCAAGATACAGGAAGA
TATCCAGAAGGAGATTGGATTCAAAATATTAACTTTAACCTTCCTGTAACGGAAGATAATGTATTGCT
ACCAACAGATCCAATTGGAGGTAAAACAGGAATTAACTATACTAATATATTTAATACTTATGGTCCTT
TAACTGCATTAAATAATGTACCACCAGTTTATCCAAATGGTCAAATTTGGGATAAAGAATTTGATACT
GACTTAAAACCAAGACTTCATGTAAATGCACCATTTGTTTGTCAAAATAATTGTCCTGGTCAATTATT
TGTAAAAGTTGCGCCTAATTTAACAAATGAATATGATCCTGATGCATCTGCTAATATGTCAAGAATTG
TGACTTACTCAGATTTTTGGTGGAAAGGTAAATTAGTATTTAAAGCTAAACTAAGAGCCTCTCATACT

TGGAATCCAATTCAACAAATGAGTATTAATGTAGATAACCAATTTAACTATGTACCAAGTAATATTGG
AGGTATGAAGATTGTATATGAAAAATCTCAACTAGCACCTAGA
'POR_KT275252_2c_PT013_12_2012'
ATGAGTGATGGAGCAGTTCAACCAGACGGTGGTCAACCTGCTGTCAGAAATGAAAGAGCAACAGGATC
TGGGAACGGGTCTGGAGGCGGGGGTGGTGGTGGTTCTGGGGGTGTGGGGATTTCTACGGGTACTTTCA
ATAATCAGACGGAATTTAAATTTTTGGAAAACGGATGGGTGGAAATCACAGCAAACTCAAGCAGACTT
GTACATTTAAATATGCCAGAAAGTGAAAATTATAGAAGAGTGGTTGTAAATAATTTGGATAAAACTGC
AGTTAACGGAAACATGGCTTTAGATGATACTCATGCACAAATTGTAACACCTTGGTCATTGGTTGATG
CAAATGCTTGGGGAGTTTGGTTTAATCCAGGAGATTGGCAACTAATTGTTAATACTATGAGTGAGTTG
CATTTAGTCAGTTTTGAACAAGAAATTTTTAATGTTGTTTTAAAGACTGTTTCAGAATCTGCTACTCA
GCCACCAACTAAAGTTTATAATAATGATTTAACTGCATCATTGATGGTTGCATTAGATAGTAATAATA
CTATGCCATTTACTCCAGCAGCTATGAGATCTGAGACATTGGGTTTTTATCCATGGAAACCAACCATA
CCAACTCCATGGAGATATTATTTTCAATGGGATAGAACATTGATACCATCTCATACTGGAACTAGTGG
CACACCAACAAATATATACCATGGTACAGATCCAGATGATGTTCAATTTTATACTATTGAAAATTCTG
TGCCAGTACACTTACTAAGAACAGGTGATGAATTTGCTACAGGAACATTTTTTTTTGATTGTAAACCA
TGTAGACTAACACATACATGGCAAACAAATAGAGCATTGGGCTTACCACCATTTCTAAATTCTTTGCC
TCAAGCTGAAGGAGGTACTAACTTTGGTTATATAGGAGTTCAACAAGATAAAAGACGTGGTGTAACTC
AAATGGGAAATACAAACTATATTACTGAAGCTACTATTATGAGACCAGCTGAGGTTGGTTATAGTGCA
CCATATTATTCTTTTGAGGCGTCTACACAAGGGCCATTTAAAACACCTATTGCAGCAGGACGGGGGGG
AGCGCAAACAGATGAAAATCAAGCAGCAGATGGTGATCCAAGATATGCATTTGGTAGACAACATGGTC
AAAAAACTACCACAACAGGAGAAACACCTGAGAGATTTACATATATAGCACATCAAGATACAGGAAGA
TATCCAGAAGGAGATTGGATTCAAAATATTAACTTTAACCTTCCTGTAACAGAAGATAATGTATTGCT
ACCAACAGATCCAATTGGAGGTAAAACAGGAATTAACTATACTAATATATTTAATACTTATGGTCCTT
TAACTGCATTAAATAATGTACCACCAGTTTATCCAAATGGTCAAATTTGGGATAAAGAATTTGATACT
GACTTAAAACCAAGACTTCATGTAAATGCACCATTTGTTTGTCAAAATAATTGTCCTGGTCAATTATT
TGTAAAAGTTGCGCCTAATTTAACAAATGAATATGATCCTGATGCATCTGCTAATATGTCAAGAATTG
TAACTTACTCAGATTTTTGGTGGAAAGGTAAATTAGTATTTAAAGCTAAACTAAGAGCCTCTCATACT
TGGAATCCAATTCAACAAATGAGTATTAATGTAGATAACCAATTTAACTATGTACCAAGTAATATTGG
AGGTATGAAAATTGTATATGAAAAATCTCAACTAGCACCTAGA
'POR_KT275253_2c_PT036_12_2012'
ATGAGTGATGGAGCAGTTCAACCAGACGGTGGTCAACCTGCTGTCAGAAATGAAAGAGCAACAGGATC
TGGGAACGGGTCTGGAGGCGGGGGTGGTGGTGGTTCTGGGGGTGTGGGGATTTCTACGGGTACTTTCA
ATAATCAGACGGAATTTAAATTTTTGGAAAACGGATGGGTGGAAATCACAGCAAACTCAAGCAGACTT
GTACATTTAAATATGCCAGAAAGTGAAAATTATAGAAGAGTGGTTGTAAATAATTTGGATAAAACTGC
AGTTAACGGAAACATGGCTTTAGATGATACTCATGCACAAATTGTAACACCTTGGTCATTGGTTGATG
CAAATGCTTGGGGAGTTTGGTTTAATCCAGGAGATTGGCAACTAATTGTTAATACTATGAGTGAGTTG
CATTTAGTTAGTTTTGAACAAGAAATTTTTAATGTTGTTTTAAAGACTGTTTCAGAATCTGCTACTCA
GCCACCAACTAAAGTTTATAATAATGATTTAACTGCATCATTGATGGTTGCATTAGATAGTAATAATA
CTATGCCATTTACTCCAGCAGCTATGAGATCTGAGACATTGGGTTTTTATCCATGGAAACCAACCATA
CCAACTCCATGGAGATATTATTTTCAATGGGATAGAACATTAATACCATCTCATACTGGAACTAGTGG
CACACCAACAAATATATACCATGGTACAGATCCAGATGATGTTCAATTTTATACTATTGAAAATTCTG
TGCCAGTACACTTACTAAGAACAGGTGATGAATTTGCTACAGGAACATTTTTTTTTGACTGTAAACCA
TGTAGACTAACACATACATGGCAAACAAATAGAGCATTGGGCTTACCACCATTTCTAAATTCTTTGCC
TCAAGCTGAAGGAGGTACTAACTTTGGTTATATAGGAGTTCAACAAGATAAAAGACGTGGTGTAACTC
AAATGGGAAATACAAACTATATTACTGAAGCTACTATTATGAGACCAGCTGAGGTTGGTTATAGTGCA
CCATATTATTCTTTTGAGGCGTCTACACAAGGGCCATTTAAAACACCTATTGCAGCAGGACGGGGGGG
AGCGCAAACAGATGAAAATCAAGCAGCAGATGGTGATCCAAGATATGCATTTGGTAGACAACATGGTC
AAAAAACTACCACAACAGGAGAAACACCTGAGAGATTTACATATATAGCACATCAAGATACAGGAAGA
TATCCAGAAGGAGATTGGATTCAAAATATTAACTTTAACCTTCCTGTAACAGAAGATAATGTATTGCT
ACCAACAGATCCAATTGGAGGTAAAACAGGAATTAACTATACTAATATATTTAATACTTATGGTCCTT
TAACTGCATTAAATAATGTACCACCAGTTTATCCAAATGGTCAAATTTGGGATAAAGAATTTGATACT
GACTTAAAACCAAGACTTCATGTAAATGCACCATTTGTTTGTCAAAATAATTGTCCTGGTCAATTATT
TGTAAAAGTTGCGCCTAATTTAACAAATGAATATGATCCTGATGCATCTGCTAATATGTCAAGAATTG
TAACTTACTCAGATTTTTGGTGGAAAGGTAAATTAGTATTTAAAGCTAAACTAAGAGCCTCTCATACT

TGGAATCCAATTCAACAAATGAGTATTAATGTAGATAACCAATTTAACTATGTACCAAGTAATATTGG
AGGTATGAAAATTGTTTATGAAAAATCTCAACTAGCACCTAGA
'POR_KT275255_2c_PT238_14_2014'
ATGAGTGATGGAGCAGTTCAACCAGACGGTGGTCAACCTGCTGTCAGAAATGAAAGAGCAACAGGATC
TGGGAACGGGTCTGGAGGCGGGGGTGGTGGTGGTTCTGGGGGTGTGGGGATTTCTACGGGTACTTTCA
ATAATCAGACGGAATTTAAATTTTTGGAAAACGGATGGGTGGAAATCACAGCAAACTCAAGCAGACTT
GTACATTTAAATATGCCAGAAAGTGAAAATTATAGAAGAGTGGTTGTAAATAATTTGGATAAAACTGC
AGTTAACGGAAACATGGCTTTAGATGATACTCATGCACAAATTGTAACACCTTGGTCATTGGTTGATG
CAAATGCTTGGGGAGTTTGGTTTAATCCAGGAGATTGGCAACTAATTGTTAATACTATGAGTGAGTTG
CATTTAGTTAGTTTTGAACAAGAAATTTTTAATGTTGTTTTAAAGACTGTTTCAGAATCTGCTACTCA
GCCACCAACTAAAGTTTATAATAATGATTTAACTGCATCATTGATGGTTGCATTAGATAGTAATAATA
CTATGCCATTTACTCCAGCAGCTATGAGATCTGAGACATTGGGTTTTTATCCATGGAAACCAACCATA
CCAACTCCATGGAGATATTATTTTCAATGGGATAGAACATTAATACCATCTCATACTGGAACTAGTGG
CACACCAACAAATATATACCATGGTACAGATCCAGATGATGTTCAATTTTATACTATTGAAAATTCTG
TGCCAGTACACTTACTAAGAACAGGTGATGAATTTGCTACAGGAACATTTTATTTTGATTGTAAACCA
TGTAGACTAACACATACATGGCAAACAAATAGAGCATTGGGCTTACCACCATTTCTAAATTCTTTGCC
CCAAGCTGAAGGAGGTACTAACTTTGGTTATATAGGAGTTCAACAAGATAAAAGACGTGGTGTAACTC
AAATGGGAAATACAAACTATATTACTGAAGCTACTATTATGAGACCAGCTGAGGTTGGTTATAGTGCA
CCATATTATTCTTTTGAGGCGTCTACACAAGGGCCATTTAAAACACCTATTGCAGCAGGACGGGGGGG
AGCGCAAACAGATGAAAATCAAGCAGCAGATGGTGATCCAAGATATGCATTTGGTAGACAACATGGTC
AAAAAACTACCACAACAGGAGAAACACCTGAGAGATTTACATATATAGCACATCAAGATACAGGAAGA
TATCCAGAAGGAGATTGGATTCAAAATATTAACTTTAACCTTCCTGTAACAGAAGATAATGTATTGCT
ACCAACAGATCCAATTGGAGGTAAAACAGGAATTAACTATACTAATATATTTAATACTTATGGTCCTT
TAACTGCATTAAATAATGTACCACCAGTTTATCCAAATGGTCAAATTTGGGATAAAGAATTTGATACT
GACTTAAAACCAAGACTTCATGTAAATGCACCATTTGTTTGTCAAAATAATTGTCCTGGTCAATTATT
TGTAAAAGTTGCGCCTAATTTAACAAATGAATATGATCCTGATGCATCTGCTAATATGTCAAGAATTG
TAACTTACTCAGATTTTTGGTGGAAAGGTAAATTAGTATTTAAAGCTAAACTAAGAGCCTCTCATACT
TGGAATCCAATTCAACAAATGAGTATTAATGTAGATAACCAATTTAACTATGTACCAAGTAATATTGG
AGGTATGAAAATTGTATATGAAAAATCTCAACTAGCACCTAGA
'ITA_KU508407_2c_25835_09_2009'
ATGAGTGATGGAGCAGTTCAACCAGACGGTGGTCAACCTGCTGTCAGAAATGAAAGAGCAACAGGATC
TGGGAACGGGTCTGGAGGCGGGGGTGGTGGTGGTTCTGGGGGTGTGGGGATTTCTACGGGTACTTTCA
ATAATCAGACGGAATTTAAATTTTTGGAAAACGGATGGGTGGAAATCACAGCAAACTCAAGCAGACTT
GTACATTTAAATATGCCAGAAAGTGAAAATTATAGAAGAGTGGTTGTAAATAATTTGGATAAAACTGC
AGTTAACGGAAACATGGCTTTAGATGATACTCATGCACAAATTGTAACACCTTGGTCATTGGTTGATG
CAAATGCTTGGGGAGTTTGGTTTAATCCAGGAGATTGGCAACTAATTGTTAATACTATGAGTGAGTTG
CATTTAGTTAGTTTTGAACAAGAAATTTTTAATGTTGTTTTAAAGACTGTTTCAGAATCTGCTACTCA
GCCACCAACTAAAGTTTATAATAATGATTTAACTGCATCATTGATGGTTGCATTAGATAGTAATAATA
CTATGCCATTTACTCCAGCAGCTATGAGATCTGAGACATTGGGTTTTTATCCATGGAAACCAACCATA
CCAACTCCATGGAGATATTATTTTCAATGGGATAGAACATTAATACCATCTCATACTGGAACTAGTGG
CACACCAACAAATATATACCATGGTACAGATCCAGATGATGTTCAATTTTATACTATTGAAAATTCTG
TGCCAGTACACCTACTAAGAACAGGTGATGAATTTGCTACAGGAACATTTTTTTTTGATTGTAAACCA
TGTAGACTAACACATACATGGCAAACAAATAGAGCATTGGGCTTACCACCATTTCTAAATTCTTTGCC
TCAAGCTGAAGGAGGTACTAACTTTGGTTATATAGGAGTTCAACAAGATAAAAGACGTGGTGTAACTC
AAATGGGAAATACAAACTATATTACTGAAGCTACTATTATGAGACCAGCTGAGGTTGGTTATAGTGCA
CCATATTATTCTTTTGAGGCGTCTACACAAGGGCCATTTAAAACACCTATTGCAGCAGGACGGGGGGG
AGCGCAAACAGATGAAAATCAAGCAGCAGATGGTGATCCAAGATATGCATTTGGTAGACAACATGGTC
AAAAAACTACCACAACAGGAGAAACACCTGAGAGATTTACATATATAGCACATCAAGATACAGGAAGA
TATCCAGAAGGAGATTGGATTCAAAATATTAACTTTAACCTTCCTGTAACAGAAGATAATGTATTGCT
ACCAACAGATCCAATTGGAGGTAAAACAGGAATTAACTATACTAATATATTTAATACTTATGGTCCTT
TAACTGCATTAAATAATGTACCACCAGTTTATCCAAATGGTCAAATTTGGGATAAAGAATTTGATACT
GACTTAAAACCAAGACTTCATGTAAATGCACCATTTGTTTGTCAAAATAATTGTCCTGGTCAATTATT
TGTAAAAGTTGCGCCTAATTTAACAAATGAATATGATCCTGATGCATCTGCTAATATGTCAAGAATTG
TAACTTACTCAGATTTTTGGTGGAAAGGTAAATTAGTATTTAAAGCTAAACTAAGAGCCTCTCATACT

TGGAATCCAATTCAACAAATGAGTATTAATGTAGATAACCAATTTAACTATGTACCAAGTAATATTGG
AGGTATGAAAATTGTATATGAAAAATCTCAACTAGCACCTAGA
'AUS_KU508691_2c_HB_2015'
ATGAGTGATGGAGCAGTTCAACCAGACGGTGGTCAACCTGCTGTCAGAAATGAAAGAGCAACAGGATC
TGGGAACGGGTCTGGAGGCGGGGGTGGTGGTGGTTCTGGGGGTGTGGGGATTTCTACGGGTACTTTCA
ATAATCAGACGGAATTTAAATTTTTGGAAAACGGATGGGTGGAAATCACAGCAAACTCAAGCAGACTT
GTACATTTAAATATGCCAGAAAGTGAAAATTATAGAAGAGTGGTTGTAAATAATTTGGATAAAACTGC
AGTTAACGGAAACATGGCTTTAGATGATACTCATGCACAAATTGTAACACCTTGGTCATTGGTTGATG
CAAATGCTTGGGGAGTTTGGTTTAATCCAGGAGATTGGCAACTAATTGTTAATACTATGAGTGAGTTG
CATTTAGTTAGTTTTGAACAAGAAATTTTTAATGTTGTTTTAAAGACTGTTTCAGAATCTGCTACTCA
GCCACCAACTAAAGTTTATAATAATGATTTAACTGCATCATTGATGGTTGCATTAGATAGTAATAATA
CTATGCCATTTACTCCAGCAGCTATGAGATCTGAGACATTGGGTTTTTATCCATGGAAACCAACCATA
CCAACTCCATGGAGATATTATTTTCAATGGGATAGAACATTAATACCATCTCATACTGGAACTAGTGG
CACACCAACAAATATATACCATGGTACAGATCCAGATGATGTTCAATTTTATACTATTGAAAATTCTG
TGCCAGTACACTTACTAAGAACAGGTGATGAATTTGCTACAGGAACATTTTTTTTTGATTGTAAACCA
TGTAGACTAACACATACATGGCAAACAAATAGAGCATTGGGCTTACCACCATTTCTAAATTCTTTGCC
TCAAGCTGAAGGAGGTACTAACTTTGGTTATATAGGAGTTCAACAAGATAAAAGACGTGGTGTAACTC
AAATGGGAAATACAAACTATATTACTGAAGCTACTATTATGAGACCAGCTGAGGTTGGTTATAGTGCA
CCATATTATTCTTTTGAGGCGTCTACACAAGGGCCATTTAAAACACCTATTGCAGCAGGACGGGGGGG
AGCGCAAACAGATGAAAATCAAGCAGCAGATGGTGATCCAAGATATGCATTTGGTAGACAACATGGTC
AAAAAACTACCACAACAGGAGAAACACCTGAGAGATTTACATATATAGCACATCAAGATACAGGAAGA
TATCCAGAAGGAGATTGGATTCAAAATATTAACTTTAACCTTCCTGTAACAGAAGATAATGTATTGCT
ACCAACAGATCCAATTGGAGGTAAAACAGGAATTAACTATACTAATATATTTAATACTTATGGTCCTT
TAACTGCATTAAATAATGTACCACCAGTTTATCCAAATGGTCAAATTTGGGATAAAGAATTTGATACT
GACTTAAAACCAAGACTTCATGTAAATGCACCATTTGTTTGTCAAAATAATTGTCCTGGTCAATTATT
TGTAAAAGTTGCGCCTAATTTAACAAATGAATATGATCCTGATGCATCTGCTAATATGTCAAGAATTG
TAACTTACTCAGATTTTTGGTGGAAAGGTAAATTAGTATTTAAAGCTAAACTAAGAGCCTCTCATACT
TGGAATCCAATTCAACAAATGAGTATTAATGTAGATAACCAATTTAACTATGTACCAAGTAATATTGG
AGGTATGAAAATTGTATATGAAAAATCTCAACTAGCACCTAGA
'AUS_KU508692_2c_FH_2015'
ATGAGTGATGGAGCAGTTCAACCAGACGGTGGTCAACCTGCTGTCAGAAATGAAAGAGCAACAGGATC
TGGGAACGGGTCTGGAGGCGGGGGTGGTGGTGGTTCTGGGGGTGTGGGGATTTCTACGGGTACTTTCA
ATAATCAGACGGAATTTAAATTTTTGGAAAACGGATGGGTGGAAATCACAGCAAACTCAAGCAGACTT
GTACATTTAAATATGCCAGAAAGTGAAAATTATAGAAGAGTGGTTGTAAATAATTTGGATAAAACTGC
AGTTAACGGAAACATGGCTTTAGATGATACTCATGCACAAATTGTAACACCTTGGTCATTGGTTGATG
CAAATGCTTGGGGAGTTTGGTTTAATCCAGGAGATTGGCAACTAATTGTTAATACTATGAGTGAGTTG
CATTTAGTTAGTTTTGAACAAGAAATTTTTAATGTTGTTTTAAAGACTGTTTCAGAATCTGCTACTCA
GCCACCAACTAAAGTTTATAATAATGATTTAACTGCATCATTGATGGTTGCATTAGATAGTAATAATA
CTATGCCATTTACTCCAGCAGCTATGAGATCTGAGACATTGGGTTTTTATCCATGGAAACCAACCATA
CCAACTCCATGGAGATATTATTTTCAATGGGATAGAACATTAATACCATCTCATACTGGAACTAGTGG
CACACCAACAAATATATACCATGGTACAGATCCAGATGATGTTCAATTTTATACTATTGAAAATTCTG
TGCCAGTACACTTACTAAGAACAGGTGATGAATTTGCTACAGGAACATTTTTTTTTGATTGTAAACCA
TGTAGACTAACACATACATGGCAAACAAATAGAGCATTGGGCTTACCACCATTTCTAAATTCTTTGCC
TCAAGCTGAAGGAGGTACTAACTTTGGTTATATAGGAGTTCAACAAGATAAAAGACGTGGTGTAACTC
AAATGGGAAATACAAACTATATTACTGAAGCTACTATTATGAGACCAGCTGAGGTTGGTTATAGTGCA
CCATATTATTCTTTTGAGGCGTCTACACAAGGGCCATTTAAAACACCTATTGCAGCAGGACGGGGGGG
AGCGCAAACAGATGAAAATCAAGCAGCAGATGGTGATCCAAGATATGCATTTGGTAGACAACATGGTC
AAAAAACTACCACAACAGGAGAAACACCTGAGAGATTTACATATATAGCACATCAAGATACAGGAAGA
TATCCAGAAGGAGATTGGATTCAAAATATTAACTTTAACCTTCCTGTAACAGAAGATAATGTATTGCT
ACCAACAGATCCAATTGGAGGTAAAACAGGAATTAACTATACTAATATATTTAATACTTATGGTCCTT
TAACTGCATTAAATAATGTACCACCAGTTTATCCAAATGGTCAAATTTGGGATAAAGAATTTGATACT
GACTTAAAACCAAGACTTCATGTAAATGCACCATTTGTTTGTCAAAATAATTGTCCTGGTCAATTATT
TGTAAAAGTTGCGCCTAATTTAACAAATGAATATGATCCTGATGCATCTGCTAATATGTCAAGAATTG
TAACTTACTCAGATTTTTGGTGGAAAGGTAAATTAGTATTTAAAGCTAAACTAAGAGCCTCTCATACT

TGGAATCCAATTCAACAAATGAGTATTAATGTAGATAACCAATTTAACTATGTACCAAGTAATATTGG
AGGTATGAAAATTGTATATGAAAAATCTCAACTAGCACCTAGA
'AUS_KU508693_2c_LW_2015'
ATGAGTGATGGAGCAGTTCAACCAGACGGTGGTCAACCTGCTGTCAGAAATGAAAGAGCAACAGGATC
TGGGAACGGGTCTGGAGGCGGGGGTGGTGGTGGTTCTGGGGGTGTGGGGATTTCTACGGGTACTTTCA
ATAATCAGACGGAATTTAAATTTTTGGAAAACGGATGGGTGGAAATCACAGCAAACTCAAGCAGACTT
GTACATTTAAATATGCCAGAAAGTGAAAATTATAGAAGAGTGGTTGTAAATAATTTGGATAAAACTGC
AGTTAACGGAAACATGGCTTTAGATGATACTCATGCACAAATTGTAACACCTTGGTCATTGGTTGATG
CAAATGCTTGGGGAGTTTGGTTTAATCCAGGAGATTGGCAACTAATTGTTAATACTATGAGTGAGTTG
CATTTAGTTAGTTTTGAACAAGAAATTTTTAATGTTGTTTTAAAGACTGTTTCAGAATCTGCTACTCA
GCCACCAACTAAAGTTTATAATAATGATTTAACTGCATCATTGATGGTTGCATTAGATAGTAATAATA
CTATGCCATTTACTCCAGCAGCTATGAGATCTGAGACATTGGGTTTTTATCCATGGAAACCAACCATA
CCAACTCCATGGAGATATTATTTTCAATGGGATAGAACATTAATACCATCTCATACTGGAACTAGTGG
CACACCAACAAATATATACCATGGTACAGATCCAGATGATGTTCAATTTTATACTATTGAAAATTCTG
TGCCAGTACACTTACTAAGAACAGGTGATGAATTTGCTACAGGAACATTTTTTTTTGATTGTAAACCA
TGTAGACTAACACATACATGGCAAACAAATAGAGCATTGGGCTTACCACCATTTCTAAATTCTTTGCC
TCAAGCTGAAGGAGGTACTAACTTTGGTTATATAGGAGTTCAACAAGATAAAAGACGTGGTGTAACTC
AAATGGGAAATACAAACTATATTACTGAAGCTACTATTATGAGACCAGCTGAGGTTGGTTATAGTGCA
CCATATTATTCTTTTGAGGCGTCTACACAAGGGCCATTTAAAACACCTATTGCAGCAGGACGGGGGGG
AGCGCAAACAGATGAAAATCAAGCAGCAGATGGTGATCCAAGATATGCATTTGGTAGACAACATGGTC
AAAAAACTACCACAACAGGAGAAACACCTGAGAGATTTACATATATAGCACATCAAGATACAGGAAGA
TATCCAGAAGGAGATTGGATGCAAAATATTAACTTTAACCTTCCTGTAACAGAAGATAATGTATTGCT
ACCAACAGATCCAATTGGAGGTAAAACAGGAATTAACTATACTAATATATTTAATACTTATGGTCCTT
TAACTGCATTAAATAATGTACCACCAGTTTATCCAAATGGTCAAATTTGGGATAAAGAATTTGATACT
GACTTAAAACCAAGACTTCATGTAAATGCACCATTTGTTTGTCAAAATAATTGTCCTGGTCAATTATT
TGTAAAAGTTGCGCCTAATTTAACAAATGAATATGATCCTGATGCATCTGCTAATATGTCAAGAATTG
TAACTTACTCAGATTTTTGGTGGAAAGGTAAATTAGTATTTAAAGCTAAACTAAGAGCCTCTCATACT
TGGAATCCAATTCAACAAATGAGTATTAATGTAGATAACCAATTTAACTATGTACCAAGTAATATTGG
AGGTATGAAAATTGTATATGAAAAATCTCAACTAGCACCTAGA
'POR_KU662349_greywolf_W33_1996'
ATGAGTGATGGAGCAGTTCAACCAGACGGTGGTCAACCTGCTGTCAGAAATGAAAGAGCTACAGGATC
TGGGAACGGGTCTGGAGGCGGGGGTGGTGGTGGTTCTGGGGGTGTGGGGATTTCTACGGGTACTTTCA
ATAATCAGACGGAATTTAAATTTTTGGAAAACGGATGGGTGGAAATCACAGCAAACTCAAGCAGACTT
GTACATTTAAATATGCCAGAAAGTGAAAATTATAGAAGAGTGGTTGTAAATAATTTGGATAAAACTGC
AGTTAACGGAAACATGGCTTTAGATGATACTCATGCACAAATTGTAACACCTTGGTCATTGGTTGATG
CAAATGCTTGGGGAGTTTGGTTTAATCCAGGAGATTGGCAACTAATTGTTAATACTATGAGTGAGTTG
CATTTAGTTAGTTTTGAACAAGAAATTTTTAATGTTGTTTTAAAGACTGTTTCAGAATCTGCTACTCA
GCCACCAACTAAAGTTTATAATAATGATTTAACTGCATCATTGATGGTTGCATTAGATAGTAATAATA
CTATGCCATTTACTCCAGCAGCTATGAGATCTGAGACATTGGGTTTTTATCCATGGAAACCAACCATA
CCAACTCCATGGAGATATTATTTTCAATGGGATAGAACATTAATACCATCTCATACTGGAACTAGTGG
CACACCAACAAATATATACCATGGTACAGATCCAGATGATGTTCAATTTTATACTATTGAAAATTCTG
TGCCAGTACACTTACTAAGAACAGGTGATGAATTTGCTACAGGAACATTTTTTTTTGATTGTAAACCA
TGTAGACTAACACATACATGGCAAACAAATAGAGCATTGGGCTTACCACCATTTCTAAATTCTTTGCC
TCAATCTGAAGGAGGTACTAACTTTGGTTATATAGGAGTTCAACAAGATAAAAGACGTGGTGTAACTC
AAATGGGAAATACAAACTATATTACTGAAGCTACTATTATGAGACCAGCTGAGGTTGGTTATAGTGCA
CCATATTATTCTTTTGAGGCGTCTACACAAGGGCCATTTAAAACACCTATTGCAGCAGGACGGGGGGG
AGCGCAAACAGATGAAAATCAAGCAGCAGATGGTGATCCAAGATATGCATTTGGTAGACAACATGGTC
AAAAAACTACCACAACAGGAGAAACACCTGAGAGATTTACATATATAGCACATCAAGATACAGGAAGA
TATCCAGAAGGAGATTGGATTCAAAATATTAACTTTAACCTTCCTGTAACAGATGATAATGTATTGCT
ACCAACAGATCCAATTGGAGGTAAAACAGGAATTAACTATACTAATATATTTAATACTTATGGTCCTT
TAACTGCATTAAATAATGTACCACCAGTTTATCCAAATGGTCAAATTTGGGATAAAGAATTTGATACT
GACTTAAAACCAAGACTTCATGTAAATGCACCATTTGTTTGTCAAAATAATTGTCCTGGTCAATTATT
TGTAAAAGTTGCGCCTAATTTAACAAATGAATATGATCCTGATGCATCTGCTAATATGTCAAGAATTG
TAACTTACTCAGATTTTTGGTGGAAAGGTAAATTAGTATTTAAAGCTAAACTAAGAGCCTCTCATACT

TGGAATCCAATTCAACAAATGAGTATTAATGTAGATAACCAATTTAACTATGTACCAAGTAATATTGG
AGGTATGAAAATTGTATATGAAAAATCTCAACTAGCACCTAGA
'POR_KU662350_greywolf_W52_2005'
ATGAGTGATGGAGCAGTTCAACCAGACGGTGGTCAACCTGCTGTCAGAAATGAAAGAGCTACAGGATC
TGGGAACGGGTCTGGAGGCGGGGGTGGTGGTGGTTCTGGGGGTGTGGGGATTTCTACGGGTACTTTCA
ATAATCAGACGGAATTTAAATTTTTGGAAAACGGATGGGTGGAAATCACAGCAAACTCAAGCAGACTT
GTACATTTAAATATGCCAGAAAGTGAAAATTATAGAAGAGTGGTTGTAAATAATTTGGATAAAACTGC
AGTTAACGGAAACATGGCTTTAGATGATATTCATGCACAAATTGTAACACCTTGGTCATTGGTTGATG
CAATTGCTTGGGGAGTTTGGTTTAATCCAGGAGATTGGCAACTAATTGTTAATACTATGAGTGAGTTG
CATTTAGTTAGTTTTGAACAAGAAATTTTTAATGTTGTTTTAAAGACTGTTTCAGAATCTGCTACTCA
GCCACCAACTAAAGTTTATAATAATGATTTAACTGCATCATTGATGGTTGCATTAGATAGTAATAATA
CTATGCCATTTACTCCAGCAGCTATGAGATCTGAGACATTGGGTTTTTATCCATGGAAACCAACCATA
CCAACTCCATGGAGATATTATTTTCAATGGGATAGAACATTAATACCATCTCATACTGGAACTAGTGG
CACACCAACAAATATATACCATGGTACAGATCCAGATGATGTTCAATTTTATAATATTGCAAATTCTG
TGCCAGTACACTTACTAAGAACAGGTGATGAATTTGCTACAGGAACATTTTTTTTTGATTGTAAACCA
TGTAGACTAACACATACATGGCAAACAAATAGAGCATTGGGCTTACCACCATTTCTAAATTCTTTGCC
TCAATCTGAAGGAGGTACTAACTTTGGTTATATAGGAGTTCAACAAGATAAAAGACGTGGTGTAACTC
AAATGGGAAATACAAACTATATTACTGAAGCTACTATTATGAGACCAGCTGAGGTTGGTTATAGTGCA
CCATATTATTCTTTTGAGGCGTCTACACAAGGGCCATTTAAAACACCTATTGCAGCAGGACGGGGGGG
AGCGCAAACAGATGAAAATCAAGCAGCAGATGGTGATCCAAGATATGCATTTGGTAGACAACATGGTC
AAAAAACTACCACAACAGGAGAAACACCTGAGAGATTTACATATATAGCACATCAAGATACAGGAAGA
TATCCAGAAGGAGATTGGATTCAAAATATTAACTTTAACCTTCCTGTAACAGATGATAATGTATTGCT
ACCAACAGATCCAATTGGAGGTAAAACAGGAATTAACTATACTAATATATTTAATACTTATGGTCCTT
TAACTGCATTAAATAATGTACCACCAGTTTATCCAAATGGTCAAATTTGGGATAAAGAATTTGATACT
GACTTAAAACCAAGACTTCATGTAAATGCACCATTTGTTTGTCAAAATAATTGTCCTGGTCAATTATT
TGTAAAAGTTGCGCCTAATTTAACAAATGAATATGATCCTGATGCATCTGCTAATATGTCAAGAATTG
TAACTTACTCAGATTTTTGGTGGAAAGGTAAATTAGTATTTAAAGCTAAACTAAGAGCCTCTCATACT
TGGAATCCAATTCAACAAATGAGTATTAATGTAGATAACCAATTTAACTATGTACCAAGTAATATTGG
AGGTATGAAAATTGTATATGAAAAATCTCAACTAGCACCTAGA
'ITA_KX434454_29451_09_2009'
ATGAGTGATGGAGCAGTTCAACCAGACGGTGGTCAGCCTGCTGTCAGAAATGAAAGAGCTACAGGATC
TGGGAACGGGTCTGGAGGCGGGGGTGGTGGTGGTTCTGGGGGTGTGGGGATTTCTACGGGTACTTTCA
ATAATCAGACGGAATTTAAATTTTTGGAAAACGGATGGGTGGAAATCACAGCAAACTCAAGCAGACTT
GTACATTTAAATATGCCAGAAAGTGAAAATTATAGAAGAGTGGTTGTAAATAATTTGGATAAAACTGC
AGTTAACGGAAACATGGCTTTAGATGATACCCATGCACAAATTGTAACACCTTGGTCATTGGTTGATG
CAAATGCTTGGGGAGTTTGGTTTAATCCAGGAGATTGGCAACTAATTGTTAATACTATGAGTGAGTTG
CATTTAGTTAGTTTTGAACAAGAAATTTTTAATGTTGTTTTAAAGACTGTTTCAGAATCTGCTACTCA
GCCACCAACTAAAGTTTATAATAATGATTTAACTGCATCATTGATGGTTGCATTAGATAGTAATAATA
CTATGCCATTTACTCCAGCAGCTATGAGATCTGAGACATTGGGTTTTTATCCATGGAAACCAACCATA
CCAACTCCATGGAGATATTATTTTCAATGGGATAGAACATTAATACCATCTCATACTGGAACTAGTGG
CACACCAACAAATATATACCATGGTACAGATCCAGATGATGTTCAATTTTATACTATTGAAAATTCTG
TGCCAGTACACTTACTAAGAACAGGTGATGAATTTGCTACAGGAACATTTTTTTTTGATTGTAAACCA
TGTAGACTAACACATACATGGCAAACAAATAGAGCATTGGGCTTACCACCATTTCTAAATTCTTTGCC
TCAAGCTGAAGGAGGTACTAACTTTGGTTATATAGGAGTTCAACAAGATAAAAGACGTGGTGTAACTC
AAATGGGAAATACAAACTATATTACTGAAGCTACTATTATGAGACCAGCTGAGGTTGGTTATAGTGCA
CCATATTATTCTTTTGAGGCGTCTACACAAGGGCCATTTAAAACACCTATTGCAGCAGGACGGGGGGG
AGCGCAAACAGATGAAAATCAAGCAGCAGATGGTGATCCAAGATATGCATTTGGTAGACAACATGGTC
AAAAAACTACCACAACAGGAGAAACACCTGAGAGATTTACATATATAGCACATCAAGATACAGGAAGA
TATCCAGAAGGAGATTGGATTCAAAATATTAACTTTAACCTTCCTGTAACAAATGATAATGTATTGCT
ACCAACAGATCCAATTGGAGGTAAAGCAGGAATTAACTATACTAATATATTTAATACTTATGGTCCTT
TAACTGCATTAAATAATGTACCACCAGTTTATCCAAATGGTCAAATTTGGGATAAAGAATTTGATACT
GACTTAAAACCAAGACTTCATGTAAATGCACCATTTGTTTGTCAAAATAATTGTCCTGGTCAATTATT
TGTAAAAGTTGCGCCTAATTTAACAAATGAATATGATCCTGATGCATCTGCTAATATGTCAAGAATTG
TAACTTACTCAGATTTTTGGTGGAAAGGTAAATTAGTATTTAAAGCTAAACTAAGAGCCTCTCATACT

TGGAATCCAATTCAACAAATGAGTATTAATGTAGATAACCAATTTAACTATGTACCAAGTAATATTGG
AGGTATGAAAATTGTATATGAAAAATCTCAACTAGCACCTAGA
'ITA_KX434456_45361_09_2009'
ATGAGTGATGGAGCAGTTCAACCAGACGGTGGTCAACCTGCTGTCAGAAATGAAAGAGCAACAGGATC
TGGGAACGGGTCTGGAGGCGGGGGTGGTGGTGGTTCTGGGGGTGTGGGGATTTCTACGGGTACTTTCA
ATAATCAGACGGAATTTAAATTTTTGGAAAACGGATGGGTGGAAATCACAGCAAACTCAAGCAGACTT
GTACATTTAAATATGCCAGAAAGTGAAAATTATAGAAGAGTGGTTGTAAATAATTTGGATAAAACTGC
AGTTAACGGAAACATGGCTTTAGATGATACTCATGCACAAATTGTAACACCTTGGTCATTGGTTGATG
CAAATGCTTGGGGAGTTTGGTTTAATCCAGGAGATTGGCAACTAATTGTTAATACTATGAGTGAGTTG
CATTTAGTTAGTTTTGAACAAGAAATTTTTAATGTTGTTTTAAAGACTGTTTCAGAATCTGCTACTCA
GCCACCAACTAAAGTTTATAATAATGATTTAACTGCATCATTGATGGTTGCATTAGATAGTAATAATA
CTATGCCATTTACTCCAGCAGCTATGAGATCTGAGACATTGGGTTTTTATCCATGGAAACCAACCATA
CCAACTCCATGGAGATATTATTTTCAATGGGATAGAACATTAATACCATCTCATACTGGAACTAGTGG
CACACCAACAAATATATACCATGGTACAGATCCAGATGATGTTCAATTTTATACTATTGAAAATTCTG
TGCCAGTACACTTACTAAGAACAGGTGATGAATTTGCTACAGGAACATTTTTTTTTGATTGTAAACCA
TGTAGACTAACACATACATGGCAAACAAATAGAGCATTGGGCTTACCACCATTTCTAAATTCTTTGCC
TCAAGCTGAAGGAGGTACTAACTTTGGTTATATAGGAGTTCAACAAGATAAAAGACGTGGTGTAACTC
AAATGGGAAATACAAACTATATTACTGAAGCTACTATTATGAGACCAGCTGAGGTTGGTTATAGTGCA
CCATATTATTCTTTTGAGGCGTCTACACAAGGGCCATTTAAAACACCTATTGCAGCAGGACGGGGGGG
AGCGCAAACAGATGAAAATCAAGCAGCAGATGGTGATCCAAGATATGCATTTGGTAGACAACATGGTC
AAAAAACTACCACAACAGGAGAAACACCTGAGAGATTTACATATATAGCACATCAAGATACAGGAAGA
TATCCAGAAGGAGATTGGATTCAAAATATTAACTTTAACCTTCCTGTAACAGAAGATAATGTATTGCT
ACCAACAGATCCAATTGGAGGTAAAACAGGAATTAACTATACTAATATATTTAATACTTATGGTCCTT
TAACTGCATTAAATAATGTACCACCAGTTTATCCAAATGGTCAAATTTGGGATAAAGAATTTGATACT
GACTTAAAACCAAGACTTCATGTAAATGCACCATTTGTTTGTCAAAATAATTGTCCTGGTCAATTATT
TGTAAAAGTTGCGCCTAATTTAACAAATGAATATGATCCTGATGCATCTGCTAATATGTCAAGAATTG
TAACTTACTCAGATTTTTGGTGGAAAGGTAAATTAGTATTTAAAGCTAAACTAAGAGCCTCTCATACT
TGGAATCCAATTCAACAAATGAGTATTAATGTAGATAACCAATTTAACTATGTACCAAGTAATATTGG
AGGTATGAAAATTGTTTATGAAAAATCTCAACTAGCACCTAGA
'ITA_KX434457_987_10_2010'
ATGAGTGATGGAGCAGTTCAACCAGACGGTGGTCAGCCTGCTGTCAGAAATGAAAGAGCGACAGGATC
TGGGAACGGGTCTGGAGGCGGGGGTGGTGGTGGTTCTGGGGGTGTGGGGATTTCTACGGGTACTTTCA
ATAATCAGACGGAATTTAAATTTTTGGAAAACGGATGGGTGGAAATCACAGCAAACTCAAGCAGACTT
GTACATTTAAATATGCCAGAAAGTGAAAATTATAGAAGAGTGGTTGTAAATAATTTGGATAAAACTGC
AGTTAACGGAAACATGGCTTTAGATGATACTCATGCACAGATTGTAACACCTTGGTCATTGGTTGATG
CAAATGCTTGGGGAGTTTGGTTTAATCCAGGAGATTGGCAACTAATTGTTAATACTATGAGTGAGTTG
CATTTAGTTAGTTTTGAACAAGAAATTTTTAATGTTGTTTTAAAGACTGTTTCAGAATCTGCTACTCA
GCCACCAACTAAAGTTTATAATAATGATTTAACTGCATCATTGATGGTTGCATTAGATAGCAATAATA
CTATGCCATTTACTCCAGCAGCTATGAGATCTGAGACATTGGGTTTTTATCCATGGAAACCAACCATA
CCAACTCCATGGAGATATTATTTTCAATGGGATAGAACATTAATACCATCTCATACTGGAACTAGTGG
CACACCAACAAATATATACCATGGTACAGATCCAGATGATGTTCAATTTTATACTATTGAAAATTCTG
TGCCAGTACACTTACTAAGAACAGGTGATGAATTTGCTACAGGAACATTTTTTTTTGATTGTAAACCA
TGTAGACTAACACATACATGGCAAACAAATAGAGCATTGGGCTTACCACCATTTCTAAATTCTTTGCC
TCAAGCTGAAGGAGGTACTAACTTTGGTTATATAGGAGTTCAACAAGATAAAAGACGTGGTGTAACTC
AAATGGGAAATACAAACTATATTACTGAAGCTACTATTATGAGACCAGCTGAGGTTGGTTATAGTGCA
CCATATTATTCTTTTGAGGCGTCTACACAAGGGCCATTTAAAACACCTATTGCAGCAGGACGGGGGGG
AGCGCAAACAGATGAAAATCAAGCAGCAGATGGTGATCCAAGATATGCATTTGGTAGACAACATGGTC
AAAAAACTACCACAACAGGAGAAACACCTGAGAGATTTACATATATAGCACATCAAGATACAGGAAGA
TATCCAGAAGGAGATTGGATTCAAAATATTAACTTTAACCTTCCTGTAACAAATGATAATGTATTGCT
ACCAACAGATCCAATTGGAGGTAAAACAGGAATTAACTATACTAATATATTTAATACTTATGGTCCTT
TAACTGCATTAAATAATGTACCACCAGTTTATCCAAATGGTCAAATTTGGGATAAAGAATTTGATACT
GACTTAAAACCAAGACTTCATGTAAATGCACCATTTGTTTGTCAAAATAATTGTCCTGGTCAATTATT
TGTAAAAGTTGCGCCTAATTTAACAAATGAATATGATCCTGATGCATCTGCTAATATGTCAAGAATTG
TAACTTACTCAGATTTTTGGTGGAAAGGTAAATTAGTATTTAAAGCTAAACTAAGAGCCTCTCATACT

TGGAATCCAATTCAACAAATGAGTATTAATGTAGATAACCAATTTAACTATGTACCAAGTAATATTGG
AGGTATGAAGATTGTATATGAAAAATCTCAACTAGCACCTAGA

'ITA_KX434458_2323_11_2011'

ATGAGTGATGGAGCAGTTCAACCAGACGGTGGTCAACCTGCTGTCAGAAATGAAAGAGCAACAGGATC
TGGGAACGGGTCTGGAGGCGGGGGTGGTGGTGGTTCTGGGGGTGTGGGGATTTCTACGGGTACTTTCA
ATAATCAGACGGAATTTAAATTTTTGGAAAACGGATGGGTGGAAATCACAGCAAACTCAAGCAGACTT
GTACATTTAAATATGCCAGAAAGTGAAAATTATAGAAGAGTGGTTGTAAATAATTTGGATAAAACTGC
AGTTAACGGAAACATGGCTTTAGATGATACTCATGCACAAATTGTAACACCTTGGTCATTGGTTGATG
CAAATGCTTGGGGAGTTTGGTTTAATCCAGGAGATTGGCAACTAATTGTTAATACTATGAGTGAGTTG
CATTTAGTTAGTTTTGAACAAGAAATTTTTAATGTTGTTTTAAAGACTGTTTCAGAATCTGCTACTCA
GCCACCAACTAAAGTTTATAATAATGATTTAACTGCATCATTGATGGTTGCATTAGATAGTAATAATA
CTATGCCATTTACTCCAGCAGCTATGAGATCTGAGACATTGGGTTTTTATCCATGGAAACCAACCATA
CCAACTCCATGGAGATATTATTTTCAATGGGATAGAACATTAATACCATCTCATACTGGAACTAGTGG
CACACCAACAAATATATACCATGGTACAGATCCAGATGATGTTCAATTTTATACTATTGAAAATTCTG
TGCCAGTACACTTACTAAGAACAGGTGATGAATTTGCTACAGGAACATTTTTTTTTGATTGTAAACCA
TGTAGACTAACACATACATGGCAAACAAATAGAGCATTGGGCTTACCACCATTTCTAAATTCTTTGCC
TCAAGCTGAAGGAGGTACTAACTTTGGTTATATAGGAGTTCAACAAGATAAAAGACGTGGTGTAACTC
AAATGGGAAATACAAACTATATTACTGAAGCTACTATTATGAGACCAGCTGAGGTTGGTTATAGTGCA
CCATATTATTCTTTTGAGGCGTCTACACAAGGGCCATTTAAAACACCTATTGCAGCAGGACGGGGGGG
AGCGCAAACAGATGAAAATCAAGCAGCAGATGGTGATCCAAGATATGCATTTGGTAGACAACATGGTC
AAAAAACTACCACAACAGGAGAAACACCTGAGAGATTTACATATATAGCACATCAAGATACAGGAAGA
TATCCAGAAGGAGATTGGATTCAAAATATTAACTTTAACCTTCCTGTAACAGAAGATAATGTATTGCT
ACCAACAGATCCAATTGGAGGTAAAACAGGAATTAACTATACTAATATATTTAATACTTATGGTCCTT
TAACTGCATTAAATAATGTACCACCAGTTTATCCAAATGGTCAAATTTGGGATAAAGAATTTGATACT
GACTTAAAACCAAGACTTCATGTAAATGCACCATTTGTTTGTCAAAATAATTGTCCTGGTCAATTATT
TGTAAAAGTTGCGCCTAATTTAACAAATGAATATGATCCTGATGCATCTGCTAATATGTCAAGAATTG
TAACTTACTCAGATTTTTGGTGGAAAGGTAAATTAGTATTTAAAGCTAAACTAAGAGCCTCTCATACT
TGGAATCCAATTCAACAAATGAGTATTAATGTAGATAACCAATTTAACTATGTACCAAGTAATATTGG
AGGTATGAAAATTGTATATGAAAAATCTCAACTAGCACCTAGA

'ITA_KX434459_27692_1_11_2011'

ATGAGTGATGGAGCAGTTCAACCAGACGGTGGTCAACCTGCTGTCAGAAATGAAAGAGCAACAGGATC
TGGGAACGGGTCTGGAGGCGGGGGTGGTGGTGGTTCTGGGGGTGTGGGGATTTCTACGGGTACTTTCA
ATAATCAGACGGAATTTAAATTTTTGGAAAACGGATGGGTGGAAATCACAGCAAACTCAAGCAGACTT
GTACATTTAAATATGCCAGAAAGTGAAAATTATAGAAGAGTGGTTGTAAATAATTTGGATAAAACTGC
AGTTAACGGAAACATGGCTTTAGATGATACTCATGCACAAATTGTAACACCTTGGTCATTGGTTGATG
CAAATGCTTGGGGAGTTTGGTTTAATCCAGGAGATTGGCAACTAATTGTTAATACTATGAGTGAGTTG
CATTTAGTTAGTTTTGAACAAGAAATTTTTAATGTTGTTTTAAAGACTGTTTCAGAATCTGCTACTCA
GCCACCAACTAAAGTTTATAATAATGATTTAACTGCATCATTGATGGTTGCATTAGATAGTAATAATA
CTATGCCATTTACTCCAGCAGCTATGAGATCTGAGACATTGGGTTTTTATCCATGGAAACCAACCATA
CCAACTCCATGGAGATATTATTTTCAATGGGATAGAACATTAATACCATCTCATACTGGAACTAGTGG
CACACCAACAAATATATACCATGGTACAGATCCAGATGATGTTCAATTTTATACTATTGAAAATTCTG
TGCCAGTACACCTACTAAGAACAGGTGATGAATTTGCTACAGGAACATTTTTTTTTGATTGTAAACCA
TGTAGACTAACACATACATGGCAAACAAATAGAGCATTGGGCTTACCACCATTTCTAAATTCTTTGCC
TCAAGCTGAAGGAGGTACTAACTTTGGTTATATAGGAGTTCAACAAGATAAAAGACGTGGTGTGACTC
AAATGGGAAATACAAACTATATTACTGAAGCTACTATTATGAGACCAGCTGAGGTTGGTTATAGTGCA
CCATATTATTCTTTTGAGGCGTCTACACAAGGGCCATTTAAAACACCTATTGCAGCAGGACGGGGGGG
AGCGCAAACAGATGAAAATCAAGCAGCAGATGGTGATCCAAGATATGCATTTGGTAGACAACATGGTC
AAAAAACTACCACAACAGGAGAAACACCTGAGAGATTTACATATATAGCACATCAAGATACAGGAAGA
TATCCAGAAGGAGATTGGATTCAAAATATTAACTTTAACCTTCCTGTAACAGAAGATAATGTATTGCT
ACCAACAGATCCAATTGGAGGTAAAACAGGAATTAACTATACTAATATATTTAATACTTATGGTCCTT
TAACTGCATTAAATAATGTACCACCAGTTTATCCAAATGGTCAAATTTGGGATAAAGAATTTGATACT
GACTTAAAACCAAGACTTCATGTAAATGCACCATTTGTTTGTCAAAATAATTGTCCTGGTCAATTATT
TGTAAAAGTTGCGCCTAATTTAACAAATGAATATGATCCTGATGCATCTGCTAATATGTCAAGAATTG
TAACTTACTCAGATTTTTGGTGGAAAGGTAAATTAGTATTTAAAGCTAAACTAAGAGCCTCTCATACT

TGGAATCCAATTCAACAAATGAGTATTAATGTAGATAACCAATTTAACTATGTACCAAGTAATATTGG
AGGTATGAAAATTGTATATGAAAAATCTCAACTAGCACCTAGA
'ITA_KX434460_52238_12_2012'
ATGAGTGATGGAGCAGTTCAACCAGACGGTGGTCAACCTGCTGTCAGAAATGAAAGAGCAACAGGATC
TGGGAACGGGTCTGGAGGCGGGGGTGGTGGTGGTTCTGGGGGTGTGGGGATTTCTACGGGTACTTTCA
ATAATCAGACGGAATTTAAATTTTTGGAAAACGGATGGGTGGAAATCACAGCAAACTCAAGCAGACTT
GTACATTTAAATATGCCAGAAAGTGAAAATTATAGAAGAGTGGTTGTAAATAATTTGGATAAAACTGC
AGTTAACGGAAACATGGCTTTAGATGATACTCATGCACAAATTGTAACACCTTGGTCATTGGTTGATG
CAAATGCTTGGGGAGTTTGGTTTAATCCAGGAGATTGGCAACTAATTGTTAATACTATGAGTGAGTTG
CATTTAGTTAGTTTTGAACAAGAAATTTTTAATGTTGTTTTAAAGACTGTTTCAGAATCTGCTACTCA
GCCACCAACTAAAGTTTATAATAATGATTTAACTGCATCATTGATGGTTGCATTAGATAGTAATAATA
CTATGCCATTTACTCCAGCAGCTATGAGATCTGAGACATTGGGTTTTTATCCATGGAAACCAACCATA
CCAACTCCATGGAGATATTATTTTCAATGGGATAGAACATTAATACCATCTCATACTGGAACTAGTGG
CACACCAACAAATATATACCATGGTACAGATCCAGATGATGTTCAATTTTATACTATTGAAAATTCTG
TGCCAGTACACTTACTAAGAACAGGTGATGAATTTGCTACAGGAACATTTTTTTTTGATTGTAAACCA
TGTAGACTAACACATACATGGCAAACAAATAGAGCATTGGGCTTACCACCATTTCTAAATTCTTTGCC
TCAAGCTGAAGGAGGTACTAACTTTGGTTATATAGGAGTTCAACAAGATAAAAGACGTGGTGTAACTC
AAATGGGAAATACAAACTATATTACTGAAGCTACTATTATGAGACCAGCTGAGGTTGGTTATAGTGCC
CCATATTATTCTTTTGAGGCGTCTACACAAGGGCCATTTAAAACACCTATTGCAGCAGGACGGGGGGG
AGCGCAAACAGATGAAAATCAAGCAGCAGATGGTGATCCAAGATATGCATTTGGTAGACAACATGGTC
AAAAAACTACCACAACAGGAGAAACACCTGAGAGATTTACATATATAGCACATCAAGATACAGGAAGA
TATCCAGAAGGAGATTGGATTCAAAATATTAACTTTAACCTTCCTGTAACAGAAGATAATGTATTGCT
ACCAACAGATCCAATTGGAGGTAAAACAGGAATTAACTATACTAATATATTTAATACTTATGGTCCTT
TAACTGCATTAAATAATGTACCACCAGTTTATCCAAATGGTCAAATTTGGGATAAAGAATTTGATACT
GACTTAAAACCAAGACTTCATGTAAATGCACCATTTGTTTGTCAAAATAATTGTCCTGGTCAATTATT
TGTAAAAGTTGCGCCTAATTTAACAAATGAATATGATCCTGATGCATCTGCTAATATGTCAAGAATTG
TAACTTACTCAGATTTTTGGTGGAAAGGTAAATTAGTATTTAAAGCTAAACTAAGAGCCTCTCATACT
TGGAATCCAATTCAACAAATGAGTATTAATGTAGATAACCAATTTAACTATGTACCAAGTAATATTGG
AGGTATGAAAATTGTATATGAGAAATCTCAACTAGCACCTAGA
'IND_KX469432_newCPV_2b_Hiller_2011'
ATGAGTGATGGAGCAGTTCAACCAGACGGTGGTCAGCCTGCTGTCAGAAATGAAAGAGCTACAGGATC
TGGGAACGGGTCTGGAGGCGGGGGTGGTGGTGGTTCTGGGGGTGTGGGGATTTCTACGGGTACTTTCA
ATAATCAGACGGAATTTAAATTTTTGGAAAACGGATGGGTGGAAATCACAGCAAACTCAAGCAGACTT
GTACATTTAAATATGCCAGAAAGTGAAAATTATAGAAGAGTGGTTGTAAATAATTTGGATAAAACTGC
AGTTAACGGAAACATGGCTTTAGATGATACTCATGCACAAATTGTAACACCTTGGTCATTGGTTGATG
CAAATGCTTGGGGAGTTTGGTTTAATCCAGGAGATTGGCAACTAATTGTTAATACTATGAGTGAGTTG
CATTTAATTAGTTTTGAACAAGAAATTTTTAATGTTGTTTTAAAGACTGTTTCAGAATCTGCTACTCA
GCCACCAACTAAAGTTTATAATAATGATTTAACTGCATCATTGATGGTTGCATTAGATAGTAATAATA
CTATGCCATTTACTCCAGCAGCTATGAGATCTGAGACATTGGGTTTTTATCCATGGAAACCAACCATA
CCAACTCCATGGAGATATTATTTTCAATGGGATAGAACATTAATACCATCTCATACTGGAACTAGTGG
CACACCAACAAATATATACCATGGTACAGATCCAGATGACGTCCAATTTTATACTATTGAAAATTCTG
TGCCAGTACACTTACTAAGAACAGGTGATGAATTTGCTACAGGAACATTTTTTTTTGATTGTAAACCA
TGTAGACTAACACATACATGGCAAACAAATAGAGCATTGGGCTTACCACCATTTCTAAATTCTTTGCC
TCAAGCTGAAGGAGGTACTAACTTTGGTTATATAGGAGTTCAACAAGATAAAAGACGTGGTGTAACTC
AAATGGGAAATACAAACTATATTACTGAAGCTACTATTATGAGACCAGCTGAGGTTGGTTATAGTGCA
CCATATTATTCTTTTGAGGCGTCTACACAAGGGCCATTTAAAACACCTATTGCAGCAGGACGGGGGGG
AGCGCAAACAGATGAAAATCAAGCAGCAGATGGTGATCCAAGATATGCATTTGGTAGACAACATGGTC
AAAAAACTACCACAACAGGAGAAACACCTGAGAGATTTACATATATAGCACATCAAGATACAGGAAGA
TATCCAGAAGGAGATTGGATTCAAAATATTAACTTTAACCTTCCTGTAACAGATGATAATGTATTGCT
ACCAACAGATCCAATTGGAGGTAAAACAGGAATTAACTATACCAATATATTTAATACTTATGGTCCTT
TAACTGCATTAAATAATGTACCACCAGTTTATCCAAATGGTCAAATTTGGGATAAAGAATTTGATACT
GACTTAAAACCAAGACTTCATGTAAATGCACCATTTGTTTGTCAAAATAATTGTCCTGGTCAATTATT
TGTAAAAGTTGCGCCTAATTTAACAAATGAATATGATCCTGATGCATCTGCTAATATGTCAAGAATTG
TAACTTACTCAGATTTTTGGTGGAAAGGTAAATTAGTATTTAAAGCTAAACTAAGAGCCTCTCATACT

TGGAATCCAATTCAACAAATGAGTATTAATGTAGATAACCAATTTAACTATGTACCAAGTAATATTGG
AGGTATGAAAATTGTATATGAAAAATCTCAACTAGCACCTAGA
'BRA_KY073269_UFMT_2015'
ATGAGTGATGGAGCAGTTCAACCAGACGGTGGTCAACCTGCTGTCAGAAATGAAAGAGCAACAGGATC
TGGGAACGGGTCTGGAGGCGGGGGTGGTGGTGGTTCTGGGGGTGTGGGGATTTCTACGGGTACTTTCA
ATAATCAGACGGAATTTAAATTTTTGGAAAACGGATGGGTGGAAATCACAGCAAACTCAAGCAGACTT
GTACATTTAAATATGCCAGAAAGTGAAAATTATAGAAGAGTGGTTGTAAATAATTTGGATAAAACTGC
AGTTAACGGAAACATGGCTTTAGATGATACTCATGCACAAATTGTAACACCTTGGTCATTGGTTGATG
CAAATGCTTGGGGAGTTTGGTTTAATCCAGGAGATTGGCAACTAATTGTTAATACTATGAGTGAGTTG
CATTTAGTTAGTTTTGAACAAGAAATTTTTAATGTTGTTTTAAAGACTGTTTCAGAATCTGCTACTCA
GCCACCAACTAAAGTTTATAATAATGATTTGACTGCATCATTGATGGTTGCATTAGATAGTAATAATA
CTATGCCATTTACTCCAGCAGCTATGAGATCTGAGACATTGGGTTTTTATCCATGGAAACCAACCATA
CCAACTCCATGGAGATATTATTTTCAATGGGATAGAACATTAATACCATCTCATACTGGAACTAGTGG
CACACCAACAAACATATACCATGGTACAGATCCAGATGATGTTCAATTTTATACTATTGAAAATTCTG
TGCCAGTACACTTACTAAGAACAGGTGATGAATTTGCTACAGGAACATTTTTTTTTGATTGTAAACCA
TGTAGACTAACACATACATGGCAAACAAATAGAGCATTGGGCTTACCACCATTTCTAAATTCTTTGCC
TCAAGCTGAAGGAGGTACTAACTTTGGTTATATAGGAGTTCAACAAGATAAAAGACGTGGTGTAACTC
AAATGGGAAATACAAACTATATTACTGAAGCTACTATTATGAGACCAGCTGAGGTTGGTTATAGTGCA
CCATATTATTCTTTTGAGGCGTCTACACAAGGGCCATTTAAAACACCTATTGCAGCAGGACGGGGGGG
AGCGCAAACAGATGAAAATCAAGCAGCAGATGGTGATCCAAGATATGCATTTGGTAGACAACATGGTC
AAAAAACTACCACAACAGGAGAAACACCTGAGAGATTTACATATATAGCACATCAAGATACAGGAAGA
TATCCAGAAGGAGATTGGATTCAAAATATTAACTTTAACCTTCCTGTAACAGAAGATAATGTATTGCT
ACCAACAGATCCAATTGGAGGTAAAACAGGAATTAACTATACTAATATATTTAATACTTATGGTCCTT
TAACTGCATTAAATAATGTACCACCAGTTTATCCAAATGGTCAAATTTGGGATAAAGAATTTGATACT
GACTTAAAACCAAGACTTCATGTAAATGCACCATTTGTTTGTCAAAATAATTGTCCTGGTCAATTATT
TGTAAAAGTTGCGCCTAATTTAACAAATGAATATGATCCTGATGCATCTGCTAATATGTCAAGAATTG
TAACTTACTCAGATTTTTGGTGGAAAGGTAAATTAGTATTTAAAGCTAAACTAAGAGCCTCTCATACT
TGGAATCCAATTCAACAAATGAGTATTAATGTAGATAACCAATTTAACTATGTACCAAGTAATATTGG
AGGTATGAAAATTGTATATGAAAAATCTCAACTAGCACCTAGA
'VAC_KY083089_Singapore_2016'
ATGAGTGATGGAGCAGTTCAACCAGACGGTGGTCAACCTGCTGTCAGAAATGAAAGAGCTACAGGATC
TGGGAACGGGTCTGGAGGCGGGGGTGGTGGTGGTTCTGGGGGTGTGGGGATTTCTACGGGTGCTTTCA
ATAATCAGACGGAATTTAAATTTTTGGAAAACGGATGGGTGGAAATCACAGCAAACTCAAGCAGACTT
GTACATTTAAATATGCCAGAAAGTGAAAATTATAGAAGAGTGGTTGTAAATAATATGGATAAAACTGC
AGTTAACGGAAACATGGCTTTAGATGATATTCATGCACAAATTGTAACACCTTGGTCATTGGTTGATG
CAAATGCTTGGGGAGTTTGGTTTAATCCAGGAGATTGGCAACTAATTGTTAATACTATGAGTGAGTTG
CATTTAGTTAGTTTTGAACAAGAAATTTTTAATGTTGTTTTAAAGACTGTTTCAGAATCTGCTACTCA
GCCACCAACTAAAGTTTATAATAATGATTTAACTGCATCATTGATGGTTGCATTAGATAGTAATAATA
CTATGCCATTTACTCCAGCAGCTATGAGATCTGAGACATTGGGTTTTTATCCATGGAAACCAACCATA
CCAACTCCATGGAGATATTATTTTCAATGGGATAGAACATTAATACCATCTCATACTGGAACTAGTGG
CACACCAACAAATATATACCATGGTACAGATCCAGATGATGTTCAATTTTATACTATTGAAAATTCTG
TGCCAGTACACTTACTAAGAACAGGTGATGAATTTGCTACAGGAACATTTTTTTTTGATTGTAAACCA
TGTAGACTAACACATACATGGCAAACAAATAGAGCATTGGGCTTACCACCATTTCTAAATTCTTTGCC
TCAATCTGAAGGAGCTACTAACTTTGGTGATATAGGAGTTCAACAAGATAAAAGACGTGGTGTAACTC
AAATGGGAAATACAAACTATATTACTGAAGCTACTATTATGAGACCAGCTGAGGTTGGTTATAGTGCA
CCATATTATTCTTTTGAGGCGTCTACACAAGGGCCATTTAAAACACCTATTGCAGCAGGACGGGGGGG
AGCGCAAACAGATGAAAATCAAGCAGCAGATGGTGATCCAAGATATGCATTTGGTAGACAACATGGTC
AAAAAACTACCACAACAGGAGAAACACCTGAGAGATTTACATATATAGCACATCAAGATACAGGAAGA
TATCCAGAAGGAGATTGGATTCAAAATATTAACTTTAACCTTCCTGTAACGAATGATAATGTATTGCT
ACCAACAGATCCAATTGGAGGTAAAACAGGAATTAACTATACTAATATATTTAATACTTATGGTCCTT
TAACTGCATTAAATAATGTACCACCAGTTTATCCAAATGGTCAAATTTGGGATAAAGAATTTGATACT
GACTTAAAACCAAGACTTCATGTAAATGCACCATTTGTTTGTCAAAATAATTGTCCTGGTCAATTATT
TGTAAAAGTTGCGCCTAATTTAACAAATGAATATGATCCTGATGCATCTGCTAATATGTCAAGAATTG
TAACTTACTCAGATTTTTGGTGGAAAGGTAAATTAGTATTTAAAGCTAAACTAAGAGCCTCTCATACT

TGGAATCCAATTCAACAAATGAGTATTAATGTAGATAACCAATTTAACTATGTACCAAGTAATATTGG
AGGTATGAAAATTGTATTTGAAAAATCTCAACTAGCACCTAGA
'VAC_KY083090_Singapore_2016'
ATGAGTGATGGAGCAGTTCAACCAGACGGTGGTCAACCTGCTGTCAGAAATGAAAGAGCTACAGGATC
TGGGAACGGGTCTGGAGGCGGGGGTGGTGGTGGTTCTGGGGGTGTGGGGATTTCTACGGGTGCTTTCA
ATAATCAGACGGAATTTAAATTTTTGGAAAACGGATGGGTGGAAATCACAGCAAACTCAAGCAGACTT
GTACATTTAAATATGCCAGAAAGTGAAAATTATAGAAGAGTGGTTGTAAATAATATGGATAAAACTGC
AGTTAACGGAAACATGGCTTTAGATGATATTCATGCACAAATTGTAACACCTTGGTTCATTGGTGATG
CAAATGCTTGGGGAGTTTGGTTTAATCCAGGAGATTGGCAACTAATTGTTAATACTATGAGTGAGTTG
CATTTAGTTAGTTTTGAACAAGAAATTTTTAATGTTGTTTTAAAGACTGTTTCAGAATCTGCTACTCA
GCCACCAACTAAAGTTTATAATAATGATTTAACTGCATCATTGATGGTTGCATTAGATAGTAATAATA
CTATGCCATTTACTCCAGCAGCTATGAGATCTGAGACATTGGGTTTTTATCCATGGAAACCAACCATA
CCAACTCCATGGAGATATTATTTTCAATGGGATAGAACATTAATACCATCTCATACTGGAACTAGTGG
CACACCAACAAATATATACCATGGTACAGATCCAGATGATGTTCAATTTTATACTATTGAAAATTCTG
TGCCAGTACACTTACTAAGAACAGGTGATGAATTTGCTACAGGAACATTTTTTTTTGATTGTAGACCA
TGTAGACTAACACATACATGGCAAACAAATAGAGCATTGGGCTTACCACCATTTCTAAATTCTTTGCC
TCAATCTGAAGGAGCTACTAACTTTGGTGATATAGGAGTTCAACAAGATAAAAGACGTGGTATAACTC
AAATGGGAAATACAAACTATATTACTGAAGCTACTATTATGAGACCAGCTGAGGTTGGTTATAGTGCA
CCATATTATTCTTTTGAGGCGTCTACACAAGGGCCATTTAAAACACCTATTGCAGCAGGACGGGGGGG
AGCGCAAACAGATGAAAATCAAGCAGCAGATGGTAATCCAAGATATGCATTTGGTAGACAACATGGTC
AAAAAACTACCACAACAGGAGAAACACCTGAGAGATTTACATATATAGCACATCAAGATACAGGAAGA
TATCCAGAAGGAGATTGGATTCAAAATATTAACTTTAACCTTCCTGTAACAAATGATAATGTATTGCT
ACCAACAGATCCAATTGGAGGTAAAACAGGAATTAACTATACTAATATATTTAATACTTATGGTCCTT
TAACTGCATTAAATAATGTACCACCAGTTTATCCAAATGGTCAAATTTGGGATAAAGAATTTGATACT
GACTTAAAACCAAGACTTCATGTAAATGCACCATTTGTTTGTCAAAATAATTGTCCTGGTCAATTATT
TGTAAAAGTTGCGCCTAATTTAACGAATGAATATGATCCTGATGCATCTGCTAATATGTCAAGAATTG
TAACTTACTCAGATTTTTGGTGGAAAGGTAAATTAGTATTTAAAGCTAAACTAAGAGCCTCTCATACT
TGGAATCCAATTCAACAAATGAGTATTAATGTAGATAACCAATTTAACTATGTACCAAGTAATATTGG
AGGTATGAAAATTGTATATGAAAAATCTCAACTAGCACCTAGA
'JPN_LC270891_2b_9985_2017'
ATGAGTGATGGAGCAGTTCAACCAGACGGTGGTCAGCCTGCTGTCAGAAATGAAAGAGCTACAGGATC
TGGGAACGGGTCTGGAGGCGGGGGTGGTGGTGGTTCTGGGGGTGTGGGGATTTCTACGGGTACTTTCA
ATAATCAGACGGAATTTAAATTTTTGGAAAACGGATGGGTGGAAATCACAGCAAACTCAAGCAGACTT
GTACATTTAAATATGCCAGAAAGTGAAAATTATAGAAGAGTGGTTGTAAATAATTTGGATAAGACTGC
AGTTAACGGAAACATGGCTTTAGATGATACCCATGCACAAATTGTAACACCTTGGTCATTGGTTGATG
CAAATGCTTGGGGAGTTTGGTTTAATCCAGGAGATTGGCAACTAATTGTTAATACTATGAGTGAGTTG
CATTTAGTTAGTTTTGAACAAGAAATTTTTAATGTTGTTTTAAAGACTGTTTCAGAATCTGCTACTCA
GCCACCAACTAAAGTTTATAATAATGATTTAACTGCATCATTGATGGTTGCATTAGATAGTAATAATA
CTATGCCATTTACTCCAGCAGCTATGAGATCTGAGACATTGGGTTTTTATCCATGGAAACCAACCATA
CCAACTCCATGGAGATATTATTTTCAATGGGATAGAACATTAATACCATCTCATACTGGAACTAGTGG
GACACCAACAAATATATACCATGGTACAGATCCAGATGATGTTCAATTTTATACTATTGAAAATTCTG
TGCCAGTACACTTACTAAGAACAGGTGATGAATTTGCTACAGGAACATTTTTTTTTGATTGTAAACCA
TGTAGACTAACACATACATGGCAAACAAATAGAGCATTGGGCTTACCACCATTTCTAAATTCTTTGCC
TCAAGCTGAAGGAGGTACTAACTTTGGTTATATAGGAGTTCAACAAGATAAAAGACGTGGTGTAACTC
AAATGGGAAATACAAACTATATTACTGAAGCTACTATTATGAGACCAGCTGAGGTTGGTTATAGTGCA
CCATATTATTCTTTTGAGGCGTCTACACAAGGGCCATTTAAAACACCTATTGCAGCAGGACGGGGGGG
AGCGCAAACAGATGAAAATCAAGCAGCAGATGGTGATCCAAGATATGCATTTGGTAGACAACATGGTC
AAAAAACTACCACAACAGGAGAAACACCTGAGAGATTTACATATATAGCACATCAAGATACAGGAAGA
TATCCAGAAGGAGATTGGATTCAAAATATTAACTTTAACCTTCCTGTAACAGATGATAATGTATTGCT
ACCAACAGATCCAATTGGAGGTAAAACAGGAATTAACTATACTAATATATTTAATACTTATGGTCCTT
TAACTGCATTAAATAATGTACCACCAGTTTATCCAAATGGTCAAATTTGGGATAAAGAATTTGATACT
GACTTAAAACCAAGACTTCATGTAAATGCACCATTTGTTTGTCAAAATAATTGTCCTGGTCAATTATT
TGTAAAAGTTGCGCCTAATTTAACAAATGAATATGATCCTGATGCATCTGCTAATATGTCAAGAATTG
TAACTTACTCAGATTTTTGGTGGAAAGGTAAATTAGTATTTAAAGCTAAACTAAGAGCCTCTCATACT

TGGAATCCAATTCAACAAATGAGTATTAATGTAGATAACCAATTTAACTATGTACCAAGTAATATTGG
AGGTATGAAAATTGTATATGAAAAATCTCAACTAGCACCTAGA

'USA_M10989_1985'

ATGAGTGATGGAGCAGTTCAACCAGACGGTGGTCAACCTGCTGTCAGAAATGAAAGAGCTACAGGATC
TGGGAACGGGTCTGGAGGCGGGGGTGGTGGTGGTTCTGGGGGTGTGGGGATTTCTACGGGTACTTTCA
ATAATCAGACGGAATTTAAATTTTTGGAAAACGGATGGGTGGAAATCACAGCAAACTCAAGCAGACTT
GTACATTTAAATATGCCAGAAAGTGAAAAGGATAGAAGAGTGGTTGTAAATAATATGGATAAAACTGC
AGTTAACGGAAACATGGCTTTAGATGATATTCATGCACAAATTGTAACACCTTGGTCATTGGTTGATG
CAAATGCTTGGGGAGTTTGGTTTAATCCAGGAGATTGGCAACTAATTGTTAATACTATGAGTGAGTTG
CATTTAGTTAGTTTTGAACAAGAAATTTTTAATGTTGTTTTAAAGACTGTTTCAGAATCTGCTACTCA
GCCACCAACTAAAGTTTATAATAATGATTTAACTGCATCATTGATGGTTGCATTAGATAGTAATAATA
CTATGCCATTTACTCCAGCAGCTATGAGATCTGAGACATTGGGTTTTTATCCATGGAAACCAACCATA
CCAACTCCATGGAGATATTATTTTCAATGGGATAGAACATTAATACCATCTCATACTGGAACTAGTGG
CACACCAACAAATATATACCATGGTACAGATCCAGATGATGTTCAATTTTATACTATTGAAAATTCTG
TGCCAGTACACTTACTAAGAACAGGTGATGAATTTGCTACAGGAACATTTTTTTTTGATTGTAAACCA
TGTAGACTAACACATACATGGCAAACAAATAGAGCATTGGGCTTACCACCATTTCTAAATTCTTTGCC
TCAATCTGAAGGAGCTACTAACTTTGGTGATATAGGAGTTCCACAAGATAAAAAACGTGGTGTAACTC
AAATGGGAAATACAAACTATATTACTGAAGCTACTATTATGAGACCAGCTGAGGTTGGTTATAGTGCA
CCATATTATTCTTTTGAGGCGTCTACACAAGGGCCATTTAAAACACCTATTGCAGCAGGACGGGGGGG
AGCGCAAACAGATGAAAATCAAGCAGCAGATGGTAATCCAAGATATGCATTTGGTAGACAACATGGTA
AAAAAACTACCACAACAGGAGAAACACCTGAGAGATTTACATATATAGCACATCAAGATACAGGAAGA
TATCCAGAAGGAGATTGGATTCAAAATATTAACTTTAACCTTCCTGTAACAAATGATAATGTATTGCT
ACCAATAGATCCAATTGGAGGTAAAACAGGAATTAACTATACTAATATATTTAATACTTATGGTCCTT
TAACTGCATTAAATAATGTACCACCAGTTTATCCAAATGGTCAAATTTGGGATAAAGAATTTGATACT
GACTTAAAACCAAGACTTCATGTAAATGCACCATTTGTTTGTCAAAATAATTGTCCTGGTCAATTATT
TGTAAAGTTGGCGCCTAATTTAACAAATGAATATGATCCTGATGCATCTGCTAATATGTCAAGAATTG
TAACTTACTCAGATTTTTGGTGGAAAGGTAAATTAGTATTTAAAGCTAAACTAAGAGCCTCTCATACT
TGGAATCCAATTCAACAAATGAGTATTAATGTAGATAACCAATTTAACTATGTACCAAGTAATATTGG
AGGTATGAAAATTGTATATGAAAAATCTCAACTAGCACCTAGA

'USA_M19296_CPV_N_1988'

ATGAGTGATGGAGCAGTTCAACCAGACGGTGGTCAACCTGCTGTCAGAAATGAAAGAGCTACAGGATC
TGGGAACGGGTCTGGAGGCGGGGGTGGTGGTGGTTCTGGGGGTGTGGGGATTTCTACGGGTACTTTCA
ATAATCAGACGGAATTTAAATTTTTGGAAAACGGATGGGTGGAAATCACAGCAAACTCAAGCAGACTT
GTACATTTAAATATGCCAGAAAGTGAAAATTATAGAAGAGTGGTTGTAAATAATATGGATAAAACTGC
AGTTAACGGAAACATGGCTTTAGATGATATTCATGCACAAATTGTAACACCTTGGTCATTGGTTGATG
CAAATGCTTGGGGAGTTTGGTTTAATCCAGGAGATTGGCAACTAATTGTTAATACTATGAGTGAGTTG
CATTTAGTTAGTTTTGAACAAGAAATTTTTAATGTTGTTTTAAAGACTGTTTCAGAATCTGCTACTCA
GCCACCAACTAAAGTTTATAATAATGATTTAACTGCATCATTGATGGTTGCATTAGATAGTAATAATA
CTATGCCATTTACTCCAGCAGCTATGAGATCTGAGACATTGGGTTTTTATCCATGGAAACCAACCATA
CCAACTCCATGGAGATATTATTTTCAATGGGATAGAACATTAATACCATCTCATACTGGAACTAGTGG
CACACCAACAAATATATACCATGGTACAGATCCAGATGATGTTCAATTTTATACTATTGAAAATTCTG
TGCCAGTACACTTACTAAGAACAGGTGATGAATTTGCTACAGGAACATTTTTTTTTGATTGTAAACCA
TGTAGACTAACACATACATGGCAAACAAATAGAGCATTGGGCTTACCACCATTTCTAAATTCTTTGCC
TCAATCTGAAGGAGCTACTAACTTTGGTGATATAGGAGTTCAACAAGATAAAAGACGTGGTGTAACTC
AAATGGGAAATACAAACTATATTACTGAAGCTACTATTATGAGACCAGCTGAGGTTGGTTATAGTGCA
CCATATTATTCTTTTGAGGCGTCTACACAAGGGCCATTTAAAACACCTATTGCAGCAGGACGGGGGGG
AGCGCAAACATATGAAAATCAAGCAGCAGATGGTGATCCAAGATATGCATTTGGTAGACAACATGGTC
AAAAAACTACCACAACAGGAGAAACACCTGAGAGATTTACATATATAGCACATCAAGATACAGGAAGA
TATCCAGAAGGAGATTGGATTCAAAATATTAACTTTAACCTTCCTGTAACGAATGATAATGTATTGCT
ACCAACAGATCCAATTGGAGGTAAAACAGGAATTAACTATACTAATATATTTAATACTTATGGTCCTT
TAACTGCATTAAATAATGTACCACCAGTTTATCCAAATGGTCAAATTTGGGATAAAGAATTTGATACT
GACTTAAAACCAAGACTTCATGTAAATGCACCATTTGTTTGTCAAAATAATTGTCCTGGTCAATTATT
TGTAAAAGTTGCGCCTAATTTAACAAATGAATATGATCCTGATGCATCTGCTAATATGTCAAGAATTG
TAACTTACTCAGATTTTTGGTGGAAAGGTAAATTAGTATTTAAAGCTAAACTAAGAGCCTCTCATACT

TGGAATCCAATTCAACAAATGAGTATTAATGTAGATAACCAATTTAACTATGTACCAAGTAATATTGG
AGGTATGAAAATTGTATATGAAAAATCTCAACTAGCACCTAGA
'USA_M23255_FPV_Cornell320_1988'
ATGAGTGATGGAGCAGTTCAACCAGACGGTGGTCAACCTGCTGTCAGAAATGAAAGAGCTACAGGATC
TGGGAACGGGTCTGGAGGCGGGGGTGGTGGTGGTTCTGGGGGTGTGGGGATTTCTACGGGTACTTTCA
ATAATCAGACGGAATTTAAATTTTTGGAAAACGGATGGGTGGAAATCACAGCAAACTCAAGCAGACTT
GTACATTTAAATATGCCAGAAAGTGAAAATTATAGAAGAGTGGTTGTAAATAATATGGATAAAACTGC
AGTTAACGGAAACATGGCTTTAGATGATATTCATGCACAAATTGTAACACCTTGGTCATTGGTTGATG
CAAATGCTTGGGGAGTTTGGTTTAATCCAGGAGATTGGCAACTAATTGTTAATACTATGAGTGAGTTG
CATTTAGTTAGTTTTGAACAAGAAATTTTTAATGTTGTTTTAAAGACTGTTTCAGAATCTGCTACTCA
GCCACCAACTAAAGTTTATAATAATGATTTAACTGCATCATTGATGGTTGCATTAGATAGTAATAATA
CTATGCCATTTACTCCAGCAGCTATGAGATCTGAGACATTGGGTTTTTATCCATGGAAACCAACCATA
CCAACTCCATGGAGATATTATTTTCAATGGGATAGAACATTAATACCATCTCATACTGGAACTAGTGG
CACACCAACAAATATATACCATGGTACAGATCCAGATGATGTTCAATTTTATACTATTGAAAATTCTG
TGCCAGTACACTTACTAAGAACAGGTGATGAATTTGCTACAGGAACATTTTTTTTTGATTGTAAACCA
TGTAGACTAACACATACATGGCAAACAAATAGAGCATTGGGCTTACCACCATTTCTAAATTCTTTGCC
TCAATCTGAAGGAGCTACTAACTTTGGTGATATAGGAGTTCAACAAGATAAAAGACGTGGTGTAACTC
AAATGGGAAATACAAACTATATTACTGAAGCTACTATTATGAGACCAGCTGAGGTTGGTTATAGTGCA
CCATATTATTCTTTTGAGGCGTCTACACAAGGGCCATTTAAAACACCTATTGCAGCAGGACGGGGGGG
AGCGCAAACAGATGAAAATCAAGCAGCAGATGGTAATCCAAGATATGCATTTGGTAGACAACATGGTC
AAAAAACTACCACAACAGGAGAAACACCTGAGAGATTTACATATATAGCACATCAAGATACAGGAAGA
TATCCAGAAGGAGATTGGATTCAAAATATTAACTTTAACCTTCCTGTAACAAATGATAATGTATTGCT
ACCAACAGATCCAATTGGAGGTAAAACAGGAATTAACTATACTAATATATTTAATACTTATGGTCCTT
TAACTGCATTAAATAATGTACCACCAGTTTATCCAAATGGTCAAATTTGGGATAAAGAATTTGATACT
GACTTAAAACCAAGACTTCATGTAAATGCACCATTTGTTTGTCAAAATAATTGTCCTGGTCAATTATT
TGTAAAAGTTGCGCCTAATTTAACAAATGAATATGATCCTGATGCATCTGCTAATATGTCAAGAATTG
TAACTTACTCAGATTTTTGGTGGAAAGGTAAATTAGTATTTAAAGCTAAACTAAGAGCCTCTCATACT
TGGAATCCAATTCAACAAATGAGTATTAATGTAGATAACCAATTTAACTATGTACCAAGTAATATTGG
AGGTATGAAAATTGTATATGAAAAATCTCAACTAGCACCTAGA
'USA_M24000_FPV_CPV_31_1988'
ATGAGTGATGGAGCAGTTCAACCAGACGGTGGTCAACCTGCTGTCAGAAATGAAAGAGCTACAGGATC
TGGGAACGGGTCTGGAGGCGGGGGTGGTGGTGGTTCTGGGGGTGTGGGGATTTCTACGGGTACTTTCA
ATAATCAGACGGAATTTAAATTTTTGGAAAACGGATGGGTGGAAATCACAGCAAACTCAAGCAGACTT
GTACATTTAAATATGCCAGAAAGTGAAAATTATAGAAGAGTGGTTGTAAATAATTTGGATAAAACTGC
AGTTAACGGAAACATGGCTTTAGATGATACTCATGCACAAATTGTAACACCTTGGTCATTGGTTGATG
CAAATGCTTGGGGAGTTTGGTTTAATCCAGGAGATTGGCAACTAATTGTTAATACTATGAGTGAGTTG
CATTTAGTTAGTTTTGAACAAGAAATTTTTAATGTTGTTTTAAAGACTGTTTCAGAATCTGCTACTCA
GCCACCAACTAAAGTTTATAATAATGATTTAACTGCATCATTAATGGTTGCATTAGATAGTAATAATA
CTATGCCATTTACTCCAGCAGCTATGAGATCTGAGACATTGGGTTTTTATCCATGGAAACCAACCATA
CCAACTCCATGGAGATATTATTTTCAATGGGATAGAACATTAATACCATCTCATACTGGAACTAGTGG
CACACCAACAAATATATACCATGGTACAGATCCAGATGATGTTCAATTTTATACTATTGAAAATTCTG
TGCCAGTACACTTACTAAGAACAGGTGATGAATTTGCTACAGGAACATTTTTTTTTGATTGTAAACCA
TGTAGACTAACACATACATGGCAAACAAATAGAGCATTGGGCTTACCACCATTTCTAAATTCTTTGCC
TCAATCTGAAGGAGGTACTAACTTTGGTTATATAGGAGTTCAACAAGATAAAAGACGTGGTGTAACTC
AAATGGGAAATACAAACTATATTACTGAAGCTACTATTATGAGACCAGCTGAGGTTGGTTATAGTGCA
CCATATTATTCTTTTGAGGCGTCTACACAAGGGCCATTTAAAACACCTATTGCAGCAGGACGGGGGGG
AGCGCAAACAGATGAAAATCAAGCAGCAGATGGTGATCCAAGATATGCATTTGGTAGACAACATGGTC
AAAAAACTACCACAACAGGAGAAACACCTGAGAGATTTACATATATAGCACATCAAGATACAGGAAGA
TATCCAGAAGGAGATTGGATTCAAAATATTAACTTTAACCTTCCTGTAACAAATGATAATGTATTGCT
ACCAACAGATCCAATTGGAGGTAAAACAGGAATTAACTATACTAATATATTTAATACTTATGGTCCTT
TAACTGCATTAAATAATGTACCACCAGTTTATCCAAATGGTCAAATTTGGGATAAAGAATTTGATACT
GACTTAAAACCAAGACTTCATGTAAATGCACCATTTGTTTGTCAAAATAATTGTCCTGGTCAATTATT
TGTAAAAGTTGCGCCTAATTTAACAAATGAATATGATCCTGATGCATCTGCTAATATGTCAAGAATTG
TAACTTACTCAGATTTTTGGTGGAAAGGTAAATTAGTATTTAAAGCTAAACTAAGAGCCTCTCATACT

TGGAATCCAATTCAACAAATGAGTATTAATATAGATAACCAATTTAACTATGTACCAAGTAATATTGG
AGGTATGAAAATTGTATATGAAAAATCTCAACTAGCACCTAGA
'USA_M24003_FPV_CPV_15_1988'
ATGAGTGATGGAGCAGTTCAACCAGACGGTGGTCAACCTGCTGTCAGAAATGAAAGAGCTACAGGATC
TGGGAACGGGTCTGGAGGCGGGGGTGGTGGTGGTTCTGGGGGTGTGGGGATTTCTACGGGTACTTTCA
ATAATCAGACGGAATTTAAATTTTTGGAAAACGGATGGGTGGAAATCACAGCAAACTCAAGCAGACTT
GTACATTTAAATATGCCAGAAAGTGAAAATTATAGAAGAGTGGTTGTAAATAATTTGGATAAAACTGC
AGTTAACGGAAACATGGCTTTAGATGATACTCATGCACAAATTGTAACACCTTGGTCATTGGTTGATG
CAAATGCTTGGGGAGTTTGGTTTAATCCAGGAGATTGGCAACTAATTGTTAATACTATGAGTGAGTTG
CATTTAGTTAGTTTTGAACAAGAAATTTTTAATGTTGTTTTAAAGACTGTTTCAGAATCTGCTACTCA
GCCACCAACTAAAGTTTATAATAATGATTTAACTGCATCATTGATGGTTGCATTAGATAGTAATAATA
CTATGCCATTTACTCCAGCAGCTATGAGATCTGAGACATTGGGTTTTTATCCATGGAAACCAACCATA
CCAACTCCATGGAGATATTATTTTCAATGGGATAGAACATTAATACCATCTCATACTGGAACTAGTGG
CACACCAACAAATATATACCATGGTACAGATCCAGATGATGTTCAATTTTATACTATTGAAAATTCTG
TGCCAGTACACTTACTAAGAACAGGTGATGAATTTGCTACAGGAACATTTTTTTTTGATTGTAAACCA
TGTAGACTAACACATACATGGCAAACAAATAGAGCATTGGGCTTACCACCATTTCTAAATTCTTTGCC
TCAATCTGAAGGAGGTACTAACTTTGGTTATATAGGAGTTCAACAAGATAAAAGACGTGGTGTAACTC
AAATGGGAAATACAAACTATATTACTGAAGCTACTATTATGAGACCAGCTGAGGTTGGTTATAGTGCA
CCATATTATTCTTTTGAGGCGTCTACACAAGGGCCATTTAAAACACCTATTGCAGCAGGACGGGGGGG
AGCGCAAACAGATGAAAATCAAGCAGCAGATGGTGATCCAAGATATGCATTTGGTAGACAACATGGTC
AAAAAACTACCACAACAGGAGAAACACCTGAGAGATTTACATATATAGCACATCAAGATACAGGAAGA
TATCCAGAAGGAGATTGGATTCAAAATATTAACTTTAACCTTCCTGTAACAAATGATAATGTATTGCT
ACCAACAGATCCAATTGGAGGTAAAACAGGAATTAACTATACTAATATATTTAATACTTATGGTCCTT
TAACTGCATTAAATAATGTACCACCAGTTTATCCAAATGGTCAAATTTGGGATAAAGAATTTGATACT
GACTTAAAACCAAGACTTCATGTAAATGCACCATTTGTTTGTCAAAATAATTGTCCTGGTCAATTATT
TGTAAAAGTTGCGCCTAATTTAACAAATGAATATGATCCTGATGCATCTGCTAATATGTCAAGAATTG
TAACTTACTCAGATTTTTGGTGGAAAGGTAAATTAGTATTTAAAGCTAAACTAAGAGCCTCTCATACT
TGGAATCCAATTCAACAAATGAGTATTAATATAGATAACCAATTTAACTATGTACCAAGTAATATTGG
AGGTATGAAAATTGTATATGAAAAATCTCAACTAGCACCTAGA
'USA_M38245_1990'
ATGAGTGATGGAGCAGTTCAACCAGACGGTGGTCAACCTGCTGTCAGAAATGAAAGAGCTACAGGATC
TGGGAACGGGTCTGGAGGCGGGGGTGGTGGTGGTTCTGGGGGTGTGGGGATTTCTACGGGTACTTTCA
ATAATCAGACGGAATTTAAATTTTTGGAAAACGGATGGGTGGAAATCACAGCAAACTCAAGCAGACTT
GTACATTTAAATATGCCAGAAAGTGAAAATTATAGAAGAGTGGTTGTAAATAATATGGATAAAACTGC
AGTTAACGGAAACATGGCTTTAGATGATATTCATGCACAAATTGTAACACCTTGGTCATTGGTTGATG
CAAATGCTTGGGGAGTTTGGTTTAATCCAGGAGATTGGCAACTAATTGTTAATACTATGAGTGAGTTG
CATTTAGTTAGTTTTGAACAAGAAATTTTTAATGTTGTTTTAAAGACTGTTTCAGAATCTGCTACTCA
GCCACCAACTAAAGTTTATAATAATGATTTAACTGCATCATTGATGGTTGCATTAGATAGTAATAATA
CTATGCCATTTACTCCAGCAGCTATGAGATCTGAGACATTGGGTTTTTATCCATGGAAACCAACCATA
CCAACTCCATGGAGATATTATTTTCAATGGGATAGAACATTAATACCATCTCATACTGGAACTAGTGG
CACACCAACAAATATATACCATGGTACAGATCCAGATGATGTTCAATTTTATACTATTGAAAATTCTG
TGCCAGTACACTTACTAAGAACAGGTGATGAATTTGCTACAGGAACATTTTTTTTTGATTGTAAACCA
TGTAGACTAACACATACATGGCAAACAAATAGAGCATTGGGCTTACCACCATTTCTAAATTCTTTGCC
TCAATCTGAAGGAGCTACTAACTTTGGTGATATAGGAGTTCAACAAGATAAAAGACGTGGTGTAACTC
AAATGGGAAATACAAACTATATTACTGAAGCTACTATTATGAGACCAGCTGAGGTTGGTTATAGTGCA
CCATATTATTCTTTTGAGGCGTCTACACAAGGGCCATTTAAAACACCTATTGCAGCAGGACGGGGGGG
AGCGCAAACAGATGAAAATCAAGCAGCAGATGGTAATCCAAGATATGCATTTGGTAGACAACATGGTC
AAAAAACTACCACAACAGGAGAAACACCTGAGAGATTTACATATATAGCACATCAAGATACAGGAAGA
TATCCAGAAGGAGATTGGATTCAAAATATTAACTTTAACCTTCCTGTAACAAATGATAATGTATTGCT
ACCAACAGATCCAATTGGAGGTAAAACAGGAATTAACTATACTAATATATTTAATACTTATGGTCCTT
TAACTGCATTAAATAATGTACCACCAGTTTATCCAAATGGTCAAATTTGGGATAAAGAATTTGATACT
GACTTAAAACCAAGACTTCATGTAAATGCACCATTTGTTTGTCAAAATAATTGTCCTGGTCAATTATT
TGTAAAAGTTGCGCCTAATTTAACAAATGAATATGATCCTGATGCATCTGCTAATATGTCAAGAATTG
TAACTTACTCAGATTTTTGGTGGAAAGGTAAATTAGTATTTAAAGCTAAACTAAGAGCCTCTCATACT

TGGAATCCAATTCAACAAATGAGTATTAATGTAGATAACCAATTTAACTATGTACCAAGTAATATTGG
AGGTATGAAAATTGTATATGAAAAATCTCAACTAGCACCTAGA
'USA_M74849_39_1995'
ATGAGTGATGGAGCAGTTCAACCAGACGGTGGTCAACCTGCTGTCAGAAATGAAAGAGCTACAGGATC
TGGGAACGGGTCTGGAGGCGGGGGTGGTGGTGGTTCTGGGGGTGTGGGGATTTCTACGGGTACTTTCA
ATAATCAGACGGAATTTAAATTTTTGGAAAACGGATGGGTGGAAATCACAGCAAACTCAAGCAGACTT
GTACATTTAAATATGCCAGAAAGTGAAAATTATAGAAGAGTGGTTGTAAATAATTTGGATAAAACTGC
AGTTAATGGAAACATGGCTTTAGATGATACTCATGCACAAATTGTAACACCTTGGTCATTGGTTGATG
CAAATGCTTGGGGAGTTTGGTTTAATCCAGGAGATTGGCAACTAATTGTTAATACTATGAGTGAGTTG
CATTTAGTTAGTTTTGAACAAGAAATTTTTAATGTTGTTTTAAAGACTGTTTCAGAATCTGCTACTCA
GCCACCAACTAAAGTTTATAATAATGATTTAACTGCATCATTGATGGTTGCATTAGATAGTAATAATA
CTATGCCATTTACTCCAGCAGCTATGAGATCTGAGACATTGGGTTTTTATCCATGGAAACCAACCATA
CCAACTCCATGGAGATATTATTTTCAATGGGATAGAACATTAATACCATCTCATACTGGAACTAGTGG
CACACCAACAAATATATACCATGGTACAGATCCAGATGATGTTCAATTTTATACTATTGAAAATTCTG
TGCCAGTACACTTACTAAGAACAGGTGATGAATTTGCTACAGGAACATTTTTTTTTGATTGTAAACCA
TGTAGACTAACACATACATGGCAAACAAATAGAGCATTGGGCTTACCACCATTTCTAAATTCTTTGCC
TCAATCTGAAGGAGGTACTAACTTTGGTTATATAGGAGTTCAACAAGATAAAAGACGTGGTGTAACTC
AAATGGGAAATACAAACTATATTACTGAAGCTACTATTATGAGACCAGCTGAGGTTGGTTATAGTGCA
CCATATTATTCTTTTGAGGCGTCTACACAAGGGCCATTTAAAACACCTATTGCAGCAGGACGGGGGGG
AGCGCAAACAGATGAAAATCAAGCAGCAGATGGTGATCCAAGATATGCATTTGGTAGACAACATGGTC
AAAAAACTACCACAACAGGAGAAACACCTGAGAGATTTACATATATAGCACATCAAGATACAGGAAGA
TATCCAGAAGGAGATTGGATTCAAAATATTAACTTTAACCTTCCTGTAACAGATGATAATGTATTGCT
ACCAACAGATCCAATTGGAGGTAAAACAGGAATTAACTATACTAATATATTTAATACTTATGGTCCTT
TAACTGCATTAAATAATGTACCACCAGTTTATCCAAATGGTCAAATTTGGGATAAAGAATTTGATACT
GACTTAAAACCAAGACTTCATGTAAATGCACCATTTGTTTGTCAAAATAATTGTCCTGGTCAATTATT
TGTAAAAGTTGCGCCTAATTTAACAAATGAATATGATCCTGATGCATCTGCTAATATGTCAAGAATTG
TAACTTACTCAGATTTTTGGTGGAAAGGTAAATTAGTATTTAAAGCTAAACTAAGAGCCTCTCATACT
TGGAATCCAATTCAACAAATGAGTATTAATGTAGATAACCAATTTAACTATGTACCAAGTAATATTGG
AGGTATGAAAATTGTATATGAAAAATCTCAACTAGCACCTAGA
'USA_M74852_133_1995'
ATGAGTGATGGAGCAGTTCAACCAGACGGTGGTCAACCTGCTGTCAGAAATGAAAGAGCTACAGGATC
TGGGAACGGGTCTGGAGGCGGGGGTGGTGGTGGTTCTGGGGGTGTGGGGATTTCTACGGGTACTTTCA
ATAATCAGACGGAATTTAAATTTTTGGAAAACGGATGGGTGGAAATCACAGCAAACTCAAGCAGACTT
GTACATTTAAATATGCCAGAAAGTGAAAATTATAGAAGAGTGGTTGTAAATAATTTGGATAAAACTGC
AGTTAACGGAAACATGGCTTTAGATGATACTCATGCACAAATTGTAACACCTTGGTCATTGGTTGATG
CAAATGCTTGGGGAGTTTGGTTTAATCCAGGAGATTGGCAACTAATTGTTAATACTATGAGTGAGTTG
CATTTAGTTAGTTTTGAACAAGAAATTTTTAATGTTGTTTTAAAGACTGTTTCAGAATCTGCTACTCA
GCCACCAACTAAAGTTTATAATAATGATTTAACTGCATCATTGATGGTTGCATTAGATAGTAATAATA
CTATGCCATTTACTCCAGCAGCTATGAGATCTGAGACATTGGGTTTTTATCCATGGAAACCAACCATA
CCAACTCCATGGAGATATTATTTTCAATGGGATAGAACATTAATACCATCTCATACTGGAACTAGTGG
CACACCAACAAATATATACCATGGTACAGATCCAGATGATGTTCAATTTTATACTATTGAAAATTCTG
TGCCAGTACACTTACTAAGAACAGGTGATGAATTTGCTACAGGAACATTTTTTTTTGATTGTAAACCA
TGTAGACTAACACATACATGGCAAACAAATAGAGCATTGGGCTTACCACCATTTCTAAATTCTTTGCC
TCAATCTGAAGGAGGTACTAACTTTGGTTATATAGGAGTTCAACAAGATAAAAGACGTGGTGTAACTC
AAATGGGAAATACAAACTATATTACTGAAGCTACTATTATGAGACCAGCTGAGGTTGGTTATAGTGCA
CCATATTATTCTTTTGAGGCGTCTACACAAGGGCCATTTAAAACACCTATTGCAGCAGGACGGGGGGG
AGCGCAAACAGATGAAAATCAAGCAGCAGATGGTGATCCAAGATATGCATTTGGTAGACAACATGGTC
AAAAAACTACCACAACAGGAGAAACACCTGAGAGATTTACATATATAGCACATCAAGATACAGGAAGA
TATCCAGAAGGAGATTGGATTCAAAATATTAACTTTAACCTTCCTGTAACAGATGATAATGTATTGCT
ACCAACAGATCCAATTGGAGGTAAAACAGGAATTAACTATACTAATATATTTAATACTTATGGTCCTT
TAACTGCATTAAATAATGTACCACCAGTTTATCCAAATGGTCAAATTTGGGATAAAGAATTTGATACT
GACTTAAAACCAAGACTTCATGTAAATGCACCATTTGTTTGTCAAAATAATTGTCCTGGTCAATTATT
TGTAAAAGTTGCGCCTAATTTAACAAATGAATATGATCCTGATGCATCTGCTAATATGTCAAGAATTG
TAACTTACTCAGATTTTTGGTGGAAAGGTAAATTAGTATTTAAAGCTAAACTAAGAGCCTCTCATACT

TGGAATCCAATTCAACAAATGAGTATTAATGTAGATAACCAATTTAACTATGTACCAAGTAATATTGG
AGGTATGAAAATTGTATATGAAAAATCTCAACTAGCACCTAGA
'USA_U22186_CPV_128_1995'
ATGAGTGATGGAGCAGTTCAACCAGACGGTGGTCAACCTGCTGTCAGAAATGAAAGAGCTACAGGATC
TGGGAACGGGTCTGGAGGCGGGGGTGGTGGTGGTTCTGGGGGTGTGGGGATTTCTACGGGTACTTTCA
ATAATCAGACGGAATTTAAATTTTTGGAAAACGGATGGGTGGAAATCACAGCAAACTCAAGCAGACTT
GTACATTTAAATATGCCAGAAAGTGAAAATTATAGAAGAGTGGTTGTAAATAATATGGATAAAACTGC
AGTTAACGGAAACATGGCTTTAGATGATATTCATGCACAAATTGTAACACCTTGGTCATTGGTTGATG
CAAATGCTTGGGGAGTTTGGTTTAATCCAGGAGATTGGCAACTAATTGTTAATACTATGAGTGAGTTG
CATTTAGTTAGTTTTGAACAAGAAATTTTTAATGTTGTTTTAAAGACTGTTTCAGAATCTGCTACTCA
GCCACCAACTAAAGTTTATAATAATGATTTAACTGCATCATTGATGGTTGCATTAGATAGTAATAATA
CTATGCCATTTACTCCAGCAGCTATGAGATCTGAGACATTGGGTTTTTATCCATGGAAACCAACCATA
CCAACTCCATGGAGATATTATTTTCAATGGGATAGAACATTAATACCATCTCATACTGGAACTAGTGG
CACACCAACAAATATATACCATGGTACAGATCCAGATGATGTTCAATTTTATACTATTGAAAATTCTG
TGCCAGTACACTTACTAAGAACAGGTGATGAATTTGCTACAGGAACATTTTTTTTTGATTGTAAACCA
TGTAGACTAACACATACATGGCAAACAAATAGAGCATTGGGCTTACCACCATTTCTAAATTCTTTGCC
TCAATCTGAAGGAGCTACTAACTTTGGTGATATAGGAGTTCAACAAGATAAAAGACGTGGTGTAACTC
AAATGGGAAATACAAACTATATTACTGAAGCTACTATTATGAGACCAGCTGAGGTTGGTTATAGTGCA
CCATATTATTCTTTTGAGGCGTCTACACAAGGGCCATTTAAAACACCTATTGCAGCAGGACGGGGGGG
AGCGCAAACAGATGAAAATCAAGCAGCAGATGGTAATCCAAGATATGCATTTGGTAGACAACATGGTA
AAAAAACTACCACAACAGGAGAAACACCTGAGAGATTTACATATATAGCACATCAAGATACAGGAAGA
TATCCAGAAGGAGATTGGATTCAAAATATTAACTTTAACCTTCCTGTAACAAATGATAATGTATTGCT
ACCAACAGATCCAATTGGAGGTAAAACAGGAATTAACTATACTAATATATTTAATACTTATGGTCCTT
TAACTGCATTAAATAATGTACCACCAGTTTATCCAAATGGTCAAATTTGGGATAAAGAATTTGATACT
GACTTAAAACCAAGACTTCATGTAAATGCACCATTTGTTTGTCAAAATAATTGTCCTGGTCAATTATT
TGTAAAAGTTGCGCCTAATTTAACAAATGAATATGATCCTGATGCATCTGCTAATATGTCAAGAATTG
TAACTTACTCAGATTTTTGGTGGAAAGGTAAATTAGTATTTAAAGCTAAACTAAGAGCCTCTCATACT
TGGAATCCAATTCAACAAATGAGTATTAATGTAGATAACCAATTTAACTATGTACCAAGTAATATTGG
AGGTATGAAAATTGTATATGAAAAATCTCAACTAGCACCTAGA
'FIN_U22192_raccoondog_RD_80_1980'
ATGAGTGATGGAGCAGTTCAACCAGACGGTGGTCAACCTGCTGTCAGAAATGAAAGAGCTACAGGATC
TGGGAACGGGTCTGGAGGCGGGGGTGGTGGTGGTTCTGGGGGTGTGGGGATTTCTACGGGTACTTTCA
ATAATCAGACGGAATTTAAATTTTTGGAAAACGGATGGGTGGAAATCACAGCAAACTCAAGCAGACTT
GTACATTTAAATATGCCAGAAAGTGAAAATTATAGAAGAGTGGTTGTAAATAATATGGATAAAACTGC
AGTTAACGGAAACATGGCTTTAGATGATATTCATGCACAAATTGTAACACCTTGGTCATTGGTTGATG
CAAATGCTTGGGGAGTTTGGTTTAATCCAGGAGATTGGCAACTAATTGTTAATACTATGAGTGAGTTG
CATTTAGTTAGTTTTGAACAAGAAATTTTTAATGTTGTTTTAAAGACTGTTTCAGAATCTGCTACTCA
GCCACCAACTAAAGTTTATAATAATGATTTAACTGCATCATTGATGGTTGCATTAGATAGTAATAATA
CTATGCCATTTACTCCAGCAGCTATGAGATCTGAGACATTGGGTTTTTATCCATGGAAACCAACCATA
CCAACTCCATGGAGATATTATTTTCAATGGGATAGAACATTAATACCATCTCATACTGGAACTAGTGG
CACACCAACAAATATATACCATGGTACAGATCCAGATGATGTTCAATTTTATACTATTGAAAATTCTG
TGCCAGTACACTTACTAAGAACAGGTGATGAATTTGCTACAGGAACATTTTTTTTTGATTGTAAACCA
TGTAGACTAACACATACATGGCAAACAAATAGAGCATTGGGCTTACCACCATTTCTAAATTCTTTGCC
TCAATCTGAAGGAGCTACTAACTTTGGTGATATAGGAGTTCAACAAGATAAAAGACGTGGTGTAACTC
AAATGGGAAATACAAACTATATTACTGAAGCTACTATTATGAGACCAGCTGAGGTTGGTTATAGTGCA
CCATATTATTCTTTTGAGGCGTCTACACAAGGGCCATTTAAAACACCTATTGCAGCAGGACGGGGGGG
AGCGCAAACAGATGAAAATCAAGCAGCAGATGGTAATCCAAGATATGCATTTGGTAGACAACATGGTC
AAAAAACTACCACAACAGGAGAAACACCTGAGAGATTTACATATATAGCACATCAAGATACAGGAAGA
TATCCAGAAGGAGATTGGATTCAAAATATTAACTTTAACCTTCCTGTAACAAATGATAATGTATTGCT
ACCAACAGATCCAATTGGAGGTAAAACAGGAATTAACTATACTAATATATTTAATACTTATGGTCCTT
TAACTGCATTAAATAATGTACCACCAGTTTATCCAAATGGTCAAATTTGGGATAAAGAATTTGATACT
GACTTAAAACCAAGACTTCATGTAAATGCACCATTTGTTTGTCAAAATAATTGTCCTGGTCAATTATT
TGTAAAAGTTGCGCCTAATTTAACAAATGAATATGATCCTGATGCATCTGCTAATATGTCAAGAATTG
TAACTTACTCAGATTTTTGGTGGAAAGGTAAATTAGTATTTAAAGCTAAACTAAGAGCCTCTCATACT

TGGAATCCAATTCAACAAATGAGTATTAATGTAGATAACCAATTTAACTATGTACCAAGTAATATTGG
AGGTATGAAAATTGTATATGAAAAATCTCAACTAGCACCTAGA

'FIN_U22193_raccoondog_RD87_1987'

ATGAGTGATGGAGCAGTTCAACCAGACGGTGGTCAACCTGCTGTCAGAAATGAAAGAGCTACAGGATC
TGGGAACGGGTCTGGAGGCGGGGGTGGTGGTGGTTCTGGGGGTGTGGGGATTTCTACGGGTACTTTCA
ATAATCAGACGGAATTTAAATTTTTGGAAAACGGATGGGTGGAAATCACAGCAAACTCAAGCAGACTT
GTACATTTAAATATGCCAGAAAGTGAAAATTATAGAAGAGTGGTTGTAAATAATATGGATAAAACTGC
AGTTAACGGAAACATGGCTTTAGATGATATTCATGCACAAATTGTAACACCTTGGTCATTGGTTGATG
CAAATGCTTGGGGGGTTTGGTTTAATCCAGGAGATTGGCAACTAATTGTTAATACTATGAGTGAGTTG
CATTTAGTTAGTTTTGAACAAGAAATTTTTAATGTTGTTTTAAAGACTGTTTCAGAATCTGCTACTCA
GCCACCAACTAAAGTTTATAATAATGATTTAACTGCATCATTGATGGTTGCATTAGATAGTAATAATA
CTATGCCATTTACTCCAGCAGCTATGAGATCTGAGACATTGGGTTTTTATCCATGGAAACCAACCATA
CCAACTCCATGGAGATATTATTTTCAATGGGATAGAACATTAATACCATCTCATACTGGAACTAGTGG
CACACCAACAAATATATACCATGGTACAGATCCAGATGATGTTCAATTTTATACTATTGAAAATTCTG
TGCCAGTACACTTACTAAGAACAGGTGATGAATTTGCTACAGGAACATTTTTTTTTGATTGTAAACCA
TGTAGACTAACACATACATGGCAAACAAATAGAGCATTGGGCTTACCACCATTTCTAAATTCTTTGCC
TCAATCTGAAGGAGCTACTAACTTTGGTGATATAGGAGTTCAACAAGATAAAAGACGTGGTGTAACTC
AAATGGGAAATACAAACTATATTACTGAAGCTACTATTATGAGACCAGCTGAGGTTGGTTATAGTGCA
CCATATTATTCTTTTGAGGCGTCTACACAAGGGCCATTTAAAACACCTATTGCAGCAGGACGGGGGGG
AGCGCAAACAGATGAAAATCAAGCAGCAGATGGTAATCCAAGATATGCATTTGGTAGACAACATGGTC
AAAAAACTACCACAACAGGAGAAACACCTGAGAGATTTACATATATAGCACATCAAGATACAGGAAGA
TATCCAGAAGGAGATTGGATTCAAAATATTAACTTTAACCTTCCTGTAACAAATGATAATGTATTGCT
ACCAACAGATCCAATTGGAGGTAAAACAGGAATTAACTATACTAATATATTTAATACTTATGGTCCTT
TAACTGCATTAAATAATGTACCACCAGTTTATCCAAATGGTCAAATTTGGGATAAAGAATTTGATACT
GACTTAAAACCAAGACTTCATGTAAATGCACCATTTGTTTGTCAAAATAATTGTCCTGGTCAATTATT
TGTAAAAGTTGCGCCTAATTTAACAAATGAATATGATCCTGATGCATCTGCTAATATGTCAAGAATTG
TAACTTACTCAGATTTTTGGTGGAAAGGTAAATTAGTATTTAAAGCTAAACTAAGAGCATCTCATACT
TGGAATCCAATTCAACAAATGAGTATTAATGTAGATAACCAATTTAACTATGTACCAAGTAATATTGG
AGGTATGAAAATTGTATATGAAAAATCTCAACTAGCACCTAGA

'USA_U22896_cat_1990'

ATGAGTGATGGAGCAGTTCAACCAGACGGTGGTCAACCTGCTGTCAGAAATGAAAGAGCTACAGGATC
TGGGAACGGGTCTGGAGGCGGGGGTGGTGGTGGTTCTGGGGGTGTGGGGATTTCTACGGGTACTTTCA
ATAATCAGACGGAATTTAAATTTTTGGAAAACGGATGGGTGGAAATCACAGCAAACTCAAGCAGACTT
GTACATTTAAATATGCCAGAAAGTGAAAATTATAGAAGAGTGGTTGTAAATAATTTGGATAAAACTGC
AGTTAATGGAAACATGGCTTTAGATGATACTCATGCACAAATTGTAACACCTTGGTCATTGGTTGATG
CAAATGCTTGGGGAGTTTGGTTTAATCCAGGAGATTGGCAACTAATTGTTAATACTATGAGTGAGTTG
CATTTAGTTAGTTTTGAACAAGAAATTTTTAATGTTGTTTTAAAGACTGTTTCAGAATCTGCTACTCA
GCCACCAACTAAAGTTTATAATAATGATTTAACTGCATCATTGATGGTTGCATTAGATAGTAATAATA
CTATGCCATTTACTCCAGCAGCTATGAGATCTGAGACATTGGGTTTTTATCCATGGAAACCAACCATA
CCAACTCCATGGAGATATTATTTTCAATGGGATAGAACATTAATACCATCTCATACTGGAACTAGTGG
CACACCAACAAATATATACCATGGTACAGATCCAGATGATGTTCAATTTTATACTATTGAAAATTCTG
TGCCAGTACACTTACTAAGAACAGGTGATGAATTTGCTACAGGAACATTTTTTTTTGATTGTAAACCA
TGTAGACTAACACATACATGGCAAACAAATAGAGCATTGGGCTTACCACCATTTCTAAATTCTTTGCC
TCAATCTGAAGGAGGTACTAACTTTGGTTATATAGGAGTTCAACAAGATAAAAGACGTGGTGTAACTC
AAATGGGAAATACAAACTATATTACTGAAGCTACTATTATGAGACCAGCTGAGGTTGGTTATAGTGCA
CCATATTATTCTTTTGAGGCGTCTACACAAGGACCATTTAAAACACCTATTGCAGCAGGACGGGGGGG
AGCGCAAACAGATGAAAATCAAGCAGCAGATGGTGATCCAAGATATGCATTTGGTAGACAACATGGTC
AAAAAACTACCACAACAGGAGAAACACCTGAGAGATTTACATATATAGCACATCAAGATACAGGAAGA
TATCCAGAAGGAGATTGGATTCAAAATATTAACTTTAACCTTCCTGTAACAGATGATAATGTATTGCT
ACCAACAGATCCAATTGGAGGTAAAACAGGAATTAACTATACTAATATATTTAATACTTATGGTCCTT
TAACTGCATTAAATAATGTACCACCAGTTTATCCAAATGGTCAAATTTGGGATAAAGAATTTGATACT
GACTTAAAACCAAGACTTCATGTAAATGCACCATTTGTTTGTCAAAATAATTGTCCTGGTCAATTATT
TGTAAAAGTTGCGCCTAATTTAACAAATGAATATGATCCTGATGCATCTGCTAATATGTCAAGAATTG
TAACTTACTCAGATTTTTGGTGGAAAGGTAAATTAGTATTTAAAGCTAAACTAAGAGCCTCTCATACT

TGGAATCCAATTCAACAAATGAGTATTAATGTAGATAACCAATTTAACTATGTACCAAGTAATATTGG
AGGTATGAAAATTGTATATGAAAAATCTCAACTAGCACCTAGA
'TAW_U72695_2a_T4_1996'
ATGAGTGATGGAGCAGTTCAACCAGACGGTGGTCAACCTGCTGTCAGAAATGAAAGAGCTACAGGATC
TGGGAACGGGTCTGGAGGCGGGGGTGGTGGTGGTTCTGGGGGTGTGGGGATTTCTACGGGTACTTTCA
ATAATCAGACAGAATTTAAATTTTTGGAAAACGGATGGGTGGAAATCACAGCAAACTCAAGCAGACTT
GTACATTTAAATATGCCAGAAAGTGAAAATTATAGAAGAGTGGTTGTAAATAATTTGGATAAAACTGC
AGTTAACGGAAACATGGCTTTAGATGATACCCATGCACAAATTGTAACACCTTGGTCATTGGTTGATG
CAAATGCTTGGGGAGTTTGGTTTAATCCAGGAGATTGGCAACTAATTGTTAATACTATGAGTGAGTTG
CATTTAGTTAGTTTTGAACAAGAAATTTTTAATGTTGTTTTAAAGACTGTTTCAGAATCTGCTACTCA
GCCACCAACTAAAGTTTATAATAATGATTTAACTGCATCATTGATGGTTGCATTAGATAGTAATAATA
CTATGCCATTTACTCCAGCAGCTATGAGATCTGAGACATTGGGTTTTTATCCATGGAAACCAACCATA
CCAACTCCATGGAGATATTATTTTCAATGGGATAGAACATTAATACCATCTCATACTGGAACTAGTGG
GACACCAACAAATATATACCATGGTACAGATCCAGATGATGTTCAATTTTATACTATTGAAAATTCTG
TGCCAGTACACTTACTAAGAACAGGTGATGAATTTGCTACAGGAACATTTTTTTTTGATTGTAAACCA
TGTAGACTAACACATACATGGCAAACAAATAGAGCATTGGGCTTACCACCATTTCTAAATTCTTTGCC
TCAAGCTGAAGGAGGTACTAACTTTGGTTATATAGGAGTTCAACAAGATAAAAGACGTGGTGTAACTC
AAATGGGAAATACAAACTATATTACTGAAGCTACTATTATGAGACCAGCTGAGGTTGGTTATAGTGCA
CCATATTATTCTTTTGAGGCGTCTACACAAGGGCCATTTAAAACACCTATTGCAGCAGGACGGGGGGG
AGCGCAAACAGATGAAAATCAAGCAGCAGATGGTGATCCAAGATATGCATTTGGTAGACAACATGGTC
AAAAAACTACCACAACAGGAGAAACACCTGAGAGATTTACATATATAGCACATCAAGATACAGGAAGA
TATCCAGAAGGAGATTGGATTCAAAATATTAACTTTAACCTTCCTGTAACAAATGATAATGTATTGCT
ACCAACAGATCCAATTGGAGGTAAAACAGGAATTAACTATACTAATATATTTAATACTTATGGTCCTT
TAACTGCATTAAATAATGTACCACCAGTTTATCCAAATGGTCAAATTTGGGATAAAGAATTTGATACT
GACTTAAAACCAAGACTTCATGTAAATGCACCATTTGTTTGTCAAAATAATTGTCCTGGTCAATTATT
TGTAAAAGTTGCGCCTAATTTAACAAATGAATATGATCCTGATGCATCTGCTAATATGTCAAGAATTG
TAACTTACTCAGATTTTTGGTGGAAAGGTAAATTAGTATTTAAAGCTAAACTAAGAGCCTCTCATACT
TGGAATCCAATTCAACAAATGAGTATTAATGTAGATAACCAATTTAACTATGTACCAAGTAATATTGG
AGGTATGAAAATTGTATATGAAAAATCTCAACTAGCACCTAGA
'TAW_U72696_2b_T10_1996'
ATGAGTGATGGAGCAGTTCAACCAGACGGTGGTCAACCTGCTGTCAGAAATGAAAGAGCTACAGGATC
TGGGAACGGGTCTGGAGGCGGGGGTGGTGGTGGTTCTGGGGGTGTGGGGATTTCTACGGGTACTTTCA
ATAATCAGACAGAATTTAAATTTTTGGAAAACGGATGGGTGGAAATCACAGCAAACTCAAGCAGACTT
GTACATTTAAATATGCCAGAAAGTGAAAATTATAGAAGAGTGGTTGTAAATAATTTGGATAAAACTGC
AGTTAACGGAAACATGGCTTTAGATGATACCCATGCACAAATTGTAACACCTTGGTCATTGGTTGATG
CAAATGCTTGGGGAGTTTGGTTTAATCCAGGAGATTGGCAACTAATTGTTAATACTATGAGTGAGTTG
CATTTAGTTAGTTTTGAACAAGAAATTTTTAATGTTGTTTTAAAGACTGTTTCAGAATCTGCTACTCA
GCCACCAACTAAAGTTTATAATAATGATTTAACTGCATCATTGATGGTTGCATTAGATAGTAATAATA
CTATGCCATTTACTCCAGCAGCTATGAGATCTGAGACATTGGGTTTTTATCCATGGAAACCAACCATA
CCAACTCCATGGAGATATTATTTTCAATGGGATAGAACATTAATACCATCTCATACTGGAACTAGTGG
GACACCAACAAATATATACCATGGTACAGATCCAGATGATGTTCAATTTTATACTATTGAAAATTCTG
TGCCAGTACACTTACTAAGAACAGGTGATGAATTTGCTACAGGAACATTTTTTTTTGATTGTAAACCA
TGTAGACTAACACATACATGGCAAACAAATAGAGCATTGGGCTTACCACCATTTCTAAATTCTTTGCC
TCAAGCTGAAGGAGGTACTAACTTTGGTTATATAGGAGTTCAACAAGATAAAAGACGTGGTGTAACTC
AAATGGGAAATACAAACTATATTACTGAAGCTACTATTATGAGACCAGCTGAGGTTGGTTATAGTGCA
CCATATTATTCTTTTGAGGCGTCTACACAAGGGCCATTTAAAACACCTATTGCAGCAGGACGGGGGGG
AGCGCAAACAGATGAAAATCAAGCAGCAGATGGTGATCCAAGATATGCATTTGGTAGACAACATGGTC
AAAAAACTACCACAACAGGAGAAACACCTGAGAGATTTACATATATAGCACATCAAGATACAGGAAGA
TATCCAGAAGGAGATTGGATTCAAAATATTAACTTTAACCTTCCTGTAACAGATGATAATGTATTGCT
ACCAACAGATCCAATTGGAGGTAAAACAGGAATTAACTATACTAATATATTTAATACTTATGGTCCTT
TAACTGCATTAAATAATGTACCACCAGTTTATCCAAATGGTCAAATTTGGGATAAAGAATTTGATACT
GACTTAAAACCAAGACTTCATGTAAATGCACCATTTGTTTGTCAAAATAATTGTCCTGGTCAATTATT
TGTAAAAGTTGCGCCTAATTTAACAAATGAATATGATCCTGATGCATCTGCTAATATGTCAAGAATTG
TAACTTACTCAGATTTTTGGTGGAAAGGTAAATTAGTATTTAAAGCTAAACTAAGAGCCTCTCATACT

TGGAATCCAATTCAACAAATGAGTATTAATGTAGATAACCAATTTAACTATGTACCAAGTAATATTGG
AGGTATGAAAATTGTATATGAAAAATCTCAACTAGCACCTAGA

'POL_Z46651_46_1994'

ATGAGTGATGGAGCAGTTCAACCAGACGGTGGTCAACCTGCTGTCAGAAATGAAAGAGCTACAGGATC
TGGGAACGGGTCTGGAGGCGGGGGTGGTGGTGGTTCTGGGGGTGTGGGGATTTCTACGGGTACTTTCA
ATAATCAGACGGAATTTAAATTTTTGGAAAACGGATGGGTGGAAATCACAGCAAACTCAAGCAGACTT
GTACATTTAAATATGCCAGAAAGTGAAAATTATAGAAGAGTGGTTGTAAATAATTTGGATAAAACTGC
AGTTAACGGAAACATGGCTTTAGATGATACTCATGCACAAATTGTAACACCTTGGTCATTGGTTGATG
CAAATGCTTGGGGAGTTTGGTTTAATCCAGGAGATTGGCAACTAATTGTTAATACTATGAGTGAGTTG
CATTTAGTTAGTTTTGAACAAGAAATTTTTAATGTTGTTTTAAAGACTGTTTCAGAATCTGCTACTCA
GCCACCAACTAAAGTTTATAATAATGATTTAACTGCATCATTGATGGTTGCATTAGATAGTAATAATA
CTATGCCATTTACTCCAGCAGCTATGAGATCTGAGACATTGGGTTTTTATCCATGGAAACCAACCATA
CCAACTCCATGGAGATATTATTTTCAATGGGATAGAACATTAATACCATCTCATACTGGAACTAGTGG
CACACCAACAAATATATACCATGGTACAGATCCAGATGATGTTCAATTTTATACTATTGAAAATTCTG
TGCCAGTACACTTACTAAGAACAGGTGATGAATTTGCTACAGGAACATTTTTTTTTGATTGTAAACCA
TGTAGACTAACACATACATGGCAAACAAATAGAGCATTGGGCTTACCACCATTTCTAAATTCTTTGCC
TCAATCTGAAGGAGGTACTAACTTTGGTTATATAGGAGTTCAACAAGATAAAAGACGTGGTGTAACTC
AAATGGGAAATACAAACTATATTACTGAAGCTACTATTATGAGACCAGCTGAGGTTGGTTATAGTGCA
CCATATTATTCTTTTGAGGCGTCTACACAAGGGCCATTTAAAACACCTATTGCAGCAGGACGGGGGGG
AGCGCAAACAGATGAAAATCAAGCAGCAGATGGTGATCCAAGATATGCATTTGGTAGACAACATGGTC
AAAAAACTACCACAACAGGAGAAACACCTGAGAGATTTACATATATAGCACATCAAGATACAGGAAGA
TATCCAGAAGGAGATTGGATTCAAAATATTAACTTTAACCTTCCTGTAACAGATGATAATGTATTGCT
ACCAACAGATCCAATTGGAGGTAAAACAGGAATTAACTATACTAATATATTTAATACTTATGGTCCTT
TAACTGCATTAAATAATGTACCACCAGTTTATCCAAATGGTCAAATTTGGGATAAAGAATTTGATACT
GACTTAAAACCAAGACTTCATGTAAATGCACCATTTGTTTGTCAAAATAATTGTCCTGGTCAATTATT
TGTAAAAGTTGCGCCTAATTTAACAAATGAATATGATCCTGATGCATCTGCTAATATGTCAAGAATTG
TAACTTACTCAGATTTTTGGTGGAAAGGTAAATTAGTATTTAAAGCTAAACTAAGAGCCTCTCATACT
TGGAATCCAATTCAACAAATGAGTATTAATGTAGATAACCAATTTAACTATGTACCAAGTAATATTGG
AGGTATGAAAATTGTATATGAAAAATCTCAACTAGCACCTAGA

'KOR_EF599097_2b_DH326_2006'

ATGAGTGATGGAGCAGTACAACCAGACGGTGGTCAACCTGCTGTCAGAAATGAAAGAGCTACAGGATC
TGGGAACGGGTCTGGAGGCGGGGGTGGTGGTGGTTCTGGGGGTGTGGGGATTTCTACGGGTACTTTCA
ATAATCAGACGGAATTTAAATTTTTGGAAAACGGATGGGTGGAAATCACAGCAAACTCAAGCAGACTT
GTACATTTAAATATGCCAGAAAGTGAAAATTATAGAAGAGTGGTTGTAAATAATTTGGATAAAACTGC
AGTTAACGGAAACATGGCTTTAGATGATACCCATGCACAAATTGTAACACCTTGGTCATTGGTTGATG
CAAATGCTTGGGGAGTTTGGTTTAATCCAGGAGATTGGCAACTAATTGTTAATACTATGAGTGAGTTG
CATTTAGTTAGTTTTGAACAAGAAATTTTTAATGTTGTTTTAAAGACTGTTTCAGAATCTGCTACTCA
GCCACCAACTAAAGTTTATAATAATGATTTAACTGCATCATTGATGGTTGCATTAGATAGTAATAATA
CTATGCCATTTACTCCAGCAGCTATGAGATCTGAGACATTGGGTTTTTATCCATGGAAACCAACCATA
CCAACTCCATGGAGATATTATTTTCAATGGGATAGAACATTAATACCATCTCATACTGGAACTAGTGG
CACACCAACAAATATATACCATGGTACAGATCCAGATGATGTTCAATTTTATACTATTGAAAATTCTG
TGCCAGTACACTTACTAAGAACAGGTGATGAATTTGCTACAGGAACATTTTTTTTTGATTGTAAACCA
TGTAGACTAACACATACATGGCAAACAAATAGAGCATTGGGCTTACCACCATTTCTAAATTCTTTGCC
TCAAGCTGAAGGAGGTACTAACTTTGGTTATATAGGAGTTCAACAAGATAAAAGACGTGGTGTAACTC
AAATGGGAAATACAAACTATATTACTGAAGCTACTATTATGAGACCAGCTGAGGTTGGTTATAGTGCA
CCATATTATTCTTTTGAGGCATCTACACAAGGGCCATTTAAAACACCTATTGCAGCAGGACGGGGGGG
AGCGCAAACAGATGAAAATCAAGCAGCAGATGGTGATCCAAGATATGCATTTGGTAGACAACATGGTC
AAAAAACTACCACAACAGGAGAAACACCTGAGAGATTTACATATATAGCACATCAAGATACAGGAAGA
TATCCAGAAGGAGATTGGATTCAAAATATTAACTTTAACCTTCCTGTAACAGATGATAATGTATTGCT
ACCAACAGATCCAATTGGAGGTAAAACAGGAATTAACTATACTAATATATTTAATACTTATGGTCCTT
TAACTGCATTAAATAATGTACCACCAGTTTATCCAAATGGTCAAATTTGGGATAAAGAATTTGATACT
GACTTAAAACCAAGACTTCATGTAAATGCACCATTTGTTTGTCAAAATAATTGTCCTGGTCAATTATT
TGTAAAAGTTGCGCCTAATTTAACAAATGAATATGATCCTGATGCATCTGCTAATATGTCAAGAATTG
TAACTTACTCAGATTTTTGGTGGAAAGGTAAATTAGTATTTAAAGCTAAACTAAGAGCCTCTCATACT

```
TGGAATCCAATTCAACAAATGAGTATTAATGTAGATAACCAATTTAACTATGTACCAAGTAATATTGG
AGGTATGAAAATTGTATATGAAAAATCTCAACTAGCACCTAGA
    'KOR_EF599098_2c_Pome_2006'
ATGAGTGATGGAGCAGTTCAACCAGACGGTGGTCAGCCTGCTGTCAGAAATGAAAGAGCTACAGGATC
TGGGAACGGGTCTGGAGGCGGGGGTGGTGGTGGTTCTGGGGGTGTGGGGATTTCTACGGGTACTTTCA
ATAATCAGACGGAATTTAAATTTTTGGAAAACGGATGGGTGGAAATCACAGCAAACTCAAGCAGACTT
GTACATTTAAATATGCCAGAAAGTGAAAATTATAGAAGAGTGGTTGTAAATAATTTGGATAAAACTGC
AGTTAACGGAAACATGGCTTTAGATGATACCCATGCACAAATTGTAACACCTTGGTCATTGGTTGATG
CAAATGCTTGGGGAGTTTGGTTTAATCCAGGAGATTGGCAACTAATTGTTAATACTATGAGTGAGTTG
CATTTAGTTAGTTTTGAACAAGAAATTTTTAATGTTGTTTTAAAGACTGTTTCAGAATCTGCTACTCA
GCCACCAACTAAAGTTTATAATAATGATTTAACTGCATCATTGATGGTTGCATTAGATAGCAATAATA
CTATGCCATTTACTCCAGCAGCTATGAGATCTGAGACATTGGGTTTTTATCCATGGAAACCAACCATA
CCAACTCCATGGAGATATTATTTTCAATGGGATAGAACATTAATACCATCTCATACTGGAACTAGTGG
CACACCAACAAATATATACCATGGTACAGATCCAGATGATGTTCAATTTTATACTATTGAAAATTCTG
TGCCAGTACACTTACTAAGAACAGGTGATGAATTTGCTACAGGAACATTTTTTTTTGATTGTAAACCA
TGTAGACTAACACATACATGGCAAACAAATAGAGCATTGGGCTTACCACCATTTCTAAATTCTTTGCC
TCAAGCTGAAGGAGATACTAACTTTGGTTATATAGGAGTTCAACAAGATAAAAGACGTGGTGTAACTC
AAATGGGAAATACAAACTATATTACTGAAGCTACTATTATGAGACCAGCTGAGGTTGGTTATAGTGCA
CCATATTATTCTTTTGAGGCGTCTACACAAGGGCCATTTAAAACACCTATTGCAGCAGGACGGGGGGG
AGCGCAAACAGATGAAAATCAAGCAGCAGATGGTGATCCAAGATATGCATTTGGTAGACAACATGGTC
AAAAAACTACCACAACAGGAGAAACACCTGAGAGATTTACATATATAGCACATCAAGATACAGGAAGA
TATCCAGAAGGAGATTGGATTCAAAATACTAACTTTAACCTTCCTGTAACAAATGATAATGTATTGCT
ACCAACAGATTCAATTGGAGGTAAAGCAGGAATTAACTATACTAATATATTTAATACTTATGGTCCTT
TAACTGCATTAAATAATGTACCACCAGTTTATCCAAATGGTCAAATTTGGGATAAAGAATTTGATACT
GACTTAAAACCAAGACTTCATGTAAATGCACCATTTGTTTGTCAAAATAATTGTCCTGGTCAATTATT
TGTAAAAGTTGCGCCTAATTTAACAAATGAATATGATCCTGATGCATCTGCTAATATGTCAAGAATTG
TAACTTACTCAGATTTTTGGTGGAAAGGTAAATTAGTATTTAAAGCTAAACTAAGAGCCTCTCATACT
TGGAATCCAATTCAACAAATGAGTATTAATGTAGATAACCAATTTAACTATGTACCAAGTAACATTGG
AGGTATGAAAATTGTATATGAGAAATCTCAACTAGCACCTAGA;
END;

BEGIN TREES;
    TREE tree =
(((((((((((((((((((((((((((((((((((((((((((((((ITA/
FJ005218/2c/330/2006,ITA/FJ005233/40/2007),POR/KT275253/2c/
PT036/12/2012),(URU/KC196096/2c/M247/2010,URU/KM457121/2c/
UY247/2010)),(URU/KC196086/2c/M55/2006,URU/KM457106/2c/
UY55/2006)),USA/JX475260/CO/704/2010),ITA/FJ005247/195/2008),ITA/
FJ005226/383/2006),GER/FJ005196/2c/G7/1997),FRA/
DQ025994/04S25/2004),FRA/DQ025960/03C4/2003),FRA/
DQ025951/03B10/2003),FRA/DQ025954/03B14/2003),USA/KJ813848/Bobcat/
ND/1162/2013),URU/KM457104/2c/UY47/2006),FRA/
DQ025969/03S5/2003),USA/KJ813858/Puma/ND/F93/2013),ITA/
FJ005248/219/2008),GER/FJ005199/2c/G172/1997),ITA/
FJ005240/208/2007),URU/KC196085/2c/M57/2007),FRA/
DQ025975/04S6/2004),USA/JX475243/ID/22772/2009),USA/JX475252/CO/
1316/2010),FRA/DQ025965/03C9/2003),ECU/KF149984/2c/ME28/2012),(ECU/
KF149962/2c/ME1/2012,(ECU/KF149963/2c/ME10/2012,(ECU/KF149964/2c/
ME23/2012,ECU/KF149969/2c/ME31/2012)))),(ARG/JF414820/Arg44/2009,
(ARG/KM236569/Cuba/2013,(ARG/JF414818/Arg32/2008,ARG/JF414821/
Arg48/2009)))),(ITA/FJ005216/2c/284/2006,(ITA/KU508407/2c/
25835/09/2009,ITA/KX434459/27692/1/11/2011))),(((URU/KM457122/2c/
UY258/2010,URU/KM457124/2c/UY307/2011),URU/KC196093/2c/M307/2011),
((URU/KC196081/2c/M95/2007,URU/KM457109/2c/UY95/2007),(URU/
```

KC196097/2c/M242/2010,(URU/KM457120/2c/UY242/2010,URU/KM457123/2c/
UY261/2008))))),(URU/KM457103/2c/UY12/2006,(ITA/FJ005209/2c/
303/2004,ITA/FJ005251/239/2008))),(URU/KC196083/2c/M82/2007,URU/
KM457108/2c/UY82/2007)),URU/KC196107/2c/M129/2008),URU/KM457131/2c/
UY368/2011),POR/KT275252/2c/PT013/12/2012),ITA/FJ005231/406/2006),
(ITA/FJ005214/2c/67/2006,BRA/KY073269/UFMT/2015)),ARG/JF414819/
Arg35/2008),USA/KJ813854/Puma/ND/F205/2013),ITA/FJ005232/411/2006),
(((URU/KC196105/2c/M152/2008,URU/KM457113/2c/UY152/2009),ITA/
FJ005212/2c/349/2004),(POR/KT275255/2c/PT238/14/2014,(ITA/
KX434460/52238/12/2012,((URU/KC196091/2c/M326/2011,URU/KM457127/2c/
UY326/2011),(((((((((URU/KC196102/2c/M185/2009,URU/KM457116/2c/
UY185/2009),(HRV/KP859577/2c/HR856/2014,ITA/KX434458/2323/11/2011)),
(URU/KC196101/2c/M187/2009,URU/KM457117/2c/UY187/2009)),AUS/
KU508693/2c/LW/2015),(URU/KC196089/2c/M349/2011,URU/KM457129/2c/
UY349/2011)),ITA/FJ005195/2c/136/2000),FRA/DQ025976/04S7/2004),(USA/
JX475273/MT/909/2012,USA/KJ813888/Coyote/MT/878/2012)),USA/KJ813843/
Bobcat/ND/1160/2013),(FRA/DQ025942/01B1/2001,(FRA/
DQ025964/03C8/2003,(ITA/FJ005206/2c/287/2004,(URU/KM457107/2c/
UY72/2007,(URU/KM457111/2c/UY120/2008,(URU/KM457112/2c/UY135/2008,
(URU/KM457125/2c/UY317/2011,(URU/KM457130/2c/UY354/2011,(URU/
KM457142/2c/UY370/2011,(HRV/KP859574/2c/HR442/2014,(HRV/KP859575/2c/
HR774/2014,(HRV/KP859576/2c/HR793/2014,(AUS/KU508691/2c/HB/2015,
(AUS/KU508692/2c/FH/2015,ITA/
KX434456/45361/09/2009))))))))))))))))),URU/KM457126/2c/
UY318/2010),(FRA/DQ025985/04S16/2004,(ITA/FJ005205/2c/279/2004,HRV/
KP859578/2c/HR859/2014))),ITA/FJ222821/2c/56/2000),(GER/FJ005260/
G82/1997,USA/KJ813846/Bobcat/ND/974/2013)),((GER/
AY742934/447/1995,RUS/JN033694/Laika/1993),(USA/AY742936/395/1998,
(USA/JX475240/AZ/16382-01/1999,(USA/JX475250/CO/728/2010,USA/
KJ813842/Bobcat/ND/502/2013))))),(((((((((((((((((USA/KJ813828/
Fisher/F1F010712/2013,USA/KJ813881/Graywolf/MI/832/2012),USA/
KJ813844/Bobcat/ND/885/2013),USA/KJ813882/Raccoon/NJ/1423/2012),USA/
KJ813851/Bobcat/ND/1168/2013),USA/JX475278/AR/1069/2012),KOR/
EU009205/2b/K029/2006),USA/JX475247/CO/1246/2010),(USA/KJ813892/
Coyote/AK/218/2013,(USA/JN867604/Dog/IL/137654/2008,USA/JX475242/WI/
18268/2002))),SAF/HQ602969/22/10SA/2010),(USA/AY742955/436/2003,FRA/
DQ025991/2b/04S22/2004)),USA/JN867602/2b/Dog/CA/148743/2008),USA/
JX475251/CO/2235/2009),USA/JN867603/2b/Dog/KS/81213/2009),USA/
KJ813852/Bobcat/ND/1170/2013),FRA/DQ025961/2b/03C5/2003),(USA/
KJ813827/Fisher/F1M111211/2013,USA/KJ813873/Graywolf/MI/850/2012)),
(ECU/KF149971/2c/ME32/2012,IND/KX469432/newCPV/2b/Hiller/2011)),
((ITA/FJ005263/42/2005,ITA/FJ005265/140/2005),(((((((((USA/
M74849/39/1995,USA/U22896/cat/1990),FRA/DQ025992/2b/04S23/2004),USA/
M74852/133/1995),POR/KU662349/greywolf/W33/1996),(POR/KU662350/
greywolf/W52/2005,POL/Z46651/46/1994)),GER/FJ005261/G162/1997),BRA/
DQ340409/2b/BR183/1985),USA/AY742932/193/1991),((USA/
AY742951/431/2003,USA/JN867605/2b/Dog/US/142805/2009),(VAC/
FJ222822/2b/FortDodge/2008,(VAC/JN625223/INDIA/vac5/2011,(USA/
EU659119/2b/CPV/410/2000,USA/EU659120/2b/CPV/411a/1998))))),((CHI/
GQ857609/CPV08/01/2008,CHI/GU569940/2b/YN0203/2002),((((((JPN/
AB115504/2c/97/008/1997,TAW/U72696/2b/T10/1996),TAW/U72695/2a/
T4/1996),(CHI/GQ857596/CPV05/01/2005,CHI/GQ857600/
CPV06/01/2006)),THA/FJ869125/KU5/2004),(USA/JX475237/CT/
372/2011,KOR/EF599097/2b/DH326/2006)),(CHI/EU483515/2b/ZD13/2007,

(JPN/LC270891/2b/9985/2017,(JPN/AB437433/1887/M/2/2008,(TAW/
FJ265781/CPV307/2005,(TWN/EF592511/TWN1/2006,TAW/FJ265775/
CPV301/2004)))))),(((VIE/AB054218/2b/cat/V123/2000,VAC/FJ222823/2b/
29/1997),ITA/FJ005264/134/2005),((THA/FJ869122/KU1/2008,THA/
FJ869123/KU3/2008),((((((THA/KP715690/VT28/2014,THA/KP715716/
VT143/2014),THA/KP715691/VT43/2014),(VIE/AB120722/2b/HCM/
18/2003,VIE/AB120724/2b/HNI/2/13/2003)),(CHI/GQ857599/
CPV05/04/2005,CHI/GQ857601/CPV06/02/2006)),CHI/GQ857605/
CPV07/03/2007),(THA/FJ869139/KU66/2003,(VIE/AB120721/2b/HCM/8/2003,
(VIE/AB054221/2b/leopard/V204/2000,(VIE/AB054224/2c/leopard/
V203/2000,(VIE/AB120725/2b/HNI/3/4/2003,(VIE/AB120723/2b/HCM/
23/2003,(VIE/AB120720/2b/HCM/6/2003,(VIE/AB054219/2b/cat/V209/2000,
(VIE/AB054220/2b/cat/V217/2000,CHI/EU145954/2b/
BJ044/20O7)))))))))))))))))),(ITA/FJ005257/54/2008,ITA/KF373611/2a/
409/2010)),(NZE/AY742933/339/1993,((VIE/AB054223/2c/leopard/
V140/2000,ITA/GU362932/cat11/2008),(NIG/HQ602995/15/10/2010,
(((((((((FRA/DQ025947/2a/02B5/2002,FRA/DQ026001/2a/04S32/2004),FRA/
DQ025962/2a/03C6/2003),ITA/KF373580/2a/581/2003),(GER/AY742935/
U6/1995,FRA/DQ025945/2a/02B3/2002)),VIE/AB054215/2a/cat/
V120/2000),ITA/FJ005255/333/2005),FRA/DQ025958/2a/03C2/2003),(ITA/
KX434457/987/10/2010,(FRA/DQ025983/2a/04S14/2004,FRA/DQ025993/2a/
04S24/2004))),(FRA/DQ025984/2a/04S15/2004,ITA/FJ005252/96/2002)),
(FRA/DQ026002/2a/04S33/2004,(ITA/KF373592/2a/329/2008,(((ITA/
AF393506/2a/699/2000,FRA/DQ025943/2a/01S1/2001),ITA/KF385388/2a/
Sicily/X83090/2009),((CHI/GQ857612/CPV08/04/2008,CHI/GU569939/2a/
YN0202/2002),(((((HUN/KF539794/H/7/2012,HUN/KF539795/H/8/2012),HUN/
KF539804/H/212/2012),(HUN/KF539793/H/5/2012,HUN/KF539797/H/
11/2012)),(HUN/KF539800/H/27/2012,(VIE/AB054217/2a/cat/
V154/2000,HUN/KF539796/H/9/2012))),(((HUN/KF539798/H/31/2012,HUN/
KF539799/H/39/2012),HUN/KF539805/H/36/2012),(ITA/AF306447/618/2000,
(FRA/DQ025944/2a/02B2/2002,(NIG/HQ602992/19/10/2010,(ITA/
AF306446/584/2000,(FRA/DQ025986/2a/04S17/2004,(ITA/KF373577/2a/
714/2001,(FRA/DQ025982/2a/04S13/2004,ITA/
FJ005253/67/2005)))))))))))))))))),((((((((THA/FJ869126/
KU5/2008,THA/FJ869137/KU52/2003),THA/FJ869134/KU23/2003),CHI/
DQ354068/2a/redpanda/RPPV/2004),KOR/EF599096/DH426/2005),(ITA/
FJ005258/80/2008,(KOR/EF599098/2c/Pome/2006,(FRA/DQ025950/2a/
02B9/2002,ITA/KX434454/29451/09/2009)))),(THA/FJ869130/KU13/2004,
(THA/FJ869138/KU53/2003,CHI/KF803615/2011/BJ/B25/2011))),(CHI/
GU569942/2a/JL0202/2002,CHI/GU569946/2a/JL0201/2002)),((USA/
AY742953/435/2003,ITA/KF373571/2a/685/1999),(THA/FJ869128/KU11/2004,
(((BRA/DQ340428/2a/BR209/1994,BRA/DQ340431/2a/BR56/1995),BRA/
DQ340411/2a/BR8/1990),(BRA/DQ340422/2a/BR22/1993,(BRA/DQ340421/2a/
BR597/1992,((((BRA/DQ340419/2a/BR570/1992,BRA/DQ340423/2a/
BR136/1993),BRA/DQ340413/2a/BR18/1990),BRA/DQ340427/2a/BR133/1994),
(BRA/DQ340414/2a/BR31/1990,(BRA/DQ340416/2a/BR47/1991,(BRA/
DQ340417/2a/BR52/1991,(BRA/DQ340418/2a/BR491/1992,(BRA/DQ340424/2a/
BR137/1993,BRA/DQ340426/2a/BR84/1994))))))))))))),CHI/KF803600/2010/
BJ/A68/2010),(((USA/EU659118/CPV/13/1981,CHI/GU569948/2a/
CC8601/1986),JPN/D26079/1993),((BRA/DQ340407/2a/BR145/1980,BRA/
DQ340408/2a/BR154/1980),(FRA/DQ025952/2a/03B12/2003,(BRA/
DQ340404/2a/BR6/1980,(BRA/DQ340405/2a/BR135/1980,(BRA/DQ340410/2a/
BR315/1986,(USA/M24000/FPV/CPV/31/1988,USA/M24003/FPV/CPV/
15/1988)))))))),((USA/JN867599/Raccoon/KY/39552/2009,USA/JN867611/

```
Raccoon/KY/358-B/2009),(USA/JN867610/Raccoon/VA/118-A/2007,(USA/
KJ813890/Redfox/MA/197/2012,(USA/JX475284/TN/26/2011,(USA/JX475239/
GA/06/2011,USA/JX475279/TN/1/2011)))))),(HUN/KF539801/H/25/2012,HUN/
KF539803/H/2/2012)),(USA/KJ813870/Raccoon/TX/1/2013,(((USA/JN867598/
Bobcat/KS/44/2010,USA/KJ813832/Fisher/ND/14/2013),(USA/KJ813831/
Fisher/ND/17/2013,USA/KJ813835/Fisher/ND/19/2013)),(USA/JX475234/ME/
258/2011,(USA/JN867618//Raccoon/WI/37/2010,(USA/JX475231/CO/
280/2011,(USA/JX475248/CO/1102/2011,(USA/JX475233/SC/182-A/2011,USA/
JX475246/CO/2503/2010))))))),((CHI/FJ231389/FPV/monkey/
BJ-22/2008,CHI/KJ170680/raccoondog/HLJ11/1/2011),((((((((CHI/
GU392242/raccoondog/HB10/2009,CHI/GU392244/raccoondog/HB7/2009),CHI/
KJ170679/raccoondog/Heb10/2/2010),CHI/GU392241/raccoondog/
HB1/2009),CHI/GU392236/fox/HB1/2009),(CHI/GU392240/raccoondog/
HB3/2009,(CHI/GU392239/raccoondog/HB6/2009,CHI/KJ194463/raccoondog/
HeB10/3/2010))),CHI/GU392237/fox/HB2/2009),(VAC/FJ011098/Intervet/
2006,(VAC/JN625222/INDIA/vac4/2011,(ITA/FJ222824/388/05/3/2005,(CHI/
FJ432718/CPV/Cv/2008,(VAC/JN625219/INDIA/vac1/2011,CHI/
KF803602/2010/BJ/A72/2010)))))),JPN/AB437434/1887/f/3/2008),
((((((VAC/GU212790/primodog/2009,VAC/GU212791/vanguard/2009),VAC/
FJ197847/Pfizer/2007),VAC/EU914139/Pfizer//2006),VAC/KY083089/
Singapore/2016),USA/M19296/CPV/N/1988),(((((USA/M23255/FPV/
Cornell320/1988,USA/M38245/1990),USA/EU659116/CPV/5/1979),(FIN/
U22192/raccoondog/RD-80/1980,FIN/U22193/raccoondog/RD87/1987)),(USA/
M10989/1985,USA/U22186/CPV/128/1995)),(VAC/JN625221/INDIA/vac3/2011,
(VAC/JN625220/INDIA/vac2/2011,(((VAC/FJ011097/Merial/2006,CHI/
GQ169553/Vac2/2007),VAC/KY083090/Singapore/2016),(CHI/GU569943/
YB8301/1983,(VAC/JN625224/INDIA/vac6/2011,ARG/KM236572/NNGag/
2012))))))))));
END;

BEGIN HYPHY;


global busted.test.bsrel_mixture_aux_1=0.7753737053701732;
busted.test.bsrel_mixture_aux_1:<1;
global busted.test.theta_GT=0.182114828971069;
global busted.test.omega2=0;
busted.test.omega2:<1;
global busted.test.omega3=15.64372143327618;
busted.test.omega3:>1;
global busted.test.theta_CT=0.7861469071992863;
global busted.test.bsrel_mixture_aux_0=0.9645280755196957;
busted.test.bsrel_mixture_aux_0:<1;
global busted.test.omega1=0;
busted.test.omega1:<1;
global busted.test.theta_CG=0.2116256644065798;
global busted.test.theta_AC=0.227270307414119;
global busted.test.theta_AT=0.07967131977770318;
global busted.test.theta_AG:=1;
busted.test_Q_component_1={61,61};
busted.test_Q_component_1[0]
[1]:=busted.test.theta_AC*busted.test.omega1*t*0.0447809360409677;
busted.test_Q_component_1[0]
[2]:=busted.test.theta_AG*t*0.1063345886498611;
```

```
busted.test_Q_component_1[0]
[3]:=busted.test.theta_AT*busted.test.omega1*t*0.419803077292116;
busted.test_Q_component_1[0]
[4]:=busted.test.theta_AC*busted.test.omega1*t*0.2496987119458206;
busted.test_Q_component_1[0]
[8]:=busted.test.theta_AG*busted.test.omega1*t*0.1886019614740855;
busted.test_Q_component_1[0]
[12]:=busted.test.theta_AT*busted.test.omega1*t*0.2208116300950855;
busted.test_Q_component_1[0]
[16]:=busted.test.theta_AC*busted.test.omega1*t*0.1512809665850036;
busted.test_Q_component_1[0]
[32]:=busted.test.theta_AG*busted.test.omega1*t*0.2936069988308121;
busted.test_Q_component_1[1]
[0]:=busted.test.theta_AC*busted.test.omega1*t*0.4290813980170551;
busted.test_Q_component_1[1]
[2]:=busted.test.theta_CG*busted.test.omega1*t*0.1063345886498611;
busted.test_Q_component_1[1]
[3]:=busted.test.theta_CT*t*0.419803077292116;
busted.test_Q_component_1[1]
[5]:=busted.test.theta_AC*busted.test.omega1*t*0.2496987119458206;
busted.test_Q_component_1[1]
[9]:=busted.test.theta_AG*busted.test.omega1*t*0.1886019614740855;
busted.test_Q_component_1[1]
[13]:=busted.test.theta_AT*busted.test.omega1*t*0.2208116300950855;
busted.test_Q_component_1[1]
[17]:=busted.test.theta_AC*busted.test.omega1*t*0.1512809665850036;
busted.test_Q_component_1[1]
[33]:=busted.test.theta_AG*busted.test.omega1*t*0.2936069988308121;
busted.test_Q_component_1[1]
[48]:=busted.test.theta_AT*busted.test.omega1*t*0.244832889475194;
busted.test_Q_component_1[2]
[0]:=busted.test.theta_AG*t*0.4290813980170551;
busted.test_Q_component_1[2]
[1]:=busted.test.theta_CG*busted.test.omega1*t*0.0447809360409677;
busted.test_Q_component_1[2]
[3]:=busted.test.theta_GT*busted.test.omega1*t*0.419803077292116;
busted.test_Q_component_1[2]
[6]:=busted.test.theta_AC*busted.test.omega1*t*0.2496987119458206;
busted.test_Q_component_1[2]
[10]:=busted.test.theta_AG*busted.test.omega1*t*0.1886019614740855;
busted.test_Q_component_1[2]
[14]:=busted.test.theta_AT*busted.test.omega1*t*0.2208116300950855;
busted.test_Q_component_1[2]
[18]:=busted.test.theta_AC*busted.test.omega1*t*0.1512809665850036;
busted.test_Q_component_1[2]
[34]:=busted.test.theta_AG*busted.test.omega1*t*0.2936069988308121;
busted.test_Q_component_1[3]
[0]:=busted.test.theta_AT*busted.test.omega1*t*0.4290813980170551;
busted.test_Q_component_1[3]
[1]:=busted.test.theta_CT*t*0.0447809360409677;
busted.test_Q_component_1[3]
[2]:=busted.test.theta_GT*busted.test.omega1*t*0.1063345886498611;
busted.test_Q_component_1[3]
[7]:=busted.test.theta_AC*busted.test.omega1*t*0.2496987119458206;
```

```
busted.test_Q_component_1[3]
[11]:=busted.test.theta_AG*busted.test.omega1*t*0.1886019614740855;
busted.test_Q_component_1[3]
[15]:=busted.test.theta_AT*busted.test.omega1*t*0.2208116300950855;
busted.test_Q_component_1[3]
[19]:=busted.test.theta_AC*busted.test.omega1*t*0.1512809665850036;
busted.test_Q_component_1[3]
[35]:=busted.test.theta_AG*busted.test.omega1*t*0.2936069988308121;
busted.test_Q_component_1[3]
[49]:=busted.test.theta_AT*busted.test.omega1*t*0.244832889475194;
busted.test_Q_component_1[4]
[0]:=busted.test.theta_AC*busted.test.omega1*t*0.3408876964850085;
busted.test_Q_component_1[4]
[5]:=busted.test.theta_AC*t*0.0447809360409677;
busted.test_Q_component_1[4]
[6]:=busted.test.theta_AG*t*0.1063345886498611;
busted.test_Q_component_1[4]
[7]:=busted.test.theta_AT*t*0.419803077292116;
busted.test_Q_component_1[4]
[8]:=busted.test.theta_CG*busted.test.omega1*t*0.1886019614740855;
busted.test_Q_component_1[4]
[12]:=busted.test.theta_CT*busted.test.omega1*t*0.2208116300950855;
busted.test_Q_component_1[4]
[20]:=busted.test.theta_AC*busted.test.omega1*t*0.1512809665850036;
busted.test_Q_component_1[4]
[36]:=busted.test.theta_AG*busted.test.omega1*t*0.2936069988308121;
busted.test_Q_component_1[4]
[50]:=busted.test.theta_AT*busted.test.omega1*t*0.244832889475194;
busted.test_Q_component_1[5]
[1]:=busted.test.theta_AC*busted.test.omega1*t*0.3408876964850085;
busted.test_Q_component_1[5]
[4]:=busted.test.theta_AC*t*0.4290813980170551;
busted.test_Q_component_1[5]
[6]:=busted.test.theta_CG*t*0.1063345886498611;
busted.test_Q_component_1[5]
[7]:=busted.test.theta_CT*t*0.419803077292116;
busted.test_Q_component_1[5]
[9]:=busted.test.theta_CG*busted.test.omega1*t*0.1886019614740855;
busted.test_Q_component_1[5]
[13]:=busted.test.theta_CT*busted.test.omega1*t*0.2208116300950855;
busted.test_Q_component_1[5]
[21]:=busted.test.theta_AC*busted.test.omega1*t*0.1512809665850036;
busted.test_Q_component_1[5]
[37]:=busted.test.theta_AG*busted.test.omega1*t*0.2936069988308121;
busted.test_Q_component_1[5]
[51]:=busted.test.theta_AT*busted.test.omega1*t*0.244832889475194;
busted.test_Q_component_1[6]
[2]:=busted.test.theta_AC*busted.test.omega1*t*0.3408876964850085;
busted.test_Q_component_1[6]
[4]:=busted.test.theta_AG*t*0.4290813980170551;
busted.test_Q_component_1[6]
[5]:=busted.test.theta_CG*t*0.0447809360409677;
busted.test_Q_component_1[6]
[7]:=busted.test.theta_GT*t*0.419803077292116;
```

```
busted.test_Q_component_1[6]
[10]:=busted.test.theta_CG*busted.test.omega1*t*0.1886019614740855;
busted.test_Q_component_1[6]
[14]:=busted.test.theta_CT*busted.test.omega1*t*0.2208116300950855;
busted.test_Q_component_1[6]
[22]:=busted.test.theta_AC*busted.test.omega1*t*0.1512809665850036;
busted.test_Q_component_1[6]
[38]:=busted.test.theta_AG*busted.test.omega1*t*0.2936069988308121;
busted.test_Q_component_1[6]
[52]:=busted.test.theta_AT*busted.test.omega1*t*0.244832889475194;
busted.test_Q_component_1[7]
[3]:=busted.test.theta_AC*busted.test.omega1*t*0.3408876964850085;
busted.test_Q_component_1[7]
[4]:=busted.test.theta_AT*t*0.4290813980170551;
busted.test_Q_component_1[7]
[5]:=busted.test.theta_CT*t*0.0447809360409677;
busted.test_Q_component_1[7]
[6]:=busted.test.theta_GT*t*0.1063345886498611;
busted.test_Q_component_1[7]
[11]:=busted.test.theta_CG*busted.test.omega1*t*0.1886019614740855;
busted.test_Q_component_1[7]
[15]:=busted.test.theta_CT*busted.test.omega1*t*0.2208116300950855;
busted.test_Q_component_1[7]
[23]:=busted.test.theta_AC*busted.test.omega1*t*0.1512809665850036;
busted.test_Q_component_1[7]
[39]:=busted.test.theta_AG*busted.test.omega1*t*0.2936069988308121;
busted.test_Q_component_1[7]
[53]:=busted.test.theta_AT*busted.test.omega1*t*0.244832889475194;
busted.test_Q_component_1[8]
[0]:=busted.test.theta_AG*busted.test.omega1*t*0.3408876964850085;
busted.test_Q_component_1[8]
[4]:=busted.test.theta_CG*busted.test.omega1*t*0.2496987119458206;
busted.test_Q_component_1[8]
[9]:=busted.test.theta_AC*busted.test.omega1*t*0.0447809360409677;
busted.test_Q_component_1[8]
[10]:=busted.test.theta_AG*t*0.1063345886498611;
busted.test_Q_component_1[8]
[11]:=busted.test.theta_AT*busted.test.omega1*t*0.419803077292116;
busted.test_Q_component_1[8]
[12]:=busted.test.theta_GT*busted.test.omega1*t*0.2208116300950855;
busted.test_Q_component_1[8]
[24]:=busted.test.theta_AC*t*0.1512809665850036;
busted.test_Q_component_1[8]
[40]:=busted.test.theta_AG*busted.test.omega1*t*0.2936069988308121;
busted.test_Q_component_1[9]
[1]:=busted.test.theta_AG*busted.test.omega1*t*0.3408876964850085;
busted.test_Q_component_1[9]
[5]:=busted.test.theta_CG*busted.test.omega1*t*0.2496987119458206;
busted.test_Q_component_1[9]
[8]:=busted.test.theta_AC*busted.test.omega1*t*0.4290813980170551;
busted.test_Q_component_1[9]
[10]:=busted.test.theta_CG*busted.test.omega1*t*0.1063345886498611;
busted.test_Q_component_1[9]
[11]:=busted.test.theta_CT*t*0.419803077292116;
```

```
busted.test_Q_component_1[9]
[13]:=busted.test.theta_GT*busted.test.omega1*t*0.2208116300950855;
busted.test_Q_component_1[9]
[25]:=busted.test.theta_AC*busted.test.omega1*t*0.1512809665850036;
busted.test_Q_component_1[9]
[41]:=busted.test.theta_AG*busted.test.omega1*t*0.2936069988308121;
busted.test_Q_component_1[9]
[54]:=busted.test.theta_AT*busted.test.omega1*t*0.244832889475194;
busted.test_Q_component_1[10]
[2]:=busted.test.theta_AG*busted.test.omega1*t*0.3408876964850085;
busted.test_Q_component_1[10]
[6]:=busted.test.theta_CG*busted.test.omega1*t*0.2496987119458206;
busted.test_Q_component_1[10]
[8]:=busted.test.theta_AG*t*0.4290813980170551;
busted.test_Q_component_1[10]
[9]:=busted.test.theta_CG*busted.test.omega1*t*0.0447809360409677;
busted.test_Q_component_1[10]
[11]:=busted.test.theta_GT*busted.test.omega1*t*0.419803077292116;
busted.test_Q_component_1[10]
[14]:=busted.test.theta_GT*busted.test.omega1*t*0.2208116300950855;
busted.test_Q_component_1[10]
[26]:=busted.test.theta_AC*t*0.1512809665850036;
busted.test_Q_component_1[10]
[42]:=busted.test.theta_AG*busted.test.omega1*t*0.2936069988308121;
busted.test_Q_component_1[10]
[55]:=busted.test.theta_AT*busted.test.omega1*t*0.244832889475194;
busted.test_Q_component_1[11]
[3]:=busted.test.theta_AG*busted.test.omega1*t*0.3408876964850085;
busted.test_Q_component_1[11]
[7]:=busted.test.theta_CG*busted.test.omega1*t*0.2496987119458206;
busted.test_Q_component_1[11]
[8]:=busted.test.theta_AT*busted.test.omega1*t*0.4290813980170551;
busted.test_Q_component_1[11]
[9]:=busted.test.theta_CT*t*0.0447809360409677;
busted.test_Q_component_1[11]
[10]:=busted.test.theta_GT*busted.test.omega1*t*0.1063345886498611;
busted.test_Q_component_1[11]
[15]:=busted.test.theta_GT*busted.test.omega1*t*0.2208116300950855;
busted.test_Q_component_1[11]
[27]:=busted.test.theta_AC*busted.test.omega1*t*0.1512809665850036;
busted.test_Q_component_1[11]
[43]:=busted.test.theta_AG*busted.test.omega1*t*0.2936069988308121;
busted.test_Q_component_1[11]
[56]:=busted.test.theta_AT*busted.test.omega1*t*0.244832889475194;
busted.test_Q_component_1[12]
[0]:=busted.test.theta_AT*busted.test.omega1*t*0.3408876964850085;
busted.test_Q_component_1[12]
[4]:=busted.test.theta_CT*busted.test.omega1*t*0.2496987119458206;
busted.test_Q_component_1[12]
[8]:=busted.test.theta_GT*busted.test.omega1*t*0.1886019614740855;
busted.test_Q_component_1[12]
[13]:=busted.test.theta_AC*t*0.0447809360409677;
busted.test_Q_component_1[12]
[14]:=busted.test.theta_AG*busted.test.omega1*t*0.1063345886498611;
```

```
busted.test_Q_component_1[12]
[15]:=busted.test.theta_AT*t*0.419803077292116;
busted.test_Q_component_1[12]
[28]:=busted.test.theta_AC*busted.test.omega1*t*0.1512809665850036;
busted.test_Q_component_1[12]
[44]:=busted.test.theta_AG*busted.test.omega1*t*0.2936069988308121;
busted.test_Q_component_1[12]
[57]:=busted.test.theta_AT*busted.test.omega1*t*0.244832889475194;
busted.test_Q_component_1[13]
[1]:=busted.test.theta_AT*busted.test.omega1*t*0.3408876964850085;
busted.test_Q_component_1[13]
[5]:=busted.test.theta_CT*busted.test.omega1*t*0.2496987119458206;
busted.test_Q_component_1[13]
[9]:=busted.test.theta_GT*busted.test.omega1*t*0.1886019614740855;
busted.test_Q_component_1[13]
[12]:=busted.test.theta_AC*t*0.4290813980170551;
busted.test_Q_component_1[13]
[14]:=busted.test.theta_CG*busted.test.omega1*t*0.1063345886498611;
busted.test_Q_component_1[13]
[15]:=busted.test.theta_CT*t*0.419803077292116;
busted.test_Q_component_1[13]
[29]:=busted.test.theta_AC*busted.test.omega1*t*0.1512809665850036;
busted.test_Q_component_1[13]
[45]:=busted.test.theta_AG*busted.test.omega1*t*0.2936069988308121;
busted.test_Q_component_1[13]
[58]:=busted.test.theta_AT*busted.test.omega1*t*0.244832889475194;
busted.test_Q_component_1[14]
[2]:=busted.test.theta_AT*busted.test.omega1*t*0.3408876964850085;
busted.test_Q_component_1[14]
[6]:=busted.test.theta_CT*busted.test.omega1*t*0.2496987119458206;
busted.test_Q_component_1[14]
[10]:=busted.test.theta_GT*busted.test.omega1*t*0.1886019614740855;
busted.test_Q_component_1[14]
[12]:=busted.test.theta_AG*busted.test.omega1*t*0.4290813980170551;
busted.test_Q_component_1[14]
[13]:=busted.test.theta_CG*busted.test.omega1*t*0.0447809360409677;
busted.test_Q_component_1[14]
[15]:=busted.test.theta_GT*busted.test.omega1*t*0.419803077292116;
busted.test_Q_component_1[14]
[30]:=busted.test.theta_AC*busted.test.omega1*t*0.1512809665850036;
busted.test_Q_component_1[14]
[46]:=busted.test.theta_AG*busted.test.omega1*t*0.2936069988308121;
busted.test_Q_component_1[14]
[59]:=busted.test.theta_AT*busted.test.omega1*t*0.244832889475194;
busted.test_Q_component_1[15]
[3]:=busted.test.theta_AT*busted.test.omega1*t*0.3408876964850085;
busted.test_Q_component_1[15]
[7]:=busted.test.theta_CT*busted.test.omega1*t*0.2496987119458206;
busted.test_Q_component_1[15]
[11]:=busted.test.theta_GT*busted.test.omega1*t*0.1886019614740855;
busted.test_Q_component_1[15]
[12]:=busted.test.theta_AT*t*0.4290813980170551;
busted.test_Q_component_1[15]
[13]:=busted.test.theta_CT*t*0.0447809360409677;
```

```
busted.test_Q_component_1[15]
[14]:=busted.test.theta_GT*busted.test.omega1*t*0.1063345886498611;
busted.test_Q_component_1[15]
[31]:=busted.test.theta_AC*busted.test.omega1*t*0.1512809665850036;
busted.test_Q_component_1[15]
[47]:=busted.test.theta_AG*busted.test.omega1*t*0.2936069988308121;
busted.test_Q_component_1[15]
[60]:=busted.test.theta_AT*busted.test.omega1*t*0.244832889475194;
busted.test_Q_component_1[16]
[0]:=busted.test.theta_AC*busted.test.omega1*t*0.3102791451089904;
busted.test_Q_component_1[16]
[17]:=busted.test.theta_AC*busted.test.omega1*t*0.0447809360409677;
busted.test_Q_component_1[16]
[18]:=busted.test.theta_AG*t*0.1063345886498611;
busted.test_Q_component_1[16]
[19]:=busted.test.theta_AT*busted.test.omega1*t*0.419803077292116;
busted.test_Q_component_1[16]
[20]:=busted.test.theta_AC*busted.test.omega1*t*0.2496987119458206;
busted.test_Q_component_1[16]
[24]:=busted.test.theta_AG*busted.test.omega1*t*0.1886019614740855;
busted.test_Q_component_1[16]
[28]:=busted.test.theta_AT*busted.test.omega1*t*0.2208116300950855;
busted.test_Q_component_1[16]
[32]:=busted.test.theta_CG*busted.test.omega1*t*0.2936069988308121;
busted.test_Q_component_1[17]
[1]:=busted.test.theta_AC*busted.test.omega1*t*0.3102791451089904;
busted.test_Q_component_1[17]
[16]:=busted.test.theta_AC*busted.test.omega1*t*0.4290813980170551;
busted.test_Q_component_1[17]
[18]:=busted.test.theta_CG*busted.test.omega1*t*0.1063345886498611;
busted.test_Q_component_1[17]
[19]:=busted.test.theta_CT*t*0.419803077292116;
busted.test_Q_component_1[17]
[21]:=busted.test.theta_AC*busted.test.omega1*t*0.2496987119458206;
busted.test_Q_component_1[17]
[25]:=busted.test.theta_AG*busted.test.omega1*t*0.1886019614740855;
busted.test_Q_component_1[17]
[29]:=busted.test.theta_AT*busted.test.omega1*t*0.2208116300950855;
busted.test_Q_component_1[17]
[33]:=busted.test.theta_CG*busted.test.omega1*t*0.2936069988308121;
busted.test_Q_component_1[17]
[48]:=busted.test.theta_CT*busted.test.omega1*t*0.244832889475194;
busted.test_Q_component_1[18]
[2]:=busted.test.theta_AC*busted.test.omega1*t*0.3102791451089904;
busted.test_Q_component_1[18]
[16]:=busted.test.theta_AG*t*0.4290813980170551;
busted.test_Q_component_1[18]
[17]:=busted.test.theta_CG*busted.test.omega1*t*0.0447809360409677;
busted.test_Q_component_1[18]
[19]:=busted.test.theta_GT*busted.test.omega1*t*0.419803077292116;
busted.test_Q_component_1[18]
[22]:=busted.test.theta_AC*busted.test.omega1*t*0.2496987119458206;
busted.test_Q_component_1[18]
[26]:=busted.test.theta_AG*busted.test.omega1*t*0.1886019614740855;
```

```
busted.test_Q_component_1[18]
[30]:=busted.test.theta_AT*busted.test.omega1*t*0.2208116300950855;
busted.test_Q_component_1[18]
[34]:=busted.test.theta_CG*busted.test.omega1*t*0.2936069988308121;
busted.test_Q_component_1[19]
[3]:=busted.test.theta_AC*busted.test.omega1*t*0.3102791451089904;
busted.test_Q_component_1[19]
[16]:=busted.test.theta_AT*busted.test.omega1*t*0.4290813980170551;
busted.test_Q_component_1[19]
[17]:=busted.test.theta_CT*t*0.0447809360409677;
busted.test_Q_component_1[19]
[18]:=busted.test.theta_GT*busted.test.omega1*t*0.1063345886498611;
busted.test_Q_component_1[19]
[23]:=busted.test.theta_AC*busted.test.omega1*t*0.2496987119458206;
busted.test_Q_component_1[19]
[27]:=busted.test.theta_AG*busted.test.omega1*t*0.1886019614740855;
busted.test_Q_component_1[19]
[31]:=busted.test.theta_AT*busted.test.omega1*t*0.2208116300950855;
busted.test_Q_component_1[19]
[35]:=busted.test.theta_CG*busted.test.omega1*t*0.2936069988308121;
busted.test_Q_component_1[19]
[49]:=busted.test.theta_CT*busted.test.omega1*t*0.244832889475194;
busted.test_Q_component_1[20]
[4]:=busted.test.theta_AC*busted.test.omega1*t*0.3102791451089904;
busted.test_Q_component_1[20]
[16]:=busted.test.theta_AC*busted.test.omega1*t*0.3408876964850085;
busted.test_Q_component_1[20]
[21]:=busted.test.theta_AC*t*0.0447809360409677;
busted.test_Q_component_1[20]
[22]:=busted.test.theta_AG*t*0.1063345886498611;
busted.test_Q_component_1[20]
[23]:=busted.test.theta_AT*t*0.419803077292116;
busted.test_Q_component_1[20]
[24]:=busted.test.theta_CG*busted.test.omega1*t*0.1886019614740855;
busted.test_Q_component_1[20]
[28]:=busted.test.theta_CT*busted.test.omega1*t*0.2208116300950855;
busted.test_Q_component_1[20]
[36]:=busted.test.theta_CG*busted.test.omega1*t*0.2936069988308121;
busted.test_Q_component_1[20]
[50]:=busted.test.theta_CT*busted.test.omega1*t*0.244832889475194;
busted.test_Q_component_1[21]
[5]:=busted.test.theta_AC*busted.test.omega1*t*0.3102791451089904;
busted.test_Q_component_1[21]
[17]:=busted.test.theta_AC*busted.test.omega1*t*0.3408876964850085;
busted.test_Q_component_1[21]
[20]:=busted.test.theta_AC*t*0.4290813980170551;
busted.test_Q_component_1[21]
[22]:=busted.test.theta_CG*t*0.1063345886498611;
busted.test_Q_component_1[21]
[23]:=busted.test.theta_CT*t*0.419803077292116;
busted.test_Q_component_1[21]
[25]:=busted.test.theta_CG*busted.test.omega1*t*0.1886019614740855;
busted.test_Q_component_1[21]
[29]:=busted.test.theta_CT*busted.test.omega1*t*0.2208116300950855;
```

```
busted.test_Q_component_1[21]
[37]:=busted.test.theta_CG*busted.test.omega1*t*0.2936069988308121;
busted.test_Q_component_1[21]
[51]:=busted.test.theta_CT*busted.test.omega1*t*0.244832889475194;
busted.test_Q_component_1[22]
[6]:=busted.test.theta_AC*busted.test.omega1*t*0.3102791451089904;
busted.test_Q_component_1[22]
[18]:=busted.test.theta_AC*busted.test.omega1*t*0.3408876964850085;
busted.test_Q_component_1[22]
[20]:=busted.test.theta_AG*t*0.4290813980170551;
busted.test_Q_component_1[22]
[21]:=busted.test.theta_CG*t*0.0447809360409677;
busted.test_Q_component_1[22]
[23]:=busted.test.theta_GT*t*0.419803077292116;
busted.test_Q_component_1[22]
[26]:=busted.test.theta_CG*busted.test.omega1*t*0.1886019614740855;
busted.test_Q_component_1[22]
[30]:=busted.test.theta_CT*busted.test.omega1*t*0.2208116300950855;
busted.test_Q_component_1[22]
[38]:=busted.test.theta_CG*busted.test.omega1*t*0.2936069988308121;
busted.test_Q_component_1[22]
[52]:=busted.test.theta_CT*busted.test.omega1*t*0.244832889475194;
busted.test_Q_component_1[23]
[7]:=busted.test.theta_AC*busted.test.omega1*t*0.3102791451089904;
busted.test_Q_component_1[23]
[19]:=busted.test.theta_AC*busted.test.omega1*t*0.3408876964850085;
busted.test_Q_component_1[23]
[20]:=busted.test.theta_AT*t*0.4290813980170551;
busted.test_Q_component_1[23]
[21]:=busted.test.theta_CT*t*0.0447809360409677;
busted.test_Q_component_1[23]
[22]:=busted.test.theta_GT*t*0.1063345886498611;
busted.test_Q_component_1[23]
[27]:=busted.test.theta_CG*busted.test.omega1*t*0.1886019614740855;
busted.test_Q_component_1[23]
[31]:=busted.test.theta_CT*busted.test.omega1*t*0.2208116300950855;
busted.test_Q_component_1[23]
[39]:=busted.test.theta_CG*busted.test.omega1*t*0.2936069988308121;
busted.test_Q_component_1[23]
[53]:=busted.test.theta_CT*busted.test.omega1*t*0.244832889475194;
busted.test_Q_component_1[24]
[8]:=busted.test.theta_AC*t*0.3102791451089904;
busted.test_Q_component_1[24]
[16]:=busted.test.theta_AG*busted.test.omega1*t*0.3408876964850085;
busted.test_Q_component_1[24]
[20]:=busted.test.theta_CG*busted.test.omega1*t*0.2496987119458206;
busted.test_Q_component_1[24]
[25]:=busted.test.theta_AC*t*0.0447809360409677;
busted.test_Q_component_1[24]
[26]:=busted.test.theta_AG*t*0.1063345886498611;
busted.test_Q_component_1[24]
[27]:=busted.test.theta_AT*t*0.419803077292116;
busted.test_Q_component_1[24]
[28]:=busted.test.theta_GT*busted.test.omega1*t*0.2208116300950855;
```

```
busted.test_Q_component_1[24]
[40]:=busted.test.theta_CG*busted.test.omega1*t*0.2936069988308121;
busted.test_Q_component_1[25]
[9]:=busted.test.theta_AC*busted.test.omega1*t*0.3102791451089904;
busted.test_Q_component_1[25]
[17]:=busted.test.theta_AG*busted.test.omega1*t*0.3408876964850085;
busted.test_Q_component_1[25]
[21]:=busted.test.theta_CG*busted.test.omega1*t*0.2496987119458206;
busted.test_Q_component_1[25]
[24]:=busted.test.theta_AC*t*0.4290813980170551;
busted.test_Q_component_1[25]
[26]:=busted.test.theta_CG*t*0.1063345886498611;
busted.test_Q_component_1[25]
[27]:=busted.test.theta_CT*t*0.419803077292116;
busted.test_Q_component_1[25]
[29]:=busted.test.theta_GT*busted.test.omega1*t*0.2208116300950855;
busted.test_Q_component_1[25]
[41]:=busted.test.theta_CG*busted.test.omega1*t*0.2936069988308121;
busted.test_Q_component_1[25]
[54]:=busted.test.theta_CT*busted.test.omega1*t*0.244832889475194;
busted.test_Q_component_1[26]
[10]:=busted.test.theta_AC*t*0.3102791451089904;
busted.test_Q_component_1[26]
[18]:=busted.test.theta_AG*busted.test.omega1*t*0.3408876964850085;
busted.test_Q_component_1[26]
[22]:=busted.test.theta_CG*busted.test.omega1*t*0.2496987119458206;
busted.test_Q_component_1[26]
[24]:=busted.test.theta_AG*t*0.4290813980170551;
busted.test_Q_component_1[26]
[25]:=busted.test.theta_CG*t*0.0447809360409677;
busted.test_Q_component_1[26]
[27]:=busted.test.theta_GT*t*0.419803077292116;
busted.test_Q_component_1[26]
[30]:=busted.test.theta_GT*busted.test.omega1*t*0.2208116300950855;
busted.test_Q_component_1[26]
[42]:=busted.test.theta_CG*busted.test.omega1*t*0.2936069988308121;
busted.test_Q_component_1[26]
[55]:=busted.test.theta_CT*busted.test.omega1*t*0.244832889475194;
busted.test_Q_component_1[27]
[11]:=busted.test.theta_AC*busted.test.omega1*t*0.3102791451089904;
busted.test_Q_component_1[27]
[19]:=busted.test.theta_AG*busted.test.omega1*t*0.3408876964850085;
busted.test_Q_component_1[27]
[23]:=busted.test.theta_CG*busted.test.omega1*t*0.2496987119458206;
busted.test_Q_component_1[27]
[24]:=busted.test.theta_AT*t*0.4290813980170551;
busted.test_Q_component_1[27]
[25]:=busted.test.theta_CT*t*0.0447809360409677;
busted.test_Q_component_1[27]
[26]:=busted.test.theta_GT*t*0.1063345886498611;
busted.test_Q_component_1[27]
[31]:=busted.test.theta_GT*busted.test.omega1*t*0.2208116300950855;
busted.test_Q_component_1[27]
[43]:=busted.test.theta_CG*busted.test.omega1*t*0.2936069988308121;
```

```
busted.test_Q_component_1[27]
[56]:=busted.test.theta_CT*busted.test.omega1*t*0.244832889475194;
busted.test_Q_component_1[28]
[12]:=busted.test.theta_AC*busted.test.omega1*t*0.3102791451089904;
busted.test_Q_component_1[28]
[16]:=busted.test.theta_AT*busted.test.omega1*t*0.3408876964850085;
busted.test_Q_component_1[28]
[20]:=busted.test.theta_CT*busted.test.omega1*t*0.2496987119458206;
busted.test_Q_component_1[28]
[24]:=busted.test.theta_GT*busted.test.omega1*t*0.1886019614740855;
busted.test_Q_component_1[28]
[29]:=busted.test.theta_AC*t*0.0447809360409677;
busted.test_Q_component_1[28]
[30]:=busted.test.theta_AG*t*0.1063345886498611;
busted.test_Q_component_1[28]
[31]:=busted.test.theta_AT*t*0.419803077292116;
busted.test_Q_component_1[28]
[44]:=busted.test.theta_CG*busted.test.omega1*t*0.2936069988308121;
busted.test_Q_component_1[28]
[57]:=busted.test.theta_CT*t*0.244832889475194;
busted.test_Q_component_1[29]
[13]:=busted.test.theta_AC*busted.test.omega1*t*0.3102791451089904;
busted.test_Q_component_1[29]
[17]:=busted.test.theta_AT*busted.test.omega1*t*0.3408876964850085;
busted.test_Q_component_1[29]
[21]:=busted.test.theta_CT*busted.test.omega1*t*0.2496987119458206;
busted.test_Q_component_1[29]
[25]:=busted.test.theta_GT*busted.test.omega1*t*0.1886019614740855;
busted.test_Q_component_1[29]
[28]:=busted.test.theta_AC*t*0.4290813980170551;
busted.test_Q_component_1[29]
[30]:=busted.test.theta_CG*t*0.1063345886498611;
busted.test_Q_component_1[29]
[31]:=busted.test.theta_CT*t*0.419803077292116;
busted.test_Q_component_1[29]
[45]:=busted.test.theta_CG*busted.test.omega1*t*0.2936069988308121;
busted.test_Q_component_1[29]
[58]:=busted.test.theta_CT*busted.test.omega1*t*0.244832889475194;
busted.test_Q_component_1[30]
[14]:=busted.test.theta_AC*busted.test.omega1*t*0.3102791451089904;
busted.test_Q_component_1[30]
[18]:=busted.test.theta_AT*busted.test.omega1*t*0.3408876964850085;
busted.test_Q_component_1[30]
[22]:=busted.test.theta_CT*busted.test.omega1*t*0.2496987119458206;
busted.test_Q_component_1[30]
[26]:=busted.test.theta_GT*busted.test.omega1*t*0.1886019614740855;
busted.test_Q_component_1[30]
[28]:=busted.test.theta_AG*t*0.4290813980170551;
busted.test_Q_component_1[30]
[29]:=busted.test.theta_CG*t*0.0447809360409677;
busted.test_Q_component_1[30]
[31]:=busted.test.theta_GT*t*0.419803077292116;
busted.test_Q_component_1[30]
[46]:=busted.test.theta_CG*busted.test.omega1*t*0.2936069988308121;
```

```
busted.test_Q_component_1[30]
[59]:=busted.test.theta_CT*t*0.244832889475194;
busted.test_Q_component_1[31]
[15]:=busted.test.theta_AC*busted.test.omega1*t*0.3102791451089904;
busted.test_Q_component_1[31]
[19]:=busted.test.theta_AT*busted.test.omega1*t*0.3408876964850085;
busted.test_Q_component_1[31]
[23]:=busted.test.theta_CT*busted.test.omega1*t*0.2496987119458206;
busted.test_Q_component_1[31]
[27]:=busted.test.theta_GT*busted.test.omega1*t*0.1886019614740855;
busted.test_Q_component_1[31]
[28]:=busted.test.theta_AT*t*0.4290813980170551;
busted.test_Q_component_1[31]
[29]:=busted.test.theta_CT*t*0.0447809360409677;
busted.test_Q_component_1[31]
[30]:=busted.test.theta_GT*t*0.1063345886498611;
busted.test_Q_component_1[31]
[47]:=busted.test.theta_CG*busted.test.omega1*t*0.2936069988308121;
busted.test_Q_component_1[31]
[60]:=busted.test.theta_CT*busted.test.omega1*t*0.244832889475194;
busted.test_Q_component_1[32]
[0]:=busted.test.theta_AG*busted.test.omega1*t*0.3102791451089904;
busted.test_Q_component_1[32]
[16]:=busted.test.theta_CG*busted.test.omega1*t*0.1512809665850036;
busted.test_Q_component_1[32]
[33]:=busted.test.theta_AC*busted.test.omega1*t*0.0447809360409677;
busted.test_Q_component_1[32]
[34]:=busted.test.theta_AG*t*0.1063345886498611;
busted.test_Q_component_1[32]
[35]:=busted.test.theta_AT*busted.test.omega1*t*0.419803077292116;
busted.test_Q_component_1[32]
[36]:=busted.test.theta_AC*busted.test.omega1*t*0.2496987119458206;
busted.test_Q_component_1[32]
[40]:=busted.test.theta_AG*busted.test.omega1*t*0.1886019614740855;
busted.test_Q_component_1[32]
[44]:=busted.test.theta_AT*busted.test.omega1*t*0.2208116300950855;
busted.test_Q_component_1[33]
[1]:=busted.test.theta_AG*busted.test.omega1*t*0.3102791451089904;
busted.test_Q_component_1[33]
[17]:=busted.test.theta_CG*busted.test.omega1*t*0.1512809665850036;
busted.test_Q_component_1[33]
[32]:=busted.test.theta_AC*busted.test.omega1*t*0.4290813980170551;
busted.test_Q_component_1[33]
[34]:=busted.test.theta_CG*busted.test.omega1*t*0.1063345886498611;
busted.test_Q_component_1[33]
[35]:=busted.test.theta_CT*t*0.419803077292116;
busted.test_Q_component_1[33]
[37]:=busted.test.theta_AC*busted.test.omega1*t*0.2496987119458206;
busted.test_Q_component_1[33]
[41]:=busted.test.theta_AG*busted.test.omega1*t*0.1886019614740855;
busted.test_Q_component_1[33]
[45]:=busted.test.theta_AT*busted.test.omega1*t*0.2208116300950855;
busted.test_Q_component_1[33]
[48]:=busted.test.theta_GT*busted.test.omega1*t*0.244832889475194;
```

```
busted.test_Q_component_1[34]
[2]:=busted.test.theta_AG*busted.test.omega1*t*0.3102791451089904;
busted.test_Q_component_1[34]
[18]:=busted.test.theta_CG*busted.test.omega1*t*0.1512809665850036;
busted.test_Q_component_1[34]
[32]:=busted.test.theta_AG*t*0.4290813980170551;
busted.test_Q_component_1[34]
[33]:=busted.test.theta_CG*busted.test.omega1*t*0.0447809360409677;
busted.test_Q_component_1[34]
[35]:=busted.test.theta_GT*busted.test.omega1*t*0.419803077292116;
busted.test_Q_component_1[34]
[38]:=busted.test.theta_AC*busted.test.omega1*t*0.2496987119458206;
busted.test_Q_component_1[34]
[42]:=busted.test.theta_AG*busted.test.omega1*t*0.1886019614740855;
busted.test_Q_component_1[34]
[46]:=busted.test.theta_AT*busted.test.omega1*t*0.2208116300950855;
busted.test_Q_component_1[35]
[3]:=busted.test.theta_AG*busted.test.omega1*t*0.3102791451089904;
busted.test_Q_component_1[35]
[19]:=busted.test.theta_CG*busted.test.omega1*t*0.1512809665850036;
busted.test_Q_component_1[35]
[32]:=busted.test.theta_AT*busted.test.omega1*t*0.4290813980170551;
busted.test_Q_component_1[35]
[33]:=busted.test.theta_CT*t*0.0447809360409677;
busted.test_Q_component_1[35]
[34]:=busted.test.theta_GT*busted.test.omega1*t*0.1063345886498611;
busted.test_Q_component_1[35]
[39]:=busted.test.theta_AC*busted.test.omega1*t*0.2496987119458206;
busted.test_Q_component_1[35]
[43]:=busted.test.theta_AG*busted.test.omega1*t*0.1886019614740855;
busted.test_Q_component_1[35]
[47]:=busted.test.theta_AT*busted.test.omega1*t*0.2208116300950855;
busted.test_Q_component_1[35]
[49]:=busted.test.theta_GT*busted.test.omega1*t*0.244832889475194;
busted.test_Q_component_1[36]
[4]:=busted.test.theta_AG*busted.test.omega1*t*0.3102791451089904;
busted.test_Q_component_1[36]
[20]:=busted.test.theta_CG*busted.test.omega1*t*0.1512809665850036;
busted.test_Q_component_1[36]
[32]:=busted.test.theta_AC*busted.test.omega1*t*0.3408876964850085;
busted.test_Q_component_1[36]
[37]:=busted.test.theta_AC*t*0.0447809360409677;
busted.test_Q_component_1[36]
[38]:=busted.test.theta_AG*t*0.1063345886498611;
busted.test_Q_component_1[36]
[39]:=busted.test.theta_AT*t*0.419803077292116;
busted.test_Q_component_1[36]
[40]:=busted.test.theta_CG*busted.test.omega1*t*0.1886019614740855;
busted.test_Q_component_1[36]
[44]:=busted.test.theta_CT*busted.test.omega1*t*0.2208116300950855;
busted.test_Q_component_1[36]
[50]:=busted.test.theta_GT*busted.test.omega1*t*0.244832889475194;
busted.test_Q_component_1[37]
[5]:=busted.test.theta_AG*busted.test.omega1*t*0.3102791451089904;
```

```
busted.test_Q_component_1[37]
[21]:=busted.test.theta_CG*busted.test.omega1*t*0.1512809665850036;
busted.test_Q_component_1[37]
[33]:=busted.test.theta_AC*busted.test.omega1*t*0.3408876964850085;
busted.test_Q_component_1[37]
[36]:=busted.test.theta_AC*t*0.4290813980170551;
busted.test_Q_component_1[37]
[38]:=busted.test.theta_CG*t*0.1063345886498611;
busted.test_Q_component_1[37]
[39]:=busted.test.theta_CT*t*0.419803077292116;
busted.test_Q_component_1[37]
[41]:=busted.test.theta_CG*busted.test.omega1*t*0.1886019614740855;
busted.test_Q_component_1[37]
[45]:=busted.test.theta_CT*busted.test.omega1*t*0.2208116300950855;
busted.test_Q_component_1[37]
[51]:=busted.test.theta_GT*busted.test.omega1*t*0.244832889475194;
busted.test_Q_component_1[38]
[6]:=busted.test.theta_AG*busted.test.omega1*t*0.3102791451089904;
busted.test_Q_component_1[38]
[22]:=busted.test.theta_CG*busted.test.omega1*t*0.1512809665850036;
busted.test_Q_component_1[38]
[34]:=busted.test.theta_AC*busted.test.omega1*t*0.3408876964850085;
busted.test_Q_component_1[38]
[36]:=busted.test.theta_AG*t*0.4290813980170551;
busted.test_Q_component_1[38]
[37]:=busted.test.theta_CG*t*0.0447809360409677;
busted.test_Q_component_1[38]
[39]:=busted.test.theta_GT*t*0.419803077292116;
busted.test_Q_component_1[38]
[42]:=busted.test.theta_CG*busted.test.omega1*t*0.1886019614740855;
busted.test_Q_component_1[38]
[46]:=busted.test.theta_CT*busted.test.omega1*t*0.2208116300950855;
busted.test_Q_component_1[38]
[52]:=busted.test.theta_GT*busted.test.omega1*t*0.244832889475194;
busted.test_Q_component_1[39]
[7]:=busted.test.theta_AG*busted.test.omega1*t*0.3102791451089904;
busted.test_Q_component_1[39]
[23]:=busted.test.theta_CG*busted.test.omega1*t*0.1512809665850036;
busted.test_Q_component_1[39]
[35]:=busted.test.theta_AC*busted.test.omega1*t*0.3408876964850085;
busted.test_Q_component_1[39]
[36]:=busted.test.theta_AT*t*0.4290813980170551;
busted.test_Q_component_1[39]
[37]:=busted.test.theta_CT*t*0.0447809360409677;
busted.test_Q_component_1[39]
[38]:=busted.test.theta_GT*t*0.1063345886498611;
busted.test_Q_component_1[39]
[43]:=busted.test.theta_CG*busted.test.omega1*t*0.1886019614740855;
busted.test_Q_component_1[39]
[47]:=busted.test.theta_CT*busted.test.omega1*t*0.2208116300950855;
busted.test_Q_component_1[39]
[53]:=busted.test.theta_GT*busted.test.omega1*t*0.244832889475194;
busted.test_Q_component_1[40]
[8]:=busted.test.theta_AG*busted.test.omega1*t*0.3102791451089904;
```

```
busted.test_Q_component_1[40]
[24]:=busted.test.theta_CG*busted.test.omega1*t*0.1512809665850036;
busted.test_Q_component_1[40]
[32]:=busted.test.theta_AG*busted.test.omega1*t*0.3408876964850085;
busted.test_Q_component_1[40]
[36]:=busted.test.theta_CG*busted.test.omega1*t*0.2496987119458206;
busted.test_Q_component_1[40]
[41]:=busted.test.theta_AC*t*0.0447809360409677;
busted.test_Q_component_1[40]
[42]:=busted.test.theta_AG*t*0.1063345886498611;
busted.test_Q_component_1[40]
[43]:=busted.test.theta_AT*t*0.419803077292116;
busted.test_Q_component_1[40]
[44]:=busted.test.theta_GT*busted.test.omega1*t*0.2208116300950855;
busted.test_Q_component_1[41]
[9]:=busted.test.theta_AG*busted.test.omega1*t*0.3102791451089904;
busted.test_Q_component_1[41]
[25]:=busted.test.theta_CG*busted.test.omega1*t*0.1512809665850036;
busted.test_Q_component_1[41]
[33]:=busted.test.theta_AG*busted.test.omega1*t*0.3408876964850085;
busted.test_Q_component_1[41]
[37]:=busted.test.theta_CG*busted.test.omega1*t*0.2496987119458206;
busted.test_Q_component_1[41]
[40]:=busted.test.theta_AC*t*0.4290813980170551;
busted.test_Q_component_1[41]
[42]:=busted.test.theta_CG*t*0.1063345886498611;
busted.test_Q_component_1[41]
[43]:=busted.test.theta_CT*t*0.419803077292116;
busted.test_Q_component_1[41]
[45]:=busted.test.theta_GT*busted.test.omega1*t*0.2208116300950855;
busted.test_Q_component_1[41]
[54]:=busted.test.theta_GT*busted.test.omega1*t*0.244832889475194;
busted.test_Q_component_1[42]
[10]:=busted.test.theta_AG*busted.test.omega1*t*0.3102791451089904;
busted.test_Q_component_1[42]
[26]:=busted.test.theta_CG*busted.test.omega1*t*0.1512809665850036;
busted.test_Q_component_1[42]
[34]:=busted.test.theta_AG*busted.test.omega1*t*0.3408876964850085;
busted.test_Q_component_1[42]
[38]:=busted.test.theta_CG*busted.test.omega1*t*0.2496987119458206;
busted.test_Q_component_1[42]
[40]:=busted.test.theta_AG*t*0.4290813980170551;
busted.test_Q_component_1[42]
[41]:=busted.test.theta_CG*t*0.0447809360409677;
busted.test_Q_component_1[42]
[43]:=busted.test.theta_GT*t*0.419803077292116;
busted.test_Q_component_1[42]
[46]:=busted.test.theta_GT*busted.test.omega1*t*0.2208116300950855;
busted.test_Q_component_1[42]
[55]:=busted.test.theta_GT*busted.test.omega1*t*0.244832889475194;
busted.test_Q_component_1[43]
[11]:=busted.test.theta_AG*busted.test.omega1*t*0.3102791451089904;
busted.test_Q_component_1[43]
[27]:=busted.test.theta_CG*busted.test.omega1*t*0.1512809665850036;
```

```
busted.test_Q_component_1[43]
[35]:=busted.test.theta_AG*busted.test.omega1*t*0.3408876964850085;
busted.test_Q_component_1[43]
[39]:=busted.test.theta_CG*busted.test.omega1*t*0.2496987119458206;
busted.test_Q_component_1[43]
[40]:=busted.test.theta_AT*t*0.4290813980170551;
busted.test_Q_component_1[43]
[41]:=busted.test.theta_CT*t*0.0447809360409677;
busted.test_Q_component_1[43]
[42]:=busted.test.theta_GT*t*0.1063345886498611;
busted.test_Q_component_1[43]
[47]:=busted.test.theta_GT*busted.test.omega1*t*0.2208116300950855;
busted.test_Q_component_1[43]
[56]:=busted.test.theta_GT*busted.test.omega1*t*0.244832889475194;
busted.test_Q_component_1[44]
[12]:=busted.test.theta_AG*busted.test.omega1*t*0.3102791451089904;
busted.test_Q_component_1[44]
[28]:=busted.test.theta_CG*busted.test.omega1*t*0.1512809665850036;
busted.test_Q_component_1[44]
[32]:=busted.test.theta_AT*busted.test.omega1*t*0.3408876964850085;
busted.test_Q_component_1[44]
[36]:=busted.test.theta_CT*busted.test.omega1*t*0.2496987119458206;
busted.test_Q_component_1[44]
[40]:=busted.test.theta_GT*busted.test.omega1*t*0.1886019614740855;
busted.test_Q_component_1[44]
[45]:=busted.test.theta_AC*t*0.0447809360409677;
busted.test_Q_component_1[44]
[46]:=busted.test.theta_AG*t*0.1063345886498611;
busted.test_Q_component_1[44]
[47]:=busted.test.theta_AT*t*0.419803077292116;
busted.test_Q_component_1[44]
[57]:=busted.test.theta_GT*busted.test.omega1*t*0.244832889475194;
busted.test_Q_component_1[45]
[13]:=busted.test.theta_AG*busted.test.omega1*t*0.3102791451089904;
busted.test_Q_component_1[45]
[29]:=busted.test.theta_CG*busted.test.omega1*t*0.1512809665850036;
busted.test_Q_component_1[45]
[33]:=busted.test.theta_AT*busted.test.omega1*t*0.3408876964850085;
busted.test_Q_component_1[45]
[37]:=busted.test.theta_CT*busted.test.omega1*t*0.2496987119458206;
busted.test_Q_component_1[45]
[41]:=busted.test.theta_GT*busted.test.omega1*t*0.1886019614740855;
busted.test_Q_component_1[45]
[44]:=busted.test.theta_AC*t*0.4290813980170551;
busted.test_Q_component_1[45]
[46]:=busted.test.theta_CG*t*0.1063345886498611;
busted.test_Q_component_1[45]
[47]:=busted.test.theta_CT*t*0.419803077292116;
busted.test_Q_component_1[45]
[58]:=busted.test.theta_GT*busted.test.omega1*t*0.244832889475194;
busted.test_Q_component_1[46]
[14]:=busted.test.theta_AG*busted.test.omega1*t*0.3102791451089904;
busted.test_Q_component_1[46]
[30]:=busted.test.theta_CG*busted.test.omega1*t*0.1512809665850036;
```

```
busted.test_Q_component_1[46]
[34]:=busted.test.theta_AT*busted.test.omega1*t*0.3408876964850085;
busted.test_Q_component_1[46]
[38]:=busted.test.theta_CT*busted.test.omega1*t*0.2496987119458206;
busted.test_Q_component_1[46]
[42]:=busted.test.theta_GT*busted.test.omega1*t*0.1886019614740855;
busted.test_Q_component_1[46]
[44]:=busted.test.theta_AG*t*0.4290813980170551;
busted.test_Q_component_1[46]
[45]:=busted.test.theta_CG*t*0.0447809360409677;
busted.test_Q_component_1[46]
[47]:=busted.test.theta_GT*t*0.419803077292116;
busted.test_Q_component_1[46]
[59]:=busted.test.theta_GT*busted.test.omega1*t*0.244832889475194;
busted.test_Q_component_1[47]
[15]:=busted.test.theta_AG*busted.test.omega1*t*0.3102791451089904;
busted.test_Q_component_1[47]
[31]:=busted.test.theta_CG*busted.test.omega1*t*0.1512809665850036;
busted.test_Q_component_1[47]
[35]:=busted.test.theta_AT*busted.test.omega1*t*0.3408876964850085;
busted.test_Q_component_1[47]
[39]:=busted.test.theta_CT*busted.test.omega1*t*0.2496987119458206;
busted.test_Q_component_1[47]
[43]:=busted.test.theta_GT*busted.test.omega1*t*0.1886019614740855;
busted.test_Q_component_1[47]
[44]:=busted.test.theta_AT*t*0.4290813980170551;
busted.test_Q_component_1[47]
[45]:=busted.test.theta_CT*t*0.0447809360409677;
busted.test_Q_component_1[47]
[46]:=busted.test.theta_GT*t*0.1063345886498611;
busted.test_Q_component_1[47]
[60]:=busted.test.theta_GT*busted.test.omega1*t*0.244832889475194;
busted.test_Q_component_1[48]
[1]:=busted.test.theta_AT*busted.test.omega1*t*0.3102791451089904;
busted.test_Q_component_1[48]
[17]:=busted.test.theta_CT*busted.test.omega1*t*0.1512809665850036;
busted.test_Q_component_1[48]
[33]:=busted.test.theta_GT*busted.test.omega1*t*0.2936069988308121;
busted.test_Q_component_1[48]
[49]:=busted.test.theta_CT*t*0.419803077292116;
busted.test_Q_component_1[48]
[51]:=busted.test.theta_AC*busted.test.omega1*t*0.2496987119458206;
busted.test_Q_component_1[48]
[54]:=busted.test.theta_AG*busted.test.omega1*t*0.1886019614740855;
busted.test_Q_component_1[48]
[58]:=busted.test.theta_AT*busted.test.omega1*t*0.2208116300950855;
busted.test_Q_component_1[49]
[3]:=busted.test.theta_AT*busted.test.omega1*t*0.3102791451089904;
busted.test_Q_component_1[49]
[19]:=busted.test.theta_CT*busted.test.omega1*t*0.1512809665850036;
busted.test_Q_component_1[49]
[35]:=busted.test.theta_GT*busted.test.omega1*t*0.2936069988308121;
busted.test_Q_component_1[49]
[48]:=busted.test.theta_CT*t*0.0447809360409677;
```

```
busted.test_Q_component_1[49]
[53]:=busted.test.theta_AC*busted.test.omega1*t*0.2496987119458206;
busted.test_Q_component_1[49]
[56]:=busted.test.theta_AG*busted.test.omega1*t*0.1886019614740855;
busted.test_Q_component_1[49]
[60]:=busted.test.theta_AT*busted.test.omega1*t*0.2208116300950855;
busted.test_Q_component_1[50]
[4]:=busted.test.theta_AT*busted.test.omega1*t*0.3102791451089904;
busted.test_Q_component_1[50]
[20]:=busted.test.theta_CT*busted.test.omega1*t*0.1512809665850036;
busted.test_Q_component_1[50]
[36]:=busted.test.theta_GT*busted.test.omega1*t*0.2936069988308121;
busted.test_Q_component_1[50]
[51]:=busted.test.theta_AC*t*0.0447809360409677;
busted.test_Q_component_1[50]
[52]:=busted.test.theta_AG*t*0.1063345886498611;
busted.test_Q_component_1[50]
[53]:=busted.test.theta_AT*t*0.419803077292116;
busted.test_Q_component_1[50]
[57]:=busted.test.theta_CT*busted.test.omega1*t*0.2208116300950855;
busted.test_Q_component_1[51]
[5]:=busted.test.theta_AT*busted.test.omega1*t*0.3102791451089904;
busted.test_Q_component_1[51]
[21]:=busted.test.theta_CT*busted.test.omega1*t*0.1512809665850036;
busted.test_Q_component_1[51]
[37]:=busted.test.theta_GT*busted.test.omega1*t*0.2936069988308121;
busted.test_Q_component_1[51]
[48]:=busted.test.theta_AC*busted.test.omega1*t*0.3408876964850085;
busted.test_Q_component_1[51]
[50]:=busted.test.theta_AC*t*0.4290813980170551;
busted.test_Q_component_1[51]
[52]:=busted.test.theta_CG*t*0.1063345886498611;
busted.test_Q_component_1[51]
[53]:=busted.test.theta_CT*t*0.419803077292116;
busted.test_Q_component_1[51]
[54]:=busted.test.theta_CG*busted.test.omega1*t*0.1886019614740855;
busted.test_Q_component_1[51]
[58]:=busted.test.theta_CT*busted.test.omega1*t*0.2208116300950855;
busted.test_Q_component_1[52]
[6]:=busted.test.theta_AT*busted.test.omega1*t*0.3102791451089904;
busted.test_Q_component_1[52]
[22]:=busted.test.theta_CT*busted.test.omega1*t*0.1512809665850036;
busted.test_Q_component_1[52]
[38]:=busted.test.theta_GT*busted.test.omega1*t*0.2936069988308121;
busted.test_Q_component_1[52]
[50]:=busted.test.theta_AG*t*0.4290813980170551;
busted.test_Q_component_1[52]
[51]:=busted.test.theta_CG*t*0.0447809360409677;
busted.test_Q_component_1[52]
[53]:=busted.test.theta_GT*t*0.419803077292116;
busted.test_Q_component_1[52]
[55]:=busted.test.theta_CG*busted.test.omega1*t*0.1886019614740855;
busted.test_Q_component_1[52]
[59]:=busted.test.theta_CT*busted.test.omega1*t*0.2208116300950855;
```

```
busted.test_Q_component_1[53]
[7]:=busted.test.theta_AT*busted.test.omega1*t*0.3102791451089904;
busted.test_Q_component_1[53]
[23]:=busted.test.theta_CT*busted.test.omega1*t*0.1512809665850036;
busted.test_Q_component_1[53]
[39]:=busted.test.theta_GT*busted.test.omega1*t*0.2936069988308121;
busted.test_Q_component_1[53]
[49]:=busted.test.theta_AC*busted.test.omega1*t*0.3408876964850085;
busted.test_Q_component_1[53]
[50]:=busted.test.theta_AT*t*0.4290813980170551;
busted.test_Q_component_1[53]
[51]:=busted.test.theta_CT*t*0.0447809360409677;
busted.test_Q_component_1[53]
[52]:=busted.test.theta_GT*t*0.1063345886498611;
busted.test_Q_component_1[53]
[56]:=busted.test.theta_CG*busted.test.omega1*t*0.1886019614740855;
busted.test_Q_component_1[53]
[60]:=busted.test.theta_CT*busted.test.omega1*t*0.2208116300950855;
busted.test_Q_component_1[54]
[9]:=busted.test.theta_AT*busted.test.omega1*t*0.3102791451089904;
busted.test_Q_component_1[54]
[25]:=busted.test.theta_CT*busted.test.omega1*t*0.1512809665850036;
busted.test_Q_component_1[54]
[41]:=busted.test.theta_GT*busted.test.omega1*t*0.2936069988308121;
busted.test_Q_component_1[54]
[48]:=busted.test.theta_AG*busted.test.omega1*t*0.3408876964850085;
busted.test_Q_component_1[54]
[51]:=busted.test.theta_CG*busted.test.omega1*t*0.2496987119458206;
busted.test_Q_component_1[54]
[55]:=busted.test.theta_CG*busted.test.omega1*t*0.1063345886498611;
busted.test_Q_component_1[54]
[56]:=busted.test.theta_CT*t*0.419803077292116;
busted.test_Q_component_1[54]
[58]:=busted.test.theta_GT*busted.test.omega1*t*0.2208116300950855;
busted.test_Q_component_1[55]
[10]:=busted.test.theta_AT*busted.test.omega1*t*0.3102791451089904;
busted.test_Q_component_1[55]
[26]:=busted.test.theta_CT*busted.test.omega1*t*0.1512809665850036;
busted.test_Q_component_1[55]
[42]:=busted.test.theta_GT*busted.test.omega1*t*0.2936069988308121;
busted.test_Q_component_1[55]
[52]:=busted.test.theta_CG*busted.test.omega1*t*0.2496987119458206;
busted.test_Q_component_1[55]
[54]:=busted.test.theta_CG*busted.test.omega1*t*0.0447809360409677;
busted.test_Q_component_1[55]
[56]:=busted.test.theta_GT*busted.test.omega1*t*0.419803077292116;
busted.test_Q_component_1[55]
[59]:=busted.test.theta_GT*busted.test.omega1*t*0.2208116300950855;
busted.test_Q_component_1[56]
[11]:=busted.test.theta_AT*busted.test.omega1*t*0.3102791451089904;
busted.test_Q_component_1[56]
[27]:=busted.test.theta_CT*busted.test.omega1*t*0.1512809665850036;
busted.test_Q_component_1[56]
[43]:=busted.test.theta_GT*busted.test.omega1*t*0.2936069988308121;
```

```
busted.test_Q_component_1[56]
[49]:=busted.test.theta_AG*busted.test.omega1*t*0.3408876964850085;
busted.test_Q_component_1[56]
[53]:=busted.test.theta_CG*busted.test.omega1*t*0.2496987119458206;
busted.test_Q_component_1[56]
[54]:=busted.test.theta_CT*t*0.0447809360409677;
busted.test_Q_component_1[56]
[55]:=busted.test.theta_GT*busted.test.omega1*t*0.1063345886498611;
busted.test_Q_component_1[56]
[60]:=busted.test.theta_GT*busted.test.omega1*t*0.2208116300950855;
busted.test_Q_component_1[57]
[12]:=busted.test.theta_AT*busted.test.omega1*t*0.3102791451089904;
busted.test_Q_component_1[57]
[28]:=busted.test.theta_CT*t*0.1512809665850036;
busted.test_Q_component_1[57]
[44]:=busted.test.theta_GT*busted.test.omega1*t*0.2936069988308121;
busted.test_Q_component_1[57]
[50]:=busted.test.theta_CT*busted.test.omega1*t*0.2496987119458206;
busted.test_Q_component_1[57]
[58]:=busted.test.theta_AC*busted.test.omega1*t*0.0447809360409677;
busted.test_Q_component_1[57]
[59]:=busted.test.theta_AG*t*0.1063345886498611;
busted.test_Q_component_1[57]
[60]:=busted.test.theta_AT*busted.test.omega1*t*0.419803077292116;
busted.test_Q_component_1[58]
[13]:=busted.test.theta_AT*busted.test.omega1*t*0.3102791451089904;
busted.test_Q_component_1[58]
[29]:=busted.test.theta_CT*busted.test.omega1*t*0.1512809665850036;
busted.test_Q_component_1[58]
[45]:=busted.test.theta_GT*busted.test.omega1*t*0.2936069988308121;
busted.test_Q_component_1[58]
[48]:=busted.test.theta_AT*busted.test.omega1*t*0.3408876964850085;
busted.test_Q_component_1[58]
[51]:=busted.test.theta_CT*busted.test.omega1*t*0.2496987119458206;
busted.test_Q_component_1[58]
[54]:=busted.test.theta_GT*busted.test.omega1*t*0.1886019614740855;
busted.test_Q_component_1[58]
[57]:=busted.test.theta_AC*busted.test.omega1*t*0.4290813980170551;
busted.test_Q_component_1[58]
[59]:=busted.test.theta_CG*busted.test.omega1*t*0.1063345886498611;
busted.test_Q_component_1[58]
[60]:=busted.test.theta_CT*t*0.419803077292116;
busted.test_Q_component_1[59]
[14]:=busted.test.theta_AT*busted.test.omega1*t*0.3102791451089904;
busted.test_Q_component_1[59]
[30]:=busted.test.theta_CT*t*0.1512809665850036;
busted.test_Q_component_1[59]
[46]:=busted.test.theta_GT*busted.test.omega1*t*0.2936069988308121;
busted.test_Q_component_1[59]
[52]:=busted.test.theta_CT*busted.test.omega1*t*0.2496987119458206;
busted.test_Q_component_1[59]
[55]:=busted.test.theta_GT*busted.test.omega1*t*0.1886019614740855;
busted.test_Q_component_1[59]
[57]:=busted.test.theta_AG*t*0.4290813980170551;
```

```
busted.test_Q_component_1[59]
[58]:=busted.test.theta_CG*busted.test.omega1*t*0.0447809360409677;
busted.test_Q_component_1[59]
[60]:=busted.test.theta_GT*busted.test.omega1*t*0.419803077292116;
busted.test_Q_component_1[60]
[15]:=busted.test.theta_AT*busted.test.omega1*t*0.3102791451089904;
busted.test_Q_component_1[60]
[31]:=busted.test.theta_CT*busted.test.omega1*t*0.1512809665850036;
busted.test_Q_component_1[60]
[47]:=busted.test.theta_GT*busted.test.omega1*t*0.2936069988308121;
busted.test_Q_component_1[60]
[49]:=busted.test.theta_AT*busted.test.omega1*t*0.3408876964850085;
busted.test_Q_component_1[60]
[53]:=busted.test.theta_CT*busted.test.omega1*t*0.2496987119458206;
busted.test_Q_component_1[60]
[56]:=busted.test.theta_GT*busted.test.omega1*t*0.1886019614740855;
busted.test_Q_component_1[60]
[57]:=busted.test.theta_AT*busted.test.omega1*t*0.4290813980170551;
busted.test_Q_component_1[60]
[58]:=busted.test.theta_CT*t*0.0447809360409677;
busted.test_Q_component_1[60]
[59]:=busted.test.theta_GT*busted.test.omega1*t*0.1063345886498611;


busted.test_Q_component_2={61,61};
busted.test_Q_component_2[0]
[1]:=busted.test.theta_AC*busted.test.omega2*t*0.0447809360409677;
busted.test_Q_component_2[0]
[2]:=busted.test.theta_AG*t*0.1063345886498611;
busted.test_Q_component_2[0]
[3]:=busted.test.theta_AT*busted.test.omega2*t*0.419803077292116;
busted.test_Q_component_2[0]
[4]:=busted.test.theta_AC*busted.test.omega2*t*0.2496987119458206;
busted.test_Q_component_2[0]
[8]:=busted.test.theta_AG*busted.test.omega2*t*0.1886019614740855;
busted.test_Q_component_2[0]
[12]:=busted.test.theta_AT*busted.test.omega2*t*0.2208116300950855;
busted.test_Q_component_2[0]
[16]:=busted.test.theta_AC*busted.test.omega2*t*0.1512809665850036;
busted.test_Q_component_2[0]
[32]:=busted.test.theta_AG*busted.test.omega2*t*0.2936069988308121;
busted.test_Q_component_2[1]
[0]:=busted.test.theta_AC*busted.test.omega2*t*0.4290813980170551;
busted.test_Q_component_2[1]
[2]:=busted.test.theta_CG*busted.test.omega2*t*0.1063345886498611;
busted.test_Q_component_2[1]
[3]:=busted.test.theta_CT*t*0.419803077292116;
busted.test_Q_component_2[1]
[5]:=busted.test.theta_AC*busted.test.omega2*t*0.2496987119458206;
busted.test_Q_component_2[1]
[9]:=busted.test.theta_AG*busted.test.omega2*t*0.1886019614740855;
busted.test_Q_component_2[1]
[13]:=busted.test.theta_AT*busted.test.omega2*t*0.2208116300950855;
busted.test_Q_component_2[1]
```

```
[17]:=busted.test.theta_AC*busted.test.omega2*t*0.1512809665850036;
busted.test_Q_component_2[1]
[33]:=busted.test.theta_AG*busted.test.omega2*t*0.2936069988308121;
busted.test_Q_component_2[1]
[48]:=busted.test.theta_AT*busted.test.omega2*t*0.244832889475194;
busted.test_Q_component_2[2]
[0]:=busted.test.theta_AG*t*0.4290813980170551;
busted.test_Q_component_2[2]
[1]:=busted.test.theta_CG*busted.test.omega2*t*0.0447809360409677;
busted.test_Q_component_2[2]
[3]:=busted.test.theta_GT*busted.test.omega2*t*0.419803077292116;
busted.test_Q_component_2[2]
[6]:=busted.test.theta_AC*busted.test.omega2*t*0.2496987119458206;
busted.test_Q_component_2[2]
[10]:=busted.test.theta_AG*busted.test.omega2*t*0.1886019614740855;
busted.test_Q_component_2[2]
[14]:=busted.test.theta_AT*busted.test.omega2*t*0.2208116300950855;
busted.test_Q_component_2[2]
[18]:=busted.test.theta_AC*busted.test.omega2*t*0.1512809665850036;
busted.test_Q_component_2[2]
[34]:=busted.test.theta_AG*busted.test.omega2*t*0.2936069988308121;
busted.test_Q_component_2[3]
[0]:=busted.test.theta_AT*busted.test.omega2*t*0.4290813980170551;
busted.test_Q_component_2[3]
[1]:=busted.test.theta_CT*t*0.0447809360409677;
busted.test_Q_component_2[3]
[2]:=busted.test.theta_GT*busted.test.omega2*t*0.1063345886498611;
busted.test_Q_component_2[3]
[7]:=busted.test.theta_AC*busted.test.omega2*t*0.2496987119458206;
busted.test_Q_component_2[3]
[11]:=busted.test.theta_AG*busted.test.omega2*t*0.1886019614740855;
busted.test_Q_component_2[3]
[15]:=busted.test.theta_AT*busted.test.omega2*t*0.2208116300950855;
busted.test_Q_component_2[3]
[19]:=busted.test.theta_AC*busted.test.omega2*t*0.1512809665850036;
busted.test_Q_component_2[3]
[35]:=busted.test.theta_AG*busted.test.omega2*t*0.2936069988308121;
busted.test_Q_component_2[3]
[49]:=busted.test.theta_AT*busted.test.omega2*t*0.244832889475194;
busted.test_Q_component_2[4]
[0]:=busted.test.theta_AC*busted.test.omega2*t*0.3408876964850085;
busted.test_Q_component_2[4]
[5]:=busted.test.theta_AC*t*0.0447809360409677;
busted.test_Q_component_2[4]
[6]:=busted.test.theta_AG*t*0.1063345886498611;
busted.test_Q_component_2[4]
[7]:=busted.test.theta_AT*t*0.419803077292116;
busted.test_Q_component_2[4]
[8]:=busted.test.theta_CG*busted.test.omega2*t*0.1886019614740855;
busted.test_Q_component_2[4]
[12]:=busted.test.theta_CT*busted.test.omega2*t*0.2208116300950855;
busted.test_Q_component_2[4]
[20]:=busted.test.theta_AC*busted.test.omega2*t*0.1512809665850036;
busted.test_Q_component_2[4]
```

```
[36]:=busted.test.theta_AG*busted.test.omega2*t*0.2936069988308121;
busted.test_Q_component_2[4]
[50]:=busted.test.theta_AT*busted.test.omega2*t*0.244832889475194;
busted.test_Q_component_2[5]
[1]:=busted.test.theta_AC*busted.test.omega2*t*0.3408876964850085;
busted.test_Q_component_2[5]
[4]:=busted.test.theta_AC*t*0.4290813980170551;
busted.test_Q_component_2[5]
[6]:=busted.test.theta_CG*t*0.1063345886498611;
busted.test_Q_component_2[5]
[7]:=busted.test.theta_CT*t*0.419803077292116;
busted.test_Q_component_2[5]
[9]:=busted.test.theta_CG*busted.test.omega2*t*0.1886019614740855;
busted.test_Q_component_2[5]
[13]:=busted.test.theta_CT*busted.test.omega2*t*0.2208116300950855;
busted.test_Q_component_2[5]
[21]:=busted.test.theta_AC*busted.test.omega2*t*0.1512809665850036;
busted.test_Q_component_2[5]
[37]:=busted.test.theta_AG*busted.test.omega2*t*0.2936069988308121;
busted.test_Q_component_2[5]
[51]:=busted.test.theta_AT*busted.test.omega2*t*0.244832889475194;
busted.test_Q_component_2[6]
[2]:=busted.test.theta_AC*busted.test.omega2*t*0.3408876964850085;
busted.test_Q_component_2[6]
[4]:=busted.test.theta_AG*t*0.4290813980170551;
busted.test_Q_component_2[6]
[5]:=busted.test.theta_CG*t*0.0447809360409677;
busted.test_Q_component_2[6]
[7]:=busted.test.theta_GT*t*0.419803077292116;
busted.test_Q_component_2[6]
[10]:=busted.test.theta_CG*busted.test.omega2*t*0.1886019614740855;
busted.test_Q_component_2[6]
[14]:=busted.test.theta_CT*busted.test.omega2*t*0.2208116300950855;
busted.test_Q_component_2[6]
[22]:=busted.test.theta_AC*busted.test.omega2*t*0.1512809665850036;
busted.test_Q_component_2[6]
[38]:=busted.test.theta_AG*busted.test.omega2*t*0.2936069988308121;
busted.test_Q_component_2[6]
[52]:=busted.test.theta_AT*busted.test.omega2*t*0.244832889475194;
busted.test_Q_component_2[7]
[3]:=busted.test.theta_AC*busted.test.omega2*t*0.3408876964850085;
busted.test_Q_component_2[7]
[4]:=busted.test.theta_AT*t*0.4290813980170551;
busted.test_Q_component_2[7]
[5]:=busted.test.theta_CT*t*0.0447809360409677;
busted.test_Q_component_2[7]
[6]:=busted.test.theta_GT*t*0.1063345886498611;
busted.test_Q_component_2[7]
[11]:=busted.test.theta_CG*busted.test.omega2*t*0.1886019614740855;
busted.test_Q_component_2[7]
[15]:=busted.test.theta_CT*busted.test.omega2*t*0.2208116300950855;
busted.test_Q_component_2[7]
[23]:=busted.test.theta_AC*busted.test.omega2*t*0.1512809665850036;
busted.test_Q_component_2[7]
```

```
[39]:=busted.test.theta_AG*busted.test.omega2*t*0.2936069988308121;
busted.test_Q_component_2[7]
[53]:=busted.test.theta_AT*busted.test.omega2*t*0.244832889475194;
busted.test_Q_component_2[8]
[0]:=busted.test.theta_AG*busted.test.omega2*t*0.3408876964850085;
busted.test_Q_component_2[8]
[4]:=busted.test.theta_CG*busted.test.omega2*t*0.2496987119458206;
busted.test_Q_component_2[8]
[9]:=busted.test.theta_AC*busted.test.omega2*t*0.0447809360409677;
busted.test_Q_component_2[8]
[10]:=busted.test.theta_AG*t*0.1063345886498611;
busted.test_Q_component_2[8]
[11]:=busted.test.theta_AT*busted.test.omega2*t*0.419803077292116;
busted.test_Q_component_2[8]
[12]:=busted.test.theta_GT*busted.test.omega2*t*0.2208116300950855;
busted.test_Q_component_2[8]
[24]:=busted.test.theta_AC*t*0.1512809665850036;
busted.test_Q_component_2[8]
[40]:=busted.test.theta_AG*busted.test.omega2*t*0.2936069988308121;
busted.test_Q_component_2[9]
[1]:=busted.test.theta_AG*busted.test.omega2*t*0.3408876964850085;
busted.test_Q_component_2[9]
[5]:=busted.test.theta_CG*busted.test.omega2*t*0.2496987119458206;
busted.test_Q_component_2[9]
[8]:=busted.test.theta_AC*busted.test.omega2*t*0.4290813980170551;
busted.test_Q_component_2[9]
[10]:=busted.test.theta_CG*busted.test.omega2*t*0.1063345886498611;
busted.test_Q_component_2[9]
[11]:=busted.test.theta_CT*t*0.419803077292116;
busted.test_Q_component_2[9]
[13]:=busted.test.theta_GT*busted.test.omega2*t*0.2208116300950855;
busted.test_Q_component_2[9]
[25]:=busted.test.theta_AC*busted.test.omega2*t*0.1512809665850036;
busted.test_Q_component_2[9]
[41]:=busted.test.theta_AG*busted.test.omega2*t*0.2936069988308121;
busted.test_Q_component_2[9]
[54]:=busted.test.theta_AT*busted.test.omega2*t*0.244832889475194;
busted.test_Q_component_2[10]
[2]:=busted.test.theta_AG*busted.test.omega2*t*0.3408876964850085;
busted.test_Q_component_2[10]
[6]:=busted.test.theta_CG*busted.test.omega2*t*0.2496987119458206;
busted.test_Q_component_2[10]
[8]:=busted.test.theta_AG*t*0.4290813980170551;
busted.test_Q_component_2[10]
[9]:=busted.test.theta_CG*busted.test.omega2*t*0.0447809360409677;
busted.test_Q_component_2[10]
[11]:=busted.test.theta_GT*busted.test.omega2*t*0.419803077292116;
busted.test_Q_component_2[10]
[14]:=busted.test.theta_GT*busted.test.omega2*t*0.2208116300950855;
busted.test_Q_component_2[10]
[26]:=busted.test.theta_AC*t*0.1512809665850036;
busted.test_Q_component_2[10]
[42]:=busted.test.theta_AG*busted.test.omega2*t*0.2936069988308121;
busted.test_Q_component_2[10]
```

```
[55]:=busted.test.theta_AT*busted.test.omega2*t*0.244832889475194;
busted.test_Q_component_2[11]
[3]:=busted.test.theta_AG*busted.test.omega2*t*0.3408876964850085;
busted.test_Q_component_2[11]
[7]:=busted.test.theta_CG*busted.test.omega2*t*0.2496987119458206;
busted.test_Q_component_2[11]
[8]:=busted.test.theta_AT*busted.test.omega2*t*0.4290813980170551;
busted.test_Q_component_2[11]
[9]:=busted.test.theta_CT*t*0.0447809360409677;
busted.test_Q_component_2[11]
[10]:=busted.test.theta_GT*busted.test.omega2*t*0.1063345886498611;
busted.test_Q_component_2[11]
[15]:=busted.test.theta_GT*busted.test.omega2*t*0.2208116300950855;
busted.test_Q_component_2[11]
[27]:=busted.test.theta_AC*busted.test.omega2*t*0.1512809665850036;
busted.test_Q_component_2[11]
[43]:=busted.test.theta_AG*busted.test.omega2*t*0.2936069988308121;
busted.test_Q_component_2[11]
[56]:=busted.test.theta_AT*busted.test.omega2*t*0.244832889475194;
busted.test_Q_component_2[12]
[0]:=busted.test.theta_AT*busted.test.omega2*t*0.3408876964850085;
busted.test_Q_component_2[12]
[4]:=busted.test.theta_CT*busted.test.omega2*t*0.2496987119458206;
busted.test_Q_component_2[12]
[8]:=busted.test.theta_GT*busted.test.omega2*t*0.1886019614740855;
busted.test_Q_component_2[12]
[13]:=busted.test.theta_AC*t*0.0447809360409677;
busted.test_Q_component_2[12]
[14]:=busted.test.theta_AG*busted.test.omega2*t*0.1063345886498611;
busted.test_Q_component_2[12]
[15]:=busted.test.theta_AT*t*0.419803077292116;
busted.test_Q_component_2[12]
[28]:=busted.test.theta_AC*busted.test.omega2*t*0.1512809665850036;
busted.test_Q_component_2[12]
[44]:=busted.test.theta_AG*busted.test.omega2*t*0.2936069988308121;
busted.test_Q_component_2[12]
[57]:=busted.test.theta_AT*busted.test.omega2*t*0.244832889475194;
busted.test_Q_component_2[13]
[1]:=busted.test.theta_AT*busted.test.omega2*t*0.3408876964850085;
busted.test_Q_component_2[13]
[5]:=busted.test.theta_CT*busted.test.omega2*t*0.2496987119458206;
busted.test_Q_component_2[13]
[9]:=busted.test.theta_GT*busted.test.omega2*t*0.1886019614740855;
busted.test_Q_component_2[13]
[12]:=busted.test.theta_AC*t*0.4290813980170551;
busted.test_Q_component_2[13]
[14]:=busted.test.theta_CG*busted.test.omega2*t*0.1063345886498611;
busted.test_Q_component_2[13]
[15]:=busted.test.theta_CT*t*0.419803077292116;
busted.test_Q_component_2[13]
[29]:=busted.test.theta_AC*busted.test.omega2*t*0.1512809665850036;
busted.test_Q_component_2[13]
[45]:=busted.test.theta_AG*busted.test.omega2*t*0.2936069988308121;
busted.test_Q_component_2[13]
```

```
[58]:=busted.test.theta_AT*busted.test.omega2*t*0.244832889475194;
busted.test_Q_component_2[14]
[2]:=busted.test.theta_AT*busted.test.omega2*t*0.3408876964850085;
busted.test_Q_component_2[14]
[6]:=busted.test.theta_CT*busted.test.omega2*t*0.2496987119458206;
busted.test_Q_component_2[14]
[10]:=busted.test.theta_GT*busted.test.omega2*t*0.1886019614740855;
busted.test_Q_component_2[14]
[12]:=busted.test.theta_AG*busted.test.omega2*t*0.4290813980170551;
busted.test_Q_component_2[14]
[13]:=busted.test.theta_CG*busted.test.omega2*t*0.0447809360409677;
busted.test_Q_component_2[14]
[15]:=busted.test.theta_GT*busted.test.omega2*t*0.419803077292116;
busted.test_Q_component_2[14]
[30]:=busted.test.theta_AC*busted.test.omega2*t*0.1512809665850036;
busted.test_Q_component_2[14]
[46]:=busted.test.theta_AG*busted.test.omega2*t*0.2936069988308121;
busted.test_Q_component_2[14]
[59]:=busted.test.theta_AT*busted.test.omega2*t*0.244832889475194;
busted.test_Q_component_2[15]
[3]:=busted.test.theta_AT*busted.test.omega2*t*0.3408876964850085;
busted.test_Q_component_2[15]
[7]:=busted.test.theta_CT*busted.test.omega2*t*0.2496987119458206;
busted.test_Q_component_2[15]
[11]:=busted.test.theta_GT*busted.test.omega2*t*0.1886019614740855;
busted.test_Q_component_2[15]
[12]:=busted.test.theta_AT*t*0.4290813980170551;
busted.test_Q_component_2[15]
[13]:=busted.test.theta_CT*t*0.0447809360409677;
busted.test_Q_component_2[15]
[14]:=busted.test.theta_GT*busted.test.omega2*t*0.1063345886498611;
busted.test_Q_component_2[15]
[31]:=busted.test.theta_AC*busted.test.omega2*t*0.1512809665850036;
busted.test_Q_component_2[15]
[47]:=busted.test.theta_AG*busted.test.omega2*t*0.2936069988308121;
busted.test_Q_component_2[15]
[60]:=busted.test.theta_AT*busted.test.omega2*t*0.244832889475194;
busted.test_Q_component_2[16]
[0]:=busted.test.theta_AC*busted.test.omega2*t*0.3102791451089904;
busted.test_Q_component_2[16]
[17]:=busted.test.theta_AC*busted.test.omega2*t*0.0447809360409677;
busted.test_Q_component_2[16]
[18]:=busted.test.theta_AG*t*0.1063345886498611;
busted.test_Q_component_2[16]
[19]:=busted.test.theta_AT*busted.test.omega2*t*0.419803077292116;
busted.test_Q_component_2[16]
[20]:=busted.test.theta_AC*busted.test.omega2*t*0.2496987119458206;
busted.test_Q_component_2[16]
[24]:=busted.test.theta_AG*busted.test.omega2*t*0.1886019614740855;
busted.test_Q_component_2[16]
[28]:=busted.test.theta_AT*busted.test.omega2*t*0.2208116300950855;
busted.test_Q_component_2[16]
[32]:=busted.test.theta_CG*busted.test.omega2*t*0.2936069988308121;
busted.test_Q_component_2[17]
```

```
[1]:=busted.test.theta_AC*busted.test.omega2*t*0.3102791451089904;
busted.test_Q_component_2[17]
[16]:=busted.test.theta_AC*busted.test.omega2*t*0.4290813980170551;
busted.test_Q_component_2[17]
[18]:=busted.test.theta_CG*busted.test.omega2*t*0.1063345886498611;
busted.test_Q_component_2[17]
[19]:=busted.test.theta_CT*t*0.419803077292116;
busted.test_Q_component_2[17]
[21]:=busted.test.theta_AC*busted.test.omega2*t*0.2496987119458206;
busted.test_Q_component_2[17]
[25]:=busted.test.theta_AG*busted.test.omega2*t*0.1886019614740855;
busted.test_Q_component_2[17]
[29]:=busted.test.theta_AT*busted.test.omega2*t*0.2208116300950855;
busted.test_Q_component_2[17]
[33]:=busted.test.theta_CG*busted.test.omega2*t*0.2936069988308121;
busted.test_Q_component_2[17]
[48]:=busted.test.theta_CT*busted.test.omega2*t*0.244832889475194;
busted.test_Q_component_2[18]
[2]:=busted.test.theta_AC*busted.test.omega2*t*0.3102791451089904;
busted.test_Q_component_2[18]
[16]:=busted.test.theta_AG*t*0.4290813980170551;
busted.test_Q_component_2[18]
[17]:=busted.test.theta_CG*busted.test.omega2*t*0.0447809360409677;
busted.test_Q_component_2[18]
[19]:=busted.test.theta_GT*busted.test.omega2*t*0.419803077292116;
busted.test_Q_component_2[18]
[22]:=busted.test.theta_AC*busted.test.omega2*t*0.2496987119458206;
busted.test_Q_component_2[18]
[26]:=busted.test.theta_AG*busted.test.omega2*t*0.1886019614740855;
busted.test_Q_component_2[18]
[30]:=busted.test.theta_AT*busted.test.omega2*t*0.2208116300950855;
busted.test_Q_component_2[18]
[34]:=busted.test.theta_CG*busted.test.omega2*t*0.2936069988308121;
busted.test_Q_component_2[19]
[3]:=busted.test.theta_AC*busted.test.omega2*t*0.3102791451089904;
busted.test_Q_component_2[19]
[16]:=busted.test.theta_AT*busted.test.omega2*t*0.4290813980170551;
busted.test_Q_component_2[19]
[17]:=busted.test.theta_CT*t*0.0447809360409677;
busted.test_Q_component_2[19]
[18]:=busted.test.theta_GT*busted.test.omega2*t*0.1063345886498611;
busted.test_Q_component_2[19]
[23]:=busted.test.theta_AC*busted.test.omega2*t*0.2496987119458206;
busted.test_Q_component_2[19]
[27]:=busted.test.theta_AG*busted.test.omega2*t*0.1886019614740855;
busted.test_Q_component_2[19]
[31]:=busted.test.theta_AT*busted.test.omega2*t*0.2208116300950855;
busted.test_Q_component_2[19]
[35]:=busted.test.theta_CG*busted.test.omega2*t*0.2936069988308121;
busted.test_Q_component_2[19]
[49]:=busted.test.theta_CT*busted.test.omega2*t*0.244832889475194;
busted.test_Q_component_2[20]
[4]:=busted.test.theta_AC*busted.test.omega2*t*0.3102791451089904;
busted.test_Q_component_2[20]
```

```
[16]:=busted.test.theta_AC*busted.test.omega2*t*0.3408876964850085;
busted.test_Q_component_2[20]
[21]:=busted.test.theta_AC*t*0.0447809360409677;
busted.test_Q_component_2[20]
[22]:=busted.test.theta_AG*t*0.1063345886498611;
busted.test_Q_component_2[20]
[23]:=busted.test.theta_AT*t*0.419803077292116;
busted.test_Q_component_2[20]
[24]:=busted.test.theta_CG*busted.test.omega2*t*0.1886019614740855;
busted.test_Q_component_2[20]
[28]:=busted.test.theta_CT*busted.test.omega2*t*0.2208116300950855;
busted.test_Q_component_2[20]
[36]:=busted.test.theta_CG*busted.test.omega2*t*0.2936069988308121;
busted.test_Q_component_2[20]
[50]:=busted.test.theta_CT*busted.test.omega2*t*0.244832889475194;
busted.test_Q_component_2[21]
[5]:=busted.test.theta_AC*busted.test.omega2*t*0.3102791451089904;
busted.test_Q_component_2[21]
[17]:=busted.test.theta_AC*busted.test.omega2*t*0.3408876964850085;
busted.test_Q_component_2[21]
[20]:=busted.test.theta_AC*t*0.4290813980170551;
busted.test_Q_component_2[21]
[22]:=busted.test.theta_CG*t*0.1063345886498611;
busted.test_Q_component_2[21]
[23]:=busted.test.theta_CT*t*0.419803077292116;
busted.test_Q_component_2[21]
[25]:=busted.test.theta_CG*busted.test.omega2*t*0.1886019614740855;
busted.test_Q_component_2[21]
[29]:=busted.test.theta_CT*busted.test.omega2*t*0.2208116300950855;
busted.test_Q_component_2[21]
[37]:=busted.test.theta_CG*busted.test.omega2*t*0.2936069988308121;
busted.test_Q_component_2[21]
[51]:=busted.test.theta_CT*busted.test.omega2*t*0.244832889475194;
busted.test_Q_component_2[22]
[6]:=busted.test.theta_AC*busted.test.omega2*t*0.3102791451089904;
busted.test_Q_component_2[22]
[18]:=busted.test.theta_AC*busted.test.omega2*t*0.3408876964850085;
busted.test_Q_component_2[22]
[20]:=busted.test.theta_AG*t*0.4290813980170551;
busted.test_Q_component_2[22]
[21]:=busted.test.theta_CG*t*0.0447809360409677;
busted.test_Q_component_2[22]
[23]:=busted.test.theta_GT*t*0.419803077292116;
busted.test_Q_component_2[22]
[26]:=busted.test.theta_CG*busted.test.omega2*t*0.1886019614740855;
busted.test_Q_component_2[22]
[30]:=busted.test.theta_CT*busted.test.omega2*t*0.2208116300950855;
busted.test_Q_component_2[22]
[38]:=busted.test.theta_CG*busted.test.omega2*t*0.2936069988308121;
busted.test_Q_component_2[22]
[52]:=busted.test.theta_CT*busted.test.omega2*t*0.244832889475194;
busted.test_Q_component_2[23]
[7]:=busted.test.theta_AC*busted.test.omega2*t*0.3102791451089904;
busted.test_Q_component_2[23]
```

```
[19]:=busted.test.theta_AC*busted.test.omega2*t*0.3408876964850085;
busted.test_Q_component_2[23]
[20]:=busted.test.theta_AT*t*0.4290813980170551;
busted.test_Q_component_2[23]
[21]:=busted.test.theta_CT*t*0.0447809360409677;
busted.test_Q_component_2[23]
[22]:=busted.test.theta_GT*t*0.1063345886498611;
busted.test_Q_component_2[23]
[27]:=busted.test.theta_CG*busted.test.omega2*t*0.1886019614740855;
busted.test_Q_component_2[23]
[31]:=busted.test.theta_CT*busted.test.omega2*t*0.2208116300950855;
busted.test_Q_component_2[23]
[39]:=busted.test.theta_CG*busted.test.omega2*t*0.2936069988308121;
busted.test_Q_component_2[23]
[53]:=busted.test.theta_CT*busted.test.omega2*t*0.244832889475194;
busted.test_Q_component_2[24]
[8]:=busted.test.theta_AC*t*0.3102791451089904;
busted.test_Q_component_2[24]
[16]:=busted.test.theta_AG*busted.test.omega2*t*0.3408876964850085;
busted.test_Q_component_2[24]
[20]:=busted.test.theta_CG*busted.test.omega2*t*0.2496987119458206;
busted.test_Q_component_2[24]
[25]:=busted.test.theta_AC*t*0.0447809360409677;
busted.test_Q_component_2[24]
[26]:=busted.test.theta_AG*t*0.1063345886498611;
busted.test_Q_component_2[24]
[27]:=busted.test.theta_AT*t*0.419803077292116;
busted.test_Q_component_2[24]
[28]:=busted.test.theta_GT*busted.test.omega2*t*0.2208116300950855;
busted.test_Q_component_2[24]
[40]:=busted.test.theta_CG*busted.test.omega2*t*0.2936069988308121;
busted.test_Q_component_2[25]
[9]:=busted.test.theta_AC*busted.test.omega2*t*0.3102791451089904;
busted.test_Q_component_2[25]
[17]:=busted.test.theta_AG*busted.test.omega2*t*0.3408876964850085;
busted.test_Q_component_2[25]
[21]:=busted.test.theta_CG*busted.test.omega2*t*0.2496987119458206;
busted.test_Q_component_2[25]
[24]:=busted.test.theta_AC*t*0.4290813980170551;
busted.test_Q_component_2[25]
[26]:=busted.test.theta_CG*t*0.1063345886498611;
busted.test_Q_component_2[25]
[27]:=busted.test.theta_CT*t*0.419803077292116;
busted.test_Q_component_2[25]
[29]:=busted.test.theta_GT*busted.test.omega2*t*0.2208116300950855;
busted.test_Q_component_2[25]
[41]:=busted.test.theta_CG*busted.test.omega2*t*0.2936069988308121;
busted.test_Q_component_2[25]
[54]:=busted.test.theta_CT*busted.test.omega2*t*0.244832889475194;
busted.test_Q_component_2[26]
[10]:=busted.test.theta_AC*t*0.3102791451089904;
busted.test_Q_component_2[26]
[18]:=busted.test.theta_AG*busted.test.omega2*t*0.3408876964850085;
busted.test_Q_component_2[26]
```

```
[22]:=busted.test.theta_CG*busted.test.omega2*t*0.2496987119458206;
busted.test_Q_component_2[26]
[24]:=busted.test.theta_AG*t*0.4290813980170551;
busted.test_Q_component_2[26]
[25]:=busted.test.theta_CG*t*0.0447809360409677;
busted.test_Q_component_2[26]
[27]:=busted.test.theta_GT*t*0.419803077292116;
busted.test_Q_component_2[26]
[30]:=busted.test.theta_GT*busted.test.omega2*t*0.2208116300950855;
busted.test_Q_component_2[26]
[42]:=busted.test.theta_CG*busted.test.omega2*t*0.2936069988308121;
busted.test_Q_component_2[26]
[55]:=busted.test.theta_CT*busted.test.omega2*t*0.244832889475194;
busted.test_Q_component_2[27]
[11]:=busted.test.theta_AC*busted.test.omega2*t*0.3102791451089904;
busted.test_Q_component_2[27]
[19]:=busted.test.theta_AG*busted.test.omega2*t*0.3408876964850085;
busted.test_Q_component_2[27]
[23]:=busted.test.theta_CG*busted.test.omega2*t*0.2496987119458206;
busted.test_Q_component_2[27]
[24]:=busted.test.theta_AT*t*0.4290813980170551;
busted.test_Q_component_2[27]
[25]:=busted.test.theta_CT*t*0.0447809360409677;
busted.test_Q_component_2[27]
[26]:=busted.test.theta_GT*t*0.1063345886498611;
busted.test_Q_component_2[27]
[31]:=busted.test.theta_GT*busted.test.omega2*t*0.2208116300950855;
busted.test_Q_component_2[27]
[43]:=busted.test.theta_CG*busted.test.omega2*t*0.2936069988308121;
busted.test_Q_component_2[27]
[56]:=busted.test.theta_CT*busted.test.omega2*t*0.244832889475194;
busted.test_Q_component_2[28]
[12]:=busted.test.theta_AC*busted.test.omega2*t*0.3102791451089904;
busted.test_Q_component_2[28]
[16]:=busted.test.theta_AT*busted.test.omega2*t*0.3408876964850085;
busted.test_Q_component_2[28]
[20]:=busted.test.theta_CT*busted.test.omega2*t*0.2496987119458206;
busted.test_Q_component_2[28]
[24]:=busted.test.theta_GT*busted.test.omega2*t*0.1886019614740855;
busted.test_Q_component_2[28]
[29]:=busted.test.theta_AC*t*0.0447809360409677;
busted.test_Q_component_2[28]
[30]:=busted.test.theta_AG*t*0.1063345886498611;
busted.test_Q_component_2[28]
[31]:=busted.test.theta_AT*t*0.419803077292116;
busted.test_Q_component_2[28]
[44]:=busted.test.theta_CG*busted.test.omega2*t*0.2936069988308121;
busted.test_Q_component_2[28]
[57]:=busted.test.theta_CT*t*0.244832889475194;
busted.test_Q_component_2[29]
[13]:=busted.test.theta_AC*busted.test.omega2*t*0.3102791451089904;
busted.test_Q_component_2[29]
[17]:=busted.test.theta_AT*busted.test.omega2*t*0.3408876964850085;
busted.test_Q_component_2[29]
```

```
[21]:=busted.test.theta_CT*busted.test.omega2*t*0.2496987119458206;
busted.test_Q_component_2[29]
[25]:=busted.test.theta_GT*busted.test.omega2*t*0.1886019614740855;
busted.test_Q_component_2[29]
[28]:=busted.test.theta_AC*t*0.4290813980170551;
busted.test_Q_component_2[29]
[30]:=busted.test.theta_CG*t*0.1063345886498611;
busted.test_Q_component_2[29]
[31]:=busted.test.theta_CT*t*0.419803077292116;
busted.test_Q_component_2[29]
[45]:=busted.test.theta_CG*busted.test.omega2*t*0.2936069988308121;
busted.test_Q_component_2[29]
[58]:=busted.test.theta_CT*busted.test.omega2*t*0.244832889475194;
busted.test_Q_component_2[30]
[14]:=busted.test.theta_AC*busted.test.omega2*t*0.3102791451089904;
busted.test_Q_component_2[30]
[18]:=busted.test.theta_AT*busted.test.omega2*t*0.3408876964850085;
busted.test_Q_component_2[30]
[22]:=busted.test.theta_CT*busted.test.omega2*t*0.2496987119458206;
busted.test_Q_component_2[30]
[26]:=busted.test.theta_GT*busted.test.omega2*t*0.1886019614740855;
busted.test_Q_component_2[30]
[28]:=busted.test.theta_AG*t*0.4290813980170551;
busted.test_Q_component_2[30]
[29]:=busted.test.theta_CG*t*0.0447809360409677;
busted.test_Q_component_2[30]
[31]:=busted.test.theta_GT*t*0.419803077292116;
busted.test_Q_component_2[30]
[46]:=busted.test.theta_CG*busted.test.omega2*t*0.2936069988308121;
busted.test_Q_component_2[30]
[59]:=busted.test.theta_CT*t*0.244832889475194;
busted.test_Q_component_2[31]
[15]:=busted.test.theta_AC*busted.test.omega2*t*0.3102791451089904;
busted.test_Q_component_2[31]
[19]:=busted.test.theta_AT*busted.test.omega2*t*0.3408876964850085;
busted.test_Q_component_2[31]
[23]:=busted.test.theta_CT*busted.test.omega2*t*0.2496987119458206;
busted.test_Q_component_2[31]
[27]:=busted.test.theta_GT*busted.test.omega2*t*0.1886019614740855;
busted.test_Q_component_2[31]
[28]:=busted.test.theta_AT*t*0.4290813980170551;
busted.test_Q_component_2[31]
[29]:=busted.test.theta_CT*t*0.0447809360409677;
busted.test_Q_component_2[31]
[30]:=busted.test.theta_GT*t*0.1063345886498611;
busted.test_Q_component_2[31]
[47]:=busted.test.theta_CG*busted.test.omega2*t*0.2936069988308121;
busted.test_Q_component_2[31]
[60]:=busted.test.theta_CT*busted.test.omega2*t*0.244832889475194;
busted.test_Q_component_2[32]
[0]:=busted.test.theta_AG*busted.test.omega2*t*0.3102791451089904;
busted.test_Q_component_2[32]
[16]:=busted.test.theta_CG*busted.test.omega2*t*0.1512809665850036;
busted.test_Q_component_2[32]
```

```
[33]:=busted.test.theta_AC*busted.test.omega2*t*0.0447809360409677;
busted.test_Q_component_2[32]
[34]:=busted.test.theta_AG*t*0.1063345886498611;
busted.test_Q_component_2[32]
[35]:=busted.test.theta_AT*busted.test.omega2*t*0.419803077292116;
busted.test_Q_component_2[32]
[36]:=busted.test.theta_AC*busted.test.omega2*t*0.2496987119458206;
busted.test_Q_component_2[32]
[40]:=busted.test.theta_AG*busted.test.omega2*t*0.1886019614740855;
busted.test_Q_component_2[32]
[44]:=busted.test.theta_AT*busted.test.omega2*t*0.2208116300950855;
busted.test_Q_component_2[33]
[1]:=busted.test.theta_AG*busted.test.omega2*t*0.3102791451089904;
busted.test_Q_component_2[33]
[17]:=busted.test.theta_CG*busted.test.omega2*t*0.1512809665850036;
busted.test_Q_component_2[33]
[32]:=busted.test.theta_AC*busted.test.omega2*t*0.4290813980170551;
busted.test_Q_component_2[33]
[34]:=busted.test.theta_CG*busted.test.omega2*t*0.1063345886498611;
busted.test_Q_component_2[33]
[35]:=busted.test.theta_CT*t*0.419803077292116;
busted.test_Q_component_2[33]
[37]:=busted.test.theta_AC*busted.test.omega2*t*0.2496987119458206;
busted.test_Q_component_2[33]
[41]:=busted.test.theta_AG*busted.test.omega2*t*0.1886019614740855;
busted.test_Q_component_2[33]
[45]:=busted.test.theta_AT*busted.test.omega2*t*0.2208116300950855;
busted.test_Q_component_2[33]
[48]:=busted.test.theta_GT*busted.test.omega2*t*0.244832889475194;
busted.test_Q_component_2[34]
[2]:=busted.test.theta_AG*busted.test.omega2*t*0.3102791451089904;
busted.test_Q_component_2[34]
[18]:=busted.test.theta_CG*busted.test.omega2*t*0.1512809665850036;
busted.test_Q_component_2[34]
[32]:=busted.test.theta_AG*t*0.4290813980170551;
busted.test_Q_component_2[34]
[33]:=busted.test.theta_CG*busted.test.omega2*t*0.0447809360409677;
busted.test_Q_component_2[34]
[35]:=busted.test.theta_GT*busted.test.omega2*t*0.419803077292116;
busted.test_Q_component_2[34]
[38]:=busted.test.theta_AC*busted.test.omega2*t*0.2496987119458206;
busted.test_Q_component_2[34]
[42]:=busted.test.theta_AG*busted.test.omega2*t*0.1886019614740855;
busted.test_Q_component_2[34]
[46]:=busted.test.theta_AT*busted.test.omega2*t*0.2208116300950855;
busted.test_Q_component_2[35]
[3]:=busted.test.theta_AG*busted.test.omega2*t*0.3102791451089904;
busted.test_Q_component_2[35]
[19]:=busted.test.theta_CG*busted.test.omega2*t*0.1512809665850036;
busted.test_Q_component_2[35]
[32]:=busted.test.theta_AT*busted.test.omega2*t*0.4290813980170551;
busted.test_Q_component_2[35]
[33]:=busted.test.theta_CT*t*0.0447809360409677;
busted.test_Q_component_2[35]
```

```
[34]:=busted.test.theta_GT*busted.test.omega2*t*0.1063345886498611;
busted.test_Q_component_2[35]
[39]:=busted.test.theta_AC*busted.test.omega2*t*0.2496987119458206;
busted.test_Q_component_2[35]
[43]:=busted.test.theta_AG*busted.test.omega2*t*0.1886019614740855;
busted.test_Q_component_2[35]
[47]:=busted.test.theta_AT*busted.test.omega2*t*0.2208116300950855;
busted.test_Q_component_2[35]
[49]:=busted.test.theta_GT*busted.test.omega2*t*0.244832889475194;
busted.test_Q_component_2[36]
[4]:=busted.test.theta_AG*busted.test.omega2*t*0.3102791451089904;
busted.test_Q_component_2[36]
[20]:=busted.test.theta_CG*busted.test.omega2*t*0.1512809665850036;
busted.test_Q_component_2[36]
[32]:=busted.test.theta_AC*busted.test.omega2*t*0.3408876964850085;
busted.test_Q_component_2[36]
[37]:=busted.test.theta_AC*t*0.0447809360409677;
busted.test_Q_component_2[36]
[38]:=busted.test.theta_AG*t*0.1063345886498611;
busted.test_Q_component_2[36]
[39]:=busted.test.theta_AT*t*0.419803077292116;
busted.test_Q_component_2[36]
[40]:=busted.test.theta_CG*busted.test.omega2*t*0.1886019614740855;
busted.test_Q_component_2[36]
[44]:=busted.test.theta_CT*busted.test.omega2*t*0.2208116300950855;
busted.test_Q_component_2[36]
[50]:=busted.test.theta_GT*busted.test.omega2*t*0.244832889475194;
busted.test_Q_component_2[37]
[5]:=busted.test.theta_AG*busted.test.omega2*t*0.3102791451089904;
busted.test_Q_component_2[37]
[21]:=busted.test.theta_CG*busted.test.omega2*t*0.1512809665850036;
busted.test_Q_component_2[37]
[33]:=busted.test.theta_AC*busted.test.omega2*t*0.3408876964850085;
busted.test_Q_component_2[37]
[36]:=busted.test.theta_AC*t*0.4290813980170551;
busted.test_Q_component_2[37]
[38]:=busted.test.theta_CG*t*0.1063345886498611;
busted.test_Q_component_2[37]
[39]:=busted.test.theta_CT*t*0.419803077292116;
busted.test_Q_component_2[37]
[41]:=busted.test.theta_CG*busted.test.omega2*t*0.1886019614740855;
busted.test_Q_component_2[37]
[45]:=busted.test.theta_CT*busted.test.omega2*t*0.2208116300950855;
busted.test_Q_component_2[37]
[51]:=busted.test.theta_GT*busted.test.omega2*t*0.244832889475194;
busted.test_Q_component_2[38]
[6]:=busted.test.theta_AG*busted.test.omega2*t*0.3102791451089904;
busted.test_Q_component_2[38]
[22]:=busted.test.theta_CG*busted.test.omega2*t*0.1512809665850036;
busted.test_Q_component_2[38]
[34]:=busted.test.theta_AC*busted.test.omega2*t*0.3408876964850085;
busted.test_Q_component_2[38]
[36]:=busted.test.theta_AG*t*0.4290813980170551;
busted.test_Q_component_2[38]
```

```
[37]:=busted.test.theta_CG*t*0.0447809360409677;
busted.test_Q_component_2[38]
[39]:=busted.test.theta_GT*t*0.419803077292116;
busted.test_Q_component_2[38]
[42]:=busted.test.theta_CG*busted.test.omega2*t*0.1886019614740855;
busted.test_Q_component_2[38]
[46]:=busted.test.theta_CT*busted.test.omega2*t*0.2208116300950855;
busted.test_Q_component_2[38]
[52]:=busted.test.theta_GT*busted.test.omega2*t*0.244832889475194;
busted.test_Q_component_2[39]
[7]:=busted.test.theta_AG*busted.test.omega2*t*0.3102791451089904;
busted.test_Q_component_2[39]
[23]:=busted.test.theta_CG*busted.test.omega2*t*0.1512809665850036;
busted.test_Q_component_2[39]
[35]:=busted.test.theta_AC*busted.test.omega2*t*0.3408876964850085;
busted.test_Q_component_2[39]
[36]:=busted.test.theta_AT*t*0.4290813980170551;
busted.test_Q_component_2[39]
[37]:=busted.test.theta_CT*t*0.0447809360409677;
busted.test_Q_component_2[39]
[38]:=busted.test.theta_GT*t*0.1063345886498611;
busted.test_Q_component_2[39]
[43]:=busted.test.theta_CG*busted.test.omega2*t*0.1886019614740855;
busted.test_Q_component_2[39]
[47]:=busted.test.theta_CT*busted.test.omega2*t*0.2208116300950855;
busted.test_Q_component_2[39]
[53]:=busted.test.theta_GT*busted.test.omega2*t*0.244832889475194;
busted.test_Q_component_2[40]
[8]:=busted.test.theta_AG*busted.test.omega2*t*0.3102791451089904;
busted.test_Q_component_2[40]
[24]:=busted.test.theta_CG*busted.test.omega2*t*0.1512809665850036;
busted.test_Q_component_2[40]
[32]:=busted.test.theta_AG*busted.test.omega2*t*0.3408876964850085;
busted.test_Q_component_2[40]
[36]:=busted.test.theta_CG*busted.test.omega2*t*0.2496987119458206;
busted.test_Q_component_2[40]
[41]:=busted.test.theta_AC*t*0.0447809360409677;
busted.test_Q_component_2[40]
[42]:=busted.test.theta_AG*t*0.1063345886498611;
busted.test_Q_component_2[40]
[43]:=busted.test.theta_AT*t*0.419803077292116;
busted.test_Q_component_2[40]
[44]:=busted.test.theta_GT*busted.test.omega2*t*0.2208116300950855;
busted.test_Q_component_2[41]
[9]:=busted.test.theta_AG*busted.test.omega2*t*0.3102791451089904;
busted.test_Q_component_2[41]
[25]:=busted.test.theta_CG*busted.test.omega2*t*0.1512809665850036;
busted.test_Q_component_2[41]
[33]:=busted.test.theta_AG*busted.test.omega2*t*0.3408876964850085;
busted.test_Q_component_2[41]
[37]:=busted.test.theta_CG*busted.test.omega2*t*0.2496987119458206;
busted.test_Q_component_2[41]
[40]:=busted.test.theta_AC*t*0.4290813980170551;
busted.test_Q_component_2[41]
```

```
[42]:=busted.test.theta_CG*t*0.1063345886498611;
busted.test_Q_component_2[41]
[43]:=busted.test.theta_CT*t*0.419803077292116;
busted.test_Q_component_2[41]
[45]:=busted.test.theta_GT*busted.test.omega2*t*0.2208116300950855;
busted.test_Q_component_2[41]
[54]:=busted.test.theta_GT*busted.test.omega2*t*0.244832889475194;
busted.test_Q_component_2[42]
[10]:=busted.test.theta_AG*busted.test.omega2*t*0.3102791451089904;
busted.test_Q_component_2[42]
[26]:=busted.test.theta_CG*busted.test.omega2*t*0.1512809665850036;
busted.test_Q_component_2[42]
[34]:=busted.test.theta_AG*busted.test.omega2*t*0.3408876964850085;
busted.test_Q_component_2[42]
[38]:=busted.test.theta_CG*busted.test.omega2*t*0.2496987119458206;
busted.test_Q_component_2[42]
[40]:=busted.test.theta_AG*t*0.4290813980170551;
busted.test_Q_component_2[42]
[41]:=busted.test.theta_CG*t*0.0447809360409677;
busted.test_Q_component_2[42]
[43]:=busted.test.theta_GT*t*0.419803077292116;
busted.test_Q_component_2[42]
[46]:=busted.test.theta_GT*busted.test.omega2*t*0.2208116300950855;
busted.test_Q_component_2[42]
[55]:=busted.test.theta_GT*busted.test.omega2*t*0.244832889475194;
busted.test_Q_component_2[43]
[11]:=busted.test.theta_AG*busted.test.omega2*t*0.3102791451089904;
busted.test_Q_component_2[43]
[27]:=busted.test.theta_CG*busted.test.omega2*t*0.1512809665850036;
busted.test_Q_component_2[43]
[35]:=busted.test.theta_AG*busted.test.omega2*t*0.3408876964850085;
busted.test_Q_component_2[43]
[39]:=busted.test.theta_CG*busted.test.omega2*t*0.2496987119458206;
busted.test_Q_component_2[43]
[40]:=busted.test.theta_AT*t*0.4290813980170551;
busted.test_Q_component_2[43]
[41]:=busted.test.theta_CT*t*0.0447809360409677;
busted.test_Q_component_2[43]
[42]:=busted.test.theta_GT*t*0.1063345886498611;
busted.test_Q_component_2[43]
[47]:=busted.test.theta_GT*busted.test.omega2*t*0.2208116300950855;
busted.test_Q_component_2[43]
[56]:=busted.test.theta_GT*busted.test.omega2*t*0.244832889475194;
busted.test_Q_component_2[44]
[12]:=busted.test.theta_AG*busted.test.omega2*t*0.3102791451089904;
busted.test_Q_component_2[44]
[28]:=busted.test.theta_CG*busted.test.omega2*t*0.1512809665850036;
busted.test_Q_component_2[44]
[32]:=busted.test.theta_AT*busted.test.omega2*t*0.3408876964850085;
busted.test_Q_component_2[44]
[36]:=busted.test.theta_CT*busted.test.omega2*t*0.2496987119458206;
busted.test_Q_component_2[44]
[40]:=busted.test.theta_GT*busted.test.omega2*t*0.1886019614740855;
busted.test_Q_component_2[44]
```

```
[45]:=busted.test.theta_AC*t*0.0447809360409677;
busted.test_Q_component_2[44]
[46]:=busted.test.theta_AG*t*0.1063345886498611;
busted.test_Q_component_2[44]
[47]:=busted.test.theta_AT*t*0.419803077292116;
busted.test_Q_component_2[44]
[57]:=busted.test.theta_GT*busted.test.omega2*t*0.244832889475194;
busted.test_Q_component_2[45]
[13]:=busted.test.theta_AG*busted.test.omega2*t*0.3102791451089904;
busted.test_Q_component_2[45]
[29]:=busted.test.theta_CG*busted.test.omega2*t*0.1512809665850036;
busted.test_Q_component_2[45]
[33]:=busted.test.theta_AT*busted.test.omega2*t*0.3408876964850085;
busted.test_Q_component_2[45]
[37]:=busted.test.theta_CT*busted.test.omega2*t*0.2496987119458206;
busted.test_Q_component_2[45]
[41]:=busted.test.theta_GT*busted.test.omega2*t*0.1886019614740855;
busted.test_Q_component_2[45]
[44]:=busted.test.theta_AC*t*0.4290813980170551;
busted.test_Q_component_2[45]
[46]:=busted.test.theta_CG*t*0.1063345886498611;
busted.test_Q_component_2[45]
[47]:=busted.test.theta_CT*t*0.419803077292116;
busted.test_Q_component_2[45]
[58]:=busted.test.theta_GT*busted.test.omega2*t*0.244832889475194;
busted.test_Q_component_2[46]
[14]:=busted.test.theta_AG*busted.test.omega2*t*0.3102791451089904;
busted.test_Q_component_2[46]
[30]:=busted.test.theta_CG*busted.test.omega2*t*0.1512809665850036;
busted.test_Q_component_2[46]
[34]:=busted.test.theta_AT*busted.test.omega2*t*0.3408876964850085;
busted.test_Q_component_2[46]
[38]:=busted.test.theta_CT*busted.test.omega2*t*0.2496987119458206;
busted.test_Q_component_2[46]
[42]:=busted.test.theta_GT*busted.test.omega2*t*0.1886019614740855;
busted.test_Q_component_2[46]
[44]:=busted.test.theta_AG*t*0.4290813980170551;
busted.test_Q_component_2[46]
[45]:=busted.test.theta_CG*t*0.0447809360409677;
busted.test_Q_component_2[46]
[47]:=busted.test.theta_GT*t*0.419803077292116;
busted.test_Q_component_2[46]
[59]:=busted.test.theta_GT*busted.test.omega2*t*0.244832889475194;
busted.test_Q_component_2[47]
[15]:=busted.test.theta_AG*busted.test.omega2*t*0.3102791451089904;
busted.test_Q_component_2[47]
[31]:=busted.test.theta_CG*busted.test.omega2*t*0.1512809665850036;
busted.test_Q_component_2[47]
[35]:=busted.test.theta_AT*busted.test.omega2*t*0.3408876964850085;
busted.test_Q_component_2[47]
[39]:=busted.test.theta_CT*busted.test.omega2*t*0.2496987119458206;
busted.test_Q_component_2[47]
[43]:=busted.test.theta_GT*busted.test.omega2*t*0.1886019614740855;
busted.test_Q_component_2[47]
```

```
[44]:=busted.test.theta_AT*t*0.4290813980170551;
busted.test_Q_component_2[47]
[45]:=busted.test.theta_CT*t*0.0447809360409677;
busted.test_Q_component_2[47]
[46]:=busted.test.theta_GT*t*0.1063345886498611;
busted.test_Q_component_2[47]
[60]:=busted.test.theta_GT*busted.test.omega2*t*0.244832889475194;
busted.test_Q_component_2[48]
[1]:=busted.test.theta_AT*busted.test.omega2*t*0.3102791451089904;
busted.test_Q_component_2[48]
[17]:=busted.test.theta_CT*busted.test.omega2*t*0.1512809665850036;
busted.test_Q_component_2[48]
[33]:=busted.test.theta_GT*busted.test.omega2*t*0.2936069988308121;
busted.test_Q_component_2[48]
[49]:=busted.test.theta_CT*t*0.419803077292116;
busted.test_Q_component_2[48]
[51]:=busted.test.theta_AC*busted.test.omega2*t*0.2496987119458206;
busted.test_Q_component_2[48]
[54]:=busted.test.theta_AG*busted.test.omega2*t*0.1886019614740855;
busted.test_Q_component_2[48]
[58]:=busted.test.theta_AT*busted.test.omega2*t*0.2208116300950855;
busted.test_Q_component_2[49]
[3]:=busted.test.theta_AT*busted.test.omega2*t*0.3102791451089904;
busted.test_Q_component_2[49]
[19]:=busted.test.theta_CT*busted.test.omega2*t*0.1512809665850036;
busted.test_Q_component_2[49]
[35]:=busted.test.theta_GT*busted.test.omega2*t*0.2936069988308121;
busted.test_Q_component_2[49]
[48]:=busted.test.theta_CT*t*0.0447809360409677;
busted.test_Q_component_2[49]
[53]:=busted.test.theta_AC*busted.test.omega2*t*0.2496987119458206;
busted.test_Q_component_2[49]
[56]:=busted.test.theta_AG*busted.test.omega2*t*0.1886019614740855;
busted.test_Q_component_2[49]
[60]:=busted.test.theta_AT*busted.test.omega2*t*0.2208116300950855;
busted.test_Q_component_2[50]
[4]:=busted.test.theta_AT*busted.test.omega2*t*0.3102791451089904;
busted.test_Q_component_2[50]
[20]:=busted.test.theta_CT*busted.test.omega2*t*0.1512809665850036;
busted.test_Q_component_2[50]
[36]:=busted.test.theta_GT*busted.test.omega2*t*0.2936069988308121;
busted.test_Q_component_2[50]
[51]:=busted.test.theta_AC*t*0.0447809360409677;
busted.test_Q_component_2[50]
[52]:=busted.test.theta_AG*t*0.1063345886498611;
busted.test_Q_component_2[50]
[53]:=busted.test.theta_AT*t*0.419803077292116;
busted.test_Q_component_2[50]
[57]:=busted.test.theta_CT*busted.test.omega2*t*0.2208116300950855;
busted.test_Q_component_2[51]
[5]:=busted.test.theta_AT*busted.test.omega2*t*0.3102791451089904;
busted.test_Q_component_2[51]
[21]:=busted.test.theta_CT*busted.test.omega2*t*0.1512809665850036;
busted.test_Q_component_2[51]
```

```
[37]:=busted.test.theta_GT*busted.test.omega2*t*0.2936069988308121;
busted.test_Q_component_2[51]
[48]:=busted.test.theta_AC*busted.test.omega2*t*0.3408876964850085;
busted.test_Q_component_2[51]
[50]:=busted.test.theta_AC*t*0.4290813980170551;
busted.test_Q_component_2[51]
[52]:=busted.test.theta_CG*t*0.1063345886498611;
busted.test_Q_component_2[51]
[53]:=busted.test.theta_CT*t*0.419803077292116;
busted.test_Q_component_2[51]
[54]:=busted.test.theta_CG*busted.test.omega2*t*0.1886019614740855;
busted.test_Q_component_2[51]
[58]:=busted.test.theta_CT*busted.test.omega2*t*0.2208116300950855;
busted.test_Q_component_2[52]
[6]:=busted.test.theta_AT*busted.test.omega2*t*0.3102791451089904;
busted.test_Q_component_2[52]
[22]:=busted.test.theta_CT*busted.test.omega2*t*0.1512809665850036;
busted.test_Q_component_2[52]
[38]:=busted.test.theta_GT*busted.test.omega2*t*0.2936069988308121;
busted.test_Q_component_2[52]
[50]:=busted.test.theta_AG*t*0.4290813980170551;
busted.test_Q_component_2[52]
[51]:=busted.test.theta_CG*t*0.0447809360409677;
busted.test_Q_component_2[52]
[53]:=busted.test.theta_GT*t*0.419803077292116;
busted.test_Q_component_2[52]
[55]:=busted.test.theta_CG*busted.test.omega2*t*0.1886019614740855;
busted.test_Q_component_2[52]
[59]:=busted.test.theta_CT*busted.test.omega2*t*0.2208116300950855;
busted.test_Q_component_2[53]
[7]:=busted.test.theta_AT*busted.test.omega2*t*0.3102791451089904;
busted.test_Q_component_2[53]
[23]:=busted.test.theta_CT*busted.test.omega2*t*0.1512809665850036;
busted.test_Q_component_2[53]
[39]:=busted.test.theta_GT*busted.test.omega2*t*0.2936069988308121;
busted.test_Q_component_2[53]
[49]:=busted.test.theta_AC*busted.test.omega2*t*0.3408876964850085;
busted.test_Q_component_2[53]
[50]:=busted.test.theta_AT*t*0.4290813980170551;
busted.test_Q_component_2[53]
[51]:=busted.test.theta_CT*t*0.0447809360409677;
busted.test_Q_component_2[53]
[52]:=busted.test.theta_GT*t*0.1063345886498611;
busted.test_Q_component_2[53]
[56]:=busted.test.theta_CG*busted.test.omega2*t*0.1886019614740855;
busted.test_Q_component_2[53]
[60]:=busted.test.theta_CT*busted.test.omega2*t*0.2208116300950855;
busted.test_Q_component_2[54]
[9]:=busted.test.theta_AT*busted.test.omega2*t*0.3102791451089904;
busted.test_Q_component_2[54]
[25]:=busted.test.theta_CT*busted.test.omega2*t*0.1512809665850036;
busted.test_Q_component_2[54]
[41]:=busted.test.theta_GT*busted.test.omega2*t*0.2936069988308121;
busted.test_Q_component_2[54]
```

```
[48]:=busted.test.theta_AG*busted.test.omega2*t*0.3408876964850085;
busted.test_Q_component_2[54]
[51]:=busted.test.theta_CG*busted.test.omega2*t*0.2496987119458206;
busted.test_Q_component_2[54]
[55]:=busted.test.theta_CG*busted.test.omega2*t*0.1063345886498611;
busted.test_Q_component_2[54]
[56]:=busted.test.theta_CT*t*0.419803077292116;
busted.test_Q_component_2[54]
[58]:=busted.test.theta_GT*busted.test.omega2*t*0.2208116300950855;
busted.test_Q_component_2[55]
[10]:=busted.test.theta_AT*busted.test.omega2*t*0.3102791451089904;
busted.test_Q_component_2[55]
[26]:=busted.test.theta_CT*busted.test.omega2*t*0.1512809665850036;
busted.test_Q_component_2[55]
[42]:=busted.test.theta_GT*busted.test.omega2*t*0.2936069988308121;
busted.test_Q_component_2[55]
[52]:=busted.test.theta_CG*busted.test.omega2*t*0.2496987119458206;
busted.test_Q_component_2[55]
[54]:=busted.test.theta_CG*busted.test.omega2*t*0.0447809360409677;
busted.test_Q_component_2[55]
[56]:=busted.test.theta_GT*busted.test.omega2*t*0.419803077292116;
busted.test_Q_component_2[55]
[59]:=busted.test.theta_GT*busted.test.omega2*t*0.2208116300950855;
busted.test_Q_component_2[56]
[11]:=busted.test.theta_AT*busted.test.omega2*t*0.3102791451089904;
busted.test_Q_component_2[56]
[27]:=busted.test.theta_CT*busted.test.omega2*t*0.1512809665850036;
busted.test_Q_component_2[56]
[43]:=busted.test.theta_GT*busted.test.omega2*t*0.2936069988308121;
busted.test_Q_component_2[56]
[49]:=busted.test.theta_AG*busted.test.omega2*t*0.3408876964850085;
busted.test_Q_component_2[56]
[53]:=busted.test.theta_CG*busted.test.omega2*t*0.2496987119458206;
busted.test_Q_component_2[56]
[54]:=busted.test.theta_CT*t*0.0447809360409677;
busted.test_Q_component_2[56]
[55]:=busted.test.theta_GT*busted.test.omega2*t*0.1063345886498611;
busted.test_Q_component_2[56]
[60]:=busted.test.theta_GT*busted.test.omega2*t*0.2208116300950855;
busted.test_Q_component_2[57]
[12]:=busted.test.theta_AT*busted.test.omega2*t*0.3102791451089904;
busted.test_Q_component_2[57]
[28]:=busted.test.theta_CT*t*0.1512809665850036;
busted.test_Q_component_2[57]
[44]:=busted.test.theta_GT*busted.test.omega2*t*0.2936069988308121;
busted.test_Q_component_2[57]
[50]:=busted.test.theta_CT*busted.test.omega2*t*0.2496987119458206;
busted.test_Q_component_2[57]
[58]:=busted.test.theta_AC*busted.test.omega2*t*0.0447809360409677;
busted.test_Q_component_2[57]
[59]:=busted.test.theta_AG*t*0.1063345886498611;
busted.test_Q_component_2[57]
[60]:=busted.test.theta_AT*busted.test.omega2*t*0.419803077292116;
busted.test_Q_component_2[58]
```

```
[13]:=busted.test.theta_AT*busted.test.omega2*t*0.3102791451089904;
busted.test_Q_component_2[58]
[29]:=busted.test.theta_CT*busted.test.omega2*t*0.1512809665850036;
busted.test_Q_component_2[58]
[45]:=busted.test.theta_GT*busted.test.omega2*t*0.2936069988308121;
busted.test_Q_component_2[58]
[48]:=busted.test.theta_AT*busted.test.omega2*t*0.3408876964850085;
busted.test_Q_component_2[58]
[51]:=busted.test.theta_CT*busted.test.omega2*t*0.2496987119458206;
busted.test_Q_component_2[58]
[54]:=busted.test.theta_GT*busted.test.omega2*t*0.1886019614740855;
busted.test_Q_component_2[58]
[57]:=busted.test.theta_AC*busted.test.omega2*t*0.4290813980170551;
busted.test_Q_component_2[58]
[59]:=busted.test.theta_CG*busted.test.omega2*t*0.1063345886498611;
busted.test_Q_component_2[58]
[60]:=busted.test.theta_CT*t*0.419803077292116;
busted.test_Q_component_2[59]
[14]:=busted.test.theta_AT*busted.test.omega2*t*0.3102791451089904;
busted.test_Q_component_2[59]
[30]:=busted.test.theta_CT*t*0.1512809665850036;
busted.test_Q_component_2[59]
[46]:=busted.test.theta_GT*busted.test.omega2*t*0.2936069988308121;
busted.test_Q_component_2[59]
[52]:=busted.test.theta_CT*busted.test.omega2*t*0.2496987119458206;
busted.test_Q_component_2[59]
[55]:=busted.test.theta_GT*busted.test.omega2*t*0.1886019614740855;
busted.test_Q_component_2[59]
[57]:=busted.test.theta_AG*t*0.4290813980170551;
busted.test_Q_component_2[59]
[58]:=busted.test.theta_CG*busted.test.omega2*t*0.0447809360409677;
busted.test_Q_component_2[59]
[60]:=busted.test.theta_GT*busted.test.omega2*t*0.419803077292116;
busted.test_Q_component_2[60]
[15]:=busted.test.theta_AT*busted.test.omega2*t*0.3102791451089904;
busted.test_Q_component_2[60]
[31]:=busted.test.theta_CT*busted.test.omega2*t*0.1512809665850036;
busted.test_Q_component_2[60]
[47]:=busted.test.theta_GT*busted.test.omega2*t*0.2936069988308121;
busted.test_Q_component_2[60]
[49]:=busted.test.theta_AT*busted.test.omega2*t*0.3408876964850085;
busted.test_Q_component_2[60]
[53]:=busted.test.theta_CT*busted.test.omega2*t*0.2496987119458206;
busted.test_Q_component_2[60]
[56]:=busted.test.theta_GT*busted.test.omega2*t*0.1886019614740855;
busted.test_Q_component_2[60]
[57]:=busted.test.theta_AT*busted.test.omega2*t*0.4290813980170551;
busted.test_Q_component_2[60]
[58]:=busted.test.theta_CT*t*0.0447809360409677;
busted.test_Q_component_2[60]
[59]:=busted.test.theta_GT*busted.test.omega2*t*0.1063345886498611;


busted.test_Q_component_3={61,61};
```

```
busted.test_Q_component_3[0]
[1]:=busted.test.theta_AC*busted.test.omega3*t*0.0447809360409677;
busted.test_Q_component_3[0]
[2]:=busted.test.theta_AG*t*0.1063345886498611;
busted.test_Q_component_3[0]
[3]:=busted.test.theta_AT*busted.test.omega3*t*0.419803077292116;
busted.test_Q_component_3[0]
[4]:=busted.test.theta_AC*busted.test.omega3*t*0.2496987119458206;
busted.test_Q_component_3[0]
[8]:=busted.test.theta_AG*busted.test.omega3*t*0.1886019614740855;
busted.test_Q_component_3[0]
[12]:=busted.test.theta_AT*busted.test.omega3*t*0.2208116300950855;
busted.test_Q_component_3[0]
[16]:=busted.test.theta_AC*busted.test.omega3*t*0.1512809665850036;
busted.test_Q_component_3[0]
[32]:=busted.test.theta_AG*busted.test.omega3*t*0.2936069988308121;
busted.test_Q_component_3[1]
[0]:=busted.test.theta_AC*busted.test.omega3*t*0.4290813980170551;
busted.test_Q_component_3[1]
[2]:=busted.test.theta_CG*busted.test.omega3*t*0.1063345886498611;
busted.test_Q_component_3[1]
[3]:=busted.test.theta_CT*t*0.419803077292116;
busted.test_Q_component_3[1]
[5]:=busted.test.theta_AC*busted.test.omega3*t*0.2496987119458206;
busted.test_Q_component_3[1]
[9]:=busted.test.theta_AG*busted.test.omega3*t*0.1886019614740855;
busted.test_Q_component_3[1]
[13]:=busted.test.theta_AT*busted.test.omega3*t*0.2208116300950855;
busted.test_Q_component_3[1]
[17]:=busted.test.theta_AC*busted.test.omega3*t*0.1512809665850036;
busted.test_Q_component_3[1]
[33]:=busted.test.theta_AG*busted.test.omega3*t*0.2936069988308121;
busted.test_Q_component_3[1]
[48]:=busted.test.theta_AT*busted.test.omega3*t*0.244832889475194;
busted.test_Q_component_3[2]
[0]:=busted.test.theta_AG*t*0.4290813980170551;
busted.test_Q_component_3[2]
[1]:=busted.test.theta_CG*busted.test.omega3*t*0.0447809360409677;
busted.test_Q_component_3[2]
[3]:=busted.test.theta_GT*busted.test.omega3*t*0.419803077292116;
busted.test_Q_component_3[2]
[6]:=busted.test.theta_AC*busted.test.omega3*t*0.2496987119458206;
busted.test_Q_component_3[2]
[10]:=busted.test.theta_AG*busted.test.omega3*t*0.1886019614740855;
busted.test_Q_component_3[2]
[14]:=busted.test.theta_AT*busted.test.omega3*t*0.2208116300950855;
busted.test_Q_component_3[2]
[18]:=busted.test.theta_AC*busted.test.omega3*t*0.1512809665850036;
busted.test_Q_component_3[2]
[34]:=busted.test.theta_AG*busted.test.omega3*t*0.2936069988308121;
busted.test_Q_component_3[3]
[0]:=busted.test.theta_AT*busted.test.omega3*t*0.4290813980170551;
busted.test_Q_component_3[3]
[1]:=busted.test.theta_CT*t*0.0447809360409677;
```

```
busted.test_Q_component_3[3]
[2]:=busted.test.theta_GT*busted.test.omega3*t*0.1063345886498611;
busted.test_Q_component_3[3]
[7]:=busted.test.theta_AC*busted.test.omega3*t*0.2496987119458206;
busted.test_Q_component_3[3]
[11]:=busted.test.theta_AG*busted.test.omega3*t*0.1886019614740855;
busted.test_Q_component_3[3]
[15]:=busted.test.theta_AT*busted.test.omega3*t*0.2208116300950855;
busted.test_Q_component_3[3]
[19]:=busted.test.theta_AC*busted.test.omega3*t*0.1512809665850036;
busted.test_Q_component_3[3]
[35]:=busted.test.theta_AG*busted.test.omega3*t*0.2936069988308121;
busted.test_Q_component_3[3]
[49]:=busted.test.theta_AT*busted.test.omega3*t*0.244832889475194;
busted.test_Q_component_3[4]
[0]:=busted.test.theta_AC*busted.test.omega3*t*0.3408876964850085;
busted.test_Q_component_3[4]
[5]:=busted.test.theta_AC*t*0.0447809360409677;
busted.test_Q_component_3[4]
[6]:=busted.test.theta_AG*t*0.1063345886498611;
busted.test_Q_component_3[4]
[7]:=busted.test.theta_AT*t*0.419803077292116;
busted.test_Q_component_3[4]
[8]:=busted.test.theta_CG*busted.test.omega3*t*0.1886019614740855;
busted.test_Q_component_3[4]
[12]:=busted.test.theta_CT*busted.test.omega3*t*0.2208116300950855;
busted.test_Q_component_3[4]
[20]:=busted.test.theta_AC*busted.test.omega3*t*0.1512809665850036;
busted.test_Q_component_3[4]
[36]:=busted.test.theta_AG*busted.test.omega3*t*0.2936069988308121;
busted.test_Q_component_3[4]
[50]:=busted.test.theta_AT*busted.test.omega3*t*0.244832889475194;
busted.test_Q_component_3[5]
[1]:=busted.test.theta_AC*busted.test.omega3*t*0.3408876964850085;
busted.test_Q_component_3[5]
[4]:=busted.test.theta_AC*t*0.4290813980170551;
busted.test_Q_component_3[5]
[6]:=busted.test.theta_CG*t*0.1063345886498611;
busted.test_Q_component_3[5]
[7]:=busted.test.theta_CT*t*0.419803077292116;
busted.test_Q_component_3[5]
[9]:=busted.test.theta_CG*busted.test.omega3*t*0.1886019614740855;
busted.test_Q_component_3[5]
[13]:=busted.test.theta_CT*busted.test.omega3*t*0.2208116300950855;
busted.test_Q_component_3[5]
[21]:=busted.test.theta_AC*busted.test.omega3*t*0.1512809665850036;
busted.test_Q_component_3[5]
[37]:=busted.test.theta_AG*busted.test.omega3*t*0.2936069988308121;
busted.test_Q_component_3[5]
[51]:=busted.test.theta_AT*busted.test.omega3*t*0.244832889475194;
busted.test_Q_component_3[6]
[2]:=busted.test.theta_AC*busted.test.omega3*t*0.3408876964850085;
busted.test_Q_component_3[6]
[4]:=busted.test.theta_AG*t*0.4290813980170551;
```

```
busted.test_Q_component_3[6]
[5]:=busted.test.theta_CG*t*0.0447809360409677;
busted.test_Q_component_3[6]
[7]:=busted.test.theta_GT*t*0.419803077292116;
busted.test_Q_component_3[6]
[10]:=busted.test.theta_CG*busted.test.omega3*t*0.1886019614740855;
busted.test_Q_component_3[6]
[14]:=busted.test.theta_CT*busted.test.omega3*t*0.2208116300950855;
busted.test_Q_component_3[6]
[22]:=busted.test.theta_AC*busted.test.omega3*t*0.1512809665850036;
busted.test_Q_component_3[6]
[38]:=busted.test.theta_AG*busted.test.omega3*t*0.2936069988308121;
busted.test_Q_component_3[6]
[52]:=busted.test.theta_AT*busted.test.omega3*t*0.244832889475194;
busted.test_Q_component_3[7]
[3]:=busted.test.theta_AC*busted.test.omega3*t*0.3408876964850085;
busted.test_Q_component_3[7]
[4]:=busted.test.theta_AT*t*0.4290813980170551;
busted.test_Q_component_3[7]
[5]:=busted.test.theta_CT*t*0.0447809360409677;
busted.test_Q_component_3[7]
[6]:=busted.test.theta_GT*t*0.1063345886498611;
busted.test_Q_component_3[7]
[11]:=busted.test.theta_CG*busted.test.omega3*t*0.1886019614740855;
busted.test_Q_component_3[7]
[15]:=busted.test.theta_CT*busted.test.omega3*t*0.2208116300950855;
busted.test_Q_component_3[7]
[23]:=busted.test.theta_AC*busted.test.omega3*t*0.1512809665850036;
busted.test_Q_component_3[7]
[39]:=busted.test.theta_AG*busted.test.omega3*t*0.2936069988308121;
busted.test_Q_component_3[7]
[53]:=busted.test.theta_AT*busted.test.omega3*t*0.244832889475194;
busted.test_Q_component_3[8]
[0]:=busted.test.theta_AG*busted.test.omega3*t*0.3408876964850085;
busted.test_Q_component_3[8]
[4]:=busted.test.theta_CG*busted.test.omega3*t*0.2496987119458206;
busted.test_Q_component_3[8]
[9]:=busted.test.theta_AC*busted.test.omega3*t*0.0447809360409677;
busted.test_Q_component_3[8]
[10]:=busted.test.theta_AG*t*0.1063345886498611;
busted.test_Q_component_3[8]
[11]:=busted.test.theta_AT*busted.test.omega3*t*0.419803077292116;
busted.test_Q_component_3[8]
[12]:=busted.test.theta_GT*busted.test.omega3*t*0.2208116300950855;
busted.test_Q_component_3[8]
[24]:=busted.test.theta_AC*t*0.1512809665850036;
busted.test_Q_component_3[8]
[40]:=busted.test.theta_AG*busted.test.omega3*t*0.2936069988308121;
busted.test_Q_component_3[9]
[1]:=busted.test.theta_AG*busted.test.omega3*t*0.3408876964850085;
busted.test_Q_component_3[9]
[5]:=busted.test.theta_CG*busted.test.omega3*t*0.2496987119458206;
busted.test_Q_component_3[9]
[8]:=busted.test.theta_AC*busted.test.omega3*t*0.4290813980170551;
```

```
busted.test_Q_component_3[9]
[10]:=busted.test.theta_CG*busted.test.omega3*t*0.1063345886498611;
busted.test_Q_component_3[9]
[11]:=busted.test.theta_CT*t*0.419803077292116;
busted.test_Q_component_3[9]
[13]:=busted.test.theta_GT*busted.test.omega3*t*0.2208116300950855;
busted.test_Q_component_3[9]
[25]:=busted.test.theta_AC*busted.test.omega3*t*0.1512809665850036;
busted.test_Q_component_3[9]
[41]:=busted.test.theta_AG*busted.test.omega3*t*0.2936069988308121;
busted.test_Q_component_3[9]
[54]:=busted.test.theta_AT*busted.test.omega3*t*0.244832889475194;
busted.test_Q_component_3[10]
[2]:=busted.test.theta_AG*busted.test.omega3*t*0.3408876964850085;
busted.test_Q_component_3[10]
[6]:=busted.test.theta_CG*busted.test.omega3*t*0.2496987119458206;
busted.test_Q_component_3[10]
[8]:=busted.test.theta_AG*t*0.4290813980170551;
busted.test_Q_component_3[10]
[9]:=busted.test.theta_CG*busted.test.omega3*t*0.0447809360409677;
busted.test_Q_component_3[10]
[11]:=busted.test.theta_GT*busted.test.omega3*t*0.419803077292116;
busted.test_Q_component_3[10]
[14]:=busted.test.theta_GT*busted.test.omega3*t*0.2208116300950855;
busted.test_Q_component_3[10]
[26]:=busted.test.theta_AC*t*0.1512809665850036;
busted.test_Q_component_3[10]
[42]:=busted.test.theta_AG*busted.test.omega3*t*0.2936069988308121;
busted.test_Q_component_3[10]
[55]:=busted.test.theta_AT*busted.test.omega3*t*0.244832889475194;
busted.test_Q_component_3[11]
[3]:=busted.test.theta_AG*busted.test.omega3*t*0.3408876964850085;
busted.test_Q_component_3[11]
[7]:=busted.test.theta_CG*busted.test.omega3*t*0.2496987119458206;
busted.test_Q_component_3[11]
[8]:=busted.test.theta_AT*busted.test.omega3*t*0.4290813980170551;
busted.test_Q_component_3[11]
[9]:=busted.test.theta_CT*t*0.0447809360409677;
busted.test_Q_component_3[11]
[10]:=busted.test.theta_GT*busted.test.omega3*t*0.1063345886498611;
busted.test_Q_component_3[11]
[15]:=busted.test.theta_GT*busted.test.omega3*t*0.2208116300950855;
busted.test_Q_component_3[11]
[27]:=busted.test.theta_AC*busted.test.omega3*t*0.1512809665850036;
busted.test_Q_component_3[11]
[43]:=busted.test.theta_AG*busted.test.omega3*t*0.2936069988308121;
busted.test_Q_component_3[11]
[56]:=busted.test.theta_AT*busted.test.omega3*t*0.244832889475194;
busted.test_Q_component_3[12]
[0]:=busted.test.theta_AT*busted.test.omega3*t*0.3408876964850085;
busted.test_Q_component_3[12]
[4]:=busted.test.theta_CT*busted.test.omega3*t*0.2496987119458206;
busted.test_Q_component_3[12]
[8]:=busted.test.theta_GT*busted.test.omega3*t*0.1886019614740855;
```

```
busted.test_Q_component_3[12]
[13]:=busted.test.theta_AC*t*0.0447809360409677;
busted.test_Q_component_3[12]
[14]:=busted.test.theta_AG*busted.test.omega3*t*0.1063345886498611;
busted.test_Q_component_3[12]
[15]:=busted.test.theta_AT*t*0.419803077292116;
busted.test_Q_component_3[12]
[28]:=busted.test.theta_AC*busted.test.omega3*t*0.1512809665850036;
busted.test_Q_component_3[12]
[44]:=busted.test.theta_AG*busted.test.omega3*t*0.2936069988308121;
busted.test_Q_component_3[12]
[57]:=busted.test.theta_AT*busted.test.omega3*t*0.244832889475194;
busted.test_Q_component_3[13]
[1]:=busted.test.theta_AT*busted.test.omega3*t*0.3408876964850085;
busted.test_Q_component_3[13]
[5]:=busted.test.theta_CT*busted.test.omega3*t*0.2496987119458206;
busted.test_Q_component_3[13]
[9]:=busted.test.theta_GT*busted.test.omega3*t*0.1886019614740855;
busted.test_Q_component_3[13]
[12]:=busted.test.theta_AC*t*0.4290813980170551;
busted.test_Q_component_3[13]
[14]:=busted.test.theta_CG*busted.test.omega3*t*0.1063345886498611;
busted.test_Q_component_3[13]
[15]:=busted.test.theta_CT*t*0.419803077292116;
busted.test_Q_component_3[13]
[29]:=busted.test.theta_AC*busted.test.omega3*t*0.1512809665850036;
busted.test_Q_component_3[13]
[45]:=busted.test.theta_AG*busted.test.omega3*t*0.2936069988308121;
busted.test_Q_component_3[13]
[58]:=busted.test.theta_AT*busted.test.omega3*t*0.244832889475194;
busted.test_Q_component_3[14]
[2]:=busted.test.theta_AT*busted.test.omega3*t*0.3408876964850085;
busted.test_Q_component_3[14]
[6]:=busted.test.theta_CT*busted.test.omega3*t*0.2496987119458206;
busted.test_Q_component_3[14]
[10]:=busted.test.theta_GT*busted.test.omega3*t*0.1886019614740855;
busted.test_Q_component_3[14]
[12]:=busted.test.theta_AG*busted.test.omega3*t*0.4290813980170551;
busted.test_Q_component_3[14]
[13]:=busted.test.theta_CG*busted.test.omega3*t*0.0447809360409677;
busted.test_Q_component_3[14]
[15]:=busted.test.theta_GT*busted.test.omega3*t*0.419803077292116;
busted.test_Q_component_3[14]
[30]:=busted.test.theta_AC*busted.test.omega3*t*0.1512809665850036;
busted.test_Q_component_3[14]
[46]:=busted.test.theta_AG*busted.test.omega3*t*0.2936069988308121;
busted.test_Q_component_3[14]
[59]:=busted.test.theta_AT*busted.test.omega3*t*0.244832889475194;
busted.test_Q_component_3[15]
[3]:=busted.test.theta_AT*busted.test.omega3*t*0.3408876964850085;
busted.test_Q_component_3[15]
[7]:=busted.test.theta_CT*busted.test.omega3*t*0.2496987119458206;
busted.test_Q_component_3[15]
[11]:=busted.test.theta_GT*busted.test.omega3*t*0.1886019614740855;
```

```
busted.test_Q_component_3[15]
[12]:=busted.test.theta_AT*t*0.4290813980170551;
busted.test_Q_component_3[15]
[13]:=busted.test.theta_CT*t*0.0447809360409677;
busted.test_Q_component_3[15]
[14]:=busted.test.theta_GT*busted.test.omega3*t*0.1063345886498611;
busted.test_Q_component_3[15]
[31]:=busted.test.theta_AC*busted.test.omega3*t*0.1512809665850036;
busted.test_Q_component_3[15]
[47]:=busted.test.theta_AG*busted.test.omega3*t*0.2936069988308121;
busted.test_Q_component_3[15]
[60]:=busted.test.theta_AT*busted.test.omega3*t*0.244832889475194;
busted.test_Q_component_3[16]
[0]:=busted.test.theta_AC*busted.test.omega3*t*0.3102791451089904;
busted.test_Q_component_3[16]
[17]:=busted.test.theta_AC*busted.test.omega3*t*0.0447809360409677;
busted.test_Q_component_3[16]
[18]:=busted.test.theta_AG*t*0.1063345886498611;
busted.test_Q_component_3[16]
[19]:=busted.test.theta_AT*busted.test.omega3*t*0.419803077292116;
busted.test_Q_component_3[16]
[20]:=busted.test.theta_AC*busted.test.omega3*t*0.2496987119458206;
busted.test_Q_component_3[16]
[24]:=busted.test.theta_AG*busted.test.omega3*t*0.1886019614740855;
busted.test_Q_component_3[16]
[28]:=busted.test.theta_AT*busted.test.omega3*t*0.2208116300950855;
busted.test_Q_component_3[16]
[32]:=busted.test.theta_CG*busted.test.omega3*t*0.2936069988308121;
busted.test_Q_component_3[17]
[1]:=busted.test.theta_AC*busted.test.omega3*t*0.3102791451089904;
busted.test_Q_component_3[17]
[16]:=busted.test.theta_AC*busted.test.omega3*t*0.4290813980170551;
busted.test_Q_component_3[17]
[18]:=busted.test.theta_CG*busted.test.omega3*t*0.1063345886498611;
busted.test_Q_component_3[17]
[19]:=busted.test.theta_CT*t*0.419803077292116;
busted.test_Q_component_3[17]
[21]:=busted.test.theta_AC*busted.test.omega3*t*0.2496987119458206;
busted.test_Q_component_3[17]
[25]:=busted.test.theta_AG*busted.test.omega3*t*0.1886019614740855;
busted.test_Q_component_3[17]
[29]:=busted.test.theta_AT*busted.test.omega3*t*0.2208116300950855;
busted.test_Q_component_3[17]
[33]:=busted.test.theta_CG*busted.test.omega3*t*0.2936069988308121;
busted.test_Q_component_3[17]
[48]:=busted.test.theta_CT*busted.test.omega3*t*0.244832889475194;
busted.test_Q_component_3[18]
[2]:=busted.test.theta_AC*busted.test.omega3*t*0.3102791451089904;
busted.test_Q_component_3[18]
[16]:=busted.test.theta_AG*t*0.4290813980170551;
busted.test_Q_component_3[18]
[17]:=busted.test.theta_CG*busted.test.omega3*t*0.0447809360409677;
busted.test_Q_component_3[18]
[19]:=busted.test.theta_GT*busted.test.omega3*t*0.419803077292116;
```

```
busted.test_Q_component_3[18]
[22]:=busted.test.theta_AC*busted.test.omega3*t*0.2496987119458206;
busted.test_Q_component_3[18]
[26]:=busted.test.theta_AG*busted.test.omega3*t*0.1886019614740855;
busted.test_Q_component_3[18]
[30]:=busted.test.theta_AT*busted.test.omega3*t*0.2208116300950855;
busted.test_Q_component_3[18]
[34]:=busted.test.theta_CG*busted.test.omega3*t*0.2936069988308121;
busted.test_Q_component_3[19]
[3]:=busted.test.theta_AC*busted.test.omega3*t*0.3102791451089904;
busted.test_Q_component_3[19]
[16]:=busted.test.theta_AT*busted.test.omega3*t*0.4290813980170551;
busted.test_Q_component_3[19]
[17]:=busted.test.theta_CT*t*0.0447809360409677;
busted.test_Q_component_3[19]
[18]:=busted.test.theta_GT*busted.test.omega3*t*0.1063345886498611;
busted.test_Q_component_3[19]
[23]:=busted.test.theta_AC*busted.test.omega3*t*0.2496987119458206;
busted.test_Q_component_3[19]
[27]:=busted.test.theta_AG*busted.test.omega3*t*0.1886019614740855;
busted.test_Q_component_3[19]
[31]:=busted.test.theta_AT*busted.test.omega3*t*0.2208116300950855;
busted.test_Q_component_3[19]
[35]:=busted.test.theta_CG*busted.test.omega3*t*0.2936069988308121;
busted.test_Q_component_3[19]
[49]:=busted.test.theta_CT*busted.test.omega3*t*0.244832889475194;
busted.test_Q_component_3[20]
[4]:=busted.test.theta_AC*busted.test.omega3*t*0.3102791451089904;
busted.test_Q_component_3[20]
[16]:=busted.test.theta_AC*busted.test.omega3*t*0.3408876964850085;
busted.test_Q_component_3[20]
[21]:=busted.test.theta_AC*t*0.0447809360409677;
busted.test_Q_component_3[20]
[22]:=busted.test.theta_AG*t*0.1063345886498611;
busted.test_Q_component_3[20]
[23]:=busted.test.theta_AT*t*0.419803077292116;
busted.test_Q_component_3[20]
[24]:=busted.test.theta_CG*busted.test.omega3*t*0.1886019614740855;
busted.test_Q_component_3[20]
[28]:=busted.test.theta_CT*busted.test.omega3*t*0.2208116300950855;
busted.test_Q_component_3[20]
[36]:=busted.test.theta_CG*busted.test.omega3*t*0.2936069988308121;
busted.test_Q_component_3[20]
[50]:=busted.test.theta_CT*busted.test.omega3*t*0.244832889475194;
busted.test_Q_component_3[21]
[5]:=busted.test.theta_AC*busted.test.omega3*t*0.3102791451089904;
busted.test_Q_component_3[21]
[17]:=busted.test.theta_AC*busted.test.omega3*t*0.3408876964850085;
busted.test_Q_component_3[21]
[20]:=busted.test.theta_AC*t*0.4290813980170551;
busted.test_Q_component_3[21]
[22]:=busted.test.theta_CG*t*0.1063345886498611;
busted.test_Q_component_3[21]
[23]:=busted.test.theta_CT*t*0.419803077292116;
```

```
busted.test_Q_component_3[21]
[25]:=busted.test.theta_CG*busted.test.omega3*t*0.1886019614740855;
busted.test_Q_component_3[21]
[29]:=busted.test.theta_CT*busted.test.omega3*t*0.2208116300950855;
busted.test_Q_component_3[21]
[37]:=busted.test.theta_CG*busted.test.omega3*t*0.2936069988308121;
busted.test_Q_component_3[21]
[51]:=busted.test.theta_CT*busted.test.omega3*t*0.244832889475194;
busted.test_Q_component_3[22]
[6]:=busted.test.theta_AC*busted.test.omega3*t*0.3102791451089904;
busted.test_Q_component_3[22]
[18]:=busted.test.theta_AC*busted.test.omega3*t*0.3408876964850085;
busted.test_Q_component_3[22]
[20]:=busted.test.theta_AG*t*0.4290813980170551;
busted.test_Q_component_3[22]
[21]:=busted.test.theta_CG*t*0.0447809360409677;
busted.test_Q_component_3[22]
[23]:=busted.test.theta_GT*t*0.419803077292116;
busted.test_Q_component_3[22]
[26]:=busted.test.theta_CG*busted.test.omega3*t*0.1886019614740855;
busted.test_Q_component_3[22]
[30]:=busted.test.theta_CT*busted.test.omega3*t*0.2208116300950855;
busted.test_Q_component_3[22]
[38]:=busted.test.theta_CG*busted.test.omega3*t*0.2936069988308121;
busted.test_Q_component_3[22]
[52]:=busted.test.theta_CT*busted.test.omega3*t*0.244832889475194;
busted.test_Q_component_3[23]
[7]:=busted.test.theta_AC*busted.test.omega3*t*0.3102791451089904;
busted.test_Q_component_3[23]
[19]:=busted.test.theta_AC*busted.test.omega3*t*0.3408876964850085;
busted.test_Q_component_3[23]
[20]:=busted.test.theta_AT*t*0.4290813980170551;
busted.test_Q_component_3[23]
[21]:=busted.test.theta_CT*t*0.0447809360409677;
busted.test_Q_component_3[23]
[22]:=busted.test.theta_GT*t*0.1063345886498611;
busted.test_Q_component_3[23]
[27]:=busted.test.theta_CG*busted.test.omega3*t*0.1886019614740855;
busted.test_Q_component_3[23]
[31]:=busted.test.theta_CT*busted.test.omega3*t*0.2208116300950855;
busted.test_Q_component_3[23]
[39]:=busted.test.theta_CG*busted.test.omega3*t*0.2936069988308121;
busted.test_Q_component_3[23]
[53]:=busted.test.theta_CT*busted.test.omega3*t*0.244832889475194;
busted.test_Q_component_3[24]
[8]:=busted.test.theta_AC*t*0.3102791451089904;
busted.test_Q_component_3[24]
[16]:=busted.test.theta_AG*busted.test.omega3*t*0.3408876964850085;
busted.test_Q_component_3[24]
[20]:=busted.test.theta_CG*busted.test.omega3*t*0.2496987119458206;
busted.test_Q_component_3[24]
[25]:=busted.test.theta_AC*t*0.0447809360409677;
busted.test_Q_component_3[24]
[26]:=busted.test.theta_AG*t*0.1063345886498611;
```

```
busted.test_Q_component_3[24]
[27]:=busted.test.theta_AT*t*0.419803077292116;
busted.test_Q_component_3[24]
[28]:=busted.test.theta_GT*busted.test.omega3*t*0.2208116300950855;
busted.test_Q_component_3[24]
[40]:=busted.test.theta_CG*busted.test.omega3*t*0.2936069988308121;
busted.test_Q_component_3[25]
[9]:=busted.test.theta_AC*busted.test.omega3*t*0.3102791451089904;
busted.test_Q_component_3[25]
[17]:=busted.test.theta_AG*busted.test.omega3*t*0.3408876964850085;
busted.test_Q_component_3[25]
[21]:=busted.test.theta_CG*busted.test.omega3*t*0.2496987119458206;
busted.test_Q_component_3[25]
[24]:=busted.test.theta_AC*t*0.4290813980170551;
busted.test_Q_component_3[25]
[26]:=busted.test.theta_CG*t*0.1063345886498611;
busted.test_Q_component_3[25]
[27]:=busted.test.theta_CT*t*0.419803077292116;
busted.test_Q_component_3[25]
[29]:=busted.test.theta_GT*busted.test.omega3*t*0.2208116300950855;
busted.test_Q_component_3[25]
[41]:=busted.test.theta_CG*busted.test.omega3*t*0.2936069988308121;
busted.test_Q_component_3[25]
[54]:=busted.test.theta_CT*busted.test.omega3*t*0.244832889475194;
busted.test_Q_component_3[26]
[10]:=busted.test.theta_AC*t*0.3102791451089904;
busted.test_Q_component_3[26]
[18]:=busted.test.theta_AG*busted.test.omega3*t*0.3408876964850085;
busted.test_Q_component_3[26]
[22]:=busted.test.theta_CG*busted.test.omega3*t*0.2496987119458206;
busted.test_Q_component_3[26]
[24]:=busted.test.theta_AG*t*0.4290813980170551;
busted.test_Q_component_3[26]
[25]:=busted.test.theta_CG*t*0.0447809360409677;
busted.test_Q_component_3[26]
[27]:=busted.test.theta_GT*t*0.419803077292116;
busted.test_Q_component_3[26]
[30]:=busted.test.theta_GT*busted.test.omega3*t*0.2208116300950855;
busted.test_Q_component_3[26]
[42]:=busted.test.theta_CG*busted.test.omega3*t*0.2936069988308121;
busted.test_Q_component_3[26]
[55]:=busted.test.theta_CT*busted.test.omega3*t*0.244832889475194;
busted.test_Q_component_3[27]
[11]:=busted.test.theta_AC*busted.test.omega3*t*0.3102791451089904;
busted.test_Q_component_3[27]
[19]:=busted.test.theta_AG*busted.test.omega3*t*0.3408876964850085;
busted.test_Q_component_3[27]
[23]:=busted.test.theta_CG*busted.test.omega3*t*0.2496987119458206;
busted.test_Q_component_3[27]
[24]:=busted.test.theta_AT*t*0.4290813980170551;
busted.test_Q_component_3[27]
[25]:=busted.test.theta_CT*t*0.0447809360409677;
busted.test_Q_component_3[27]
[26]:=busted.test.theta_GT*t*0.1063345886498611;
```

```
busted.test_Q_component_3[27]
[31]:=busted.test.theta_GT*busted.test.omega3*t*0.2208116300950855;
busted.test_Q_component_3[27]
[43]:=busted.test.theta_CG*busted.test.omega3*t*0.2936069988308121;
busted.test_Q_component_3[27]
[56]:=busted.test.theta_CT*busted.test.omega3*t*0.244832889475194;
busted.test_Q_component_3[28]
[12]:=busted.test.theta_AC*busted.test.omega3*t*0.3102791451089904;
busted.test_Q_component_3[28]
[16]:=busted.test.theta_AT*busted.test.omega3*t*0.3408876964850085;
busted.test_Q_component_3[28]
[20]:=busted.test.theta_CT*busted.test.omega3*t*0.2496987119458206;
busted.test_Q_component_3[28]
[24]:=busted.test.theta_GT*busted.test.omega3*t*0.1886019614740855;
busted.test_Q_component_3[28]
[29]:=busted.test.theta_AC*t*0.0447809360409677;
busted.test_Q_component_3[28]
[30]:=busted.test.theta_AG*t*0.1063345886498611;
busted.test_Q_component_3[28]
[31]:=busted.test.theta_AT*t*0.419803077292116;
busted.test_Q_component_3[28]
[44]:=busted.test.theta_CG*busted.test.omega3*t*0.2936069988308121;
busted.test_Q_component_3[28]
[57]:=busted.test.theta_CT*t*0.244832889475194;
busted.test_Q_component_3[29]
[13]:=busted.test.theta_AC*busted.test.omega3*t*0.3102791451089904;
busted.test_Q_component_3[29]
[17]:=busted.test.theta_AT*busted.test.omega3*t*0.3408876964850085;
busted.test_Q_component_3[29]
[21]:=busted.test.theta_CT*busted.test.omega3*t*0.2496987119458206;
busted.test_Q_component_3[29]
[25]:=busted.test.theta_GT*busted.test.omega3*t*0.1886019614740855;
busted.test_Q_component_3[29]
[28]:=busted.test.theta_AC*t*0.4290813980170551;
busted.test_Q_component_3[29]
[30]:=busted.test.theta_CG*t*0.1063345886498611;
busted.test_Q_component_3[29]
[31]:=busted.test.theta_CT*t*0.419803077292116;
busted.test_Q_component_3[29]
[45]:=busted.test.theta_CG*busted.test.omega3*t*0.2936069988308121;
busted.test_Q_component_3[29]
[58]:=busted.test.theta_CT*busted.test.omega3*t*0.244832889475194;
busted.test_Q_component_3[30]
[14]:=busted.test.theta_AC*busted.test.omega3*t*0.3102791451089904;
busted.test_Q_component_3[30]
[18]:=busted.test.theta_AT*busted.test.omega3*t*0.3408876964850085;
busted.test_Q_component_3[30]
[22]:=busted.test.theta_CT*busted.test.omega3*t*0.2496987119458206;
busted.test_Q_component_3[30]
[26]:=busted.test.theta_GT*busted.test.omega3*t*0.1886019614740855;
busted.test_Q_component_3[30]
[28]:=busted.test.theta_AG*t*0.4290813980170551;
busted.test_Q_component_3[30]
[29]:=busted.test.theta_CG*t*0.0447809360409677;
```

```
busted.test_Q_component_3[30]
[31]:=busted.test.theta_GT*t*0.419803077292116;
busted.test_Q_component_3[30]
[46]:=busted.test.theta_CG*busted.test.omega3*t*0.2936069988308121;
busted.test_Q_component_3[30]
[59]:=busted.test.theta_CT*t*0.244832889475194;
busted.test_Q_component_3[31]
[15]:=busted.test.theta_AC*busted.test.omega3*t*0.3102791451089904;
busted.test_Q_component_3[31]
[19]:=busted.test.theta_AT*busted.test.omega3*t*0.3408876964850085;
busted.test_Q_component_3[31]
[23]:=busted.test.theta_CT*busted.test.omega3*t*0.2496987119458206;
busted.test_Q_component_3[31]
[27]:=busted.test.theta_GT*busted.test.omega3*t*0.1886019614740855;
busted.test_Q_component_3[31]
[28]:=busted.test.theta_AT*t*0.4290813980170551;
busted.test_Q_component_3[31]
[29]:=busted.test.theta_CT*t*0.0447809360409677;
busted.test_Q_component_3[31]
[30]:=busted.test.theta_GT*t*0.1063345886498611;
busted.test_Q_component_3[31]
[47]:=busted.test.theta_CG*busted.test.omega3*t*0.2936069988308121;
busted.test_Q_component_3[31]
[60]:=busted.test.theta_CT*busted.test.omega3*t*0.244832889475194;
busted.test_Q_component_3[32]
[0]:=busted.test.theta_AG*busted.test.omega3*t*0.3102791451089904;
busted.test_Q_component_3[32]
[16]:=busted.test.theta_CG*busted.test.omega3*t*0.1512809665850036;
busted.test_Q_component_3[32]
[33]:=busted.test.theta_AC*busted.test.omega3*t*0.0447809360409677;
busted.test_Q_component_3[32]
[34]:=busted.test.theta_AG*t*0.1063345886498611;
busted.test_Q_component_3[32]
[35]:=busted.test.theta_AT*busted.test.omega3*t*0.419803077292116;
busted.test_Q_component_3[32]
[36]:=busted.test.theta_AC*busted.test.omega3*t*0.2496987119458206;
busted.test_Q_component_3[32]
[40]:=busted.test.theta_AG*busted.test.omega3*t*0.1886019614740855;
busted.test_Q_component_3[32]
[44]:=busted.test.theta_AT*busted.test.omega3*t*0.2208116300950855;
busted.test_Q_component_3[33]
[1]:=busted.test.theta_AG*busted.test.omega3*t*0.3102791451089904;
busted.test_Q_component_3[33]
[17]:=busted.test.theta_CG*busted.test.omega3*t*0.1512809665850036;
busted.test_Q_component_3[33]
[32]:=busted.test.theta_AC*busted.test.omega3*t*0.4290813980170551;
busted.test_Q_component_3[33]
[34]:=busted.test.theta_CG*busted.test.omega3*t*0.1063345886498611;
busted.test_Q_component_3[33]
[35]:=busted.test.theta_CT*t*0.419803077292116;
busted.test_Q_component_3[33]
[37]:=busted.test.theta_AC*busted.test.omega3*t*0.2496987119458206;
busted.test_Q_component_3[33]
[41]:=busted.test.theta_AG*busted.test.omega3*t*0.1886019614740855;
```

```
busted.test_Q_component_3[33]
[45]:=busted.test.theta_AT*busted.test.omega3*t*0.2208116300950855;
busted.test_Q_component_3[33]
[48]:=busted.test.theta_GT*busted.test.omega3*t*0.244832889475194;
busted.test_Q_component_3[34]
[2]:=busted.test.theta_AG*busted.test.omega3*t*0.3102791451089904;
busted.test_Q_component_3[34]
[18]:=busted.test.theta_CG*busted.test.omega3*t*0.1512809665850036;
busted.test_Q_component_3[34]
[32]:=busted.test.theta_AG*t*0.4290813980170551;
busted.test_Q_component_3[34]
[33]:=busted.test.theta_CG*busted.test.omega3*t*0.0447809360409677;
busted.test_Q_component_3[34]
[35]:=busted.test.theta_GT*busted.test.omega3*t*0.419803077292116;
busted.test_Q_component_3[34]
[38]:=busted.test.theta_AC*busted.test.omega3*t*0.2496987119458206;
busted.test_Q_component_3[34]
[42]:=busted.test.theta_AG*busted.test.omega3*t*0.1886019614740855;
busted.test_Q_component_3[34]
[46]:=busted.test.theta_AT*busted.test.omega3*t*0.2208116300950855;
busted.test_Q_component_3[35]
[3]:=busted.test.theta_AG*busted.test.omega3*t*0.3102791451089904;
busted.test_Q_component_3[35]
[19]:=busted.test.theta_CG*busted.test.omega3*t*0.1512809665850036;
busted.test_Q_component_3[35]
[32]:=busted.test.theta_AT*busted.test.omega3*t*0.4290813980170551;
busted.test_Q_component_3[35]
[33]:=busted.test.theta_CT*t*0.0447809360409677;
busted.test_Q_component_3[35]
[34]:=busted.test.theta_GT*busted.test.omega3*t*0.1063345886498611;
busted.test_Q_component_3[35]
[39]:=busted.test.theta_AC*busted.test.omega3*t*0.2496987119458206;
busted.test_Q_component_3[35]
[43]:=busted.test.theta_AG*busted.test.omega3*t*0.1886019614740855;
busted.test_Q_component_3[35]
[47]:=busted.test.theta_AT*busted.test.omega3*t*0.2208116300950855;
busted.test_Q_component_3[35]
[49]:=busted.test.theta_GT*busted.test.omega3*t*0.244832889475194;
busted.test_Q_component_3[36]
[4]:=busted.test.theta_AG*busted.test.omega3*t*0.3102791451089904;
busted.test_Q_component_3[36]
[20]:=busted.test.theta_CG*busted.test.omega3*t*0.1512809665850036;
busted.test_Q_component_3[36]
[32]:=busted.test.theta_AC*busted.test.omega3*t*0.3408876964850085;
busted.test_Q_component_3[36]
[37]:=busted.test.theta_AC*t*0.0447809360409677;
busted.test_Q_component_3[36]
[38]:=busted.test.theta_AG*t*0.1063345886498611;
busted.test_Q_component_3[36]
[39]:=busted.test.theta_AT*t*0.419803077292116;
busted.test_Q_component_3[36]
[40]:=busted.test.theta_CG*busted.test.omega3*t*0.1886019614740855;
busted.test_Q_component_3[36]
[44]:=busted.test.theta_CT*busted.test.omega3*t*0.2208116300950855;
```

```
busted.test_Q_component_3[36]
[50]:=busted.test.theta_GT*busted.test.omega3*t*0.244832889475194;
busted.test_Q_component_3[37]
[5]:=busted.test.theta_AG*busted.test.omega3*t*0.3102791451089904;
busted.test_Q_component_3[37]
[21]:=busted.test.theta_CG*busted.test.omega3*t*0.1512809665850036;
busted.test_Q_component_3[37]
[33]:=busted.test.theta_AC*busted.test.omega3*t*0.3408876964850085;
busted.test_Q_component_3[37]
[36]:=busted.test.theta_AC*t*0.4290813980170551;
busted.test_Q_component_3[37]
[38]:=busted.test.theta_CG*t*0.1063345886498611;
busted.test_Q_component_3[37]
[39]:=busted.test.theta_CT*t*0.419803077292116;
busted.test_Q_component_3[37]
[41]:=busted.test.theta_CG*busted.test.omega3*t*0.1886019614740855;
busted.test_Q_component_3[37]
[45]:=busted.test.theta_CT*busted.test.omega3*t*0.2208116300950855;
busted.test_Q_component_3[37]
[51]:=busted.test.theta_GT*busted.test.omega3*t*0.244832889475194;
busted.test_Q_component_3[38]
[6]:=busted.test.theta_AG*busted.test.omega3*t*0.3102791451089904;
busted.test_Q_component_3[38]
[22]:=busted.test.theta_CG*busted.test.omega3*t*0.1512809665850036;
busted.test_Q_component_3[38]
[34]:=busted.test.theta_AC*busted.test.omega3*t*0.3408876964850085;
busted.test_Q_component_3[38]
[36]:=busted.test.theta_AG*t*0.4290813980170551;
busted.test_Q_component_3[38]
[37]:=busted.test.theta_CG*t*0.0447809360409677;
busted.test_Q_component_3[38]
[39]:=busted.test.theta_GT*t*0.419803077292116;
busted.test_Q_component_3[38]
[42]:=busted.test.theta_CG*busted.test.omega3*t*0.1886019614740855;
busted.test_Q_component_3[38]
[46]:=busted.test.theta_CT*busted.test.omega3*t*0.2208116300950855;
busted.test_Q_component_3[38]
[52]:=busted.test.theta_GT*busted.test.omega3*t*0.244832889475194;
busted.test_Q_component_3[39]
[7]:=busted.test.theta_AG*busted.test.omega3*t*0.3102791451089904;
busted.test_Q_component_3[39]
[23]:=busted.test.theta_CG*busted.test.omega3*t*0.1512809665850036;
busted.test_Q_component_3[39]
[35]:=busted.test.theta_AC*busted.test.omega3*t*0.3408876964850085;
busted.test_Q_component_3[39]
[36]:=busted.test.theta_AT*t*0.4290813980170551;
busted.test_Q_component_3[39]
[37]:=busted.test.theta_CT*t*0.0447809360409677;
busted.test_Q_component_3[39]
[38]:=busted.test.theta_GT*t*0.1063345886498611;
busted.test_Q_component_3[39]
[43]:=busted.test.theta_CG*busted.test.omega3*t*0.1886019614740855;
busted.test_Q_component_3[39]
[47]:=busted.test.theta_CT*busted.test.omega3*t*0.2208116300950855;
```

```
busted.test_Q_component_3[39]
[53]:=busted.test.theta_GT*busted.test.omega3*t*0.244832889475194;
busted.test_Q_component_3[40]
[8]:=busted.test.theta_AG*busted.test.omega3*t*0.3102791451089904;
busted.test_Q_component_3[40]
[24]:=busted.test.theta_CG*busted.test.omega3*t*0.1512809665850036;
busted.test_Q_component_3[40]
[32]:=busted.test.theta_AG*busted.test.omega3*t*0.3408876964850085;
busted.test_Q_component_3[40]
[36]:=busted.test.theta_CG*busted.test.omega3*t*0.2496987119458206;
busted.test_Q_component_3[40]
[41]:=busted.test.theta_AC*t*0.0447809360409677;
busted.test_Q_component_3[40]
[42]:=busted.test.theta_AG*t*0.1063345886498611;
busted.test_Q_component_3[40]
[43]:=busted.test.theta_AT*t*0.419803077292116;
busted.test_Q_component_3[40]
[44]:=busted.test.theta_GT*busted.test.omega3*t*0.2208116300950855;
busted.test_Q_component_3[41]
[9]:=busted.test.theta_AG*busted.test.omega3*t*0.3102791451089904;
busted.test_Q_component_3[41]
[25]:=busted.test.theta_CG*busted.test.omega3*t*0.1512809665850036;
busted.test_Q_component_3[41]
[33]:=busted.test.theta_AG*busted.test.omega3*t*0.3408876964850085;
busted.test_Q_component_3[41]
[37]:=busted.test.theta_CG*busted.test.omega3*t*0.2496987119458206;
busted.test_Q_component_3[41]
[40]:=busted.test.theta_AC*t*0.4290813980170551;
busted.test_Q_component_3[41]
[42]:=busted.test.theta_CG*t*0.1063345886498611;
busted.test_Q_component_3[41]
[43]:=busted.test.theta_CT*t*0.419803077292116;
busted.test_Q_component_3[41]
[45]:=busted.test.theta_GT*busted.test.omega3*t*0.2208116300950855;
busted.test_Q_component_3[41]
[54]:=busted.test.theta_GT*busted.test.omega3*t*0.244832889475194;
busted.test_Q_component_3[42]
[10]:=busted.test.theta_AG*busted.test.omega3*t*0.3102791451089904;
busted.test_Q_component_3[42]
[26]:=busted.test.theta_CG*busted.test.omega3*t*0.1512809665850036;
busted.test_Q_component_3[42]
[34]:=busted.test.theta_AG*busted.test.omega3*t*0.3408876964850085;
busted.test_Q_component_3[42]
[38]:=busted.test.theta_CG*busted.test.omega3*t*0.2496987119458206;
busted.test_Q_component_3[42]
[40]:=busted.test.theta_AG*t*0.4290813980170551;
busted.test_Q_component_3[42]
[41]:=busted.test.theta_CG*t*0.0447809360409677;
busted.test_Q_component_3[42]
[43]:=busted.test.theta_GT*t*0.419803077292116;
busted.test_Q_component_3[42]
[46]:=busted.test.theta_GT*busted.test.omega3*t*0.2208116300950855;
busted.test_Q_component_3[42]
[55]:=busted.test.theta_GT*busted.test.omega3*t*0.244832889475194;
```

```
busted.test_Q_component_3[43]
[11]:=busted.test.theta_AG*busted.test.omega3*t*0.3102791451089904;
busted.test_Q_component_3[43]
[27]:=busted.test.theta_CG*busted.test.omega3*t*0.1512809665850036;
busted.test_Q_component_3[43]
[35]:=busted.test.theta_AG*busted.test.omega3*t*0.3408876964850085;
busted.test_Q_component_3[43]
[39]:=busted.test.theta_CG*busted.test.omega3*t*0.2496987119458206;
busted.test_Q_component_3[43]
[40]:=busted.test.theta_AT*t*0.4290813980170551;
busted.test_Q_component_3[43]
[41]:=busted.test.theta_CT*t*0.0447809360409677;
busted.test_Q_component_3[43]
[42]:=busted.test.theta_GT*t*0.1063345886498611;
busted.test_Q_component_3[43]
[47]:=busted.test.theta_GT*busted.test.omega3*t*0.2208116300950855;
busted.test_Q_component_3[43]
[56]:=busted.test.theta_GT*busted.test.omega3*t*0.244832889475194;
busted.test_Q_component_3[44]
[12]:=busted.test.theta_AG*busted.test.omega3*t*0.3102791451089904;
busted.test_Q_component_3[44]
[28]:=busted.test.theta_CG*busted.test.omega3*t*0.1512809665850036;
busted.test_Q_component_3[44]
[32]:=busted.test.theta_AT*busted.test.omega3*t*0.3408876964850085;
busted.test_Q_component_3[44]
[36]:=busted.test.theta_CT*busted.test.omega3*t*0.2496987119458206;
busted.test_Q_component_3[44]
[40]:=busted.test.theta_GT*busted.test.omega3*t*0.1886019614740855;
busted.test_Q_component_3[44]
[45]:=busted.test.theta_AC*t*0.0447809360409677;
busted.test_Q_component_3[44]
[46]:=busted.test.theta_AG*t*0.1063345886498611;
busted.test_Q_component_3[44]
[47]:=busted.test.theta_AT*t*0.419803077292116;
busted.test_Q_component_3[44]
[57]:=busted.test.theta_GT*busted.test.omega3*t*0.244832889475194;
busted.test_Q_component_3[45]
[13]:=busted.test.theta_AG*busted.test.omega3*t*0.3102791451089904;
busted.test_Q_component_3[45]
[29]:=busted.test.theta_CG*busted.test.omega3*t*0.1512809665850036;
busted.test_Q_component_3[45]
[33]:=busted.test.theta_AT*busted.test.omega3*t*0.3408876964850085;
busted.test_Q_component_3[45]
[37]:=busted.test.theta_CT*busted.test.omega3*t*0.2496987119458206;
busted.test_Q_component_3[45]
[41]:=busted.test.theta_GT*busted.test.omega3*t*0.1886019614740855;
busted.test_Q_component_3[45]
[44]:=busted.test.theta_AC*t*0.4290813980170551;
busted.test_Q_component_3[45]
[46]:=busted.test.theta_CG*t*0.1063345886498611;
busted.test_Q_component_3[45]
[47]:=busted.test.theta_CT*t*0.419803077292116;
busted.test_Q_component_3[45]
[58]:=busted.test.theta_GT*busted.test.omega3*t*0.244832889475194;
```

```
busted.test_Q_component_3[46]
[14]:=busted.test.theta_AG*busted.test.omega3*t*0.3102791451089904;
busted.test_Q_component_3[46]
[30]:=busted.test.theta_CG*busted.test.omega3*t*0.1512809665850036;
busted.test_Q_component_3[46]
[34]:=busted.test.theta_AT*busted.test.omega3*t*0.3408876964850085;
busted.test_Q_component_3[46]
[38]:=busted.test.theta_CT*busted.test.omega3*t*0.2496987119458206;
busted.test_Q_component_3[46]
[42]:=busted.test.theta_GT*busted.test.omega3*t*0.1886019614740855;
busted.test_Q_component_3[46]
[44]:=busted.test.theta_AG*t*0.4290813980170551;
busted.test_Q_component_3[46]
[45]:=busted.test.theta_CG*t*0.0447809360409677;
busted.test_Q_component_3[46]
[47]:=busted.test.theta_GT*t*0.419803077292116;
busted.test_Q_component_3[46]
[59]:=busted.test.theta_GT*busted.test.omega3*t*0.244832889475194;
busted.test_Q_component_3[47]
[15]:=busted.test.theta_AG*busted.test.omega3*t*0.3102791451089904;
busted.test_Q_component_3[47]
[31]:=busted.test.theta_CG*busted.test.omega3*t*0.1512809665850036;
busted.test_Q_component_3[47]
[35]:=busted.test.theta_AT*busted.test.omega3*t*0.3408876964850085;
busted.test_Q_component_3[47]
[39]:=busted.test.theta_CT*busted.test.omega3*t*0.2496987119458206;
busted.test_Q_component_3[47]
[43]:=busted.test.theta_GT*busted.test.omega3*t*0.1886019614740855;
busted.test_Q_component_3[47]
[44]:=busted.test.theta_AT*t*0.4290813980170551;
busted.test_Q_component_3[47]
[45]:=busted.test.theta_CT*t*0.0447809360409677;
busted.test_Q_component_3[47]
[46]:=busted.test.theta_GT*t*0.1063345886498611;
busted.test_Q_component_3[47]
[60]:=busted.test.theta_GT*busted.test.omega3*t*0.244832889475194;
busted.test_Q_component_3[48]
[1]:=busted.test.theta_AT*busted.test.omega3*t*0.3102791451089904;
busted.test_Q_component_3[48]
[17]:=busted.test.theta_CT*busted.test.omega3*t*0.1512809665850036;
busted.test_Q_component_3[48]
[33]:=busted.test.theta_GT*busted.test.omega3*t*0.2936069988308121;
busted.test_Q_component_3[48]
[49]:=busted.test.theta_CT*t*0.419803077292116;
busted.test_Q_component_3[48]
[51]:=busted.test.theta_AC*busted.test.omega3*t*0.2496987119458206;
busted.test_Q_component_3[48]
[54]:=busted.test.theta_AG*busted.test.omega3*t*0.1886019614740855;
busted.test_Q_component_3[48]
[58]:=busted.test.theta_AT*busted.test.omega3*t*0.2208116300950855;
busted.test_Q_component_3[49]
[3]:=busted.test.theta_AT*busted.test.omega3*t*0.3102791451089904;
busted.test_Q_component_3[49]
[19]:=busted.test.theta_CT*busted.test.omega3*t*0.1512809665850036;
```

```
busted.test_Q_component_3[49]
[35]:=busted.test.theta_GT*busted.test.omega3*t*0.2936069988308121;
busted.test_Q_component_3[49]
[48]:=busted.test.theta_CT*t*0.0447809360409677;
busted.test_Q_component_3[49]
[53]:=busted.test.theta_AC*busted.test.omega3*t*0.2496987119458206;
busted.test_Q_component_3[49]
[56]:=busted.test.theta_AG*busted.test.omega3*t*0.1886019614740855;
busted.test_Q_component_3[49]
[60]:=busted.test.theta_AT*busted.test.omega3*t*0.2208116300950855;
busted.test_Q_component_3[50]
[4]:=busted.test.theta_AT*busted.test.omega3*t*0.3102791451089904;
busted.test_Q_component_3[50]
[20]:=busted.test.theta_CT*busted.test.omega3*t*0.1512809665850036;
busted.test_Q_component_3[50]
[36]:=busted.test.theta_GT*busted.test.omega3*t*0.2936069988308121;
busted.test_Q_component_3[50]
[51]:=busted.test.theta_AC*t*0.0447809360409677;
busted.test_Q_component_3[50]
[52]:=busted.test.theta_AG*t*0.1063345886498611;
busted.test_Q_component_3[50]
[53]:=busted.test.theta_AT*t*0.419803077292116;
busted.test_Q_component_3[50]
[57]:=busted.test.theta_CT*busted.test.omega3*t*0.2208116300950855;
busted.test_Q_component_3[51]
[5]:=busted.test.theta_AT*busted.test.omega3*t*0.3102791451089904;
busted.test_Q_component_3[51]
[21]:=busted.test.theta_CT*busted.test.omega3*t*0.1512809665850036;
busted.test_Q_component_3[51]
[37]:=busted.test.theta_GT*busted.test.omega3*t*0.2936069988308121;
busted.test_Q_component_3[51]
[48]:=busted.test.theta_AC*busted.test.omega3*t*0.3408876964850085;
busted.test_Q_component_3[51]
[50]:=busted.test.theta_AC*t*0.4290813980170551;
busted.test_Q_component_3[51]
[52]:=busted.test.theta_CG*t*0.1063345886498611;
busted.test_Q_component_3[51]
[53]:=busted.test.theta_CT*t*0.419803077292116;
busted.test_Q_component_3[51]
[54]:=busted.test.theta_CG*busted.test.omega3*t*0.1886019614740855;
busted.test_Q_component_3[51]
[58]:=busted.test.theta_CT*busted.test.omega3*t*0.2208116300950855;
busted.test_Q_component_3[52]
[6]:=busted.test.theta_AT*busted.test.omega3*t*0.3102791451089904;
busted.test_Q_component_3[52]
[22]:=busted.test.theta_CT*busted.test.omega3*t*0.1512809665850036;
busted.test_Q_component_3[52]
[38]:=busted.test.theta_GT*busted.test.omega3*t*0.2936069988308121;
busted.test_Q_component_3[52]
[50]:=busted.test.theta_AG*t*0.4290813980170551;
busted.test_Q_component_3[52]
[51]:=busted.test.theta_CG*t*0.0447809360409677;
busted.test_Q_component_3[52]
[53]:=busted.test.theta_GT*t*0.419803077292116;
```

```
busted.test_Q_component_3[52]
[55]:=busted.test.theta_CG*busted.test.omega3*t*0.1886019614740855;
busted.test_Q_component_3[52]
[59]:=busted.test.theta_CT*busted.test.omega3*t*0.2208116300950855;
busted.test_Q_component_3[53]
[7]:=busted.test.theta_AT*busted.test.omega3*t*0.3102791451089904;
busted.test_Q_component_3[53]
[23]:=busted.test.theta_CT*busted.test.omega3*t*0.1512809665850036;
busted.test_Q_component_3[53]
[39]:=busted.test.theta_GT*busted.test.omega3*t*0.2936069988308121;
busted.test_Q_component_3[53]
[49]:=busted.test.theta_AC*busted.test.omega3*t*0.3408876964850085;
busted.test_Q_component_3[53]
[50]:=busted.test.theta_AT*t*0.4290813980170551;
busted.test_Q_component_3[53]
[51]:=busted.test.theta_CT*t*0.0447809360409677;
busted.test_Q_component_3[53]
[52]:=busted.test.theta_GT*t*0.1063345886498611;
busted.test_Q_component_3[53]
[56]:=busted.test.theta_CG*busted.test.omega3*t*0.1886019614740855;
busted.test_Q_component_3[53]
[60]:=busted.test.theta_CT*busted.test.omega3*t*0.2208116300950855;
busted.test_Q_component_3[54]
[9]:=busted.test.theta_AT*busted.test.omega3*t*0.3102791451089904;
busted.test_Q_component_3[54]
[25]:=busted.test.theta_CT*busted.test.omega3*t*0.1512809665850036;
busted.test_Q_component_3[54]
[41]:=busted.test.theta_GT*busted.test.omega3*t*0.2936069988308121;
busted.test_Q_component_3[54]
[48]:=busted.test.theta_AG*busted.test.omega3*t*0.3408876964850085;
busted.test_Q_component_3[54]
[51]:=busted.test.theta_CG*busted.test.omega3*t*0.2496987119458206;
busted.test_Q_component_3[54]
[55]:=busted.test.theta_CG*busted.test.omega3*t*0.1063345886498611;
busted.test_Q_component_3[54]
[56]:=busted.test.theta_CT*t*0.419803077292116;
busted.test_Q_component_3[54]
[58]:=busted.test.theta_GT*busted.test.omega3*t*0.2208116300950855;
busted.test_Q_component_3[55]
[10]:=busted.test.theta_AT*busted.test.omega3*t*0.3102791451089904;
busted.test_Q_component_3[55]
[26]:=busted.test.theta_CT*busted.test.omega3*t*0.1512809665850036;
busted.test_Q_component_3[55]
[42]:=busted.test.theta_GT*busted.test.omega3*t*0.2936069988308121;
busted.test_Q_component_3[55]
[52]:=busted.test.theta_CG*busted.test.omega3*t*0.2496987119458206;
busted.test_Q_component_3[55]
[54]:=busted.test.theta_CG*busted.test.omega3*t*0.0447809360409677;
busted.test_Q_component_3[55]
[56]:=busted.test.theta_GT*busted.test.omega3*t*0.419803077292116;
busted.test_Q_component_3[55]
[59]:=busted.test.theta_GT*busted.test.omega3*t*0.2208116300950855;
busted.test_Q_component_3[56]
[11]:=busted.test.theta_AT*busted.test.omega3*t*0.3102791451089904;
```

```
busted.test_Q_component_3[56]
[27]:=busted.test.theta_CT*busted.test.omega3*t*0.1512809665850036;
busted.test_Q_component_3[56]
[43]:=busted.test.theta_GT*busted.test.omega3*t*0.2936069988308121;
busted.test_Q_component_3[56]
[49]:=busted.test.theta_AG*busted.test.omega3*t*0.3408876964850085;
busted.test_Q_component_3[56]
[53]:=busted.test.theta_CG*busted.test.omega3*t*0.2496987119458206;
busted.test_Q_component_3[56]
[54]:=busted.test.theta_CT*t*0.0447809360409677;
busted.test_Q_component_3[56]
[55]:=busted.test.theta_GT*busted.test.omega3*t*0.1063345886498611;
busted.test_Q_component_3[56]
[60]:=busted.test.theta_GT*busted.test.omega3*t*0.2208116300950855;
busted.test_Q_component_3[57]
[12]:=busted.test.theta_AT*busted.test.omega3*t*0.3102791451089904;
busted.test_Q_component_3[57]
[28]:=busted.test.theta_CT*t*0.1512809665850036;
busted.test_Q_component_3[57]
[44]:=busted.test.theta_GT*busted.test.omega3*t*0.2936069988308121;
busted.test_Q_component_3[57]
[50]:=busted.test.theta_CT*busted.test.omega3*t*0.2496987119458206;
busted.test_Q_component_3[57]
[58]:=busted.test.theta_AC*busted.test.omega3*t*0.0447809360409677;
busted.test_Q_component_3[57]
[59]:=busted.test.theta_AG*t*0.1063345886498611;
busted.test_Q_component_3[57]
[60]:=busted.test.theta_AT*busted.test.omega3*t*0.419803077292116;
busted.test_Q_component_3[58]
[13]:=busted.test.theta_AT*busted.test.omega3*t*0.3102791451089904;
busted.test_Q_component_3[58]
[29]:=busted.test.theta_CT*busted.test.omega3*t*0.1512809665850036;
busted.test_Q_component_3[58]
[45]:=busted.test.theta_GT*busted.test.omega3*t*0.2936069988308121;
busted.test_Q_component_3[58]
[48]:=busted.test.theta_AT*busted.test.omega3*t*0.3408876964850085;
busted.test_Q_component_3[58]
[51]:=busted.test.theta_CT*busted.test.omega3*t*0.2496987119458206;
busted.test_Q_component_3[58]
[54]:=busted.test.theta_GT*busted.test.omega3*t*0.1886019614740855;
busted.test_Q_component_3[58]
[57]:=busted.test.theta_AC*busted.test.omega3*t*0.4290813980170551;
busted.test_Q_component_3[58]
[59]:=busted.test.theta_CG*busted.test.omega3*t*0.1063345886498611;
busted.test_Q_component_3[58]
[60]:=busted.test.theta_CT*t*0.419803077292116;
busted.test_Q_component_3[59]
[14]:=busted.test.theta_AT*busted.test.omega3*t*0.3102791451089904;
busted.test_Q_component_3[59]
[30]:=busted.test.theta_CT*t*0.1512809665850036;
busted.test_Q_component_3[59]
[46]:=busted.test.theta_GT*busted.test.omega3*t*0.2936069988308121;
busted.test_Q_component_3[59]
[52]:=busted.test.theta_CT*busted.test.omega3*t*0.2496987119458206;
```

```
busted.test_Q_component_3[59]
[55]:=busted.test.theta_GT*busted.test.omega3*t*0.1886019614740855;
busted.test_Q_component_3[59]
[57]:=busted.test.theta_AG*t*0.4290813980170551;
busted.test_Q_component_3[59]
[58]:=busted.test.theta_CG*busted.test.omega3*t*0.0447809360409677;
busted.test_Q_component_3[59]
[60]:=busted.test.theta_GT*busted.test.omega3*t*0.419803077292116;
busted.test_Q_component_3[60]
[15]:=busted.test.theta_AT*busted.test.omega3*t*0.3102791451089904;
busted.test_Q_component_3[60]
[31]:=busted.test.theta_CT*busted.test.omega3*t*0.1512809665850036;
busted.test_Q_component_3[60]
[47]:=busted.test.theta_GT*busted.test.omega3*t*0.2936069988308121;
busted.test_Q_component_3[60]
[49]:=busted.test.theta_AT*busted.test.omega3*t*0.3408876964850085;
busted.test_Q_component_3[60]
[53]:=busted.test.theta_CT*busted.test.omega3*t*0.2496987119458206;
busted.test_Q_component_3[60]
[56]:=busted.test.theta_GT*busted.test.omega3*t*0.1886019614740855;
busted.test_Q_component_3[60]
[57]:=busted.test.theta_AT*busted.test.omega3*t*0.4290813980170551;
busted.test_Q_component_3[60]
[58]:=busted.test.theta_CT*t*0.0447809360409677;
busted.test_Q_component_3[60]
[59]:=busted.test.theta_GT*busted.test.omega3*t*0.1063345886498611;


busted.test_pi={
{0.04851315318630076}
{0.005063058944109833}
{0.0120224885348396}
{0.04746412007342106}
{0.03553566757602913}
{0.003708668015554472}
{0.008806419042069661}
{0.03476725551605885}
{0.02684073359811472}
{0.002801224150253312}
{0.00665164786776096}
{0.02626033804620581}
{0.03142462619383732}
{0.003279620561974613}
{0.007787624248545315}
{0.03074510999519717}
{0.02365327100386314}
{0.002468565686787404}
{0.005861733587247011}
{0.02314180013707198}
{0.01732591514410903}
{0.001808213316879193}
{0.00429369361698128}
{0.01695126502340225}
{0.01308657764010232}
```

```
{0.001365776281621247}
{0.003243104587208041}
{0.01280359761557127}
{0.01532151902605711}
{0.001599025188990042}
{0.003796965868611359}
{0.01499021133438374}
{0.04590640890752055}
{0.004791006952663272}
{0.01137648737543148}
{0.04491374321018998}
{0.03362623905896859}
{0.003509391149457637}
{0.00833322608414545}
{0.03289911587860135}
{0.02539850764185784}
{0.002650706722552576}
{0.00629423665276 2}
{0.02484929832883466}
{0.02973609516331416}
{0.003103397587894434}
{0.007369173918647609}
{0.02909309122674739}
{0.003995123005879771}
{0.03745265464068252}
{0.02804023508899723}
{0.002926409720619427}
{0.0069489073113242}
{0.02743390189543365}
{0.00221037028619279}
{0.005248635600898597}
{0.02072132318203182}
{0.02479632341713619}
{0.002587859968121455}
{0.006145003868206126}
{0.02426013554572825}
};
Model
busted.test=("Exp(busted.test_Q_component_1)*busted.test.bsrel_mixtu
re_aux_0+Exp(busted.test_Q_component_2)*(1-
busted.test.bsrel_mixture_aux_0)*busted.test.bsrel_mixture_aux_1+Exp
(busted.test_Q_component_3)*(1-busted.test.bsrel_mixture_aux_0)*(1-
busted.test.bsrel_mixture_aux_1)",busted.test_pi,EXPLICIT_FORM_MATRI
X_EXPONENTIAL);
TRY_NUMERIC_SEQUENCE_MATCH=0;
ACCEPT_ROOTED_TREES=0;

UseModel (busted.test);
Tree
RkVCgCgf.tree_id_0=(((((((((((((((((((((((((((((((((((((((((((((((((
(((((ITA_FJ005218_2c_330_2006,ITA_FJ005233_40_2007)Node53,POR_KT2752
53_2c_PT036_12_2012)Node52,
(URU_KC196096_2c_M247_2010,URU_KM457121_2c_UY247_2010)Node57)Node51,
(URU_KC196086_2c_M55_2006,URU_KM457106_2c_UY55_2006)Node60)Node50,US
```

A_JX475260_CO_704_2010)Node49,ITA_FJ005247_195_2008)Node48,ITA_FJ005
226_383_2006)Node47,GER_FJ005196_2c_G7_1997)Node46,FRA_DQ025994_04S2
5_2004)Node45,FRA_DQ025960_03C4_2003)Node44,FRA_DQ025951_03B10_2003)
Node43,FRA_DQ025954_03B14_2003)Node42,USA_KJ813848_Bobcat_ND_1162_20
13)Node41,URU_KM457104_2c_UY47_2006)Node40,FRA_DQ025969_03S5_2003)No
de39,USA_KJ813858_Puma_ND_F93_2013)Node38,ITA_FJ005248_219_2008)Node
37,GER_FJ005199_2c_G172_1997)Node36,ITA_FJ005240_208_2007)Node35,URU
_KC196085_2c_M57_2007)Node34,FRA_DQ025975_04S6_2004)Node33,USA_JX475
243_ID_22772_2009)Node32,USA_JX475252_CO_1316_2010)Node31,FRA_DQ0259
65_03C9_2003)Node30,ECU_KF149984_2c_ME28_2012)Node29,
(ECU_KF149962_2c_ME1_2012,(ECU_KF149963_2c_ME10_2012,
(ECU_KF149964_2c_ME23_2012,ECU_KF149969_2c_ME31_2012)Node88)Node86)N
ode84)Node28,(ARG_JF414820_Arg44_2009,(ARG_KM236569_Cuba_2013,
(ARG_JF414818_Arg32_2008,ARG_JF414821_Arg48_2009)Node95)Node93)Node9
1)Node27,(ITA_FJ005216_2c_284_2006,
(ITA_KU508407_2c_25835_09_2009,ITA_KX434459_27692_1_11_2011)Node100)
Node98)Node26,
(((URU_KM457122_2c_UY258_2010,URU_KM457124_2c_UY307_2011)Node105,URU
_KC196093_2c_M307_2011)Node104,
((URU_KC196081_2c_M95_2007,URU_KM457109_2c_UY95_2007)Node110,
(URU_KC196097_2c_M242_2010,
(URU_KM457120_2c_UY242_2010,URU_KM457123_2c_UY261_2008)Node115)Node1
13)Node109)Node103)Node25,(URU_KM457103_2c_UY12_2006,
(ITA_FJ005209_2c_303_2004,ITA_FJ005251_239_2008)Node120)Node118)Node
24,
(URU_KC196083_2c_M82_2007,URU_KM457108_2c_UY82_2007)Node123)Node23,U
RU_KC196107_2c_M129_2008)Node22,URU_KM457131_2c_UY368_2011)Node21,PO
R_KT275252_2c_PT013_12_2012)Node20,ITA_FJ005231_406_2006)Node19,
(ITA_FJ005214_2c_67_2006,BRA_KY073269_UFMT_2015)Node130)Node18,ARG_J
F414819_Arg35_2008)Node17,USA_KJ813854_Puma_ND_F205_2013)Node16,ITA_
FJ005232_411_2006)Node15,
(((URU_KC196105_2c_M152_2008,URU_KM457113_2c_UY152_2009)Node138,ITA_
FJ005212_2c_349_2004)Node137,(POR_KT275255_2c_PT238_14_2014,
(ITA_KX434460_52238_12_2012,
((URU_KC196091_2c_M326_2011,URU_KM457127_2c_UY326_2011)Node147,
(((((((((URU_KC196102_2c_M185_2009,URU_KM457116_2c_UY185_2009)Node1
59,
(HRV_KP859577_2c_HR856_2014,ITA_KX434458_2323_11_2011)Node162)Node15
8,
(URU_KC196101_2c_M187_2009,URU_KM457117_2c_UY187_2009)Node165)Node15
7,AUS_KU508693_2c_LW_2015)Node156,
(URU_KC196089_2c_M349_2011,URU_KM457129_2c_UY349_2011)Node169)Node15
5,ITA_FJ005195_2c_136_2000)Node154,FRA_DQ025976_04S7_2004)Node153,
(USA_JX475273_MT_909_2012,USA_KJ813888_Coyote_MT_878_2012)Node174)No
de152,USA_KJ813843_Bobcat_ND_1160_2013)Node151,
(FRA_DQ025942_01B1_2001,(FRA_DQ025964_03C8_2003,
(ITA_FJ005206_2c_287_2004,(URU_KM457107_2c_UY72_2007,
(URU_KM457111_2c_UY120_2008,(URU_KM457112_2c_UY135_2008,
(URU_KM457125_2c_UY317_2011,(URU_KM457130_2c_UY354_2011,
(URU_KM457142_2c_UY370_2011,(HRV_KP859574_2c_HR442_2014,
(HRV_KP859575_2c_HR774_2014,(HRV_KP859576_2c_HR793_2014,
(AUS_KU508691_2c_HB_2015,
(AUS_KU508692_2c_FH_2015,ITA_KX434456_45361_09_2009)Node204)Node202)
Node200)Node198)Node196)Node194)Node192)Node190)Node188)Node186)Node

184)Node182)Node180)Node178)Node150)Node146)Node144)Node142)Node136)
Node14,URU_KM457126_2c_UY318_2010)Node13,(FRA_DQ025985_04S16_2004,
(ITA_FJ005205_2c_279_2004,HRV_KP859578_2c_HR859_2014)Node210)Node208
)Node12,ITA_FJ222821_2c_56_2000)Node11,
(GER_FJ005260_G82_1997,USA_KJ813846_Bobcat_ND_974_2013)Node214)Node1
0,((GER_AY742934_447_1995,RUS_JN033694_Laika_1993)Node218,
(USA_AY742936_395_1998,(USA_JX475240_AZ_16382_01_1999,
(USA_JX475250_CO_728_2010,USA_KJ813842_Bobcat_ND_502_2013)Node225)No
de223)Node221)Node217)Node9,
((((((((((((((((USA_KJ813828_Fisher_F1F010712_2013,USA_KJ813881_Gr
aywolf_MI_832_2012)Node245,USA_KJ813844_Bobcat_ND_885_2013)Node244,U
SA_KJ813882_Raccoon_NJ_1423_2012)Node243,USA_KJ813851_Bobcat_ND_1168
_2013)Node242,USA_JX475278_AR_1069_2012)Node241,KOR_EU009205_2b_K029
_2006)Node240,USA_JX475247_CO_1246_2010)Node239,
(USA_KJ813892_Coyote_AK_218_2013,
(USA_JN867604_Dog_IL_137654_2008,USA_JX475242_WI_18268_2002)Node256)
Node254)Node238,SAF_HQ602969_22_10SA_2010)Node237,
(USA_AY742955_436_2003,FRA_DQ025991_2b_04S22_2004)Node260)Node236,US
A_JN867602_2b_Dog_CA_148743_2008)Node235,USA_JX475251_CO_2235_2009)N
ode234,USA_JN867603_2b_Dog_KS_81213_2009)Node233,USA_KJ813852_Bobcat
_ND_1170_2013)Node232,FRA_DQ025961_2b_03C5_2003)Node231,
(USA_KJ813827_Fisher_F1M111211_2013,USA_KJ813873_Graywolf_MI_850_201
2)Node268)Node230,
(ECU_KF149971_2c_ME32_2012,IND_KX469432_newCPV_2b_Hiller_2011)Node27
1)Node229,((ITA_FJ005263_42_2005,ITA_FJ005265_140_2005)Node275,
((((((((USA_M74849_39_1995,USA_U22896_cat_1990)Node287,FRA_DQ02599
2_2b_04S23_2004)Node286,USA_M74852_133_1995)Node285,POR_KU662349_gre
ywolf_W33_1996)Node284,
(POR_KU662350_greywolf_W52_2005,POL_Z46651_46_1994)Node293)Node283,G
ER_FJ005261_G162_1997)Node282,BRA_DQ340409_2b_BR183_1985)Node281,USA
_AY742932_193_1991)Node280,
((USA_AY742951_431_2003,USA_JN867605_2b_Dog_US_142805_2009)Node300,
(VAC_FJ222822_2b_FortDodge_2008,(VAC_JN625223_INDIA_vac5_2011,
(USA_EU659119_2b_CPV_410_2000,USA_EU659120_2b_CPV_411a_1998)Node307)
Node305)Node303)Node299)Node279,
((CHI_GQ857609_CPV08_01_2008,CHI_GU569940_2b_YN0203_2002)Node311,
((((((JPN_AB115504_2c_97_008_1997,TAW_U72696_2b_T10_1996)Node320,TA
W_U72695_2a_T4_1996)Node319,
(CHI_GQ857596_CPV05_01_2005,CHI_GQ857600_CPV06_01_2006)Node324)Node3
18,THA_FJ869125_KU5_2004)Node317,
(USA_JX475237_CT_372_2011,KOR_EF599097_2b_DH326_2006)Node328)Node316
,(CHI_EU483515_2b_ZD13_2007,(JPN_LC270891_2b_9985_2017,
(JPN_AB437433_1887_M_2_2008,(TAW_FJ265781_CPV307_2005,
(TWN_EF592511_TWN1_2006,TAW_FJ265775_CPV301_2004)Node339)Node337)Nod
e335)Node333)Node331)Node315,
(((VIE_AB054218_2b_cat_V123_2000,VAC_FJ222823_2b_29_1997)Node344,ITA
_FJ005264_134_2005)Node343,
((THA_FJ869122_KU1_2008,THA_FJ869123_KU3_2008)Node349,
(((((THA_KP715690_VT28_2014,THA_KP715716_VT143_2014)Node357,THA_KP7
15691_VT43_2014)Node356,
(VIE_AB120722_2b_HCM_18_2003,VIE_AB120724_2b_HNI_2_13_2003)Node361)N
ode355,
(CHI_GQ857599_CPV05_04_2005,CHI_GQ857601_CPV06_02_2006)Node364)Node3
54,CHI_GQ857605_CPV07_03_2007)Node353,(THA_FJ869139_KU66_2003,

(VIE_AB120721_2b_HCM_8_2003,(VIE_AB054221_2b_leopard_V204_2000,
(VIE_AB054224_2c_leopard_V203_2000,(VIE_AB120725_2b_HNI_3_4_2003,
(VIE_AB120723_2b_HCM_23_2003,(VIE_AB120720_2b_HCM_6_2003,
(VIE_AB054219_2b_cat_V209_2000,
(VIE_AB054220_2b_cat_V217_2000,CHI_EU145954_2b_BJ044_2007)Node384)No
de382)Node380)Node378)Node376)Node374)Node372)Node370)Node368)Node35
2)Node348)Node342)Node314)Node310)Node278)Node274)Node228)Node8,
(ITA_FJ005257_54_2008,ITA_KF373611_2a_409_2010)Node387)Node7,
(NZE_AY742933_339_1993,
((VIE_AB054223_2c_leopard_V140_2000,ITA_GU362932_cat11_2008)Node393,
(NIG_HQ602995_15_10_2010,
(((((((((FRA_DQ025947_2a_02B5_2002,FRA_DQ026001_2a_04S32_2004)Node4
07,FRA_DQ025962_2a_03C6_2003)Node406,ITA_KF373580_2a_581_2003)Node40
5,
(GER_AY742935_U6_1995,FRA_DQ025945_2a_02B3_2002)Node412)Node404,VIE_
AB054215_2a_cat_V120_2000)Node403,ITA_FJ005255_333_2005)Node402,FRA_
DQ025958_2a_03C2_2003)Node401,(ITA_KX434457_987_10_2010,
(FRA_DQ025983_2a_04S14_2004,FRA_DQ025993_2a_04S24_2004)Node420)Node4
18)Node400,
(FRA_DQ025984_2a_04S15_2004,ITA_FJ005252_96_2002)Node423)Node399,
(FRA_DQ026002_2a_04S33_2004,(ITA_KF373592_2a_329_2008,
(((ITA_AF393506_2a_699_2000,FRA_DQ025943_2a_01S1_2001)Node432,ITA_KF
385388_2a_Sicily_X83090_2009)Node431,
((CHI_GQ857612_CPV08_04_2008,CHI_GU569939_2a_YN0202_2002)Node437,
(((((HUN_KF539794_H_7_2012,HUN_KF539795_H_8_2012)Node444,HUN_KF53980
4_H_212_2012)Node443,
(HUN_KF539793_H_5_2012,HUN_KF539797_H_11_2012)Node448)Node442,
(HUN_KF539800_H_27_2012,
(VIE_AB054217_2a_cat_V154_2000,HUN_KF539796_H_9_2012)Node453)Node451
)Node441,
(((HUN_KF539798_H_31_2012,HUN_KF539799_H_39_2012)Node458,HUN_KF53980
5_H_36_2012)Node457,(ITA_AF306447_618_2000,
(FRA_DQ025944_2a_02B2_2002,(NIG_HQ602992_19_10_2010,
(ITA_AF306446_584_2000,(FRA_DQ025986_2a_04S17_2004,
(ITA_KF373577_2a_714_2001,
(FRA_DQ025982_2a_04S13_2004,ITA_FJ005253_67_2005)Node474)Node472)Nod
e470)Node468)Node466)Node464)Node462)Node456)Node440)Node436)Node430
)Node428)Node426)Node398)Node396)Node392)Node390)Node6,
((((((((THA_FJ869126_KU5_2008,THA_FJ869137_KU52_2003)Node484,THA_FJ8
69134_KU23_2003)Node483,CHI_DQ354068_2a_redpanda_RPPV_2004)Node482,K
OR_EF599096_DH426_2005)Node481,(ITA_FJ005258_80_2008,
(KOR_EF599098_2c_Pome_2006,
(FRA_DQ025950_2a_02B9_2002,ITA_KX434454_29451_09_2009)Node494)Node49
2)Node490)Node480,(THA_FJ869130_KU13_2004,
(THA_FJ869138_KU53_2003,CHI_KF803615_2011_BJ_B25_2011)Node499)Node49
7)Node479,
(CHI_GU569942_2a_JL0202_2002,CHI_GU569946_2a_JL0201_2002)Node502)Nod
e478,((USA_AY742953_435_2003,ITA_KF373571_2a_685_1999)Node506,
(THA_FJ869128_KU11_2004,
(((BRA_DQ340428_2a_BR209_1994,BRA_DQ340431_2a_BR56_1995)Node513,BRA_
DQ340411_2a_BR8_1990)Node512,(BRA_DQ340422_2a_BR22_1993,
(BRA_DQ340421_2a_BR597_1992,
((((BRA_DQ340419_2a_BR570_1992,BRA_DQ340423_2a_BR136_1993)Node524,BR
A_DQ340413_2a_BR18_1990)Node523,BRA_DQ340427_2a_BR133_1994)Node522,

(BRA_DQ340414_2a_BR31_1990,(BRA_DQ340416_2a_BR47_1991,
(BRA_DQ340417_2a_BR52_1991,(BRA_DQ340418_2a_BR491_1992,
(BRA_DQ340424_2a_BR137_1993,BRA_DQ340426_2a_BR84_1994)Node537)Node53
5)Node533)Node531)Node529)Node521)Node519)Node517)Node511)Node509)No
de505)Node477)Node5,CHI_KF803600_2010_BJ_A68_2010)Node4,
(((USA_EU659118_CPV_13_1981,CHI_GU569948_2a_CC8601_1986)Node543,JPN_
D26079_1993)Node542,
((BRA_DQ340407_2a_BR145_1980,BRA_DQ340408_2a_BR154_1980)Node548,
(FRA_DQ025952_2a_03B12_2003,(BRA_DQ340404_2a_BR6_1980,
(BRA_DQ340405_2a_BR135_1980,(BRA_DQ340410_2a_BR315_1986,
(USA_M24000_FPV_CPV_31_1988,USA_M24003_FPV_CPV_15_1988)Node559)Node5
57)Node555)Node553)Node551)Node547)Node541)Node3,
((USA_JN867599_Raccoon_KY_39552_2009,USA_JN867611_Raccoon_KY_358_B_2
009)Node563,(USA_JN867610_Raccoon_VA_118_A_2007,
(USA_KJ813890_Redfox_MA_197_2012,(USA_JX475284_TN_26_2011,
(USA_JX475239_GA_06_2011,USA_JX475279_TN_1_2011)Node572)Node570)Node
568)Node566)Node562)Node2,
(HUN_KF539801_H_25_2012,HUN_KF539803_H_2_2012)Node575)Node1,
(USA_KJ813870_Raccoon_TX_1_2013,
(((USA_JN867598_Bobcat_KS_44_2010,USA_KJ813832_Fisher_ND_14_2013)Nod
e582,
(USA_KJ813831_Fisher_ND_17_2013,USA_KJ813835_Fisher_ND_19_2013)Node5
85)Node581,(USA_JX475234_ME_258_2011,
(USA_JN867618_Raccoon_WI_37_2010,(USA_JX475231_CO_280_2011,
(USA_JX475248_CO_1102_2011,
(USA_JX475233_SC_182_A_2011,USA_JX475246_CO_2503_2010)Node596)Node59
4)Node592)Node590)Node588)Node580)Node578,
((CHI_FJ231389_FPV_monkey_BJ_22_2008,CHI_KJ170680_raccoondog_HLJ11_1
_2011)Node600,
((((((((CHI_GU392242_raccoondog_HB10_2009,CHI_GU392244_raccoondog_H
B7_2009)Node611,CHI_KJ170679_raccoondog_Heb10_2_2010)Node610,CHI_GU3
92241_raccoondog_HB1_2009)Node609,CHI_GU392236_fox_HB1_2009)Node608,
(CHI_GU392240_raccoondog_HB3_2009,
(CHI_GU392239_raccoondog_HB6_2009,CHI_KJ194463_raccoondog_HeB10_3_20
10)Node619)Node617)Node607,CHI_GU392237_fox_HB2_2009)Node606,
(VAC_FJ011098_Intervet_2006,(VAC_JN625222_INDIA_vac4_2011,
(ITA_FJ222824_388_05_3_2005,(CHI_FJ432718_CPV_Cv_2008,
(VAC_JN625219_INDIA_vac1_2011,CHI_KF803602_2010_BJ_A72_2010)Node631)
Node629)Node627)Node625)Node623)Node605,JPN_AB437434_1887_f_3_2008)N
ode604,
((((((VAC_GU212790_primodog_2009,VAC_GU212791_vanguard_2009)Node640,
VAC_FJ197847_Pfizer_2007)Node639,VAC_EU914139_Pfizer_2006)Node638,VA
C_KY083089_Singapore_2016)Node637,USA_M19296_CPV_N_1988)Node636,
(((((USA_M23255_FPV_Cornell320_1988,USA_M38245_1990)Node651,USA_EU65
9116_CPV_5_1979)Node650,
(FIN_U22192_raccoondog_RD_80_1980,FIN_U22193_raccoondog_RD87_1987)No
de655)Node649,
(USA_M10989_1985,USA_U22186_CPV_128_1995)Node658)Node648,
(VAC_JN625221_INDIA_vac3_2011,(VAC_JN625220_INDIA_vac2_2011,
(((VAC_FJ011097_Merial_2006,CHI_GQ169553_Vac2_2007)Node667,VAC_KY083
090_Singapore_2016)Node666,(CHI_GU569943_YB8301_1983,
(VAC_JN625224_INDIA_vac6_2011,ARG_KM236572_NNGag_2012)Node673)Node67
1)Node665)Node663)Node661)Node647)Node635)Node603)Node599);

RkVCgCgf.tree_id_0.ITA_FJ005218_2c_330_2006.t=0.008522004575430796;
RkVCgCgf.tree_id_0.ITA_FJ005233_40_2007.t=0;
RkVCgCgf.tree_id_0.Node53.t=0.008520909738546798;
RkVCgCgf.tree_id_0.POR_KT275253_2c_PT036_12_2012.t=0.017799633707015
53;
RkVCgCgf.tree_id_0.Node52.t=0;
RkVCgCgf.tree_id_0.URU_KC196096_2c_M247_2010.t=0;
RkVCgCgf.tree_id_0.URU_KM457121_2c_UY247_2010.t=0;
RkVCgCgf.tree_id_0.Node57.t=0.008521413852143505;
RkVCgCgf.tree_id_0.Node51.t=0;
RkVCgCgf.tree_id_0.URU_KC196086_2c_M55_2006.t=0;
RkVCgCgf.tree_id_0.URU_KM457106_2c_UY55_2006.t=0;
RkVCgCgf.tree_id_0.Node60.t=0.01623128966839967;
RkVCgCgf.tree_id_0.Node50.t=0;
RkVCgCgf.tree_id_0.USA_JX475260_CO_704_2010.t=0.008523961554794388;
RkVCgCgf.tree_id_0.Node49.t=0;
RkVCgCgf.tree_id_0.ITA_FJ005247_195_2008.t=0.008523928761488284;
RkVCgCgf.tree_id_0.Node48.t=0;
RkVCgCgf.tree_id_0.ITA_FJ005226_383_2006.t=0.0078552801167865;
RkVCgCgf.tree_id_0.Node47.t=0;
RkVCgCgf.tree_id_0.GER_FJ005196_2c_G7_1997.t=0.008521610359879818;
RkVCgCgf.tree_id_0.Node46.t=0;
RkVCgCgf.tree_id_0.FRA_DQ025994_04S25_2004.t=0.008521057894952996;
RkVCgCgf.tree_id_0.Node45.t=0;
RkVCgCgf.tree_id_0.FRA_DQ025960_03C4_2003.t=0.008521413880864654;
RkVCgCgf.tree_id_0.Node44.t=0;
RkVCgCgf.tree_id_0.FRA_DQ025951_03B10_2003.t=0.008523928720876187;
RkVCgCgf.tree_id_0.Node43.t=0;
RkVCgCgf.tree_id_0.FRA_DQ025954_03B14_2003.t=0.007822273191514618;
RkVCgCgf.tree_id_0.Node42.t=0;
RkVCgCgf.tree_id_0.USA_KJ813848_Bobcat_ND_1162_2013.t=0.007802397399
179064;
RkVCgCgf.tree_id_0.Node41.t=0;
RkVCgCgf.tree_id_0.URU_KM457104_2c_UY47_2006.t=0.008009150426893217;
RkVCgCgf.tree_id_0.Node40.t=0;
RkVCgCgf.tree_id_0.FRA_DQ025969_03S5_2003.t=0.008545505312771403;
RkVCgCgf.tree_id_0.Node39.t=0;
RkVCgCgf.tree_id_0.USA_KJ813858_Puma_ND_F93_2013.t=0.008522496206135
332;
RkVCgCgf.tree_id_0.Node38.t=0;
RkVCgCgf.tree_id_0.ITA_FJ005248_219_2008.t=0.007833327655959135;
RkVCgCgf.tree_id_0.Node37.t=0;
RkVCgCgf.tree_id_0.GER_FJ005199_2c_G172_1997.t=0.008523331159429343;
RkVCgCgf.tree_id_0.Node36.t=0;
RkVCgCgf.tree_id_0.ITA_FJ005240_208_2007.t=0.007802397393924357;
RkVCgCgf.tree_id_0.Node35.t=0;
RkVCgCgf.tree_id_0.URU_KC196085_2c_M57_2007.t=0.008547262448657165;
RkVCgCgf.tree_id_0.Node34.t=0;
RkVCgCgf.tree_id_0.FRA_DQ025975_04S6_2004.t=0.008548541976782736;
RkVCgCgf.tree_id_0.Node33.t=0;
RkVCgCgf.tree_id_0.USA_JX475243_ID_22772_2009.t=0.008523961567131809
;
RkVCgCgf.tree_id_0.Node32.t=0;
RkVCgCgf.tree_id_0.USA_JX475252_CO_1316_2010.t=0.008522496162600625;

```
RkVCgCgf.tree_id_0.Node31.t=0;
RkVCgCgf.tree_id_0.FRA_DQ025965_03C9_2003.t=0.008547456870230227;
RkVCgCgf.tree_id_0.Node30.t=0;
RkVCgCgf.tree_id_0.ECU_KF149984_2c_ME28_2012.t=0.008548542021241315;
RkVCgCgf.tree_id_0.Node29.t=0;
RkVCgCgf.tree_id_0.ECU_KF149962_2c_ME1_2012.t=0;
RkVCgCgf.tree_id_0.ECU_KF149963_2c_ME10_2012.t=0.008493926458210623;
RkVCgCgf.tree_id_0.ECU_KF149964_2c_ME23_2012.t=0.008495403884833349;
RkVCgCgf.tree_id_0.ECU_KF149969_2c_ME31_2012.t=0.008495357560807604;
RkVCgCgf.tree_id_0.Node88.t=0;
RkVCgCgf.tree_id_0.Node86.t=0;
RkVCgCgf.tree_id_0.Node84.t=0.008521413877477216;
RkVCgCgf.tree_id_0.Node28.t=0;
RkVCgCgf.tree_id_0.ARG_JF414820_Arg44_2009.t=0;
RkVCgCgf.tree_id_0.ARG_KM236569_Cuba_2013.t=0.008496914608188424;
RkVCgCgf.tree_id_0.ARG_JF414818_Arg32_2008.t=0.008495486792921121;
RkVCgCgf.tree_id_0.ARG_JF414821_Arg48_2009.t=0.00781454571158968;
RkVCgCgf.tree_id_0.Node95.t=0;
RkVCgCgf.tree_id_0.Node93.t=0;
RkVCgCgf.tree_id_0.Node91.t=0.008521413870322157;
RkVCgCgf.tree_id_0.Node27.t=0;
RkVCgCgf.tree_id_0.ITA_FJ005216_2c_284_2006.t=0.008521310458068065;
RkVCgCgf.tree_id_0.ITA_KU508407_2c_25835_09_2009.t=0;
RkVCgCgf.tree_id_0.ITA_KX434459_27692_1_11_2011.t=0.0085118996220365
85;
RkVCgCgf.tree_id_0.Node100.t=0.008531157746805899;
RkVCgCgf.tree_id_0.Node98.t=0;
RkVCgCgf.tree_id_0.Node26.t=0;
RkVCgCgf.tree_id_0.URU_KM457122_2c_UY258_2010.t=0;
RkVCgCgf.tree_id_0.URU_KM457124_2c_UY307_2011.t=0;
RkVCgCgf.tree_id_0.Node105.t=0;
RkVCgCgf.tree_id_0.URU_KC196093_2c_M307_2011.t=0;
RkVCgCgf.tree_id_0.Node104.t=0.008547456873762816;
RkVCgCgf.tree_id_0.URU_KC196081_2c_M95_2007.t=0;
RkVCgCgf.tree_id_0.URU_KM457109_2c_UY95_2007.t=0;
RkVCgCgf.tree_id_0.Node110.t=0.007728294421387746;
RkVCgCgf.tree_id_0.URU_KC196097_2c_M242_2010.t=0;
RkVCgCgf.tree_id_0.URU_KM457120_2c_UY242_2010.t=0;
RkVCgCgf.tree_id_0.URU_KM457123_2c_UY261_2008.t=0;
RkVCgCgf.tree_id_0.Node115.t=0;
RkVCgCgf.tree_id_0.Node113.t=0.008545594845561097;
RkVCgCgf.tree_id_0.Node109.t=0.007952732124081487;
RkVCgCgf.tree_id_0.Node103.t=0;
RkVCgCgf.tree_id_0.Node25.t=0;
RkVCgCgf.tree_id_0.URU_KM457103_2c_UY12_2006.t=0.01620858447196524;
RkVCgCgf.tree_id_0.ITA_FJ005209_2c_303_2004.t=0;
RkVCgCgf.tree_id_0.ITA_FJ005251_239_2008.t=0.008498475854201638;
RkVCgCgf.tree_id_0.Node120.t=0.008523961567013212;
RkVCgCgf.tree_id_0.Node118.t=0;
RkVCgCgf.tree_id_0.Node24.t=0;
RkVCgCgf.tree_id_0.URU_KC196083_2c_M82_2007.t=0;
RkVCgCgf.tree_id_0.URU_KM457108_2c_UY82_2007.t=0;
RkVCgCgf.tree_id_0.Node123.t=0.01779404700703355;
RkVCgCgf.tree_id_0.Node23.t=0;
```

RkVCgCgf.tree_id_0.URU_KC196107_2c_M129_2008.t=0.01772934759352004;
RkVCgCgf.tree_id_0.Node22.t=0;
RkVCgCgf.tree_id_0.URU_KM457131_2c_UY368_2011.t=0.01773663499191544;
RkVCgCgf.tree_id_0.Node21.t=0;
RkVCgCgf.tree_id_0.POR_KT275252_2c_PT013_12_2012.t=0.01773262123026717;
RkVCgCgf.tree_id_0.Node20.t=0;
RkVCgCgf.tree_id_0.ITA_FJ005231_406_2006.t=0.01772829975954765;
RkVCgCgf.tree_id_0.Node19.t=0;
RkVCgCgf.tree_id_0.ITA_FJ005214_2c_67_2006.t=0.008500740948480089;
RkVCgCgf.tree_id_0.BRA_KY073269_UFMT_2015.t=0.008502332932799998;
RkVCgCgf.tree_id_0.Node130.t=0.008586024353189822;
RkVCgCgf.tree_id_0.Node18.t=0;
RkVCgCgf.tree_id_0.ARG_JF414819_Arg35_2008.t=0.01773141944574295;
RkVCgCgf.tree_id_0.Node17.t=0;
RkVCgCgf.tree_id_0.USA_KJ813854_Puma_ND_F205_2013.t=0.01773286130051387;
RkVCgCgf.tree_id_0.Node16.t=0;
RkVCgCgf.tree_id_0.ITA_FJ005232_411_2006.t=0.01772956445061999;
RkVCgCgf.tree_id_0.Node15.t=0;
RkVCgCgf.tree_id_0.URU_KC196105_2c_M152_2008.t=0;
RkVCgCgf.tree_id_0.URU_KM457113_2c_UY152_2009.t=0;
RkVCgCgf.tree_id_0.Node138.t=0.01569922717331853;
RkVCgCgf.tree_id_0.ITA_FJ005212_2c_349_2004.t=0.008550781436437878;
RkVCgCgf.tree_id_0.Node137.t=0;
RkVCgCgf.tree_id_0.POR_KT275255_2c_PT238_14_2014.t=0.0176921294691045;
RkVCgCgf.tree_id_0.ITA_KX434460_52238_12_2012.t=0.01775501977718361;
RkVCgCgf.tree_id_0.URU_KC196091_2c_M326_2011.t=0;
RkVCgCgf.tree_id_0.URU_KM457127_2c_UY326_2011.t=0;
RkVCgCgf.tree_id_0.Node147.t=0.01593812648653514;
RkVCgCgf.tree_id_0.URU_KC196102_2c_M185_2009.t=0;
RkVCgCgf.tree_id_0.URU_KM457116_2c_UY185_2009.t=0;
RkVCgCgf.tree_id_0.Node159.t=0.008548262559565808;
RkVCgCgf.tree_id_0.HRV_KP859577_2c_HR856_2014.t=0.01543898378343869;
RkVCgCgf.tree_id_0.ITA_KX434458_2323_11_2011.t=0;
RkVCgCgf.tree_id_0.Node162.t=0;
RkVCgCgf.tree_id_0.Node158.t=0;
RkVCgCgf.tree_id_0.URU_KC196101_2c_M187_2009.t=0;
RkVCgCgf.tree_id_0.URU_KM457117_2c_UY187_2009.t=0;
RkVCgCgf.tree_id_0.Node165.t=0.008531777549293299;
RkVCgCgf.tree_id_0.Node157.t=0;
RkVCgCgf.tree_id_0.AUS_KU508693_2c_LW_2015.t=0.007639420840992625;
RkVCgCgf.tree_id_0.Node156.t=0;
RkVCgCgf.tree_id_0.URU_KC196089_2c_M349_2011.t=0;
RkVCgCgf.tree_id_0.URU_KM457129_2c_UY349_2011.t=0;
RkVCgCgf.tree_id_0.Node169.t=0.008505102160486739;
RkVCgCgf.tree_id_0.Node155.t=0;
RkVCgCgf.tree_id_0.ITA_FJ005195_2c_136_2000.t=0.007818528286864695;
RkVCgCgf.tree_id_0.Node154.t=0;
RkVCgCgf.tree_id_0.FRA_DQ025976_04S7_2004.t=0.008545000325267232;
RkVCgCgf.tree_id_0.Node153.t=0;
RkVCgCgf.tree_id_0.USA_JX475273_MT_909_2012.t=0.008545226352285999;
RkVCgCgf.tree_id_0.USA_KJ813888_Coyote_MT_878_2012.t=0;

RkVCgCgf.tree_id_0.Node174.t=0.008438059846068562;
RkVCgCgf.tree_id_0.Node152.t=0;
RkVCgCgf.tree_id_0.USA_KJ813843_Bobcat_ND_1160_2013.t=0.016467894824
41192;
RkVCgCgf.tree_id_0.Node151.t=0;
RkVCgCgf.tree_id_0.FRA_DQ025942_01B1_2001.t=0;
RkVCgCgf.tree_id_0.FRA_DQ025964_03C8_2003.t=0.008549644043655439;
RkVCgCgf.tree_id_0.ITA_FJ005206_2c_287_2004.t=0;
RkVCgCgf.tree_id_0.URU_KM457107_2c_UY72_2007.t=0;
RkVCgCgf.tree_id_0.URU_KM457111_2c_UY120_2008.t=0;
RkVCgCgf.tree_id_0.URU_KM457112_2c_UY135_2008.t=0;
RkVCgCgf.tree_id_0.URU_KM457125_2c_UY317_2011.t=0;
RkVCgCgf.tree_id_0.URU_KM457130_2c_UY354_2011.t=0;
RkVCgCgf.tree_id_0.URU_KM457142_2c_UY370_2011.t=0;
RkVCgCgf.tree_id_0.HRV_KP859574_2c_HR442_2014.t=0;
RkVCgCgf.tree_id_0.HRV_KP859575_2c_HR774_2014.t=0;
RkVCgCgf.tree_id_0.HRV_KP859576_2c_HR793_2014.t=0;
RkVCgCgf.tree_id_0.AUS_KU508691_2c_HB_2015.t=0;
RkVCgCgf.tree_id_0.AUS_KU508692_2c_FH_2015.t=0;
RkVCgCgf.tree_id_0.ITA_KX434456_45361_09_2009.t=0.008549644047113536
;
RkVCgCgf.tree_id_0.Node204.t=0;
RkVCgCgf.tree_id_0.Node202.t=0;
RkVCgCgf.tree_id_0.Node200.t=0;
RkVCgCgf.tree_id_0.Node198.t=0;
RkVCgCgf.tree_id_0.Node196.t=0;
RkVCgCgf.tree_id_0.Node194.t=0;
RkVCgCgf.tree_id_0.Node192.t=0;
RkVCgCgf.tree_id_0.Node190.t=0;
RkVCgCgf.tree_id_0.Node188.t=0;
RkVCgCgf.tree_id_0.Node186.t=0;
RkVCgCgf.tree_id_0.Node184.t=0;
RkVCgCgf.tree_id_0.Node182.t=0;
RkVCgCgf.tree_id_0.Node180.t=0;
RkVCgCgf.tree_id_0.Node178.t=0;
RkVCgCgf.tree_id_0.Node150.t=0;
RkVCgCgf.tree_id_0.Node146.t=0;
RkVCgCgf.tree_id_0.Node144.t=0;
RkVCgCgf.tree_id_0.Node142.t=0;
RkVCgCgf.tree_id_0.Node136.t=0;
RkVCgCgf.tree_id_0.Node14.t=0;
RkVCgCgf.tree_id_0.URU_KM457126_2c_UY318_2010.t=0.01501797992491238;
RkVCgCgf.tree_id_0.Node13.t=0;
RkVCgCgf.tree_id_0.FRA_DQ025985_04S16_2004.t=0.008512255471080088;
RkVCgCgf.tree_id_0.ITA_FJ005205_2c_279_2004.t=0;
RkVCgCgf.tree_id_0.HRV_KP859578_2c_HR859_2014.t=0.008469149252466888
;
RkVCgCgf.tree_id_0.Node210.t=0.008496876892035288;
RkVCgCgf.tree_id_0.Node208.t=0.008571521414287112;
RkVCgCgf.tree_id_0.Node12.t=0.008549644789669802;
RkVCgCgf.tree_id_0.ITA_FJ222821_2c_56_2000.t=0;
RkVCgCgf.tree_id_0.Node11.t=0.007839243553248044;
RkVCgCgf.tree_id_0.GER_FJ005260_G82_1997.t=0.02776535551976233;
RkVCgCgf.tree_id_0.USA_KJ813846_Bobcat_ND_974_2013.t=0.0077723345666

99335;
RkVCgCgf.tree_id_0.Node214.t=0.008572539682642091;
RkVCgCgf.tree_id_0.Node10.t=0;
RkVCgCgf.tree_id_0.GER_AY742934_447_1995.t=0.008508983314023584;
RkVCgCgf.tree_id_0.RUS_JN033694_Laika_1993.t=0.03707362009148909;
RkVCgCgf.tree_id_0.Node218.t=0;
RkVCgCgf.tree_id_0.USA_AY742936_395_1998.t=0;
RkVCgCgf.tree_id_0.USA_JX475240_AZ_16382_01_1999.t=0;
RkVCgCgf.tree_id_0.USA_JX475250_CO_728_2010.t=0.008474701448005845;
RkVCgCgf.tree_id_0.USA_KJ813842_Bobcat_ND_502_2013.t=0.0084771886331
8931;
RkVCgCgf.tree_id_0.Node225.t=0;
RkVCgCgf.tree_id_0.Node223.t=0.01774642540423018;
RkVCgCgf.tree_id_0.Node221.t=0.008532435439697943;
RkVCgCgf.tree_id_0.Node217.t=0.008534010722782013;
RkVCgCgf.tree_id_0.Node9.t=0;
RkVCgCgf.tree_id_0.USA_KJ813828_Fisher_F1F010712_2013.t=0.0177099409
8536554;
RkVCgCgf.tree_id_0.USA_KJ813881_Graywolf_MI_832_2012.t=0.01760459480
934884;
RkVCgCgf.tree_id_0.Node245.t=0;
RkVCgCgf.tree_id_0.USA_KJ813844_Bobcat_ND_885_2013.t=0.0079961195558
94375;
RkVCgCgf.tree_id_0.Node244.t=0;
RkVCgCgf.tree_id_0.USA_KJ813882_Raccoon_NJ_1423_2012.t=0.00846301304
9538408;
RkVCgCgf.tree_id_0.Node243.t=0;
RkVCgCgf.tree_id_0.USA_KJ813851_Bobcat_ND_1168_2013.t=0.008462078561
962384;
RkVCgCgf.tree_id_0.Node242.t=0;
RkVCgCgf.tree_id_0.USA_JX475278_AR_1069_2012.t=0.008463968356501268;
RkVCgCgf.tree_id_0.Node241.t=0;
RkVCgCgf.tree_id_0.KOR_EU009205_2b_K029_2006.t=0.008462272614077213;
RkVCgCgf.tree_id_0.Node240.t=0;
RkVCgCgf.tree_id_0.USA_JX475247_CO_1246_2010.t=0.008463968419954742;
RkVCgCgf.tree_id_0.Node239.t=0;
RkVCgCgf.tree_id_0.USA_KJ813892_Coyote_AK_218_2013.t=0.0085099421718
86373;
RkVCgCgf.tree_id_0.USA_JN867604_Dog_IL_137654_2008.t=0.0085101076282
37009;
RkVCgCgf.tree_id_0.USA_JX475242_WI_18268_2002.t=0.008490477446573795
;
RkVCgCgf.tree_id_0.Node256.t=0;
RkVCgCgf.tree_id_0.Node254.t=0.008494092257245369;
RkVCgCgf.tree_id_0.Node238.t=0;
RkVCgCgf.tree_id_0.SAF_HQ602969_22_10SA_2010.t=0;
RkVCgCgf.tree_id_0.Node237.t=0;
RkVCgCgf.tree_id_0.USA_AY742955_436_2003.t=0;
RkVCgCgf.tree_id_0.FRA_DQ025991_2b_04S22_2004.t=0;
RkVCgCgf.tree_id_0.Node260.t=0;
RkVCgCgf.tree_id_0.Node236.t=0;
RkVCgCgf.tree_id_0.USA_JN867602_2b_Dog_CA_148743_2008.t=0.0084619745
19230782;
RkVCgCgf.tree_id_0.Node235.t=0;

```
RkVCgCgf.tree_id_0.USA_JX475251_CO_2235_2009.t=0.008464590304979723;
RkVCgCgf.tree_id_0.Node234.t=0;
RkVCgCgf.tree_id_0.USA_JN867603_2b_Dog_KS_81213_2009.t=0.00846314598
0110833;
RkVCgCgf.tree_id_0.Node233.t=0;
RkVCgCgf.tree_id_0.USA_KJ813852_Bobcat_ND_1170_2013.t=0.008487748030
310572;
RkVCgCgf.tree_id_0.Node232.t=0;
RkVCgCgf.tree_id_0.FRA_DQ025961_2b_03C5_2003.t=0.0084888169155225;
RkVCgCgf.tree_id_0.Node231.t=0;
RkVCgCgf.tree_id_0.USA_KJ813827_Fisher_F1M111211_2013.t=0;
RkVCgCgf.tree_id_0.USA_KJ813873_Graywolf_MI_850_2012.t=0.00845972967
901314;
RkVCgCgf.tree_id_0.Node268.t=0.008379730953936184;
RkVCgCgf.tree_id_0.Node230.t=0.0177302887842563;
RkVCgCgf.tree_id_0.ECU_KF149971_2c_ME32_2012.t=0.02522723656791472;
RkVCgCgf.tree_id_0.IND_KX469432_newCPV_2b_Hiller_2011.t=0.0276120796
2341367;
RkVCgCgf.tree_id_0.Node271.t=0.007966422093327772;
RkVCgCgf.tree_id_0.Node229.t=0.008536706718222519;
RkVCgCgf.tree_id_0.ITA_FJ005263_42_2005.t=0;
RkVCgCgf.tree_id_0.ITA_FJ005265_140_2005.t=0.01777859892539869;
RkVCgCgf.tree_id_0.Node275.t=0.008103534677276288;
RkVCgCgf.tree_id_0.USA_M74849_39_1995.t=0;
RkVCgCgf.tree_id_0.USA_U22896_cat_1990.t=0.008582720653399779;
RkVCgCgf.tree_id_0.Node287.t=0.008557733344088995;
RkVCgCgf.tree_id_0.FRA_DQ025992_2b_04S23_2004.t=0.0177577057093161;
RkVCgCgf.tree_id_0.Node286.t=0;
RkVCgCgf.tree_id_0.USA_M74852_133_1995.t=0;
RkVCgCgf.tree_id_0.Node285.t=0;
RkVCgCgf.tree_id_0.POR_KU662349_greywolf_W33_1996.t=0;
RkVCgCgf.tree_id_0.Node284.t=0;
RkVCgCgf.tree_id_0.POR_KU662350_greywolf_W52_2005.t=0.02992800561711
69;
RkVCgCgf.tree_id_0.POL_Z46651_46_1994.t=0;
RkVCgCgf.tree_id_0.Node293.t=0;
RkVCgCgf.tree_id_0.Node283.t=0;
RkVCgCgf.tree_id_0.GER_FJ005261_G162_1997.t=0.02788603696981435;
RkVCgCgf.tree_id_0.Node282.t=0;
RkVCgCgf.tree_id_0.BRA_DQ340409_2b_BR183_1985.t=0.008558562161830355
;
RkVCgCgf.tree_id_0.Node281.t=0.008060667269899218;
RkVCgCgf.tree_id_0.USA_AY742932_193_1991.t=0;
RkVCgCgf.tree_id_0.Node280.t=0;
RkVCgCgf.tree_id_0.USA_AY742951_431_2003.t=0;
RkVCgCgf.tree_id_0.USA_JN867605_2b_Dog_US_142805_2009.t=0.0079377443
11929871;
RkVCgCgf.tree_id_0.Node300.t=0;
RkVCgCgf.tree_id_0.VAC_FJ222822_2b_FortDodge_2008.t=0.00780291808798
0765;
RkVCgCgf.tree_id_0.VAC_JN625223_INDIA_vac5_2011.t=0.0078713185401353
52;
RkVCgCgf.tree_id_0.USA_EU659119_2b_CPV_410_2000.t=0;
RkVCgCgf.tree_id_0.USA_EU659120_2b_CPV_411a_1998.t=0.00779387568120
```

```
299;
RkVCgCgf.tree_id_0.Node307.t=0.007816408510882443;
RkVCgCgf.tree_id_0.Node305.t=0;
RkVCgCgf.tree_id_0.Node303.t=0.007855808628859295;
RkVCgCgf.tree_id_0.Node299.t=0.01498790799383721;
RkVCgCgf.tree_id_0.Node279.t=0;
RkVCgCgf.tree_id_0.CHI_GQ857609_CPV08_01_2008.t=0.008397524657702309
;
RkVCgCgf.tree_id_0.CHI_GU569940_2b_YN0203_2002.t=0.00787048457131769
3;
RkVCgCgf.tree_id_0.Node311.t=0.02790519814309516;
RkVCgCgf.tree_id_0.JPN_AB115504_2c_97_008_1997.t=0.00855155351334259
9;
RkVCgCgf.tree_id_0.TAW_U72696_2b_T10_1996.t=0;
RkVCgCgf.tree_id_0.Node320.t=0;
RkVCgCgf.tree_id_0.TAW_U72695_2a_T4_1996.t=0.00782002531030208;
RkVCgCgf.tree_id_0.Node319.t=0.008570817194713667;
RkVCgCgf.tree_id_0.CHI_GQ857596_CPV05_01_2005.t=0.008524319102257082
;
RkVCgCgf.tree_id_0.CHI_GQ857600_CPV06_01_2006.t=0.008057237541828681
;
RkVCgCgf.tree_id_0.Node324.t=0.01622135368879309;
RkVCgCgf.tree_id_0.Node318.t=0;
RkVCgCgf.tree_id_0.THA_FJ869125_KU5_2004.t=0;
RkVCgCgf.tree_id_0.Node317.t=0.008531189784487322;
RkVCgCgf.tree_id_0.USA_JX475237_CT_372_2011.t=0;
RkVCgCgf.tree_id_0.KOR_EF599097_2b_DH326_2006.t=0.008526304538440723
;
RkVCgCgf.tree_id_0.Node328.t=0.008527526714329093;
RkVCgCgf.tree_id_0.Node316.t=0;
RkVCgCgf.tree_id_0.CHI_EU483515_2b_ZD13_2007.t=0.008479517651549777;
RkVCgCgf.tree_id_0.JPN_LC270891_2b_9985_2017.t=0.01772746947710514;
RkVCgCgf.tree_id_0.JPN_AB437433_1887_M_2_2008.t=0.008532683850670903
;
RkVCgCgf.tree_id_0.TAW_FJ265781_CPV307_2005.t=0;
RkVCgCgf.tree_id_0.TWN_EF592511_TWN1_2006.t=0.008531072816743434;
RkVCgCgf.tree_id_0.TAW_FJ265775_CPV301_2004.t=0.01778323770838885;
RkVCgCgf.tree_id_0.Node339.t=0;
RkVCgCgf.tree_id_0.Node337.t=0;
RkVCgCgf.tree_id_0.Node335.t=0.008506430141336714;
RkVCgCgf.tree_id_0.Node333.t=0;
RkVCgCgf.tree_id_0.Node331.t=0.008505081135379226;
RkVCgCgf.tree_id_0.Node315.t=0.008531101067816817;
RkVCgCgf.tree_id_0.VIE_AB054218_2b_cat_V123_2000.t=0;
RkVCgCgf.tree_id_0.VAC_FJ222823_2b_29_1997.t=0.02590086127183903;
RkVCgCgf.tree_id_0.Node344.t=0;
RkVCgCgf.tree_id_0.ITA_FJ005264_134_2005.t=0.03869005542161161;
RkVCgCgf.tree_id_0.Node343.t=0;
RkVCgCgf.tree_id_0.THA_FJ869122_KU1_2008.t=0.01777552588302886;
RkVCgCgf.tree_id_0.THA_FJ869123_KU3_2008.t=0;
RkVCgCgf.tree_id_0.Node349.t=0.008531644416068428;
RkVCgCgf.tree_id_0.THA_KP715690_VT28_2014.t=0.008451913541337145;
RkVCgCgf.tree_id_0.THA_KP715716_VT143_2014.t=0;
RkVCgCgf.tree_id_0.Node357.t=0.008478737914326443;
```

RkVCgCgf.tree_id_0.THA_KP715691_VT43_2014.t=0;
RkVCgCgf.tree_id_0.Node356.t=0.02908345824926212;
RkVCgCgf.tree_id_0.VIE_AB120722_2b_HCM_18_2003.t=0.00853593846258074
4;
RkVCgCgf.tree_id_0.VIE_AB120724_2b_HNI_2_13_2003.t=0;
RkVCgCgf.tree_id_0.Node361.t=0;
RkVCgCgf.tree_id_0.Node355.t=0;
RkVCgCgf.tree_id_0.CHI_GQ857599_CPV05_04_2005.t=0.008465930900974359
;
RkVCgCgf.tree_id_0.CHI_GQ857601_CPV06_02_2006.t=0.008525654364609307
;
RkVCgCgf.tree_id_0.Node364.t=0.008541149339613408;
RkVCgCgf.tree_id_0.Node354.t=0;
RkVCgCgf.tree_id_0.CHI_GQ857605_CPV07_03_2007.t=0.01614412640159393;
RkVCgCgf.tree_id_0.Node353.t=0.01771919030986227;
RkVCgCgf.tree_id_0.THA_FJ869139_KU66_2003.t=0.008531644417191901;
RkVCgCgf.tree_id_0.VIE_AB120721_2b_HCM_8_2003.t=0.0239828123904500б;
RkVCgCgf.tree_id_0.VIE_AB054221_2b_leopard_V204_2000.t=0.00784597309
1593746;
RkVCgCgf.tree_id_0.VIE_AB054224_2c_leopard_V203_2000.t=0.02494301109
451504;
RkVCgCgf.tree_id_0.VIE_AB120725_2b_HNI_3_4_2003.t=0;
RkVCgCgf.tree_id_0.VIE_AB120723_2b_HCM_23_2003.t=0.00855697596939545
2;
RkVCgCgf.tree_id_0.VIE_AB120720_2b_HCM_6_2003.t=0.008557557394748542
;
RkVCgCgf.tree_id_0.VIE_AB054219_2b_cat_V209_2000.t=0.008049202802760
584;
RkVCgCgf.tree_id_0.VIE_AB054220_2b_cat_V217_2000.t=0.017758065923208
26;
RkVCgCgf.tree_id_0.CHI_EU145954_2b_BJ044_2007.t=0.01775126353421432;
RkVCgCgf.tree_id_0.Node384.t=0;
RkVCgCgf.tree_id_0.Node382.t=0;
RkVCgCgf.tree_id_0.Node380.t=0;
RkVCgCgf.tree_id_0.Node378.t=0;
RkVCgCgf.tree_id_0.Node376.t=0;
RkVCgCgf.tree_id_0.Node374.t=0;
RkVCgCgf.tree_id_0.Node372.t=0;
RkVCgCgf.tree_id_0.Node370.t=0;
RkVCgCgf.tree_id_0.Node368.t=0;
RkVCgCgf.tree_id_0.Node352.t=0;
RkVCgCgf.tree_id_0.Node348.t=0.008558874073305488;
RkVCgCgf.tree_id_0.Node342.t=0.00855831724900275;
RkVCgCgf.tree_id_0.Node314.t=0;
RkVCgCgf.tree_id_0.Node310.t=0;
RkVCgCgf.tree_id_0.Node278.t=0;
RkVCgCgf.tree_id_0.Node274.t=0;
RkVCgCgf.tree_id_0.Node228.t=0;
RkVCgCgf.tree_id_0.Node8.t=0.007823900149177077;
RkVCgCgf.tree_id_0.ITA_FJ005257_54_2008.t=0.008530106516233491;
RkVCgCgf.tree_id_0.ITA_KF373611_2a_409_2010.t=0;
RkVCgCgf.tree_id_0.Node387.t=0.008532365392056122;
RkVCgCgf.tree_id_0.Node7.t=0;
RkVCgCgf.tree_id_0.NZE_AY742933_339_1993.t=0.02778115754431505;

RkVCgCgf.tree_id_0.VIE_AB054223_2c_leopard_V140_2000.t=0.01602301764
930932;
RkVCgCgf.tree_id_0.ITA_GU362932_cat11_2008.t=0.02349040384043101;
RkVCgCgf.tree_id_0.Node393.t=0;
RkVCgCgf.tree_id_0.NIG_HQ602995_15_10_2010.t=0.00850976924814408;
RkVCgCgf.tree_id_0.FRA_DQ025947_2a_02B5_2002.t=0.008487193531986523;
RkVCgCgf.tree_id_0.FRA_DQ026001_2a_04S32_2004.t=0.00848990495490159;
RkVCgCgf.tree_id_0.Node407.t=0;
RkVCgCgf.tree_id_0.FRA_DQ025962_2a_03C6_2003.t=0.008462203057553106;
RkVCgCgf.tree_id_0.Node406.t=0;
RkVCgCgf.tree_id_0.ITA_KF373580_2a_581_2003.t=0;
RkVCgCgf.tree_id_0.Node405.t=0;
RkVCgCgf.tree_id_0.GER_AY742935_U6_1995.t=0;
RkVCgCgf.tree_id_0.FRA_DQ025945_2a_02B3_2002.t=0;
RkVCgCgf.tree_id_0.Node412.t=0;
RkVCgCgf.tree_id_0.Node404.t=0;
RkVCgCgf.tree_id_0.VIE_AB054215_2a_cat_V120_2000.t=0.017596602793301
56;
RkVCgCgf.tree_id_0.Node403.t=0;
RkVCgCgf.tree_id_0.ITA_FJ005255_333_2005.t=0.01765390099405599;
RkVCgCgf.tree_id_0.Node402.t=0;
RkVCgCgf.tree_id_0.FRA_DQ025958_2a_03C2_2003.t=0.008459620780533972;
RkVCgCgf.tree_id_0.Node401.t=0;
RkVCgCgf.tree_id_0.ITA_KX434457_987_10_2010.t=0.008429227696130283;
RkVCgCgf.tree_id_0.FRA_DQ025983_2a_04S14_2004.t=0;
RkVCgCgf.tree_id_0.FRA_DQ025993_2a_04S24_2004.t=0.02740101949583216;
RkVCgCgf.tree_id_0.Node420.t=0;
RkVCgCgf.tree_id_0.Node418.t=0.008465197579397529;
RkVCgCgf.tree_id_0.Node400.t=0;
RkVCgCgf.tree_id_0.FRA_DQ025984_2a_04S15_2004.t=0;
RkVCgCgf.tree_id_0.ITA_FJ005252_96_2002.t=0.0177151742576136;
RkVCgCgf.tree_id_0.Node423.t=0.008486448003014607;
RkVCgCgf.tree_id_0.Node399.t=0.008485215299562089;
RkVCgCgf.tree_id_0.FRA_DQ026002_2a_04S33_2004.t=0.008511173914655411
;
RkVCgCgf.tree_id_0.ITA_KF373592_2a_329_2008.t=0.007912951695578116;
RkVCgCgf.tree_id_0.ITA_AF393506_2a_699_2000.t=0;
RkVCgCgf.tree_id_0.FRA_DQ025943_2a_01S1_2001.t=0.008486321774998716;
RkVCgCgf.tree_id_0.Node432.t=0;
RkVCgCgf.tree_id_0.ITA_KF385388_2a_Sicily_X83090_2009.t=0.0080765959
66150629;
RkVCgCgf.tree_id_0.Node431.t=0.007805859094485544;
RkVCgCgf.tree_id_0.CHI_GQ857612_CPV08_04_2008.t=0.008445261620248591
;
RkVCgCgf.tree_id_0.CHI_GU569939_2a_YN0202_2002.t=0.00785275438416000
2;
RkVCgCgf.tree_id_0.Node437.t=0.01776224575782464;
RkVCgCgf.tree_id_0.HUN_KF539794_H_7_2012.t=0;
RkVCgCgf.tree_id_0.HUN_KF539795_H_8_2012.t=0.01770124070831584;
RkVCgCgf.tree_id_0.Node444.t=0;
RkVCgCgf.tree_id_0.HUN_KF539804_H_212_2012.t=0.024019634740704;
RkVCgCgf.tree_id_0.Node443.t=0;
RkVCgCgf.tree_id_0.HUN_KF539793_H_5_2012.t=0;
RkVCgCgf.tree_id_0.HUN_KF539797_H_11_2012.t=0.01470514294638514;

```
RkVCgCgf.tree_id_0.Node448.t=0.008509494893512827;
RkVCgCgf.tree_id_0.Node442.t=0.007806111468688637;
RkVCgCgf.tree_id_0.HUN_KF539800_H_27_2012.t=0.008506676342280767;
RkVCgCgf.tree_id_0.VIE_AB054217_2a_cat_V154_2000.t=0.025849991086781
84;
RkVCgCgf.tree_id_0.HUN_KF539796_H_9_2012.t=0.00850707708967849;
RkVCgCgf.tree_id_0.Node453.t=0;
RkVCgCgf.tree_id_0.Node451.t=0.008509615532390482;
RkVCgCgf.tree_id_0.Node441.t=0;
RkVCgCgf.tree_id_0.HUN_KF539798_H_31_2012.t=0.0084164662545249;
RkVCgCgf.tree_id_0.HUN_KF539799_H_39_2012.t=0.01772274264976291;
RkVCgCgf.tree_id_0.Node458.t=0.008521754806556413;
RkVCgCgf.tree_id_0.HUN_KF539805_H_36_2012.t=0;
RkVCgCgf.tree_id_0.Node457.t=0.008493918850595274;
RkVCgCgf.tree_id_0.ITA_AF306447_618_2000.t=0;
RkVCgCgf.tree_id_0.FRA_DQ025944_2a_02B2_2002.t=0;
RkVCgCgf.tree_id_0.NIG_HQ602992_19_10_2010.t=0.008509950678713091;
RkVCgCgf.tree_id_0.ITA_AF306446_584_2000.t=0.008509132164908234;
RkVCgCgf.tree_id_0.FRA_DQ025986_2a_04S17_2004.t=0.007829194665919793
;
RkVCgCgf.tree_id_0.ITA_KF373577_2a_714_2001.t=0.008483642966506662;
RkVCgCgf.tree_id_0.FRA_DQ025982_2a_04S13_2004.t=0.01613368026113351;
RkVCgCgf.tree_id_0.ITA_FJ005253_67_2005.t=0.01491085089400893;
RkVCgCgf.tree_id_0.Node474.t=0;
RkVCgCgf.tree_id_0.Node472.t=0;
RkVCgCgf.tree_id_0.Node470.t=0;
RkVCgCgf.tree_id_0.Node468.t=0;
RkVCgCgf.tree_id_0.Node466.t=0;
RkVCgCgf.tree_id_0.Node464.t=0;
RkVCgCgf.tree_id_0.Node462.t=0;
RkVCgCgf.tree_id_0.Node456.t=0;
RkVCgCgf.tree_id_0.Node440.t=0;
RkVCgCgf.tree_id_0.Node436.t=0;
RkVCgCgf.tree_id_0.Node430.t=0;
RkVCgCgf.tree_id_0.Node428.t=0;
RkVCgCgf.tree_id_0.Node426.t=0;
RkVCgCgf.tree_id_0.Node398.t=0.008509764663605061;
RkVCgCgf.tree_id_0.Node396.t=0;
RkVCgCgf.tree_id_0.Node392.t=0.008533786570610939;
RkVCgCgf.tree_id_0.Node390.t=0;
RkVCgCgf.tree_id_0.Node6.t=0;
RkVCgCgf.tree_id_0.THA_FJ869126_KU5_2008.t=0.007802079727715247;
RkVCgCgf.tree_id_0.THA_FJ869137_KU52_2003.t=0;
RkVCgCgf.tree_id_0.Node484.t=0;
RkVCgCgf.tree_id_0.THA_FJ869134_KU23_2003.t=0;
RkVCgCgf.tree_id_0.Node483.t=0;
RkVCgCgf.tree_id_0.CHI_DQ354068_2a_redpanda_RPPV_2004.t=0.0079232667
56788283;
RkVCgCgf.tree_id_0.Node482.t=0;
RkVCgCgf.tree_id_0.KOR_EF599096_DH426_2005.t=0.008505273437947464;
RkVCgCgf.tree_id_0.Node481.t=0.008505299824641531;
RkVCgCgf.tree_id_0.ITA_FJ005258_80_2008.t=0;
RkVCgCgf.tree_id_0.KOR_EF599098_2c_Pome_2006.t=0.04699516895595939;
RkVCgCgf.tree_id_0.FRA_DQ025950_2a_02B9_2002.t=0;
```

RkVCgCgf.tree_id_0.ITA_KX434454_29451_09_2009.t=0;
RkVCgCgf.tree_id_0.Node494.t=0;
RkVCgCgf.tree_id_0.Node492.t=0.008504990495979038;
RkVCgCgf.tree_id_0.Node490.t=0.007822815543564921;
RkVCgCgf.tree_id_0.Node480.t=0;
RkVCgCgf.tree_id_0.THA_FJ869130_KU13_2004.t=0;
RkVCgCgf.tree_id_0.THA_FJ869138_KU53_2003.t=0;
RkVCgCgf.tree_id_0.CHI_KF803615_2011_BJ_B25_2011.t=0.008514046860206
318;
RkVCgCgf.tree_id_0.Node499.t=0;
RkVCgCgf.tree_id_0.Node497.t=0.008514047619701063;
RkVCgCgf.tree_id_0.Node479.t=0;
RkVCgCgf.tree_id_0.CHI_GU569942_2a_JL0202_2002.t=0;
RkVCgCgf.tree_id_0.CHI_GU569946_2a_JL0201_2002.t=0.02505048722586394
;
RkVCgCgf.tree_id_0.Node502.t=0.01620629759975022;
RkVCgCgf.tree_id_0.Node478.t=0.008531288195176642;
RkVCgCgf.tree_id_0.USA_AY742953_435_2003.t=0.01775394707152952;
RkVCgCgf.tree_id_0.ITA_KF373571_2a_685_1999.t=0;
RkVCgCgf.tree_id_0.Node506.t=0;
RkVCgCgf.tree_id_0.THA_FJ869128_KU11_2004.t=0.01782012134327324;
RkVCgCgf.tree_id_0.BRA_DQ340428_2a_BR209_1994.t=0;
RkVCgCgf.tree_id_0.BRA_DQ340431_2a_BR56_1995.t=0.00803350491541302;
RkVCgCgf.tree_id_0.Node513.t=0;
RkVCgCgf.tree_id_0.BRA_DQ340411_2a_BR8_1990.t=0.01775260118207725;
RkVCgCgf.tree_id_0.Node512.t=0;
RkVCgCgf.tree_id_0.BRA_DQ340422_2a_BR22_1993.t=0.007845777280376694;
RkVCgCgf.tree_id_0.BRA_DQ340421_2a_BR597_1992.t=0.008532282474658355
;
RkVCgCgf.tree_id_0.BRA_DQ340419_2a_BR570_1992.t=0.008562008335050355
;
RkVCgCgf.tree_id_0.BRA_DQ340423_2a_BR136_1993.t=0.008531286173906531
;
RkVCgCgf.tree_id_0.Node524.t=0;
RkVCgCgf.tree_id_0.BRA_DQ340413_2a_BR18_1990.t=0.008532282529632862;
RkVCgCgf.tree_id_0.Node523.t=0;
RkVCgCgf.tree_id_0.BRA_DQ340427_2a_BR133_1994.t=0;
RkVCgCgf.tree_id_0.Node522.t=0;
RkVCgCgf.tree_id_0.BRA_DQ340414_2a_BR31_1990.t=0;
RkVCgCgf.tree_id_0.BRA_DQ340416_2a_BR47_1991.t=0;
RkVCgCgf.tree_id_0.BRA_DQ340417_2a_BR52_1991.t=0;
RkVCgCgf.tree_id_0.BRA_DQ340418_2a_BR491_1992.t=0;
RkVCgCgf.tree_id_0.BRA_DQ340424_2a_BR137_1993.t=0;
RkVCgCgf.tree_id_0.BRA_DQ340426_2a_BR84_1994.t=0;
RkVCgCgf.tree_id_0.Node537.t=0;
RkVCgCgf.tree_id_0.Node535.t=0;
RkVCgCgf.tree_id_0.Node533.t=0;
RkVCgCgf.tree_id_0.Node531.t=0;
RkVCgCgf.tree_id_0.Node529.t=0;
RkVCgCgf.tree_id_0.Node521.t=0;
RkVCgCgf.tree_id_0.Node519.t=0;
RkVCgCgf.tree_id_0.Node517.t=0.008530929163812394;
RkVCgCgf.tree_id_0.Node511.t=0.008530779823963818;
RkVCgCgf.tree_id_0.Node509.t=0;

```
RkVCgCgf.tree_id_0.Node505.t=0;
RkVCgCgf.tree_id_0.Node477.t=0;
RkVCgCgf.tree_id_0.Node5.t=0;
RkVCgCgf.tree_id_0.CHI_KF803600_2010_BJ_A68_2010.t=0.054881479716290
44;
RkVCgCgf.tree_id_0.Node4.t=0.008060945152370392;
RkVCgCgf.tree_id_0.USA_EU659118_CPV_13_1981.t=0.01771830040883346;
RkVCgCgf.tree_id_0.CHI_GU569948_2a_CC8601_1986.t=0.00780434475717918
4;
RkVCgCgf.tree_id_0.Node543.t=0;
RkVCgCgf.tree_id_0.JPN_D26079_1993.t=0.01782595242213514;
RkVCgCgf.tree_id_0.Node542.t=0.008531059165447299;
RkVCgCgf.tree_id_0.BRA_DQ340407_2a_BR145_1980.t=0;
RkVCgCgf.tree_id_0.BRA_DQ340408_2a_BR154_1980.t=0;
RkVCgCgf.tree_id_0.Node548.t=0.008559962263540929;
RkVCgCgf.tree_id_0.FRA_DQ025952_2a_03B12_2003.t=0.02768732143482441;
RkVCgCgf.tree_id_0.BRA_DQ340404_2a_BR6_1980.t=0;
RkVCgCgf.tree_id_0.BRA_DQ340405_2a_BR135_1980.t=0;
RkVCgCgf.tree_id_0.BRA_DQ340410_2a_BR315_1986.t=0.02492009356339868;
RkVCgCgf.tree_id_0.USA_M24000_FPV_CPV_31_1988.t=0.008566460036639724
;
RkVCgCgf.tree_id_0.USA_M24003_FPV_CPV_15_1988.t=0;
RkVCgCgf.tree_id_0.Node559.t=0.007831806894144771;
RkVCgCgf.tree_id_0.Node557.t=0;
RkVCgCgf.tree_id_0.Node555.t=0;
RkVCgCgf.tree_id_0.Node553.t=0;
RkVCgCgf.tree_id_0.Node551.t=0;
RkVCgCgf.tree_id_0.Node547.t=0;
RkVCgCgf.tree_id_0.Node541.t=0;
RkVCgCgf.tree_id_0.Node3.t=0.01034501063760017;
RkVCgCgf.tree_id_0.USA_JN867599_Raccoon_KY_39552_2009.t=0;
RkVCgCgf.tree_id_0.USA_JN867611_Raccoon_KY_358_B_2009.t=0.0085335072
3543287;
RkVCgCgf.tree_id_0.Node563.t=0.008534993088737534;
RkVCgCgf.tree_id_0.USA_JN867610_Raccoon_VA_118_A_2007.t=0.0085610313
35726195;
RkVCgCgf.tree_id_0.USA_KJ813890_Redfox_MA_197_2012.t=0.0078138194892
6564;
RkVCgCgf.tree_id_0.USA_JX475284_TN_26_2011.t=0;
RkVCgCgf.tree_id_0.USA_JX475239_GA_06_2011.t=0.008537614880801464;
RkVCgCgf.tree_id_0.USA_JX475279_TN_1_2011.t=0.0162166921042001;
RkVCgCgf.tree_id_0.Node572.t=0;
RkVCgCgf.tree_id_0.Node570.t=0;
RkVCgCgf.tree_id_0.Node568.t=0;
RkVCgCgf.tree_id_0.Node566.t=0;
RkVCgCgf.tree_id_0.Node562.t=0.01963764208892254;
RkVCgCgf.tree_id_0.Node2.t=0;
RkVCgCgf.tree_id_0.HUN_KF539801_H_25_2012.t=0.00848549454370072;
RkVCgCgf.tree_id_0.HUN_KF539803_H_2_2012.t=0.008144876155365565;
RkVCgCgf.tree_id_0.Node575.t=0.01752909735733479;
RkVCgCgf.tree_id_0.Node1.t=0;
RkVCgCgf.tree_id_0.USA_KJ813870_Raccoon_TX_1_2013.t=0.00855790104923
1792;
RkVCgCgf.tree_id_0.USA_JN867598_Bobcat_KS_44_2010.t=0;
```

RkVCgCgf.tree_id_0.USA_KJ813832_Fisher_ND_14_2013.t=0.00860885302022
7902;
RkVCgCgf.tree_id_0.Node582.t=0.00858081786997947;
RkVCgCgf.tree_id_0.USA_KJ813831_Fisher_ND_17_2013.t=0;
RkVCgCgf.tree_id_0.USA_KJ813835_Fisher_ND_19_2013.t=0.00860758198866
8841;
RkVCgCgf.tree_id_0.Node585.t=0.00858081789319723 8;
RkVCgCgf.tree_id_0.Node581.t=0;
RkVCgCgf.tree_id_0.USA_JX475234_ME_258_2011.t=0.008554559589748533;
RkVCgCgf.tree_id_0.USA_JN867618_Raccoon_WI_37_2010.t=0;
RkVCgCgf.tree_id_0.USA_JX475231_CO_280_2011.t=0;
RkVCgCgf.tree_id_0.USA_JX475248_CO_1102_2011.t=0.008581928494088641;
RkVCgCgf.tree_id_0.USA_JX475233_SC_182_A_2011.t=0.008552799205858306
;
RkVCgCgf.tree_id_0.USA_JX475246_CO_2503_2010.t=0.008553477982676737;
RkVCgCgf.tree_id_0.Node596.t=0;
RkVCgCgf.tree_id_0.Node594.t=0;
RkVCgCgf.tree_id_0.Node592.t=0;
RkVCgCgf.tree_id_0.Node590.t=0;
RkVCgCgf.tree_id_0.Node588.t=0;
RkVCgCgf.tree_id_0.Node580.t=0.008556874575401791;
RkVCgCgf.tree_id_0.Node578.t=0.01279359511910699;
RkVCgCgf.tree_id_0.CHI_FJ231389_FPV_monkey_BJ_22_2008.t=0.0306509454
8024807;
RkVCgCgf.tree_id_0.CHI_KJ170680_raccoondog_HLJ11_1_2011.t=0.02356344
962053135;
RkVCgCgf.tree_id_0.Node600.t=0.01615654858996591;
RkVCgCgf.tree_id_0.CHI_GU392242_raccoondog_HB10_2009.t=0.01477309929
465336;
RkVCgCgf.tree_id_0.CHI_GU392244_raccoondog_HB7_2009.t=0.016193401499
26003;
RkVCgCgf.tree_id_0.Node611.t=0;
RkVCgCgf.tree_id_0.CHI_KJ170679_raccoondog_Heb10_2_2010.t=0.00781915
0569245819;
RkVCgCgf.tree_id_0.Node610.t=0;
RkVCgCgf.tree_id_0.CHI_GU392241_raccoondog_HB1_2009.t=0.008525349167
766802;
RkVCgCgf.tree_id_0.Node609.t=0;
RkVCgCgf.tree_id_0.CHI_GU392236_fox_HB1_2009.t=0;
RkVCgCgf.tree_id_0.Node608.t=0.007945771148306295;
RkVCgCgf.tree_id_0.CHI_GU392240_raccoondog_HB3_2009.t=0;
RkVCgCgf.tree_id_0.CHI_GU392239_raccoondog_HB6_2009.t=0.007996794100
600483;
RkVCgCgf.tree_id_0.CHI_KJ194463_raccoondog_HeB10_3_2010.t=0.00852702
5978602656;
RkVCgCgf.tree_id_0.Node619.t=0;
RkVCgCgf.tree_id_0.Node617.t=0;
RkVCgCgf.tree_id_0.Node607.t=0;
RkVCgCgf.tree_id_0.CHI_GU392237_fox_HB2_2009.t=0.02538549791109774;
RkVCgCgf.tree_id_0.Node606.t=0.008153070893989313;
RkVCgCgf.tree_id_0.VAC_FJ011098_Intervet_2006.t=0.04186954181496733;
RkVCgCgf.tree_id_0.VAC_JN625222_INDIA_vac4_2011.t=0.0150553053304889
2;
RkVCgCgf.tree_id_0.ITA_FJ222824_388_05_3_2005.t=0;

RkVCgCgf.tree_id_0.CHI_FJ432718_CPV_Cv_2008.t=0.007958492003244895;
RkVCgCgf.tree_id_0.VAC_JN625219_INDIA_vac1_2011.t=0.0177651574833238
8;
RkVCgCgf.tree_id_0.CHI_KF803602_2010_BJ_A72_2010.t=0;
RkVCgCgf.tree_id_0.Node631.t=0.007847455139695194;
RkVCgCgf.tree_id_0.Node629.t=0;
RkVCgCgf.tree_id_0.Node627.t=0;
RkVCgCgf.tree_id_0.Node625.t=0.008129889686133847;
RkVCgCgf.tree_id_0.Node623.t=0.007836001268611163;
RkVCgCgf.tree_id_0.Node605.t=0;
RkVCgCgf.tree_id_0.JPN_AB437434_1887_f_3_2008.t=0.01626634907648924;
RkVCgCgf.tree_id_0.Node604.t=0.0162788730259433;
RkVCgCgf.tree_id_0.VAC_GU212790_primodog_2009.t=0.01555201802132118;
RkVCgCgf.tree_id_0.VAC_GU212791_vanguard_2009.t=0.008296940477524994
;
RkVCgCgf.tree_id_0.Node640.t=0.02597729341659282;
RkVCgCgf.tree_id_0.VAC_FJ197847_Pfizer_2007.t=0;
RkVCgCgf.tree_id_0.Node639.t=0.007847730529653598;
RkVCgCgf.tree_id_0.VAC_EU914139_Pfizer_2006.t=0.03334043163247082;
RkVCgCgf.tree_id_0.Node638.t=0;
RkVCgCgf.tree_id_0.VAC_KY083089_Singapore_2016.t=0;
RkVCgCgf.tree_id_0.Node637.t=0.01625714251286312;
RkVCgCgf.tree_id_0.USA_M19296_CPV_N_1988.t=0.008087860900021107;
RkVCgCgf.tree_id_0.Node636.t=0.008556823616892688;
RkVCgCgf.tree_id_0.USA_M23255_FPV_Cornell320_1988.t=0;
RkVCgCgf.tree_id_0.USA_M38245_1990.t=0;
RkVCgCgf.tree_id_0.Node651.t=0;
RkVCgCgf.tree_id_0.USA_EU659116_CPV_5_1979.t=0;
RkVCgCgf.tree_id_0.Node650.t=0;
RkVCgCgf.tree_id_0.FIN_U22192_raccoondog_RD_80_1980.t=0;
RkVCgCgf.tree_id_0.FIN_U22193_raccoondog_RD87_1987.t=0.0179145423336
5554;
RkVCgCgf.tree_id_0.Node655.t=0;
RkVCgCgf.tree_id_0.Node649.t=0;
RkVCgCgf.tree_id_0.USA_M10989_1985.t=0.0569746564302582;
RkVCgCgf.tree_id_0.USA_U22186_CPV_128_1995.t=0;
RkVCgCgf.tree_id_0.Node658.t=0.008137150356557505;
RkVCgCgf.tree_id_0.Node648.t=0;
RkVCgCgf.tree_id_0.VAC_JN625221_INDIA_vac3_2011.t=0.0078895956745978
26;
RkVCgCgf.tree_id_0.VAC_JN625220_INDIA_vac2_2011.t=0.060327629573944;
RkVCgCgf.tree_id_0.VAC_FJ011097_Merial_2006.t=0.02617125201773527;
RkVCgCgf.tree_id_0.CHI_GQ169553_Vac2_2007.t=0.02506724904286855;
RkVCgCgf.tree_id_0.Node667.t=0.008466192789585 66;
RkVCgCgf.tree_id_0.VAC_KY083090_Singapore_2016.t=0.04157158804738977
;
RkVCgCgf.tree_id_0.Node666.t=0;
RkVCgCgf.tree_id_0.CHI_GU569943_YB8301_1983.t=0.01628911801156396;
RkVCgCgf.tree_id_0.VAC_JN625224_INDIA_vac6_2011.t=0.0162901481769721
2;
RkVCgCgf.tree_id_0.ARG_KM236572_NNGag_2012.t=0;
RkVCgCgf.tree_id_0.Node673.t=0;
RkVCgCgf.tree_id_0.Node671.t=0.01526649735932325;
RkVCgCgf.tree_id_0.Node665.t=0;

```
RkVCgCgf.tree_id_0.Node663.t=0.01492437502124897;
RkVCgCgf.tree_id_0.Node661.t=0.01631915646924444;
RkVCgCgf.tree_id_0.Node647.t=0.007856298588104963;
RkVCgCgf.tree_id_0.Node635.t=0;
RkVCgCgf.tree_id_0.Node603.t=0;
RkVCgCgf.tree_id_0.Node599.t=0.02264381271867432;
DataSet busted.codon_data = ReadDataFile(USE_NEXUS_FILE_DATA);
DataSetFilter busted.filter.default =
CreateFilter(busted.codon_data,
3,"0-1742","99,103,303,212,283,208,273,200,105,100,91,55,39,35,37,25
7,272,44,261,106,92,104,207,45,193,199,43,223,218-221,168,269,166,16
9,98,305,315,284,286,211,205,276,213,282,285,271,95,107,206,275,217,
292,302,101,97,318,167,260,102,216,279,96,304,316,210,289,215,280,30
0,314,214,281,308,209,290,90,46,201,266,254,29,42,94,274,277,278,287
,
291,293,297-299,306,307,312,288,50,93,301,121,113,256,22,170,24,191,
197,253,249,264,255,265,258,202,82,195,268,181,192,163,27,52,179,198
,
180,259,40,248,263,222,317,115,117,328,333,53,329,309,310,336,114,62
,
20,25,182,122,175,87,88,145,158,8,335,334,140,142,131,189,337,84,321
,
15,127,80,126,2,123,116,129,130,294,296,295,11,13,141,143,144,138,10
,
5,7,14,12,9,3,4,83,111,228,21,6,149,165,33,56,41,226,23,32,0,110,38,
313,48,54,49,108,57,227,19,30,229,146,157,231,232,240,230,234,237,1,
233,235,236,241,18,31,164,17,51,225,47,109,132,136,135,79,81,112,338
,
34,311,134,137,244,159,161,26,224,133,77,78,64,72,71,70,73,65,76,66-
69,74,75,242,86,162,28,60,61,36,58,59,63,325,326,178,184,183,267,204
,
190,203,238,239,262,177,251,250,252,188,185,186,196,187,194,125,246,
155,156,245,154,150,153,152,247,151,119,174,124,128,171,243,16,147,1
48,120,89,319,323,324,327,85,331,332,322,330,173,172,118,139,320,160
,176,270","TAA,TAG,TGA");
ASSUME_REVERSIBLE_MODELS=1;
LikelihoodFunction RkVCgCgf.likelihoodFunction =
(busted.filter.default,RkVCgCgf.tree_id_0);

END;
* Log(L) = -6179.57, AIC-c = 13752.05 (694 estimated parameters)
* For *test* branches, the following rate distribution for branch-
site combinations was inferred

|          Selection mode           |     dN/dS     |Proportion, %|
Notes               |
|-----------------------------------|---------------|-------------|-
----------------------------------|
|        Negative selection         |     0.000     |    2.750    |
|
|        Negative selection         |     0.000     |   96.453    |
Collapsed rate class        |
|      Diversifying selection       |    15.644     |    0.797    |
|
```

Performing the constrained (dN/dS > 1 not allowed) model fit
* Log(L) = -6195.14, AIC-c = 13781.19 (693 estimated parameters)
* For *test* branches under the null (no dN/dS > 1 model), the following rate distribution for branch-site combinations was inferred

Selection mode	dN/dS	Proportion, %	Notes
Negative selection	0.000	20.744	
Negative selection	0.000	68.278	Collapsed rate class
Neutral evolution	1.000	10.977	

Branch-site unrestricted statistical test of episodic diversification [BUSTED]
Likelihood ratio test for episodic diversifying positive selection, **p =   0.0000**.
