# Supplementary material for: Evidence of CPV2c introgression into Croatia and novel insights into phylogeny and cell tropism
Source: Sci Rep. 2019 Nov 15;9:16909. doi: 10.1038/s41598-019-53422-9 (PMC6858334; doi:10.1038/s41598-019-53422-9)
Supplement: Supplementary file 7 — The adaptive Branch-Site Random Effects Likelihood method [file 41598_2019_53422_MOESM7_ESM.pdf]

Evidence of CPV2c introgression into Croatia and novel insights into phylogeny and cell tropism

Dinko Novosel, Tamas Tuboly, Gyula Balka, Levente Szeredi, Ivana Lojkic, Andreja Jungic, Zaklin Acinger Rogic, Tahar Ait Ali, Attila Csagola

Supplementary info file 7.

Results of selection using The adaptive Branch-Site Random Effects Likelihood method

```
/HYPHY 2.3.13.20180601beta(MPI) for Darwin on x86_64\
***************** TYPES OF STANDARD ANALYSES *****************


    (1) Selection Analyses
    (2) Evolutionary Hypothesis Testing
    (3) Relative evolutionary rate inference
    (4) Basic Analyses
    (5) Codon Selection Analyses
    (6) Compartmentalization
    (7) Data File Tools
    (8) Miscellaneous
    (9) Model Comparison
    (10) Kernel Analysis Tools
    (11) Molecular Clock
    (12) Phylogeny Reconstruction
    (13) Positive Selection
    (14) Recombination
    (15) Selection/Recombination
    (16) Relative Rate
    (17) Relative Ratio
    (18) Substitution Rates

1
 Please select type of analyses you want to list (or press ENTER to
process custom batch file):











***************** FILES IN 'Selection Analyses' *****************


    (1) [MEME] Test for episodic site-level selection using MEME
(Mixed Effects Model of Evolution).
```

```
    (2) [FEL] Test for pervasive site-level selection using FEL
(Fixed Effects Likelihood).
    (3) [SLAC] Test for pervasive site-level selection using SLAC
(Single Likelihood Ancestor Counting).
    (4) [FUBAR] Test for pervasive site-level selection using FUBAR
(Fast Unconstrained Bayesian AppRoximation for inferring selection).
    (5) [BUSTED] Test for episodic gene-wide selection using BUSTED
(Branch-site Unrestricted Statistical Test of Episodic
Diversification).
    (6) [aBSREL] Test for lineage-specific evolution using the
branch-site method aBS-REL (Adaptive Branch-Site Random Effects
Likelihood).
    (7) [RELAX] Test for relaxation of selection pressure along a
specified set of test branches using RELAX (a random effects test of
selection relaxation).

 Please select the analysis you would like to perform (or press
ENTER to return to the list of analysis types):6

Analysis Description
--------------------
aBSREL (Adaptive branch-site random effects likelihood) uses an
adaptive
random effects branch-site model framework to test whether each
branch
has evolved under positive selection, using a procedure which infers
an
optimal number of rate categories per branch.

- __Requirements__: in-frame codon alignment and a phylogenetic tree

- __Citation__: Less Is More: An Adaptive Branch-Site Random Effects
Model for Efficient
Detection of Episodic Diversifying Selection (2015). Mol Biol Evol
32
(5): 1342-1353

- __Written by__: Sergei L Kosakovsky Pond, Ben Murrell, Steven
Weaver and Temple iGEM /
UCSD viral evolution group

- __Contact Information__: spond@temple.edu

- __Analysis Version__: 2.0



####Choose Genetic Code

1. [**Universal**] Universal code. (Genebank transl_table=1).
2. [**Vertebrate mtDNA**] Vertebrate mitochondrial DNA code.
(Genebank transl_table=2).
3. [**Yeast mtDNA**] Yeast mitochondrial DNA code. (Genebank
transl_table=3).
```

```
4. [**Mold/Protozoan mtDNA**] Mold, Protozoan and Coelenterate
mitochondrial DNA and the Mycloplasma/Spiroplasma code. (Genebank
transl_table=4).
5. [**Invertebrate mtDNA**] Invertebrate mitochondrial DNA code.
(Genebank transl_table=5).
6. [**Ciliate Nuclear**] Ciliate, Dasycladacean and Hexamita Nuclear
code. (Genebank transl_table=6).
7. [**Echinoderm mtDNA**] Echinoderm mitochondrial DNA code.
(Genebank transl_table=9).
8. [**Euplotid Nuclear**] Euplotid Nuclear code. (Genebank
transl_table=10).
9. [**Alt. Yeast Nuclear**] Alternative Yeast Nuclear code.
(Genebank transl_table=12).
10. [**Ascidian mtDNA**] Ascidian mitochondrial DNA code. (Genebank
transl_table=13).
11. [**Flatworm mtDNA**] Flatworm mitochondrial DNA code. (Genebank
transl_table=14).
12. [**Blepharisma Nuclear**] Blepharisma Nuclear code. (Genebank
transl_table=15).
13. [**Chlorophycean mtDNA**] Chlorophycean Mitochondrial Code
(transl_table=16).
14. [**Trematode mtDNA**] Trematode Mitochondrial Code
(transl_table=21).
15. [**Scenedesmus obliquus mtDNA**] Scenedesmus obliquus
mitochondrial Code (transl_table=22).
16. [**Thraustochytrium mtDNA**] Thraustochytrium Mitochondrial Code
(transl_table=23).
17. [**Pterobranchia mtDNA**] Pterobranchia Mitochondrial Code
(transl_table=24).
18. [**SR1 and Gracilibacteria**] Candidate Division SR1 and
Gracilibacteria Code (transl_table=25).
19. [**Pachysolen Nuclear**] Pachysolen tannophilus Nuclear Code
(transl_table=26).

>Please choose an option (or press q to cancel selection):1


>Select a coding sequence alignment file (`/Users/dinkonovosel/
hyphy/res/TemplateBatchFiles/SelectionAnalyses/`) /Users/
dinkonovosel/CPV_VP2_cod_sel.txt


>A tree was found in the data file:
`((((((((((((((((((((((((((((((((((((((((((((((((((((((ITA/
FJ005218/2c/330/2006,ITA/FJ005233/40/2007),POR/KT275253/2c/
PT036/12/2012),(URU/KC196096/2c/M247/2010,URU/KM457121/2c/
UY247/2010)),(URU/KC196086/2c/M55/2006,URU/KM457106/2c/
UY55/2006)),USA/JX475260/CO/704/2010),ITA/FJ005247/195/2008),ITA/
FJ005226/383/2006),GER/FJ005196/2c/G7/1997),FRA/
DQ025994/04S25/2004),FRA/DQ025960/03C4/2003),FRA/
DQ025951/03B10/2003),FRA/DQ025954/03B14/2003),USA/KJ813848/Bobcat/
ND/1162/2013),URU/KM457104/2c/UY47/2006),FRA/
DQ025969/03S5/2003),USA/KJ813858/Puma/ND/F93/2013),ITA/
FJ005248/219/2008),GER/FJ005199/2c/G172/1997),ITA/
```

FJ005240/208/2007),URU/KC196085/2c/M57/2007),FRA/
DQ025975/04S6/2004),USA/JX475243/ID/22772/2009),USA/JX475252/CO/
1316/2010),FRA/DQ025965/03C9/2003),ECU/KF149984/2c/ME28/2012),(ECU/
KF149962/2c/ME1/2012,(ECU/KF149963/2c/ME10/2012,(ECU/KF149964/2c/
ME23/2012,ECU/KF149969/2c/ME31/2012)))),(ARG/JF414820/Arg44/2009,
(ARG/KM236569/Cuba/2013,(ARG/JF414818/Arg32/2008,ARG/JF414821/
Arg48/2009)))),(ITA/FJ005216/2c/284/2006,(ITA/KU508407/2c/
25835/09/2009,ITA/KX434459/27692/1/11/2011))),(((URU/KM457122/2c/
UY258/2010,URU/KM457124/2c/UY307/2011),URU/KC196093/2c/M307/2011),
((URU/KC196081/2c/M95/2007,URU/KM457109/2c/UY95/2007),(URU/
KC196097/2c/M242/2010,(URU/KM457120/2c/UY242/2010,URU/KM457123/2c/
UY261/2008))))),(URU/KM457103/2c/UY12/2006,(ITA/FJ005209/2c/
303/2004,ITA/FJ005251/239/2008))),(URU/KC196083/2c/M82/2007,URU/
KM457108/2c/UY82/2007)),URU/KC196107/2c/M129/2008),URU/KM457131/2c/
UY368/2011),POR/KT275252/2c/PT013/12/2012),ITA/FJ005231/406/2006),
(ITA/FJ005214/2c/67/2006,BRA/KY073269/UFMT/2015)),ARG/JF414819/
Arg35/2008),USA/KJ813854/Puma/ND/F205/2013),ITA/FJ005232/411/2006),
(((URU/KC196105/2c/M152/2008,URU/KM457113/2c/UY152/2009),ITA/
FJ005212/2c/349/2004),(POR/KT275255/2c/PT238/14/2014,(ITA/
KX434460/52238/12/2012,((URU/KC196091/2c/M326/2011,URU/KM457127/2c/
UY326/2011),((((((((((URU/KC196102/2c/M185/2009,URU/KM457116/2c/
UY185/2009),(HRV/KP859577/2c/HR856/2014,ITA/KX434458/2323/11/2011)),
(URU/KC196101/2c/M187/2009,URU/KM457117/2c/UY187/2009)),AUS/
KU508693/2c/LW/2015),(URU/KC196089/2c/M349/2011,URU/KM457129/2c/
UY349/2011)),ITA/FJ005195/2c/136/2000),FRA/DQ025976/04S7/2004),(USA/
JX475273/MT/909/2012,USA/KJ813888/Coyote/MT/878/2012)),USA/KJ813843/
Bobcat/ND/1160/2013),(FRA/DQ025942/01B1/2001,(FRA/
DQ025964/03C8/2003,(ITA/FJ005206/2c/287/2004,(URU/KM457107/2c/
UY72/2007,(URU/KM457111/2c/UY120/2008,(URU/KM457112/2c/UY135/2008,
(URU/KM457125/2c/UY317/2011,(URU/KM457130/2c/UY354/2011,(URU/
KM457142/2c/UY370/2011,(HRV/KP859574/2c/HR442/2014,(HRV/KP859575/2c/
HR774/2014,(HRV/KP859576/2c/HR793/2014,(AUS/KU508691/2c/HB/2015,
(AUS/KU508692/2c/FH/2015,ITA/
KX434456/45361/09/2009)))))))))))))))))),URU/KM457126/2c/
UY318/2010),(FRA/DQ025985/04S16/2004,(ITA/FJ005205/2c/279/2004,HRV/
KP859578/2c/HR859/2014))),ITA/FJ222821/2c/56/2000),(GER/FJ005260/
G82/1997,USA/KJ813846/Bobcat/ND/974/2013)),((GER/
AY742934/447/1995,RUS/JN033694/Laika/1993),(USA/AY742936/395/1998,
(USA/JX475240/AZ/16382-01/1999,(USA/JX475250/CO/728/2010,USA/
KJ813842/Bobcat/ND/502/2013))))),(((((((((((((((((USA/KJ813828/
Fisher/F1F010712/2013,USA/KJ813881/Graywolf/MI/832/2012),USA/
KJ813844/Bobcat/ND/885/2013),USA/KJ813882/Raccoon/NJ/1423/2012),USA/
KJ813851/Bobcat/ND/1168/2013),USA/JX475278/AR/1069/2012),KOR/
EU009205/2b/K029/2006),USA/JX475247/CO/1246/2010),(USA/KJ813892/
Coyote/AK/218/2013,(USA/JN867604/Dog/IL/137654/2008,USA/JX475242/WI/
18268/2002))),SAF/HQ602969/22/10SA/2010),(USA/AY742955/436/2003,FRA/
DQ025991/2b/04S22/2004)),USA/JN867602/2b/Dog/CA/148743/2008),USA/
JX475251/CO/2235/2009),USA/JN867603/2b/Dog/KS/81213/2009),USA/
KJ813852/Bobcat/ND/1170/2013),FRA/DQ025961/2b/03C5/2003),(USA/
KJ813827/Fisher/F1M111211/2013,USA/KJ813873/Graywolf/MI/850/2012)),
(ECU/KF149971/2c/ME32/2012,IND/KX469432/newCPV/2b/Hiller/2011)),
((ITA/FJ005263/42/2005,ITA/FJ005265/140/2005),(((((((((USA/
M74849/39/1995,USA/U22896/cat/1990),FRA/DQ025992/2b/04S23/2004),USA/
M74852/133/1995),POR/KU662349/greywolf/W33/1996),(POR/KU662350/

greywolf/W52/2005,POL/Z46651/46/1994)),GER/FJ005261/G162/1997),BRA/
DQ340409/2b/BR183/1985),USA/AY742932/193/1991),((USA/
AY742951/431/2003,USA/JN867605/2b/Dog/US/142805/2009),(VAC/
FJ222822/2b/FortDodge/2008,(VAC/JN625223/INDIA/vac5/2011,(USA/
EU659119/2b/CPV/410/2000,USA/EU659120/2b/CPV/411a/1998))))),((CHI/
GQ857609/CPV08/01/2008,CHI/GU569940/2b/YN0203/2002),(((((((JPN/
AB115504/2c/97/008/1997,TAW/U72696/2b/T10/1996),TAW/U72695/2a/
T4/1996),(CHI/GQ857596/CPV05/01/2005,CHI/GQ857600/
CPV06/01/2006)),THA/FJ869125/KU5/2004),(USA/JX475237/CT/
372/2011,KOR/EF599097/2b/DH326/2006)),(CHI/EU483515/2b/ZD13/2007,
(JPN/LC270891/2b/9985/2017,(JPN/AB437433/1887/M/2/2008,(TAW/
FJ265781/CPV307/2005,(TWN/EF592511/TWN1/2006,TAW/FJ265775/
CPV301/2004)))))),(((VIE/AB054218/2b/cat/V123/2000,VAC/FJ222823/2b/
29/1997),ITA/FJ005264/134/2005),((THA/FJ869122/KU1/2008,THA/
FJ869123/KU3/2008),(((((THA/KP715690/VT28/2014,THA/KP715716/
VT143/2014),THA/KP715691/VT43/2014),(VIE/AB120722/2b/HCM/
18/2003,VIE/AB120724/2b/HNI/2/13/2003)),(CHI/GQ857599/
CPV05/04/2005,CHI/GQ857601/CPV06/02/2006)),CHI/GQ857605/
CPV07/03/2007),(THA/FJ869139/KU66/2003,(VIE/AB120721/2b/HCM/8/2003,
(VIE/AB054221/2b/leopard/V204/2000,(VIE/AB054224/2c/leopard/
V203/2000,(VIE/AB120725/2b/HNI/3/4/2003,(VIE/AB120723/2b/HCM/
23/2003,(VIE/AB120720/2b/HCM/6/2003,(VIE/AB054219/2b/cat/V209/2000,
(VIE/AB054220/2b/cat/V217/2000,CHI/EU145954/2b/
BJ044/20O7)))))))))))))))))),(ITA/FJ005257/54/2008,ITA/KF373611/2a/
409/2010)),(NZE/AY742933/339/1993,((VIE/AB054223/2c/leopard/
V140/2000,ITA/GU362932/cat11/2008),(NIG/HQ602995/15/10/2010,
((((((((((FRA/DQ025947/2a/02B5/2002,FRA/DQ026001/2a/04S32/2004),FRA/
DQ025962/2a/03C6/2003),ITA/KF373580/2a/581/2003),(GER/AY742935/
U6/1995,FRA/DQ025945/2a/02B3/2002)),VIE/AB054215/2a/cat/
V120/2000),ITA/FJ005255/333/2005),FRA/DQ025958/2a/03C2/2003),(ITA/
KX434457/987/10/2010,(FRA/DQ025983/2a/04S14/2004,FRA/DQ025993/2a/
04S24/2004))),(FRA/DQ025984/2a/04S15/2004,ITA/FJ005252/96/2002)),
(FRA/DQ026002/2a/04S33/2004,(ITA/KF373592/2a/329/2008,(((ITA/
AF393506/2a/699/2000,FRA/DQ025943/2a/01S1/2001),ITA/KF385388/2a/
Sicily/X83090/2009),((CHI/GQ857612/CPV08/04/2008,CHI/GU569939/2a/
YN0202/2002),(((((HUN/KF539794/H/7/2012,HUN/KF539795/H/8/2012),HUN/
KF539804/H/212/2012),(HUN/KF539793/H/5/2012,HUN/KF539797/H/
11/2012)),(HUN/KF539800/H/27/2012,(VIE/AB054217/2a/cat/
V154/2000,HUN/KF539796/H/9/2012))),(((HUN/KF539798/H/31/2012,HUN/
KF539799/H/39/2012),HUN/KF539805/H/36/2012),(ITA/AF306447/618/2000,
(FRA/DQ025944/2a/02B2/2002,(NIG/HQ602992/19/10/2010,(ITA/
AF306446/584/2000,(FRA/DQ025986/2a/04S17/2004,(ITA/KF373577/2a/
714/2001,(FRA/DQ025982/2a/04S13/2004,ITA/
FJ005253/67/2005))))))))))))))))),(((((((THA/FJ869126/
KU5/2008,THA/FJ869137/KU52/2003),THA/FJ869134/KU23/2003),CHI/
DQ354068/2a/redpanda/RPPV/2004),KOR/EF599096/DH426/2005),(ITA/
FJ005258/80/2008,(KOR/EF599098/2c/Pome/2006,(FRA/DQ025950/2a/
02B9/2002,ITA/KX434454/29451/09/2009)))),(THA/FJ869130/KU13/2004,
(THA/FJ869138/KU53/2003,CHI/KF803615/2011/BJ/B25/2011))),(CHI/
GU569942/2a/JL0202/2002,CHI/GU569946/2a/JL0201/2002)),((USA/
AY742953/435/2003,ITA/KF373571/2a/685/1999),(THA/FJ869128/KU11/2004,
(((BRA/DQ340428/2a/BR209/1994,BRA/DQ340431/2a/BR56/1995),BRA/
DQ340411/2a/BR8/1990),(BRA/DQ340422/2a/BR22/1993,(BRA/DQ340421/2a/
BR597/1992,((((BRA/DQ340419/2a/BR570/1992,BRA/DQ340423/2a/

BR136/1993),BRA/DQ340413/2a/BR18/1990),BRA/DQ340427/2a/BR133/1994),
(BRA/DQ340414/2a/BR31/1990,(BRA/DQ340416/2a/BR47/1991,(BRA/
DQ340417/2a/BR52/1991,(BRA/DQ340418/2a/BR491/1992,(BRA/DQ340424/2a/
BR137/1993,BRA/DQ340426/2a/BR84/1994)))))))))))),CHI/KF803600/2010/
BJ/A68/2010),(((USA/EU659118/CPV/13/1981,CHI/GU569948/2a/
CC8601/1986),JPN/D26079/1993),((BRA/DQ340407/2a/BR145/1980,BRA/
DQ340408/2a/BR154/1980),(FRA/DQ025952/2a/03B12/2003,(BRA/
DQ340404/2a/BR6/1980,(BRA/DQ340405/2a/BR135/1980,(BRA/DQ340410/2a/
BR315/1986,(USA/M24000/FPV/CPV/31/1988,USA/M24003/FPV/CPV/
15/1988)))))))),((USA/JN867599/Raccoon/KY/39552/2009,USA/JN867611/
Raccoon/KY/358-B/2009),(USA/JN867610/Raccoon/VA/118-A/2007,(USA/
KJ813890/Redfox/MA/197/2012,(USA/JX475284/TN/26/2011,(USA/JX475239/
GA/06/2011,USA/JX475279/TN/1/2011)))))),(HUN/KF539801/H/25/2012,HUN/
KF539803/H/2/2012)),(USA/KJ813870/Raccoon/TX/1/2013,(((USA/JN867598/
Bobcat/KS/44/2010,USA/KJ813832/Fisher/ND/14/2013),(USA/KJ813831/
Fisher/ND/17/2013,USA/KJ813835/Fisher/ND/19/2013)),(USA/JX475234/ME/
258/2011,(USA/JN867618//Raccoon/WI/37/2010,(USA/JX475231/CO/
280/2011,(USA/JX475248/CO/1102/2011,(USA/JX475233/SC/182-A/2011,USA/
JX475246/CO/2503/2010))))))),((CHI/FJ231389/FPV/monkey/
BJ-22/2008,CHI/KJ170680/raccoondog/HLJ11/1/2011),((((((((CHI/
GU392242/raccoondog/HB10/2009,CHI/GU392244/raccoondog/HB7/2009),CHI/
KJ170679/raccoondog/Heb10/2/2010),CHI/GU392241/raccoondog/
HB1/2009),CHI/GU392236/fox/HB1/2009),(CHI/GU392240/raccoondog/
HB3/2009,(CHI/GU392239/raccoondog/HB6/2009,CHI/KJ194463/raccoondog/
HeB10/3/2010))),CHI/GU392237/fox/HB2/2009),(VAC/FJ011098/Intervet/
2006,(VAC/JN625222/INDIA/vac4/2011,(ITA/FJ222824/388/05/3/2005,(CHI/
FJ432718/CPV/Cv/2008,(VAC/JN625219/INDIA/vac1/2011,CHI/
KF803602/2010/BJ/A72/2010)))))),JPN/AB437434/1887/f/3/2008),
((((((VAC/GU212790/primodog/2009,VAC/GU212791/vanguard/2009),VAC/
FJ197847/Pfizer/2007),VAC/EU914139/Pfizer//2006),VAC/KY083089/
Singapore/2016),USA/M19296/CPV/N/1988),(((((USA/M23255/FPV/
Cornell320/1988,USA/M38245/1990),USA/EU659116/CPV/5/1979),(FIN/
U22192/raccoondog/RD-80/1980,FIN/U22193/raccoondog/RD87/1987)),(USA/
M10989/1985,USA/U22186/CPV/128/1995)),(VAC/JN625221/INDIA/vac3/2011,
(VAC/JN625220/INDIA/vac2/2011,(((VAC/FJ011097/Merial/2006,CHI/
GQ169553/Vac2/2007),VAC/KY083090/Singapore/2016),(CHI/GU569943/
YB8301/1983,(VAC/JN625224/INDIA/vac6/2011,ARG/KM236572/NNGag/
2012))))))))))`

>Would you like to use it (y/n)? y

>Loaded a multiple sequence alignment with **339** sequences, **581** codons, and **1** partitions from `/Users/dinkonovosel/CPV_VP2_cod_sel.txt`

####Choose the set of branches to test for selection

1. [**All**] Include all branches in the analysis
2. [**Internal**] Include all internal branches in the analysis
3. [**Leaves**] Include all leaf branches in the analysis
4. [**Unlabeled branches**] Set of 675 unlabeled branches

>Please choose an option (or press q to cancel selection):1

* Selected 675 branches for testing: `ITA_FJ005218_2c_330_2006,
ITA_FJ005233_40_2007, Node53, POR_KT275253_2c_PT036_12_2012, Node52,
URU_KC196096_2c_M247_2010, URU_KM457121_2c_UY247_2010, Node57,
Node51, URU_KC196086_2c_M55_2006, URU_KM457106_2c_UY55_2006, Node60,
Node50, USA_JX475260_CO_704_2010, Node49, ITA_FJ005247_195_2008,
Node48, ITA_FJ005226_383_2006, Node47, GER_FJ005196_2c_G7_1997,
Node46, FRA_DQ025994_04S25_2004, Node45, FRA_DQ025960_03C4_2003,
Node44, FRA_DQ025951_03B10_2003, Node43, FRA_DQ025954_03B14_2003,
Node42, USA_KJ813848_Bobcat_ND_1162_2013, Node41,
URU_KM457104_2c_UY47_2006, Node40, FRA_DQ025969_03S5_2003, Node39,
USA_KJ813858_Puma_ND_F93_2013, Node38, ITA_FJ005248_219_2008,
Node37, GER_FJ005199_2c_G172_1997, Node36, ITA_FJ005240_208_2007,
Node35, URU_KC196085_2c_M57_2007, Node34, FRA_DQ025975_04S6_2004,
Node33, USA_JX475243_ID_22772_2009, Node32,
USA_JX475252_CO_1316_2010, Node31, FRA_DQ025965_03C9_2003, Node30,
ECU_KF149984_2c_ME28_2012, Node29, ECU_KF149962_2c_ME1_2012,
ECU_KF149963_2c_ME10_2012, ECU_KF149964_2c_ME23_2012,
ECU_KF149969_2c_ME31_2012, Node88, Node86, Node84, Node28,
ARG_JF414820_Arg44_2009, ARG_KM236569_Cuba_2013,
ARG_JF414818_Arg32_2008, ARG_JF414821_Arg48_2009, Node95, Node93,
Node91, Node27, ITA_FJ005216_2c_284_2006,
ITA_KU508407_2c_25835_09_2009, ITA_KX434459_27692_1_11_2011,
Node100, Node98, Node26, URU_KM457122_2c_UY258_2010,
URU_KM457124_2c_UY307_2011, Node105, URU_KC196093_2c_M307_2011,
Node104, URU_KC196081_2c_M95_2007, URU_KM457109_2c_UY95_2007,
Node110, URU_KC196097_2c_M242_2010, URU_KM457120_2c_UY242_2010,
URU_KM457123_2c_UY261_2008, Node115, Node113, Node109, Node103,
Node25, URU_KM457103_2c_UY12_2006, ITA_FJ005209_2c_303_2004,
ITA_FJ005251_239_2008, Node120, Node118, Node24,
URU_KC196083_2c_M82_2007, URU_KM457108_2c_UY82_2007, Node123,
Node23, URU_KC196107_2c_M129_2008, Node22,
URU_KM457131_2c_UY368_2011, Node21, POR_KT275252_2c_PT013_12_2012,
Node20, ITA_FJ005231_406_2006, Node19, ITA_FJ005214_2c_67_2006,
BRA_KY073269_UFMT_2015, Node130, Node18, ARG_JF414819_Arg35_2008,
Node17, USA_KJ813854_Puma_ND_F205_2013, Node16,
ITA_FJ005232_411_2006, Node15, URU_KC196105_2c_M152_2008,
URU_KM457113_2c_UY152_2009, Node138, ITA_FJ005212_2c_349_2004,
Node137, POR_KT275255_2c_PT238_14_2014, ITA_KX434460_52238_12_2012,
URU_KC196091_2c_M326_2011, URU_KM457127_2c_UY326_2011, Node147,
URU_KC196102_2c_M185_2009, URU_KM457116_2c_UY185_2009, Node159,
HRV_KP859577_2c_HR856_2014, ITA_KX434458_2323_11_2011, Node162,
Node158, URU_KC196101_2c_M187_2009, URU_KM457117_2c_UY187_2009,
Node165, Node157, AUS_KU508693_2c_LW_2015, Node156,
URU_KC196089_2c_M349_2011, URU_KM457129_2c_UY349_2011, Node169,
Node155, ITA_FJ005195_2c_136_2000, Node154, FRA_DQ025976_04S7_2004,
Node153, USA_JX475273_MT_909_2012, USA_KJ813888_Coyote_MT_878_2012,
Node174, Node152, USA_KJ813843_Bobcat_ND_1160_2013, Node151,
FRA_DQ025942_01B1_2001, FRA_DQ025964_03C8_2003,
ITA_FJ005206_2c_287_2004, URU_KM457107_2c_UY72_2007,
URU_KM457111_2c_UY120_2008, URU_KM457112_2c_UY135_2008,
URU_KM457125_2c_UY317_2011, URU_KM457130_2c_UY354_2011,

URU_KM457142_2c_UY370_2011, HRV_KP859574_2c_HR442_2014,
HRV_KP859575_2c_HR774_2014, HRV_KP859576_2c_HR793_2014,
AUS_KU508691_2c_HB_2015, AUS_KU508692_2c_FH_2015,
ITA_KX434456_45361_09_2009, Node204, Node202, Node200, Node198,
Node196, Node194, Node192, Node190, Node188, Node186, Node184,
Node182, Node180, Node178, Node150, Node146, Node144, Node142,
Node136, Node14, URU_KM457126_2c_UY318_2010, Node13,
FRA_DQ025985_04S16_2004, ITA_FJ005205_2c_279_2004,
HRV_KP859578_2c_HR859_2014, Node210, Node208, Node12,
ITA_FJ222821_2c_56_2000, Node11, GER_FJ005260_G82_1997,
USA_KJ813846_Bobcat_ND_974_2013, Node214, Node10,
GER_AY742934_447_1995, RUS_JN033694_Laika_1993, Node218,
USA_AY742936_395_1998, USA_JX475240_AZ_16382_01_1999,
USA_JX475250_CO_728_2010, USA_KJ813842_Bobcat_ND_502_2013, Node225,
Node223, Node221, Node217, Node9,
USA_KJ813828_Fisher_F1F010712_2013,
USA_KJ813881_Graywolf_MI_832_2012, Node245,
USA_KJ813844_Bobcat_ND_885_2013, Node244,
USA_KJ813882_Raccoon_NJ_1423_2012, Node243,
USA_KJ813851_Bobcat_ND_1168_2013, Node242,
USA_JX475278_AR_1069_2012, Node241, KOR_EU009205_2b_K029_2006,
Node240, USA_JX475247_CO_1246_2010, Node239,
USA_KJ813892_Coyote_AK_218_2013, USA_JN867604_Dog_IL_137654_2008,
USA_JX475242_WI_18268_2002, Node256, Node254, Node238,
SAF_HQ602969_22_10SA_2010, Node237, USA_AY742955_436_2003,
FRA_DQ025991_2b_04S22_2004, Node260, Node236,
USA_JN867602_2b_Dog_CA_148743_2008, Node235,
USA_JX475251_CO_2235_2009, Node234,
USA_JN867603_2b_Dog_KS_81213_2009, Node233,
USA_KJ813852_Bobcat_ND_1170_2013, Node232,
FRA_DQ025961_2b_03C5_2003, Node231,
USA_KJ813827_Fisher_F1M111211_2013,
USA_KJ813873_Graywolf_MI_850_2012, Node268, Node230,
ECU_KF149971_2c_ME32_2012, IND_KX469432_newCPV_2b_Hiller_2011,
Node271, Node229, ITA_FJ005263_42_2005, ITA_FJ005265_140_2005,
Node275, USA_M74849_39_1995, USA_U22896_cat_1990, Node287,
FRA_DQ025992_2b_04S23_2004, Node286, USA_M74852_133_1995, Node285,
POR_KU662349_greywolf_W33_1996, Node284,
POR_KU662350_greywolf_W52_2005, POL_Z46651_46_1994, Node293,
Node283, GER_FJ005261_G162_1997, Node282,
BRA_DQ340409_2b_BR183_1985, Node281, USA_AY742932_193_1991, Node280,
USA_AY742951_431_2003, USA_JN867605_2b_Dog_US_142805_2009, Node300,
VAC_FJ222822_2b_FortDodge_2008, VAC_JN625223_INDIA_vac5_2011,
USA_EU659119_2b_CPV_410_2000, USA_EU659120_2b_CPV_411a_1998,
Node307, Node305, Node303, Node299, Node279,
CHI_GQ857609_CPV08_01_2008, CHI_GU569940_2b_YN0203_2002, Node311,
JPN_AB115504_2c_97_008_1997, TAW_U72696_2b_T10_1996, Node320,
TAW_U72695_2a_T4_1996, Node319, CHI_GQ857596_CPV05_01_2005,
CHI_GQ857600_CPV06_01_2006, Node324, Node318, THA_FJ869125_KU5_2004,
Node317, USA_JX475237_CT_372_2011, KOR_EF599097_2b_DH326_2006,
Node328, Node316, CHI_EU483515_2b_ZD13_2007,
JPN_LC270891_2b_9985_2017, JPN_AB437433_1887_M_2_2008,
TAW_FJ265781_CPV307_2005, TWN_EF592511_TWN1_2006,
TAW_FJ265775_CPV301_2004, Node339, Node337, Node335, Node333,

Node331, Node315, VIE_AB054218_2b_cat_V123_2000,
VAC_FJ222823_2b_29_1997, Node344, ITA_FJ005264_134_2005, Node343,
THA_FJ869122_KU1_2008, THA_FJ869123_KU3_2008, Node349,
THA_KP715690_VT28_2014, THA_KP715716_VT143_2014, Node357,
THA_KP715691_VT43_2014, Node356, VIE_AB120722_2b_HCM_18_2003,
VIE_AB120724_2b_HNI_2_13_2003, Node361, Node355,
CHI_GQ857599_CPV05_04_2005, CHI_GQ857601_CPV06_02_2006, Node364,
Node354, CHI_GQ857605_CPV07_03_2007, Node353,
THA_FJ869139_KU66_2003, VIE_AB120721_2b_HCM_8_2003,
VIE_AB054221_2b_leopard_V204_2000,
VIE_AB054224_2c_leopard_V203_2000, VIE_AB120725_2b_HNI_3_4_2003,
VIE_AB120723_2b_HCM_23_2003, VIE_AB120720_2b_HCM_6_2003,
VIE_AB054219_2b_cat_V209_2000, VIE_AB054220_2b_cat_V217_2000,
CHI_EU145954_2b_BJ044_2007, Node384, Node382, Node380, Node378,
Node376, Node374, Node372, Node370, Node368, Node352, Node348,
Node342, Node314, Node310, Node278, Node274, Node228, Node8,
ITA_FJ005257_54_2008, ITA_KF373611_2a_409_2010, Node387, Node7,
NZE_AY742933_339_1993, VIE_AB054223_2c_leopard_V140_2000,
ITA_GU362932_cat11_2008, Node393, NIG_HQ602995_15_10_2010,
FRA_DQ025947_2a_02B5_2002, FRA_DQ026001_2a_04S32_2004, Node407,
FRA_DQ025962_2a_03C6_2003, Node406, ITA_KF373580_2a_581_2003,
Node405, GER_AY742935_U6_1995, FRA_DQ025945_2a_02B3_2002, Node412,
Node404, VIE_AB054215_2a_cat_V120_2000, Node403,
ITA_FJ005255_333_2005, Node402, FRA_DQ025958_2a_03C2_2003, Node401,
ITA_KX434457_987_10_2010, FRA_DQ025983_2a_04S14_2004,
FRA_DQ025993_2a_04S24_2004, Node420, Node418, Node400,
FRA_DQ025984_2a_04S15_2004, ITA_FJ005252_96_2002, Node423, Node399,
FRA_DQ026002_2a_04S33_2004, ITA_KF373592_2a_329_2008,
ITA_AF393506_2a_699_2000, FRA_DQ025943_2a_01S1_2001, Node432,
ITA_KF385388_2a_Sicily_X83090_2009, Node431,
CHI_GQ857612_CPV08_04_2008, CHI_GU569939_2a_YN0202_2002, Node437,
HUN_KF539794_H_7_2012, HUN_KF539795_H_8_2012, Node444,
HUN_KF539804_H_212_2012, Node443, HUN_KF539793_H_5_2012,
HUN_KF539797_H_11_2012, Node448, Node442, HUN_KF539800_H_27_2012,
VIE_AB054217_2a_cat_V154_2000, HUN_KF539796_H_9_2012, Node453,
Node451, Node441, HUN_KF539798_H_31_2012, HUN_KF539799_H_39_2012,
Node458, HUN_KF539805_H_36_2012, Node457, ITA_AF306447_618_2000,
FRA_DQ025944_2a_02B2_2002, NIG_HQ602992_19_10_2010,
ITA_AF306446_584_2000, FRA_DQ025986_2a_04S17_2004,
ITA_KF373577_2a_714_2001, FRA_DQ025982_2a_04S13_2004,
ITA_FJ005253_67_2005, Node474, Node472, Node470, Node468, Node466,
Node464, Node462, Node456, Node440, Node436, Node430, Node428,
Node426, Node398, Node396, Node392, Node390, Node6,
THA_FJ869126_KU5_2008, THA_FJ869137_KU52_2003, Node484,
THA_FJ869134_KU23_2003, Node483, CHI_DQ354068_2a_redpanda_RPPV_2004,
Node482, KOR_EF599096_DH426_2005, Node481, ITA_FJ005258_80_2008,
KOR_EF599098_2c_Pome_2006, FRA_DQ025950_2a_02B9_2002,
ITA_KX434454_29451_09_2009, Node494, Node492, Node490, Node480,
THA_FJ869130_KU13_2004, THA_FJ869138_KU53_2003,
CHI_KF803615_2011_BJ_B25_2011, Node499, Node497, Node479,
CHI_GU569942_2a_JL0202_2002, CHI_GU569946_2a_JL0201_2002, Node502,
Node478, USA_AY742953_435_2003, ITA_KF373571_2a_685_1999, Node506,
THA_FJ869128_KU11_2004, BRA_DQ340428_2a_BR209_1994,
BRA_DQ340431_2a_BR56_1995, Node513, BRA_DQ340411_2a_BR8_1990,

Node512, BRA_DQ340422_2a_BR22_1993, BRA_DQ340421_2a_BR597_1992, BRA_DQ340419_2a_BR570_1992, BRA_DQ340423_2a_BR136_1993, Node524, BRA_DQ340413_2a_BR18_1990, Node523, BRA_DQ340427_2a_BR133_1994, Node522, BRA_DQ340414_2a_BR31_1990, BRA_DQ340416_2a_BR47_1991, BRA_DQ340417_2a_BR52_1991, BRA_DQ340418_2a_BR491_1992, BRA_DQ340424_2a_BR137_1993, BRA_DQ340426_2a_BR84_1994, Node537, Node535, Node533, Node531, Node529, Node521, Node519, Node517, Node511, Node509, Node505, Node477, Node5, CHI_KF803600_2010_BJ_A68_2010, Node4, USA_EU659118_CPV_13_1981, CHI_GU569948_2a_CC8601_1986, Node543, JPN_D26079_1993, Node542, BRA_DQ340407_2a_BR145_1980, BRA_DQ340408_2a_BR154_1980, Node548, FRA_DQ025952_2a_03B12_2003, BRA_DQ340404_2a_BR6_1980, BRA_DQ340405_2a_BR135_1980, BRA_DQ340410_2a_BR315_1986, USA_M24000_FPV_CPV_31_1988, USA_M24003_FPV_CPV_15_1988, Node559, Node557, Node555, Node553, Node551, Node547, Node541, Node3, USA_JN867599_Raccoon_KY_39552_2009, USA_JN867611_Raccoon_KY_358_B_2009, Node563, USA_JN867610_Raccoon_VA_118_A_2007, USA_KJ813890_Redfox_MA_197_2012, USA_JX475284_TN_26_2011, USA_JX475239_GA_06_2011, USA_JX475279_TN_1_2011, Node572, Node570, Node568, Node566, Node562, Node2, HUN_KF539801_H_25_2012, HUN_KF539803_H_2_2012, Node575, Node1, USA_KJ813870_Raccoon_TX_1_2013, USA_JN867598_Bobcat_KS_44_2010, USA_KJ813832_Fisher_ND_14_2013, Node582, USA_KJ813831_Fisher_ND_17_2013, USA_KJ813835_Fisher_ND_19_2013, Node585, Node581, USA_JX475234_ME_258_2011, USA_JN867618_Raccoon_WI_37_2010, USA_JX475231_CO_280_2011, USA_JX475248_CO_1102_2011, USA_JX475233_SC_182_A_2011, USA_JX475246_CO_2503_2010, Node596, Node594, Node592, Node590, Node588, Node580, Node578, CHI_FJ231389_FPV_monkey_BJ_22_2008, CHI_KJ170680_raccoondog_HLJ11_1_2011, Node600, CHI_GU392242_raccoondog_HB10_2009, CHI_GU392244_raccoondog_HB7_2009, Node611, CHI_KJ170679_raccoondog_Heb10_2_2010, Node610, CHI_GU392241_raccoondog_HB1_2009, Node609, CHI_GU392236_fox_HB1_2009, Node608, CHI_GU392240_raccoondog_HB3_2009, CHI_GU392239_raccoondog_HB6_2009, CHI_KJ194463_raccoondog_HeB10_3_2010, Node619, Node617, Node607, CHI_GU392237_fox_HB2_2009, Node606, VAC_FJ011098_Intervet_2006, VAC_JN625222_INDIA_vac4_2011, ITA_FJ222824_388_05_3_2005, CHI_FJ432718_CPV_Cv_2008, VAC_JN625219_INDIA_vac1_2011, CHI_KF803602_2010_BJ_A72_2010, Node631, Node629, Node627, Node625, Node623, Node605, JPN_AB437434_1887_f_3_2008, Node604, VAC_GU212790_primodog_2009, VAC_GU212791_vanguard_2009, Node640, VAC_FJ197847_Pfizer_2007, Node639, VAC_EU914139_Pfizer_2006, Node638, VAC_KY083089_Singapore_2016, Node637, USA_M19296_CPV_N_1988, Node636, USA_M23255_FPV_Cornell320_1988, USA_M38245_1990, Node651, USA_EU659116_CPV_5_1979, Node650, FIN_U22192_raccoondog_RD_80_1980, FIN_U22193_raccoondog_RD87_1987, Node655, Node649, USA_M10989_1985, USA_U22186_CPV_128_1995, Node658, Node648, VAC_JN625221_INDIA_vac3_2011, VAC_JN625220_INDIA_vac2_2011, VAC_FJ011097_Merial_2006, CHI_GQ169553_Vac2_2007, Node667, VAC_KY083090_Singapore_2016, Node666, CHI_GU569943_YB8301_1983, VAC_JN625224_INDIA_vac6_2011, ARG_KM236572_NNGag_2012, Node673, Node671, Node665, Node663, Node661, Node647, Node635, Node603,

Node599`

Obtaining branch lengths and nucleotide substitution biases under the nucleotide GTR model
* Log(L) = -6510.63, AIC-c = 14388.84 (683 estimated parameters)

Fitting the baseline model with a single dN/dS class per branch, and no site-to-site variation.
* Log(L) = -5978.34, AIC-c = 14703.71 (1364 estimated parameters)
* Branch-level non-synonymous/synonymous rate ratio distribution has median 0.21, and 95% of the weight in 0.10 - 10000000000.00

Determining the optimal number of rate classes per branch using a step up procedure

Branch	Length	Rates	Max. dN/dS	Log(L)	AIC-c	Best AIC-c so far
USA_M10989_1985	0.00	2	117.48 (1.54%)	-5975.45	14702.00	14702.00
USA_M10989_1985	0.00	3	>1000 (0.27%)	-5974.96	14705.06	14702.00
VAC_JN625220_INDIA_vac2_2011	0.00	2	2.22 (4.91%)	-5975.43	14706.02	14702.00
KOR_EF599098_2c_Pome_2006	0.00	2	0.25 (23.61%)	-5975.45	14706.06	14702.00
CHI_KF803600_2010_BJ_A68_2010	0.00	2	451.85 (0.35%)	-5968.13	14691.42	14691.42
CHI_KF803600_2010_BJ_A68_2010	0.00	3	530.66 (0.31%)	-5968.12	14695.44	14691.42
VAC_FJ011098_Intervet_2006	0.00	2	0.27 (49.77%)	-5968.13	14695.48	14691.42
VAC_KY083090_Singapore_2016	0.00	2	>1000 (0.57%)	-5956.37	14671.94	14671.94
VAC_KY083090_Singapore_2016	0.00	3	>1000 (0.56%)	-5956.37	14676.00	14671.94
ITA_FJ005264_134_2005	0.00	2	0.00 (99.29%)	-5956.37	14676.00	14671.94
RUS_JN033694_Laika_1993	0.00	2	430.60 (0.19%)	-5949.10	14661.47	14661.47
RUS_JN033694_Laika_1993	0.00	3	431.12 (0.19%)	-5949.10	14665.52	14661.47
CHI_FJ231389_FPV_monkey_BJ_22_20...	0.00	2	0.75 (9.85%)	-5949.10	14665.52	14661.47
POR_KU662350_greywolf_W52_2005	0.00	2	>1000 (15.78%)	-5949.11	14665.55	14661.47
VAC_EU914139_Pfizer_2006	0.00	2	>1000 (0.59%)	-5942.71	14652.74	14652.74
VAC_EU914139_Pfizer_2006	0.00	3	>1000 (0.52%)	-5942.71	14656.79	14652.74
GER_FJ005261_G162_1997	0.00	2	0.00 (98.04%)	-5942.71	14656.80	14652.74

Node311	0.00	2	0.00 (98.04%)	-5942.71	14656.80	14652.74
NZE_AY742933_339_1993	0.00	2	0.25 (0.00%)	-5942.71	14656.80	14652.74
GER_FJ005260_G82_1997	0.00	2	0.00 (98.61%)	-5942.71	14656.80	14652.74
FRA_DQ025952_2a_03B12_2003	0.00	2	0.25 (0.00%)	-5942.71	14656.80	14652.74
IND_KX469432_newCPV_2b_Hiller_20...	0.00	2	0.25 (0.00%)	-5942.71	14656.80	14652.74
FRA_DQ025993_2a_04S24_2004	0.00	2	0.00 (98.04%)	-5942.71	14656.80	14652.74
CHI_GQ169553_Vac2_2007	0.00	2	0.88 (9.36%)	-5942.72	14656.81	14652.74
VAC_FJ011097_Merial_2006	0.00	2	0.99 (10.01%)	-5942.71	14656.80	14652.74
VAC_FJ222823_2b_29_1997	0.00	2	0.99 (10.10%)	-5942.71	14656.80	14652.74
CHI_GU569946_2a_JL0201_2002	0.00	2	0.98 (9.92%)	-5942.72	14656.81	14652.74
Node640	0.00	2	1.01 (9.93%)	-5942.71	14656.80	14652.74
VIE_AB054224_2c_leopard_V203_200...	0.00	2	0.98 (9.75%)	-5942.72	14656.81	14652.74
CHI_GU392237_fox_HB2_2009	0.00	2	0.98 (10.20%)	-5942.71	14656.80	14652.74
ECU_KF149971_2c_ME32_2012	0.00	2	1.02 (9.66%)	-5942.72	14656.81	14652.74
VIE_AB054217_2a_cat_V154_2000	0.00	2	1.00 (10.22%)	-5942.71	14656.80	14652.74
ITA_GU362932_cat11_2008	0.00	2	0.74 (5.10%)	-5942.71	14656.80	14652.74
BRA_DQ340410_2a_BR315_1986	0.00	2	226.24 (0.23%)	-5937.11	14645.59	14645.59
BRA_DQ340410_2a_BR315_1986	0.00	3	209.02 (0.24%)	-5937.11	14649.65	14645.59
VIE_AB120721_2b_HCM_8_2003	0.00	2	0.74 (5.05%)	-5937.11	14649.65	14645.59
Node356	0.00	2	>1000 (0.15%)	-5927.49	14630.41	14630.41
Node356	0.00	3	797.28 (0.19%)	-5927.58	14634.66	14630.41
HUN_KF539804_H_212_2012	0.00	2	0.50 (25.00%)	-5927.49	14634.47	14630.41
CHI_KJ170680_raccoondog_HLJ11_1_...	0.00	2	>1000 (0.61%)	-5922.78	14625.06	14625.06
CHI_KJ170680_raccoondog_HLJ11_1_...	0.00	3	>1000 (0.49%)	-5922.77	14629.08	14625.06
Node599	0.00	2	57.47 (45.66%)	-5922.81	14629.17	14625.06
Node562	0.00	2	50.93 (56.45%)	-5922.81	14629.16	14625.06
FIN_U22193_raccoondog_RD87_1987	0.00	2	0.00 (98.04%)	-5922.78	14629.11	14625.06

JPN_D26079_1993	0.00	2	0.00 (98.30%)	-5922.78	14629.11	14625.06
THA_FJ869128_KU11_2004	0.00	2	0.00 (98.30%)	-5922.78	14629.11	14625.06
Node123	0.00	2	0.00 (98.04%)	-5922.78	14629.11	14625.06
POR_KT275253_2c_PT036_12_2012	0.00	2	0.00 (98.04%)	-5922.78	14629.11	14625.06
ITA_FJ005265_140_2005	0.00	2	0.25 (0.00%)	-5922.78	14629.11	14625.06
VAC_JN625219_INDIA_vac1_2011	0.00	2	0.25 (0.00%)	-5922.78	14629.11	14625.06
THA_FJ869122_KU1_2008	0.00	2	0.25 (0.00%)	-5922.78	14629.11	14625.06
TAW_FJ265775_CPV301_2004	0.00	2	0.00 (98.30%)	-5922.78	14629.11	14625.06
ITA_KX434460_52238_12_2012	0.00	2	0.00 (98.04%)	-5922.78	14629.11	14625.06
FRA_DQ025992_2b_04S23_2004	0.00	2	0.00 (98.04%)	-5922.78	14629.11	14625.06
CHI_EU145954_2b_BJ044_2007	0.00	2	0.25 (0.00%)	-5922.78	14629.11	14625.06
VIE_AB054220_2b_cat_V217_2000	0.00	2	0.25 (0.00%)	-5922.78	14629.11	14625.06
Node223	0.00	2	0.00 (98.30%)	-5922.78	14629.11	14625.06
BRA_DQ340411_2a_BR8_1990	0.00	2	0.25 (0.00%)	-5922.78	14629.11	14625.06
USA_AY742953_435_2003	0.00	2	0.25 (0.00%)	-5922.78	14629.11	14625.06
USA_KJ813854_Puma_ND_F205_2013	0.00	2	0.00 (98.04%)	-5922.78	14629.11	14625.06
POR_KT275252_2c_PT013_12_2012	0.00	2	0.00 (98.04%)	-5922.78	14629.11	14625.06
URU_KM457131_2c_UY368_2011	0.00	2	0.25 (0.00%)	-5922.78	14629.11	14625.06
URU_KC196107_2c_M129_2008	0.00	2	0.25 (0.00%)	-5922.78	14629.11	14625.06
ARG_JF414819_Arg35_2008	0.00	2	0.00 (98.04%)	-5922.78	14629.11	14625.06
Node575	0.00	2	1.00 (100.00%)	-5923.21	14629.97	14625.06
ITA_FJ005231_406_2006	0.00	2	0.25 (0.00%)	-5922.78	14629.11	14625.06
ITA_FJ005232_411_2006	0.00	2	0.00 (98.04%)	-5922.78	14629.11	14625.06
JPN_LC270891_2b_9985_2017	0.00	2	0.25 (0.00%)	-5922.78	14629.11	14625.06
Node230	0.00	2	0.25 (0.00%)	-5922.78	14629.11	14625.06
USA_EU659118_CPV_13_1981	0.00	2	0.25 (0.00%)	-5922.78	14629.11	14625.06
Node437	0.00	2	0.25 (0.00%)	-5922.78	14629.11	14625.06

```
|       ITA_FJ005252_96_2002        |   0.00   |    2     |    0.00
(98.30%)   |   -5922.78    |   14629.11     |    14625.06     |
|USA_KJ813828_Fisher_F1F010712_20...|   0.00   |    2     |    0.00
(98.30%)   |   -5922.78    |   14629.11     |    14625.06     |
|       HUN_KF539795_H_8_2012       |   0.00   |    2     |    0.25
( 0.00%)   |   -5922.78    |   14629.11     |    14625.06     |
|       ITA_FJ005255_333_2005       |   0.00   |    2     |    0.25
( 0.00%)   |   -5922.78    |   14629.11     |    14625.06     |
|      HUN_KF539799_H_39_2012       |   0.00   |    2     |    0.25
( 0.00%)   |   -5922.78    |   14629.11     |    14625.06     |
|USA_KJ813881_Graywolf_MI_832_201...|   0.00   |    2     |    0.00
(98.30%)   |   -5922.78    |   14629.11     |    14625.06     |
|   VIE_AB054215_2a_cat_V120_2000   |   0.00   |    2     |    0.00
(98.04%)   |   -5922.78    |   14629.11     |    14625.06     |
|              Node600              |   0.00   |    2     |    0.25
(10.07%)   |   -5922.78    |   14629.11     |    14625.06     |
|              Node604              |   0.00   |    2     |    0.51
( 1.93%)   |   -5922.78    |   14629.11     |    14625.06     |
|    JPN_AB437434_1887_f_3_2008     |   0.00   |    2     |    0.52
( 2.07%)   |   -5922.78    |   14629.11     |    14625.06     |
|   VAC_JN625224_INDIA_vac6_2011    |   0.00   |    2     |    0.48
( 2.06%)   |   -5922.78    |   14629.11     |    14625.06     |
|USA_KJ813843_Bobcat_ND_1160_2013...|   0.00   |    2     |    0.50
( 2.33%)   |   -5922.78    |   14629.11     |    14625.06     |
|              Node147              |   0.00   |    2     |    0.49
( 2.12%)   |   -5922.78    |   14629.11     |    14625.06     |
|              Node353              |   0.00   |    2     |  436.50
( 0.20%)   |   -5922.14    |   14627.83     |    14625.06     |
|      USA_JX475279_TN_1_2011       |   0.00   |    2     |    0.54
( 1.90%)   |   -5922.78    |   14629.11     |    14625.06     |
|              Node502              |   0.00   |    2     |    0.68
( 2.03%)   |   -5922.78    |   14629.11     |    14625.06     |
|   POR_KT275255_2c_PT238_14_2014   |   0.00   |    2     |  436.47
( 0.20%)   |   -5922.14    |   14627.83     |    14625.06     |
|     URU_KM457103_2c_UY12_2006     |   0.00   |    2     |    0.73
( 1.82%)   |   -5922.78    |   14629.11     |    14625.06     |
|              Node324              |   0.00   |    2     |    0.68
( 1.89%)   |   -5922.78    |   14629.11     |    14625.06     |
|              Node60               |   0.00   |    2     |    0.69
( 1.91%)   |   -5922.78    |   14629.11     |    14625.06     |
|CHI_GU392244_raccoondog_HB7_2009...|   0.00   |    2     |    0.25
(25.07%)   |   -5922.78    |   14629.11     |    14625.06     |
|              Node138              |   0.00   |    2     |    0.25
(21.87%)   |   -5922.78    |   14629.11     |    14625.06     |
|    CHI_GQ857605_CPV07_03_2007     |   0.00   |    2     |    0.25
(24.93%)   |   -5922.78    |   14629.11     |    14625.06     |
|VIE_AB054223_2c_leopard_V140_200...|   0.00   |    2     |    0.24
(24.91%)   |   -5922.78    |   14629.11     |    14625.06     |
|    FRA_DQ025982_2a_04S13_2004     |   0.00   |    2     |    0.25
(25.07%)   |   -5922.78    |   14629.11     |    14625.06     |
|    HRV_KP859577_2c_HR856_2014     |   0.00   |    2     |   1.00
(100.00%)   |   -5923.17    |   14629.88     |    14625.06     |
|    URU_KM457126_2c_UY318_2010     |   0.00   |    2     |   >1000
(11.08%)   |   -5922.80    |   14629.15     |    14625.06     |
```

HUN_KF539797_H_11_2012	0.00	2	1.00 (100.00%)	-5923.17	14629.88	14625.06
ITA_FJ005253_67_2005	0.00	2	1.00 (100.00%)	-5923.17	14629.88	14625.06
Node299	0.00	2	1.00 (100.00%)	-5923.16	14629.87	14625.06
Node637	0.00	2	62.57 (24.42%)	-5922.81	14629.16	14625.06
CHI_GU392242_raccoondog_HB10_200...	0.00	2	1.00 (100.00%)	-5923.17	14629.88	14625.06
VAC_JN625221_INDIA_vac3_2011	0.00	2	1.00 (100.00%)	-5923.16	14629.87	14625.06
Node671	0.00	2	1.00 (100.00%)	-5923.16	14629.87	14625.06
Node663	0.00	2	1.00 (100.00%)	-5923.16	14629.87	14625.06
VAC_JN625222_INDIA_vac4_2011	0.00	2	1.00 (100.00%)	-5923.16	14629.87	14625.06
VAC_GU212790_primodog_2009	0.00	2	57.07 (32.93%)	-5922.81	14629.16	14625.06
Node578	0.00	2	1.00 (100.00%)	-5923.11	14629.77	14625.06
Node3	0.00	2	68.61 (28.96%)	-5922.80	14629.14	14625.06
USA_KJ813832_Fisher_ND_14_2013	0.00	2	0.00 (99.90%)	-5922.78	14629.11	14625.06
USA_KJ813835_Fisher_ND_19_2013	0.00	2	0.25 (0.00%)	-5922.78	14629.11	14625.06
Node667	0.00	2	0.25 (0.00%)	-5922.78	14629.11	14625.06
USA_U22896_cat_1990	0.00	2	0.00 (98.05%)	-5922.78	14629.11	14625.06
Node130	0.00	2	0.00 (98.04%)	-5922.78	14629.11	14625.06
USA_JX475248_CO_1102_2011	0.00	2	0.25 (0.00%)	-5922.78	14629.11	14625.06
Node582	0.00	2	0.25 (0.00%)	-5922.78	14629.11	14625.06
Node585	0.00	2	0.00 (98.04%)	-5922.78	14629.11	14625.06
Node208	0.00	2	0.00 (98.05%)	-5922.78	14629.11	14625.06
Node636	0.00	2	0.25 (0.00%)	-5922.78	14629.11	14625.06
Node661	0.00	2	0.25 (0.00%)	-5922.78	14629.11	14625.06
Node319	0.00	2	0.00 (98.03%)	-5922.78	14629.11	14625.06
USA_M24000_FPV_CPV_31_1988	0.00	2	0.00 (98.04%)	-5922.78	14629.11	14625.06
Node342	0.00	2	0.25 (0.00%)	-5922.78	14629.11	14625.06
Node548	0.00	2	0.00 (98.04%)	-5922.78	14629.11	14625.06

```
|     CHI_GU569943_YB8301_1983       |   0.00   |    2     |     0.25
( 0.00%)   |   -5922.78    |   14629.11     |    14625.06     |
|USA_JN867610_Raccoon_VA_118_A_20...|   0.00   |    2     |     0.25
( 0.00%)   |   -5922.78    |   14629.11     |    14625.06     |
|               Node348              |   0.00   |    2     |     0.00
(98.05%)   |   -5922.78    |   14629.11     |    14625.06     |
|    VIE_AB120720_2b_HCM_6_2003      |   0.00   |    2     |     0.00
(88.40%)   |   -5922.78    |   14629.11     |    14625.06     |
|               Node580              |   0.00   |    2     |     0.25
( 0.00%)   |   -5922.78    |   14629.11     |    14625.06     |
|    BRA_DQ340409_2b_BR183_1985      |   0.00   |    2     |     0.00
(98.03%)   |   -5922.78    |   14629.11     |    14625.06     |
|               Node214              |   0.00   |    2     |     0.00
(98.05%)   |   -5922.78    |   14629.11     |    14625.06     |
|    VIE_AB120723_2b_HCM_23_2003     |   0.00   |    2     |     0.25
( 0.00%)   |   -5922.78    |   14629.11     |    14625.06     |
|               Node287              |   0.00   |    2     |     0.25
( 0.00%)   |   -5922.78    |   14629.11     |    14625.06     |
|  USA_KJ813870_Raccoon_TX_1_2013    |   0.00   |    2     |     0.25
( 0.00%)   |   -5922.78    |   14629.11     |    14625.06     |
|     USA_JX475234_ME_258_2011       |   0.00   |    2     |     0.25
( 0.00%)   |   -5922.78    |   14629.11     |    14625.06     |
|    ITA_KX434456_45361_09_2009      |   0.00   |    2     |     0.00
(98.05%)   |   -5922.78    |   14629.11     |    14625.06     |
|      FRA_DQ025964_03C8_2003        |   0.00   |    2     |     0.00
(98.04%)   |   -5922.78    |   14629.11     |    14625.06     |
|               Node12               |   0.00   |    2     |     0.25
( 0.00%)   |   -5922.78    |   14629.11     |    14625.06     |
|     USA_JX475246_CO_2503_2010      |   0.00   |    2     |     0.25
( 0.00%)   |   -5922.78    |   14629.11     |    14625.06     |
|    JPN_AB115504_2c_97_008_1997     |   0.00   |    2     |     0.25
( 0.00%)   |   -5922.78    |   14629.11     |    14625.06     |
|    USA_JX475233_SC_182_A_2011      |   0.00   |    2     |     0.00
(98.04%)   |   -5922.78    |   14629.11     |    14625.06     |
|               Node104              |   0.00   |    2     |     0.00
(98.04%)   |   -5922.78    |   14629.11     |    14625.06     |
|               Node113              |   0.00   |    2     |     0.00
(98.04%)   |   -5922.78    |   14629.11     |    14625.06     |
|     USA_JX475273_MT_909_2012       |   0.00   |    2     |     0.00
(98.04%)   |   -5922.78    |   14629.11     |    14625.06     |
|      FRA_DQ025969_03S5_2003        |   0.00   |    2     |     0.25
( 0.00%)   |   -5922.78    |   14629.11     |    14625.06     |
|    BRA_DQ340419_2a_BR570_1992      |   0.00   |    2     |     0.25
( 0.00%)   |   -5922.78    |   14629.11     |    14625.06     |
|     ECU_KF149984_2c_ME28_2012      |   0.00   |    2     |     0.00
(98.04%)   |   -5922.78    |   14629.11     |    14625.06     |
|      FRA_DQ025975_04S6_2004        |   0.00   |    2     |     0.00
(98.04%)   |   -5922.78    |   14629.11     |    14625.06     |
|      FRA_DQ025976_04S7_2004        |   0.00   |    2     |     0.00
(98.04%)   |   -5922.78    |   14629.11     |    14625.06     |
|      FRA_DQ025965_03C9_2003        |   0.00   |    2     |     0.00
(98.04%)   |   -5922.78    |   14629.11     |    14625.06     |
|     URU_KC196085_2c_M57_2007       |   0.00   |    2     |     0.25
( 0.00%)   |   -5922.78    |   14629.11     |    14625.06     |
```

```
|      USA_JX475239_GA_06_2011       |   0.00   |    2     |    0.25
( 0.00%)   |   -5922.78    |   14629.11    |    14625.06     |
|     ITA_FJ005212_2c_349_2004       |   0.00   |    2     |    0.00
(98.04%)   |   -5922.78    |   14629.11    |    14625.06     |
|              Node364               |   0.00   |    2     |    0.00
(98.04%)   |   -5922.78    |   14629.11    |    14625.06     |
|    VIE_AB120722_2b_HCM_18_2003     |   0.00   |    2     |    0.25
( 0.00%)   |   -5922.78    |   14629.11    |    14625.06     |
|              Node563               |   0.00   |    2     |    0.25
( 0.00%)   |   -5922.78    |   14629.11    |    14625.06     |
|              Node229               |   0.00   |    2     |    0.00
(98.05%)   |   -5922.78    |   14629.11    |    14625.06     |
|USA_JN867611_Raccoon_KY_358_B_20...|   0.00   |    2     |    0.00
(98.04%)   |   -5922.78    |   14629.11    |    14625.06     |
|              Node392               |   0.00   |    2     |    0.25
( 0.00%)   |   -5922.78    |   14629.11    |    14625.06     |
|      HUN_KF539801_H_25_2012        |   0.00   |    2     |    0.25
( 0.00%)   |   -5922.78    |   14629.11    |    14625.06     |
|              Node387               |   0.00   |    2     |    0.00
(98.05%)   |   -5922.78    |   14629.11    |    14625.06     |
|              Node217               |   0.00   |    2     |    0.00
(98.05%)   |   -5922.78    |   14629.11    |    14625.06     |
|    JPN_AB437433_1887_M_2_2008      |   0.00   |    2     |    0.00
(98.04%)   |   -5922.78    |   14629.11    |    14625.06     |
|      THA_FJ869139_KU66_2003        |   0.00   |    2     |    0.25
( 0.00%)   |   -5922.78    |   14629.11    |    14625.06     |
|    CHI_GQ857596_CPV05_01_2005      |   0.00   |    2     |    0.00
(98.15%)   |   -5922.78    |   14629.11    |    14625.06     |
|              Node349               |   0.00   |    2     |    0.00
(98.04%)   |   -5922.78    |   14629.11    |    14625.06     |
|    BRA_DQ340421_2a_BR597_1992      |   0.00   |    2     |    0.25
( 0.00%)   |   -5922.78    |   14629.11    |    14625.06     |
|     BRA_DQ340413_2a_BR18_1990      |   0.00   |    2     |    0.25
( 0.00%)   |   -5922.78    |   14629.11    |    14625.06     |
|              Node165               |   0.00   |    2     |    0.00
(98.04%)   |   -5922.78    |   14629.11    |    14625.06     |
|              Node100               |   0.00   |    2     |    0.00
(98.04%)   |   -5922.78    |   14629.11    |    14625.06     |
|              Node315               |   0.00   |    2     |    0.25
( 0.00%)   |   -5922.78    |   14629.11    |    14625.06     |
|              Node511               |   0.00   |    2     |    0.25
( 0.00%)   |   -5922.78    |   14629.11    |    14625.06     |
|              Node221               |   0.00   |    2     |    0.00
(98.03%)   |   -5922.78    |   14629.11    |    14625.06     |
|              Node542               |   0.00   |    2     |    0.25
( 0.00%)   |   -5922.78    |   14629.11    |    14625.06     |
|              Node478               |   0.00   |    2     |    0.25
( 0.00%)   |   -5922.78    |   14629.11    |    14625.06     |
|              Node517               |   0.00   |    2     |    0.00
(98.04%)   |   -5922.78    |   14629.11    |    14625.06     |
|              Node317               |   0.00   |    2     |    0.00
(98.03%)   |   -5922.78    |   14629.11    |    14625.06     |
|              Node159               |   0.00   |    2     |    0.00
(98.04%)   |   -5922.78    |   14629.11    |    14625.06     |
```

```
|      TWN_EF592511_TWN1_2006       |   0.00   |    2     |    0.25
( 0.00%)   |   -5922.78    |   14629.11    |    14625.06     |
|    BRA_DQ340423_2a_BR136_1993     |   0.00   |    2     |    0.25
( 0.00%)   |   -5922.78    |   14629.11    |    14625.06     |
|       ITA_FJ005257_54_2008        |   0.00   |    2     |    0.25
( 0.00%)   |   -5922.78    |   14629.11    |    14625.06     |
|    KOR_EF599097_2b_DH326_2006     |   0.00   |    2     |    0.00
(98.03%)   |   -5922.78    |   14629.11    |    14625.06     |
|     USA_JX475260_CO_704_2010      |   0.00   |    2     |    0.00
(98.04%)   |   -5922.78    |   14629.11    |    14625.06     |
|              Node328              |   0.00   |    2     |    0.25
( 0.00%)   |   -5922.78    |   14629.11    |    14625.06     |
|      FRA_DQ025960_03C4_2003       |   0.00   |    2     |    0.00
(98.04%)   |   -5922.78    |   14629.11    |    14625.06     |
|      GER_FJ005196_2c_G7_1997      |   0.00   |    2     |    0.00
(98.04%)   |   -5922.78    |   14629.11    |    14625.06     |
|    CHI_GQ857601_CPV06_02_2006     |   0.00   |    2     |    0.25
( 0.00%)   |   -5922.78    |   14629.11    |    14625.06     |
|CHI_GU392241_raccoondog_HB1_2009...|   0.00   |    2     |    0.25
( 0.00%)   |   -5922.78    |   14629.11    |    14625.06     |
|     GER_FJ005199_2c_G172_1997     |   0.00   |    2     |    0.00
(98.04%)   |   -5922.78    |   14629.11    |    14625.06     |
|    USA_JX475243_ID_22772_2009     |   0.00   |    2     |    0.00
(98.04%)   |   -5922.78    |   14629.11    |    14625.06     |
|      FRA_DQ025951_03B10_2003      |   0.00   |    2     |    0.25
( 0.00%)   |   -5922.78    |   14629.11    |    14625.06     |
|       ITA_FJ005247_195_2008       |   0.00   |    2     |    0.25
( 0.00%)   |   -5922.78    |   14629.11    |    14625.06     |
|              Node120              |   0.00   |    2     |    0.00
(98.04%)   |   -5922.78    |   14629.11    |    14625.06     |
|     USA_JX475252_CO_1316_2010     |   0.00   |    2     |    0.00
(98.04%)   |   -5922.78    |   14629.11    |    14625.06     |
|   USA_KJ813858_Puma_ND_F93_2013   |   0.00   |    2     |    0.25
( 0.00%)   |   -5922.78    |   14629.11    |    14625.06     |
|     ITA_FJ005218_2c_330_2006      |   0.00   |    2     |    0.25
( 0.00%)   |   -5922.78    |   14629.11    |    14625.06     |
|              Node57               |   0.00   |    2     |    0.25
( 0.00%)   |   -5922.78    |   14629.11    |    14625.06     |
|      FRA_DQ025994_04S25_2004      |   0.00   |    2     |    0.00
(98.04%)   |   -5922.78    |   14629.11    |    14625.06     |
|     ITA_FJ005216_2c_284_2006      |   0.00   |    2     |    0.00
(98.04%)   |   -5922.78    |   14629.11    |    14625.06     |
|              Node84               |   0.00   |    2     |    0.00
(98.04%)   |   -5922.78    |   14629.11    |    14625.06     |
|              Node91               |   0.00   |    2     |    0.25
( 0.00%)   |   -5922.78    |   14629.11    |    14625.06     |
|              Node53               |   0.00   |    2     |    0.25
( 0.00%)   |   -5922.78    |   14629.11    |    14625.06     |
|   ITA_KX434459_27692_1_11_2011    |   0.00   |    2     |    0.00
(98.04%)   |   -5922.78    |   14629.11    |    14625.06     |
|      FRA_DQ025985_04S16_2004      |   0.00   |    2     |    0.25
( 0.00%)   |   -5922.78    |   14629.11    |    14625.06     |
|    FRA_DQ026002_2a_04S33_2004     |   0.00   |    2     |    0.25
( 0.00%)   |   -5922.78    |   14629.11    |    14625.06     |
```

CHI_KF803615_2011_BJ_B25_2011	0.00	2	0.25 (0.00%)	-5922.78	14629.11	14625.06
Node451	0.00	2	0.25 (0.00%)	-5922.78	14629.11	14625.06
Node497	0.00	2	0.25 (0.00%)	-5922.78	14629.11	14625.06
NIG_HQ602995_15_10_2010	0.00	2	0.00 (98.02%)	-5922.78	14629.11	14625.06
GER_AY742934_447_1995	0.00	2	0.00 (98.02%)	-5922.78	14629.11	14625.06
Node448	0.00	2	0.25 (0.00%)	-5922.78	14629.11	14625.06
USA_KJ813892_Coyote_AK_218_2013	0.00	2	0.25 (0.00%)	-5922.78	14629.11	14625.06
ITA_AF306446_584_2000	0.00	2	0.25 (0.00%)	-5922.78	14629.11	14625.06
USA_JN867604_Dog_IL_137654_2008	0.00	2	0.25 (0.00%)	-5922.78	14629.11	14625.06
Node398	0.00	2	0.25 (0.00%)	-5922.78	14629.11	14625.06
NIG_HQ602992_19_10_2010	0.00	2	0.25 (0.00%)	-5922.78	14629.11	14625.06
Node335	0.00	2	0.00 (98.02%)	-5922.78	14629.11	14625.06
HUN_KF539800_H_27_2012	0.00	2	0.00 (98.04%)	-5922.78	14629.11	14625.06
HUN_KF539796_H_9_2012	0.00	2	0.25 (0.00%)	-5922.78	14629.11	14625.06
KOR_EF599096_DH426_2005	0.00	2	0.25 (0.00%)	-5922.78	14629.11	14625.06
Node331	0.00	2	0.00 (98.03%)	-5922.78	14629.11	14625.06
Node481	0.00	2	0.25 (0.00%)	-5922.78	14629.11	14625.06
ITA_FJ005214_2c_67_2006	0.00	2	0.00 (98.04%)	-5922.78	14629.11	14625.06
Node492	0.00	2	0.25 (0.00%)	-5922.78	14629.11	14625.06
BRA_KY073269_UFMT_2015	0.00	2	0.00 (98.04%)	-5922.78	14629.11	14625.06
ITA_FJ005251_239_2008	0.00	2	0.00 (98.04%)	-5922.78	14629.11	14625.06
ARG_KM236569_Cuba_2013	0.00	2	0.25 (0.00%)	-5922.78	14629.11	14625.06
Node210	0.00	2	0.25 (0.00%)	-5922.78	14629.11	14625.06
ARG_JF414818_Arg32_2008	0.00	2	0.00 (98.04%)	-5922.78	14629.11	14625.06
Node457	0.00	2	0.25 (0.00%)	-5922.78	14629.11	14625.06
ECU_KF149963_2c_ME10_2012	0.00	2	0.25 (0.00%)	-5922.78	14629.11	14625.06
ECU_KF149964_2c_ME23_2012	0.00	2	0.00 (98.04%)	-5922.78	14629.11	14625.06

```
|             Node254            |   0.00   |    2     |    0.25
( 0.00%)   |   -5922.78    |   14629.11    |    14625.06     |
|    USA_JX475242_WI_18268_2002    |   0.00   |    2     |    0.25
( 0.00%)   |   -5922.78    |   14629.11    |    14625.06     |
|     ECU_KF149969_2c_ME31_2012    |   0.00   |    2     |    0.00
(98.04%)   |   -5922.78    |   14629.11    |    14625.06     |
|             Node423            |   0.00   |    2     |    0.25
( 0.00%)   |   -5922.78    |   14629.11    |    14625.06     |
|     FRA_DQ025961_2b_03C5_2003    |   0.00   |    2     |    0.25
( 0.00%)   |   -5922.78    |   14629.11    |    14625.06     |
|     FRA_DQ025947_2a_02B5_2002    |   0.00   |    2     |    0.00
(98.04%)   |   -5922.78    |   14629.11    |    14625.06     |
|USA_KJ813852_Bobcat_ND_1170_2013...|   0.00   |    2     |    0.00
(98.03%)   |   -5922.78    |   14629.11    |    14625.06     |
|     FRA_DQ025943_2a_01S1_2001    |   0.00   |    2     |    0.25
( 0.00%)   |   -5922.78    |   14629.11    |    14625.06     |
|             Node399            |   0.00   |    2     |    0.25
( 0.00%)   |   -5922.78    |   14629.11    |    14625.06     |
|    CHI_GQ857612_CPV08_04_2008    |   0.00   |    2     |    0.25
( 0.00%)   |   -5922.78    |   14629.11    |    14625.06     |
|    CHI_GQ857609_CPV08_01_2008    |   0.00   |    2     |    0.00
(98.03%)   |   -5922.78    |   14629.11    |    14625.06     |
|     ITA_KF373577_2a_714_2001     |   0.00   |    2     |    0.25
( 0.00%)   |   -5922.78    |   14629.11    |    14625.06     |
|    FRA_DQ026001_2a_04S32_2004    |   0.00   |    2     |    0.25
( 0.00%)   |   -5922.78    |   14629.11    |    14625.06     |
|     CHI_EU483515_2b_ZD13_2007    |   0.00   |    2     |    0.00
(98.02%)   |   -5922.78    |   14629.11    |    14625.06     |
|    VAC_GU212791_vanguard_2009    |   0.00   |    2     |    0.25
( 0.00%)   |   -5922.78    |   14629.11    |    14625.06     |
|             Node357            |   0.00   |    2     |    0.25
( 0.00%)   |   -5922.78    |   14629.11    |    14625.06     |
|  USA_KJ813842_Bobcat_ND_502_2013  |   0.00   |    2     |    0.00
(98.03%)   |   -5922.78    |   14629.11    |    14625.06     |
|     USA_JX475250_CO_728_2010     |   0.00   |    2     |    0.00
(98.02%)   |   -5922.78    |   14629.11    |    14625.06     |
|    HRV_KP859578_2c_HR859_2014    |   0.00   |    2     |    0.00
(98.02%)   |   -5922.78    |   14629.11    |    14625.06     |
|             Node418            |   0.00   |    2     |    0.25
( 0.00%)   |   -5922.78    |   14629.11    |    14625.06     |
|     USA_JX475278_AR_1069_2012    |   0.00   |    2     |    0.25
( 0.00%)   |   -5922.78    |   14629.11    |    14625.06     |
|     USA_JX475251_CO_2235_2009    |   0.00   |    2     |    0.00
(98.04%)   |   -5922.78    |   14629.11    |    14625.06     |
|     USA_JX475247_CO_1246_2010    |   0.00   |    2     |    0.25
( 0.00%)   |   -5922.78    |   14629.11    |    14625.06     |
|USA_KJ813882_Raccoon_NJ_1423_201...|   0.00   |    2     |    0.00
(98.02%)   |   -5922.78    |   14629.11    |    14625.06     |
|USA_JN867603_2b_Dog_KS_81213_200...|   0.00   |    2     |    0.25
( 0.00%)   |   -5922.78    |   14629.11    |    14625.06     |
|USA_JN867602_2b_Dog_CA_148743_20...|   0.00   |    2     |    0.25
( 0.00%)   |   -5922.78    |   14629.11    |    14625.06     |
|    CHI_GQ857599_CPV05_04_2005    |   0.00   |    2     |    0.25
( 0.00%)   |   -5922.78    |   14629.11    |    14625.06     |
```

```
|     FRA_DQ025962_2a_03C6_2003     |   0.00   |    2     |    0.25
( 0.00%)   |   -5922.78    |   14629.11    |    14625.06     |
|      HUN_KF539798_H_31_2012       |   0.00   |    2     |    0.25
( 0.00%)   |   -5922.78    |   14629.11    |    14625.06     |
|     FRA_DQ025958_2a_03C2_2003     |   0.00   |    2     |    0.25
( 0.00%)   |   -5922.78    |   14629.11    |    14625.06     |
|USA_KJ813851_Bobcat_ND_1168_2013...|   0.00   |    2     |    0.00
(98.03%)   |   -5922.78    |   14629.11    |    14625.06     |
|     KOR_EU009205_2b_K029_2006     |   0.00   |    2     |    0.00
(98.04%)   |   -5922.78    |   14629.11    |    14625.06     |
|USA_KJ813873_Graywolf_MI_850_201...|   0.00   |    2     |    0.25
( 0.00%)   |   -5922.78    |   14629.11    |    14625.06     |
|      THA_KP715690_VT28_2014       |   0.00   |    2     |    0.25
( 0.00%)   |   -5922.78    |   14629.11    |    14625.06     |
|     ITA_KX434457_987_10_2010      |   0.00   |    2     |    0.25
( 0.00%)   |   -5922.78    |   14629.11    |    14625.06     |
|              Node174              |   0.00   |    2     |   1.00
(100.00%)   |   -5922.98    |   14629.50    |    14625.06     |
|  USA_KJ813844_Bobcat_ND_885_2013  |   0.00   |    2     |   62.46
(19.97%)   |   -5922.80    |   14629.15    |    14625.06     |
|ITA_KF385388_2a_Sicily_X83090_20...|   0.00   |    2     |  457.64
(80.11%)   |   -5922.79    |   14629.12    |    14625.06     |
|              Node268              |   0.00   |    2     |   97.81
( 6.60%)   |   -5922.80    |   14629.15    |    14625.06     |
|              Node281              |   0.00   |    2     |   59.85
(18.88%)   |   -5922.80    |   14629.15    |    14625.06     |
|              Node458              |   0.00   |    2     |  434.60
(80.01%)   |   -5922.78    |   14629.12    |    14625.06     |
|              Node275              |   0.00   |    2     |   1.00
(100.00%)   |   -5922.97    |   14629.49    |    14625.06     |
|              Node169              |   0.00   |    2     |   8.74
(100.00%)   |   -5922.81    |   14629.16    |    14625.06     |
|              Node625              |   0.00   |    2     |  494.56
(74.25%)   |   -5922.79    |   14629.12    |    14625.06     |
|USA_KJ813848_Bobcat_ND_1162_2013...|   0.00   |    2     |   1.00
(100.00%)   |   -5922.97    |   14629.50    |    14625.06     |
|     BRA_DQ340431_2a_BR56_1995     |   0.00   |    2     |   66.24
(17.96%)   |   -5922.80    |   14629.15    |    14625.06     |
|       ITA_FJ005240_208_2007       |   0.00   |    2     |   1.00
(100.00%)   |   -5922.97    |   14629.50    |    14625.06     |
|CHI_KJ194463_raccoondog_HeB10_3_...|   0.00   |    2     |  23.87
(100.00%)   |   -5922.79    |   14629.13    |    14625.06     |
|CHI_DQ354068_2a_redpanda_RPPV_20...|   0.00   |    2     |   79.95
(15.19%)   |   -5922.80    |   14629.15    |    14625.06     |
|              Node490              |   0.00   |    2     |   >1000
(13.08%)   |   -5922.79    |   14629.12    |    14625.06     |
|               Node4               |   0.00   |    2     |  412.94
(80.14%)   |   -5922.79    |   14629.12    |    14625.06     |
|   VIE_AB054219_2b_cat_V209_2000   |   0.00   |    2     |   1.00
(100.00%)   |   -5922.97    |   14629.49    |    14625.06     |
|     ITA_KF373592_2a_329_2008      |   0.00   |    2     |   >1000
(14.35%)   |   -5922.79    |   14629.12    |    14625.06     |
|    CHI_GU569948_2a_CC8601_1986    |   0.00   |    2     |  428.68
(80.24%)   |   -5922.79    |   14629.12    |    14625.06     |
```

CHI_GQ857600_CPV06_01_2006	0.00	2	1.00 (100.00%)	-5922.97	14629.50	14625.06
Node109	0.00	2	214.53 (58.87%)	-5922.79	14629.12	14625.06
URU_KM457104_2c_UY47_2006	0.00	2	808.49 (41.41%)	-5922.79	14629.12	14625.06
Node559	0.00	2	397.93 (80.24%)	-5922.79	14629.12	14625.06
Node11	0.00	2	66.65 (22.17%)	-5922.80	14629.15	14625.06
ITA_FJ005195_2c_136_2000	0.00	2	9.57 (100.00%)	-5922.81	14629.16	14625.06
HUN_KF539803_H_2_2012	0.00	2	23.87 (100.00%)	-5922.79	14629.13	14625.06
Node110	0.00	2	1.00 (100.00%)	-5922.97	14629.50	14625.06
ITA_FJ005226_383_2006	0.00	2	8.58 (100.00%)	-5922.81	14629.16	14625.06
FRA_DQ025954_03B14_2003	0.00	2	1.00 (100.00%)	-5922.97	14629.50	14625.06
Node8	0.00	2	398.99 (80.24%)	-5922.79	14629.12	14625.06
ARG_JF414821_Arg48_2009	0.00	2	1.00 (100.00%)	-5922.98	14629.50	14625.06
Node442	0.00	2	23.67 (100.00%)	-5922.79	14629.13	14625.06
BRA_DQ340422_2a_BR22_1993	0.00	2	399.93 (80.23%)	-5922.79	14629.12	14625.06
USA_EU659120_2b_CPV_411a_1998	0.00	2	1.00 (100.00%)	-5922.97	14629.49	14625.06
TAW_U72695_2a_T4_1996	0.00	2	42.70 (31.18%)	-5922.80	14629.15	14625.06
Node606	0.00	2	394.79 (80.10%)	-5922.79	14629.12	14625.06
VIE_AB054221_2b_leopard_V204_200...	0.00	2	72.21 (20.19%)	-5922.80	14629.15	14625.06
ITA_FJ005248_219_2008	0.00	2	399.62 (97.65%)	-5922.79	14629.12	14625.06
Node271	0.00	2	41.01 (29.51%)	-5922.80	14629.15	14625.06
THA_FJ869126_KU5_2008	0.00	2	13.18 (98.17%)	-5922.80	14629.15	14625.06
USA_KJ813846_Bobcat_ND_974_2013	0.00	2	1.00 (100.00%)	-5922.97	14629.49	14625.06
USA_KJ813890_Redfox_MA_197_2012	0.00	2	23.80 (100.00%)	-5922.79	14629.13	14625.06
FRA_DQ025986_2a_04S17_2004	0.00	2	76.48 (18.85%)	-5922.80	14629.15	14625.06
CHI_GU569939_2a_YN0202_2002	0.00	2	475.39 (24.49%)	-5922.79	14629.12	14625.06
CHI_GU392239_raccoondog_HB6_2009...	0.00	2	23.87 (100.00%)	-5922.79	14629.13	14625.06
VAC_FJ222822_2b_FortDodge_2008	0.00	2	1.00 (100.00%)	-5922.97	14629.49	14625.06

CHI_GU569940_2b_YN0203_2002	0.00	2	40.39 (25.98%)	-5922.80	14629.15	14625.06
Node431	0.00	2	449.47 (80.22%)	-5922.79	14629.12	14625.06
Node307	0.00	2	1.00 (100.00%)	-5922.97	14629.50	14625.06
Node303	0.00	2	64.29 (20.39%)	-5922.80	14629.15	14625.06
CHI_KJ170679_raccoondog_Heb10_2_...	0.00	2	403.43 (80.24%)	-5922.79	14629.12	14625.06
VAC_JN625223_INDIA_vac5_2011	0.00	2	1.00 (100.00%)	-5922.97	14629.50	14625.06
Node608	0.00	2	396.95 (80.26%)	-5922.79	14629.12	14625.06
USA_JN867605_2b_Dog_US_142805_20...	0.00	2	1.00 (100.00%)	-5922.97	14629.49	14625.06
AUS_KU508693_2c_LW_2015	0.00	2	9.62 (100.00%)	-5922.81	14629.16	14625.06
USA_M19296_CPV_N_1988	0.00	2	385.40 (80.14%)	-5922.79	14629.12	14625.06
Node658	0.00	2	364.52 (80.12%)	-5922.79	14629.12	14625.06
Node647	0.00	2	285.06 (82.01%)	-5922.79	14629.12	14625.06
Node666	0.00	2	1.00 (100.00%)	-5922.97	14629.49	14625.06
Node673	0.00	2	50.44 (26.21%)	-5922.80	14629.15	14625.06
Node631	0.00	2	1.00 (100.00%)	-5922.97	14629.49	14625.06
CHI_FJ432718_CPV_Cv_2008	0.00	2	63.31 (18.96%)	-5922.80	14629.15	14625.06
Node639	0.00	2	347.89 (80.61%)	-5922.79	14629.12	14625.06
Node623	0.00	2	1.00 (100.00%)	-5922.97	14629.49	14625.06
Node245	0.00	2	0.25 (2.00%)	-5922.78	14629.11	14625.06
Node243	0.00	2	0.25 (1.96%)	-5922.78	14629.11	14625.06
Node242	0.00	2	0.25 (1.96%)	-5922.78	14629.11	14625.06
Node244	0.00	2	0.25 (2.00%)	-5922.78	14629.11	14625.06
Node241	0.00	2	0.25 (1.96%)	-5922.78	14629.11	14625.06
Node320	0.00	2	0.25 (1.96%)	-5922.78	14629.11	14625.06
Node32	0.00	2	0.25 (1.96%)	-5922.78	14629.11	14625.06
Node337	0.00	2	0.25 (1.96%)	-5922.78	14629.11	14625.06
Node158	0.00	2	0.25 (1.96%)	-5922.78	14629.11	14625.06

```
|              Node333            |   0.00   |    2     |    0.25
( 1.96%)   |   -5922.78    |   14629.11    |    14625.06     |
|              Node339            |   0.00   |    2     |    0.25
( 1.96%)   |   -5922.78    |   14629.11    |    14625.06     |
|              Node162            |   0.00   |    2     |    0.25
( 1.96%)   |   -5922.78    |   14629.11    |    14625.06     |
|              Node16             |   0.00   |    2     |    0.25
( 1.96%)   |   -5922.78    |   14629.11    |    14625.06     |
|              Node33             |   0.00   |    2     |    0.25
( 1.96%)   |   -5922.78    |   14629.11    |    14625.06     |
|              Node300            |   0.00   |    2     |    0.25
( 1.96%)   |   -5922.78    |   14629.11    |    14625.06     |
|              Node239            |   0.00   |    2     |    0.25
( 1.96%)   |   -5922.78    |   14629.11    |    14625.06     |
|              Node240            |   0.00   |    2     |    0.25
( 2.00%)   |   -5922.78    |   14629.11    |    14625.06     |
|              Node24             |   0.00   |    2     |    0.25
( 1.96%)   |   -5922.78    |   14629.11    |    14625.06     |
|    HRV_KP859574_2c_HR442_2014    |   0.00   |    2     |    0.25
( 1.96%)   |   -5922.78    |   14629.11    |    14625.06     |
|              Node34             |   0.00   |    2     |    0.25
( 1.96%)   |   -5922.78    |   14629.11    |    14625.06     |
|       GER_AY742935_U6_1995       |   0.00   |    2     |    0.25
( 1.96%)   |   -5922.78    |   14629.11    |    14625.06     |
|              Node344            |   0.00   |    2     |    0.25
( 1.96%)   |   -5922.78    |   14629.11    |    14625.06     |
|              Node343            |   0.00   |    2     |    0.25
( 1.96%)   |   -5922.78    |   14629.11    |    14625.06     |
|              Node157            |   0.00   |    2     |    0.25
( 1.96%)   |   -5922.78    |   14629.11    |    14625.06     |
|              Node154            |   0.00   |    2     |    0.25
( 1.96%)   |   -5922.78    |   14629.11    |    14625.06     |
|              Node310            |   0.00   |    2     |    0.25
( 1.96%)   |   -5922.78    |   14629.11    |    14625.06     |
|              Node305            |   0.00   |    2     |    0.25
( 1.96%)   |   -5922.78    |   14629.11    |    14625.06     |
|              Node31             |   0.00   |    2     |    0.25
( 1.96%)   |   -5922.78    |   14629.11    |    14625.06     |
|              Node314            |   0.00   |    2     |    0.25
( 1.96%)   |   -5922.78    |   14629.11    |    14625.06     |
|              Node318            |   0.00   |    2     |    0.25
( 1.96%)   |   -5922.78    |   14629.11    |    14625.06     |
|              Node156            |   0.00   |    2     |    0.25
( 1.96%)   |   -5922.78    |   14629.11    |    14625.06     |
|              Node155            |   0.00   |    2     |    0.25
( 1.96%)   |   -5922.78    |   14629.11    |    14625.06     |
|              Node316            |   0.00   |    2     |    0.25
( 1.96%)   |   -5922.78    |   14629.11    |    14625.06     |
|              Node238            |   0.00   |    2     |    0.25
( 1.96%)   |   -5922.78    |   14629.11    |    14625.06     |
|              Node28             |   0.00   |    2     |    0.25
( 1.96%)   |   -5922.78    |   14629.11    |    14625.06     |
|              Node280            |   0.00   |    2     |    0.25
( 1.96%)   |   -5922.78    |   14629.11    |    14625.06     |
```

```
|              Node282              |  0.00   |   2     |   0.25
( 1.96%)   |   -5922.78     |   14629.11    |   14625.06     |
|              Node151              |  0.00   |   2     |   0.25
( 1.96%)   |   -5922.78     |   14629.11    |   14625.06     |
|              Node279              |  0.00   |   2     |   0.25
( 1.96%)   |   -5922.78     |   14629.11    |   14625.06     |
|              Node293              |  0.00   |   2     |   0.25
( 1.96%)   |   -5922.78     |   14629.11    |   14625.06     |
|              Node152              |  0.00   |   2     |   0.25
( 1.96%)   |   -5922.78     |   14629.11    |   14625.06     |
|              Node278              |  0.00   |   2     |   0.25
( 1.96%)   |   -5922.78     |   14629.11    |   14625.06     |
|              Node29               |  0.00   |   2     |   0.25
( 1.96%)   |   -5922.78     |   14629.11    |   14625.06     |
|              Node283              |  0.00   |   2     |   0.25
( 1.96%)   |   -5922.78     |   14629.11    |   14625.06     |
|              Node234              |  0.00   |   2     |   0.25
( 1.96%)   |   -5922.78     |   14629.11    |   14625.06     |
|              Node235              |  0.00   |   2     |   0.25
( 2.00%)   |   -5922.78     |   14629.11    |   14625.06     |
|              Node237              |  0.00   |   2     |   0.25
( 2.00%)   |   -5922.78     |   14629.11    |   14625.06     |
|              Node236              |  0.00   |   2     |   0.25
( 1.96%)   |   -5922.78     |   14629.11    |   14625.06     |
|              Node233              |  0.00   |   2     |   0.25
( 1.96%)   |   -5922.78     |   14629.11    |   14625.06     |
|              Node23               |  0.00   |   2     |   0.25
( 1.96%)   |   -5922.78     |   14629.11    |   14625.06     |
|              Node188              |  0.00   |   2     |   0.25
( 1.96%)   |   -5922.78     |   14629.11    |   14625.06     |
|              Node232              |  0.00   |   2     |   0.25
( 1.96%)   |   -5922.78     |   14629.11    |   14625.06     |
|              Node231              |  0.00   |   2     |   0.25
( 1.96%)   |   -5922.78     |   14629.11    |   14625.06     |
|              Node30               |  0.00   |   2     |   0.25
( 1.96%)   |   -5922.78     |   14629.11    |   14625.06     |
|              Node153              |  0.00   |   2     |   0.25
( 1.96%)   |   -5922.78     |   14629.11    |   14625.06     |
|              Node286              |  0.00   |   2     |   0.25
( 1.96%)   |   -5922.78     |   14629.11    |   14625.06     |
|              Node284              |  0.00   |   2     |   0.25
( 1.96%)   |   -5922.78     |   14629.11    |   14625.06     |
|              Node285              |  0.00   |   2     |   0.25
( 1.96%)   |   -5922.78     |   14629.11    |   14625.06     |
|              Node274              |  0.00   |   2     |   0.25
( 1.96%)   |   -5922.78     |   14629.11    |   14625.06     |
|              Node150              |  0.00   |   2     |   0.25
( 2.00%)   |   -5922.78     |   14629.11    |   14625.06     |
|              Node25               |  0.00   |   2     |   0.25
( 1.96%)   |   -5922.78     |   14629.11    |   14625.06     |
|              Node256              |  0.00   |   2     |   0.25
( 1.96%)   |   -5922.78     |   14629.11    |   14625.06     |
|              Node27               |  0.00   |   2     |   0.25
( 1.96%)   |   -5922.78     |   14629.11    |   14625.06     |
```

| Node260 | 0.00 | 2 | 0.25
(1.96%) | -5922.78 | 14629.11 | 14625.06 |
| Node26 | 0.00 | 2 | 0.25
(1.96%) | -5922.78 | 14629.11 | 14625.06 |
| Node186 | 0.00 | 2 | 0.25
(1.96%) | -5922.78 | 14629.11 | 14625.06 |
| Node204 | 0.00 | 2 | 0.25
(1.96%) | -5922.78 | 14629.11 | 14625.06 |
| Node198 | 0.00 | 2 | 0.25
(1.96%) | -5922.78 | 14629.11 | 14625.06 |
| Node21 | 0.00 | 2 | 0.25
(1.96%) | -5922.78 | 14629.11 | 14625.06 |
| Node202 | 0.00 | 2 | 0.25
(1.96%) | -5922.78 | 14629.11 | 14625.06 |
| Node2 | 0.00 | 2 | 0.25
(1.96%) | -5922.78 | 14629.11 | 14625.06 |
| Node20 | 0.00 | 2 | 0.25
(1.96%) | -5922.78 | 14629.11 | 14625.06 |
| Node200 | 0.00 | 2 | 0.25
(1.96%) | -5922.78 | 14629.11 | 14625.06 |
| Node196 | 0.00 | 2 | 0.25
(1.96%) | -5922.78 | 14629.11 | 14625.06 |
| Node184 | 0.00 | 2 | 0.25
(1.96%) | -5922.78 | 14629.11 | 14625.06 |
| Node225 | 0.00 | 2 | 0.25
(1.96%) | -5922.78 | 14629.11 | 14625.06 |
| Node228 | 0.00 | 2 | 0.25
(1.96%) | -5922.78 | 14629.11 | 14625.06 |
| Node182 | 0.00 | 2 | 0.25
(2.00%) | -5922.78 | 14629.11 | 14625.06 |
| Node192 | 0.00 | 2 | 0.25
(2.00%) | -5922.78 | 14629.11 | 14625.06 |
| Node180 | 0.00 | 2 | 0.25
(1.96%) | -5922.78 | 14629.11 | 14625.06 |
| Node22 | 0.00 | 2 | 0.25
(1.96%) | -5922.78 | 14629.11 | 14625.06 |
| Node218 | 0.00 | 2 | 0.25
(1.96%) | -5922.78 | 14629.11 | 14625.06 |
| HRV_KP859575_2c_HR774_2014 | 0.00 | 2 | 0.25
(1.96%) | -5922.78 | 14629.11 | 14625.06 |
| ITA_FJ005209_2c_303_2004 | 0.00 | 2 | 0.25
(1.96%) | -5922.78 | 14629.11 | 14625.06 |
| ITA_FJ005233_40_2007 | 0.00 | 2 | 0.25
(1.96%) | -5922.78 | 14629.11 | 14625.06 |
| ITA_FJ005258_80_2008 | 0.00 | 2 | 0.25
(1.96%) | -5922.78 | 14629.11 | 14625.06 |
| ITA_FJ005206_2c_287_2004 | 0.00 | 2 | 0.25
(1.96%) | -5922.78 | 14629.11 | 14625.06 |
| Node10 | 0.00 | 2 | 0.25
(1.96%) | -5922.78 | 14629.11 | 14625.06 |
| Node1 | 0.00 | 2 | 0.25
(1.96%) | -5922.78 | 14629.11 | 14625.06 |
| ITA_FJ005205_2c_279_2004 | 0.00 | 2 | 0.25
(2.00%) | -5922.78 | 14629.11 | 14625.06 |

ITA_FJ005263_42_2005	0.00	2	0.25 (1.96%)	-5922.78	14629.11	14625.06
ITA_KF373611_2a_409_2010	0.00	2	0.25 (1.96%)	-5922.78	14629.11	14625.06
ITA_KX434454_29451_09_2009	0.00	2	0.25 (1.96%)	-5922.78	14629.11	14625.06
ITA_KU508407_2c_25835_09_2009	0.00	2	0.25 (1.96%)	-5922.78	14629.11	14625.06
ITA_KX434458_2323_11_2011	0.00	2	0.25 (1.96%)	-5922.78	14629.11	14625.06
ITA_FJ222821_2c_56_2000	0.00	2	0.25 (1.96%)	-5922.78	14629.11	14625.06
ITA_FJ222824_388_05_3_2005	0.00	2	0.25 (1.96%)	-5922.78	14629.11	14625.06
ITA_KF373580_2a_581_2003	0.00	2	0.25 (1.96%)	-5922.78	14629.11	14625.06
ITA_KF373571_2a_685_1999	0.00	2	0.25 (1.96%)	-5922.78	14629.11	14625.06
ITA_AF393506_2a_699_2000	0.00	2	0.25 (1.96%)	-5922.78	14629.11	14625.06
HUN_KF539793_H_5_2012	0.00	2	0.25 (1.96%)	-5922.78	14629.11	14625.06
HUN_KF539794_H_7_2012	0.00	2	0.25 (1.96%)	-5922.78	14629.11	14625.06
Node36	0.00	2	0.25 (1.96%)	-5922.78	14629.11	14625.06
Node355	0.00	2	0.25 (1.96%)	-5922.78	14629.11	14625.06
Node35	0.00	2	0.25 (1.96%)	-5922.78	14629.11	14625.06
Node352	0.00	2	0.25 (1.96%)	-5922.78	14629.11	14625.06
Node354	0.00	2	0.25 (1.96%)	-5922.78	14629.11	14625.06
HRV_KP859576_2c_HR793_2014	0.00	2	0.25 (2.00%)	-5922.78	14629.11	14625.06
Node361	0.00	2	0.25 (1.96%)	-5922.78	14629.11	14625.06
Node105	0.00	2	0.25 (1.96%)	-5922.78	14629.11	14625.06
ITA_AF306447_618_2000	0.00	2	0.25 (1.96%)	-5922.78	14629.11	14625.06
Node103	0.00	2	0.25 (1.96%)	-5922.78	14629.11	14625.06
HUN_KF539805_H_36_2012	0.00	2	0.25 (1.96%)	-5922.78	14629.11	14625.06
Node368	0.00	2	0.25 (1.96%)	-5922.78	14629.11	14625.06
Node37	0.00	2	0.25 (1.96%)	-5922.78	14629.11	14625.06
Node115	0.00	2	0.25 (1.96%)	-5922.78	14629.11	14625.06
Node370	0.00	2	0.25 (1.96%)	-5922.78	14629.11	14625.06

```
|              Node194             |   0.00   |    2     |    0.25
( 1.96%)   |   -5922.78    |   14629.11     |    14625.06     |
|              Node190             |   0.00   |    2     |    0.25
( 1.96%)   |   -5922.78    |   14629.11     |    14625.06     |
|              Node19              |   0.00   |    2     |    0.25
( 1.96%)   |   -5922.78    |   14629.11     |    14625.06     |
|              Node18              |   0.00   |    2     |    0.25
( 1.96%)   |   -5922.78    |   14629.11     |    14625.06     |
|              Node17              |   0.00   |    2     |    0.25
( 1.96%)   |   -5922.78    |   14629.11     |    14625.06     |
|              Node178             |   0.00   |    2     |    0.25
( 1.96%)   |   -5922.78    |   14629.11     |    14625.06     |
|              Node15              |   0.00   |    2     |    0.25
( 1.96%)   |   -5922.78    |   14629.11     |    14625.06     |
|              Node136             |   0.00   |    2     |    0.25
( 1.96%)   |   -5922.78    |   14629.11     |    14625.06     |
|              Node13              |   0.00   |    2     |    0.25
( 1.96%)   |   -5922.78    |   14629.11     |    14625.06     |
|              Node118             |   0.00   |    2     |    0.25
( 1.96%)   |   -5922.78    |   14629.11     |    14625.06     |
|              Node137             |   0.00   |    2     |    0.25
( 1.96%)   |   -5922.78    |   14629.11     |    14625.06     |
|              Node146             |   0.00   |    2     |    0.25
( 1.96%)   |   -5922.78    |   14629.11     |    14625.06     |
|              Node144             |   0.00   |    2     |    0.25
( 1.96%)   |   -5922.78    |   14629.11     |    14625.06     |
|              Node14              |   0.00   |    2     |    0.25
( 1.96%)   |   -5922.78    |   14629.11     |    14625.06     |
|              Node142             |   0.00   |    2     |    0.25
( 1.96%)   |   -5922.78    |   14629.11     |    14625.06     |
|    FRA_DQ025991_2b_04S22_2004    |   0.00   |    2     |    0.25
( 1.96%)   |   -5922.78    |   14629.11     |    14625.06     |
|     BRA_DQ340414_2a_BR31_1990    |   0.00   |    2     |    0.25
( 1.96%)   |   -5922.78    |   14629.11     |    14625.06     |
|    BRA_DQ340408_2a_BR154_1980    |   0.00   |    2     |    0.25
( 1.96%)   |   -5922.78    |   14629.11     |    14625.06     |
|     BRA_DQ340416_2a_BR47_1991    |   0.00   |    2     |    0.25
( 1.96%)   |   -5922.78    |   14629.11     |    14625.06     |
|    BRA_DQ340424_2a_BR137_1993    |   0.00   |    2     |    0.25
( 1.96%)   |   -5922.78    |   14629.11     |    14625.06     |
|    BRA_DQ340418_2a_BR491_1992    |   0.00   |    2     |    0.25
( 1.96%)   |   -5922.78    |   14629.11     |    14625.06     |
|     BRA_DQ340417_2a_BR52_1991    |   0.00   |    2     |    0.25
( 2.00%)   |   -5922.78    |   14629.11     |    14625.06     |
|    BRA_DQ340407_2a_BR145_1980    |   0.00   |    2     |    0.25
( 1.96%)   |   -5922.78    |   14629.11     |    14625.06     |
|      ARG_KM236572_NNGag_2012     |   0.00   |    2     |    0.25
( 1.96%)   |   -5922.78    |   14629.11     |    14625.06     |
|      ARG_JF414820_Arg44_2009     |   0.00   |    2     |    0.25
( 1.96%)   |   -5922.78    |   14629.11     |    14625.06     |
|      AUS_KU508691_2c_HB_2015     |   0.00   |    2     |    0.25
( 1.96%)   |   -5922.78    |   14629.11     |    14625.06     |
|    BRA_DQ340405_2a_BR135_1980    |   0.00   |    2     |    0.25
( 1.96%)   |   -5922.78    |   14629.11     |    14625.06     |
```

```
|     BRA_DQ340404_2a_BR6_1980      |   0.00   |    2     |     0.25
( 1.96%)   |   -5922.78    |   14629.11     |    14625.06     |
|      AUS_KU508692_2c_FH_2015      |   0.00   |    2     |     0.25
( 2.00%)   |   -5922.78    |   14629.11     |    14625.06     |
|     BRA_DQ340426_2a_BR84_1994     |   0.00   |    2     |     0.25
( 1.96%)   |   -5922.78    |   14629.11     |    14625.06     |
|     FRA_DQ025944_2a_02B2_2002     |   0.00   |    2     |     0.25
( 1.96%)   |   -5922.78    |   14629.11     |    14625.06     |
|      FRA_DQ025942_01B1_2001       |   0.00   |    2     |     0.25
( 1.96%)   |   -5922.78    |   14629.11     |    14625.06     |
|     FRA_DQ025945_2a_02B3_2002     |   0.00   |    2     |     0.25
( 1.96%)   |   -5922.78    |   14629.11     |    14625.06     |
|    FRA_DQ025984_2a_04S15_2004     |   0.00   |    2     |     0.25
( 1.96%)   |   -5922.78    |   14629.11     |    14625.06     |
|    FRA_DQ025983_2a_04S14_2004     |   0.00   |    2     |     0.25
( 1.96%)   |   -5922.78    |   14629.11     |    14625.06     |
|     FRA_DQ025950_2a_02B9_2002     |   0.00   |    2     |     0.25
( 1.96%)   |   -5922.78    |   14629.11     |    14625.06     |
|FIN_U22192_raccoondog_RD_80_1980...|   0.00   |    2     |     0.25
( 1.96%)   |   -5922.78    |   14629.11     |    14625.06     |
|     CHI_GU392236_fox_HB1_2009     |   0.00   |    2     |     0.25
( 1.96%)   |   -5922.78    |   14629.11     |    14625.06     |
|    BRA_DQ340428_2a_BR209_1994     |   0.00   |    2     |     0.25
( 2.00%)   |   -5922.78    |   14629.11     |    14625.06     |
|    BRA_DQ340427_2a_BR133_1994     |   0.00   |    2     |     0.25
( 1.96%)   |   -5922.78    |   14629.11     |    14625.06     |
|CHI_GU392240_raccoondog_HB3_2009...|   0.00   |    2     |     0.25
( 1.96%)   |   -5922.78    |   14629.11     |    14625.06     |
|     ECU_KF149962_2c_ME1_2012      |   0.00   |    2     |     0.25
( 1.96%)   |   -5922.78    |   14629.11     |    14625.06     |
|   CHI_KF803602_2010_BJ_A72_2010   |   0.00   |    2     |     0.25
( 1.96%)   |   -5922.78    |   14629.11     |    14625.06     |
|    CHI_GU569942_2a_JL0202_2002    |   0.00   |    2     |     0.25
( 1.96%)   |   -5922.78    |   14629.11     |    14625.06     |
|              Node372              |   0.00   |    2     |     0.25
( 2.00%)   |   -5922.78    |   14629.11     |    14625.06     |
|       THA_FJ869125_KU5_2004       |   0.00   |    2     |     0.25
( 2.00%)   |   -5922.78    |   14629.11     |    14625.06     |
|      THA_FJ869130_KU13_2004       |   0.00   |    2     |     0.25
( 1.96%)   |   -5922.78    |   14629.11     |    14625.06     |
|      THA_FJ869137_KU52_2003       |   0.00   |    2     |     0.25
( 1.96%)   |   -5922.78    |   14629.11     |    14625.06     |
|      THA_FJ869134_KU23_2003       |   0.00   |    2     |     0.25
( 1.96%)   |   -5922.78    |   14629.11     |    14625.06     |
|       THA_FJ869123_KU3_2008       |   0.00   |    2     |     0.25
( 2.00%)   |   -5922.78    |   14629.11     |    14625.06     |
|  POR_KU662349_greywolf_W33_1996   |   0.00   |    2     |     0.25
( 2.00%)   |   -5922.78    |   14629.11     |    14625.06     |
|        POL_Z46651_46_1994         |   0.00   |    2     |     0.25
( 1.96%)   |   -5922.78    |   14629.11     |    14625.06     |
|     SAF_HQ602969_22_10SA_2010     |   0.00   |    2     |     0.25
( 2.00%)   |   -5922.78    |   14629.11     |    14625.06     |
|      TAW_U72696_2b_T10_1996       |   0.00   |    2     |     0.25
( 1.96%)   |   -5922.78    |   14629.11     |    14625.06     |
```

```
|     TAW_FJ265781_CPV307_2005       |   0.00    |    2      |     0.25
( 1.96%)   |   -5922.78    |   14629.11     |     14625.06      |
|      THA_FJ869138_KU53_2003        |   0.00    |    2      |     0.25
( 1.96%)   |   -5922.78    |   14629.11     |     14625.06      |
|     URU_KC196093_2c_M307_2011      |   0.00    |    2      |     0.25
( 1.96%)   |   -5922.78    |   14629.11     |     14625.06      |
|     URU_KC196091_2c_M326_2011      |   0.00    |    2      |     0.25
( 1.96%)   |   -5922.78    |   14629.11     |     14625.06      |
|     URU_KC196096_2c_M247_2010      |   0.00    |    2      |     0.25
( 1.96%)   |   -5922.78    |   14629.11     |     14625.06      |
|     URU_KC196101_2c_M187_2009      |   0.00    |    2      |     0.25
( 1.96%)   |   -5922.78    |   14629.11     |     14625.06      |
|     URU_KC196097_2c_M242_2010      |   0.00    |    2      |     0.25
( 1.96%)   |   -5922.78    |   14629.11     |     14625.06      |
|     URU_KC196089_2c_M349_2011      |   0.00    |    2      |     0.25
( 1.96%)   |   -5922.78    |   14629.11     |     14625.06      |
|      THA_KP715716_VT143_2014       |   0.00    |    2      |     0.25
( 1.96%)   |   -5922.78    |   14629.11     |     14625.06      |
|      THA_KP715691_VT43_2014        |   0.00    |    2      |     0.25
( 1.96%)   |   -5922.78    |   14629.11     |     14625.06      |
|     URU_KC196081_2c_M95_2007       |   0.00    |    2      |     0.25
( 1.96%)   |   -5922.78    |   14629.11     |     14625.06      |
|     URU_KC196086_2c_M55_2006       |   0.00    |    2      |     0.25
( 1.96%)   |   -5922.78    |   14629.11     |     14625.06      |
|     URU_KC196083_2c_M82_2007       |   0.00    |    2      |     0.25
( 1.96%)   |   -5922.78    |   14629.11     |     14625.06      |
|              Node98                |   0.00    |    2      |     0.25
( 1.96%)   |   -5922.78    |   14629.11     |     14625.06      |
|              Node627               |   0.00    |    2      |     0.25
( 1.96%)   |   -5922.78    |   14629.11     |     14625.06      |
|              Node619               |   0.00    |    2      |     0.25
( 1.96%)   |   -5922.78    |   14629.11     |     14625.06      |
|              Node629               |   0.00    |    2      |     0.25
( 1.96%)   |   -5922.78    |   14629.11     |     14625.06      |
|              Node638               |   0.00    |    2      |     0.25
( 1.96%)   |   -5922.78    |   14629.11     |     14625.06      |
|              Node635               |   0.00    |    2      |     0.25
( 1.96%)   |   -5922.78    |   14629.11     |     14625.06      |
|              Node617               |   0.00    |    2      |     0.25
( 2.00%)   |   -5922.78    |   14629.11     |     14625.06      |
|              Node607               |   0.00    |    2      |     0.25
( 1.96%)   |   -5922.78    |   14629.11     |     14625.06      |
|              Node605               |   0.00    |    2      |     0.25
( 1.96%)   |   -5922.78    |   14629.11     |     14625.06      |
|              Node609               |   0.00    |    2      |     0.25
( 2.00%)   |   -5922.78    |   14629.11     |     14625.06      |
|              Node611               |   0.00    |    2      |     0.25
( 2.00%)   |   -5922.78    |   14629.11     |     14625.06      |
|              Node610               |   0.00    |    2      |     0.25
( 1.96%)   |   -5922.78    |   14629.11     |     14625.06      |
|              Node648               |   0.00    |    2      |     0.25
( 1.96%)   |   -5922.78    |   14629.11     |     14625.06      |
|              Node88                |   0.00    |    2      |     0.25
( 1.96%)   |   -5922.78    |   14629.11     |     14625.06      |
```

```
|              Node86              |   0.00   |    2     |    0.25
( 1.96%)   |   -5922.78    |   14629.11    |    14625.06     |
|               Node9              |   0.00   |    2     |    0.25
( 1.96%)   |   -5922.78    |   14629.11    |    14625.06     |
|              Node95              |   0.00   |    2     |    0.25
( 1.96%)   |   -5922.78    |   14629.11    |    14625.06     |
|              Node93              |   0.00   |    2     |    0.25
( 1.96%)   |   -5922.78    |   14629.11    |    14625.06     |
|               Node7              |   0.00   |    2     |    0.25
( 1.96%)   |   -5922.78    |   14629.11    |    14625.06     |
|              Node650             |   0.00   |    2     |    0.25
( 1.96%)   |   -5922.78    |   14629.11    |    14625.06     |
|              Node649             |   0.00   |    2     |    0.25
( 1.96%)   |   -5922.78    |   14629.11    |    14625.06     |
|              Node651             |   0.00   |    2     |    0.25
( 2.00%)   |   -5922.78    |   14629.11    |    14625.06     |
|              Node665             |   0.00   |    2     |    0.25
( 1.96%)   |   -5922.78    |   14629.11    |    14625.06     |
|              Node655             |   0.00   |    2     |    0.25
( 1.96%)   |   -5922.78    |   14629.11    |    14625.06     |
|     URU_KC196102_2c_M185_2009    |   0.00   |    2     |    0.25
( 1.96%)   |   -5922.78    |   14629.11    |    14625.06     |
|   USA_JX475240_AZ_16382_01_1999  |   0.00   |    2     |    0.25
( 1.96%)   |   -5922.78    |   14629.11    |    14625.06     |
|     USA_JX475237_CT_372_2011     |   0.00   |    2     |    0.25
( 1.96%)   |   -5922.78    |   14629.11    |    14625.06     |
|      USA_JX475284_TN_26_2011     |   0.00   |    2     |    0.25
( 1.96%)   |   -5922.78    |   14629.11    |    14625.06     |
|  USA_KJ813831_Fisher_ND_17_2013  |   0.00   |    2     |    0.25
( 1.96%)   |   -5922.78    |   14629.11    |    14625.06     |
|USA_KJ813827_Fisher_F1M111211_20...|   0.00   |    2     |    0.25
( 2.00%)   |   -5922.78    |   14629.11    |    14625.06     |
|     USA_JX475231_CO_280_2011     |   0.00   |    2     |    0.25
( 1.96%)   |   -5922.78    |   14629.11    |    14625.06     |
|   USA_EU659119_2b_CPV_410_2000   |   0.00   |    2     |    0.25
( 1.96%)   |   -5922.78    |   14629.11    |    14625.06     |
|      USA_EU659116_CPV_5_1979     |   0.00   |    2     |    0.25
( 1.96%)   |   -5922.78    |   14629.11    |    14625.06     |
|  USA_JN867598_Bobcat_KS_44_2010  |   0.00   |    2     |    0.25
( 1.96%)   |   -5922.78    |   14629.11    |    14625.06     |
|  USA_JN867618_Raccoon_WI_37_2010 |   0.00   |    2     |    0.25
( 1.96%)   |   -5922.78    |   14629.11    |    14625.06     |
|USA_JN867599_Raccoon_KY_39552_20...|   0.00   |    2     |    0.25
( 2.00%)   |   -5922.78    |   14629.11    |    14625.06     |
|  USA_KJ813888_Coyote_MT_878_2012 |   0.00   |    2     |    0.25
( 1.96%)   |   -5922.78    |   14629.11    |    14625.06     |
|    VAC_KY083089_Singapore_2016   |   0.00   |    2     |    0.25
( 1.96%)   |   -5922.78    |   14629.11    |    14625.06     |
|     VAC_FJ197847_Pfizer_2007     |   0.00   |    2     |    0.25
( 1.96%)   |   -5922.78    |   14629.11    |    14625.06     |
|   VIE_AB054218_2b_cat_V123_2000  |   0.00   |    2     |    0.25
( 2.00%)   |   -5922.78    |   14629.11    |    14625.06     |
|   VIE_AB120725_2b_HNI_3_4_2003   |   0.00   |    2     |    0.25
( 1.96%)   |   -5922.78    |   14629.11    |    14625.06     |
```

VIE_AB120724_2b_HNI_2_13_2003	0.00	2	0.25 (1.96%)	-5922.78	14629.11	14625.06
USA_U22186_CPV_128_1995	0.00	2	0.25 (1.96%)	-5922.78	14629.11	14625.06
USA_M24003_FPV_CPV_15_1988	0.00	2	0.25 (1.96%)	-5922.78	14629.11	14625.06
USA_M23255_FPV_Cornell320_1988	0.00	2	0.25 (1.96%)	-5922.78	14629.11	14625.06
USA_M38245_1990	0.00	2	0.25 (1.96%)	-5922.78	14629.11	14625.06
USA_M74852_133_1995	0.00	2	0.25 (2.00%)	-5922.78	14629.11	14625.06
USA_M74849_39_1995	0.00	2	0.25 (2.00%)	-5922.78	14629.11	14625.06
USA_AY742955_436_2003	0.00	2	0.25 (2.00%)	-5922.78	14629.11	14625.06
URU_KM457113_2c_UY152_2009	0.00	2	0.25 (1.96%)	-5922.78	14629.11	14625.06
URU_KM457112_2c_UY135_2008	0.00	2	0.25 (1.96%)	-5922.78	14629.11	14625.06
URU_KM457116_2c_UY185_2009	0.00	2	0.25 (1.96%)	-5922.78	14629.11	14625.06
URU_KM457120_2c_UY242_2010	0.00	2	0.25 (1.96%)	-5922.78	14629.11	14625.06
URU_KM457117_2c_UY187_2009	0.00	2	0.25 (1.96%)	-5922.78	14629.11	14625.06
URU_KM457111_2c_UY120_2008	0.00	2	0.25 (1.96%)	-5922.78	14629.11	14625.06
URU_KM457106_2c_UY55_2006	0.00	2	0.25 (1.96%)	-5922.78	14629.11	14625.06
URU_KC196105_2c_M152_2008	0.00	2	0.25 (1.96%)	-5922.78	14629.11	14625.06
URU_KM457107_2c_UY72_2007	0.00	2	0.25 (1.96%)	-5922.78	14629.11	14625.06
URU_KM457109_2c_UY95_2007	0.00	2	0.25 (1.96%)	-5922.78	14629.11	14625.06
URU_KM457108_2c_UY82_2007	0.00	2	0.25 (1.96%)	-5922.78	14629.11	14625.06
URU_KM457121_2c_UY247_2010	0.00	2	0.25 (1.96%)	-5922.78	14629.11	14625.06
URU_KM457142_2c_UY370_2011	0.00	2	0.25 (1.96%)	-5922.78	14629.11	14625.06
URU_KM457130_2c_UY354_2011	0.00	2	0.25 (1.96%)	-5922.78	14629.11	14625.06
USA_AY742932_193_1991	0.00	2	0.25 (1.96%)	-5922.78	14629.11	14625.06
USA_AY742951_431_2003	0.00	2	0.25 (1.96%)	-5922.78	14629.11	14625.06
USA_AY742936_395_1998	0.00	2	0.25 (2.00%)	-5922.78	14629.11	14625.06
URU_KM457129_2c_UY349_2011	0.00	2	0.25 (1.96%)	-5922.78	14629.11	14625.06
URU_KM457123_2c_UY261_2008	0.00	2	0.25 (1.96%)	-5922.78	14629.11	14625.06

```
|    URU_KM457122_2c_UY258_2010      |   0.00   |    2     |    0.25
( 1.96%)   |   -5922.78    |   14629.11    |    14625.06     |
|    URU_KM457124_2c_UY307_2011      |   0.00   |    2     |    0.25
( 1.96%)   |   -5922.78    |   14629.11    |    14625.06     |
|    URU_KM457127_2c_UY326_2011      |   0.00   |    2     |    0.25
( 1.96%)   |   -5922.78    |   14629.11    |    14625.06     |
|    URU_KM457125_2c_UY317_2011      |   0.00   |    2     |    0.25
( 2.00%)   |   -5922.78    |   14629.11    |    14625.06     |
|              Node603               |   0.00   |    2     |    0.25
( 1.96%)   |   -5922.78    |   14629.11    |    14625.06     |
|              Node44                |   0.00   |    2     |    0.25
( 1.96%)   |   -5922.78    |   14629.11    |    14625.06     |
|              Node440               |   0.00   |    2     |    0.25
( 1.96%)   |   -5922.78    |   14629.11    |    14625.06     |
|              Node443               |   0.00   |    2     |    0.25
( 1.96%)   |   -5922.78    |   14629.11    |    14625.06     |
|              Node441               |   0.00   |    2     |    0.25
( 1.96%)   |   -5922.78    |   14629.11    |    14625.06     |
|              Node436               |   0.00   |    2     |    0.25
( 1.96%)   |   -5922.78    |   14629.11    |    14625.06     |
|              Node428               |   0.00   |    2     |    0.25
( 1.96%)   |   -5922.78    |   14629.11    |    14625.06     |
|              Node426               |   0.00   |    2     |    0.25
( 1.96%)   |   -5922.78    |   14629.11    |    14625.06     |
|              Node43                |   0.00   |    2     |    0.25
( 1.96%)   |   -5922.78    |   14629.11    |    14625.06     |
|              Node432               |   0.00   |    2     |    0.25
( 1.96%)   |   -5922.78    |   14629.11    |    14625.06     |
|              Node430               |   0.00   |    2     |    0.25
( 1.96%)   |   -5922.78    |   14629.11    |    14625.06     |
|              Node444               |   0.00   |    2     |    0.25
( 1.96%)   |   -5922.78    |   14629.11    |    14625.06     |
|              Node468               |   0.00   |    2     |    0.25
( 1.96%)   |   -5922.78    |   14629.11    |    14625.06     |
|              Node466               |   0.00   |    2     |    0.25
( 1.96%)   |   -5922.78    |   14629.11    |    14625.06     |
|              Node47                |   0.00   |    2     |    0.25
( 1.96%)   |   -5922.78    |   14629.11    |    14625.06     |
|              Node472               |   0.00   |    2     |    0.25
( 1.96%)   |   -5922.78    |   14629.11    |    14625.06     |
|              Node470               |   0.00   |    2     |    0.25
( 1.96%)   |   -5922.78    |   14629.11    |    14625.06     |
|              Node464               |   0.00   |    2     |    0.25
( 1.96%)   |   -5922.78    |   14629.11    |    14625.06     |
|              Node453               |   0.00   |    2     |    0.25
( 2.00%)   |   -5922.78    |   14629.11    |    14625.06     |
|              Node45                |   0.00   |    2     |    0.25
( 1.96%)   |   -5922.78    |   14629.11    |    14625.06     |
|              Node456               |   0.00   |    2     |    0.25
( 1.96%)   |   -5922.78    |   14629.11    |    14625.06     |
|              Node462               |   0.00   |    2     |    0.25
( 1.96%)   |   -5922.78    |   14629.11    |    14625.06     |
|              Node46                |   0.00   |    2     |    0.25
( 1.96%)   |   -5922.78    |   14629.11    |    14625.06     |
```

Node420	0.00	2	0.25	(1.96%)	-5922.78	14629.11	14625.06
Node39	0.00	2	0.25	(1.96%)	-5922.78	14629.11	14625.06
Node384	0.00	2	0.25	(1.96%)	-5922.78	14629.11	14625.06
Node390	0.00	2	0.25	(1.96%)	-5922.78	14629.11	14625.06
Node396	0.00	2	0.25	(1.96%)	-5922.78	14629.11	14625.06
Node393	0.00	2	0.25	(2.00%)	-5922.78	14629.11	14625.06
Node382	0.00	2	0.25	(2.00%)	-5922.78	14629.11	14625.06
Node376	0.00	2	0.25	(1.96%)	-5922.78	14629.11	14625.06
Node374	0.00	2	0.25	(1.96%)	-5922.78	14629.11	14625.06
Node378	0.00	2	0.25	(2.00%)	-5922.78	14629.11	14625.06
Node380	0.00	2	0.25	(2.00%)	-5922.78	14629.11	14625.06
Node38	0.00	2	0.25	(1.96%)	-5922.78	14629.11	14625.06
Node40	0.00	2	0.25	(1.96%)	-5922.78	14629.11	14625.06
Node407	0.00	2	0.25	(1.96%)	-5922.78	14629.11	14625.06
Node406	0.00	2	0.25	(1.96%)	-5922.78	14629.11	14625.06
Node41	0.00	2	0.25	(1.96%)	-5922.78	14629.11	14625.06
Node42	0.00	2	0.25	(1.96%)	-5922.78	14629.11	14625.06
Node412	0.00	2	0.25	(1.96%)	-5922.78	14629.11	14625.06
Node405	0.00	2	0.25	(1.96%)	-5922.78	14629.11	14625.06
Node401	0.00	2	0.25	(1.96%)	-5922.78	14629.11	14625.06
Node400	0.00	2	0.25	(1.96%)	-5922.78	14629.11	14625.06
Node402	0.00	2	0.25	(2.00%)	-5922.78	14629.11	14625.06
Node404	0.00	2	0.25	(2.00%)	-5922.78	14629.11	14625.06
Node403	0.00	2	0.25	(1.96%)	-5922.78	14629.11	14625.06
Node474	0.00	2	0.25	(1.96%)	-5922.78	14629.11	14625.06
Node547	0.00	2	0.25	(1.96%)	-5922.78	14629.11	14625.06
Node543	0.00	2	0.25	(1.96%)	-5922.78	14629.11	14625.06

Node551	0.00	2	0.25 (1.96%)	-5922.78	14629.11	14625.06
Node555	0.00	2	0.25 (1.96%)	-5922.78	14629.11	14625.06
Node553	0.00	2	0.25 (1.96%)	-5922.78	14629.11	14625.06
Node541	0.00	2	0.25 (1.96%)	-5922.78	14629.11	14625.06
Node531	0.00	2	0.25 (2.00%)	-5922.78	14629.11	14625.06
Node529	0.00	2	0.25 (1.96%)	-5922.78	14629.11	14625.06
Node533	0.00	2	0.25 (2.00%)	-5922.78	14629.11	14625.06
Node537	0.00	2	0.25 (2.00%)	-5922.78	14629.11	14625.06
Node535	0.00	2	0.25 (1.96%)	-5922.78	14629.11	14625.06
Node557	0.00	2	0.25 (1.96%)	-5922.78	14629.11	14625.06
Node592	0.00	2	0.25 (1.96%)	-5922.78	14629.11	14625.06
Node590	0.00	2	0.25 (1.96%)	-5922.78	14629.11	14625.06
Node594	0.00	2	0.25 (2.00%)	-5922.78	14629.11	14625.06
Node6	0.00	2	0.25 (2.00%)	-5922.78	14629.11	14625.06
Node596	0.00	2	0.25 (1.96%)	-5922.78	14629.11	14625.06
Node588	0.00	2	0.25 (1.96%)	-5922.78	14629.11	14625.06
Node568	0.00	2	0.25 (2.00%)	-5922.78	14629.11	14625.06
Node566	0.00	2	0.25 (1.96%)	-5922.78	14629.11	14625.06
Node570	0.00	2	0.25 (2.00%)	-5922.78	14629.11	14625.06
Node581	0.00	2	0.25 (1.96%)	-5922.78	14629.11	14625.06
Node572	0.00	2	0.25 (2.00%)	-5922.78	14629.11	14625.06
Node524	0.00	2	0.25 (2.00%)	-5922.78	14629.11	14625.06
Node49	0.00	2	0.25 (1.96%)	-5922.78	14629.11	14625.06
Node484	0.00	2	0.25 (1.96%)	-5922.78	14629.11	14625.06
Node494	0.00	2	0.25 (1.96%)	-5922.78	14629.11	14625.06
Node5	0.00	2	0.25 (1.96%)	-5922.78	14629.11	14625.06
Node499	0.00	2	0.25 (1.96%)	-5922.78	14629.11	14625.06

Node483	0.00	2	0.25 (2.00%)	-5922.78	14629.11	14625.06
Node479	0.00	2	0.25 (1.96%)	-5922.78	14629.11	14625.06
Node477	0.00	2	0.25 (1.96%)	-5922.78	14629.11	14625.06
Node48	0.00	2	0.25 (1.96%)	-5922.78	14629.11	14625.06
Node482	0.00	2	0.25 (1.96%)	-5922.78	14629.11	14625.06
Node480	0.00	2	0.25 (1.96%)	-5922.78	14629.11	14625.06
Node50	0.00	2	0.25 (1.96%)	-5922.78	14629.11	14625.06
Node52	0.00	2	0.25 (1.96%)	-5922.78	14629.11	14625.06
Node519	0.00	2	0.25 (1.96%)	-5922.78	14629.11	14625.06
Node521	0.00	2	0.25 (1.96%)	-5922.78	14629.11	14625.06
Node523	0.00	2	0.25 (2.00%)	-5922.78	14629.11	14625.06
Node522	0.00	2	0.25 (1.96%)	-5922.78	14629.11	14625.06
Node513	0.00	2	0.25 (2.00%)	-5922.78	14629.11	14625.06
Node506	0.00	2	0.25 (2.00%)	-5922.78	14629.11	14625.06
Node505	0.00	2	0.25 (1.96%)	-5922.78	14629.11	14625.06
Node509	0.00	2	0.25 (1.96%)	-5922.78	14629.11	14625.06
Node512	0.00	2	0.25 (2.00%)	-5922.78	14629.11	14625.06
Node51	0.00	2	0.25 (1.96%)	-5922.78	14629.11	14625.06

Rate class analyses summary
* 8 branches with **2** rate classes
* 667 branches with **1** rate classes

Improving parameter estimates of the adaptive rate class model
* Log(L) = -5921.08, AIC-c = 14621.65 (1380 estimated parameters)

Testing selected branches for selection

Branch	Rates	Max. dN/dS	Test LRT	Uncorrected p-value
USA_M10989_1985	2	>1000 (0.33%)	7.08	0.01024
VAC_JN625220_INDIA_vac2_2011	1	0.10 (100.00%)	0.00	1.00000

KOR_EF599098_2c_Pome_2006	1	0.21 (100.00%)	0.00	1.00000
CHI_KF803600_2010_BJ_A68_2010	2	482.13 (0.35%)	12.88	0.00055
VAC_FJ011098_Intervet_2006	1	0.14 (100.00%)	0.00	1.00000
VAC_KY083090_Singapore_2016	2	>1000 (0.57%)	23.39	0.00000
ITA_FJ005264_134_2005	1	0.00 (100.00%)	0.00	1.00000
RUS_JN033694_Laika_1993	2	433.78 (0.19%)	10.62	0.00171
CHI_FJ231389_FPV_monkey_BJ_22_20...	1	0.61 (100.00%)	0.00	1.00000
POR_KU662350_greywolf_W52_2005	1	>1000 (100.00%)	1.50	0.18606
VAC_EU914139_Pfizer_2006	2	>1000 (0.49%)	15.10	0.00018
GER_FJ005261_G162_1997	1	0.00 (100.00%)	0.00	1.00000
Node311	1	0.00 (100.00%)	0.00	1.00000
NZE_AY742933_339_1993	1	0.00 (100.00%)	0.00	1.00000
GER_FJ005260_G82_1997	1	0.00 (100.00%)	0.00	1.00000
FRA_DQ025952_2a_03B12_2003	1	0.00 (100.00%)	0.00	1.00000
IND_KX469432_newCPV_2b_Hiller_20...	1	0.00 (100.00%)	0.00	1.00000
FRA_DQ025993_2a_04S24_2004	1	0.00 (100.00%)	0.00	1.00000
CHI_GQ169553_Vac2_2007	1	0.10 (100.00%)	0.00	1.00000
VAC_FJ011097_Merial_2006	1	0.10 (100.00%)	0.00	1.00000
VAC_FJ222823_2b_29_1997	1	0.10 (100.00%)	0.00	1.00000
CHI_GU569946_2a_JL0201_2002	1	0.10 (100.00%)	0.00	1.00000
Node640	1	0.10 (100.00%)	0.00	1.00000
VIE_AB054224_2c_leopard_V203_200...	1	0.10 (100.00%)	0.00	1.00000
CHI_GU392237_fox_HB2_2009	1	0.10 (100.00%)	0.00	1.00000
ECU_KF149971_2c_ME32_2012	1	0.10 (100.00%)	0.00	1.00000
VIE_AB054217_2a_cat_V154_2000	1	0.10 (100.00%)	0.00	1.00000
ITA_GU362932_cat11_2008	1	0.41 (100.00%)	0.00	1.00000
BRA_DQ340410_2a_BR315_1986	2	226.67 (0.23%)	8.06	0.00624

VIE_AB120721_2b_HCM_8_2003	1	0.41 (100.00%)	0.00	1.00000
Node356	2	>1000 (0.17%)	16.73	0.00008
HUN_KF539804_H_212_2012	1	0.42 (100.00%)	0.00	1.00000
CHI_KJ170680_raccoondog_HLJ11_1_...	2	>1000 (0.60%)	10.23	0.00208
Node599	1	>1000 (100.00%)	1.12	0.22975
Node562	1	>1000 (100.00%)	1.00	0.24477
FIN_U22193_raccoondog_RD87_1987	1	0.00 (100.00%)	0.00	1.00000
JPN_D26079_1993	1	0.00 (100.00%)	0.00	1.00000
THA_FJ869128_KU11_2004	1	0.00 (100.00%)	0.00	1.00000
Node123	1	0.00 (100.00%)	0.00	1.00000
POR_KT275253_2c_PT036_12_2012	1	0.00 (100.00%)	0.00	1.00000
ITA_FJ005265_140_2005	1	0.00 (100.00%)	0.00	1.00000
VAC_JN625219_INDIA_vac1_2011	1	0.00 (100.00%)	0.00	1.00000
THA_FJ869122_KU1_2008	1	0.00 (100.00%)	0.00	1.00000
TAW_FJ265775_CPV301_2004	1	0.00 (100.00%)	0.00	1.00000
ITA_KX434460_52238_12_2012	1	0.00 (100.00%)	0.00	1.00000
FRA_DQ025992_2b_04S23_2004	1	0.00 (100.00%)	0.00	1.00000
CHI_EU145954_2b_BJ044_2007	1	0.00 (100.00%)	0.00	1.00000
VIE_AB054220_2b_cat_V217_2000	1	0.00 (100.00%)	0.00	1.00000
Node223	1	0.00 (100.00%)	0.00	1.00000
BRA_DQ340411_2a_BR8_1990	1	0.00 (100.00%)	0.00	1.00000
USA_AY742953_435_2003	1	0.00 (100.00%)	0.00	1.00000
USA_KJ813854_Puma_ND_F205_2013	1	0.00 (100.00%)	0.00	1.00000
POR_KT275252_2c_PT013_12_2012	1	0.00 (100.00%)	0.00	1.00000
URU_KM457131_2c_UY368_2011	1	0.00 (100.00%)	0.00	1.00000
URU_KC196107_2c_M129_2008	1	0.00 (100.00%)	0.00	1.00000
ARG_JF414819_Arg35_2008	1	0.00 (100.00%)	0.00	1.00000

```
|              Node575              |      1     |  >1000 (100.00%)
|         0.83         |       0.26997      |
|        ITA_FJ005231_406_2006        |      1     |   0.00 (100.00%)
|         0.00         |       1.00000      |
|        ITA_FJ005232_411_2006        |      1     |   0.00 (100.00%)
|         0.00         |       1.00000      |
|      JPN_LC270891_2b_9985_2017      |      1     |   0.00 (100.00%)
|         0.00         |       1.00000      |
|              Node230              |      1     |   0.00 (100.00%)
|         0.00         |       1.00000      |
|      USA_EU659118_CPV_13_1981      |      1     |   0.00 (100.00%)
|         0.00         |       1.00000      |
|              Node437              |      1     |   0.00 (100.00%)
|         0.00         |       1.00000      |
|        ITA_FJ005252_96_2002        |      1     |   0.00 (100.00%)
|         0.00         |       1.00000      |
|USA_KJ813828_Fisher_F1F010712_20...|      1     |   0.00 (100.00%)
|         0.00         |       1.00000      |
|        HUN_KF539795_H_8_2012        |      1     |   0.00 (100.00%)
|         0.00         |       1.00000      |
|        ITA_FJ005255_333_2005        |      1     |   0.00 (100.00%)
|         0.00         |       1.00000      |
|       HUN_KF539799_H_39_2012       |      1     |   0.00 (100.00%)
|         0.00         |       1.00000      |
|USA_KJ813881_Graywolf_MI_832_201...|      1     |   0.00 (100.00%)
|         0.00         |       1.00000      |
|   VIE_AB054215_2a_cat_V120_2000   |      1     |   0.00 (100.00%)
|         0.00         |       1.00000      |
|              Node600              |      1     |   0.20 (100.00%)
|         0.00         |       1.00000      |
|              Node604              |      1     |   0.20 (100.00%)
|         0.00         |       1.00000      |
|     JPN_AB437434_1887_f_3_2008     |      1     |   0.21 (100.00%)
|         0.00         |       1.00000      |
|   VAC_JN625224_INDIA_vac6_2011    |      1     |   0.21 (100.00%)
|         0.00         |       1.00000      |
|USA_KJ813843_Bobcat_ND_1160_2013...|      1     |   0.21 (100.00%)
|         0.00         |       1.00000      |
|              Node147              |      1     |   0.21 (100.00%)
|         0.00         |       1.00000      |
|              Node353              |      1     |   0.21 (100.00%)
|         0.00         |       1.00000      |
|       USA_JX475279_TN_1_2011       |      1     |   0.21 (100.00%)
|         0.00         |       1.00000      |
|              Node502              |      1     |   0.21 (100.00%)
|         0.00         |       1.00000      |
|   POR_KT275255_2c_PT238_14_2014   |      1     |   0.21 (100.00%)
|         0.00         |       1.00000      |
|      URU_KM457103_2c_UY12_2006      |      1     |   0.21 (100.00%)
|         0.00         |       1.00000      |
|              Node324              |      1     |   0.21 (100.00%)
|         0.00         |       1.00000      |
|              Node60               |      1     |   0.21 (100.00%)
|         0.00         |       1.00000      |
```

```
|CHI_GU392244_raccoondog_HB7_2009...|      1     |   0.21 (100.00%)
|        0.00        |       1.00000      |
|              Node138              |      1     |   0.21 (100.00%)
|        0.00        |       1.00000      |
|    CHI_GQ857605_CPV07_03_2007     |      1     |   0.21 (100.00%)
|        0.00        |       1.00000      |
|VIE_AB054223_2c_leopard_V140_200...|      1     |   0.21 (100.00%)
|        0.00        |       1.00000      |
|    FRA_DQ025982_2a_04S13_2004     |      1     |   0.21 (100.00%)
|        0.00        |       1.00000      |
|    HRV_KP859577_2c_HR856_2014     |      1     |  >1000 (100.00%)
|        0.74        |       0.28458      |
|    URU_KM457126_2c_UY318_2010     |      1     |  >1000 (100.00%)
|        0.74        |       0.28506      |
|      HUN_KF539797_H_11_2012       |      1     |  >1000 (100.00%)
|        0.74        |       0.28464      |
|       ITA_FJ005253_67_2005        |      1     |  >1000 (100.00%)
|        0.72        |       0.28881      |
|              Node299              |      1     |  >1000 (100.00%)
|        0.71        |       0.28994      |
|              Node637              |      1     |  >1000 (100.00%)
|        0.75        |       0.28408      |
|CHI_GU392242_raccoondog_HB10_200...|      1     |  >1000 (100.00%)
|        0.71        |       0.28963      |
|   VAC_JN625221_INDIA_vac3_2011    |      1     |  >1000 (100.00%)
|        0.70        |       0.29175      |
|              Node671              |      1     |  >1000 (100.00%)
|        0.74        |       0.28552      |
|              Node663              |      1     |  >1000 (100.00%)
|        0.75        |       0.28375      |
|   VAC_JN625222_INDIA_vac4_2011    |      1     |  >1000 (100.00%)
|        0.71        |       0.29032      |
|    VAC_GU212790_primodog_2009     |      1     |  >1000 (100.00%)
|        0.71        |       0.29025      |
|              Node578              |      1     |  >1000 (100.00%)
|        0.61        |       0.30744      |
|               Node3               |      1     |  >1000 (100.00%)
|        0.43        |       0.34498      |
|  USA_KJ813832_Fisher_ND_14_2013   |      1     |   0.00 (100.00%)
|        0.00        |       1.00000      |
|  USA_KJ813835_Fisher_ND_19_2013   |      1     |   0.00 (100.00%)
|        0.00        |       1.00000      |
|              Node667              |      1     |   0.00 (100.00%)
|        0.00        |       1.00000      |
|        USA_U22896_cat_1990        |      1     |   0.00 (100.00%)
|        0.00        |       1.00000      |
|              Node130              |      1     |   0.00 (100.00%)
|        0.00        |       1.00000      |
|     USA_JX475248_CO_1102_2011     |      1     |   0.00 (100.00%)
|        0.00        |       1.00000      |
|              Node582              |      1     |   0.00 (100.00%)
|        0.00        |       1.00000      |
|              Node585              |      1     |   0.00 (100.00%)
|        0.00        |       1.00000      |
```

Node208	1	0.00 (100.00%)	0.00	1.00000
Node636	1	0.00 (100.00%)	0.00	1.00000
Node661	1	0.00 (100.00%)	0.00	1.00000
Node319	1	0.00 (100.00%)	0.00	1.00000
USA_M24000_FPV_CPV_31_1988	1	0.00 (100.00%)	0.00	1.00000
Node342	1	0.00 (100.00%)	0.00	1.00000
Node548	1	0.00 (100.00%)	0.00	1.00000
CHI_GU569943_YB8301_1983	1	0.00 (100.00%)	0.00	1.00000
USA_JN867610_Raccoon_VA_118_A_20...	1	0.00 (100.00%)	0.00	1.00000
Node348	1	0.00 (100.00%)	0.00	1.00000
VIE_AB120720_2b_HCM_6_2003	1	0.00 (100.00%)	0.00	1.00000
Node580	1	0.00 (100.00%)	0.00	1.00000
BRA_DQ340409_2b_BR183_1985	1	0.00 (100.00%)	0.00	1.00000
Node214	1	0.00 (100.00%)	0.00	1.00000
VIE_AB120723_2b_HCM_23_2003	1	0.00 (100.00%)	0.00	1.00000
Node287	1	0.00 (100.00%)	0.00	1.00000
USA_KJ813870_Raccoon_TX_1_2013	1	0.00 (100.00%)	0.00	1.00000
USA_JX475234_ME_258_2011	1	0.00 (100.00%)	0.00	1.00000
ITA_KX434456_45361_09_2009	1	0.00 (100.00%)	0.00	1.00000
FRA_DQ025964_03C8_2003	1	0.00 (100.00%)	0.00	1.00000
Node12	1	0.00 (100.00%)	0.00	1.00000
USA_JX475246_CO_2503_2010	1	0.00 (100.00%)	0.00	1.00000
JPN_AB115504_2c_97_008_1997	1	0.00 (100.00%)	0.00	1.00000
USA_JX475233_SC_182_A_2011	1	0.00 (100.00%)	0.00	1.00000
Node104	1	0.00 (100.00%)	0.00	1.00000
Node113	1	0.00 (100.00%)	0.00	1.00000
USA_JX475273_MT_909_2012	1	0.00 (100.00%)	0.00	1.00000

```
|        FRA_DQ025969_03S5_2003        |      1     |   0.00 (100.00%)
|        0.00         |        1.00000        |
|      BRA_DQ340419_2a_BR570_1992      |      1     |   0.00 (100.00%)
|        0.00         |        1.00000        |
|       ECU_KF149984_2c_ME28_2012      |      1     |   0.00 (100.00%)
|        0.00         |        1.00000        |
|        FRA_DQ025975_04S6_2004        |      1     |   0.00 (100.00%)
|        0.00         |        1.00000        |
|        FRA_DQ025976_04S7_2004        |      1     |   0.00 (100.00%)
|        0.00         |        1.00000        |
|        FRA_DQ025965_03C9_2003        |      1     |   0.00 (100.00%)
|        0.00         |        1.00000        |
|       URU_KC196085_2c_M57_2007       |      1     |   0.00 (100.00%)
|        0.00         |        1.00000        |
|        USA_JX475239_GA_06_2011       |      1     |   0.00 (100.00%)
|        0.00         |        1.00000        |
|       ITA_FJ005212_2c_349_2004       |      1     |   0.00 (100.00%)
|        0.00         |        1.00000        |
|               Node364                |      1     |   0.00 (100.00%)
|        0.00         |        1.00000        |
|      VIE_AB120722_2b_HCM_18_2003     |      1     |   0.00 (100.00%)
|        0.00         |        1.00000        |
|               Node563                |      1     |   0.00 (100.00%)
|        0.00         |        1.00000        |
|               Node229                |      1     |   0.00 (100.00%)
|        0.00         |        1.00000        |
|USA_JN867611_Raccoon_KY_358_B_20...|      1     |   0.00 (100.00%)
|        0.00         |        1.00000        |
|               Node392                |      1     |   0.00 (100.00%)
|        0.00         |        1.00000        |
|        HUN_KF539801_H_25_2012        |      1     |   0.00 (100.00%)
|        0.00         |        1.00000        |
|               Node387                |      1     |   0.00 (100.00%)
|        0.00         |        1.00000        |
|               Node217                |      1     |   0.00 (100.00%)
|        0.00         |        1.00000        |
|      JPN_AB437433_1887_M_2_2008      |      1     |   0.00 (100.00%)
|        0.00         |        1.00000        |
|        THA_FJ869139_KU66_2003        |      1     |   0.00 (100.00%)
|        0.00         |        1.00000        |
|      CHI_GQ857596_CPV05_01_2005      |      1     |   0.00 (100.00%)
|        0.00         |        1.00000        |
|               Node349                |      1     |   0.00 (100.00%)
|        0.00         |        1.00000        |
|      BRA_DQ340421_2a_BR597_1992      |      1     |   0.00 (100.00%)
|        0.00         |        1.00000        |
|       BRA_DQ340413_2a_BR18_1990      |      1     |   0.00 (100.00%)
|        0.00         |        1.00000        |
|               Node165                |      1     |   0.00 (100.00%)
|        0.00         |        1.00000        |
|               Node100                |      1     |   0.00 (100.00%)
|        0.00         |        1.00000        |
|               Node315                |      1     |   0.00 (100.00%)
|        0.00         |        1.00000        |
```

```
|              Node511              |      1     |   0.00 (100.00%)
|         0.00        |       1.00000      |
|              Node221              |      1     |   0.00 (100.00%)
|         0.00        |       1.00000      |
|              Node542              |      1     |   0.00 (100.00%)
|         0.00        |       1.00000      |
|              Node478              |      1     |   0.00 (100.00%)
|         0.00        |       1.00000      |
|              Node517              |      1     |   0.00 (100.00%)
|         0.00        |       1.00000      |
|              Node317              |      1     |   0.00 (100.00%)
|         0.00        |       1.00000      |
|              Node159              |      1     |   0.00 (100.00%)
|         0.00        |       1.00000      |
|       TWN_EF592511_TWN1_2006       |      1     |   0.00 (100.00%)
|         0.00        |       1.00000      |
|     BRA_DQ340423_2a_BR136_1993     |      1     |   0.00 (100.00%)
|         0.00        |       1.00000      |
|        ITA_FJ005257_54_2008        |      1     |   0.00 (100.00%)
|         0.00        |       1.00000      |
|     KOR_EF599097_2b_DH326_2006     |      1     |   0.00 (100.00%)
|         0.00        |       1.00000      |
|      USA_JX475260_CO_704_2010      |      1     |   0.00 (100.00%)
|         0.00        |       1.00000      |
|              Node328              |      1     |   0.00 (100.00%)
|         0.00        |       1.00000      |
|       FRA_DQ025960_03C4_2003       |      1     |   0.00 (100.00%)
|         0.00        |       1.00000      |
|       GER_FJ005196_2c_G7_1997      |      1     |   0.00 (100.00%)
|         0.00        |       1.00000      |
|     CHI_GQ857601_CPV06_02_2006     |      1     |   0.00 (100.00%)
|         0.00        |       1.00000      |
|CHI_GU392241_raccoondog_HB1_2009...|      1     |   0.00 (100.00%)
|         0.00        |       1.00000      |
|      GER_FJ005199_2c_G172_1997     |      1     |   0.00 (100.00%)
|         0.00        |       1.00000      |
|     USA_JX475243_ID_22772_2009     |      1     |   0.00 (100.00%)
|         0.00        |       1.00000      |
|       FRA_DQ025951_03B10_2003      |      1     |   0.00 (100.00%)
|         0.00        |       1.00000      |
|        ITA_FJ005247_195_2008       |      1     |   0.00 (100.00%)
|         0.00        |       1.00000      |
|              Node120              |      1     |   0.00 (100.00%)
|         0.00        |       1.00000      |
|      USA_JX475252_CO_1316_2010     |      1     |   0.00 (100.00%)
|         0.00        |       1.00000      |
|    USA_KJ813858_Puma_ND_F93_2013   |      1     |   0.00 (100.00%)
|         0.00        |       1.00000      |
|      ITA_FJ005218_2c_330_2006      |      1     |   0.00 (100.00%)
|         0.00        |       1.00000      |
|              Node57               |      1     |   0.00 (100.00%)
|         0.00        |       1.00000      |
|       FRA_DQ025994_04S25_2004      |      1     |   0.00 (100.00%)
|         0.00        |       1.00000      |
```

ITA_FJ005216_2c_284_2006	1	0.00 (100.00%)	0.00	1.00000
Node84	1	0.00 (100.00%)	0.00	1.00000
Node91	1	0.00 (100.00%)	0.00	1.00000
Node53	1	0.00 (100.00%)	0.00	1.00000
ITA_KX434459_27692_1_11_2011	1	0.00 (100.00%)	0.00	1.00000
FRA_DQ025985_04S16_2004	1	0.00 (100.00%)	0.00	1.00000
FRA_DQ026002_2a_04S33_2004	1	0.00 (100.00%)	0.00	1.00000
CHI_KF803615_2011_BJ_B25_2011	1	0.00 (100.00%)	0.00	1.00000
Node451	1	0.00 (100.00%)	0.00	1.00000
Node497	1	0.00 (100.00%)	0.00	1.00000
NIG_HQ602995_15_10_2010	1	0.00 (100.00%)	0.00	1.00000
GER_AY742934_447_1995	1	0.00 (100.00%)	0.00	1.00000
Node448	1	0.00 (100.00%)	0.00	1.00000
USA_KJ813892_Coyote_AK_218_2013	1	0.00 (100.00%)	0.00	1.00000
ITA_AF306446_584_2000	1	0.00 (100.00%)	0.00	1.00000
USA_JN867604_Dog_IL_137654_2008	1	0.00 (100.00%)	0.00	1.00000
Node398	1	0.00 (100.00%)	0.00	1.00000
NIG_HQ602992_19_10_2010	1	0.00 (100.00%)	0.00	1.00000
Node335	1	0.00 (100.00%)	0.00	1.00000
HUN_KF539800_H_27_2012	1	0.00 (100.00%)	0.00	1.00000
HUN_KF539796_H_9_2012	1	0.00 (100.00%)	0.00	1.00000
KOR_EF599096_DH426_2005	1	0.00 (100.00%)	0.00	1.00000
Node331	1	0.00 (100.00%)	0.00	1.00000
Node481	1	0.00 (100.00%)	0.00	1.00000
ITA_FJ005214_2c_67_2006	1	0.00 (100.00%)	0.00	1.00000
Node492	1	0.00 (100.00%)	0.00	1.00000
BRA_KY073269_UFMT_2015	1	0.00 (100.00%)	0.00	1.00000

ITA_FJ005251_239_2008	1	0.00 (100.00%)	0.00	1.00000
ARG_KM236569_Cuba_2013	1	0.00 (100.00%)	0.00	1.00000
Node210	1	0.00 (100.00%)	0.00	1.00000
ARG_JF414818_Arg32_2008	1	0.00 (100.00%)	0.00	1.00000
Node457	1	0.00 (100.00%)	0.00	1.00000
ECU_KF149963_2c_ME10_2012	1	0.00 (100.00%)	0.00	1.00000
ECU_KF149964_2c_ME23_2012	1	0.00 (100.00%)	0.00	1.00000
Node254	1	0.00 (100.00%)	0.00	1.00000
USA_JX475242_WI_18268_2002	1	0.00 (100.00%)	0.00	1.00000
ECU_KF149969_2c_ME31_2012	1	0.00 (100.00%)	0.00	1.00000
Node423	1	0.00 (100.00%)	0.00	1.00000
FRA_DQ025961_2b_03C5_2003	1	0.00 (100.00%)	0.00	1.00000
FRA_DQ025947_2a_02B5_2002	1	0.00 (100.00%)	0.00	1.00000
USA_KJ813852_Bobcat_ND_1170_2013...	1	0.00 (100.00%)	0.00	1.00000
FRA_DQ025943_2a_01S1_2001	1	0.00 (100.00%)	0.00	1.00000
Node399	1	0.00 (100.00%)	0.00	1.00000
CHI_GQ857612_CPV08_04_2008	1	0.00 (100.00%)	0.00	1.00000
CHI_GQ857609_CPV08_01_2008	1	0.00 (100.00%)	0.00	1.00000
ITA_KF373577_2a_714_2001	1	0.00 (100.00%)	0.00	1.00000
FRA_DQ026001_2a_04S32_2004	1	0.00 (100.00%)	0.00	1.00000
CHI_EU483515_2b_ZD13_2007	1	0.00 (100.00%)	0.00	1.00000
VAC_GU212791_vanguard_2009	1	0.00 (100.00%)	0.00	1.00000
Node357	1	0.00 (100.00%)	0.00	1.00000
USA_KJ813842_Bobcat_ND_502_2013	1	0.00 (100.00%)	0.00	1.00000
USA_JX475250_CO_728_2010	1	0.00 (100.00%)	0.00	1.00000
HRV_KP859578_2c_HR859_2014	1	0.00 (100.00%)	0.00	1.00000
Node418	1	0.00 (100.00%)	0.00	1.00000

USA_JX475278_AR_1069_2012	1	0.00 (100.00%)	0.00	1.00000
USA_JX475251_CO_2235_2009	1	0.00 (100.00%)	0.00	1.00000
USA_JX475247_CO_1246_2010	1	0.00 (100.00%)	0.00	1.00000
USA_KJ813882_Raccoon_NJ_1423_201...	1	0.00 (100.00%)	0.00	1.00000
USA_JN867603_2b_Dog_KS_81213_200...	1	0.00 (100.00%)	0.00	1.00000
USA_JN867602_2b_Dog_CA_148743_20...	1	0.00 (100.00%)	0.00	1.00000
CHI_GQ857599_CPV05_04_2005	1	0.00 (100.00%)	0.00	1.00000
FRA_DQ025962_2a_03C6_2003	1	0.00 (100.00%)	0.00	1.00000
HUN_KF539798_H_31_2012	1	0.00 (100.00%)	0.00	1.00000
FRA_DQ025958_2a_03C2_2003	1	0.00 (100.00%)	0.00	1.00000
USA_KJ813851_Bobcat_ND_1168_2013...	1	0.00 (100.00%)	0.00	1.00000
KOR_EU009205_2b_K029_2006	1	0.00 (100.00%)	0.00	1.00000
USA_KJ813873_Graywolf_MI_850_201...	1	0.00 (100.00%)	0.00	1.00000
THA_KP715690_VT28_2014	1	0.00 (100.00%)	0.00	1.00000
ITA_KX434457_987_10_2010	1	0.00 (100.00%)	0.00	1.00000
Node174	1	>1000 (100.00%)	0.36	0.35925
USA_KJ813844_Bobcat_ND_885_2013	1	>1000 (100.00%)	0.37	0.35847
ITA_KF385388_2a_Sicily_X83090_20...	1	>1000 (100.00%)	0.35	0.36200
Node268	1	>1000 (100.00%)	0.37	0.35842
Node281	1	>1000 (100.00%)	0.36	0.35946
Node458	1	>1000 (100.00%)	0.36	0.35919
Node275	1	>1000 (100.00%)	0.37	0.35892
Node169	1	>1000 (100.00%)	0.36	0.35929
Node625	1	>1000 (100.00%)	0.36	0.36034
USA_KJ813848_Bobcat_ND_1162_2013...	1	>1000 (100.00%)	0.37	0.35881
BRA_DQ340431_2a_BR56_1995	1	>1000 (100.00%)	0.37	0.35907
ITA_FJ005240_208_2007	1	>1000 (100.00%)	0.37	0.35881

CHI_KJ194463_raccoondog_HeB10_3_...	1	>1000 (100.00%)	0.35	0.36381
CHI_DQ354068_2a_redpanda_RPPV_20...	1	>1000 (100.00%)	0.37	0.35814
Node490	1	>1000 (100.00%)	0.37	0.35854
Node4	1	>1000 (100.00%)	0.35	0.36373
VIE_AB054219_2b_cat_V209_2000	1	>1000 (100.00%)	0.36	0.35947
ITA_KF373592_2a_329_2008	1	>1000 (100.00%)	0.37	0.35886
CHI_GU569948_2a_CC8601_1986	1	>1000 (100.00%)	0.37	0.35909
CHI_GQ857600_CPV06_01_2006	1	>1000 (100.00%)	0.47	0.33538
Node109	1	>1000 (100.00%)	0.33	0.36743
URU_KM457104_2c_UY47_2006	1	>1000 (100.00%)	0.36	0.35939
Node559	1	>1000 (100.00%)	0.34	0.36570
Node11	1	>1000 (100.00%)	0.36	0.35966
ITA_FJ005195_2c_136_2000	1	>1000 (100.00%)	0.36	0.35967
HUN_KF539803_H_2_2012	1	>1000 (100.00%)	0.34	0.36575
Node110	1	>1000 (100.00%)	0.34	0.36555
ITA_FJ005226_383_2006	1	>1000 (100.00%)	0.36	0.35936
FRA_DQ025954_03B14_2003	1	>1000 (100.00%)	0.36	0.35943
Node8	1	>1000 (100.00%)	0.36	0.35952
ARG_JF414821_Arg48_2009	1	>1000 (100.00%)	0.37	0.35897
Node442	1	>1000 (100.00%)	0.37	0.35887
BRA_DQ340422_2a_BR22_1993	1	>1000 (100.00%)	0.37	0.35894
USA_EU659120_2b_CPV_411a_1998	1	>1000 (100.00%)	0.34	0.36563
TAW_U72695_2a_T4_1996	1	>1000 (100.00%)	0.36	0.35945
Node606	1	>1000 (100.00%)	0.34	0.36607
VIE_AB054221_2b_leopard_V204_200...	1	>1000 (100.00%)	0.37	0.35895
ITA_FJ005248_219_2008	1	>1000 (100.00%)	0.37	0.35886
Node271	1	>1000 (100.00%)	0.46	0.33741

THA_FJ869126_KU5_2008	1	>1000 (100.00%)	0.37	0.35878
USA_KJ813846_Bobcat_ND_974_2013	1	>1000 (100.00%)	0.36	0.35931
USA_KJ813890_Redfox_MA_197_2012	1	>1000 (100.00%)	0.37	0.35904
FRA_DQ025986_2a_04S17_2004	1	>1000 (100.00%)	0.37	0.35831
CHI_GU569939_2a_YN0202_2002	1	>1000 (100.00%)	0.36	0.36008
CHI_GU392239_raccoondog_HB6_2009...	1	>1000 (100.00%)	0.35	0.36386
VAC_FJ222822_2b_FortDodge_2008	1	>1000 (100.00%)	0.35	0.36219
CHI_GU569940_2b_YN0203_2002	1	>1000 (100.00%)	0.36	0.36032
Node431	1	>1000 (100.00%)	0.37	0.35890
Node307	1	>1000 (100.00%)	0.34	0.36574
Node303	1	>1000 (100.00%)	0.38	0.35654
CHI_KJ170679_raccoondog_Heb10_2_...	1	>1000 (100.00%)	0.34	0.36570
VAC_JN625223_INDIA_vac5_2011	1	>1000 (100.00%)	0.34	0.36574
Node608	1	>1000 (100.00%)	0.34	0.36397
USA_JN867605_2b_Dog_US_142805_20...	1	>1000 (100.00%)	0.34	0.36566
AUS_KU508693_2c_LW_2015	1	>1000 (100.00%)	0.36	0.35960
USA_M19296_CPV_N_1988	1	>1000 (100.00%)	0.37	0.35887
Node658	1	>1000 (100.00%)	0.34	0.36582
Node647	1	>1000 (100.00%)	0.35	0.36292
Node666	1	>1000 (100.00%)	0.36	0.35930
Node673	1	>1000 (100.00%)	0.34	0.36442
Node631	1	>1000 (100.00%)	0.34	0.36422
CHI_FJ432718_CPV_Cv_2008	1	>1000 (100.00%)	0.34	0.36424
Node639	1	>1000 (100.00%)	0.35	0.36289
Node623	1	>1000 (100.00%)	0.36	0.36067
Node245	1	1.00 (100.00%)	0.00	1.00000
Node243	1	1.00 (100.00%)	0.00	1.00000

```
|             Node242             |      1     |    1.00 (100.00%)
|        0.00        |       1.00000       |
|             Node244             |      1     |    1.00 (100.00%)
|        0.00        |       1.00000       |
|             Node241             |      1     |    1.00 (100.00%)
|        0.00        |       1.00000       |
|             Node320             |      1     |    1.00 (100.00%)
|        0.00        |       1.00000       |
|             Node32              |      1     |    1.00 (100.00%)
|        0.00        |       1.00000       |
|             Node337             |      1     |    1.00 (100.00%)
|        0.00        |       1.00000       |
|             Node158             |      1     |    1.00 (100.00%)
|        0.00        |       1.00000       |
|             Node333             |      1     |    1.00 (100.00%)
|        0.00        |       1.00000       |
|             Node339             |      1     |    1.00 (100.00%)
|        0.00        |       1.00000       |
|             Node162             |      1     |    1.00 (100.00%)
|        0.00        |       1.00000       |
|             Node16              |      1     |    1.00 (100.00%)
|        0.00        |       1.00000       |
|             Node33              |      1     |    1.00 (100.00%)
|        0.00        |       1.00000       |
|             Node300             |      1     |    1.00 (100.00%)
|        0.00        |       1.00000       |
|             Node239             |      1     |    1.00 (100.00%)
|        0.00        |       1.00000       |
|             Node240             |      1     |    1.00 (100.00%)
|        0.00        |       1.00000       |
|             Node24              |      1     |    1.00 (100.00%)
|        0.00        |       1.00000       |
|    HRV_KP859574_2c_HR442_2014    |      1     |    1.00 (100.00%)
|        0.00        |       1.00000       |
|             Node34              |      1     |    1.00 (100.00%)
|        0.00        |       1.00000       |
|       GER_AY742935_U6_1995       |      1     |    1.00 (100.00%)
|        0.00        |       1.00000       |
|             Node344             |      1     |    1.00 (100.00%)
|        0.00        |       1.00000       |
|             Node343             |      1     |    1.00 (100.00%)
|        0.00        |       1.00000       |
|             Node157             |      1     |    1.00 (100.00%)
|        0.00        |       1.00000       |
|             Node154             |      1     |    1.00 (100.00%)
|        0.00        |       1.00000       |
|             Node310             |      1     |    1.00 (100.00%)
|        0.00        |       1.00000       |
|             Node305             |      1     |    1.00 (100.00%)
|        0.00        |       1.00000       |
|             Node31              |      1     |    1.00 (100.00%)
|        0.00        |       1.00000       |
|             Node314             |      1     |    1.00 (100.00%)
|        0.00        |       1.00000       |
```

```
|                Node318              |      1     |    1.00 (100.00%)
|         0.00         |        1.00000       |
|                Node156              |      1     |    1.00 (100.00%)
|         0.00         |        1.00000       |
|                Node155              |      1     |    1.00 (100.00%)
|         0.00         |        1.00000       |
|                Node316              |      1     |    1.00 (100.00%)
|         0.00         |        1.00000       |
|                Node238              |      1     |    1.00 (100.00%)
|         0.00         |        1.00000       |
|                Node28               |      1     |    1.00 (100.00%)
|         0.00         |        1.00000       |
|                Node280              |      1     |    1.00 (100.00%)
|         0.00         |        1.00000       |
|                Node282              |      1     |    1.00 (100.00%)
|         0.00         |        1.00000       |
|                Node151              |      1     |    1.00 (100.00%)
|         0.00         |        1.00000       |
|                Node279              |      1     |    1.00 (100.00%)
|         0.00         |        1.00000       |
|                Node293              |      1     |    1.00 (100.00%)
|         0.00         |        1.00000       |
|                Node152              |      1     |    1.00 (100.00%)
|         0.00         |        1.00000       |
|                Node278              |      1     |    1.00 (100.00%)
|         0.00         |        1.00000       |
|                Node29               |      1     |    1.00 (100.00%)
|         0.00         |        1.00000       |
|                Node283              |      1     |    1.00 (100.00%)
|         0.00         |        1.00000       |
|                Node234              |      1     |    1.00 (100.00%)
|         0.00         |        1.00000       |
|                Node235              |      1     |    1.00 (100.00%)
|         0.00         |        1.00000       |
|                Node237              |      1     |    1.00 (100.00%)
|         0.00         |        1.00000       |
|                Node236              |      1     |    1.00 (100.00%)
|         0.00         |        1.00000       |
|                Node233              |      1     |    1.00 (100.00%)
|         0.00         |        1.00000       |
|                Node23               |      1     |    1.00 (100.00%)
|         0.00         |        1.00000       |
|                Node188              |      1     |    1.00 (100.00%)
|         0.00         |        1.00000       |
|                Node232              |      1     |    1.00 (100.00%)
|         0.00         |        1.00000       |
|                Node231              |      1     |    1.00 (100.00%)
|         0.00         |        1.00000       |
|                Node30               |      1     |    1.00 (100.00%)
|         0.00         |        1.00000       |
|                Node153              |      1     |    1.00 (100.00%)
|         0.00         |        1.00000       |
|                Node286              |      1     |    1.00 (100.00%)
|         0.00         |        1.00000       |
```

```
|              Node284              |     1    |   1.00 (100.00%)
|        0.00        |       1.00000     |
|              Node285              |     1    |   1.00 (100.00%)
|        0.00        |       1.00000     |
|              Node274              |     1    |   1.00 (100.00%)
|        0.00        |       1.00000     |
|              Node150              |     1    |   1.00 (100.00%)
|        0.00        |       1.00000     |
|              Node25               |     1    |   1.00 (100.00%)
|        0.00        |       1.00000     |
|              Node256              |     1    |   1.00 (100.00%)
|        0.00        |       1.00000     |
|              Node27               |     1    |   1.00 (100.00%)
|        0.00        |       1.00000     |
|              Node260              |     1    |   1.00 (100.00%)
|        0.00        |       1.00000     |
|              Node26               |     1    |   1.00 (100.00%)
|        0.00        |       1.00000     |
|              Node186              |     1    |   1.00 (100.00%)
|        0.00        |       1.00000     |
|              Node204              |     1    |   1.00 (100.00%)
|        0.00        |       1.00000     |
|              Node198              |     1    |   1.00 (100.00%)
|        0.00        |       1.00000     |
|              Node21               |     1    |   1.00 (100.00%)
|        0.00        |       1.00000     |
|              Node202              |     1    |   1.00 (100.00%)
|        0.00        |       1.00000     |
|               Node2               |     1    |   1.00 (100.00%)
|        0.00        |       1.00000     |
|              Node20               |     1    |   1.00 (100.00%)
|        0.00        |       1.00000     |
|              Node200              |     1    |   1.00 (100.00%)
|        0.00        |       1.00000     |
|              Node196              |     1    |   1.00 (100.00%)
|        0.00        |       1.00000     |
|              Node184              |     1    |   1.00 (100.00%)
|        0.00        |       1.00000     |
|              Node225              |     1    |   1.00 (100.00%)
|        0.00        |       1.00000     |
|              Node228              |     1    |   1.00 (100.00%)
|        0.00        |       1.00000     |
|              Node182              |     1    |   1.00 (100.00%)
|        0.00        |       1.00000     |
|              Node192              |     1    |   1.00 (100.00%)
|        0.00        |       1.00000     |
|              Node180              |     1    |   1.00 (100.00%)
|        0.00        |       1.00000     |
|              Node22               |     1    |   1.00 (100.00%)
|        0.00        |       1.00000     |
|              Node218              |     1    |   1.00 (100.00%)
|        0.00        |       1.00000     |
|    HRV_KP859575_2c_HR774_2014     |     1    |   1.00 (100.00%)
|        0.00        |       1.00000     |
```

ITA_FJ005209_2c_303_2004	1	1.00 (100.00%)	0.00	1.00000
ITA_FJ005233_40_2007	1	1.00 (100.00%)	0.00	1.00000
ITA_FJ005258_80_2008	1	1.00 (100.00%)	0.00	1.00000
ITA_FJ005206_2c_287_2004	1	1.00 (100.00%)	0.00	1.00000
Node10	1	1.00 (100.00%)	0.00	1.00000
Node1	1	1.00 (100.00%)	0.00	1.00000
ITA_FJ005205_2c_279_2004	1	1.00 (100.00%)	0.00	1.00000
ITA_FJ005263_42_2005	1	1.00 (100.00%)	0.00	1.00000
ITA_KF373611_2a_409_2010	1	1.00 (100.00%)	0.00	1.00000
ITA_KX434454_29451_09_2009	1	1.00 (100.00%)	0.00	1.00000
ITA_KU508407_2c_25835_09_2009	1	1.00 (100.00%)	0.00	1.00000
ITA_KX434458_2323_11_2011	1	1.00 (100.00%)	0.00	1.00000
ITA_FJ222821_2c_56_2000	1	1.00 (100.00%)	0.00	1.00000
ITA_FJ222824_388_05_3_2005	1	1.00 (100.00%)	0.00	1.00000
ITA_KF373580_2a_581_2003	1	1.00 (100.00%)	0.00	1.00000
ITA_KF373571_2a_685_1999	1	1.00 (100.00%)	0.00	1.00000
ITA_AF393506_2a_699_2000	1	1.00 (100.00%)	0.00	1.00000
HUN_KF539793_H_5_2012	1	1.00 (100.00%)	0.00	1.00000
HUN_KF539794_H_7_2012	1	1.00 (100.00%)	0.00	1.00000
Node36	1	1.00 (100.00%)	0.00	1.00000
Node355	1	1.00 (100.00%)	0.00	1.00000
Node35	1	1.00 (100.00%)	0.00	1.00000
Node352	1	1.00 (100.00%)	0.00	1.00000
Node354	1	1.00 (100.00%)	0.00	1.00000
HRV_KP859576_2c_HR793_2014	1	1.00 (100.00%)	0.00	1.00000
Node361	1	1.00 (100.00%)	0.00	1.00000
Node105	1	1.00 (100.00%)	0.00	1.00000

```
|        ITA_AF306447_618_2000       |     1    |   1.00 (100.00%)
|        0.00        |       1.00000       |
|              Node103               |     1    |   1.00 (100.00%)
|        0.00        |       1.00000       |
|      HUN_KF539805_H_36_2012        |     1    |   1.00 (100.00%)
|        0.00        |       1.00000       |
|              Node368               |     1    |   1.00 (100.00%)
|        0.00        |       1.00000       |
|              Node37                |     1    |   1.00 (100.00%)
|        0.00        |       1.00000       |
|              Node115               |     1    |   1.00 (100.00%)
|        0.00        |       1.00000       |
|              Node370               |     1    |   1.00 (100.00%)
|        0.00        |       1.00000       |
|              Node194               |     1    |   1.00 (100.00%)
|        0.00        |       1.00000       |
|              Node190               |     1    |   1.00 (100.00%)
|        0.00        |       1.00000       |
|              Node19                |     1    |   1.00 (100.00%)
|        0.00        |       1.00000       |
|              Node18                |     1    |   1.00 (100.00%)
|        0.00        |       1.00000       |
|              Node17                |     1    |   1.00 (100.00%)
|        0.00        |       1.00000       |
|              Node178               |     1    |   1.00 (100.00%)
|        0.00        |       1.00000       |
|              Node15                |     1    |   1.00 (100.00%)
|        0.00        |       1.00000       |
|              Node136               |     1    |   1.00 (100.00%)
|        0.00        |       1.00000       |
|              Node13                |     1    |   1.00 (100.00%)
|        0.00        |       1.00000       |
|              Node118               |     1    |   1.00 (100.00%)
|        0.00        |       1.00000       |
|              Node137               |     1    |   1.00 (100.00%)
|        0.00        |       1.00000       |
|              Node146               |     1    |   1.00 (100.00%)
|        0.00        |       1.00000       |
|              Node144               |     1    |   1.00 (100.00%)
|        0.00        |       1.00000       |
|              Node14                |     1    |   1.00 (100.00%)
|        0.00        |       1.00000       |
|              Node142               |     1    |   1.00 (100.00%)
|        0.00        |       1.00000       |
|    FRA_DQ025991_2b_04S22_2004      |     1    |   1.00 (100.00%)
|        0.00        |       1.00000       |
|     BRA_DQ340414_2a_BR31_1990      |     1    |   1.00 (100.00%)
|        0.00        |       1.00000       |
|    BRA_DQ340408_2a_BR154_1980      |     1    |   1.00 (100.00%)
|        0.00        |       1.00000       |
|     BRA_DQ340416_2a_BR47_1991      |     1    |   1.00 (100.00%)
|        0.00        |       1.00000       |
|    BRA_DQ340424_2a_BR137_1993      |     1    |   1.00 (100.00%)
|        0.00        |       1.00000       |
```

```
|     BRA_DQ340418_2a_BR491_1992      |      1     |   1.00 (100.00%)
|        0.00         |       1.00000       |
|      BRA_DQ340417_2a_BR52_1991      |      1     |   1.00 (100.00%)
|        0.00         |       1.00000       |
|     BRA_DQ340407_2a_BR145_1980      |      1     |   1.00 (100.00%)
|        0.00         |       1.00000       |
|       ARG_KM236572_NNGag_2012       |      1     |   1.00 (100.00%)
|        0.00         |       1.00000       |
|       ARG_JF414820_Arg44_2009       |      1     |   1.00 (100.00%)
|        0.00         |       1.00000       |
|       AUS_KU508691_2c_HB_2015       |      1     |   1.00 (100.00%)
|        0.00         |       1.00000       |
|     BRA_DQ340405_2a_BR135_1980      |      1     |   1.00 (100.00%)
|        0.00         |       1.00000       |
|      BRA_DQ340404_2a_BR6_1980       |      1     |   1.00 (100.00%)
|        0.00         |       1.00000       |
|       AUS_KU508692_2c_FH_2015       |      1     |   1.00 (100.00%)
|        0.00         |       1.00000       |
|      BRA_DQ340426_2a_BR84_1994      |      1     |   1.00 (100.00%)
|        0.00         |       1.00000       |
|      FRA_DQ025944_2a_02B2_2002      |      1     |   1.00 (100.00%)
|        0.00         |       1.00000       |
|       FRA_DQ025942_01B1_2001        |      1     |   1.00 (100.00%)
|        0.00         |       1.00000       |
|      FRA_DQ025945_2a_02B3_2002      |      1     |   1.00 (100.00%)
|        0.00         |       1.00000       |
|     FRA_DQ025984_2a_04S15_2004      |      1     |   1.00 (100.00%)
|        0.00         |       1.00000       |
|     FRA_DQ025983_2a_04S14_2004      |      1     |   1.00 (100.00%)
|        0.00         |       1.00000       |
|      FRA_DQ025950_2a_02B9_2002      |      1     |   1.00 (100.00%)
|        0.00         |       1.00000       |
|FIN_U22192_raccoondog_RD_80_1980...|      1     |   1.00 (100.00%)
|        0.00         |       1.00000       |
|      CHI_GU392236_fox_HB1_2009      |      1     |   1.00 (100.00%)
|        0.00         |       1.00000       |
|     BRA_DQ340428_2a_BR209_1994      |      1     |   1.00 (100.00%)
|        0.00         |       1.00000       |
|     BRA_DQ340427_2a_BR133_1994      |      1     |   1.00 (100.00%)
|        0.00         |       1.00000       |
|CHI_GU392240_raccoondog_HB3_2009...|      1     |   1.00 (100.00%)
|        0.00         |       1.00000       |
|      ECU_KF149962_2c_ME1_2012       |      1     |   1.00 (100.00%)
|        0.00         |       1.00000       |
|    CHI_KF803602_2010_BJ_A72_2010    |      1     |   1.00 (100.00%)
|        0.00         |       1.00000       |
|     CHI_GU569942_2a_JL0202_2002     |      1     |   1.00 (100.00%)
|        0.00         |       1.00000       |
|               Node372               |      1     |   1.00 (100.00%)
|        0.00         |       1.00000       |
|        THA_FJ869125_KU5_2004        |      1     |   1.00 (100.00%)
|        0.00         |       1.00000       |
|       THA_FJ869130_KU13_2004        |      1     |   1.00 (100.00%)
|        0.00         |       1.00000       |
```

THA_FJ869137_KU52_2003	1	1.00 (100.00%)	0.00	1.00000
THA_FJ869134_KU23_2003	1	1.00 (100.00%)	0.00	1.00000
THA_FJ869123_KU3_2008	1	1.00 (100.00%)	0.00	1.00000
POR_KU662349_greywolf_W33_1996	1	1.00 (100.00%)	0.00	1.00000
POL_Z46651_46_1994	1	1.00 (100.00%)	0.00	1.00000
SAF_HQ602969_22_10SA_2010	1	1.00 (100.00%)	0.00	1.00000
TAW_U72696_2b_T10_1996	1	1.00 (100.00%)	0.00	1.00000
TAW_FJ265781_CPV307_2005	1	1.00 (100.00%)	0.00	1.00000
THA_FJ869138_KU53_2003	1	1.00 (100.00%)	0.00	1.00000
URU_KC196093_2c_M307_2011	1	1.00 (100.00%)	0.00	1.00000
URU_KC196091_2c_M326_2011	1	1.00 (100.00%)	0.00	1.00000
URU_KC196096_2c_M247_2010	1	1.00 (100.00%)	0.00	1.00000
URU_KC196101_2c_M187_2009	1	1.00 (100.00%)	0.00	1.00000
URU_KC196097_2c_M242_2010	1	1.00 (100.00%)	0.00	1.00000
URU_KC196089_2c_M349_2011	1	1.00 (100.00%)	0.00	1.00000
THA_KP715716_VT143_2014	1	1.00 (100.00%)	0.00	1.00000
THA_KP715691_VT43_2014	1	1.00 (100.00%)	0.00	1.00000
URU_KC196081_2c_M95_2007	1	1.00 (100.00%)	0.00	1.00000
URU_KC196086_2c_M55_2006	1	1.00 (100.00%)	0.00	1.00000
URU_KC196083_2c_M82_2007	1	1.00 (100.00%)	0.00	1.00000
Node98	1	1.00 (100.00%)	0.00	1.00000
Node627	1	1.00 (100.00%)	0.00	1.00000
Node619	1	1.00 (100.00%)	0.00	1.00000
Node629	1	1.00 (100.00%)	0.00	1.00000
Node638	1	1.00 (100.00%)	0.00	1.00000
Node635	1	1.00 (100.00%)	0.00	1.00000
Node617	1	1.00 (100.00%)	0.00	1.00000

Node607	1	1.00 (100.00%)	0.00	1.00000
Node605	1	1.00 (100.00%)	0.00	1.00000
Node609	1	1.00 (100.00%)	0.00	1.00000
Node611	1	1.00 (100.00%)	0.00	1.00000
Node610	1	1.00 (100.00%)	0.00	1.00000
Node648	1	1.00 (100.00%)	0.00	1.00000
Node88	1	1.00 (100.00%)	0.00	1.00000
Node86	1	1.00 (100.00%)	0.00	1.00000
Node9	1	1.00 (100.00%)	0.00	1.00000
Node95	1	1.00 (100.00%)	0.00	1.00000
Node93	1	1.00 (100.00%)	0.00	1.00000
Node7	1	1.00 (100.00%)	0.00	1.00000
Node650	1	1.00 (100.00%)	0.00	1.00000
Node649	1	1.00 (100.00%)	0.00	1.00000
Node651	1	1.00 (100.00%)	0.00	1.00000
Node665	1	1.00 (100.00%)	0.00	1.00000
Node655	1	1.00 (100.00%)	0.00	1.00000
URU_KC196102_2c_M185_2009	1	1.00 (100.00%)	0.00	1.00000
USA_JX475240_AZ_16382_01_1999	1	1.00 (100.00%)	0.00	1.00000
USA_JX475237_CT_372_2011	1	1.00 (100.00%)	0.00	1.00000
USA_JX475284_TN_26_2011	1	1.00 (100.00%)	0.00	1.00000
USA_KJ813831_Fisher_ND_17_2013	1	1.00 (100.00%)	0.00	1.00000
USA_KJ813827_Fisher_F1M111211_20...	1	1.00 (100.00%)	0.00	1.00000
USA_JX475231_CO_280_2011	1	1.00 (100.00%)	0.00	1.00000
USA_EU659119_2b_CPV_410_2000	1	1.00 (100.00%)	0.00	1.00000
USA_EU659116_CPV_5_1979	1	1.00 (100.00%)	0.00	1.00000
USA_JN867598_Bobcat_KS_44_2010	1	1.00 (100.00%)	0.00	1.00000

USA_JN867618_Raccoon_WI_37_2010	1	1.00 (100.00%)	0.00	1.00000
USA_JN867599_Raccoon_KY_39552_20...	1	1.00 (100.00%)	0.00	1.00000
USA_KJ813888_Coyote_MT_878_2012	1	1.00 (100.00%)	0.00	1.00000
VAC_KY083089_Singapore_2016	1	1.00 (100.00%)	0.00	1.00000
VAC_FJ197847_Pfizer_2007	1	1.00 (100.00%)	0.00	1.00000
VIE_AB054218_2b_cat_V123_2000	1	1.00 (100.00%)	0.00	1.00000
VIE_AB120725_2b_HNI_3_4_2003	1	1.00 (100.00%)	0.00	1.00000
VIE_AB120724_2b_HNI_2_13_2003	1	1.00 (100.00%)	0.00	1.00000
USA_U22186_CPV_128_1995	1	1.00 (100.00%)	0.00	1.00000
USA_M24003_FPV_CPV_15_1988	1	1.00 (100.00%)	0.00	1.00000
USA_M23255_FPV_Cornell320_1988	1	1.00 (100.00%)	0.00	1.00000
USA_M38245_1990	1	1.00 (100.00%)	0.00	1.00000
USA_M74852_133_1995	1	1.00 (100.00%)	0.00	1.00000
USA_M74849_39_1995	1	1.00 (100.00%)	0.00	1.00000
USA_AY742955_436_2003	1	1.00 (100.00%)	0.00	1.00000
URU_KM457113_2c_UY152_2009	1	1.00 (100.00%)	0.00	1.00000
URU_KM457112_2c_UY135_2008	1	1.00 (100.00%)	0.00	1.00000
URU_KM457116_2c_UY185_2009	1	1.00 (100.00%)	0.00	1.00000
URU_KM457120_2c_UY242_2010	1	1.00 (100.00%)	0.00	1.00000
URU_KM457117_2c_UY187_2009	1	1.00 (100.00%)	0.00	1.00000
URU_KM457111_2c_UY120_2008	1	1.00 (100.00%)	0.00	1.00000
URU_KM457106_2c_UY55_2006	1	1.00 (100.00%)	0.00	1.00000
URU_KC196105_2c_M152_2008	1	1.00 (100.00%)	0.00	1.00000
URU_KM457107_2c_UY72_2007	1	1.00 (100.00%)	0.00	1.00000
URU_KM457109_2c_UY95_2007	1	1.00 (100.00%)	0.00	1.00000
URU_KM457108_2c_UY82_2007	1	1.00 (100.00%)	0.00	1.00000
URU_KM457121_2c_UY247_2010	1	1.00 (100.00%)	0.00	1.00000

```
|     URU_KM457142_2c_UY370_2011      |      1     |   1.00 (100.00%)
|       0.00        |       1.00000       |
|     URU_KM457130_2c_UY354_2011      |      1     |   1.00 (100.00%)
|       0.00        |       1.00000       |
|        USA_AY742932_193_1991        |      1     |   1.00 (100.00%)
|       0.00        |       1.00000       |
|        USA_AY742951_431_2003        |      1     |   1.00 (100.00%)
|       0.00        |       1.00000       |
|        USA_AY742936_395_1998        |      1     |   1.00 (100.00%)
|       0.00        |       1.00000       |
|     URU_KM457129_2c_UY349_2011      |      1     |   1.00 (100.00%)
|       0.00        |       1.00000       |
|     URU_KM457123_2c_UY261_2008      |      1     |   1.00 (100.00%)
|       0.00        |       1.00000       |
|     URU_KM457122_2c_UY258_2010      |      1     |   1.00 (100.00%)
|       0.00        |       1.00000       |
|     URU_KM457124_2c_UY307_2011      |      1     |   1.00 (100.00%)
|       0.00        |       1.00000       |
|     URU_KM457127_2c_UY326_2011      |      1     |   1.00 (100.00%)
|       0.00        |       1.00000       |
|     URU_KM457125_2c_UY317_2011      |      1     |   1.00 (100.00%)
|       0.00        |       1.00000       |
|             Node603                 |      1     |   1.00 (100.00%)
|       0.00        |       1.00000       |
|             Node44                  |      1     |   1.00 (100.00%)
|       0.00        |       1.00000       |
|             Node440                 |      1     |   1.00 (100.00%)
|       0.00        |       1.00000       |
|             Node443                 |      1     |   1.00 (100.00%)
|       0.00        |       1.00000       |
|             Node441                 |      1     |   1.00 (100.00%)
|       0.00        |       1.00000       |
|             Node436                 |      1     |   1.00 (100.00%)
|       0.00        |       1.00000       |
|             Node428                 |      1     |   1.00 (100.00%)
|       0.00        |       1.00000       |
|             Node426                 |      1     |   1.00 (100.00%)
|       0.00        |       1.00000       |
|             Node43                  |      1     |   1.00 (100.00%)
|       0.00        |       1.00000       |
|             Node432                 |      1     |   1.00 (100.00%)
|       0.00        |       1.00000       |
|             Node430                 |      1     |   1.00 (100.00%)
|       0.00        |       1.00000       |
|             Node444                 |      1     |   1.00 (100.00%)
|       0.00        |       1.00000       |
|             Node468                 |      1     |   1.00 (100.00%)
|       0.00        |       1.00000       |
|             Node466                 |      1     |   1.00 (100.00%)
|       0.00        |       1.00000       |
|             Node47                  |      1     |   1.00 (100.00%)
|       0.00        |       1.00000       |
|             Node472                 |      1     |   1.00 (100.00%)
|       0.00        |       1.00000       |
```

```
|              Node470              |     1    |    1.00 (100.00%)
|        0.00        |       1.00000       |
|              Node464              |     1    |    1.00 (100.00%)
|        0.00        |       1.00000       |
|              Node453              |     1    |    1.00 (100.00%)
|        0.00        |       1.00000       |
|              Node45               |     1    |    1.00 (100.00%)
|        0.00        |       1.00000       |
|              Node456              |     1    |    1.00 (100.00%)
|        0.00        |       1.00000       |
|              Node462              |     1    |    1.00 (100.00%)
|        0.00        |       1.00000       |
|              Node46               |     1    |    1.00 (100.00%)
|        0.00        |       1.00000       |
|              Node420              |     1    |    1.00 (100.00%)
|        0.00        |       1.00000       |
|              Node39               |     1    |    1.00 (100.00%)
|        0.00        |       1.00000       |
|              Node384              |     1    |    1.00 (100.00%)
|        0.00        |       1.00000       |
|              Node390              |     1    |    1.00 (100.00%)
|        0.00        |       1.00000       |
|              Node396              |     1    |    1.00 (100.00%)
|        0.00        |       1.00000       |
|              Node393              |     1    |    1.00 (100.00%)
|        0.00        |       1.00000       |
|              Node382              |     1    |    1.00 (100.00%)
|        0.00        |       1.00000       |
|              Node376              |     1    |    1.00 (100.00%)
|        0.00        |       1.00000       |
|              Node374              |     1    |    1.00 (100.00%)
|        0.00        |       1.00000       |
|              Node378              |     1    |    1.00 (100.00%)
|        0.00        |       1.00000       |
|              Node380              |     1    |    1.00 (100.00%)
|        0.00        |       1.00000       |
|              Node38               |     1    |    1.00 (100.00%)
|        0.00        |       1.00000       |
|              Node40               |     1    |    1.00 (100.00%)
|        0.00        |       1.00000       |
|              Node407              |     1    |    1.00 (100.00%)
|        0.00        |       1.00000       |
|              Node406              |     1    |    1.00 (100.00%)
|        0.00        |       1.00000       |
|              Node41               |     1    |    1.00 (100.00%)
|        0.00        |       1.00000       |
|              Node42               |     1    |    1.00 (100.00%)
|        0.00        |       1.00000       |
|              Node412              |     1    |    1.00 (100.00%)
|        0.00        |       1.00000       |
|              Node405              |     1    |    1.00 (100.00%)
|        0.00        |       1.00000       |
|              Node401              |     1    |    1.00 (100.00%)
|        0.00        |       1.00000       |
```

```
|                Node400                |      1     |    1.00 (100.00%)
|         0.00        |        1.00000        |
|                Node402                |      1     |    1.00 (100.00%)
|         0.00        |        1.00000        |
|                Node404                |      1     |    1.00 (100.00%)
|         0.00        |        1.00000        |
|                Node403                |      1     |    1.00 (100.00%)
|         0.00        |        1.00000        |
|                Node474                |      1     |    1.00 (100.00%)
|         0.00        |        1.00000        |
|                Node547                |      1     |    1.00 (100.00%)
|         0.00        |        1.00000        |
|                Node543                |      1     |    1.00 (100.00%)
|         0.00        |        1.00000        |
|                Node551                |      1     |    1.00 (100.00%)
|         0.00        |        1.00000        |
|                Node555                |      1     |    1.00 (100.00%)
|         0.00        |        1.00000        |
|                Node553                |      1     |    1.00 (100.00%)
|         0.00        |        1.00000        |
|                Node541                |      1     |    1.00 (100.00%)
|         0.00        |        1.00000        |
|                Node531                |      1     |    1.00 (100.00%)
|         0.00        |        1.00000        |
|                Node529                |      1     |    1.00 (100.00%)
|         0.00        |        1.00000        |
|                Node533                |      1     |    1.00 (100.00%)
|         0.00        |        1.00000        |
|                Node537                |      1     |    1.00 (100.00%)
|         0.00        |        1.00000        |
|                Node535                |      1     |    1.00 (100.00%)
|         0.00        |        1.00000        |
|                Node557                |      1     |    1.00 (100.00%)
|         0.00        |        1.00000        |
|                Node592                |      1     |    1.00 (100.00%)
|         0.00        |        1.00000        |
|                Node590                |      1     |    1.00 (100.00%)
|         0.00        |        1.00000        |
|                Node594                |      1     |    1.00 (100.00%)
|         0.00        |        1.00000        |
|                 Node6                 |      1     |    1.00 (100.00%)
|         0.00        |        1.00000        |
|                Node596                |      1     |    1.00 (100.00%)
|         0.00        |        1.00000        |
|                Node588                |      1     |    1.00 (100.00%)
|         0.00        |        1.00000        |
|                Node568                |      1     |    1.00 (100.00%)
|         0.00        |        1.00000        |
|                Node566                |      1     |    1.00 (100.00%)
|         0.00        |        1.00000        |
|                Node570                |      1     |    1.00 (100.00%)
|         0.00        |        1.00000        |
|                Node581                |      1     |    1.00 (100.00%)
|         0.00        |        1.00000        |
```

```
|              Node572              |     1    |   1.00 (100.00%)
|        0.00        |       1.00000       |
|              Node524              |     1    |   1.00 (100.00%)
|        0.00        |       1.00000       |
|              Node49               |     1    |   1.00 (100.00%)
|        0.00        |       1.00000       |
|              Node484              |     1    |   1.00 (100.00%)
|        0.00        |       1.00000       |
|              Node494              |     1    |   1.00 (100.00%)
|        0.00        |       1.00000       |
|               Node5               |     1    |   1.00 (100.00%)
|        0.00        |       1.00000       |
|              Node499              |     1    |   1.00 (100.00%)
|        0.00        |       1.00000       |
|              Node483              |     1    |   1.00 (100.00%)
|        0.00        |       1.00000       |
|              Node479              |     1    |   1.00 (100.00%)
|        0.00        |       1.00000       |
|              Node477              |     1    |   1.00 (100.00%)
|        0.00        |       1.00000       |
|              Node48               |     1    |   1.00 (100.00%)
|        0.00        |       1.00000       |
|              Node482              |     1    |   1.00 (100.00%)
|        0.00        |       1.00000       |
|              Node480              |     1    |   1.00 (100.00%)
|        0.00        |       1.00000       |
|              Node50               |     1    |   1.00 (100.00%)
|        0.00        |       1.00000       |
|              Node52               |     1    |   1.00 (100.00%)
|        0.00        |       1.00000       |
|              Node519              |     1    |   1.00 (100.00%)
|        0.00        |       1.00000       |
|              Node521              |     1    |   1.00 (100.00%)
|        0.00        |       1.00000       |
|              Node523              |     1    |   1.00 (100.00%)
|        0.00        |       1.00000       |
|              Node522              |     1    |   1.00 (100.00%)
|        0.00        |       1.00000       |
|              Node513              |     1    |   1.00 (100.00%)
|        0.00        |       1.00000       |
|              Node506              |     1    |   1.00 (100.00%)
|        0.00        |       1.00000       |
|              Node505              |     1    |   1.00 (100.00%)
|        0.00        |       1.00000       |
|              Node509              |     1    |   1.00 (100.00%)
|        0.00        |       1.00000       |
|              Node512              |     1    |   1.00 (100.00%)
|        0.00        |       1.00000       |
|              Node51               |     1    |   1.00 (100.00%)
|        0.00        |       1.00000       |
```

Adaptive branch site random effects likelihood test
Likelihood ratio test for episodic diversifying positive selection
at Holm-Bonferroni corrected _p =   0.0500_ found **1** branches

under selection among **675** tested.

* VAC_KY083090_Singapore_2016, p-value = 0.00186
