# Supplementary material for: Evidence of CPV2c introgression into Croatia and novel insights into phylogeny and cell tropism
Source: Sci Rep. 2019 Nov 15;9:16909. doi: 10.1038/s41598-019-53422-9 (PMC6858334; doi:10.1038/s41598-019-53422-9)
Supplement: Supplementary file 8 — Detect relaxed selection in codon-based phylogenetic framework [file 41598_2019_53422_MOESM8_ESM.pdf]

Evidence of CPV2c introgression into Croatia and novel insights into phylogeny and cell tropism

Dinko Novosel, Tamas Tuboly, Gyula Balka, Levente Szeredi, Ivana Lojkic, Andreja Jungic, Zaklin Acinger Rogic, Tahar Ait Ali, Attila Csagola

Supplementary info file 8.

Results of selection using Random Effects Test of Selection Relaxation method

Analysis Description

RELAX (a random effects test of selection relaxation) uses a random effects branch-site model framework to test whether a set of 'Test' branches evolves under relaxed selection relative to a set of 'Reference' branches (R), as measured by the relaxation parameter (K). Version 2.1 adds a check for stability in K estimates to try to mitigate convergence problems

- __Requirements__: in-frame codon alignment and a phylogenetic tree, with at least two
groups of branches defined using the {} notation (one group can be defined as all unlabeled branches)

- __Citation__: RELAX: Detecting Relaxed Selection in a Phylogenetic Framework (2015).
Mol Biol Evol 32 (3): 820-832

- __Written by__: Sergei L Kosakovsky Pond, Ben Murrell, Steven Weaver and Temple iGEM /
UCSD viral evolution group

- __Contact Information__: spond@temple.edu

- __Analysis Version__: 2.1

####Choose Genetic Code

1. [**Universal**] Universal code. (Genebank transl_table=1).
2. [**Vertebrate mtDNA**] Vertebrate mitochondrial DNA code. (Genebank transl_table=2).
3. [**Yeast mtDNA**] Yeast mitochondrial DNA code. (Genebank transl_table=3).
4. [**Mold/Protozoan mtDNA**] Mold, Protozoan and Coelenterate mitochondrial DNA and the Mycloplasma/Spiroplasma code. (Genebank transl_table=4).
5. [**Invertebrate mtDNA**] Invertebrate mitochondrial DNA code. (Genebank transl_table=5).
6. [**Ciliate Nuclear**] Ciliate, Dasycladacean and Hexamita Nuclear code. (Genebank transl_table=6).
7. [**Echinoderm mtDNA**] Echinoderm mitochondrial DNA code. (Genebank

transl_table=9).
8. [**Euplotid Nuclear**] Euplotid Nuclear code. (Genebank transl_table=10).
9. [**Alt. Yeast Nuclear**] Alternative Yeast Nuclear code. (Genebank transl_table=12).
10. [**Ascidian mtDNA**] Ascidian mitochondrial DNA code. (Genebank transl_table=13).
11. [**Flatworm mtDNA**] Flatworm mitochondrial DNA code. (Genebank transl_table=14).
12. [**Blepharisma Nuclear**] Blepharisma Nuclear code. (Genebank transl_table=15).
13. [**Chlorophycean mtDNA**] Chlorophycean Mitochondrial Code (transl_table=16).
14. [**Trematode mtDNA**] Trematode Mitochondrial Code (transl_table=21).
15. [**Scenedesmus obliquus mtDNA**] Scenedesmus obliquus mitochondrial Code (transl_table=22).
16. [**Thraustochytrium mtDNA**] Thraustochytrium Mitochondrial Code (transl_table=23).
17. [**Pterobranchia mtDNA**] Pterobranchia Mitochondrial Code (transl_table=24).
18. [**SR1 and Gracilibacteria**] Candidate Division SR1 and Gracilibacteria Code (transl_table=25).
19. [**Pachysolen Nuclear**] Pachysolen tannophilus Nuclear Code (transl_table=26).

>Please choose an option (or press q to cancel selection):

>Select a coding sequence alignment file (`/home/datamonkey/datamonkey-js-server/production/app/relax/../../.hyphy/res//TemplateBatchFiles/SelectionAnalyses/`)

>Please select a tree file for the data: (`/home/datamonkey/datamonkey-js-server/production/app/relax/../../.hyphy/res//TemplateBatchFiles/SelectionAnalyses/`)
>Loaded a multiple sequence alignment with **291** sequences, **581** codons, and **1** partitions from `/home/datamonkey/datamonkey-js-server/production/app/relax/output/5d7bfccc9ad01c7476fcd241`

####Choose the set of branches to use as the _test_ set

1. [**Unlabeled branches**] Set of 573 unlabeled branches
2. [**TEST**] Set TEST with 6 branches

>Please choose an option (or press q to cancel selection):
1. [**Unlabeled branches**] Set of 573 unlabeled branches
2. [**TEST**] Set TEST with 6 branches

>Please choose an option (or press q to cancel selection):

Branch sets for RELAX analysis
* Selected 6 branches as the _test_ set: `FRA_DQ025985_04S16_2004, ITA_FJ005205_2C_279_2004, HRV_KP859578_2C_HR859_2014, Node445, Node443, ITA_FJ005232_411_2006`

* Selected 573 branches as the _reference_ set:
`VIE_AB054215_2A_CAT_V120_2000, FRA_DQ025947_2A_02B5_2002,
ITA_FJ005255_333_2005, Node29, FRA_DQ025962_2A_03C6_2003, Node28,
Node26, FRA_DQ026001_2A_04S32_2004, Node25, GER_AY742935_U6_1995,
Node24, FRA_DQ025958_2A_03C2_2003, Node23,
FRA_DQ025983_2A_04S14_2004, FRA_DQ025993_2A_04S24_2004, Node37,
ITA_KX434457_987_10_2010, Node36, Node22,
FRA_DQ025984_2A_04S15_2004, ITA_FJ005252_96_2002, Node41, Node21,
VIE_AB054217_2A_CAT_V154_2000, HUN_KF539796_H_9_2012, Node50,
HUN_KF539800_H_27_2012, Node49, HUN_KF539793_H_5_2012,
HUN_KF539797_H_11_2012, Node55, HUN_KF539794_H_7_2012,
HUN_KF539795_H_8_2012, Node59, HUN_KF539804_H_212_2012, Node58,
Node54, Node48, CHI_GQ857612_CPV08_04_2008,
CHI_GU569939_2A_YN0202_2002, Node63, Node47, ITA_AF306446_584_2000,
FRA_DQ025982_2A_04S13_2004, ITA_FJ005253_67_2005, Node73, Node71,
FRA_DQ025986_2A_04S17_2004, Node70, ITA_KF373577_2A_714_2001,
Node69, HUN_KF539798_H_31_2012, HUN_KF539799_H_39_2012, Node79,
HUN_KF539805_H_36_2012, Node78, Node68, ITA_AF306447_618_2000,
Node67, NIG_HQ602992_19_10_2010, Node66, Node46,
ITA_AF393506_2A_699_2000, FRA_DQ025943_2A_01S1_2001, Node86,
ITA_KF385388_2A_SICILY_X83090_2009, Node85, Node45,
ITA_KF373592_2A_329_2008, Node44, Node20,
FRA_DQ026002_2A_04S33_2004, Node19, NIG_HQ602995_15_10_2010,
Node18, VIE_AB054223_2C_LEOPARD_V140_2000,
ITA_GU362932_CAT11_2008, Node93, Node17, NZE_AY742933_339_1993,
Node16, JPN_AB437434_1887_F_3_2008, CHI_GU392236_FOX_HB1_2009,
CHI_GU392241_RACCOONDOG_HB1_2009,
CHI_GU392242_RACCOONDOG_HB10_2009,
CHI_GU392244_RACCOONDOG_HB7_2009, Node112, Node110,
CHI_KJ170679_RACCOONDOG_HEB10_2_2010, Node109, Node107,
CHI_GU392239_RACCOONDOG_HB6_2009,
CHI_GU392240_RACCOONDOG_HB3_2009, Node117,
CHI_KJ194463_RACCOONDOG_HEB10_3_2010, Node116, Node106,
CHI_GU392237_FOX_HB2_2009, Node105, Node103,
USA_EU659116_CPV_5_1979, FIN_U22193_RACCOONDOG_RD87_1987,
Node124, VAC_FJ011097_MERIAL_2006, CHI_GQ169553_VAC2_2007, Node133,
VAC_KY083090_SINGAPORE_2016, Node132, CHI_GU569943_YB8301_1983,
VAC_JN625224_INDIA_VAC6_2011, ARG_KM236572_NNGAG_2012, Node139,
Node137, Node131, VAC_FJ011098_INTERVET_2006, Node130,
VAC_JN625220_INDIA_VAC2_2011, Node129,
VAC_JN625221_INDIA_VAC3_2011, Node128, ITA_FJ222824_388_05_3_2005,
CHI_FJ432718_CPV_CV_2008, VAC_JN625219_INDIA_VAC1_2011,
CHI_KF803602_2010_BJ_A72_2010, Node151, Node149, Node147,
VAC_JN625222_INDIA_VAC4_2011, Node146, USA_M10989_1985,
USA_U22186_CPV_128_1995, Node155, Node145, Node127, Node123,
VAC_EU914139_PFIZER_2006, VAC_FJ197847_PFIZER_2007,
VAC_GU212790_PRIMODOG_2009, VAC_GU212791_VANGUARD_2009,
Node164, Node162, Node160, VAC_KY083089_SINGAPORE_2016, Node159,
USA_M19296_CPV_N_1988, Node158, Node122, Node102,
CHI_FJ231389_FPV_MONKEY_BJ_22_2008,

CHI_KJ170680_RACCOONDOG_HLJ11_1_2011, Node169, Node101,
CHI_KF803600_2010_BJ_A68_2010, Node100,
USA_JN867598_BOBCAT_KS_44_2010, USA_KJ813832_FISHER_ND_14_2013,
Node178, USA_KJ813831_FISHER_ND_17_2013,
USA_KJ813835_FISHER_ND_19_2013, Node181, Node177,
USA_JN867618_RACCOON_WI_37_2010, USA_JX475233_SC_182_A_2011,
USA_JX475246_CO_2503_2010, Node188, USA_JX475234_ME_258_2011,
Node187, Node185, USA_JX475248_CO_1102_2011, Node184, Node176,
USA_KJ813870_RACCOON_TX_1_2013, Node175,
USA_JN867599_RACCOON_KY_39552_2009,
USA_JN867611_RACCOON_KY_358_B_2009, Node195,
USA_JN867610_RACCOON_VA_118_A_2007, USA_JX475239_GA_06_2011,
USA_JX475279_TN_1_2011, Node202, Node200, USA_JX475284_TN_26_2011,
Node199, USA_KJ813890_REDFOX_MA_197_2012, Node198, Node194,
Node174, HUN_KF539801_H_25_2012, HUN_KF539803_H_2_2012, Node207,
Node173, Node99, JPN_D26079_1993, USA_EU659118_CPV_13_1981,
CHI_GU569948_2A_CC8601_1986, Node213, Node211,
FRA_DQ025952_2A_03B12_2003, BRA_DQ340404_2A_BR6_1980,
BRA_DQ340410_2A_BR315_1986, Node220, USA_M24000_FPV_CPV_31_1988,
USA_M24003_FPV_CPV_15_1988, Node223, Node219, Node217,
BRA_DQ340407_2A_BR145_1980, Node216, Node210, Node98,
FRA_DQ025950_2A_02B9_2002, KOR_EF599098_2C_POME_2006, Node232,
CHI_DQ354068_2A_REDPANDA_RPPV_2004, Node231,
KOR_EF599096_DH426_2005, THA_FJ869126_KU5_2008,
THA_FJ869134_KU23_2003, Node238, Node236, Node230,
ITA_FJ005258_80_2008, Node229, THA_FJ869130_KU13_2004,
CHI_KF803615_2011_BJ_B25_2011, Node242, Node228,
CHI_GU569942_2A_JL0202_2002, CHI_GU569946_2A_JL0201_2002, Node245,
Node227, Node97, Node15, USA_AY742953_435_2003,
ITA_KF373571_2A_685_1999, Node248, Node14, ITA_FJ005257_54_2008,
ITA_KF373611_2A_409_2010, Node251, Node13,
VIE_AB054218_2B_CAT_V123_2000, VAC_FJ222823_2B_29_1997, Node262,
ITA_FJ005264_134_2005, Node261, VIE_AB054219_2B_CAT_V209_2000,
VIE_AB054220_2B_CAT_V217_2000, VIE_AB120723_2B_HCM_23_2003,
CHI_EU145954_2B_BJ044_20O7, Node278, Node276, Node274,
VIE_AB120720_2B_HCM_6_2003, Node273, VIE_AB120725_2B_HNI_3_4_2003,
Node272, VIE_AB054221_2B_LEOPARD_V204_2000, Node271,
VIE_AB054224_2C_LEOPARD_V203_2000, Node270,
VIE_AB120721_2B_HCM_8_2003, Node269, THA_FJ869139_KU66_2003,
Node268, VIE_AB120722_2B_HCM_18_2003,
VIE_AB120724_2B_HNI_2_13_2003, Node290, CHI_GQ857599_CPV05_04_2005,
CHI_GQ857601_CPV06_02_2006, Node293, Node289,
THA_KP715690_VT28_2014, THA_KP715716_VT143_2014, Node297,
THA_KP715691_VT43_2014, Node296, Node288,
CHI_GQ857605_CPV07_03_2007, Node287, Node267,
THA_FJ869122_KU1_2008, THA_FJ869123_KU3_2008, Node302, Node266,
Node260, JPN_AB115504_2C_97_008_1997, TAW_U72696_2B_T10_1996,
Node309, TAW_U72695_2A_T4_1996, Node308,
CHI_GQ857596_CPV05_01_2005, CHI_GQ857600_CPV06_01_2006, Node313,
Node307, THA_FJ869125_KU5_2004, Node306, BRA_DQ340411_2A_BR8_1990,

BRA_DQ340428_2A_BR209_1994, BRA_DQ340431_2A_BR56_1995, Node321, Node319, BRA_DQ340413_2A_BR18_1990, BRA_DQ340419_2A_BR570_1992, Node328, BRA_DQ340423_2A_BR136_1993, Node327, BRA_DQ340414_2A_BR31_1990, Node326, BRA_DQ340421_2A_BR597_1992, Node325, BRA_DQ340422_2A_BR22_1993, Node324, Node318, THA_FJ869128_KU11_2004, Node317, Node305, Node259, CHI_GQ857609_CPV08_01_2008, CHI_GU569940_2B_YN0203_2002, Node336, Node258, FRA_DQ025992_2B_04S23_2004, POR_KU662349_GREYWOLF_W33_1996, POR_KU662350_GREYWOLF_W52_2005, Node344, Node342, USA_M74849_39_1995, USA_U22896_CAT_1990, Node347, Node341, GER_FJ005261_G162_1997, Node340, BRA_DQ340409_2B_BR183_1985, Node339, Node257, USA_AY742932_193_1991, USA_AY742951_431_2003, USA_JN867605_2B_DOG_US_142805_2009, Node355, USA_EU659119_2B_CPV_410_2000, USA_EU659120_2B_CPV_411A_1998, Node360, VAC_JN625223_INDIA_VAC5_2011, Node359, VAC_FJ222822_2B_FORTDODGE_2008, Node358, Node354, Node352, Node256, ITA_FJ005263_42_2005, Node255, JPN_AB437433_1887_M_2_2008, TWN_EF592511_TWN1_2006, TAW_FJ265775_CPV301_2004, Node374, TAW_FJ265781_CPV307_2005, Node373, Node371, JPN_LC270891_2B_9985_2017, Node370, CHI_EU483515_2B_ZD13_2007, Node369, USA_JX475237_CT_372_2011, KOR_EF599097_2B_DH326_2006, Node380, Node368, USA_AY742955_436_2003, USA_JX475278_AR_1069_2012, USA_KJ813851_BOBCAT_ND_1168_2013, USA_KJ813881_GRAYWOLF_MI_832_2012, USA_KJ813882_RACCOON_NJ_1423_2012, Node401, Node399, Node397, USA_KJ813844_BOBCAT_ND_885_2013, Node396, Node394, USA_KJ813828_FISHER_F1F010712_2013, Node393, USA_JN867604_DOG_IL_137654_2008, USA_JX475242_WI_18268_2002, Node407, USA_KJ813892_COYOTE_AK_218_2013, Node406, Node392, USA_JX475251_CO_2235_2009, Node391, USA_JN867603_2B_DOG_KS_81213_2009, Node390, KOR_EU009205_2B_K029_2006, Node389, USA_JN867602_2B_DOG_CA_148743_2008, Node388, USA_JX475247_CO_1246_2010, USA_KJ813852_BOBCAT_ND_1170_2013, Node415, Node387, USA_KJ813827_FISHER_F1M111211_2013, USA_KJ813873_GRAYWOLF_MI_850_2012, Node418, Node386, FRA_DQ025961_2B_03C5_2003, Node385, ITA_FJ005265_140_2005, Node384, SAF_HQ602969_22_10SA_2010, Node383, Node367, ECU_KF149971_2C_ME32_2012, IND_KX469432_NEWCPV_2B_HILLER_2011, Node424, Node366, Node254, Node12, GER_AY742934_447_1995, RUS_JN033694_LAIKA_1993, Node428, USA_AY742936_395_1998, USA_JX475240_AZ_16382_01_1999, USA_JX475250_CO_728_2010, USA_KJ813842_BOBCAT_ND_502_2013, Node435, Node433, Node431, Node427, Node11, GER_FJ005260_G82_1997, USA_KJ813846_BOBCAT_ND_974_2013, Node438, Node10, ITA_FJ222821_2C_56_2000, Node9, Node442, Node8, URU_KM457126_2C_UY318_2010, Node7, URU_KC196081_2C_M95_2007, URU_KC196093_2C_M307_2011, URU_KC196097_2C_M242_2010, Node452, Node450, Node6, FRA_DQ025964_03C8_2003, POR_KT275253_2C_PT036_12_2012, Node456, URU_KC196086_2C_M55_2006,

Node455, Node5, ITA_FJ005240_208_2007, ITA_FJ005248_219_2008, Node460, Node4, URU_KC196083_2C_M82_2007, URU_KC196101_2C_M187_2009, Node463, Node3, GER_FJ005196_2C_G7_1997, URU_KC196091_2C_M326_2011, Node467, GER_FJ005199_2C_G172_1997, USA_JX475252_CO_1316_2010, Node470, Node466, Node2, FRA_DQ025954_03B14_2003, USA_KJ813843_BOBCAT_ND_1160_2013, Node476, ECU_KF149984_2C_ME28_2012, Node475, ITA_FJ005195_2C_136_2000, POR_KT275252_2C_PT013_12_2012, Node480, Node474, ITA_FJ005218_2C_330_2006, ITA_FJ005233_40_2007, Node484, USA_JX475260_CO_704_2010, Node483, Node473, Node1, FRA_DQ025942_01B1_2001, ITA_FJ005214_2C_67_2006, BRA_KY073269_UFMT_2015, Node493, Node491, FRA_DQ025951_03B10_2003, ITA_FJ005231_406_2006, Node497, HRV_KP859577_2C_HR856_2014, Node496, Node490, ITA_FJ005209_2C_303_2004, ITA_FJ005251_239_2008, Node503, URU_KM457103_2C_UY12_2006, Node502, ITA_FJ005226_383_2006, URU_KC196096_2C_M247_2010, Node507, Node501, Node489, FRA_DQ025960_03C4_2003, ARG_JF414819_ARG35_2008, Node514, URU_KC196107_2C_M129_2008, AUS_KU508693_2C_LW_2015, Node517, Node513, FRA_DQ025969_03S5_2003, URU_KC196085_2C_M57_2007, Node520, Node512, ITA_FJ005247_195_2008, ITA_KX434460_52238_12_2012, Node523, Node511, FRA_DQ025975_04S6_2004, URU_KC196102_2C_M185_2009, ECU_KF149962_2C_ME1_2012, ECU_KF149963_2C_ME10_2012, Node534, ECU_KF149964_2C_ME23_2012, Node533, ECU_KF149969_2C_ME31_2012, Node532, Node530, Node528, FRA_DQ025994_04S25_2004, POR_KT275255_2C_PT238_14_2014, Node539, Node527, ITA_FJ005212_2C_349_2004, URU_KC196105_2C_M152_2008, Node543, USA_JX475243_ID_22772_2009, USA_KJ813858_PUMA_ND_F93_2013, Node546, Node542, Node526, Node510, Node488, FRA_DQ025965_03C9_2003, ARG_JF414818_ARG32_2008, ARG_KM236569_CUBA_2013, Node557, ARG_JF414821_ARG48_2009, Node556, ARG_JF414820_ARG44_2009, Node555, Node553, USA_JX475273_MT_909_2012, USA_KJ813888_COYOTE_MT_878_2012, Node562, Node552, USA_KJ813848_BOBCAT_ND_1162_2013, URU_KM457104_2C_UY47_2006, Node565, Node551, URU_KC196089_2C_M349_2011, USA_KJ813854_PUMA_ND_F205_2013, Node568, Node550, FRA_DQ025976_04S7_2004, URU_KM457131_2C_UY368_2011, Node572, ITA_FJ005216_2C_284_2006, ITA_KU508407_2C_25835_09_2009, ITA_KX434459_27692_1_11_2011, Node577, Node575, Node571, Node549`

####RELAX analysis type

1. [**All**] [Default] Fit descriptive models AND run the relax test (4 models)
2. [**Minimal**] Run only the RELAX test (2 models)

>Please choose an option (or press q to cancel selection):

Obtaining branch lengths and nucleotide substitution biases under the nucleotide GTR model

* Log(L) = -6479.61, AIC-c = 14134.58 (587 estimated parameters)

Obtaining the global omega estimate based on relative GTR branch lengths and nucleotide substitution biases
* Log(L) = -6189.55, AIC-c = 13573.31 (595 estimated parameters)
* non-synonymous/synonymous rate ratio for *Reference* =   0.1231
* non-synonymous/synonymous rate ratio for *Test* =   0.0000

Improving branch lengths, nucleotide substitution biases, and global dN/dS ratios under a full codon model
* Log(L) = -6189.55, AIC-c = 13573.31 (595 estimated parameters)
* non-synonymous/synonymous rate ratio for *Reference* =   0.1232
* non-synonymous/synonymous rate ratio for *Test* =   0.0000

Fitting the general descriptive (separate k per branch) model

* Log(L) = -5961.56, AIC-c = 14293.63 (1177 estimated parameters)
* The following baseline rate distribution for branch-site combinations was inferred

Selection mode	dN/dS	Proportion, %	Notes
Negative selection	0.742	97.486	
Negative selection	0.793	2.507	
Diversifying selection	16.204	0.007	Not supported by data

* Branch-level relaxation or intensification parameter distribution has mean 5.80, median 5.35, and 95% of the weight in 1.55 - 7.86

Fitting the alternative model to test K != 1
* Log(L) = -6176.41, AIC-c = 13555.09 (599 estimated parameters)
* Relaxation/intensification parameter (K) =   11.55
* The following rate distribution was inferred for **test** branches

Selection mode	dN/dS	Proportion, %	Notes
Negative selection	0.000	87.363	
Negative selection	0.165	12.567	
Diversifying selection	9999844607.5...	0.070	

* The following rate distribution was inferred for **reference** branches

Selection mode	dN/dS	Proportion, %	Notes
Negative selection	0.000	87.363	
Negative selection	0.856	12.567	
Diversifying selection	7.344	0.070	

* Potential convergence issues due to flat likelihood surfaces; checking to see

whether K > 1 or K < 1 is robustly inferred

Potential for highly unreliable K inference due to multiple local maxima in the likelihood function, treat results with caution
> Relaxation parameter reset to opposite mode of evolution from that obtained in the initial optimization.
* Log(L) = -6158.70, AIC-c = 13519.67 (599 estimated parameters)
* Relaxation/intensification parameter (K) = 0.00
* The following rate distribution was inferred for **test** branches

Selection mode	dN/dS	Proportion, %	Notes
Negative selection	0.000	90.939	
Neutral evolution	1.000	9.025	
Diversifying selection	1.000	0.036	Collapsed rate class

* The following rate distribution was inferred for **reference** branches

Selection mode	dN/dS	Proportion, %	Notes
Negative selection	0.000	90.939	
Neutral evolution	1.000	9.025	
Diversifying selection	145.064	0.036	

Fitting the null (K := 1) model
* Log(L) = -6159.25, AIC-c = 13518.75 (598 estimated parameters)
* The following rate distribution for test/reference branches was inferred

Selection mode	dN/dS	Proportion, %	Notes
Negative selection	0.004	90.943	
Negative selection	0.965	9.023	
Diversifying selection	154.361	0.035	

Test for relaxation (or intensification) of selection [RELAX]
Likelihood ratio test **p = 0.2974**.
>No significant evidence for relaxation (or intensification) of selection among **test** branches _relative_ to the **reference** branches at P<=0.05

Fitting the partitioned descriptive model (separate distributions for *test* and *reference* branches)
