# Supplementary figures and images for: Evidence of CPV2c introgression into Croatia and novel insights into phylogeny and cell tropism
Source: Sci Rep. 2019 Nov 15;9:16909. doi: 10.1038/s41598-019-53422-9 (PMC6858334; doi:10.1038/s41598-019-53422-9)

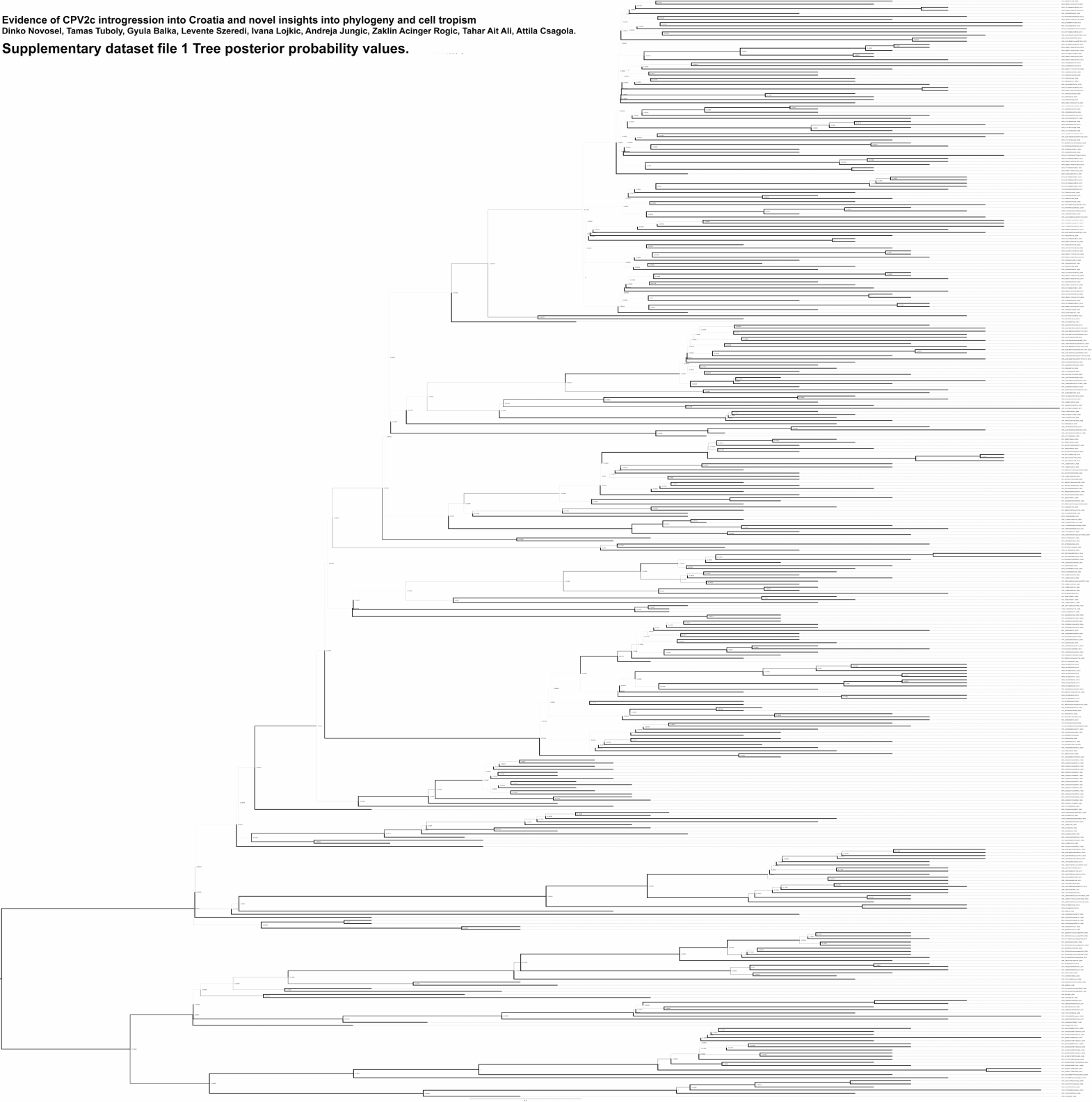

Supplement: Supplementary file 9 — Posterior probability tree [file 41598_2019_53422_MOESM9_ESM.pdf]
